# Exploring autophagy with Gene Ontology

**DOI:** 10.1080/15548627.2017.1415189

**Published:** 2018-02-17

**Authors:** Paul Denny, Marc Feuermann, David P. Hill, Ruth C. Lovering, Helene Plun-Favreau, Paola Roncaglia

**Affiliations:** a Functional Gene Annotation, Institute of Cardiovascular Science, University College London, London, UK; b SIB Swiss Institute of Bioinformatics, Geneva, Switzerland; c The Jackson Laboratory, Bar Harbor, ME, USA; d Department of Molecular Neuroscience, UCL Institute of Neurology, London, UK; e European Bioinformatics Institute (EMBL-EBI), European Molecular Biology Laboratory, Wellcome Genome Campus, Hinxton, Cambridge, UK; f The Gene Ontology Consortium

**Keywords:** annotation, autophagy, biocuration, curation, enrichment analysis, Gene Ontology, network analysis, Parkinson disease

## Abstract

Autophagy is a fundamental cellular process that is well conserved among eukaryotes. It is one of the strategies that cells use to catabolize substances in a controlled way. Autophagy is used for recycling cellular components, responding to cellular stresses and ridding cells of foreign material. Perturbations in autophagy have been implicated in a number of pathological conditions such as neurodegeneration, cardiac disease and cancer. The growing knowledge about autophagic mechanisms needs to be collected in a computable and shareable format to allow its use in data representation and interpretation. The Gene Ontology (GO) is a freely available resource that describes how and where gene products function in biological systems. It consists of 3 interrelated structured vocabularies that outline what gene products do at the biochemical level, where they act in a cell and the overall biological objectives to which their actions contribute. It also consists of ‘annotations’ that associate gene products with the terms. Here we describe how we represent autophagy in GO, how we create and define terms relevant to autophagy researchers and how we interrelate those terms to generate a coherent view of the process, therefore allowing an interoperable description of its biological aspects. We also describe how annotation of gene products with GO terms improves data analysis and interpretation, hence bringing a significant benefit to this field of study.

## Introduction

The Gene Ontology (GO) is a freely available public resource that describes how genes act in biological systems and places those descriptions in a computable format [1]. One part of the resource, the ontology, consists of 3 interrelated structured vocabularies that describe the biochemical activity of a gene product (i.e. a protein or an RNA), the subcellular location of action of a gene product, and the overall biological objective that the gene product's function helps to achieve. The ontology is structured with relationships between vocabulary terms that can be used to understand how the terms fit together to form a coherent picture of biology. Terms contain human-readable textual definitions used by biocurators during the annotation process (see below), and computable definitions used for automated reasoning (see Results). The other part of the GO resource consists of associations of gene products with terms, called annotations. Manual annotations are created by biocurators who search and read the published literature, and use the reported results to make an evidence-supported association of a gene product with an appropriate GO term [2]. The GO is used for many purposes, ranging from simple examination of annotations to answer the question “what does this gene do?” to sophisticated computational analysis used to interpret large datasets [3,4]. In fact, GO is arguably the most widely used bioinformatics resource in functional analysis of genes and proteins, with a PubMed search using the string “gene ontology” returning more than 12,700 articles as of May 2017.

Autophagy is a ubiquitous cellular process used as a mechanism to maintain cellular homeostasis and to respond to cellular stress [5]. During autophagy, cellular components are degraded and, in most cases, recycled for later use. Autophagy can be accomplished by several distinct mechanisms and can have distinct degradation targets [5–8]. Given its importance in normal physiological processes, disruptions in autophagy or its regulation have been associated with a variety of pathological conditions [9–18]. For example, in the nervous system, disruptions in mitophagy, a type of macroautophagy that degrades mitochondria, have been associated with Parkinson disease [10]. Autophagic clearance of protein aggregates has been presented as a possible contributor to the pathology of Alzheimer disease [11], Huntington disease [12], amyotrophic lateral sclerosis [13] and Parkinson disease [14]. Beyond the nervous system, autophagy has also been implicated in cardiovascular disease [15], cancer [16], immune system function [17] and recently in Niemann-Pick disease [18]. Considering the importance of autophagy in both the normal function of cells and its implication in pathogenesis, we have focused on improving the representation of autophagy in GO from both ontology-development and annotation approaches.

Here, we describe our work to expand and refine the GO terms that represent autophagy with an emphasis on macroautophagy. We explain the rationale behind the creation of new terms related to autophagy and we detail our methodology to create computable definitions. The result is a representation of autophagy that reflects current knowledge, but is flexible enough to allow for expansion and revision if and as new terms are needed for annotation. We also report on strategies to annotate genes using the new GO representation. Finally, we illustrate how data analysis can be improved by our work. Overall, the enhanced GO resources in the field of autophagy that result from our efforts allow significant improvements in the capture of autophagy-related information and data analysis, thereby bringing benefit to the research community.

## Results

### Different types of autophagy require specific biological process terms

In GO, terms are described in a hierarchical fashion where specific terms are defined by their relationship with more general terms and by how they differ from their general ‘parents.’ The structure of the GO is such that any given ‘child’ term may be a subtype of more than one parent, and any ‘parent’ term may have any number of ‘child’ terms (or none). For example, ‘mitophagy’ is a subtype of (is_a) ‘selective autophagy’. A variety of relations are used between GO terms, in addition to the standard ‘is_a’ relation; e.g., functions and processes can have ‘occurs_in’ relations with respect to cellular components (‘mitochondrial RNA processing’ occurs_in ‘mitochondrion’) [19]. GO terms are defined in 2 ways: a human-readable textual definition and, wherever possible, a computable definition that can be interpreted computationally. The latter definitions are determined by axioms, i.e. statements consisting of necessary and sufficient relationships to define the term with respect to other GO terms or with respect to GO and other specialized ontologies that are imported into GO [20]. These axioms are used by computational reasoners to infer relationships between terms [21] (thereby automatically placing them within the ontology structure) and to ensure that terms are logically consistent [22].

**Figure 1.**
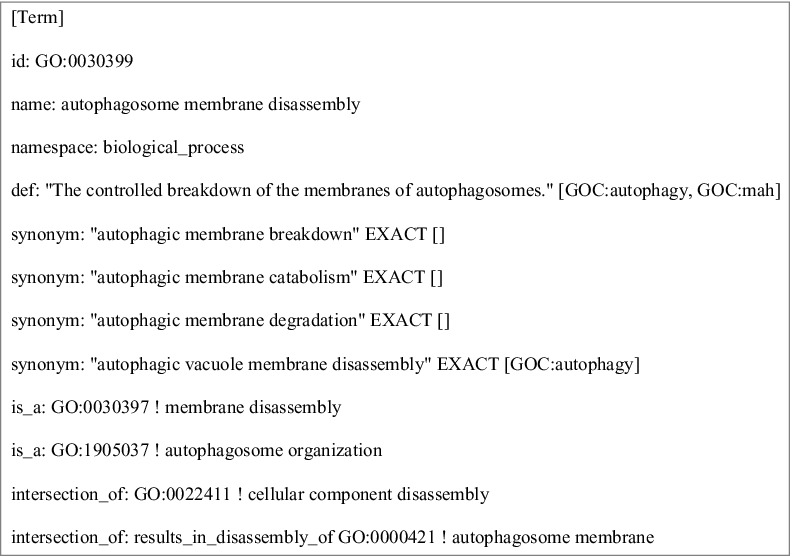
Information associated with the GO term ‘autophagosome membrane disassembly’ (GO:0030399). The ontology stanza, in obo format,^105^ shows the term and various metadata associated with it. The textual definition labeled ‘def’ is the human-readable definition used by biocurators. The ‘intersection_of’ tags specify the necessary and sufficient conditions to define the term in a computable format. The stanza also contains other information related to a term, such as synonyms.^105^

In addition to relationships inferred by computable definitions, GO terms can have additional links with other terms in the ontology. Figure 1 shows an example of the GO term for ‘autophagosome membrane disassembly’ (GO:0030399). The textual definition of the term, “The controlled breakdown of the membranes of autophagosomes,” is a human-readable definition available to biomedical users and biocurators. The listed synonyms are used for searching purposes. The lines that begin with ‘is_a’ in Figure 1 are relationships between the term and other terms in the ontology. These required relationships are either manually asserted or are asserted after computational reasoning [22]. In this example, the membrane disassembly term can be inferred by reasoning and the autophagosome organization term was added by a biocurator. Finally, the computable definition is given by the 2 lines labeled ‘intersection_of’ in Figure 1. The computable definition says that the term can be expressed as a type of cellular component disassembly that disassembles an autophagosome membrane.

In considering the structure of GO with respect to autophagy, we first determined how the children of autophagy were ‘different’ from the parent term. The parent term ‘autophagy’ (GO:0006914) is textually defined as “The cellular catabolic process in which cells digest parts of their own cytoplasm; allows for both recycling of macromolecular constituents under conditions of cellular stress and remodeling the intracellular structure for cell differentiation”. Because different types of autophagy use different mechanisms that can be shared with other cellular processes, it was not possible to determine the ‘necessary and sufficient’ conditions for a computable definition of the generic term using the existing structure of the ontology, so the term was manually placed in the ontology structure. Different types of autophagy can be distinguished in 2 ways: the mechanism by which the autophagic process proceeds, and the target of the autophagic process. Both of these distinguishing features have been used to describe subtypes of autophagy. A list of autophagy-related terms available in GO is shown in Table S1. Also the AmiGO2 browser [23] allows interactive navigation of the ontology structure, and provides a view of term definitions, synonyms and references, in a comprehensive and user-friendly way. GO ontology files are freely available for download [24].

**Figure 2.**
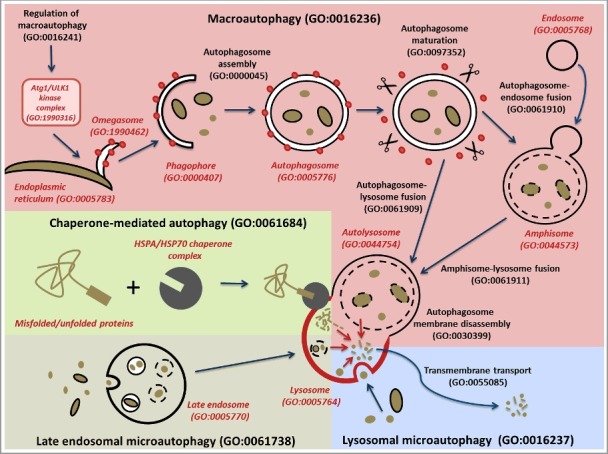
Schematic representation of the different types of autophagy. The main autophagy-related GO terms and their corresponding IDs are indicated. The different types of autophagy are represented with different background colors: macroautophagy in pink; chaperone-mediated autophagy, green; late endosomal microautophagy, cream; and lysosomal microautophagy, blue. The major steps of macroautophagy are shown, from the formation of omegasomes to the fusion with the lysosome, via engulfment of macroautophagy targets by the phagophore, the formation of a closed autophagosome, and the maturation of the autophagosome through removal of ATG proteins such as Atg8-family proteins. The branching pathway via an amphisome is also represented. Black lines correspond to membranes (apart from the lysosomal membrane which is in red). Red dots correspond to ATG proteins such as Atg8-family protein members required for the autophagosome assembly and removed during autophagosome maturation. The scissors represent proteins such as ATG4 and the PI3P phosphatase that strip ATG proteins from the outer autophagosome membrane during the maturation step. The red arrows within the lysosome represent lysosomal breakdown of the autophagy targets. The CMA-targeting motif that is recognized by a cytosolic chaperone is indicated by a rectangle on the misfolded/unfolded protein. To distinguish between biological process and cellular component GO terms, the latter are in red italics.

Mechanistically, 4 subtypes of autophagy have been described in GO: macroautophagy (often referred to in the literature as autophagy), lysosomal microautophagy, late endosomal microautophagy and chaperone-mediated autophagy (Figure 2). Chaperone-mediated autophagy (GO:0061684) specifically degrades proteins that contain a chaperone-mediated autophagy (CMA)-targeting motif that is recognized by a cytosolic chaperone and targets them to the lysosome where they translocate directly across the membrane in a LAMP2-dependent manner [8]. Lysosomal microautophagy (GO:0016237) occurs by the direct engulfment of cytoplasmic materials by the lysosome [6]. Late endosomal microautophagy (GO:0061738) delivers chaperone-tagged materials to a late endosomal compartment for degradation [7]. Finally, macroautophagy (GO:0016236), the main focus of this project, is the engulfment of cytosolic material and organelles by double-membrane transient structures called phagophores that mature into autophagosomes [5,25]. Biocurators use this feature to differentiate the use of the term autophagy in the literature when it refers to macroautophagy. Each of these types of autophagy is defined such that they can be distinguished from one another.

**Figure 3.**
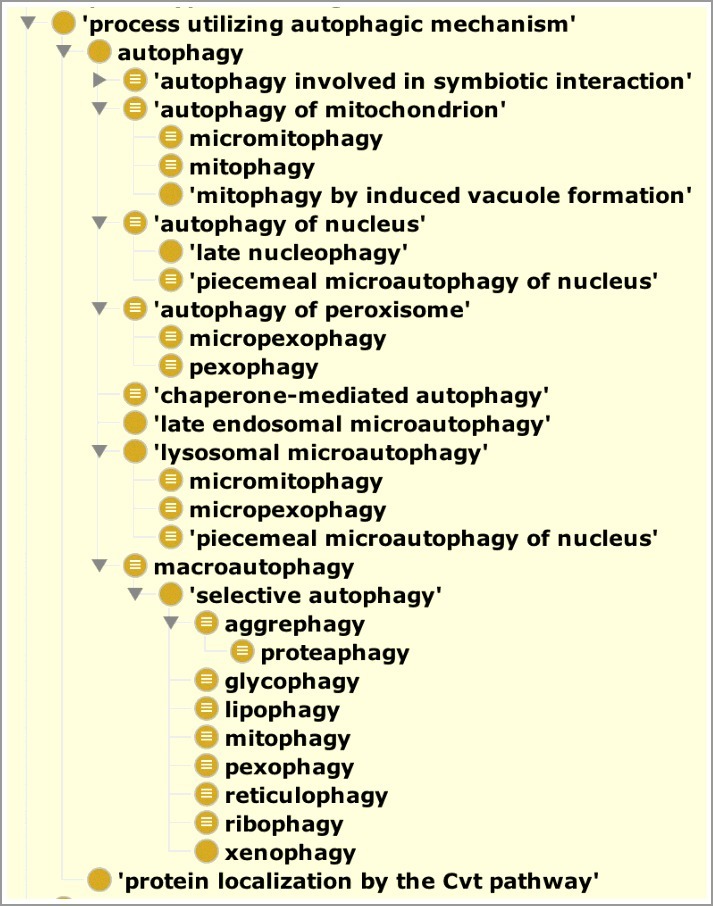
A view of the ontology showing the ‘is_a’ neighborhood around autophagy. This figure was generated by the Protégé ontology editing tool^33^ and shows the ‘is_a’ hierarchy of the autophagy branch of the ontology. Each indentation indicates a term that is a type of its parent. The grouping term ‘process utilizing autophagic mechanism’ is used to group conventional autophagy with the Cvt pathway, both of which use common mechanisms. General terms ‘autophagy of X’ are used to group types of autophagy that can use different mechanisms to degrade similar targets. For example, ‘mitophagy’ is a child of both ‘autophagy of mitochondrion’ and ‘macroautophagy’. Macroautophagy of specific targets are grouped under a term to describe the ‘selective autophagy’ pathway and nomenclature has been assigned to be consistent with the names that are used prominently in the literature. To view the entire structure of this branch see Table S1; for a dynamic view we also recommend the 'inferred view’ tab in the AmiGO2 browser.^23^

The second way to distinguish types of autophagy is by the target that is degraded in the process [26]. For example, ‘autophagy of mitochondrion’ (GO:0000422), ‘autophagy of nucleus’ (GO:0044804) and ‘autophagy of peroxisome’ (GO:0030242) describe the autophagic degradation of mitochondria, nuclei and peroxisomes respectively without describing the precise autophagic mechanism used for the degradation. Figure 3 shows a graphical view of the subtype structure of the ontology directly below the most general grouping term, ‘process utilizing autophagic mechanism’ (GO:0061919, see below).

Whenever possible, distinguishing features based on other ontology terms are used to create computable definitions. Using distinguishing features allows for expansion and refinement of the ontology as research in the area proceeds. For example, if an additional mechanistic type of autophagy needs to be described, it can be added as a new term with respect to the features that make it unique. This strategy is similar to the one recently adopted for creating computable definitions for biochemical pathways in GO [27].

The term ‘autophagy involved in symbiotic interaction’ (GO:0075071) (Figure 3) allows for annotation of gene products that play a role in autophagic processes that occur as part of symbiotic interactions between organisms [28]. In addition to its original meaning of self-eating, macroautophagy can also degrade invading pathogens such as bacteria [29]. Therefore, the more specific term ‘xenophagy’ (GO:0098792) is a child of ‘selective autophagy’ which is a child of ‘macroautophagy’ (Figure 3).

The Cvt pathway [30] (GO:0032258, ‘protein localization by the Cvt pathway’; synonym: cytoplasm-to-vacuole targeting pathway) was not made a subtype of autophagy in GO because we restricted the use of the term ‘autophagy’ to represent only catabolic processes [31]. However, as the Cvt pathway uses much of the same machinery as autophagy and is closely related to it, we created a new grouping term, called ‘process utilizing autophagic mechanism’ (GO:0061919), that includes conventional ‘catabolic’ autophagy and the Cvt pathway (Figure 3 and Table S1). This grouping term will also be used for other processes that utilize autophagy machinery if and as they are required for annotation.

In addition to describing the types of autophagy, we also described subprocesses and specialized components that play roles in autophagy, focusing on our annotation-driven efforts to curate genes involved in macroautophagic processes (see below). Macroautophagy occurs through the formation of a specific organelle called the autophagosome [9] (GO:0005776, Figure 2). The steps of the autophagosome assembly, as well as the different cellular components involved, have been described in Feuermann et al [32]. We have improved the definitions of autophagy-related terms by adding appropriate relationships between some processes and the relevant cellular component terms. For example, we made ‘autolysosome’ (GO:0044754) a necessary participant in the process of ‘chaperone-mediated autophagy’ (GO:0061684), and we made the ‘omegasome’ (GO:1990462) and the ‘phagophore’ (GO:0061908) participate in the process of ‘autophagosome assembly’ (GO:0000045).

**Figure 4.**
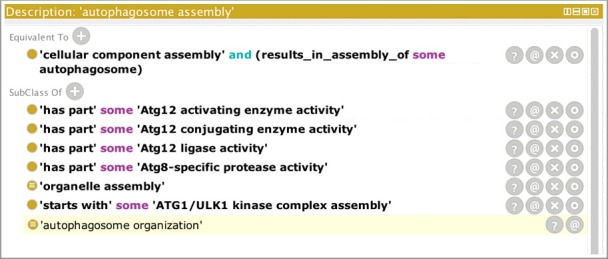
The GO term ‘autophagosome assembly’ showing relationships to molecular functions. A screenshot of the Protégé ontology-editing tool^33^ focusing on ‘autophagosome assembly’ (GO:0000045). The top part of the panel displays the equivalence axiom for the term ‘autophagosome assembly’ which states that the term is equivalent to a type of ‘cellular component assembly’ that results in the assembly of an ‘autophagosome’. The lower part of the panel shows the relations between this term and other terms in the ontology. The last row displays the result of computational reasoning and shows that, based on the equivalence axiom, ‘autophagosome assembly’ (GO:0000045) is a type of ‘autophagosome organization’ (GO:1905037). Rows that are not shaded in the lower part of the panel indicate relationships that have been asserted by an editor. For example, the ‘autophagosome assembly’ process has been asserted as a type of ‘organelle assembly’ (GO:0070925), and the ‘starts_with’ row indicates that the process starts with the creation of the Atg1/ULK1 complex. The ‘has_part’ rows show GO molecular functions that are necessary for autophagosome assembly to occur.

In some cases, the molecular functions that are used to carry out the steps of the autophagic programs have been well characterized, and where possible, we have added relationships in the ontology between those molecular functions and the corresponding autophagic processes. Figure 4 shows a screenshot of the Protégé ontology-editing tool [33] focusing on ‘autophagosome assembly’ (GO:0000045). The ‘has_part’ rows show GO molecular functions that are necessary for autophagosome assembly to occur and therefore link the GO processes with the GO molecular functions in a biologically meaningful way.

### ‘Autophagy’ versus ‘regulation of autophagy’

Autophagy plays a crucial physiological role; therefore it has to be tightly regulated. Indeed, defects in the regulation of autophagy are associated with many diseases [6]. Many signaling pathways such as TOR signaling and the Toll-like receptor-signaling pathway regulate autophagy, and these are triggered by events such as starvation, stress response or pathogen recognition [34–38]. To distinguish between processes involved in the regulation of autophagy (i.e. upstream of autophagy) and autophagy itself, a clear beginning of the autophagy process needed to be defined. Our revision of the autophagy ontology domain has led to clearer boundaries for the process terms, the most straightforward case being the macroautophagy subclass, which begins with the assembly of the Atg1/ULK1 kinase complex by upstream signaling [39]. Activation of the Atg1/ULK1 kinase complex is the first step in autophagosome assembly; therefore, in the ontology, ‘autophagosome assembly’ (GO:0000045) has a ‘starts_with’ relationship with ‘Atg1/ULK1 complex assembly’ (GO:1904745; Figure 4). The activation of BECN1 is also required for autophagosome assembly and is generally considered to follow ULK1 activation [39]. By defining the start of the autophagosome assembly process, any autophagy-associated functions or processes that are upstream of this complex assembly step, such as inhibition of MTOR or activation of AMPK, are considered to regulate the process, rather than be part of the process. The situation is less obvious when it comes to microautophagy or chaperone-mediated autophagy. However, we have defined the start of these processes as the step when a protein or organelle is marked for degradation. The autophagy terms are defined as ending with transmembrane transport because although not studied in as much detail, our current understanding is that this is the last step in which the products of the catabolic process are transported out of the lysosome and before the lysosome is recycled [40].

### Impact of ontology revisions on existing annotations

**Figure 5.**
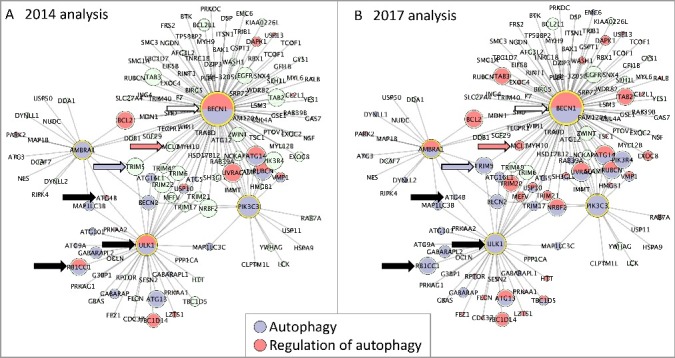
Protein interactome network associated with 4 key autophagy proteins. The in silico human interactome associated with AMBRA1, BECN1, PIK3C3, ULK1 assembled with Cytoscape^58^ and analyzed with BinGO.^60^ Each node is a protein and each edge is an interaction between 2 proteins. GO terms associated with each protein are indicated by the node color (blue indicates ‘autophagy’ and red ‘regulation of autophagy’), green nodes indicate proteins that are not annotated to either of the selected GO terms. The size of each node is proportional to the number of times the interaction has been captured as an annotation. (**A**) BinGO analysis using the 2014 GO annotation file, 29 proteins are associated with the GO term ‘autophagy’, 18 with ‘regulation of autophagy’. (**B**) BinGO analysis using the 2017 GO annotation file, 45 proteins are associated with the GO term ‘autophagy’, 39 with ‘regulation of autophagy’. The black arrows indicate proteins associated with the GO terms ‘autophagy’ and ‘regulation of autophagy’ in 2014, but now associated with only ‘autophagy’ (February 2017). The blue and red arrows indicate TRIM5 and MCL1 (Q9C035 and Q07820); these proteins were not associated with any autophagy-related term in 2014 but are now associated with ‘autophagy’ or ‘regulation of autophagy’, respectively. The white arrow indicates BECN1; this remains associated with both ‘autophagy’ and ‘regulation of autophagy’ terms.

Providing clearer definitions of where processes begin led us to review previous GO annotations. Examining existing annotations revealed that around half of the proteins annotated to ‘regulation of autophagy’ (or to its positive and negative regulation children terms) were also annotated to ‘autophagy’ (or to a child term). Having now demarcated the beginning and end of autophagy, we reviewed instances of such double annotations and refined them so that genes were associated with the most correct term based on available published experimental evidence. For example, several human proteins, including ULK1, RB1CC1 and ATG4B (UniProt IDs O75385, Q8TDY2 and Q9Y4P1 respectively), had been annotated with both terms, but following a review we removed the annotations to the regulation term (see Figure 5, black arrows). Some gene products are still associated with both ‘regulation of autophagy’ or the ‘autophagy’ terms. This occurs because the exact nature of how a gene product acts is still not fully understood, or because the original biocurator chose the term which best captured the data presented in the publication being curated, in the species being curated, and, in the absence of more precise information on that species, that specific annotation may not be revised at present. For example, human BECN1 (Q14457) is currently annotated with both ‘regulation of autophagy’ and ‘autophagy’ terms; the ‘autophagy’ annotation is supported by a direct assay, whereas ‘regulation of autophagy’ is derived from an annotation of an orthologous protein (see Figure 5, white arrow).

### Improving the annotation of proteins involved in autophagy

Our effort in curating proteins involved in or regulating autophagy resulted in new knowledge available to the scientific community, and quantifiable in >1,200 autophagy domain annotations associated with 474 human proteins (as of March 2017). Breaking down the progress we made, on January 1, 2014 the GO human proteome data included only 114 manual annotations using an autophagy domain GO term, associated with 95 proteins. Since then, 184 human proteins have been curated with autophagy terms based on experimental data, leading to the creation of 436 annotations. Additionally, there are now 200 author-statement supported annotations and >50 sequence-similarity supported annotations. The application of computational and manual approaches has provided an additional 330 annotations, through the transfer of experimentally supported annotations from model organism proteins to the reviewed human orthologs. In addition, there are a further 245 annotations based on other computational pipelines [41]. Below, we describe the approaches we took in curating autophagy players, and the results we obtained, in more detail.

Published literature on autophagy is abundant, with >2,500 papers in PubMed referring to mitophagy, microautophagy, xenophagy, pexophagy or chaperone-mediated autophagy, as of March 2017. Manual curation of autophagy literature was achieved following the selection of >200 papers containing relevant experimental data, as described in Materials and Methods. These GO annotations enable capture of the role of autophagy players in a detailed and comprehensive manner, and make this information freely available to the community in an electronic format from the GO Consortium (GOC) website [24]. We carried out the curation process by reviewing the most current literature available (March 2017), and by consulting with experts in the field when possible. Notably, comprehensive curation of each paper led to the capture of additional cellular roles (i.e. not specific to autophagy) of the autophagy-related gene products. So, although we focused on macroautophagy, we also captured information about the broader range of biology, resulting in a wider spectrum of annotations. An example is shown in Table 1, where manual curation captured experimental information about 8 proteins from a single scientific article; note that annotations refer not only to processes connected with autophagy, but also to metabolism, binding to specific chemical compounds, and cellular locations.

**Table 1.** Sample output of manual GO curation of a scientific article.

Gene Symbol	GO Identifier	GO Term Name	Evidence
***Biological process***			
*PIP4K2A*	GO:0010506	regulation of autophagy	IMP
*PIP4K2A*	GO:2000786	positive regulation of autophagosome assembly	IMP
*PIP4K2B*	GO:0010506	regulation of autophagy	IMP
*PIP4K2B*	GO:2000786	positive regulation of autophagosome assembly	IMP
*MTMR3*	GO:0042149	cellular response to glucose starvation	IMP
*MTMR3*	GO:2000785	regulation of autophagosome assembly	IMP
*PIKFYVE*	GO:2000785	regulation of autophagosome assembly	IMP
*PIP4K2C*	GO:0010506	regulation of autophagy	IMP
*PIP4K2C*	GO:2000786	positive regulation of autophagosome assembly	IMP
*ZFYVE1*	GO:0009267	cellular response to starvation	IDA
*ZFYVE1*	GO:0016236	macroautophagy	IDA
***Molecular function***			
*WIPI2*	GO:0010314	phosphatidylinositol-5-phosphate binding	IDA
*WIPI2*	GO:0010314	phosphatidylinositol-5-phosphate binding	IDA
*WIPI2*	GO:0032266	phosphatidylinositol-3-phosphate binding	IDA
***Cellular component***			
*PIP4K2A*	GO:0005776	autophagosome	IMP
*PIP4K2B*	GO:0005776	autophagosome	IMP
*PIP4K2C*	GO:0005776	autophagosome	IMP
*ING2*	GO:0005634	nucleus	IMP
*ING2*	GO:0005886	plasma membrane	IMP
*ZFYVE1*	GO:0016020	membrane	IDA
*WIPI2*	GO:0016020	membrane	IDA

Experimental data described by Vicinanza et al [106]. were selected for annotation as they describe the role and location of 8 autophagy-related human proteins. The QuickGO [91,92] filter option was used to retrieve all manual experimental annotations associated with this paper. Gene symbol, HGNC gene symbol; GO Identifier, Gene Ontology unique numerical identifier; GO Term Name, Gene Ontology unique descriptive label; Evidence, evidence code used in annotation (IDA, Inferred from Direct Assay; IMP, Inferred from Mutant Phenotype) [42].

We focused our curation on human proteins; however, when experimental data was not available for human, model organisms were used (in particular mouse and *Saccharomyces cerevisiae*). These nonhuman annotations have been propagated to human proteins, and tagged with appropriate evidence codes according to their origin. As an example, the function of *S. cerevisiae* Atg2 (UniProt identifier: P53855) has been extensively studied, but experimental data are rather poor for the corresponding human homologs ATG2A and ATG2B (Q2TAZ0 and Q96BY7). Propagation of annotations from yeast to human via the Phylogenetic Annotation and INference Tool (PAINT) pipeline developed by the GOC [32,42,43] enabled the association of ‘autophagosome assembly’ (GO:0000045) and ‘autophagy of mitochondrion’ (GO:0000422) with the 2 human homologs (see Materials and Methods for details).

There are several different types of autophagy, which range from the relatively simple process of CMA involving only about 10 distinct proteins [8] to more complex processes, such as macroautophagy [9]. Research into these different processes is varied, with some key model organisms used predominantly to investigate specific types of autophagy (such as the use of yeast to investigate microautophagy), while other autophagy processes are investigated with a range of species (such as human, mouse, rat, fly, worm and yeast for mitophagy). Thus the manual annotations created reflect, to some extent, the scientific approaches used to investigate the various autophagy processes. Microautophagy research has almost exclusively been conducted in yeast, whereas this process is poorly characterized in mammals [6,7,44]. Consequently, as of March 2017, while 52 yeast proteins are associated with a microautophagy-related term, based on experimental data, the same is true for only 5 human proteins. In contrast, experimental data supports the association of a similar number of yeast and human proteins with mitophagy-related terms (33 and 43, respectively). This highlights the usefulness of capturing and integrating knowledge from nonhuman organisms.

### Quality control checks for GO annotation coverage

To evaluate the breadth of our annotation focus and to help detect any gaps in coverage, we compared our annotations with 7 other independent, complementary, knowledge-base resources that also have representations of autophagy: Reactome [45], PD-map [46], Autophagy Regulatory Network [47], iLIR database [48], Viralzone [49], Human Autophagy Database [50], and Autophagy Database [51]. Reactome has reciprocal links with GO, providing >98,000 annotations to the GOC dataset as of February 2017. UniProtKB has identifiers for all 67 human proteins in the Reactome macroautophagy pathway [52] and 135 proteins associated with autophagy in the PD-map [53] were downloaded. These were compared with the list of proteins annotated with the GO terms ‘autophagy’ (or one of its descendants) in the GOC database. Eight of the 67 proteins in the Reactome macroautophagy pathway were not associated with the GO term ‘macroautophagy’ (CHMP2A, CHMP2B, CHMP3, CHMP4C, CHMP6, CHMP7, TSC1 and TSC2; UniProt IDs O43633, Q9UQN3, Q9Y3E7, Q96CF2, Q96FZ7, Q8WUX9, Q92574 and P49815 respectively). These discrepancies were investigated, and literature-based knowledge was identified to support the association of all but one of these proteins (CHMP7) with a ‘macroautophagy’ GO term. A comparison between the list of proteins associated with autophagy in PD-map [46] with those associated with the autophagy GO term detected 81 discrepancies. This comparison led to the association of a further 53 proteins with autophagy-relevant GO terms. For example, CAMKK2 (Q96RR4) was associated in PD-map with the autophagy domain, but not with any autophagy GO terms. A focused PubMed search identifies that Sinha et al [54]. demonstrate that thyroid hormone-induced autophagy is mediated by PRKAA1/AMPK (Q13131), and that CAMKK2 is required for phosphorylation of PRKAA1. As PRKAA1/AMPK was shown to phosphorylate ULK1 (O75385) [54], leading to its mitochondrial recruitment and to initiation of mitophagy, the term ‘positive regulation of autophagy’ was associated with CAMKK2. For the remaining 28 discrepancies in the PD-map versus GO comparison, there was no literature to support a role of those proteins in autophagy and the PD-map group are reviewing this branch of their resource. Overall, a comparison of the GOC resource with 2 independent ones (Reactome and PD-map) led to the addition of GO annotations to autophagy-related terms for 60 proteins.

As for other resources, we verified that all proteins indicated as ‘core autophagy proteins’ by the Autophagy Regulatory Network [47] are associated with autophagy terms in GO [55]. We also found good overlap of human proteins annotated to GO autophagy terms and autophagy proteins listed in the Human Autophagy Database [50] and the Autophagy Database [51]. An incomplete overlap is very likely due to the different scope of those resources, which include proteins involved in not ‘strictly-autophagic’ processes such as apoptosis. Comparison with the autophagy-related database iLIR [48], listing LC3-interacting region-containing proteins, was slightly out of scope due to its inclusion of proteins that are targets of autophagy rather than active players in the process. Lastly, the ViralZone [49] resource includes details of autophagic processes from a virus perspective, such as ‘induction by virus of host autophagy’. ViralZone integrates with GO as well as UniProtKB [56] and should therefore be considered as a collaborative effort that partially overlaps with GO, rather than a resource for comparison.

A “guilt-by-association” approach was also used to investigate missing annotations. “Guilt-by-association” would predict that some of the proteins associated with the GO term ‘autophagosome’ might have a role in autophagosome assembly. Of the 90 reviewed human protein IDs associated with the GO term ‘autophagosome’, 80 were also associated with the GO term ‘autophagy’. A review of the literature supporting the association of the ‘autophagosome’ term with the remaining 10 proteins led to the association of ‘regulation of autophagy’ (or a more specific child term) with 5 of these proteins (WASHC1, HTT, TICAM1, FYCO1 and OSBPL7; A8K0Z3, P42858, Q8IUC6, Q9BQS8 and Q9BZF2 respectively). Two proteins were associated with the term ‘autophagosome’ because they are targets of autophagy (ORAI1, IL1B; Q96D31, P01584), but do not contribute to the macroautophagy process. Consequently, these protein records were revised and the ‘autophagosome’ GO annotations removed. The GO term ‘autophagosome’ is associated with 2 other gene products based on their homology to the murine *Washc1* gene by computational pipelines. These annotations have been retained, although these proteins (WASH2P, WASH3P; Q6VEQ5, C4AMC7) are listed in UniProtKB as pseudogenes. The remaining protein, RPN2 (P04844), is a component of the endoplasmic reticulum membrane that is also part of autophagosomes [57]. However, as this protein is quickly degraded following integration of the endoplasmic reticulum membrane into the autophagosomes, the GO term autophagosome was removed from this protein record [57].

Autophagy-related GO terms were also reviewed to check that these were associated with appropriate proteins. The majority of the 124 autophagy-related GO terms have been associated with at least one protein (Table S1), although 28 of these terms have not been used directly in an annotation. Some of these unused terms are highly descriptive child terms such as ‘negative regulation of macroautophagy by TORC1 signaling’; whereas others are parent terms, such as ‘Atg1/ULK1 kinase complex assembly’, which provide the opportunity to group annotations to their more specific child terms. The application of these autophagy-relevant GO terms appears to confirm reasonable annotation coverage of this domain.

### Cytoscape analysis

To create a limited autophagy interactome, 4 ‘core autophagy’ proteins [39] were selected to seed a human protein network using Cytoscape [58]. These proteins, AMBRA1, BECN1, PIK3C3 (yeast Vps34) and ULK1 (Q9C0C7, Q14457, Q8NEB9 and O75385), are targets of well-characterized post-translational modifications that lead to activation of autophagy. The resulting network includes 146 proteins and 530 interactions. GOlorize [59] and BinGO [60] analyses identified 600 GO biological process terms as enriched within this network, as well as 120 enriched cellular component terms and 67 molecular function terms (Table S2). With this many terms enriched, it is necessary to consider how informative the identified terms are, as many provide very little specific information about the processes, functions or locations associated with this network. For example, ‘organelle assembly’ is highly enriched in this dataset, with 24 proteins in the network associated with this term, but a similarly enriched term is ‘autophagosome assembly,’ with 14 proteins associated. The latter term is more informative (than the former) because it names the specific complex assembled by 10% of the proteins in the network. The BinGO analysis demonstrated that the proteins associated with these seed proteins have roles in the various processes that lead to autophagy, such as response to stress and nutrient levels, signaling and intracellular transport, as well as the downstream impact of macromolecule metabolism (Table 2). In addition, the enriched terms also reflect the importance of autophagy in many biological pathways, including development, immunity and cell cycle [8,9,61,62] (Table 2).

**Table 2.** Comparison of functional analysis of the protein network associated with 4 autophagy-relevant proteins using Gene Ontology (full list of enriched terms in Table S2).

		February 2017 dataset, X = 137 proteins, N = 19945			January 2014 dataset, X = 128 proteins, N = 31060		
GO Identifier	GO Term Name	corrected p-value	x	n	corrected p-value	x	n
**Autophagy related Biological Process terms **							
6914	autophagy	3.27E-48	45	252	2.59E-34	29	186
10506	regulation of autophagy	6.26E-37	39	283	2.58E-21	18	105
16236	macroautophagy	3.53E-38	33	145	3.36E-21	15	53
16241	regulation of macroautophagy	1.05E-18	20	138	1.44E-04	4	31
45	autophagosome assembly	4.19E-19	14	37	1.04E-20	14	44
2000785	regulation of autophagosome assembly	1.60E-07	7	36	#N/A	#N/A	#N/A
422	autophagy of mitochondrion	3.45E-11	9	30	#N/A	#N/A	#N/A
1903146	regulation of autophagy of mitochondrion	2.76E-06	7	56	#N/A	#N/A	#N/A
32006	regulation of TOR signaling	3.09E-06	8	85	6.28E-06	6	60
**Biological Process > 20% interactome proteins enriched**							
6996	organelle organization	1.26E-17	68	3209	8.93E-13	50	3718
33043	regulation of organelle organization	2.87E-10	33	1191	2.44E-05	17	1011
6950	response to stress	1.70E-10	57	3271	1.48E-06	44	4628
80134	regulation of response to stress	2.98E-06	28	1351	3.52E-03	14	1169
31667	response to nutrient levels	6.26E-12	22	403	2.25E-03	9	489
7154	cell communication	1.73E-04	61	5442	2.69E-05	54	7137
10646	regulation of cell communication	1.34E-06	47	3108	1.79E-05	34	3395
7165	signal transduction	1.95E-02	49	4973	1.52E-05	51	6410
9966	regulation of signal transduction	5.33E-07	45	2801	1.81E-06	34	3029
6915	apoptotic process	2.09E-05	21	908	3.09E-09	25	1200
42981	regulation of apoptotic process	1.02E-07	32	1450	2.87E-08	29	1853
48856	anatomical structure development	2.64E-02	49	5069	3.82E-03	46	6992
48731	system development	1.63E-02	43	4154	7.89E-03	38	5642
48468	cell development	4.88E-02	18	1458	3.47E-03	20	2081
7049	cell cycle	1.11E-09	34	1334	4.34E-10	31	1730
51726	regulation of cell cycle	5.57E-09	29	1048	2.87E-08	23	1158
16192	vesicle-mediated transport	7.40E-07	34	1769	1.21E-04	20	1564
60627	regulation of vesicle-mediated transport	1.19E-05	15	453	#N/A	#N/A	#N/A
2376	immune system process	1.77E-03	33	2513	7.76E-06	31	2783
2682	regulation of immune system process	1.52E-03	23	1447	1.11E-02	16	1681
43170	macromolecule metabolic process	9.01E-03	71	7709	8.04E-04	66	10738
60255	regulation of macromolecule metabolic process	4.57E-04	64	6015	7.60E-04	51	7470
**Autophagy related Cellular Component terms**		**February 2017 dataset, X = 133 proteins, N = 18984**			**January 2014 dataset, X = 132 proteins, N = 33766**		
5776	autophagosome	8.25E-34	26	84	1.09E-23	16	55
421	autophagosome membrane	3.01E-19	13	29	4.28E-14	9	27
407	phagophore assembly site	6.80E-21	14	30	9.89E-15	10	36
34045	phagophore assembly site membrane	7.72E-13	8	14	2.68E-12	7	15
5764	lysosome	1.92E-02	11	633	9.77E-03	8	600
**Cellular Component terms > 20% interactome proteins enriched **							
5634	Nucleus	3.40E-02	63	7090	#N/A	#N/A	#N/A
31410	cytoplasmic vesicle	5.05E-11	46	2156	4.97E-10	31	1978
5856	cytoskeleton	3.48E-06	35	2005	4.21E-06	32	3151

A protein network associated with 4 seed proteins (AMBRA1, BECN1, PIK3C3, ULK1) was created using Cytoscape. GO enrichment analysis was then conducted using the BinGO plugin [60] within Cytoscape [58] using either the 2017 or the 2014 GOC annotation files. The selected significantly enriched biological process and cellular component terms identified in the 2 analyses are either autophagy-relevant or represent annotations associated with over 20% of the proteins analyzed. In addition, the associated-process or regulation term data is also included in the table. n indicates the number of protein IDs associated with the GO term, x is the number of protein IDs in both the submitted list and associated with the GO term. #N/A indicates GO terms that are not significantly enriched in the analysis using the 2014 GOC files, but are enriched in the 2017 analysis. In the 2017 and 2014 files the total number of human proteins associated with the biological process domain is: 19,945 and 31,060 (respectively). The total number of human proteins associated with the cellular component domain is: 18,984 and 33,766 (2017 and 2014 files respectively). Note that the number of proteins associated with the human proteome in the GOC annotation files is reduced due to the removal of redundant protein identifiers. Also the label of GO:0000422 (currently ‘autophagy of mitochondrion’, previously ‘mitophagy’), and GO:1903146 (currently ‘regulation of autophagy of mitochondrion’, previously ‘regulation of mitophagy’) have been modified recently to be more consistent with literature usage. For space constraints, GO IDs are simplified here, e.g. GO ID 6914 is GO:0006914.

A comparison of the GO terms enriched in this dataset using the GO terms available in January 2014 vs. February 2017 identified that there have been considerable changes in the number of human proteins associated with the GO autophagy domain (Table 2). In January 2014 there were only 186 proteins associated with the ‘autophagy’ terms. That number increased to 252 proteins by February 2017. There was also a doubling of proteins associated with the ‘regulation of autophagy’, i.e. 105 in January 2014 and 238 in February 2017. This increase in the number of proteins associated with these terms is reflected in the number of proteins in the autophagy network; 65 proteins, of the 146 proteins in this interactome, are now associated with an autophagy-related GO term, whereas in 2014 there were only 35 proteins associated with these terms (examples of proteins now associated with autophagy-relevant terms are indicated by blue or red arrows in Figure 5). The change in the number of proteins associated with autophagy-related GO terms is not simply a reflection of an increase in the number of autophagy-related annotations. This annotation project also reviewed all proteins which had been associated with both ‘regulation of autophagy’ and ‘autophagy’ terms. Using the new autophagy term definitions, that describe the start and finish of the autophagy processes, the appropriate GO term could be more accurately associated with these proteins. As mentioned earlier, in 2014 ULK1 was associated with both ‘regulation of autophagy’ and ‘autophagy’ GO terms (black arrow, Figure 5A). Since we have defined the start of macroautophagy to be the formation of the Atg1/ULK1 complex, we have considered the action of ULK1 kinase to be integral to this process. Therefore, we consider its activity to have a direct role in autophagy, and the ‘regulation of autophagy’ annotations associated with ULK1 were reviewed and changed to ‘autophagy’ or child terms.

The functional analysis of this autophagosome assembly interactome also demonstrates that the *P* values associated with the significantly enriched GO terms have decreased, following the additional annotations created during the past 3 y. Furthermore, the role of this interactome in ‘autophagy of mitochondrion’ can now be identified, whereas using the 2014 annotation files this process was not significantly enriched.

### Gene set enrichment analysis results

Many studies have suggested that dysregulated autophagy is associated with the development of Parkinson disease [63–65]. To illustrate the importance of our work towards improved interpretation of high-throughput biomedical data, we reanalyzed a previously published Parkinson disease dataset [4] using the most recent GO data, taking advantage of the added wealth and depth of knowledge provided by our autophagy improvements. The dataset [4] contains blood transcriptomes from newly diagnosed, drug-naïve Parkinson disease patients and from age- and gender-matched controls, and was chosen because, to date, it is the largest published transcriptomic profiling of untreated PD patients (containing 40 patients), allowing for a better evaluation of disease mechanisms not yet confounded by pharmacological treatment. GO annotations used at the time of the original publication predated the autophagy ontology and curation effort described here (as well as others about cell death and signaling). Full results from our reanalysis are shown in Tables S3, S4 and S5, while a summary list of autophagy-relevant results from the same Gene Set Enrichment Analysis (GSEA) is shown in Table 3.

**Table 3.** Summary of autophagy-relevant GO gene set enrichment analysis (GSEA) results from a dataset of drug-naïve, sporadic Parkinson disease patients.

GO Identifier	GO Term Name
GO:0005776	autophagosome
GO:0000421	autophagosome membrane
GO:0000407	phagophore assembly site
GO:0034045	phagophore assembly site membrane
GO:0006914	autophagy
GO:1905037	autophagosome organization
GO:0044804	autophagy of nucleus
GO:0016236	macroautophagy
GO:1903008	organelle disassembly
GO:0061726	mitochondrion disassembly

Analysis was performed as detailed in Materials and Methods. Gene sets relevant to autophagy and enriched in patients vs. controls at nominal *P* value <5% were selected from Tables S3, S4 and S5, and sorted by false discovery rate (smallest first). Note that the label of GO:0044804 (currently ‘autophagy of nucleus’) was previously ‘nucleophagy’, and has been modified recently to be more consistent with literature usage.

Cellular component GO gene sets enriched in patients vs. controls (Table S3) highlight the presence of transcripts related to autophagosomal structures. The following gene sets are among the top 5 significant results: ‘autophagosome’, ‘autophagosome membrane’, ‘phagophore assembly site’, and ‘phagophore assembly site membrane’ (Table 3). For the top significant gene set in Table 3 (‘autophagosome’), which is also the top result of our GSEA overall (see Tables S3, S4 and S5), the subset of genes from the gene set that contribute most to the enrichment result are listed in Table S6.

Among molecular function GO gene sets, the top result GO:0032266 ‘phosphatidylinositol-3-phosphate binding’ (Table S4) is also consistent with the formation of autophagosomes. Axe et al [66]. follow the dynamics of several phosphatidylinositol 3-phosphate (PtdIns3P)-binding proteins during amino-acid starvation and induction of autophagy, and show that at least some autophagosomes are formed in a starvation-induced, PtdIns3P-enriched membrane compartment, called the omegasome, dynamically connected to the endoplasmic reticulum. PtdIns3P may play a role in providing localization clues and facilitating the fusion step at the final stage of autophagosome formation [66]. More recently, it has been confirmed that PtdIns3P-binding proteins participate in signaling events that lead to autophagosome assembly and activity [67]. These findings are in line with the presence of 5 other GO gene sets related to phosphatidylinositol binding in our results (Table S4) (‘phosphatidylinositol-5-phosphate binding’, ‘phosphatidylinositol-4-phosphate binding’, ‘phosphatidylinositol-3,4,5-trisphosphate binding, ‘phosphatidylinositol-3,5-bisphosphate binding’ and ‘phosphatidylinositol-3,4-bisphosphate binding’), as well as enrichment of the term ‘1-phosphatidylinositol-3-kinase activity’. Together, these enriched molecular functions support the involvement of autophagy in Parkinson disease.

Biological process gene sets related to autophagy are found among results (‘autophagy’, ‘autophagosome organization’, ‘autophagy of nucleus’, ‘macroautophagy’, ‘organelle disassembly’ and ‘mitochondrion disassembly’) (Table 3, Table S5), though with limited significance values. GO gene sets related to cholesterol transport, leukocyte activation and development are among processes overrepresented in patients vs. controls; this is expected given the source of the samples (blood transcriptomes). However, notably, the top significant gene set (‘toll-like receptor 4 signaling pathway’) refers to a cascade of events known to regulate autophagy. Toll-Like Receptor (TLR) signaling in general links autophagy to innate immunity [34], with TLR4 signaling shown to induce autophagy via BECN1 [38]. TLR4 signaling has also been shown to be involved in autophagy cell protection against ethanol toxicity in mouse astrocytes and neurons [68], and the term ‘cellular response to ethanol’ is found further down the list (Table S5). TLR signaling has indeed been associated with Parkinson disease [69]. Starvation, and particularly nitrogen starvation, induces autophagy, via the TOR signaling pathway [35], and GO terms describing the cellular response to nitrogen starvation, ‘cellular response to nitrogen compound’ and ‘cellular response to nitrogen levels’, are present among the top significant gene sets. Furthermore, autophagy regulates macrophage foam cell formation and function [70,71] and terms relevant to these processes also appear among enrichment results, such as ‘regulation of macrophage-derived foam cell differentiation’ and its positive and negative children [70], or ‘regulation of cholesterol storage’ [71] (Table S5).

### Discussion

Many different resources now provide descriptions of the functions and locations of gene products and the cellular and molecular pathways that are essential for life. However, only a small percentage of the available biological literature is currently represented in computer accessible resources. Comprehensive annotation of the human and model-organism genomes is an ongoing task that is far from complete. Providing gene product annotations using GO terms requires considerable interaction between the ontology developers, expert scientists and the biocurators creating the annotations. This project aimed to focus on the use of GO to describe the cellular pathways associated with autophagy. Primary literature identified for gene curation was the major source of data to support both the expansion of the ontology to describe this domain and the creation of gene product annotations. Three sources were used to identify proteins with a role in autophagy, the literature, Reactome [45] and PD-Map [46].

#### An improved ontological representation of autophagy

Our work has resulted in improvement of the ontological representation of autophagy in GO. We have systematically classified the types of autophagy by either mechanism or target. In many cases, this method has allowed us to create equivalence axiom-based definitions of terms that are used to computationally classify the terms. We have also used our focused effort to interrelate terms from the 3 parts of GO, biological process, molecular function and cellular component. By precisely defining terms, we could refine the representation of the start and end of many autophagy processes. Defining the start and end of processes results in more precise annotation of gene products whose function has been elucidated. If the gene products lie within the subprocesses or functions that we have defined as parts of an autophagic process, then they can be annotated directly with those terms. If the functions of the gene products lie outside of our defined parts but impinge upon the execution of autophagy by controlling the internal parts, then those gene products regulate autophagy. However, in some cases proteins play dual roles, and might therefore be tagged with both process and regulation terms. As an example, the human autophagy receptor CALCOCO2 independently regulates targeting of bacteria to autophagosomes and promotes pathogen-containing autophagosome maturation by interacting with the Atg8-family homologs [72]. Our work leaves the representation as up-to-date as possible now, but allows for changes as the field progresses by adding new mechanistic or target-based subprocesses and giving us the ability to easily modify existing equivalence axioms as they are required for necessity and sufficiency. For example, if warranted, we could add a new class to represent noncanonical (ULK1-dependent) macroautophagy [73], and easily examine the existing terms to see if they are specific enough to be renamed ‘canonical macroautophagy’ or if they represent a generic form and require additional terms to represent ‘canonical autophagy’. The current ontology structure also allows for the straightforward addition of more types of selective autophagy if and as they are required for annotation. Our approach also allows for refinement of definition axioms and asserted relationships as knowledge is accumulated.

#### Improved curation of autophagy players and its effect on data analysis

This autophagy-focused annotation project has led to a more complete representation of autophagy in the GO database, providing over 1,200 GO annotations describing the role of 474 human proteins in autophagy. This has been achieved following a review of recent literature and a comparison of GO annotations with those provided by PD-map and Reactome. Comprehensive GO annotation of this domain was also demonstrated by verifying that core autophagy proteins listed in 5 autophagy-specific databases [47–51] are associated with an autophagy-relevant GO term. In addition, good depth of annotation was achieved, with highly specific GO terms associated with many proteins; consequently, two-thirds of autophagy-related terms are associated with at least one human protein. Furthermore, many of the experimentally supported annotations have been propagated to orthologous proteins across over 100 different species.

The impact of this project can be visualized by overlaying enriched GO terms onto the in silico interaction network associated with 4 of the key autophagosome assembly proteins (Figure 5). This shows that 65 proteins in this interactome are now associated with an autophagy term, whereas in 2014 there were only 35 proteins. Our in silico network was seeded with only 4 proteins because many autophagy-associated proteins have multiple roles and therefore create very complex networks. For example, Behrends et al [74]. constructs an experimentally-supported autophagy interaction network (AIN) with 409 interactors. In contrast, if the in silico analysis is seeded with Behrends’ 65 bait proteins, the network extends to more than 2700 proteins, including 400 of the AIN proteins. The impact of continued annotation by GO biocurators is also demonstrated by reanalysis, using g:profiler [75], of the 409 AIN proteins [74] (data not shown). In 2010 Behrends et al [74]. demonstrated that between 4 and 7% of this network was associated with one of the following GO terms: ‘vesicle transport’, ‘proteolysis’, ‘signal transduction’ and ‘phosphorylation’ (with *P* values of 10^−5^ to 10^−9^). Since then there has been a 3-fold increase in the number of annotations associated with these proteins. Consequently, in July 2017 25% of proteins in this network were identified as associated with each of those terms (with *P* values of 10^−6^ to 10^−13^), with 44% associated with ‘transport’ (*P* value 10^−12^). In addition, the most significantly enriched term in the 2017 analysis was ‘macroautophagy’, with 60 proteins associated with this term, and a *P* value of 10^−40^.

However, there are still many proteins in both of these interactomes not annotated to an autophagy-relevant biological process term. Additional literature searches may support the creation of missing autophagy annotations, or suggest that these additional interacting proteins reflect the multifunctional roles of the 4 seed proteins in this network. Alternatively, identification of the role of these proteins in autophagy may require further experimental investigation.

Our reanalysis of gene set enrichment in a cohort of newly diagnosed, drug-free Parkinson disease patients highlighted the presence of transcripts that are known to play a role in autophagy. Notably, autophagy is not detected as a dysregulated pathway in a recent analysis of the same dataset [76] carried out before the completion of this work. Despite the statistical limitations of results coming from a single dataset, findings from our reanalysis pointing to the interplay of autophagy and Parkinson disease are confirmed in recent literature [63–65].

#### Autophagy through evolution and taxonomic constraints

Much of our understanding of the molecular mechanisms mediating macroautophagy results from genetic studies in yeast, in which more than 40 autophagy-related (*ATG*) genes have been identified [77]. Most of these genes are well conserved across eukaryotes including human and are essential for the formation and expansion of autophagosomes in most of these organisms [5,25,26]. Many studies have also been performed in human as well as model organisms such as mouse, and to a lesser extent in *Drosophila melanogaster, Caenorhabditis elegans* or *Arabidopsis thaliana*. The high conservation of the genes and mechanisms involved in macroautophagy allows for the propagation of information coming from experimental data obtained from one eukaryotic model organism to most other eukaryotes. Validated procedures for propagating annotations, such as using PAINT, the Ensembl Compara pipeline and curating to ISS evidence, led to a significant increase in the coverage of autophagy annotation. However, due to some exceptions, caution is required in propagating data across some species. For example, in methylotrophic yeast such as *Pichia pastoris*, the utilization of methanol as a carbon source requires peroxisomes. However, peroxisomes are not necessary when these cells grow on other carbon sources such as glucose or ethanol and, in fact, these organelles are then degraded via pexophagy. These yeast express pexophagy-specific genes, including *ATG28* and the peroxisome receptor for pexophagy *ATG30* [78,79]. Despite the many experimental data available for *Pichia pastoris*, its unusual metabolism prevents this species from being considered as a model for the annotation of autophagic processes in other eukaryotes. Another tricky situation occurs in algal genomes where autophagy-related (ATG) proteins are conserved in green algae, but have not been detected in red algal genomes. TOR signaling is conserved in both red and green algae [80] and is a major regulator of autophagy in eukaryotes [26]. However, as autophagy does not occur in red algae, the ‘regulation of autophagy’ annotations associated with TOR and its interacting proteins cannot be propagated to these species.

Moreover, the evolution of these proteins correlates with the increased complexity of organisms in the course of evolution. Yeast Atg8 and mammalian homologs of this, ubiquitin-like protein, are key players in macroautophagy [26], mediating membrane tethering and fusion. It is covalently bound to phosphatidylethanolamine (PE) on the phagophore membrane and remains bound through the maturation process of the autophagosome. Only one gene coding for an Atg8 protein has been identified in yeast, but the family is expanded in animals where 2 Atg8-like subfamilies have been described: the MAP1LC3/LC3 (microtubule associated protein 1 light chain 3) subfamily including MAP1LC3A, B, B2 and C) and the GABARAP (GABA type A receptor-associated protein) subfamily including GABARAP, GABARAPL1, GABARAPL2 and the pseudogene GABARAPL3. The LC3 subfamily is involved in elongation of the preautophagosome membrane, whereas the GABARAP subfamily is essential for a later stage in autophagosome maturation [81]. Furthermore, the number of members in each subfamily varies from one lineage to the other, reflecting duplication and loss events during evolution [82]. Both subfamilies are involved in macroautophagy, but the members of the GABARAP subfamily play additional roles in diverse membrane trafficking processes, such as transport from the Golgi to the plasma membrane, endoplasmic reticulum to Golgi transport and intra-Golgi transport.

#### Perspectives

The work described here is not meant to be an endpoint, but rather a starting point, in providing a good representation of autophagy in a comprehensive resource. It will allow continued capture of the new knowledge in this domain in a comprehensive, interoperable and computer-readable way. For example, additional types of selective autophagy, such as zymophagy, can easily be added to the ontology as they are studied and the terms are required for annotation [83]. The incorporation of computable definitions for many autophagy-related GO terms, and the alignment of autophagy representation in GO with other bioinformatics resources, such as Reactome [45] and PD-map [46], contributes to optimal database integration. Specifically, the improvement of the ontology and increase of annotation coverage will be instrumental in the interpretation of gene enrichment analysis. In addition, our annotations will improve the interpretation of autophagy networks, such as those created by Behrends et al [74]. by providing additional insights into the functional role of the proteins identified as coimmunoprecipitated with known or predicted autophagy proteins.

Autophagy plays an important role in the response to starvation [35] and in development, differentiation, aging and cell death [61], but also in the defense response against intracellular pathogens [29]. Moreover, defects in autophagy are also related to an increasing number of diseases, from degenerative diseases to cancer [9–18]. Medical research will therefore benefit from an accurate ontological representation of autophagy, and ongoing update of autophagy knowledge in the GO resource enables better capture of recent findings in the field. Our improved GO resource for autophagy research is not only useful for human, but for most eukaryotic organisms, and can also highlight some exceptions to canonical mechanisms as mentioned earlier. Finally, the value that our work brings to the ontology and annotation knowledgebase underlines the usefulness of focusing efforts on specific cellular processes, and might serve as a paradigm to improve the representation of many other areas of biology.

Our work established solid foundations to enable continued incorporation of new findings in the field. For example, experimental knowledge of full details of the end of autophagic processes is still incomplete. As new data emerge, the GO resource will be able to capture them and make them available to the community, building upon the effort described here. To this aim, the GO Consortium always welcomes feedback from the scientific community. We warmly encourage autophagy researchers to contact us if they wish to suggest additions or changes to the ontology and/or publications describing novel characteristics of proteins or RNAs involved in autophagy [84].

### Materials and methods

#### Ontology building

Ontology development was accomplished by our working group consisting of Gene Ontology (GO) developers and biocurators, and was led as an extension of work by the UCL functional annotation team focusing on Parkinson disease [85]. A strategy to refine the GO resource by phylogenetic annotation [32] was also applied. To ensure an accurate representation of the processes, we reached out to external experts in several ways, e.g. by contacting autophagy researchers at the same institution as working group members and by presenting the GO autophagy project at meetings, workshops and conferences [86]. The working group met at regular monthly intervals to update progress, discuss issues, and plan and assign upcoming tasks. To keep track of ontology development needs, record discussions and allow other members of the GOC to contribute, we used a designated issue tracker on the GO GitHub repository [87], and tagged tickets with an ‘autophagy’ label. Ontology editors implemented changes using the ontology-editing tools Protégé [33], OBO-Edit [88] or TermGenie [21].

#### Annotation

##### Identifying experimental data to annotate

The PubMed database [89] was used to locate recent papers reviewing the literature for microautophagy, chaperone-mediated autophagy or mitophagy. These reviews were then used to identify lists of proteins involved in these processes. Subsequently, searches of PubMed, with each individual gene symbol, name or synonym and additional filters, were conducted to provide a comprehensive coverage of the role of these proteins with respect to autophagy. The following filters were applied to relevant gene-symbol and name searches: ‘AND microautophagy’, ‘AND mitophagy’, or ‘AND chaperone-mediated autophagy’. The selection of papers to curate was then based on whether: 1) they contained experimental data; 2) new information would be added to the current GO annotation data associated with the protein; 3) it was possible to identify the species the protein or expression construct was derived from. Only papers that met all 3 criteria were curated. The choice of papers curated was, therefore, influenced by the information captured previously (i.e. papers already annotated with existing autophagy terms were excluded). While human autophagy players are the primary focus of this project, mouse and *Saccharomyces cerevisiae* gene products were also curated where information was available.

##### Gene Ontology annotation – manual curation process

Manual GO annotation was performed essentially as described by Patel et al [90]. The most specific GO terms, representing the experimental data presented in each paper, were identified using the AmiGO or QuickGO browsers [23,91,92] and a consistent annotation approach was used [2]. The type of experimental data reported in the paper guides the selection of evidence codes associated with each annotation [2,42]. Guidelines for the use and interpretation of autophagy assays are used to guide annotations [26]. Most of these annotations will be transferred automatically to orthologous proteins from other species by the Ensembl Compara pipeline [93], and tagged with the IEA (Inferred from Electronic Annotation) evidence code. However, on inspection of many protein annotation records, it was noted that there were no extant electronically-generated annotations. In these cases, in order to complete the manual annotation process, orthologous human, mouse and rat proteins were identified using the HUGO Gene Nomenclature Committee ortholog prediction tool (HCOP) [94], and GO annotations with experimental evidence codes were transferred as previously described [90]. These annotations were tagged with the ISS (Inferred from Sequence Similarity) evidence code.

Autophagy-related annotations have also been propagated to other species using the PAINT tool developed by the GOC and applying the IBA (Inferred from Biological Ancestor) evidence code [43]. PAINT uses a tree-based approach to manually annotate homologs and paralogs based on PANTHER family predictions, integrating GO annotations from evolutionarily related genes across about 100 different organisms [95–97].

To provide information about the current GO annotations the QuickGO browser [91,92] was used to downloaded all the annotations (and annotation statistics) associated with the autophagy-related terms (using the ‘GO Identifier’ filter option with the GO IDs listed Table S1, and selecting the ‘exact match’ option). The number of proteins associated with each autophagy-related GO term was listed in the statistics download file ‘goid’ tab, following the application of the ‘Evidence’ filter ‘Manual All’ (this provides a list of all annotations supported by one of the following evidence codes: IMP, IGI, IPI, IDA, IEP, EXP, ISS, TAS, NAS, ND, IC, RCA, IBA, IBD, IKR, IRD, ISA, ISM, ISO, IGC [42]). To extract this equivalent data for the human proteome, the ‘Taxon’ filter ‘9606 human’ was selected.

A breakdown of the source and evidence supporting each human protein annotation was extracted using the QuickGO browser [91,92] filtered to retrieve only autophagy-related terms associated with human protein IDs. As the downloaded file included both nonreviewed and reviewed UniProt IDs, the UniProt Retrieve/ID mapping tool [98] was used to identify only the reviewed IDs. The 474 reviewed human protein IDs were then included in the ‘Gene Product ID’ QuickGO filter and used, along with the autophagy-related GO IDs in the ‘GO Identifier’ filter option. The resulting annotations and statistics data was then downloaded so that the number and source of annotations associated with these autophagy-related GO terms could be calculated (from the ‘evidence’ and ‘assigned’ tabs in the statistics downloaded file). The searches occurred on March 8, 2017.

#### Functional analysis

##### Cytoscape

Four human proteins (symbol and UniProtKB ID, respectively: AMBRA1, Q9C0C7; BECN1, Q14457; PIK3C3, Q8NEB9; ULK1, O75385) were used to seed a network of human proteins constructed using Cytoscape [58] version 3.3.0 (March 1, 2017). The following parameters were applied: Hypergeometric test, Benjamini & Hochberg False Discovery Rate correction, significance level 0.05: the human proteome was used as the reference set. The network view was modified using the following options: NetworkAnalyzer: ‘map nodes size’, degree, low values to small sizes; edit, ‘remove duplicate edges’ and selectively removed all edges describing identical protein binding interactions; layout, ‘prefuse force directed’, all nodes. The GO terms associated with this network were then identified using GOlorize [59] with the BinGO plugin [60] within the Cytoscape tool, and including the GO term ontology [99] (February 10, 2017) and either the January 20, 2014 (gene_association.goa_human.129) or February 13, 2017 gene association files [100] (the latter required combining 4 files: goa_human.gaf.165, goa_human_isoform.gaf.165, goa_human_complex.gaf.165, goa_human_rna.gaf.165). Enriched GO terms with fewer than 3 protein associated were removed from the result tables.

##### Gene Set Enrichment Analysis

The Gene Set Enrichment Analysis (GSEA) desktop tool v2.2.3 was downloaded from the Broad Institute website [101–103] GSEA was run on the normalized, unfiltered microarray dataset from Calligaris et al [4]., as suggested in the tool's implementation. We used a collection of Gene Ontology (GO) gene sets provided on request by the Molecular Signatures Database (MSigDB) staff, according to the procedure used to generate the GO C5 collection for the current (v5.2) release of the MSigDB database, with the only difference being newer versions of the sources: (1) gene2go (downloaded on September 27, 2016 from the NCBI ftp server) and (2) go-basic.obo (downloaded on September 27, 2016 from GO). The MSigDB documentation [104] outlines the procedure. GSEA was performed separately on each of the C5 subcollections (biological process, molecular function and cellular component), using the default setting, except for excluding gene sets with fewer than 5 genes. The array type was indicated as HG − U133A 2.0. All files used to perform the GSEA analysis are available as supplemental material (Files S1-4, Table S7).

## Supplementary Material

supp_data_1415189.zip
