# Supplementary material for: Exploring autophagy with Gene Ontology
Source: Autophagy. 2018 Feb 17;14(3):419–36. doi: 10.1080/15548627.2017.1415189 (PMC5915032; doi:10.1080/15548627.2017.1415189)
Supplement: supp_data_1415189.zip [file kaup-14-03-1415189-s001.zip › supp_data_1415189/supp_data_1415189_s01.docx]

**File S1.** Biological process GO gene set used to perform Gene Set Enrichment Analysis. Analysis was performed as detailed in Materials and Methods.

GO_REGULATION_OF_CARDIAC_MUSCLE_CONTRACTION_BY_CALCIUM_ION_SIGNALING Any process that modulates the frequency, rate or extent of cardiac muscle contraction by changing the calcium ion signals that trigger contraction. CACNA1C CALM2 HDAC4 CASQ2 ATP2A2 GSTM2 ATP1A2 SLC8A1 CALM1 RYR2 GSTO1 FKBP1B CAMK2D CLIC2 SLC9A1 PRKACA PLN ATP1B1 DMD ANK2 HRC CALM3

GO_RESPONSE_TO_MOLECULE_OF_BACTERIAL_ORIGIN Any process that results in a change in state or activity of an organism (in terms of movement, secretion, enzyme production, gene expression, etc.) as a result of a stimulus by molecules of bacterial origin such as peptides derived from bacterial flagellin. VCAM1 GSTP1 XBP1 CNR2 UGT1A1 PTGS2 DUSP10 IDO1 MAPK3 PALM3 HAVCR2 MTA1 TNFRSF8 SPON2 LILRB2 IL12RB2 DCN GCH1 CCL20 TNFRSF25 CMPK2 TNFRSF1A AICDA REN RIPK2 IRAK3 PTPN22 ALAD TFPI RELT CYP1A2 MAPKAPK2 CXCL2 JUN ELANE CD86 CXCL1 CNR1 CSF3 ADAM9 CXCL13 CD14 JUNB CD40 KLRK1 SELP TRIB1 CCL5 GNG12 LY96 TNFRSF4 STAR NGFR MPO F2R C4B ABCA1 AXL FOXP1 SRR SELE MEF2C FASLG NR1H3 SPARC RELA HNRNPA0 CD180 MAOB ZC3H12A PPARGC1A IL12A IRAK1 TICAM2 PTGIR CASP9 ABCC8 ZFP36 LGALS9 PPARD PELI1 SCARB1 CCL3 S100A14 CSF2 PTAFR IRF5 TLR5 CSF2RB FZD5 PTGER1 TREM2 EPO HSPD1 TBXA2R FMO1 ABR TNFRSF21 TLR1 MGST1 RPS6KA3 TRIM6 TNFRSF11A TNFRSF1B PLCG2 LITAF TNFRSF10B HCK ACP5 IFNG JUND FGF10 CCR7 ABL1 CCRN4L TNFRSF10D TJP1 GNRH1 TNF ATP4B TNFRSF10C TNFAIP3 IFNAR1 IL13 SIRT2 MTDH C19orf29 CEBPB CXCL10 CYP1A1 P2RX7 CDC73 NOS3 PRKCE WNT5A LTBR JAK2 TLR2 IL1B OTUD5 MAPKAPK3 NFKBIB HDAC5 IL18BP ADAMTS13 UPF1 STAP1 CXCL16 NFKBIA PRPF8 TNIP2 MAPK8 PLSCR4 MAPK1 SBNO2 IL12B RPS6KB1 CASP3 IRG1 CARD9 PYCARD PTGER4 FOS DAB2IP NLRP3 PPBP TNFRSF11B HMGB2 B2M NOD2 CXCL3 SNCA TNFRSF10A MRC1 ASS1 TIRAP ALPL PF4 TRAF6 COMT SLPI HDAC1 ARG1 LYN PAF1 ICAM1 CNP TGFB1 TNIP3 PLSCR3 MAPK14 IRF3 MGST2 ADAM17 FAS S100A7 SRC THBD CEBPE CCR5 LOXL1 BCL10 CHUK NFKB2 PCK1 CYP27B1 CDK19 PTGER2 HSF1 IL23R NR1D1 C5AR1 CD36 LBP CXCL9 SHPK IRF8 AKT1 PTGFR SLC11A1 BDKRB1 PDE4B CPS1 IL10 CD80 KCNJ8 CD27 NFKB1 RARA PABPN1 TNFRSF18 SSC5D IL18 EDNRB SERPINE1 PDE4D CD96 NFKBIL1 PRDX3 IL6 CD6 TLR6 ANKRD1 PENK CCL2 TAC1 ADM EDN1 IL24 LTA TLR9 CXCL5 IL8 AKAP8 PTGES CASP8 CXCL6 TSPO OPRM1 TNFSF4 TNFRSF6B BCR SELS FER APOB IRAK2 CX3CR1 ERBB2IP CTSG TICAM1 ADH5 CASP1 CD24 LILRB1 TH GFI1 PCK2 NR1H4 HDAC2 NOTCH1 NOS2 PF4V1 GJB6 TIMP4 IL10RA CTR9 MALT1 OPRK1 KLRC4-KLRK1 S100A8 HAMP TLR4 CXCL11 TNFRSF14 SCGB1A1 LIAS AKIRIN2 TNFRSF9 CITED1

GO_NEGATIVE_REGULATION_OF_CELL_JUNCTION_ASSEMBLY Any process that stops, prevents or reduces the frequency, rate or extent of cell junction assembly. THBS1 TNF PTEN ACE PHLDB2 CORO1C ARHGAP6 IKBKB ROCK2 SRC APOD ITGB1BP1 EPB49 MMP14 ROCK1 DLC1 BCAS3 ACVRL1 CLASP2 RCC2

GO_COFACTOR_CATABOLIC_PROCESS The chemical reactions and pathways resulting in the breakdown of a cofactor, a substance that is required for the activity of an enzyme or other protein. ACAT1 CBR3 ACOT7 ASPDH NUDT12 NUDT7 BLVRA BLVRB MTHFS ALDH1L1 CYP4F11 VCP UGT1A4 UGT1A1 CYP4F2 HMOX1 PDXP ALDH1L2 AMBP HMOX2

GO_CELLULAR_MACROMOLECULE_LOCALIZATION Any process in which a macromolecule is transported to, and/or maintained in, a specific location at the level of a cell. Localization at the cellular level encompasses movement within the cell, from within the cell to the cell surface, or from one location to another at the surface of a cell. AP1G1 STX5 TMEM48 CEP68 WRB VPS26B POM121 LRWD1 NRXN2 SRP54 PES1 PEX1 FGF7 NUP93 ATG4D AP2B1 SEC24D CACNB3 SYTL2 TSNARE1 ID1 RANBP6 ARF4 AP2S1 TIMM22 ACTN2 EGF SH3BP4 WASH3P CEP72 RIMS1 HSPA9 TOMM7 TBC1D2B CROCC EHD3 SPO11 STX18 SSNA1 ADIPOQ GSG2 RPS4X HSP90B1 FUT10 MAGI2 EMP2 BCL6 UFD1L NFKBIE FAM83H ATP6V1D GIPC1 RPS4Y1 TRAM1 SYNGAP1 PMPCA MACF1 IPO9 FLOT1 USO1 ERBB2IP KPNA4 PEX2 STX19 NUP205 GOPC FERMT2 CD63 MZT1 TGFBRAP1 BCAP31 RPS2 TMSB10 GAK PEX6 CNTLN PEX7 TBC1D10A TOMM22 ZDHHC23 PAF1 IMMP2L ITGB1BP1 TIMM17A NLGN2 RPL23A MSX1 TRAPPC8 AP4B1 AP1B1 ATG9A HM13 KDELR1 SNX15 CD81 DCHS1 SUMO1 COPG RAE1 RTP2 VCL RUNDC1 CLTC BAG6 RPL36 AP3D1 GET4 BBS2 AP1M1 MARCH5 DRD1 SPRN EPB41L3 PPIL2 SKAP1 STAM SUFU NUP98 COG3 C11orf73 BCAP29 STXBP6 CAV3 NUP107 TRAF3IP1 POM121L12 RPS27 COPZ2 YBX1 VPS16 MLPH TBC1D22A PIH1D1 RAB26 RPLP1 GRID2 SEC63 NUP188 KLHL21 C12orf11 SMURF1 H2AFY2 GSN RPS15A BRAF RPGR VTI1A CASC3 GOLGA1 PEX10 NUP88 SHH CLTA MCM3AP SEC61G DNAJA3 RPS6 SURF4 SEC23B RPL19 STX1A TRIP11 DLG1 SEC61A1 TOMM40L RIC3 TBC1D30 RPS19 SNX5 TBC1D2 RAMP2 DLG4 STX1B AP3B1 TOMM34 TMEM30A F11R RAB7A OS9 GAS6 TBC1D8 MTX1 RPL27A RAB10 PIK3C3 LACRT RPS25 TBC1D16 SMAD7 AP3S2 RGPD6 LIN7B CPE TAF8 SYK PRKCZ JAK2 ATG3 EXOC3L4 KPNA2 ARHGEF2 RASSF9 SRPR REEP2 ARF6 SOX9 SPTBN1 HPS4 CDH2 XPO5 AGRN AKT2 PPP1R15A ID4 RPL36A ANKRD32 STX2 STX10 LAMA5 RAB34 AP1AR NUP133 MICALL1 HERC2 RUVBL2 TBC1D22B TMEM33 FGF10 TNFAIP3 PACS1 LMNA RHBDD1 SHANK3 RAD21 EXOC1 TOMM5 VAMP2 CDK1 VPS45 TSPAN5 NUPL2 JUN SUN2 PRPF18 ESR1 IFT81 VAPA VPS29 BAG4 EIF2D NAGPA EXOSC2 PDCD6 SEC23IP EFCAB4A GGA3 VHLL CCHCR1 MAPK3 CCDC99 COL1A1 LRP4 PDE2A COG7 VAMP5 RAB38 TOB1 STX4 TLN2 ANK2 PIGK KCNB1 TULP1 RERE RHOB BECN1P1 BECN1 SCG5 TTC7B ERP29 OXA1L PEX3 DDX3X RPS26 RB1 TBC1D5 POLA2 PAN3 KDELR3 HSPA4 RGPD1 KLC1 LATS2 RPS12 TOM1L2 RPL31 TIMM13 PIBF1 RPSA NKD2 CSE1L TOLLIP ANK1 HSPB11 RAB31 KPNA3 SEC61B RFTN1 CELSR1 SEC16B UGT8 NIPBL AUP1 FCHO2 NR5A1 SSB CLASP2 FAM126A TRAM2 ACAP2 SYTL3 GRIK2 OBFC2B TAF3 BMP4 DNAJA1 MFF TMED9 AMN AP1M2 TMED10 BMF SYNJ2BP KCNIP3 STX7 RAB3GAP2 PYGO1 RAB35 KRT18 LIN7A ATG4A RPS21 CTCF FYB ARL3 ZDHHC3 PACSIN1 PEX19 RPL12 FBLN5 AP1S1 TBC1D25 NACC2 SPATA7 PHB2 PARK2 CLTCL1 IMMP1L VPS18 XPOT TBC1D21 CORO1B NEDD4 BCL3 CHML TRAK1 VPS41 SNUPN ZDHHC18 ABCA12 HOMER3 HSPA8 EFR3A XPO1 RAPSN SNX33 YWHAH UBA52 SKP1 ATP1B3 RAB27A TSPO BRCA2 ITGB2 KPNA1 AKR1C3 STX3 ATG4C AP1S2 SELS WRAP53 ICMT TGFB1 EXOSC10 WNT11 RPLP0 EIF5A NUP50 YWHAB HEPACAM ICK BBS12 SRP72 CTNNA1 CEP250 PLK1 EGR2 LOC100507003 PPP1R10 C5orf42 HHEX TBC1D1 VAMP3 ASPSCR1 SRGN ADORA1 LATS1 IPO5 WASL SUN5 SLC9A3R1 ZFAND6 MESDC2 NFKBIA VPS13C CAPN3 DPP6 SNX11 MID1 PAX6 SPAG5 SEC13 TOPORS KPNA7 G3BP2 RPL4 NLRP5 KEAP1 XPO4 EYA1 CNTNAP1 TMSB4X CHMP4A TIMM44 BAD HERPUD1 GPAA1 TSPAN14 ASNA1 SRP19 SYNE2 TNFAIP2 BMP7 RPS5 IFT20 IFT27 ABRA MIPEP VCP ATP1B1 LONP2 ROCK1 RAMP1 TERT KPNA6 TIMM17B PKP2 FLNB RPS10 TCTN1 WDR45L ATM RPS27A MYADM SSR2 ARFGAP3 CCDC101 SNX13 NUP153 COPB2 C14orf133 FBXO7 POM121C BSG NCOA4 SSX2IP STX11 RHOD RPH3AL SNX1 IPO4 ARL2 NAPB RAB8B AKAP5 TNKS2 TTC7A RPS15 PAM16 RANBP1 TNPO2 TOR1A SIX3 EXPH5 LMAN1 MIS12 PKD1 MID2 AHCYL1 HIST1H1B DMD DYNLT1 MRAP TIMM50 RPS3 SEC24B RPL9 RPL10A TBC1D3B DZIP1 RPS11 FCER1G RPL32 ANXA2 ZP3 AURKA FAF2 POM121L2 NUP155 DNM1L STAM2 RER1 RRAGC SYNDIG1 RANBP2 PDIA3 CNTNAP2 PACSIN2 RAB7L1 AP4E1 EXOC3 UNC93B1 RPLP2 S100A10 TTC30A TBCK MIOS TNFRSF1A ZFYVE27 RPL13A MORC3 LRRC4 PSMD10 UBAC2 SYTL5 GDAP1 ATG4B DHX9 C4orf49 TOM1 EXOC3L1 PACS2 TMED2 NXT1 RPL13 RABGEF1 ACD MRAP2 PRKG2 C18orf55 ATP9B SGSM1 TMCO6 KATNB1 RPL15 CCDC14 RPL26 CDH1 SYNRG C7orf59 NAPG DLG2 RDX VPS11 TTC30B HBXIP USP6NL BLZF1 ARL2BP STX8 CHCHD4 TERF2IP VTI1B TIAM1 DERL1 TOR1AIP2 KIF13A RPS14 PIGR AKAP12 ITGA4 AMOT DNAJC19 RPL21 NEDD1 TOM1L1 TMEM150A F2R SRP9 ADPRH TIMM9 ARL6 COLQ STYX COPB1 CEP57 DLG3 SAR1A MDM2 FGF13 BBS1 RPL7 EHD2 GOLGA4 ETV5 RPL26L1 RFTN2 SRI ITGB7 TBC1D8B ZFAND2B TSPAN17 GRIK5 RPS20 WIPI1 STX16 CALCR KIF13B NFKBIL1 RPL37A CTNNB1 IL10 RTP4 RPS29 COX18 TSPAN15 DCP1A RPS23 TBC1D14 PEX12 LIN7C AHI1 FGF9 NUP35 CTAGE5 ROCK2 NPM1 EVI5L MTBP TINF2 TSPAN33 POM121B PEX26 SEC23A KPNB1 C2orf71 CLIP1 MYO5A COPA NLGN1 RPS8 CTDNEP1 GOLGA7 ARFIP1 CHAMP1 SNF8 PPP3CA KCNIP4 JUB CD24 TBC1D12 HES1 TGFB3 RPL14 EFR3B RIMS2 AP3M1 SUN3 TIMM10 SPTBN4 SNX9 RPL8 WAPAL NUP54 LTBP2 WIPI2 TIA1 FLOT2 RABGAP1L TGFB2 PFN4 RAPGEF2 VPS26A HTR2A BUB1B SNX2 SMO PEX5 RTP1 CDC37 GOLGA7B TMSB15A KCNB2 MTX3 YWHAZ SYTL1 NUPL1 ANK3 TULP2 IPO11 RPL41 TLN1 CAV1 TMSB15B CDC42 GRIN3B CCDC41 JUP AXIN2 DYNC2H1 GCC1 COPZ1 SIN3A PRKCI GAS8 WDR35 AP4M1 AP2M1 CRB3 TMEM188 RAPGEF6 VPS13A CNGB1 TBC1D10C PEX13 PKD2 RPL10 MDFI ANGPT1 TNPO1 SORL1 ZP2 IFT46 RAB11B SNX8 RPS24 SSR1 DIAPH1 SPCS2 HSP90AA1 IFT122 RAB32 OPRD1 FAF1 NUP214 JAK3 TTC26 PRICKLE1 GRTP1 COPG2 TBC1D9B APBA1 TBC1D9 ANKRD13C RNF128 TAP2 ATG9B SH3GLB1 STX12 GRPEL2 RPL39 BICD1 PADI6 RAB13 PTCH1 NDUFA13 ARCN1 CHP C6orf170 DBN1 CENPA STX17 TRNT1 TOMM70A TSPAN10 MXI1 SDCBP RGPD4 VPS28 RPS13 TOMM40 GLRB PDZD11 TIMM23 SNX17 DNLZ TEX15 MUSK BCL2L11 PINX1 STRADA EXOC8 ZW10 AP3S1 ATR ADPRHL1 TMSB4Y SHANK1 P2RY1 SUN1 TOR1AIP1 PIKFYVE BID STXBP4 RPL30 IFT140 VPS36 HK2 RRAGA XPO6 SEC24A CTSA GPR158 AGT SYNE1 CC2D2A YWHAQ H2AFY SIX1 IFT74 C15orf2 DCTN2 CTTN CSRP3 MADCAM1 TAOK2 C17orf28 SNX10 CBLB CCDC22 TIMM8B NCF1 RPS28 NRXN1 ARL6IP1 RANBP17 RPL3 RSG1 CASC5 PTPRU TMEM107 LRRK2 GCKR STX6 LAMP1 CD4 SYS1 WDR19 PTTG1IP CDK5 AP2A2 TRAK2 MEX3D RPS18 SNX31 RPH3A PMAIP1 DERL2 GGA1 OR1D2 WDR45 TOMM20 DNAJB6 DUSP16 VAMP7 DSCR3 CHM RPL37 EHD1 PLEKHF1 ATRX IPO13 NGFR SEC62 PCM1 RPL34 PEX16 MYO7A SPCS1 GGA2 PEX14 TONSL RYR2 RPL3L TUB DDX1 TAP1 SIL1 RAC1 LAMTOR2 CLTB GNPTG TULP3 USP4 WLS CSNK1E SEH1L SLC25A6 SH3PXD2B RPL22 KCNA1 TBC1D3C PKP3 CNTN2 RPL18 CRB1 MAPRE1 PML TXNIP SYVN1 CRIPT RILPL2 TBC1D26 RGPD3 FIS1 PIK3R1 MYO1C RTN2 KIAA0753 SNX16 INPP5K ARL1 RAB8A NUP85 XPO7 RILPL1 PDIA2 SCARB2 SEL1L KDELR2 HPS6 RPS16 TLK1 OBSCN RAN TNPO3 PIP5K1A TMEM88 RAB3GAP1 PRPF19 SRP68 EXOSC3 RPS9 RAB6A PARP3 NUTF2 NFASC RPL6 ZBTB16 LAMTOR1 ZDHHC22 VPS35 STIL CACNB2 NUBP1 MTCH2 SGSM2 RTP3 GBF1 EXOC3L2 ABCA7 IFT43 RPL29 AKAP6 STXBP1 MAPK1 RPL18A CHMP4B ESCO2 AP1G2 DVL1 VAMP4 DPP10 NRAS AFG3L2 TWF1 BAX NOP58 STAT3 SPAG4 GCC2 SNX6 WNT3A IFT57 LOC100652748 AGER DISC1 AP3B2 ZNF385A IKBKB GPIHBP1 SLC11A1 AURKB AKT1 RPL28 MTHFSD TRPS1 KIF20B GPHN SNX18 PICK1 TULP4 MYO6 OSBPL8 NPLOC4 PTPRK MTERFD2 LRP6 RPL7A TMEM129 SIX4 TBC1D15 GOSR2 EGFR LAMTOR3 PARD3 RRAGB TWF2 EVI5 IPO8 TTC21A RCC2 IFNG CSNK1D FCN1 MT3 RPL27 DVL2 SCIN TERF2 SCN3B CACNA1A TBC1D10B APPBP2 ARFRP1 RPL23 KPNA5 IFIT1 MFN2 GDF5 DAG1 TESC FLNA REEP1 SNAPIN ZFYVE16 RAB33B OPTN KIAA0528 WNT4 RPS7 FXC1 NUP62CL RGPD8 HSPA5 STRADB ANKRD50 RPAIN OBSL1 AZI1 TPR ITGAL GOLPH3 YWHAG POT1 CD74 PLRG1 APOE PALM FAM83D WNT5A RELN NCKIPSD MAP7 SUPT7L RAB11FIP3 BANP RRAGD VPS13D GRPEL1 RAB3IP AIP DENND4C C2CD3 RBM22 HOOK3 RPL11 TNF AP2A1 PEX5L AP1S3 TBC1D13 SSR3 RGPD5 RPL5 VPS39 RAP2A RIT2 EZH2 EZR IPO7 FRMD6 SYTL4 BBIP1 TOMM20L VPS25 UBL4A SIX2 PIK3R4 HK1 RAMP3 SAR1B SNX27 NUP62 RABGAP1 PAQR3 TRAM1L1 SRPRB AP3M2 RPL24 MOAP1 SAMM50 TBC1D3H NRP1 PINK1 PIGW BBS4 GSK3B ARMCX3 THRA ZFYVE9 RPL38 TTC21B MYRIP TNKS HEY2 FNTA RPS3A RPL17 MTX2 MPP5 RPL35A RAB11A CALR RPS17 TNIK WNK3 RPL35 HGS SEC24C HDAC6 SRP14 C14orf49 C2orf85 YWHAE NAPA BBS9 SYNGR1 RANGRF NPC1 FAM178A NSF TBC1D4 PREB SGSM3 GNPTAB RGPD2 TSC2 SLU7

GO_COPPER_ION_HOMEOSTASIS Any process involved in the maintenance of an internal steady state of copper ions within an organism or cell. ATP7B CUTC SLC31A1 ATOX1 COMMD1 PRNP ATP7A APP ARF1 MT2A CCDC22 PRND SCO2 XIAP SLC31A2 SCO1

GO_CELL_DIVISION The process resulting in division and partitioning of components of a cell to form more cells; may or may not be accompanied by the physical separation of a cell into distinct, individually membrane-bounded daughter cells. ING2 KIF4A NCAPG KIF2B C15orf23 LIG3 ENSA DIXDC1 JTB NUP37 NCAPG2 SETD8 SEPT4 CCNK SEPT10 CDK1 SEPT14 MAP4 CCNE1 CUZD1 ANKRD53 LIG4 BRCC3 CKS2 CDC6 KIF2C SKA3 RHOB SEPT3 ANAPC10 NUMBL CDK5 ANAPC4 BECN1 PHF13 CDK6 SYCP2 TERF1 SMC3 KATNB1 SMC4 POU3F2 CCDC99 AHCTF1 GNAI2 ARL8B CASC5 PIK3CB NEK2 NOX5 MYH9 MAU2 TNKS BIRC6 TSG101 LATS2 KIF13A VANGL2 PDS5B INO80 LRRCC1 BBS4 BCAR1 CHMP1A CETN1 WNT7A SMC1A BRSK2 RB1 ZNF830 NUMA1 CENPJ ACTR2 ZNF207 IST1 ZFYVE26 PKN2 NSMCE2 BCL2L1 KIF14 AATF DPPA3 CEP55 CNTRL RAB11A RCC1 CDC25A RGS14 MAPRE3 USP37 KLHL9 ALKBH4 DCT SPC24 USP8 NEDD1 DOCK7 CDK2 CDC23 NCAPH BIRC5 SON CCND2 ANAPC13 CCDC124 CEP63 RCC2 PAPD7 PARD6B CENPE SPIRE2 SENP5 PELO NDC80 PARD3B DYNC1LI1 CUL3 CENPA NCAPD3 CENPV SYCE1 HAUS8 NSL1 CHMP6 TPR PDS5A BUB3 ITGB3BP NEDD9 TUBA1C NCAPD2 CCNG1 SYCE3 SDCCAG3 TPRA1 CCNE2 MAP9 PIK3C3 RAB10 FZR1 PARD6G SPECC1L KIAA1383 CETN2 SPTBN1 MASTL ARF6 FAM83D PRKCE DYNLT3 CCNY MIS12 SKA1 FSD1 DYNLT1 MYH10 KIFC1 RAB11FIP3 PAFAH1B1 LMLN SPAST NUDC CHMP5 CENPC1 CEP164 HAUS2 KIF18B RUVBL1 STRA13 ARHGEF2 TACC1 ANAPC5 PLK5 ANKLE2 NEK4 ARHGEF11 STAG2 CLASP1 CD2AP TPX2 KIF4B RAD21 CDC26 NSUN2 CABLES2 SIRT2 RPS3 FMN2 GNAI3 HAUS7 NR3C1 AURKA APC SEPT11 ANAPC7 C7orf11 RHOA ZW10 CDK7 NDE1 NEK9 PARD6A CHMP1B CDC16 NUMB ARL8A SEPT5 ECT2 CDC25C TGFB2 SOX5 RNF8 ZWINT CDC20 SYCP3 DCLRE1A MITD1 TIAL1 RASA1 PPP2R2D HAUS6 ZBTB16 BUB1B FBXL7 PDCD6IP RACGAP1 CKS1B TIMELESS SNX9 ZFYVE19 INCENP SEPT12 NUSAP1 ANAPC2 WAPAL CCNB1 KIT CHMP4B ARPP19 HELLS WASL CCNB3 ASPM PTTG1 CCNT2 TXNL4A FAM175B REEP4 KIAA0196 ANAPC16 C19orf21 TP63 MAD2L1 CDK4 NUF2 CDC14A PLK1 PPP1CC CENPW CKAP2 ANK3 SMC5 SSSCA1 CINP LATS1 SPC25 CDC123 NEK6 CIB1 BORA TOP1 FGFR2 USP16 CUL7 UBE2C SETDB2 KLHL13 CHFR CSNK1A1 ANXA11 TIPIN AURKC CHMP4A MAD2L2 C1orf96 ANAPC1 TTC19 SNX18 KIF20B CDC7 VPS4A RBBP8 SEPT2 SPAG5 RALA BIN3 HAUS4 WNT3A C11orf51 DSN1 FZD7 CIT DIS3L2 FAM64A SKA2 CCNT1 ERCC2 AURKB CECR2 CCND1 NEK3 CETN3 PARD3 TTC28 DAPK3 TOP2A BABAM1 MIS18A KNTC1 VRK1 EVI5 REEP3 STAMBP PMF1 LLGL2 CENPT C12orf11 KLHL21 CCNO CCNA2 POU3F3 STOX1 CDK3 KLHDC5 CDK20 NEK1 ZNF16 BOD1 ETV5 TUBB SAC3D1 CDCA8 UBE2S KLHL22 KIF2A BUB1 CCNB2 USP9X OIP5 SPICE1 ACTR3 WWTR1 DCTN3 RALB SYCP1 STAG1 SGOL2 CDC25B CLASP2 WEE1 CCNF CHMP3 KIF20A MAPRE2 FGF13 PAPD5 PPP1R1C ZWILCH MAD1L1 CHMP2B USP44 CENPF CHMP4C NUP43 CDK14 CCNA1 RECQL5 SPG20 MIS18BP1 RAB11FIP4 ARL3 ROPN1B KIF23 SMC2 GNAI1 CDCA5 MAPRE1 HOXB4 POGZ SEH1L MCMBP GOLGA2 STRA8 PRPF40A MAEA BOD1P RHOC CTDP1 RAB35 ZFP36L2 KATNA1 LIG1 MARK4 CHEK2 TEX14 USP39 ANLN VPS4B HMGA2 CABLES1 HEPACAM2 SPIRE1 FBXO5 PPP1CB PSRC1 CDCA2 SEPT6 PRC1 CCNG2 CHMP2A SYCE2 FGFR1 ROCK2 CCND3 C9orf114 TEAD3 SEPT9 HAUS5 KIF11 TRIOBP ERCC6L DIAPH2 TUBA1A PPP1CA NEK7 SGOL1 CKAP5 ESPL1 APITD1 BRE RAN BRCA2 CHMP7 ANAPC11 FIGN CNTROB HAUS1 CDCA3 E4F1 TUBA1B LEF1 SNX33 ACTR8 HAUS3 NOTCH1 SEPT7 ZC3HC1 SEPT1 LZTS2 STMN1 UBE2I

GO_REGULATION_OF_HAIR_CYCLE Any process that modulates the frequency, rate or extent of the cyclical phases of growth (anagen), regression (catagen), quiescence (telogen), and shedding (exogen) in the life of a hair. GAL CLOCK FA2H CDH3 TERT TRPV3 ARNTL WNT10B TRADD FOXN1 FST HPSE TNF NIPBL SMO PER1 MSX2 TGFB2 SMAD4 KRT17 DKK4 MYSM1

GO_RESPONSE_TO_ORGANIC_CYCLIC_COMPOUND Any process that results in a change in state or activity of a cell or an organism (in terms of movement, secretion, enzyme production, gene expression, etc.) as a result of an organic cyclic compound stimulus. HCFC1 PDCD7 RORA DHODH TGFBR2 MSTN PTCH1 HEY1 PSMB2 PTAFR OGG1 ID3 PER1 TET2 DUSP1 PELI1 HNF4A AIFM1 GPI NR1I2 SP5 MUL1 KLF2 RYR3 LDHA CARM1 PAK3 IFNA21 NPAS4 MMP14 AR NPFF ABHD2 PSPH RAMP2 PRKRA STC1 DUOX1 PFKFB1 MAVS RHOXF1 INHBA NR6A1 ENG NR3C1 CXCL10 DUSP6 PLA2G4F PPP5C P2RY1 H2AFZ RUVBL2 AVP CYBB PRKCG P2RX2 ERRFI1 IL13 CAT GPD1 CPN1 KRAS ACSBG1 SLC8A3 IFNE MSX2 HCN3 FGF10 ABL1 BID SPP1 MED16 RNLS MTAP PDGFRB EGLN2 HDAC5 MED24 JAK2 CHRNG CNGA3 GOT1 UTS2 CHRNB1 AQP8 PTK2B ATP2B4 PRKCE SLC26A3 CYP1A1 PTN DEFA1B PTPRU COL1A1 LRRK2 EFNA5 PDE2A MYOD1 DDX54 CPT1A MAPK3 UBR5 EPHX1 NR1I3 DDX58 HCN4 GHRHR CDK5 ASNS BECN1 SMAD3 UGT1A1 GCLM TARBP2 MED13 WNT7B SOD1 STC2 CD4 ABCA3 VCAM1 SMAD2 GSTP1 GABRG2 PPARG JUN ESR1 TIPARP ADCY1 MED1 CDK1 CCNE1 BRCA1 NR0B2 CYP1A2 APEX1 SLIT3 BGLAP SLC6A3 CYBA ABCC2 CDKN1B C17orf28 IFNA1 CA9 HNRNPD PGR REN MED4 DEFA3 IFNA10 RBM14 NCOA6 OXT RFTN1 IGFBP5 IMPACT WNT8B BCL2L1 NR5A1 KCNE1 PIM1 CHRNA6 RBFOX2 MED30 JUNB SDK1 CNR1 ADAM9 LOX RB1 STRN3 CALM2 MBD2 PMAIP1 ZC3H12A PPARGC1A SPARC ATF1 TAT ADA SSTR4 GRIP1 NTRK3 ADSS LY6D ANXA3 CREB1 MED12 PCNA EIF2C2 DGCR8 IL4 ABCA1 TADA3 SSTR5 STAT5B POU4F2 IFNA13 ID1 SSTR1 ZFP36L2 PPP2R2A ESRRA MED14 IL18 CALCR UCN GNAI1 RARA HSD11B2 PRKACA IFNB1 P2RY12 MLC1 THBS1 CTNNB1 KCNJ8 MRPL44 DRD2 EIF4A3 IL10 UBE2L3 ISL1 MDM2 PDE3A CHRNA5 CASP8 TEK SELL PAX4 THRAP3 FGF23 CHRNB2 ELK1 ADM EDN1 RALB DSG2 NEDD8 CCL2 RFTN2 PIAS1 SOX30 HDAC2 PPARGC1B NR1H4 CHRNA3 CASP1 S100B CCDC62 CIB2 CLDN4 PCK2 CHRNA2 FLOT1 GPER TICAM1 PRKAA1 LEF1 PAQR8 CHRNA1 TGFB3 ATP1A1 RBBP7 CYP7A1 NR4A1 RXRG ABCB4 COLEC12 RGS19 KCNJ11 HNMT GJB2 FOXA1 OPRM1 ZFP36L1 NTRK1 NR3C2 DROSHA CITED1 UBE3A ADIPOQ ROCK2 LRP8 HSP90B1 DPYSL2 NPM1 HOMER1 NR2F2 AREG F7 SCGB1A1 RXRA IFNA7 CD83 AGTR2 KDM1A POSTN HNRNPU SLIT2 OPRK1 TAF2 NKX2-2 ESRRB IL22 TMF1 TAF7 MED17 ETS1 DAXX ASS1 PTGDS ACSL1 G6PD SOCS2 RAE1 MBD1 APOA2 NOD2 DRD1 HMGB2 AXIN2 CASP3 IRG1 CDK4 SLC34A1 NR1D2 FOSL1 JUP WFDC1 AACS PRKACB CATSPER2 NKX6-1 CAV1 PTPRN MSX1 DDX21 P2RX5 CCNB1 PLA2G5 OPRL1 LYN AMH CHRNB4 KAT5 SRD5A1 TRH CHRNA4 HTR2A NR4A3 SMO PPARA FOSB ZCCHC11 RAP1A DHH CDC5L TGFB2 COMT PDXP HDAC1 ITPR2 RAPGEF2 DRD5 NR2C1 P2RX3 MBD4 ATP1A2 GSTM2 CCNA2 BTG2 BMP2 CRHBP PGRMC2 BRAF ZNF703 CATSPERB EIF2C3 CALM1 SRC IDH1 PAPPA IRF3 PARP1 HOXB13 ESRRG ADCY5 NR2F1 PAM DICER1 HSP90AA1 DIAPH1 CPS1 ACACB PDE4B SSTR2 MSN GPLD1 TRPV1 CCND1 SIN3A CD36 SLC3A2 TMEM173 KIAA0146 EEF2 IHH SLC9A1 NR1D1 REST HSF1 CST3 LGALS1 CYP27B1 ABCA2 ALDH1A2 KLF9 CHRNB3 APP SLC6A4 TBXA2R HSPD1 NCOA3 EPO GNAT3 RNF4 GPR83 ACR MKI67 OR51E2 FOLR2 CYP24A1 NCOA4 CCL3 OXTR BSG GABRB3 PLIN2 PPARD ZFP36 ADCY3 KLF5 ARNT2 CASQ2 TRIM25 PANX1 UCP3 BAK1 AQP1 TERT JUND DNMT3B WNT10B CATSPERG GH1 NQO1 IFNA2 IFIT1 RGS20 CD38 BMP6 KCNMB1 RPL32 CDK7 CEBPB ADAR CHRNA7 HTR3A WT1 SIRT2 NDUFS4 GLI2 TXN2 ADCY6 CFTR CRYAB FHL2 SNIP1 NRIP1 TNF AGXT GNRH1 IFNA17 DHX36 KCNQ1 BIRC2 SRRT HCN2 PAQR5 PRKAR2B HTR1B CALCOCO1 SMYD3 FKBP4 IL1B PPAP2A NR2E1 TLR2 HRH1 UCN3 VDR SAFB SSH1 PDX1 IFNA14 NR4A2 CREM ADCY2 GNG2 BLM PTEN P2RX7 IFNA4 FGFR4 HOMER2 AXIN1 CATSPER1 CHKA IFNW1 GABRB2 RAPGEF1 LPHN3 GABRA1 PTGS2 METTL3 PIAS2 NOX4 C18orf26 LEP HMGCS1 CATSPER4 CHRNE C4orf49 TPCN2 SLC5A5 ADCYAP1R1 BTRC EZR MBD3 HNF1B IFNK PAK4 EZH2 FGF8 ASCL1 IGFBP2 TFPI ALAD RAMP3 RIPK2 NKX3-1 ABCC4 RNF14 IRAK3 ABP1 RAD51 PAQR7 DHFR TACR3 DHFRP1 IL1RN PAK2 OTC ACTA1 AANAT STAR CTSL2 CALR SLC16A1 PMVK PLN RYR1 AIF1 FOLR1 CCL5 CYP1B1 FBXO32 KAT2A DRD3 CIDEA NPC1 MDK NR1H2 CRH THRA CASP9 MYOG MAOB RELA SLC22A6 PAQR9 APLP1 CDH1 SMAD1 NR1H3 WNT7A MTHFR MEF2C TYMS GBA UGT3A2 SRR LUM SRSF2 SLC26A6 KLF4 IFNA16 TNC GRIN1 TIAM1 SEPN1 TYR IL6 NR5A2 AKAP9 GABRB1 NR2E3 EEF2K PDE4D CAD P2RX1 PAQR6 NME1 FOXO1 NR2C2 EDNRB SREBF1 TRIM63 MTOR TLR3 NFKB1 HMOX1 CALM3 IGFBP7 GIP PHB IFNA6 SMAD5 IFNA5 NR0B1 ALDH3A1 ITGA6 TPH2 FLT3 IFNA8 CDKN1A SDC1 PTGES HPCA RYR2 OSBPL7 SST SERPINF1 DNM2 MSR1 TAC1 BMP4 ANKRD1 SLC26A5 PENK DDX1 CDKN2D SLC6A1 DNAJA1 ACAT1 ANXA1 NOTCH1 ANXA7 DEFA1 TH KDM3A YWHAH RARG INPP5K VGF RAN AKR1C3 SFRP1 RORC PKLR ARID1A TMEM146 TSPO NCOA1 LIN28A ENDOG BCL2 SMAD9 THRB MMP19 KCNK1 TRIP4 PADI2 C19orf2 PIK3CG TXNIP HSP90AB1 ADCYAP1 PARK2 NR2F6 EFTUD2 KALRN HNRNPA2B1 GCLC HAMP NEDD4 DRD4 LIN28B CHEK2 ATP2B1 APOA4 ABCD3 CEBPA CA2 TRPM2 EIF2C4 AKAP6 CCL1 SNCA CHRND ESR2 KYNU CTNNA1 P2RX4 ATP1A3 ZC3HAV1 HNF4G TRPC3 MGMT STAT3 CARD9 SSTR3 FOS MAPK1 PTH RPS6KB1 HLCS PEA15 P2RY11 NFKBIA P2RY6 GNB1 TGFBR1 NASP TGFB1 RPLP0 EIF2C1 ICAM1 HTR5A P2RY13 DDX18 ANGPT2 DUOX2 AQP9 RAPGEF3 ARG1 RXRB SMPD1 ALPL CTGF IL15 DNTT ABAT BMP7 DGKQ ACSL3 PPP1R9B CCR5 SRD5A2 ITGA2 TGFB1I1 KCNC2 THBD MMP15 FAS LRP6 RORB RAP1B TRPA1 GNAO1 PMEPA1 DSG1 SCGB2A1 NAMPT FOXF1 LONP2 NEFL AVPR1A RARB EGFR MB21D1 P2RX6 HCN1 AQP3 TFAP4 LYPD1 STAT1 PTGFR AKT1 BCHE SLC7A11 FKBP1B CATSPER3 RBBP8 EPB49 SLC8A1 FOXO3 FECH DEFB1 CDO1 BAD DNMT3A PTGER2 PCK1

GO_CELLULAR_RESPONSE_TO_BIOTIC_STIMULUS Any process that results in a change in state or activity of a cell (in terms of movement, secretion, enzyme production, gene expression, etc.) as a result of a biotic stimulus, a stimulus caused or produced by a living organism. NOS3 CDC73 CTR9 PRKCE WNT5A CLEC7A TMCO1 TLR4 OPRK1 HAMP KLRC4-KLRK1 NFKBIB HDAC5 TXNIP IL1B TLR2 WFS1 CXCL16 UPF1 STAP1 ADAMTS13 TNFSF4 TSPO PPP1R15B ABL1 IRAK2 TNFAIP3 CX3CR1 TNF SELS SIRT2 TICAM1 MTDH APAF1 NOS2 NR1H4 CXCL10 CEBPB C19orf29 LILRB1 GFI1 EME1 ANKRD1 CCL2 BTK TLR6 IL24 LITAF TNFRSF1B AKAP8 HCK IFNG IL8 ATG10 TP53 SCARB1 PPARD ZFP36 IL10 CD80 HSPA5 TLR5 PTAFR RARA MUS81 CCL3 SYK NFKB1 CSF2 EIF2AK3 PDE4D TREM2 PABPN1 SERPINE1 FZD5 IL18 EDNRB TLR1 CD6 IL6 NFKBIL1 TBXA2R FBXO18 ABCA1 HSF1 CDK19 DDIT3 NR1D1 AXL SHPK GSK3B LBP NR1H3 MEF2C CD36 TICAM2 IRAK1 PDE4B CD180 AKT1 RELA IRF8 IL12A PPARGC1A ZC3H12A PLSCR3 CD40 CSF3 CD14 ADAM9 IRF3 CCL5 MAPK14 KLRK1 LY96 CEBPE SRC STAR CCR5 AICDA ARG1 TIRAP CCL20 CMPK2 TRAF6 TFPI RIPK2 PTPN22 LYN ICAM1 PAF1 CD86 TGFB1 TNIP3 MAPK1 PLSCR4 XBP1 MAPK8 IL12B SBNO2 PRPF8 TNIP2 NFKBIA CCDC47 GSTP1 B2M HMGB2 PYCARD IRG1 DAB2IP NLRP3 SPON2 HAVCR2 LILRB2 MAPK3 SLX4 MRC1 ASS1

GO_NEGATIVE_REGULATION_OF_INTERLEUKIN_8_PRODUCTION Any process that stops, prevents, or reduces the frequency, rate, or extent of interleukin-8 production. C19orf29 TLR9 ANXA1 ELANE KLF4 PTPN22 BPI MAP2K5 ARRB1 SSC5D BCL3 OTUD7B ANXA4 IL10

GO_N_TERMINAL_PROTEIN_AMINO_ACID_ACETYLATION The acetylation of the N-terminal amino acid of proteins. NAA60 CREBBP EP300 NAA50 C7orf52 NAA15 NAA16 NAA10 NAA20 NAA25 NAA30 AANAT NAT9 NAA11 KAT2B

GO_CARTILAGE_CONDENSATION The condensation of mesenchymal cells that have been committed to differentiate into chondrocytes. SOX9 TGFB2 ROR2 UNCX PKD1 MGP CTGF COL11A1 MAPK14 FGF6 BMPR1B OTOR BARX2 WNT7A ALX1 BMP1 FGF4 MYCN COL2A1 THRA MYF5

GO_GLUCOSE_CATABOLIC_PROCESS The chemical reactions and pathways resulting in the breakdown of glucose, the aldohexose gluco-hexose. PFKFB3 PFKL PKLR HK1 BAD HK2 PFKFB4 ENO3 ENO1 GAPDHS ALDOA PGK1 PFKFB2 PFKP GPI PGAM1 PFKFB1 TKTL1 PFKM TPI1 GCK GAPDH ENO2 ALDOB LRP5 HK3 PGAM2 PKM2 ALDOC

GO_RESPONSE_TO_VITAMIN_A Any process that results in a change in state or activity of a cell or an organism (in terms of movement, secretion, enzyme production, gene expression, etc.) as a result of a vitamin A stimulus. GATA4 TYMS EPO DNMT3B PITX2 PPARG CYP1A1 ALDH1A2 ARG1 PPARD SLC34A1 TSHB HAMP DNMT3A CAT RARA

GO_INTERMEDIATE_FILAMENT_ORGANIZATION Control of the spatial distribution of intermediate filaments; includes organizing filaments into meshworks, bundles, or other structures, as by cross-linking. GFAP KRT14 DSP SHH KRT71 MTM1 PKP1 NEFH AGFG1 DNAJB6 KRT20 KRT17 DES NEFL KRT2 PKP2 KRT25 VIM KRT9 NEFM

GO_TRNA_WOBBLE_BASE_MODIFICATION The process in which the nucleotide at position 34 in the anticodon of a tRNA is post-transcriptionally modified. TRMU MTO1 MOCS3 IKBKAP URM1 KIAA1456 ALKBH8 CTU2 GTPBP3 ELP3 CTU1 ADAT2

GO_ERBB_SIGNALING_PATHWAY A series of molecular signals initiated by binding of a ligand to a member of the ERBB family of receptor tyrosine kinases on the surface of a cell, and ending with regulation of a downstream cellular process, e.g. transcription. HSP90AA1 ABL1 RPS6KA5 GAB1 TDGF1 EGFR NRG4 ADAM17 SOCS5 KRAS SRC ERBB2IP STUB1 EREG FAM59A PIK3C2A SOS1 UBA52 USP8 EPS8 NCK2 FAM83B PIK3R1 SOX9 IQGAP1 PTK2B HRAS PIGR GRB2 SHC1 SHC3 NRG2 RNF41 ERBB4 MVP ERBB3 BCAR1 EFEMP1 UBB AKT1 VIL1 UBC PTK6 AREG RASSF2 DGKD FAM83A MAPK1 EGF PTPN11 NRAS PTK2 CSK HBEGF CPNE3 NRG1 EPS15 CDC37 ARF4 FES PDPK1 GRB7 MYOC PAG1 MATK PIK3CA PLCG1 ERBB2 RPS27A KIF16B PLCE1 CBLB CUL5 BTC CBL PTPRR PXN REPS2 CAMLG TGFB1

GO_SISTER_CHROMATID_COHESION The cell cycle process in which the sister chromatids of a replicated chromosome are associated with each other. REC8 DSCC1 BUB1 BUB1B RAD21L1 NUP37 NDEL1 RANGAP1 BIRC5 KIF2B CDC20 KIF2A CDCA8 ZWINT NDC80 WAPAL CENPQ KIF2C CENPF ZWILCH KIF22 MAD1L1 CLASP2 RCC2 HORMAD2 STAG1 SGOL2 PAPD7 NUP160 INCENP CENPE RAD51C CENPK DDX11 MAD2L1 MAPRE1 CENPI PDS5A SMC3 CDCA5 NUF2 CENPA NUP43 MLF1IP NSL1 PPP2R1A ESCO1 CASC5 CENPO HDAC8 SMC5 SPC25 MRE11A MCMBP CENPN POGZ BUB3 SEH1L PLK1 ITGB3BP AHCTF1 STRA8 PPP1CC CCDC99 B9D2 SKA1 CLIP1 PAFAH1B1 LOC728637 PDS5B MAU2 NAA50 NUP107 MIS12 RPS27 SKA2 CENPP SEC13 GSG2 SMC1A DSN1 RB1 ERCC6L AURKB CENPC1 CENPH NUDC CENPL NUP98 PHB2 RANBP2 NIPBL RAD21 SGOL1 CKAP5 APITD1 PMF1 TAOK1 KNTC1 SMC1B CLASP1 STAG2 NDE1 ZW10 NUP133 NUP85 SPC24 FBXW7 HORMAD1 CENPT KIF18A XPO1 CENPM

GO_POSITIVE_REGULATION_OF_RECEPTOR_RECYCLING Any process that activates or increases the frequency, rate or extent of receptor recycling. RAB7L1 EPS15 PSEN1 ECE1 BVES NSF D4S234E VAMP3 SCRIB INPP5F SNCA RAMP3 ARAP1

GO_LYMPHOCYTE_ACTIVATION_INVOLVED_IN_IMMUNE_RESPONSE A change in morphology and behavior of a lymphocyte resulting from exposure to a specific antigen, mitogen, cytokine, chemokine, cellular ligand, or soluble factor, leading to the initiation or perpetuation of an immune response. STAT6 FOXP1 VAMP7 TLR4 CD1C PTK2B HMGB1 IFNA16 IFNA14 GATA3 IL4 MFNG NOTCH2 LGALS1 IRF4 BCL3 IFNA4 LY9 ATP7A CD180 SLC11A1 GPR183 LFNG ADA CLEC4E IFNA7 EXOSC6 GAPT RORC UNC13D IFNE F2RL1 RAB27A IFNA17 LCP1 ABL1 IL18R1 MSH2 IFNA10 FCER1G LILRB1 RC3H2 CCR6 EOMES LEF1 CORO1A APBB1IP IFNA2 PLCG2 AICDA IFNA1 SLAMF6 CD40LG EXOSC3 CDH17 RNF8 SEMA4A KLRF2 RC3H1 LIG4 CD86 RNF168 ERCC1 IFNK BATF MSH6 IFNA21 TNFSF18 IFNA8 ICAM1 RPS6 VAMP2 TSC1 CLEC4D IFNG ITGAL IFNB1 PTGER4 NKX2-3 EXO1 RELB PSEN1 IL12B CD244 NBN PLCL2 IFNA5 IFNA6 EIF2AK4 MLH1 SWAP70 IL6 IFNA13 RORA HSPD1 ITFG2 DLL1 ZFPM1 IFNW1 ITM2A

GO_LOCALIZATION_WITHIN_MEMBRANE Any process in which a substance or cellular entity, such as a protein complex or organelle, is transported to, and/or maintained in, a specific location within a membrane. MUSK ITGA4 RAPSN MIA3 SYNGAP1 FLNA NRXN1 GRIK5 PICK1 CD24 RILPL1 RFTN1 LRP4 NUP133 IFT122 WDR19 DVL1 RER1 COLQ CNIH2 DLG3 ITGAL CD2 SHANK3 CDK5 ATP1B1 ITGB2 DLG2 RALA ITGB1BP1 CDH13 RILPL2 DNAJA3 GRIK2 CACNA1A MADCAM1 DLG1 TBC1D20 SSNA1 TMEM48 APOE RELN AGRN RAB1A CDH2 MAGI2 ITGB7 DOCK2 NRXN2 FNTA REEP2 RFTN2 GLRB ETV5 DLG4 PIGR NLGN1 GPHN MAL CLIP1 RAC1

GO_REGULATION_OF_ARF_PROTEIN_SIGNAL_TRANSDUCTION Any process that modulates the frequency, rate or extent of ARF protein signal transduction. CYTH4 PSD CYTH1 IQSEC1 PSD2 IQSEC3 PSD3 CYTH2 IQSEC2 ARFGEF2 PSD4 GBF1 KIAA1244 ARFGEF1 CYTH3 FBXO8

GO_DIOL_METABOLIC_PROCESS The chemical reactions and pathways involving a diol, a compound that contains two hydroxy groups, generally assumed to be, but not necessarily, alcoholic. GBA SPTLC1 ACER3 ACER2 DEGS2 SPHK1 SPTLC2 ACER1 SPHK2 SGPP1 SGPP2

GO_NEGATIVE_REGULATION_OF_ESTABLISHMENT_OF_PROTEIN_LOCALIZATION_TO_PLASMA_MEMBRANE Any process that decreases the frequency, rate or extent of the directed movement of a protein to a specific location in the plasma membrane. PPP2R5A TMEM59 LRRC15 RHOQ GOPC LYPLA1 PID1 CSK BCL2L1 PPFIA1 TMBIM1 PKDCC

GO_RESPONSE_TO_INTERFERON_GAMMA Any process that results in a change in state or activity of a cell or an organism (in terms of movement, secretion, enzyme production, gene expression, etc.) as a result of an interferon-gamma stimulus. Interferon-gamma is also known as type II interferon. IL12RB1 LGALS9 CCL24 AQP4 TLR3 CCL3 IRF5 PTAFR CCL3L3 CCL18 TRIM31 CALCOCO2 CX3CL1 SP100 CCL2 IRF9 TRIM21 EDN1 IRF2 TRIM34 CCL15 CCL19 CCL14 IRF7 HCK IFNG SEC61A1 IFI30 FCGR1A TRIM25 CCL22 CAMK2G CCL8 CCL4 TRIM68 HLA-DQA2 NOS2 HLA-DRB3 HLA-F HLA-B HLA-C HLA-E WNT5A TRIM62 CAMK2B UBD CCL25 JAK2 CCL23 OASL PML ADAMTS13 OAS3 CITED1 CXCL16 CCL21 VCAM1 IL12B CCL4L2 IRG1 GBP2 CD58 HLA-DPB1 CCL17 MEFV HLA-G XCL2 CD44 B2M XCL1 IRF6 JAK1 CCL7 PRKCD EPRS NCAM1 KYNU SNCA GCH1 CAMK2A CCL16 MRC1 CIITA CCL1 ASS1 HLA-DQB2 MT2A CCL20 RPL13A IFNGR2 FCGR1B HLA-DRB4 HLA-DRA BST2 HLA-DQB1 ICAM1 CCL3L1 HLA-DPA1 SYNCRIP HLA-DRB1 NMI CD86 HLA-DRB5 NUB1 DAPK3 HLA-DQA1 TDGF1 IFITM2 CAMK2D IRF3 AIF1 CCL26 IFNGR1 CCL5 HLA-A TRIM22 GBP1 SLC30A8 HLA-H STAR TRIM38 TRIM8 C19orf66 OAS1 TRIM5 IRF4 CYP27B1 SLC26A6 IL23R GBP6 IFITM3 DAPK1 IFITM1 MID1 CCL13 IRF1 OAS2 IRF8 TRIM26 GAPDH STAT1 GBP5 SLC11A1 CCL11

GO_FC_RECEPTOR_SIGNALING_PATHWAY A series of molecular signals initiated by the binding of the Fc portion of an immunoglobulin to an Fc receptor on the surface of a signal-receiving cell, and ending with regulation of a downstream cellular process, e.g. transcription. The Fc portion of an immunoglobulin is its C-terminal constant region. PLCG2 ACTR3 NFATC3 RAC1 IGKC BTK ELMO1 RPS27A PRKCQ MS4A2 IGKV1-5 MAPK10 VAV2 PSMD13 PSMB8 WIPF1 FCGR1A HCK CLEC4D PSMD11 IGKV2-40 PSMF1 WASF2 SYK PSMB4 NFKB1 ARPC4 PTK2 PSMB2 PSMB9 IGHV2-5 VAV3 WIPF3 PSMB7 PSMC3 PSMC1 CYFIP1 PSMD5 CALM3 PAK1 PSMC2 MAP2K7 IGHG1 IKBKG DOCK1 IGLC1 MYH2 PIK3R2 PDPK1 TAB1 NCKAP1 IGHG4 MYO1G PSMD8 UBE2N PSMB6 GRB2 SHC1 NCKIPSD FCGR2A NCKAP1L PSME3 LOC440786 MYO1C PIK3R1 PRKCE MALT1 LAT PSME2 MAP2K4 IGHV3-23 PSMA8 MYO10 PSMA5 CD247 IGKV3D-20 IGHE WIPF2 HSP90AB1 FER FCER1A NFATC1 PLD2 KRAS ABL1 PSMD14 GRAP2 PSMA2 PPP3CA SKP1 MAPK9 UBA52 PSMC6 FCER1G PSMD12 NFATC2 IGKV3-20 ARPC1B ARPC1A FYN PSME1 PSMA1 PSMD10 PSMB11 IGKV4-1 PSMB10 ELMO2 CARD11 PSMB5 NR4A3 NCK1 FCGR3A ARPC3 FCGR1B TRAF6 CUL1 PSMD3 LAT2 FGR PPAPDC1A JUN BTRC PSMA3 PSMD9 BAIAP2 TEC LYN PSMC4 IGHG2 BRK1 GAB2 PSMA6 FOS NRAS CDC42 IGHG3 PSMB3 NFKBIA LCP2 CRK MAPK1 PSMC5 MAPK8 KIT WASL VAV1 PSMD1 PIK3CB ACTB PIK3CA PLCG1 MAPK3 LIMK1 ABI1 ARPC5 PRKCD HRAS PIGR PSMB1 PSMA7 PSME4 PSMA4 MAP3K7 PSMD6 IGKV1D-33 MAP3K1 UBB RELA CALM2 UBC IGLC7 PSMD2 WAS UBE2D1 ARPC2 PPP3CB IKBKB IGLC3 CLEC4E PSMD7 FBXW11 YES1 IGLC6 CD3G ITK TAB3 HSP90AA1 UBE2D2 IGKV3D-11 IGLC2 PLA2G6 ACTR2 PPP3R1 SOS1 TAB2 IGLV1-51 UBE2V1 CHUK PSMD4 PAK2 BCL10 CALM1 ACTG1 SRC FCGRT CYFIP2 IGLV7-43

GO_PROTEIN_CARBOXYLATION The addition of a carboxy group to a protein amino acid. F9 F7 VKORC1L1 PROZ VKORC1 PROS1 GGCX F10 F2 BGLAP PROC

GO_REGIONALIZATION The pattern specification process that results in the subdivision of an axis or axes in space to define an area or volume in which specific patterns of cell differentiation will take place or in which cells interpret a specific environment. ZBTB16 NODAL SMO WNT1 HOXC8 MSGN1 HHIP TGFBR1 DLX2 GREM2 POGLUT1 MSX1 ALX1 TP63 CXXC4 HOXB3 AXIN2 PCDH8 DYNC2H1 TBR1 HOXD8 PSEN1 HIPK2 AIDA FOXA2 NKX6-1 HHEX SEMA3F NEUROD1 DLL1 FKBP8 MEGF8 HES3 RFX4 PBX3 BMPR1B LDB1 TRA2B EGR2 ABI1 BASP1 GATA4 ACVR2A HOXA3 HOXB7 GDF11 KDM2B DLL4 WNT3 SETDB2 DKK1 FGFR2 SMAD4 HIPK1 LMX1B ALDH1A2 TRAF3IP1 VAX2 FOXJ1 MESP1 CDON PCSK6 SUFU WNT2B HOXD13 PAX6 WNT3A IFT57 MEOX2 SHH MEOX1 FOXF1 IFT172 ACVR2B GSX2 RPGRIP1L IFT122 CRB2 CDX4 EVX1 BMP2 PITX2 BTG2 T PBX2 MDFI LRP6 TULP3 RIPPLY2 FOXB1 EDN1 WLS BMP4 NOG CITED2 RNF2 ISL1 HOXD9 SEMA3C SMAD6 SEMA3A TBX6 HOXC11 BPTF EGF HOXC9 SRF MNS1 CTNNB1 CELSR2 OTX1 HOXD11 EN1 DMRT3 RING1 EMX2 RIPPLY1 HOXB4 SP8 FOXC1 DLL3 HOXC5 NKX2-2 MLLT3 TDRD5 FOXC2 PKD1L1 NRARP NR2F2 YY1 PLD6 AHI1 HES7 NBL1 HOXA5 PCSK5 GDF3 ARC SFRP1 RGS19 FOXA1 HES1 NOTCH1 RARG POFUT1 NKX6-2 HES5 EOMES LEF1 TBX1 SIX2 TBX3 NKX3-1 CHSY1 MTF2 ZIC3 EPB41L5 CDX1 PAX7 CDX2 HOXB5 HNF1B PBX1 SENP2 COBL NTF4 TCF15 MYF6 IRX1 ASCL1 FGF8 ACD IFT52 DSCAML1 DPCD HOXD3 NRP1 SMAD3 LRP5L RBPJ DBX1 TMED2 SMAD2 FEZF2 WDR19 WNT7B FOXH1 SOX1 GRSF1 NKX2-5 TMEM107 LRP4 AXIN1 FOXD1 PALB2 WNT8A FEZF1 MED12 VANGL2 DDIT3 CYP26B1 NOTO PCGF2 LHX2 HEY2 CTNNBIP1 DISP1 SMARCD3 BMPR2 CRKL LFNG TTC21B WNT7A HOXA2 MEF2C HOXB9 HOXB6 WNT8B OTX2 MLL TDGF1 KAT2A HOXA11 CELSR1 ARL13B GSC IRX3 NKX2-1 GLI3 DLX1 HSPB11 BARX1 MAFB SOX17 HOXB1 ETS2 NLE1 HOXC4 TCTN1 RGS20 FBXL15 ATM SNAI1 EP300 PROP1 CER1 LRP5 INTU DVL2 LHX3 MYF5 LHX1 TCAP CHRD TSHZ1 ROR2 ALX4 GREM1 HOXA10 OVOL2 C6orf170 HOXA7 SKI GLI1 IRX2 SCMH1 OSR1 FZD5 PTCH1 KDM6A TBX20 BMI1 WNT5A EMX1 HOXD10 SIX3 MIB1 RELN PGAP1 HOXB2 FOXN4 SSBP3 PAX8 IFT140 PLXNA2 PRKDC SFRP2 HOXC13 BMPR1A XRCC2 GPC3 FRS2 GPR56 HOXA9 CYP26C1 MSX2 DMRTA2 HOXB8 FGF10 DUSP6 DNAAF1 PAX1 AURKA GBX2 HOXC6 HOXA6 PAX2 GLI2 HOXD4 HOXC10 WT1

GO_NEGATIVE_REGULATION_OF_EPITHELIAL_TO_MESENCHYMAL_TRANSITION Any process that decreases the rate, frequency, or extent of epithelial to mesenchymal transition. Epithelial to mesenchymal transition where an epithelial cell loses apical/basolateral polarity, severs intercellular adhesive junctions, degrades basement membrane components and becomes a migratory mesenchymal cell. FOXA2 FOXA1 HPN C18orf1 OVOL2 PPP2CA FUZ ADIPOR1 NOG TGFB2 SFRP1 TRIM62 STRAP DAB2IP PBLD SFRP2 MAD2L2 SMAD7 BMP5 TBX5 DACT3 VASN SDHAF2 NKX2-1 EFNA1

GO_CREATINE_METABOLIC_PROCESS The chemical reactions and pathways involving creatine (N-(aminoiminomethyl)-N-methylglycine), a compound synthesized from the amino acids arginine, glycine, and methionine that occurs in muscle. CKB CKMT2 CKM GATM CKMT1A SLC6A8 GHR CKMT1B GAMT STAT5A STAT5B

GO_POSITIVE_REGULATION_OF_ADAPTIVE_IMMUNE_RESPONSE Any process that activates or increases the frequency, rate, or extent of an adaptive immune response. FOXP3 TGFB1 TRAF2 MSH6 TBX21 RSAD2 IFNG LTA HPX UNG SASH3 CCL19 NCR3 RIPK2 PRKCQ EXOSC3 PVRL2 BTK TRAF6 IL6 STX7 HSPD1 XCL1 TFRC FZD5 B2M PYCARD NLRP3 CD28 IL12RB1 IL12B CD226 EIF2AK4 CD80 CD4 TNFSF13 ZBTB1 SLC11A1 SIRT1 IL12A IL27RA TNFSF13B ADA IL1B PRKCZ STAT6 MAP3K7 IL23R IL4 GATA3 WHSC1 C3 TNFRSF13C MALT1 FCER2 HLA-E P2RX7 PAXIP1 ANXA1 ZP3 CLCF1 FCER1G IL23A IL2 CCR2 IL6ST EXOSC6 SOCS5 TNF PVR NLRP10 FCER1A FADD TNFSF4 CD40 HLX

GO_PITUITARY_GLAND_DEVELOPMENT The progression of the pituitary gland over time from its initial formation until its mature state. The pituitary gland is an endocrine gland that secretes hormones that regulate many other glands. GLI1 GLI2 GHRH POU3F2 PITX1 SOX3 NKX2-1 PITX2 BMP2 WNT4 SALL1 DRD2 SOX2 FGF10 HES1 TBX19 GHRHR BMPR1A RBPJ ADCYAP1 PAX6 CDH1 SRD5A1 FGF8 GSX1 GATA2 POU1F1 NR0B1 MSX1 LHX3 ISL1 WNT5A BMP4 OTP NOG ALDH1A2 SIX3 PROP1 ETS1 KDM1A CREB1 DUOX2 SLC6A3

GO_MALE_MEIOSIS_I A cell cycle process comprising the steps by which a cell progresses through male meiosis I, the first meiotic division in the male germline. SYCP3 MOV10L1 BRCA2 BRDT REC8 C17orf104 HSPA2 ING2 DMRTC2 MEI1 CCNA1 C16orf73 DMC1 UBR2 TRIP13 RAD51C SLC2A8 SPO11

GO_POSITIVE_REGULATION_OF_INNATE_IMMUNE_RESPONSE Any process that activates or increases the frequency, rate or extent of the innate immune response, the organism's first line of defense against infection. UBE2N NLRC5 MYD88 COCH PLSCR1 PSMD8 PDPK1 TLR1 RPS6KA3 TAB1 TRIM6 PIK3C3 IKBKG PSMC2 LSM14A HSPD1 LGALS9 PAK1 RELB PSMD5 SH2D1A REG3G CTSS POLR3B PSMC1 PSMC3 POLR3F CLEC4C PSMB9 PSMB2 PSMB7 PRKACA TLR5 TLR3 PSMB4 IFNB1 SYK S100A14 NFKB1 UBE2K PAK3 PIK3AP1 PSMF1 CLEC4A AP1G1 DMBT1 IRF7 MAP2K6 HCK FCN1 CLEC4D PSMD11 PSMB8 PSMD13 CASP8 ICAM2 SH2D1B MAVS EP300 BTK CREBBP RPS27A TLR6 RPS19 ADAM8 TLR9 NCR3 PLCG2 TRAF3 PSME1 PSMA1 EREG TICAM1 POLR3C FYN PGLYRP3 NR1H4 PSMC6 PSMD12 FCER1G SKP1 UBA52 SCARA3 PSMA2 BIRC2 BIRC3 PSMD14 RPS6KA5 IRAK2 CADM1 KRAS ITGB2 TNFAIP3 PVR COLEC12 TLR7 POLR3D TLR2 TBK1 CYLD MAPKAPK3 TANK TNIP1 PSMA5 HSP90B1 PSME2 RIPK1 PSMA8 PGLYRP1 WNT5A PRKCE MALT1 CLEC7A PSME3 HLA-E PRKACG TLR4 CD1D UBE2D3 PSMB6 HMGB1 KLRC4-KLRK1 POLR3G HAVCR2 PRKCD DDX58 VAV1 TRIL PSMD1 PSMC5 LAMP1 PRKACB IL12B CD226 NFKBIA TNIP2 MARCO NRAS IL21 HMGB2 NOD2 PSMB3 CARD9 PYCARD PSMA6 PSMC4 FAM105B LYN UNC93B1 BTRC CD86 TXK TNIP3 PSMD9 PSMA3 IFNK MAPKAPK2 PSMD3 PGLYRP2 MED1 ICAM3 FFAR2 SLAMF6 PVRL2 TIRAP CTSK CUL1 TRAF6 HPX CARD11 PSMB5 PIK3R4 IFI16 IRAK4 PSMD10 RIPK2 PSMB11 PSMB10 IRAK3 LY96 SRC BCL10 PSMD4 CHUK PAK2 UBE2V1 LGMN TRIM5 TLR10 TAB2 RFTN1 CLEC6A CTSL1 UBE2D2 MB21D1 CD14 IRF3 TAB3 CCL5 FADD KLRK1 ITCH SIN3A LBP MMP2 RASGRP1 ITGAM CD36 IFIH1 TLR8 IKBKE PSMD7 FBXW11 CLEC4E IRAK1 RAF1 UBE2D1 CNPY3 TICAM2 IKBKB CTSB NLRC4 RELA UBB CD180 PSMD2 LAG3 GBP5 UBC IL12A MAP3K1 AIM2 STAT5B PSMD6 TMEM173 MAP3K7 CRTAM PSMB1 PSMA7 HRAS PGLYRP4 PSMA4 CD209 NOD1 PSME4

GO_ISOPRENOID_BIOSYNTHETIC_PROCESS The chemical reactions and pathways resulting in the formation of any isoprenoid compound, isoprene (2-methylbuta-1,3-diene) or compounds containing or derived from linked isoprene (3-methyl-2-butenylene) residues. MVK PMVK RDH10 DHRS9 BCMO1 DHDDS MVD IDI2 ALDH8A1 IDI1 NUS1 DPAGT1 GGPS1 HMGCR FDPS CYP1A1 ALDH1A2 RBP1 ISPD FDFT1 COQ2 ALDH1A3 HMGCS1 HMGCS2 PDSS2 PDSS1

GO_PROTEIN_STABILIZATION Any process involved in maintaining the structure and integrity of a protein and preventing it from degradation or aggregation. EP300 GTPBP4 USP2 STX12 SOX17 TELO2 TESC HSPA1B CDKN1A TAF9B WNT10B TSC1 MT3 IFI30 MUL1 CPN2 PHB CREBL2 SMAD7 PEX19 CDC37L1 A1CF CCT3 FLNA PDCD10 CHP HSPA1A HCFC1 HSPD1 CHEK2 GNAQ STK3 HPS4 ZBED3 PIK3R1 PTEN TSPAN1 HIST1H1B CCT8 DNLZ PIN1 SYVN1 HSP90AB1 AHSP WFS1 PHB2 PARK2 SAV1 RASSF2 PIM2 SEL1L ATP1B3 PEX6 IGF1 STXBP4 LAMP2 USP33 TCP1 SOX4 GOLGA7 NAA15 USP7 FBXW7 CCT6A PFN2 APOA1 MORC3 SUMO1 CCT4 SMO TMEM88 STK4 VHL UBE2B DVL3 CSN3 ZSWIM7 WIZ MSX1 FLOT2 AAK1 TAF9 USP19 LAMP1 PFN1 ANK2 DVL1 TNIP2 NLK PINK1 SMAD3 CDC37 PRKCD CRTAP STXBP1 BAG6 COG7 BAG3 PPIB FKBPL CCT2 CLU PPAP2B CREB1 CCT7 PARK7 NAPG USP27X COG3 GPIHBP1 PPARGC1A GAPDH PER3 ZNF207 HSP90AA1 NAA16 ATP1B1 MDM4 CCT5 USP13 DSG1 GTSE1 CALR LEPRE1 TBRG1 C19orf39 IFT46 ATP1B2

GO_SPLICEOSOMAL_SNRNP_ASSEMBLY The aggregation, arrangement and bonding together of one or more snRNA and multiple protein components to form a ribonucleoprotein complex that is involved in formation of the spliceosome. SRSF10 WDR77 GEMIN7 SNRPG GEMIN5 LSM4 SRSF12 TGS1 GEMIN6 GEMIN4 GEMIN2 CD2BP2 SNRPF SNRPD1 PRPF3 SMN2 SART1 PRPF6 SNRPB PRMT5 CLNS1A SNRPC C20orf4 SNRPD3 RBM22 PRPF8 PRPF19 PRPF31 GEMIN8 SART3 SNUPN DDX20 SNRPD2 STRAP USP4 PRMT7 SMN1 SNRPE

GO_REGULATION_OF_ENERGY_HOMEOSTASIS Any process that modulates the frequency, rate or extent of energy homeostasis. FLCN OPRK1 TRPV4 BMP8A NR1D2 LEPR NR4A3 CD36 PASK PRKAA2 METRNL PRKAA1 FOXO1 DLL1 PPARGC1A MLXIPL GHRL

GO_MITOCHONDRIAL_ELECTRON_TRANSPORT_UBIQUINOL_TO_CYTOCHROME_C The transfer of electrons from ubiquinol to cytochrome c that occurs during oxidative phosphorylation, mediated by the multisubunit enzyme known as complex III. UQCRH UQCRQ UQCRC1 UQCRC2 PMPCB UQCRFS1 UQCR11 UQCRB UQCRHL MECP2 CYC1 CYCS C11orf83 UQCR10

GO_MALE_MEIOSIS A cell cycle process by which the cell nucleus divides as part of a meiotic cell cycle in the male germline. TRIP13 KIF18A TEX15 C11orf20 FANCA EIF2C4 TDRD12 TDRD9 TEX11 DMRTC2 C16orf73 SPDYA MEI1 CCNA1 TAF1L MLH1 MAEL TDRKH BRCA2 SYCP2 BRDT MLH3 RAD51C SLC2A8 SPO11 C2orf65 UBR2 DMC1 PIM2 HSPA2 ING2 TEX14 DDX4 SYCP3 MOV10L1 REC8 C17orf104 LOC728637 CYP26B1 DPEP3 ASZ1

GO_CELLULAR_RESPONSE_TO_INSULIN_STIMULUS Any process that results in a change in state or activity of a cell (in terms of movement, secretion, enzyme production, gene expression, etc.) as a result of an insulin stimulus. Insulin is a polypeptide hormone produced by the islets of Langerhans of the pancreas in mammals, and by the homologous organs of other organisms. GCK ATP6V0C ATP6V0E2 ATP6V0B IDE ATP6V1G2 APRT TRIB3 ATP6V0A4 ATP6V1E1 SLC25A33 SRSF5 ATP6V0D2 HDAC9 IGF2 CCL2 ATP6V1E2 RAB10 CEACAM1 EEF2K PDPK1 KIAA0528 PIK3R2 CPEB1 RAB13 PDE3B SMARCC1 ATP6AP1 FOXO1 CPEB2 ATP6V1B2 KL SREBF1 GHSR PAK1 USF1 PLA2G1B ADIPOQ EIF4EBP1 SLC2A8 PRKCZ HDAC5 YWHAG GCLC GRB2 UCP2 SHC1 MYO5A GOT1 KIAA0889 ATP6V1D AKT2 FOXC2 PIK3R1 BAIAP2L2 ATP6V1H DENND4C SLC2A4 RAB8A PCSK9 AP3S1 PTPN2 SH2B2 ERRFI1 INPP5K WDTC1 PIK3C2A FER SELS PKLR PRKDC CAV2 ATP6V1A PIK3R3 ATP6V0D1 ZFP36L1 ATP6V1B1 STXBP4 ATP6V0E1 PDK2 SOCS7 PPARG FOXO4 PHIP BAIAP2 INHBB ATP6V1C2 SRD5A1 ATP6V1G1 APOBEC1 RPE65 VAMP2 APPL1 IGF1R IRS4 INSR ATP6V0A2 CAPN10 ATP6V0A1 PIK3CA PTPRA PPAT BAIAP2L1 GHRHR ATP6V1F SP1 SORBS1 PTPN1 PDK4 GSTP1 RHOQ XBP1 AKT1 GPLD1 STAT1 IRS1 EIF4EBP2 PRKCI GSK3A BCAR1 SLC9A1 IGFBP1 ZFP106 PCK1 ATP6V1G3 STAR SOS1 RAB31 LPIN1 SRC TCIRG1 NAMPT KAT2B ENPP1 TBC1D4 ATP6V1C1 PARP1 IRS2 INS GAB1

GO_T_CELL_MEDIATED_IMMUNITY Any process involved in the carrying out of an immune response by a T cell. FOXP3 CTSH DLG1 CD46 SLC11A1 RFTN1 ICAM1 RAET1E MICA MYO1G SLAMF1 BTN3A3 JAG1 CRTAM CADM1 CD8A BTN3A2 CD55 IL31RA MICB KDM5D GNL1 RAB27A IL4 GATA3 DENND1B CTSC EMP2

GO_LEFT_RIGHT_AXIS_SPECIFICATION The establishment, maintenance and elaboration of the left/right axis. The left/right axis is defined by a line that runs orthogonal to both the anterior/posterior and dorsal/ventral axes. Each side is defined from the viewpoint of the organism rather of the observer (as per anatomical axes). AHI1 EPB41L5 HSPB11 CITED2 SETDB2 MNS1 PITX2 DLL1 IFT172 SMO PKD1L1 ARL13B NOTCH1

GO_EMBRYONIC_EYE_MORPHOGENESIS The process occurring in the embryo by which the anatomical structures of the post-embryonic eye are generated and organized. FZD5 FOXF2 TWIST1 PAX2 TH AXIN1 FBN2 SP3 RARG WNT16 BMP7 MFAP2 RARB HIPK2 TBX2 FRS2 ZEB1 PROX1 ARID1A SOX11 STRA6 PAX6 EFEMP1 TFAP2A VAX2 SIX3 HIPK1 WNT5A PHACTR4 FBN1 KDM2B FOXL2 ALDH1A3

GO_POSITIVE_REGULATION_OF_CELL_DIVISION Any process that activates or increases the frequency, rate or extent of cell division. MRGPRX2 IL1B TAL1 FGF9 CIT VEGFA SVIL AURKB OPN1MW2 SSTR5 FGFR2 OR2A4 CSPP1 FGF1 PRKCE PTN AURKC PDGFC YBX1 PROK1 GIPC1 GKN1 RXFP3 SPAST KIF20B SIRT2 FAM59A EREG FGF4 OR1A2 PDGFB VEGFC TGFA RHOA PDGFA ECT2 DRD3 PKN2 MDK TAS2R13 PDGFD TGFB3 CAT KIF14 SHH OPN1MW FIGF FGF2 THBS4 RACGAP1 CDC25B FGF8 ITGB1BP1 TGFB1 IL1A CDC6 PGF TGFB2 ZNF16 FGF3 IGF2 FGF7 TAS1R2 FGF6 NKX3-1 BTC FGF5 MACC1 PKP4 KIAA1383 DRD2 VEGFB CUL3 CENPV HTR2B KIF23 CDC42 PPBP OPN1LW OSM CXCR5 KIF3B

GO_REGULATION_OF_LONG_TERM_NEURONAL_SYNAPTIC_PLASTICITY A process that modulates long-term neuronal synaptic plasticity, the ability of neuronal synapses to change long-term as circumstances require. Long-term neuronal synaptic plasticity generally involves increase or decrease in actual synapse numbers. GRIK2 SNCA GRM5 RAB8A NEURL EPHB2 KCNJ10 NETO1 SYNGAP1 RAB11A NF1 SYP AGT DLG4 HRAS KRAS CAMK2B SHANK3 SYNGR1 GRIN1 NPTN DRD2 KIT

GO_NEGATIVE_REGULATION_OF_TUMOR_NECROSIS_FACTOR_MEDIATED_SIGNALING_PATHWAY Any process that decreases the rate or extent of the tumor necrosis factor-mediated signaling pathway. The tumor necrosis factor-mediated signaling pathway is the series of molecular signals generated as a consequence of tumor necrosis factor binding to a cell surface receptor. APOA1 GSTP1 F2RL1 PYDC1 ZNF675 PIAS4 PYDC2 GAS6 NOL3 TRAIP RFFL ADIPOQ PTPN2 PELI3 NR1H4 NLRP2P

GO_SUBPALLIUM_DEVELOPMENT The process whose specific outcome is the progression of the subpallium over time, from its formation to the mature structure. The subpallium is the base region of the telencephalon. INHBA RARB DRD2 GSX2 BBS2 CNTNAP2 SHANK3 OGDH ALDH1A3 MKKS SLITRK5 DRD1 HPRT1 FGF8 DLX1 ASCL1 BBS4 GLI3 FOXP2 BCL11B SECISBP2 DLX2

GO_CELLULAR_RESPONSE_TO_OSMOTIC_STRESS Any process that results in a change in state or activity of a cell (in terms of movement, secretion, enzyme production, gene expression, etc.) as a result of a stimulus indicating an increase or decrease in the concentration of solutes outside the organism or cell. RCSD1 TRPV4 SERPINB6 STK39 ZFP36L1 CAPN3 TSPO XRCC6 MYLK CAB39 SLC12A6 RELB SCARA3 XRCC5 ARHGEF2 SLC2A4 CSDA DDX3X PKD2 OXSR1 ERRFI1 OSR1 AQP1 EPO

GO_RESPONSE_TO_INORGANIC_SUBSTANCE Any process that results in a change in state or activity of a cell or an organism (in terms of movement, secretion, enzyme production, gene expression, etc.) as a result of an inorganic substance stimulus. SDC1 PTGES SLC30A10 ITPKB FGF23 TFR2 RYR2 IL1A MDM2 TPH2 SLC30A3 CASP8 KHK SLC26A5 PENK IL18RAP PRDX2 MT1F FABP4 DTYMK SLC6A1 DNM2 SERPINF1 EDN1 FOXO1 SOD2 KCNMB3 SPAG16 EIF2AK3 MST4 CBX8 PRDX3 IL6 MTL5 EEF2K PEF1 EIF4A3 EIF2S1 GIP HMOX1 DRD2 CALM3 KCNA1 APTX THBS1 ZACN CYP11B2 TXNIP SCGB1A1 PARK2 MT1A BCL2 ATP7A HOMER1 AREG KCNIP2 SLC30A7 MT1M IQGAP1 ACER1 NEDD4 EGLN1 CA2 ETS1 CEBPA CPNE6 NEDD4L APOA4 SLC40A1 STAT6 ALOX5AP S100A8 HAMP GCLC UCP2 PRKAA1 DPEP1 KPNA4 SLC25A12 CYP11B1 ANXA7 HDAC2 FBP1 SYT1 ANXA1 PRDX1 MAPK9 PPP3CA TH D2HGDH SLC30A1 TNFSF4 ENDOG EEF1A2 NTRK1 PPP1R15B CD9 TSPO AKR1C3 MTR APOB PKLR LCE1D ICAM1 ZNF277 APOBEC1 TFAP2A TRAF2 CCNB1 NET1 ATF2 RPLP0 ARG1 NUDT1 KCNK3 FGG FGA HBA2 HBA1 MT2A NR4A3 KCNMB2 KCNMB4 ZNF658 GUCA1A ATP5D AQP9 CYB5A FOSB PON1 GLRA1 LONP1 ASS1 ANK3 TRPM2 SLC25A13 SNCA G6PD SLC25A24 PTH MEF2A CAV1 CAPN3 B2M CPNE3 TRPC3 TNFRSF11B FOS AKR1B1 FOSL1 ATP13A2 CASP3 CDK4 SLC34A1 MNAT1 FKBP1B SLC8A1 EPB49 CHP2 SCN5A ALOX15 CACNA1H SLC14A2 PRNP CACNA1G AQP3 CPS1 CCND1 SIRT1 GPLD1 STAT1 HSF1 ANXA11 TRPC1 CST3 KCNMA1 HAAO EEF2 HSD17B1 KIAA0146 EIF2B5 ADCY10 DNMT3A BAD FECH SLC30A8 ATP7B KCNC2 CYP2R1 KRT8 SRC PKD2 CALM1 FAS CCNA2 ATF4 ABAT KCNA5 C12orf5 BRAF CRHBP KCNC1 TRPC6 S100A13 EGFR PAM AVPR1A NEFL PDGFD CYP2E1 GNAO1 PARP1 SHH MTF1 TRPA1 KRT14 JUND AQP1 TERT LDHA HVCN1 MT3 WNT10B TTN SLC30A6 CASQ2 RYR3 CYBRD1 BAK1 KLF2 UCP3 APBB1 SIPA1 BMP6 CDK2 MT1E SLC11A2 SORD AQP2 CCL19 NQO1 TSHB SLC25A23 MT1H PDCD10 SEC31A PTCH1 SLC30A5 APP ALG2 HSPD1 XRCC4 DUSP1 BSG HSPA5 TXNRD2 GPI ABCC8 AIFM1 CACYBP OGG1 CSF2 HFE NDRG1 IL18BP ITPR3 ADNP SLC30A4 PDGFRB NEUROD2 MT1G GUCY1B3 ADAMTS13 GDI1 WNT5A ATP2B4 STK24 GLRX2 NOS3 P2RX7 PTEN CYP1A1 BLM AHCYL1 CNGA3 CRIP1 FXN CPNE7 PDX1 PTK2B PAX2 RPS3 FABP1 PGAM2 KDM6B PPP5C KCNMB1 CYBB MB PPIF ECT2 CLIC4 ABL1 LOXL2 CCR7 FGB TNFAIP3 CRYAB CAT GNRH1 CDK1 ACO1 CYP1A2 FUS ASCL1 ZNF580 EZH2 NFE2L2 MMP3 JUN LIG4 GGH CD86 GLRA2 TFAP2B ADCY1 SLFN14 HP HNRNPD RAD51 CDKN1B CASR MT1B GIPR ATIC APEX1 STK25 IGFBP2 ALAD SOD3 PDCD6 ABP1 SLC6A3 BGLAP PRKCD FMR1 ID2 PLEKHA1 TNNT2 COL1A1 MAP3K5 LRRK2 GART FSTL3 GSS SLC30A2 CPNE1 ERCC6 VCAM1 RHOB TRPV6 SOD1 CPNE2 BECN1 PTGS2 SLC41A1 CUTA SETX HMGCS1 SPARC MAPK7 CDH1 MEF2C ATF1 TAT ADA PARK7 CASP9 MYOG PPARGC1A STIM1 ZC3H12A CALM2 RELA PPP2CB MAOB BNIP3 MT1X LRP11 S100A16 IL4 GPX1 C20orf111 TF PCNA CREB1 TNNC1 PCGF2 C1QA KLF4 AXL CALR QDPR IMPACT ALS2CR8 NFATC4 HDAC6 AANAT HBB ACTA1 OTC RASA4 MPO COL18A1 ATRX STAR CPOX JUNB ENTPD6 ADAM9 CD14 RASGRP2 NPC1 PLN CAMK2D ADPRHL2 TFF1 MLL KCNH1 CYP1B1 CCS

GO_ESTABLISHMENT_OF_PROTEIN_LOCALIZATION_TO_MEMBRANE The directed movement of a protein to a specific location in a membrane. PACSIN1 ACTN2 ATG4A RPS21 KRT18 RPS23 TSPAN15 COX18 RPL12 RPS29 PEX19 TOMM7 RPL18 RTP4 PKP3 BMF RPS20 TIMM22 RPL37A RAB3GAP2 RPL22 SRP54 RFTN2 RPL26L1 GOLGA4 ATG4D ZFAND2B AMN TSPAN17 FAM126A CLASP2 RPL7 RPL3L BBS1 WRB TRAM2 RPS16 GAK TOMM22 EFR3B RPL14 PIP5K1A ATG4C GOLGA7 EFR3A INPP5K FLOT1 MACF1 TRAM1 CD24 RILPL1 UBA52 RPS2 PEX26 MYO1C TSPAN33 RPS4Y1 RPS8 CLIP1 RILPL2 EHD3 RPS4X ROCK2 VAMP4 RPL41 CHMP4B RPL18A SLC9A3R1 BBS2 BAX JUP GRIN3B RTP2 RTP3 GOLGA7B RTP1 CACNB2 PEX5 ADORA1 GET4 BAG6 ANK3 RPL36 VAMP3 KCNB2 RPL29 RPS9 VPS35 SRP72 RPL6 NFASC SPTBN4 TIMM10 RAB3GAP1 RPLP0 FLOT2 SRP68 RPL23A ICMT RPL8 NUP54 HSP90AA1 SSR1 RPS24 EGFR PARD3 ATP1B1 RPL10 RPL7A SMURF1 SEC63 SRP19 ASNA1 TSPAN14 RPLP1 RAB26 RAB11B BRAF RPS15A IFT20 RPS5 RPS27 CAV3 RPL28 BAD CHMP4A PRKCI GCC2 RPL4 IKBKB TSPAN10 RPS25 BSG GAS6 NDUFA13 REEP1 F11R RAB13 PTCH1 RPL39 RAB10 WNT4 RPL27A RPS7 OPTN MYADM RPS27A RPS19 RPL23 RPS10 SSR2 TESC RPL27 RPL19 RPS6 ROCK1 ARFRP1 SEC61A1 PKP2 RPL30 BID NUP155 TNF PACS1 RPL11 RPS11 RAB3IP RPL10A BCL2L11 RPL9 RPS3 MICALL1 RAB34 LAMA5 ANXA2 RPL32 CDH2 SPTBN1 REEP2 SRPR RPL36A RPS13 SDCBP GOLPH3 RPS15 PALM TTC7A ATG3 RAB8B RPL13 SYS1 KCNB1 ANK2 TMED2 SAMM50 MOAP1 RPL24 PACS2 VAMP5 TTC7B RPL15 PRKG2 RABGEF1 RPL3 ATG4B ARL6IP1 RPS28 SRPRB TRAM1L1 RPL13A TNFRSF1A TAOK2 UBL4A NCF1 RPL5 SSR3 VAMP2 S100A10 RPLP2 EZR TSPAN5 NSF NPC1 SEC62 COLQ PEX16 RPL34 CHM RPL37 ANK1 RPL21 RPL35 NKD2 RPSA WNK3 RPS17 RAB11A ARL6 TIMM9 C2orf85 RFTN1 C14orf49 SRP14 SRP9 TMEM150A RAB31 RPS3A HSPA4 MPP5 RPL35A RPL31 RPL17 RPS12 RPS14 KIF13A VAMP7 RDX RPS18 PEX3 CDH1 RPL26 OXA1L BLZF1 PMAIP1 RPS26 RPL38

GO_NEGATIVE_REGULATION_OF_DEPHOSPHORYLATION Any process the stops, prevents, or reduces the frequency, rate or extent of removal of phosphate groups from a molecule. DLG3 MKI67IP TNF WNK1 ELFN2 PPP1R16B ELL YWHAE PCIF1 RGN SH2D4A MYO1D INPP5K PPP4R4 PPP1R35 KIAA0430 PPP1R36 MPHOSPH10 PPP1R15A RBM26 NCKAP1L GRXCR1 CEP192 CRY2 MASTL IQGAP1 C19orf2 IKBKB ROCK2 TSKS DLG2 FKBP1A FKBP1B FARP1 SWAP70 ZCCHC9 SPRED1 GPATCH2 PKMYT1 PPP2R4 ZFYVE1 RRP1B CASC5 CHP LMTK3 SYTL2 CD2BP2 CCDC8 PPP1R27 SFI1 FKBP15 PPP1R11 PPP1R37 SPOCD1 CNST BAG4 IGBP1 RIMBP2 SEMA4D PPP1R39 SLC7A14 YWHAB TMEM225 TGFB2 ELFN1 TIPRL UBN1 WDR81 CAMSAP3 TMEM132D PPP1R26 CSRNP2 KIAA1244 CSRNP3 PCDH11X ROCK1

GO_NEGATIVE_REGULATION_OF_INFLAMMATORY_RESPONSE Any process that stops, prevents, or reduces the frequency, rate or extent of the inflammatory response. TNFAIP8L2 C1QTNF3 CD276 TNFRSF1A RPS19 APOA1 KRT1 NPY5R SOCS3 SERPINF1 TNFRSF1B NLRP6 PPARA SPN PTGIS FAM105B MMP26 ACP5 CHID1 NT5E NOV ELANE ISL1 FOXP3 PPARG IL20RB TEK FAM132A IL12B PPARD GSTP1 GHSR IL10 RABGEF1 MEFV DUSP10 NLRP3 IL2RA NFKB1 WFDC1 CYP19A1 PTGER4 SMAD3 CNR2 IRG1 PSMB4 NLRX1 METRNL PRKCD SHARPIN PYDC2 GHRL ABR O3FAR1 RORA ADORA1 PROC GPX1 IL4 PGLYRP1 GATA3 IL22RA2 NDFIP1 ETS1 GBA ASH1L KLF4 HGF APCS NR1H3 ADCYAP1 MAPK7 MVK APOD SAA1 AGER ADA APOE ADIPOQ NLRP12 ZC3H12A SMPDL3B INS IL1R2 FEM1A TNFAIP3 PBK SOCS5 CX3CR1 FOXF1 CUEDC2 SELS CALCRL BCR GPER C5orf30 IL2 GPR17 PTPN2 NR1H4 TYRO3 CHRNA7 TNFAIP6

GO_REGULATION_OF_BILE_ACID_METABOLIC_PROCESS Any process that modulates the frequency, rate or extent of bile acid metabolic process. PANK2 C10orf112 FGF19 PROX1 SIRT1 NR1D1 STAR CYP7A1 STARD4 NR1H4 FGFR4

GO_DNA_BIOSYNTHETIC_PROCESS The cellular DNA metabolic process resulting in the formation of DNA, deoxyribonucleic acid, one of the two main types of nucleic acid, consisting of a long unbranched macromolecule formed from one or two strands of linked deoxyribonucleotides, the 3'-phosphate group of each constituent deoxyribonucleotide being joined in 3',5'-phosphodiester linkage to the 5'-hydroxyl group of the deoxyribose moiety of the next one. WRNIP1 NBN PHB EXO1 RMI2 USP43 POLB WRN ACD UBA7 POLD3 POLE2 RFC5 POLD4 TERF1 POLI UBE2L6 LIN9 PALB2 RFC1 POLE3 POLE4 RPA2 SPATA22 MRE11A XRCC3 LIG3 RFC2 RAD51 CCDC111 TK2 RPS27A ATM LOC100133495 CTGF CDKN2D POLQ TEX12 SYCP1 POLH POLA1 TERT PAPD7 C1orf124 KAT5 WRAP53 NOP10 RCHY1 REV1 RAD51C BRCA1 TRIM25 LIG4 PAPD5 TERF2 ERVK-6 CENPF POLG PTGES3 LOC100128274 CHRAC1 ERVK-7 C1orf86 ERVK-8 BRCA2 VCP KIAA0101 POLD1 DTL POLK RAD51AP1 XRCC2 ERVK-10 NPLOC4 POLG2 ISG15 SMG7 GAR1 PINX1 RAD50 BRIP1 POLL TK1 POLN DNTT POLD2 USP10 UBA52 HMBOX1 RMI1 RPA3 REV3L LIG1 DNA2 BLM UFD1L PCNA TOP3A MAD2L2 RPA1 RAD51D RFC3 POT1 PPIA DKC1 MYBBP1A TEP1 POLM TYMS RBBP8 RFC4 NHP2 ZBTB1 POLA2 TNKS1BP1 RAD51B SIRT1 UBC TERF2IP BARD1 POLE UBB

GO_ADHERENS_JUNCTION_ORGANIZATION A process that is carried out at the cellular level which results in the assembly, arrangement of constituent parts, or disassembly of an adherens junction. An adherens junction is a cell junction at which the cytoplasmic face of the plasma membrane is attached to actin filaments. CDH3 ARHGAP6 RAB8B WHAMM BCL2 CDH12 RASSF8 RHOD CDH1 CDH13 CDH15 CDH10 PTK2B CDH2 ARHGEF7 CDH11 CADM2 HIPK1 NUMB JUB LAMA5 FERMT2 SRC TRIP6 DLC1 ACTN1 ZNF703 ITGA2 PTPRK THY1 TBCD PIP5K1A ANG CDH24 PVR CADM1 PVRL4 CDH7 TESK2 PVRL3 DLG5 CTTN PKP2 MLLT4 CDH9 RCC2 RAMP2 CDH5 PIP5K1C CDH17 CDH4 PVRL2 TAOK2 CDH8 CADM3 PDPK1 VCL CTNNA1 CDH6 CTNND1 JUP PVRL1 DSP SMAD7 CDC42 CSK ACTN3 NUMBL CDH18 SORBS1 ACTN2 CTNNB1

GO_CATECHOLAMINE_BIOSYNTHETIC_PROCESS The chemical reactions and pathways resulting in the formation of any of a group of physiologically important biogenic amines that possess a catechol (3,4-dihydroxyphenyl) nucleus and are derivatives of 3,4-dihydroxyphenylethylamine. DAO HAND2 PAH DBH DDC INSM1 PNMT HDC GCH1 SNCA TH TGFB2 GATA3 NR4A2 GPR37 AGTR2 SLC6A3

GO_REGULATION_OF_FEEDING_BEHAVIOR Any process that modulates the rate, frequency or extent of the behavior associated with the intake of food. MC3R MC4R INSL5 NPY2R LEPR MTOR NAPEPLD STAT3 NR4A3 TRH UCN RXFP4 TACR3 AGRP EIF2AK4 QRFP C16orf80 CNR1 INS RETN

GO_MUSCLE_CONTRACTION A process in which force is generated within muscle tissue, resulting in a change in muscle geometry. Force generation involves a chemo-mechanical energy conversion step that is carried out by the actin/myosin complex activity, which generates force through ATP hydrolysis. MKKS GNAO1 ATP1B1 RYR1 CAMK2D KCNE1 TRIM72 MYL4 TNNT1 MYH8 MYL9 NEB EMD TMOD3 PLA2G6 HSP90AA1 P2RX6 ACTA1 ADRA1A CHUK ATP1A2 ANKRD2 KCNA5 EDN2 CALD1 SMPX MYOF CHRM3 SCN4B CALM1 SLC6A8 EEF2 SCN7A KCNMA1 DYSF TNNC1 NOS1 EDN3 SLC9A1 TNNT3 ASPH CAV3 TNNC2 PTGER3 MYOM1 SSTR2 MYBPC2 MYL12A MYLPF SMTN JSRP1 TRPV1 TNNI1 CALM2 DMPK SORBS3 FKBP1B TPM3 EDNRA MYH14 SLC8A1 TPM1 CACNA1H SCN5A DRD1 HCN4 ACTA2 MYOT STAC TACR2 RPS6KB1 CHRNE BBS2 KCNJ2 ANK2 MYH3 TLN1 TPCN2 MYLK SORBS1 TNNT2 NKX2-5 PIK3CA MYL6 CHRND CACNA1C CACNB2 MYH6 TMOD1 VCL ATP1A3 LMOD3 MYL7 GLRA1 UTRN VIPR1 TPM2 HTR2A GJC1 RCSD1 MYOM2 MYL1 KCNE1L LMOD2 MYL12B GJA5 MYH4 P2RX3 CCDC78 LTB4R CSRP3 GDNF KCNE2 KCNH2 CHRNB4 ATP8A2 TNNI2 CRYAB GPD1L ATP1A1 LMOD1 SLMAP AGT PPP1R13L MYL10 GAMT CACNG1 NUP155 KCNQ1 MYLK2 STAC3 MYL3 ALDOA SCN2B SRSF1 ARHGEF11 MB MYH1 FXYD1 ITGB5 PIK3C2A NDUFS6 CACNA2D1 FGF12 CHRNA1 PGAM2 HSBP1 P2RX2 UTS2 CHRNB1 GAA MYL5 DMD TBX20 TPM4 ADRBK1 MYBPC3 HTR7 HRC KCNJ12 NEDD4L ROCK2 CKMT2 BDKRB2 HOMER1 KCNIP2 SSPN MYH13 SGCA TNNI3 CHRNG SULF1 COL4A3BP RAP1GDS1 OXTR MTOR TRIM63 SYNM ACTN2 VEGFB SNTA1 DRD2 CALM3 TAZ DES ACTN3 HTR2B SMAD5 ACTC1 MYL6B SNTB1 MYH2 STBD1 GALR2 MYH11 TMOD2 ITGA1 PDE4D EDNRB GJA1 PABPN1 CHRNB2 EDN1 MYL2 NMUR1 SULF2 TTN PKP2 DLG1 CASQ2 TRDN SCN4A SCN3B HTR1D ARG2 MYH7 DTNA ROCK1 KBTBD10 TCAP SCN1B CLCN1 MYBPC1 CACNA1S PXN TMOD4 VIM ACTG2 MAP2K6 RYR2

GO_BETA_CATENIN_DESTRUCTION_COMPLEX_DISASSEMBLY The disaggregation of a beta-catenin destruction complex into its constituent components. APC DVL2 FRAT2 LRP5 AXIN1 FZD1 GSK3B WNT8A LRP6 FZD5 FRAT1 WNT3A DVL3 WNT8B FZD2 FAM123B CTNNB1 CSNK1A1 PPP1CA CAV1 WNT1 DVL1

GO_POSITIVE_REGULATION_OF_TYROSINE_PHOSPHORYLATION_OF_STAT5_PROTEIN Any process that activates or increases the frequency, rate or extent of the introduction of a phosphate group to a tyrosine residue of a Stat5 protein. EPO IL23A FYN ERBB4 IL2 JAK2 CSF2 IL31RA GH1 IL23R IL12B IL4 KIT IL3 IGF1 GHR

GO_REGULATION_OF_TRANSFERASE_ACTIVITY Any process that modulates the frequency, rate or extent of transferase activity, the catalysis of the transfer of a group, e.g. a methyl group, glycosyl group, acyl group, phosphorus-containing, or other groups, from one compound (generally regarded as the donor) to another compound (generally regarded as the acceptor). Transferase is the systematic name for any enzyme of EC class 2. TNIK IKBKAP RGN HGS RGS14 TOM1L1 AVPI1 FLRT2 LRRTM3 PAK2 F2R TAB2 CSNK2B DDR2 PFKFB2 PDE5A FAM150A PDE6G CTC1 CCL5 INCA1 TAB3 KAT2B TSC2 ANKRD54 PTPRJ PARP16 FPR1 GSK3B RIPK3 PSMD7 PARK7 CRKL FAM58BP RAF1 IRAK1 IRS1 TNKS ACE MARK2 PSMD6 PHPT1 TIAM1 CLU NCK2 PROK1 MAP3K7 VANGL2 PDGFC STK11 PDCD4 GBA MAP3K3 PSMA4 KLF4 EDN3 ZAK BCCIP PSMB1 HRAS RAPGEF1 PLCG1 PAQR3 PIK3CB NUP62 PRKAB2 DCUN1D5 SPDYE4 DCN CHTF18 AXIN1 CSF1R MOS CRK ARHGEF5 LCP2 SLC27A1 NOX4 C18orf26 RFC5 ACD ANAPC4 DUSP10 PSMA6 PINK1 LEP PROK2 COX11 DVL3 EZH2 CBL PSMA3 BTRC MAGEC2 CDC6 MEN1 INSR RFC2 S100A12 CDK5RAP3 RAP2B ERBB2 PIK3R4 NCK1 DSCC1 CDK5R2 LRRC4 IRAK3 ATG14 PSMD10 RIPK2 NKX3-1 IL23A RPS3 RAC2 LRRC4B ADCY6 ZP3 RHOA ADAR MAGED1 CDK7 PPP1R1B PPP1R9A PSMD12 CALCA PPP1R3G PDGFB ANAPC7 CDC16 CDC25C HMBOX1 AZU1 CHRNA7 TPX2 PRKAR2B CCR7 RANBP2 TNF WNK1 LRRC19 SFRP2 FRS2 FCER1A SPDYE5 PIK3R6 BAG5 POT1 MAP2K5 EPHA8 YWHAG CD74 MAPKAPK3 IL1B SOCS6 APOE AKAP13 ITGB3 CCNH SMYD3 MAP2K4 STAP1 PODNL1 PSME2 DAB1 FGF1 WNT5A SESN2 CCNY STK3 P2RX7 RELN PKD1 NCKAP1L PTEN BLM ADCY2 UNC119 EPO UBE2N BUB3 FZD5 PSMD8 GCG CCNG1 CCNE2 PRKAB1 APP C1orf88 GTF2H1 FBXO7 GREM1 PSMC3 HSPA5 STRADB ADCY3 SPRED1 ZFP36 DUSP14 VAV3 OBFC1 GRM4 PROX1 SH3BP5 PKIB PSMF1 EIF4A2 MT3 TNFRSF10B MAPK15 TTN PSMB8 FLRT1 DVL2 TP53 BAK1 CDKN2B VAPB RPS27A C13orf15 FZD10 ATM BGN GTPBP4 DNAJC3 FAM58A GH1 TESC ZNF675 GADD45G ELP4 OSBPL8 NF1 MMD2 LRP6 BMP7 TGFA PRKAG1 STOX1 C12orf5 SPDYE6 CLSPN F2 DUSP8 INS ADORA2B RGS4 EGFR LAMTOR3 MYOCD IRS2 SPDYE3 C21orf7 ADAM17 PRLR WASH1 ANG RFC3 SNX6 CEP85 AGER PPM1E WNT3A TFAP4 TOPORS TP73 CCNT1 UBE2D1 CRIPAK SLC11A1 NF2 SIRT1 SPAG9 AKT1 ZP4 PRKAG2 AURKB SOCS4 ADRA2B MADD CCNL2 C1QTNF2 HGF BAD IL23R CNPPD1 SERPINB3 MAPK8IP3 KIDINS220 PLK1 FGD2 FEM1B LIMK1 HHEX GHRL TNFRSF10A LATS1 SNCA ADORA1 CBLC CIB1 KIAA1804 PEA15 SLC9A3R1 MAPK1 PSEN1 DVL1 CCNT2 ASPN MAP3K15 MAD2L1 ADRA2C ANAPC16 LAX1 DAB2IP BAX MNAT1 STK4 CKS1B CDC27 PSMC4 SPRY1 HERC5 MAGEA2B WRAP53 SRCIN1 UCHL1 PPP1R1A PIK3IP1 TRAF2 TGFB1 ANAPC2 TGFBR1 UVRAG WNT11 SMPD1 PPM1F ERN1 TIRAP HMGCR FBXO43 PKIA SERINC2 IGF1R PSMB10 SH3BP5L CCT4 GADD45A STUB1 WEE2 INPP5K RGS3 SERINC5 AMBRA1 UBA52 FBXW7 UBE2I SKP1 PSMA2 LRRC66 ARRB1 CCNYL2 IGF1 EEF1A2 MAP3K4 CSF1 UBASH3B MAP3K11 SOCS5 IRAK2 TCP1 SFRP1 HSP90AB1 LTF ADCYAP1 PIK3CG MAP3K14 TNFAIP8L3 SAA1 TNFSF15 PARK2 CCND3 PSMA5 NPRL2 PLA2G1B C5 MALT1 DRD4 HSPA2 MAP2K2 DEPTOR PIK3R1 CEBPA GHR TLR4 FBXO5 TRIB2 GNB2L1 SHC1 FGFR1OP DCUN1D1 NRG1 MAP2K1 PIK3R2 MIDN IL6 CEACAM1 CDKN1C GPRC5B PKMYT1 CALM3 PPP2R3C MAPK8IP1 SASH1 HSPH1 HTR2B GRHL2 DCUN1D4 PSMB7 ARRDC3 CHCHD10 PTPN11 CAMKK2 TLR3 MTOR MAP3K8 CDKN1A GADD45B CDKN2C GBAS SPDYE1 RBL1 PPP1R1C DUSP12 FLT3 DTNBP1 EIF2AK2 MAP4K5 PTPLAD1 PRKRIP1 BMP4 DUSP9 OBFC2B DNAJA1 CAMK1 CDKN2D SOCS1 TLR9 ADAM8 RAC1 LAMTOR2 SERTAD1 SHB CDKN3 ARRDC4 WWTR1 PIBF1 MAP3K2 CSPG4 MAPRE3 PDGFRA CDC25A IQGAP3 ADRA2A HIPK3 TDGF1 GAB1 CD40 FEM1A ADAM9 GRM1 KIF14 TRIM27 TRIB1 GSK3A ERP29 DKC1 MAPK7 MVP CCNYL1 RB1 PSMD2 UBC CALM2 KITLG UBB ZYG11A GPS2 PPP2R5A TSG101 FAM20A DUSP16 LATS2 UBE2E1 NTRK3 EPM2AIP1 PSMA7 PRKCD TRAF4 NGF NRK CCNC MAPK3 PIK3CA GNAI2 RTN4RL2 MAP3K5 NEK2 PSMD1 GCKR EFNA5 LRRK2 MRE11A CISH MAP3K13 CHAD SPRED2 FZD4 TARBP2 GMFG GSTP1 PIK3R5 ERCC6 ANAPC10 PPP2R1A CD4 FLT1 SOD1 WNT7B CDK5 TERF1 CCNE1 CCNK CDK1 H2AFY CAMK2N1 CKS2 PKN1 FGR PPARG ELANE MAS1 ADCY1 MAPKAPK2 PSMD3 NEK10 CBLB CXCR4 CORO1C KIAA0226 TAOK2 CDKN1B JTB PSMB5 MAGEA2 PTPN22 PYDC1 GPS1 PRKAR1B PSMB11 PSMA1 THY1 FAM150B COPS8 PSME1 PTPRC ERRFI1 FZD8 STRADA PINX1 DIRAS3 DUSP6 CXCL10 PTPN2 PSMC6 APC CAMK2N2 ECT2 NPFFR2 SOCS7 CDK5R1 CCDC88A LRRC15 ABL1 PPP2CA HYAL2 CDKN2A FGF10 ZYG11B KRAS PIK3R3 TNFAIP3 CDC26 DIRAS2 DBF4B FGF2 PARM1 AGT BIRC7 LRRC4C S1PR2 PRKCZ ADNP JAK2 DUSP19 RFC4 MAP3K12 TXN PDGFRB KIAA1199 NEURL RASSF2 ANAPC5 PSMA8 AKT1S1 MAP2K3 RIPK1 CHI3L1 ATP2B4 CAB39 RPTOR MAPK11 CDC20B LPAR1 TRAF7 ADCY8 BMI1 PTPN6 CHORDC1 PTK2B PDCD10 GCN1L1 MSTN GAS6 TAB1 NRG3 CHP ILK TSPYL2 MMD TGFBR2 FZR1 IKBKG MST1 PSMC2 PAK1 ZFYVE28 DUSP1 PSMD5 PSMC1 MAPKAPK5 PSMB2 CSK SMAD7 SYK SHC2 PAK3 PSMD11 MAP2K6 TSC1 CENPE LRP5 MUL1 HSPB1 VAV2 ELP3 LPAR3 MAPK10 DLG1 ADCY9 ADRB2 CDK2 PFKFB1 CDC23 PLCE1 CD40LG PRKAR1A PRKCQ MAP3K10 CYR61 CCND2 IGF2 CCL19 TNFRSF11A MAP3K9 RPLP1 SERINC1 MIF IBTK SRC MDFIC NTF3 MDFI DIRAS1 CALM1 PKD2 DOK7 CCNA2 ANGPT1 AFAP1L2 PSMD4 SORL1 BRAF BMP2 MUC20 LMO4 PDGFA PPEF2 CERS1 MAP3K6 PDGFD VAC14 TEN1 ADCY5 FAF1 ACVR2B MAPK14 RTN4R PPP1R3F SDC4 SPDYE2L ADCY7 DUSP4 RASGRP1 ERBB3 VEGFA C11orf51 CCND1 FGFR3 UBE2C EPHA1 CAV3 MAP3K1 MBIP RGS2 ALS2 CCT2 CDC14C MAD2L2 LDB2 EPHA4 NOD1 PSME4 ANAPC1 PIH1D1 RBL2 C5AR1 CDC37 SOCS2 CDK12 SFN TRPT1 CARTPT ABI1 LDB1 LRRTM4 DAXX MAP4K1 ACSL1 PRKACB CCNB3 IL12B SPDYA KIT PSMC5 DCUN1D3 NVL NRBF2 CCL21 CAV1 CD300A PTPN1 AIDA PIF1 FOXA2 SPDYE7P NOD2 PSMB3 CDC42 DUSP5 PDE6H MST1R CDK4 CARD14 PYCARD LYN DUSP26 ITGB1BP1 SNX9 EFNA1 PSMD9 CCNB1 FLRT3 PRKAR2A DCUN1D2 GP1BA TGFB2 RAP1A TRAF6 CUL1 CDC20 RAPGEF2 CD81 HTR2A SOCS3 C10orf46 BUB1B PRKAA1 EPGN EREG ADARB1 RAP2C DUSP2 DUSP18 RAD50 CCNL1 TAOK3 FGF16 RPS2 SNF8 DUSP7 CD24 JUB CHRNA3 CARD10 HEXIM2 NEK7 NTRK1 PSMD14 MAP4K2 ANAPC11 TGFB3 MYCNOS MLLT1 FGFR1 GNG3 SPRY2 ADIPOQ PODN NR2F2 PTK6 TNFSF11 NPM1 HMGN1 TINF2 DUSP22 LRP8 ZNF622 LRTM1 LAT TAF7 IQGAP1 DSTYK ALK GNAQ RTN4RL1 NYX PRKACG EMP2 PSME3 GMFB DBNDD2 PSRC1 DUSP3 PIN1 PILRB PSMB6 DUSP21 ITGA1 VLDLR DUS2L IL18 ANGPT4 STK38 NLRC5 PABPN1 PDPK1 PRDX3 ODZ1 MAP2K7 PNKP NBN DRD2 EGF CTNNB1 LRRTM1 UBE2L3 CDK5RAP1 PRKACA UCN PTK2 PSMB9 THBS1 ADCY4 PSMB4 CDC25B TRIB3 CCKBR SMG8 PSMD13 FGF13 FGF18 USP44 MAP4K3 PKIG TEK ZGPAT CDC14B GPRC5A TPD52L1 HEXIM1 DBNL ZNF16 TLR6 FABP4 UBE2S ZER1 ZFP91 RALB EDN1 TELO2

GO_LOCOMOTION Self-propelled movement of a cell or organism from one location to another. SEMA6B NTN3 PRKCQ SLC26A8 CD248 CEACAM8 TNFRSF11A CCL19 CCL14 CXCR6 SCN1B RFX3 RSPH9 SEMA6A VAV2 SCARB1 CENPV KAL1 PTAFR HBEGF DRGX SLC22A16 CSK LRRC6 ZNF280B RAB13 TUBB3 SKI JAM3 SPTBN5 ILK SPTB JAM2 TBX20 FAP B3GNT1 PAFAH1B1 TSPAN1 LPAR1 CH25H SPTA1 FAM40B SPEM1 SDCBP SOX8 VAX1 PARP9 GRIN2C PDGFRB ARTN CD2AP TGFBR3 C12orf55 CDK5R1 GPR56 PLD2 KRAS PLXNA2 CCR2 PTPRC NDN CMKLR1 SCG2 P2RY1 CD177 CDH4 TAOK2 CORO1C CXCR4 SIRPG ZNF280C SORBS2 SIX1 C16orf45 CMTM2 BRK1 FFAR2 C11orf34 CXCL1 CTTN ELANE MADCAM1 PKN1 FGR FEZF2 CD244 CCL17 CNR2 CD44 NPHP4 CDK5 XCL1 DOK2 PRKCD NRXN1 TWIST1 LRRK2 SLC9A10 OR1D2 CCDC164 POU4F2 IL4 GPX1 FPR3 ECSCR SIAH1 NTRK3 EFNB1 SSTR4 DNAH3 TRPM4 CXCL12 KITLG CCNYL1 ZFAND5 ADAM9 PCM1 CXCL13 C10orf92 EVL PLXNC1 OTX2 PDGFRA NDNF TYRO3 GLI3 NGFR AMICA1 TMEM201 PIK3C2G ELMO1 WWC1 ARRB2 NDEL1 CCL2 TNFAIP1 EFNB3 ITGB7 EDN1 IL17RA USP9X FOXB1 PGK2 FBXO45 EXT1 BIN2 HAND2 ISL1 FGF13 CCL27 IL10 SEMA6D DCDC2 CTNNB1 DRD2 THBS1 P2RY12 CXCR5 PTK2 ANGPT4 ITGA1 STARD13 GP6 SCRIB ERG PLXNB1 MATK DOCK1 RHOC NOX1 ETS1 MYO5A ROBO1 FOXC1 SETD2 CCL23 GPR183 NR2F2 ITGA11 DPYSL2 NEO1 MATN2 HES1 GDF7 CSPG4P5 CD84 CCL22 IER2 FER OGDH JUB CD24 PF4 RAPGEF2 SEMA4A ITGAV TGFB2 NODAL SMO PSTPIP2 NR4A3 CHN1 ELMO2 SPTBN4 SH3KBP1 SEMA7A LYN FLRT3 SNAI2 NET1 KLF7 EFNA4 EFNA1 BAMBI CAV1 NKX6-1 KIT JUP PAK7 FOXN1 CDC42 SAA2 NCAM1 FSCN3 PLD1 ANK3 ISPD GPR15 FOXJ1 GATA3 EPHA4 SLC9A1 KRT16 SATB2 SLC3A2 PRKCI VPS4A CXCL9 CDKL5 CCR9 BDKRB1 GPLD1 GAS8 VEGFA CCL28 ITGA3 BVES HSP90AA1 DNAAF2 JAK3 PEX13 SRC EPHB2 SLC7A6 CCR6 GLG1 EDN2 TNS1 DNAH7 NTRK2 MIXL1 ANGPT1 WDPCP POU4F3 EPHB4 PROP1 SOX17 SPN EFHC1 FN1 KIF5B PKN3 DAG1 CCL15 SORD DCLK1 IFNG NAV1 SPTAN1 ALCAM LHX3 SLIRP OVOL2 CUL3 LGALS9 CCL3 ITGAL FOLR2 PSTPIP1 RASGEF1A FLNA RELN CLRN1 FAM83D WNT5A PEAK1 DAB1 NR4A2 FMNL3 UNC5B MYH10 HRH1 PPAP2A GOLPH3 CD74 PLXNA1 SPATA13 ITGA9 DNAH5 DYX1C1 NTN1 ITGB3 ENAH AMOTL1 KIRREL3 RLTPR BARHL1 SRPX2 IL16 CCKAR TNF FZD3 MDGA1 GLI2 HSD3B7 RET VEGFC EPYC RHOA DIXDC1 EPB41L5 GAPDHS SEMA4D DLX5 ERBB2 CCL20 ATP2B2 ING2 ETV4 SFTPD SEMA4F NCK1 TNK2 SIX2 PREX1 APBB2 SDC3 FPR2 CXCL2 PAK4 EZR OPHN1 ARHGEF5 ATP5B SOX1 USP24 RPL24 NRP1 LEP LPHN3 RARRES2 MMP9 JAK1 FEZ1 POU3F2 CATSPER1 PIK3CB PTPRA LCK FGFR4 FSCN2 NCK2 AXL CTNNA2 HRAS LHX2 VANGL2 SEMA4G WNT7A HOXA2 BCAR1 BBS4 L1CAM GSK3B IL12A DOCK4 NLRP12 MDK PKN2 YWHAE IST1 TUBB2B CYP1B1 TSC2 PTPRJ CREB3 WDR52 FOLR1 SLC16A1 LSP1 B4GALT1 SPNS1 CCR3 ARL13B NOG ADAMTS12 INPP5B MBOAT7 RAC1 ADAM8 ANKS1A CMTM4 LYST SRGAP2 SLC7A7 CSF3R SEMA3G C1orf187 SOX10 LMX1A PARVA NDRG4 CNTN2 RYK EGR3 AGTR1 EDNRB GJA1 NRG1 MKL1 CCL3L3 PDE4D LAMB1 CEACAM1 SGK196 KRT2 JMY MAP2K1 PLXND1 RALBP1 TREM1 IL6 NRXN3 RAB1A PIK3R1 VPS4B ANLN S100A8 GRB2 SAA1 NRCAM ERBB4 PML PTP4A3 LRRC16A DPP4 PLA2G1B LDHC CXCL6 CHL1 PIP5K1A NLRP10 ASAP3 EPHB1 BTG1 CORO1A CCL4 TBX1 FGF19 PLXNA3 DEFA1 NFATC2 VASP ANXA1 BCL11B NUP85 CTGF RHOG DPYSL5 RIC8A LHX9 SLIT1 CKLF CCDC40 NFASC ICAM1 GDNF ASPM DVL1 RPS6KB1 CCL4L2 MAPK1 PSEN1 NKX2-3 DAB2IP BAX EPHA7 NRAS GBF1 PTPRO MAPK8IP3 ACVRL1 BMPR1B SEMA3F SHROOM2 DMRT1 HGF KCTD13 CELSR3 DEFB1 WNT3A DISC1 SDCCAG8 SLC7A11 STAT1 SOX18 CCL11 EPHA2 DAPK2 CACNA1I EGFR NEFL ANG JAGN1 CCL26 ADAM17 CELF3 ITGAX SEMA6C THBD T ITGA2 PTPRK CCR5 ATP1B2 F2 PPP1R9B CD34 SIX4 EPS8 RPS19 CYR61 ENG SNAI1 CHGA FMNL1 PIP5K1C OR8A1 TBX21 ISL2 HCK MMP14 INPP5D CCK LRP5 CER1 ELP3 ROBO2 GPR77 FAT1 LHX6 SYK CMTM8 F11R RAB7A GAS6 PLAUR PDCD10 FES PHOX2B CCR1 NRG3 CDH2 CCDC39 ITGA5 LOC100506013 SEMA3B SPTBN1 NOS3 ATP2B4 MYO18A HMGB1 PTK2B PTPN6 PHACTR4 XCR1 PRKCZ TNN CCL25 JAK2 PRM3 CXCL16 NEURL ABI2 DOCK8 FGF10 CCDC88A SOCS7 MSX2 F2RL1 FGF2 FIGF CD151 GBX1 KDR PSG2 KIF5C SEMA4C CMTM5 SEMA5B NANOS1 HIST1H2BA NDE1 CXCL10 EMR2 LAMA5 APC CXCL17 GBX2 DDX4 APOA1 ANO6 PTPRM SLIT3 UNC5A PTPRF GPC6 CUZD1 DPYSL4 CDK1 GPR33 EPHA5 NTN4 VCAM1 RERE FLT1 DCC SLC7A9 FZD4 EPX XCL2 MERTK MAPK3 PRKG1 LTB4R2 VHLL CCL16 NRD1 EFNA5 FOXD1 MMP1 COL1A1 PIK3CA CMTM6 DEFA1B CAP1 STAT5B BDNF MYH9 SLC16A8 CREB1 SELE ITGAM PPIA ATP1A4 EFNA2 DGKZ GPR44 PPP3CB EFNB2 CELSR1 CYP7B1 TDGF1 S100A9 RBFOX2 SELP PLEKHO1 CXCR2 HOXB9 CSPG4 TMEFF2 MET AIMP1 TRY6 SOS1 DNAH6 TEKT3 DNAH17 NUP93 UNK ARHGEF16 FGF7 SEMA3A IL8 FOXE1 AVL9 MMP12 TEK CEND1 SELL SIAH2 ENPP2 SLC7A5 PROCR GCNT1 SRF S100A2 ZEB2 ABL2 MYO1G PRPF40A ITGB1 ARF4 EMX2 EFNA3 ID1 SDC2 PDPK1 EMP2 DNAI1 DST FUT10 TMF1 GRIN2A GPM6A SPNS3 TNFSF12 DCX ZRANB1 ARX GIPC1 SLIT2 FGFR1 CXCL11 CCRL2 PTK6 TNFSF11 LAMB2 GRB14 NTRK1 PRSS3 C3orf39 CCL8 ARC PEX7 APOB NR4A1 NFIB LEF1 TBX5 KIAA0319 CXCR1 PF4V1 CD63 DCHS1 RNF165 COL5A1 PIK3CD ITGB1BP1 ALX1 CCL3L1 TNFRSF12A POU4F1 PROS1 GPR37 CATSPER2 CCL21 IL12B FOSL1 CDC42BPB PPIL2 DRD1 HMGB2 ZAP70 PPBP CXCL3 UNC5D ARPC5 MEGF8 ITGB4 LGALS3 SEMA4B SPTBN2 CXCR3 PPAP2B WNT3 ZNF280D DOCK2 C5AR1 KIAA1598 RALA FAT2 LOC100507050 SEMA5A CCL13 CDH13 SDC4 LBP MSN C1orf190 VIL1 PDE4B AKAP4 MAPK14 ETV1 SLC7A8 SHH SRGAP2P1 NUP188 C12orf11 DBH SEMA3D NTF3 SERPIND1 MIF PLXNB3 PDGFA CMTM3 RANBP9 CXCR7 ATP1A2 CX3CL1 GPR124 INSL6 ROCK1 PLXNA4 THBS4 UNC5C LHX1 TMEM18 PRKX PLAT ADCY3 PPARD BSG CXCL14 GREM1 PROX1 CXorf41 RNASE2 PALLD WASF2 GPR32 HEXB VAV3 MITF DNAH2 TNP1 SCYL3 FERMT1 PDILT TYK2 APP ASTN1 ATOH1 SIX3 FUT8 RHBDF1 COL1A2 PTEN NCKAP1L ACVR1 PODXL2 SMCP TOR1A IL1B NR2E1 PLXNB2 EPHA8 DDIT4 ROBO3 DDR1 S100P LOXL2 RPS6KA5 ZNF280A CCR7 PPID PTK7 NRTN TNR CLN6 TLX3 SAA4 MAG GPC1 HRG RAC2 OLIG3 KIF26B CHRNA7 AZU1 JPH3 FCER1G PDGFB CALCA C9orf46 NEUROD4 S100A12 CCR4 CEACAM6 CDK5R2 ASCL1 FGF8 ZNF580 CNTN4 C3AR1 PROK2 SGPL1 TXNDC3 CSF1R CD48 CATSPER4 LECT2 ALKBH1 DPCD PLEKHG5 ID2 FEZF1 SEMA3E SPON2 CCL7 NRP2 OLR1 APC2 PROC PLA2G10 PLCG1 PDPN CTHRC1 TIAM1 MARK2 DNAH1 TYMP EDN3 MEF2C FPR1 ARSB GATA2 ENPEP ARID5B APCDD1 CD2 HTR6 MAPT CCL5 SELPLG ITGA4 AMOT LHX4 C15orf38 CCRL1 PLP2 SRMS PAXIP1 FLRT2 NKX2-1 CCBP2 DNAJA1 GAP43 BMP4 UMOD EPHB3 CXCL5 BOC TTLL5 SDC1 SEMA3C ESAM SLC7A10 ITGA6 KIF26A NOV DNAH8 B3GNT2 ROPN1B HTR2B ARHGAP35 TOP2B CCL24 CDC42BPA PTPN11 PVRL1 SPAG16 PHACTR1 CCL18 GFRA3 PIK3R2 DEFB4A C5 LAMC1 CXADR NEDD4 CORO1B SHC1 USP9Y FSCN1 HOXA5 CRMP1 PIK3CG TSPO LCP1 TMEM146 ATP1B3 IL6R DNER IL17RC ITGB2 CX3CR1 USP33 CMTM1 FYN HIF1A PLTP PRSS37 DEPDC1B S1PR1 NOTCH1 FUT7 PODXL IRAK4 SIRPA ANGPT2 STYK1 SLC16A3 SPNS2 LY6K LGI1 WNT11 LAMA2 RTN4 TGFBR1 TGFB1 PLAU OGN SPOCK1 CD58 DNAH11 EGR2 EPHA3 VAV1 CCL1 AAMP GRB7 KIF5A BARHL2 BRAT1 MARK1 SMAD4 CCR8 BIN3 CMTM7 CCR10 CATSPER3 TNS3 YES1 PAX6 MESP1 CD47 LIMD1 CYSLTR1 ELMO3 FEZ2 PECAM1 PLA2G6 SBDS ATP1B1 TEKT2 SYNE2 PITX2 POU3F3 BMP7 FMOD

GO_FATTY_ACID_ELONGATION The elongation of a fatty acid chain by the sequential addition of two-carbon units. TECR PTPLB PTPLAD2 ELOVL1 ELOVL4 ELOVL5 ELOVL7 ELOVL2 ELOVL6 PTPLAD1 ELOVL3

GO_ACTIN_FILAMENT_ORGANIZATION A process that is carried out at the cellular level which results in the assembly, arrangement of constituent parts, or disassembly of cytoskeletal structures comprising actin filaments. Includes processes that control the spatial distribution of actin filaments, such as organizing filaments into meshworks, bundles, or other structures, as by cross-linking. C2orf62 EZR CTTN BAIAP2 AKAP2 BRK1 COBL CDC42EP2 TRPV4 NCK1 FAM101A ARPC3 HCLS1 INPPL1 PREX1 SORBS2 MICALL2 LMOD2 PDXP GHRL TNNT2 WASF1 LMOD3 ZNF664-FAM101A ABI1 TMSB15A CTNNA1 TMOD1 ARPC5 FSCN3 BAIAP2L1 PLS1 TMSB15B MAGEL2 CDC42 ADD2 SORBS1 MICAL1 WASL MSRB2 IQGAP2 VIL1 ARHGAP6 ARPC2 WAS WHAMM CCL11 BIN3 EPB49 PRKCI FRYL BCAR1 WASH6P SORBS3 KCTD13 KIAA1598 TMSB4X MAD2L2 CAP1 SHROOM2 FSCN2 NCK2 CAV3 DSTN ARAP1 PPP1R9B ACTA1 ARF1 SHROOM1 GSN EPS8 SRC WASF3 ACTN1 MYO1B ANG TRIM27 RND1 WASH1 AIF1 DIAPH1 CORO7 EVL TMOD3 ACTR2 NEBL WIPF1 TTN DLG1 SPIRE2 SCIN TMOD4 ZYX MICAL2 TTC17 TCAP LIMA1 ACTR3 RAC1 FHOD3 CFL1 TNFAIP1 PHACTR1 JMY DBN1 TMOD2 MICAL3 FLNA NEDD9 ITGB1 PDCD10 PROX1 PDLIM3 ARPC4 SWAP70 GHSR ACTR3B FAT1 PACSIN1 ACTC1 RHOF SHROOM4 PLS3 CUL3 KPTN SRF WASH3P PLA2G1B ARHGEF2 HSP90B1 BCL2 FSCN1 JAK2 COBLL1 RHOD LRRC16A FMN1 ERMN ADD1 PTK2B DPYSL3 CCDC155 SPIRE1 SPTA1 RUFY3 EMP2 CLRN1 ESPN BAIAP2L2 CORO1B CAP2 TMSB4Y FAM101B TMSB10 ITGB5 PPP1R9A RHOA ACTN4 PPARGC1B VASP ACTR3C ARPC1B CFL2 ARPC1A CORO1A SEPX1 FMN2 FRY RAC2 LMOD1 GDPD2 PAWR ABI2 ARPC5L ABL1 ALDOA LCP1 RLTPR DIAPH2 ARRB1

GO_NUCLEUS_LOCALIZATION Any process in which the nucleus is transported to, and/or maintained in, a specific location within the cell. DCTN4 SYNE2 HHEX DOCK7 NTN1 WDR81 SUN2 WDR43 SLC9A3R1 CAV3 PCM1 TMEM201 PTK2 CDC42 BIN1 SLIT1 HOOK3 MYH10 CEP120 PAFAH1B1

GO_BONE_TRABECULA_MORPHOGENESIS The process of shaping a trabecula in bone. A trabecula is a tissue element in the form of a small beam, strut or rod. MSX2 THBS3 MMP2 SBNO2 WNT10B SEMA4D PPARGC1B COL1A1 SFRP1 PLXNB1 FBN2

GO_POSITIVE_REGULATION_OF_TRANSLATIONAL_INITIATION Any process that activates or increases the frequency, rate or extent of translational initiation. RXRA IMPACT YTHDF2 DDX3X POLR2G POLR2D YTHDF1 UHMK1 CDC123 BOLL RPS6KB1 DAZL KHDRBS1 EIF2AK4 EIF2B5 METTL3 NCK1 DNAJC3 CCL5 TNF PPP1R15A DAZ3 RPS6KB2 DAZ1

GO_POSITIVE_REGULATION_OF_CELL_GROWTH Any process that activates or increases the frequency, rate, extent or direction of cell growth. EXOSC2 CYBA PSMD10 H3F3C NOL8 ZFYVE27 SEMA4D EXOSC4 HPN ERBB2 CDH4 TGFB2 LGI1 TNFRSF12A ANAPC2 TGFBR1 SYT14L MAP1B C6orf108 SEMA7A NRP1 H3F3B SYT2 CDC42 TAF9 USP47 CIB1 EFNA5 SPHK1 AKAP6 CPNE5 RND2 LIMK1 DSCAM MEGF8 SFN INO80 KDM2B IGFBP1 KIAA1598 SLC9A1 NTRK3 N6AMT1 WNT3 MTPN KRT17 BDNF CPNE9 CDKL5 DERL2 CXCL12 AKT1 BMPR2 SYT4 VEGFA WNT3A DISC1 DDX3X TRIM32 L1CAM SEMA5A HDGFRP2 GSK3B MAPT H3F3A ADAM17 TWF2 EGFR INS IST1 S100A9 AVPR1A F2 SMURF1 RAB11A SUPV3L1 SLC25A33 EDN1 FN1 NCBP1 SYT17 PRSS2 CDKN2AIP CD38 GOLGA4 NDEL1 UNC13A PPP1R1C LPAR3 MUL1 TAF9B MMP14 RPS6KA1 CRABP2 EXTL3 MTOR HBEGF EXOSC9 UCN CSNK2A1 RIMS1 ZNF639 UTS2R SRF RPS6KA3 ILK ADNP2 DDX39B SYT3 NRG1 ALOX12 FGFR1OP S100A8 HAMP PAFAH1B1 PTK2B PIN1 HIST1H1B FXN SLC44A4 RUFY3 CPNE6 NEDD4L ADAM10 RASAL1 NTN1 CXCL16 BCL2 IL9 ADNP PARK2 SDCBP MAP2K5 SFRP2 SFRP1 RIMS2 TRPV2 IGF1 ISLR2 AVP IL9R SYT1 HYAL1 TRPC5 IL2 LEF1 MACF1 ITSN2

GO_POSITIVE_REGULATION_OF_MAP_KINASE_ACTIVITY Any process that activates or increases the frequency, rate or extent of MAP kinase activity. MAP3K11 KRAS TNF MAP4K2 IRAK2 DIRAS2 TGFB3 FGF2 FCER1A FRS2 PIK3R6 ARRB1 IGF1 MAP3K4 FGF10 MAGED1 DUSP6 TAOK3 PDGFB UBA52 DUSP7 CD24 JUB CHRNA7 PRKAA1 RPS3 EPGN FZD8 TLR4 LPAR1 SHC1 PTK2B C5 FGF1 WNT5A DRD4 IQGAP1 MAP2K2 ALK P2RX7 GHR MAPK11 AKAP13 PDGFRB TNFSF11 MAP2K4 MAP2K3 RIPK1 PLA2G1B MAP2K5 BIRC7 PIK3CG S1PR2 CD74 SAA1 FGFR1 MAPKAPK3 GNG3 DUSP19 IL1B CSK GRM4 THBS1 PTPN11 SYK PAK1 EGF SASH1 MAPKAPK5 HTR2B TAB1 MAP2K1 ILK ODZ1 MAP2K7 IKBKG ITGA1 PDCD10 FZD5 NRG1 TLR9 ADAM8 CCL19 EDN1 MAP3K9 TNFRSF11A GH1 PTPLAD1 MAP4K5 DBNL DUSP9 CD40LG PLCE1 RPS27A TLR6 MAP3K10 FZD10 MUL1 DVL2 LPAR3 MAPK10 FLT3 FGF18 TPD52L1 SHC2 PAK3 MAP2K6 TAB3 MAPK14 GAB1 ADORA2B TDGF1 EGFR CD40 PDE5A MAP3K6 PDGFD PDE6G GRM1 TGFA AVPI1 IQGAP3 ADRA2A BMP2 MUC20 TAB2 PDGFA MIF MDFIC MAP3K2 SRC CSPG4 DIRAS1 MDFI NTF3 PROK1 VANGL2 C1QTNF2 MAP3K7 PDGFC NTRK3 EPHA4 HGF ZAK NOD1 EDN3 HRAS C5AR1 ADRA2B MADD TIAM1 VEGFA TP73 IRAK1 UBC SPAG9 UBB KITLG FPR1 ERP29 AGER RASGRP1 NOX4 NOD2 DUSP5 ADRA2C PDE6H DAB2IP MST1R PEA15 KIAA1804 MOS FZD4 KIT MAPK1 PSEN1 PIK3R5 ERCC6 ARHGEF5 FLT1 WNT7B SOD1 PTPN1 GHRL DAXX PIK3CB MAP4K1 MAP3K5 LRRK2 AXIN1 MAPK8IP3 FGD2 MAPK3 HTR2A RIPK2 INSR NEK10 S100A12 CXCR4 ERN1 TRAF6 ERBB2 CD81 EFNA1 TRAF2 TGFB1 PKN1 ELANE MAPKAPK2 CDK1 PROK2 DVL3 EZH2

GO_REGULATION_OF_SECRETION Any process that modulates the frequency, rate or extent of the controlled release of a substance from a cell or a tissue. IRS2 CACNA1I AVPR1A PRKCA PLA2G6 ADORA2B EGFR INS ANG LLGL2 FOXF1 SYT14 RAP1B CPLX3 SLC25A5 NF1 GSDMD KCNC2 SLC30A8 MYO6 ACSL3 BLK KCNA5 ABAT ARF1 CD34 NPR1 SYT5 SMAD4 GPR68 AIM2 ADRA2B BAD RHBDD3 RHBDF2 KCNG2 TLR8 BANK1 FKBP1B SNX6 SLC24A6 SYT15 HRH3 DOC2A SLC9A3R1 CPLX1 CD58 PTGER4 ATP13A2 GAB2 NLRP3 PNKD ADRA2C P2RX4 GALR1 STXBP3 SNCA ADORA1 CAMK2A SRGN VAMP3 TRPM2 NEUROD1 CRY1 GHRL STXBP1 HMGCR C2CD4C FGG C1QTNF3 VPS35 HADH RAPGEF3 SRCIN1 RAB3GAP1 GDNF GAL TGFB1 TRAF2 TRIM16 IGF1 TNFSF4 ARRB1 RAB27A IL17RC SFRP1 CPLX2 SOX4 BCR NLRP10 CADM1 HCAR2 IL2 SERP1 SYBU HIF1A KCNS3 LILRB1 IL17F RAB3A NOS2 NOTCH1 RIMS4 ANXA1 TAC4 SYT16 VPS4B DRD4 C5 ADRBK1 S100A8 C2CD4B RAB2B TLR4 TNFRSF14 PARK2 PASK SAA1 PML PRAM1 RAB9A ADCYAP1 PLA2G1B HTR2B CPB2 HMOX1 SCAMP5 GIP SYN1 PTPN11 SREBF1 AGTR1 C19orf26 SLC25A6 NRG1 GJA1 EDNRB ARNTL CEACAM1 IL6 MIDN SOCS1 SLC6A1 RAB5A ANKRD1 NNAT MARCKS IL1RL1 TAC1 WLS STXBP2 TLR9 ADAM8 CHMP3 SYT13 NLRP2 NPHS1 HMGN3 INHA C12orf4 SDC1 CLASP2 SYTL3 FAM132A DTNBP1 NOV IL1A SLC25A4 NSF CRP NR1H2 CD14 ANO1 PFKFB2 NAPA CIDEA DRD3 IL1RAPL1 CD2 CCL5 TMEM27 HGS SLC16A1 WNK3 RAB11FIP1 SIRT3 F2R LLGL1 SERGEF CACNA1D TIAM1 PHPT1 RAB3B MYRIP KCNN4 SNAP25 BMP8A CARD8 EDN3 GNAS C2CD2L FOXP1 FFAR1 MEF2C CLEC4E MCU TCF7L2 NR1H3 NCS1 MAOB IRS1 NTSR1 ARL2BP TRIM9 NLRP12 SYT9 ICA1 CRH GATA2 CASP5 CSF1R LEP PINK1 OSM RABGEF1 SYT2 RAB27B HAVCR2 FMR1 CACNA1C HAP1 PLA2G10 VTCN1 PFN2 NKX3-1 SYTL5 ACVR1C NLRP6 CDK5R2 NPY5R INHBB SYTL4 IL17A EZR CLOCK PPID CCR7 LGI3 HTR1B DNM1L KCNQ1 GPAM C2CD4D FCER1A RASL10B WNK1 CCKAR TNF IFNAR1 CFTR SNCAIP RAC2 CHRNA7 SEPT5 PLA2R1 VEGFC PPP1R9A FCER1G ZP3 RHBDF1 CLEC9A P2RX7 EXPH5 G6PC2 WNT5A SYT11 RAB11FIP3 PDX1 UCN3 PAEP CYP4A11 IL1B TLR2 PAX8 ARL2 CD74 GOLPH3 RPH3AL CRHR1 SYT7 RAB8B SLC2A1 CHMP6 OXCT1 PIM3 LGALS9 PPARD CCL3 HFE OXTR RAPGEF4 OR51E2 C1QTNF1 SNCG GCG PYDC2 HSPD1 TNFRSF21 TRIM6 RAB33B SMPD3 C13orf15 CD274 CD276 CD38 BMP6 FN1 IFNA2 KIF5B IFNG ITPR1 FCN1 AQP1 RAB11FIP5 PANX1 CACNA1A SCIN FOXP3 NLRP2P IL13RA2 PAM NANOGNB DNAJC1 RAB3D MAPK14 LPL UNC13D ADCY5 IRF3 CACNA2D2 STK39 SRC GBP1 MIF RAB26 TLR10 BRAF CRHBP GDF9 CORIN TARDBP ANGPT1 ARHGEF7 GATA3 ACCN2 NR1D1 REST PFKL RAB21 CRTAM RASGRP1 ERBB3 RALA VPS4A SDC4 IL4R GPLD1 STXBP6 SIDT2 TRPV1 CACNA1G ACHE FOXA2 CD300A NKX6-1 SCT AACS TACR2 PDE8B PYCARD STAM ZAP70 NOD2 APOA2 CARTPT O3FAR1 SYTL1 ANXA4 TRAF6 FFAR3 FGA IL1RAP RAP1A TGFB2 COMT HDAC1 ITPR2 TRH HTR2A KCNMB4 CHRNA4 CHRNB4 ALOX15B POMC LYN OPRL1 PDCD6IP ALOX12B NLGN2 GPR27 PSMD9 OPRM1 CADPS2 CD84 SLC30A1 KCNJ11 RIMS2 FER NDUFAF2 TC2N TGFB3 ARFIP1 SLAMF1 GPER GHRH HTR2C CHRNA3 PPP3CA CASP1 PCK2 SNF8 SYT1 NR1H4 HLA-E CHD7 TMF1 OPRK1 NLGN1 UCP2 NMU AGTR2 POSTN FGFR1 GLUD1 RFX6 CHMP2A DPYSL2 TNFSF11 TNFRSF9 ADIPOQ GHSR IL26 IL10 RIMS1 EGF UTS2R DRD2 P2RY12 KDM5B SOX11 UCN SSC5D GRIK5 KISS1 PDPK1 PER2 SYTL2 SYT8 HCRT TLR6 SRI UNC13A KRT20 EDN1 ILDR1 IL17RA RALB CHRNB2 SYT6 TMBIM6 SYT17 TFR2 FGF23 AP1G1 FAM3D ISL1 NMB SYT10 CNR1 ADAM9 S100A9 CHRNA6 CD40 HTR1A IL17RB CYP4F2 AVPR2 GLUL TRIM27 LEPRE1 TNFRSF4 OXT RAB3C FGF20 CLEC6A ADRA2A ATP2A2 SYT12 TSG101 IL4 PTGER3 SSTR5 VAMP7 SGK1 CREB1 ADA BRP44 SNX4 GLMN SLC2A2 PPIA ERP29 TRPM4 CXCL12 APLN ZC3H12A KCNA2 BRSK2 RPH3A PPP3CB SYT4 TRPV6 STX4 KCNB1 CPLX4 C2CD4A XBP1 LRRC32 LAMP1 CD244 GHRHR CDK5 SCG5 NPPB RSG1 CLEC5A TWIST1 NRXN1 CPT1A LGR4 LRRK2 EFNA5 CAPN10 DPH3 SIRT4 ENSA DOC2B APOA1 GJA5 RIMS3 REN PYDC1 CYBA CHIA TRPV4 PTPN22 NPY2R GIPR CDK16 NR0B2 PCLO SYT14L VAMP2 FFAR2 IL11 EPHA5 TFAP2B FGR CLASP1 STXBP4 FGB FGF10 ABL1 HYAL2 RAB15 IL1R2 F2RL1 SEC24A EXOC1 AGT STXBP5L IL36RN CSN2 CCR2 IL13 P2RY2 C6orf125 VAMP8 P2RY1 AVP LIF MAPK11 SPTBN1 MYO18A PRKCE HMGB1 NPVF UTS2 ENY2 JAK2 PRKCZ ITPR3 SDCBP HNF4A POFUT2 ABCC8 GPR77 RETN LACRT SYK GOLPH3L PTAFR TLR5 SYT3 GAS6 NLRP1 IL33 RAB7A ATP6AP1 STX1B BTN2A2 PIK3C3 ABR RBP4 FES TLR1 STXBP5 MTNR1B INHBA GLP1R IL5 CHGA STC1 CD40LG GRM7 PFKM TNFRSF11A C12orf39 FOXL2 CACNA1E KCNC4 NPFF CCL19 STX1A MAP2K6 RSAD2 APBB1 CCK GCK UNC13B WNK4 RFX3 VSNL1 LRP5

GO_RESPONSE_TO_UV_B Any process that results in a change in state or activity of a cell or an organism (in terms of movement, secretion, enzyme production, gene expression, etc.) as a result of a UV-B radiation stimulus. UV-B radiation (UV-B light) spans the wavelengths 280 to 315 nm. STK11 CRIP1 HYAL3 IVL IL12B ERCC6 HYAL2 BCL2 MSH2 RELA HMGN1 MME IL12A MFAP4 XPC CDKN1A HYAL1

GO_NEGATIVE_REGULATION_OF_DENDRITIC_SPINE_DEVELOPMENT Any process that decreases the rate, frequency, or extent of dendritic spine development, the process whose specific outcome is the progression of the dendritic spine over time, from its formation to the mature structure. PLK2 FSTL4 PTEN HDAC2 SRGAP2P1 EFNA1 DNM3 NGEF ASAP1 NLGN1 NLGN3

GO_REGULATION_OF_VASCULAR_ENDOTHELIAL_GROWTH_FACTOR_RECEPTOR_SIGNALING_PATHWAY Any process that modulates the frequency, rate or extent of vascular endothelial growth factor receptor signaling pathway activity. HIF1A MYOF PRKCB MT3 IL1B FGF9 VEGFA HHEX VEGFC ITGB3 MMRN2 GRB10 FGF18 TMEM204 NEDD4 FZD4 PRKD2 FGF10 ITGA5 VTN FLT1 PTPN1 ARNT PDCD6 DAB2IP

GO_OXALOACETATE_METABOLIC_PROCESS The chemical reactions and pathways involving oxaloacetate, the anion of oxobutanedioic acid, an important intermediate in metabolism, especially as a component of the TCA cycle. GHR PCK1 MDH2 STAT5A STAT5B MDH1 PCK2 ACLY NIT2 MDH1B GOT2 GOT1

GO_POSITIVE_REGULATION_OF_GLUCOSE_METABOLIC_PROCESS Any process that increases the rate, frequency or extent of glucose metabolism. Glucose metabolic processes are the chemical reactions and pathways involving glucose, the aldohexose gluco-hexose. PTPN2 PPP1R3G SRC RGN GCG FOXO1 PHKG2 KAT2B SLC45A3 ACTN3 IGF1 DGAT2 SORBS1 IRS2 DYRK2 INS ARPP19 PTH KAT2A PMAIP1 PPARGC1A IRS1 GCK GPLD1 AKT1 FAM48A EPM2AIP1 PPARA WDR5 C1QTNF2 SMEK2 IGF2 AKT2 INSR

GO_C_TERMINAL_PROTEIN_AMINO_ACID_MODIFICATION The alteration of the C-terminal amino acid residue in a protein. FOLH1B ATG4A AGBL1 ATG4B LCMT1 ATG12 ATG5 ATG4C AGBL4 ICMT FOLH1 ATG4D ATG7 AGTPBP1

GO_NEUTROPHIL_ACTIVATION_INVOLVED_IN_IMMUNE_RESPONSE The change in morphology and behavior of a neutrophil resulting from exposure to a cytokine, chemokine, cellular ligand, or soluble factor, leading to the initiation or perpetuation of an immune response. TYROBP VAMP2 ANXA3 STXBP2 FCER1G PPBP VAMP7 ZAP70 STXBP3 VAMP8 SYK

GO_POSITIVE_REGULATION_OF_INSULIN_RECEPTOR_SIGNALING_PATHWAY Any process that increases the frequency, rate or extent of insulin receptor signaling. INS OSBPL8 ADIPOR1 SRC PRKCZ SERPINA12 IGF2 NR1H4 SIRT1 IRS1 LEP FAM132A NUCKS1

GO_GOLGI_TO_PLASMA_MEMBRANE_PROTEIN_TRANSPORT The directed movement of proteins from the Golgi to the plasma membrane in transport vesicles that move from the trans-Golgi network to the plasma membrane. BBS1 RAB10 ARFRP1 BLZF1 RAB34 RAB31 VAMP3 ANK3 OPTN GOLGA7 GCC2 RAB26 GOLPH3 VAMP2 MACF1 AMN VAMP7 KIF13A NSF VAMP4 SYS1 SPTBN1 KRT18 GOLGA4 VAMP5 BBS2

GO_BONE_MINERALIZATION The deposition of hydroxyapatite, a form of calcium phosphate with the formula Ca10(PO4)6(OH)2, in bone tissue. FGFR3 ERCC2 WNT11 CER1 ZBTB40 ALOX15 ASGR2 BGLAP MINPP1 DUOX2 CLEC3B CYP27B1 PTN FBXL15 TUFT1 ANO6 IBSP GPNMB FGFR2 BMP2 PKDCC RSPO2 PHEX LGR4 EIF2AK3 GPC3 SBDS LEP AXIN2 ASPN PHOSPHO1 PTGS2 IGF1 MMP13 SBNO2 IFITM5 PTH1R KLF10

GO_CELLULAR_RESPONSE_TO_VITAMIN_D Any process that results in a change in state or activity of a cell (in terms of movement, secretion, enzyme production, gene expression, etc.) as a result of a vitamin D stimulus. IL15 PTN FGF23 TNC PENK ATP2B1 BGLAP SFRP1 PIM1 VDR CYP24A1

GO_PURINERGIC_NUCLEOTIDE_RECEPTOR_SIGNALING_PATHWAY The series of molecular signals generated as a consequence of a receptor binding to an extracellular purine nucleotide to initiate a change in cell activity. P2RY13 P2RX4 P2RX2 P2RY2 P2RY10 P2RX1 P2RX5 GPR34 P2RY1 P2RY8 ANO6 P2RY11 P2RX6 P2RY6 P2RX7 GPR171 P2RX3 P2RY12 GPR87 P2RY14 P2RY4 PTAFR

GO_CITRULLINE_METABOLIC_PROCESS The chemical reactions and pathways involving citrulline, N5-carbamoyl-L-ornithine, an alpha amino acid not found in proteins. ASS1 OTC CPS1 DDAH1 PADI3 PADI2 ALDH18A1 CAD PADI6 PADI4 DDAH2 PADI1

GO_NEGATIVE_REGULATION_OF_PEPTIDYL_THREONINE_PHOSPHORYLATION Any process that decreases the frequency, rate or extent of peptidyl-threonine phosphorylation. Peptidyl-threonine phosphorylation is the phosphorylation of peptidyl-threonine to form peptidyl-O-phospho-L-threonine. SMAD7 DDIT4 CALM2 CALM3 SIRT2 SPRED2 INPP5K PPEF2 PARD3 SPRED1 SPRY2 CALM1 EPB49

GO_POSITIVE_REGULATION_OF_ENDOTHELIAL_CELL_DIFFERENTIATION Any process that activates or increases the frequency, rate or extent of endothelial cell differentiation. BMP4 S1PR2 CTNNB1 ACVRL1 BMP6 ATOH8 ALOX12 GDF2 ETV2 BTG1 NOTCH1 CDH5 CLDN5 PROC TMEM100

GO_NEGATIVE_REGULATION_OF_LEUKOCYTE_APOPTOTIC_PROCESS Any process that stops, prevents, or reduces the frequency, rate or extent of leukocyte apoptotic process. BCL10 FCER1G RAG1 LILRB1 SLC39A10 MIF HIF1A PIP VHLL IL2 MERTK GAS6 NOD2 CCL5 IDO1 JAK3 SELS CD27 FADD CXCR2 GPAM HSH2D DOCK8 CCL21 IRS2 CCR7 EFNA1 KITLG CXCL12 AURKB CD74 ADA IRF7 ITPKB NOC2L FOXP1 CCL19 HCLS1 AXL PRKCQ BMP4 SLC46A2 BCL6 TSC22D3

GO_NEGATIVE_REGULATION_OF_LYMPHOCYTE_MEDIATED_IMMUNITY Any process that stops, prevents, or reduces the frequency, rate, or extent of lymphocyte mediated immunity. SERPINB4 CD96 HAVCR2 MICA PTPRC XCL1 SLAMF1 CLEC4G PPP3CB CR1 IL20RB FOXP3 CEACAM1 DUSP22 LILRB1 ARRB2 C4BPB LGALS9 C4BPA HLA-E NDFIP1 BCL6 FOXJ1 IL7R IFNA2 THOC1 SPN SERPINB9 PTPN6 IFNB1 HFE

GO_PROTEIN_K63_LINKED_DEUBIQUITINATION A protein deubiquitination process in which a K63-linked ubiquitin chain, i.e. a polymer of ubiquitin formed by linkages between lysine residues at position 63 of the ubiquitin monomers, is removed from a protein. OTUD7A BRCC3 USP8 OTUD1 USP20 UIMC1 USP17L2 CYLD OTUD5 USP27X SHMT2 TNFAIP3 USP25 YOD1 BRE USP33 USP13 OTUB2 ZRANB1 ATXN3 USP16 PSMD14 FAM175B BABAM1 OTUD7B

GO_REGULATION_OF_INSULIN_LIKE_GROWTH_FACTOR_RECEPTOR_SIGNALING_PATHWAY Any process that modulates the frequency, rate or extent of insulin-like growth factor receptor signaling. IGF1 ATXN7 GHSR WNT1 IGFBP3 IGFBP4 TRIM72 IGFBP1 NKX3-1 GH1 BMP5 GHRHR AR IGFBP2 KIAA1161 CILP PHIP GHRH BMP2 IGFBP5 CDH3 IGFBP6 POU1F1

GO_ALKALOID_METABOLIC_PROCESS The chemical reactions and pathways involving alkaloids, nitrogen containing natural products which are not otherwise classified as peptides, nonprotein amino acids, amines, cyanogenic glycosides, glucosinolates, cofactors, phytohormones or primary metabolites (such as purine or pyrimidine bases). BCHE CYP3A5 TH NUDT12 NAMPT CYP3A4 DDC CYP2D6 NAPRT1 PTGS2 CYP1A2 APOA1BP PTGIS CARKD

GO_RESPONSE_TO_EXTRACELLULAR_STIMULUS Any process that results in a change in state or activity of a cell or an organism (in terms of movement, secretion, enzyme production, gene expression, etc.) as a result of an extracellular stimulus. LRP11 TNC IL4 ABCA1 MMP7 TYR DDIT3 GBA SRSF2 OMA1 AXL BMP8A HMGCL SPARC BBS4 OGT CHMP1A MTHFR MEF2C TYMS ADA TBC1D5 BMPR2 MBD2 RELA G6PC ARSB ZC3H12A PPARGC1A SLC22A3 PMAIP1 KAT2A C2 WDR59 SLC38A3 MTMR3 CNR1 GLUL AIF1 FOLR1 MKKS PIM1 UCN2 NENF IMPACT CTSL2 SLC16A1 DNAJC15 OTC ACTA1 OXT STAR MPO NUAK2 DSC2 ADSL ENSA APOA1 MIOS IGFBP2 ALAD PIK3R4 GIPR BGLAP ATG14 CHSY1 ABP1 TRPV4 ACVR1C CYBA PRKAA2 CBL NFE2L2 INHBB ASCL1 JUN PPARG MBD3 FOXO4 GCLM XBP1 SREBF2 GSS STC2 TPCN2 SOD1 VCAM1 KCNB1 GSTP1 CD4 PTGS2 SLC6A19 BECN1 BECN1P1 ASNS LEP HMGCS1 ULK1 UGT1A1 BHLHA15 MYOD1 GABARAPL3 PIK3C2B GNAI2 COL1A1 GAST ADIPOR2 LRRK2 SIRT6 DAPL1 GLRX2 NR4A2 PTEN HDDC3 CYP1A1 PTN P2RX7 RPTOR VDR UCN3 GCGR PDX1 MYH13 SLC39A4 ADNP SPP1 APOE PEMT PDK2 DAP CCRN4L PRLH RRAGC CAT CCKAR SLC39A5 SFRP2 RRAGA SIRT2 GAS2L1 ACSL4 PAX2 RRAGD TXN2 BRIP1 APAF1 PCSK9 CXCL10 POR ABCG5 RMI1 CHRNA7 GABARAPL2 ABCG8 CYBB PFKFB1 HTR4 STC1 SIPA1 NPRL3 RPS19 SH3GLB1 ADRB1 C12orf39 SORD PSPH AQP2 NPFF TSHB NQO1 LDHA DNMT3B TSC1 PYY RRP8 RASGRP4 TRIM25 KIAA1324 CDKN2B ASGR1 ADRB2 UCP3 CCK TP53 PPY KLF10 OXCT1 PPARD SLC2A1 AIFM1 ZFP36 HSPA5 WRN OGG1 FOLR2 CYP24A1 HFE ATG7 DEPDC5 PROX1 ADRB3 BCAS3 EPO LIPG GAS6 GCG GCN1L1 NPY PIK3C3 SLC6A4 ATF3 TBXA2R FADS1 TGFBR2 WNT4 DHODH PYY3 HSF1 CST3 PCK1 ALDH1A2 CYP27B1 FOXO3 EEF2 DNMT3A FKBP1B WNT2B SLC8A1 ACACB CPS1 AQP3 CCL28 SSTR2 AKT1 STAT1 SIRT5 TRPV1 CCND1 BCHE SIRT1 AVPR1A ADSSL1 RRAGB SLC27A4 ADORA2B MAP1LC3B EGFR EHMT2 MYOCD CD3E MAP1LC3B2 FOXA3 ITGA2 GSDMD SRD5A2 CLPS PICK1 EIF2A C6orf127 SRC BCL10 BMP7 ATF4 PPP1R9B ACSL3 PITX2 SKP2 COMT AES ARG1 NUDT1 HMGCR ALPL IL15 IFI16 GNPAT PPARA NUDT15 SERPINC1 LYN ICAM1 KDM4A SRD5A1 POMC TGFB1 SLC1A2 MAX ERCC1 WNT11 HAT1 PPM1D PTH HLCS BBS2 AACS RPS6KB1 FBXO22 FOXA2 PDK4 TNFRSF11B MGMT NOD2 SLC34A1 CBS FOS SSTR3 FOSL1 PYY2 CARTPT PON1 KYNU GATA4 MBD1 ASS1 MAP1LC3A GHRL G6PD FNIP1 ACSL1 UPP1 RNF152 ATP2B1 SETDB1 ATG5 POSTN UCP2 GCLC GALP HAMP USF2 F7 MYBBP1A ADCYAP1 EIF4EBP1 MC4R BCL2 NPRL2 WDR24 ADIPOQ MAP1LC3C WNT9B CADPS2 NCOA1 TSPO ALB OPRM1 NTRK1 VGF USP33 GDF3 AKR1C3 ULK2 SFRP1 PKLR HSPA8 PRKAA1 HTR2C NMUR2 GHRH TTPA AHCY CLIC5 NR1H4 ACAT1 TH COX4I1 PENK CCL2 CDKN2D LTA DAD1 RALB ADM FSTL1 CPEB4 CDKN1A LOC389493 PCSK1N SST FGF23 KIF26A MDM2 FGF21 GABARAPL1 EIF2AK2 ALDH3A1 ITGA6 TPH2 TBL2 ZFYVE1 HMOX1 GIP SRF USF1 EIF2S1 ARHGAP35 GHSR EIF2AK4 UCN SREBF1 RARA HSD11B2 MTOR RB1CC1 SLC38A2 TBL1XR1 SEH1L CAD EIF2AK3 SLC25A25 FOXO1 IL6 SSTR1

GO_LENS_FIBER_CELL_DEVELOPMENT The process whose specific outcome is the progression of a lens fiber cell over time, from its formation to the mature structure. Cell development does not include the steps involved in committing a cell to a lens fiber cell fate. A lens fiber cell is any of the elongated, tightly packed cells that make up the bulk of the mature lens in a camera-type eye. TMOD1 CRYGB WNT7A VIM WNT7B EPHA2 WNT5B TBC1D20 BFSP2 PROX1 FRS2

GO_POSITIVE_REGULATION_OF_VIRAL_GENOME_REPLICATION Any process that activates or increases the frequency, rate or extent of viral genome replication. CCL5 RAD23A NUCKS1 TOP2A DDB1 TARBP2 CD28 SRPK2 PKN2 PPID ADAR NR5A2 NOTCH1 STAU1 TRIM38 ADARB1 PPIE SRPK1 LARP1 YTHDC2 PPIH PTPLAD1 PPIB IFIT1 VAPA FKBP6 VAPB PABPC1 TBC1D20 PPIA STOM DDX3X

GO_REGULATION_OF_LIPID_KINASE_ACTIVITY Any process that modulates the frequency, rate or extent of lipid kinase activity, the catalysis of the transfer of a phosphate group, usually from ATP, to a simple or complex lipid. CCL21 CCR7 FLT1 EEF1A2 PIK3R5 NRBF2 KIT PIK3R6 DAB2IP FGF2 CDC42 PTK2 WASH1 VAC14 PIK3R3 NOD2 VAV3 PDGFRA SRC F2 PIK3R2 PDGFB AMBRA1 CD81 PIK3R1 KIAA0226 PPP2R5A ATG14 RBL2 PTK2B KLF4 PIK3R4 CCL19 RAC1 RBL1 TNFAIP8L3 LYN EPHA8 IRS1 FLT3 FGFR3 TEK TGFB1 FGR VAV2 RB1 PIK3IP1 CCKBR PDGFRB

GO_REGULATION_OF_SPINDLE_ORGANIZATION Any process that modulates the rate, frequency or extent of the assembly, arrangement of constituent parts, or disassembly of the microtubule spindle. VPS4B CHMP4C CHMP4B TPR SENP6 C1orf96 RNF4 CHMP2A CHMP3 CHMP1A CHMP5 PLK1 CEP97 PDCD6IP CHMP2B CHMP1B

GO_CELLULAR_RESPONSE_TO_DEXAMETHASONE_STIMULUS Any process that results in a change in state or activity of a cell (in terms of movement, secretion, enzyme production, gene expression, etc.) as a result of a dexamethasone stimulus. MSTN ERRFI1 SRD5A1 FOXO1 DNMT3B AQP1 SMYD3 STAR CRH RPL32 ASS1 TFAP4 CASP9 IL6 TGFB1 CCL2 EGFR ARG1 FBXO32 IFNB1 FECH HNRNPU SERPINF1 AGTR2

GO_GOLGI_VESICLE_TRANSPORT The directed movement of substances into, out of or within the Golgi apparatus, mediated by vesicles. VAMP4 VPS13C PROS1 COG5 TRAPPC9 LMF1 F9 DYNC2H1 RAB14 KIF3B F10 MCFD2 GBF1 RAB2A SGSM2 TMEM115 EXOC2 ANK3 SPTBN2 KDELR1 HTT ARF5 RAB6A COPG SNX2 ATP8A2 RAB6C SPTBN4 PITPNB TRAPPC4 RACGAP1 UVRAG TRAPPC8 COMMD1 CUX1 DYNLL2 GOLGB1 GOLGA1 DCTN1 RINT1 CORO7 VTI1A DYNC1LI2 SNX8 INS MYO1B VCP KIF18A DCTN5 CYTH3 PICK1 GOSR2 F2 SORL1 KLHL12 TGFA ARF1 DYNLL1 KIF5A LMAN1L KLC2 VPS13A LMAN2 SEC22C ARF3 COPZ2 COG3 ATP8A1 GOLGA3 PRKCI PROZ AP3B2 F8 RAB41 COPZ1 GCC2 GCC1 STXBP6 NRBP1 AP4M1 BCAP29 SEC13 MON2 ANKRD28 KIF23 ARL3 CNIH3 TBC1D14 GORASP1 TMED7 SEC24D ANKFY1 DCTN4 WIPI1 YIF1A KIF1C GOLGA2 TMED10 RAB35 ARF4 TRAPPC10 STEAP2 TRAPPC3 KIF3A KIF2A YKT6 GOLGA4 COG8 DCTN3 F5 TMED9 DYNC1H1 DNM2 STX5 AP1G1 KIF22 TBC1D20 KIF26A CREB3L2 CTSC ERGIC2 TMED3 KDELR2 GAK ARL1 USO1 CHIC2 COG1 TEX261 RAB6B GOLGA5 BCAP31 RAB8A ERGIC1 GOPC GOSR1 RAB1A MYO5A COPA SEC23A STX18 F7 EHD3 RP2 C16orf62 ATP9A AREG DYNC2LI1 CTSZ KIF11 RGPD3 CTAGE5 TRAPPC6B SYS1 TMED2 DYNC1I2 ANK2 STX4 CNIH4 LAMP1 STX6 SEC22B COPE ATP9B KIF3C ARFGEF2 DOPEY2 ATL2 SAR1B COG7 PROC RAB1B USE1 SCYL1 SERPINA1 CCDC22 KIF4A VAPA KIF2B OSBPL5 BGLAP SEC23IP PPP6C TGOLN2 CCDC93 SCFD1 RAB7B RGPD5 DCTN6 DYNC1I1 RAB7L1 VAMP2 KIF2C TRAPPC5 DCTN2 SEC16B RANGRF NSF COPB1 NAPA CNIH2 RGPD2 BET1 SORT1 FOLR1 PREB YIF1B ZFYVE20 ARFGAP1 ANK1 GRIA1 SEC24C TRAPPC1 NKD2 NBAS COG2 TFG LLGL1 KIF15 RGPD1 SEC16A VTI1B DDHD2 KLC1 VAMP7 TRAPPC2 NAPG SNAP23 ATL3 COL7A1 KDELR3 ARFGAP2 WHAMM SCAMP2 KLHL20 RGPD8 STX17 CUL3 SCAMP3 RGPD6 GOLPH3L KIFAP3 GAS6 ARCN1 CNIH PPP6R3 SEC31A VPS54 HYOU1 EPS15 ARFGAP3 COG6 RAB10 COPB2 SPTBN5 RAB33B OPTN LMAN2L BNIP1 NRBP2 KIF16B VAPB PRKD1 CENPE CSNK1D SEC23B SURF4 SPTAN1 EXOC5 COPG2 MPPE1 TRIP11 RER1 EXOC1 SEC24A RAB1C KIF4B AP2A1 PACS1 TAPBP MIA3 SEC24B COG4 VAMP8 SEC22A GABARAPL2 BET1L AP1AR CD59 PPP6R1 ZW10 STX10 LMAN1 MYO18A SPTB SPTBN1 TRAPPC6A TRAPPC2P1 DNAJC28 SPAST CD55 SPTA1 BET3L IER3IP1 RGPD4 SNX1 GOLPH3 SCAMP1

GO_POSITIVE_REGULATION_OF_CELLULAR_AMIDE_METABOLIC_PROCESS Any process that activates or increases the frequency, rate or extent of the chemical reactions and pathways involving amides. RXRA SAMD4A GUF1 EIF5A2 DDX3X PASK PICALM EIF6 SMPD2 PABPC1 CSDA PION NSUN4 DEFB103A RPS4X ZC3H12A NPM1 USP16 KRT17 BARHL2 NCK2 CLU KHDRBS1 BCL3 EIF2B5 PPP1R15A POU5F1 EEF2 LARP1 PTK2B CCDC56 GNB2L1 IMPACT ITGA2 C6orf125 EIF5AL1 WIBG SERP1 NCOR2 CPEB3 PRR16 POLR2D LRRTM3 RMND1 IMP3 C1QBP PLA2G6 BOLL DND1 DEFB103B LIN28A TNF CCL5 SOX4 MPV17L2 FAM129A YTHDF2 POLDIP3 NFE2L2 TGFB1 POLR2G YTHDF1 CIRBP ESR1 EIF5A NSMAF HNRNPD RPS9 ERBB2 SMPD3 RBMS3 CYR61 TNFRSF1A DNAJC3 NCK1 DAZ3 DAZ1 CSNK1E PRKCD FMR1 UPF3A DDX39B MAPK3 UPF3B IL6 UHMK1 CDC123 TARBP2 CD28 MAPK1 RPS27L UQCC NCOR1 EIF4A3 RPS6KB1 CDK5RAP1 DAZL RBM3 BCDIN3D EIF2AK4 METTL3 UCN LARP4B PAIP1 PIWIL2 PTAFR CDK4 MTOR ELAVL1 THBS1 RPS6KB2 PINK1

GO_FATTY_ACYL_COA_METABOLIC_PROCESS The chemical reactions and pathways involving a fatty-acyl-CoA, any derivative of coenzyme A in which the sulfhydryl group is in thiolester linkage with a fatty-acyl group. ACOT8 ACSL1 HSD17B12 ACSL3 ACOT7 ELOVL4 HSD17B4 FASN ACACA ACSL4 SLC25A1 THEM4 ACSM2A TECR PTPLB AGK ACSF3 ACOT12 SCD5 ACSBG1 SCD ELOVL7 DGAT1 ELOVL2 DGAT2 ACSBG2 PTPLA ELOVL6 GCDH ACSL6 FAR2 PPT2 ACLY ELOVL1 ACSL5 ACOT9 PPT1 ACOT4 THEM5 ELOVL5 ELOVL3 ACOT13 ACSF2 ACOT11 LOC344967 ACOT6 ACOT1 FAR1 ACOT2

GO_GLYCOLIPID_CATABOLIC_PROCESS The chemical reactions and pathways resulting in the breakdown of glycolipid, a class of 1,2-di-O-acylglycerols joined at oxygen 3 by a glycosidic linkage to a carbohydrate part (usually a mono-, di- or tri-saccharide). GBA NEU3 PNLIPRP2 HEXB NAGA NEU4 GALC GBA2 GLA GM2A NEU1 NEU2 GBA3

GO_REGULATION_OF_RELEASE_OF_SEQUESTERED_CALCIUM_ION_INTO_CYTOSOL Any process that modulates the frequency, rate or extent of the release into the cytosolic compartment of calcium ions sequestered in the endoplasmic reticulum or mitochondria. PKD2 CALM1 CORO1A IL13 THY1 GPER F2 GSTM2 ATP1A2 F2R CXCL10 JPH3 UBASH3B DIAPH1 ABL1 NPSR1 CAMK2D PLN CXCL11 SLC8A1 CXCL9 FKBP1B FKBP1A CALM2 CASQ1 NTSR1 JSRP1 BDKRB1 KIAA1199 TRPC1 SEPN1 CHD7 HRC PRKCE PTK2B PTPN6 CD19 MYO5A DMD NOS1 JPH4 XCL1 PDE4D SNCA CACNA1C JPH2 HAP1 PDPK1 PLCG1 AKAP6 DHRS7C CAPN3 ANK2 LACRT CALM3 BAX CLIC2 PRKACA DRD1 F2RL3 RYR2 JPH1 LYN GSTO1 TGFB1 CASQ2 TRDN HTT PRKD1 SRI CYBA

GO_G_PROTEIN_COUPLED_PURINERGIC_RECEPTOR_SIGNALING_PATHWAY The series of molecular signals generated as a consequence of a receptor binding to an extracellular purine or purine derivative and transmitting the signal to a heterotrimeric G-protein complex to initiate a change in cell activity. P2RY13 ADORA3 P2RY2 P2RY10 P2RY8 GNAI2 GPR34 ADORA1 P2RY1 ADORA2A ADORA2B P2RY11 P2RY6 GPR171 P2RY4 PTAFR GPR87 P2RY12 ADCY5 P2RY14

GO_PROTEIN_LOCALIZATION_TO_CHROMATIN Any process in which a protein is transported to, or maintained at, a part of a chromosome that is organized into chromatin. CCDC101 PLK1 ESCO2 H2AFY EZH2 H2AFY2 WAPAL HIST1H1B RAD21 RUVBL2 ESR1 LRWD1 PIH1D1

GO_PROTEIN_LOCALIZATION_TO_NONMOTILE_PRIMARY_CILIUM A process in which a protein is transported to, or maintained in, a location within a nonmotile primary cilium. TULP2 ARL6 BBIP1 TMEM107 C2orf71 TULP3 C5orf42 TULP1 LOC100507003 TCTN1 CC2D2A TUB BBS4 SPATA7

GO_TRANSCRIPTION_FROM_MITOCHONDRIAL_PROMOTER The synthesis of RNA from a mitochondrial DNA template, usually by a specific mitochondrial RNA polymerase. C17orf42 MTERFD1 SLC25A33 PPARGC1B MRPL12 POLRMT MTERF TFAM MTERFD2 C10orf2 TFB2M MTERFD3

GO_REGULATION_OF_CYSTEINE_TYPE_ENDOPEPTIDASE_ACTIVITY Any process that modulates the frequency, rate or extent of cysteine-type endopeptidase activity. HIP1 POR IFI6 CASP1 BCAP31 ACER2 AVP DPEP1 FABP1 GPER DLC1 RPS3 PAX2 LEF1 BCL2L11 APAF1 CRYAB TNF TBC1D10A MTCH1 SFRP2 DAP TNFSF10 ARRB1 LAMP3 TRIAP1 BIRC2 BIRC3 CRADD CST9 BID HSPE1 RIPK1 CST8 FIS1 EGLN3 PML BIRC7 MAP2K5 JAK2 TNFSF15 MYC S100A8 ROBO1 GNB2L1 HMGB1 CIDEB IL6 PRDX3 RPS6KA3 CST2 CASP2 HSPD1 EIF2AK3 P2RX1 IFI27 ALOX12 NDUFA13 HTRA2 MKL1 BIRC8 GAS6 PLAUR PIDD NLRP1 PSMB9 SERPINB9 CD27 CST6 THBS1 SOX2 SOX7 LGALS9 CASP7 RPS27L NGFRAP1 AIFM1 GPI CSNK2A1 CST4 MUL1 MDM2 CCK SIAH2 BAK1 CASP8 AQP1 RPS6KA1 SENP1 WNT9A MT3 TNFRSF10B NLRP2 APOPT1 CST9LP1 CST1 FOXL2 NLE1 BIRC5 ARRB2 CDKN2D CYR61 GSN F2R PAK2 BCL10 NGFR F3 CST11 NEK5 NOL3 RNF34 CYFIP2 FAS MAGEA3 XDH SRC VCP PDCD2 PCID2 FADD S100A9 YWHAE RAF1 NLRP12 HBXIP CSTL1 TFAP4 VEGFA CASP9 RFFL NLRC4 AKT1 VIL1 BCL2L10 PMAIP1 SIRT1 BCL2L13 TNFAIP8 PARK7 ARL6IP5 DAPK1 FASLG DDX3X IFT57 WNT3A BCL2L12 PIH1D1 KLF4 CARD8 NOD1 REST BAD HERPUD1 DHCR24 HGF GPX1 XIAP AIM2 ASPH HSF1 CST3 DNAJB6 CST7 BOK LCK CYCS FNIP1 RAG1 SNCA USP47 TNFRSF10A MAP3K5 ARL6IP1 SFN CST5 MMP9 NGF CD44 EPHA7 TP63 MGMT MEFV SMAD3 CASP8AP2 BAX PYCARD CASP3 NLRP3 TNFSF14 MICAL1 NAIP ANP32B CTSH PPARG TRAF2 TFAP2B SH3RF1 KIAA0141 PRDX5 TRADD CST9L IFI16 NKX3-1 COL4A3 ACVR1C PDCD6 NODAL PPM1F CDKN1B FAM162A PDCD5 DIABLO IGBP1 PERP CTGF

GO_MITOCHONDRIAL_ELECTRON_TRANSPORT_NADH_TO_UBIQUINONE The transfer of electrons from NADH to ubiquinone that occurs during oxidative phosphorylation, mediated by the multisubunit enzyme known as complex I. NDUFV1 NDUFA8 NDUFA6 COQ9 NDUFS8 DLD NDUFB4 NDUFB6 NDUFAB1 NDUFS1 NDUFA2 NDUFA5 NDUFA10 NDUFB10 NDUFB1 NDUFV3 NDUFS4 NDUFS6 NDUFS5 NDUFV2 NDUFAF1 NDUFB11 NDUFA13 NDUFC2-KCTD14 NDUFA9 NDUFS7 NDUFB9 NDUFS2 NDUFA7 NDUFB8 NDUFC2 NDUFA1 NDUFB2 NDUFB5 NDUFB3 NDUFA4 NDUFC1 NDUFA12 NDUFB7 NDUFA11 NDUFS3 NDUFA3

GO_REGULATION_OF_TELOMERASE_RNA_LOCALIZATION_TO_CAJAL_BODY Any process that modulates the frequency, rate or extent of telomerase RNA localization to Cajal body. DKC1 CCT3 CCT6A NOP10 CCT2 CCT8 SHQ1 NAF1 RUVBL1 NHP2 TCP1 CCT7 CCT4 RUVBL2 CCT5

GO_SUCKLING_BEHAVIOR Specific behavior of a newborn or infant mammal that results in the derivation of nourishment from the breast. POU4F1 UBE2Q1 APP OXTR DACH1 DERL2 UBR3 HAND2 GLS CNTFR PEX13 HELT GRIN1

GO_POSITIVE_REGULATION_OF_CYTOSKELETON_ORGANIZATION Any process that activates or increases the frequency, rate or extent of the formation, arrangement of constituent parts, or disassembly of cytoskeletal structures. WIPF3 WDR1 ARPC4 PROX1 KIRREL GPR65 MTOR CCL24 ACTN2 CYFIP1 WASH3P PAK1 SWAP70 CDC42EP5 ACTR3B MYOC ID1 WNT4 FES JMY ODZ1 MLST8 BCAS3 NCKAP1 ACTR3 RAC1 EDN1 TAC1 CDC42EP1 HAX1 C13orf15 WIPF1 DLG1 SCIN SPIRE2 TEK CDC42EP3 PFN3 MYLK3 NPHS1 HCK TGFB3 LMOD1 SFRP1 FER RLTPR F2RL1 ARPC5L ABI2 CLASP1 CCR7 RHOA PLK4 ACTR3C VASP PLXNA3 RPS3 CFL2 ARPC1A ARPC1B SYNPO2L SPIRE1 LPAR1 CLIP1 SPAST PSRC1 PTK2B GRB2 PRKCE RANBP1 ARF6 BAIAP2L2 P2RX7 VPS4B MYO1C NCKAP1L ROCK2 PFDN2 STAP1 ARHGEF15 WIPF2 LRRC16A FMN1 ARL2 SLAIN2 NOX4 SYNPO MAGEL2 CDK5 KATNB1 BAIAP2L1 PYCARD FAM21C SMAD3 WASL PFN1 ARHGEF5 CCL21 WASF1 SERPINF2 ARPC5 NES SYNPO2 LIMK1 NCK1 BAG4 HCLS1 ARPC3 TRPV4 RAPGEF3 CEP120 RICTOR LMOD2 PPM1F CDKN1B PDXP PFN2 CTGF APOA1 ARHGEF10 CTTN TGFBR1 S100A10 WNT11 BRK1 ICAM1 CDC42EP2 ITGB1BP1 BAIAP2 ANKRD53 TRIM27 FAM179B WASH1 CCL26 STMN2 MAPT BMP10 PLEK ACTR2 CDC42EP4 EVL CSF3 GSN BRAF DSTN NTF3 NTRK3 FHOD1 HRAS CAV3 EPHA1 C15orf62 RGS2 NCK2 ARPC2 WAS CCL11 WHAMM IQGAP2 NF2 VIL1 SEMA5A WASH6P SORBS3 SDC4 PPM1E TPM1 ALOX15

GO_NEGATIVE_REGULATION_OF_ANDROGEN_RECEPTOR_SIGNALING_PATHWAY Any process that decreases the rate, frequency, or extent of the androgen receptor signaling pathway. SIRT1 HEYL NODAL SFRP1 SMARCA4 TCF21 FOXP1 PIAS2 FOXH1 DAB2 HDAC1 PHB

GO_ASTROCYTE_DIFFERENTIATION The process in which a relatively unspecialized cell acquires the specialized features of an astrocyte. An astrocyte is the most abundant type of glial cell. Astrocytes provide support for neurons and regulate the environment in which they function. STAT3 GFAP PTPN11 KRAS DRD1 SHH CDK6 ABL1 MAPK1 S100A9 LAMC3 EGFR S100B SOX6 DLL1 NOTCH1 MAP2K1 MAPK3 TSPAN2 POU3F2 NF1 HES5 GCM1 SMO PLP1 S100A8 EIF2B5 TLR4 PPAP2B NKX2-2 SOX9 MT1X LAMB2 MT3 TAL1 CNTF VIM AGER PAX6 SOX8

GO_REGULATION_OF_PHOSPHORUS_METABOLIC_PROCESS Any process that modulates the frequency, rate or extent of the chemical reactions and pathways involving phosphorus or compounds containing phosphorus. ADCY7 DUSP4 ERBB3 RASGRP1 RTN4R CXCL9 CDON BDKRB1 GPLD1 BARD1 VEGFA PRNP ALS2 DYNLL1 TMEM188 HSF1 MAP3K1 FGFR2 FBXO18 RGS2 EPHA4 TBC1D10C MPHOSPH10 MAD2L2 LDB2 NSD1 BPGM MDFIC GBP1 SRC PKD2 MDFI CRYBA1 IBTK KCTD20 SORL1 GDF9 DNAJC27 LMO4 CDA NTRK2 ANGPT1 TARDBP PPP1R16B TNFRSF19 FABP3 NPTN PPEF2 ADCY5 FAF1 ACVR2B GPR87 RPS6KA4 OPRD1 KIAA1244 SPTBN4 GABBR2 SNX9 LYN SEMA7A DUSP15 PRKAR2A FLRT3 EFNA1 POU1F1 CRLF1 PF4 RAPGEF2 DRD5 GP1BA HDAC1 RAP1A TGFB2 NODAL PPP2R2D RNF149 GUCA1A HTR2A CDC37 CARTPT TRPT1 SFN O3FAR1 AIDA FOXA2 CAV1 PRKACB KIT HIPK2 MST1R PDE6H AXIN2 CARD14 NOD2 SPDYE7P CDC42 CCL23 IFNA7 FGF9 GPR161 NPM1 GPR183 BDKRB2 NR2F2 AREG ZNF622 DUSP22 LRP8 ROCK2 ATP7A CDK10 CNTN1 NOX1 RBM26 PRKACG CDK2AP1 IL20 TAF7 LAT PHLPP1 NYX ALK IQGAP1 DUSP3 PPP1R35 OPRK1 DUSP21 PIN1 PILRB DBNDD2 AGTR2 GMFB DUSP18 DUSP2 SLAMF1 EREG GHRH PRKAA1 ADARB1 GDF15 FGF16 NDRG2 SNF8 PRDX1 CHRNA3 JUB KNDC1 CD24 DUSP7 RRAS CCNL1 HDAC2 PPARGC1B TAOK3 OPRM1 HES1 GDF7 CHRM5 HEXIM2 TNFRSF6B CCL22 PRKCDBP MLLT1 FER MAP4K2 TGFB3 CDC25B HAND2 PKIG MAP4K3 FGF18 ZGPAT SYMPK GPR116 ISL1 FGF13 RIMBP2 WWC1 PPP1R39 CCL2 ARRB2 PLA2G2A HAX1 ADM EDN1 FBLN1 CALCR ANGPT4 TNFRSF18 GUCA1B NLRC5 ITGA1 VLDLR HRH4 RRP1B PPP2R2A WNT16 BMPER KIRREL2 PRDX3 LRRTM1 NME2 IL26 CDK5RAP1 CTNNB1 DRD2 THBS1 FARP1 ACVR1B UCN PTK2 CNTF MVP SSTR4 OGT GRM8 GLMN ZC3H12A KITLG CALM2 CCNYL1 BMP3 GRXCR1 GPS2 IL4 DUSP16 NTRK3 C1orf177 MAP3K2 CDC25A MAPRE3 PDGFRA TNFRSF4 IMPACT FGF20 NGFR TRIM5 IQGAP3 DOCK7 ADAM9 PPP2R5B CSF3 KIF14 FOXM1 H2AFY TTK SYT14L CCNE1 AHSG MAS1 GATA1 INPP5F CKS2 ELANE FGR PKN1 TNFRSF25 NEK10 TAOK2 TMEM225 KIAA0226 CXCR4 CBLB CORO1C CLIP3 PTPN22 TRPV4 PSAP PRKAR1B PYDC1 GIPR HCLS1 HTR1E HPX INPP5J XCL1 CCNC PRKCD TRAF4 DSCAM TWIST1 RTN4RL2 MAP3K5 LRRK2 MRE11A NPPC GCKR CASC5 GNAI2 PPP2R1A CD4 ERCC6 GMFG GSTP1 WNT7B SOD1 CBFA2T3 GPATCH2 CHAD CD244 INHBE TARBP2 FIZ1 CCL17 PPP2R1B SMAD3 CD44 GHRHR CDK5 DUSP19 SDCBP LRRC4C RIPK1 MAP2K3 KIAA1199 TXN PDGFRB RASSF2 IL29 BTBD10 PRKCE MASTL SMG5 PAFAH1B1 PPP1R36 LPAR1 TRAF7 MUSK CCR2 PTPRC DIRAS3 GNAI3 STRADA DLC1 C6orf125 COPS8 FAM150B THY1 ECT2 GRB10 PPIF SMG6 NPFFR2 P2RY1 PPP5C HYAL2 PPP2CA CDKN2A FGB TGFBR3 CDK5R1 LRRC15 IFNE AGT PIK3R3 KRAS DIRAS2 PRKDC PPP1R26 TSC1 MAP2K6 CNKSR3 PAK3 IFNA21 UBN1 ADRB2 VAV2 PRKD1 GLP1R IGF2 C18orf1 SLC7A14 INHBA MAVS PRR5 CD40LG PLCE1 PPP2R5E GRM7 PFKFB1 GUCA1C SLC25A23 TNFRSF11A RUNDC3A CCL19 CCL14 AR MSTN ADRB3 KDM4D RDBP GDF6 MST1 CHP MLXIPL ILK ACTN3 DUSP1 ZFYVE28 PER1 PTAFR CSK CHP2 WNT3A BMP1 AGER EPB49 ALOX15 CTDSPL SNX6 SLC11A1 AKT1 IKBKB TFAP4 CCNT1 FZD7 CCL11 EPHA2 CCNL2 MADD PLCL1 NPR1 ADRA2B HGF SERPINB3 CELSR3 KIAA0430 IL23R STRAP C1QTNF2 ACVR2A SH2D4A LRP6 AKTIP MYO1D GADD45G OSBPL8 ITGA2 ELP4 CLSPN C12orf5 STOX1 ATPIF1 TRPC6 F2 PRKAG1 TGFA ELFN2 CD3E IRS2 PARD3 ADORA2B RGS4 LAMTOR3 BMP10 EGFR WDFY2 ENPP1 ANG ADAM17 PRLR BMP8B CCL26 C21orf7 UCHL1 P2RY13 ICAM1 WDR81 RSPO1 CTGF HMGCR PPP2R2B SMPD1 LAMTOR1 IGFBP4 RAPGEF3 CDK9 IGF1R PKIA CD2BP2 CTDSP1 ABCA7 KIDINS220 ZMYND11 MAPK8IP3 PPP1R11 ACVRL1 HDAC3 CIB1 PPP1R17 CBLC SNCA AKAP6 DVL1 CCNT2 FAM20C CCL4L2 ARHGAP8 ARPP19 MAPK1 PSEN1 STAP2 DAB2IP LAX1 TWF1 BAX CARD9 STAT3 MAP3K15 ASPN EPHA7 TNFRSF11B MECOM SAA1 ERBB4 C3orf33 PML FKBP1A HSP90AB1 TNFAIP8L3 MAP3K14 CDCA2 C19orf2 PLA2G1B NPRL2 BCL2 IL22RA2 EGLN1 PIK3R1 GHR CEBPA CHEK2 MAP2K2 DEPTOR GNB2L1 GRM3 GRB2 MAPK8IP2 TLR4 IL2 CCL4 TBX1 INPP5K WEE2 FGF19 FBXW7 MAP3K4 CCNYL2 CSF1 ELL LRRC66 WNT9B SFRP1 MAP3K11 SOCS5 EPHB1 IRAK2 VIP HPCA RBL1 MAP3K8 INHA DTNBP1 NOG PPP2R5D SOCS1 MAP4K5 PPP1R14A PRKRIP1 WWTR1 LAMTOR2 RAC1 ADAM8 SHB EDNRB FKBP15 NRG1 GCNT2 FOXO1 CCL3L3 PDE4D EEF2K GALR2 CEACAM1 CDKN1C ACMSD MYOC MAP2K1 AKAP9 IL6 MIDN SASH1 MAPK8IP1 IFNA5 PKMYT1 NCOR1 SOX2 CD27 VIPR2 TLR3 NDRG4 RYK TSKS PARK7 WNT7A PARP16 C1QL4 NPNT GSK3B IL12A APLP1 NTSR1 GRM2 FAM58BP CRH CRKL PTGIR MYOG MAP4K4 NLRP12 CTDSP2 PTHLH CLU CEP192 NCK2 PRMT1 BMP8A ZAK IFNA16 HRAS VANGL2 PROK1 STK11 PDGFC XDH HGS TNIK WNK3 PPP1R12A IKBKAP PPME1 LRRTM3 PAK2 RXFP2 PDE6G PFKFB2 GDF2 ECD CSNK2B ADRA1B PLEK YWHAE DDR2 KAT2B TSC2 ANKRD54 PTPRJ INCA1 RAP2A DVL3 INHBB RIT2 CBL EZH2 SENP2 SH3RF1 MARVELD3 MEN1 CDC6 IFNK EZR IL3 SEMA4D GAPDHS ERBB2 CCL20 RAD51 GDF1 IRAK3 RIPK2 NKX3-1 NCK1 NPY5R TNK2 PIK3R4 MMP9 PPP1R27 RAPGEF1 IFNW1 HAVCR2 SPHK1 PIK3CB NUP62 PAQR3 FGFR4 ARHGEF5 ADCYAP1R1 PPP2R4 VRK2 NRP1 PINK1 LEP PDCL3 DUSP10 RARRES2 HRH1 GPR133 YWHAG MAP2K5 CD74 TLR7 PALM CRHR1 APOE SOCS6 TNIP1 ITGB3 FLCN IFNA4 BLM PPP1R42 RELN FAM83D CCNY WNT5A SESN2 DAB1 CRY2 FGF1 HRC STK3 UNC119 PELI2 PWP1 IFNAR1 GPR55 IL23A SIRT2 CDC25C RET MAGED1 ADAR CDK7 VEGFC ODAM RHOA PPP6R1 PPP2R3B PRKAR2B SPDYE5 SRPX2 MKI67IP TNF WNK1 IL6ST GPD1L IFNG CSNK1D RBPMS MT3 TNFRSF10B TP53 PGAM4 CAPRIN2 CDKN2B BAK1 FLRT1 DVL2 NLRP2P GDF5 GADD45GIP1 SLC25A33 TESC SPN FN1 GH1 DAG1 CCL15 ARNT BGN ADRB1 FAM58A FZD5 CCNG1 COPS5 ATF3 SPINK1 CCNE2 GLIPR2 C1orf88 CREBL2 HSPA5 STRADB LGALS9 UBE2K CSF2 CCL3 HFE C1QTNF1 NPHP3 PPP1R14B KL EDNRA SPDYE2L ARL6IP5 CD36 CCL13 SDC4 FGFR3 CCND1 PPP1R2P3 TRAF3IP1 AKAP8L PPAP2B MC2R DKK1 MBIP EPHA1 CAV3 NOD1 C5AR1 RBL2 PIH1D1 S100A7 IDH1 SET CALM1 DIRAS1 NTF3 RPLP1 EHD4 MIF BMP2 BRAF PDGFA MUC20 CLCF1 CCNA2 DOK7 RANBP9 ADRA1A CXCR7 AFAP1L2 BCL10 MAP3K6 PDGFD FAM129A PBLD MAPK14 PBK NELF STK39 VAC14 ME2 DUSP26 ITGB1BP1 MAGI3 CCL3L1 OPRL1 CCNB1 PLA2G5 ALOX12B TRAF6 FGA CD81 WNT1 SNX25 RICTOR PPARA SOCS3 C10orf46 LDB1 PLCB1 LRRTM4 ABI1 CDK12 SOCS2 ACSL1 FNIP1 DAXX LMTK3 MAP4K1 NRBF2 GPR37 PTPN1 CD300A CCL21 SPDYA IL12B CCNB3 SCT PYCARD CDK4 DRD1 DUSP5 FGFR1 SQSTM1 SPRY2 CXCL11 GNG3 TNFSF11 PTK6 HSP90B1 ADIPOQ VRK3 PODN EMP2 LRTM1 RTN4RL1 GNAQ PGAM1 MAGI2 DSTYK PSRC1 ASH1L PPP4R4 TMEM102 SLIT2 RAP2C TRPC5 RAD50 EPGN SYNGAP1 HTR2C FLOT1 IL34 GPER FBP1 NTRK1 KSR1 PPP1R15B CARD10 CCL8 PPP2R3A MYCNOS CSRNP2 FGF23 TRIB3 TEK TPD52L1 EDA2R GPRC5A ENPP2 ME1 CCKBR SMG8 TLR6 ITLN1 FABP4 HEXIM1 ZNF16 DBNL RALB TELO2 FGF7 ZFP91 STK38 IL18 CCDC8 TBL1XR1 DUS2L KISS1 ODZ1 MAP2K7 CD109 SYTL2 ID1 IFNA13 PDPK1 SWAP70 CD80 ZCCHC9 NBN PRMT5 EGF ZFYVE1 PTH1R RB1CC1 IFNB1 ADCY4 PRKACA ERP29 GSK3A MAPK7 C17orf87 UBC RPS6KA6 PPARGC1A UBB APLN BMPR2 RB1 CAP1 MINK1 TSG101 IGFBP3 PPP2R5A ABCA1 TADA3 ENTPD5 FAM20A NOS1 LATS2 CSPG4 SMG7 FGF4 DNAJC15 PIBF1 PCIF1 IFNA10 ADRA2A APOC2 GRM1 TDGF1 HIPK3 GAB1 CD40 INHBC NENF CAMK2D AVPR2 TRIM27 TRIB1 CAMK2N1 CDK1 CCNK IL11 ADCY1 MAPKAPK2 JUN RCVRN JTB APOA1 ENSA REN SAMSN1 CDKN1B IFNA1 NPY2R GPS1 BAG4 NBR1 NRK NGF DDRGK1 MAPK3 LTB4R2 CCL16 PDE2A EFNA5 PIK3CA TIMP3 LRP4 PIK3R5 FLT1 MAP3K13 CISH MICAL1 SPRED2 FZD4 FLT3LG IL21 XCL2 UNC5CL ADNP PRKCZ CCL25 JAK2 BIRC7 S1PR2 AKT1S1 ARHGEF2 MAP3K12 NEURL RPTOR CDH2 ZBED3 CAB39 MAPK11 AKT2 ITGA5 CHI3L1 ROS1 SOX9 NOS3 CAP2 ATP2B4 HMGB1 PTK2B CHORDC1 PTPN6 PPP1R15A ADCY8 IL13 ERRFI1 FZD8 AVP CAMK2N2 DUSP6 CXCL10 PTPN2 C19orf29 LIF APC ABL1 FGF10 CCDC88A SOCS7 TAOK1 F2RL1 NUAK1 FGF2 BMPR1A TNFAIP3 KDR GPD1 SEMA4C DBF4B PPP1R7 CENPE PCDH11X CSRNP3 SPPL3 SHC2 FZD1 ADORA2A PPP1R2 GCK CCK ADCY9 LRP5 DLG1 LPAR3 MAPK10 ELP3 GUCA2B MUL1 HSPB1 CYR61 CCND2 ENG IL5 PRKD2 MAP3K10 PRKAR1A CHGA TNFRSF1B RAMP2 TIMP2 MAP3K9 DACT1 SPRY4 GCN1L1 LHCGR C19orf10 PLAUR GAS6 PPP6R3 ATP6AP1 PDCD10 MMD TGFBR2 IKBKG CCR1 MLST8 NRG3 TAB1 TSPYL2 GPR77 MAPKAPK5 LACRT PAK1 PPP2R2C EIF2AK1 SYK SMAD7 PPM1E TLR8 YES1 SORBS3 CEP85 BANK1 MID1 FKBP1B PAX6 SIRT1 NF2 ZP4 PRKAG2 SPAG9 FNIP2 TP73 FKTN CRIPAK PPP1R14C SOCS4 BRAT1 SMAD4 GDF11 BAD CNPPD1 RNF41 DNAJC10 RAP1B FAS MMD2 NF1 EDAR SPDYE6 DUSP8 CYSLTR2 ARF1 BMP7 STK40 OSTN BMP15 MYOCD INS PRKCA PLA2G6 VCP PMEPA1 SPDYE3 WASH1 HERC5 UBE2B OXER1 PPP1R1A SRCIN1 CKS1B STK4 TEC SPRY1 WNT11 UVRAG INSM1 TRAF2 PIK3IP1 TGFBR1 CAMSAP3 TMEM132D TGFB1 IGBP1 ERN1 HTT IL15 TIRAP YWHAB PPM1F KLB FGG ELFN1 SH3BP5L GADD45A FGD2 SFI1 GALR1 PLK1 TNFRSF10A PPP1R2P9 LATS1 ADORA1 GHRL CCL1 HHEX RAD17 ATXN7 P2RY11 KIAA1804 PEA15 SLC9A3R1 PTH LMO3 MNAT1 ITSN1 THPO STAT2 LEFTY1 ADRA2C TNFSF15 MC4R GUCA2A TNFRSF14 PHB2 PARK2 ADCYAP1 LTF PIK3CG CCND3 MALT1 C5 HSPA2 MOB1B DRD4 SHC1 GFRA2 MYC TRIB2 PKHD1 RGS3 FYN HIF1A HES5 UBA52 AMBRA1 NOS2 EEF1A2 IGF1 TSPO UBASH3B VTN IL6R ARRB1 BRD4 HCRTR1 HUS1 CALCRL FZD2 ITGB2 GUCY1A3 GDF3 CAV2 SPDYE1 ITPKB CDKN1A GADD45B CDKN2C SMAD6 IFNA8 APIP AKAP8 FLT3 EIF2AK2 GDF10 ITGA6 GRIK2 FGF21 IL1A PPP1R1C DUSP12 DNAJA1 CDKN2D CAMK1 PTPLAD1 DUSP9 BMP4 CTF1 CNST RLN2 CDKN3 CCDC88C GUCY1A2 TLR9 IL24 SERTAD1 SYNJ2BP FGFR1OP EIF2AK3 PDZD3 CCL18 LRRK1 PIK3R2 HSPH1 EIF2AK4 IFNA6 HTR2B PHB CCL24 GPRC5B PPP2R3C CALM3 PTPN11 GPR65 MTOR CAMKK2 NQO2 AIPL1 EIF6 LEFTY2 DLG2 MEF2C FPR1 RIPK3 ARR3 ARL2BP IRS1 TERF2IP RAF1 IRAK1 TIAM1 MARK2 ACE C3 EDN3 KLF4 MAP3K3 BCCIP MAP3K7 RHOH PDCD4 GBA RGN RGS14 AKAP12 SIRT3 FAM59A TAB2 AVPI1 TOM1L1 FLRT2 F2R PPP1R2P1 PDE5A FAM150A DRD3 STYX ITCH DLG3 CCL5 TAB3 AIF1 PTPRR COX11 PHIP FGF8 RELT PROK2 TNFSF18 PRKAA2 STOML2 GALR3 HDAC4 MOB2 TNFRSF1A PFN2 RAP2B WNK2 INSR S100A12 CDK5RAP3 NLRP6 PSMD10 MTNR1A ATG14 STK25 LRRC4 ARAF CDK5R2 EIF3A SERPINF2 MCM7 FMR1 PPP1R37 CCL7 SLC39A10 SIRT6 MC3R DCN AXIN1 PROM2 PRKAB2 PID1 PLCG1 SPDYE4 CRK SLC27A1 LCP2 PLCL2 CSF1R MOS IL31RA OSM RABGEF1 NOX4 C18orf26 MRAP2 MC5R GPR37L1 MAPKAPK3 WFS1 IL1B TBK1 DAB2 PPP2R5C EPHA8 PLXNB2 AKAP5 SMYD3 CCNH STAP1 PODNL1 DDIT4 MAP2K4 AKAP13 P2RX7 ADCY2 NCKAP1L PKD1 PTEN ACVR1 ANKRD6 IFNA14 LTBR MRAP BMP5 DMD PGAM2 ADCY6 HRG RPS3 NDUFS4 LRRC4B RAC2 CHRNA7 AZU1 PARD6A PPP1R9A PPP1R1B ZP3 ANXA2 PDGFB CALCA RPS6KA5 HTR1B TPX2 AMBP CCR7 DNM1L IFNA17 ANKLE2 SFRP2 PIK3R6 FCER1A FRS2 FLT4 RANBP2 GABBR1 LRRC19 LEMD2 FSHR ROCK1 PKIB RAMP1 THBS4 TIPRL TTN ATM FZD10 RPS27A C13orf15 VAPB MYADM BMP6 CX3CL1 GNAL SPOCD1 IFNA2 ZNF675 PPP1R14D PRR5L DNAJC3 GTPBP4 ACR GCG EPO TREM2 GTF2H1 FBXO7 TRIM6 PRKAB1 APP MC1R TSHR ADCY3 SPRED1 BCAR3 ULK4 GREM1 KIRREL PROX1 SH3BP5 VAV3 DUSP14 GRM4

GO_KERATINOCYTE_PROLIFERATION The multiplication or reproduction of keratinocytes, resulting in the expansion of a cell population. Keratinocytes are epidermal cells which synthesize keratin and undergo a characteristic change as they move upward from the basal layers of the epidermis to the cornified (horny) layer of the skin. KLK8 FGF10 PTCH1 IRF6 CDH13 EREG PPARD KRT2 SDR16C5 FERMT1 TP63 WNT16

GO_PROTEIN_LOCALIZATION_TO_LYSOSOME A process in which a protein is transported to, or maintained in, a location within a lysosome. HBXIP SORL1 HSPA8 AP3B1 SNX16 GCC2 RAB7A HGS ZFYVE16 C7orf59 NCOA4 RNF128 GNPTG GNPTAB NAGPA AP3M1 SCARB2 WASH3P NEDD4 SH3BP4 CD81

GO_POSITIVE_REGULATION_OF_BLOOD_PRESSURE Any process in which the force of blood traveling through the circulatory system is increased. CARTPT PDE4D ADH5 ID2 F11R ADRA1A CHRNA7 OXT ADORA1 AVP TBXA2R AVPR1A UTS2R ADRA1D ADRA1B CNR1 WNK1 OR51E2 HSD11B2 AGT OXTR AVPR2 CYP11B2 TPM1 ADIPOQ NR2F2 ACE QRFP DRD5 TACR3 GLP1R C12orf39 ADRB1 NPFF UTS2 CYBA

GO_GLIAL_CELL_DIFFERENTIATION The process in which a relatively unspecialized cell acquires the specialized features of a glial cell. KLF15 ADAM22 CDKN2C KCNJ10 VIM MT3 MMP24 DAG1 FGF5 GCM1 GAP43 NAB2 MYOC MAP2K1 EIF2B4 ILK ZNF488 ODZ4 PHOX2B SKI TSPAN2 NRG1 CNTN2 SOX11 CSK PTPN11 SOX10 C11orf9 EIF2B1 SOX2 CTNNB1 LAMB2 NDRG1 SOX8 OLIG2 TAL1 METRN TLR2 C1orf130 LPAR1 TLR4 ID4 S100A8 PLP1 EMX1 NKX2-2 SOX9 CDH2 AKT2 PTEN RELN NOTCH1 EIF2B3 S100B NKX6-2 PAX2 HES5 SIRT2 GPC1 NFIB LEF1 KRAS OLIG1 NLGN3 DNER SLC8A3 SOX4 ABL1 HES1 SRSF1 VTN CD9 FGF10 ARHGEF10 NAB1 TGFB1 LAMA2 CNP LYN ASCL1 SH3TC2 SMO ERBB2 NEUROD4 DLL1 PTPRZ1 POU3F2 EGR2 ID2 MAPK3 CDK6 DRD1 DUSP10 CDK5 STAT3 LAMC3 MAPK1 HDAC10 GSTP1 SOD1 NKX6-1 PHGDH ERCC2 AKT1 PAX6 AGER CNTF ERBB3 MED12 HDAC11 EIF2B5 EIF2B2 PRDM8 TRPC4 MPP5 MT1X BOK PPAP2B CLU MXRA8 FA2H NKX2-1 SOX6 GLI3 NTRK2 PICK1 WASF3 NF1 LGI4 SHH POU3F1 GFAP PARD3 DICER1 EGFR S100A9 DTX1

GO_MATING The pairwise union of individuals for the purpose of sexual reproduction, ultimately resulting in the formation of zygotes. HEXB OXTR PI3 KLK14 EDDM3A DRD1 NCOA1 SERPINE2 AVPR1A TH OXT SLC6A4 AVP PPP1R1B ABAT APP UBE2Q1 DDO P2RX1 MTNR1A MAPK8IP2 TAC1 ACVR2A SEMG1 DRD5 PTEN GRIN1 OR2H2 DMRTA1 THRB NHLH2 THRA C10orf125

GO_ACTIN_FILAMENT_BASED_PROCESS Any cellular process that depends upon or alters the actin cytoskeleton, that part of the cytoskeleton comprising actin filaments and their associated proteins. CORO2B ARHGAP6 ARPC2 WHAMM WAS BRSK2 IQGAP2 CASQ1 DIAPH3 TRIM32 BCAR1 TPM3 TPM1 FRYL INF2 SELE TNNC1 TNNT3 MYRIP MYH9 TNNC2 FSCN2 CAP1 NCK2 DNAJB6 MINK1 PARVB ACTA1 ALKBH4 LLGL1 ADRA2A NUAK2 TNIK CALR SCN4B AMOT PLEK2 PDGFRA RND1 TRIM27 FRMD3 AIF1 MYL4 KCNE1 CAMK2D MYO7A FAM40A PLEK ACTR2 TRIP10 S100A9 FHL3 EVL FRMD6 CTTN MOB2 CXCL1 EZR OPHN1 CSRP3 SUN2 BRK1 INSRR CDC42EP2 COBL BAIAP2 RAP2A AKAP2 ATP2C1 HCLS1 NCK1 SORBS2 PREX1 TRPV4 MYL1 LMOD2 GJA5 CORO1C SSH2 TAOK2 EPB41L5 FGD6 PFN2 MYPN TNNT2 NKX2-5 MYL6 CAPN10 CACNA1C MYOZ1 ABLIM3 MAPKAP1 LMOD3 PRKG1 ANTXR1 CDK5 NPHP4 MAGEL2 MTSS1 KLHL1 BAIAP2L1 MICAL1 YSK4 MSRB2 GMFG ADD2 ANK2 MYH3 ARHGEF5 KCNJ2 PDGFRB ARHGEF2 SDCBP COBLL1 RHOD FMN1 S1PR2 MYOZ2 PRKCZ JAK2 SPTA1 CCDC155 RHOU DMD PAFAH1B1 ADD1 MYH10 PTK2B TPM4 SSH1 DPYSL3 PHACTR4 ARF6 MYO18A CAP2 BAIAP2L2 FMNL3 ESPN NCKAP1L CLRN1 IQSEC1 RHOA PPP1R9A TMSB4Y DLC1 RAC2 SEPX1 ARPC1A FMN2 KRAS TNF GPD1L PACSIN2 RLTPR PTK7 KCNQ1 NUP155 EPB41L2 ABI2 ARPC5L ABL1 FGF10 ARHGEF11 SCN2B ALDOA TTN WIPF1 CYTH2 SCN3B PKP2 CASQ2 SCIN DLG1 SPIRE2 MYH7 NPHS2 FLNB SCN1B TCAP ROCK1 CAPZB PFN3 AQP1 MYBPC1 ZYX IQSEC2 VIM FMNL1 PIP5K1C MYOZ3 MYL2 PDLIM7 PRKAR1A FHOD3 CORO2A MYH2 SPTBN5 ABR DBN1 TMOD2 MYH11 FLNA NEDD9 PDCD10 MRAS F11R RAB13 RAP1GDS1 ARPC4 PDLIM3 OBSL1 PALLD PROX1 WASF2 CUL3 PAK1 ACTN3 IQSEC3 MYL6B ACTR3B ACTC1 FAT1 MYBPC2 IKBKB CCL11 NF2 VIL1 C1orf190 TNNI1 WASH6P SORBS3 EPB49 ABLIM2 MYH14 RALA BIN3 SCN5A PRKCI FMNL2 FGD4 TMSB4X MAD2L2 KCTD13 MLPH KIAA1598 SLC9A1 CAV3 ATXN3 DOCK2 EPB41L1 SHROOM2 FOXJ1 DYNLL1 LDB3 SHROOM1 ARF1 FARP2 SIX4 EPS8 KLHL17 MKLN1 GSN KCNA5 DSTN XIRP1 TNXB TRPM7 PPP1R9B FRMD5 ARAP1 PDGFA MYO6 CORO6 WASF3 ACTN1 NF1 XIRP2 KRT8 SRC SYNE2 ACTG1 ARHGEF18 WASH1 MYO1B LLGL2 MYH8 TNNT1 ANG NEB BMP10 TMOD3 DIAPH1 FNBP1L CORO7 C2orf62 KCNE2 RND3 KCNH2 RACGAP1 TNNI2 ARHGAP26 EPB41L4B FAM101A ARPC3 INPPL1 TPM2 GJC1 CFLAR EPB41L4A KCNE1L RICTOR PDXP RHOG MYH4 MICALL2 GHRL WASF1 CTNNA1 CACNB2 FSCN3 ARPC5 MYH6 TMOD1 FLII ABI1 FGD2 ZNF664-FAM101A TMSB15A LIMK1 TMSB15B EPB41L3 CDC42 CDC42BPB PLS1 WASL NPHP1 KIT PFN1 MEF2A RHOQ SLC9A3R1 CAPN3 CNN3 SORBS1 PTPN1 TLN1 ROCK2 RHOJ BCL2 TRIOBP HSP90B1 ARFIP2 CDC42BPG PLA2G1B WIPF2 LRRC16A DAAM2 TNNI3 FSCN1 SPIRE1 SIPA1L1 SHC1 ERMN MYO5A MYBPC3 ARHGEF17 CORO1B CXADR MYO1E EMP2 RUFY3 NEDD4L BCL6 PPARGC1B S1PR1 ANXA1 VASP ACTR3C ACTN4 RAC3 ITGB5 FAM101B TMSB10 FRY FGF12 INPP5K SDAD1 CACNA2D1 CORO1A CFL2 ARPC1B GAS2L3 PAWR OBSCN GDPD2 ITPKA MYLIP PIP5K1A KRT19 LMOD1 FER BCR MYO9B ARRB1 PRKCDBP DIAPH2 MYL3 TESK2 LCP1 CNN1 EPB41 DTNBP1 EHD2 NEBL LIMA1 KBTBD10 FGD5 MYLK3 SRGAP2 MICAL2 TMOD4 FGD3 LIMCH1 RYR2 TTC17 FGD1 ACTR3 RAC1 FGF7 PARVG DAAM1 TNFAIP1 ANKRD1 MOBP CFL1 ELMO1 PDPK1 MICAL3 PHACTR1 JMY NISCH ITGB1 GJA1 MKL1 WDR1 WIPF3 PARVA CNN2 GPR65 ABL2 PLS3 ACTN2 SHROOM4 RHOF DES WASH3P SNTA1 CDC42BPA SRF KPTN KIF23 SWAP70 ARHGAP35 PACSIN1 GHSR

GO_SARCOPLASMIC_RETICULUM_CALCIUM_ION_TRANSPORT The directed movement of calcium ions (Ca2+) into, out of or within the sarcoplasmic reticulum. NOL3 FKBP1B CACNG1 RYR2 ANK2 RYR1 TRDN ATP2A2 CCL3 SLN CCR5

GO_METANEPHRIC_MESENCHYME_DEVELOPMENT The biological process whose specific outcome is the progression of a metanephric mesenchyme from an initial condition to its mature state. This process begins with the formation of metanephric mesenchyme and ends with the mature structure. Metanephric mesenchyme is the tissue made up of loosely connected mesenchymal cells in the metanephros. SIX4 TCF21 BMP7 PDGFRB SHH STAT1 WNT4 SMAD4 WT1 PAX2 PKD2 SIX1 BASP1 OSR1

GO_SOMATIC_STEM_CELL_DIVISION The self-renewing division of a somatic stem cell, a stem cell that can give rise to cell types of the body other than those of the germ-line. WNT3A FGFR1 LEF1 WNT7A HOXB4 ZFP36L2 NUMB TEAD3 RAB10 DOCK7 DCT FGF13 FZD7 NOTCH1 ASPM NUMBL SOX5 TGFB2 KIT FGFR2 VANGL2

GO_POSITIVE_REGULATION_OF_VASCULAR_ENDOTHELIAL_GROWTH_FACTOR_PRODUCTION Any process that increases or activates the frequency, rate, or extent of production of vascular endothelial growth factor. GATA4 BRCA1 IL1B CCBE1 EIF2AK3 C3AR1 HIF1A RORA TGFB1 ISL1 ATF4 IL1A NOX1 SULF2 C5 C3 ADORA2B FLT4 CYP1B1 C5AR1 NODAL PTGS2 IL6ST HPSE SULF1 ARNT

GO_DEFENSE_RESPONSE_TO_FUNGUS Reactions triggered in response to the presence of a fungus that act to protect the cell or organism. DCD C10orf99 LTF ELANE IL17A TGFB1 CHGA HTN1 S100A12 HAMP ADM S100A8 IL17RA DEFA5 HTN3 SPON2 CTSG HRG MPO CLEC6A DEFA1B DEFA3 DEFA1 APP S100A9 DEFA6 GNLY ANG COTL1 IL17RC NLRP10 DEFA4 JAGN1 IL36RN

GO_REGULATION_OF_GRANULOCYTE_CHEMOTAXIS Any process that modulates the rate, frequency or extent of granulocyte chemotaxis. Granulocyte chemotaxis is the movement of a granulocyte in response to an external stimulus. CCL21 CCR7 GPR77 CSF1 CAMK1D DAPK2 THBS1 CXCR2 S100A14 RARRES2 CCL5 XCL1 CXCL3 S100A7 RAC2 CMKLR1 JAM3 IL23A C1QBP TIRAP NCKAP1L IL4 MPP1 CCL2 C5AR1 TRPV4 RAC1 CCL19 SLIT2 IL8 CD74 THBS4 LBP TNFSF18 C3AR1 CXCL2 CXCL1

GO_PURINE_CONTAINING_COMPOUND_SALVAGE Any process that generates a purine-containing compound, any nucleobase, nucleoside, nucleotide or nucleic acid that contains a purine base, from derivatives of them without de novo synthesis. CECR1 GMPR ADA ADK APRT AMPD2 AMPD1 PNP DCK DGUOK MTAP PRTFDC1 AMPD3 ADAL GMPR2 HPRT1

GO_ENTRY_INTO_HOST Penetration by an organism into the body, tissues, or cells of the host organism. The host is defined as the larger of the organisms involved in a symbiotic interaction. ITGB6 F11R TFRC GAS6 SLC20A2 NCAM1 EPS15 CLEC5A ITGB1 NUP153 HSPA1A CR1 MRC1 CD4 CD80 CAV1 ZNF639 LAMP1 SCARB1 XPR1 CLDN1 PVRL1 IDE CLEC4G ICAM1 UVRAG CD86 SLC10A1 ITGAV CD81 WWP2 PVRL2 CDHR3 GYPA CXCR4 ITGB7 EFNB3 HSPA1B DAG1 HTR2A MOG SLAMF1 RPSA TNFRSF4 HAVCR1 ITGA2 TYRO3 CCR5 KPNA3 VAMP8 ITGB5 HSBP1L1 HYAL2 NPC1 LAMP3 SCARB2 ITCH WWP1 SELPLG PVR ACE2 PVRL4 CAV2 CLEC4M TNFRSF14 PPIA GPR172A DPP4 SLC1A5 CTSB ITGB3 EFNB2 CD46 SIVA1 CR2 ITGA5 VPS18 CXADR ANPEP CD209 AXL CD55 DYNLT1 GPR172B LDLR ADRBK1 SERPINB3

GO_NEUROTRANSMITTER_TRANSPORT The directed movement of a neurotransmitter into, out of or within a cell, or between cells, by means of some agent such as a transporter or pore. Neurotransmitters are any chemical substance that is capable of transmitting (or inhibiting the transmission of) a nerve impulse from a neuron to another cell. STX1B SYT3 SNAPIN SLC6A6 SYTL2 SYT8 SLC6A4 SCRIB SLC6A11 LIN7A SLC6A17 SYN3 RIMS1 GABRQ STX11 GRM4 SYN1 PPFIA4 LIN7B SLC17A7 SV2A STX1A PPFIA2 SYT13 SNAP47 KCNJ10 SLC18A2 CASK OTOF CADPS SYTL3 CACNA1A UNC13B APBA1 NRXN2 UNC13A SLC6A1 PIP5K1C SYT6 SYT17 SLC6A20 HSPA8 STX2 BZRAP1 SYT1 RIMS4 STX19 SLC18A3 NAAA TH SEPT5 RAB3A CADPS2 SLC5A7 C2CD4D STX3 SLC1A3 CACNA1B SLC6A15 SLC6A5 CPLX2 RIMS2 PTPRN2 TC2N LIN7C SLC6A7 RPH3AL SYNJ1 PARK2 DGKI PPT1 DNAJC5 SYT7 CHAT SNPH SLC6A9 SYT16 NRXN3 P2RX7 SLC17A8 PDZD11 C2CD4B SLC38A1 SYT11 NLGN1 GABRA2 PLDN SLC6A16 NRXN1 PPFIA1 STXBP1 SYTL1 SLC6A12 SNCA SLC22A2 SLC32A1 C2CD4A PSEN1 CPLX4 CPLX1 GAD1 STX4 SLC6A13 DVL1 CDK5 SYT2 SLC6A19 SYN2 VAMP2 SYT14L PCLO DDC SYTL4 SLC18A1 RIMS3 DOC2B C2CD4C SLC6A18 SYTL5 SLC6A3 GAD2 NF1 SLC6A2 SNAP29 SLC6A8 SLC6A14 CPLX3 ATP1A2 SV2B NANOGNB SYT10 GLUL SYT14 SLC22A1 SYT15 PARK7 WNT7A SNAP23 PPFIA3 TRIM9 RPH3A SYT9 HRH3 DOC2A ICA1 SYT4 SLC22A3 SYT12 BRSK1 SYT5 SV2C SNAP25 LPHN1 BAIAP3 SLC17A6

GO_PARAXIAL_MESODERM_DEVELOPMENT The process whose specific outcome is the progression of the paraxial mesoderm over time, from its formation to the mature structure. The paraxial mesoderm is the mesoderm located bilaterally adjacent to the notochord and neural tube. SMAD3 TEAD2 BMPR1A DLL3 FOXC1 FOXC2 SMAD2 EPB41L5 WNT5A WNT11 POGLUT1 NUP133 LEF1 FGFR1 WNT3A TCF15 YAP1 EXOC4

GO_REGULATION_OF_PROTEIN_KINASE_B_SIGNALING Any process that modulates the frequency, rate or extent of protein kinase B signaling, a series of reactions mediated by the intracellular serine/threonine kinase protein kinase B. CSF3 CCR7 HYAL2 PRKCDBP EGFR INS DRD3 TSC2 PTPRJ AKR1C3 TNF XDH SRC OSBPL8 INPP5K GPER CRYBA1 MTDH PDGFA NTRK2 C1QBP IGFBP5 F3 STOX1 ANGPT1 RRAS ZP3 PTEN SFRP5 FLCN GPX1 GATA3 STK3 MAGI2 CHI3L1 PHLPP1 MEIS3 CAV3 TSPYL5 AXL KLF4 GNB2L1 DDIT3 PKHD1 STK11 MTM1 RNF41 SPRY2 PARK7 TNFAIP8L3 SEMA5A F7 BANK1 PIK3CG TCF7L2 SIRT1 TNFSF11 EPHA2 TXN IL26 PHLDA3 CCL21 MC1R PIK3R5 SLC9A3R1 CPNE1 DRD2 CD28 AKR1C2 LEP MTOR CCL3 ITSN1 THPO MST1R THBS1 PINK1 F10 PTK2 HPSE C1QTNF1 HBEGF NOX4 C19orf10 NRG1 GAS6 RAPGEF1 PLEKHA1 MSTN IL18 ARFGEF1 FAM110C GCNT2 CIB1 IL6 SESN3 ILK MYOC IGF2 INSR ARRB2 HAX1 RICTOR PDCD6 RASD2 LMBRD1 CCL19 BAG4 DAG1 HCLS1 ADAM8 LEMD2 KIAA1161 ITGB1BP1 FAM132A INPP5F TEK TGFBR1 DLG1 MUL1 TGFB1

GO_REGULATION_OF_ENDOTHELIAL_CELL_DEVELOPMENT Any process that modulates the frequency, rate or extent of endothelial cell development. IKBKB CLDN5 ROCK2 CDH5 TNF PROC S1PR2 S1PR3 PPP1R12A ROCK1 IL1B TNFRSF1A

GO_ENDOCARDIAL_CUSHION_FORMATION The developmental process pertaining to the initial formation of an endocardial cushion. The endocardial cushion is a specialized region of mesenchymal cells that will give rise to the heart septa and valves. FGF8 SNAI2 BMP7 MSX1 NOTCH1 ENG SNAI1 SMAD4 BMP4 MSX2 BMPR1A TBX20 HEYL DCHS1 RBPJ TMEM100 BMP5 HEY1

GO_PURINE_NUCLEOSIDE_MONOPHOSPHATE_BIOSYNTHETIC_PROCESS The chemical reactions and pathways resulting in the formation of purine nucleoside monophosphate, a compound consisting of a purine base linked to a ribose or deoxyribose sugar esterified with phosphate on the sugar. ATP5J ATP5H STOML2 LDHC PRKAG2 TGFB1 ATP5A1 ATP5I ADA AMPD1 APRT ATP6V0A4 ATP5S COX5B GBAS ATP5D IMPDH2 ATIC HPRT1 LHPP ATP5F1 PFAS ADSS AK4 ADSL ATP5J2 PAICS ATP5L ATP5L2 AK2 ATP6V0A2 ATP6V0A1 GART ATP5C1 AK1 C11orf83 PRPS1 GMPS ATP5G2 SLC25A13 SURF1 PPAT ATP5O AMPD2 C16orf7 AK5 ATP5EP2 ADK PKLR IMPDH1 AKD1 TCIRG1 ATP5G1 CHCHD10 AK3 ATP5E PRTFDC1 ATP5B ALDOA ADSSL1 AMPD3 ATP5G3

GO_RESPONSE_TO_PLATELET_DERIVED_GROWTH_FACTOR Any process that results in a change in state or activity of a cell or an organism (in terms of movement, secretion, enzyme production, gene expression, etc.) as a result of a platelet-derived growth factor stimulus. YES1 HAS2 RDX CBL HAS1 FYN HYAL1 ERRFI1 SRC CCNA2 PDGFRB CORO1B IQGAP1 CCL2 PTN PDGFD CREB1 FER

GO_REGULATION_OF_CELL_CYCLE_G2_M_PHASE_TRANSITION Any process that modulates the frequency, rate or extent of cell cycle G2/M phase transition. CCND1 CENPF NPM1 TP53 PBX1 ZNF830 FOXO4 GPR132 RAD51B H2AFY WNT10B RAD51C RCC2 CDC7 SIN3A CDK1 PKIA VPS4B HMGA2 ATM SMARCD3 BLM FHL1 NEK10 CDK5RAP3 HSPA2 STOX1 CLSPN USP47 PHOX2B MRE11A APP CCNA2 TOPBP1 TAOK3 PAXIP1 ATF5 MIIP GTSE1 RAB11A KCNH5 HUS1B RNASEH2B PLK1 ANKRD32 SYF2 HUS1 CAMK2D CDK4 FANCI KIF14 DBF4B FOXN3 CTC1 RINT1 NAE1 CCNB3 NBN C15orf42 BRD4

GO_NEGATIVE_REGULATION_OF_TYPE_2_IMMUNE_RESPONSE Any process that stops, prevents, or reduces the frequency, rate, or extent of a type 2 immune response. SOCS5 ANXA1 STAT6 IFNA2 IFNB1 IL27RA CCR2 IL29 BCL6 HLX NDFIP1

GO_NEUROGENESIS Generation of cells within the nervous system. CDC20 RAPGEF2 LRFN2 SEMA4A SMARCE1 HDAC1 RAP1A PRDM6 TGFB2 ZFHX3 LZTS1 SMO NR4A3 CHN1 FAIM2 SPTBN4 FERD3L PDLIM5 LYN TRAPPC4 SEMA7A FLRT3 SOX14 KLF7 ARHGEF10 EFNA4 EFNA1 HHIP FOXA2 NKX6-1 HIPK2 TRAPPC9 MEF2A KIT ATXN10 HOXB3 CABP4 NEGR1 DYNC2H1 B2M IFRD1 CDC42 MBD1 PPP1CC NCAM1 HES3 PBX3 ANK3 ISPD LMX1B ALS2 ALDH1A2 CNGB1 MFRP GATA3 FGFR2 PRDM8 EPHA4 RAB21 NKD1 SATB2 PRKCI ERBB3 RTN4R CDON CDKL5 DGUOK RAPH1 VEGFA CIT ITGA3 HSP90AA1 DICER1 NPTN MEIS1 PHOX2A PEX13 EPHB2 EFHD1 SORL1 BTG2 LMO4 NTRK2 ASAP1 DFNA5 FARP2 WDPCP EVX1 TDP2 LPPR5 SLC39A12 HDAC9 ETV5 CCL2 GOLGA4 NDEL1 EFNB3 ADM SYT17 USP9X FOXB1 FBXO45 KCNJ10 MMP24 EXT1 HAND2 MDM2 COPS2 KLF15 MYCN ISL1 FGF13 NME2 SEMA6D CDK5RAP1 SALL1 DCDC2 UNCX CYFIP1 CTNNB1 DRD2 PLAG1 P2RY12 RARA WDR1 UCN FARP1 PTK2 SLITRK4 VLDLR ITGA1 GPC2 SCRIB SCLT1 PLXNB1 WNT16 KIF13B UHMK1 PER2 SNX3 RUFY3 CDH11 OLFM1 ALK CRTC1 IQGAP1 ROBO1 NEUROG2 MAP2 NLGN1 DNM3 PIN1 POSTN DLL3 C2orf71 NEFH CLIP1 SIPA1L1 SCARF1 AHI1 CCDC66 AREG GPR183 NR2F2 LRP8 DPYSL2 ATP7A NEO1 CNTN1 MATN2 OPRM1 GDF7 HES1 FOXA1 SRSF1 CD9 LRFN5 NLGN3 RIMS2 IER2 ULK2 NIF3L1 OGDH OLIG1 GNGT1 RAC3 UBA6 CHRNA3 PPP3CA C3orf17 KNDC1 SYT1 HDAC2 CDH4 DUOXA1 SSH2 CXCR4 TRPV4 CEP120 ZNF280C CDK5RAP2 CCDC64 SYT14L SIX1 CDK16 C16orf45 MAP4 COBL WDR36 INPP5F CTTN PAX7 LIG4 GSTP1 SOD1 WNT7B FEZF2 CPNE1 RAP1GAP FEV NPHP4 CDK5 INPP5J NRXN1 DSCAM TWIST1 SPG11 BTG4 RTN4RL2 LRP1 LRRK2 NGRN GRXCR1 POU4F2 EIF2B2 NCDN OMG SIAH1 WDR5 MED12 VAMP7 NTRK3 EFNB1 SGK1 CNTF GRIP1 MYCL1 PROX2 MAPK6 CXCL12 LRFN4 PCM1 SDK1 EVL CNR1 PPP2R5B PLXNC1 OTX2 MYO7A RND1 EHD1 NDNF IMPACT FGF20 GLI3 NGFR DOCK7 IRX3 MUTED PRKD1 SEMA6B NRBP2 INHBA NTN3 OTP PRKCQ ARHGEF1 GCM1 GPR173 VIM SCN1B OPA1 CARM1 PAK3 EN2 ETV6 SEMA6A STMN4 KAL1 DRGX CSK ZNF280B RAB13 PTCH1 NPY TUBB3 LRFN1 JAM3 SKI SPTBN5 ERCC3 GDF6 DBN1 EIF2B4 HERC1 ATF5 SECISBP2 ILK ECM2 SPTB DGKG SNPH PTPRD TBX20 ATP8B1 PITX3 PAFAH1B1 B3GNT1 DPYSL3 SPTA1 LPAR1 NDRG1 SOX8 HDAC5 LRRC4C GDI1 VAX1 DDX6 RASAL1 ARTN NEUROD2 FAT4 CDK5R1 GPR56 IFT140 KRAS PLXNA2 CCR2 NDN SLC4A7 FAM150B THY1 ECT2 NUMB EIF2B3 PRDM16 P2RY1 SHANK1 FBXO38 DPYSL5 RGMA LHX9 SNW1 SLIT1 CASZ1 SH3TC2 NFASC BLOC1S2 LRFN3 UCHL1 ATP8A2 KDM4A RACGAP1 WDR81 CNP HOXC8 PRPF19 GDNF ANAPC2 DLX2 DVL1 ASPM LAMC3 MAPK1 PSEN1 DAB2IP AFG3L2 BAX STAT3 EPHA7 SLC4A10 NRAS TBC1D24 TRPM1 SLC45A3 CTDSP1 ASCL2 LIMK1 MAPK8IP3 KIDINS220 PTPRO PLDN BMPR1B CAMK2A CIB1 ROGDI STXBP1 BEND6 SEMA3F CHD5 BOK UFL1 ADRA2B HIPK1 KIF20B HGF CELSR3 WNT3A SEPT2 AGER DISC1 LINGO1 SDCCAG8 DDX56 WNT2B BCHE AKT1 FZD7 EPHA2 PRDM12 TWF2 EGFR PARD3 NEFL NTM USP21 STMN2 IFT172 BAI1 GNAO1 CPEB3 RORB SEMA6C LRP6 RUNX1 PICK1 PTPRK TRPC6 F2 PPP1R9B FA2H SIX4 CTNND2 CFL1 NOG KIF3A MBOAT7 PENK LINGO2 FGF5 HPRT1 RAC1 ANKS1A TULP3 ZSWIM6 LTA OMP KLK8 PRDM1 WEE1 FSTL4 SRGAP2 CBFA2T2 DTNBP1 SEMA3G KEL NEUROG1 CAMK1D C1orf187 BLOC1S3 ATCAY ROM1 SOX2 KCNA1 SOX10 LMX1A NDRG4 RYK CNTN2 CRB1 GJA1 EDNRB RHEB MKL1 NRG1 NME1 RNF10 CSNK1E LAMB1 TRIM67 GALR2 CDKN1C EEF2K ENC1 GABRB1 SGK196 MAP2K1 MYOC PLXND1 IL6 NRXN3 BHLHB9 S100A8 HEYL GRB2 MAPK8IP2 NRN1L TLR4 KALRN HMG20B NR2F6 TAL1 ERBB4 NRCAM LRRC55 ATAT1 STMN3 BCL2 LIN28A ISLR2 CSF1 NCOA1 CHL1 WNT9B PTCHD2 ONECUT2 SFRP1 EPHB1 SLC1A3 EOMES IL2 NKX6-2 LRTOMT STMN1 TH PLXNA3 TBCE ANXA1 VASP BCL11B TTL RAB8A DIXDC1 SEMA4D DLX5 ERBB2 ATP2B2 ETV4 SEMA4F NCK1 SS18L1 PREX1 APBB2 BAIAP2 RAP2A RIT2 EZH2 CBLN1 RPE65 SLITRK3 KLK6 PAK4 CRTAC1 EZR LINGO3 CUX2 OPHN1 SOX1 ARHGAP4 RPL24 NRP1 SETX HOXD3 ULK1 LEP KLHL1 CDK6 SYT2 SLITRK6 DUSP10 LPHN3 KIF17 RND2 RAPGEF1 FEZ1 WNT8A POU3F2 VWC2L C5orf13 NKX2-5 PTPRA PAQR3 FSCN2 GRIN1 NCK2 CLU RRN3 HEY2 PRMT1 AXL TCF4 CTNNA2 HRAS SNAP25 MPP5 LHX2 VANGL2 SEMA4G STK11 WNT7A HOXA2 BBS4 L1CAM GSK3B PROM1 SMARCC2 YWHAE NAPA IST1 TUBB2B DTX1 FOLR1 RAB11A CALR TNIK ZNF521 MXRA8 HDAC6 SPEN NFATC4 PAK2 LLGL1 GDF5 POU4F3 VWC2 FN1 KIF5B DAG1 DCLK1 IFNG CSNK1D LLPH NAV1 MT3 PDE6C WNT9A TLE6 HDGFRP3 CAPRIN2 SPTAN1 CACNA1A NGEF ALCAM LHX3 FLRT1 RNF6 XRCC5 GABRB3 CCL3 SLITRK5 OBSL1 C17orf96 FZD5 ZNF536 HTRA2 SNAPIN YAP1 WNT4 HOXD1 NCAM2 TNFRSF21 FOXO6 IGSF9 RELN WNT5A DAB1 NR4A2 SPAST LBX1 PLP1 UNC5B SSH1 MYH10 NCKIPSD JAG1 YWHAG PLXNA1 FMN1 DYX1C1 PALM FKBP4 NTN1 ODZ3 APOE ENAH MCOLN3 KIRREL3 CLMN SERPINE2 BARHL1 ZC4H2 HOOK3 CCKAR WNK1 IL6ST FZD3 OPCML MDGA1 LHX8 FIG4 GLI2 HOXC10 APOA5 SIRT2 RET RSPO2 CEBPB EPYC VEGFC RHOA MICALL2 WNT1 LST1 SOX5 IL1RAP RNF165 DCHS1 DFNB31 NPTX1 GSX1 SCRT1 TNFRSF12A POU4F1 LRRC38 SZT2 ZNF217 SLITRK2 CASP3 DRD1 HMGB2 EPB41L3 LDB1 VCL ABI1 CNTN6 UNC5D PTPRZ1 ARFGEF1 SKOR2 MEGF8 GDPD5 LINGO4 CPNE5 SPTBN2 SEMA4B PRKCH LGALS1 PPAP2B WNT3 DKK1 ZNF280D REST KIAA1598 EEF2 C1orf96 RP1L1 EIF2B5 NKX2-8 CNO LOC100507050 NEFM POU3F4 SUFU SEMA5A LRRN1 SDC4 CPNE9 S100A6 RNF112 ERCC2 DAPK3 GSX2 NR2F1 POU3F1 ETV1 SHH NELF SRGAP2P1 RP1 SMURF1 SEMA3D NTF3 WASF3 GRID2 BMP2 BRAF PLXNB3 CLCF1 CAPRIN1 RANBP9 ITM2C SHOX2 MAP1S UNC13A FABP7 DBNL CHRNB2 UNK SEMA3A TBX6 MCF2 PTPRG CEND1 SIAH2 FZD9 GIGYF2 RIMS1 SPG20 PRMT5 SRF ABL2 GORASP1 SOX11 ADNP2 TSPAN2 RAP1GAP2 ITGB1 PLK2 C12orf52 ARF4 EMX2 ODZ1 EFNA3 EN1 ID1 DMRT3 SDC2 CHD7 ZNF335 NEDD4L CPNE6 BCL6 TMEM106B GRIN2A GPM6A RTN4RL1 NKX2-2 GNAQ MAGI2 DCX KDM1A ARX SLIT2 FGFR1 OLIG2 METRN PICALM TEAD3 PTK6 STRN THOC2 LAMB2 HMG20A NTNG1 NTRK1 GAK C3orf39 PEX7 MTR NFIB TRPC5 LEF1 FAM5C LRRC70 MACF1 SYNGAP1 FLOT1 GPER GFI1 KIAA0319 S100B CLIC5 APOA1 SMARCA1 PTPRM SF3A2 VAPA SLIT3 BIN1 UNC5A PTPRF NFE2L2 MAP1B OMD DPYSL4 NTF4 CDK1 COL25A1 MED1 EPHA5 NTN4 PBX1 ADCY1 GRIN3A PPARG JUN TULP1 RERE DCC XBP1 FZD4 BECN1 TMC1 NGF MAPK3 PRKG1 VHLL GABRA5 CLN5 NAGLU FOXD1 EFNA5 LRP4 MINK1 SMARCD3 MT1X CAMSAP2 CDH23 BDNF KANK1 CREB1 HDAC11 NOS1 SPINK5 ATF1 TRIM32 EFNA2 HS6ST1 UBB DAGLA PHGDH PPP3CB SYT4 BMPR2 BRSK2 RB1 EFNB2 RASGRF1 CELSR1 COL3A1 FAM5B S100A9 RBFOX2 TSKU GFAP GPRIN1 UGT8 WNT8B CSPG4 NAP1L2 C7orf51 NOTCH3 WDR62 SOS1 VSX1 DCT FBXW8 NAB2 SEZ6 TIMP2 DLG4 OR8A1 MMP14 ISL2 C10orf125 CDNF PARD6B ELL3 PRDM13 FZD1 CRX ADAM22 APBB1 CCK CERS2 TRIP11 LPAR3 ELP3 MANF MUL1 ROBO2 PAK1 LHX6 EIF2B1 ID3 NLGN4X ABT1 FRMD7 C1QL1 EIF4ENIF1 IL33 SYT3 GAS6 EIF4G1 STX1B TMEM30A FES MMD ZNF488 RAB10 RORA PHOX2B NRG3 CUL4B CDH2 PTN MIB1 AGRN AKT2 SEMA3B CHODL ARF6 STK24 SOX9 SPTBN1 HOXD10 HMGB1 PTK2B C1orf130 CAMK2B ID4 USH2A ADNP APOD PRKCZ SKOR1 TNN JAK2 RDH13 FOXN4 ARHGEF2 SPP1 NEURL ABL1 SLC12A5 FGF10 CCDC88A SOCS7 MTCH1 FIGF BMPR1A GBX1 GBA2 SLC8A3 PRRX1 KIF5C SLC9A6 SEMA4C SHANK3 FZD8 SEMA5B NANOS1 LPPR4 NDE1 LIF NUP133 MICALL1 GBX2 CEP290 AMIGO1 PIGT SRCIN1 PREX2 LAMA2 WNT11 LGI1 INSM1 DYNLL2 RTN4 LHFPL5 TGFBR1 CAMSAP3 CUX1 TGFB1 TBR1 OGN TCF3 SLC9A3R1 MNX1 HDAC10 SPOCK1 OLFM3 RAB17 RPGRIP1 NOM1 ADRA2C LSM1 WNT5B EGR2 SALL3 PQBP1 CTNNA1 DLL1 EPHA3 PCDH12 STRC GHRL NEUROD1 EYA1 VAX2 KIF5A BARHL2 DLL4 CUL7 SMAD4 MARK1 GLDN SDK2 DNMT3A TRPC4 KCTD11 CNTNAP1 FOXO3 FKBP1B PAX6 MESP1 NF2 SCRT2 FEZ2 SPAG9 TOPORS TP73 PAPD4 HCN1 IFT27 NEK3 RARB LLGL2 PDZD7 LRTM2 S1PR5 LGI4 ARHGDIA MMD2 UBE4B NF1 CAMSAP1 IFT20 SOX6 PITX2 ARF1 BMP7 FMOD CAMK1 PTF1A WNT10A GAP43 BMP4 ANKRD1 NNAT CTF1 DNM2 ZHX2 TLR9 SERPINF1 EPHB3 MDGA2 BOC CDKN2C SEMA3C CRABP2 HOXD9 GCM2 SARM1 KIF26A B3GNT2 ARL3 CELSR2 EIF2AK4 PACSIN1 XK TOP2B ARHGAP35 ZNHIT2 GPRC5B FAM134C CHAC1 XRN2 PTPN11 C11orf9 PVRL1 MTOR WNT2 TRIM11 ARNTL SPINT1 RAB35 ODZ4 NR2E3 GFRA3 SOX3 IRX5 ZMYND8 APOA4 PMP22 NEDD4 GNAT2 NBL1 PARK2 MKS1 ADCYAP1 CRMP1 MAN2A1 KCNIP2 PPT1 NEUROG3 TSPO BTBD3 VTN DCLK2 DNER SOX4 FZD2 CX3CR1 STX3 ITPKA USP33 HELT FYN UQCRQ SKIL FRY HIF1A HES5 ITSN2 YWHAH RAB3A NOTCH1 S1PR1 EML1 DHFRP1 ZFYVE27 NEUROD4 DHFR SLITRK1 CDK5RAP3 BHLHE22 CCR4 BAI3 STK25 CDK5R2 ASCL1 FGF8 CNTN4 RAB7L1 ZNF259 LHX5 NAB1 MOB2 NUMBL CSF1R MTMR2 KATNB1 RBPJ DBX1 LTK ALKBH1 GPR37L1 DSCAML1 ID2 FEZF1 GABRB2 SEMA3E FMR1 CYB5D2 SPON2 ANKRD27 GNAT1 NRP2 AXIN1 EIF4E PLA2G10 HAP1 TIAM1 CTHRC1 MARK2 BRSK1 AVIL MTPN PRELP TNC EDN3 KLF4 MYEF2 UBE2V2 LUM UST GBA TGIF2 MEF2C FRYL NCS1 CDH1 RELA ARSB GATA2 APCDD1 FAM150A DRD3 MAPT IL1RAPL1 WNT6 DLX1 RGS14 SPRY3 LHX4 DAGLB STAR FLRT2 NKX2-1 FZD10 JAG2 BMP6 BTBD6 RUNX2 TCTN1 SLC11A2 SSH3 LRRN2 WNT10B LEMD2 PLXNA4 DNMT3B KIAA1486 TERT UNC5C NRL LHX1 KERA ULK4 ATL1 PROX1 MECP2 HEXB TCF12 EPO BCL6B BLOC1S1 SLC6A4 FBXO7 APP RTN4IP1 ASTN1 ATOH1 GP5 SIX3 PTEN EMX1 TOR1A MAP6 DYNLT1 BMP5 TLR2 NR2E1 PLXNB2 EPHA8 PLK5 STAP1 DDIT4 ROBO3 RPS6KA5 TLX2 SRRT ZNF280A RUNX3 DMRTA2 PTK7 GNRH1 SFRP2 NRTN XRCC2 BAG5 FRS2 USH1G CNTNAP2 ZEB1 DBC1 TRPV2 TNR TLX3 MAG GPC1 ADCY6 PAX2 ACSL4 SEC24B OLIG3 AGTPBP1 KIF26B AZU1 NTNG2 PPP1R9A AURKA PCSK9

GO_TRANSMEMBRANE_RECEPTOR_PROTEIN_SERINE_THREONINE_KINASE_SIGNALING_PATHWAY A series of molecular signals initiated by the binding of an extracellular ligand to a receptor on the surface of the target cell where the receptor possesses serine/threonine kinase activity, and ending with regulation of a downstream cellular process, e.g. transcription. GDF2 BMP15 COL3A1 ADAM9 BMP10 PARD3 INHBC AKAP4 ACVR2B MAPK14 PARP1 AMHR2 FURIN ARHGEF18 BMP8B SRC SMURF1 MTMR4 PTPRK TGFB1I1 T CHRDL1 GDF9 ZNF8 BMP2 BMP7 RBM14 FMOD BMP3 BTBD11 SMAD4 FNTA GDF11 BMP8A TMEM100 MAP3K7 CREB1 ACVR2A LTBP1 TGIF2 BMP1 LEFTY2 FAM83G SMAD1 UBC UBB ZFYVE9 BMPR2 UBE2D1 SMURF2 IRAK1 SMAD2 FOXH1 USP15 HIPK2 TOB1 INHBE FOS SMAD3 NLK LEFTY1 SLC33A1 EGR1 CGN MAPK3 ACVRL1 ATOH8 WFIKKN2 MEGF8 BMPR1B MYH6 NKX2-5 FSHB LRP4 RNF111 APOA1 DLX5 WNT1 RGMA CFC1 ZCCHC12 TGFB2 NODAL GDF1 ACVR1C INHBB FGF8 AKAP2 CBL AFP DUSP15 RGMB EID2 GREM2 MSX1 TGFB1 JUN BAMBI LTBP2 TGFBR1 ETV2 GDF7 TGFBR3 MSX2 DSG4 BMPR1A HPGD UBE2M SLC39A5 LNPEP TGFB3 GDF3 SKIL LEF1 GDF15 STUB1 UBA52 ITGB5 PARD6A FERMT2 RHOA TGFBRAP1 PDGFB FUT8 COL1A2 ACVR1 SUB1 PPM1L LOC100506013 MAGI2 SPTBN1 UBE2D3 USP9Y BMP5 SCXB PRKCZ FKBP1A PML DUSP22 CITED1 SMAD9 LTBP4 SMAD5 HNF4A ROR2 KLF10 HFE CHRD ACVR1B PTK2 HFE2 SMAD7 MSTN F11R ZFYVE16 GCNT2 SKI ITGB1 TGFBR2 GDF6 TWSG1 TAB1 ID1 NOG RPS27A ENG GDF5 INHBA CITED2 BMP6 CCL2 ARRB2 BMP4 RUNX2 NEDD8 NUP93 AKAP3 CDH5 CLDN5 USP9X ZYX ZCCHC18 RYR2 VIM SMAD6 FSTL1 PXN INHA GDF10 CER1

GO_REGULATION_OF_EXCRETION Any process that modulates the frequency, rate, or extent of excretion, the elimination by an organism of the waste products that arise as a result of metabolic activity. COMT DRD2 AVPR1A STC1 UTS2R SLC9A3R1 DRD3 NPR1 NPPB AGTR2 C12orf39 AGTR1 UTS2 TAC1 AGT AVPR2 EDN1 EDNRB NPHS1 CORIN ADORA1 OXT AVP

GO_ORGANELLE_FISSION The creation of two or more organelles by division of one organelle. CHMP3 WEE1 CLASP2 AKAP8 STAG1 CENPF CHMP2B KIF22 MAD1L1 PAPD5 KLHL22 CDCA8 MFF CNTD1 SYCP1 DCTN3 ACTR3 DNM2 CCNB2 OIP5 CDK11B BOD1P C15orf43 GOLGA2 MCMBP SEH1L KATNA1 CTDP1 FBXW5 MIS18BP1 TOP2B CCNA1 PKMYT1 CHMP4C MAPRE1 CDCA5 SMC2 PEX19 SYCE2 SLC2A8 PARK2 CCNG2 PLD6 PRC1 CDCA2 TRIOBP FIS1 HAUS5 CLTCL1 GEM DMC1 HMGA2 VPS4B ANLN CHEK2 HSPA2 ASZ1 FBXO5 SPIRE1 HEPACAM2 ACTR8 SNX33 WEE2 E4F1 HAUS1 UBE2I LZTS2 ZC3HC1 HAUS3 FIGN BRCA2 CHMP7 RAN ESPL1 CKAP5 UBE2B INCENP RAD51C RACGAP1 CDC27 ERCC1 SIRT7 ANAPC2 MND1 FBXL7 HAUS6 DCLRE1A CENPW SLX4 CENPN PLK1 ACOT8 SPATA22 LATS1 SPC25 SMC5 EIF2C4 TAF1L ASPM PTTG1 WASL ARPP19 CHMP4B KIF3B CDC14A LEPREL4 MAD2L1 KIAA0196 ANAPC16 HAUS4 SEPT2 SPAG5 C2orf65 RBBP8 AURKB SKA2 TIPIN CSNK1A1 CHFR KLHL13 SETDB2 XIAP USP16 STAG3 KIF20B HGF SNX18 C17orf104 DUSP13 KIAA0430 MYBL2 AURKC CHMP4A TOP3A CENPT OFD1 CDK3 HORMAD1 STOX1 EPS8 VRK1 PBRM1 KNTC1 ZFP42 TUBGCP6 BABAM1 DCTN1 TOP2A NEK3 BOLL REEP3 RCC2 CEP63 DYNC1LI1 TTN SPIRE2 ATM FKBP6 NCAPH BIRC5 ANAPC13 REC8 TEX12 MKI67 SYCE3 CCNG1 BIRC8 NCAPD2 FLNA BUB3 SMARCA4 MAP9 CHMP6 NSL1 CUL3 PDS5A TPR CEP164 NUDC PLK5 UBR2 RUVBL1 MIS12 DYNLT3 FAM83D CETN2 SPAST CCNB1IP1 KIFC1 RAD51D DYNLT1 CCDC155 HAUS7 C11orf20 PTTG3P RPS3 SIRT2 CDC25C CDC16 ARL8A RHOA AURKA ANAPC7 TPX2 BIRC2 NEK4 MTFR1 DNM1L ANKLE2 NSUN2 XRCC2 KIF4B C11orf85 KIF2C CDC6 KIF2B RAD51 ING2 TUBGCP5 NCAPG2 SYCE1L DSCC1 GDAP1 TEX11 XRCC3 NUP62 TUBGCP3 SKA3 RPL24 DMRTC2 KATNB1 COX10 ANAPC4 CHMP1A WRAP73 ZNF830 NAA50 BIRC6 TNKS CORT CYP26B1 SRSF2 PDS5B LOC728637 MOV10L1 KLHL9 RGS14 RAB11A PEX11A CEP55 PPP1R12A TDRD9 NEDD1 EME2 C16orf73 ZNF268 CEP57 NSMCE2 MAPRE2 CCNF CDC25B CCDC79 HFM1 SGOL2 USP44 RAD54B YEATS4 ZWILCH TMEM48 PSMD13 FANCM KIF2A UBE2S SAC3D1 NDEL1 BOD1 NEK1 C11orf80 KLHDC5 RAD21L1 SPICE1 BUB1 USP9X STRA8 POGZ KIF23 TDRKH RECQL5 NUP43 SPANXA2-OT1 GGNBP1 SPO11 CHMP2A MSH4 KIF11 ERCC6L RAD51B GSG2 TEX14 MARK4 PSRC1 DNM3 KPNB1 CLIP1 RAD50 EREG CDCA3 DNM1P46 SEPT7 CHAMP1 MZT1 MSH2 TUBGCP2 TUBGCP4 ANAPC11 BRE APITD1 SGOL1 PRDM9 SNX9 TIMELESS MLH3 PDCD6IP MSH6 CCNB1 ARHGEF10 WAPAL NUSAP1 CDC20 ZWINT RNF8 MSH5 BUB1B PPP2R2D SYCP3 NEK6 RNF212 SSSCA1 PEX11B CLTC BAG6 REEP4 FAM175B TXNL4A TACC3 SPDYA HELLS NUF2 C19orf21 RRS1 VPS4A NLRC4 MSH3 DIS3L2 FAM64A CIT C11orf51 DSN1 DLGAP5 AKAP8L HSF1 UBE2C KLHDC3 BORA ANAPC1 C1orf96 MAD2L2 KLHL21 C12orf11 KIF18A CDK11A FAM54B TDRD12 CCNA2 MIS18A TTC28 CETN3 PMF1 PBK NUP88 CENPE RPS6 OPA1 PAPD7 HORMAD2 NDC80 DNM1P34 MUL1 RAD54L PRKAR1A EME1 CDC23 CDK2 TUBB3 NEDD9 ITGB3BP KIAA1383 FZR1 SUGT1 MLH1 HAUS8 SYCE1 CENPV NCAPD3 CENPA HAUS2 BIRC7 CENPC1 CEP57L1 CHMP5 PIM2 ANAPC5 SLC25A46 STRA13 ARHGEF2 KIF18B MASTL VCPIP1 LMLN PAFAH1B1 DNM1 FSD1 SKA1 FANCA BCL2L11 MTFP1 PINX1 FAM54A KIF25 FMN2 TEX15 TRIP13 CHMP1B NEK9 NDE1 ZW10 EML4 C7orf11 PPP5C NR3C1 ABL1 SMC1B CD2AP BIRC3 CLASP1 STAG2 SUN1 CDC26 RAD21 ANKRD53 MX2 CDK1 CCNK DCTN2 CKS2 BRCC3 JTB DDX4 ENSA C15orf23 NCAPG KIF4A SETD8 NUP37 CCDC99 NEK2 MRE11A CASC5 MX1 PPP2R1A ANAPC10 MAEL MEI1 SMC4 SMC3 TERF1 PHF13 SYCP2 BECN1 BRDT TTYH1 CETN1 C17orf87 LRRCC1 LFNG NUMA1 NOLC1 RB1 BRSK2 SMC1A SMCR7L FANCD2 MAU2 TADA3 INO80 DPEP3 LATS2 ERCC4 USP37 MAPRE3 CDC25A RCC1 PIBF1 PEX11G KIF15 SPC24 SEC16B ZNF207 ACTR2 NIPBL KIF14 PTTG2

GO_CELLULAR_PROTEIN_COMPLEX_DISASSEMBLY The disaggregation of a protein complex into its constituent components, occurring at the level of an individual cell. Protein complexes may have other associated non-protein prosthetic groups, such as nucleic acids, metal ions or carbohydrate groups. MRPL15 DSTN STMN1 MRPS25 MRPL10 KIF19 MRPL14 MRPL47 HSPA8 MRPS22 GSPT2 DNAJC6 KIF18A CFL2 MRPL4 MRPL9 MRPS24 MRPS7 MRPL33 KIF14 MRPS10 MRPS31 MRPL32 MRPL40 MRPL35 MRPS33 GAK MRPL20 MRPS28 C12orf65 NAPA GFM2 MRPS26 MRPS5 STMN3 KIF18B AURKAIP1 VIL1 MRPS18B VPS4A MRPL54 NAPB MRPL38 MRPL27 SYNJ1 MRPL3 MRPS12 C1orf96 N6AMT1 MRPS14 MRPS11 MTRF1 MRPL21 MRPS9 MRPL13 MRPL19 MRPL39 MRPL48 VPS4B MRPL51 MRRF PTCD3 MRPS18C MRPL41 GSPT1 MRPL46 MRPS6 TRMT112 MRPS35 MICAL3 MRPS30 MRPS27 MRPL49 APEH MRPS15 MRPS23 MRPL18 MRPL22 MRPL11 MRPL34 MRPS16 MTRF1L MRPL52 MRPS34 MRPL12 MRPS17 NCKAP5L MRPL55 MICAL1 MRPL24 MRPL44 MRPL16 MRPS21 STMN4 ETF1 MRPL50 KIF2C MRPL28 MRPL43 MRPL1 MRPL36 DAP3 PEX14 MICAL2 KIF24 MRPL53 MRPS36 MRPL23 MRPS18A ICT1 MRPL42 MRPL45 CHCHD1 KIF2B ABCE1 MRPL2 MRPL37 MRPL30 CFL1 MRPL17 GADD45GIP1

GO_CYTOKINE_PRODUCTION The appearance of a cytokine due to biosynthesis or secretion following a cellular stimulus, resulting in an increase in its intracellular or extracellular levels. IL18 GAS6 TUSC2 CD96 PRKCD SEH1L HSPD1 IL12RB2 TREM1 IL25 CD4 CAMK4 MAF LIPA CD226 LCP2 CMA1 IL12B CD28 KIT IL31RA NLRP3 PYCARD LEP RBPJ NOD2 PTAFR IDO1 TRIM56 PTGS2 IRF7 PNP LYN STOML2 WNT11 BATF MR1 TXK DLG1 FOXP3 TLR6 C13orf15 KLRF2 VTCN1 FABP4 IL1RAP CHGA S100A12 NFATC2IP BTN3A2 C20orf160 IRAK4 CCL19 TLR9 PIK3CD EOMES IL19 DBH SLAMF1 IFNAR1 HIF1A CEBPE NFAT5 IL17F RFTN1 LILRB1 CASP1 NFATC2 LTBP3 NOS2 HEG1 PLA2R1 NOTCH1 NR1H4 BCL10 CCR7 S100A13 S100A9 DENND1B GPAM TNFSF4 F2RL1 AVPR2 AGT GPR56 TRIM27 PCSK5 PAWR IRF3 FGFR1 IL1B CD36 RASGRP1 TLR2 TBK1 ITK PIK3CG SIRT1 GBP5 SLC11A1 ADAMTS3 PLA2G1B NLRC4 CD46 RAB1A BTN3A1 CHI3L1 AIM2 NOX5 ABCA1 AZI2 IL4 GATA3 S1PR3 ASH1L CD55 NOD1 S100A8 HMGB1 TLR4 TMEM173 FOXP1

GO_REGULATION_OF_TORC1_SIGNALING Any process that modulates the frequency, rate or extent of TORC1 signaling. SESN2 SEH1L RNF152 ATM RRAGC GATSL3 STK11 SESN3 DEPDC5 RRAGA GATSL2 SESN1 PIH1D1 TELO2

GO_POSITIVE_REGULATION_OF_NEUROTRANSMITTER_TRANSPORT Any process that activates or increases the frequency, rate or extent of the directed movement of a neurotransmitter into, out of or within a cell, or between cells, by means of some agent such as a transporter or pore. DTNBP1 SNCA UNC13B NLGN1 PARK2 STX1A UNC13A RAB3GAP1 STX1B TACR2 RAB3B DRD4 GPER DRD2

GO_REGULATION_OF_PHOSPHOLIPASE_C_ACTIVITY Any process that modulates the frequency, rate or extent of phospholipase C activity. HTR2A LPAR1 SELE C5AR1 GNA15 GNAQ NMUR1 PRKCE TXK RASGRP4 PDGFRB ESR1 ITK RASGRP1 FGFR1 EDNRA PTAFR AGT ANG ABL2 FGF2 KIT AVPR1A EGFR ADCYAP1R1 FLT1 HTR2B ABL1 ADRA1A S1PR4 PLCB2 LPAR2 PRKCD BICD1 GPR55 PDGFRA AVPR1B

GO_MITOCHONDRIAL_MEMBRANE_ORGANIZATION A process that is carried out at the cellular level which results in the assembly, arrangement of constituent parts, or disassembly of a mitochondrial membrane, either of the lipid bilayer surrounding a mitochondrion. SLC25A5 ZNF205 BCL2L11 RHOT1 TIMM50 EYA2 THEM4 NOL3 PPIF YWHAH HEBP2 TFDP1 TIMM9 PPP3R1 PPP1R13B ATPIF1 RHOT2 BID HSP90AA1 ACAA2 E2F1 MPV17L YWHAE BNIP3L HK2 TOMM22 CHCHD3 BCL2L1 GSK3A CHCHD6 YWHAG TP53BP2 GSK3B PMAIP1 BNIP3 TP73 BCL2 TOMM20 DYNLL1 PPP3CC HSPA4 IMMT APOO GZMB TMEM102 NMT1 BAD C2orf18 SLC9A1 OMA1 C22orf29 BMF NDUFA13 MTCH2 SFN CAMK2A SNCA TIMM22 HSPA1A YWHAZ C11orf83 NAIF1 MOAP1 SAMM50 TAZ MAPK8 AFG3L2 COX18 STAT3 BAX TOMM7 TP63 TIMM10 APOOL OPA1 YWHAQ TP53 CNP BAK1 CASP8 ATF2 DYNLL2 MUL1 TOMM20L LETM1 YWHAB MFN2 C19orf70 ALKBH7 BLOC1S2

GO_ACTIVATION_OF_INNATE_IMMUNE_RESPONSE Any process that initiates an innate immune response. Innate immune responses are defense responses mediated by germline encoded components that directly recognize components of potential pathogens. Examples of this process include activation of the hypersensitive response of Arabidopsis thaliana and activation of any NOD or TLR signaling pathway in vertebrate species. PRKCD HAVCR2 DDX58 PSMD1 TRIL PRKACB PSMC5 TNIP2 NFKBIA NOD2 PSMB3 NRAS MARCO PSMA6 PYCARD CARD9 FAM105B LYN PSMC4 UNC93B1 PSMA3 PSMD9 TNIP3 CD86 BTRC FFAR2 ICAM3 PGLYRP2 PSMD3 MAPKAPK2 CUL1 TRAF6 TIRAP CTSK PIK3R4 IFI16 PSMB5 CARD11 IRAK3 PSMB10 RIPK2 PSMB11 PSMD10 IRAK4 LY96 SRC TRIM5 LGMN UBE2V1 PSMD4 PAK2 CHUK BCL10 RFTN1 CLEC6A TLR10 TAB2 CTSL1 CD14 MB21D1 UBE2D2 TAB3 IRF3 ITCH FADD KLRK1 SIN3A LBP CLEC4E PSMD7 FBXW11 IKBKE ITGAM IFIH1 CD36 TLR8 CTSB IKBKB CNPY3 TICAM2 UBE2D1 RAF1 IRAK1 UBC PSMD2 CD180 UBB NLRC4 RELA AIM2 MAP3K1 PSMD6 MAP3K7 TMEM173 NOD1 PSME4 PSMA4 CD209 HRAS PGLYRP4 PSMB1 PSMA7 UBE2N PSMD8 MYD88 TAB1 RPS6KA3 TLR1 PDPK1 HSPD1 PSMC2 LSM14A PIK3C3 IKBKG REG3G CTSS PSMD5 RELB LGALS9 PAK1 CLEC4C PSMC3 PSMC1 TLR5 PSMB7 PRKACA PSMB9 PSMB2 NFKB1 S100A14 SYK TLR3 PSMB4 DMBT1 CLEC4A PIK3AP1 PAK3 PSMF1 CLEC4D PSMD11 FCN1 IRF7 MAP2K6 HCK PSMB8 PSMD13 ICAM2 CASP8 MAVS TLR6 RPS27A BTK CREBBP EP300 TLR9 ADAM8 TRAF3 PLCG2 TICAM1 PSMA1 PSME1 FYN PSMD12 FCER1G NR1H4 PGLYRP3 PSMC6 UBA52 SKP1 PSMA2 BIRC2 SCARA3 PSMD14 RPS6KA5 BIRC3 TNFAIP3 ITGB2 KRAS IRAK2 COLEC12 TLR7 MAPKAPK3 CYLD TBK1 TLR2 PSMA5 TANK TNIP1 PSMA8 PSME2 RIPK1 HSP90B1 MALT1 PRKCE PGLYRP1 PRKACG PSME3 CLEC7A TLR4 HMGB1 KLRC4-KLRK1 PSMB6 UBE2D3

GO_OUTFLOW_TRACT_SEPTUM_MORPHOGENESIS The process in which the anatomical structures of the outflow tract septum are generated and organized. The outflow tract septum is a partition in the outflow tract. SMAD6 FGF8 TBX1 DVL3 ISL1 BMPR2 NKX2-5 TGFBR2 FGFR2 MSX2 SMAD4 RARB TBX2 TGFB2 BMP4 GATA6 ACVR1 ZFPM2 ENG RARA TBX20 BMPR1A PARVA

GO_DNA_LIGATION The re-formation of a broken phosphodiester bond in the DNA backbone, carried out by DNA ligase. LIG3 TOP2A LIG1 APTX PARP3 XRCC6 HMGB2 PARP1 MGMT POLB C9orf142 HMGB1 PARP2 LIG4 XRCC4 XRCC1

GO_CAMERA_TYPE_EYE_PHOTORECEPTOR_CELL_DIFFERENTIATION The process in which a relatively unspecialized cell acquires the specialized features of a photoreceptor cell in a camera-type eye. PDE6C ROM1 SOX8 SOX9 THY1 DSCAM HCN1 RP1 PTN RORB TOPORS SDK2 GNAT2 CABP4 PROM1

GO_CELL_CELL_SIGNALING_INVOLVED_IN_CARDIAC_CONDUCTION Any process that mediates the transfer of information from one cell to another and contributes to the heart process that regulates cardiac muscle contraction; beginning with the generation of an action potential in the sinoatrial node and ending with regulation of contraction of the myocardium. KCNA5 CACNA1C CASQ2 PKP2 CACNA1G SCN3B GJA1 RYR2 SCN10A SCN5A CACNB2 SCN1B SCN4B GJC1 KCNE1L HCN4 ANK2 CACNA1D KCNQ1 NUP155 RNF207 GJA5

GO_POSITIVE_REGULATION_OF_PROTEIN_AUTOPHOSPHORYLATION Any process that activates or increases the frequency, rate or extent of the phosphorylation by a protein of one or more of its own residues. RAP2B PDGFD IQGAP1 GREM1 CALM3 INS NEK10 ACE NBN PDGFC RAD50 CALM1 RAP2C RAP2A SRC CALM2 PDGFA MRE11A RASSF2 PDGFB TOM1L1 VEGFA VEGFC

GO_TRNA_METHYLATION The posttranscriptional addition of methyl groups to specific residues in a tRNA molecule. WDR4 RG9MTD1 THUMPD2 MTO1 TRMT6 RG9MTD2 METTL19 TRMT1L TRMT61A METTL2A GTPBP3 NSUN2 HSD17B10 THUMPD3 METTL1 C9orf156 KIAA1456 FTSJ1 TRMT61B TRMT11 TRDMT1 TARBP1 TRMT1 NSUN6 TRMT5 CCDC76 TRMT112 ALKBH8 METTL2B LCMT2

GO_MODIFICATION_BY_SYMBIONT_OF_HOST_MORPHOLOGY_OR_PHYSIOLOGY The process in which a symbiont organism effects a change in the structure or processes of its host organism. PTX3 BCL2L11 KPNA4 ZC3HAV1 SLC25A6 PABPN1 C9 SUGT1 KPNA3 HIPK2 EIF2AK4 HYAL2 CD4 ALB KPNA1 BCL2L1 RRAGA ATG7 SERPINB9 SMAD3 CCNK RXRA CLEC4M PARK2 TYMS TGFB1 TNIP1 KPNA2 ZC3H12A KPNA7 CASP8 EIF2AK2 CPSF4 INSR VAPA KPNA5 VAPB ITGAV KPNB1 DAG1 NTRK3 CD209 BAD APCS

GO_SOMATIC_DIVERSIFICATION_OF_IMMUNE_RECEPTORS_VIA_SOMATIC_MUTATION The process in which immune receptor genes are diversified through somatic mutation. POLQ POLM MLH1 EXO1 AICDA PMS2P1 PMS2P5 MSH6 UNG MSH2 ADAR PMS2 POLL POLB

GO_NEGATIVE_REGULATION_OF_STRESS_ACTIVATED_PROTEIN_KINASE_SIGNALING_CASCADE Any process that stops, prevents, or reduces the frequency, rate or extent of signaling via the stress-activated protein kinase signaling cascade. FKTN DLG1 TP73 EZR AKT1 MEN1 MARVELD3 PARK2 DUSP19 PDCD4 MYC DACT1 ZNF675 SERPINB3 PAFAH1B1 PTPN22 DUSP3 GPS2 DNAJA1 IGBP1 TAOK3 HDAC3 FOXO1 ZMYND11 MECOM DUSP10 FOXM1 PBK PER1 SFRP1 SFRP2 PINK1 ITCH F2RL1 NCOR1 HIPK3 AMBP AIDA GSTP1 MAPK8IP1 HSPH1

GO_REGULATION_OF_ESTABLISHMENT_OF_PROTEIN_LOCALIZATION_TO_CHROMOSOME Any process that modulates the frequency, rate or extent of the directed movement of a protein to a specific location on a chromosome. TCP1 CCT7 CCT5 CCT4 TERF1 KIAA0146 CCT8 WRAP53 CCT2 CCT6A DKC1

GO_BUNDLE_OF_HIS_CELL_TO_PURKINJE_MYOCYTE_COMMUNICATION The process that mediates interactions between a bundle of His cell and its surroundings that contributes to the process of the bundle of His cell communicating with a Purkinje myocyte in cardiac conduction. Encompasses interactions such as signaling or attachment between one cell and another cell, between a cell and an extracellular matrix, or between a cell and any other aspect of its environment. SCN5A SCN10A GJA5 DSC2 KCNA5 DSG2 JUP PKP2 CTNNA3 TNNI3K DSP

GO_REGULATION_OF_PROTEIN_LOCALIZATION_TO_CELL_SURFACE Any process that modulates the frequency, rate or extent of protein localization to the cell surface. CTNNB1 TOR1A EGF KCNAB2 CAV3 ACTN2 NEDD4L RANGRF STX4 STX3 GPD1L LEPROT TM9SF4 TNF GPM6B HFE ABCA12 RAB11FIP5 ERBB4 SNX33 NRG1 SYNJ2BP GBF1 TAX1BP3 RAB11B ASTN2 COMMD1

GO_ROSTROCAUDAL_NEURAL_TUBE_PATTERNING The process in which the neural tube is divided into specific regions along the rostrocaudal axis. HES3 PAX6 HES1 WNT1 FGF8 LRP6 EN1 GBX2 SOX17 KDM2B SSBP3

GO_AMINOGLYCAN_METABOLIC_PROCESS The chemical reactions and pathways involving aminoglycans, any polymer containing amino groups that consists of more than about 10 monosaccharide residues joined to each other by glycosidic linkages. PGLYRP2 GUSB CHST2 TGFB1 B3GNT3 OMD GPC6 SDC3 XYLT2 CHST5 IDS CHSY1 CHST6 CHIA GAL3ST3 CHST3 B3GNT4 B4GALT7 B3GNT8 IL15 GPC5 NAGLU DCN B3GAT3 SPOCK2 EGFLAM GPC4 HS6ST2 HS3ST2 ITIH4 CHST9 CD44 VCAN HS3ST1 CHST14 HS3ST5 OGN FUCA1 ARSB AKT1 CYTL1 CHI3L2 XYLT1 CHST12 ACPL2 NCAN HS3ST3A1 SDC4 HS6ST1 PGLYRP4 SLC9A1 HEXA LYG1 NDST1 HGF IDUA HYAL3 LUM UST NDST4 SGSH UGDH CHST11 B3GAT2 PRELP HPSE2 B4GALT6 FMOD ANGPT1 CHST15 ITIH2 CHST13 CSPG4 B4GALT1 LYVE1 NDNF EXTL1 PIM1 OVGP1 HGSNAT STAB2 ITIH3 ST3GAL4 SLC35D2 CSGALNACT2 B3GNT2 EXT1 CHID1 HAS1 B3GALT6 EXTL3 SDC1 ABCC5 GALNS EXT2 SPACA3 BGN DSE ITIH1 SDC2 CHP IMPAD1 DSEL MKI67 BCAN GLCE GPC2 GCNT2 EXTL2 HEXB SPOCK3 CHPF2 HPSE GLB1 CHSY3 NDST3 ST3GAL6 KERA HS2ST1 HMMR B4GALT4 GNS KIAA1199 PDGFRB B4GALT2 ST3GAL2 IL1B CHST1 HS3ST6 B3GNT1 ITIH5 FOXC1 NDST2 HS3ST3B1 AGRN SLC35D1 CTBS B3GNT7 PGLYRP1 LYG2 CHI3L1 CHPF CSGALNACT1 CSPG5 ST3GAL1 PGLYRP3 GALNT5 GPC1 ST3GAL3 HYAL1 HAS2 GXYLT1 GPC3 FGF2 CLN6 ACAN HAS3 B4GALT5 ITIH6 HSPG2 HYAL2 CHIT1 CHST7 B4GALT3 HYAL4 B3GAT1

GO_ASYMMETRIC_PROTEIN_LOCALIZATION Any process in which a protein is transported to, or maintained in, a specific location such that it is distributed asymmetrically. DYNC2H1 MPP5 VANGL2 MAL CELSR1 SHROOM2 HCN1 COLQ NAPA ARF4 SCRIB RAB10 DLG5 GOPC ERBB2IP RDX WNT7A SHROOM3

GO_LIPID_MODIFICATION The covalent alteration of one or more fatty acids in a lipid, resulting in a change in the properties of the lipid. IMPAD1 PIK3R2 PTPRQ PIK3C3 ALOX12 LPPR1 SLC27A2 SPHK2 NRG1 HBEGF DGKA INPP5A LCAT PIP4K2C KL PTPN11 PIP5K1B PIP5KL1 PPARD EGF CD28 CD80 CYP4V2 FGF18 INPP5D ACAD8 PLA2G7 SYNJ2 SLC25A17 FGF23 FAM126A SOAT1 FGF17 PIP5K1C FGF7 PIPSL ACAT2 FGF5 HADHA FGF6 IVD ETFB ADIPOR1 EPHX2 PIK3C2G INPP5B HIBCH LPPR5 POR PDGFB ACAT1 FGF16 LPPR4 CYP3A5 EREG PRKAA1 PIK3C2A PHYH HAO1 INPP5K EFR3A FGF19 CPT1C SMG1 DGKB PEX2 FYN B4GALNT2 ECI2 ACOX1 ABCD1 ACOX2 FGF2 PIP5K1A MTMR8 PEX7 PIK3R6 EFR3B FRS2 AMACR NUDT19 INPP1 ACADL PIKFYVE GCDH FGF10 EHHADH PDGFRB AUH MTMR7 LPPR2 ADIPOQ CYP2C8 ACAD10 ACSM1 PIK3CG FGFR1 CYP3A7 DGKI CYGB ERBB4 FGF9 PPAP2A FGF22 SYNJ1 PPAP2C NRG2 CPT1B SCP2 CD19 GRB2 ACAD9 SESN2 MTMR1 FGF1 DGKG PIK3R1 CYP1A1 ABCD3 PTEN ADH7 CYP3A4 PIK3CB PIK3CA LCK FGFR4 HAO2 ACOT8 ABCD2 VAV1 PI4KB SPHK1 ADIPOR2 PIK3C2B IMPA2 BDH2 CPT1A PI4KAP1 INPP5J PI4K2B PI4K2A ECHDC2 MTMR2 LEP TTC7B IP6K1 KIT ACAD11 PIK3R5 ACADS HACL1 CERK TRAT1 CD86 PPAPDC1A PPARG CROT INPP5F ACOX3 CPT2 FGF8 INPPL1 PIK3CD B4GALNT1 TMEM55A BTC HADH MTMR6 SOAT2 DECR1 TMEM55B KLB SLC35C1 FGF3 ERBB2 FA2H TMEM150A INPP5E CRAT FGF20 DGKQ PDGFA ACOXL ECHS1 ALDH3A2 OCRL PEX13 FGF4 HSD17B4 ECI1 PDGFRA PI4KAP2 ETFDH NRG4 AGK MAPK14 ACADVL EGFR SACM1L GAB1 ACAA2 ECH1 IRS2 MTMR3 PIP4K2A DGKD ACAA1 DGKZ FGFR3 PPARGC1A IRS1 KITLG IP6K2 ETFA A3GALT2P ERBB3 LPPR3 IMPA1 PPAPDC1B MTM1 HADHB BPNT1 PIP4K2B ACADM FGFR2 ACADSB DGKE PI4KA PPAP2B

GO_ASSOCIATIVE_LEARNING Learning by associating a stimulus (the cause) with a particular outcome (the effect). NPS FOXB1 CTNS CHRNB2 TAC1 RIC8A GRM7 PIAS1 DRD5 HMGCR APP ITGB1 GABRA5 NETO1 ATP1A3 UCN CDK5 DRD1 NDRG4 MTOR MECP2 FOS MEIS2 DRD2 KIT SRF TBR1 EIF4A3 ADCY3 PDE1B NEUROD2 B4GALT2 CRH PPT1 NPTX2 HRH2 HRH1 CREB1 SNAP25 DDHD2 OPRK1 LRRN4 ACCN2 GRIN2A RELN GRIN1 ADAM2 DEAF1 ATP1A2 NRGN PPP1R1B SHANK1 BTG2 CHRNA7 BRAF IFT20 RIN1 NF1 SYNGAP1 HIF1A RGS14 DBH KRAS TNR MLL NLGN3 NPTN DRD3 TANC1 ABL1 CLN3

GO_PEROXISOMAL_TRANSPORT Transport of substances into, out of or within a peroxisome, a small, membrane-bounded organelle that uses dioxygen (O2) to oxidize organic molecules. RAB8B PEX14 PEX13 PEX2 PEX5 PEX12 PEX3 LONP2 PEX5L PEX7 PEX1 ABCD1 PEX16 PEX19 ZFAND6 PEX26 PEX10 PEX6

GO_INNER_EAR_RECEPTOR_STEREOCILIUM_ORGANIZATION A process that is carried out at the cellular level which results in the assembly, arrangement of constituent parts, or disassembly of a stereocilium. A stereocilium is an actin-based protrusion from the apical surface of inner ear receptor cells. CTHRC1 GRXCR1 HES1 SOD1 CDH23 ATP2B2 SLC9A3R1 IFT27 PDZD7 USH1G MYO7A DFNB31 VANGL2 HES5 MKS1 SDC4 SEC24B IFT20 SCRIB STRC TRIP11 CLIC5 FAT4 LHFPL5

GO_RESPONSE_TO_MAGNESIUM_ION Any process that results in a change in state or activity of a cell or an organism (in terms of movement, secretion, enzyme production, gene expression, etc.) as a result of a magnesium ion stimulus. FGF23 KCNC2 SLFN14 D2HGDH SNCA CCND1 RYR3 FBP1 ANK3 MDM2 CD14 ENTPD6 KCNA1 BMP6 SLC34A1 THBS1 SLC41A1 CNGA3 TNFRSF11B

GO_PROTEIN_MANNOSYLATION The addition of a mannose residue to a protein acceptor molecule. FKTN GYLTL1B POMT2 SDF2 TMEM5 DPY19L2P2 NUS1 LARGE DPM3 POMT1 DPY19L4 DPY19L3 DPM2 DPY19L2 DPY19L1 ALG5 SDF2L1 B3GNT1 DPM1 C3orf39 FKRP ISPD

GO_KINETOCHORE_ORGANIZATION A process that is carried out at the cellular level which results in the assembly, arrangement of constituent parts, or disassembly of the kinetochore, a multisubunit complex that is located at the centromeric region of DNA and provides an attachment point for the spindle microtubules. CENPH POGZ CENPT CENPC1 CENPA CENPE MIS12 CENPW APITD1 SMC2 STRA13 CENPF SMC4

GO_NEGATIVE_REGULATION_OF_CARDIAC_MUSCLE_CELL_APOPTOTIC_PROCESS Any process that decreases the rate or extent of cardiac cell apoptotic process, a form of programmed cell death induced by external or internal signals that trigger the activity of proteolytic caspases whose actions dismantle a cardiac muscle cell and result in its death. SIRT5 SFRP2 ILK PDPK1 NKX2-5 SIRT4 NFE2L2 JAK2 MYOCD NOL3 HEY2 HAND2 RGL2 HSF1

GO_DOPAMINE_RECEPTOR_SIGNALING_PATHWAY The series of molecular signals generated as a consequence of a dopamine receptor binding to one of its physiological ligands. KLF16 GNB1 GNA14 GSK3B FLNA GSK3A ADCY6 GNAI3 CALY GPR52 RGS9 GNAS GNAO1 DRD1 HMP19 ADCY5 GNA11 DRD4 DRD2 GNAQ GNAL GNA15 SLC9A3R1 DRD3 ARRB2 GPR21 DRD5 D4S234E GNG2 OPRM1

GO_NEGATIVE_REGULATION_OF_OSSIFICATION Any process that stops, prevents, or reduces the frequency, rate or extent of bone formation. ECM1 TGFB1 HDAC4 LRP5 TWIST2 GATA1 GDF10 MEN1 UCMA HAND2 AHSG SMAD6 FGF23 NBR1 FAM101A BCOR CHSY1 C14orf169 SEMA4D NOG LRP4 ID1 TWSG1 CCR1 SRGN TWIST1 SKI ID2 PTCH1 ZNF664-FAM101A CHRD CDK6 ID3 RBPJ CCL3 SMAD3 AXIN2 GREM1 TOB1 GFRA4 CITED1 BCL2 TMEM64 AREG LIMD1 TRPM4 SUFU MEF2C MEPE HOXA2 STATH PTK2B TPH1 SOX9 CALCA NOTCH1 SOST FAM101B IGFBP5 HIF1A RORB SMURF1 HDAC7 TNF SFRP1 CCRN4L OSTN

GO_INTESTINAL_EPITHELIAL_CELL_DEVELOPMENT The process whose specific outcome is the progression of a columnar/cuboidal epithelial cell of the intestine over time, from its formation to the mature structure. NKX3-2 C1GALT1 C12orf5 CDKN1A HIF1A PRDM1 SPDEF YIPF6 HOXA5 TYMS KLF5

GO_BIOLOGICAL_ADHESION The attachment of a cell or organism to a substrate, another cell, or other organism. Biological adhesion includes intracellular attachment between membrane regions. CDHR1 FZD8 PKM2 TNFAIP6 CAPZA1 TIGIT FIBP APC PAX1 MICALL1 PTPN2 AHSA1 LAMA5 EMR2 DOCK8 ALDOA ABL1 DSG4 NUAK1 F2RL1 TJP1 IGFALS ICAM5 GPA33 CD151 PVRL4 HAS3 SH3GL1 TNN KIFC3 ADAMTS13 NKAP SPP1 COL6A6 PCDHB3 ITGA5 CDH2 STK24 SPTBN1 SOX9 PATZ1 GP9 ARF6 PTK2B ICOS PTPN6 PCDHA6 HMGB1 CD1D GAS6 F11R GCN1L1 EIF4G1 HIST1H3I RAB10 CADM3 RORA CCR1 TGFBR2 TROAP HIST1H3F FAT1 ROBO2 CDH18 CAPG CLDN1 NLGN4X SYK TBX21 PCDH11X HCK CLDN17 CLEC4D RSAD2 MPL DNAJA3 RPS6 ICAM2 FNDC3A UNC45A ADAM22 CASK DLG1 HSPB1 PCDHA3 CYR61 AMTN PRKD2 ENG PAICS COL12A1 TMEM8B PIP5K1C LPP TM9SF4 SNX5 MAFB AIMP1 NOTCH3 RPSA DGCR6 ATP2A2 PARVB IFNA10 CELSR1 COL3A1 S100A9 EMD GPR18 PCDHGC4 SELP COL13A1 EMILIN2 DDX3X ITGAM TTYH1 DIAPH3 SPACA4 PCDH1 EPDR1 RPS26 EFNB2 PPP3CB PCDHB6 MYH9 STAT5B CDH23 CD1C LPHN1 ARHGAP18 CDH10 LY6D SELE MERTK EGR1 TGFBI PKP4 SCARF2 ARHGAP5 RHOB TLN2 VCAM1 FZD4 PCDHGA7 ZC3H15 PCDHB15 OMD CUZD1 MLLT4 MPDZ PCDHGB3 CDH16 BCAM PCDHA1 ERC1 MPP7 VAPA COL16A1 IFNA1 PTPRM PSMB11 BGLAP PCDHGB2 SIGLEC16 PTPRF SIGLEC5 LEF1 USO1 ERBB2IP MACF1 PCDHB16 SEPT7 ITGB5 CLDN4 RPS2 CD63 FERMT2 ZFP36L1 IL18R1 PFKP AZGP1 PCDHGB1 ICAM4 TBC1D10A PDXDC1 PICALM LY9 LGALS3BP PKD1L1 LAMB2 HLA-E DST CLEC7A EMP2 CHD7 FAT3 PSMB6 GIPC1 PLEC MYO1G STK38 BCAN MAEA ITGB1 ODZ1 TRO ZFP36L2 IFNA13 CNTNAP5 ID1 PDPK1 SWAP70 GIGYF2 CD80 LYPD3 SRF TREML2 GCNT1 EFS ACTN2 IFNB1 ZAN ABL2 SERBP1 HPSE STX5 HAS1 GPRC5A TEK SELL MPRIP DSG3 CD200 RNASE10 DBNL NRXN2 YKT6 KIAA1524 SND1 DSG2 ARHGEF16 ACTG1 ITGAD EIF2A PCDHGA5 IDH1 BZW1 FREM2 GRID2 EHD4 TRPM7 RC3H2 BRAF PCMT1 PLXNB3 CXCR7 HAPLN2 ITGA10 CLDN6 OLA1 IGSF9B DGCR2 CLDN12 TMOD3 PGM5 MFGE8 MYL9 SHH SIGLEC6 CNO CD36 STRCP1 SEMA5A CDH13 CDHR2 FAT2 ANXA9 STXBP6 MSN CGREF1 EVPL MUC4 PPAP2B DOCK2 LRRC59 EPHA1 DSC3 VMP1 KIAA1598 EDA EEF2 CNTN6 FASN VCL CGN ABI1 ITGB4 UNC5D RAG1 SMAGP PPFIA1 CXCR3 SPTBN2 ACHE PTPN1 CD300A CD226 CCL21 WISP2 PFN1 HIST1H3H CHST4 IL12B ZAP70 PCDHB10 COL14A1 CNTNAP3 VCAN SIGLEC11 ITGB1BP1 DEFB118 RND3 TNFRSF12A RPL23A NT5E NLGN2 MKL2 CD81 WNT1 MICALL2 ANGPTL3 FGA COL8A1 DHX29 PVRL2 COL4A3 COL5A1 VWA2 DCHS1 PIK3CD CLDN16 PCDH11Y INPPL1 CDH22 RAC2 MYBPH EBI3 CHRNA7 FCER1G ANXA2 CCR7 AMBP LOXL2 EEF1D DDR1 S100P IFNA17 PTK7 PCDH19 COL4A6 DOCK9 CNTNAP2 PACSIN2 MICB OTOA MAG ACAN SVEP1 TNR IL1B SIGLEC14 SNX1 PCDHA8 COBLL1 RHOD EPHA8 PLXNB2 CLDN14 CLDN11 RUVBL1 ACVR1 PKD1 GP5 CERCAM P2RX7 TOR1A RANBP1 PODXL2 SSPO FREM1 IFNA14 ADD1 CCT8 BMP5 FERMT1 ASTN1 PCDHA12 C1orf38 APP KIAA1462 CLSTN3 PNN BSG RELB PPARD WASF2 KIRREL DDOST CTNND1 SSX2IP ZYX THBS4 CLEC4A ROCK1 CLDN3 FLNB PRKX PKP2 PLIN3 PCDHGA2 TRIM25 VAPB RUNX2 JAG2 CX3CL1 IFNA2 LRRN2 CDH5 ITGA4 CLDN22 FLRT2 HBB FOLR4 SIGLEC1 SIGLEC12 LAMA4 EPCAM IL1RAPL1 CD2 SELPLG CCL5 RDX ARVCF CLEC4E CDH1 RIPK3 CD22 MFAP4 SPG7 MEGF10 WAS PKNOX1 PCDHB7 DNAJB1 TIAM1 MARK2 TNC RHOH CD99L2 ANTXR1 LSAMP ITGB6 SPON2 SPON1 PCDHAC2 OLR1 NRP2 SIGLEC7 PDPN PDLIM1 BAIAP2L1 RPL15 CNTN3 METAP1 AKIP1 MTSS1 DSCAML1 CD8B CD7 EXOC3 TNFSF18 CNTN4 RAB7L1 AOC3 STOML2 CD86 EFHD2 RAP2B TMEM2 CLDN2 BYSL CARD11 ITGB8 FYN HES5 HSPA8 ITGA7 AMIGO3 S1PR1 LCP1 VTN PCDHB11 PRDX6 RAG2 RAB27A RORC SOX4 IL7R CADM1 PVR ITGB2 FERMT3 CX3CR1 PAK6 FSCN1 PCDHB1 NLGN4Y PIK3CG LGALS4 HIST1H3A ARFIP2 SEPT9 PCDH20 SATB1 CCND3 CEL APOA4 COL5A3 HIST1H3D BCL3 LAMC1 CXADR NEDD4 CORO1B MALT1 AIRE PCDHB8 MYBPC3 SHC1 PKHD1 IL32 GOLGA2 ODZ4 H1FX LAMB4 IFNA6 GRHL2 ADAM23 ROPN1B CELSR2 EIF2AK4 IGFBP7 KRT18 PVRL1 FBLN5 CD72 CNN2 FBLIM1 BOC ITPKB EPHB3 IFNA8 ESAM SMAD6 PCDHB12 PCDHB18 ITGA6 CDH19 CHMP2B SLURP1 NOV TSPAN32 SPAM1 ATXN2L CNTN5 PARVG CNTNAP4 SUSD5 NCR3 UMOD FAS PLA2G2D TGFB1I1 CYFIP2 LYVE1 LGALS7B TNXB BMP7 PRKCA PECAM1 INS PCDHGC3 FOXF1 FADD ATP1B1 CHST10 COL28A1 SORBS3 COL19A1 FKBP1B TACSTD2 CD47 EIF3E IRF4 ENO1 EPB41L1 MEGF11 DLL4 REG3A CCR8 PCDHGA8 ICOSLG SDK2 GLDN PCDHGA11 TPBG CNTNAP1 FMNL2 CLIC1 VWF ZC3HAV1 CC2D1A FAM129B CTNNA1 PCDH12 STAB1 STXBP3 STRC VAV1 SLA2 EPHA3 VAMP3 CLDN7 CNN3 PCDHGA1 ENTPD1 NPHP1 CD58 PTGER4 SPOCK1 EDIL3 AMELX SRCIN1 CDH9 RGMB LAMA2 LAMA3 PCDH9 CAMSAP3 TGFB1 RTN4 DLG5 FUT7 PODXL PERP IL15 FGG AMIGO1 SLAMF6 HEPACAM CDH17 YWHAB EIF4G2 PSMB10 LAD1 SIRPA CRNN BCL2L11 PTPRC THY1 CEP41 DLC1 NUMB FGB ISLR CDH7 CD2AP LRRC15 CDK5R1 GPR56 IFNE PRKDC PTPRT HDLBP CDHR4 GP1BB SDCBP CHMP5 NDRG1 DDX6 CDH3 ITGAE CTNNA3 TNKS1BP1 FAT4 MB21D2 WISP3 SH3GLB2 JAM2 PRKCE ECM2 LMLN PTPRD TAGLN2 FAP ITGB3BP JAM3 NEDD9 DBN1 ILK EGFL6 CLDN18 HCFC1 ACTN3 SGCE LRRFIP1 SCARB1 KAL1 CDH6 NOP56 TSC1 MICA MYBPC1 LDHA UBFD1 IFNA21 CAPZB SCN1B APBA1 PVRL3 PLCB3 PCDH17 MAGI1 CD40LG CLDN8 PKP1 SH3GLB1 CCL19 TECTA CLDN5 EHD1 PDGFRA HAPLN4 TNFRSF4 CALD1 PCDHGA3 COL18A1 SP3 TYRO3 GLI3 AGGF1 AMICA1 PCDHA5 SLK SDK1 PUF60 RS1 ADAM9 CDHR5 PLXNC1 AATF CCS RPL34 KIF14 ADA ELF4 CD3G KITLG CXCL12 LFNG WHAMM ARHGAP6 CLSTN2 MYL12A ADAM2 PI4KA DNAJB6 IL4 THBS2 CLINT1 OMG CDSN CD209 CLDN23 C1orf177 EFNB1 ASTL EIF5 COMP THBS3 DSCAM LILRB2 NRXN1 MRE11A EGFL7 CADM4 PTPRU PCBP1 ARGLU1 WNT7B CD4 LAMB3 TNFSF14 SMAD3 NPHP4 CDK5 CD44 PCDHA4 HAPLN3 CLDN15 BATF GATA1 LIG4 MADCAM1 TES CTTN NINJ1 TAOK2 IBSP DSC2 SRPX CDH4 SIRPG PTPN22 S100A11 MOG SORBS2 CLDN20 ARHGAP1 SLAMF1 ARFIP1 PCDHGA6 PPP3CA JUB CD24 PRDX1 HES1 CD9 CD84 FER RPL14 NLGN3 PCDH18 TRIM29 IFNA7 SCARF1 AHNAK SSPN ITGA11 CD97 GPR183 ATP7A NID1 CNTN1 NEO1 TMPO GPNMB IQGAP1 DSC1 LAT CDH11 LAMC2 NLGN1 ROBO1 STAT6 PRPH2 POSTN CD33 ITGA1 CD96 SCRIB CD6 KIF13B KIRREL2 COL6A1 GLOD4 NME2 CTNNB1 CD28 P2RY12 CD8A CFDP1 THBS1 MMP24 PXN LIMA1 PPL CASP8 SYMPK RSL1D1 NLRC3 EEF1G AMIGO2 ITGB7 CCL2 BZW2 TRIP6 SRC APBB1IP ACTN1 DPT CCR6 RAB11B ASAP1 SLAMF7 RARS ANGPT1 FARP2 RADIL BVES PCDH10 CLDN9 STAB2 HABP2 NPTN MSLNL SIGLEC9 GOLGA3 PCDHGA4 SLC3A2 HAPLN1 NCAN SIGLEC8 VNN1 NHEJ1 CLDN19 IRF1 CCR9 CDON EMILIN1 DCHS2 ITGA3 MYBPC2 CLSTN1 ADAM15 PCDHB9 ARHGEF7 KLC2 IZUMO1 GATA3 CADM2 FGFRL1 FBN1 EPHA4 LYPD5 CRTAM SFN NCAM1 TSTA3 LIMS2 YWHAZ ADAM12 TLN1 SORBS1 IGSF5 PPFIBP1 OLFM4 KIT PHLDB2 NEGR1 JUP FOXN1 NCSTN CDC42 GAPVD1 B2M SNX9 PDLIM5 PCDHA9 COL6A2 TJP2 FLRT3 FLOT2 CTNNAL1 FREM3 EFNA1 SEMA4A ITGAV TGFB2 GP1BA COL15A1 SNX2 ELMO2 PCDH7 AJAP1 IFNAR1 OPCML PDZD2 HIST1H3C SLC9A3R2 IL23A RET C10orf54 CEBPB MCAM KIRREL3 SRPX2 CTPS ATP4B ACE2 TNF SNED1 NUDC CD74 ITGA9 GOLPH3 TNIP1 ITGB3 NTN1 ZBTB1 ODZ3 PCDHGA10 GNE PCDHAC1 MSLN RELN IGSF9 IFNA4 CDH26 CSRP1 DAB1 PEAK1 MYH10 CDH15 LARP1 FZD5 TMEM8A EPS15 FLNA SPECC1L WNT4 ITGA8 HSPD1 NCAM2 VEZT PCDHB13 HSPA5 PCDHB2 FXC1 GPR98 CBLL1 ITGAL PSTPIP1 PPFIA2 IFNG UBAP2 CSNK1D LASP1 RCC2 SPTAN1 FLRT1 FOXP3 ALCAM PCDHA11 EPHB4 CAST PCDHA2 CLCA2 FBLN7 CD276 SPN CDC42EP1 FN1 CRISP2 KIF5B DAG1 B4GALT1 CCR3 USP8 PAK2 PPME1 PKN2 CLDN10 YWHAE IST1 DDR2 EPS15L1 PLEK CYP1B1 LPXN PCDHGB7 EMR1 EPS8L1 PARK7 PCDH15 CLEC4M COL17A1 L1CAM COL7A1 BCAR1 NPNT CASS4 PTPRS APLP1 CRKL NCK2 AZI2 BAG3 LAMA1 CYP26B1 AXL IFNA16 CTNNA2 GNAS FOXP1 MMRN1 STK11 RAPGEF1 FEZ1 IFNW1 LCK SCYL1 ACTB PIK3CB PCDHB5 AMBN NID2 HNRNPK RPL24 MPZL2 LEP HOXD3 LPHN3 CDK6 CBL ESYT2 BAIAP2 FPR2 CBLN1 PAK4 IFNK ICAM3 EZR SEMA4D EPB41L5 CDH8 CDHR3 RIPK2 WISP1 PREX1 NCK1 ATP2C1 ATIC CCL4 EOMES IL2 CORO1A HIST1H3G LILRB1 PCDHA7 ANXA1 VASP BCL11B TESK2 TNFSF4 PPP1R13L CHL1 COL6A5 TXNDC9 MUC16 HIST1H3E CDH24 PIP5K1A CKAP5 EPHB1 RAN NRCAM JMJD6 FKBP1A HSP90AB1 DPP4 BCL2 COL6A3 BMX ANLN NRXN3 RAB1A BTN3A1 KTN1 HIST1H3B S100A8 GNB2L1 EPS8L2 LAMB1 CEACAM1 RPL22 IL6 HSPA1A IFNA5 EPN2 PCDHGB6 ROM1 NINJ2 PARVA MAPRE1 CNTN2 PKP3 NPHS1 CD3D CD99 SRGAP2 MPZL3 TMEM47 HIST1H3J PCDHGC5 CSF3R ODZ2 RC3H1 CTNND2 ADAMTS12 RANGAP1 STK10 CNTNAP3B CCNB2 RAC1 ADAM8 TINAG RPL7A LRP6 ABCF3 ITGAX THEMIS PTPRK ITGA2 MYO6 VASN ATP1B2 CEACAM5 MKLN1 CD34 FNBP1L CD3E TWF2 BMP10 EGFR DSG1 CDH20 NTM IGSF11 MYO1B PCDHA13 ADAM17 PRLR BAI1 PCDHGB5 ITGA2B CD164 WNT3A SEPT2 PLEKHA7 ITK STAT1 SLC11A1 SLC7A11 FZD7 EPHA2 CCL11 TCF7 CDH12 PCDHA10 PCDHGA12 EIF2S3 SIGLEC10 CELSR3 PCDHB14 ZFPM1 PCDHB4 CIB1 PCDHGA9 RPL29 EIF4H STXBP1 TINAGL1 PSEN1 CHMP4B HSH2D LAMC3 PCDH8 BAX CD93 DAB2IP NKX2-3 IL7 TWF1 DSP TNFSF8 ICAM1 LYPLA2 CSTA ITGBL1 COL8A2 CTGF RIC8A LEPR NFASC LRFN3 PCDHGB4 RPL6

GO_TRIGLYCERIDE_CATABOLIC_PROCESS The chemical reactions and pathways resulting in the breakdown of a triglyceride, any triester of glycerol. FABP1 FABP2 APOA5 PNPLA5 PNPLA1 PNPLA3 CPS1 FABP6 APOE FABP7 PNPLA4 APOC3 FABP3 FABP4 APOA1 FABP9 LIPE FABP5 DDHD2 LIPC LPL MGLL FABP12 APOB PNPLA2

GO_HEME_BIOSYNTHETIC_PROCESS The chemical reactions and pathways resulting in the formation of heme, any compound of iron complexed in a porphyrin (tetrapyrrole) ring, from less complex precursors. TSPO CPOX SLC25A38 FECH PPOX FXN SLC25A39 ALAD COX10 SLC11A2 COX15 HMBS UROS IBA57 ATPIF1 UROD TMEM14C NFE2L1 ALAS1 ALAS2

GO_REPRODUCTION The production of new individuals that contain some portion of genetic material inherited from one or more parent organisms. FOXO3 C17orf104 BAD STAG3 DNMT3A SMAD4 POLR1B PHC2 CUL7 PSG7 NLRP5 ZP4 MCM8 SPAG9 SIRT1 CATSPER3 C2orf65 NAMPT DEDD FETUB FOXA3 BOLL AVPR1A PLA2G6 TOP2A SPANXA1 BMP15 MYOCD FABP9 ZFP42 ABAT CDX4 BMP7 HORMAD1 PITX2 PARN ANGPT2 FBXO43 ZFAT DUOX2 MND1 NUDT1 ARG1 ZMIZ1 TGFBR1 NASP TGFB1 PRDX4 STK4 LY6K UBE2B ADRA2C FANCL C8orf42 SSTR3 LEPREL4 PLA2G3 ADAM29 ADAM18 NPHP1 SUN5 TAF1L EIF2C4 FSHB GHRL PCDH12 ZNF296 PLK1 CTNNA1 CRTAP SLX4 FEM1B BASP1 CABS1 USP42 RAD1 SPIRE1 USP9Y ASZ1 TSPY2 SOHLH2 CXADR HSPA2 HERPUD2 TBC1D21 PIWIL4 TDRD5 IRX5 MMP19 SPANXA2 NDRG3 ZNF541 ADCYAP1 SLC2A8 PHB2 SYCE2 CADM1 ESPL1 BRCA2 PCSK5 AKR1C3 HPGD HUS1 DIAPH2 ARRB1 TMEM146 DNAH9 FAM75D3 NOTCH1 PRSS37 PAFAH1B2 PSAPL1 TAF4B HIF1A MORC1 ADIG SKIL TTPA PLA2G4C SERPINF1 ZFP41 DNM2 EPN1 PRL RLN2 TAC1 STRBP ASB1 BMP4 DNAJA1 OSGIN2 SPAM1 IL1A DLD NR0B1 HOXD9 DMRTC1 TTLL5 SDC1 EIF2S2 SMC2 HSF2BP ELSPBP1 WNT2 PARP11 MTOR PTPN11 MNS1 XRN2 ARID4B GIP IGFBP7 CCNA1 DLX3 CDY2B GRHL2 SMAD5 TOP2B ROPN1B LINC00085 EPOR SOX3 SPINT1 PYGO1 NDP P2RX1 SPAG16 CCT3 GOLGA2 DMRTC1B ARNTL C15orf43 MOV10L1 LOC728637 SLC26A6 TNC ACE BIRC6 CORT UBTFL1 ARID5B ZNF830 CASP5 GATA2 HSD17B3 MKKS CEP57 STYX C16orf73 HOXA11 EME2 TBPL1 NKX2-1 OR10J1 FOLR4 STAR CABYR LHX4 PTCHD3 CTSL2 RGN DNAJC19 TBX3 UBAP2L BYSL ADAD1 ITGB8 MTNR1A FAM9C SEPT4 INSR PAX5 CLOCK SGPL1 SLC2A14 PROK2 C11orf85 CBX2 FGF8 ALKBH1 DPCD IDO1 SPIN3 SHCBP1L MLL4 RBPJ ATP8B3 CATSPER4 DMRTC2 MOS TXNDC3 TSSK6 TMED2 AFF4 PLCD1 ADIPOR2 DCN C20orf152 RNF114 PPAT ANTXR1 CRIP1 CCT8 VCX UMPS CCDC155 BMP5 CCNB1IP1 SMCP GAL3ST1 DMRT2 PKD1 ACVR1 PTEN RUVBL1 TSNAX CLDN11 BIK HERC4 IL1B GJB3 XRCC2 FRS2 SFRP2 NSUN2 GNRH1 DMRTA2 DND1 PRM1 TNFSF10 BIRC2 NLRP14 AMBP DDR1 PDGFB AURKA CALCA PPP1R1B ZP3 TFAP2C CHRNA7 FAM75A6 HTRA1 PCYT1B GNPDA1 TSPY4 ACSL4 PTTG3P PGAM2 C11orf20 DZIP1 INSL6 ZPBP2 SERPINA5 CATSPERG FKBP6 JAG2 BMP6 ATM SPIRE2 IRF2BPL KLF1 COL9A3 LHX1 SPATA2 HVCN1 FSHR ZMYND15 KPNA6 IQCF1 TMEM119 NCOA4 HEXB OXTR TRIM36 BSG TRIM28 HOXA10 EXO1 PPARD LIMK2 ADCY3 BCL2L2 APP C14orf133 PDILT SLC6A4 WDR43 BUB3 TNP1 EPO NCAPD2 GLI1 SPHK2 ACR EIF2B5 IHH RPL10L KLHDC3 WNT3 VMP1 FANCF PPAP2B OSBP2 MC2R PSG4 DSN1 SSTR2 MSH3 CCND1 RECK SHH MAPK14 HOXB13 AKAP4 MFGE8 FAM75C1 C17orf46 CCT6B CORIN RQCD1 KIF18A CATSPERB DBH KRT8 IDH1 SOCS3 PSG11 PRSS21 PABPC1L C20orf165 FOSB DAZ1 MSH5 DHH CCDC63 RNF8 PACRG PVRL2 PTPRN UBE2A CCNB1 MSH6 MLH3 OPRL1 IMMP2L AFP ZPBP DEFB118 TXNDC8 PRDM9 SRD5A1 DRD1 HMGB2 CYP19A1 IRG1 FOSL1 SPDYA CCNB3 IL12B BBS2 CATSPER2 BAG6 ZNF35 GLRA1 PLCB1 SLIT2 UCP2 GNAQ PLCD3 TMF1 ESRRB FUT10 TEX14 BCL6 TIMP4 CHD7 EMP2 RAD51B RSPH1 CITED1 DDX25 UBE3A YY1 TEAD3 RXRA PRSS42 SPO11 PSG3 APITD1 SLC4A2 ODF2 PSG6 TUBGCP4 CDY1B EXD1 H2AFX APOB KRT19 ARID4A ZFP36L1 SPACA7 NTRK1 MSH2 CLIC5 GOPC CLDN4 BCAP31 SPESP1 RAD50 LEF1 PAQR8 PKDREJ KIAA1524 DSG2 RNASE10 OCA2 RAD23B ETNK2 TBC1D20 PSMD13 TMEM48 RAD54B PDE3A SPATA20 HFM1 CCDC79 CYLC2 HAS1 FAM75D4 STRA6 WIPF3 TAC3 PRKACA TSPY1 ZAN SLC38A2 NBN MAK GHSR ZGLP1 DMRT3 KHDRBS3 SYT8 TRO KISS1 CASP2 ITGB1 TDRD1 PRM2 MAGED2 UTF1 SPA17 MYH9 MORN2 STAT5B QKI TSSK1B AGRP DMRTA1 SMC1A NPAS1 BMPR2 DIAPH3 INSL3 NUMA1 C17orf87 LHB ATP1A4 HS6ST1 TRIM27 NIPBL TSPY10 NR5A1 CCT5 SLC38A3 ACTR2 GAB1 JUNB CYP7B1 RBMY1J RPL39L SOS1 OXT C9orf79 TMEM203 DDX20 C19orf20 SLIT3 SPIN1 COL16A1 REN DDX4 PPARG CSDE1 ESR1 PBX1 TIPARP IFT81 PSG9 MED1 PAIP2 CDK1 RDH10 PTGIS ST14 FIGLA PIWIL2 SMC3 SMC4 NOBOX HIST1H1T FZD4 FSTL3 UPRT ACRBP STC2 MAEL DAZ4 TSNAXIP1 UBE2J1 DPY19L2P2 ANTXR2 CCIN MERTK PLEKHA1 NRK EGR1 ID4 MAFF SCXB PTK2B SOX9 NOS3 C9orf11 PATZ1 HOXD10 C15orf60 ATP2B4 ROS1 CYP1A1 CELF4 MLL2 PTN SPP1 NEURL TFCP2L1 PIM2 NSDHL SPIN4 ZNF318 KRT9 PRM3 ADNP EDDM3A RAD21 RHBDD1 PSG2 BMPR1A GAMT STAG2 BIRC3 BRD2 FGF10 LGR5 DMRTB1 ETV2 LIF TNFAIP6 HIST1H2BA HERC2 AVP TRIP13 STX2 FOXL2 AKAP3 RAMP2 PRKAR1A EME1 CAPZA3 RHOXF1 FBXW8 CYR61 SNAI1 ASF1B DLG1 RNF38 FNDC3A ELL3 HORMAD2 C10orf125 RPS6 TSPY3 MMP14 RLN1 HEY1 CCNI TLR5 SPATA9 ROR2 SYCE1 HNF4A MLH1 FAM75A5 CDY2A ROBO2 MGST1 TGFBR2 TSGA10 SERPINB5 OSR1 LHCGR TOP3A AURKC ACVR2A BPY2C DUSP13 DEFB1 KIAA0430 DHCR24 DMRT1 OR2H2 HSD17B2 CHD5 BOK TFEB TCF7 GALNTL5 PTGFR AKT1 BCL2L10 SPAG4 WNT2B FAM75A7 PMCH TCFL5 MMP2 C5orf32 RBBP8 PRLR CELF3 FAM9B CLGN ANG DSG1 EGFR TUBD1 CALR3 CYLC1 RBMY1B SMARCA2 SPINT2 TUBGCP6 SIX4 PRKAG1 UBE2Q1 TRPC6 WDR48 T SRD5A2 ITGA2 SFMBT1 THBD PICK1 LRP6 HSD17B4 TIAL1 LHX9 ZBTB16 CCT4 LEPR GMCL1 ALPL RBMY1F HOOK1 WDR81 ERCC1 ICAM1 RACGAP1 RAD51C GJA10 KIAA0196 TRPC3 BAX STAT3 YBX2 AKR1B1 FOS TAF7L MAPK1 RPS6KB1 DAZL ASPM PTTG1 TRPM2 RPL29 EIF4H PNOC CIB1 SPATA22 PAFAH1B3 BMPR1B GATA4 ASCL2 NUPR1 FBXO5 MAPK8IP2 GRB2 CEBPA EGLN1 UTP14C C10orf27 THRB BCL2 ATAT1 DMC1 PLCZ1 TDRD6 HSP90AB1 PLD6 BTG1 VGF SFRP1 TCP1 ARID1A NPPA WNT9B SPATA6 KLF17 TESK2 NCOA1 CSF1 RFX2 MAP3K4 LIN28A TRPC7 ANXA1 KDM3A TH RARG HAS2 WEE2 CNTFR NUP210L EOMES PUM1 ACTR3 SPATA19 SYCP1 CNTD1 WFDC2 CITED2 INPP5B FANCG NOG SPACA1 PLCD4 ZP1 INHA SRY PRDM1 TLR3 OR7C1 CENPI ODF1 BPTF KCNU1 MAP2K1 GABRB1 CDKN1C UMODL1 FAM50A SMARCC1 CAD HIST1H1A NR2C2 STK11 CYP26B1 MEIG1 AXL NANOS2 HEY2 PLAC1 FAM9A GRIN1 PTHLH CD46 CRH CTSB CSDA RRM1 RBM7 THRA TSPY8 SMAD1 EIF5A2 BBS4 PARK7 WNT7A MICALCL APOL2 HMX3 E2F1 PRLHR RXFP2 WDR77 TDRD9 HAND1 B4GALT1 CALR MMP23B SRPK1 C22orf28 TEX19 IGFBP2 NPY5R HSF2 PSG5 NKX3-1 SYCE1L TUBGCP5 OOEP RAD51 ING2 PAQR7 PANK2 CDX2 MEN1 PGF SENP2 INHBB PTGS2 XKRY HMGCS1 LEP SETX HILS1 PRDM14 ADCYAP1R1 TUBGCP3 TXNDC2 NUP62 SPEF2 XRCC3 CATSPER1 TEX11 HAVCR2 DPY19L2P1 RNF151 MYCBPAP MMP9 VDR KIFC1 RAD51D CREM SEPP1 CETN2 STK3 WNT5A SOX15 NANOS3 UBR2 TAF4 FKBP4 PSG1 CRHR1 C9orf24 SOHLH1 PPAP2A NRIP1 SERPINE2 CLIC4 HOXA9 GMCL1P1 GATA6 PAQR5 DACH2 EPYC NPM2 CEBPB CDC25C BPY2 SPAG8 ZNF148 SIRT2 WT1 GLI2 LHX8 TOB2 WBP2NL CFTR ODF3 SPACA3 FAM75B TEX12 ARNT SEMG1 REC8 TESC SOX17 CDYL CD38 LHX3 SLIRP GPR149 BAK1 H1FOO KLHL10 DEFB126 E2F7 MYCBP HFE CSF2 AZI1 IQCG DAZAP1 LGALS9 EPAS1 MEA1 ADAM28 STS OVOL2 FAM75D1 ENDOU ADAM20 SPIN2A WNT4 SPATA5 FZD5 MKI67 SYCE3 ADCY10 C5orf41 PIWIL3 PSME4 KDM2B GATA3 IZUMO1 FGFR2 RGS2 HSF1 ADAM15 CST3 CCT2 DYNLL1 FOXJ1 CYP27B1 ITGA3 VEGFA HOXD13 IL4R GOLGA3 ADCY7 PAPPA TSSK2 NKAPL ADC AMHR2 IGF2R H3F3A CDY1 PI3 KDM5A PAM BSPH1 RBM15 RSPO3 THEG OVGP1 SLCO4C1 TDRD12 CRHBP ZP2 GDF9 CCR6 PKD2 MDFI SRC SYCP3 TNP2 DAZ3 NME5 TSSK3 DNMT3L NODAL UBXN8 COMT TGFB2 PCSK4 DRD5 KDM1B WAPAL AMH TXNRD3 SBF1 ALOX15B PZP SPIN2B SPTBN4 TP63 BPY2B H3F3B MST1R KIT ADAM21 RNF212 MAMLD1 SAFB2 PTGDR FSCN3 MAST2 FOXC1 SETD2 SIX5 OPRK1 MCM9 CTCFL LRMP ETS1 SPANXB1 DEAF1 PRKACG CREB3L4 NR2F2 AREG SCGB1A1 MSH4 FGF9 KLK14 SGOL1 TGFB3 STOX2 LNPEP WDR33 GJB2 FOXA1 OPRM1 GDF7 HES1 ACSBG2 CD9 TUBGCP2 CELF1 SOX30 INSL4 CTDNEP1 EREG DDO USP9X SYT6 RAD21L1 EDN1 ADM C11orf80 ETV5 ARRB2 FAM75A3 CCL2 PIAS1 RNF2 FANCM ADAM30 CASP8 SGOL2 CDC25B OVOL1 CCNF UCN PTK2 ACVR1B HSD11B2 RARA PLAG1 GGNBP1 KDM5B CTNNB1 SPANXA2-OT1 SALL1 IL10 TDRKH PRDX3 SSTR1 MTL5 HUS1B TSSK4 H1FNT STRA8 FBLN1 GGNBP2 AGFG1 PCNA SIAH1 ANKRD49 DPEP3 EIF2B2 CCT7 IL4 FANCD2 POU4F2 ADAM2 MMP7 DNAJB6 OR1D2 PYGO2 NHLH2 CCNYL1 KITLG MBD2 LFNG POMZP3 GGT1 ADA CRISP1 BCL2L1 IGSF8 FAM75A1 ZSCAN2 PTTG2 GPR64 CNR1 ANKRD7 ATRX SP3 IGFBP5 TYRO3 ERCC4 PDGFRA PIWIL1 B4GALNT1 TCF21 SF1 PSAP LRGUK NPR2 PGM3 ZFPM2 CKS2 ODF4 TDRD7 GATA1 MAS1 INSRR CDK16 C15orf2 SPAG6 TESK1 GHRHR SYCP2 BRDT TERF1 TARBP2 MEI1 WDR19 SOD1 FAM75C2 WNT7B PPP2R1A SLC9A10 CASC5 NPPC MRE11A NEK2 ADAMTS2 SPATA24 LGR4 DPY19L2 ASTL SPEM1 TBP PAFAH1B1 MASTL IL11RA GLRB ALKBH5 PRMT7 SLC26A3 STRA13 DDX6 DNALI1 DACH1 PSMC3IP SOX8 RPS6KA2 RNF17 PRKDC ACOX1 GPR56 AGT PHLDA2 C12orf55 SUN1 PPP2CA SMC1B PLK4 PLA2G4B ZW10 CDKL2 CYP17A1 TEX15 FMN2 E2F8 BCL2L11 FANCA GTSF1 AR NPFF ABHD2 GCM1 SLC26A8 STC1 CDK2 RAD54L INHBA AAAS NR6A1 SPAG1 SPATA16 PVRL3 ETV6 TTC26 TCP11 MAP2K6 TUBG1 GGN SLC22A16 LRRC6 PTAFR OAZ3 NCAPD3 RNASE9 LCN6 EIF2B4 DHODH RBP4 FOXF2 SCMH1 JAM3 ADAMTS1 GJB5 MSTN RAB13

GO_NEGATIVE_REGULATION_OF_CELL_AGING Any process that decreases the rate, frequency, or extent of cell aging. Cell aging is the progression of the cell from its inception to the end of its lifespan. TWIST1 TERT MIF ZKSCAN3 BCL2L12 TERF2 SIRT1 FZR1 ABL1 WNT1 PTEN BCL6 FOXM1 MARCH5 CDK6 PRKDC

GO_NEGATIVE_T_CELL_SELECTION The process of elimination of immature T cells which react strongly with self-antigens. ZAP70 SHH SPN GLI3 CD74 THEMIS DOCK2 CD28 AIRE FAS CCR7 CD3E

GO_POSITIVE_REGULATION_OF_INTERFERON_GAMMA_PRODUCTION Any process that activates or increases the frequency, rate, or extent of interferon-gamma production. Interferon-gamma is also known as type II interferon. TNF IL21 FADD KLRK1 HLA-DPB1 CD2 TLR3 PYCARD CD244 IL12B LGALS9 IL12RB1 TNFSF4 ABL1 CD3E CD14 IL18R1 CD226 ZP3 HSPD1 IL12RB2 IL23A EBI3 HAVCR2 ZFPM1 PDE4D IL18 CCR2 FZD5 SLAMF1 IFNAR1 IL2 TLR4 TLR9 SASH3 LTA IL27 KLRC4-KLRK1 PTPN22 IL23R RIPK2 HRAS WNT5A TNFRSF13C CEBPG SLAMF6 CD276 BCL3 IL29 PDE4B ISL1 TXK TICAM2 SLC11A1 IL12A IRF8 HLA-DPA1 IL27RA TLR7 TLR8 RASGRP1 IL1B

GO_NEGATIVE_REGULATION_OF_IMMUNE_EFFECTOR_PROCESS Any process that stops, prevents, or reduces the frequency, rate, or extent of an immune effector process. HLX INS CD84 C4BPA TNFSF4 IL7R ITCH FOXF1 THOC1 CUEDC2 JAK3 MICB FER BCR SOCS5 TNF PRKDC TGFB3 CCR2 DAK PTPRC SERPING1 SLAMF1 SEC14L1 C1QBP TRIM38 HTRA1 LILRB1 ANGPT1 ANXA1 CD59 PGLYRP3 IL29 TRAF3IP1 BCL6 HLA-E NDFIP1 FOXJ1 IL4 PGLYRP1 CD55 PTPN6 SPINK5 SERPINB4 IL4R PPM1B DUSP22 PCBP2 PPP3CB IL10 CD300A TARBP2 LGALS9 HMOX1 IL2RA SERPINB9 IFNB1 HFE RABGEF1 DUSP10 IL33 APOA2 XCL1 NLRX1 CD96 HAVCR2 CEACAM1 ABR LGALS3 CR1 RPS19 TSPAN6 APOA1 RC3H1 DHX58 ARRB2 C4BPB IFIT1 TGFB2 IFNA2 IRAK3 SPN A2M TMBIM6 MICA CLEC4G BST2 TNFSF18 PGLYRP2 EIF2AK2 IL13RA2 MUL1 IL20RB FOXP3 TGFB1

GO_GUANOSINE_CONTAINING_COMPOUND_BIOSYNTHETIC_PROCESS The chemical reactions and pathways resulting in the formation of guanosine-containing compounds (guanosines). NME2 NME7 PRTFDC1 TXNDC3 NME2P1 NME9 NME6 IMPDH1 NME5 NME3 IMPDH2 HPRT1 NME1 GUK1 GMPS NME4

GO_POSITIVE_REGULATION_OF_POTASSIUM_ION_TRANSMEMBRANE_TRANSPORT Any process that activates or increases the frequency, rate or extent of potassium ion transmembrane transport. AKAP9 AKAP6 GAL KCNMB1 GALR2 KCNC1 ATP1B2 KCNH2 KCNC2 DNM2 ATP1B1 NPPA KCNE1 EDN3 KCNE1L OPRK1 ACTN2 RNF207 ATP1B3 AMIGO1 KCNQ1 KCNJ2 ANK2 ANO6 NOS1AP

GO_POSITIVE_REGULATION_OF_B_CELL_DIFFERENTIATION Any process that activates or increases the frequency, rate or extent of B cell differentiation. NCKAP1L MMP14 BTK PRDM1 PPP2R3C XBP1 STAT5B SYK INPP5D ATP11C PCID2 BAD CD27 ZAP70

GO_REGULATION_OF_GROWTH Any process that modulates the frequency, rate or extent of the growth of all or part of an organism so that it occurs at its proper speed, either globally or in a specific part of the organism's development. MKKS ITCH TMEM97 PTPRJ MAPT COLQ DRD3 CHPT1 IST1 GDF2 PAK2 SCGB3A1 RAB11A SEMA4G STK11 MTPN KRT17 HEY2 ATRN ING3 RAI1 CSDA BLZF1 BCAR1 BBS4 L1CAM IP6K2 GSK3B ENOX2 MEF2C OSM SYT2 TKT NRP1 LEP RBPJ ARHGAP4 TAF9 WWC2 EBAG9 NKX2-5 USP47 ADIPOR2 SPHK1 POU3F2 RND2 SEMA3E IGFBP2 APBB2 WISP1 MORF4L2 PSMD10 SEMA4F INSR ING2 ZFYVE27 SEMA4D HPN ERBB2 EZR IGFBP6 HNF1B SGPL1 CCDC85B ZNF259 SERTAD3 FGF8 TRPV2 TNF TNR CRYAB SFRP2 GPR21 GPAM SERPINE2 DDR1 PRLH GATA6 ZP3 HTRA1 HRG WT1 HYAL1 HIST1H1B TM7SF4 SESN2 WNT5A BRD8 SOX15 STK3 FLCN PTEN NTN1 APOE RUVBL1 SOCS6 LTBP4 MT1G TNKS2 VPRBP MAP2K5 PLXNA1 WFS1 WRN EXOSC9 PROX1 RNF6 PPARD GREM1 CLSTN3 MBD5 ESM1 SMARCA4 APP RAB33B SLC6A4 MT1H YAP1 HTRA2 SASH3 ADRB1 SOX17 SLC25A33 GH1 FN1 CD38 SIPA1 GDF5 PRSS2 TP53 CAPRIN2 PLXNA4 IGFBPL1 IFNG MT3 ADAM17 IGSF11 CREG1 ENPP1 EGFR BMP10 INS AVPR1A SMARCA2 TWF2 MYOCD HLX SIX4 F2 PPP1R9B SUPV3L1 CRIM1 SEMA6C KCTD11 DIO3 GJD4 SOCS4 PPIB SMAD4 ACTL6A ENO1 NPR1 BARHL2 ZMAT3 TP73 SIRT1 IRF8 AKT1 DACT3 WNT3A DISC1 EPHA7 SPOCK1 DAB2IP IL7 AFG3L2 STAT3 RPS6KB1 CRLF3 CAPN3 GHRL AKAP6 SEMA3F DLL1 CIB1 LATS1 ACVRL1 MYH6 ESR2 BASP1 LIMK1 NOL8 IGFBP4 FHL1 STAT5A PPM1F HEPACAM CTGF MT2A TIRAP RTN4 TGFBR1 ANAPC2 TGFB1 GAL RERG LGI1 WNT11 STK4 BST2 ATP8A2 C6orf108 SOCS5 BTG1 NPPA SFRP1 PAPPA2 RAG2 TBX2 IGF1 CSF1 ISLR2 NOTCH1 TTL IL9R PLXNA3 HIF1A ITSN2 E4F1 SERP1 IL2 SLC44A4 HAMP S100A8 SHC1 GNB2L1 MT1M HMGA2 GHR PPT1 BCL2 C19orf2 PML NDRG3 ERBB4 NRCAM PARK2 WNT2 AGTR1 PTPN11 LMX1A FBLN5 MTOR PHB IGFBP7 C1orf187 PLXND1 NAIF1 ING5 CTDP1 CEACAM1 KCNK2 TRIM40 FGFR1OP CDK11B GJA1 DDX39B NRG1 NCBP1 DNM2 EPC1 LTA PRL SGK3 GAP43 BMP4 MT1F OSGIN2 NDNL2 NOG CDKN2AIP SOCS1 CDKN2D BRMS1L KIF26A PPP1R1C NOV SEMA3G PRAME CDKN1A CDKN2C RPS6KA1 SEMA3C TP53TG5 FSTL4 CRABP2 EXTL3 TAF9B NIPBL KIF14 NDUFS3 IL17RB CAMK2D PLXNC1 S100A9 OSBP RASGRP2 FGF20 MPO IGFBP5 ING1 BNIPL PLAC8 CREB1 NTRK3 MTM1 SGK1 LATS2 N6AMT1 INO80 STAT5B KIAA1967 MT1X BDNF IGFBP3 TSG101 BMPR2 SYT4 RB1 EI24 DERL2 CXCL12 TRIM32 GSK3A HDGFRP2 FXYD2 DDX3X GHRHR NPPB CDK5 SMAD3 DCC CISH PPP2R1A MAEL SOD1 STC2 PIK3CA NPPC EFNA5 SGK2 MYOD1 DSCAM NRK PHF17 SLIT3 H3F3C EXOSC2 CYBA FAM107A SLC6A3 LRP12 CDH4 TAOK2 CDKN1B MT1B ZFPM2 CTTN ELANE PPARG WDR36 CDK1 SIX1 SYT14L H2AFY MORF4L1 MAP1B PLXNA2 SEMA4C PRKDC FGF2 BMPR1A GPC3 AGT TGFBR3 GAMT SOCS7 UBE2E3 CDK5R1 CDKN2A HYAL2 PPP2CA ABL1 ARHGEF11 DUSP6 POR NANOS1 AVP RUVBL2 CSPG5 NDN SEMA5B BCL2L11 MUSK FXN MUC12 TBX20 NME6 PAFAH1B1 PTK2B WWC3 SEMA3B WISP3 TMC8 AGRN MAPK11 TFCP2L1 C8orf44-SGK3 RASAL1 CXCL16 SAV1 SPP1 EGLN2 CD320 SDCBP ST7L ADNP HBEGF FOXK1 OGFR DCBLD2 CSNK2A1 ACTN3 HNF4A ZNF639 NRG3 RPS6KA3 TSPYL2 ILK RBP4 TGFBR2 NEDD9 ADRB3 PTCH1 MSTN HTRA3 NDUFA13 SYT3 GAS6 FRZB AR TMEM8B MYL2 PLCE1 PRKCQ ADIPOR1 CYR61 SEMA6B MT1E INHBA EAF2 LPAR3 SEMA6A MUL1 ADRB2 APBB1 TNK1 CARM1 MMP14 TCHP CACNA2D2 HOXB13 MAPK14 H3F3A LGMN CDK11A GDF9 CDA FOXS1 SEMA3D SMURF1 H2AFY2 RAB21 NKD1 MAD2L2 ING4 KDM2B KIAA1598 TSPYL5 SLC9A1 CAMP IGFBP1 WNT3 CAV3 HSF1 FGFR2 CYP27B1 ADAM15 FLVCR1 CLSTN1 VEGFA MBL2 ACACB CPNE9 BDKRB1 FGFR3 CDKL5 VIL1 AGPAT6 RTN4R SEMA5A LBP CDHR2 CD36 IFRD1 CDC42 EPB41L3 WFDC1 PLS1 H3F3B BBS2 IL12B WISP2 DCUN1D3 DMAP1 NKX6-1 SEMA4B CPNE5 G6PD SOCS2 HTRA4 SFN MEGF8 PLCB1 AGR2 SOCS3 SMO AES TGFB2 EXOSC4 SEMA4A POU1F1 PNPT1 GNG4 MSX1 CCNB1 NET1 TNFRSF12A SEMA7A KAT5 SPTBN4 ALOX15B RBBP7 RIMS2 ULK2 MFI2 ARMC10 BAP1 CELF1 FBP1 SYT1 GHRH MACF1 CTSG TBX5 TRPC5 LEF1 ARX FOXC1 SLIT2 SERTAD2 AGTR2 PSRC1 PIN1 OLFM1 KAZALD1 CHD7 RUFY3 BCL6 NEDD4L CPNE6 FOXC2 ADAM10 MT1A PTK6 DPYSL2 IL9 FGFR1 SQSTM1 FGF9 OSGIN1 UCN ACVR1B PTK2 NPY1R SRF UTS2R SH3BP4 DRD2 RIMS1 SPG20 GHSR SEMA6D IL10 MCTS1 C20orf20 FTO TRO ALOX12 HOPX TFRC ADNP2 STRA8 SYT17 EDN1 GOLGA4 NDEL1 UNC13A WWC1 FGF13 TLL2 YEATS4 KIAA1109 SEMA3A

GO_INFLAMMATORY_RESPONSE_TO_ANTIGENIC_STIMULUS An inflammatory response to an antigenic stimulus, which can be include any number of T cell or B cell epitopes. IL2RA IL31RA SERPINC1 CXCR2 HMGB1 RBPJ NPFF TNF HMGB2 PNMA1 OPRM1 GATA3 CYSLTR1 CD6 ELANE NOTCH1 IL25 AHCY IL20RB KDM6B IL5RA RASGRP1 ICAM1

GO_REGULATION_OF_STEROL_TRANSPORT Any process that modulates the frequency, rate or extent of the directed movement of sterols into, out of or within a cell, or between cells, by means of some agent such as a transporter or pore. ABCA1 APOA1 ABCA2 APOC3 APOA4 ABCA12 LAMTOR1 SCP2 NR1H3 ADIPOQ APOE PPARG SIRT1 SREBF2 EGF NR1H2 APOC2 NFKBIA TSPO SHH ABCB4 NFKB1 ABCG1 LEP PON1 APOA5 APOA2 CETP PTCH1 LIPG ABCA7 PLA2G10 ABCG5 ABCG8 APOC1 NUS1 LRP1 PLTP

GO_REGULATION_OF_CELL_ADHESION Any process that modulates the frequency, rate or extent of attachment of a cell to another cell or to the extracellular matrix. DACT2 KIAA0922 SPACA7 MUC1 HES1 FOXA1 GRAP2 MUC21 PAWR MFI2 FLOT1 MACF1 SLAMF1 LEF1 ACTN4 PDCD1 CD24 ACER2 RAC3 IQGAP1 LAT FOXC2 ETS1 ADAM10 BCL6 HLA-E TNFSF9 EMP2 RELL2 POSTN PPP1CB TMEM102 DUSP3 SCGB1A1 TNFSF13B CD247 CD83 TGM2 DROSHA NID1 ROCK2 ADIPOQ DUSP22 TNFSF11 CD28 CTNNB1 PLA2G2F SRF IL10 SWAP70 CD80 PTK2 RARA IFNB1 THBS1 TFE3 IL2RA ABL2 ARHGDIG ALOX12 TNFRSF18 IL18 TFRC SERPINE1 FBLN1 PDPK1 DOCK1 CD6 PLXNB1 EFNB3 RNASE10 CCL2 MIP PTPRG IL8 DHPS TEK DAPK3 SHH JAK3 UNC13D FAF1 ZNF703 APBB1IP GBP1 SRC BCL10 WDPCP GSN ANGPT1 PLXNB3 BRAF BMP2 GATA3 EPHA1 ATXN3 SLC46A2 LGALS1 ARHGEF7 PPAP2B FOXJ1 ADAM15 MAD2L2 IHH SLC9A1 SDC4 CDH13 SEMA5A IL4R IRF1 VNN1 RASGRP1 CD36 ERBB3 PRNP ITGA3 CCL28 VEGFA LAG3 OLFM4 IL12B PHLDB2 CCL21 CD300A CAV1 FOXA2 CDC42 FOXN1 ZAP70 NOD2 PDCD1LG2 PYCARD SKAP1 FAM21C AGR2 LDB1 ANK3 AP3D1 ASS1 HLA-DQB2 SIT1 LGALS3 RAG1 ABI3BP TGFB2 HLA-DRB4 ITGAV WNT1 FGA COL8A1 TRAF6 HLA-DOA NR4A3 RASA1 RICTOR PPARA NODAL TMIGD2 ZNF683 LYN HLA-DPA1 CLEC4G EPB41L4B DUSP26 ITGB1BP1 HLA-DRB1 EFNA1 NET1 SNAI2 FLOT2 NUAK1 FGB CLASP1 PPP2CA ABL1 CDKN2A KDR GPR56 ONECUT1 THY1 DLC1 PTPRC FANCA CCR2 AP1AR LAMA5 PTPN2 TIGIT JAM2 SOX9 ECM2 PRKCE ITGA5 HLA-DMB PTN IL29 PLEKHA2 CTLA4 SPTA1 CD1D ICOS PTPN6 PTK2B GPM6B HMGB1 CD55 PRELID1 CCL25 JAK2 CD5 PRKCZ APOD CCDC80 NKAP PAK1 IL12RB1 PELI1 CSK SMAD7 CHRD KIFAP3 PTAFR SYK PDE3B AP3B1 ILK EGFL6 MLST8 BTN2A2 FES TGFBR2 HOXA7 SMOC2 PRKAR1A CD40LG PRKCQ PRR5 IGF2 PRKD2 CYR61 FBLN2 CCL19 PAK3 DNAJA3 TSC1 MMP14 CASK DLG1 ADAM22 MYF5 RREB1 HLA-DQA1 SLK HLA-DRB5 CXCL13 ADAM9 RND1 KIF14 ZBTB7B NDNF SOS1 GLI3 IL4 FANCD2 KANK1 STAT5B TNFRSF13C MINK1 EFNB1 IL28B ADAMDEC1 ARHGDIB CD209 GLMN MAPK7 CD3G CCDC88B ADA SPINK5 EFNB2 ARPC2 ARHGAP6 CXCL12 FZD4 LRRC32 TNFSF14 XBP1 FSTL3 CD244 SOD1 VCAM1 PPP2R1A NFKBID CD4 STX4 IL21 CD44 SMAD3 DSCAM PRKCD LILRB2 EGFLAM PRKG1 XCL1 PIK3CA COL1A1 EFNA5 TGFBI PKP4 COL16A1 CORO1C NINJ1 APOA1 IL27 BAG4 SIRPG TRPV4 NPY2R LMO1 PTPN22 IFT74 HLA-DQB1 ELANE C11orf34 TNFSF4 VTN CSF1 UBASH3B IGF1 STX3 SOCS5 FERMT3 SFRP1 ONECUT2 IL7R HAS2 IL2 FYN CORO1A NOTCH1 ANXA1 S1PR1 LILRB1 HLA-DQA2 MALT1 PIK3R1 GRB2 KLRC4-KLRK1 RASAL3 PIK3CG MAP3K14 LRRC16A PML TNFRSF14 SAA1 BCL2 VPS33B TRBC1 DPP4 NRARP TRIOBP SOX2 CYTH1 HSPH1 CELSR2 EGR3 DMP1 VIT MTOR PIEZO1 PTPN11 GCNT2 IL6 RSU1 MYOC VSIG4 CEACAM1 BMP4 SERPINI1 PTPN23 CITED2 RC3H1 SHB ADAM8 RAC1 DNM2 C20orf160 MAP3K8 CLASP2 PNP PRDM1 EPHB3 ITPKB CD3D MAD1L1 ITGA6 KNG1 PRKCA LMO7 HLX CD3E SPINT2 PRLR TBCD FADD FOXF1 PLA2G2D ITGA2 NF1 CYTH3 ARHGDIA BMP7 ABAT CLPTM1 IRF4 IL23R ICOSLG BAD YES1 CD164 ALOX15 EPB49 AGER DISC1 WNT3A EPHA2 FZD7 AKT1 TACSTD2 ZP4 NF2 CD47 TRAC PLAU EPHA7 EDIL3 LAX1 IL7 HLA-DPB1 SPOCK1 NLRP3 BTLA SPOCK2 ACVRL1 PTPRO FAM116A CLDN7 VAV1 CIB1 HSD17B12 EPHA3 DLL1 FGG PPM1F PODXL IL15 ANGPT2 HLA-DRA PLG ZBTB16 ICAM1 FXYD5 TGFB1 TRAF2 LAMA2 LAMA3 GPAM SERPINE2 CLC CCR7 DDR1 IL6ST TNR TNF ZEB1 PIK3R6 GNRH1 SFRP2 MYB MIA3 RAC2 GLI2 IL23A HRG EBI3 B4GALNT2 HYAL1 CD59 ZP3 HLA-DRB3 VEGFC CEBPB RET AZU1 KIF26B ZNF645 C1QBP DAB1 WNT5A NCKAP1L PTEN PKD1 HHLA2 FLCN FMN1 CD74 PLXNB2 MAP2K5 EPHA8 RHOD IL1B ARL2 ZBTB1 SOCS6 GREM1 LGALS9 CAMK4 VAV3 C1QTNF1 HFE CBLL1 FLNA BCAS3 EPO TNFRSF21 PAG1 SOX13 HSPD1 WNT4 CD276 CX3CL1 SIPA1 VWC2 JAG2 MYADM CD274 C13orf15 PRSS2 GTPBP4 SASH3 FN1 TESC SPN IFNA2 RCC2 ROCK1 KIAA0748 IFNG FOXP3 TRBV12-3 IL20RB PRKX LIMS1 CYTIP DTX1 EPCAM PDE5A LAMA4 AIF1 CCL5 ADAMTS18 LPXN SART1 CYP1B1 PTPRJ KLRK1 CD2 PPP1R12A CALR ITGA4 PAK2 IL1RN TNC LAMA1 ZC3H8 NCK2 NDFIP1 MAP3K7 CYP26B1 KLF4 GSK3B RIPK3 CDH1 NPNT RDX TPM1 CD46 CRKL IL12A ATP5B CRK IL28A IL1RL2 IDO1 HLA-G CDK6 LEP HAVCR2 EMID2 SERPINF2 SEMA3E MAPKAP1 LCK MARCH7 PIK3CB TNFAIP8L2 ERBB2 VTCN1 SEMA4D EPB41L5 IGFBP2 CARD11 DOCK5 PREX1 NCK1 UTRN RIPK2 SFTPD TNFSF18 PTPRR S100A10 CD86 MEN1 IL36B

GO_BEHAVIORAL_RESPONSE_TO_COCAINE Any process that results in a change in the behavior of an organism as a result of a cocaine stimulus. DRD3 DRD2 DRD4 SDK1 DRD1 ABAT HTR2A CDK5 KALRN HOMER1 SNCA OPRK1 HOMER2

GO_STRIATED_MUSCLE_ADAPTATION Any process in which striated muscle adapts, with consequent modifications to structural and/or functional phenotypes, in response to a stimulus. Stimuli include contractile activity, loading conditions, substrate supply, and environmental factors. These adaptive events occur in both muscle fibers and associated structures (motoneurons and capillaries), and they involve alterations in regulatory mechanisms, contractile properties and metabolic capacities. CFLAR TRIM63 NPPA GATA6 IL15 ACTN3 HEY2 RPS6KB1 INPP5F PPP3CA PPARGC1A MYH7 ATP2A2 KLF15 GSN HDAC4 MYOG ACTA1 MYOC CAMTA2 EZH2 MEF2C MSTN MYH6 MYOD1 TCAP KDM4A

GO_NECROTIC_CELL_DEATH A type of cell death that is morphologically characterized by an increasingly translucent cytoplasm, swelling of organelles, minor ultrastructural modifications of the nucleus (specifically, dilatation of the nuclear membrane and condensation of chromatin into small, irregular, circumscribed patches) and increased cell volume (oncosis), culminating in the disruption of the plasma membrane and subsequent loss of intracellular contents. Necrotic cells do not fragment into discrete corpses as their apoptotic counterparts do. Moreover, their nuclei remain intact and can aggregate and accumulate in necrotic tissues. TRAF2 TICAM2 MAP3K5 PPIF TRPM7 CASP1 RIPK1 TMEM123 TICAM1 SIRT2 RIPK3 LY96 CYLD FAS MLKL FASLG TLR4 TNF IRF3 ALKBH7 FADD TLR3 PYGL BAX BIRC2 DNM1L CD14 PGAM5

GO_POSITIVE_REGULATION_OF_THYMOCYTE_AGGREGATION Any process that activates or increases the frequency, rate or extent of thymocyte aggregation. VNN1 GLI2 ADA KIAA0748 RASGRP1 EGR3 FOXP3 SHH IHH ADAM8 IL7R

GO_DETECTION_OF_LIGHT_STIMULUS The series of events in which a light stimulus (in the form of photons) is received and converted into a molecular signal. RGS9BP RPE65 PDE6C OPN1SW CCDC66 GJA10 ATP8A2 AIPL1 RHO GNB1 RRH ACCN1 EYS OPN1MW2 ABCA4 GNAQ GNA11 PDE6A GUCY2F PITPNM1 PDE6B CNGB1 RCVRN SAG GUCA1A UNC119 OPN4 PLEKHB1 RGR BEST1 OPN3 GNAT2 GNGT1 SEMA5B GUCA1B PDC CACNA1F RP1 CACNA2D4 GRM6 ELOVL4 RDH11 GNGT2 NR2E3 OPN5 GNAT1 SDR16C5 ARRB1 TULP1 RS1 CDS1 PDE6G CNGA1 GRK1 TRPC3 OPN1LW OPN1MW CABP4

GO_AMINOGLYCAN_BIOSYNTHETIC_PROCESS The chemical reactions and pathways resulting in the formation of aminoglycans, any polymer containing amino groups that consists of more than about 10 monosaccharide residues joined to each other by glycosidic linkages. GPC5 B3GNT8 DSE EXT2 CHST3 B3GNT4 BGN B4GALT7 CHSY1 CHST6 ABCC5 EXTL3 SDC1 CHST5 XYLT2 EXT1 B3GNT3 OMD B3GALT6 SDC3 HAS1 GPC6 CSGALNACT2 CHST2 B3GNT2 SLC35D2 ST3GAL4 ST3GAL6 OGN KERA HS3ST5 CHST9 CHPF2 CHST14 CHSY3 NDST3 VCAN HS3ST1 GPC2 GLCE HS6ST2 GCNT2 HS3ST2 EXTL2 GPC4 DSEL B3GAT3 BCAN SDC2 DCN UGDH CHST11 CHPF PRELP B3GAT2 SLC35D1 NDST4 AGRN B3GNT7 UST LUM NDST2 HS3ST3B1 NDST1 HEXA B3GNT1 SDC4 HS6ST1 HS3ST6 CHST1 IL1B ST3GAL2 HS3ST3A1 NCAN CHST12 ACPL2 B4GALT2 KIAA1199 PDGFRB HS2ST1 CYTL1 XYLT1 B4GALT4 CHST7 B3GAT1 B4GALT3 HSPG2 HAS3 B4GALT5 ACAN EXTL1 GPC3 HAS2 B4GALT1 CHST13 HYAL1 GPC1 ST3GAL3 CSPG4 FMOD B4GALT6 CHST15 GALNT5 ANGPT1 CSPG5 CSGALNACT1 ST3GAL1

GO_CHONDROITIN_SULFATE_CATABOLIC_PROCESS The chemical reactions and pathways resulting in the breakdown of chondroitin sulfate, any member of a group of 10-60 kDa glycosaminoglycans, widely distributed in cartilage and other mammalian connective tissues, the repeat units of which consist of beta-(1,4)-linked D-glucuronyl beta-(1,3)-N-acetyl-D-galactosamine sulfate. HYAL4 BCAN NCAN HYAL1 CSPG4 BGN VCAN IDUA DCN HEXB ARSB CSPG5 IDS HEXA

GO_ETHER_METABOLIC_PROCESS The chemical reactions and pathways involving organic ethers, any anhydride of the general formula R1-O-R2, formed between two identical or nonidentical organic hydroxy compounds. TXN2 TXNDC8 FAR1 TXNDC2 CYP2W1 TMEM86B TXNL1 AGMO ALOX12 PEX7 AGPS TXN GNPAT

GO_INSEMINATION The introduction of semen or sperm into the genital tract of a female. P2RX1 SERPINE2 AVPR1A DDO EDDM3A KLK14 SEMG1 ACVR2A SLC6A4 TAC1 OXT OXTR

GO_CALCIUM_ION_TRANSMEMBRANE_TRANSPORT A process in which a calcium ion is transported from one side of a membrane to the other by means of some agent such as a transporter or pore. FKBP1B MCU NCS1 TRPM4 CATSPER3 SLC24A2 SLC24A6 CACNA1H SLC8A1 CACNA1G SLC24A1 TRPV1 ACCN2 C9orf7 GRIN1 CACNA1D NALCN CACNG4 TRPC1 DDIT3 PKD1L3 TRPC4 DENND5A IBTK NOL3 CCDC90A MICU1 PKD2 ATP2A2 F2R TRPC6 ORAI3 CCR5 TRPM7 PKD2L2 CACNA1I ERO1L RYR1 TRPA1 CACNA2D2 IL1RAPL1 CACNA2D3 TRPC4AP GRIN3A CACNG5 ITPR2 ATP2B2 ITGAV ANO6 HTR2A ATP2C1 HTR1E PKD2L1 CACNG2 TRPV4 CACNB2 XCL1 CACNA2D4 TMC1 CACNA1F TRPM2 LCK MCOLN2 CATSPER1 JPH2 CACNA1C PDE2A TRPV5 SLC24A5 PSEN1 CCL21 TRPV6 TPCN2 TRPM6 CATSPER2 CLCA3P TRPC3 EFHA1 TRPM1 CACNG6 TRPM8 CATSPER4 GRIN3B FKBP1A ITPR3 CACNG8 TMC2 PKD1L1 ORAI1 ATP2B4 CHRNA10 GRIN2A GPM6A ATP2B1 PRKCE PKD1 P2RX7 CCDC109B ATP2C2 TMCO1 ATP2A1 TRPV3 ATP2A3 CACNA2D1 HTR2C PTPRC TRPC5 ZP3 ATP2B3 ORAI2 JPH3 CACNB4 STIM2 CACNG1 MCOLN3 CCR7 DENND5B OPRM1 HTR1B TRPC7 CACNB1 CHERP CACNA1B TRPV2 SLC8A3 FGF2 LOXHD1 CACNG3 CACNA1S RYR2 TPCN1 TRPM3 ITPR1 CALHM1 TRDN CACNA1A PANX1 RYR3 HTR1D GRM7 C22orf32 TMEM37 SLC24A4 CACNA1E RASA3 PKDREJ CCL19 CHRNA9 CUL5 SLC25A23 MCOLN1 PLCG2 SLC25A25 GAS6 CACNB3 HTR1F CACNG7 DRD2 TRPM5 HTR2B SLC24A3 PKD1L2 P2RY12 CCL3 SLC8A2

GO_POSITIVE_REGULATION_OF_BONE_REMODELING Any process that activates or increases the frequency, rate or extent of bone remodeling. CA2 TFRC MC4R EGFR ATP6AP1 TNFSF11 TM7SF4 SYK FSHB ADAM8 TMEM64 SPP1 PPARGC1B

GO_REGULATION_OF_T_CELL_MEDIATED_IMMUNITY Any process that modulates the frequency, rate, or extent of T cell mediated immunity. LILRB1 CEACAM1 HSPD1 IL6 STX7 ZP3 XCL1 SLAMF1 FZD5 PTPRC IL23A HFE IFNB1 NLRP3 IL7R FADD PVR B2M CLC IL12RB1 TNFSF4 IL12B DUSP22 IL12A WAS FOXP3 IL20RB TRAF2 PPP3CB ZBTB1 IL1B CLEC4G AGER PRKCZ RSAD2 RIPK3 TRPM4 SPN IL23R NCR3 HMGB1 IFNA2 MAP3K7 SASH3 HLA-E TRAF6 P2RX7 GATA3 MALT1 PVRL2

GO_NEGATIVE_REGULATION_OF_INTRACELLULAR_SIGNAL_TRANSDUCTION Any process that stops, prevents or reduces the frequency, rate or extent of intracellular signal transduction. PTPN6 TMEM161A PDX1 GATSL2 PAFAH1B1 RASAL2 PTEN P2RX7 SFRP5 FLCN TNFAIP8L1 ATP2B4 SESN2 DAB1 WWC3 RIPK1 PODNL1 DDIT4 ARHGEF2 UBR2 AKT1S1 TNIP1 TANK RASSF2 APOE RASAL1 SOCS6 IL1B DUSP19 FIGNL1 WFS1 CD74 LRRC4C BAG5 SFRP2 LRRC19 GPD1L TMEM127 TNFAIP3 GATSL3 AMBP HYAL2 ABL1 PPP2CA SERPINE2 F2RL1 LRRC15 SOCS7 C1QBP PPIF LIF CREB3L1 DUSP6 PTPN2 MGRN1 ERRFI1 LRRC4B SEC14L1 DLC1 ZNF675 TIMP2 DACT1 LITAF SPRY4 LMBRD1 DAG1 BGN MYADM DHX58 ADIPOR1 ATM SNAI1 MFN2 NPRL3 CASQ2 TRDN FLRT1 DLG1 HSPB1 MUL1 LEMD2 TSC1 ITPR1 ELL3 OPA1 CNKSR3 TNK1 GSTO1 PER1 DEPDC5 CLIC2 MCL1 TRIM59 CSK DUSP14 SPRED1 MAPKAPK5 ACTN3 DUSP1 CUL3 RORA ATF3 SPINK1 BCL2L2 CHP OPTN HTRA2 NDUFA13 GCG PLAUR PYDC2 MSTN PDE3B EPO CDC34 HYOU1 SCAI RRM2B CARD8 KLF4 DDIT3 GBA PDCD4 STK11 MTM1 DUSP16 RHOH CLU C9orf89 KANK1 GRINA RRN3 GPX1 GPS2 KIAA1967 PPP2CB CXCL12 RELA CALM2 RPS6KA6 ZC3H12A NLRP12 CSDA PARK7 DDX3X FBXW11 MAPK7 TRIM32 C1QL4 GSK3A TSC2 PTPRJ CREB3 ITCH BCL2L1 NDUFS3 RASA2 ARHGAP42 FOXM1 HIPK3 DRD3 PLEK SESN1 DYRK1A RASA4 LRRTM3 FLRT2 RGS14 XDH HGS DAK PIBF1 SIRT3 PSMD10 GPS1 PTPN22 IRAK3 NLRP6 PDCD6 LRRC4 TSPAN6 CCDC22 WNK2 PARL CDK5RAP3 ING2 NONO MEN1 ESR1 CRTC3 INPP5F MARVELD3 EZR NFE2L2 PTPRR NOC2L PRKAA2 SENP2 PINK1 DUSP10 RABGEF1 CD44 PTTG1IP PPP2R1A NFKBID GSTP1 WWC2 XBP1 CPNE1 SPRED2 TAF9 CHAD TXNDC3 CISH LRRK2 USP47 DCN TPT1 RTN4RL2 SESN3 NUP62 TIMP3 PAQR3 EIF3A RAPGEF1 MMP9 PLEKHA1 MAPKAP1 TWIST1 PRKCD DUSP21 PIN1 ASH1L RASAL3 DUSP3 GNB2L1 PKHD1 MYC KDM1A SLIT2 TLR4 BCL6 RNF152 DEPTOR RTN4RL1 NYX MAGI2 PHLPP1 LRTM1 C19orf2 DUSP22 BDKRB2 PODN NPRL2 BCL2 VRK3 ADIPOQ STMN3 SPRY2 IVNS1ABP C3orf33 PARK2 CYLD SYVN1 FKBP1A SFRP1 SELS SOCS5 MUC1 ARRB1 PRKCDBP TBC1D7 LRRC66 TRIAP1 JUB DUSP7 NDRG2 TAOK3 FBP1 NR1H4 TLE1 OTUD7A DUSP18 DUSP2 RGS3 ERBB2IP ARAP3 INPP5K GPER HIF1A SYNGAP1 PRKAA1 RASA3 TMBIM6 WWC1 SOCS1 CDKN2D DNAJA1 DUSP9 SRI BMP4 TNFAIP1 IL1RL1 ARRB2 CASP8 NLRC3 NOV MDM2 TAF9B UBR1 THBS1 RASA4B LEPROT PHLDA3 ARHGAP35 IL10 HSPH1 MAPK8IP1 LRRTM1 SH3BP4 CALM3 DRD2 PHB NCOR1 TRIM67 MYOC HSPA1A SYNJ2BP STK38 FBLN1 ITGB1 TRIM40 ARNTL FOXO1 WTIP SOD2 SERPINB3 HERPUD1 KCTD13 TBC1D10C VDAC2 PGAP2 SMAD4 RGS2 MBIP SOCS4 CAV3 STAT1 RFFL AKT1 LIMD1 SIRT1 BCL2L10 G3BP2 NF2 FKTN PRNP EPHA2 ITGA3 TP73 DUSP4 TXNDC12 ZNF385A BCL2L12 BANK1 PPM1B FKBP1B VNN1 RTN4R STAMBP FAF1 PBK RGS4 PPEF2 INS DUSP8 SORL1 USP10 CXCR7 GSTM2 BMP7 ADRA1A RANBP9 CALM1 PKD2 GPR97 MAGEA3 GBP1 SRC HDAC7 MIF NOL3 NF1 RNF34 CRYBA1 RASA1 NME5 IGF1R SOCS3 RNF149 HMGCR OTUD7B IGBP1 C1QTNF3 TGFB2 GP1BA SMPD1 FLRT3 SNAI2 TNIP3 PIK3IP1 TRAP1 UCHL1 UBE2B ITGB1BP1 DUSP26 PPM1A SPRY1 LYN PYCARD DAB2IP NLRP3 LAX1 LMO3 EPM2A MECOM DUSP5 ASPN AIDA PTPN1 CD300A CAV1 NFKBIA SLC9A3R1 FNIP1 CIB1 CBLC SLA2 NEUROD1 LRRTM4 ZMYND11 NLRX1 HDAC3 SOCS2

GO_REGULATION_OF_NEURON_MIGRATION Any process that modulates the frequency, rate or extent of neuron migration. ULK4 RAPGEF2 RELN NKX6-1 COL3A1 SRGAP2P1 ZSWIM6 NELF GPR56 STAT3 KIAA1598 GNRH1 DAB2IP KIF20B SCRT1 SRGAP2 PLXNB2 UNC5D NRG1 ERBB4 SEMA3A GPR173 SEMA6A FLRT2 NRG3 SOX14 SCRT2

GO_RETINOL_METABOLIC_PROCESS The chemical reactions and pathways involving retinol, one of the three compounds that makes up vitamin A. ADH7 ALDH1A1 DGAT2 ALDH1A2 DGAT1 RETSAT RDH14 PNPLA4 RDH12 TTR CYP1B1 PLB1 DHRS3 ADH4 DHRS4 AKR1C3 AWAT2 ALDH1A3 RPE65 LRAT RDH8 RDH13 RDH10 BCMO1 DHRS9 RDH11 RBP4 SDR16C5 RDH5

GO_CALCIUM_DEPENDENT_CELL_CELL_ADHESION_VIA_PLASMA_MEMBRANE_CELL_ADHESION_MOLECULES The attachment of one cell to another cell via adhesion molecules that require the presence of calcium for the interaction. CDH23 PCDHB2 CDH17 PCDHB11 CDH2 PCDHB9 PCDHB6 PCDHB13 PCDHB3 ATP2C1 PCDHGB4 PCDHB10 PCDHB14 PCDHGC3 DCHS1 DSG1 NLGN1 CDH22 CDH13 ARVCF PCDHB5 CDH16 PCDHB4 PCDH12 JUB PCDHB16

GO_G1_DNA_DAMAGE_CHECKPOINT A cell cycle checkpoint that detects and negatively regulates progression from G1 to S phase in the cell cycle in response to DNA damage. MDM2 TP53 WAC CCNB1 CDKN1A CDK1 CARM1 TFDP2 CNOT2 E2F7 PCBP4 C2orf29 CNOT7 GADD45A PLAGL1 CDKN1B CDK2 ATM RPS27A C13orf15 EP300 CASP2 RPA2 PLK2 SFN PIDD TP63 BAX RPS27L GIGYF2 TP73 TNKS1BP1 CENPJ NPM1 UBC CCND1 UBB PML PLK3 ZNF385A CNOT10 PCNA CNOT6 CNOT4 GML RFWD3 RBL2 FBXO31 CHEK2 PRMT1 CNOT8 E2F4 AURKA CDC25C ARID3A UBA52 BTG2 TFDP1 GTSE1 RQCD1 CNOT3 CNOT6L MDM4 PRKDC SOX4 TRIAP1 CNOT1 E2F1 MUC1 CRADD

GO_REGULATION_OF_GLUCOSE_TRANSPORT Any process that modulates the frequency, rate or extent of glucose transport. Glucose transport is the directed movement of the hexose monosaccharide glucose into, out of or within a cell, or between cells, by means of some agent such as a transporter or pore. CLTCL1 AKT1 PRKAG2 IRS1 ADIPOQ PRKCZ IL1B PRKCI NUP35 NUP98 GSK3A RTN2 C1QTNF2 PIK3R1 NUP107 AKT2 C3 GRB10 NGFR NUP205 NR1H4 NUP133 NUP85 NUP188 PRKAA1 FGF19 INPP5K OSBPL8 ENPP1 HK2 AGT SELS GPC3 NUP214 SLC25A27 MAPK14 RANBP2 TNF NUP88 PIK3R3 OSTN IGF1 MZB1 IRS2 REPIN1 INS NUP155 POM121 FAM132A GCK NUP50 SLC1A2 FGF21 TMEM48 NUP54 NUPL2 NUP160 NFE2L2 TRIB3 TERT CLIP3 NUP37 NUP93 APPL1 LMBRD1 NR4A3 NUP210 AAAS FFAR3 ITLN1 INSR RAP1A SIRT6 NUP153 CAPN10 GCKR ADIPOR2 O3FAR1 PID1 NUP62 PIK3R2 KIAA0528 NUPL1 ASPSCR1 PRKCB RAE1 SEH1L RNASEL PTPN11 TPR LEP RARRES2 CREBL2 SORBS1 PEA15 NUP43 RPS6KB1 POM121C GIP PTH RHOQ ARPP19

GO_GOLGI_RIBBON_FORMATION The formation of a continuous ribbon of interconnected Golgi stacks of flat cisternae. OPTN VAMP4 STX16 VTI1A PRMT5 MYO18A GCC2 GOLGA2 STX6 TMED5 GOLPH3

GO_DETECTION_OF_LIGHT_STIMULUS_INVOLVED_IN_SENSORY_PERCEPTION The series of events in which a light stimulus is received by a cell and converted into a molecular signal as part of the sensory perception of light. CNGB1 TULP1 GUCY2F GNAT2 BEST1 ATP8A2 GJA10 CACNA1F GRM6 CACNA2D4 RGS9BP SEMA5B RPE65 CCDC66 EYS SDR16C5 GNAT1

GO_POSITIVE_REGULATION_OF_CELLULAR_COMPONENT_ORGANIZATION Any process that activates or increases the frequency, rate or extent of a process involved in the formation, arrangement of constituent parts, or disassembly of cell structures, including the plasma membrane and any external encapsulating structures such as the cell wall and cell envelope. ABCB4 LMOD1 MFI2 NTRK1 NEK7 HNMT TADA2A MAPK9 SEPT7 ACTR3C HUWE1 CD63 CCDC165 HIP1 SYNPO2L TRPC5 RAD50 EPGN HSPA1L MACF1 FLOT1 GPER PSRC1 CCDC56 KDM1A SLIT2 GNL3 CPNE6 SGIP1 NEDD4L BCL6 TMEM106B GPM6A PPP3CC LRTM1 MAGI2 PTK6 FGFR1 SLAIN2 METRN STX18 SYNJ1 ABL2 CCP110 WIPF3 TOMM7 LEPROT SWAP70 TAF1 RIMS1 PNKP NBN LOC388630 ACTN2 EGF SRF WASH3P RPA2 ODZ1 MAP2K7 FAM20B ID1 PDPK1 ANKFY1 CYB5R1 ITGB1 RALB FAM115A BCLAF1 ARHGEF16 TLR6 HRK SHOX2 UNC13A TEK PLEKHM2 CUL4A ENPP2 CHD1L MAPK14 PARP1 FAM179B MRGPRE CDC42EP4 MIS18A CNOT1 MFGE8 BRAF PLXNB3 CAPRIN1 GSN SMURF1 NTF3 UBL5 KIAA1598 PIH1D1 KIAA0146 AKAP8L WNT3 EPHA1 CAV3 UBE2C WDR75 DOCK2 CPNE9 GBP5 MSN ERCC2 VIL1 SART3 RALA CD36 WBP2 WASH6P LRRN1 SEMA5A SDC4 FOSL1 PYCARD MARCH5 CCL21 SLITRK2 PFN1 FNIP1 WBP11 CPNE5 WDR61 PLCB1 NBPF3 PTX3 CAND1 ARPC5 MEGF8 SUMO1 RICTOR CHTOP FGA MICALL2 WNT1 GPHA2 IL1RAP CCNB1 TNFRSF12A NLGN2 MSX1 ITGB1BP1 PRDM9 PAF1 FGF2 DPCR1 KDR HAS3 RNF20 SHANK3 ABI2 ABL1 CLASP1 MSX2 ZDHHC8 F2RL1 RUVBL2 APC LIF PSMC6 RBX1 ANKRD32 LRRTM2 PTK2B KCTD17 CAMK2B ZBED3 PTN MIB1 AGRN AKT2 ARF6 SOX9 SPTBN1 HPS4 SLMO1 NKAP PEMT NEURL ADNP LRRC24 DDHD1 CAPG SYK DTWD2 FRMD7 ROBO2 PSMC1 MAPKAPK5 PAK1 FES PSMC2 MLST8 CUL4B SYT3 PLAUR GAS6 TMEM30A SEZ6 PRKD2 IL5 FBXW8 HNF1A LDLRAP1 POLR3A CCK APBB1 UNC13B WIPF1 LRP5 DLG1 LPAR3 MUL1 IDE CNOT2 HCK CD53 SURF4 CCT5 MPV17L2 TRIM27 NIPBL APOC2 ACTR2 PPP3R1 OXT ANK1 NAP1L2 NKD2 LPHN1 FHOD1 PLAGL2 CREB1 SELE NOS1 TF TSG101 BDNF CENPJ PAN3 PPARGC1A SYT4 BMPR2 RB1 TBC1D5 ATG13 ATF1 GSK3A STOM PIWIL2 FLT3LG BECN1 STX4 KCNB1 TULP1 CTBP1 SCYL2 EFNA5 MMP1 LRP4 NGF MAPK3 MERTK ABLIM3 BAG4 DOC2B APOA1 ANO6 MPP7 PDCD5 LMOD2 SF3A2 CDKN1B GTF2H4 COL16A1 PPARG JUN MAP1B BRCA1 MMP3 NFE2L2 BCR NPEPPS ESPL1 RNF31 STX3 ITPKA ATPBD4 IGF1 ENDOG C4A LCP1 VTN RAB27A ARRB1 JDP2 YWHAH AMIGO3 AMBRA1 FYN SKIL HIF1A ITSN2 STUB1 SPIRE1 ZMYND8 CORO1B TRIOBP SEPT9 PPT1 FSCN1 PHB2 PARK2 ADCYAP1 PIEZO1 GPR65 MTOR MLLT11 SREBF1 ARHGAP35 PACSIN1 CCL24 GPRC5B FAM134C MNS1 RAB3GAP2 BMF SYNJ2BP CCT3 CNST TAC1 DNM2 TMED9 SERPINF1 MFF CAMK1 OBFC2B PTPN23 ANKRD1 IL1A APOPT1 EPHB3 CLASP2 SDC1 AKAP8 CRABP2 OSCP1 VCP WASH1 LRTM2 INS KCNN3 PLA2G6 ARAP1 ARF1 BMP7 FAS ARHGDIA BAD TPBG ACTL6A CUL7 SMAD4 CD47 SIRT1 NF2 FNIP2 PIP4K2A TP73 PPM1E SORBS3 FKBP1B WASL NPHP1 DLL1 EPHA3 GHRL SMC5 USP36 SLX4 GTF2F2 HTT YWHAB CCT6A CDH17 PPM1F FGG AMIGO1 EIF5A WNT11 DYNLL2 CUX1 TGFBR1 TGFB1 UBE2B WRAP53 PSMC4 TRPV2 RPS6KA5 RER1 SYNDIG1 CCR7 DNM1L DHX36 PTK7 TNFSF10 C1QBP AZU1 TADA2B FCER1G PPP1R9A ANXA2 RMND1 AURKA PDGFB PCSK9 ZNF205 HRG RPS3 RAC2 LRRC4B MYB TM7SF4 BMP5 HIST1H1B CCT8 P2RX7 LMAN1 CIDEB NCKAP1L RANBP1 TOR1A BAIAP2L2 TNKS2 BIK PLK5 STAP1 RUVBL1 RAB8B SMCR7 ARL2 IL1B DAB2 PLXNB2 PROX1 OXTR KIRREL RNASE2 MECP2 WASF2 PIP4K2C CLSTN3 PSMC3 TRIM28 GREM1 GTF2H1 FBXO7 MITF NCKAP1 GCG UBE2N EPO TREM2 BCAS3 ATM C13orf15 MYADM FYCO1 LIMS1 SPIRE2 WNT10B MIER1 ROCK1 FAM115C SH2B1 DNMT3B PKIB IL1RAPL1 MAPT OSTBETA NSMCE2 NR1H2 CD14 FAM150A DRD3 CEP135 C2 C4B PAXIP1 FLRT2 EDN3 UBE2V2 CNOT6 GBA TIAM1 MARK2 AVIL PIP4K2B C3 ARSB GATA2 VPS11 WAS TPM1 BAIAP2L1 KATNB1 SHCBP1L ACD NOX4 LTK SYNPO CRK C4orf49 DMRTC2 DCN PROM2 HAP1 SERPINF2 EPT1 FMR1 ANKRD27 BAI3 UBE2J2 STK25 ARIH2 PFN2 ZFYVE27 UGCG CTSK MTF2 INSR SLITRK1 STOML2 HNF1B ZBTB17 MOB2 HDAC4 S100A10 MYF6 PHIP FGF8 IGJ NLGN3 RIMS2 FAM154A FER ANAPC11 TGFB3 HES1 BAP1 MUC1 RAC3 JUB SLC17A9 ACTN4 SYT1 TTC15 EREG GDF15 ROBO1 GZMB NLGN1 DNM3 CLIP1 RUFY3 NOX1 SNX3 SETDB1 CTR9 CRTC1 IQGAP1 LRP8 TINF2 NSUN4 ROCK2 CNTN1 SCARF1 AHI1 WIPF2 RTEL1 WDR1 UBE2L3 NME2 LRRTM1 CDK5RAP1 CTNNB1 CYFIP1 DRD2 CD28 PLXNB1 DOCK1 SERPINE1 SLITRK4 VLDLR EDN1 SYT17 AMIGO2 LPPR5 ARRB2 GOLGA4 NDEL1 CCL2 HAX1 CASP8 EHD2 ISL1 TFR2 BAI2 MYLK3 FAF1 UNC13D FRMPD4 RPS6KA4 GPR26 TSGA13 HSP90AA1 SFPQ NPTN NTRK2 ANGPT1 TDRD12 SRC CNOT6L EPHB2 EPHA4 STARD7 RAB21 DLGAP5 CCT2 DYNLL1 ARHGEF7 C6orf89 HSF1 GATA3 RGS2 CDKL5 MBL2 VEGFA CLSTN1 ITGA3 PRKCI IL4R VPS4A CXCL9 SIN3A ELMOD1 NEGR1 FAM21C PLS1 TP63 B2M CDC42 CAV1 SORBS1 PHLDB2 RHOQ MAPK8 PSMC5 PHF1 OLFM4 KIT YWHAZ WASF1 ANK3 AGR2 NES SFN ARPC3 CDC20 RAPGEF2 ITGAV FAM162A RAP1A TGFB2 PDXP ZNF304 FLRT3 SNAI2 NET1 ARHGEF10 NUSAP1 DDB2 SNX9 LYN SEMA7A PDCD6IP HK2 GPC3 AGT BID FGB CDK5R1 PPIF CDKL2 SHANK1 SMARCB1 PLK4 FBXO38 ATR BCL2L11 ARPC1A ERCC5 EIF5AL1 GTF2H5 C6orf125 FAM150B PTPRD PAFAH1B1 DPYSL3 UBE2D3 RHOU TSPAN1 LPAR1 ESPN PRKCE PFDN2 ARHGEF15 KIAA1199 ACCN1 RASAL1 PDGFRB NEUROD2 VPS28 SDCBP ARPC4 ERCC3 ABR RDBP JARID2 CHP ILK BRD7 GLS2 BICD1 CDC34 PLCG2 GCM1 CLEC16A CCL19 C20orf123 AR SLX1B SH3GLB1 PRKD1 IGF2 PRKCQ MYF5 SFRP4 ADRB2 LRSAM1 C2orf89 TPPP TSC1 SCN1B MYBPC1 HNRNPA1 OPA1 PAK3 ATAD1 ADAM9 CXCL13 EVL CNR1 PPP2R5B CSF3 RREB1 FGF20 ATRX NGFR GCFC1 ERCC4 EHD1 NDNF CCT7 DDHD2 VAMP7 IL28B RPA1 NTRK3 SMCR7L THBS2 GPX1 IL4 C15orf62 PMAIP1 MAPK6 CXCL12 ARPC2 CLSTN2 WHAMM CNTF DKC1 OGT WDR46 SMAD3 TERF1 NPHP4 C10orf129 CDK5 UQCC TNFSF14 NEK2 MRE11A RTF1 PRKCD NRXN1 MYOD1 DSCAM TWIST1 CYBA CLIP3 BTC TRPV4 CEP120 HCLS1 PHF19 CDH4 CORO1C CTTN PAX7 LIG4 SYT14L H2AFY ANKRD53 BRK1 AHSG COBL YWHAQ TCP1 SFRP1 EPHB1 MAP3K4 ISLR2 IL9R FBXW7 PLXNA3 TFDP1 NFATC2 ANXA1 VASP CFL2 ARPC1B IL2 VPS37B HAS2 GNB2L1 EPS8L2 GRB2 TLR4 KALRN NRXN3 PIK3R1 VPS4B MYO1C BHLHB9 FIS1 ATAT1 BCL2 TAL1 PML PLD6 LRRC16A NDRG4 CDCA5 PSMB7 GTF2H2 NEUROG1 CAMK1D CDC42EP5 BBC3 CPB2 TRIM67 EEF2K ENC1 JMY PAN2 MAP2K1 MYOC IL6 NRG1 NME1 ACTR3 RAC1 NMUR1 TUB LINGO2 ODZ2 NPHS1 HPCA CDC42EP3 CBFA2T2 STMN2 BAI1 CCL26 TWF2 FUZ PRDM12 FNBP1L BMP10 SAE1 NEFL DNMT1 DSTN ATPIF1 ACSL3 TGFA TET1 CPEB3 ITGAX PICK1 KIAA1731 ITGA2 HGF SLN AIM2 DMRT1 RPL28 LMAN2 SLC11A1 AURKB AKT1 IRF8 CCL11 EPHA2 WNT3A EPB49 DISC1 ALOX15 ATP8A1 DDX56 TP53BP2 DAB2IP BAX DPP10 TAPT1 DVL1 KIAA0947 PSEN1 MAPK1 U2AF2 CIB1 SNCA SYNPO2 ABCA7 LIMK1 KIDINS220 CCT4 RAPGEF3 SNW1 CDK9 CBLN2 CTGF RHOG ERCC1 ANAPC2 ATP8A2 RAB3GAP1 ICAM1 SRPX2 ACE2 TNF SNIP1 ARPC5L PHLDB1 RLTPR XPA HMBOX1 RET RHOA NPM2 HYAL1 CSNK2A2 FIG4 APOA5 SIRT2 NMT1 SPAST NCKIPSD CLRN1 RELN WNT5A PALM NTN1 APOE ODZ3 YWHAG POT1 FMN1 CSF2 CBLL1 HFE TPR SLITRK5 OBSL1 OBFC1 ACTR3B CUL3 UBL4B WNT4 KIAA0528 FOXO6 FZD5 PSMD8 LRRC46 HTRA2 FLNA FN1 GH1 CDC42EP1 KIF5B DAG1 SPACA3 CALCOCO2 TP53 CAPRIN2 BAK1 KIAA1324 SLX1A SCIN TERF2 FLRT1 FOXP3 IFNG LLPH MAPK15 HHAT PFN3 EPS8L1 PTPRJ RNF40 MLL TMEM27 RANGRF FAM178A PLEK YWHAE IST1 DDR2 E2F1 KAT2A PPP1R13B HDAC6 LRRTM3 SCGB3A1 CALR RAB11A WNK3 AXL HRAS STK11 MED25 OR13C4 CLU NCK2 TNKS RPA3 RRN3 AUTS2 IQGAP2 WRAP73 MIEN1 BNIP3 MYOG CRKL CALY WNT7A GTF2H3 PARK7 EIF5A2 BBS4 L1CAM GSK3B SETX NRP1 PINK1 SYT2 SLITRK6 LPHN3 MAGEL2 MOAP1 ARHGEF5 DDB1 SREBF2 HNRNPK NKX2-5 MMP9 RND2 FEZ1 RAPGEF1 SFTPD RAMP3 NCK1 SS18L1 DOCK5 PREX1 EPB41L5 EED SEMA4D ERBB2 ING2 SLITRK3 EZR CUX2 BAIAP2 RIT2 CBL EZH2 PDZK1 CDC42EP2 CBLN1

GO_DIGESTIVE_SYSTEM_PROCESS A physical, chemical, or biochemical process carried out by living organisms to break down ingested nutrients into components that may be easily absorbed and directed into metabolism. AQP1 HRH2 CD36 SLC26A7 ACO1 EZR ZNF830 CEL CCKBR MOGAT2 SLC9A4 VIL1 TJP2 SOX9 SERPINA3 MUC4 VDR MUC6 TLR9 STATH TLR4 KCNN4 SLC26A6 STRAP LDLR NMU COPA NPC1L1 SOAT2 FABP1 CHRM3 MUC2 PNLIP F11R AQP5 SLC5A1 ADRA2A GHRL ABCG5 RBP4 ABCG8 SCT KCNQ1 SCARB1 CHRM5 DRD3 VSIG1 SLC46A1 CHRM1 UCN NOD2 MUC13 LEP PLS1 NPR3 TFF1 GCNT3 AKR1C1 PBLD

GO_CELL_PROLIFERATION The multiplication or reproduction of cells, resulting in the expansion of a cell population. KITLG DAGLA PPP3CB BMPR2 NANOG ELF4 DKC1 ENTPD5 C1orf177 PCNA IGFBP3 CR2 GPX1 NOX5 TRIM71 GLI3 IGFBP5 DCT DOCK7 KIF15 IFNA10 MET CDC25A CSPG4 FGF4 CSE1L TNFRSF4 HTR1A PIM1 BCL2L1 LRRC17 GLUL LOC93622 ELN IGSF8 TRIM27 KIF14 PCM1 RASGRF1 USPL1 CD40 GAB1 JUNB ESR1 MAS1 MED1 LIG4 CXCL1 CKS2 DCTN2 CUZD1 SIX1 MORF4L1 CDK1 PDAP1 MCM10 CEP120 BIN1 APOA1 IFNA1 CDV3 TGFBI NPPC MRE11A LRP1 NRD1 GNAI2 UBR5 EGR1 IRF6 TWIST1 TUSC2 ATOH8 LGR4 PRKCD BCAT1 CDK5 PDK1 FZD6 LIPA RERE WNT7B GSTP1 XBP1 TNFSF14 CBFA2T3 TACC1 PIM2 NKAP DACH1 PEMT FAT4 TXN PDGFRB ARTN LY86 CD5 PTPN6 PTK2B TBX20 ABCB1 FAP PAFAH1B1 ID4 TSPAN1 CYP1A1 BTBD10 UHRF1 SOX9 PAK1IP1 ROS1 GINS4 NUMB PAX1 GBX2 NDE1 LAMA5 E2F8 PTPRC TRNP1 ELF5 AGT GPC3 IFNE FIGF CD151 PDXK PRKDC DOCK8 LGR5 FGF10 POC1B ABL1 F2RL1 TGFBR3 CDK5R1 ETV6 DLG1 CER1 APPL2 MMP14 YME1L1 MPL IFNA21 BOP1 RPS6 MELK AR INSIG1 FBXW8 CYR61 PRKD1 NR6A1 STC1 PRKAR1A PLCE1 CD40LG NAB2 ARHGEF1 RAB10 DBN1 RORA FES TGFBR2 ATF5 ILK USP28 LIPG GAS6 PTCH1 OSR1 NPY IL33 SKI TAF8 SYK COL4A3BP SCARB1 SIDT2 ERCC2 SART3 FGFR3 CCND1 SRA1 EDNRA CDH13 SEMA5A C19orf80 C5AR1 CLK1 TXNRD1 KRT16 EEF2 CRTAM IMPDH2 RPS27 DLGAP5 FGFR2 DOCK2 C6orf89 ZNRF3 BMP2 RC3H2 SRC MIF H3F3A ASCC3 FBXO4 TXLNA FURIN SHH RPL23A ORC3 MSX1 HHIP IL5RA ITGB1BP1 GMNC COL4A3 DCHS1 BUB1B NR4A3 IFI16 CD81 WNT1 CUL1 COL8A1 TGFB2 SOX5 MAP4K1 NES PURB AXIN2 H3F3B PAK7 FOXN1 NCSTN TP63 ACHE NKX6-1 TACC3 HELLS KIT DUSP22 AREG TEAD3 TNFSF11 CITED1 THAP1 PICALM PSPHP1 IFNA7 FGFR1 RXRA TNFSF13B BHLHE41 GNL3 STAT6 FOXC1 ARX KDM1A FOXC2 EMP2 TNFSF9 ALK INSL4 S100B PRDX1 SDR16C5 GRPR LEF1 SLAMF1 EREG FER MIA RBBP7 ZFP36L1 CHRM1 TACC2 CHRM5 FZD9 TEK COPS2 FGF13 CCKBR C2orf29 MAPRE2 OGFOD1 HAND2 CUL5 SALL4 BUB1 IRF2 FGF7 DTYMK PES1 FABP7 NDEL1 OCA2 ERG SCRIB ZFP36L2 EMX2 IFNA13 WNT16 BMPER MATK ANGPT4 NAA60 IFNB1 TSPY1 IL2RA SOX11 IL10 UBE2L3 MTCP1 PRMT5 CTNNB1 NBN UNCX CD180 MEGF10 BLZF1 ENPEP RAF1 RRM1 MXD1 WNT7A TYMS MEF2C CD79A TCF7L2 SMAD1 BCAR1 HRAS PDS5B UBE2V2 ZAK IFNA16 FAM83B CLU TYR VTI1B AZI2 RRN3 ACE DAGLB PA2G4 USP8 CHRM4 CHRM3 USP13 TCF19 TNFRSF17 ECD CTC1 DDR2 KAT2A ADRA1B HNF1B IFNK PAK4 PRDM4 LHX5 KIF2C CD86 BTRC FRAT2 FGF8 ASCL1 PROK2 TNFSF18 PDZK1 ZNF259 NKX3-1 RIPK2 NCAPG2 POLA1 BYSL ARIH2 SIX2 ERBB2 NEUROD4 DLX5 DIXDC1 CDK5RAP3 APC2 GNAT1 PIK3CB NKX2-5 AMBN PDPN ID2 POU3F2 PALB2 IFNW1 MCM7 NPR3 MTSS1 OSM LTK NUMBL RNF43 IL15RA CSF1R PLCL2 DDIT4 TBK1 REG1B FIGNL1 MS4A1 CD74 NUDC PSMG1 GOLPH3 DAB2 PLXNB2 MYH10 IFNA14 POLR3G CRIP1 LARP1 ISG20 GNG2 RHBDF1 PTEN PRG4 SIX3 P2RX7 IFNA4 WNT5A FAM83D FGF1 CDC25C CDC16 MTCP1NB CEBPB CDK7 GINS1 EBI3 IL23A GLI2 SLC39A5 FRS2 ZEB1 SETMAR CTPS SFRP2 FZD3 HOOK3 TNF SRRT PPP1R8 LOXL2 TPX2 IFNA17 IFLTD1 BAK1 TP53 PRKX DVL2 PELO RASGRP4 WNT10B MT3 PYY HDGFRP3 FGF6 WDR12 IFNA2 CD276 SIPA1 WNT4 HSPD1 FERMT1 BCL2L2 SPHK2 GCG MKI67 OSR2 EPS15 CD160 YAP1 PROX1 KLF10 DAZAP1 XRCC5 PPARD STAT1 AURKB AKT1 TACSTD2 SLC11A1 NF2 EPHA2 FAM83A LRP2 RETNLB WNT3A DISC1 WNT2B FKBP1B REG3A BAD HGF ACVR2A BOK BRAT1 SMAD4 DMRT1 CDK3 IFT20 SKP2 PITX2 EPS8 TGFA BMP7 POU3F3 CD34 FTSJ2 LGI4 LRP6 RAP1B ITGA2 RUNX1 SBDS CREG1 MDM4 HDGF EVI5 IRS2 UHRF2 EGFR BMP10 LGI1 ERCC1 TRAIP IFNAR2 GNB1 PRPF19 NASP TGFB1 UCHL1 BST2 AMELX C6orf108 TNFSF8 CDC27 RACGAP1 APPL1 SNW1 LHX9 STIL FBXL7 PSMB10 RAPGEF3 IGFBP4 CKLF CDK9 COL8A2 CTGF ERN1 IL15 SLAMF6 C3orf58 FSHB HHEX NAA35 IGF2BP1 PLK1 ZMYND11 GPC4 ACVRL1 BAX MNAT1 THPO STAT3 CDC14A MECOM RPGRIP1 ASPM MAPK1 PSEN1 HLCS ADRA1D NRARP BCL2 SATB1 CCND3 SH2D2A FSCN1 ERBB4 PIK3CG TLR4 HMGA2 KHDRBS1 MALT1 BTN3A1 MMP16 LILRB1 CSGALNACT1 TFDP1 IL9R NOTCH1 EML1 ANXA7 TBX1 GFI1B DDX41 FYN E4F1 SOX4 IL7R IMPDH1 CADM1 EPHB1 BRCA2 GAPT MAP3K11 TSPO CSF1 IGF1 RAG2 TNFSF4 EHF CENPF FLT3 NOV ALKBH3 IL1A KLK8 PRDM1 ITPKB IFNA8 SRGAP2 RPS6KA1 EIF2S2 PRL FGF5 CTF1 SHB SERPINF1 HPRT1 RAC1 EMP1 SLC29A2 RC3H1 CITED2 MUSTN1 BMP4 DPH1 PENK KRT2 ODZ4 CEACAM1 IL6 MAP2K1 CDK11B NDP CD70 HOXB4 NDRG4 MAPRE1 RYK WNT2 IFNA6 GRHL2 IFNA5 KCNA1 HMOX1

GO_MYELOID_LEUKOCYTE_MEDIATED_IMMUNITY Any process involved in the carrying out of an immune response by a myeloid leukocyte. CTSG SPON2 TUSC2 TREM1 IL6 STXBP3 VAMP8 AZU1 KIT S100A13 CXCL6 CAMK4 PPBP PI4K2A ADAM17 JAGN1 CPLX2 SERPINB9 CCL3 PLA2G3 PIK3CG LYN MRGPRX2 VAMP2 RASGRP1 SNAP23 CXCL5 ELANE MILR1 PLA2G1B LAT2 CHGA LAT ACE BTK MLL5 VAMP7 PIK3CD ANXA3 STXBP2 NR4A3 IRAK4

GO_VASCULAR_ENDOTHELIAL_GROWTH_FACTOR_SIGNALING_PATHWAY A series of molecular signals initiated by the binding of a vascular endothelial growth factor (VEGF) to a receptor on the surface of the target cell, and ending with regulation of a downstream cellular process, e.g. transcription. NRP1 NRP2 FLT3 FLT4 PDGFRB KDR FOXC1 VEGFA HSPB1 PRKD1 PRKD2 MYO1C PDGFRA FLT1

GO_REGULATION_OF_ER_TO_GOLGI_VESICLE_MEDIATED_TRANSPORT Any process that modulates the rate, frequency, or extent of ER to Golgi vesicle-mediated transport, the directed movement of substances from the endoplasmic reticulum (ER) to the Golgi, mediated by COP II vesicles. Small COP II coated vesicles form from the ER and then fuse directly with the cis-Golgi. Larger structures are transported along microtubules to the cis-Golgi. YIPF5 GAS1 RINT1 SCFD1 STX18 INSIG1 SEC24B SORL1 ARF1 RNF139 TBC1D20

GO_POSITIVE_REGULATION_OF_NEURAL_PRECURSOR_CELL_PROLIFERATION Any process that activates or increases the frequency, rate or extent of neural precursor cell proliferation. NOTCH1 DCT VEGFC GNG5 GLI3 HIF1A GLI1 GLI2 NES GPR37L1 FZD3 SHH CX3CR1 GPR56 PROX1 PTCHD2 SOX10 CTNNB1 EGF DMRTA2 DRD2 SETD1A ASPM INSM1 VEGFA LHX1 LHX5 CDON ELL3 LYN PAX6 NR2E1 ASCL1 WNT3A DISC1 KDM1A SMO KIAA1524 OTP DLL4 SMARCD3 ZNF335

GO_AMP_METABOLIC_PROCESS The chemical reactions and pathways involving AMP, adenosine monophosphate. NT5E AK1 PRPS1 AK3 ADSS AKD1 ADSSL1 APRT AMPD2 AK2 ADK AMPD3 ADSL AK4

GO_ERROR_PRONE_TRANSLESION_SYNTHESIS The conversion of DNA-damage induced single-stranded gaps into large molecular weight DNA after replication by using a specialized DNA polymerase or replication complex to insert a defined nucleotide across the lesion. This process does not remove the replication-blocking lesions and causes an increase in the endogenous mutation level. For example, in E. coli, a low fidelity DNA polymerase, pol V, copies lesions that block replication fork progress. This produces mutations specifically targeted to DNA template damage sites, but it can also produce mutations at undamaged sites. RPA3 RFC2 REV3L RPS27A PCNA POLE2 RFC5 MAD2L2 RPA1 POLI POLK RFC3 RFC1 REV1 RFC4 UBA52 UBC RPA2 UBB

GO_AXIS_ELONGATION The developmental growth that results in the elongation of a line that defines polarity or symmetry in an anatomical structure. FGF10 TNC TRIM28 MAGI2 PTK7 BMP4 FGFR2 SOX9 FGF1 WNT5A SFRP1 SFRP2 VANGL2 MED12 SHH LRP6 SPRY2 WNT3A SPRY1 HOXD13 YAP1 RDH10 ESR1 WNT11 MED1 AREG TGFB1

GO_REGULATION_OF_CELL_DEVELOPMENT Any process that modulates the rate, frequency or extent of the progression of the cell over time, from its formation to the mature structure. Cell development does not include the steps involved in committing a cell to a specific fate. CPNE5 SEMA4B GDPD5 SIGLEC15 G6PD UNC5D PTPRZ1 SKOR2 HES3 MEGF8 MBD1 B2M HMGB2 IFRD1 CDC42 HOXB3 AXIN2 NEGR1 FAM21C KIT OLFM4 NKX6-1 POU4F1 EFNA1 NET1 SOX14 TNFRSF12A SEMA7A LYN SCRT1 ITGB1BP1 PDLIM5 FERD3L FAM101A CHN1 NODAL ZFHX3 LZTS1 SMO HDAC1 RAP1A IL1RAP CDC20 FGA RAPGEF2 SEMA4A CLCF1 CAPRIN1 BMP2 BRAF PLXNB3 SORL1 NTRK2 ASAP1 GRID2 GBP1 EPHB2 SEMA3D SMURF1 NTF3 SHH NELF SRGAP2P1 UNC13D DICER1 NPTN GSX2 NR2F1 MEIS1 VEGFA ITGA3 CDKL5 CPNE9 DGUOK CDON TRPV1 RNF112 LOC100507050 RTN4R SEMA5A PRKCI RAB21 REST FBN1 EPHA4 KIAA1598 WNT3 DKK1 CAV3 GATA3 PRKCH ARHGEF7 LGALS1 KIF13B ID1 DOCK1 PER2 SDC2 PLXNB1 VLDLR RAP1GAP2 PLK2 FBLN1 WDR1 RARA SOX11 PTK2 ABL2 PLAG1 GORASP1 P2RY12 SRF CTNNB1 PRMT5 DRD2 NME2 RIMS1 SPG20 SEMA6D CDK5RAP1 MDM2 MYCN ISL1 FGF13 PDE3A MYLK3 PTPRG SEMA3A TBX6 CHRNB2 SYT17 EDN1 ETV5 GOLGA4 NDEL1 SHOX2 UNC13A ITM2C LPPR5 SLC39A12 HDAC9 SYT1 ACTN4 HDAC2 RAC3 GFI1 KIAA0319 CHRNA3 FAM101B PPP3CA KNDC1 C3orf17 FAM5C SYNGAP1 MACF1 GPER TRPC5 NLGN3 RIMS2 MFI2 ULK2 GAK NTRK1 FOXA1 HES1 OPRM1 GDF7 HMG20A ADIPOQ ROCK2 CNTN1 PTK6 GPR183 TNFSF11 LRP8 DPYSL2 FGFR1 SCARF1 OLIG2 METRN KDM1A POSTN SLIT2 DLL3 SIPA1L1 CLIP1 ROBO1 NLGN1 PIN1 DNM3 TMEM106B OLFM1 NKX2-2 MAGI2 CRTC1 IQGAP1 CHD7 SNX3 ZNF335 RUFY3 BCL6 CPNE6 NEDD4L LRP4 LRRK2 NPPC LRP1 EFNA5 MYOD1 DSCAM NGF INPP5J CDK5 RAP1GAP DCC CPNE1 PPP2R1A FEZF2 CTTN LIG4 PPARG WDR36 MED1 INPP5F PBX1 C16orf45 NTF4 COBL CDK1 SIX1 SYT14L NFE2L2 MAP1B SLIT3 BIN1 PTPRF TRPV4 CDK5RAP2 CDH4 DUOXA1 NPR2 SF3A2 SSH2 CXCR4 CORO1C APOA1 DOCK7 IRX3 DCT FGF20 GLI3 NGFR NDNF NOTCH3 IMPACT NAP1L2 KIF14 TRIB1 GFAP FAM5B PLXNC1 RREB1 PCM1 COL3A1 SDK1 PPP2R5B CNR1 ARPC2 BMPR2 SYT4 MAPK6 CXCL12 MYCL1 TRIM32 GSK3A CNTF ATF1 CREB1 VAMP7 NTRK3 NOS1 OMG KANK1 BDNF SMARCD3 CLDN18 NRG3 ILK MMD FES ZNF488 GDF6 DBN1 PHOX2B EIF4G1 SKI STX1B ATP6AP1 TMEM30A SYT3 FRMD7 EIF4ENIF1 ROBO2 LPAR3 SEMA6A MUL1 MYF5 CRX RFX3 APBB1 CERS2 C10orf125 SCN1B ELL3 OPA1 CARM1 PAK3 GPR173 VIM ISL2 CLDN5 FRZB SEZ6 TIMP2 OTP ARHGEF1 SEMA6B RPS19 PRKD1 IL5 ENG FBXW8 AP1AR LIF FBXO38 ECT2 NUMB SHANK1 TYROBP FAM150B SEMA5B THY1 CCR2 PRRX1 PLXNA2 SEMA4C SHANK3 BMPR1A GPR56 CDK5R1 CCDC88A ABL1 FGB RASAL1 SPP1 NEURL NEUROD2 GDI1 VAX1 DDX6 ARHGEF2 SOX8 S1PR2 LRRC4C ADNP LPAR1 CAMK2B ID4 PTPRD PAFAH1B1 PTK2B DPYSL3 SEMA3B CHODL ARF6 SOX9 STK24 CDH2 MIB1 PTN AKAP6 BEND6 NEUROD1 SEMA3F DLL1 EPHA3 CIB1 KIDINS220 PTPRO MAPK8IP3 CTNNA1 ZNF664-FAM101A CTDSP1 PQBP1 ASCL2 LIMK1 EPHA7 LSM1 ADRA2C SLC45A3 SPOCK1 DAB2IP RAB17 STAT3 FBXO22 TBR1 OGN TCF3 PSEN1 DVL1 ASPM RTN4 PRPF19 ANAPC2 CUX1 GAL TGFB1 DLX2 KDM4A SRCIN1 ATP8A2 SLIT1 CASZ1 SNW1 RGMA AMIGO1 FGG ARF1 BMP7 FMOD F2 TRPC6 NF1 S1PR5 CPEB3 SEMA6C MMD2 ARHGDIA STMN2 BMP10 INS NEFL RARB TWF2 IKBKB TP73 NF2 SCRT2 SPAG9 AKT1 TACSTD2 DDX56 PAX6 FKBP1B WNT3A DISC1 EPB49 LINGO1 KCTD11 FOXO3 BAD KIF20B HGF DLL4 CUL7 ADRA2B SMAD4 GPR68 UFL1 EYA1 SLC9B2 BARHL2 MAP2K1 MYOC SOX3 PLXND1 IL6 CTDP1 EEF2K SPINT1 TRIM67 ENC1 ODZ4 ARNTL NME1 CSNK1E RNF10 EDNRB TRIM11 DDX39B RHEB NRG1 CNTN2 WNT2 LMX1A SOX10 MTOR NDRG4 FAM134C GPRC5B SOX2 NEUROG1 EIF2AK4 CAMK1D C1orf187 PACSIN1 XK ARHGAP35 SARM1 SEMA3G KEL SEMA3C FSTL4 SRGAP2 CBFA2T2 CRABP2 EPHB3 KLK8 ZHX2 RAC1 DNM2 ZSWIM6 SHB SERPINF1 LTA ANKRD1 BMP4 CFL1 NOG CAMK1 NOTCH1 TTL BCL11B YWHAH PLXNA3 LILRB1 HIF1A NKX6-2 ITSN2 HES5 WEE2 HAS2 TTPA FYN EOMES IL2 SKIL CX3CR1 ITPKA PTCHD2 SFRP1 LIN28A IGF1 TSPO NCOA1 ISLR2 CSF1 NEUROG3 BCL2 TRIOBP LTF ADCYAP1 LRRC16A CRMP1 MAN2A1 HMG20B NBL1 ERBB4 NRCAM PARK2 KALRN HEYL HAMP PMP22 NEDD4 ZMYND8 BHLHB9 EIF4E PAQR3 NKX2-5 HAP1 VWC2L C5orf13 PROC FMR1 CYB5D2 POU3F2 ANKRD27 ID2 FEZF1 RND2 RAPGEF1 SEMA3E FEZ1 SYT2 LTK DUSP10 GPR37L1 SETX HOXD3 NRP1 KATNB1 ARHGAP4 NUMBL CRK MOB2 CLOCK HDAC4 CUX2 S100A10 KLK6 CNTN4 RAB7L1 MYF6 ASCL1 BAIAP2 RAP2A RIT2 EZH2 NCK1 STK25 SS18L1 DOCK5 PREX1 BAI3 SEMA4F CDK5RAP3 TNFRSF1A DIXDC1 SEMA4D ZFYVE27 SPEN NFATC4 FLRT2 STAR RAB11A CALR TNIK PPP1R12A DLX1 RGS14 MAPT IL1RAPL1 DRD3 IST1 DTX1 HOXA11 PDE5A FAM150A GATA2 MYOG CRKL ARSB RELA NCS1 L1CAM GSK3B WNT7A HOXA2 MEF2C UST SEMA4G TGIF2 STK11 TCF4 KLF4 UBE2V2 SNAP25 RRN3 HEY2 S1PR3 TIAM1 MARK2 GRIN1 AVIL APP RTN4IP1 TNFRSF21 FOXO6 ATOH1 SLC6A4 FBXO7 BCL6B EPO FLNA YAP1 ZNF536 TCF12 SNAPIN OBSL1 PROX1 XRCC5 RNF6 ULK4 LIMS1 CAPRIN2 CACNA1A NGEF ROCK1 PLXNA4 TLE6 DNMT3B TERT RCC2 IFNG CSNK1D LLPH MT3 CDH5 SSH3 FN1 VWC2 BMP6 GPR124 GDF5 MYADM VEGFC PPP1R9A EPYC RHOA AURKA C1QBP RET FIG4 GLI2 SIRT2 ADCY6 HOOK3 TRPV2 TNF TNR TLX3 FZD3 MAG IL6ST SFRP2 GNRH1 XRCC2 BAG5 ZEB1 DBC1 DMRTA2 SERPINE2 PTK7 TLX2 SRRT NTN1 ODZ3 APOE AKAP13 PLK5 PALM FKBP4 STAP1 YWHAG PLXNB2 PLXNA1 IL1B JAG1 TLR2 NR2E1 NCKIPSD BMP5 LBX1 SSH1 DYNLT1 WNT5A EMX1 DAB1 SIX3 RELN PTEN

GO_SKELETAL_MUSCLE_CONTRACTION A process in which force is generated within skeletal muscle tissue, resulting in a change in muscle geometry. Force generation involves a chemo-mechanical energy conversion step that is carried out by the actin/myosin complex activity, which generates force through ATP hydrolysis. In the skeletal muscle, the muscle contraction takes advantage of an ordered sarcomeric structure and in most cases it is under voluntary control. TNNT1 SYNM MYH8 RCSD1 TNNC1 GAA EEF2 CHRNB1 CCDC78 HSP90AA1 MYH3 STAC3 TNNC2 TNNT3 RPS6KB1 TNNI1 MB JSRP1 HOMER1 MYH7 CHRND CHUK TNNI2 ATP8A2 TNNI3 MYH14 CHRNA1 TCAP DMPK

GO_FERTILIZATION The union of gametes of opposite sexes during the process of sexual reproduction to form a zygote. It involves the fusion of the gametic nuclei (karyogamy) and cytoplasm (plasmogamy). SPTBN4 ZPBP LY6K CDK1 CCT4 OOEP TNP2 DUOX2 UBAP2L PVRL2 PCSK4 UBXN8 TEX11 SPATA22 CATSPER1 SPEF2 PLCB1 ASTL GLRA1 CATSPER4 ATP8B3 MST1R XKRY BAX H3F3B BPY2B SYCP2 TRPC3 ADAM21 MAEL TUBGCP3 PRDM14 CATSPER2 TARBP2 POMZP3 NLRP5 ZP4 CSDA CD46 CRISP1 PARK7 CATSPER3 ATP1A4 SPA17 CCT7 BPY2C OR1D2 ADAM2 CCT2 IZUMO1 SMAD4 ZP2 TRPC6 TDRD9 OR10J1 TDRD12 UBE2Q1 FOLR4 CATSPERB T WDR48 B4GALT1 AKAP4 FETUB CCT5 H3F3A IGSF8 CLGN BCL2L1 OVGP1 HOXA11 C16orf73 MFGE8 BSPH1 FNDC3A ZP1 ADAM30 HOXD9 PLCD4 PVRL3 SPAM1 SPAG1 SLIRP KLHL10 DEFB126 TTLL5 HVCN1 RAD21L1 ABHD2 REC8 PKDREJ SYT6 AKAP3 SPACA3 INSL6 AR ZPBP2 SERPINA5 CATSPERG FOXL2 AAAS RNASE10 UMODL1 ADAM20 SYT8 ACR STRA8 CCT3 TNP1 ZAN KDM5B TRIM36 HEXB ELSPBP1 SLC22A16 ROPN1B LINC00085 TDRKH KCNU1 LCN6 ADCY3 HOXA10 NR2F2 DNALI1 UBE3A PLCZ1 CCT8 LRMP FUT10 GLRB SMCP C9orf11 HOXD10 SPESP1 BPY2 PRSS37 NPM2 ZP3 WBP2NL STX2 GNPDA1 TEX15 WEE2 SPAG8 TCP1 APOB KLK14 TRPC7 CD9 SPACA7 HOXA9 CLIC4 TMEM146

GO_RESPONSE_TO_TOXIC_SUBSTANCE Any process that results in a change in state or activity of a cell or an organism (in terms of movement, secretion, enzyme production, gene expression, etc.) as a result of a toxic stimulus. GSTP1 ERCC6 TXNDC2 MBP TRPM6 SOD1 RPS6KB1 EPX MAPK1 FOS PINK1 CDK4 GPX2 BAX KDM3B MGMT ASNS PTGS2 NUPR1 MAPK3 EPHX1 PXDN PTGS1 PON1 GPX7 GSTK1 CHKA SCN9A GPX6 GSTO2 IYD EHMT1 ASS1 HBA2 HBA1 GSTZ1 HP ARG1 IPCEF1 RAD51 AQP10 PXDNL MGST3 DUOX2 ALAD SOD3 SRD5A1 SCFD1 PRDX5 TXNRD3 UBIAD1 CDK1 LYN CCNB1 C10orf58 PRDX4 CNP INMT DDC WAPAL C9orf21 MOSC1 CPOX S100A9 CERS1 NEFL AHR SLC7A8 PON3 CCS CYP1B1 BPHL CCL5 MGST2 OPRD1 SLC6A14 DHX15 FAS MOSC2 KCNC2 MPO SESN1 CES1 KCNC1 LTC4S STAR HDAC6 HBB GSTM2 GSR GSTM3 CHUK CST3 MT1X GPX1 TXNRD1 DNMT3A FECH PON2 TYMS PARK7 CDH1 SLC7A11 GSTT1 MAOB CPS1 TXNRD2 AIFM1 DHRS2 DRD2 GPX4 SLC23A1 SRF NXN CLIC2 KDM5B CCL3 GPX5 BLMH HTRA2 SOD2 LPO GJC2 APOM PDZD3 SLC6A4 GABRB1 PRDX3 MGST1 EPHX2 SLC6A1 PENK PRDX2 DUOX1 TXNDC17 NQO1 MT3 SDC1 SCN1B DNMT3B GSTO1 CDKN1A GPX8 GUCY2C HTR1D NXNL1 BAK1 EIF2AK2 MDM2 CYP2F1 SLC18A2 PRDX6 SEPW1 ALB SCN2B SLC30A1 SELS GSTA1 TP53INP1 CAT TTPA CCL4 KDM6B FABP1 XPA PRDX1 TH PPP1R9A APOA4 CYP1A1 SESN2 SLC22A8 NOS3 GPX3 KDM1A NXNL2 TPO CYGB SLC30A4 PARK2 TLR2 MPST SRSF9 ABTB2 ADAMTS13 GLYAT SRXN1 GSTM1 PDGFRB APOE BCL2 ATP7A

GO_RESPONSE_TO_RADIATION Any process that results in a change in state or activity of a cell or an organism (in terms of movement, secretion, enzyme production, gene expression, etc.) as a result of an electromagnetic radiation stimulus. Electromagnetic radiation is a propagating wave in space with electric and magnetic components. These components oscillate at right angles to each other and to the direction of propagation. RNF168 SLC1A2 GNB1 TGFB1 UBE2A TMEM109 ABCA4 PPM1D SNAI2 NET1 MSH6 ERCC1 ICAM1 KAT5 APOBEC1 UBE2B RAD51C DDB2 GJA10 ATP8A2 IFI16 GUCA1A ANGPT2 OPN4 C9orf80 RIC8A GADD45A RNF8 RBM4B HMGCR CRY1 TOPBP1 ATP1A3 NETO1 CACNA2D4 RAD9A PPP1CC TP63 DRD1 POLB MGMT SLC4A10 TRPM1 TRPC3 MEIS2 FOS CABP4 BAX CASP3 NPHP1 IL12B DCUN1D3 TLK2 KIT MAPK8 INTS7 TP73 SLC24A1 PAPD4 TRIM13 CCL11 SIRT1 RAD9B CCND1 AURKB AKT1 ERCC2 NHEJ1 LZIC HRH2 SAG KIAA0146 LRRN4 DNMT3A TSPYL5 FECH HSF1 PITPNM1 GATA3 BRAT1 FKBPL TIPIN HIST3H2A EYA1 CNGB1 ATF4 ATP1A2 MBD4 IFT20 BRAF C12orf5 MME KCNC1 THBD UBE4B NF1 PTPRK KCNC2 RP1 CYP2R1 DBH PBK PARP1 GRK1 FBXO4 TP53BP1 MAPK14 KRT14 DTL EGFR NPTN CERS1 XRCC6 BABAM1 MDM2 COPS3 EYS MAP4K3 POLG CDKN1A TROVE2 REV1 CHRNB2 DNM2 USP2 ELK1 FOXB1 RGR PLEKHB1 POLH CCL2 PENK PIAS1 OBFC2B GNA11 FANCG CDKN2D RAD18 C12orf32 ELOVL4 XRRA1 NR2E3 MAP2K7 CLK2 ITGB1 GUCA1B PDC OPN1LW GTF2H2 MTOR NDRG4 EIF2S1 PNKP USF1 USP1 C15orf42 DRD2 KIAA1530 EIF2AK4 RAD51B BCL2 OPN1MW2 YY1 HMGN1 DMC1 OPN1SW PML CCDC66 XPC ERCC8 RHO NR2F6 RAD1 PPP1CB MYC KDM1A DCLRE1C NMU HAMP CHEK1 GNAT2 OPRK1 GRB2 CHEK2 GRIN2A GNAQ CRTC1 NEDD4 DEAF1 ZRANB3 BCL3 PIK3R1 MSH2 ANXA1 SDR16C5 TH RDH11 OPN5 HIF1A SYNGAP1 PRKAA1 GNGT1 GRM6 BRCA2 BRE SLC1A3 H2AFX HUS1 NLGN3 SFRP1 OPN1MW TRIAP1 ARRB1 MAP3K4 NTRK1 OPRM1 PPP1CA SDF4 BRCC3 CLOCK ELANE KRT13 RRH LIG4 JUN INTS3 MEN1 RPE65 NOC2L C1orf124 BRCA1 BEST1 ALAD CYBA CCR4 FBXL21 RFWD2 RAD51 BHLHE40 RCVRN USP47 GNAT1 SERPINB13 XRCC3 GNGT2 SWI5 FMR1 CCL7 MTA1 ID2 CACNA1F EGR1 ASNS NOX4 NPHP4 PTGS2 CDK5 HMGCS1 NUCKS1 DDB1 VCAM1 ERCC6 TULP1 PDE1B RHOB CASP9 RRM1 SMC1A IL12A PMAIP1 CXCL12 RELA TRIM32 MFAP4 AIPL1 CREB1 HYAL3 PCNA STK11 ZAK INO80 DDHD2 KIAA1967 FANCD2 GPX1 IKBIP AGRP TYR ADAM2 BRSK1 GRIN1 AANAT NFATC4 PAXIP1 STAR UIMC1 IMPACT ERCC4 CDC25A RGS14 NIPBL KIAA0101 HOXA1 BCL2L1 POLD1 MLL DRD3 JUNB N4BP1 PDE6G COL3A1 RS1 CIRBP MAPK10 TP53 BAK1 PDE6C DNMT3B RGS9BP AQP1 JUND SIK1 NPS CTNS TNFRSF11A GUCY2F PDE6A IVL RAD54L ATM CCDC111 EP300 CREBBP OBFC2A USP28 APP CUL4B ERCC3 FAM175A XRCC4 YAP1 KDM4D F11R OGG1 WRN MECP2 PER1 XRCC5 DUSP1 MC1R CNGA1 CDS1 ACCN1 ZBTB1 TXN B4GALT2 TANK FBXL3 RDH13 HRH1 FIGNL1 PLK3 OPN3 CRIP1 UNC119 ACTR5 TMEM161A RAD51D RFWD3 CRY2 FEN1 PTN BLM PDE6B ABCG5 CXCL10 PPP1R1B ATR RUVBL2 ECT2 XPA GTF2H5 DYNLRB1 RBM4 SEMA5B AEN HYAL1 ERCC5 TP53INP1 KRAS CAT CRYAB PRKDC RHBDD1 SFRP2 RAD51AP1 XRCC2 C10orf116 SCARA3 EEF1D HYAL2 EYA3 TANC1 PPID

GO_WHITE_FAT_CELL_DIFFERENTIATION The process in which a relatively unspecialized cell acquires specialized features of a white adipocyte, an animal connective tissue cell involved in energy storage. White adipocytes have cytoplasmic lipids arranged in a unique vacuole. CEBPA FGF10 FABP4 TBL1XR1 ADIG CTBP1 AACS CTBP2 PRDM16 SNAI2 SIRT1 PER2 PPARG

GO_SIGNAL_TRANSDUCTION_IN_RESPONSE_TO_DNA_DAMAGE A cascade of processes induced by the detection of DNA damage within a cell. CASP2 SFN PLK2 PIDD TP63 BAX HIPK2 NBN PRPF19 MDM2 BATF CCNB1 TP53 CEP63 CARM1 CDK1 CDKN1A CNOT7 BRCA1 C2orf29 PCBP4 CNOT2 TFDP2 E2F7 KAT5 GADD45A CDK2 CDC5L CDKN1B PLAGL1 SP100 WNT1 EP300 C13orf15 RPS27A ATM AURKA PAXIP1 TFDP1 BTG2 UBA52 USP10 CDC25C ATRX ARID3A RQCD1 MIF MYO6 GTSE1 CNOT6L CNOT3 PRKDC MDM4 FOXM1 BRCA2 SOX4 MAPK14 E2F1 TRIAP1 CNOT1 CRADD MUC1 BID ABL1 TNKS1BP1 SMC1A TP73 TFAP4 CASP9 UBB NPM1 UBC RPS6KA6 CENPJ NDRG1 PML CNOT10 ZNF385A PLK3 MAD2L2 PGAP2 PCNA FOXO3 RBL2 GRB2 GML CNOT6 CNOT4 ING4 PRMT1 SESN2 CHEK2 BCL3 CNOT8 E2F4

GO_NEGATIVE_REGULATION_OF_TELOMERASE_ACTIVITY Any process that stops or reduces the activity of the enzyme telomerase, which catalyzes of the reaction: deoxynucleoside triphosphate + DNA(n) = diphosphate + DNA(n+1). PPARG OBFC1 TEN1 TP53 TERF1 TINF2 MEN1 POT1 CERS1 CTC1 SRC PINX1 PIF1

GO_MONOCARBOXYLIC_ACID_TRANSPORT The directed movement of monocarboxylic acids into, out of or within a cell, or between cells, by means of some agent such as a transporter or pore. SLCO3A1 OSTBETA SLC10A2 SLC5A6 SLC16A5 ABCD1 SLCO1C1 SLCO2A1 STARD10 ABCB4 SLC25A20 DRD3 SLC27A4 ALB NMB FABP3 NCOA1 SLCO1A2 NOS2 ANXA1 NR1H4 PLA2G4F ABCB11 SLCO1B3 SLC16A7 PROCA1 FABP1 PLA2G2D SLC16A1 NMUR2 ACSL4 ACACA GOT2 SLC16A9 CPT1B BRP44L SLC16A8 SLC26A6 ATP8B1 SLC16A6 DRD4 NCOA2 ACE ABCD3 PLA2G2C SLC10A6 LCN12 ACACB APOE PNPLA8 FABP6 PLA2G1B PRKAG2 PLA2G4A BDKRB2 RXRA SLC10A3 SLC16A2 BRP44 ABCC3 SLC22A13 SLC16A11 LEP OC90 AKR1C1 SLC16A12 LOC347411 PLA2G3 SLC27A6 SLC16A4 BSG SLC9A3R1 PLA2G2F DRD2 PLIN2 PPARD SLC27A1 SLC16A13 SLC5A8 PRKAB2 PLA2G10 ACSL1 ABCD2 CEACAM1 SLCO1B1 SLC27A2 SLC10A4 CPT1A SLC16A14 EMB SLCO2B1 PPARA ABCC4 ABCC2 PLA2G12A AQP9 SLC5A12 PLA2G2A LCN1 SLC10A1 PLA2G2E PPARG CROT PLA2G12B SLC10A5 PLA2G5 SLC27A5 PRKAA2 SLC25A17 CPT2 OSTalpha MFSD2A SLC16A3 AKR1C4

GO_MITOCHONDRIAL_GENOME_MAINTENANCE The maintenance of the structure and integrity of the mitochondrial genome; includes replication and segregation of the mitochondrial chromosome. SESN2 MRPL39 DNA2 SLC25A36 MEF2A AKT3 CCDC111 MRPL17 PIF1 C20orf72 PARP1 MPV17 SLC25A33 RRM2B DNAJA3 OPA1 POLG2 C10orf2 LONP1 SLC25A4 C17orf42 POLG

GO_PHOSPHATIDYLETHANOLAMINE_BIOSYNTHETIC_PROCESS The chemical reactions and pathways resulting in the formation of phosphatidylethanolamine, any of a class of glycerophospholipids in which a phosphatidyl group is esterified to the hydroxyl group of ethanolamine. CEPT1 AGXT2L1 CHKA ETNK1 CHKB ETNK2 PHOSPHO1 LPIN2 SLC27A1 ALOX15 LPIN1 PISD LPIN3 PCYT2 EPT1

GO_MEMBRANE_HYPERPOLARIZATION The process in which membrane potential increases with respect to its steady-state potential, usually from negative potential to a more negative potential. For example, during the repolarization phase of an action potential the membrane potential often becomes more negative or hyperpolarized before returning to the steady-state resting potential. SLC26A3 PARK7 CFTR PRKCZ CRTC1 CASP1 KCNQ3 KCNA5 CACNG2 ATP1A1 ADIPOQ

GO_REGULATION_OF_ALPHA_BETA_T_CELL_ACTIVATION Any process that modulates the frequency, rate or extent of alpha-beta T cell activation. CD28 IL12RB1 LGALS9 IL12B CD244 CD300A CD80 HSPH1 ZAP70 RARA SYK HFE NLRP3 AP3B1 XCL1 IL18 AP3D1 IL6 TGFBR2 PRKCQ RC3H1 IL27 SHB SASH3 CCL19 RIPK2 ZBTB16 ZNF683 TNFSF18 PNP PRDM1 ITPKB IFNG FOXP3 CD86 TNFSF4 HLX CD3E SOCS5 SHH JAK3 ZBTB7B MYB IL23A EBI3 CCR2 ANXA1 LILRB1 GLI3 GATA3 IL4 NCKAP1L IRF4 BCL6 HLA-E IHH IL23R HMGB1 RASAL3 CD55 IL4R IRF1 TNFRSF14 ADA AGER PRKCZ CD83 NKAP IL12A

GO_SERINE_FAMILY_AMINO_ACID_METABOLIC_PROCESS The chemical reactions and pathways involving amino acids of the serine family, comprising cysteine, glycine, homoserine, selenocysteine and serine. AGXT CBS SHMT2 MTRR BAAT GLDC EIF4A3 SEPHS2 SARS GCLM GART SEPSECS MRI1 AHCY GCSH DAO SDS GCAT DHFRL1 CDO1 SHMT1 GCLC AHCYL1 SRR PSPH MTHFD1 DHFRP1 PSAT1 THNSL2 DHFR SLC25A32 CTH GLYAT PHGDH AHCYL2 TDH AGXT2 DMGDH AMT MPST APIP GGT1

GO_INORGANIC_ION_IMPORT_INTO_CELL The directed movement of inorganic ions from outside of a cell into the cytoplasmic compartment. This may occur via transport across the plasma membrane or via endocytosis. SLC9A5 TRPM2 SLC9A10 TRPV1 TRPV5 SLC9A11 STEAP2 STEAP3 SLC9A4 SLC31A1 SLC9A8 STEAP4 TFR2 PICALM SLC9A2 STEAP1B SLC9A6 SLC8A3 HFE SLC9A1 ATP2B4 SLC9A7 SLC9A3 STEAP1 TRPV6 TF SLC9A9

GO_HISTONE_H4_K16_ACETYLATION The modification of histone H4 by the addition of an acetyl group to a lysine residue at position 16 of the histone. MEAF6 PHF16 C12orf41 KAT8 MSL1 PHF15 ING4 KIAA1310 WDR5 MLL MSL3 OGT C2orf67 PHF17 MCRS1 HCFC1 PHF20 KAT7 MSL2 KIAA1267

GO_IMMUNE_RESPONSE Any immune system process that functions in the calibrated response of an organism to a potential internal or invasive threat. SUSD4 TRIL CCL7 SPON2 ITM2A IGHG3 IL1RL2 CD7 OSM CD8B RBPJ GBP2 IL31RA KIR2DS5 CSF1R PLCL2 LCP2 TRGC2 IL28A IL17A CD86 TRGV3 TNFSF18 IGJ UNC93B1 RELT C8orf84 FCGR3A CCR4 NLRP6 S100A12 TNFAIP8L2 VTCN1 RPL13A TNFRSF1A PAX5 FCGR1B CCBP2 IL1RN C4B SRMS STAR CCRL1 LY96 AIF1 FCN3 TRIM4 CCL5 KLRK1 KLRC2 ITCH C2 DEFB103B TNFRSF17 FCGR3B CLEC10A CD14 CLNK IGKV3D-11 IRAK1 DEFB105B WAS TRIM26 CD180 IP6K2 DEFB108P1 MEF2C SNAP23 DAPK1 FASLG CD79A CLEC4E IFITM3 SLC26A6 PIGR APCS IL28RA C3 POU2AF1 APP TYK2 PAG1 IFI27 TREM2 IGHG4 TCF12 PRKCB PYDC2 APOBEC3F TRIM56 NFIL3 SHMT2 EXO1 TRIM28 CXCL14 RELB POLR3F PSMB8 DEFB123 IL13RA2 TRIM25 MS4A2 DEFB129 CLEC4A TRDC IGKV2-40 HIST1H2BE IFNA2 BMP6 CX3CL1 RPS27A C13orf15 CD274 ZP3 FCER1G HIST1H2BI AZU1 C1QBP FBXO9 SEC14L1 EBI3 GZMA BPIFB3 MICB ZEB1 TNFSF10 IFNA17 CCR7 DEFB132 LILRB3 HIST2H2BE IL18BP NCR2 TLR2 TBK1 IGKV3D-20 IL1B RNASE7 SIGLEC14 CLEC2A APOBEC3A CRIP1 ISG20 MID2 APOBEC3G POLR3G LTBR IFNA14 LILRA2 FCGR1C CCL1 VAV1 RNF19B STXBP3 ITFG2 SLA2 DLL1 TNFRSF10A BTLA KYNU KLRG1 FCGR2B ZC3HAV1 BLNK SAMHD1 STAT2 PTGER4 CD58 RAB17 PLA2G3 NLRP3 MNX1 SBNO2 MBP IGHV1OR15-1 TGFB1 KIR3DL1 NMI IGHA1 RNF168 ZBP1 DEFB128 FAM105B TEC TRIM14 BST2 STYK1 HERC5 HIST1H2BF GNL1 IGKV4-1 IRAK4 PSMB10 C8G SLAMF6 SLPI CDH17 TIRAP NCF4 FUT7 IL15 IFNGR2 NFKB2 CYSLTR2 GSDMD IGHV1OR21-1 FAS LILRA4 LILRB4 FADD PLA2G6 S100A13 DEFB110 TRIM13 POU2F2 IGLC7 SIRT1 CYSLTR1 TBKBP1 YES1 MID1 DEFB113 HRH2 CCR10 IGLC3 IFIH1 TLR8 CCR8 CD1E ICOSLG LY75 FCN2 MASP1 BTNL8 IRF4 CCL18 DEFB115 LIME1 CD1B VSIG4 CD70 IL32 TRIM11 PVRL1 GPR65 NFKB1 FYB CCL24 TRIM15 IFNA6 EIF2AK4 CTSW IL1A SARM1 EIF2AK2 IFNA8 AKAP8 H2BFS SEMA3C SMAD6 IRF7 CXCL5 POLR3K NLRP2 KIR2DS1 IL24 TLR9 STXBP2 NCR3 IL1RL1 NOS2 NOTCH1 IFI6 HLA-DQA2 UBA52 CYP11B1 FYN TRIM35 CADM1 CX3CR1 GAPT RORC IL7R RAB27A IL6R C4A LCP1 VTN CTSC CST9 TRBC1 IGHV3-23 IL27RA PIK3CG POLR3D LTF OASL TNFRSF14 TNFSF15 KLRC4-KLRK1 NEDD4 C5 AIRE MALT1 APOA4 DEFB4A BCL3 CR1 DEFA1B CCL16 TNFRSF8 DDX58 IRF6 EGR1 XCL2 IL21 XBP1 VCAM1 PPARG PGLYRP2 SECTM1 VAMP2 IL27 VIPR1 KLRD1 SIGLEC16 CHIA AICDA IFNA1 KLK3 VPREB1 CAPZA2 DEFA3 IFNA10 RFTN1 CLEC6A IGLV1-51 LILRA1 IGLV7-43 TRIM22 TOLLIP IFNGR1 TRIM27 CXCR2 CAMK2D C4BPA S100A9 TDGF1 CD40 HLA-DRB5 GPR44 APLN UBB GZMH UBC CRCP TRIM32 DDX3X APOBEC3C GBP6 ADAMDEC1 ANXA3 CD1C CRISP3 C1QA C8orf4 CEBPG TNFRSF13C APOBEC3H HIST1H2BG IGLC1 GZMM ERAP1 RORA CCR1 IKBKG FES GAS6 IL33 TLR5 IRF5 SYK SH2D1A POLR3B GPI MLH1 DLG1 SEC61A1 INPP5D TNK1 RPS6 DMBT1 NCF2 HCK GTPBP1 RSAD2 CLEC4D IGKC TNFRSF1B POLR3A CHGA C4BPB SP2 DHX58 PRKD2 IL5 IRGM PGLYRP3 LIF IL36A C19orf29 CXCL10 HLA-F TRIM68 CYBB CAPZA1 TYROBP IL13 BMPR1A FCGR2C F2RL1 DENND1B CHIT1 LAMP3 CAMK2G ABL1 CXCL16 SEMG2 ADAMTS13 ARHGEF2 PRELID1 JAK2 CCL25 RAET1L CAMK2B DEFB107A CD1D ICOS PTPN6 TRIM62 PTK2B HMGB1 CFH C7 COLEC10 NOTCH2 LOC440786 HLA-DMB LGALS3 RAG1 SIGLEC15 PTX3 EPRS LILRA5 CXCL3 PPBP ZAP70 DEFB124 HMGB2 IRG1 SKAP1 PYCARD PADI4 IGSF6 CHST4 IL12B CD1A CCL21 LAIR1 HLA-DRB1 MSH6 IGHG2 CCL3L1 DEFB118 HLA-DPA1 C1RL VPREB3 HLA-DOA PIK3CD IL1RAP RNF8 LST1 FGA TRAF6 MARCH1 BCL10 PRG2 EFCAB4B RC3H2 CFHR1 MIF ITGAD S100A7 RAET1E IRF3 DEFB106A IGHV4OR15-8 DEFB104B DAPK3 TREML1 PCBP2 SMPDL3B GBP5 IFITM1 MASP2 LBP CCL13 OAS2 CD36 DEFB116 IGHM PRF1 C5AR1 NOD1 EDA DOCK2 LGALS1 IFNA13 ODZ1 LILRA3 IL18 DEFA5 MYO1G IFNB1 SERPINB9 ABL2 NBN DEFB135 BPIFB1 RAET1G SWAP70 ENPP2 FCGR1A SH2D1B CHID1 IL8 IRF2 IK TRAF3 DBNL KLRF2 TLR6 DEFB112 MSH2 OTUD7A IGKV3-20 PF4V1 S100B PDCD1 IL34 GPER LEF1 RGS1 IL17B CCL8 CFP CFI IL18R1 CITED1 LY9 ORAI1 DEFB103A AKIRIN2 PTK6 TNFSF11 MRGPRX2 CXCL11 KIR2DS4 CD83 IL9 TNFSF12 MFNG IL22 CLEC7A HLA-E BCL6 EMP2 SH2D6 DDX60 C9 IL25 LCK TUBB4B GCH1 HAVCR2 IFNW1 KIR3DS1 JAK1 PI4K2A HLA-G MILR1 IPO7 MR1 IFNK CXCL2 IL36B LCN2 TRAF3IP2 TNK2 RIPK2 SFTPD CCL20 SEMA4D IL3 APOBEC3B IKBKAP HLA-H SRPK1 DAK C6orf57 C1S EMR1 DEFB114 TICAM2 CD46 GAPDH IL12A LRRC33 ILF2 CLEC4M FOXP1 TNFRSF13B KDM5D HRAS IFNA16 ULBP3 AXL IGKV1D-33 CLU KAAG1 ECSIT TNFRSF21 ENDOU HSPD1 CNIH PPP1R14B PSTPIP1 EXOSC9 ITGAL CCL3 CSF2 RNASEL LGALS9 FCRL4 CAMK4 NLRP2P FOXP3 IL20RB TRBV12-3 ALCAM TNFRSF10B DEFB126 CFB FCN1 IFNG SEMG1 CCL15 TRIM21 SPN BTN3A2 C6 IFIT1 CD276 SP100 CALCOCO2 HLA-DRB3 TRIM10 ADAR CEBPB IGLL1 LTB SIRT2 IL23A IFNAR1 KDM6B TNF IL37 IL16 DEFA4 ZBTB1 CRHR1 TLR7 CD74 BPIFA1 UBD JAG1 IGHE MS4A1 WNT5A PRG4 IFNA4 MRC1 CIITA SNCA ATP6V0A2 XAF1 CAMK2A ZFPM1 APOBEC3D GBF1 TNFRSF11B FRK MEFV CARD9 PRG3 IL7 LAX1 DAB2IP NKX2-3 HLA-DPB1 PSEN1 COLEC11 CCL4L2 NAIP TINAGL1 CTSH TXK OTUB1 MARCH8 ERCC1 LAT2 IFNAR2 HIST1H2BK ICAM1 TNFSF8 IGF1R HLA-DRA DEFB131 DEFB125 IGHD AQP9 HLA-DOB SERPINC1 EXOSC3 MT2A OTUD7B KLHL6 TRIM38 CCR5 BLK THEMIS SLC30A8 TINAG HLA-A ISG15 EXOSC6 CCL26 ADAM17 ANG ENPP1 MB21D1 TFEB TCF7 CCL11 CNPY3 DEFB133 IKBKB IRF8 CD300E STAT1 SLC11A1 ITK CD164 IKBKE AGER IL23R DEFB1 PGLYRP4 RFX1 AIM2 SUSD2 CST7 ATG12 DEFB127 IL6 TREM1 PKHD1L1 TRIM31 CCL3L3 MYD88 TLR3 C8B CD27 IFNA5 CD8BP CLEC4C IGKV1-5 PNP PRDM1 LYST LTA TRIM34 IRF9 TAP1 RC3H1 KRT1 ANXA1 DEFA1 LILRB1 ENPP3 POLR3C EOMES SERINC5 CCL4 IL2 CORO1A NLRP10 CPLX2 TNFSF4 CSF1 CXCL6 BCL2 IFI35 GEM PLA2G1B OAS3 MAP3K14 DEFB121 PML DEFB105A LILRB5 IGLL5 SAA1 ATG5 REL TLR4 HAMP S100A8 PGLYRP1 BTN3A1 BMX MX1 MAP3K5 CLEC5A C1QB PRKCD LGR4 PXDN LILRB2 XCL1 CD44 CNR2 SMAD3 CCL17 TNFSF14 DEFA6 CD244 CD4 MADCAM1 FGR LIG4 ELANE CXCL1 LTB4R BATF SYNCRIP FFAR2 MX2 HLA-DQB1 NCF1 PYDC1 CYBA TNFRSF25 ERAP2 TRIM5 NGFR C19orf66 OAS1 TNFRSF4 FTH1 HLA-DMA IL2RG C1R ULBP1 HLA-DQA1 CSF3 IGLC2 CXCL13 CXCL12 LFNG IGLC6 TRPM4 ADA ELF4 VAMP7 IL28B CD209 IL4 CASP4 CR2 TLR1 IGHG1 JAM3 RPL39 CFHR5 CFD NLRP1 CSK COL4A3BP PTAFR IL12RB1 CMA1 IFNA21 TSC1 HIST1H2BC MICA IL36G TAP2 CCL14 NPFF CCL19 TNFRSF11A CEACAM8 PRKRA ANKRD17 PLCG2 CD40LG MAVS BTK PRKD1 IL10RB VAMP8 CMKLR1 SIRPB1 DEFB108B TAPBP SEPX1 CCR2 IL36RN TNFRSF10C TNFRSF10D CD79B IFNE IL1R2 TGFBR3 FGB RNF135 TNFSF13 ANKHD1 BTN3A3 LY86 CTLA4 CD55 PRKCE HLA-B IL29 LILRA6 ASS1 SIT1 HLA-DQB2 KIR2DL3 C8A NCAM1 NLRX1 RNASE3 MARCO FCAMR B2M NOD2 POLR3H PDCD1LG2 MST1R KIT TRAT1 ZNF683 LYN SEMA7A YTHDF2 IL5RA IFI16 NR4A3 TMIGD2 DPP8 HLA-DRB4 SEMA4A PF4 FFAR3 IGHA2 CHUK TLR10 S1PR4 TRIM8 SLAMF7 CCR6 IRAK1BP1 IL19 APBB1IP CD300LB GBP1 SRC OPRD1 GCNT3 IFITM2 JAK3 UNC13D CTSL1 NUB1 BST1 IFIT2 MYLPF CCL28 MBL2 HIST1H2BJ NLRC4 CCR9 RNF125 CXCL9 IL4R VNN1 IRF1 RASGRP1 TMEM173 KRT16 CRTAM CAMP IFIT3 GATA3 DEFB119 FOXJ1 ADAM15 CYP27B1 CD6 MATK KIR2DL1 C1QC CD96 DEFB107B NLRC5 TNFRSF18 SSC5D IGHV2-5 PTK2 CXCR5 AQP4 TFE3 CD8A THBS1 IL2RA CD28 CTSS IL10 ULBP2 IL26 CCL27 IFI30 IL1R1 IFIT5 SERINC3 APOL1 HMHB1 DEFB104A EDN1 ADM TNFAIP1 IL18RAP CCL2 TUBB AHCY NR1H4 FCAR PRDX1 ADARB1 CTSG POLR3E TICAM1 SLAMF1 SERPING1 ST6GAL1 MAP4K2 FER COLEC12 TNFRSF6B F12 CCL22 CD84 OPRM1 KIR2DS2 SRPK2 HMGB3 ATP7A DEFB134 CD97 TNFRSF9 GPR183 BPI DEFB106B TNFSF13B CCL23 IFNA7 STAT6 PNMA1 DCLRE1C OPRK1 GZMB LAT ETS1 IL20 HLA-C TNFSF9

GO_REGULATION_OF_GASTRULATION Any process that modulates the rate or extent of gastrulation. Gastrulation is the complex and coordinated series of cellular movements that occurs at the end of cleavage during embryonic development of most animals. OTX2 SFRP2 BMPR1A PHLDB2 PHLDB1 IL10 HNF4A CLASP1 FOXA2 DUSP6 IL1RN ODZ4 OSR2 OSR1 SCXB DAG1 TGIF2 GNB2L1 NODAL KLF4 SOX17 COL5A1 DKK1 APOA1 COL5A2 MYADM ADIPOQ FZD7 LHX1 MESP1 HNF1B MAP2K5 CLASP2 MAPK7 WNT3A FGFR1

GO_NUCLEOBASE_METABOLIC_PROCESS The chemical reactions and pathways involving a nucleobase, a nitrogenous base that is a constituent of a nucleic acid, e.g. the purines: adenine, guanine, hypoxanthine, xanthine and the pyrimidines: cytosine, uracil, thymine. APRT TYMS ADA RRM1 UCK2 CPS1 PAICS TYMP ADAL GDA ACPP AOX1 HPRT1 KDM1A UMPS PRHOXNB GMPR2 SHMT1 UCKL1 CAD XDH CECR1 PPAT GMPR PRPS1 GMPS DHODH DPYD DPYS GART CDA CMPK1 MAPK1 UPRT PRTFDC1 ALDH6A1 TET2 TTR UCK1 MTOR

GO_DORSAL_VENTRAL_AXIS_SPECIFICATION The establishment, maintenance and elaboration of the dorsal/ventral axis. The dorsal/ventral axis is defined by a line that runs orthogonal to both the anterior/posterior and left/right axes. The dorsal end is defined by the upper or back side of an organism. The ventral end is defined by the lower or front side of an organism. WNT3 CTNNB1 EGF RGS20 RGS19 SMAD2 VAX2 CXXC4 BMPR1A AXIN2 LRP5L SFRP1 SMAD6 SENP2 PAX6 FZD5 LRP6 MDFI LRP5 AXIN1

GO_TOOTH_MINERALIZATION The process in which calcium salts are deposited into calcareous tooth structures such as dental enamel, dentin and cementum. CNNM4 FOXO1 FAM20C MSX2 TBX1 WDR72 AMELX ALPL FAM20A COL1A1 STIM1 PVRL1 PPARA

GO_EMBRYONIC_MORPHOGENESIS The process in which anatomical structures are generated and organized during the embryonic phase. The embryonic phase begins with zygote formation. The end of the embryonic phase is organism-specific. For example, it would be at birth for mammals, larval hatching for insects and seed dormancy in plants. BAX PCDH8 COL11A1 DVL1 PSEN1 SLC9A3R1 MAPK1 FRAS1 DLL1 STRC LATS1 HHEX C5orf42 NEUROD1 GATA4 RIC8A ZBTB16 STIL ZIC1 CCDC40 CEP290 LAMA3 WNT11 TBX15 DLX2 TGFB1 TGFBR1 LHFPL5 GDNF COL2A1 ATP8A2 STK4 PDZD7 FOXF1 IFT172 FUZ SPINT2 HLX SP9 RARB RPGRIP1L PITX2 BMP7 SIX4 LMBR1 PBX2 LRP6 MMP15 ITGA2 T KIF20B ACVR2A POU5F1 EYA1 VAX2 DLL4 SETDB2 SMAD4 HIPK1 NF2 LRIG3 MESP1 SOX18 TCF7 EPHA2 KIAA1715 WNT3A IFT57 MMP2 GNA12 PAX6 NDRG4 HOXC11 NEUROG1 ARHGAP35 HTR2B GRHL2 TRIM15 HOXC9 SOX2 CDKN1C SPINT1 ODZ4 NAT8B IMPAD1 SLC39A3 GJA1 SP8 HOXB4 LAMB1 WLS RAC1 CHRNA9 TULP3 NOG LUZP1 BMP4 HOXD9 DLD MICAL2 RYR2 CRABP2 ARID1A SOX4 BCR FZD2 SFRP1 ITGB2 GDF3 ECE1 VTN NCOA1 TBX2 WNT9B RARG TH NOTCH1 VASP TBX1 EOMES HES5 ITGA7 HIF1A GNB2L1 GRB2 IRX5 HMGA2 MMP16 HOXA5 TAL1 MKS1 HOXD3 IFT52 SLITRK6 ACD DSCAML1 TMED2 HIRA CACNA1C AXIN1 NKX2-5 GRSF1 SPEF2 ID2 MMP9 MTHFD1 SIX2 ALX3 TBX3 EPB41L5 PAX5 DLX5 HPN ZIC3 ATP2B2 HNF1B ZBTB17 APLNR HOXB5 DVL3 GRHL3 FGF8 BBS5 HOXD12 TSC2 MKKS HMX3 FOLR1 HOXB6 HOXA11 KAT2A HAND1 ARL13B GSC AMOT WNT6 ITGA4 NOTO PCGF2 CYP26B1 KLF4 ZAK LHX2 VANGL2 GNAS CTHRC1 GATA2 RPL38 MEF2C HOXA2 WNT7A BBS4 SMAD1 COL7A1 PROX1 NPHP3 OVOL2 CUL3 TSHZ1 HOXA10 GREM1 TRIM28 SOX7 ALX4 RPS7 WNT4 ITGA8 ATOH1 OSR2 FZD5 GLI1 YAP1 EXOC4 IRX2 HOXB1 SOX17 FN1 EXT2 POU4F3 KIF16B GDF5 JAG2 RUNX2 TCTN1 LHX1 ARFRP1 B9D1 DVL2 WNT9A SFRP2 USH1G ZEB1 FRS2 FZD3 TLX2 MFAP2 ATP6V1B1 GATA6 C2CD3 HOXA9 PTK7 FBN2 RET RSPO2 DNAAF1 VEGFC HYAL1 KDM6B GLI2 HOXA6 PAX2 HOXC10 SEC24B LBX1 PDX1 BMP5 SIX3 ACVR1 WNT5A CDC73 STK3 MBNL1 HOXB2 NTN1 ITGB3 PAX8 PLXNB2 HOXB3 DYNC2H1 TP63 DUSP5 MTHFD1L FOXA2 PRKACB HIPK2 PFN1 LDB1 HES3 ITGB4 MEGF8 NODAL SMO NR4A3 SOCS3 COL8A1 TRAF6 ITGAV WNT1 CFC1 HDAC1 TGFB2 FLRT3 EFNA1 TFAP2A MSX1 GREM2 ALX1 PAF1 ACVR2B C1orf172 SHH RECK IFT122 RSPO3 SLC39A1 LMO4 CRB2 MIXL1 BCL10 WDPCP EPHB2 MDFI PKD2 PITX1 KDM2B TXNRD1 FBN1 NDST1 ALDH1A3 HOXA3 HOXB7 IHH SOBP TRAF3IP1 ALDH1A2 FLVCR1 PPAP2B DKK1 WNT3 FGFR2 GATA3 UGDH CDON ITGA3 RALA SATB2 DUSP4 POU3F4 SUFU SDC4 HOXD13 PRKACA RARA STRA6 SOX11 IL10 OTX1 SALL1 EIF4A3 AFF3 CTNNB1 SRF SCRIB PTPRQ TWSG1 COL6A1 EN1 WNT16 ITGB1 SYF2 SALL4 ADM EDN1 RNF2 SHOX2 MYCN TBX6 FOXE1 EXT1 HAND2 TGFB3 ZFP36L1 HES1 GDF7 ITGB5 HESX1 CLIC5 HDAC2 HSBP1 DUSP2 LEF1 EYA2 TBX5 SETD2 FOXC1 GJB6 DEAF1 CHD7 FOXC2 CTR9 GNAQ ZNF281 AHI1 FGFR1 FGF9 SPRY2 EFEMP1 LIAS SMAD3 FZD6 MMP8 CFC1B SMAD2 MYH3 FLT1 SOD1 FOXH1 WNT7B WDR19 LAMB3 LEO1 NAGLU CCDC103 RTF1 LRP4 TMEM107 MAPK3 ATOH8 TWIST1 TEAD2 TCF21 GJA5 MED1 PBX1 SIX1 IRX1 ST14 CC2D2A RDH10 COBL OTX2 MYO7A NIPBL WNT8B HOXA1 HMX2 CELSR1 GLI3 SP3 SOS1 IRX3 FGF4 PDGFRA MED12 LATS2 DNAJB6 BBS7 TRIM71 CHST11 BMPR2 NANOG ZNF358 GLMN CHRD DLX6 GPI POFUT2 ROR2 DUSP1 RBP4 TGFBR2 ABR HOXA7 C6orf170 TMIE OSR1 PTCH1 GJB5 SHROOM3 MYO15A SKI FOXF2 COL12A1 DACT1 HOXA13 HOXC4 GCM1 ETS2 PRKRA FRZB MAFB FOXL2 SNAI1 ENG CYR61 INHBA CREBBP INSIG1 PRKAR1A MYF5 FZD1 INTU CER1 LRP5 DLG1 PRICKLE1 MMP14 TSC1 FBXW4 RPS6 TCAP TBX4 BMPR1A FGF2 GPC3 PRRX1 SEMA4C HOXB8 ETV2 FGF10 MAB21L2 MSX2 C2orf49 NKX3-2 LAMA5 LRIG1 GBX2 APAF1 BCL2L11 RBPMS2 ELF5 DLC1 HOXD4 TBX20 INSIG2 PHACTR4 SCXB KDM6A MIB1 CCDC39 ITGA5 LOC100506013 HOXD10 SOX9 CHRNA10 IPMK FAM48A KCNQ4 FOXN4 FOXI1 COL4A2 SOX8

GO_EXTRACELLULAR_MATRIX_ASSEMBLY The aggregation, arrangement and bonding together of the extracellular matrix. ATP7A TGFB1 HAPLN2 MYH11 PLOD3 TNXB HAS2 MFAP4 GAS6 HAS1 HAS3 FBLN5 THSD4 GPM6B RAMP2 LOX

GO_RESPONSE_TO_PURINE_CONTAINING_COMPOUND Any process that results in a change in state or activity of a cell or an organism (in terms of movement, secretion, enzyme production, gene expression, etc.) as a result of a purine-containing compound stimulus. TYR SEPN1 PCK1 ADSS CDO1 SLC26A6 SPARC SLC8A1 EPB49 CPS1 TRPV1 PPARGC1A RELA STAT1 JUNB HCN1 P2RX6 RYR1 KCNE1 THBD RAP1B AANAT GSTM2 DNTT IGFBP5 BRAF CRHBP STAR DGKQ OXT ITPR2 REN HDAC1 PDXP RAP1A P2RX3 RAPGEF2 APEX1 RAPGEF3 AQP9 SLC6A3 FOSB DUOX2 SRD5A1 EZR PPARG JUN PTPRN PLA2G5 P2RX5 RPLP0 AACS SLC5A5 P2RY11 SOD1 NOX4 TRPC3 PTGS2 HCN4 FOS FOSL1 HMGCS1 RAPGEF1 P2RX4 ASS1 AKAP6 COL1A1 TRPM2 PDE2A CREM P2RX7 PTEN SLC26A3 AQP8 KDM1A CNGA3 SSH1 PTK2B PIK3CG IL1B ADIPOQ MMP19 CITED1 AREG HSP90B1 HCN3 BIRC2 KCNJ11 KCNQ1 ZFP36L1 HCN2 VGF GPD1 PKLR SLC8A3 AGXT PRKAA1 NDUFS4 WT1 INPP5K CFTR P2RX2 HDAC2 PPARGC1B CASP1 CIB2 PENK CCL2 PFKFB1 STC1 DUOX1 SLC6A1 SDC1 DNMT3B CARM1 JUND AQP1 LDHA RYR2 WNT10B PAX4 CASQ2 SELL RYR3 TEK PDE3A PANX1 ALDH3A1 DUSP1 BSG PTAFR SREBF1 P2RY12 PER1 NME1 P2RX1 CAD APP AKAP9 IL6 HSPD1 DHODH EEF2K SLC6A4

GO_POSITIVE_REGULATION_OF_SEQUENCE_SPECIFIC_DNA_BINDING_TRANSCRIPTION_FACTOR_ACTIVITY Any process that activates or increases the frequency, rate or extent of activity of a transcription factor, any factor involved in the initiation or regulation of transcription. MAP3K7 ANXA3 RNF41 NOD1 EDA CEBPG MTPN AIM2 IL4 PRKCH PPAP2B CLU IKBKB VEGFA ARID5B TRIM13 IRAK1 NHLH2 UBC PPARGC1A UBB RELA TRIM26 TERF2IP NLRC4 AKT1 CYTL1 TRIM32 RIPK3 WNT3A AGER PARK7 PRKCI TRIM27 SHH TAB3 OPRD1 RPS6KA4 TP53BP1 CD40 INS S100A9 DDR2 ABRA MYOCD RHEBL1 PPP2R5B TRIM5 NFKB2 UBE2V1 CHUK BCL10 TRIM38 TRIM8 TAB2 TRIM22 LRP6 CARD11 NODAL IRAK3 SMO RIPK2 CFLAR IL1RAP S100A12 CTH TRAF6 ERC1 WNT1 TIRAP TRAF2 CLOCK HDAC4 PPARG TGFB1 EPHA5 ESR1 ATF2 CRTC3 TRIM14 AMH ICAM1 TNFSF18 SYT14L RAB7B TRADD NOD2 NLRP3 FOSL1 PINK1 PSMA6 JUP PYCARD CARD14 MAP3K13 TCF3 KIT TRAPPC9 FZD4 HIPK2 ARHGEF5 NKX6-1 CAPN3 NFKBIA NEUROD1 CAMK2A CIB1 RNF25 NFAM1 SPHK1 ESR2 DDRGK1 DDX58 KDM1A MID2 TLR4 TRAF5 S100A8 NEUROG2 TRIM62 RIPK4 PPRC1 TRIM37 MALT1 WNT5A ALK NKX2-2 STK3 CRTC1 PTEN RELN NEUROG3 SAV1 NEUROD2 ZIC2 NPM1 TNFSF11 LRP8 PLA2G1B RIPK1 ARHGEF2 LTF NFKBIB HDAC5 STK36 PRKCZ PHB2 IL1B JAK2 TLR2 ITGB2 TNF KRAS CAT RNF31 IRAK2 FZD2 AGT FER TAF12 RPS6KA5 NTRK1 FOXA1 SMARCB1 PPARGC1B UBA52 PPP3CA TICAM1 MTDH TLR9 ADAM8 AR TRIM34 EDN1 HSPA1B TRIM21 TNFRSF11A MAVS PRKCQ CD40LG PRKD1 PRKD2 RPS27A C13orf15 IL5 BTK EP300 LRP5 TRIM25 DVL2 FZD1 EIF2AK2 EDA2R CRTC2 WNT10B WNT2 NFKB1 TRAF1 BEX1 TLR3 LRRFIP1 SRF CTNNB1 BUD31 GREM1 LGALS9 NEUROG1 CAMK1D TRIM15 IL10 GTF2A2 MBTPS2 TRIM52 SMARCA4 RBCK1 TAB1 HSPA1A IL6 PRDX3 TRIM31 JMY IKBKG UBE2N NDP TSSK4 PIDD PRKCB MYD88

GO_ORGANONITROGEN_COMPOUND_CATABOLIC_PROCESS The chemical reactions and pathways resulting in the breakdown of organonitrogen compound. NOS2 ACAT1 AHCY DDO FTCD C9orf3 GLUD2 LRTOMT PRODH2 ACY1 LNPEP NPEPPS PAOX OGDH ECE1 HMOX2 HSPG2 HNMT HYAL4 ACADL PM20D1 HMMR PPT1 AUH AGPHD1 GLUD1 NT5C CRYM MPST LTA4H AMDHD1 NEIL2 PGLYRP1 ACER1 UPP1 AGXT2L2 ACMSD SMOX PDE7B PPM1K ADO ASPG SDC2 BCAN NEU2 BLMH PDE1A DAO PDE4D GPC2 NT5C2 GLB1 ALDH6A1 NEU3 PDE11A HPSE SMUG1 ALDH1L2 CHAC1 CNDP2 SAT2 HMOX1 PDE3A ACAD8 DLD DHPS ALDH1L1 BLVRB CHID1 PNP SDC1 SHMT1 HPRT1 UNG TRHDE HPSE2 HINT1 CDA TET1 ABAT FMOD OAT MBD4 UROC1 PDE7A DBH BCKDHB LYVE1 DDAH2 NUDT9 DTD1 PDE9A VCP OVGP1 AQPEP PRTFDC1 STAB2 GLS HGSNAT SMPDL3B FUCA1 CHI3L2 NUDT4 PDE4B MTHFS NCAN AMT GM2A SDC4 MAOA CDO1 PGLYRP4 SLC9A1 HEXA NEIL1 LYG1 HAAO HIBADH SGSH SAT1 DERA DIO3 CYP3A4 TPP1 UPB1 NUDT11 CECR1 GPC4 GCSH ASL KYNU TET3 SMPD4 HAL CBS PDE8B VCAN SAMHD1 BCAT2 SARDH NTHL1 OGN NT5E NT5C3 CTSH TGFB1 APOBEC1 TDH NEU1 NUDT15 IDS ENTPD4 MCCC2 GDA SMPD1 NUDT1 THNSL2 ARG1 GSTZ1 GPC5 COMT PRODH HDC NEU4 ASPDH CYP3A5 CSPG5 DPYS PGLYRP3 CARNS1 GPC1 HYAL1 FGF2 GBA2 PIPOX AGXT GPT GPC3 ACE2 CAT RNPEP CPN1 ACAN HYAL2 AMBP GCDH CHIT1 NUDT18 ASRGL1 NUDT12 ADAMTS13 MTAP KIAA1199 BHMT SLC25A21 GNS ASPA FAH ENPP4 BLVRA AFMID MCCC1 CHAC2 APOBEC3G SLC44A1 GPT2 GOT1 GOT2 APOBEC3A AGRN CTBS CHI3L1 NOS3 LYG2 DPYD CHP ERAP1 TDO2 APOBEC2 ABHD12 NT5C1B GLS2 GCAT PAH PDE3B NUDT3 TET2 PDE4C HEXB IDO2 OGG1 AASS MTRR A2LD1 APOBEC3F UPP2 KERA IDE GBA3 AGXT2 PDE4A PDE8A NUDT10 GALNS BGN CYP2D6 PDE10A SPACA3 HIBCH SMPD3 IVD ADAL GALC OTC ALDH5A1 ENPP7 XDH CSPG4 HPD NT5C1A QDPR BCKDK SDS HSD17B10 PCYOX1 TST HOGA1 DLST DDAH1 GLUL PDE5A GLDC AADAT GLA AMPD3 MAOB ARSB KMO ENPEP RNPEPL1 TAT ADA DBT HMGCL GGT1 IDUA HYAL3 ALDH4A1 LUM ENOSF1 GBA CNDP1 NOS1 APOBEC3C C14orf126 APOBEC3H ACE PRELP PGCP GPX1 ANPEP TYMP ACADSB BCKDHA PDE2A DCN NAGLU SPHK1 DUT NT5M NUDT16 CHDH PPAT NUDT7 ALDH7A1 MAT1A LOC440434 UGT1A1 SMPDL3A CD44 IDO1 BCAT1 ALLC SATL1 PCYOX1L PDE1B ITPA TDG GAD1 PGLYRP2 SGPL1 GUSB GPC6 PCBD1 SDC3 OMD DMGDH CCBL1 CHIA UGT1A4 XPNPEP1 SLC6A3 GAD2 AOX1 HGD ERAP2 CTH IL4I1 AICDA

GO_SMALL_MOLECULE_CATABOLIC_PROCESS The chemical reactions and pathways resulting in the breakdown of small molecules, any low molecular weight, monomeric, non-encoded molecule. FTCD DDO PEX2 CPT1C INPP5K PHYH HAO1 GLUD2 ADH5 CYP39A1 AHCY FBP1 NOS2 ACAT1 CBR3 EHHADH ALDH1A1 PFKP ACADL INPP1 NUDT19 HNMT CYP7A1 AMDHD2 PEX7 PKLR PRODH2 OGDH ABCD1 AKR1C3 SYNJ1 GK GLUD1 SCARF1 CRYM GPD2 ALDOB AGPHD1 ADIPOQ HK3 AUH CEL ABCD3 CYP46A1 PGAM1 FUT4 FUT10 AMDHD1 ACAD9 SCP2 BLMH SLC27A2 DAO ACMSD AGXT2L2 ADO PPM1K ASPG AKR1A1 PFKFB3 GK2 ALDH6A1 RBKS GLB1 ALDH1B1 XYLB FGF23 CRYL1 KHK ACAD8 DLD TKTL1 ETFB SHMT1 FAAH HADHA CYP4F11 LPIN2 BCKDHB LPIN1 HSD17B4 UROC1 PEX13 ALDH3B2 ALDH3A2 DDAH2 ALDOC PGM2 OAT INPP5E ABAT NUDT5 ACAA2 GLS PGM5 SLC27A4 ACADVL NUDT9 DTD1 FUT9 ETFA MTHFS AMT SRD5A3 TPI1 ACAA1 ENO1 CYP27B1 GNPDA2 HIBADH ACADM C17orf48 PFKL CDO1 BAD HADHB HAAO NPL ASL PON1 GCSH KYNU BDH2 ABCD2 ACOT8 GK5 C9orf103 HACL1 ACAD11 GALT BCAT2 PCCB ACSF3 CBS HAL PNKD AKR1B10 RENBP PLA2G15 ALDH2 AKR1D1 TDH FUT5 CYP4F12 CROT ACOT4 NAGK FUT7 RPE GSTZ1 THNSL2 NUDT1 ARG1 NUDT15 HADH LIPE ENO3 PFKFB4 QPRT MCCC2 PGAM2 ECI2 PKM2 MIOX CARNS1 DHDH GNPDA1 HDC PRODH GCDH ALDOA ASRGL1 NUDT18 CYP26C1 AMACR GPT PIPOX AGXT HK2 OXCT2 ACOX2 ADH4 ACOX1 AFMID SULT2A1 MCCC1 ACAD10 ASPA FAH MTMR7 SLC25A21 APOE ADH7 PTEN CYP1A1 MMAA FUT8 NOS3 FUT6 SESN2 CPT1B SNX17 GOT2 GOT1 GPT2 CYP4A11 GLS2 NUDT3 PAH GCAT PCCA GALE ENO2 TDO2 GPI PDDC1 FBP2 OXCT1 PPARD SCARB1 MTRR INPP5A AASS IDO2 CYP24A1 MT3 AGXT2 GALM ESD SLC25A17 GCK LRP5 MUT HIBCH DCXR GALK1 PFKFB1 PFKM IVD GLYCTK ACAT2 CRABP1 PRHOXNB SORD HPD ECI1 DAK SDS BCKDK QDPR ECHS1 ACOXL CRAT OTC ALDH5A1 BDH1 GLDC AADAT PFKFB2 ECH1 PON3 HSD17B10 CYP4F2 CYP4F3 GLUL DDAH1 PGM1 DLST HOGA1 ETFDH DBT PARK7 TRERF1 TAT HMGCL NTSR1 GAPDH KMO HAGH C14orf126 BCKDHA ACADSB CYP26B1 NOS1 ENOSF1 ALDH3B1 ALDH4A1 IMPA1 PPAT MAT1A CPT1A ALDH7A1 LPIN3 IMPA2 NUDT16 FUT2 ACADS PGK1 GAD1 MCEE LEP MTMR2 ECHDC2 BCAT1 IDO1 PCBD1 CPT2 CCBL1 FUT1 CYP26A1 ACOX3 LOC729020 GAPDHS IL4I1 CTH GAD2 DECR1 HK1 HGD

GO_POSITIVE_REGULATION_OF_FATTY_ACID_BIOSYNTHETIC_PROCESS Any process that activates or increases the frequency, rate or extent of the chemical reactions and pathways resulting in the formation of fatty acids. APOA1 NR1H2 RGN APOC2 APOA4 NR1H3 AVPR1A APOA5 AVP MID1IP1 SLC45A3 PTGS2 ANXA1 MLXIPL

GO_RESPONSE_TO_GLUCAGON Any process that results in a change in state or activity of a cell or an organism (in terms of movement, secretion, enzyme production, gene expression, etc.) as a result of a glucagon stimulus. GLP2R ADCY3 GNG7 PRKAR2B GNB2 RPS6KB1 PRKACB ADCY4 ADCY5 SREBF1 CYC1 PRKACA GNG12 GCG ADCY6 GNG8 QDPR GNG5 GNB4 CRY1 ASS1 CCNA2 GNG2 PCK1 ADCY2 GLP1R PRKACG PRKAR1A PFKFB1 ARG1 PRKAR1B GNG10 CDO1 ADCY8 CREB1 GCGR GNB3 GNG3 GNG13 ADCY7 ADCY9 ADCY1 PRKAR2A GNB1 CPS1 GNG4 GNG11 FGF21

GO_LENS_MORPHOGENESIS_IN_CAMERA_TYPE_EYE The process in which the anatomical structures of the lens are generated and organized. The lens is a transparent structure in the eye through which light is focused onto the retina. An example of this process is found in Mus musculus. TDRD7 EPHA2 TBC1D20 PVRL3 SKI PVRL1 PITX3 PROX1 SOX11 SOX1 SHROOM2 SIX3 CRYGB BMP4 HIPK1 MEIS1 HIPK2 CTNNB1 BCAR3

GO_MICROTUBULE_CYTOSKELETON_ORGANIZATION_INVOLVED_IN_MITOSIS Any microtubule cytoskeleton organization that is involved in mitosis. KPNB1 SPICE1 AURKC TUBGCP5 MYBL2 KIFC1 XIAP CHEK2 BIRC5 KIF4A KIF2A ARHGEF10 WRAP73 KIF11 NLRC4 AURKB RACGAP1 BIRC7 KIF4B CDC14A TUBGCP4 KIF3B BIRC2 ZNF207 TUBGCP6 TUBGCP3 TPX2 FAM175B KIF23 TUBGCP2 TACC3 CLASP1 BIRC3 MAP9 RHOA MZT1 KIAA1383 NEK2 FLNA RAB11A GOLGA2 PIBF1 OFD1 BIRC8

GO_POSITIVE_REGULATION_OF_COLLAGEN_METABOLIC_PROCESS Any process that increases the frequency, rate or extent of the chemical reactions and pathways resulting in the metabolism of collagen, any of a group of fibrous proteins of very high tensile strength that form the main component of connective tissue in animals. CCL2 BMP4 RETN ENG C13orf15 CTGF SCXB UTS2 TGFB3 UCN SERPINF2 ITGA2 SERPINB7 AMELX CBX8 PDGFRB C1orf9 TGFB1 HDAC2 F2R WNT4 F2

GO_REGULATION_OF_CATECHOLAMINE_METABOLIC_PROCESS Any process that modulates the frequency, rate or extent of the chemical reactions and pathways involving catecholamines. PARK2 PARK7 VHLL SNCA MAOB ABAT GPR37 TACR3 PDE1B COMT DRD4 NR4A2 PNKD SLC6A3 HTR1A DRD1 HPRT1 CHRNB2

GO_REGULATION_OF_GLIOGENESIS Any process that modulates the frequency, rate or extent of gliogenesis, the formation of mature glia. CERS2 MYCN PRPF19 PPARG DLX2 IFNG EZH2 KDM4A C16orf45 LYN CDK1 TERT BIN1 LTA NOG ETV5 HDAC1 CXCR4 SPINT1 ZNF488 ODZ4 TNFRSF21 ID2 MBD1 ASCL2 RHEB SKI PTPRZ1 RNF10 SOX10 PLAG1 MTOR P2RY12 CNTN2 WDR1 DUSP10 SOX11 GPR37L1 SLC45A3 NKX6-1 PRMT5 CTNNB1 GPR183 NF2 STAP1 RNF112 RELA TP73 OLIG2 PRKCI NR2E1 TLR2 ADCYAP1 SOX8 EPHA4 CREB1 NTRK3 ID4 CDH2 TIAM1 PTN UFL1 PRKCH DAB1 EMX1 NKX2-2 BMP2 F2 CLCF1 NOTCH1 LIF HDAC2 CCR2 DLX1 HES5 NKX6-2 NF1 SIRT2 GFAP SHH MAG IL6ST LIN28A HES1 TSPO CSF1 DICER1 DRD3 GSX2 SERPINE2

GO_CATION_TRANSPORT The directed movement of cations, atoms or small molecules with a net positive charge, into, out of or within a cell, or between cells, by means of some agent such as a transporter or pore. ITPR3 CHRNG SELK ATOX1 KCNQ4 GRIN2C ACCN1 SLC23A2 NDUFA4L2 PRKCE SLC5A2 CHRNA10 ATP2B4 TCN1 KCNG3 SLC36A2 CHRNB1 JPH4 SLC44A1 CNGA3 SLC4A5 PTPRC KIAA1919 CSN2 P2RX2 KCNK17 SLC4A7 ATP6V0E1 SLC12A5 CAMK2G HCN3 TMEM38A FGF2 SLC41A2 SLC8A3 SLC9A6 SLC25A37 TSC1 ATP6V0B ATP6V0A4 SLC13A2 SCN1B HTR3B CACNA1S COX4I2 WNK4 RYR3 KCND3 GCK SLC47A1 COMMD9 SLC13A1 SLC12A4 TRDN CASK ATP6V0E2 STEAP1 SLC9A9 SLC35G1 GRM7 CDK2 PLCG2 SLC25A23 RAMP2 UQCRHL COX6A2 KCNC4 CCL19 ATP6V0D2 CACNA1E SLC30A5 NPY GAS6 ATP6AP1 MMGT1 KCNK12 CCR1 CHP HTR1F SLC5A1 ABCC8 NIPA2 SLC39A9 RHCE SLC17A4 SLC22A16 CCT8L2 TTYH1 FXYD2 COX17 TRPM4 FTL ATP1A4 SLC22A1 SLC22A3 ATP5J KCNA2 STIM1 SLC10A6 ATP6V1G3 TF SLC22A14 CDH23 NOX5 C15orf48 NNT MAGED2 PKD1L3 KCNAB1 DDIT3 SGK1 RHD HTR3C ATP5O CCDC90A PSEN2 SLC2A10 ATP2A2 HTR3D SLC46A1 VDAC1 CHRNA6 SLC38A3 CAMK2D KCNE1 CCS KCNH1 SLC25A27 FTH1 KCNH7 TRPC4AP SLC31A1 NIPAL2 SLC4A8 SLC35A4 SCN11A GRIN3A COMMD3 ANO6 COX6A1 CDKN1B GJA5 KCNK10 TRPV4 FAM26D HTR1E FAM26F TMC1 CACNA1F XCL1 KCNA10 EFCAB4A KCNH5 UQCRFS1 PDE2A RHCG CALHM2 SLC38A11 SLC9A10 ANK2 CLCA3P KCND1 KCNB1 COX7A2P2 SLC12A1 TRPV6 SLC24A5 NIPA1 HTR3E CACNG6 HCN4 SLC41A1 SLC5A11 EFHA1 CDK5 GJA4 PICALM ATP5H MRS2 TMEM66 ORAI1 SLC25A28 PKD1L1 ATP7A SRL NOX1 NEDD4L KCNJ12 SLC30A7 GRIN2A GPM6A KCNJ9 LMTK2 UCP2 TMCO1 ATP6V1D SLC40A1 STEAP1B CHRNA1 SFXN1 TRPC5 NMUR2 HTR2C ATP2A3 ATP2A1 SLC9A11 FTHL17 CHRNA2 SLC39A14 COX4I1 CHRNA3 PPP3CA KCNIP4 CACNB4 SLC39A7 ATP2B3 CHERP OPRM1 DENND5B CACNG1 SLC5A7 SLC9A7 SLC30A1 KCNJ11 ATP5G3 SLC30A9 CCL8 SLC17A2 MFI2 SLC44A3 COX5A SLC6A15 ATP6V1A SLC5A6 ATP1A1 SLC10A2 KCNJ10 TPCN1 TFR2 SLC30A10 ATP13A4 CUTC SLC34A2 KCNV2 HTR1D STEAP3 CHRNA5 SLC30A3 COX6B1 CALHM1 SLC39A12 SLC24A4 SRI KCNAB2 SLC5A12 KCNA4 SLC44A5 CUL5 CHRNB2 KCNK18 PKDREJ TFRC KCNV1 FTMT CACNB3 SLC25A25 TRAPPC10 CACNG7 KCNS1 UQCRFS1P1 KCNJ1 HEPH KCNJ8 SLC23A1 DRD2 SLC38A2 SLC8A2 P2RY12 PKD1L2 HEPHL1 SLC24A3 COX8C SLC22A5 SLC3A2 CACNA1H CNNM2 SLC24A2 ATP10D SLC13A4 TRPV1 SCNN1D KCNC3 CACNA1G CYP27B1 CNGB1 CACNG4 SLC35A5 SCO2 SLC25A14 ACCN2 REST CP SLC9A1 DENND5A SLC4A11 KCNK6 CNGA2 SURF1 KCNF1 SLC5A9 PKD2 SLC6A8 IBTK NOL3 TRPM7 ZP2 RAB11B EFCAB4B ADRA1A SLC2A12 ATP1A2 TUSC3 SLC39A1 RHAG KCNIP1 SLC5A3 TCIRG1 SLC7A8 CACNA2D2 ANXA6 ATP6AP1L JPH1 NIPAL3 CHRNB4 KCNJ16 P2RX5 KCNE2 GIF SLC13A5 SLC4A4 P2RX3 ITGAV CLCA1 KCNJ4 ITPR2 CYB5A PKD2L1 KCNE1L SLC2A6 COX6B2 KCNMB4 CHRNA4 HTR2A TMCO3 CACNA2D4 SCN10A SLC22A2 SCN9A MCOLN2 KCNB2 CLTC TRPM6 CATSPER2 CCL21 CAV1 CNNM4 SLC38A8 SLC15A1 CALHM3 GRIN3B SLC13A3 SLC34A1 KCNQ3 KCNJ13 SLC33A1 KCNK16 ACCN5 SLC39A4 SLC30A4 UCP1 SLC36A1 CACHD1 SLC5A10 FKBP4 KCNA3 SLC10A7 P2RX7 SLC17A8 CCDC109B PKD1 ATP6V1H ATP5L2 KCNG4 MAGT1 VDR SCNN1A PIEZO2 GAR1 KCNK9 HTR3A KCNJ3 SEC14L1 FXYD4 CHRNA7 SCNN1B STIM2 JPH3 ORAI2 ZP3 SLC18A3 TMEM165 KCNMB1 KLHL3 ATP5E HTR1B HCN2 SCN2B ATP6V1B1 ATP6V0D1 CCR7 MCOLN3 SLC38A6 KCNQ1 SLC39A5 CACNA1B SLC39A6 ATP4B TRPV2 ITPR1 TRPM3 MT3 RAMP1 HVCN1 AQP1 FTH1P19 ATP5S SLC10A5 ATP6V0C CACNA1A UCP3 PANX1 SCN4A SCN3B SLC30A6 SLC10A1 TMEM175 UQCR11 TMEM37 C22orf32 KCNAB3 SCO1 KCND2 MCOLN1 ATP6V1E1 TMEM38B SLC11A2 PRKCB SLC9B1 MFSD3 SLC10A4 SCN8A SCARA5 CHRNB3 KCNK15 UQCR10 SLC20A1 ATP5G2 CNGA1 ATP1B4 NDUFA4 SLC5A4 SLC9B1P1 CCL3 HFE ATP4A NCS1 MCU COX7B2 SLC15A2 FAM26E NALCN NDFIP1 UQCRH GRIN1 CACNA1D KCNT2 KCNH6 ATP5J2 C9orf7 KCNN4 SLC22A4 KCNE4 SCN4B SLC15A4 KCNJ6 ACCN4 ATP5C1 KCNK7 F2R SLC9A3 SLC44A2 ANO1 NSF ERO1L IL1RAPL1 CCL5 SCN1A SLC15A3 PLN ATP6V1C1 RYR1 COX7C ATP6V1G1 COX11 ATP5A1 COX15 KCNJ15 SCNN1G SLC9A8 STOML2 SLC41A3 KCNN1 TCN2 SLC35A1 CASR REP15 HPN ATP2B2 CACNG2 RAMP3 ATP2C1 SLC9A2 SLC20A2 SLC22A18 BHLHA15 SLC2A9 ATP6V0A1 KCNJ18 TRPV5 SLC39A10 SLC39A8 TPT1 CATSPER1 JPH2 CACNA1C KCNQ2 LCK PANX3 KCNJ2 TPCN2 ATP5B SLC5A5 KCNE3 CHRNE SLC30A2 CATSPER4 TRPM8 SLC38A4 SCN3A COX10 ANO10 SLC22A13 CACNG8 ATP5I SLC39A2 SLC2A8 SLC34A3 FKBP1A PLCZ1 KCNIP2 KCNH4 KCNK1 SLC39A11 TMC2 VPS4B ATP2B1 ARMC1 COX7B KCNQ5 KCNK5 SLC38A1 ATP2C2 SLC44A4 FYN CORO1A CNGB3 UQCRQ KCNG1 UQCRC1 KCNS3 SLC35A3 CACNA2D1 TRPV3 ABCB11 NIPAL4 BSPRY KCNN2 TRPC7 CACNB1 ATP1B3 IREB2 C1orf31 COX8A ATP13A5 CALCRL NIPAL1 KCNA7 RYR2 CACNG3 SLC17A7 LOXHD1 ATP6V1G2 SLC9A4 SLC2A13 SLC17A5 KCNA6 NMUR1 DNM2 CHRNA9 ATP12A RASA3 SLC39A3 KCNJ14 P2RX1 KCNMB3 STEAP2 UQCRB ATP6V1E2 KCNK2 ABCC9 KCNIP3 NDUFA9 SLC17A1 SLC5A8 KCNU1 HTR2B KCNA1 TRPM5 PIEZO1 PVRL1 ATP6V1B2 ZACN SLC8A1 ATP8A1 KCNG2 SCN5A CATSPER3 SLC24A6 COX5B FKBP1B CYSLTR1 SLC11A1 SLC24A1 KCNS2 KCNH8 RHBG SHROOM2 KCNT1 TRPC1 SLC35A2 SLC9B2 ATP5L SLC12A6 SLC12A8 SLC17A6 SLN ATP13A3 TRPC4 SCN7A KCNMA1 ATP5F1 MICU1 C16orf7 ATP5EP2 SLC30A8 KCNC2 ATP7B SLC38A7 KCNA5 CCR5 KCNH3 ATP1B2 TRPC6 ORAI3 KCNC1 KCNK13 HCN1 P2RX6 CACNA1I SLC38A10 COX7A2L SLC31A2 KCNN3 PKD2L2 CACNA2D3 ACCN3 SLC12A9 ATP5G1 TRPA1 ATP1B1 ATP6V1C2 SCN2A SLC39A13 KCNJ5 SLC12A2 KCNH2 SLC4A9 CACNG5 COX7A2 CTGF TMEM63C KCNK3 SLC12A3 ATP5D MTMR6 KCNMB2 STEAP4 ATP1A3 P2RX4 SLC36A3 CACNB2 COX6C CAMK2A CHRND ATP6V0A2 SLC9A5 TRPM2 SLC17A3 ATP13A1 PSEN1 SLC9A3R1 ATP13A2 COX7A1 KCNK4 CNGA4 SLC4A10 ATP6V1F SLC12A7 TRPM1 TRPC3

GO_ENERGY_HOMEOSTASIS Any process involved in the balance between food intake (energy input) and energy expenditure. ACACB SLC35D3 MRAP2 PIK3CA NR4A3 CRTC3 STAT3 OMA1 EDN2 MEX3C LEPR AMPD3 AMPD2 PRCP

GO_DORSAL_VENTRAL_NEURAL_TUBE_PATTERNING The process in which the neural tube is regionalized in the dorsoventral axis. FKBP8 GLI3 PAX7 GSC C6orf170 WNT3A PTCH1 GLI2 SMO TULP3 SHH FOXA1 WDR19 IFT122 PSEN1 TCTN1 BMP4

GO_RNA_SPLICING_VIA_TRANSESTERIFICATION_REACTIONS Splicing of RNA via a series of two transesterification reactions. SNRPD2 LUC7L3 TFIP11 PCF11 PSIP1 SMN1 CELF4 FRG1 SMNDC1 LUC7L2 PRMT7 ZMAT2 BCAS2 SCAF11 RAVER2 SF3A3 NUDT21 PLRG1 CWC27 DNAJC8 SNRPF SUGP1 SMN2 SRRT CDK13 CLNS1A HELB CDC40 RBM22 SF3B1 SRRM1 C19orf29 SRSF6 LSM3 SKIV2L2 WDR83 SF3B14 ZRSR2 NHP2L1 NCBP2 PHF5A PAPOLA PPIH HNRNPA3 DHX32 DHX35 HNRNPH1 HNRNPL RBPMS PTBP2 HNRNPA1 FIP1L1 CCAR1 NCBP2L DDX5 PNN METTL14 BUD31 PDCD7 PRPF4 SRSF10 SF3B5 THOC4 TRA2A DBR1 SRSF2 RNF113B DHX8 RNPS1 SF3B4 SNRNP35 HTATSF1 CSTF2 CPSF2 HNRNPA0 HNRNPM CPSF7 LSM7 TXNL4B DHX38 SART1 SLU7 RBMX BUD13 RBM41 DCPS USP49 CWC15 WDR77 SPEN ELAVL2 GEMIN2 CLP1 ZMAT5 MAGOH SNRNP27 GPATCH1 DDX20 HNRNPH3 U2AF1L4 LSM6 POLR2I CPSF3 SF1 PRPF40B SNRPE SF3A2 KIAA1875 HNRNPD SNRNP70 NONO PRPF18 SYNCRIP POLR2J XAB2 PRPF31 FUS POLR2F SETX METTL3 NOVA1 PSPC1 SNRNP40 PPIL1 HNRNPK DDX46 SNRPB SNRPD3 AQR POLR2A SRSF4 RBM17 DHX9 LUC7L SNRPB2 PCBP1 GEMIN7 RALY PPIE SNRPN SRSF12 HNRNPA2B1 SNUPN KDM1A POLR2C HNRNPU USP39 POLR2L DHX16 YTHDC1 SRSF9 LSM2 HNRNPF SNRPA1 HNRNPUL1 RNF113A CSTF1 EFTUD2 SFSWAP NAA38 HNRNPR RAVER1 SF3B2 SRSF1 SRPK2 POLR2B CELF1 NOVA2 DDX39A RBM5 GEMIN4 DDX41 SNRNP200 HSPA8 SF3A1 USP4 HNRNPC SRSF5 NCBP1 DDX1 GTF2F1 CTNNBL1 PTBP1 SF3B3 LSM5 RNPC3 DDX23 GPKOW POLR2E ELAVL1 PRPF39 SNRPD1 PPIL3 C20orf4 PRMT5 EIF4A3 ISY1 UPF3B PRPF40A PABPN1 GEMIN6 DDX39B SRRM2 ZRSR1 SYF2 TGS1 ZCCHC8 STRAP RSRC1 YBX1 MPHOSPH10 PRPF4B KHSRP GEMIN8 SART3 PCBP2 WBP4 PABPC1 ESRP2 RBMX2 SNRNP48 CELF3 PRPF3 DQX1 CASC3 C2orf3 SFPQ SNRPA RBM15 PPWD1 SNRNP25 SNRPG LOC100130932 GEMIN5 LSM4 POLR2D ZCRB1 DHX15 NOL3 POLR2K UBL5 SNW1 GTF2F2 CDC5L HNRNPH2 POLR2G PRPF19 CPSF1 POLR2H PRPF6 CSTF3 DGCR14 LSM1 SRSF11 PRPF8 TXNL4A SNRPC CWC22 U2AF2 SRSF7 WBP11 RBM8A PQBP1 TRA2B SRSF3 CD2BP2 CRNKL1

GO_REGULATION_OF_STRIATED_MUSCLE_CELL_DIFFERENTIATION Any process that modulates the frequency, rate or extent of striated muscle cell differentiation. FLT3LG PROX1 RBPJ MTOR FBXO22 TNFSF14 GREM1 XBP1 NKX2-5 AKAP6 CTDP1 G6PD HDAC3 HOPX BHLHA15 MYOD1 NRG1 DDX39B NLN EDN1 MORF4L2 CFLAR HDAC1 SHOX2 BMP4 HDAC9 HDAC4 SMYD1 MSX1 TGFB1 NOV MYF5 EHD2 CD53 MYLK3 SIK1 MYF6 EZH2 MMP14 MAML1 MAMSTR SHH TRIM72 MAPK14 BMP10 MYOCD IGF1 RBM24 NFATC2 CXCL10 SCGB3A1 NOTCH1 NEK5 ANKRD2 SOX6 BMP2 CEACAM5 GDF15 FLOT1 EHD1 TBX1 YBX1 CYP26B1 HAMP PIN1 PPAPDC3 CAV3 DKK1 SMAD4 IL4 MYOG THRA AKAP13 BCL2 EFNB2 MESP1 IL4R BHLHE41 GSK3A TRIM32 DMPK HDAC5 CXCL9 WNT3A RBM38 MEF2C

GO_ANTERIOR_POSTERIOR_AXIS_SPECIFICATION The establishment, maintenance and elaboration of the anterior/posterior axis. The anterior-posterior axis is defined by a line that runs from the head or mouth of an organism to the tail or opposite end of the organism. LDB1 FZD5 BASP1 LRP6 SKI WNT8A T WT1 RIPPLY1 HHEX CDX4 AURKA TMED2 FOXA2 TDGF1 HOXD8 SRF CTNNB1 OTX2 GPC3 FRS2 SHH GDF3 TBX6 PLD6 NRARP LHX1 PGAP1 PCSK6 CER1 CDX2 EPB41L5 RNF2 TDRD5 CDX1 WNT5A WNT3 HEY2 BMP4 SMAD4 WLS NODAL ETS2 KDM6A SIX2 DDIT3 TBX3 RIPPLY2

GO_CELLULAR_RESPONSE_TO_LIGHT_STIMULUS Any process that results in a change in state or activity of a cell (in terms of movement, secretion, enzyme production, gene expression, etc.) as a result of a light stimulus, electromagnetic radiation of wavelengths classified as infrared, visible or ultraviolet light. MFAP4 XPC OPN1SW RHO CASP9 TP73 ZBTB1 PAPD4 SIRT1 OPN1MW2 AURKB YY1 CRY2 NEDD4 PTN HIST3H2A PIK3R1 PDE6B CNGB1 PCNA OPN3 HYAL3 KDM1A MYC STK11 ACTR5 SAG CRIP1 CHEK1 INO80 TMEM161A GNGT1 IMPACT PTPRK HYAL1 CDC25A ERCC4 NFATC4 ATF4 ATR XPA MME RUVBL2 OPN5 RDH11 TRIAP1 N4BP1 CERS1 HYAL2 PDE6G RS1 PPID PBK TP53INP1 RHBDD1 GRK1 OPN1MW POLD1 CDKN1A AQP1 NOC2L DDB2 MDM2 RRH GNB1 TP53 BAK1 ERCC1 PDE6A CREBBP EP300 OPN4 POLH FMR1 USP28 CRY1 C12orf32 CUL4B GNAT1 USP47 DDB1 EIF2S1 EIF2AK4 MC1R CNGA1 OPN1LW TP63 TRPM1 PTGS2 BAX

GO_SEROTONIN_METABOLIC_PROCESS The chemical reactions and pathways involving serotonin (5-hydroxytryptamine), a monoamine neurotransmitter occurring in the peripheral and central nervous systems, also having hormonal properties. ATP2B2 GRIN2A SRD5A1 PDE1B BTBD9 DDC ATP7A RNF180 TPH2 TPH1 HTR1A

GO_NEGATIVE_REGULATION_OF_SUBSTRATE_ADHESION_DEPENDENT_CELL_SPREADING Any process that stops, prevents or reduces the frequency, rate or extent of substrate adhesion-dependent cell spreading. AP1AR ACTN4 POSTN MFI2 EFNA5 TACSTD2 RCC2 CORO1C KANK1 EPB49 FBLN1 GBP1 ITGB1BP1

GO_HEART_PROCESS A circulatory system process carried out by the heart. The heart is a hollow, muscular organ, which, by contracting rhythmically, keeps up the circulation of the blood. The heart is a hollow, muscular organ, which, by contracting rhythmically, keeps up the circulation of the blood. TNNC1 DMD GAA ADRBK1 SLC9A1 MYBPC3 GPX1 ACE SGCG CAV3 NEDD4L SGCD AKAP13 TNNI1 RPS6KA2 SCN5A TNNI3 SLC8A1 TPM1 ATP1B1 PLN GPD1L ATP1A1 CAMK2D KCNE1 MYL4 DNM1L KCNQ1 NUP155 PPP1R13L SCN2B SRSF1 MYLK2 MYL3 ATP1A2 SGCZ KCNA5 CACNA2D1 SCN4B FGF12 SRC MYL1 RAMP3 MYL2 KCNE1L GJC1 CHGA GJA5 CSRP3 TRDN PKP2 CASQ2 SCN3B TTN KCNE2 MYH7 TCAP SCN1B KCNH2 RYR2 MAP2K6 TNNI2 CDC42 MTOR VEGFB SNTA1 TAZ SOD1 CAV1 ACTC1 SMAD5 KCNJ2 ANK2 NKX2-5 TNNT2 PIK3CA CACNA1C MYH6 PDE4D CACNB2 ATP1A3 GJA1

GO_C21_STEROID_HORMONE_METABOLIC_PROCESS The chemical reactions and pathways involving C21-steroid hormones, steroid compounds containing 21 carbons which function as hormones. SRD5A1 CYP11B1 CACNA1H CYP11B2 AFP DHRS9 LHB CYP17A1 FDXR STARD5 STAR FDX1L FSHB CYP11A1 DHRS2 STAT5B AKR1B1 ADM AKR1C1 AKR1C2 AKR1C3 AKR1D1 STARD3 FDX1

GO_DORSAL_SPINAL_CORD_DEVELOPMENT The process whose specific outcome is the progression of the dorsal region of the spinal cord over time, from its formation to the mature structure. The dorsal region of the mature spinal cord contains neurons that process and relay sensory input. GSX2 UNCX WNT1 GDF7 C1orf187 HOXB8 DRGX PROX1 LBX1 RFX4 PBX3 MDGA1 TAL1 GSX1 WNT3A ASCL1 PAX7 LHX3 LMO4 LHX5 LHX1

GO_REGULATION_OF_INTERLEUKIN_8_SECRETION Any process that modulates the frequency, rate or extent of interleukin-8 secretion. NLRP10 PYCARD CD58 PTPN22 CD2 LGALS9 F2RL1 CD244 WNT5A ANXA4 GPR77 CRP HYAL2 F2R ANXA1 FFAR2 FCN1 SSC5D

GO_POSITIVE_REGULATION_OF_T_HELPER_1_TYPE_IMMUNE_RESPONSE Any process that activates or increases the frequency, rate, or extent of a T-helper 1 type immune response. ANXA1 SOCS5 CCL19 NLRP10 IL23R RIPK2 SLC11A1 IL27RA IL12RB1 IL12B IL23A HLX XCL1 CCR2 CD80

GO_KINETOCHORE_ASSEMBLY The aggregation, arrangement and bonding together of a set of components to form the kinetochore, a multisubunit complex that is located at the centromeric region of DNA and provides an attachment point for the spindle microtubules. APITD1 STRA13 CENPF CENPH POGZ CENPA CENPC1 CENPT MIS12 CENPE CENPW

GO_DNA_INTEGRATION The process in which a segment of DNA is incorporated into another, usually larger, DNA molecule such as a chromosome. GIN1 LOC100133495 ERVK-7 ERVK-10 RLF SCAND3 KRBA2 NYNRIN SETMAR BANF1 ERVK-6 SMARCB1 THAP9 ERVK-8

GO_REGULATION_OF_NEUROBLAST_PROLIFERATION Any process that modulates the frequency, rate or extent of neuroblast proliferation. NF1 PAX6 HIF1A CTNNA1 DISC1 NOTCH1 TGFB1 DCT VEGFC VEGFA LRRK2 VAX1 GLI3 DMRTA2 CTNNB1 OTP DRD2 SMARCD3 SIX3 ZNF335 ASPM FZD3 KCTD11 SHH CX3CR1 SMO SOX10

GO_REGULATION_OF_CELL_MIGRATION_INVOLVED_IN_SPROUTING_ANGIOGENESIS Any process that modulates the frequency, rate or extent of cell migration involved in sprouting angiogenesis. Cell migration involved in sprouting angiogenesis is the orderly movement of endothelial cells into the extracellular matrix in order to form new blood vessels contributing to the process of sprouting angiogenesis. FOXC2 HDAC9 DLL4 SRPX2 KLF4 PTGS2 NR2E1 MEOX2 HDAC7 ITGB1BP1 PDCD10 HDAC5 STARD13 MAP2K5 CIB1 MMRN2 NOTCH1 VEGFA RHOA

GO_ORGANELLE_FUSION The creation of a single organelle from two or more organelles. SNX14 GDAP1 VAMP3 SYTL1 VAMP4 STX4 C2CD4A EEA1 STX6 VAMP5 BAX SEC22B AFG3L2 SYT2 SYT14L VPS39 CDK1 VAMP2 UVRAG SYTL4 STOML2 WIPI2 USP30 DOC2B C2CD4C ST20 KIAA0226 SYTL5 ZFYVE20 NKD2 SNAP29 GOSR2 SYT10 FAM73A VTI1A BET1 VCP VAMP1 SYT14 SNAP23 VPS4A SYT15 MFN1 STX8 LIX1 SYT9 RPH3A DOC2A VPS11 SYT4 SYT12 SMCR7L TSG101 SYT5 VTI1B SNAP25 VPS16 SEC22C DYSF VAMP7 SYT3 UBQLN1 ANKFY1 EPG5 SNAPIN STX16 RAB7A STX1B C14orf133 STX7 TSNARE1 RAB39 KIAA0528 SYTL2 SYT8 STX17 STX11 VAV3 CHMP3 STX1A SAMD9L SYT13 STX5 SNAP47 OPA1 RAB20 SYTL3 VPS33A BAK1 CHMP2B TMEM175 MFF BNIP1 MFN2 YKT6 MCOLN1 LIX1L STX12 SYT6 SYT17 GNAI3 STX2 USO1 VAMP8 SEC22A SYT1 RAB34 TGFBRAP1 RAB8A STX19 STX10 GOSR1 CLN3 C2CD4D CYP26C1 CHCHD3 TC2N STX3 CAV2 VPS8 FAM73B BCL2A1 TECPR1 RPH3AL PLD6 SYT7 FIS1 SMCR7 VPS33B LRMP SYT16 VCPIP1 SYT11 VPS41 C2CD4B

GO_LIPID_LOCALIZATION Any process in which a lipid is transported to, or maintained in, a specific location. ABCG4 ABCG5 PLA2G4F PROCA1 ABCG8 APOA5 FABP1 ACSL4 CFTR NRIP1 TNF PNPLA2 ATP10A SLCO1A2 APOE SYT7 PRELID1 IL1B HDLBP APOD C11orf2 GOT2 CPT1B ATP8B1 ABCB1 SLMO1 NCOA2 ATP10B P2RX7 STARD4 CFHR4 SLCO1B1 RBP4 TMEM30A SLC10A4 LIPG COL4A3BP APOL6 ABCC3 HEXB SCARB1 PLIN2 ABCA9 PPARD GLTPD2 STARD6 ABCA6 SLC10A5 ATP11A SLC27A5 SLC25A17 ANO3 ANO7 SOAT1 APOL4 OSBPL6 C12orf39 SERPINA5 STX12 LIPC LDLRAP1 INHBA SLC10A1 PITPNM3 STAR CES1 SLCO1B3 ABCA10 ABCA13 SPNS1 CETP OSTBETA APOL2 SLCO2A1 CLN8 DRD3 CIDEA NPC1 DGAT2 NMB OSBP APOC2 APOF SLC10A6 LCN12 FABP6 STARD5 APOC4 SLC10A3 VAMP7 STARD3 KCNN4 LDLR PITPNA ABCA1 STAT5B ACE CLU PLA2G2C ATP8B4 SFTPA1 PLA2G10 PRKAB2 APOC1 CHKA SLC22A9 CPT1A ESYT3 ABHD4 ESYT1 LEP BSCL2 ATP8B3 ATP9B C2orf43 FZD4 SLC27A1 ABCA3 PPARG STOML2 FFAR2 GULP1 CPT2 PRKAA2 OSTalpha ESYT2 PLEKHA8 AKR1C4 B4GALNT1 OSBPL1A ABCC2 ABCC4 ACVR1C OSBPL2 PSAP OSBPL5 ANO6 APOA1 NOS2 ANXA1 NR1H4 PLTP ARV1 ABCB11 GLTPD1 RFT1 NMUR2 OSBPL3 ATP8B2 PITPNC1 APOL3 SLC10A2 ABCD1 SLCO1C1 ABCB4 STARD10 APOB SLC25A20 TRIAP1 TSPO ALB NCOA1 PRELID2 DGAT1 CEL PNPLA8 PLA2G1B ATP9A BDKRB2 APOL5 TNFAIP8L3 RXRA PLIN5 AGTR2 SPNS3 SCP2 ABCA12 APOO DRD4 FITM2 APOA4 ABCD3 ATP11B VPS4B SLMO2 CEACAM1 APOM SLC27A2 VLDLR PNLIP LCAT OSBPL9 PLA2G2F DRD2 PLA2G2E PLA2G12B APOL1 OSBPL7 NPC2 ANO4 PLEKHA8P1 SLCO2B1 MSR1 PLA2G12A PLA2G2A APOLD1 APOC3 NME4 ACSL3 SORL1 ABCA8 OSBPL11 OSBPL8 PLA2G2D LRP6 ACACA SLCO3A1 PCTP ABCG1 ENPP1 SLC27A4 FABP3 GPIHBP1 ACACB PRKAG2 PLA2G4A GLTP GM2A VPS4A ATP10D LBP CD36 ATP8A1 C20orf79 STARD7 MTTP PITPNM1 OSBP2 ABCA2 CRY1 GHRL ACSL1 ABCD2 ABCA7 APOBR APOA2 OSBPL10 ABCA5 OC90 AKR1C1 PLA2G3 SLC27A6 SLC9A3R1 CAV1 APOH LRP10 CROT ABCA4 PLA2G5 PITPNB SPNS2 MFSD2A ATP8A2 TMEM30B LPA PPARA SIGMAR1 NPC1L1 AQP9 SOAT2 ATP11C ANO9 LCN1 FITM1 ANGPTL3

GO_REGULATION_OF_DELAYED_RECTIFIER_POTASSIUM_CHANNEL_ACTIVITY Any process that modulates the frequency, rate or extent of delayed rectifier potassium channel activity. RNF207 VAMP2 NOS1AP KCNG1 KCNE3 KCNAB1 KCNS2 ANK3 AKAP6 KCNS1 KCNRG KCNE2 KCNE1 SUMO1 NPPA

GO_RESPONSE_TO_REACTIVE_OXYGEN_SPECIES Any process that results in a change in state or activity of a cell or an organism (in terms of movement, secretion, enzyme production, gene expression, etc.) as a result of a reactive oxygen species stimulus. Reactive oxygen species include singlet oxygen, superoxide, and oxygen free radicals. MDM2 UCP3 KLF2 BAK1 PXN AQP1 GPX8 LDHA SDC1 NUDT2 MT3 CCL19 DNM2 EDN1 NQO1 CDK2 PRDX2 IL18RAP BTK IL6 PRDX3 CBX8 MST4 HSPD1 PDCD10 SOD2 FOXO1 GPX5 DUSP1 HMOX1 AIFM1 APTX TXNRD2 ATP7A BCL2 TXN APOE PDGFRB GUCY1B3 AREG IL18BP SCGB1A1 TXNIP APOD PLK3 FXN MPV17 STAT6 PTK2B UCP2 NOS3 STK24 GLRX2 GPX3 APOA4 ETS1 EGLN1 ANXA1 PPARGC1B HDAC2 PPP5C ECT2 PRDX1 CYBB MB PPIF DPEP1 FABP1 PAX2 PRKAA1 RPS3 KDM6B TXN2 KPNA4 HYAL1 CAT CRYAB MTR TNFAIP3 AKR1C3 FER PDK2 CCR7 PPP1R15B HYAL2 ABL1 PRDX6 PTPRN JUN TRAF2 NET1 CDK1 NFE2L2 MMP3 PRDX5 ZNF580 EZH2 P4HB ZNF277 SOD3 NR4A3 STK25 APEX1 CYBA HNRNPD ARG1 HP HBA1 HBA2 CYCS COL1A1 MAP3K5 GPX7 PRKCD PLEKHA1 NOX4 GPX2 CASP3 AKR1B1 FOSL1 SETX FOS CRYGD TXNDC3 SOD1 RHOB ERCC6 GSTP1 PPP2CB STAT1 BNIP3 RELA SIRT1 PPARGC1A MAPK7 FKBP1B PARK7 ADA SLC8A1 TPM1 EEF2 PCNA BAD KLF4 AXL TXNRD1 PCGF2 NOX5 GPX1 HSF1 CST3 C20orf111 GSR HBB HDAC6 CCNA2 TRPC6 STAR KCNA5 MPO PTPRK KCNC2 IMPACT PKD2 PDGFRA SRC S100A7 TRPA1 GNAO1 CCS CYP1B1 ADPRHL2 ROMO1 CYP2E1 ERO1L PDGFD ADAM9

GO_CELLULAR_RESPONSE_TO_NITROGEN_LEVELS Any process that results in a change in state or activity of a cell (in terms of movement, secretion, enzyme production, gene expression, etc.) as a result of a stimulus reflecting the presence, absence, or concentration of inorganic nitrogen. GABARAPL3 MAP1LC3B MAP1LC3A MAP1LC3C MAP1LC3B2 BECN1 BECN1P1 NPRL2 ATG5 RB1CC1 GABARAPL2 GABARAPL1 ATG7

GO_REGULATION_OF_HETEROTYPIC_CELL_CELL_ADHESION Any process that modulates the frequency, rate, or extent of heterotypic cell-cell adhesion. CD44 TNF KLF4 FGG MYADM IL10 FGB FGA APOA1 IL1RN ADIPOQ BMP7 FLOT2 FLOT1 MAPK7 GCNT2 MAP2K5 IL1B ALOX15

GO_RESPONSE_TO_MECHANICAL_STIMULUS Any process that results in a change in state or activity of a cell or an organism (in terms of movement, secretion, enzyme production, gene expression, etc.) as a result of a mechanical stimulus. COL3A1 PKD2L2 JUNB EGFR CD40 ETV1 MAPK14 PDZD7 ACCN3 FADD TRPA1 MKKS SCN1A FAS PKD2 MAP3K2 SRC ITGA2 KCNC1 SOST BTG2 MPO KCNA5 BCL10 ATP1A2 ACTA1 MMP7 LRP11 TNC DNAH1 MAP3K1 CAV3 BAG3 MTPN SLC9A1 DENND5A BAD PKD1L3 TLR8 ANKRD23 SLC8A1 IRF1 RELA AKT1 MBD2 CXCL12 BNIP3 STAT1 BDKRB1 RAF1 CASP5 NFKBIA KCNJ2 KIT MAPK8 TNFSF14 RPS6KB1 CASP8AP2 KCNK4 PTGER4 JUP FOS MEIS2 FOSL1 PTGS2 SLITRK6 COL11A1 ASNS GATA4 MAPK3 TMC1 TNFRSF8 NRXN1 STRC PDE2A DCN TNFRSF10A COL1A1 HPN P2RX3 HTT TNFRSF1A ATP2B2 FOSB PKD2L1 BGLAP GADD45A CYBA TRPV4 HTR2A IGFBP2 ANGPT2 ATP8A2 INHBB HABP4 CCNB1 PPARG TGFB1 JUN CSRP3 LHFPL5 HDAC4 CRADD DENND5B ENDOG NTRK1 SERPINE2 SUN1 AGT CNTNAP2 CLCN6 NPPA SHANK3 SLC1A3 ATP1A1 MAG FYN PIEZO2 IL13 GLI2 CASP1 P2RY1 XPA CXCL10 ETS1 PKD1 P2RX7 CHRNA10 SOX9 CHI3L1 GCLC PTK2B LTBR CHEK1 DMD TLR4 SCXB POSTN IL1B SMPD2 TLR7 MAP3K14 XPC TXNIP MAP2K4 TMC2 PKD1L1 ACCN1 GPI DRD2 RETN KCNA1 TLR3 THBS1 CNN2 NFKB1 SLC38A2 STRA6 UCN PKD1L2 TLR5 MYD88 ABHD12 GJA1 PTCH1 KCNK2 CASP2 TGFBR2 FOXP2 IL6 ENG NRXN2 ANKRD1 SLC26A5 BMP6 CCL2 STRBP EDN1 PSPH PKDREJ RAC1 CHRNA9 TNFRSF10B RYR2 MMP14 ANO3 LOXHD1 JUND AQP1 TCAP BAK1 CASP8 TTN

GO_REGULATION_OF_ACUTE_INFLAMMATORY_RESPONSE_TO_ANTIGENIC_STIMULUS Any process that modulates the frequency, rate, or extent of an acute inflammatory response to an antigenic stimulus. C3 ADCYAP1 BTK CNR1 CCR7 FCER1G NPY5R ZP3 IL20RB SPN FCER1A SELS

GO_PYRIMIDINE_RIBONUCLEOTIDE_METABOLIC_PROCESS The chemical reactions and pathways involving a pyrimidine ribonucleotide, a compound consisting of nucleoside (a pyrimidine base linked to a ribose sugar) esterified with a phosphate group at either the 3' or 5'-hydroxyl group of the sugar. NME9 CTPS NME6 NME5 ENTPD4 UCK1 NME3 CTPS2 UMPS AK3 NME2 UPP2 NME7 UPRT CMPK1 TXNDC3 NME2P1 UPP1 DHODH NME4 UCK2 UCKL1 AK5 NME1 CAD

GO_MITOCHONDRION_LOCALIZATION Any process in which a mitochondrion or mitochondria are transported to, and/or maintained in, a specific location within the cell. KIF1B MUL1 UBB PLIN5 OPA1 UCHL1 MAP1B SLC4A5 MTM1 SPAST LRPPRC PVRL2 MAP1S BRAT1 MFN2 MARK2 UXT RHOT2 HDAC6 KIAA0664 LRRK2 HIF1A BHLHA15 ATP2A1 SYNJ2BP RHOT1 FEZ1 MSTO1 MAPT ATCAY C4orf49 NEFL DNM1L KAT2A MEF2A ALB

GO_REGULATION_OF_ACROSOME_REACTION Any process that modulates the frequency, rate or extent of the acrosome reaction. ZP2 SPINK1 POMZP3 ZP4 ZP3 CRISP1 PLCB1 GLRA1 CACNA1H B4GALT1 PLB1 SERPINA10 SPINK2 SPINK13 FAM170B IQCF1 SPINK8

GO_OXALATE_TRANSPORT The directed movement of oxalate into, out of or within a cell, or between cells, by means of some agent such as a transporter or pore. Oxalate, or ethanedioic acid, occurs in many plants and is highly toxic to animals. SLC26A6 AGXT SLC26A8 SLC26A9 SLC26A11 SLC26A1 SLC26A5 SLC26A3 SLC26A4 SLC26A10 SLC26A2 SLC26A7

GO_REACTIVE_NITROGEN_SPECIES_METABOLIC_PROCESS The chemical reactions and pathways involving a reactive nitrogen species. NQO1 GCHFR CYP1B1 SUOX SLC7A2 NOS1 MOSC1 TLR6 NOS3 GCH1 ARG2 RORA AKT1 POR SPR CPS1 NOS2 TLR2 DDAH2 MOSC2

GO_POSITIVE_REGULATION_OF_OSSIFICATION Any process that activates or increases the frequency, rate or extent of bone formation. BMP7 CEBPB CALCA CEBPD GLI3 BMP2 FBN2 OXT TOB2 NIPBL GDPD2 IL6ST TGFB3 BMPR1A SFRP2 ACVR2B DDR2 MSX2 IL6R IGF1 GDF2 BMPR2 C1orf9 LTF NPNT IFITM1 SMAD1 SLC8A1 MEF2C JAG1 ZHX3 ACVR2A GNAS C2orf28 ID4 HGF GPM6B WNT5A P2RX7 CTNNBIP1 CTHRC1 CEBPA ACVR1 NELL1 ILK IL6 WNT4 PKDCC NPPC BMPR1B ATP6AP1 GJA1 OSR2 OSR1 CLIC1 TP63 TMEM119 KL SOX11 SMAD3 FAM20C PTH CTNNB1 SMAD5 WNT7B TFAP2A TGFB1 ADRB2 JUND WNT10B ZBTB16 TAC1 PDLIM7 BMP6 TGFB2 BMP4 CD276 RUNX2 CYR61 PRKD1 ANO6

GO_REGULATION_OF_EXOSOMAL_SECRETION Any process that modulates the frequency, rate or extent of exosomal secretion. CHMP6 VPS4B SMPD3 TSG101 ATP13A2 STAM HGS IFNG RAB7A CHMP2A CHMP3 SDC1 SDCBP VPS4A PDCD6IP SDC4 SNF8

GO_GLUTAMATE_RECEPTOR_SIGNALING_PATHWAY A series of molecular signals initiated by the binding of glutamate to a glutamate receptor on the surface of a target cell, and ending with regulation of a downstream cellular process, e.g. transcription. GRM4 TRPM1 GRIN3B CDK5R1 CLN3 KCNB1 GRM1 SSTR1 APP HOMER2 GRIK5 HOMER3 GRID2 FMR1 GRIK3 PLCB1 ATP1A3 GRM6 GRIA1 GRIA2 PTK2B GRM3 GRIN2A GNAQ GRIA4 GRM7 GRIN1 GRM2 GRIN2C GRM5 GRIN3A GRIN2B HOMER1 GRID1 GRIK2 GRM8 CPEB4 GRIK4 GRIK1 GRIA3 GRIN2D

GO_REGULATION_OF_RENAL_SYSTEM_PROCESS Any process that modulates the frequency, rate or extent of a system process, a multicellular organismal process carried out by the renal system. OXT ADORA1 AVP F2R PDGFB CORIN GAS6 GJA1 EDNRB INPP5K PTPRO AGT AVPR2 AGTR1 OR51E2 NPPB UTS2R AVPR1A F2RL1 DRD2 DRD3 ADIPOQ TAC1 CYBA EDN1 C12orf39 AGTR2 UTS2 NPR1 EMP2 STC1 GJA5 COMT

GO_PHOSPHOLIPID_BIOSYNTHETIC_PROCESS The chemical reactions and pathways resulting in the formation of phospholipids, any lipid containing phosphoric acid as a mono- or diester. PTPMT1 CPNE1 PIK3R5 PIGK SLC27A1 CPNE3 PI4K2B PI4K2A INPP4A MTMR2 DPM2 LPIN3 EPT1 PIK3C2B PIGC MBOAT2 PIGQ INPP5J PIGV PIK3CA PIK3CB FGFR4 AGPAT1 CECR5 CHKA PI4KB INPP4B TAMM41 KLB GGPS1 FITM1 FGF3 PIK3R4 INPPL1 ABHD5 PIK3CD LPCAT3 MTMR6 LPCAT4 PTDSS2 SAMD8 PIGZ PIGT DHDDS FGF8 PIGF PIGB IDI1 CRLS1 PLA2G5 SGMS2 INPP5F GAB1 SACM1L PLA2G6 CHPT1 MTMR3 IRS2 PLA2G16 VAC14 DPM1 PMVK MTMR4 SERINC1 PIGX FGF4 OCRL LPIN1 ARF1 INPP5E ETNK1 FGF20 AGPAT5 PIGY PIP4K2B FGFR2 FAR1 DGKE SGMS1 FDPS PI4KA PGAP2 IMPA1 MTM1 ARF3 AGPAT4 LPGAT1 GPAA1 AGPAT6 PIGW AGPAT9 MVK PIGS ALOX15 PIP4K2A PIGH MTMR14 FGFR3 PLA2G4A SRD5A3 IRS1 CDIPT PPARD TAZ PIGL CDS1 KL PIP4K2C PIGP PIGM CDS2 PTPN11 CEPT1 PIP5K1B HEXB SPTLC2 PLSCR1 PISD PLAUR SPHK2 PIK3R2 PIGG FADS1 PIGU PIK3C3 MBOAT1 MBOAT7 ATM DOLPP1 PIK3C2G FGF17 LPIN2 TLR9 FGF7 ALG5 SERINC4 PIP5K1C PLCG2 SPTLC1 FGF5 FGF6 SYNJ2 AGPAT2 FGF23 AGPAT3 MPPE1 ETNK2 INPP5D FGF18 TPTE2 PIKFYVE GPAM FGF10 LPCAT1 PHOSPHO1 CWH43 PIK3R3 PCYT1A PIP5K1A PIGN FGF2 FRS2 SH3YL1 PIK3R6 FIG4 PGAP3 PIK3C2A SERAC1 PIGO FGF19 INPP5K PCYT1B SERINC5 IDI2 CHKB FGF16 NUS1 JUB DOLK FGF1 PCYT2 MTMR1 FITM2 PIGA PIK3R1 PTEN CPNE6 CPNE7 AGXT2L1 GRB2 LPCAT2 LCLAT1 PLD6 DPM3 PIK3CG PGS1 FGFR1 FGF22 SYNJ1 MVD FGF9 ISYNA1 MTMR7 GPAT2 PTDSS1 PEMT PGAP1

GO_ANTEROGRADE_AXONAL_TRANSPORT The directed movement of organelles or molecules along microtubules from the cell body toward the cell periphery in nerve cell axons. BLOC1S1 PLDN SPG7 AP3B1 CNO SNAPIN AP3B2 HAP1 AP3D1 MUTED AP3S1 KIF1B AP3M2 DTNBP1 KIF4A NEFL C4orf49 SOD1 BLOC1S3 AP3S2 BLOC1S2 RAB21 AP3M1 KIF1A KIF3B SPAST

GO_RESPONSE_TO_NERVE_GROWTH_FACTOR A process that results in a change in state or activity of a cell or an organism (in terms of movement, secretion, enzyme production, gene expression, etc.) as a result of a nerve growth factor stimulus. PDPK1 CALCA MICALL1 ID1 APP KCNC1 CIB1 RAB35 EEF2K KCNC2 CSNK1E KIDINS220 EHD1 RAPGEF1 CORO1A STMN2 SORT1 E2F1 KAT2A BPTF EIF4A3 NTRK1 ACAP2 AKT1 TRPV1 CBL CSNK1D ARPC3 FOXO3 CREB1 EEF2 TAC1 CDC5L MAGI2 RAP1A RAPGEF2 PTN

GO_KIDNEY_MORPHOGENESIS Morphogenesis of a kidney. A kidney is an organ that filters the blood and excretes the end products of body metabolism in the form of urine. CTNNBIP1 EYA1 CALB1 PKD1 FOXJ1 FGF1 GATA3 SOX9 SMAD4 VANGL2 MYC AGTR2 PAX8 AHI1 NPNT SOX8 FMN1 TACSTD2 PDGFRB FAT4 BCL2 CITED1 KLHL3 HOXA11 HES1 FGF10 WNT9B FGF2 GPC3 SOX4 GCNT3 ADAMTS16 SHH GCNT4 WNT6 PKD2 HES5 PAX2 WT1 BMP2 GLI3 KIF26B LAMA5 IRX3 NOG WNT1 HNF1A BMP4 DCHS1 SMO WWTR1 TCF21 SIX2 IRX1 SIX1 FGF8 LHX1 WNK4 PGF HNF1B WNT11 PBX1 DLG1 GDNF GZF1 PRKX WNT7B SALL1 GCNT1 CTNNB1 GREM1 NPHP3 PTCH1 OSR1 LGR4 IRX2 WNT4 FRAS1 LRRK2 FOXD1 HOXD11 ILK

GO_RETINA_VASCULATURE_DEVELOPMENT_IN_CAMERA_TYPE_EYE The process whose specific outcome is the progression of the vasculature of the retina over time, from its formation to the mature structure. PDGFRA ACVRL1 HIF1A NDP ARHGEF15 BMPR2 RHOJ PDGFRB LRP5 COL4A1 ROM1 CLIC4 FZD4 NRP1 ACVR2B CYP1B1

GO_ORGANIC_HYDROXY_COMPOUND_TRANSPORT The directed movement of an organic hydroxy compound (organic alcohol) into, out of or within a cell, or between cells, by means of some agent such as a transporter or pore. An organic hydroxy compound is an organic compound having at least one hydroxy group attached to a carbon atom. CADPS SLC2A13 SLC18A2 SLC10A5 AQP1 SLC27A5 AKR1C4 NPC2 SOAT1 OSTalpha AQP2 MSR1 SLCO2B1 AQP7P3 STX12 AQP9 SOAT2 NPC1L1 AQP10 SLC6A3 LIPC LDLRAP1 OSBPL5 SLC18A1 APOA1 MIP SLC10A1 APOC3 STARD4 GHRL RBP4 SLCO1B1 AQP6 CEACAM1 SLC22A2 APOC1 SNCA SLC6A4 SLC10A4 NISCH P2RX1 APOM AQP5 APOA2 PNLIP ABCA7 LIPG DRD1 ABCA5 ABCC3 SLC5A11 LCAT MECP2 AQP4 AKR1C1 SYK SCARB1 CAV1 FABP6 CEL APOE AQP3 SLC10A6 PGAP1 SYT7 SLC22A3 STARD5 AQP11 SLC10A3 MFSD10 SLC22A1 VPS4A RXRA PARK2 CD36 PARK7 AQP8 AGTR2 ATP8B1 SCP2 LDLR SLC26A6 C20orf79 NCOA2 ABCA1 ABCA2 VPS4B CLU APOA4 FCER1G ABCG4 ABCG5 NR1H4 ABCG8 SLCO1B3 ABCA13 CES1 ABCB11 ARV1 STAR SLC6A2 AQP7 APOA5 CFTR CETP LRP6 SLCO1C1 OSTBETA SLC10A2 APOB ACCN3 SLC5A3 ABCG1 FCER1A CHRM5 SLCO1A2 NCOA1 APOC2 ALB NPC1 OSBP

GO_NEGATIVE_REGULATION_OF_CELL_SUBSTRATE_ADHESION Any process that decreases the frequency, rate or extent of cell-substrate adhesion. Cell-substrate adhesion is the attachment of a cell to the underlying substrate via adhesion molecules. FZD7 CASK ARHGAP6 MEN1 TACSTD2 C11orf34 NF2 RCC2 CLASP2 MMP14 ITGB1BP1 APOD EPB49 ANGPT2 POSTN PLG RASA1 KANK1 CORO1C ADAM15 BCL6 WNT1 PTEN LGALS1 PIK3R1 ACTN4 AP1AR NOTCH1 MYOC COL1A1 EFNA5 ACER2 HOXA7 NF1 PTPRO DLC1 GCNT2 ACVRL1 BCAS3 SEMA3E FBLN1 GBP1 SRC SERPINE1 MFI2 SPOCK1 TBCD THBS1 FZD4 PHLDB2 CDKN2A

GO_CELLULAR_RESPONSE_TO_GAMMA_RADIATION Any process that results in a change in state or activity of a cell (in terms of movement, secretion, enzyme production, gene expression, etc.) as a result of a gamma radiation stimulus. Gamma radiation is a form of electromagnetic radiation (EMR) or light emission of a specific frequency produced from sub-atomic particle interaction, such as electron-positron annihilation and radioactive decay. Gamma rays are generally characterized as EMR having the highest frequency and energy, and also the shortest wavelength, within the electromagnetic radiation spectrum. WRN ELK1 NOX4 KDM1A BCL2L1 CRYAB H2AFX CYBA TSPYL5 XRCC5 TLK2 RAD51 ATM XRCC6 ATR TMEM109 YAP1 GTF2H5 EGR1

GO_CELL_ADHESION_MEDIATED_BY_INTEGRIN The attachment of a cell, either to another cell or to an underlying substrate such as the extracellular matrix, via an integrin, a heterodimeric adhesion receptor formed by the non-covalent association of particular alpha and beta subunits. ITGAV ITGA5 VTN ADAM9 COL16A1 FBN1 ADAM17 ITGB6 ITGA2 ICAM1 ITGB1 NPNT ITGA11 ITGA6 ITGB3 NOV

GO_RESPONSE_TO_FIBROBLAST_GROWTH_FACTOR Any process that results in a change in state or activity of a cell or an organism (in terms of movement, secretion, enzyme production, gene expression, etc.) as a result of a fibroblast growth factor stimulus. POSTN POLR2C NDST1 GRB2 GCLC FGFRL1 FGF1 TRIM71 DLL4 TNC IQGAP1 DSTYK FGFR2 POLR2L HNRNPF FAT4 CPS1 HNRNPM FGFR3 UBC UBB SCGB1A1 FGFR1 FGF22 FGF9 CCL5 FGF2 FRS2 CEP57 NR4A1 ESRP2 IER2 SFRP1 TDGF1 RBFOX2 HYAL2 CXCL13 FGF10 ZFP36L1 FIBP POLR2D FLRT2 UBA52 POLR2B FGF16 FGF20 KCNC1 STAR FGF19 FGF12 POLR2K HYAL1 FGF4 TBX1 FGF17 POLR2I ELK1 FRS3 NCBP1 FGF7 GTF2F2 FGF5 FGF6 TIAL1 CCL2 CDC5L KLB RPS27A KIF16B NOG CTGF NCBP2 FGF3 GTF2F1 FGF21 SHOC2 HNRNPH1 POLR2G FLRT1 TIA1 SNAI2 POLR2J LHX1 FLRT3 FGF18 PTBP1 POLR2F HNRNPA1 FGF23 POLR2E ITGB1BP1 IL8 FGF8 CBL CD44 KL EGR3 GALNT3 KAL1 RAB14 KDM5B PTPN11 SETX POLR2H SHCBP1 POLR2A PTH SPRED2 MAPK1 GCLM SPRED1 ZFP36 COL1A1 ESRP1 FGFR4 ZFP36L2 SNCA FGFBP1 MAPK3

GO_ACUTE_INFLAMMATORY_RESPONSE Inflammation which comprises a rapid, short-lived, relatively uniform response to acute injury or antigenic challenge and is characterized by accumulations of fluid, plasma proteins, and granulocytic leukocytes. An acute inflammatory response occurs within a matter of minutes or hours, and either resolves within a few days or becomes a chronic inflammatory response. ANO6 HP SERPINC1 FN1 NPFF CD163 KLKB1 TFR2 ORM1 AHSG PTGES ICAM1 MYLK3 IL1A ELANE VCAM1 REG3G IL31RA ITIH4 EIF2AK1 UGT1A1 HFE STAT3 OGG1 PLSCR1 APOA2 SAA2 NUPR1 PRCP SERPINF2 EPO ORM2 SERPINA1 MRGPRX1 ASS1 CD6 IL6 CTNNBIP1 ACVR1 IL22 GATA3 SERPINA3 HAMP S100A8 APCS DEFB1 REG3A F8 SAA1 IL1B VNN1 LBP TRPV1 MBL2 OPRM1 CRP INS IL6R F12 TNFSF4 CXCR2 APOL2 SAA4 B4GALT1 SIGIRR F3 F2 CEBPB

GO_POSITIVE_REGULATION_OF_MITOCHONDRION_ORGANIZATION Any process that increases the frequency, rate or extent of a process involved in the formation, arrangement of constituent parts, or disassembly of a mitochondrion. TDRD12 ATPIF1 PPP3R1 PPP1R13B UBL5 ITGAX MRGPRE MPV17L2 SAE1 KCNN3 YWHAE PLA2G6 E2F1 KAT2A GPR26 TSGA13 MIS18A VPS11 TP73 PMAIP1 PPARGC1A BNIP3 GSK3A GSK3B TP53BP2 ATG13 PARK7 PLAGL2 IL28B BAD STARD7 DDHD2 RPL28 WDR75 SMCR7L ACTL6A OR13C4 DYNLL1 YWHAZ DCN WBP11 EPT1 SFN ABLIM3 NBPF3 MMP9 USP36 MARCH5 TP63 BECN1 C10orf129 WDR46 ELMOD1 PINK1 PYCARD BAX SREBF2 C4orf49 UQCC U2AF2 MAPK8 MOAP1 ZBTB17 DYNLL2 STOML2 YWHAQ ARIH2 UBE2J2 PDCD5 FAM162A YWHAB GPHA2 HTT UGCG CTSK MICALL2 AMBRA1 SLC17A9 RMND1 HUWE1 C1QBP IL9R YWHAH FBXW7 PPIF CDKL2 TFDP1 HIF1A HSPA1L RAC2 C6orf125 GPER ZNF205 BCL2L11 CSNK2A2 KDR NPEPPS SNIP1 RNF31 ATPBD4 HK2 DPCR1 HNMT DNM1L ZDHHC8 TNFSF10 BID HES1 BAP1 RUVBL1 SMCR7 BCL2 BIK NKAP FIS1 PEMT NSUN4 YWHAG PLD6 PARK2 RHOU NMT1 GZMB CCDC56 UBE2D3 PPP3CC SLMO1 HPS4 ZBED3 CIDEB FAM20B FBXO7 CYB5R1 FZD5 PSMD8 LRRC46 BMF GLS2 HTRA2 PLAUR NRG1 TOMM7 PSMB7 LEPROT DDHD1 RNASE2 DTWD2 MLLT11 BBC3 UBL4B UBE2L3 CDK5RAP1 MUL1 TP53 CASP8 BAK1 HHAT MYBPC1 OPA1 OSCP1 APOPT1 SH3GLB1 NMUR1 POLR3A MFF HRK

GO_POSITIVE_REGULATION_OF_PROTEIN_SUMOYLATION Any process that activates or increases the frequency, rate or extent of the addition of SUMO groups to a protein. PIAS1 RWDD3 TOLLIP MUL1 PIAS4 GNL3 HDAC4 ARNT PIAS3 RASD2 UBE2I

GO_BRAIN_MORPHOGENESIS The process in which the anatomical structures of the brain are generated and organized. The brain is one of the two components of the central nervous system and is the center of thought and emotion. It is responsible for the coordination and control of bodily activities and the interpretation of information from the senses (sight, hearing, smell, etc.). SLIT1 FOXO3 PAFAH1B1 CTNNA2 SMO DUOX2 WNT5A EMX1 FANCD2 ZNF335 CDH2 PROP1 PTEN UCHL5 BBS4 FGF8 FZD6 MKKS SLC4A10 SHANK3 FZD3 BBS2 PSEN1 GSX2 GDF7 OTX1 SPEF2 HHEX SOS1 HESX1 SLC6A4 PAX2 NF1

GO_POSITIVE_REGULATION_OF_AMINE_TRANSPORT Any process that activates, maintains or increases the frequency, rate or extent of the directed movement of amines into, out of or within a cell, or between cells, by means of some agent such as a transporter or pore. KCNB1 RAB3B TACR2 SLC38A3 DRD2 DRD4 NPY2R OXTR PINK1 ADRBK1 OPRK1 AGT CHRNB2 TMEM27 STX1A CARTPT ARL6IP1 OXT CXCL12 ABAT GDNF

GO_NEGATIVE_REGULATION_OF_CYTOKINE_PRODUCTION Any process that stops, prevents, or reduces the rate of production of a cytokine. GATA3 HSF1 FOXJ1 NDFIP1 TRAF3IP1 GBA IL23R HGF AXL KLF4 LBP PPM1B BANK1 IFIH1 IKBKE AGER PRNP EPHA2 NLRP12 PCBP2 UBB LAG3 SLC11A1 ZC3H12A UBC IL12A CIDEA IRF3 FURIN RPS6KA4 TRIM27 JAK3 GPR18 CUEDC2 ITCH CRYBA1 PIBF1 HDAC7 ISG15 GBP1 C5orf30 PRG2 CD34 ANGPT1 TGFB2 C1QTNF3 ANXA4 OTUD7B APOA1 NUTF2 PYDC1 IRAK3 SFTPD PTPN22 BST2 ORM1 INHBB POMC KAT5 HERC5 TGFB1 ELANE NMI EZR TRAIP WNT11 PGLYRP2 TIA1 EPX LRRC32 IL12B GSTP1 TAX1BP1 IDO1 MEFV UBA7 RABGEF1 PDCD1LG2 IRG1 PTGER4 PYCARD NLRP3 TWIST1 HAVCR2 ZFPM1 LGR4 TUSC2 NLRX1 DDX58 XCL1 APOA2 GHRL O3FAR1 DLL1 SRGN GPNMB PGLYRP1 NCKAP1L BCL6 IL29 BCL3 REL TLR4 TRIB2 PIN1 SYT11 HMGB1 SCGB1A1 MAP2K5 LTF PML TBK1 CD83 OTUD5 CYLD FGFR1 APOD RNF135 ADIPOQ CDH3 TNFRSF9 BPI F2RL1 TNFSF4 ARRB1 IL1R2 CD84 TSPO GATA6 CD2AP RPS6KA5 TGFB3 TNF SOCS5 IL36RN TNFAIP3 SELS CMKLR1 SIGIRR IL23A SLAMF1 LEF1 ERBB2IP ERRFI1 IL13 PGLYRP3 NR1H4 ANXA1 C19orf29 CD24 LILRB1 CHRNA7 UBA52 TIGIT NDRG2 C1QBP CD276 ASB1 MAVS IL1RL1 ARRB2 DHX58 INHBA BTK RPS27A CD274 C13orf15 TLR9 RNF128 RAC1 FN1 IFNA2 TRAF3 RNF216 INHA AKAP8 CHID1 ACP5 IFNG MUL1 IL20RB FOXP3 NLRP2P NLRC3 TRIM25 KLF2 INPP5D LGALS9 HMOX1 RELB IL10 GPR77 GHSR ZFP36 MC1R CSK RARA UBE2L6 HFE IFNB1 THBS1 NFKB1 CD96 NLRC5 GAS6 IL33 SSC5D PYDC2 TNFRSF21 IL6 BTN2A2 NFKBIL1 VSIG4 CEACAM1

GO_NEUTRAL_LIPID_CATABOLIC_PROCESS The chemical reactions and pathways resulting in the breakdown of neutral lipids, lipids only soluble in solvents of very low polarity. APOC3 FABP4 FABP3 APOA1 FABP9 FABP7 ABHD6 PNPLA4 LIPC LPL FABP12 MGLL DDHD2 PNPLA2 APOB LIPE FABP5 ABHD2 ABHD16A APOA2 ABHD12 PNPLA1 APOA5 FABP2 FABP1 PNPLA5 DAGLA CPS1 PNPLA3 APOE FABP6

GO_REGULATION_OF_MITOTIC_CELL_CYCLE Any process that modulates the rate or extent of progress through the mitotic cell cycle. TMEM8B ANKRD17 TIMP2 DACT1 NLE1 CCND2 ATM RPS27A C13orf15 IGF2 EP300 OBFC2A GADD45GIP1 EME1 CDC23 CDK2 TP53 DYNC1LI1 CDKN2B NDC80 LRP5 DLG1 PSMB8 PSMD11 E2F7 CNOT2 SIK1 CENPE WNT10B LSM11 IDAS RPS6 SH2B1 WNT9A TERT PSMF1 CARM1 RCC2 TPR OBSL1 PSMB2 CSNK2A1 PSMC1 PSMC3 CUL3 PSMD5 DUSP1 PIM3 ERCC3 FZR1 PSMC2 PHOX2B FBXO7 MAP9 SMARCA4 APP UBE2E2 CUL4B BRD7 PIDD TPRA1 PTCH1 MKI67 PSMD8 BUB3 NME6 PAFAH1B1 FAP PTPN6 RFWD3 BLM CYP1A1 PKD1 PTEN RANBP1 DYNLT3 USP22 CDC73 PLK5 PIM2 ANAPC5 PSMA8 PSME2 PDGFRB TNKS1BP1 PLK3 IL1B PLRG1 SDCBP CHMP5 SETMAR PRCC DBC1 DBF4B PRKDC NEK11 FZD3 RNF20 CDC26 ABL1 BID TPX2 CDK13 FGF10 CRADD CDC25C ARID3A CDC16 CHMP1B ZW10 NPM2 PSMD12 PDGFB AURKA ANAPC7 APC PSMC6 BTG3 MIIP CSNK2A2 PSMA1 PSME1 SIRT2 ANKRD32 BTC PSMD10 NKX3-1 PSMB11 APEX1 CDC45 PSMB5 INSR PLAGL1 CDKN1B CDK5RAP3 INTS3 PSMD3 CDC6 PBX1 KANK2 FOXO4 CKS2 PSMA3 GPR132 BTRC ASCL1 LCMT1 H2AFY TTK PHIP ANKRD53 EZH2 FGF8 SENP2 CDK1 PSMA6 SMAD3 ASNS BECN1 ANAPC4 ANAPC10 DDB1 RPL24 BTG4 USP47 PSMD1 NEK2 XRCC3 MRE11A SPHK1 EIF4E PID1 LSM10 PDPN ID2 PHF17 KCNH5 TOM1L2 INO80 EDN3 PSMA4 HRAS CNOT4 CNOT6 PSMB1 PSMA7 PCNA UBE2E1 PRMT2 CTDSP2 PSMD6 SMARCD3 FBXO31 STAT5B RPA3 TNKS PRMT1 CENPJ UBC PSMD2 UBB ZNF830 L3MBTL1 RB1 SMC1A PSMD7 DDX3X CNOT10 RDX CHMP1A USP17L2 CAMK2D PCID2 RNF40 AATF ZNF268 FANCI KIF14 AIF1 BCL2L1 NSMCE2 CTC1 EME2 FAM178A PKN2 ECD DRD3 FAM5B E2F1 ZNF207 ATRX TOM1L1 CNOT3 RAB11A GTSE1 RCC1 EDN1 PTPN3 USP2 BUB1 SHB KLHL22 OBFC2B BMP4 USP44 CENPF CUL4A CHMP2B MAD1L1 ZWILCH MDM2 IL1A PPP1R1C PSMD13 TFDP2 OVOL1 C2orf29 CHMP3 CNOT7 ZNF655 CDKN1A CDC25B MAD2L1BP CEP97 PTPN11 CDCA5 PSMB4 PSMB7 PSMB9 NEUROG1 GIGYF2 FOXN3 IL10 TOP2B PKMYT1 NBN RPS27L EGF PRMT5 CHMP4C CTNNB1 CD28 C15orf42 SCRIB CDKN1C ZFP36L2 CASP2 RPA2 MAEA CCDC8 MDC1 PLK2 SYF2 HUS1B DUSP3 PSRC1 CHEK1 PSMB6 PIN1 FOXC1 FBXO5 POLDIP2 E2F4 HMGA2 VPS4B CUL9 ANLN RPRM PSME3 TEX14 CHEK2 MCPH1 HSPA2 MAGI2 KIF11 NPM1 MTBP THAP1 PSMA5 RAD51B CDK10 BCL2 PSMG2 CYLD CHMP2A TAL1 PHB2 EIF4EBP1 NACC2 PML XPC MEPCE HUS1 SOX4 ANAPC11 BRCA2 ESPL1 CAV2 PSMD14 NEK7 IGF1 NAE1 HES1 FOXA1 ZFP36L1 MUC1 TRIAP1 PSMA2 BRD4 UBA52 SKP1 TFDP1 TAOK3 TTC15 MSH2 ANXA1 TRIM35 SLFN11 EREG FAM5C EPGN E4F1 BUB1B PSMB10 GADD45A CDK9 PKIA FBXO43 CUL1 GAS1 CDC20 PARP3 ZWINT FHL1 PDXP CCNB1 ATF2 PNPT1 PSMD9 ANAPC2 TGFB1 NUSAP1 PCBP4 RAD51C CKS1B PDCD6IP CDC27 PSMC4 CDC14A BAX CDK4 HECA TP63 ANGEL2 MAD2L1 PSMB3 ANAPC16 CDC42 TACC3 RPS6KB1 CCNB3 SLC9A3R1 PSMC5 CHMP4B NEK6 CDC123 RAD17 SMC5 CLTC TOPBP1 PLCB1 GBF1 CTDSP1 RAD9A SFN PLK1 RNASEH2B PSME4 KIF20B GML RBL2 ANAPC1 MYO16 C1orf96 MAD2L2 DLGAP5 CNOT8 TIPIN CHFR DMRT1 UBE2C CUL7 BORA SIRT1 RAD9B CCND1 AKT1 AURKAIP1 ERCC2 TP73 UBE2D1 C11orf51 ZNF385A CTDSPL CEP85 SIN3A CDC7 MDM4 ADAM17 KNTC1 RINT1 DAPK3 TOP2A TTC28 EGFR INS CNOT1 SAE1 PRKCA TMOD3 GEN1 CLSPN STOX1 KCNA5 BTG2 PPP1R9B TGFA BMP7 CCNA2 PSMD4 AFAP1L2 CNOT6L C12orf11 PKD2 RQCD1

GO_VASCULAR_SMOOTH_MUSCLE_CELL_DIFFERENTIATION The process in which a relatively unspecialized cell acquires specialized features of a vascular smooth muscle cell. PITX2 ADM MESP1 RAMP2 VEGFA EPC1 NOTCH1 MYOCD ENG HES1 GATA6 SGCB SRF HEY2

GO_PHOSPHATE_ION_HOMEOSTASIS Any process involved in the maintenance of an internal steady state of phosphate ions within an organism or cell. FGFR4 GCM2 SLC34A1 XPR1 ENPP1 TFAP2B SFRP4 SLC34A2 GPCPD1 SLC9A3R1 SLC34A3 FGF23

GO_PROTEIN_LOCALIZATION_TO_CELL_SURFACE A process in which a protein is transported to, or maintained in, a location within the external part of the cell wall and/or plasma membrane. WNT11 RIC3 ANGPT1 PTPRU GPIHBP1 VCL FCN1 SMURF1 FLNA PTPRK USP4 FBLN5 MRAP MRAP2 FGF7 PIGK EMP2 ANK2 MESDC2 FGF10 ARF6 CTNNB1

GO_NON_CANONICAL_WNT_SIGNALING_PATHWAY The series of molecular signals initiated by binding of a Wnt protein to a frizzled family receptor on the surface of the target cell, followed by propagation of the signal via effectors other than beta-catenin. MYOC PSMC2 AP2S1 WNT4 AP2B1 PSMD8 FZD5 PSMB2 PSMB9 RYK PRICKLE2 PSMB7 PSMB4 PSMD5 CTNNB1 CALM3 ROR2 TNRC6A CELSR2 PSMC3 PSMC1 DVL2 PSMB8 PSMD13 FZD9 FZD1 SFRP4 MOV10 PSMF1 TNRC6C PSMD11 PRICKLE1 RAC1 FRZB PDE6A DAAM1 ARRB2 PLCB3 FZD10 RPS27A PSMC6 RHOA PSMD12 PARD6A PPP3CA UBA52 PSME1 PSMA1 LEF1 FZD8 AP2A1 NFATC1 FZD3 SFRP1 FZD2 SFRP2 WNT9B PTK7 PSMA2 ROR1 PSMD14 PSMA5 PSME2 PSMA8 PSMB6 GNAT2 VANGL1 MAGI2 WNT5A PDE6B GNG2 PSME3 SFRP5 CLTC EIF2C4 CAMK2A PSMD1 PLCB2 GPC4 PLCB1 CDC42 PSMB3 NLK FZD6 PSMA6 FZD4 PSMC5 PFN1 DVL1 GNB1 PSMA3 PSMD9 WNT11 PSMD3 EIF2C1 PSMC4 TNRC6B GRHL3 GPC6 DVL3 PSMB5 PSMB11 SMO PSMD10 PSMB10 WNT1 PSMD4 PPP3R1 ZNRF3 EIF2C3 CALM1 SMURF1 GNAO1 PDE6G CELSR1 FZD7 SMURF2 PPP3CB AP2M1 UBB CALM2 UBC PSMD2 TCF7L2 AP2A2 ARHGEF19 WNT7A PSMD7 MAP3K7 VANGL2 MED12 CELSR3 EIF2C2 PSMA7 PSMB1 PSME4 PSMA4 CTHRC1 TIAM1 PSMD6

GO_MODULATION_BY_VIRUS_OF_HOST_MORPHOLOGY_OR_PHYSIOLOGY The process in which a virus effects a change in the structure or processes of its host organism. HIPK2 CD4 HYAL2 EIF2AK4 KPNA1 BCL2L1 RRAGA SMAD3 ATG7 ZC3HAV1 BCL2L11 KPNA4 PABPN1 SLC25A6 KPNA3 INSR CPSF4 KPNA5 VAPA VAPB DAG1 KPNB1 NTRK3 BAD CD209 RXRA CCNK CLEC4M TYMS PARK2 TGFB1 KPNA7 KPNA2 ZC3H12A EIF2AK2 CASP8

GO_PROTEIN_LOCALIZATION_TO_KINETOCHORE Any process in which a protein is transported to, or maintained at, the kinetochore. BUB1B AURKB MTBP ZW10 CASC5 CHAMP1 GSG2 CCDC99 MIS12 CDK1 RCC2

GO_HEART_DEVELOPMENT The process whose specific outcome is the progression of the heart over time, from its formation to the mature structure. The heart is a hollow, muscular organ, which, by contracting rhythmically, keeps up the circulation of the blood. WNT8A TCF25 TWIST1 ID2 MAPK3 TNNT2 NKX2-5 SIRT6 ADIPOR2 CACNA1C CCDC103 SMAD2 ANK2 TMED2 FOXH1 IFT52 NOX4 NRP1 RBPJ SMAD3 CC2D2A SENP2 BBS5 CDK1 DVL3 SIX1 IFT74 FGF8 MBD3 PPARG JUN APLNR CSRP3 MED1 MOSPD3 GATA1 ZIC3 INSR GJA5 ZFPM2 ERBB2 MMP21 MTHFD1 NPY5R SORBS2 TBX3 SCUBE1 NPY2R TEAD2 NKX3-1 CALR SGCB PDGFRA HSPB11 ARL13B NFATC4 FLRT2 GLI3 HAND1 SOS1 NCOA6 TAB2 OXT GAB1 TDGF1 KAT2A LOX COL3A1 MKKS NIPBL FOLR1 RYR1 PLN CAMK2D TSC2 BBS4 GSK3A SPARC COX17 PROX2 SMAD1 TPM1 MEF2C GATA2 BMPR2 CRKL PPP3CB RAF1 MBD2 SGCG HEY2 CCDC164 BBS7 SMARCD3 PCNA MED12 VANGL2 TNNC1 KCNAB1 NTRK3 NOTO YAP1 BAZ1B OSR1 PTCH1 ADAMTS1 C6orf170 TAB1 ILK TGFBR2 RBP4 MEF2BNB ADAP2 MYH11 PPARD ECE2 TAZ GREM1 OXCT1 ACTC1 OVOL2 NPHP3 CACYBP OBSL1 COL4A3BP HEY1 SMAD7 PDLIM3 CPE PROX1 OXTR ID3 TCAP BICC1 PRICKLE1 SIK1 TSC1 LEMD2 TTN TRIP11 CER1 DVL2 PKP2 MYH7 FZD1 NPRL3 ASXL1 PLCE1 PRKAR1A SNAI1 FHOD3 ENG ATM CYR61 EP300 EPHB4 CLDN5 CDKL1 SOX17 MYL2 RAMP2 GLI2 DLC1 WT1 ALPK3 SEC24B KDM6B DNAAF1 PDGFB MB TGFBR3 C2CD3 PTK7 MSX2 GATA6 IFT140 PRKDC LMNA DHRS3 FHL2 SFRP2 ZFP161 BMPR1A FRS2 GPC3 AGT MAP2K5 RBM20 RPS6KA2 JAG1 SAV1 AKAP13 PDGFRB FAT4 DNAH5 MAP2K4 FOXN4 WNT5A LOC100506013 NOTCH2 STK3 SOX9 MIB1 PTN CCDC39 PSKH1 ACVR1 PKD1 PTEN SCXB GAA LRRC10 BMP5 KDM6A SNX17 CRIP1 DSCR6 TBX20 LBX1 MYH10 ACVRL1 ZFPM1 MEGF8 MYH6 MBD1 GATA4 CCM2 HHEX C5orf42 C3orf58 DLL1 MEF2A MAPK1 PSEN1 DVL1 FOXL1 CBY1 LEFTY1 COL11A1 DSP CDC42 DYNC2H1 DNAH11 MNAT1 AXIN2 STK4 PDLIM5 MAML1 EFNA1 RTN4 IRX4 POU4F1 TGFB1 MSX1 TGFBR1 COL2A1 CCNB1 SNAI2 FLRT3 AP1B1 WNT11 ATF2 TGFB2 GALNT11 MEF2D ZMIZ1 GATA5 CCDC40 NODAL DCHS1 GJC1 COL5A1 BCOR STIL PPARA SMO UBE4B FREM2 NF1 DCTN5 T XIRP2 LRP6 MTERFD2 PKD2 BMP7 ADRA1A MIXL1 XIRP1 PITX2 BMP2 SOX6 IFT20 LMO4 RBM15 BMP10 RARB RPGRIP1L PAM MYOCD BVES IFT122 SHH FOXF1 ACVR2B IFT172 SUFU SLC8A1 IFT57 EDNRA WNT3A ERBB3 SCN5A VEGFA LRP2 ITGA3 MESP1 DAND5 TNNI1 SOX18 CAV3 CRELD1 SETDB2 DLL4 SMAD4 GATA3 FGFR2 TRAF3IP1 ALDH1A2 SGCD EYA1 FOXJ1 ADAM15 IHH ADAM19 LTBP1 TRPS1 FBN1 REST NDST1 TMEM100 FGFRL1 SLC9A1 HOPX NEXN AP2B1 CAD NDUFV2 ARMC4 ITGB1 GJA1 NRG1 PLXND1 EPOR WNT16 MAP2K1 SH3PXD2B PTCD2 ID1 KCNK2 ODZ4 ERG ACTN2 ADAMTS6 SALL1 VEGFB CTNNB1 SRF KCNJ8 HTR2B GRHL2 WNT2 RARA PTK2 STRA6 NPY1R SOX11 PARVA PTPN11 RB1CC1 WDR69 NDRG4 MTOR SEMA3C SMAD6 SIN3B MYLK3 HAND2 TFDP2 MICAL2 RYR2 PRDM1 SMYD1 MDM2 ISL1 TEK NEBL HEXIM1 SHOX2 GNA11 ANKRD1 BMP4 SRI NOG HDAC9 CITED2 LUZP1 DNM2 DSG2 SALL4 ADM NKX2-6 EDN1 C20orf160 HIF1A FGF19 FGF12 HAS2 TBX5 GYS1 TBX1 EOMES SMYD2 SGCZ HEG1 S1PR1 NOTCH1 POFUT1 TH PPP1R13L TBX2 ECE1 MYL3 MYLK2 ZFP36L1 HES1 PCSK5 NPPA ARID1A CALCRL FZD2 SOX4 FKBP1A POU6F1 JMJD6 TMEM65 RXRA AHI1 TNNI3 ERBB4 OLFM1 WHSC1 NEDD4 CXADR GNAQ EGLN1 CHD7 FOXC2 HNRNPU SETD2 FOXC1 AGTR2 NEK8 SHC1 HEYL ROBO1 MYBPC3 ADRBK1

GO_REGULATION_OF_ENDOTHELIAL_CELL_MIGRATION Any process that modulates the rate, frequency, or extent of the orderly movement of an endothelial cell into the extracellular matrix to form an endothelium. AAMP PDPK1 BMPER PLCG1 CIB1 CEACAM1 NRP2 DCN MMRN2 STARD13 PDCD10 ALOX12 ATOH8 ACVRL1 BCAS3 PRCP SYNJ2BP ANGPT4 PTK2 PTGS2 DAB2IP PROX1 NRP1 THBS1 SCARB1 RHOB SASH1 APOH HSPB1 TGFB1 EFNA1 FGF18 TEK NFE2L2 ZNF580 ITGB1BP1 SERPINF1 ANGPT2 CCDC23 EDN1 PDCD6 STC1 BMP4 SP100 PTPRM GPR124 STAT5A SEMA4A HDAC9 PRKD1 PRKD2 C13orf15 PDGFB NOTCH1 RHOA VEGFC ANGPT1 FGF16 NF1 HRG MET FGF4 HDAC7 KDR SRPX2 MAPK14 AGT FLT4 FGF2 VASH1 PRKCA BMP10 AMOTL1 TDGF1 CSNK2B CXCL13 GDF2 ITGB3 EPHA2 APOE ROCK2 VEGFA BMPR2 GPLD1 AKT1 NR2F2 ZC3H12A SEMA5A HDAC5 BCAR1 MAP2K5 SPARC WNT7A NR2E1 FGFR1 CCBE1 KRIT1 MEOX2 AGTR2 FOXP1 ANXA3 SLIT2 PTK2B KLF4 HMGB1 SH3BGRL3 GATA3 DLL4 WNT5A FGF1 FOXC2 ETS1 PPAP2B EMP2

GO_PROTEIN_TARGETING The process of targeting specific proteins to particular regions of the cell, typically membrane-bounded subcellular organelles. Usually requires an organelle specific protein sequence motif. RPL38 ZFYVE9 POLA2 RPS26 PAN3 CHCHD4 TRAK2 RPL26 PEX3 RPS18 RPS14 RPS12 RPL35A MTX2 RPL17 RPL31 VTI1B HSPA4 OR1D2 TOMM20 RGPD1 RPS3A SRP9 F2R SRP14 IPO13 KPNA3 SEC61B ARL6 C2orf85 TIMM9 RPS17 WNK3 RPSA AKAP12 TIMM13 CSE1L NKD2 RPL21 DNAJC19 RPL35 HGS CHM ANK1 RPL37 RPL34 PEX16 GNPTAB RGPD2 CEP57 TSC2 SEC62 YWHAE SYNGR1 IPO7 RPLP2 JUN SSR3 YWHAQ RPL5 RGPD5 C15orf2 SIX2 PIK3R4 NCF1 NAGPA CBLB TAOK2 TIMM8B VPS25 TOMM20L RPL13A NUP62 PDE2A SRPRB TRAM1L1 GCKR VHLL RPS28 ARL6IP1 GDAP1 RANBP17 ATG4B MAPK3 RPL3 BECN1 BECN1P1 PTTG1IP RABGEF1 C18orf55 PRKG2 TMCO6 RPL15 KATNB1 C4orf49 RPL24 TOB1 PACS2 SYS1 KCNB1 ANK2 SAMM50 TMED2 RPL13 RERE RAB8B ATG3 KPNA2 PALM TOMM40 AKAP5 RASSF9 RPS15 SDCBP RHOD YWHAG IPO4 RPS13 VPS28 RGPD4 JAK2 NCKIPSD TIMM23 DNLZ RPL36A SRPR RANBP1 PAM16 HPS4 SPTBN1 TNPO2 SIX3 ANXA2 RPL32 MICALL1 NUP133 AIP RPS3 TIMM50 VPS13D RPL9 RPL10A RAB3IP GRPEL1 RPS11 TNF RANBP2 RPL11 PACS1 LMNA VPS36 PDIA3 PEX5L TOMM5 AGT AP1S3 POM121L2 RBM22 NUP155 BID RPL30 STXBP4 TRIP11 SEC61A1 TOMM40L RPS6 RPL19 PRICKLE1 KPNA6 IFNG RPL27 MT3 TIMM17B DAG1 GRPEL2 SSR2 TESC RPL23 RPS10 MFN2 KPNA5 RPS27A RPS19 GDF5 MYADM RAB33B OPTN MTX1 RPL27A NUP153 RPS7 FBXO7 AP3B1 RPL39 TOMM34 RAB7A OS9 PTCH1 ZFYVE16 GAS6 NDUFA13 NCOA4 RPAIN RGPD6 SYK TPR POM121C FXC1 NUP62CL BSG TRNT1 TOMM70A RGPD8 RPS25 C11orf73 SLC11A1 KPNA7 AKT1 RPL4 GCC1 GCC2 LOC100652748 PRKCI NUP98 TRPS1 CHMP4A TIMM44 RPL28 VPS13A POM121L12 NUP107 RPS27 TNPO1 SORL1 RPS5 RPS15A GOSR2 RPLP1 NUP188 SEC63 SRP19 PEX13 RPL7A RPL10 MTERFD2 SMURF1 NUP88 OPRD1 MCM3AP IPO8 LONP2 SEC61G NUP214 JAK3 PARD3 SSR1 RPS24 VTI1A MIPEP ABRA GOLGA1 PEX10 SPCS2 HSP90AA1 ICMT RPL8 NUP54 TGFB1 RPL23A LTBP2 TRAPPC8 SRP68 NUP50 RPLP0 IMMP2L TIMM10 TIMM17A RPL6 NFASC NUTF2 SRP72 YWHAB TGFB2 RPS9 RAB6A AKAP6 KCNB2 YWHAZ MTX3 RPL29 ANK3 NUPL1 RPL36 ADORA1 CDC37 RAE1 PEX5 RTP1 RTP2 RTP3 GOLGA7B PPP1R10 SPRN DRD1 AFG3L2 NOP58 STAT3 IPO5 IPO11 CHMP4B MAPK1 RPL18A ZFAND6 NFKBIA VPS13C RPL41 RGPD3 FIS1 RPS4X IMMP1L EHD3 PML TXNIP PEX12 NUP35 FGF9 PHB2 KPNB1 GIPC1 SNUPN VPS41 RPS8 RPS4Y1 NLGN1 POM121B FUT10 NEDD4 PIK3R1 MYO1C BCL3 TRAK1 BCL6 PEX26 NUP205 NUP85 UBA52 SNF8 RPS2 PPP3CA HSPA8 PMPCA TRAM1 HOMER3 SNX16 INPP5K IPO9 GOLGA7 PEX2 ERBB2IP KPNA4 RAN AKR1C3 KPNA1 TGFB3 ATG4C RPL14 PIP5K1A PEX7 TNPO3 AP3M1 TOMM22 SCARB2 RAB27A PEX6 RPS16 TSPO HES1 TRAM2 POM121 RPL7 RPL3L SPCS1 PEX14 PEX1 GNPTG ZFAND2B ATG4D NUP93 RPL26L1 GOLGA4 SRP54 MFF KIF13B RANBP6 RPL22 TIMM22 RPL37A AP1M2 RPS20 SYNJ2BP SLC25A6 RPL18 TOMM7 RTP4 RPS29 RPL12 PEX19 RPS23 ATG4A RPS21 WASH3P FYB EGF CTNNB1 ZDHHC3

GO_PRESYNAPSE_ASSEMBLY NA WNT3A DVL1 WNT7A PTEN WNT5A FZD1 PTPRD NLGN3 NLGN1 NLGN4X IL1RAPL1 NLGN2 LRP4

GO_NEUROTRANSMITTER_METABOLIC_PROCESS The chemical reactions and pathways involving neurotransmitters, any of a group of substances that are released on excitation from the axon terminal of a presynaptic neuron of the central or peripheral nervous system and travel across the synaptic cleft to either excite or inhibit the target cell. ALDH5A1 ABAT DAGLA CACNA1A MAOB TH DAGLB CHAT SLC22A2 LRTOMT NAALAD2 PAH ALDH9A1 DBH SLC44A4 NOS1 PRIMA1 GAD2 SLC6A3 GCHFR MAOA COMT SLC5A7 GAD1 COLQ HNMT ACHE CLN3

GO_PROSTATE_GLANDULAR_ACINUS_DEVELOPMENT The progression of a glandular acinus of the prostate gland over time, from its initial formation to the mature structure. The glandular acini are the saclike structures of the gland. FOXA1 FGFR2 RXRA HOXD13 FRS2 ESR1 HOXB13 SFRP1 WDR77 TP63 NOTCH1

GO_REGULATION_OF_NEURAL_PRECURSOR_CELL_PROLIFERATION Any process that modulates the frequency, rate or extent of neural precursor cell proliferation. SLC6A4 LIMS2 LRRK2 SPINT1 ILK RAPGEF1 NES ID2 SKOR2 GLI1 FOXO1 CTNNA1 PTPRZ1 SOX10 PROX1 GPR37L1 ASPM DRD2 EGF CTNNB1 SHCBP1 CEND1 LHX1 LHX5 TGFB1 INSM1 ASCL1 PTBP2 ELL3 LYN SMO KIAA1524 OTP GNG5 BTG2 GLI3 DCT NOTCH1 VEGFC SIRT2 NF1 GLI2 HIF1A GPR56 PTCHD2 FZD3 CX3CR1 SHH SPINT2 DMRTA2 SETD1A VAX1 NF2 CDON GATA2 VEGFA NR2E1 DISC1 WNT3A PAX6 ADCYAP1 KDM2B KCTD11 FOXO3 KDM1A SMARCD3 ZNF335 SIX3 CDH2 EMX1 WNT5A DLL4 TRIM71

GO_MRNA_3_END_PROCESSING Any process involved in forming the mature 3' end of an mRNA molecule. SRRM1 DDX39A POLR2D CSTF2T MAGOH SRSF6 CLP1 TUT1 THOC1 SLU7 PAPOLG THOC6 CASC3 SRSF1 CDC40 SRSF9 THOC2 PABPC1 CPSF6 CPSF7 CSTF1 CPSF4L SSU72 NUDT21 THOC3 DHX38 RNPS1 SARNP ZC3H3 SRSF2 AHCYL1 PCF11 THOC7 SLBP CPSF2 CSTF2 CDC73 MTPAP LEO1 PAPOLB RBM8A GRSF1 APP THOC4 UPF3B SRSF3 PABPN1 DDX39B ZNF473 CSTF3 FIP1L1 SRSF11 EIF4A3 SRSF4 SRSF7 U2AF2 SYMPK PNPT1 CPSF1 ZC3H11A LSM11 POLDIP3 PAF1 THOC5 PABPC1L CHTOP NCBP1 CPSF3 SRSF5 NCBP2 CPSF4 PAPOLA

GO_NEURON_DEATH The process of cell death in a neuron. POLB TP63 CDK5 BCL2L1 FADD SLC25A27 NLRP8 BAG5 BAX PSEN1 ENDOG BID NAE1 AIFM1 APP TNFRSF21 LRRK2 APAF1 NLRP1 BIRC8 FAS ATN1 SIAH1 SLC9A1 DIABLO FAM162A BOK POU4F3 MAGI1 ATM MAX LIG4 BCL2 RB1 GRIK2 KLK6 BNIP3 NLRP5 GAPDH MEOX2 PIGT ERBB3 DPYSL4 SDIM1 CBL

GO_CALCIUM_MEDIATED_SIGNALING_USING_INTRACELLULAR_CALCIUM_SOURCE A series of molecular signals in which a cell uses calcium ions released from an intracellular store to convert a signal into a response. CCL20 VCAM1 TMEM110 DEFB1 GPR143 SELP LPHN1 PRKACA KDR RYR2 EPB49 FKBP1B NTRK2 PTGFR AZU1 HOMER2 FIS1 BCAP31

GO_RETINAL_METABOLIC_PROCESS The chemical reactions and pathways involving retinal, a compound that plays an important role in the visual process in most vertebrates. In the retina, retinal combines with opsins to form visual pigments. Retinal is one of the forms of vitamin A. ALDH1A2 RPE65 BCMO1 RDH10 AKR1C1 RDH11 CYP1B1 SDR16C5 ALDH8A1 AKR1C3 ALDH1A3 BCO2

GO_CELL_FATE_COMMITMENT The commitment of cells to specific cell fates and their capacity to differentiate into particular kinds of cells. Positional information is established through protein signals that emanate from a localized source within a cell (the initial one-cell zygote) or within a developmental field. MYF6 ASCL1 SIX1 GSX1 FGF8 PAF1 NTF4 WNT11 HNF1B BATF GATA1 POU4F1 POU1F1 PAX7 TGFBR1 PPARG DLX2 GAS1 GATA5 NEUROD4 WNT1 SOX5 SLAMF6 POU6F2 TGFB2 NODAL SMO SOX12 SIX2 TBX3 ID2 GATA4 WNT8A MYOD1 ZFPM1 DLL1 AXIN1 FKBP8 LEO1 APC2 LATS1 RTF1 NKX2-5 NEUROD1 SMAD2 FEZF2 FOXA2 SOX1 PRDM14 WNT7B NKX6-1 TBR1 TCF3 PSEN1 MNX1 NRP1 IL7 DBX1 RBPJ FEV IFRD1 TBX19 FOXN1 DSCAML1 CDC42 WNT5B WNT3A SATB2 WNT7A NANOG MEF2C HOXA2 SUFU WNT2B TCF7L2 SMAD1 PAX6 MESP1 CDON SOX18 GATA2 MYOG CHD5 EYA1 IRF4 BARHL2 DLL4 WNT3 IL4 HEY2 GATA3 FGFR2 SMAD4 KLF4 CYP26B1 POU5F1 TRPS1 NTRK3 LATS2 WNT6 DLX1 ZNF521 NOTCH3 PITX1 BMP2 SOX6 GLI3 DOCK7 GSC NKX2-1 EVX1 HOXA11 SMARCC2 EBF2 GSX2 SHH WNT8B TBX6 PRDM1 WNT10B ISL2 WNT9A MYF5 PTCH2 LHX3 ISL1 FOXP3 FGF13 PTF1A JAG2 WNT10A RUNX2 GAP43 BMP4 SOX17 ETS2 GCM1 MYL2 NKX6-3 C17orf96 PTCH1 NRG1 ITGB1 WNT4 RAB10 RORA ODZ4 WNT16 IL6 DMRT3 ATOH1 NEUROG1 TRIM15 SMAD5 ROR2 EPAS1 SOX2 CTNNB1 PROX1 WNT2 RARA MCL1 OLIG2 ERBB4 SPRY2 TAL1 JAG1 NR2E1 PML SOX8 NR2F2 TEAD3 FOXN4 LY9 BCL2 C8orf22 ACVR1 FOXC2 NOTCH2 WNT5A SOX9 NKX2-2 CDC73 HOXD10 CTR9 LBX1 ARX STAT6 KDM6B EOMES TBX1 NOTCH4 EYA2 HES5 ELF5 NKX6-2 GLI2 PAX2 HOXC10 WT1 OLIG3 CEBPB NOTCH1 APC BCL11B ETV2 FOXA1 GATA6 GDF7 HES1 FGF10 RAG2 DMRTA2 WNT9B TBX2 ONECUT2 PPDPF GBX1 SFRP1 ONECUT1 RORC PRRX1 TLX3 PRKDC OLIG1

GO_PROTEIN_LOCALIZATION_TO_VACUOLE A process in which a protein is transported to, or maintained at, a location in a vacuole. SCARB2 WASH3P SH3BP4 VTI1A VPS13C TNFAIP3 BECN1P1 BECN1 NCOA4 VPS36 NRP1 GNPTAB PACSIN2 AP3M1 SNX16 AP3B1 HSPA8 VPS13D TMEM30A TOLLIP HGS RAB7A SMURF1 ZFYVE16 MICALL1 SNF8 SORL1 RAB35 GOSR2 ARF6 VPS13A VTI1B NEDD4 VPS25 CD81 RNF128 PIK3R4 VPS41 GNPTG SH3GLB1 NAGPA GCC2 VPS28 C7orf59 ACAP2 HBXIP TRAPPC8

GO_REGULATION_OF_B_CELL_MEDIATED_IMMUNITY Any process that modulates the frequency, rate, or extent of B cell mediated immunity. PVRL2 EXOSC3 C4BPB WHSC1 C3 IL4 FOXJ1 BTK FCER2 NDFIP1 BCL6 LTA HPX STAT6 UNG PTPN6 IL27RA IFNG TBX21 TGFB1 FOXP3 TNFSF13 MSH6 CD40 CD28 TNFSF4 C4BPA IL10 CD226 TNF EXOSC6 SUPT6H THOC1 FCER1A TFRC XCL1 IL2 FCER1G CLCF1 CR1 PAXIP1 APLF

GO_CELL_PROJECTION_ORGANIZATION A process that is carried out at the cellular level which results in the assembly, arrangement of constituent parts, or disassembly of a prolongation or process extending from a cell, e.g. a flagellum or axon. SRF CYFIP1 DRD2 DCDC2 ACTN2 C16orf80 FARP1 PTK2 UCN P2RY12 CCP110 ARMC4 ITGB1 VLDLR ITGA1 SLITRK4 TSPAN2 SDC2 EFNA3 TMEM141 UHMK1 PLXNB1 SCRIB ARF4 SCLT1 TEKT3 DBNL NEK1 EFNB3 NDEL1 MAP1S USP9X FOXB1 CHRNB2 FGD1 ADM MCF2 FGD5 LIMA1 TTC17 EXT1 IFT80 SEMA3A FBXO45 IQCB1 ISL1 ABLIM1 BBS1 SIAH2 LRFN5 GDF7 HES1 MATN2 NTRK1 NTNG1 MTR ULK2 FAM154A NLGN3 FLOT1 MTSS1L LRRC70 SYNGAP1 RAP2C NFIB PCDP1 HDAC2 CLIC5 UBA6 JUB S100B SEPT7 RAC3 TTLL1 RTN4RL1 MARK4 IQGAP1 TMEM106B CDH11 GPM6A E2F4 EMP2 ZNF335 DNAI1 NEFH C2orf71 ARX ATP6V1D POSTN SLIT2 MAP2 NLGN1 DNM3 CLUAP1 ROBO1 EHD3 ZMYND10 CROCC PICALM SCARF1 AHI1 ATP7A RSPH1 CNTN1 NEO1 SSNA1 LOC100653515 LAMB2 STRN DPYSL2 AREG DYNC2LI1 SLITRK2 KIT MEF2A BBS2 CCL21 HEATR2 SZT2 EPB41L3 CDC42 DYNC2H1 ATXN10 CCDC41 ARFGEF1 ARPC5 RFX4 MEGF8 NCAM1 PTPRZ1 UNC5D VCL ABI1 NES ANK3 WASF1 SPTBN2 LINGO4 GDPD5 RAP1A TGFB2 CCDC63 LST1 TTBK2 PVRL2 RAPGEF2 MICALL2 SEMA4A LRFN2 CCDC78 GALNT11 POC1A NPTX1 CHN1 DFNB31 NR4A3 INPPL1 WDR60 NME5 RNF165 SNX2 TRAPPC4 SEMA7A LYN SPTBN4 LRRC38 EFNA4 POU4F1 KLF7 EFNA1 C2orf62 FLRT3 DNAAF2 DICER1 RPGR IFT122 SRGAP2P1 TCHP SHH FAM179B TTLL8 ETV1 AKAP4 WASF3 EFHD1 NTF3 RP1 EPHB2 WDPCP GSN RANBP9 DNAH7 PDGFA IFT46 BTG2 ASAP1 TMEM231 PLXNB3 GATA3 FGFR2 WNT3 RAPGEF6 ZNF280D CNGB1 FOXJ1 ARHGEF7 ISPD TRAF3IP1 ALS2 TMEM17 RP1L1 FGD4 KIAA1598 EPHA4 CDH13 SEMA5A LRRN1 SDC4 LOC100507050 NEFM C14orf45 RTN4R CNO NKX2-8 WDR35 ITGA3 VEGFA S100A6 CCDC37 RAPH1 PAK1 TTC8 STMN4 ROBO2 E2F5 LRRC6 FOPNL DRGX KAL1 JAM3 LRFN1 TUBB3 ZNF280B CCDC67 RAB13 NPY HERC1 CUL4B ILK C6orf170 RAB10 PHOX2B SPTBN5 TSGA10 PRKCQ NTN3 DLG4 CCDC13 TTC26 PAK3 CDNF PARD6B SCN1B OR8A1 TSC1 ISL2 KIF24 SEMA6A LPAR3 SPAG1 MANF VAV2 TRIP11 INTU APBB1 CCK RSPH9 RFX3 C12orf55 CDK5R1 CCDC88A SLC12A5 TANC1 POC1B ABL1 CD2AP KIF5C SLC9A6 PLXNA2 SHANK3 KRAS IFT140 ONECUT1 GBX1 SEMA5B CEP41 NDN PTPDC1 MICALL1 GBX2 LAMA5 SHANK1 LPPR4 NUMB SPTB SPTBN1 ECM2 ARF6 SEMA3B RTTN CCDC39 SPTA1 LPAR1 KCTD17 PTK2B B3GNT1 HMGB1 PAFAH1B1 S1PR2 SDCBP TNN JAK2 APOD PRKCZ NEURL FAT4 ARTN VAX1 CPNE1 DCC SOD1 TULP1 WNT7B FEZF2 WDR19 RERE NPHP4 CDK5 SPG11 DSCAM ABLIM3 NRXN1 MAPK3 PRKG1 RSG1 PLEKHA1 TCTN2 NGF TMEM107 LRP4 CCDC103 LRRK2 FOXD1 EFNA5 RTN4RL2 SNX10 VAPA LRGUK PTPRM CDH4 ANO6 APOA1 FGD6 PTPRF C19orf20 SLIT3 BAG4 UNC5A ZNF280C CCDC64 NTF4 COBL CC2D2A MAP4 MAP1B CDK16 DPYSL4 OMD SPAG6 IFT74 GRIN3A JUN CTTN FBF1 NTN4 ADCY1 IFT81 COL25A1 EPHA5 RBFOX2 ACTR2 PLXNC1 EVL CNR1 RASGRF1 PCM1 C10orf92 ARHGEF4 UGT8 CCDC42B MYO7A GPRIN1 TSKU GFAP OTX2 PLEKHO1 IMPACT PIBF1 C7orf51 HSPB11 EHD1 OCRL MUTED DOCK7 C6orf165 PARVB NGFR SOS1 CEP152 GLI3 MYH9 BDNF POU4F2 CAMSAP2 CDH23 MINK1 CCDC164 BBS7 GRXCR1 EFNB1 UNC119B SGK1 CREB1 NCDN OMG WDR5 SIAH1 KIF27 CCDC113 EFNA2 TRIM32 GRIP1 TTYH1 RB1 BRSK2 WHAMM EFNB2 PHGDH PPP3CB LRFN4 UBB CXCL12 CENPJ MNS1 ATCAY BLOC1S3 TOP2B PACSIN1 ARHGAP35 CELSR2 ARL3 EIF2AK4 C1orf187 RYK CNTN2 MTOR PVRL1 PTPN11 LMX1A PARVA SPAG16 LAMB1 MKL1 GJA1 IL6 MYOC GFRA3 MST4 PLXND1 RAB35 HYDIN GALR2 GAP43 PTPN23 LINGO2 CTNND2 KIF3A NOG ZSWIM6 HPRT1 EMP1 RAC1 ACTR3 DNM2 TLR9 TCTN3 WWTR1 SRGAP2 CEP97 TTLL5 TROVE2 SEMA3C KLK8 BOC WEE1 FGD3 EPHB3 B3GNT2 KIF26A ITGA6 CCDC11 DTNBP1 CHL1 TSPO BTBD3 RFX2 SPATA6 DNAH9 STX3 EPHB1 USP33 ITPKA ONECUT2 PIP5K1A INPP5K MYO1A FRY HES5 FYN CORO1A NOTCH1 VASP S1PR1 RAB8A BCL11B NUP85 C10orf90 TBCE PLXNA3 TMEM138 RAB3A STMN1 NEDD4 PMP22 CORO1B APOA4 TEKT4 NRXN3 RAB1A PKHD1 NRN1L TMEM237 MAPK8IP2 GRB2 LRRC16A CRMP1 MKS1 ADCYAP1 FSCN1 NBL1 NRCAM LRRC55 STK36 BCL2 KIAA1377 ATMIN STMN3 ARFIP2 SLC9A3R1 MAPK1 MNX1 NPHP1 OGN MYLK EMP3 DVL1 NRAS TAPT1 TBC1D24 EPHA7 RAB17 IQUB DAB2IP AFG3L2 CDC14A PLA2G3 PLDN CEP250 BMPR1B KIDINS220 MAPK8IP3 NUBP1 PTPRO CTNNA1 PQBP1 CCDC28B EGR2 LOC100507003 FGD2 SEMA3F C5orf42 GHRL IFT43 STXBP1 STRC BBS10 CAMK2A EPHA3 AMIGO1 ICK CEP290 DPYSL5 CCDC40 BBS12 NFASC BLOC1S2 LRFN3 SLIT1 KIAA1009 LHX9 CCDC114 TTLL3 PREX2 UCHL1 FXYD5 ATP8A2 UBE2B GDNF LHFPL5 CAMSAP3 RTN4 LGI1 LAMA2 CNP NEFL NEK3 IFT27 RPGRIP1L EGFR PARD3 FNBP1L PRDM12 FUZ TWF2 LRTM2 BAI1 GNAO1 IFT172 TTC21A USP21 PDZD7 OFD1 PTPRK UBE4B PICK1 KIAA1731 SEMA6C C5orf30 TEKT2 RAP1B CCNO FMOD C1orf192 ARF1 BMP7 KIF19 PPP1R9B CAMSAP1 IFT20 SMAD4 VAX2 KIF5A CNTNAP1 CELSR3 KIF20B GLDN CCDC151 PAX6 LINGO1 IFT57 WNT3A SEPT2 DISC1 AGER TOPORS FEZ2 AKT1 FAM161A ATL1 KERA CUL3 KLF5 SSX2IP TRIM59 VAV3 SLITRK5 NPHP3 C19orf51 WASF2 CXorf41 IQCG AZI1 MECP2 BLOC1S1 FLNA SNAPIN NCAM2 ATOH1 C1orf88 APP ITGA8 TCTN1 POU4F3 LRRN2 DCLK1 DAG1 KIF5B SLC11A2 HDGFRP3 UNC5C CEP89 IDAS PLXNA4 ROCK1 SH2B1 KIAA1486 LLPH CSNK1D FLRT1 LHX3 B9D1 EXOC5 ALCAM SPTAN1 CACNA1A LHX1 CLMN C2CD3 KIRREL3 ENAH ZNF280A CCR7 ATP6V0D1 RPS6KA5 FZD3 CCKAR TNR NRTN SPAG17 CNTNAP2 PACSIN2 USH1G SEC24B RAC2 APOA5 PAX2 GLI2 ACSL4 RAB23 GPC1 DZIP1 AURKA PPP1R9A EPYC NTNG2 DNAAF1 KIF26B TMEM67 TOR1A NR4A2 AK7 WNT5A EMX1 DAB1 RELN PTEN GP5 IGSF9 P2RX7 DZIP1L DYNLT1 MYH10 MAP6 SPAST UNC5B PLP1 SNX1 PLXNA1 FMN1 SPATA13 GOLPH3 CEP164 RHOD KIAA0586 PLXNB2 EPHA8 NR2E1 ROBO3 APOE RAB8B NTN1 KIAA1984 DNAH5 DYX1C1 RPL24 TXNDC3 CSF1R NUMBL ALKBH1 DSCAML1 SLITRK6 IFT52 KLHL1 MTMR2 NRP1 ULK1 MTSS1 IFT88 SPON2 ANKRD27 FEZ1 FEZF1 KIF17 HAP1 PLA2G10 SPEF2 PTPRA KIAA1751 C5orf13 NRP2 SLITRK1 ARHGEF6 DHFR ATP2B2 ZFYVE27 ERBB2 DLX5 DHFRP1 RSPH4A RAP2B NUBP2 APBB2 NCK1 SS18L1 SEMA4F ETV4 ABCC4 BBS5 C21orf2 CNTN4 ZNF259 PCNT TMEM216 FGF8 ARHGEF26 BAIAP2 RAP2A DNAI2 OPHN1 EZR CRTAC1 LINGO3 TTC30A PAK4 SLITRK3 BBIP1 BBS9 PLEK FOLR1 AIF1 MKKS MAPT RAB11A SPRY3 SNAP29 TNIK FLRT2 PAK2 LLGL1 NKX2-1 RAB25 HDAC6 ARL13B ARL6 LHX4 C11orf63 DNAH1 PRMT1 TNC PRELP BRSK1 AVIL NCK2 CTHRC1 TIAM1 GBA STK11 VANGL2 B9D2 LUM HRAS LHX2 NOTO CTNNA2 CDH1 L1CAM GSK3B BBS4 WNT7A HOXA2 FRYL RDX TPM1 MAP4K4 TTC30B C21orf59 ALMS1 WRAP73 TTC21B

GO_NUCLEOTIDE_SUGAR_METABOLIC_PROCESS The cellular chemical reactions and pathways involving nucleotide-sugars, any nucleotide-carbohydrate in which the distal phosphoric residue of a nucleoside 5'-diphosphate is in glycosidic linkage with a monosaccharide or monosaccharide derivative. B4GALNT2 MGAT1 EXTL2 SLC35A3 MPI GNPDA1 CSGALNACT1 TSTA3 GALT PMM2 GNPNAT1 AMDHD2 DPM1 RENBP TGDS GFPT1 PMM1 UAP1L1 UGP2 UAP1 UGGT1 GNE GUK1 GFPT2 SLC35D1 UXS1 PGM3 UGGT2 NAGK FUT8 GNPDA2 UGDH DPAGT1 GMDS GMPPB

GO_TETRAPYRROLE_METABOLIC_PROCESS The chemical reactions and pathways involving tetrapyrroles, natural pigments containing four pyrrole rings joined by one-carbon units linking position 2 of one pyrrole ring to position 5 of the next. HMBS CTRC PRSS1 MMADHC SUCLA2 CTRB2 IBA57 CTRB1 BDH2 ATPIF1 UROD CUBN ALAS1 ABCD4 RSAD1 AMBP PRSS3 TSPO HMOX2 HMOX1 CPOX MMAB PPOX UGT1A1 EIF2AK1 SLC25A39 MTR MTRR COX10 BLVRB BLVRA COX15 CYP1A2 UROS ABCC1 GIF TCN2 ABCB6 NFE2L1 CD320 MMACHC TMEM14C ALAS2 MMAA MUT CYP1A1 SLC25A38 FECH UGT1A4 AMN TCN1 HPX ALAD FXN SPTA1 SLC11A2 LMBRD1

GO_REGULATION_OF_CELL_SUBSTRATE_ADHESION Any process that modulates the frequency, rate or extent of cell-substrate adhesion. Cell-substrate adhesion is the attachment of a cell to the underlying substrate via adhesion molecules. PTK2 DMP1 VIT THBS1 GREM1 ILK RSU1 DOCK1 EGFL6 MYOC HOXA7 WNT4 GCNT2 BCAS3 FLNA SERPINE1 FBLN1 DNM2 RAC1 FN1 SMOC2 VWC2 MYADM CYR61 FBLN2 CASK LIMS1 ITGA6 TEK MYF5 RCC2 CLASP2 ROCK1 TSC1 MMP14 KDR MFI2 SFRP1 ONECUT1 ONECUT2 CCR7 VTN FGB CSF1 CLASP1 CDKN2A DDR1 ACTN4 NOTCH1 AP1AR VEGFC RAC3 ACER2 C1QBP RAC2 HAS2 MACF1 DLC1 HRG PLEKHA2 RELL2 POSTN PTK2B GPM6B IQGAP1 ECM2 PRKCE ITGA5 PTEN BCL6 PIK3R1 PTN EMP2 BCL2 NID1 ROCK2 CCDC80 TRIOBP FMN1 LRRC16A RHOD CCL25 JAK2 PRKCZ ARL2 APOD CDC42 CDK6 EDIL3 FAM21C SMAD3 SPOCK1 OLFM4 FZD4 PHLDB2 CCL21 PLAU CRK COL1A1 PIK3CB EFNA5 HSD17B12 CIB1 ABI3BP EPHA3 EMID2 SPOCK2 ACVRL1 PTPRO EGFLAM SEMA3E AGR2 LDB1 ANGPT2 PREX1 DOCK5 PLG UTRN RASA1 NPY2R COL16A1 CORO1C PPM1F FGG WNT1 COL8A1 EPB41L5 NINJ1 APOA1 FGA S100A10 MEN1 NET1 C11orf34 ITGB1BP1 PTPRJ UNC13D TBCD FOXF1 RREB1 DAPK3 SLK WDPCP BRAF NF1 NDNF CALR GBP1 SRC SLC9A1 KANK1 EPHA1 ATXN3 LGALS1 ARHGEF7 MINK1 ADAM15 ITGA3 FZD7 CRKL VEGFA CCL28 ARPC2 ARHGAP6 TACSTD2 NF2 GSK3B SDC4 CDH13 NPNT CD36 ALOX15 EPB49 DISC1

GO_POSITIVE_REGULATION_OF_LIPID_STORAGE Any process that increases the rate, frequency or extent of lipid storage. Lipid storage is the accumulation and maintenance in cells or tissues of lipids, compounds soluble in organic solvents but insoluble or sparingly soluble in aqueous solvents. Lipid reserves can be accumulated during early developmental stages for mobilization and utilization at later stages of development. MSR1 NFKB1 APOB LPL FITM2 SCARB1 SREBF2 CIDEA C3 C7orf68 NR1H2 FITM1 PLA2G10 ACACB ZC3H12A APOC4 PLIN5 OSBPL11 IKBKE EHD1 CD36

GO_POSITIVE_REGULATION_OF_MRNA_METABOLIC_PROCESS Any process that activates or increases the frequency, rate or extent of mRNA metabolic process. HNRNPR PAF1 TNRC6C TNRC6B THRAP3 GTPBP1 CNOT7 APOBEC1 POLR2G PRPF19 NANOS3 PNPT1 PABPC1 UPF1 ZC3H12A CCNB1 CDC73 SNRNP70 RC3H1 KHSRP CELF4 CNOT8 NCBP2 SF3B4 NCBP1 PRR5L SNW1 EIF2C2 NANOS2 HSPA8 CPEB3 ZC3HAV1 TRA2B C11orf35 HSPA1A BTG2 LEO1 NANOS1 ZFP36L2 TOB1 DAZAP1 CNOT1 ZFP36 ZFP36L1 HMX2 CELF3 RBMX SLC39A5

GO_POSITIVE_REGULATION_OF_SECRETION Any process that activates or increases the frequency, rate or extent of the controlled release of a substance from a cell or a tissue. OXT CLEC6A F2R HGS TNFRSF4 SIRT3 CD2 CYP4F2 IL17RB GLUL TMEM27 NMB SYT10 ADAM9 CD14 ANO1 NR1H2 PFKFB2 S100A9 DRD3 CXCL12 NTSR1 APLN SYT9 NLRP12 PPP3CB CRH CASP5 SYT4 GATA2 BRP44 SNX4 CLEC4E MCU GLMN TCF7L2 PPIA NCS1 TRPM4 KCNN4 EDN3 CARD8 VAMP7 C2CD2L FFAR1 CREB1 TSG101 PHPT1 IL4 MYRIP CAPN10 PLA2G10 CLEC5A TWIST1 HAVCR2 LEP PINK1 GHRHR CDK5 NPPB RAB27B STX4 KCNB1 XBP1 CSF1R LAMP1 CD244 FFAR2 SYTL4 FGR IL17A EZR NR0B2 INHBB NKX3-1 PYDC1 PTPN22 CYBA TRPV4 CHIA NPY2R CDK5R2 GIPR VTCN1 DOC2B P2RY1 VAMP8 AVP PLA2R1 FCER1G VEGFC ZP3 IFNAR1 IL13 P2RY2 CFTR EXOC1 SEC24A AGT FCER1A RASL10B STXBP5L TNF CLASP1 PPID FGB HYAL2 ABL1 RAB15 DNM1L F2RL1 SYT7 RAB8B IL1B JAK2 PRKCZ RPH3AL GOLPH3 SDCBP PDX1 HMGB1 UCN3 PAEP CYP4A11 MAPK11 CLEC9A P2RX7 EXPH5 SPTBN1 MYO18A WNT5A PRKCE HSPD1 RBP4 STXBP5 TRIM6 GCG RAB7A NLRP1 IL33 ATP6AP1 STX1B CCL3 SYK HFE OXTR OR51E2 GOLPH3L PTAFR C1QTNF1 RETN OXCT1 LGALS9 LACRT PPARD GCK PANX1 APBB1 CCK UNC13B SCIN VSNL1 STX1A MAP2K6 IFNG FCN1 AQP1 TNFRSF11A FOXL2 KIF5B CCL19 SMPD3 INHBA CD274 IL5 C13orf15 CD276 CD38 BMP6 PFKM ACSL3 BLK TARDBP ARF1 ABAT CD34 SRC MIF GSDMD SLC30A8 LPL MAPK14 UNC13D ANG IRF3 IRS2 CACNA1I AVPR1A PLA2G6 EGFR ADORA2B INS RAB3D GPLD1 TRPV1 CACNA1G RASGRP1 VPS4A SDC4 IL4R BAD CRTAM NPR1 ARHGEF7 GATA3 SMAD4 GPR68 AIM2 ADORA1 SNCA VAMP3 TRPM2 GHRL STXBP1 P2RX4 CARTPT GALR1 PTGER4 CD58 PYCARD STAM NLRP3 ATP13A2 GAB2 ZAP70 NOD2 ACHE NKX6-1 SCT AACS TACR2 ALOX12B GDNF TGFB1 GAL TRIM16 GPR27 NLGN2 PSMD9 ALOX15B OPRL1 PDCD6IP RAB3GAP1 VPS35 TRH FGA IL1RAP C1QTNF3 FGG TGFB2 CASP1 PCK2 IL17F SNF8 RAB3A SYT1 NR1H4 IL2 HCAR2 SLAMF1 SERP1 GPER GHRH HIF1A IL17RC SOX4 NLRP10 CADM1 TGFB3 IGF1 CADPS2 RAB27A TNFSF4 ARRB1 PLA2G1B DPYSL2 TNFSF11 TNFRSF14 GLUD1 FGFR1 SAA1 CHMP2A RFX6 RAB9A ADCYAP1 NLGN1 OPRK1 ADRBK1 S100A8 NMU AGTR2 POSTN RAB2B HLA-E TAC4 VPS4B TMF1 C5 KISS1 IL6 SYTL2 GJA1 EDNRB PTPN11 SOX11 UCN IL26 IL10 HTR2B DRD2 SCAMP5 CPB2 GIP FAM132A DTNBP1 ISL1 IL1A TFR2 NLRP2 INHA CLASP2 AP1G1 SDC1 TAC1 EDN1 ILDR1 IL17RA WLS CHRNB2 ADAM8 SLC6A1 RAB5A SRI NNAT ANKRD1 IL1RL1 UNC13A

GO_CELLULAR_HORMONE_METABOLIC_PROCESS The chemical reactions and pathways involving any hormone, naturally occurring substances secreted by specialized cells that affects the metabolism or behavior of other cells possessing functional receptors for the hormone, as carried out by individual cells. AKR1B15 COMT SCPEP1 HSD17B8 HSD3B1 CRABP1 ADM UGT1A3 HSD17B6 SULT1A1 AFP RDH10 DHRS9 CYP26A1 RPE65 LRAT CRABP2 SRD5A1 AKR1C4 SULT1E1 UGT1A9 ALDH8A1 UGT1A8 UGT2B7 MED1 FDXR ESR1 SGPL1 TIPARP SCARB1 RDH12 PNPLA4 DHRS2 AKR1D1 DHRS4 AKR1B1 UGT1A1 AKR1C2 CYP19A1 AKR1C1 BCMO1 PLEKHA1 FSHB FDX1L CYP3A4 CYP11A1 WNT4 RBP4 HSD17B7 HSD17B12 STAT5B UGT2B11 UGT1A7 RETSAT ALDH1A2 ADH7 ALDH1A3 AWAT2 FDX1 STARD3 HSD17B1 CYP26B1 PLB1 CYP11B2 RDH13 RDH8 HSD17B3 LHB CACNA1H BCO2 RDH5 PPARGC1A SRD5A3 HSD17B11 STARD5 CYP26C1 RDH14 HSD3B2 DGAT1 RBP1 ECE1 ALDH1A1 DGAT2 SHH AKR1C3 ADH4 DHRS3 CYP1B1 HSD17B14 TTR UGT2B4 SRD5A2 CYP17A1 HSD17B4 CYP21A2 ASMT CYP11B1 AANAT SDR16C5 STAR RDH11

GO_NEGATIVE_REGULATION_OF_PROTEIN_METABOLIC_PROCESS Any process that stops, prevents, or reduces the frequency, rate or extent of chemical reactions and pathways involving a protein. PARP10 TAF9 PPP2R4 PSMA6 PINK1 DUSP10 METTL3 ANAPC4 PDCL3 RABGEF1 EIF3A RAPGEF1 MMP9 ITM2A SERPINF2 FMR1 USP47 DCN HAP1 EIF4E NUP62 PID1 PAQR3 SEMA4D RPL13A PAX5 WNK2 CDK5RAP3 MTF2 UBAC2 PSMD10 ATG14 SFTPD RASD2 IRAK3 NLRP6 WFDC10B LRRC4 TFPI NCK1 SPP2 CCDC23 CBL PTPRR NOC2L INHBB SH3RF1 SENP2 WFDC6 MEN1 PROL1 MARVELD3 ILF3 OPHN1 EZR PSMA3 NR1H2 YWHAE N4BP1 TSC2 PTPRJ ITCH KAT2B INCA1 SERPINA12 RGS14 RGN XDH CALR SIRT3 CST11 FLRT2 LRRTM3 PAK2 SRP9 HDAC6 C4B CLU NCK2 CTDSP2 PSMD6 BIRC6 C3 CNOT6 PSMB1 KLF4 NANOS2 PSMA4 GBA PDCD4 EIF6 DAPK1 PARK7 PSMD7 COL7A1 USP17L2 SERPINH1 SMAD1 C1QL4 PURA GAPDH RELA RAF1 HBXIP NLRP12 ZFP36 WFDC13 PLAT SPRED1 TNRC6A PSMC3 NXN GREM1 NGFRAP1 SH3BP5 CPEB2 TPR HFE KIRREL MECP2 PEBP1 DUSP14 SPINT3 BIRC8 RNF4 PSMD8 BUB3 FLNA FBXO7 CST2 SPINK1 ATF3 NELL1 APP CAST MYADM RPS27A CD276 BIRC5 ZNF675 TESC TRIM21 NLE1 SCO1 SERPINA5 DNAJC3 DAG1 PRR5L BGN GTPBP4 MT3 LEMD2 AQP1 PSMF1 ROCK1 SENP1 DNMT3B WNT9A PKIB CDKN2B BAK1 TIPRL CAPRIN2 FLRT1 SPINK7 FOXP3 PSMB8 NLRP2P SPINK8 SRRT AMBP PRKAR2B SERPINE2 BIRC2 BAG5 SFRP2 LRRC19 CRYAB WNK1 SNIP1 GPD1L RNF139 TNF CSTB PWP1 LRRC4B FABP1 SIRT2 HRG PTTG3P PAX2 RPS3 PARD6A CDC16 CD59 ANAPC7 PSMD12 ADAR PPP1R1B ANXA2 PTEN NCKAP1L GNL3L FLCN P2RX7 SESN2 CRY2 BANP SERPINA11 SSPO DMD IL1B YWHAG SAMD4B MAP2K5 PSME2 UPF1 PODNL1 DDIT4 SMYD3 TNIP1 NANOS3 ITGB3 SERPINA6 APOE SOCS6 NAIP CAPN3 SERPINB10 DVL1 PTTG1 PSEN1 TLK2 SLC9A3R1 USP19 BAX STAT3 PRG3 SPOCK1 ATP13A2 DAB2IP LMO3 LAX1 MECOM ANAPC16 RENBP MAD2L1 ASPN SPINK13 CTDSP1 CRTAP LIMK1 ABCA7 IGF2BP1 SPOCK2 ZFPM1 CST5 PLK1 ZMYND11 HDAC3 PPP1R11 CIB1 LATS1 CBLC SNCA ACOT8 EIF2C4 GRB7 CRY1 GHRL HHEX USP14 HMGCR IGBP1 PPM1F YWHAB SMPD1 SLPI LCN1 GADD45A BCOR WFDC8 SH3BP5L SERPINC1 PSMB10 IGF1R PKIA FBXO43 LPA UCHL1 PRDX5 PPP1R1A BST2 SRCIN1 UBE2B MAGEA2B SPRY1 ZNF451 CDC27 PSMC4 TNRC6B KDM4A EIF2C1 NMI ANAPC2 OTUB1 TGFB1 CSTA INSM1 SPINT2 MYOCD DNMT1 PARD3 RGS4 EGFR INS ANG DEDD FETUB PAPLN ENPP1 SPINK14 MDM4 PMEPA1 MAGEA3 LRP6 CPEB3 DNAJC10 ISG15 CRIM1 NF1 F2 DUSP8 ATF4 BMP7 CNOT8 CST7 UFL1 SMAD4 XIAP SOCS4 SERPINB3 STRAP HGF DHCR24 HERPUD1 TNFAIP8 COL28A1 PPM1E EPB49 CEP85 BANK1 PAX6 FKBP1B SNX6 CTDSPL PRKAG2 OVOS AKT1 RFFL SIRT1 LIMD1 NF2 EIF3E FKTN PRMT3 CRIPAK UBE2D1 CSTL1 IKBKB TP73 TFAP4 HSPH1 CST4 MAPK8IP1 EIF2AK4 CHAC1 CPB2 ANXA8 CALM3 PHB NCOR1 MTOR CD27 NFKB1 PSMB7 MKL1 SYNJ2BP NRG1 RNF222 EDNRB PDE4D EIF2AK3 SMARCC1 TRIM40 WTIP FOXO1 FGFR1OP CEACAM1 CDKN1C SPINT1 UMODL1 ENC1 LRRK1 IL6 HSPA1A SOCS1 SPOPL RC3H1 CDKN2D NOG DNAJA1 CARD18 DUSP9 ASB1 BMP4 SERPINI1 PRKRIP1 UBE2G2 WFDC2 AZIN1 WWTR1 USP4 SHB CST1 SERPINF1 LXN PUM1 CNOT7 FXR1 CPEB4 INHA CDKN2C RPS6KA1 GADD45B CDKN1A SMAD6 ZC3H12D SNX12 SPINK4 EIF2AK2 ITIH3 PATL2 DTNBP1 KNG1 MOV10 PPP1R1C TSPO UBASH3B C4A VTN LIN28A CST9 IREB2 ARRB1 LRRC66 PSMA2 TRIAP1 SFRP1 SOX4 RAN SOCS5 IL2 SPI1 RGS3 FYN DPEP1 WEE2 INPP5K TMEM59 FRY SERPINA7 KDM3A LILRB1 UBA52 NOS2 IGF2BP2 IFI6 COL6A3 CEBPA BCL3 DEPTOR LIN28B C5 FHIT HNRNPA2B1 GCLC GFRA2 GNB2L1 FBXO5 MYC USP25 TRIB2 TLR4 EIF4EBP1 PARK2 C3orf33 HMG20B HNRNPR PML HSP90AB1 FKBP1A SERPINE3 R3HDML SPINK6 PSMA5 PPP2R1A SMAD2 ANAPC10 GMFG GSTP1 TOB1 TRIM39 TNFSF14 TARBP2 SPRED2 CHAD CTBP1 MICAL1 CISH SMAD3 EIF4EBP3 CDK5 CD44 UBR5 INPP5J SERPINB7 NGF TWIST1 ARL6IP1 UBXN1 PRKCD LRRK2 PSMD1 SERPINB13 RTN4RL2 UBE2J1 CR1 HDAC8 TIMP3 LOC100127983 SERPINA1 SIRT4 SERPINB11 CDKN1B CBLB HNRNPD CORO1C A2ML1 SAMSN1 GPS1 PRKAR1B SERPINA10 PYDC1 PTPN22 WFDC5 MAGEA2 SERPINB6 PSMB5 BRCA1 CAMK2N1 H2AFY PAIP2 CDK1 AHSG PSMD3 SYNCRIP INPP5F MAS1 SERPINA2 TFAP2B ELANE JUN PKN1 CLN8 C4BPA HIPK3 AATF PCID2 SPINK9 TRIB1 TRIM27 PTTG2 FOXM1 CNOT3 LEPRE1 IMPACT PIBF1 NGFR IGFBP5 QKI IGFBP3 DNAJB6 HHATL DGCR8 FXR2 GPX1 SERPINA3 TRIM71 GPS2 KIAA1967 DERL3 PSMA7 EIF2C2 UBE2E1 C2orf28 NTRK3 MTM1 LATS2 DUSP16 MVP DDX3X SPINK5 GLMN MAPK7 MEX3D OGT DERL2 UBB CALM2 UBC RPS6KA6 ZC3H12A PSMD2 PPARGC1A RB1 EIF4EBP2 GPI CSNK2A1 PSMC1 DUSP1 PSMD5 METTL14 ZFYVE28 PER1 EIF2AK1 KAL1 SMAD7 PSMB2 CSK EIF4ENIF1 GAS6 PLAUR OS9 SKI SERPINB5 ITIH1 PSMC2 FZR1 MLXIPL ILK SPINT4 CHP JARID2 RPS6KA3 EIF2B4 C18orf1 INHBA ENG PRKAR1A CDK2 CDC23 C4BPB SPINK2 TIMP2 PRKRA DACT1 EIF4E2 HSPA1B CST9LP1 SPRY4 RNF128 CLEC16A A2M TSC1 PSMD11 IDE CNOT2 CNKSR3 APBB1 WAC INPP5D DLG1 HSPB1 LRP5 CIRBP BIRC3 HYAL2 CDKN2A ABL1 PPP2CA IL1R2 LRRC15 F2RL1 LAMP3 SOCS7 AGT GPC3 ERLEC1 PRKDC CDC26 TNFAIP3 ITIH6 PINX1 PRKCG FMN2 ERRFI1 PTPRC PSME1 RBM4 THY1 SIGIRR PSMA1 EIF2B3 GABARAPL2 AVP NANOS1 CAMK2N2 APC LIF PSMC6 POR DUSP6 PTPN2 SMARCB1 PPP5C C19orf29 CELF4 XPO5 ZBED3 MASTL ATP2B4 PRKCE CHORDC1 PTPN6 CD55 PAFAH1B1 ITIH5 PPP1R15A DUSP19 VPS28 PRKCZ LRRC4C SERPINB4 BIRC7 UCHL5 SDCBP AKT1S1 ANAPC5 RASSF2 GRIN2C BACE2 PTPN1 AIDA CD300A CAV1 PROS1 PSMC5 PHF1 TRIP12 RILP PYCARD ITIH4 WFDC1 PI15 DUSP5 SERPINB2 PSMB3 YOD1 LRRTM4 SFN EPRS WFIKKN2 SOCS2 FNIP1 RAG1 G6PD ZNF540 BAG6 SPINLW1 ITGAV WNT1 CDC20 TIMP1 GAS1 ZCCHC11 SNX25 HDAC1 GP1BA COL4A3 HMSD BUB1B SOCS3 IFI16 RNF149 PZP CST9L DUSP26 ITGB1BP1 LYN FLRT3 PRKAR2A C3P1 FLOT2 CCNB1 TIA1 ECM1 PSMD9 PPP1R16B OVOS2 DAPK3 DICER1 SERPINA9 DNAJC1 HRSP12 WFDC3 PPEF2 CNOT1 FAM129A KDM5A PBLD PI3 SUPT6H FURIN RECK SHH PBK ADC STK39 CALM1 ITIH2 SET SERPINA4 SERPIND1 NTF3 GBP1 SRC CNOT6L NOL3 RNF34 IBTK MIF RQCD1 PAIP2B CRYBA1 EIF2C3 BTG2 ITM2B SORL1 GLG1 PSMD4 ANGPT1 CRB2 TARDBP RANBP9 CAPRIN1 CST3 PPAP2B FOXJ1 KBTBD5 KHSRP TRAF3IP1 RGS2 DKK1 WFIKKN1 CAV3 UBE2C EPHA1 MBIP PIH1D1 ANAPC1 RGP1 MAD2L2 IGF2BP3 EIF2B5 TBC1D10C TFPI2 DUSP4 BCL2L12 SAMD4A SIN3A SUFU RTN4R VIL1 NLRC4 BDKRB1 LAG3 C11orf51 PRNP PIAS3 VEGFA GHSR IL10 CDK5RAP1 GIGYF2 SWAP70 LRRTM1 SNTA1 CTNNB1 MRPL44 EIF2S1 EIF4A3 SERPINB9 PSMB4 THBS1 CST6 SPOCK3 PSMB9 UCN RARA SERPINE1 CPEB1 STK38 FBLN1 DUS2L CD109 C7orf30 CPAMD8 ZFP36L2 PER2 PRDX3 PDPK1 KIRREL2 APLP2 FABP4 SERPINI2 ITM2C ARRB2 HEXIM1 PTPN3 SERPINB12 EDN1 UNK SERPINB1 PCSK1N SERPINB8 TNRC6C TRIB3 SIAH2 CASP8 GPRC5A ZGPAT USP44 PKIG KLF15 SVIP SF3B3 PSMD13 MDM2 ZFP36L1 PPP1R15B PSMD14 CLN3 WFDC10A HEXIM2 MLLT1 ANAPC11 PI16 DUSP18 SERPING1 LEF1 DUSP2 ADARB1 GPER WFDC12 JUB DUSP7 ACER2 NDRG2 TAOK3 HDAC2 CELF1 AGAP2 PSME3 SNX3 TIMP4 GNAQ NYX RTN4RL1 TAF7 GRIN2A LRTM1 PSMB6 DUSP21 CARD17 PIN1 DUSP3 ASH1L PPP4R4 CARD16 DBNDD2 GMFB KDM1A GIPC1 SLIT2 PICALM SPRY2 DUSP22 KIAA1432 CST8 TINF2 NPM1 NR2F2 PTK6 BDKRB2 PODN DROSHA VRK3 ROCK2 ADIPOQ

GO_T_CELL_SELECTION The process in which T cells that express T cell receptors that are restricted by self MHC protein complexes and tolerant to self antigens are selected for further maturation. ZFPM1 THEMIS FAS IL6 BCL11B BRAF GLI3 SRF CD28 CD3E CD4 CCR7 SHH ZAP70 SYK CD74 ITPKB CD3D FOXP3 BCL2 LY9 BATF JAG2 AIRE IL4 SLAMF6 GATA3 DOCK2 IL15 IRF4 CD1D STK11 CARD11 STAT6 SPN

GO_ICOSANOID_TRANSPORT The directed movement of icosanoids into, out of or within a cell, or between cells, by means of some agent such as a transporter or pore. Icosanoids are unsaturated C20 fatty acids and skeletally related compounds. PLA2G2D NMUR2 PLA2G1B PLA2G4A PLA2G12B PROCA1 BDKRB2 PLA2G5 NOS2 ANXA1 PLA2G2E PLA2G10 PNPLA8 PLA2G4F NMB PLA2G2C PLA2G2F DRD2 DRD4 PLA2G2A ACE DRD3 LEP OC90 SLCO2A1 ABCC2 ABCC4 PLA2G3 PLA2G12A SLCO3A1

GO_POSITIVE_REGULATION_OF_NEUTROPHIL_MIGRATION Any process that activates or increases the frequency, rate or extent of neutrophil migration. IL1A IL1R1 CXCL1 C1QBP CXCL2 RAC2 CD74 THBS4 LBP C3AR1 IL23A XCL1 CXCL3 IL8 CCL19 RAC1 ADAM8 C5AR1 CXCR2 TIRAP CCR7 NCKAP1L CCL21 CAMK1D DAPK2

GO_DNA_STRAND_ELONGATION The DNA metabolic process in which a DNA strand is synthesized by adding nucleotides to the 3' end of an existing DNA stand. PARP3 FEN1 NBN LIG3 DNA2 LIG1 POLA1 POLD1 PCNA PARP1 POLD3 DCLRE1B POLD4 PRIM1 PARP2 GINS2 RAD50 RFC3 PRIM2 TERT GINS1 RNASEH2A GINS3 GINS4 RNASEH1 MRE11A RFC4 LIG4 POLA2 POLD2

GO_SNRNA_PROCESSING Any process involved in the conversion of a primary small nuclear RNA (snRNA) transcript into a mature snRNA molecule. EXOSC5 EXOSC3 EXOSC4 INTS7 CPSF3L INTS9 EXOSC6 EXOSC7 EXOSC9 INTS12 EXOSC2 TUT1 INTS6 INTS8 INTS1 C16orf57 INTS4 EXOSC8 INTS10 INTS3 INTS5 INTS2

GO_RETINA_HOMEOSTASIS A tissue homeostatic process involved in the maintenance of an internal equilibrium within the retina of the eye, including control of cellular proliferation and death and control of metabolic function. AZGP1 POC1B ALB MKKS POTEF USH1G RP1 CDHR1 ACTG1 IGHA2 PRDX1 ARMS2 CIB2 CDH23 DRAM2 POTEI CLRN1 CNGB1 TF RP1L1 USH2A NXNL2 PIGR CROCC BBS4 LTF SPATA7 POTEE AIPL1 RHO POTEJ ZG16B PCDH15 CDH3 PROM1 BBS2 GPR98 MAK RDH12 ERCC6 CST4 SOD1 TULP1 NPHP3 B2M IGHG3 NPHP4 PIP VHLL ACTB BBS10 TUB LCN1 KRT1 LYZ IGKC DFNB31 USH1C BBS12 IGJ RPE65 IQCB1 HSPB1 IGHA1 ABCA4 PROL1 NXNL1 WDR36 BBS1

GO_POSITIVE_REGULATION_OF_RECEPTOR_INTERNALIZATION Any process that activates or increases the frequency, rate or extent of receptor internalization. NTF3 SYNJ2BP WNT3A AHI1 PICK1 FMR1 SFRP4 CD63 TBC1D5 PCSK9 ANGPT1 VEGFA SCYL2 ARRB1 DRD2 MAGI2 GREM1 ARRB2 GH1 SYK ATAD1 PLCG2 SELE

GO_GLOMERULUS_DEVELOPMENT The progression of the glomerulus over time from its initial formation until its mature state. The glomerulus is a capillary tuft which forms a close network with the visceral epithelium (podocytes) and the mesangium to form the filtration barrier and is surrounded by Bowman's capsule in nephrons of the vertebrate kidney. The glomerulus is part of the nephron and is restricted to one body segment. MTSS1 ACTA2 SULF1 HES1 PECAM1 KIRREL3 CD24 PDGFB NOTCH1 CD34 ANGPT1 PDGFRA EGR1 BASP1 OSR1 NOTCH3 WT1 COL4A4 AMPD2 LGR4 PTPRO COL4A3 GPR4 NUP93 WWTR1 HEYL ANGPT2 MPV17 TCF21 VANGL2 FOXJ1 FOXC2 PODXL MYO1E IQGAP1 MAGI2 BMP4 SULF2 PLCE1 PROM1 LHX1 NPHS2 BCL2 NID1 KLF15 ENPEP LAMB2 PDGFRB MEF2C JAG1 NPHS1

GO_CELLULAR_AMIDE_METABOLIC_PROCESS The chemical reactions and pathways involving an amide, any derivative of an oxoacid in which an acidic hydroxy group has been replaced by an amino or substituted amino group, as carried out by individual cells. EIF3E AKT1 RPL4 GGTA1P PABPC1 RPS4Y2 TARS MTHFS SLC25A18 GM2A GSTA5 EIF2S3 RPL28 SLC19A1 ST8SIA4 NDUFA7 CYP2C9 MME RPS5 CERS3 F2 ERO1LB SLC25A10 SLC25A5 CPEB3 VARS RPL7A MTERFD2 MRPS22 GARS TARS2 NAMPT NARS MRPL33 MGST2 MIPEP GTPBP2 SARS2 EIF3I MRPS33 EIF4E3 EGFR EIF5A RPLP0 SEC11A CTSH MRPS2 RPL39P5 TUFM AARS2 EIF4G2 CERS6 CERS5 MTHFD2L RPL6 MRPL17 STAT5A SMPD1 ARG1 RPS9 PPA1 SLC25A24 MRPL46 EIF4H RPL29 FSHB HARS CLIC1 SLC25A1 BDH2 MRPL34 FAM57B GSTK1 RPS6KB2 SMPD4 ST6GALNAC4 PLA2G15 C7orf36 F10 MRPS34 EIF4E1B SLC25A48 EEFSEC PSEN1 RPL18A CPD ORMDL1 IMMP1L ST8SIA2 EFTUD2 EIF4EBP1 GUF1 AMDHD1 GCLC CEBPA GHR MRPL41 PTCD3 MRPL39 ABCF1 DEGS2 UBA52 CGA BACE1 MRPL14 MRPL10 DEGS1 ASMT FTCD MRPL4 FARS2 RPL36AL C9orf3 GATC DPEP1 ST6GALNAC2 IGHMBP2 MRPS31 ACY1 NPEPPS PCSK5 MCART6 EEF1A2 ECE1 RPS16 SLC25A20 ARG2 PTBP1 RPL3L EIF2AK2 SLC25A4 DLD ETF1 MRPL53 ALDH1L1 EIF2S2 SPCS1 SSB CPEB4 RPL7L1 CHCHD1 SHMT1 SLC25A15 ALOXE3 TRHDE HRASLS5 RPL22 EEF2K NAT8B DDX3Y SLC25A6 FTSJ1 CAD P2RX1 GFM1 MRPL22 MCART1 EIF3M RPL18 NEU3 RPL12 GTF2H2 SLC25A39 EIF2AK4 CPXM2 GSTM5 RPS21 MRPL16 C15orf42 CHAC1 EIF1B MMACHC AASDH RPL38 GGT6 ENPEP MTHFR EIF6 MRPL38 PARK7 GTF2H3 RPL26 EIF5A2 SLC25A3 GGT7 RPS14 RARS2 RPL35A RPL17 MRPS11 CNDP1 GBA SGMS1 RPS3A MRPL19 ACE CLIC3 ST8SIA6 PGCP MRPL15 AANAT OTC RPL21 ENPP7 RPL35 SLC25A44 MRPS24 RPS17 PCSK7 GSTT2B PSTK FOLR1 EIF3CL MRPS5 MRPS26 AEBP1 MRPL28 SGPL1 RPLP2 EARS2 RPL5 DAP3 SAMD8 XPNPEP1 MRPL45 MTHFD1 NMRAL1 ITGB8 EIF3H DHFRP1 RPL13A UGCG DHFR MRPL30 GSTO2 ASAH1 PROC EIF1AD MRPS6 EIF4E EIF3G MRPS35 QARS EIF3A SLC25A43 MRPS23 LARS2 GDAP1 MRPL55 RPL15 GGTLC2 PTGS2 PAIP1 CERK RPL13 SPPL2A RPL24 SLC25A29 CPA3 PDF RPS15 NUDT12 SLC25A21 C9orf41 TNIP1 MRPL54 AARSD1 ST3GAL2 UCP1 MRPS9 FARSB LARP1 CLIC6 CPXM1 DMD P2RX7 MRPL48 GAL3ST1 GLRX2 EIF1AY NEU4 RMND1 RPL32 RPL10A RPS11 SELT RPS3 EFTUD1 RPL9 PPA2 NOA1 MTFMT MRPS10 RPL11 ACE2 SLC25A30 CLN6 EEF1D MRPL35 CLIC4 OPLAH NSMAF UCP3 SPPL2B RPL27 GBA3 EIF4A2 SLC25A17 SPTLC1 SLC25A33 TSHB CTNS SLC25A45 YARS2 PRHOXNB RPS27A SMPD3 NHP2L1 GADD45GIP1 RPL23 RPS10 RPS7 EIF5B GSTA3 MRPS16 HTRA2 MRTO4 COPS5 SPTLC2 HEXB CPEB2 MTRR PARS2 SHMT2 EIF1AX CDKAL1 RSL24D1 ECE2 PCSK6 RPL22L1 ORMDL2 GSTT1 PCBP2 AURKAIP1 SMPDL3B SLC25A47 NARS2 SPCS3 FDXACB1 CPS1 MCART2 MRPL3 PROZ RPL10L ASAH2 EIF4B EEF2 MRPS14 EIF2B5 IGF2BP3 RPS27 ST6GALNAC5 SLC25A42 GGT3P SLC25A14 RPS15A LRRC47 ABTB1 HARS2 CORIN RARS GSTM2 GSTM3 SEPSECS IDH1 UROC1 SLC25A31 RPL10 EIF2A EIF1 RPLP1 MRP63 AGK FURIN AQPEP SPCS2 PAM C12orf65 RPS24 SARS CERS1 ALOX12B SLC25A22 RPL8 EIF3C RPL23A POMC AARS NEU1 IMMP2L MRPL36 MRPL42 MRPL23 MRPS36 HM13 GSTZ1 PET112 DHX29 DNPEP MRPL37 MRPL49 PABPC4 MRPS30 CDC123 G6PD APEH ASS1 TPP1 RPL36 SLC25A13 TRMT112 EIF3K SLC25A11 ORMDL3 METTL17 ASL EPRS MRPL18 EIF3B GGTLC1 HAL NCSTN C7orf44 MTHFD1L PROS1 SEC11B RPL41 QRSL1 CARS2 F9 MRPL24 PM20D1 CTSZ RPS4X SLC25A28 GSTM1 WARS2 DALRD3 EIF3J LTA4H F7 UCP2 HBS1L EIF3L RPS4Y1 RPS8 MRPL21 MRPS12 SLC25A41 MRPL13 SLC25A32 ACER1 RPS2 ACER2 SLC25A2 CLIC5 WARS APOA1BP SLC25A12 MRPL9 MRPL47 CARKD CTSG RPL14 MRPL40 GSTA1 LNPEP TRNAU1AP CLN3 GFM2 RSL1D1 CPZ MTIF2 RPL7 ST3GAL4 DHPS PCSK1N MARS2 MRPL43 RRBP1 ICT1 ST3GAL5 MRPS18A NAPRT1 PCSK1 SLC25A26 NAT8 EEF1G RPL26L1 SLC25A38 UNC13A RPL37AP8 ABCE1 EIF3D RPL37A YARS PTRH1 RPS20 CPEB1 NEU2 SLC25A25 RPS23 RPS29 SOX11 MCTS1 ALDH1L2 RPS27L EIF2S1 CNDP2 MRPL44 DIO2 EIF4EBP2 NHP2 RPS26 RNPEPL1 RPS18 MRPL27 GGT1 LHB EIF2B2 MTRF1 RPS12 EIF2C2 RPL31 SLC25A16 N6AMT1 MRPL51 GSPT1 HAGH STAT5B EEF1A1P5 EIF4A1 VARS2 ANPEP GPX1 GALC RPL39L ALDH5A1 GSR GGT2 C20orf173 RPL37 AIMP1 RPSA SEC11C PSEN2 POLG2 MRPL32 MTERFD1 SLC25A27 EIF4G3 SLC25A35 UGT8 RPL34 SLC46A1 GLA CLN8 SPTSSA SGMS2 ETHE1 GLO1 PGLYRP2 DARS PTGIS MRPL1 GGT5 EEF1A1 BGLAP EIF2D SLC25A19 MARS B4GALNT1 ERAP2 NAGS ST8SIA3 REN MRPL2 DARS2 FPGS NGF EIF5 LARS RPL3 RPS28 ST6GALNAC1 SLC25A40 MRPS15 HMGN5 CLN5 MRPL11 LOC440434 MTIF3 SMPDL3A BECN1 ALLC SCG5 MRPL52 GGCT GSTP1 PPP2R1A SOD1 GSS LOC255308 GCLM MTHFD2 FARSA SLC25A34 ADAMTS13 RPL13AP3 BACE2 TARSL2 RPS13 SMPD2 MRPS18B CHAC2 RPL36A RPLP0P6 EIF3F IARS GSTM4 MRPS18C MRRF EEF1E1 THOP1 MRPS25 ST3GAL1 ST6GALNAC6 EIF2B3 ST6GALNAC3 ST3GAL3 CARNS1 CERS4 EIF5AL1 TAPBP RBM4 GSPT2 AGT GBA2 MRPS7 TSFM CPN1 RNPEP PPP2CA RPL30 GGTLC3 DENR MRPL20 MRPS28 IARS2 CERS2 CARS IMP5 MRPL50 GSTT2 IDE GTPBP1 SPPL3 SLC25A37 GSTO1 RPS6 RPL19 NLN EIF4E2 SLC25A23 KARS EEF1B2 RPS19 GSTA4 GSTA2 SLC25A36 AIMP2 MRPS27 SECISBP2L ACER3 RPL27A GDAP1L1 ERAP1 EIF2B4 SECISBP2 MGST1 GCN1L1 GAS6 RPL39 EIF4G1 PADI6 PCSK2 CPE EIF2B1 CLIC2 MTRF1L MRPS17 MRPL12 CPM COL4A3BP MRPS21 RPS25 CMA1 ST3GAL6

GO_POSITIVE_REGULATION_OF_HAIR_CYCLE Any process that activates or increases the frequency, rate or extent of the cyclical phases of growth (anagen), regression (catagen), quiescence (telogen), and shedding (exogen) in the life of a hair. HPSE FST GAL FOXN1 TNF TGFB2 TERT MSX2 KRT17 TRADD WNT10B

GO_DOUBLE_STRAND_BREAK_REPAIR The repair of double-strand breaks in DNA via homologous and nonhomologous mechanisms to reform a continuous DNA helix. GEN1 SFPQ ZFYVE26 C16orf73 BABAM1 XRCC6 EME2 DTX3L NSMCE2 KDM2A PARP1 VCP TP53BP1 WDR48 UIMC1 SHFM1 ERCC4 PAXIP1 KIAA0415 C19orf39 APLF FBXO18 RPA3 XRCC6BP1 EYA1 KIAA0146 HIST1H4A RPA1 MAD2L2 KIAA0430 UBE2V2 C17orf104 C9orf142 INO80 CDC7 FAN1 NHEJ1 NSMCE1 RBBP8 BACH1 POLM MCM8 BARD1 C7orf49 HIST2H4B ESCO2 DCLRE1B NUCKS1 SETX MMS22L MTA1 PALB2 SLX4 GINS2 RECQL4 SMC5 HIST3H3 SWI5 XRCC3 MRE11A CIB1 RAD51 HIST1H4J LIG3 HIST2H4A RNF8 MND1 PARP3 SFR1 CDC45 DCLRE1A RAD52 POLA1 C20orf29 SUMO1 ZSWIM7 RAD51C BRCA1 MORF4L1 KAT5 LIG4 PRPF19 RNF168 BRCC3 ERCC1 UVRAG EYA3 PSMD14 RAD21 PRKDC BRE BRCA2 XRCC2 RAD51AP1 HUS1 H2AFX SETMAR TRIP13 EXD2 SMC6 RAD50 BRIP1 SMARCA5 POLN MSH2 POLL HIST1H4L RNF138 HERC2 GINS4 RIF1 HIST1H4C DNA2 MCM9 FEN1 WHSC1 CHEK2 BLM ACTR5 DCLRE1C CCDC155 RAD51D HELQ HIST1H4I HIST1H4H HIST1H4D RAD51B RECQL PARP9 HIST4H4 DMC1 YY1 XRCC5 NBN RECQL5 MLH1 HIST1H4K GGN WRN CDCA5 KDM4D HUS1B BAZ1B UBE2N MDC1 HIST1H4B RPA2 XRCC4 FAM175A OBFC2B EME1 RAD54L OBFC2A DDX1 POLQ HIST1H4F TDP2 ATM SLX1B PIAS4 REC8 RAD21L1 PAPD7 SMARCAD1 TONSL TDP1 SLX1A HIST1H4E XRCC1 RAD54B APBB1

GO_REGULATION_OF_HISTONE_MODIFICATION Any process that modulates the frequency, rate or extent of the covalent alteration of a histone. EID1 CDK9 CHTOP SNW1 BCOR RAPGEF3 ZNF304 ING2 MTF2 EED CAMK1 PRKD1 PAX5 ATM PHF19 PRKD2 ISL1 PAX7 OTUB1 TGFB1 FOXP3 TP53 CCNB1 SNAI2 ZNHIT1 PAF1 ZNF451 AKAP8 KDM4A DNMT3B BRCA1 UBE2B H2AFY NOC2L SREBF1 UCN PIWIL2 MLL4 MECP2 PINK1 CTCF CTNNB1 PHF1 MAPK8 DMRTC2 TRIP12 CTBP1 WDR61 BRD7 RTF1 JARID2 RDBP SNCA TWIST1 UBE2N SKI FMR1 UBR5 MAPK3 GCG NSD1 HIST1H1B NOS1 KDM1A PIH1D1 CHEK1 GATA3 SMAD4 CTR9 TADA3 DPPA2 IWS1 AUTS2 C6orf89 CTCFL TAF7 BCL6 AKAP8L ZNF335 FLCN PYGO2 GATA2 VEGFA SART3 SIRT1 PPARGC1A USP17L2 SIN3A HDAC5 OGT IL1B WBP2 MTHFR RPS6KA4 SUPT6H RNF20 NIPBL RNF40 MLL CAMK2D KDM5A DNMT1 SETD7 JDP2 ARRB1 KAT2A TADA2A ZNF274 MUC1 RPS6KA5 PRDM12 MYOCD LIF PAXIP1 SMARCB1 TET1 KDM3A TADA2B ATRX RUVBL2 GFI1 MYB NAP1L2 SET GFI1B SPI1 GCFC1

GO_REGULATION_OF_CELL_MORPHOGENESIS_INVOLVED_IN_DIFFERENTIATION Any process that modulates the frequency, rate or extent of cell morphogenesis contributing to cell differentiation. Cell morphogenesis involved in differentiation is the change in form (cell shape and size) that occurs when relatively unspecialized cells acquire specialized structural and/or functional features that characterize the cells, tissues, or organs of the mature organism or some other relatively stable phase of the organism's life history. FN1 SSH3 SEMA6B FBXW8 MYADM ARHGEF1 CAPRIN2 NGEF CACNA1A LIMS1 SEMA6A LPAR3 MT3 PLXNA4 PAK3 RCC2 OBSL1 ROBO2 RNF6 ILK FLNA PAFAH1B1 PTPRD SSH1 CAMK2B RELN PTEN SEMA3B DAB1 WNT5A GDI1 APOE NTN1 SPP1 ADNP NR2E1 PLXNB2 LRRC4C PLXNA1 TNR TRPV2 SEMA4C MAG SHANK3 PLXNA2 TLX2 ABL1 FGB CDK5R1 C1QBP RET EPYC PPP1R9A RHOA AP1AR SEMA5B THY1 SEMA4F BAI3 SS18L1 STK25 SLIT3 DOCK5 PREX1 APOA1 SEMA4D ZFYVE27 CDH4 CORO1C SSH2 WDR36 CTTN CUX2 S100A10 RAP2A BAIAP2 MAP1B NRP1 CDK5 CRK FEZF2 DCC ARHGAP4 EFNA5 LRRK2 LRP4 NGF SEMA3E RND2 DSCAM POU3F2 ANKRD27 OMG SEMA4G UST STK11 NTRK3 VAMP7 MARK2 TIAM1 GRIN1 BDNF KANK1 CXCL12 CRKL BMPR2 ARPC2 WNT7A GSK3B L1CAM MAPT IL1RAPL1 PLXNC1 RREB1 IST1 NGFR NFATC4 TNIK RAB11A CALR RAC1 DNM2 CHRNB2 CFL1 SHOX2 NDEL1 GOLGA4 SARM1 KEL SEMA3G FGF13 EPHB3 SEMA3A SEMA3C CRABP2 FSTL4 GORASP1 CNTN2 PTK2 C1orf187 ARHGAP35 XK SEMA6D SPG20 SRF EEF2K PLXNB1 PLXND1 MAP2K1 KIF13B MYOC SDC2 ID1 DOCK1 FBLN1 NRG1 ROBO1 DNM3 NLGN1 SLIT2 POSTN KALRN SIPA1L1 RUFY3 BHLHB9 OLFM1 NEDD4 TRIOBP DPYSL2 LRP8 NEUROG3 NRCAM METRN LRRC16A NLGN3 ULK2 MFI2 ITPKA ISLR2 RAC3 YWHAH KNDC1 CHRNA3 PLXNA3 PPP3CA TTL ACTN4 TRPC5 SKIL SYNGAP1 MACF1 HAS2 LZTS1 SLIT1 CHN1 FGA RAPGEF2 SEMA4A FGG AMIGO1 NET1 TNFRSF12A RTN4 EFNA1 ANAPC2 CUX1 ITGB1BP1 PDLIM5 SRCIN1 SEMA7A FAM21C IFRD1 EPHA7 CDC42 NKX6-1 OGN TBR1 OLFM4 PSEN1 CIB1 SEMA4B SEMA3F LIMK1 PQBP1 PTPRO MEGF8 SKOR2 EPHA4 KIAA1598 RAB21 ARHGEF7 BARHL2 CUL7 WNT3 CDKL5 TACSTD2 VEGFA EPB49 DISC1 WNT3A LINGO1 RTN4R LOC100507050 SEMA5A UNC13D NELF TWF2 NEFL PLXNB3 BRAF NTRK2 TRPC6 CAPRIN1 FMOD EPHB2 GBP1 ARHGDIA SEMA6C SEMA3D

GO_REGULATION_OF_CELL_MATRIX_ADHESION Any process that modulates the frequency, rate or extent of attachment of a cell to the extracellular matrix. LIMS1 S100A10 CASK C11orf34 TEK MYF5 ROCK1 CLASP2 RCC2 ITGB1BP1 MMP14 TSC1 RAC1 UTRN RASA1 PPM1F COL16A1 CORO1C EPB41L5 NINJ1 MYOC PIK3CB ILK HOXA7 WNT4 EPHA3 CIB1 EFNA5 BCAS3 ACVRL1 LDB1 SERPINE1 SEMA3E CDK6 PTK2 THBS1 SMAD3 PHLDB2 GREM1 PLAU CCL21 ARHGAP6 ROCK2 CCL28 VEGFA BCL2 NF2 RHOD CDH13 FMN1 GSK3B SDC4 APOD EPB49 DISC1 PRKCZ CCL25 CD36 POSTN PLEKHA2 PTK2B GPM6B SLC9A1 EPHA1 IQGAP1 EMP2 PIK3R1 MINK1 BCL6 ADAM15 PTEN VEGFC WDPCP ACER2 HRG MACF1 DLC1 NF1 SRC KDR ONECUT2 ONECUT1 PTPRJ SFRP1 DAPK3 CDKN2A DDR1 CLASP1 SLK CSF1 CCR7

GO_DNA_DOUBLE_STRAND_BREAK_PROCESSING The 5' to 3' exonucleolytic resection of the DNA at the site of the break to form a 3' single-strand DNA overhang. RAD52 SLX1B SETMAR UBE2V2 NBN DNA2 ATM BLM RNF138 SLX1A BARD1 MRE11A EXD2 UBE2N KAT5 RBBP8 SLX4 RAD50 BRCA1 SMARCAD1

GO_REGULATION_OF_HISTONE_H3_K9_ACETYLATION Any process that modulates the frequency, rate or extent of histone H3-K9 acetylation. CAMK1 BRCA1 NAP1L2 SMAD4 HDAC5 GATA3 ZNF451 PIH1D1 MLL CHEK1 RAPGEF3 PIWIL2 SMARCB1

GO_NEGATIVE_CHEMOTAXIS The directed movement of a motile cell or organism towards a lower concentration of a chemical. WNT5A SEMA3B SEMA6B APOA1 SEMA4D ITGAV SEMA4A SLIT1 SLIT3 SEMA4G SLIT2 ROBO1 SEMA4F PLXNA4 SEMA3C SEMA7A SEMA5A SEMA3A SEMA6A SEMA3G ITGB3 FLRT3 SEMA6D RYK EPHA7 SEMA4C SEMA5B SEMA3D SEMA3E NRG1 SEMA6C RHOA NRG3 SEMA4B FLRT2 SEMA3F PLXNA3 PDGFA EFNA5

GO_EMBRYONIC_HEART_TUBE_DEVELOPMENT The process whose specific outcome is the progression of the embryonic heart tube over time, from its formation to the mature structure. The heart tube forms as the heart rudiment from the heart field. VANGL2 FOXC1 IHH LBX1 TBX20 NOTO WNT5A SETDB2 CCDC39 TRAF3IP1 MIB1 BBS7 FOXC2 ACVR1 MESP1 SOX18 FOXN4 BBS4 SUFU WNT3A IFT57 AHI1 MEF2C SHH MKKS FOLR1 IFT172 C2CD3 TBX2 HES1 IFT122 DNAAF1 ARL13B NOTCH1 HAND1 HIF1A PKD2 CCDC40 TBX3 NKX2-6 SOX17 NODAL TEAD2 SMO STIL ZIC3 GJA5 ENG CITED2 MED1 BBS5 HAND2 MICAL2 FGF8 RYR2 IFT52 NPHP3 SMAD3 NDRG4 SRF PSEN1 CTNNB1 TMED2 FOXH1 OVOL2 HHEX NKX2-5 DLL1 TGFBR2 CCDC103 YAP1 MEGF8 GJA1 GATA4

GO_CELLULAR_RESPONSE_TO_ALCOHOL Any process that results in a change in state or activity of a cell (in terms of movement, secretion, enzyme production, gene expression, etc.) as a result of an alcohol stimulus. DNMT3A KLF4 SMO KIAA0146 INHBA BLM ITPR2 PRKCE ABCA1 RAD51 KLF2 LRP8 GPLD1 GLRA2 ADCY7 OSBPL7 BRCA1 CDH1 JUP MLC1 UGT1A1 HMGCS1 CYP7A1 C18orf26 TP53INP1 AACS CTNNB1 CYBB KCNMB1 PTCH1 LRP6 GLRA1 SPI1 PRKAA1 CTNNA1 CCL7

GO_POSITIVE_REGULATION_OF_RECEPTOR_MEDIATED_ENDOCYTOSIS Any process that activates or increases the frequency, rate or extent of receptor mediated endocytosis, the uptake of external materials by cells, utilizing receptors to ensure specificity of transport. MAGI2 TOR1A LDLRAP1 ARRB2 TF SGIP1 SELE RAB21 CCL19 GH1 NLGN1 PLCG2 DAB2 CBL AHI1 WNT3A TBC1D5 VEGFA PPT1 SFRP4 GREM1 DRD2 ARRB1 SCYL2 HNRNPK WASL VTN CCL21 B2M HFE SYK ATAD1 APOA5 FMR1 BICD1 PICK1 SYNJ2BP NTF3 SERPINE1 HIP1 PCSK9 CD63 ANGPT1

GO_RESPONSE_TO_CAFFEINE Any process that results in a change in state or activity of a cell or an organism (in terms of movement, secretion, enzyme production, gene expression, etc.) as a result of a caffeine stimulus. Caffeine is an alkaloid found in numerous plant species, where it acts as a natural pesticide that paralyzes and kills certain insects feeding upon them. SEPN1 HDAC1 RYR1 SLC8A1 RYR2 DNMT3B PRKAA1 CAD RYR3 PPARGC1A DHODH GSTM2 PPARG HDAC2 IL6 CASQ2

GO_REGULATION_OF_ACTIN_FILAMENT_BASED_PROCESS Any process that modulates the frequency, rate or extent of any cellular process that depends upon or alters the actin cytoskeleton. TRIOBP ARFIP2 ROCK2 FSCN1 VILL PARK2 WIPF2 LRRC16A MYBPC3 GRB2 SPIRE1 SLIT2 GMFB PIK3R1 MYO1C OR2A4 CORO1B TMSB10 ACTR3C S1PR1 VASP ARPC1B CFL2 SYNPO2L CORO1A ARFIP1 INPP5K LMOD1 SFRP1 FER ASAP3 ATP1A1 TGFB3 MYLK2 RNF207 TEK PLEKHH2 TMOD4 NPHS1 RYR2 CDC42EP3 LIMA1 CLASP2 MYLK3 EDN1 DSG2 TAC1 RAC1 ACTR3 HAX1 SRI JMY ODZ1 MYOC ID1 PDE4D GPR65 ABL2 CNN2 MTOR WIPF3 WDR1 SWAP70 CDC42EP5 ARHGAP35 CCL24 ACTN2 CYFIP1 FCHSD1 WASH3P NF2 VIL1 TACSTD2 CIT PDE4B CCL11 PPM1E EPB49 ALOX15 SCN5A SORBS3 WASH6P SEMA5A SDC4 SH3BGRL3 SLC9A1 TMSB4X EPHA1 CAV3 SMAD4 BRAF DSTN PDGFA ARAP1 ARF1 GSN EPS8 ATP1A2 FCHSD2 ARHGDIA NTF3 PICK1 CCL26 WASH1 TWF2 CDC42EP4 BMP10 DAPK3 NEB PAM TMOD3 WNT11 ARHGEF10 TGFBR1 TGFB1 ITGB1BP1 SPTBN4 BST2 ICAM1 SUMO1 ARHGAP28 RAPGEF3 RASA1 RICTOR ARPC3 CTGF PFN4 PPM1F PDXP TGFB2 SIGLEC15 EPHA3 LATS1 SPTBN2 PPFIA1 WASF1 SYNPO2 LIMK1 GATA4 TMSB15A ARFGEF1 ARPC5 TMOD1 JUP TWF1 PYCARD PTGER4 FAM21C TMSB15B DSP CAV1 CCL21 WASL PHLDB2 RHOQ PFN1 STAP1 ARHGEF15 AKAP13 PDGFRB CTNNA3 RHOD FMN1 ADD1 PTK2B SSH1 GPM6B LPAR1 SPTA1 NCKAP1L PRKCE ARF6 SPTB SPTBN1 BAIAP2L2 CAPZA1 TMSB4Y SHANK1 ODAM PPP1R9A RHOA AP1AR ARPC1A HRG DLC1 SHANK3 ARPC5L ABI2 ABL1 CD2AP CLASP1 CCR7 CCDC88A RLTPR CDK5R1 F2RL1 SPTAN1 WIPF1 SCIN SPIRE2 PKP2 DLG1 HCK TSC1 ROCK1 CAPZB PAK3 PFN3 CDC42EP1 SSH3 FZD10 C13orf15 FHOD3 MYADM CAPZA3 STC1 WNT4 SPTBN5 FES MLST8 TMOD2 ILK F11R NCKAP1 JAM3 BCAS3 KIRREL PROX1 WASF2 CAPG FRMD7 ARPC4 NOS1AP ACTR3B PAK1 IQGAP2 CXCL12 ARPC2 ARHGAP6 ALMS1 WAS WHAMM TPM1 RDX MEF2C BBS4 ARHGEF19 TRPM4 ARHGDIB FHOD1 ARHGAP18 HRAS VANGL2 NTRK3 TNNC1 PHPT1 AVIL NCK2 MTPN KANK1 MYH9 C15orf62 C15orf38 ATP2A2 TMEFF2 PDGFRA CTSL2 CAMK2D TRIM27 MKKS PLN CELSR1 EVL CSF3 PLEK ACTR2 EPHA5 EZR CTTN FRMD6 S100A10 GRHL3 BAIAP2 BRK1 CDC42EP2 NCK1 BAG4 HCLS1 PREX1 PFN2 DIXDC1 APOA1 CAPZA2 LMOD2 SSH2 GJA5 DSC2 LRP1 CACNA1C EFNA5 TNNT2 LMOD3 SEMA3E SERPINF2 PRKCD SMAD3 BAIAP2L1 SYNPO NOX4 MAGEL2 CDK5 ARHGEF5 KCNJ2 ANK2 GMFG CRK ADD2 CSF1R

GO_MRNA_METABOLIC_PROCESS The chemical reactions and pathways involving mRNA, messenger RNA, which is responsible for carrying the coded genetic 'message', transcribed from DNA, to sites of protein assembly at the ribosomes. CPSF3 SNRPE RPL13A PRPF40B RBM23 KIAA1875 SNRNP70 POLR2J RPLP2 PRPF31 RPL5 FUS ZNF259 RPL15 DOM3Z SETX NOVA1 METTL3 PAIP1 PPIL1 RPL13 RPL24 HNRNPK SRSF4 AQR POLR2A LUC7L MTPAP DHX9 LSM10 EIF4E GRSF1 CLASRP SNRPN ERI1 FMR1 RPL35A CNOT6 RPL17 SRSF2 NANOS2 CSDC2 RNPS1 RPS14 SF3B4 SNRNP35 HTATSF1 RPS3A CSTF2 SLBP HNRNPA0 MAGOHB RPL38 LSM7 CNOT10 GTF2H3 TXNL4B RPL26 SART1 SLU7 PATL1 ARL6IP4 RBMX ECD BUD13 RBM41 PNRC2 KIAA1429 SPEN WDR77 PPP4R2 SRPK1 RNASE4 RPL21 RPL35 ZMAT5 RPS17 SNRNP27 WDR83 ZRSR2 SF3B14 NHP2L1 RPS27A ATM PHF5A PPIH SON RPL23 RPS10 HNRNPA3 DHX32 DHX35 C17orf85 HNRNPH1 PELO RPL27 RBPMS HNRNPL PTBP2 EIF4A2 FASTKD5 NSRP1 FIP1L1 RNASEL EXOSC9 DDX5 TSEN2 ZFP36 PNN RNGTT GTF2H1 RPS7 THOC4 SRSF10 APP SCAF1 TRA2A MRTO4 SNRPD2 TFIP11 PSIP1 AHCYL1 RBM28 FRG1 CDC73 ZMAT2 BCAS2 PRPF38A MBNL1 RPS15 SCAF11 UPF1 CCNH CPSF6 NUDT21 PLRG1 RBM20 CWC27 SAMD4B SMN2 ACIN1 RPL11 SUGP1 PPP1R8 CDK13 SRRT CDC40 RBM22 C1QBP ADAR CDK7 RPL32 WIBG RPS11 RPL10A LSM3 RPL9 RPS3 SNW1 RPL6 GTF2F2 RBMY1F ERN1 RPS9 EXOSC3 RPLP0 EIF2C1 EXOSC10 POLR2G PRPF19 WTAP ADARB2 APOBEC1 POLR2H MNAT1 PRPF6 CSTF3 LSM1 SRSF11 SUGP2 TSEN34 PRPF8 DHX34 CWC22 RPL18A U2AF2 SRSF7 EIF2C4 RPL29 ESRP1 RBM8A PQBP1 CD2BP2 SRSF3 FTSJD1 ZRANB2 CRNKL1 STRAP PRPF38B THOC7 AFF2 CNOT8 AKAP17A RPL28 RPL4 GEMIN8 WBP4 EIF3E PAPD4 KHDRBS2 PABPC1 TRUB1 RBM25 SMG9 SNRNP48 THOC6 EXOSC7 EXOSC6 PRPF3 CELF3 PAPOLG ELAVL4 RBMY1B DQX1 C2orf3 ROD1 SNRPA PPWD1 H2AFB3 LSM4 LOC100130932 RPS5 SNRPG RPL7A PARN DHX15 ZCRB1 SUPV3L1 USP4 HNRNPC HNRPLL NCBP1 ZHX2 RC3H1 DDX1 PCBP3 RBM27 CIR1 CPSF4 PTBP1 KIN RPL3L MOV10 PATL2 PAPD5 ETF1 CNOT7 DDX23 POLR2E GPKOW SSB POLDIP3 A1CF ELAVL1 PRPF39 RPL12 GTF2H2 RPL18 PPIL3 XRN2 RPS21 PAN2 RPL22 UPF3B CELF5 DDX39B ZNF326 GEMIN6 SRRM2 HNRNPA2B1 SARNP RBFOX3 PABPC3 SNUPN POLR2L KHDRBS1 RBM15B IWS1 DHX16 SRSF9 YTHDC1 HNRNPF SNRPA1 RNF113A HNRNPUL1 EFTUD2 CSTF1 NAA38 SFSWAP HNRNPR JMJD6 RPS16 SF3B2 RBMXL1 UBA52 MBNL3 NOVA2 DDX39A PDE12 GEMIN4 SMG1 DDX41 HSPA8 GPATCH1 EXOSC2 DDX20 POLR2I SF1 LSM6 HNRNPH3 U2AF1L4 HNRNPD AICDA SF3A2 GTF2H4 NONO SYNCRIP SLFN14 CSDE1 PRPF18 XAB2 POLR2F BRDT HNRNPA1L2 PSPC1 SNRNP40 PPP2R1A SNRPD3 SNRPB DDX46 PDCD11 LEO1 RBM17 SNRPB2 SREK1IP1 NUDT16 GEMIN7 PCBP1 RALY RPL3 PPIE TTF2 RPS28 SRSF12 UPF3A EIF2C2 DBR1 CNOT4 RPL31 DHX8 DCP1B RNF113B RPS12 GSPT1 APOBEC4 QKI EIF4A1 CPSF2 KIAA1967 TSEN15 CEBPG PPARGC1A PAN3 ZC3H12A HNRNPM CPSF7 RPS26 DDX47 MBNL2 RPS18 DHX38 DKC1 RPL34 SCAF8 EDC3 PUF60 RBMY1J RBFOX2 DCPS CWC15 USP49 AGGF1 ELAVL2 CLP1 H2AFB2 GEMIN2 RPL37 MAGOH SMG7 CNOT3 RPSA NBAS RBMY1C SKIV2L2 NCBP2 RPS19 ZCCHC6 PAPOLA RBM11 CNOT2 C16orf57 RPL19 HNRNPA1 RPS6 LSM11 FTSJD2 ZNF473 CCAR1 NCBP2L MLH1 RPS25 BUD31 METTL14 PRPF4 XRN1 DIS3L PDCD7 ERCC3 RPL27A APOBEC2 SF3B5 RBM10 CELF6 RPL39 EIF4G1 LUC7L3 DCP2 RPL36A PCF11 CELF4 SMN1 SMG5 LUC7L2 SMNDC1 RBFOX1 PRMT7 ALKBH5 DDX6 SRRM4 CELF2 TNKS1BP1 RAVER2 TSEN54 RBM39 SF3A3 RNMT SSU72 POP4 RPS13 RBBP6 EDC4 THOC1 SNRPF DNAJC8 RPL30 PPP2CA HELB CLNS1A SF3B1 CCRN4L SRRM1 SMG6 C19orf29 CTIF SRSF6 RBM4 GSPT2 GTF2H5 THOC5 PABPC1L CHTOP EXOSC4 RBM4B CDC5L ZCCHC11 HNRNPH2 RPL23A PNPT1 RPL8 CPSF1 PAF1 DGCR14 H2AFB1 RPL41 TXNL4A SNRPC LGALS3 WBP11 RPL36 PAPOLB UPF2 TRA2B CDK12 ZCCHC8 RSRC1 YBX1 EIF4B KHSRP RPS27 AKAP8L PRPF4B EXOSC5 AURKAIP1 SART3 ERCC2 PCBP2 FAM103A1 DIS3L2 PHRF1 RBM38 CPSF4L THOC3 SAMD4A RBMX2 ESRP2 SCNM1 SUPT6H CASC3 SAP18 EXOSC1 RPS24 SFPQ CNOT1 RBM15 SNRNP25 RNH1 GEMIN5 RPS15A POLR2D SRSF8 TARDBP RPL10 CNOT6L CSTF2T POLR2K NOL3 RQCD1 EIF2C3 RPLP1 UBL5 SRSF5 GTF2F1 CTNNBL1 RPL26L1 SREK1 RPL7 SYMPK SMG8 LSM5 SF3B3 THRAP3 C2orf29 RNPC3 ZC3H11A RPS23 DIS3 DCP1A RPS29 SNRPD1 PRKACA PRMT5 C20orf4 EIF4A3 RPL37A ISY1 ZFP36L2 PPP2R2A PTCD2 UHMK1 KHDRBS3 PRPF40A PABPN1 ZRSR1 CPEB1 RPS20 SYF2 TGS1 RPS8 RPS4Y1 ZC3H3 HNRNPU KDM1A POLR2C USP39 ERN2 RBM26 THOC2 RPS4X LSM2 NT5C3L RAVER1 SUPT5H RPL14 WDR33 ZFP36L1 SRSF1 SRPK2 EXOSC8 POLR2B RPS2 CELF1 RBM5 TUT1 ADARB1 SF3A1 SNRNP200

GO_RESPONSE_TO_COCAINE Any process that results in a change in state or activity of a cell or an organism (in terms of movement, secretion, enzyme production, gene expression, etc.) as a result of a cocaine stimulus. Cocaine is a crystalline alkaloid obtained from the leaves of the coca plant. HOMER1 DPYSL2 CRH MDM2 EFTUD2 HSP90AB1 DNMT3B HDAC5 DNMT3A SLC6A3 PTK2B OPRK1 CHRNB2 DNM2 HTR2A KALRN TIAM1 TACR3 DRD5 SLC6A1 SMPD1 DRD4 HSPD1 CRHBP SNCA OXT HOMER2 CCNA2 ABAT HDAC2 MBD1 HTR3A OXTR MTOR DRD1 CDK5 LPHN3 HTR1B CNR1 OPRM1 SDK1 HSP90AA1 DRD3 HNMT DRD2

GO_CELL_SUBSTRATE_ADHERENS_JUNCTION_ASSEMBLY The aggregation, arrangement, and bonding together of a set of components to form a cell-substrate adherens junction. TRIP6 ACTN1 THY1 ITGA2 RCC2 PTPRK RHOD DLC1 JUB WHAMM PDPK1 BCL2 ARHGAP6 LAMA5 FERMT2 CTTN TESK2 ARHGEF7 SORBS1 ACTN3 TAOK2 ACTN2 PTK2B PIP5K1A

GO_NEGATIVE_REGULATION_OF_PHOSPHOPROTEIN_PHOSPHATASE_ACTIVITY Any process that stops or reduces the activity of a phosphoprotein phosphatase. IKBKB PPP1R15A ROCK2 TNF TIPRL PPP4R4 ROCK1 PPP1R11 FKBP1A CRY2 PPP2R4 MASTL FKBP1B RGN NCKAP1L

GO_REGULATION_OF_SYNAPTIC_TRANSMISSION_GLUTAMATERGIC Any process that modulates the frequency, rate or extent of glutamatergic synaptic transmission, the process of communication from a neuron to another neuron across a synapse using the neurotransmitter glutamate. ADORA1 LRRK2 ATP1A2 SYT1 GRM6 NRXN1 GRIK3 NLGN3 OXTR ATAD1 TNR DRD1 UCN SHANK3 GLUL PTGS2 GRM4 HTR1B NTRK1 CNR1 GRM1 EGFR PLA2G6 DRD2 PSEN1 SERPINE2 GRIK2 GRM5 NLGN2 GRM2 OPHN1 IQSEC2 DGKI GRIK1 NPS MEF2C GRM8 RAB3GAP1 ADCYAP1 NPY2R GRM3 MAPK8IP2 NLGN1 PTK2B HTR2A KALRN RELN UNC13A DKK1 GRM7

GO_RIBONUCLEOPROTEIN_COMPLEX_BIOGENESIS A cellular process that results in the biosynthesis of constituent macromolecules, assembly, and arrangement of constituent parts of a complex containing RNA and proteins. Includes the biosynthesis of the constituent RNA and protein molecules, and those macromolecular modifications that are involved in synthesis or assembly of the ribonucleoprotein complex. RPL24 TARBP2 SNRPD3 SNRPB LOC255308 NSUN3 PDCD11 TAF9 EBNA1BP2 RPL13 SHQ1 RPL15 SETX WDR46 PPAN MRPL11 ERI1 RPS28 SRSF12 RPL3 EIF3A EIF5 EIF3G GTPBP10 GEMIN7 C1D LUC7L KIAA1875 SF3A2 RPL13A SNRPE TFB2M NIP7 DDX20 EIF3H BYSL LTV1 SF1 EIF2D SENP3 RRP7B RIOK2 LSM6 UTP11L TEX10 EXOSC2 MRPL1 RPL5 NOC2L XAB2 FCF1 RPLP2 PRPF31 NSUN5 RRP9 PRPF18 WDR36 DDX52 NUFIP1 EIF3CL MPHOSPH6 EDC3 RPL34 SLU7 SART1 AATF MPV17L2 MTERFD1 PATL1 NOL11 RPS17 RPSA CLP1 DDX28 C7orf60 GEMIN2 RPL37 RPL21 RPL35 GNL2 PA2G4 FTSJ3 UTP15 LSG1 NAF1 WDR77 METTL16 RRN3 RCL1 RPS3A NOL9 MRPS11 CIRH1A RPL35A EIF2C2 RPL17 RPL31 RPS12 RPS14 TXNL4B RPL26 DKC1 DDX3X EIF6 RPS18 RPS26 CCDC59 NOLC1 RPL38 DDX47 NHP2 THUMPD1 DCAF13 RSL24D1 RPS25 TBL3 NOP56 EXOSC9 ABT1 PNO1 RNASEL MRTO4 BICD1 RPL39 SRSF10 NGDN DIS3L LSM14A WDR43 RPL27A RPS7 RPL23 BRIX1 RPS10 WDR18 NHP2L1 RPS27A RPS19 NPM3 ZRSR2 GTPBP4 NLE1 WDR12 SKIV2L2 BOP1 RPL19 RPS6 NSUN5P2 TSC1 RPL27 CSNK1D PTBP2 NOL6 RRP8 CIRBP CDC40 CLNS1A MRPL20 DENR SF3B1 NAT10 CCRN4L CCDC86 RBM22 WDR3 RPL30 ERI2 SNRPF SMN2 RPL11 MRPS7 NOA1 EFTUD1 LSM3 RPL9 RPS3 UTP23 GTF2H5 WDR37 GAR1 UTP14A RPS11 SRSF6 RPL10A MDN1 IMP3 RPL32 ADAR RUVBL2 C1QBP LUC7L2 PRMT7 CELF4 SMN1 GNL3L FRG1 VCX RPLP0P6 EIF3F ISG20 PSIP1 LUC7L3 MRPS9 HEATR1 SNRPD2 RPL36A DHX37 SF3A3 RPS13 POP4 RUVBL1 DDX6 SCAF11 MRM1 RPS15 METTL15 RPL18A SNRPC NVL GTPBP5 UTP6 RPL41 PRPF8 TXNL4A C7orf36 NOM1 DDX27 NOP58 EIF3B PRPF6 TSR1 CRNKL1 DDX51 FBL ERAL1 EIF3K CD2BP2 GRB7 RPL36 EIF2C4 AAMP TRMT112 EIF4H RPL29 SETD4 CDC123 WBP11 PELP1 RPS9 WDR55 NOP14 EXOSC3 EXOSC4 RPP30 NOL8 ZNF658 RPL6 PWP2 RPP25 GNL1 NSUN5P1 PUM2 C14orf169 ZNHIT6 MRPL36 SRFBP1 DDX21 EIF3C PRPF19 RPL23A RNMTL1 DDX49 ISG20L2 RPL8 TSR2 RRP15 RPLP0 RRNAD1 EIF2C1 EXOSC10 NOC4L RIOK1 C2orf3 RPS24 DICER1 EXOSC1 EIF3I EXOSC7 PRPF3 EXOSC6 NUP88 SBDS WBSCR22 RPP38 NOL3 RPLP1 EIF2C3 RPL10 EIF2A RPL7A FTSJ2 MTERFD2 FBLL1 LAS1L POLR2D MINA NOP16 GEMIN5 LSM4 RPS15A RPS5 SNRPG WDR75 SUV39H1 EXOSC5 RPL28 EIF2S3 RPS27 EIF4B MPHOSPH10 PIH1D1 RPL10L STRAP RPP40 METTL15P1 DDX56 YBEY RPF1 MTG1 RBFA RRS1 UTP18 FDXACB1 DIS3L2 UTP3 SART3 GEMIN8 RPL4 C16orf42 EIF3E FAM103A1 SIRT1 LIMD1 C15orf42 PRMT5 XRN2 C20orf4 RPS27L RPS21 DDX31 BMS1 EIF4A3 MCTS1 KRR1 RPF2 MAK16 RPS29 RPL12 SNRPD1 RPL18 RPS23 DHX30 DIS3 EIF3M PRPF39 CSNK1E TGS1 NOP2 DDX39B GEMIN6 RPS20 ATXN2 RPL37A ISY1 C7orf30 RRP7A RRP1B RPL22 EIF3D ABCE1 IMP4 RPL26L1 WDR74 ORAOV1 RC3H1 DDX1 DIEXF PES1 ATXN2L SRSF5 PIH1D2 DYNC1H1 RPS10P5 USP4 OGFOD1 UTP20 RPL7L1 CNOT7 NOP10 DDX23 RRP36 PAPD5 DIMT1 NOB1 FASTKD2 RPL7 RPL3L RSL1D1 PATL2 SRSF1 RPS16 SRPK2 RAN TFB1M RPL14 XPO1 SDAD1 SF3A1 SNRNP200 GEMIN4 RIOK3 CELF1 RBM5 EXOSC8 MRPL10 UBA52 RPS2 EMG1 USP39 UTP14C RPP21 CHD7 GNL3 SNUPN RPS8 EIF3L RPS4Y1 RRP1 NMD3 SFSWAP EIF3J ERI3 DROSHA NSA2 NSUN4 ZNF622 RPS4X SRSF9 NPM1 SURF6 YTHDC1

GO_POSITIVE_REGULATION_OF_NIK_NF_KAPPAB_SIGNALING Any process that activates or increases the frequency, rate or extent of NIK/NF-kappaB signaling. NOD1 CARD14 TLR3 TNF MAP3K7 NOD2 ZFP91 TLR6 TRAF6 SASH1 TIRAP RC3H1 CHI3L1 MALT1 CARD10 EIF2C1 TNFRSF10A MAS1 RC3H2 EIF2AK2 TRAF2 IRAK1 ACTN4 TNFSF15 TNFRSF10B RPS3 EIF2C3 CALR HAVCR2 TRAF4 COPS8

GO_REGULATION_OF_ACTIN_FILAMENT_BUNDLE_ASSEMBLY Any process that modulates the frequency, rate or extent of the assembly of actin filament bundles. SDC4 SORBS3 LRRC16A BBS4 PARK2 RDX TPM1 PPM1E ALMS1 ROCK2 ARHGAP6 VIL1 TACSTD2 ARHGEF15 NF2 EPHA1 PIK3R1 LPAR1 SLC9A1 FHOD1 INPP5K DLC1 TMEFF2 SYNPO2L S1PR1 RHOA PPP1R9A SHANK1 ARAP1 BRAF PLEK CLASP1 EVL SHANK3 TGFB3 ASAP3 MKKS SFRP1 PFN3 CLASP2 ROCK1 TSC1 ITGB1BP1 TGFBR1 S100A10 ARHGEF10 WNT11 PPM1F C13orf15 APOA1 PFN2 CTGF RAC1 BAG4 TAC1 ARHGAP28 RAPGEF3 SERPINF2 LIMK1 SYNPO2 PPFIA1 ID1 MYOC WNT4 PFN1 PAK1 PHLDB2 SWAP70 ARHGEF5 FRMD7 SYNPO NOX4 SMAD3 WASF2 MTOR PTGER4 GPR65

GO_REGULATION_OF_GENE_SILENCING Any process that modulates the rate, frequency, or extent of gene silencing, the transcriptional or post-transcriptional process carried out at the cellular level that results in long-term gene inactivation. ASF1A MAP2K1 ADAR TET1 ATAD2B HIST1H3F JUB HIST1H3G HIST1H3C HIST1H3I WTIP FMR1 NCOR2 ELAVL1 HIST1H3E STAT3 DND1 DNMT1 PHF8 ATAD2 HIST1H3H TRIM28 EGFR NCOR1 BCDIN3D ZFP36 PHF2 UHRF2 LIN28A TGFB1 ESR1 SIRT1 LIMD1 ZC3H12A HIST1H3J TERT SIN3A HMGA1 HIST1H3A MIER1 APOBEC1 FXR1 H2AFY CDC45 PUM1 POU5F1 PUM2 HIST1H3B MAP2K2 CDK2 ZNF304 AICDA TRIM71 HIST1H3D UHRF1 XPO5 SETDB1

GO_REGULATION_OF_HEART_GROWTH Any process that modulates the rate or extent of heart growth. Heart growth is the increase in size or mass of the heart. CDK1 GSK3A ERBB4 FGF9 MEF2C FGFR1 TGFBR1 ACACB SAV1 TP73 CCNB1 HEY2 FGFR2 CAV3 MAPK11 ZFPM2 PTEN NOG PIN1 EDN1 TBX20 HAMP MYH6 TBX5 WT1 DDX39B GJA1 BASP1 NOTCH1 CTDP1 DUSP6 NKX2-5 AKAP6 KCNK2 G6PD FGF20 RBP4 TGFBR2 TBX2 BMP10 TGFBR3 GATA6 IGF1 WNT2 MAPK14 MTOR RBPJ FGF2 PROX1 BMPR1A

GO_REGULATION_OF_CAMP_DEPENDENT_PROTEIN_KINASE_ACTIVITY Any process that modulates the frequency, rate or extent of cAMP-dependent protein kinase activity. PRKAR2A PKIG SIRT1 NPFFR2 MST1 PRKAR1B ADIPOQ PKIA PRKAR2B RAPGEF2 PKIB SESN2 ATP2B4 PRKAR1A

GO_POSITIVE_REGULATION_OF_GROWTH Any process that activates or increases the rate or extent of growth, the increase in size or mass of all or part of an organism. IL2 LEF1 HYAL1 TRPC5 SERP1 WT1 TBX5 MACF1 ITSN2 GHRH IL9R AVP SYT1 CELF1 NOTCH1 ZP3 CSF1 ISLR2 GATA6 IGF1 TBX2 GPAM RAG2 GPR21 TGFBR3 SFRP1 RIMS2 BMPR1A SFRP2 FGF2 PRKDC TRPV2 PARK2 ERBB4 FGF9 WFS1 IL9 FGFR1 ADNP MAP2K5 SDCBP BCL2 CXCL16 NTN1 RASAL1 TFCP2L1 ADAM10 GHR CPNE6 AGRN NEDD4L CHD7 RUFY3 SOX15 PIN1 PTK2B PAFAH1B1 HAMP TBX20 S100A8 SLC44A4 FXN HIST1H1B ARX NRG1 SYT3 DDX39B ADNP2 YAP1 HOPX FGFR1OP ALOX12 TGFBR2 ILK RPS6KA3 GHSR ZNF639 RIMS1 CSNK2A1 ACTN3 DRD2 UTS2R SRF PPARD MTOR PROX1 UCN WNT2 EXOSC9 HBEGF MMP14 TAF9B CRABP2 EXTL3 RPS6KA1 MUL1 LPAR3 PPP1R1C CDKN2AIP PRSS2 UNC13A NDEL1 GOLGA4 CD38 FN1 GH1 EDN1 SLC25A33 SASH3 SYT17 NCBP1 SMURF1 FOXS1 SUPV3L1 RAB11A F2 HLX OSBP TWF2 AVPR1A IST1 S100A9 INS EGFR BMP10 MAPT H3F3A MAPK14 ADAM17 MKKS NIPBL CACNA2D2 MEF2C DDX3X DISC1 WNT3A GSK3B SEMA5A HDGFRP2 L1CAM BBS4 TRIM32 VIL1 AKT1 DERL2 CXCL12 CPNE9 CDKL5 ACACB VEGFA SYT4 BMPR2 CSDA HEY2 FGFR2 PPIB BDNF KRT17 HSF1 WNT3 STAT5B DIO3 MTPN KIAA1598 IGFBP1 SLC9A1 KDM2B INO80 MTM1 N6AMT1 NTRK3 CREB1 LIMK1 RND2 AGR2 BASP1 PLCB1 DSCAM SFN MEGF8 POU3F2 MYOD1 SPHK1 EFNA5 CIB1 USP47 DLL1 AKAP6 CPNE5 GHRL CAPN3 BBS2 RPS6KB1 TAF9 H3F3B RBPJ PLS1 LEP IL7 NRP1 CDC42 GHRHR SYT2 ATP8A2 C6orf108 FGF8 MAP1B SYT14L SPTBN4 SEMA7A CDK1 ZNF259 TNFRSF12A LGI1 CCNB1 TGFBR1 ANAPC2 POU1F1 EZR ZFPM2 ERBB2 ZFYVE27 SEMA4D HPN EXOSC4 TGFB2 PPM1F CDH4 INSR SLC6A3 PSMD10 SMO CYBA EXOSC2 NOL8 H3F3C

GO_CELLULAR_COMPONENT_MAINTENANCE The organization process that preserves a cellular component in a stable functional or structural state. RAB3A KIFC3 PARD6A CTTN NLGN2 ITGA3 PKP2 CAMSAP3 PLEKHA7 MTHFR RASSF8 SUPV3L1 PICK1 MTMR2 BAI3 C1QL1 ITPKA MYOCD MYADM TANC1 CSF1R

GO_NEGATIVE_REGULATION_OF_PROTEOLYSIS_INVOLVED_IN_CELLULAR_PROTEIN_CATABOLIC_PROCESS Any process that stops, prevents or reduces the frequency, rate or extent of proteolysis involved in cellular protein catabolic process. PDCL3 SHH PBK HFE BAG5 USP19 LAMP3 TAF9 TRIM39 N4BP1 TLK2 PSEN1 BAG6 GABARAPL2 SMARCC1 UBXN1 PRKCG GIPC1 CLEC16A MTM1 SCO1 FHIT KIAA1967 KBTBD5 WNT1 USP14 OPHN1 SVIP WAC SUFU HSP90AB1 SENP1 SDCBP PML PSMF1 PARK7

GO_PROTEIN_TARGETING_TO_LYSOSOME The process of directing proteins towards the lysosome using signals contained within the protein. SORL1 ZFYVE16 RAB7A HGS GCC2 HSPA8 AP3B1 SNX16 AP3M1 GNPTAB NAGPA GNPTG NCOA4 NEDD4 WASH3P SCARB2

GO_POSITIVE_REGULATION_OF_DNA_REPLICATION Any process that activates or increases the frequency, rate or extent of DNA replication. PDGFB IL6 NPM2 PDGFA ATRX EREG CALR EPO GLI1 GLI2 E2F8 PDGFRA TGFB3 DBF4B UCN CDC42 OBFC1 CACYBP CSF2 EGFR INS FGF10 CTC1 IGF1 JUN WIZ PLA2G1B KITLG MAP2K4 STOML2 MAS1 NPM1 AREG CDK1 CDC7 ATF1 E2F7 IGF1R PDGFC RAC1 PCNA BMP5 HRAS INO80 KCTD13 SHC1 DNA2 CDK2 BMP4 TNFAIP1 INSR BMP6 CST3 IL3

GO_GAMETE_GENERATION The generation and maintenance of gametes in a multicellular organism. A gamete is a haploid reproductive cell. SLC9A10 DAZ4 TSNAXIP1 UBE2J1 CASC5 DPY19L2P2 ADAMTS2 SPATA24 LGR4 CCIN MERTK DPY19L2 PLEKHA1 PIWIL2 BRDT NOBOX HIST1H1T TARBP2 MEI1 FSTL3 ACRBP FAM75C2 SOD1 MAEL ODF4 IFT81 TDRD7 MAS1 PAIP2 C15orf2 CDK16 FIGLA SPAG6 TESK1 DDX20 B4GALNT1 C19orf20 SPIN1 LRGUK PGM3 DDX4 RPL39L SOS1 ATRX C9orf79 TYRO3 TMEM203 PIWIL1 BCL2L1 ZSCAN2 FAM75A1 TRIM27 TSPY10 GPR64 RBMY1J CNR1 PYGO2 CCNYL1 DIAPH3 INSL3 POMZP3 GGT1 ATP1A4 AGFG1 ANKRD49 SIAH1 SPA17 FANCD2 MORN2 POU4F2 QKI TSSK1B TSGA10 SCMH1 JAM3 ADAMTS1 GGN CCNI SLC22A16 SPATA9 OAZ3 RNASE9 CDY2A MLH1 FAM75A5 ASF1B SPATA16 PVRL3 FNDC3A ETV6 ELL3 TTC26 RPS6 TSPY3 TCP11 AR FOXL2 ABHD2 SLC26A8 CAPZA3 RHOXF1 NR6A1 TNFAIP6 HIST1H2BA HERC2 TRIP13 TEX15 FMN2 BCL2L11 GTSF1 RHBDD1 ACOX1 C12orf55 GAMT BIRC3 BRD2 LGR5 NEURL DDX6 SOX8 SPIN4 KRT9 PRM3 RNF17 RPS6KA2 SPEM1 PTK2B TBP PAFAH1B1 NOS3 SOX9 ATP2B4 PATZ1 ROS1 ALKBH5 PRMT7 SLC26A3 MLL2 CELF4 BAG6 FSCN3 ZNF35 MAST2 TP63 HMGB2 BPY2B H3F3B FOSL1 KIT BBS2 CATSPER2 CCNB1 AMH TXNRD3 IMMP2L AFP DEFB118 TXNDC8 SBF1 SPIN2B PRSS21 SYCP3 DAZ3 PABPC1L TNP2 C20orf165 NME5 TSSK3 DNMT3L DAZ1 DHH CCDC63 RNF8 PCSK4 PACRG PVRL2 KDM1B SLCO4C1 CCT6B TDRD12 GDF9 CCR6 CATSPERB SRC TSSK2 NKAPL ADC IGF2R H3F3A CDY1 KDM5A BSPH1 FAM75C1 C17orf46 THEG SSTR2 IL4R GOLGA3 ADCY10 PIWIL3 RPL10L PSME4 KDM2B RGS2 WNT3 HSF1 FANCF OSBP2 DYNLL1 PPAP2B FOXJ1 SSTR1 KHDRBS3 MTL5 ITGB1 TDRD1 TSSK4 H1FNT PRM2 STRA8 GGNBP2 FAM75D4 WIPF3 PRKACA RARA GGNBP1 TSPY1 CTNNB1 MAK ZGLP1 TDRKH TBC1D20 TMEM48 PDE3A SPATA20 CDC25B OVOL1 CYLC2 USP9X RAD21L1 KIAA1524 ETV5 OCA2 FAM75A3 PIAS1 RAD23B RNF2 SOX30 CELF1 MSH2 GOPC BCAP31 CTDNEP1 EREG PAQR8 SLC4A2 ODF2 WDR33 CDY1B APOB H2AFX ARID4A ACSBG2 RSPH1 DDX25 CREB3L4 YY1 MSH4 SPO11 PRSS42 FOXC1 SIX5 MCM9 TMF1 CTCFL BCL6 SPANXB1 DEAF1 PRKACG SPEF2 NUP62 CATSPER1 C20orf152 RNF114 DPY19L2P1 MYCBPAP RNF151 PTGS2 DPCD LEP SPIN3 MLL4 SHCBP1L SETX CATSPER4 DMRTC2 HILS1 TXNDC3 PRDM14 ADCYAP1R1 TSSK6 AFF4 TXNDC2 CLOCK SLC2A14 SGPL1 PROK2 INHBB ADAD1 HSF2 SEPT4 FAM9C PAQR7 ING2 PANK2 PAX5 RXFP2 TBPL1 TDRD9 CABYR CTSL2 PTCHD3 CALR SRPK1 RGN TEX19 MKKS CEP57 STYX E2F1 HOXA11 ZNF830 CSDA TSPY8 SMAD1 EIF5A2 BBS4 MICALCL STK11 MOV10L1 SLC26A6 AXL CYP26B1 MEIG1 NANOS2 ACE FAM9A BCL2L2 FAM75D1 PDILT C14orf133 SPIN2A WNT4 TNP1 GLI1 SPATA5 SYCE3 MYCBP IQCF1 TMEM119 HEXB AZI1 IQCG HOXA10 DAZAP1 MEA1 LIMK2 ADAM28 SLIRP GPR149 SPATA2 ZMYND15 FSHR KLHL10 ZPBP2 SERPINA5 INSL6 FAM75B CATSPERG REC8 SOX17 CDYL FKBP6 JAG2 ATM NPM2 ZP3 FAM75A6 CDC25C BPY2 ZNF148 PCYT1B SPAG8 WT1 TSPY4 PGAM2 TOB2 C11orf20 ODF3 CFTR DZIP1 NRIP1 NSUN2 PRM1 DND1 HOXA9 GMCL1P1 NLRP14 PAQR5 NANOS3 RUVBL1 CLDN11 TSNAX UBR2 BIK C9orf24 SOHLH1 HERC4 PPAP2A VCX CCDC155 KIFC1 CCNB1IP1 CREM CETN2 GAL3ST1 ACVR1 FSHB SPATA22 CIB1 PAFAH1B3 ZNF296 BMPR1B CRTAP CABS1 KIAA0196 FANCL C8orf42 BAX YBX2 PLA2G3 SSTR3 TAF7L ADAM18 ADAM29 RPS6KB1 NPHP1 SUN5 DAZL PTTG1 ASPM TGFBR1 TGFB1 HOOK1 WDR81 ERCC1 PRDX4 RACGAP1 RAD51C GJA10 UBE2B ANGPT2 TIAL1 ZBTB16 GMCL1 ZMIZ1 RBMY1F PRKAG1 HORMAD1 WDR48 SFMBT1 PICK1 PARN FAM9B CELF3 ANG DEDD FOXA3 BOLL TUBD1 SPANXA1 CALR3 CYLC1 RBMY1B SMARCA2 BMP15 FABP9 GALNTL5 SPAG9 MCM8 AKT1 SIRT1 BCL2L10 SPAG4 FAM75A7 PMCH CATSPER3 TCFL5 C2orf65 AURKC ACVR2A FOXO3 BPY2C DUSP13 C17orf104 KIAA0430 DEFB1 BAD DNMT3A SMAD4 PHC2 DMRT1 CHD5 PYGO1 FAM50A SPAG16 HIST1H1A ARNTL NR2C2 HSF2BP PARP11 MTOR OR7C1 XRN2 MNS1 ARID4B CCNA1 ODF1 CDY2B SMAD5 ROPN1B SPACA1 NR0B1 DLD TTLL5 EIF2S2 PRDM1 ZFP41 PUM1 DNM2 SPATA19 STRBP SYCP1 CNTD1 BMP4 WFDC2 INPP5B FANCG DNAJA1 NOTCH1 KDM3A PAFAH1B2 TAF4B ADIG NUP210L MORC1 SKIL CADM1 BTG1 BRCA2 HPGD DIAPH2 TMEM146 RFX2 KLF17 SPATA6 TESK2 LIN28A FAM75D3 DNAH9 C10orf27 BCL2 ATAT1 MMP19 DMC1 SPANXA2 TDRD6 NDRG3 ZNF541 PLD6 USP42 FBXO5 USP9Y TSPY2 ASZ1 SOHLH2 HSPA2 CXADR HERPUD2 TBC1D21 PIWIL4 TDRD5 UTP14C

GO_REGULATION_OF_HEART_CONTRACTION Any process that modulates the frequency, rate or extent of heart contraction. Heart contraction is the process in which the heart decreases in volume in a characteristic way to propel blood through the body. ADA CACNA1H FXYD2 SCN5A KCNG2 TPM1 SLC8A1 ATP1A4 FKBP1B DMPK TRPM4 GSK3A CALM2 APLN CASQ1 ZC3H12A STIM1 FXYD3 CACNA1G PDE4B THRA CACNG4 CACNA1D NPR1 TRPC1 HEY2 RGS2 CAV3 ASPH SLC9A1 EDN3 NOS1 KCNK6 CALM1 SCN4B OXT ATP1B2 EDN2 KCNA5 GSTM2 CORIN ATP1A2 ATP2A2 ADRA1A RANGRF HSP90AA1 BVES PDE5A YWHAE AVPR1A BMP10 ADRA1B CACNA2D3 KCNE1 CAMK2D MYL4 KCNIP1 RYR1 ATP1B1 PLN FXYD7 CACNA2D2 GNAO1 SPTBN4 HSPB7 FXYD6 KCNH2 KCNE2 CACNG5 CSRP3 HDAC4 CTGF TACR3 TGFB2 GJA5 DSC2 S100A1 ATP2B2 KCNK3 NPR2 ITPR2 KCNJ4 KCNE1L GJC1 SUMO1 CACNG2 P2RX4 GATA4 SCN10A CACNA1F ATP1A3 CACNA2D4 MYH6 CACNB2 CACNA1C NPPC ADORA1 MC3R NKX2-5 TNNT2 CAV1 KCNJ2 ANK2 KCND1 MEF2A JUP HCN4 CACNG6 GLRX3 DSP SLC1A1 JAK2 CACNG8 TNNI3 ITPR3 PIK3CG TMEM65 EHD3 FOXN4 KCNIP2 CELF2 CTNNA3 THRB KCNK1 KCNJ12 IRX5 CXADR NOS3 ATP2B4 HRC ATP2B1 ADRBK1 DMD AGTR2 AHCYL1 UTS2 GAA IL2 ATP2A3 CACNA2D1 ATP2A1 TH CHRNA7 KCNIP4 FXYD1 CACNB4 FXYD4 CALCA ATP2B3 SCN2B MYL3 CACNB1 NUP155 KCNQ1 RNF207 ATP1B3 KCNJ11 TBX2 CACNG1 AGT SLC8A3 NPPA ATP1A1 GPD1L ACE2 CACNA1B POPDC2 RYR2 IFNG SEMA3A ITPR1 CACNA1S CACNG3 CYP2J2 GSTO1 SCN1B RYR3 KCND3 MYH7 PKP2 CASQ2 TRDN SCN3B MDM2 GLP1R STC1 CHGA SRI MYL2 TAC1 ADM EDN1 DSG2 KCND2 C12orf39 ADRB1 NPFF CACNB3 KCNJ14 GJA1 PDE4D HOPX ABCC9 CACNG7 KCNK15 KCNIP3 AKAP9 NOS1AP SNTA1 DRD2 DES CALM3 CHRM2 EPAS1 SLC8A2 CLIC2 SREBF1 SMAD7 UCN TNNI3K HBEGF PRKACA

GO_COFACTOR_BIOSYNTHETIC_PROCESS The chemical reactions and pathways resulting in the formation of a cofactor, a substance that is required for the activity of an enzyme or other protein. ATPIF1 ACSL3 ACOT7 NADKD1 ALAS1 MMADHC PNPO NMNAT2 ACACA MOCS1 TPK1 ISCU THEM4 QDPR NAMPT COQ5 PDSS1 TECR PPOX AGK ELOVL7 PDHX NMNAT3 NADSYN1 C9orf95 CPOX ACSL5 TMEM14C KMO ELOVL5 ACACB ALAS2 BRP44 ITGB1BP3 MTHFS LOC344967 FECH GPHN HAAO SLC25A16 COQ3 HSD17B12 ADCK3 GART ACSL1 NARFL ACOT8 GCH1 FPGS NFS1 FASN DHFRL1 IBA57 MAT1A KYNU NUBP1 BDH2 MLYCD SLC25A1 ACOT12 ACSF3 SCD5 MOCOS CIAO1 PANK4 COX10 ELOVL6 MTHFD1L HSCB COQ4 COASY DLAT NFE2L1 PANK3 FLAD1 ACSS2 ACOT4 PDHA1 COX15 PCBD1 UBIAD1 MOCS3 ELOVL3 ACOT6 PDSS2 NADK PDHA2 ALAD NUBP2 MTHFD2L NDOR1 ATIC MTHFD1 QPRT PANK2 DHFRP1 DCAKD DHFR ASPDH UROD ACAT1 RSAD1 SPR PANK1 ACSL4 ACSBG1 PTPLB PDXK SCD ACSBG2 TSPO GCDH PPT2 PDHB MOCS2 ACSL6 FAM96B ACLY ABCB6 MTHFD2 PPT1 AFMID UROS ACOT13 ACSF2 LIAS BRP44L FXN SPTA1 NMNAT1 ACOT1 MAT2B ISCA1 ISCA2 NDUFA9 ELOVL4 SUCLA2 HMBS ACSS1 PPCS MMS19 MAT2A SLC25A39 PTS FAM96A LIPT2 PTPLA ELOVL2 ELOVL1 THEM5 ACOT9 ME1 DLD PCBD2 COQ9 RFK ACOT11 PNP CIAPIN1 PPCDC NFU1 COQ7 COQ6 NAPRT1 COQ2 SLC11A2 SLC25A38 ACOT2

GO_PARASYMPATHETIC_NERVOUS_SYSTEM_DEVELOPMENT The process whose specific outcome is the progression of the parasympathetic nervous system over time, from its formation to the mature structure. The parasympathetic nervous system is one of the two divisions of the vertebrate autonomic nervous system. Parasympathetic nerves emerge cranially as pre ganglionic fibers from oculomotor, facial, glossopharyngeal and vagus and from the sacral region of the spinal cord. Most neurons are cholinergic and responses are mediated by muscarinic receptors. The parasympathetic system innervates, for example: salivary glands, thoracic and abdominal viscera, bladder and genitalia. NRP1 HOXB1 PHOX2A HES1 NAV2 HOXB2 PLXNA3 PHOX2B TFAP2A GDNF SEMA3F SEMA3A EGR2 SIX1 TBX1 PLXNA4 HES3

GO_DOPAMINE_METABOLIC_PROCESS The chemical reactions and pathways involving dopamine, a catecholamine neurotransmitter and a metabolic precursor of noradrenaline and adrenaline. AGTR2 DRD1 HPRT1 SNCB SLC6A3 MAOA NR4A2 TGFB2 COMT DRD4 DRD2 DRD3 GRIN2A NPR1 GPR37 ATP7A DDC TH MAOB SNCA GCH1 DAO DBH PARK2 SNCAIP

GO_PHOSPHATE_ION_TRANSPORT The directed movement of phosphate into, out of or within a cell, or between cells, by means of some agent such as a transporter or pore. SLC25A3 SLC17A7 SLC34A2 IP6K2 SLC34A3 SLC20A2 FGFR1 SLC37A4 ADAMTS8 SLC20A1 SLC37A1 ANKH SLC37A2 SLC37A3 SLC17A1 SLC17A3 SLC17A4 SLC34A1 XPR1 SLC17A2

GO_ESTROGEN_BIOSYNTHETIC_PROCESS The chemical reactions and pathways resulting in the formation of estrogens, C18 steroid hormones that can stimulate the development of female sexual characteristics. Also found in plants. AKR1B15 RDH8 HSD17B8 HSD17B1 HSD3B1 HSD17B11 HSD17B7 HSD17B12 CYP19A1 HSD17B14 STAR

GO_REGULATION_OF_SUPEROXIDE_METABOLIC_PROCESS Any process that modulates the rate, frequency, or extent of superoxide metabolism, the chemical reactions and pathways involving superoxide, the superoxide anion O2- (superoxide free radical), or any compound containing this species. FBLN5 AATF SYK PON3 AGT CYBA TNF SOD1 CRP SZT2 DHFRP1 GSTP1 F2RL1 DHFR EGFR GCH1 TGFB1 BMP7 PARK7 NFE2L2 CD36 RGN ACP5 PRKCD

GO_GLUTAMATE_SECRETION The controlled release of glutamate by a cell. The glutamate is the most abundant excitatory neurotransmitter in the nervous system. SLC1A2 STXBP1 GRM2 SYT1 PPFIA1 PPFIA3 APBA1 TRPV1 RAB3A UNC13B NTRK2 VAMP2 SLC17A7 NF1 BZRAP1 PPFIA2 STX1A SLC1A1 SLC1A7 GLS2 GIPC1 SLC1A3 PPFIA4 SLC38A2 SNAP25 CPLX1 SLC1A6 GLS RIMS1

GO_NEGATIVE_REGULATION_OF_GLIAL_CELL_PROLIFERATION Any process that stops or decreases the rate or extent of glial cell proliferation. PTN HES1 TSPO ASCL2 SKI DICER1 ADCYAP1 TERT RNF10 CERS2 NOTCH1 SOX11

GO_MYELOID_DENDRITIC_CELL_DIFFERENTIATION The process in which a monocyte acquires the specialized features of a dendritic cell, an immunocompetent cell of the lymphoid and hemopoietic systems and skin. UBD SPI1 TGFBR2 BATF CD86 TGFB1 TRAF6 CAMK4 IRF4 DHRS2 RELB IL4 PSEN1 CSF2 BATF3 LTBR TM7SF4 RBPJ BATF2

GO_EPIBOLY The expansion of one cell sheet over other cells or yolk. COL5A1 HBEGF ADAM17 DDR1 CYR61 ARHGAP35 LCP1 ITGA5 RLTPR MSX2 TOR1A C11orf34 CEACAM1 JUB RHOA RHOC TMEFF2 WNT7A FLNA ACVRL1 MEGF8 PDCD10 MMP12

GO_REGULATION_OF_INTRACELLULAR_PROTEIN_TRANSPORT Any process that modulates the frequency, rate or extent of the directed movement of proteins within cells. BCAS3 FLNA TMEM30A IFI27 OAZ1 OS9 LRRC46 PYDC2 PSMD8 FZD5 GAS6 GLS2 HTRA2 SIAH3 CHP RBCK1 KIAA0528 LACRT LGALS9 GREM1 UBL4B TRIM28 TMEM110 OGG1 MXI1 AZI1 RNASE2 TPR DTWD2 FAM115C HHAT MYBPC1 SPPL3 RBPMS FZD1 TP53 BMP6 MAVS SP100 POLR3A RGS20 SULF2 PRR5L CCL19 TM9SF4 KIF5B SH3GLB1 LITAF DACT1 IL23A RAC2 RAB23 CSNK2A2 RHOA ECT2 CDKL2 CDK5R1 RBM22 ZDHHC8 HYAL2 TNF SNIP1 ERLEC1 BMPR1A SFRP2 DPCR1 DAB2 TLR7 APOD PLK3 JAK2 TLR2 IL1B RUVBL1 KIAA1199 TXN SMCR7 PEMT NKAP GDI1 RANBP3L HPS4 SLMO1 ZBED3 XPO5 SFRP5 AKT2 PKD1 RHOU MBTPS1 UBE2D3 PTPN14 PRKCD EPT1 ABLIM3 NUCB1 DDX58 UBR5 NUP62 UBE2J1 PDE2A SPHK1 APC2 AXIN1 LRRK2 SREBF2 DYRK2 TNFSF14 ANP32B KCNB1 PARP10 PTGS2 CDK5 C10orf129 PINK1 WDR46 SMAD3 LEP ZNF259 RAB7L1 PDZK1 VAMP2 CDK1 PCNT ZBTB17 MED1 PDCD5 PARL CCDC22 ERBB2 CTSK UGCG SIRT4 ARIH2 HCLS1 BAG4 UBE2J2 UBAC2 PSMD10 GTSE1 EDEM1 GLI3 PPP3R1 EMD KAT2A PCM1 CSF3 SEC16B OSTBETA KCNE1 GSK3A STOM GSK3B TCF7L2 MAPK7 USP17L2 CDH1 ATG13 FBXW11 PARK7 THRA VPS11 PPP3CB NLRP12 BRSK2 ZC3H12A DERL2 C8orf4 KANK1 SMCR7L OR13C4 PLAGL2 IL28B VAMP7 DERL3 CYB5R1 IL18 NRG1 UHMK1 FAM20B PIK3R2 IL6 ODZ1 NFKBIL1 EGF UBE2L3 IL10 TOMM7 PRKACA PSMB7 LEPROT SREBF1 PTPN11 CD27 TLR3 MTOR CDKN1A OSCP1 TONSL CTDSPL2 HPCA MDM2 NOV SVIP PKIG HAX1 RANGAP1 NDEL1 UBE2G2 CCL2 NMUR1 BMP4 SRI CAMK1 MFF TLR9 CABP1 FAM115A NUP93 WWTR1 HSPA1L XPO1 INPP5K SLC17A9 CCDC165 HUWE1 FBXW7 IL9R BCAP31 PRDX1 PPP3CA HNMT RGS19 BNIP3L IGF1 CHRM1 IL18R1 CHERP LCP1 BAP1 HES1 NPEPPS TGFB3 ATPBD4 RNF31 SOX4 HSP90AB1 CYLD PARK2 ERBB4 ADIPOQ FIS1 GNAQ MYO1C PIK3R1 BCL3 RUFY3 NFKBIE TLR4 AGTR2 HDAC3 SFN NBPF3 USP36 B3GAT3 AKAP6 NUPL1 ANK3 CIB1 WBP11 IL12B IPO5 KIAA0947 DMAP1 SLC9A3R1 U2AF2 NFKBIA PTPN1 YOD1 CDC42 JUP ELMOD1 ATP13A2 NLRP3 DAB2IP AXIN2 PPM1A ITGB1BP1 TMEM30B NUP54 BAMBI TGFB1 TGFBR1 SNAI2 GPHA2 C1QTNF3 MICALL2 CEP290 SH3TC2 PKIA NUTF2 ZIC1 NODAL RAPGEF3 SMO UBL5 RANBP3 NOL3 EDAR NF1 EDEM2 ITGAX MDFIC TRIP6 MDFI PKD2 BMP7 TDRD12 ANGPT1 OAZ2 SORL1 DNAJC27 ATPIF1 SAE1 KCNN3 EGFR TSGA13 GPR26 HSP90AA1 MIS18A PARP1 SHH MRGPRE PBLD FAF1 MAPK14 SUFU DACT3 GCC2 PPM1B SEMA5A EPB49 CHP2 WNT3A CD36 FZD7 FAM89B G3BP2 SIRT1 BARD1 RPL28 SMAD4 KEAP1 XPO4 WDR75 ACTL6A TBC1D10C SLC35D3 TMEM173 EDA KIF20B STARD7 SLC9A1

GO_REGULATION_OF_CATION_CHANNEL_ACTIVITY Any process that modulates the frequency, rate or extent of cation channel activity. MYO5A JPH4 NOS1 DMD KCNAB1 SEPN1 DRD4 HRC JSRP1 HOMER1 STIM1 CRHR1 NTSR1 CALM2 KCNIP2 PDE4B KCNS2 AHNAK FKBP1A EHD3 STOM FKBP1B NPPA CAMK2D KCNE1 PLN CACNB1 GNB5 FGF14 PLA2G6 KCNJ11 RNF207 EFCAB4B KCNRG PIRT CACNB4 STIM2 JPH3 GSTM2 KCNG1 PKD2 CALM1 FGF12 CACNA2D1 SUMO1 PLCG2 KCNE1L KIF5B HTT S100A1 SRI KCNE2 GAL TRDN CASQ2 JPH1 GSTO1 VAMP2 OPRL1 CLIC2 STAC PRKACA C19orf26 TMEM110 ANK2 CAV1 KCNE3 NOS1AP ACTN2 CTSS DRD2 CALM3 GALR2 KCNS1 JPH2 AKAP6 ANK3 HAP1 MMP9 CACNB3 UBQLN1 FMR1 EPO CACNB2 PDE4D

GO_PROTEIN_TRANSPORT_ALONG_MICROTUBULE The directed movement of a protein along a microtubule, mediated by motor proteins. RPGR IFT27 TRAF3IP1 ARL3 PCM1 IFT122 WDR19 ICK IFT140 SSX2IP BBS12 AZI1 DYNC2H1 TTC21A TTC26 IFT74 IFT57 HSPB11 SSNA1 IFT43 WDR35 TTC30B TTC21B IFT20 IFT81 TTC30A IFT46

GO_REGULATION_OF_PROTEIN_LOCALIZATION_TO_CHROMOSOME_TELOMERIC_REGION Any process that modulates the frequency, rate or extent of protein localization to chromosome, telomeric region. CCT8 GNL3 TERF1 CCT7 TCP1 CCT4 CCT5 DKC1 CCT6A WRAP53 CCT2 H2AFY GNL3L

GO_POST_GOLGI_VESICLE_MEDIATED_TRANSPORT The directed movement of substances from the Golgi to other parts of the cell, including organelles and the plasma membrane, mediated by small transport vesicles. MYO5A TGOLN2 DNM2 VAMP7 KIF16B CCDC22 OSBPL5 VTI1B VPS13A COMMD1 AP4M1 C16orf62 STXBP6 SCAMP2 SCAMP1 EXOC5 CCDC93 SNAP23 AP3B2 SCFD1 RAB7L1 AP1G1 EHD3 RP2 VAMP2 RAB14 MYO1B SORT1 SCAMP3 EXOC1 AP2A1 MON2 VTI1A SYS1 ARL3 STX4 VPS13C KLHL20 CORO7 LAMP1 GAK SORL1 EXOC2 STEAP2 VAMP8 GOSR2 RAB10 GOPC ARF1 LLGL1 AP1AR ARFGEF2 GBF1 WIPI1 DOPEY2 CHIC2 ZFYVE20 ANKFY1 EPS15 VPS54

GO_PRIMARY_AMINO_COMPOUND_METABOLIC_PROCESS The chemical reactions and pathways involving primary amino compound. BTBD9 DDC ADC RNF180 ATP7A CHKA TPH2 HTR1A TPH1 GRIN2A ATP2B2 SRD5A1 AGMAT PDE1B

GO_POSITIVE_REGULATION_OF_PHOSPHATASE_ACTIVITY Any process that increases the rate or frequency of phosphatase activity. Phosphatases catalyze the hydrolysis of phosphoric monoesters, releasing inorganic phosphate. SMAD3 AGTR2 PPP1R15A CD300A CALM3 MAGI2 PPP2R4 PLEK GPLD1 CALM2 BMP2 SLC39A10 PPARGC1B PDGFRB VRK3 AKAP6 CALM1 MEF2C JAK2 CHP2 RGN IFNG RIPK3 ITGA2 PPP1R12A ITGA1 NPNT HSP90AB1

GO_AMYLOID_PRECURSOR_PROTEIN_METABOLIC_PROCESS The chemical reactions and pathways involving amyloid precursor protein (APP), the precursor of beta-amyloid, a glycoprotein associated with Alzheimer's disease. CLN3 APH1A PICALM ACHE LDLRAP1 PSEN1 PSEN2 DHCR24 SORL1 PSENEN ABCG1 KLK6 DLG1 NCSTN

GO_ORGANIC_ACID_BIOSYNTHETIC_PROCESS The chemical reactions and pathways resulting in the formation of organic acids, any acidic compound containing carbon in covalent linkage. FADS1 PYCRL DPYD ACMSD ALDH18A1 PRKAB1 ELOVL4 EIF2B4 PER2 ASPG ACSS1 GLS2 SUCLA2 ALOX12 SLC27A2 CAD PADI6 PAH EIF2B1 SYK LIPT2 MTRR SHMT2 GOT1L1 BAAT ELOVL2 PTPLA AKR1A1 PDDC1 ELOVL1 DECR2 DHPS ALDH8A1 AGXT2 PTGES SLC27A5 APIP EDN1 FOLH1 CYP27A1 LIPC SHMT1 MGLL PSPH ALOXE3 PLOD1 ACSM3 PRKAG3 PTPLAD1 SC5DL PADI3 ABCB11 CYP39A1 PLA2G4F MCAT DEGS1 AHCY C5orf4 C1orf93 PADI1 GLUD2 PRKAA1 HSD3B7 NOXRED1 PKLR PTPLB FCER1A GPT AMACR AGXT CYP7A1 SCD AKR1C3 SLC1A3 ACOX2 MTR GGTLC3 SEPHS2 CBR4 NDUFAB1 ACLY ACSM1 PLA2G1B LDHC PADI2 BHMT FADS3 PNPLA8 MTAP ASPA UROS GLUD1 LIAS LTA4H CD74 ALOX5AP PLP1 MYO5A SCP2 CH25H GPT2 GOT2 GOT1 ABCD3 CYP46A1 CYP1A1 PECR ACOT8 FADS6 HSD17B12 PTGDS ASS1 PRKAB2 UPB1 OLAH FASN AGMO MLYCD PTGS1 ASL KYNU NAALAD2 DHFRL1 AASDHPPT PTPLAD2 GGTLC2 GGTLC1 SCD5 PRG3 CBS ACSF3 IDO1 ASNS AKR1D1 PTGS2 BCAT1 BCAT2 ELOVL6 GAD1 PADI4 XBP1 PLA2G5 ALOX12B PTGES3 TBXAS1 ACSS2 AKR1C4 GADL1 ALOX15B BRCA1 PTGIS RDH10 ELOVL3 DHRS9 CCBL1 PRKAA2 GGT5 PTGES2 FADS2 LGSN MTHFD1 MTHFD2L HSD17B8 HPGDS DHFRP1 NAGS PSAT1 THNSL2 DHFR ALOX5 CTH LOC643181 ACOT7 STAR ACSL3 EDN2 LTC4S FA2H ABAT PRKAG1 OTC CBR1 OAT MRI1 HSD17B4 RGN GGT2 ACACA SDS MIF TECR LPL PYCR1 MSMO1 HOGA1 MGST2 GLUL ELOVL7 RBP1 ADI1 CYP8B1 CYP7B1 PYCR2 GLS ENOPH1 PLA2G4A THNSL1 BHMT2 PRKAG2 GGTA1P GGT6 PHGDH ELOVL5 ACACB KMO CPS1 PARK7 ALOX15 ASNSD1 GGT1 GGT7 FOLH1B EIF2B2 CDO1 LOC344967 SRR ALDH1A3 ALDH4A1 NANP HAAO ALDH1A2 OXSM QKI GGT3P MECR

GO_NEGATIVE_REGULATION_OF_CATION_TRANSMEMBRANE_TRANSPORT Any process that stops, prevents or reduces the frequency, rate or extent of cation transmembrane transport. STK39 C19orf26 PLN CLIC2 CAMK2D KCNE1 DRD3 ACTN2 SLC30A1 DRD2 CALM3 SLC9A3R1 CAV1 KCNE3 GNB5 ANK3 GSTM2 PCSK9 ATP1A2 ADRA2A KCNRG FMR1 EPO FGF12 OSR1 MMP9 PKD2 UBQLN1 CALM1 TLR9 KCNAB1 SUMO1 KCNE1L SLN CAV3 PRKCE NEDD4 DRD4 SRI CAB39 NEDD4L KEL TGFB1 TRDN CASQ2 CRHR1 COMMD1 KCNE2 NTSR1 CALM2 GSTO1 KCNH2 FKBP1A FKBP1B OPRL1 OXSR1

GO_POSITIVE_REGULATION_OF_SYNAPTIC_TRANSMISSION Any process that activates or increases the frequency, rate or extent of synaptic transmission, the process of communication from a neuron to a target (neuron, muscle, or secretory cell) across a synapse. CUX2 NLGN2 LAMA2 LGI1 UNC13B GRIK2 DTNBP1 VAMP2 RAB3GAP1 STX1A NPS IFNG SNAP47 IQSEC2 KIF5B CHRNB2 DLG4 TAC1 CCL2 ARRB2 UNC13A HAP1 SNCA ADORA1 KISS1 PLK2 STX1B NRXN1 FMR1 NETO1 PTGS2 DRD1 OXTR PINK1 MECP2 SLC8A2 RETN MAPK1 DRD2 GIP TACR2 STX4 CLSTN3 LRRTM1 RIMS1 DVL1 CRH CLSTN2 CLSTN1 NTSR1 CA7 SLC24A2 ADCYAP1 WNT7A NR2E1 PRKCZ ITPR3 UTS2 PTK2B NLGN1 LRRTM2 SNAP25 NMU DRD4 PRKCE RAB3B GRIN2A SHANK2 SYT12 GRIN1 PTEN RELN CA2 PTN SYT1 ABAT IGSF9B ADRA1A OXT SHANK1 S100B NTRK2 BRAF FLOT1 GPER RGS14 RAPSN STX3 SLC1A3 GLUL SHANK3 TNR RIMS2 SLC8A3 GFAP NLGN3 SERPINE2 EGFR NPTN NTRK1

GO_REGULATION_OF_RESPIRATORY_GASEOUS_EXCHANGE_BY_NEUROLOGICAL_SYSTEM_PROCESS A process carried out by the nervous system that is required for the proper control of respiratory gaseous exchange. This process occurs in the respiratory center of the brain in vertebrates. PBX3 GLS GSX2 GLRA1 NLGN2 ATP1A2 TLX3 NLGN3 MECP2 TSHZ3 PHOX2B ADORA1 NLGN1

GO_LIPID_DIGESTION The whole of the physical, chemical, and biochemical processes carried out by living organisms to break down ingested lipids into components that may be easily absorbed and directed into metabolism. LIPK PLA2G1B ABCG8 LIPM ABCG5 CEL LIPG CD36 PNLIP CLPS LIPC AKR1C1 LIPH LDLR SOAT2 NPC1L1 PNLIPRP2 ARX APOA4 LIPI LIPN LMF1

GO_ENDOTHELIAL_CELL_MIGRATION The orderly movement of an endothelial cell into the extracellular matrix to form an endothelium. PTK2B TNFSF12 FAP ROBO1 SLIT2 GIPC1 PTEN EPHB4 APOA1 EMP2 CORO1B GPX1 NOS3 MYH9 GPR124 SOX18 GPLD1 STAT1 DPP4 EFNB2 PRKX NOV TGFBR1 VEGFA CDH13 PTP4A3 NR4A1 CYP1B1 FGF2 NRP1 S100A2 PTK2 EGR3 ITGB2 KDR PRSS3 CXCL13 S100P LOXL2 GREM1 SRF TDGF1 SCARB1 SCG2 TRY6 ID1 PAXIP1 PLXND1 PIK3CA RHOA AMOT PLEKHG5 RAB13 ITGB1 STARD13 VHLL

GO_LAYER_FORMATION_IN_CEREBRAL_CORTEX The detachment of cells from radial glial fibers at the appropriate time when they cease to migrate and form distinct layer in the cerebral cortex. RELN NR2E1 C16orf45 MBOAT7 SOCS7 CDK5R1 CTNNB1 DAB2IP GLI3 PAFAH1B1 GPR56 CDK5 CDK5R2

GO_REGULATION_OF_NECROPTOTIC_PROCESS Any process that modulates the rate, frequency or extent of a necroptotic process, a necrotic cell death process that results from the activation of endogenous cellular processes, such as signaling involving death domain receptors or Toll-like receptors. BIRC3 CAV1 RIPK3 BIRC2 RIPK1 CFLAR ARHGEF2 FADD RBCK1 SLC25A4 CSDA

GO_CELLULAR_RESPONSE_TO_ENDOGENOUS_STIMULUS Any process that results in a change in state or activity of a cell (in terms of movement, secretion, enzyme production, gene expression, etc.) as a result of a stimulus arising within the organism. ACVR2B TCIRG1 JAK3 ESRP2 ADCY5 ESRRG FURIN NELF PARP1 AMHR2 DIAPH1 PDGFD NR2F1 CTSL1 NTRK2 GDF9 PGRMC2 BMP2 CRHBP GSTM2 ATP1A2 POLR2D CCNA2 SMURF1 SRC POLR2K NR1D1 NDST1 TMEM100 IGFBP1 FGFRL1 SLC9A1 FBN1 NOD1 REST KIAA0146 EEF2 GATA3 FGFR2 FBXO18 HSF1 VIL1 GPLD1 TRPV1 FGFR3 PDE4B CPS1 SSTR2 CD36 CACNA1H PRKCI ADCY7 SIN3A SIK2 CDH13 IRG1 AKR1C1 SLC34A1 JUP NR1D2 SLC33A1 DRD1 NOD2 SORBS1 NKX6-1 CAV1 PTPN1 USP15 HIPK2 KIT RHOQ BBS2 PRKACB O3FAR1 ACSL1 SIGLEC15 ASS1 DAXX GLRA1 CGN FGFBP1 MEGF8 PTGDR SOCS2 WFIKKN2 FOSB PPARA NODAL TRH ARPC3 NR4A3 RAPGEF2 WNT1 DRD5 CD81 GATA5 NR2C1 CDC5L TGFB2 PDXP RAP1A SOX5 ITPR2 ZCCHC12 EID2 SNAI2 TIA1 FLRT3 PRKAR2A MSX1 GREM2 BAMBI GNG4 LTBP2 DDC MAX SRD5A1 PPM1A ITGB1BP1 KAT5 DUSP15 LYN FER NR4A1 APOB RXRG UBE2M ATP1A1 TGFB3 ATP6V1A ZFP36L1 CD9 OPRM1 GDF7 NR3C2 NTRK1 KCNJ11 ITGB5 CIB2 POLR2B FGF16 NR1H4 TGFBRAP1 HDAC2 PPARGC1B FERMT2 LEF1 PAQR8 GPER GDF15 WDTC1 PRKAA1 UCP2 MYO5A AGTR2 HNRNPU SLIT2 KDM1A POSTN KIAA0889 POLR2C ATP6V1D FOXC2 PRKACG DSTYK MAGI2 IQGAP1 MED17 ESRRB TAF7 HSP90B1 DUSP22 NR2F2 CITED1 ADIPOQ UBE3A FGF22 GNG3 FGF9 GNG13 FGFR1 RXRA IFNB1 P2RY12 ADCY4 KDM5B PTK2 PRKACA RARA COL4A1 GHSR UBE2L3 SH3BP4 CTNNB1 ACTN2 EIF4A3 CTSS GNB2 ZFP36L2 PDPK1 ID1 TWSG1 COL6A1 SSTR1 HCRTR2 MED14 CPEB1 AVPR1B ITGB1 ESRRA NEDD8 EDN1 SRSF5 FGF7 CLEC3B USP9X HDAC9 GTF2F1 PIAS1 CCL2 GNRHR ARRB2 PDE3A FGF18 KLF15 ISL1 MDM2 THRAP3 FGF23 HAS1 UBR1 IL8 PXN TRIB3 NR5A1 AVPR2 SORT1 KCNE1 BCL2L1 COL3A1 CXCL13 ADAM9 MED30 RBFOX2 JUNB GAB1 TDGF1 SOS1 NCOA6 CHRDL1 IGFBP5 SESN1 FGF20 GNB4 DEFA3 ADRA2A RAB31 RBM14 EHD1 FGF4 IMPACT GNG10 LATS2 ASIP CRHR2 CREB1 MED12 BMP3 ATP6V1G3 AGRP TADA3 SSTR5 CASP4 TRIM71 POU4F2 STAT5B UBB PPARGC1A UBC HNRNPM SMURF2 RB1 EIF4EBP2 BMPR2 GRIP1 SSTR4 MAPK7 GSK3A SMAD3 UGT1A1 RAP1GAP HCN4 GHRHR SP1 NLK BECN1 CD44 ASNS SOD1 FOXH1 GABRG2 SMAD2 GSTP1 SPRED2 XBP1 TOB1 GCLM MED13 EFNA5 LRRK2 NPFFR1 PDE2A CAPN10 LRP4 SESN3 DEFA1B PIK3CA COL1A1 NR1I3 XCL1 MAPK3 UBR5 EGR1 MYOD1 PRKCD DDX54 PRKAR1B PTPN22 CYBA POLR2I BAG4 APEX1 SLIT3 APOA1 MED4 COL16A1 PGR HNRNPD REN KLF3 PDCD5 GATA1 ADCY1 ESR1 MED1 MAS1 JUN PPARG GLRA2 SHOC2 BRCA1 AKR1C4 NR0B2 CAPN2 EEF1A1 POLR2F CCNE1 VAMP2 SLC8A3 RRAGA BMPR1A FGF2 PRKDC GPD1 PIK3R3 FGF10 FGB STXBP4 ETV2 HYAL2 ABL1 ATP6V0E1 MSX2 DSG4 SOCS7 HCN3 TGFBR3 NPFFR2 CYBB SLC2A4 NR3C1 MICALL1 FIBP AP3S1 PTPN2 POR SH2B2 APAF1 ERRFI1 PIK3C2A UBE2D3 RXFP4 ADCY8 GOT1 GCGR SCXB AQP8 AKT2 SLC26A3 PTN RPTOR ATP2B4 SOX9 PRKCE NCOA2 PLOD3 ARHGEF2 EGLN2 FAT4 NEURL MED16 PDGFRB JAK2 PRKCZ UROS COL4A2 MED24 HDAC5 SYK KAL1 CSK HFE2 HEY1 SMAD7 CHRD PTAFR AIFM1 HNF4A NR1I2 ROBO2 PAK1 DUSP1 ROR2 RORA PHOX2B GDF6 RAB10 TGFBR2 CYP11A1 TAB1 LHCGR MSTN F11R RAB13 PDE3B ATP6AP1 SKI LMO2 RAMP2 ATP6V0D2 AR FGF17 CLDN5 ABHD2 INHBA IGF2 GLP1R ENG NR6A1 ADIPOR1 NCBP2 STC1 EME1 PRKAR1A RHOXF1 KLF2 ADCY9 GCK RYR3 ATP6V0E2 CER1 NPAS4 ATP6V0B ZCCHC18 IDE GPR173 CARM1 HNRNPA1 QRFPR ATP6V0A4 NAMPT SCGB2A1 STMN2 ENPP1 ARHGEF18 PRLR BMP8B PMEPA1 IRS2 BMP15 SPINT2 HCN1 RARB DNMT1 AVPR1A INS RRAGB KLF11 LAMTOR3 BMP10 EGFR PARD3 PPP1R9B SOX6 FMOD BMP7 ACACA RORB LPIN1 RAP1B CPEB3 TGFB1I1 PTPRK T FECH DNMT3A BAD ZFP106 FOXO3 ACVR2A PCK1 SMAD4 PTGER2 DLL4 AKT1 PTGFR STAT1 SIRT1 UBE2D1 TFAP4 GNB3 EPB49 SLC8A1 BMP1 YES1 MMP2 FAM83G POLR2H STAT3 PTGER4 AKR1C2 RAB14 SSTR3 FOS DAB2IP AKR1B1 ATP6V1F HNF4G LEFTY1 P2RY11 PDK4 P2RY6 PTH MAPK1 RPS6KB1 IPO5 LATS1 SNCA CIB1 ATP6V0A2 CAMK2A AKAP6 FSHB GHRL ESRP1 SLX4 P2RX4 ATP1A3 BMPR1B ESR2 MYH6 ACVRL1 CTNNA1 KIDINS220 APPL1 TIAL1 RAPGEF3 AQP9 LAMTOR1 LEPR GTF2F2 IGF1R IRS4 CTGF KLB STAT5A RGMA ARG1 RXRB RPLP0 TGFB1 CTSH COL2A1 POLR2G GNB1 TGFBR1 ATP6V1C2 APOBEC1 RGMB ICAM1 SELS RORC SFRP1 PKLR ARID1A HPGD P2RY4 CAV2 CX3CR1 AKR1C3 RAN ITGB2 NCOA1 TNFSF4 HCRTR1 GNG5 TH KDM3A PTPN12 UBA52 RARG YWHAH RAB8A ANXA1 NOTCH1 DEFA1 TBX1 CYP11B1 SKIL CORO1A FYN FGF12 FGF19 INPP5K HAS2 GRB2 GCLC USP9Y SHC1 HEYL POLR2L GHR PIK3R1 CA2 NEDD4 CORO1B ATP2B1 PLA2G1B C19orf2 PADI2 STK16 SMAD9 THRB TRIP4 HNRNPF EIF4EBP1 PARK2 SLC2A8 NR2F6 PIK3CG FKBP1A ADCYAP1 CYP11B2 PML MUS81 ATP6V1B2 PTPN11 NFKB1 EGR3 SREBF1 AGTR1 WNT2 SMAD5 IGFBP7 USF1 BPTF PHB NR2E3 ATP6V1E2 GABRB1 RAB35 UMODL1 SPINT1 EEF2K CEACAM1 PIK3R2 IL6 AKAP9 NR5A2 GPR152 SMARCC1 CAD PDE4D CSNK1E NR2C2 GCNT2 FOXO1 PAQR6 TAC1 PRL FGF5 SERPINF1 NCBP1 LAMTOR2 DNM2 FRS3 SOCS1 ADAMTS12 DNAJA1 NOG BMP4 ANKRD1 WNT10A PENK RANGAP1 PTBP1 GDF10 FLT3 AGTRAP NR0B1 ACAP2 FGF21 RYR2 POLR2E SST GH2 FSTL1 ATP6V1G2 CPEB4 AKAP8 SMAD6 CEP57 KAT2B FBXO32 AIF1 FOLR1 RYR1 TBC1D4 ATP6V1C1 MKKS CCL5 NPC1 GDF2 NR1H2 KAT2A E2F1 CSNK2B SOST STAR ZNF8 GPR22 FLRT2 AANAT GNG12 FAM59A CALR MTMR4 SLC26A6 KLF4 BMP8A PDGFC MAP3K7 SEPN1 TNC FNTA RELA IRS1 APLP1 PAQR9 HBXIP CASP9 THRA ZFYVE9 PTGIR CRH CTSB MEF2C RDX WNT7A LEFTY2 C7orf59 SMAD1 CDH1 NR1H3 BBS4 BCAR1 BAIAP2L1 HMGCS1 LEP SETX PTGS2 PIAS2 GABRA1 NOX4 SLC5A5 SLC27A1 SHCBP1 C4orf49 POLR2A ADIPOR2 TNMD ATP6V0A1 FGFR4 NKX2-5 PTPRA PLCG1 PID1 NUP62 PPAT RAPGEF1 GABRB2 MCM7 RIPK2 NKX3-1 ABP1 RNF14 GDF1 IGFBP2 DLX5 COL5A2 ERBB2 FGF3 EPB41L5 PAQR7 RAD51 INSR SNRNP70 CDC6 MEN1 PGF POLR2J HDAC4 FOXO4 EZR FGF8 EZH2 PHIP CBL BAIAP2 INHBB ATP6V1G1 RPE65 PRKAA2 ZNF259 ZEB1 FRS2 SLC39A5 NRIP1 FHL2 TNF PAQR5 ATP6V0D1 GATSL3 GNG7 GATA6 ATP6V1B1 HCN2 HTR1B RRAGC PRKAR2B KCNQ1 PDK2 PARD6A DENND4C PCSK9 CALCA PDGFB RPL32 FCER1G CEBPB CDK7 RHOA ADCY6 HYAL1 CFTR GAS2L1 WT1 SIRT2 PAX2 RRAGD SSH1 VDR BMP5 SAFB AHCYL1 ACVR1 COL1A2 BLM ADCY2 GNG2 FUT8 ADAMTS7 BAIAP2L2 NR4A2 FGF1 SESN2 WNT5A ATP6V1H UBR2 CALCOCO1 FKBP4 CRHR1 SMYD3 GNG11 NR2E1 PPAP2A PAX8 HRH1 EPHA8 YWHAG CPEB2 HFE OXTR KL OR51E2 GALNT3 NCOA4 FOLR2 KLF5 SPRED1 ADCY3 TSHR GLP2R ZFP36 KLF10 PPARD GABRB3 SLC6A4 WNT4 KIAA0528 KLF9 APP GCG ZFYVE16 GNG8 RNF4 NCOA3 TREM2 GH1 WWOX FGF6 ATP6V1E1 SLC25A33 CDH5 RPS27A KIF16B GDF5 RUNX2 BMP6 UCP3 KLF1 ATP6V0C LHX1 SLC38A9 CASQ2 FLRT1 HNRNPH1 LIMS1 MT3 WNT10B CSNK1D SIK1 ZYX GNRHR2 JUND AQP1 DNMT3B APRT FSHR

GO_REGULATION_OF_CELL_GROWTH_INVOLVED_IN_CARDIAC_MUSCLE_CELL_DEVELOPMENT Any process that modulates the rate, frequency, or extent of the growth of a cardiac muscle cell, where growth contributes to the progression of the cell over time from its initial formation to its mature state. IGF1 DDX39B CAV3 GSK3A HAMP EDN1 MTOR G6PD PIN1 AKAP6 CTDP1

GO_DNA_REPLICATION The cellular metabolic process in which a cell duplicates one or more molecules of DNA. DNA replication begins when specific sequences, known as origins of replication, are recognized and bound by initiation proteins, and ends when the original DNA molecule has been completely duplicated and the copies topologically separated. The unit of replication usually corresponds to the genome of the cell, an organelle, or a virus. The template for replication can either be an existing DNA molecule or RNA. ACHE C20orf72 RMI2 CDT1 POLI NUCKS1 MMS22L POLD4 POLE2 POLD3 RFC5 POLB RAD9A SLX4 GINS2 RECQL4 MCM7 MRE11A CINP CHTF18 POLE3 TOPBP1 DUT RAD17 NT5M PARP3 ATRIP SSBP1 RAD51 RFC2 LIG3 DBF4 BAZ1A GMNC NFIA POLA1 MCM10 C20orf43 CDC45 NOL8 DSCC1 ORC2 CDK9 BRCA1 KAT5 PARP2 CDK1 PRIM2 TIMELESS CCNE1 ZNF259 SPHAR CDC6 ORC3 NASP LIG4 REPIN1 EME2 TOP2A DTD1 POLD1 DTL RRM2 MCM3AP SSRP1 MCM3 KIAA0101 PARP1 C10orf2 SET CDC25A NFIX POLG2 NCOA6 RNASEH1 GINS3 ATRX CLSPN RBM14 TBRG1 FAM111A TIPIN FBXO18 TOP1 REV3L SLBP CORT RPA3 RRM2B ORC1 ING4 KCTD13 NAP1L1 RPA1 PRIM1 TOP3A PCNA C8orf45 NUP98 RBBP8 SIN3A CDC7 RFC3 PURA POLE BARD1 MCM8 SIRT1 RAD9B ORC6 POLA2 ZNF830 RRM1 SUPT16H RECQL5 TOP2B EGF C15orf42 EXO1 NBN PNKP WRNIP1 RTEL1 RPAIN WRN STRA8 DONSON RFC1 CDC34 MCMBP RPA2 INO80E ING5 THOC4 C12orf32 CCNE2 POLQ TFAM ATM CCDC111 EME1 CDK2 TNFAIP1 SLC25A33 NFIC POLH DNAJC2 REV1 TONSL SMARCAL1 DNAJA3 LRWD1 KIN POLG C17orf42 FANCM IGF1 HELB CCDC88A POLK SETMAR CHAF1A MCM4 IGHMBP2 THOC1 HUS1 APITD1 BRCA2 RBBP7 BRIP1 RAD50 PTMS NFIB RBBP4 GINS1 E4F1 ANKRD32 BOD1L ORC5 RMI1 MCM2 GINS4 CDC25C KAT7 POLD2 POLN ATR POLL RBMS1 BLM ZRANB3 CDK2AP1 MCM9 LIG1 DNA2 CHAF1B FEN1 RPA4 CHEK1 MCM5 RAD1 ORC4 RBBP6 MCM6 TOP1MT HMGA1 UPF1 STRA13 RNASEH2A RFC4

GO_REGULATION_OF_STEM_CELL_DIFFERENTIATION Any process that modulates the frequency, rate or extent of stem cell differentiation. N4BP2L2 GATA6 HES1 BMPR1A STAT3 YAP1 HES5 HOXB4 TBX5 PWP1 PDGFRA GATA4 NKX2-5 TRIM6 LTBP3 NOTCH1 ZFP36L2 SOX6 KDM3A SOX5 TGFB2 SOX9 ENG FOXC1 REST TEAD2 GSK3B PRICKLE1 WNT3A NFE2L2 JAG1 GDNF H1FOO TACSTD2 SP7 EIF2AK2

GO_REGULATION_OF_PROTEIN_EXIT_FROM_ENDOPLASMIC_RETICULUM Any process that modulates the frequency, rate or extent of the directed movement of proteins from the endoplasmic reticulum. TMEM30A GCC2 TMEM30B EDEM2 OS9 EDEM1 UBE2J1 BRSK2 SVIP DERL2 SORL1 BCAP31 UBE2G2 SEC16B SLC35D3 OSTBETA YOD1 TM9SF4 DERL3 UBAC2 ERLEC1

GO_REGULATION_OF_TELOMERE_MAINTENANCE_VIA_TELOMERE_LENGTHENING Any process that modulates the frequency, rate or extent of telomere maintenance via telomere lengthening. CCT3 MAPK3 PINX1 SRC ATR SMG6 HMBOX1 MAP2K7 NEK2 MAPK1 CTNNB1 NAT10 PNKP MAPKAPK5 PIF1 NEK7 CTC1 MAP3K4 OBFC1 PARP1 ACD RTEL1 TERF1 TEN1 TCP1 CCT5 HNRNPA1 POT1 PKIB DKC1 WRAP53 MAPK15 TERF2 TINF2 AURKB TNKS2 SMG5 PRKCQ TNKS CCT6A CCT2 ATM GNL3L CCT8 HNRNPC HNRNPU CCT7 CCT4

GO_POSITIVE_REGULATION_OF_VASODILATION Any process that activates or increases the frequency, rate or extent of vasodilation. ALOX12 ADCYAP1 PTGDR GPER GJA1 CPS1 KCNMB1 NOS2 NPPC APLN EGFR PTPRM INS PPARD NOS3 HMOX1 GJA5 F2RL1 PLA2G6 EPHX2 SCPEP1 PTAFR UTS2 AGTR2 NOS1 UCN ADM AGT

GO_RESPONSE_TO_NUTRIENT Any process that results in a change in state or activity of a cell or an organism (in terms of movement, secretion, enzyme production, gene expression, etc.) as a result of a nutrient stimulus. LTA TSHB DAD1 NQO1 STC1 CCL2 PENK CDKN2D MDM2 TRIM25 UCP3 ALDH3A1 CDKN2B LDHA DNMT3B SST FGF23 CYP24A1 FOLR2 OGG1 RARA MTOR OXCT1 SRF HMOX1 USF1 PPARD SSTR1 SLC6A4 TGFBR2 TBXA2R EPO GAS6 LIPG VDR POSTN GCGR PDX1 GCLC HAMP ATP2B1 CYP1A1 PTEN PTN SETDB1 SPP1 ADIPOQ PEMT F7 USF2 CAT CCKAR AKR1C3 SFRP1 SFRP2 PKLR PDK2 TSPO NCOA1 NR1H4 AHCY ABCG5 CXCL10 POR COX4I1 ABCG8 CYBB ACSL4 BRIP1 TXN2 TTPA APAF1 ALAD IGFBP2 GNPAT GIPR BGLAP SERPINC1 ARG1 ALPL ENSA HMGCR ADSL APOA1 IL15 TGFB1 PPARG HAT1 ERCC1 ASCL1 TNFRSF11B PTGS2 NOD2 MGMT BECN1 SLC6A19 LEP HMGCS1 SLC34A1 UGT1A1 XBP1 GCLM PTH RPS6KB1 AACS HLCS TPCN2 FOXA2 STC2 CD4 VCAM1 GSTP1 GNAI2 COL1A1 ASS1 ACSL1 ADIPOR2 KYNU GATA4 EEF2 SRSF2 DNMT3A IL4 ABCA1 TNC HSF1 TYR CYP27B1 ALDH1A2 AQP3 SSTR2 CCL28 STAT1 RELA ARSB BCHE CCND1 HMGCL FKBP1B OGT CHMP1A SPARC ADA MTHFR MEF2C TYMS SLC8A1 FOLR1 PIM1 C2 EGFR SLC27A4 CNR1 CD3E OTC BMP7 STAR ACSL3 PITX2 ITGA2 SLC16A1

GO_SNORNA_METABOLIC_PROCESS The chemical reactions and pathways involving snoRNA, small nucleolar RNA, any of a class of small RNAs that are associated with the eukaryotic nucleus as components of small nucleolar ribonucleoproteins. They participate in the processing or modifications of many RNAs, mostly ribosomal RNAs (rRNAs) though snoRNAs are also known to target other classes of RNA, including spliceosomal RNAs, tRNAs, and mRNAs via a stretch of sequence that is complementary to a sequence in the targeted RNA. EXOSC2 RNF113B EXOSC6 NUDT16 GAR1 FBL NOP10 RNF113A EXOSC4 FBLL1 EXOSC3 EXOSC5 DKC1

GO_REGULATION_OF_NITRIC_OXIDE_SYNTHASE_BIOSYNTHETIC_PROCESS Any process that modulates the frequency, rate or extent of the chemical reactions and pathways resulting in the formation of a nitric-oxide synthase enzyme. TLR2 MAP2K6 JAK2 MAP2K4 CCL20 FCER2 GSTP1 PGGT1B CCL2 SELS NAMPT EDN1 TLR9 TLR4 KDR NOD2

GO_REGULATION_OF_OXIDOREDUCTASE_ACTIVITY Any process that modulates the frequency, rate or extent of oxidoreductase activity, the catalysis of an oxidation-reduction (redox) reaction, a reversible chemical reaction in which the oxidation state of an atom or atoms within a molecule is altered. One substrate acts as a hydrogen or electron donor and becomes oxidized, while the other acts as hydrogen or electron acceptor and becomes reduced. AGT CCS KRAS TNF DDAH1 ABL1 CNR1 HSP90AA1 INS EGFR GLA ZDHHC21 GFI1 EDN2 HDAC6 SPR POR RGN IL13 GZMA CALM1 HIF1A DDAH2 NOD1 PTK2B ACVR2A AGTR2 FXN VDR CYP27B1 ECSIT FCER2 CAV3 ATP2B4 NOS3 CDH3 AKT1 CALM2 APOE ATP7A CYGB PARK2 PARK7 IL1B OXA1L RIPK3 NFKB1 ABL2 NPR3 CNR2 LEP NOD2 PTS PRKG2 AGTR1 SZT2 CAV1 HTR2B NOS1AP WASL SCARB1 CALM3 GCH1 SNCA LRRK2 NOSIP FTMT GCHFR CYBA EDN1 DNM2 NOSTRIN DHFRP1 DRD5 SIRT4 HP DHFR LYPLA1 TERF2 GDNF RFK FGF23 IFNG MT3 DAOA TERT

GO_CLATHRIN_COAT_ASSEMBLY The process that results in the assembly of clathrin triskelia into the ordered structure known as a clathrin cage. D4S234E PICALM CALY AP2B1 EPS15 PIK3C2A CLINT1 HMP19 AP2S1 FCHO1 FCHO2 SNAP91 HIP1

GO_ORGANONITROGEN_COMPOUND_BIOSYNTHETIC_PROCESS The chemical reactions and pathways resulting in the formation of organonitrogen compound. ADCY6 PADI1 RPS11 HYAL1 RPL10A GPC1 EFTUD1 RPL9 RPS3 SELT ASPDH EIF1AY PADI3 HDC DCK RMND1 RPL32 ATP5E EEF1D OPLAH MRPL35 PPA2 NOA1 AGXT PTPLB CTPS ACAN AK3 MRPS10 MTFMT SLC25A30 RPL11 CHST1 ST3GAL2 IL1B PPAP2A AARSD1 MRPL54 HS3ST6 UCP1 SRM PDF RPS15 TNIP1 BHMT SLC25A21 ADCY2 P2RX7 GAL3ST1 ATP5L2 NR4A2 MRPL48 MRPS9 TPH1 DMD UMPS GOT2 FARSB NDST2 GPT2 LARP1 MRPS16 SPHK2 EIF5B GSTA3 ADK OAZ1 COPS5 SPTLC2 AMPD2 MRTO4 LCMT2 RPS7 ATP5G2 ADCY3 UPP2 CMPK1 RSL24D1 KERA TYW5 CPEB2 PTS CDKAL1 EIF1AX CHPF2 MTRR PARS2 CHSY3 SHMT2 RPL27 PCBD2 B3GALT6 EIF4A2 ATP5S SLC25A17 AQP1 APRT PPCDC UCP3 SGPP1 NHP2L1 GADD45GIP1 DSE VAPB RPS27A RPL23 RPS10 GUCY2F ABCC5 KDSR SLC25A33 SPTLC1 EXT2 PRHOXNB SLC11A2 SLC25A45 BGN YARS2 PLOD1 SGPP2 MRPS24 RPL21 MOCS1 SLC25A44 RPL35 B4GALT1 RPS17 TK1 TBPL1 OTC MRI1 MRPL15 AANAT ATP5C1 MRPS5 EIF3CL NMNAT3 MRPS26 AMPD3 CHPT1 CPOX EXTL1 GSTT2B PYCR1 PPOX PSTK TYMS A3GALT2P ITGB1BP3 MRPL38 EIF6 GTF2H3 GGT7 SLC25A3 FOLH1B RPL26 EIF5A2 AASDH EIF1B MMACHC KMO RPL38 GGT6 AGMAT RPS3A SGMS1 ST8SIA6 TYMP PRELP ATP5J2 MRPL19 ODC1 RPL35A RPL17 RPS14 RARS2 MRPS11 ST8SIA5 GBA ST8SIA1 LUM SRR UST ALDH3B1 ALDH4A1 PPAT QARS SLC25A43 EIF3A MRPS23 LARS2 SPHK1 EIF1AD GART DCN GCH1 GSTO2 ATP6V0A1 MRPS35 MRPS6 DUT EIF4E EIF3G PDCL SLC27A1 RPL13 ATP5B RPL24 SLC25A29 GAD1 TXNDC3 RPL15 NPR3 MRPL55 CHST9 PANK4 PAIP1 MOCOS PDCL3 HS3ST1 COX10 COX15 ATP5A1 SDC3 RPL5 GADL1 CCBL1 DAP3 HDHD1 ELOVL3 CHST5 MRPL28 SGPL1 STOML2 CHST2 RPLP2 EARS2 RPL13A PANK2 PSAT1 B3GNT8 UGCG DHFRP1 CMPK2 DCAKD CTH MRPL30 DHFR SAMD8 MRPL45 CHSY1 PTDSS2 ALAD EIF3H ATIC MTHFD1 LGSN NMRAL1 ASMT PAPSS2 MRPL4 GATC HAS2 ST6GALNAC2 RPL36AL FARS2 AK5 SPTSSB MRPL14 CSGALNACT1 MRPL10 TH UBA52 DEGS2 DEGS1 ACAT1 RSAD1 RPS16 TSPO EEF1A2 SLC25A20 PDCL2 B4GALT3 CALCRL TXNDC9 PKLR NPPA MRPS31 IGHMBP2 IMPDH1 SLC1A3 PAOX PCSK5 GUCY1A3 MCART6 EIF4EBP1 ATP5I EFTUD2 GUF1 PADI2 LDHC ST8SIA2 CEBPA MRPL41 NMNAT1 PTCD3 UPP1 ABCF1 MRPL39 GCLC DSEL SLC25A6 P2RX1 CAD GFM1 MRPL22 DAO FTSJ1 GCNT2 PPCS NME1 ACMSD ALDH18A1 RPL22 PYCRL EEF2K ASPG ELOVL4 DDX3Y HTR2B EIF2AK4 MRPL16 C15orf42 CHAC1 GSTM5 RPS21 MTOR MCART1 EIF3M GOT1L1 GTF2H2 SLC25A39 RPL12 RPL18 CHCHD10 MRPL53 TAAR1 EXTL3 GUCY2C RPL7L1 APIP GBAS CPEB4 SDC1 EIF2S2 PNP SSB PTBP1 EIF2AK2 RPL3L TPH2 ARG2 B3GNT2 GUK1 SLC25A4 ETF1 PTPLAD1 CHCHD1 SHMT1 AZIN1 DCTD GUCY1A2 SLC25A15 ALOXE3 HPRT1 RPL7A VARS MTERFD2 SLC25A10 SLC25A5 CPEB3 ALDH3B2 MRPS22 ATP5EP2 C16orf7 ATPIF1 CERS3 NDUFA7 RPS5 FMOD B4GALT6 FA2H ABAT PAPSS1 BBOX1 GTPBP2 SARS2 EIF3I MRPS33 EIF4E3 EGFR AKD1 GARS NAMPT TARS2 NARS MGST2 MRPL33 ATP5G1 MTHFS TARS RPS4Y2 ACPL2 COX5B SLC25A18 ASNSD1 AKT1 BHMT2 PRKAG2 RPL4 ENOPH1 XYLT1 EIF3E TMEM14C ALAS2 PABPC1 NPR1 ST8SIA4 ATP5L EIF2S3 RPL28 FECH HEXA NME9 CDO1 GPHN ATP5F1 HAAO GMPR HARS MRPL34 FAM57B GSTK1 SLC25A1 BDH2 KYNU UCKL1 GPC4 SNCA SLC25A24 PPA1 ATP6V0A2 UPB1 EIF4H MRPL46 RPL29 SLC25A48 EEFSEC EIF4E1B RPL18A OGN PRG3 MMAB CBS NME3 RPS6KB2 SMPD4 C7orf36 MRPS34 ST6GALNAC4 MRPS2 RPL39P5 TUFM AARS2 XYLT2 RPLP0 EIF5A TGFB1 INSM1 TK2 MRPL17 RPS9 GPC5 ARG1 SMPD1 CERS5 CERS6 EIF4G2 ATP5D RPL6 MTHFD2L GUCY2D EIF5AL1 ST3GAL3 CERS4 PANK1 CARNS1 RBM4 GSPT2 CSPG5 ST6GALNAC6 EIF2B3 ST3GAL1 MRPS25 UROD PRPS1 ST6GALNAC3 SPR ALDOA RPL30 CHST7 GAMT DENR MRPL20 MRPS28 MOCS2 GPT GPC3 B4GALT5 HAS3 TSFM MRPS7 AFMID RPS13 TARSL2 SMPD2 UROS QTRTD1 MRPS18B RPL13AP3 FARSA B4GALT4 SLC25A34 MTHFD2 GUCY1B3 PTDSS1 ASPA B4GALT2 MTAP KIAA1199 PDGFRB MMAA AGRN MRRF MRPS18C GSTM4 EEF1E1 PPM1L GMPR2 LPCAT2 RPL36A B3GNT1 CHAC2 NME6 PFAS ADCY8 FXN GOT1 RPLP0P6 EIF3F SPTA1 IARS GLS2 GCN1L1 PLSCR1 PADI6 GLCE PAH RPL39 EIF4G1 MRPS27 SECISBP2L DPYD DHODH ACER3 RPL27A SECISBP2 MGST1 EIF2B4 MRPS21 RPS25 ELOVL2 OAZ3 ST3GAL6 UCK1 QTRT1 EIF2B1 MRPS17 MRPL12 COL4A3BP CTPS2 MTRF1L GTPBP1 AGXT2 SLC25A37 RFK ESD RPL19 RPS6 GSTO1 ATP6V0A4 SLC35D2 CERS2 ADCY9 CARS ADORA2A IARS2 MRPL50 CSGALNACT2 GUCA2B SLC35B2 AK4 EEF1B2 RPS19 GSTA4 PRKD1 ERH AIMP2 ADAL SLC25A36 GSTA2 PAICS FOLH1 EIF4E2 SLC25A23 RAMP2 KARS PSPH CHST13 C20orf173 PNPO ATP5O AIMP1 RPL37 TPK1 GGT2 CSPG4 POLG2 RPSA A4GALT QDPR NADKD1 ACOT7 CHST15 RPL39L CLN8 C9orf95 SLC25A27 EIF4G3 MRPL32 MTERFD1 UCN2 GLUL SLC25A35 RPL34 UGT8 MRPL27 ADA HS3ST3A1 RPS18 CHST12 GGT1 HS6ST1 ATP5J DIO2 RPS26 EIF4EBP2 PHGDH NHP2 GSPT1 HAGH AMD1 MRPL51 EEF1A1P5 EIF4A1 VARS2 CHST11 EIF2C2 RPL31 MTRF1 EIF2B2 RPS12 N6AMT1 ADSS SLC25A16 LARS RPL3 CHDH EIF5 AASDHPPT MRPL11 RPS28 ALDH7A1 HS3ST2 ST6GALNAC1 MRPS15 SLC25A40 DARS2 NPPC NFS1 GMPS FPGS C11orf83 ELOVL6 HS3ST5 UPRT GSTP1 LOC255308 GCLM GSS COASY MTIF3 PTPLAD2 NPPB BCAT1 GGCT MRPL52 CHST14 ASNS DARS OMD PCBD1 GPC6 EEF1A1 GGT5 MOCS3 MRPL1 ADCY1 PANK3 SPTSSA SGMS2 FLAD1 ADSL NAGS VAPA MRPL2 NPR2 ST8SIA3 SLC6A3 MGST3 HPGDS SLC25A19 MARS CHST3 B4GALNT1 EIF2D B4GALT7 MRPL9 WARS SLC25A12 NOXRED1 GLUD2 HTR2C MRPL47 SLC25A2 ACER2 RPS2 AHCY GALNT5 HSPG2 GFM2 ATP5G3 SLC26A1 B3GAT1 SEPHS2 MRPL40 RPL14 TRNAU1AP MTR GSTA1 SPTLC3 GLUD1 DALRD3 EIF3J HS2ST1 RPS4X ABCB6 ATP5H WARS2 PNMT SLC25A28 GSTM1 SLC35D1 B3GNT7 SLC25A41 SLC25A32 ACER1 TMLHE CHPF MRPL13 EIF3L RPS8 RPS4Y1 UCP2 HBS1L MRPL21 AGTR2 PPAP2C HS3ST3B1 MRPS12 PDC HMBS SUCLA2 BCAN SULT1A2 RPS20 CPEB1 GPC2 EXTL2 SLC25A25 RPL37A SMOX LARGE EIF3D SDC2 PTRH1 YARS MCTS1 NME2 SLC26A2 MRPL44 CNDP2 RPS27L EIF2S1 AKR1A1 RPS23 ADCY4 RPS29 SOX11 NDST3 EXT1 HAS1 RRBP1 HAND2 SLC35B3 ALDH9A1 MRPL43 MARS2 ST3GAL4 RPL7 ELOVL1 RSL1D1 MTIF2 CKM DHPS ME1 DTYMK EEF1G ABCE1 RPL26L1 SLC25A38 RPL37AP8 ICT1 ADM ST3GAL5 MRPS18A PCSK1 NAPRT1 SLC25A26 RPL10 EIF2A DBH EIF1 NMNAT2 SLC25A31 SURF1 ALDH3A2 MRP63 RPLP1 CDA ABTB1 OAZ2 LRRC47 RPS15A GSTM2 OAT RARS NME4 GSTM3 SEPSECS ALAS1 HARS2 ANGPT1 AK1 NADSYN1 PRTFDC1 ADI1 PYCR2 GLS C12orf65 RPS24 SARS CERS1 ADSSL1 TCIRG1 ADCY5 AGK FURIN ELOVL7 ADC MRPL3 NCAN ADCY7 MCART2 SDC4 GSTT1 PCBP2 CYTL1 THNSL1 PLA2G4A AURKAIP1 CSNK1G2 RPL22L1 DGUOK ELOVL5 FDXACB1 CPS1 NARS2 SLC25A47 NDST4 ST6GALNAC5 RPS27 SLC25A42 PPAP2B SAT1 GATA3 SLC25A14 UGDH ACADM GGT3P B3GAT2 NDST1 RPL10L EIF2B5 MRPS14 ADCY10 IGF2BP3 IMPDH2 EIF4B EEF2 CECR1 B3GAT3 METTL17 EIF3K SLC25A11 DHFRL1 NAALAD2 IBA57 MRPL18 HS6ST2 EPRS ASL CDC123 APEH MRPL49 PABPC4 MRPS30 RPL36 TRMT112 SLC25A13 ASS1 RPL41 MTHFD1L GPR37 BCAT2 PADI4 NME2P1 MRPL24 QRSL1 CARS2 EIF3B C7orf44 VCAN B3GNT3 AARS MRPL36 AMPD1 ALOX12B NFE2L1 PLA2G5 SLC25A22 EIF3C UCK2 RPL23A DDC NT5E RPL8 GATM NME7 AK2 DHX29 TGFB2 MRPL37 SMS GSTZ1 THNSL2 PET112 NME5 CHST6 MRPL42 NADK MRPL23 MRPS36 B3GNT4 QPRT CSAD

GO_INDOLE_CONTAINING_COMPOUND_METABOLIC_PROCESS The chemical reactions and pathways involving compounds that contain an indole (2,3-benzopyrrole) skeleton. AANAT DDC RNF180 ATP7A TDO2 KMO TPH2 ACMSD KYNU CCBL1 SRD5A1 AFMID ASMT IDO1 BTBD9 HAAO IDO2 SLC34A1 HTR1A TPH1 GRIN2A ATP2B2 GCDH AADAT PDE1B

GO_CELLULAR_RESPONSE_TO_PEPTIDE Any process that results in a change in state or activity of a cell (in terms of movement, secretion, enzyme production, gene expression, etc.) as a result of a peptide stimulus. LYN SRD5A1 ATP6V1C2 APOBEC1 MAX GNG4 TGFB1 GNB1 PRKAR2A STAT5A ARG1 CDC5L WNT1 NR4A3 IRS4 IGF1R APPL1 SOCS2 ASS1 ATP6V0A2 CAMK2A PRKACB RHOQ MAPK1 CAV1 SORBS1 NKX6-1 PDK4 PTPN1 NOD2 ATP6V1F AKR1B1 STAT3 ADCY7 GNB3 PRKCI CPS1 AKT1 GPLD1 STAT1 HSF1 PCK1 FOXO3 FBN1 ZFP106 NOD1 SLC9A1 IGFBP1 RAP1B SRC LPIN1 CCNA2 CRHBP INS KLF11 IRS2 PARP1 PRLR ENPP1 ADCY5 TCIRG1 NAMPT JAK3 TRIB3 ATP6V1G2 PXN GH2 MDM2 FGF21 AGTRAP KLF15 CCL2 GNRHR RANGAP1 HDAC9 SRSF5 EDN1 PRL FOXO1 SMARCC1 GPR152 CPEB1 PIK3R2 PDPK1 IL6 ID1 UMODL1 EEF2K CEACAM1 ATP6V1E2 USF1 ACTN2 GNB2 GHSR PRKACA PTK2 SREBF1 AGTR1 NFKB1 ADCY4 ATP6V1B2 CYP11B2 GNG13 GNG3 EIF4EBP1 SLC2A8 ADIPOQ PLA2G1B PRKACG PIK3R1 CA2 FOXC2 GHR ATP6V1D KIAA0889 AGTR2 SHC1 MYO5A UCP2 GCLC GRB2 WDTC1 GPER INPP5K FYN CYP11B1 RAB8A GNG5 ZFP36L1 ATP6V1A CAV2 PKLR SELS FER NR4A1 VAMP2 RPE65 BAIAP2 INHBB ATP6V1G1 PHIP FOXO4 PPARG MAS1 CDC6 ADCY1 MEN1 KLF3 INSR APEX1 CYBA PTPN22 RIPK2 PRKAR1B PRKCD PPAT MAPK3 PIK3CA PTPRA ATP6V0A1 CAPN10 C4orf49 XBP1 GSTP1 SP1 GHRHR BAIAP2L1 GSK3A BCAR1 EIF4EBP2 RELA IRS1 STAT5B CASP4 ATP6V1G3 CRHR2 CREB1 GNG10 KLF4 GNG12 RAB31 GNB4 SOS1 STAR GAB1 ATP6V1C1 TBC1D4 KAT2B APRT ATP6V0A4 GPR173 IDE GNRHR2 ATP6V0B ATP6V0E2 ATP6V0C KLF2 GCK ADCY9 KLF1 PRKAR1A GLP1R IGF2 ATP6V0D2 SLC25A33 GH1 ATP6V1E1 PDE3B ATP6AP1 GNG8 RAB13 GCG KIAA0528 CYP11A1 RAB10 PAK1 KLF10 KLF5 ADCY3 GLP2R KL CSK CPEB2 YWHAG HDAC5 PRKCZ JAK2 GNG11 CRHR1 ARHGEF2 ATP6V1H BAIAP2L2 NR4A2 AKT2 GNG2 ADCY2 GCGR AHCYL1 RXFP4 GOT1 ADCY8 PIK3C2A ERRFI1 ADCY6 SH2B2 AP3S1 PTPN2 POR PCSK9 DENND4C SLC2A4 SOCS7 PDK2 PRKAR2B ATP6V0E1 ATP6V0D1 GNG7 ATP6V1B1 STXBP4 PIK3R3 PRKDC

GO_NUCLEAR_TRANSCRIBED_MRNA_CATABOLIC_PROCESS_EXONUCLEOLYTIC The chemical reactions and pathways resulting in the breakdown of the transcript body of a nuclear-transcribed mRNA that occurs when the ends are not protected by the 5'-cap or the 3'-poly(A) tail. LSM6 EDC3 EXOSC9 LSM1 EXOSC7 EXOSC6 EXOSC2 PATL1 DCP1B DIS3 CNOT6 DCP1A EDC4 DCP2 EXOSC3 DCPS EXOSC5 CNOT8 EXOSC1 EXOSC4 LSM7 DIS3L2 POLR2G LSM5 LSM2 LSM4 EXOSC8 DDX6 LSM3 NT5C3L CNOT7

GO_THIOESTER_BIOSYNTHETIC_PROCESS The chemical reactions and pathways resulting in the formation of a thioester, a compound of general formula RC(=O)SR' in which the linking oxygen in an ester is replaced by a sulfur atom. They are the product of esterification between a carboxylic acid and a thiol. THEM4 ACSL4 SLC25A1 MLYCD FASN ACACA ACSS1 ACAT1 ELOVL4 HSD17B12 ACSL1 ACSL3 ACOT7 ACOT8 PPT2 PDHB ACSL6 PTPLA ACSBG2 GCDH ELOVL6 ELOVL2 ELOVL7 PDHX SCD ACSF3 ACOT12 SCD5 ACSBG1 PTPLB AGK TECR ACOT13 ACSF2 ACOT11 ELOVL3 PDHA1 BRP44 ACACB ELOVL5 THEM5 ACOT9 PPT1 ACSS2 DLD ACOT4 ELOVL1 ACSL5 ACLY DLAT ACOT2 ACOT1 PDHA2 LOC344967 ACOT6 BRP44L

GO_LIPID_HOMEOSTASIS Any process involved in the maintenance of an internal steady state of lipid within an organism or cell. ABCA12 LDLR ACAD9 GOT1 ABCA2 APOA4 CEBPA SESN2 FITM2 CAV3 ACADM ABCA1 ACADSB ORMDL2 SIRT1 ACAD10 APOC4 ORMDL1 ACSM1 G6PC APOE CTAGE5 MALL GPIHBP1 ETFA NR1H3 USF2 C19orf80 PTCHD2 ABCB4 PNPLA2 ACSM2A TMEM97 APOB ACADVL SLC25A27 LPL CYP7A1 POLD1 ABCG1 ACOX2 MYLIP NR1H2 DGAT1 DGAT2 NPC1 GCDH IRS2 FABP3 APOC2 INS ACADL GPAM PLSCR3 FBXW7 ABCG8 ACOXL NUS1 CD24 ABCG5 PCSK9 NR1H4 PNPLA3 CETP PNPLA1 ACACA EHD1 PRKAA1 APOA5 SOAT2 LAMTOR1 LIPC ASGR2 ACSM3 ANGPTL3 EPHX2 ANGPTL4 APOA1 FABP4 APOC3 IVD LDLRAP1 C10orf112 C1QTNF3 ACAD8 LRP5 PKP2 PPARG NPC2 SOAT1 ACOX3 PNPLA5 PRKAA2 NR1D2 AKR1C1 LCAT COL4A3BP ACAD11 ACADS HNF4A CAV1 MED13 SCARB1 PNPLA4 USF1 XBP1 GIP RORA GCKR NR5A2 PLA2G10 MLXIPL APOA2 IL18 ORMDL3 LIPG AMPD2 APOM

GO_L_ASCORBIC_ACID_METABOLIC_PROCESS The chemical reactions and pathways involving L-ascorbic acid, (2R)-2-[(1S)-1,2-dihydroxyethyl]-4-hydroxy-5-oxo-2,5-dihydrofuran-3-olate; L-ascorbic acid is vitamin C and has co-factor and anti-oxidant activities in many species. CYB5R3 GSTO2 CYB5A GCLC SLC23A2 SLC2A3 RGN SLC2A1 AKR1A1 GSTO1 SLC23A1

GO_PURINE_DEOXYRIBONUCLEOTIDE_METABOLIC_PROCESS The chemical reactions and pathways involving purine deoxyribonucleotide, a compound consisting of deoxyribonucleoside (a purine base linked to a deoxyribose sugar) esterified with a phosphate group at either the 3' or 5'-hydroxyl group of the sugar. AKD1 DGUOK NUDT15 NUDT16 SAMHD1 GUK1 ADA NUDT18 ADK NUDT1 AK5

GO_REGULATION_OF_MESONEPHROS_DEVELOPMENT Any process that modulates the rate, frequency or extent of mesonephros development. Mesonephros development is the process whose specific outcome is the progression of the mesonephros over time, from its formation to the mature structure. The mesonephros is an endocrine and metabolic organ that filters the blood and excretes the end products of body metabolism in the form of urine. SOX9 GATA3 GREM1 BMP4 NOG HOXB7 SIX2 AGTR2 SMO AGT PAX2 LGR4 SOX8 WNT2B WT1 PAX8 SIX1 BASP1 MAGED1 VEGFA GDNF SIX4 TGFB1 LHX1 HNF1B TACSTD2

GO_NOTOCHORD_DEVELOPMENT The process whose specific outcome is the progression of the notochord over time, from its formation to the mature structure. The notochord is a mesoderm-derived structure located ventral of the developing nerve cord. In vertebrates, the notochord serves as a core around which other mesodermal cells form the vertebrae. In the most primitive chordates, which lack vertebrae, the notochord persists as a substitute for a vertebral column. COBL T YAP1 GLI1 GLI2 EPHA2 COL2A1 EFNA1 CRB2 WNT11 SOX9 WNT5A NOG GDF3 KDM6A ID3 STIL TEAD2 NOTO

GO_REGULATION_OF_PROTEIN_MODIFICATION_BY_SMALL_PROTEIN_CONJUGATION_OR_REMOVAL Any process that modulates the frequency, rate or extent of protein modification by small protein conjugation or removal. DNAJA1 NDNL2 SPOPL UBE2S ARRB2 PIAS1 USP4 ARRDC4 ZER1 TRIB3 USP44 CDC14B SIAH2 PSMD13 UBE2L3 SASH1 DCUN1D4 EGF CTNNB1 FAM123B MTOR PSMB4 ARRDC3 PSMB7 PTK2 PSMB9 RNF222 DCUN1D1 TRIM67 HSPA1A PER2 PSME3 MALT1 CTR9 PSMB6 GCLC PIN1 FBXO5 KDM1A GNL3 HMG20B PARK2 PML HSP90AB1 FKBP1A PSMA5 UBE3A PSMD14 TSPO TBC1D7 PSMA2 ARRB1 SOX4 ANAPC11 APITD1 FYN TICAM1 STUB1 UBE2I UBA52 FBXW7 SKP1 NDFIP2 RNF180 CUL1 TRAF6 CDC20 WNT1 DCUN1D2 BUB1B PSMB10 CDK9 FBXO43 MAGEA2B NHLRC1 CDC27 PSMC4 COMMD1 CCNB1 PSMD9 NMI OTUB1 ANAPC2 TGFBR1 CAPN3 CAV1 TRIP12 FBXO22 DCUN1D3 PSEN1 PSMC5 MAD2L1 PSMB3 ANAPC16 FEM1B LIMK1 PLK1 CRY1 DAXX UFL1 KBTBD5 CHFR XIAP UBE2C CAV3 PSME4 HERPUD1 TSPYL5 ANAPC1 CDC14C MAD2L2 DISC1 SUFU SART3 PIAS3 TOPORS PRMT3 UBE2D1 C11orf51 HSP90AA1 FBXO4 VCP WASH1 ISG15 SMURF1 WDR48 SKP2 ANGPT1 PSMD4 BCL10 RPS27A HSPBP1 AIMP2 CDK2 CDC23 HSPA1B TRIM21 GTPBP4 ARNT PIAS4 PSMD11 PRICKLE1 RCHY1 FBXO2 PSMF1 ADRB2 PSMB8 MUL1 PSMC3 HSPA5 PSMC1 PELI1 RWDD3 NXN PSMD5 HFE SMAD7 PSMB2 RNF4 PSMD8 UBQLN1 UBE2N BUB3 FZR1 PSMC2 CHP GNL3L CDC20B PTEN PRKCE BMI1 PTK2B VPS28 WFS1 PSMA8 ANAPC5 PSME2 STRA13 UBXN2A TANK ABL1 BIRC3 ZYG11B BIRC2 BAG5 TNFAIP3 RNF20 RNF139 CDC26 PINX1 PRKCG PSMA1 RPS3 PSME1 CDC16 PSMD12 ANAPC7 PSMC6 CCDC22 CDK5RAP3 PTPN22 SEPT4 MAGEA2 RASD2 RIPK2 PSMD10 PSMB11 CCDC23 PSMB5 C1orf124 BRCA1 SENP2 CDK1 MAGEC2 PSMD3 PSMA3 HDAC4 BTRC ANAPC10 PARP10 TRIM39 PINK1 PSMA6 PDCL3 PTTG1IP ANAPC4 CDK5 EGR1 UBR5 MTA1 UBXN1 PSMD1 LRRK2 AXIN1 SPHK1 RNF111 DCUN1D5 HDAC8 DERL1 PSMD6 NDFIP1 CLU ZYG11A PSMA4 PSMA7 PSMB1 UBE2E1 PSMD7 RASSF5 PARK7 OGT PHF23 GLMN UBC PSMD2 ZC3H12A UBB FEM1A N4BP1 ITCH AVPR2 RNF40 FANCI TOLLIP PAXIP1

GO_SPERMATID_NUCLEUS_DIFFERENTIATION The specialization of the spermatid nucleus during the development of a spermatid into a mature male gamete competent for fertilization. SYCP3 AGFG1 HMGB2 SYCP1 PSME4 DMRTC2 TMF1 PVRL2 TSSK6 CHD5 TBPL1 PYGO2 GOPC KDM3A HIST1H2BA PYGO1 TNP1 H1FNT SRPK1

GO_MYELOID_CELL_DEVELOPMENT The process whose specific outcome is the progression of a myeloid cell over time, from its formation to the mature structure. SOX6 G6PD C6orf25 ANXA2 LRRK1 HBZ SRC TSPAN2 ABI1 MAEA L3MBTL3 EPO ZFPM1 PTPN11 WASF2 FLI1 ROD1 FAM20C SRF TMOD3 KIT MEIS1 RHAG ARID4A MED1 GATA1 ERCC2 KLF2 PIP4K2A VPS33B EPB49 TAL1 ZNF385A JMJD6 RPS6 PAFAH1B1 PTPN6 SLC11A2 FOXP1 BPGM BCL6 EP300 FLVCR1 NCKAP1L EPB42

GO_MICROGLIAL_CELL_ACTIVATION The change in morphology and behavior of a microglial cell resulting from exposure to a cytokine, chemokine, cellular ligand, or soluble factor. IL4 TLR7 TLR2 TLR8 CLU IL13 TLR6 IL33 JUN AIF1 CX3CR1 AZU1 TLR3 SNCA TRPV1

GO_NEGATIVE_REGULATION_OF_PEPTIDASE_ACTIVITY Any process that stops or reduces the rate of peptidase activity, the hydrolysis of peptide bonds within proteins. RPS6KA3 APP PRDX3 SPINT4 IL6 SPINK1 UMODL1 SPINT1 CST2 CD109 CPAMD8 ITIH1 SERPINB5 SERPINE1 BIRC8 MKL1 GAS6 PLAUR SPINT3 PEBP1 SPOCK3 THBS1 KAL1 CST6 CD27 SERPINB9 NGFRAP1 ANXA8 CST4 GPI CSNK2A1 WFDC13 MDM2 SPINK7 KNG1 CASP8 SPINK4 SIAH2 ITIH3 WNT9A RPS6KA1 PSMF1 AQP1 SERPINB8 MT3 PCSK1N LXN A2M SERPINB1 CST9LP1 SERPINF1 SERPINA5 CST1 SERPINB12 NLE1 SPINK2 TIMP2 WFDC2 ARRB2 BIRC5 SERPINI1 CARD18 SERPINI2 CDKN2D APLP2 CAST POR IFI6 AVP PAX2 SERPINA7 PTTG3P HRG WFDC12 FABP1 DPEP1 CSTB SERPING1 LEF1 ITIH6 PI16 CRYAB SFRP2 GPC3 AGT TRIAP1 BIRC2 LAMP3 ARRB1 SERPINE2 WFDC10A CST9 C4A AMBP VTN BIRC3 SPINK8 SERPINA6 SPINK6 R3HDML CST8 MAP2K5 SERPINE3 UCHL5 BIRC7 SERPINB4 PICALM ITIH5 CARD16 SERPINA11 SSPO CARD17 C5 TIMP4 COL6A3 SERPINA1 TIMP3 CR1 SERPINB13 USP47 SNCA FNIP1 RAG1 WFIKKN2 SERPINF2 UBXN1 CST5 ARL6IP1 SFN SPOCK2 NGF MMP9 SERPINB7 SPINK13 SERPINB2 CD44 RENBP PI15 WFDC1 ITIH4 SPOCK1 MICAL1 TNFSF14 PTTG1 PROS1 SERPINB10 NAIP CSTA ECM1 SERPINA2 PROL1 TFAP2B C3P1 WFDC6 AHSG SH3RF1 CST9L PZP BST2 PRDX5 SPP2 IFI16 LPA TFPI SERPINB6 WFDC10B SERPINC1 HMSD WFDC5 SERPINA10 COL4A3 WFDC8 LCN1 A2ML1 SLPI IGBP1 TIMP1 SERPINB11 SPINLW1 USP14 C4B CRB2 PAK2 SORL1 CST11 NGFR CRIM1 NOL3 RNF34 SRC SERPIND1 SERPINA4 MAGEA3 ITIH2 SERPINA12 PTTG2 FURIN RECK PI3 SPINK14 PCID2 SPINK9 PAPLN FETUB SERPINA9 WFDC3 OVOS2 YWHAE SPINT2 VEGFA CSTL1 HBXIP RAF1 VIL1 NLRC4 RFFL AKT1 OVOS SERPINH1 COL7A1 BCL2L12 SPINK5 DDX3X COL28A1 TNFAIP8 PARK7 TFPI2 DHCR24 HGF HERPUD1 KLF4 PIH1D1 SERPINB3 C3 BIRC6 XIAP WFIKKN1 SERPINA3 GPX1 CST7 DNAJB6 CST3

GO_POSITIVE_REGULATION_OF_ASTROCYTE_DIFFERENTIATION Any process that activates or increases the frequency, rate or extent of astrocyte differentiation. SERPINE2 ID2 HES1 BIN1 CLCF1 MAG IL6ST PRPF19 NOTCH1 LIF BMP2

GO_MEMBRANE_DISASSEMBLY The controlled breakdown of any cell membrane in the context of a normal process such as autophagy. LMNA NUP88 RANBP2 NUP214 TPR BANF1 NUP155 PRKCA POM121C NUP43 EMD VRK1 NUP85 NUP133 NUPL1 NUP62 NUP205 NEK6 NEK9 NUP153 PLK1 SEH1L CTDNEP1 RAE1 LPIN1 NUP188 PRKCB CCNB2 NUP210 NUP93 NUP37 PAFAH1B1 TMEM188 NDEL1 AKAP8L NUP107 AAAS NUPL2 NUP54 TMEM48 NUP50 POM121 CCNB1 CDK1 NUP98 NUP35 NUP160

GO_LACTATION The secretion of milk by the mammary gland. DHODH XDH PPAT CSN2 HIF1A CAD ATP7B OXTR HK2 GHRHR PRLR DDR1 CAV1 NCOA1 UPRT PAM CCND1 MED1 SLC29A1 APLN RPLP0 VEGFA NEURL ATP7A CSN3 ERBB4 APRT AGPAT6 USF2 CDO1 SERPINC1 SLC6A3 PRL CREB1 FOXB1 UMPS VDR STAT5A ATP2B2 STAT5B

GO_RESPONSE_TO_MANGANESE_ION Any process that results in a change in state or activity of a cell or an organism (in terms of movement, secretion, enzyme production, gene expression, etc.) as a result of a manganese ion stimulus. ATF4 PTGS2 ATP13A2 D2HGDH LRRK2 SLFN14 TH EIF2S1 ARG1 EIF2AK3 ADAM9 HSPA5 TSPO PARK2

GO_NEGATIVE_REGULATION_OF_CANONICAL_WNT_SIGNALING_PATHWAY Any process that decreases the rate, frequency, or extent of the Wnt signaling pathway through beta-catenin, the series of molecular signals initiated by binding of a Wnt protein to a frizzled family receptor on the surface of the target cell, followed by propagation of the signal via beta-catenin, and ending with a change in transcription of target genes. PSME4 PSMA4 IGFBP1 PSMB1 PSMA7 FOXO3 NKD1 DDIT3 MAD2L2 LATS2 CTHRC1 PSMD6 CSNK1A1 DKK1 UBC MESP1 LIMD1 PSMD2 UBB PSMD7 GSK3A DACT3 GSK3B TCF7L2 MAPK14 SHH FUZ BMP2 KLHL12 GLI3 SOST ZNRF3 PSMD4 HECW1 LRP6 NKD2 PSMB10 IGFBP4 PSMD10 PSMB11 DKK2 INVS IGFBP2 PSMB5 HDAC1 AES SNAI2 PSMD3 WNT11 PSMA3 IGFBP6 PSMD9 DVL3 TMEM88 STK4 PSMC4 DAB2IP PSMA6 AXIN2 PSMB3 FZD6 NPHP4 WNT5B DVL1 PFDN5 CAV1 SCYL2 PSMC5 DKK3 PSMD1 AXIN1 APC2 LATS1 NKX2-5 LRP4 FAM123A EGR1 PHF17 RAPGEF1 PTPRO MCC PSMB6 MLLT3 CDH2 SFRP5 DKK4 PSME3 ANKRD6 WNT5A SOX9 STK3 PSMA8 TLE2 PSME2 APOE PSMA5 TMEM64 CYLD PARK2 DAB2 SFRP2 SFRP1 GPC3 PSMD14 SOSTDC1 KIAA0922 PSMA2 RGS19 LZTS2 UBA52 PSMD12 G3BP1 NOTCH1 APC PSMC6 LEF1 PSMA1 PSME1 RBX1 SOX17 WWTR1 TBX18 DACT1 FRZB NOTUM RPS27A NOG KREMEN2 RGS20 FZD1 SFRP4 SIAH2 DVL2 CHD8 ISL1 PSMB8 PSMD13 PSMD11 PRICKLE1 BICC1 PSMF1 FAM123B CTNND1 SOX10 PSMB4 NPHP3 PSMB7 PSMB2 PSMB9 C1orf187 PSMC3 PSMC1 ROR2 CUL3 PSMD5 SOX2 GREM1 WNT4 PSMC2 SDHAF2 PSMD8 GLI1 FOXO1

GO_MRNA_CLEAVAGE Any process in which a pre-mRNA or mRNA molecule is cleaved at specific sites or in a regulated manner. TUT1 EIF2C3 CPSF4L RNASE4 CLP1 CSTF1 CSTF2T POP4 CPSF1 EIF2C4 EIF2C1 MOV10 CSTF2 CPSF4 CPSF2 ERN1 NCBP2 NCBP1 WDR33 CPSF3 PCF11 EIF2C2 CSTF3

GO_REGULATION_OF_CHOLESTEROL_HOMEOSTASIS Any process that modulates the frequency, rate or extent of cholesterol homeostasis. NR1H4 FGFR4 RORA LDLR NR1D1 SEC24A NR1H3 SREBF2 RALY NPC1 HNF4A NR1H2

GO_POSITIVE_REGULATION_OF_ORGANELLE_ASSEMBLY Any process that activates or increases the frequency, rate or extent of organelle assembly. CNOT2 FSCN1 STX18 BBS4 SDCBP RAB3GAP1 SDC1 SDC4 PDCD6IP MYLK3 CENPJ WRAP73 PAN3 SEPT9 LRSAM1 MSN KIAA1324 PIP4K2A IL5 HTT VPS4B PIP4K2B EDN1 CEP120 RALB CNOT6 KCTD17 SH3GLB1 SRC CNOT6L KIAA1731 RAB3GAP2 SEPT7 PAN2 MAPK9 PLK4 HAP1 ARHGEF5 FUZ ARHGAP35 LCP1 BMP10 CNOT1 CEP135 MNS1 CSF2 PROX1 CAPG CCP110 FAM154A TNF PIP4K2C TAPT1

GO_ACETYL_COA_BIOSYNTHETIC_PROCESS The chemical reactions and pathways resulting in the formation of acetyl-CoA, a derivative of coenzyme A in which the sulfhydryl group is acetylated. PDHA2 ACAT1 ACSS2 PDHX DLD ACLY BRP44L DLAT PDHB MLYCD BRP44 PDHA1 ACSS1

GO_NUCLEOTIDE_EXCISION_REPAIR_DNA_INCISION A process that results in the endonucleolytic cleavage of the damaged strand of DNA. The incision occurs at the junction of single-stranded DNA and double-stranded DNA that is formed when the DNA duplex is unwound. RPS27A RPA3 RFC2 GTF2H4 PCNA RPA1 GTF2H3 DDB2 RFC3 FAN1 CUL4A UBC ERCC2 ERCC1 UBB RFC4 NTHL1 DDB1 CHD1L POLD1 POLK PARP1 OGG1 RFC5 POLD3 POLD4 GTF2H2 ERCC5 ERCC4 GTF2H5 RBX1 RFC1 UBA52 XPA ERCC3 GTF2H1 RPA2 CUL4B BIVM POLD2

GO_POSITIVE_REGULATION_OF_CHROMOSOME_SEGREGATION Any process that activates or increases the frequency, rate or extent of chromosome segregation, the process in which genetic material, in the form of chromosomes, is organized and then physically separated and apportioned to two or more sets. H2AFY SMC6 RCC2 SIRT2 ANKRD32 CCNB1 CDC6 TTC15 SMC5 RB1 DLGAP5 RAD18 FAM178A SFPQ CUL3 BECN1 ANAPC11 NSMCE2 ESPL1
[truncated: 3,555,042 more chars]
