# Supplementary material for: Exploring autophagy with Gene Ontology
Source: Autophagy. 2018 Feb 17;14(3):419–36. doi: 10.1080/15548627.2017.1415189 (PMC5915032; doi:10.1080/15548627.2017.1415189)
Supplement: supp_data_1415189.zip [file kaup-14-03-1415189-s001.zip › supp_data_1415189/supp_data_1415189_s02.docx]

**File S2.** Cellular component GO gene set used to perform Gene Set Enrichment Analysis. Analysis was performed as detailed in Materials and Methods.

GO_PRERIBOSOME Any complex of pre-rRNAs, ribosomal proteins, and associated proteins formed during ribosome biogenesis. RRP15 RPLP0 RRP9 RSL1D1 NOC4L WDR36 NOL6 NOB1 RPLP2 FCF1 RRP36 NOC2L BOP1 UTP20 SRFBP1 UTP11L TEX10 WDR12 NIP7 LTV1 RRP7B PWP2 NHP2L1 PES1 DIEXF NOP14 IMP4 WDR74 RRP7A RRP1B RPS7 AAMP NGDN FBL PPAN MRTO4 TSR1 NOP58 WDR46 NOP56 EBNA1BP2 UTP6 KRR1 MAK16 TBL3 PDCD11 BMS1 ZNF622 DCAF13 UTP18 UTP3 NSA2 EIF6 RPF1 RRS1 HEATR1 RRP1 RPLP0P6 MPHOSPH10 UTP14C EMG1 LAS1L MDN1 FTSJ3 IMP3 UTP14A WDR37 FBLL1 RPLP1 RPSA UTP23 CCDC86 WDR3

GO_NADH_DEHYDROGENASE_COMPLEX An integral membrane complex that possesses NADH oxidoreductase activity. The complex is one of the components of the electron transport chain. It catalyzes the transfer of a pair of electrons from NADH to a quinone. NDUFAB1 NDUFB6 NDUFB4 NDUFS8 PARK7 NDUFA6 NDUFV1 NDUFA8 NDUFB1 NDUFV3 NDUFA10 NDUFB10 FOXRED1 NDUFA5 NDUFA2 NDUFS1 NDUFC2 SNCA NDUFB8 NDUFA7 NDUFS2 NDUFB9 NDUFS7 NDUFA9 NDUFA13 WDR93 NDUFB11 NDUFAF1 NDUFV2 NDUFS5 NDUFS4 NDUFS6 NDUFA3 NDUFS3 NDUFA11 NDUFB7 NDUFC1 NDUFA12 NDUFA4 NDUFB3 NDUFB5 NDUFA1 NDUFB2

GO_MHC_CLASS_II_PROTEIN_COMPLEX A transmembrane protein complex composed of an MHC class II alpha and MHC class II beta chain, and with or without a bound peptide or polysaccharide antigen. HLA-DRB1 HLA-DQB2 HLA-DRB3 HLA-DQA2 CD74 HLA-DPA1 HLA-DQB1 HLA-DOA HLA-DMA HLA-DRA HLA-DPB1 HLA-DOB HLA-DRB4 HLA-DQA1 HLA-DMB HLA-DRB5

GO_STAGA_COMPLEX A large multiprotein complex that possesses histone acetyltransferase and is involved in regulation of transcription. The composition is similar to that of the SAGA complex; for example, the human complex contains the transcription-transformation cofactor TRRAP, hGCN5L acetylase, novel human ADA-like and SPT-like cofactors, and a subset of TAFs. ATXN7 TADA1 TAF10 TAF12 KAT2A TADA3 SAP130 TAF9 TADA2B TAF5L TRRAP SUPT7L TAF6L SUPT3H

GO_NODE_OF_RANVIER An axon part that is a gap in the myelin where voltage-gated sodium channels cluster and saltatory conduction is executed. SPOCK1 SCN8A KCNQ2 KCNQ3 CNTN2 SCN1A MYOC DAG1 BIN1 NFASC DLG1 ANK3 SPTBN4 SCN2A SCN1B

GO_MICROTUBULE Any of the long, generally straight, hollow tubes of internal diameter 12-15 nm and external diameter 24 nm found in a wide variety of eukaryotic cells; each consists (usually) of 13 protofilaments of polymeric tubulin, staggered in such a manner that the tubulin monomers are arranged in a helical pattern on the microtubular surface, and with the alpha/beta axes of the tubulin subunits parallel to the long axis of the tubule; exist in equilibrium with pool of tubulin monomers and can be rapidly assembled or disassembled in response to physiological stimuli; concerned with force generation, e.g. in the spindle. ARHGAP4 SKA3 MTUS1 TUBGCP3 DYNC1I2 PBXIP1 GRAMD3 KATNB1 TTLL11 KIF6 KLC4 KIF3C MTA1 FAM110C GABARAPL3 FEZ1 EIF3A KIF17 ARFGEF2 TTLL4 EML2 TUBB4A TUBB4B APC2 AXIN1 NEK2 SRPRB KIF4A CDK5RAP3 KIF2B C17orf28 C15orf23 C19orf20 CDK5RAP2 TUBG2 TUBGCP5 TRPV4 KATNAL1 TUBB6 CDK1 KIF21B MAP4 DYNC1I1 PCNT MAP1B SPAG6 WDR47 DNAI2 DCTN2 TPPP3 KIF2C ZNF207 TUBB2B EMD KATNAL2 MAP1A KIF14 DNAL4 MAPT CEP57 CCT5 TUBB2A GTSE1 RAB11A MAPRE3 RGS14 C6orf165 HDAC6 DNAH6 SCTR KIF15 ARL6 TUBA3D TRIM55 DYNLRB2 DNAH1 CAMSAP2 TIAM1 TMEM214 KIF13A KLC1 DNAH10 ACTR1A CCT7 INO80 KIF27 TUBA8 DNAH3 TUBB8 RASSF5 JAKMIP1 HOOK2 WHAMM CALM2 NUMA1 STIM1 CENPJ TTLL9 CUL3 HAUS8 KIFAP3 NCKAP5L BCAS3 DNAH2 TUBA1C BICD1 TUBB3 BIRC8 SHROOM3 MAP9 WDR43 KIAA1383 BIRC5 RGS20 DCXR KIF16B TUBA3E KIF5B LRPPRC RCC2 FAM82A2 LRRC49 NAV1 MT3 CENPE CSNK1D TUBG1 KIF24 DLG1 TTLL7 MAP6D1 TPPP DYNC1LI1 APPBP2 DNM1L BIRC2 BIRC3 CLASP1 TPX2 KIF4B RUSC1 KIF5C TUBAL3 TUBA4B HOOK3 SLC8A3 SPAG17 ODF3L2 SIRT2 DYNLRB1 KIF25 HAUS7 AURKA CYP2A6 APC NDE1 ZW10 EML4 KIF26B CDC16 KIF9 KIFC2 TEKT1 SNPH TBCB REEP2 CSPP1 DYNLT3 TTLL13 CCT8 SKA1 FSD1 MAP7 DNM1 MID2 DYNLT1 KIFC1 PAFAH1B1 MAP6 SPAST CCDC50 NUDC KIF12 NDRG1 CEP57L1 BIRC7 DNAH12 KRIT1 CEP170 HAUS2 KIF18B NINL KIF1B TBCC SVIL ARHGEF2 KIFC3 FKBP4 DNAH5 STAU2 ASPM DVL1 REEP4 FAM175B POLB MEFV TUBE1 DNAH11 DYNC2H1 AXIN2 KIF3B PLK1 HDAC3 CKAP2 CLTC MAP1LC3A NEK6 CCT6A TRIM54 IGBP1 TBL1X KIAA1009 INVS HAUS6 CCT4 CDC27 TTLL3 TCTE3 RACGAP1 INCENP NUSAP1 CAMSAP3 HOOK1 DYNLL2 ACTR1B WDR81 RAB3D TUBD1 MAP1LC3B DCTN1 RADIL PARP4 TUBGCP6 KNTC1 NICN1 DYNC1LI2 FAM179B TTLL8 REEP3 MAP1LC3B2 C16orf48 TBCD KIF18A CALM1 TEKT2 KLHL21 RP1 BCL10 SHROOM1 KIF19 DNAH7 CRHBP CAMSAP1 C9orf9 XIAP DYNLL1 CCT2 KIF5A TUBA4A PTPN20B SHROOM2 KLC2 AURKC C1orf96 RP1L1 KIF20B MID1 C18orf10 SPAG5 SLC8A1 DISC1 HAUS4 EML5 SKA2 GAS8 NLRC4 AURKB CLMP SNTB2 CLIP2 CALM3 TUBA3C NCOR1 C16orf80 ARL3 HSPH1 KIF23 MTUS2 RASSF1 MID1IP1 KIF1A TRIM63 MAPRE1 TUBB1 TBL1XR1 KLC3 CCT3 KIF1C GOLGA2 KIF13B CAPN6 KATNA1 FAM82A1 KCNAB2 DNAH17 TEKT3 MAP1S NDEL1 TUBB KIF3A KLHL22 KIF2A RASSF3 DYNC1H1 DNM2 MDM1 EML3 CLASP2 TTLL5 MAPRE2 KIF20A FGF13 DNAH8 KIF26A SARM1 GABARAPL1 KIF22 DNAH14 RGS19 TUBA1A DNAH9 NEK7 TCP11L1 TUBGCP2 KIAA0284 CKAP5 ODF2 MAP3K11 FIGN KIF21A TBCA SELS FAM154A TCP1 TUBGCP4 DCLK2 FAM82B SYBU HAUS1 MACF1 TUBA1B GAS2L3 SERP1 EML1 HAUS3 EML6 TBCE LZTS2 TTLL1 STMN1 IQGAP1 NIN MAP2K2 DST NEIL2 DNAI1 TEKT4 CLIP1 DNM3 MAP2 DCX MYO5A PSRC1 PRC1 TTLL6 SYNJ1 SPRY2 SLAIN2 CYLD SAA1 SS18 ATAT1 MAP1LC3C DPYSL2 HAUS5 DYNC2LI1 SEPT9 KIF11

GO_I_BAND A region of a sarcomere that appears as a light band on each side of the Z disc, comprising a region of the sarcomere where thin (actin) filaments are not overlapped by thick (myosin) filaments; contains actin, troponin, and tropomyosin; each sarcomere includes half of an I band at each end. FKBP1B FKBP1A SCN5A FBXL22 SLC8A1 MYOZ2 PDE4B PPP3CB CASQ1 HOMER1 ATP2B4 HRC KY CAV3 PPP2R5A BAG3 DNAJB6 CACNA1D KBTBD5 LDB3 SMN1 DST FRG1 NOS1 SMTNL1 DMD MTM1 PPP1R12A STUB1 PSEN2 ACTN1 SYNE2 ANK1 SYNPO2L XIRP2 KRT8 CFL2 KCNN2 ACTN4 ADRA1A FERMT2 PARVB PPP1R12B PPP3CA KCNA5 FLNC ANKRD2 NEB PGM5 BMP10 AHNAK2 ALDOA MYL3 FHL3 RYR1 CRYAB FHL2 SMN2 SCN1A OBSCN MYL9 MURC KRT19 FBXO32 KAT2B KCNE1 AKAP4 SLC4A1 CACNA1S TCAP RYR2 JPH1 HSPB1 SCN3B CASQ2 CSRP3 HDAC4 MYZAP TTN FLNB NEBL RYR3 MYH7 ANKRD1 S100A1 MYL12B SRI FHOD3 TRIM54 SORBS2 BIN1 IGFN1 MYOZ3 SYNC MYH6 NEXN ITGB1BP2 SYNPO2 ANK3 MYPN CACNA1C JPH2 MYOZ1 SCN8A PAK1 DES FBP2 CTNNB1 ACTN2 FBXO22 CAPN3 ACTC1 NOS1AP ANK2 GLRX3 PDLIM3 OBSL1 SYNPO TRIM63 MYOT PALLD JUP PARVA

GO_NUCLEAR_REPLICATION_FORK The Y-shaped region of a nuclear replicating DNA molecule, resulting from the separation of the DNA strands and in which the synthesis of new strands takes place. Also includes associated protein complexes. TIPIN ZRANB3 RPA3 BCAS2 TOP1 CDC5L MCM10 POLA1 RPA4 PCNA CDC45 RPA1 TONSL PLRG1 SMARCAD1 TOP1MT SMARCAL1 PURA POLE POLA2 PRPF19 HELB CHRAC1 MCM3 MMS22L ZMIZ2 POLD1 POLD3 POLE2 POLD4 SMARCA5 GINS2 ERCC5 PURB BAZ1B XPA GINS4 RPA2 POLE3 POLE4

GO_CONDENSED_CHROMOSOME A highly compacted molecule of DNA and associated proteins resulting in a cytologically distinct structure. SPC25 RNF212 TEX11 NEK2 TOPBP1 SMC5 CENPO CASC5 RAD9A CCDC99 PPP1CC AHCTF1 CENPW PLK1 CENPN SMC3 NUF2 LEPREL4 SMC4 ANAPC16 HMGB2 DDX11 MAD2L1 SYCP2 SKA3 MLF1IP KIAA1267 SUN2 KIF2C CCNB1 CDX2 LIG4 WAPAL BRCA1 RAD51C INCENP H2AFY MLH3 DCTN6 DYNC1I1 CCDC75 NUP37 HJURP SYCE1L BUB1B SYCP3 SS18L1 ORC2 ZWINT PMS2P3 CBX3 NCAPG MSH5 KIF2B RAD51 C15orf23 HORMAD1 SUV420H1 SPC24 CENPM DCTN5 EIF2C3 CENPT RCC1 TP53BP1 PMF1 BANF1 NSMCE2 KNTC1 ZNF207 TOP2A AURKB RAD9B SMC1A DSN1 SKA2 L3MBTL1 SPAG5 RRS1 SEPT2 CENPH EID3 CHMP1A NSMCE1 SUV420H2 LOC728637 STAG3 AURKC CSNK1A1 NUP107 SUV39H1 FANCD2 XRCC4 NUDCD2 SYCE3 BOD1P FBXO28 MKI67 BUB3 HUS1B SEH1L BAZ1B ITGB3BP NCAPD2 CDCA5 SYN1 CFDP1 SMC2 CENPK KIFAP3 PHF2 MIS18BP1 SYCE1 NSL1 MLH1 CTCF CENPA NUP43 CENPV NCAPD3 DYNC1LI1 ZWILCH LRWD1 MAD1L1 CENPF NOL6 TTN NDC80 CENPE TUBG1 HORMAD2 AKAP8 SGOL2 CLASP2 RAD21L1 DCTN3 SYCP1 LRPPRC TEX12 BUB1 REC8 NDNL2 PES1 BIRC5 NCAPH CDK2 FKBP6 BOD1 RANGAP1 NDEL1 SEPT7 ZNF276 UBE2I NUP85 NUP133 AURKA CHAMP1 CEBPB NDE1 ZW10 RGS12 PINX1 RAD50 SMARCA5 SMC6 ADD3 PHF6 HUS1 H2AFX SETMAR SGOL1 RAD21 NSMCE4A APITD1 MKI67IP BRCA2 TOP3B CLASP1 SMC1B BRD4 DMC1 STRA13 C9orf114 ERCC6L RASSF2 RSPH1 MSH4 SYCE2 SEPT6 PMS1 CENPC1 CHEK1 CCNB1IP1 HMGB1 SKA1 CCDC155 RAD1 MIS12 MEAF6 BLM HSPA2 TEX14 DYNLT3

GO_HISTONE_DEACETYLASE_COMPLEX A protein complex that possesses histone deacetylase activity. HDAC11 SALL2 TBL1X TAF6L APPL1 HEY2 SAP130 ING2 HDAC1 CIR1 TBL1Y PHF21A HDAC9 BRMS1 PHF12 CHD5 SAP30 GATAD2B BRMS1L APPL2 MBD3 HDAC4 MBD2 C14orf43 SIN3A HDAC5 NACC2 SIN3B ZNF541 TAL1 TRERF1 SATB2 MORF4L1 MECOM CHD3 FAM60A NRIP1 RBBP7 CBX5 SUDS3 HDAC10 ZNF217 SALL1 NCOR1 RERE SAP18 CSNK2A1 DNTTIP1 HDAC2 HDAC8 HDAC6 HINT1 RBBP4 TBL1XR1 HDAC3 GATAD2A CHD4 NCOR2 HDAC7 MTA2

GO_INTRINSIC_COMPONENT_OF_MITOCHONDRIAL_OUTER_MEMBRANE The component of the mitochondrial outer membrane consisting of the gene products and protein complexes having either part of their peptide sequence embedded in the hydrophobic region of the membrane or some other covalently attached group such as a GPI anchor that is similarly embedded in the membrane. TOMM22 PINK1 TOMM7 FUNDC1 TOMM20 TOMM20L FUNDC2 MFN2 C4orf49 BNIP3 BAK1 TOMM40 ABCB6 FIS1 MUL1 RHOT2 ARMCX3 SYNJ2BP RHOT1 GDAP1 DMPK CPT1A

GO_PLASMA_MEMBRANE_PROTEIN_COMPLEX Any protein complex that is part of the plasma membrane. CACNA1H KCNC3 CACNA1G ITGA3 PDE4B AP2M1 SNTB2 CAV3 SGCD ABCA2 CACNG4 KCNK6 GNA13 CATSPERB GRID2 KCNF1 KRT8 APBB1IP CALM1 TRIP6 ITGAD CHUK ITGA10 BCL10 ATP1A2 BMP2 SACM1L PGM5 SHISA7 CACNA2D2 CLTA FAF1 KCNIP1 HLA-DPA1 LYN CHRNB4 GABBR2 GRIN2D TRAT1 HLA-DRB1 GNG4 FLOT2 KCNJ16 KCNE2 KCNJ4 HLA-DRB4 TRAF6 ITGAV KCNMB4 CHRNA4 HLA-DOA SUMO1 CFLAR KCNE1L C8A ITGB4 CACNA2D4 SCN10A HLA-DQB2 KCNB2 CLTC SCN9A CATSPER2 SORBS1 B2M ZAP70 GRIN3B JUP SKAP1 CASP3 KCNQ3 SSPN LIN7C GJA4 GNG13 PICALM CD247 SYNJ1 GNG3 NEO1 GJA3 ABCB6 ITGA11 GRIN2A NOX1 GJB6 HLA-C HLA-E SGIP1 NLGN1 C1orf101 FLOT1 GNGT1 CPT1C CHRNA1 SGCZ CHRNA2 CHRNA3 KCNIP4 ITGB5 CACNB4 CACNG1 GJB2 RGS19 KCNJ11 ATP1A1 KRT19 PORCN KCNV2 GRIA3 TFR2 CHRNA5 CASP8 ITGB7 KCNAB2 TLR6 CHRNB2 TRAF3 KCNA4 ITGA1 GRIK5 ITGB1 NDUFV2 AP2B1 TFRC CACNB3 ABCF2 KCNV1 CD6 CACNG7 KCNS1 AP2S1 CNIH3 CTNNB1 SNTA1 KCNJ8 KCNJ1 GNAI1 ACVR1B CD8A CD3G AP2A2 ATP1A4 ITGAM FXYD2 SNTG2 TBC1D5 KCNA2 CALM2 MYH9 TF KCNAB1 NOS1 GNG10 SGCB TOLLIP HTR3C HTR3D CD40 HLA-DQA1 CNIH2 CHRNA6 HLA-DRB5 GRM1 HLA-DMA COL13A1 KCNE1 KCNH1 VAMP2 GJB4 HLA-DQB1 CTTN SCN11A GRIN3A GRIN2B GJA5 CORO1C MPP7 NCF1 CYBA SLC6A3 CACNA1F KCNA10 GNAI2 LRP4 CD4 KCND1 VCAM1 KCNB1 HTR3E CACNG6 NCF1C SDCBP GRIK4 CHRNG GRIN2C ITGAE RIPK1 KCNQ4 GLRB C7 CHRNA10 GRIA4 CDH2 IL29 HLA-DMB HLA-B ITGA5 CHRNB1 KCNG3 PTK2B PTPN6 NOXA1 FZD8 GNAI3 HLA-F IL10RB APC CYBB FXYD1 SHANK1 TGFBR3 BMPR1A CD79B SCN1B OSMR NCF2 HTR3B CACNA1S STX1A LRP5 DLG1 TRDN KCND3 LDLRAP1 APH1A ENG KCNC4 APH1B CACNA1E DLG4 GJB5 SNTB1 TLR1 STXBP5 TGFBR2 SGCE IL12RB1 NOS1AP ABCC8 HFE2 SMAD7 SYK CATSPER3 GM2A GNA12 WNT3A GJB1 ITGA2B KCNG2 SCN5A IKBKB KCNS2 GJD4 KCNT1 CNTNAP1 ACVR2A SCN7A KCNMA1 IL23R KCNC2 ITGA2 ITGAX HLA-A FAS LRP6 EPS8 KCNA5 ATP1B2 KCNC1 EGFR CD3E CACNA1I SHISA8 GNAO1 ATP1B1 CACNA2D3 FADD KCNH2 SCN2A KCNJ5 GJA10 TRAF2 LRRC26 GNB1 TGFBR1 CACNG5 AMIGO1 GNAZ NCF4 KCNMB2 HLA-DRA IGF1R RGS9 HLA-DOB GJC1 C8G CACNB2 CTNNA1 BMPR1B ATP1A3 ABCA7 CHRND RGS7 PSEN1 NOXO1 DPP6 HLA-DPB1 OLFM3 ABCB8 SGCA CACNG8 KCNH4 KCNK1 KCNIP2 C5 SHANK2 HSPA2 KHDRBS1 GNAT2 SHC1 SNTG1 GRB2 KCNQ5 KCNS3 ITGA7 CACNA2D1 CNTFR KCNG1 ABCD4 GJD2 HLA-DQA2 GNG5 TMEM146 ATP1B3 IL6R ARRB1 CACNB1 IGF1 VTN ITGB2 RNF31 INHA CACNG3 CD3D KCNA7 TSPAN32 ITGA6 GRIK2 GNA15 KCNA6 CHRNA9 ADAM8 CLTB SHISA6 ATP12A TMED10 OLFM2 PDE4D KCNMB3 GJC2 GJA9 GJA1 KCNJ14 NCF1B AKAP9 KCNIP3 IL6 GNA14 CEACAM1 KCNK2 ABCC9 KCNA1 CALM3 ABHD6 KCNU1 CNTN2 PVRL1 C8B ZACN NPNT CDH1 CD79A MAGEE1 DLG2 IRAK1 IRS1 KCNT2 SGCG IL28RA CACNA1D GRIN1 KCNN4 SNAP25 HLA-H SCN4B ITGA4 GRIA1 GNG12 RGS11 GJD3 KCNJ6 EPS15L1 SCN1A NOX3 DLG3 CBL GJB7 TRADD STOML2 KCNN1 MR1 INSR DIABLO TNK2 UTRN ITGB8 CARD11 ACVR1C CACNG2 GJA8 GRIA2 ITGB6 C9 VWC2L GNAT1 CATSPER1 CACNA1C GNGT2 KCNQ2 APC2 CHRNE KCNJ2 KCNE3 CD8B SCN3A HLA-G NOX4 CATSPER4 DAB2 CD74 ITGA9 GJB3 TLR2 GNG11 ITGB3 KCNA3 GNG2 ACVR1 DMD TRAF5 GLRA3 KCNG4 HTR3A KCNJ3 ABCG5 FCER1G SLC18A3 HLA-DRB3 KCNMB1 ABCG8 CASP10 CHRNA7 BIRC2 KCNQ1 SCN2B HCN2 GNG7 FKRP CACNA1B ATP4B GJC3 SHISA9 GABBR1 AP2A1 IL6ST CNTNAP2 ALCAM SCN4A CASQ2 SCN3B CACNA1A MS4A2 VWC2 DAG1 CATSPERG KCND2 C6 PSENEN EPS15 GNAT3 GNG8 ABHD12 HTRA2 CSF2RB CHRNB3 ITGA8 SCN8A RGS6 IL13RA1 ATP1B4 HFE ITGAL

GO_PLATELET_ALPHA_GRANULE A secretory organelle found in blood platelets, which is unique in that it exhibits further compartmentalization and acquires its protein content via two distinct mechanisms: (1) biosynthesis predominantly at the megakaryocyte (MK) level (with some vestigial platelet synthesis) (e.g. platelet factor 4) and (2) endocytosis and pinocytosis at both the MK and circulating platelet levels (e.g. fibrinogen (Fg) and IgG). APP SERPINA1 STXBP1 SRGN SNCA STXBP3 PHACTR2 SERPINF2 ORM2 CYB5R1 CFD SERPINE1 TMX3 VWF GAS6 PPBP THBS1 ACTN2 EGF VEGFB PROS1 TGFB1 KNG1 AHSG ORM1 PCDH7 PLG A2M F5 SERPINA5 FN1 TGFB2 FGG PF4 FGA TIMP1 APLP2 IGF2 VEGFC ACTN4 PDGFB PDGFA HRG ACTN1 SERPING1 A1BG TGFB3 SELP FIGF PECAM1 SERPINE2 IGF1 FGB CD9 ISLR ALDOA ALB VEGFA VPS33B ITGB3 SPARC F8 CD36 ITGA2B LEFTY2 TMSB4X MMRN1 VAMP7 HGF F13A1 THBS2 SERPINA3 QSOX1 CLU

GO_INTRACILIARY_TRANSPORT_PARTICLE_B The larger subcomplex of the intraciliary transport particle; characterized complexes have molecular weights around 550 kDa. IFT27 TRAF3IP1 TRIM59 IFT52 IFT172 RABL5 CLUAP1 IFT88 TTC26 HSPB11 IFT80 IFT57 KIF17 IFT74 TTC30B IFT46 TTC30A IFT81 IFT20

GO_EXTRACELLULAR_MATRIX A structure lying external to one or more cells, which provides structural support for cells or tissues; may be completely external to the cell (as in animals and bacteria) or be part of the cell (as in plants). MFAP1 CMA1 CHADL RPS25 KAL1 ADAMTS1 ADAMTS20 PLSCR1 HIST1H4B ILK EGFL6 SMOC2 MUC5AC ADAMTSL3 NTN3 MMP28 HIST1H4F AMTN CYR61 RPS19 FBLN2 TECTA CD248 TIMP2 COL12A1 MMP14 VIM HSPB1 DLG1 CASK TGFBR3 FGF10 RPL30 PRKDC LMNA GPC3 CD151 SERAC1 COL9A2 PKM2 LAMA5 WISP3 ECM2 CHI3L1 AGRN COL22A1 PTN USH2A ADAMTS14 COL4A2 TNN RPS13 COL10A1 HIST1H4D COL6A6 CCDC80 ADAMTS13 ENTPD2 LAMB3 CHAD WNT7B SOD1 MMP25 MMP8 TPSAB1 SMC3 PXDN EGFLAM COMP SFTPA1 MYL6 DEFA1B SERPINA1 TIMP3 MMP1 COL1A1 EGFL7 EFNA5 TGFBI ADAMTS2 IBSP COL16A1 RBP3 MATN1 HIST1H4J MFAP5 HIST2H4A SOD3 MMP21 SLIT3 B4GALT7 THSD4 AHSG HAPLN3 OMD MMP3 GPC6 TFF3 NTN4 CRISPLD2 S100A9 COL3A1 LOX MGP MMP20 EMILIN2 WNT8B ADAMTS16 ELN DSPP HAPLN4 COL11A2 NDNF ATP5O LEPRE1 COL18A1 DGCR6 EIF4A1 CANX MYH9 THBS2 TOMM20 HMCN2 MMP7 ADAMTSL5 MMP10 EMILIN3 CRISP3 SPARC CRIP2 RPS18 POMZP3 HNRNPM COL4A1 SBSN HPSE SPOCK3 HSPA9 PRSS36 THBS1 GPC2 PLEC ZG16 SSC5D BCAN FBLN1 RPS20 SERPINE1 COL6A1 WNT16 ARF4 TUBB CLEC3B DYNC1H1 HADHA MMP12 MMP24 ADAMTS5 CPZ CFP HSPG2 PFKP MATN2 TGFB3 CTSG OLFML2A ERBB2IP PRDX1 KAZALD1 NYX LAMC2 NAV2 DST TIMP4 RELL2 HNRNPU SLIT2 POSTN MMP11 EFEMP1 LMCD1 FGF9 LGALS3BP TGM2 PODN NID1 LAMB2 HSP90B1 ADAMTS3 RPS4X WISP2 ACHE COL14A1 VCAN JUP PTPRZ1 ADAMTS15 NES CLTC LGALS3 ABI3BP TGFB2 WNT1 COL8A1 TIMP1 COL4A3 COL5A1 EMID1 VWA2 COL15A1 COL6A2 ECM1 APOH LTBP2 FREM3 SFTPA2 ADAM11 FLRT3 SFPQ MFGE8 HSP90AA1 SHH LPL PI3 FREM2 DPT ACTG1 HAPLN2 ZP2 LOXL1 HPSE2 GLG1 RPS15A FGFR2 WNT3 LGALS1 MUC4 CST3 CCT2 TFPI2 EEF2 FBN1 LOC100507050 MEPE DCD HAPLN1 IMPG2 NCAN VEGFA GPLD1 CPA6 EMILIN1 PCSK6 CDON KERA DDX5 PLAT HSPA5 FLNA COCH ADAMTSL2 HSPD1 WNT4 RPS7 CTSD RPL23 RPS10 VWC2 FBLN7 PRSS2 ENAM VWA1 BGN DAG1 FN1 SPN THBS4 WNT9A RPL27 CILP2 WNT10B MMP26 FLRT1 COL9A3 FLNB SERPINE2 COL4A6 MFAP2 MMP13 LOXL2 ACAN PCOLCE TNR RPL11 OTOA SFRP2 RPL9 RPS3 OPTC RPS11 GPC1 LTBP3 PDGFB HIST1H4L ANXA2 ZP3 EPYC ODAM FBN2 TGM4 HTRA1 FGF1 WNT5A RELN COL1A2 RPN1 ADAMTS7 TFIP11 COL21A1 MATN3 FREM1 HIST1H4I ADAMTS17 HIST1H4H CCBE1 LTBP4 APOE NTN1 COL24A1 PODNL1 HNRNPK GANAB HIST2H4B NID2 ATP5B MMP17 ADAMTS4 RARRES2 SPARCL1 ADAMTS10 IMPG1 SPON1 SPON2 EMID2 WNT8A MMP9 AMBN TUBB4B DCN COL5A2 ANGPTL4 C8orf84 SFTPD ADAMTS9 WISP1 RTBDN P4HB ATP5A1 CRTAC1 AEBP1 COLQ LAMA4 EFEMP2 ADAMTS18 CASP14 MMP23B RPS17 CALR COL9A1 WNT6 FLRT2 SOST F3 TNC PRELP LAMA1 CLU LECT1 RPS3A CTHRC1 MMRN1 SMOC1 HIST1H4A LUM CKAP4 RPL35A APCS RPS14 COL7A1 MFAP4 NPNT LEFTY2 WNT7A COL17A1 GAPDH APLP1 IGFBP7 ADAMTS6 CPXM2 RPL12 HIST1H4K WNT2 DMP1 VIT PRTN3 FBLN5 LAMB1 NDP SLC25A6 LAMB4 MYOC MMRN2 RPL22 BMP4 CLEC14A WNT10A IL1RL1 ADAMTSL1 KRT1 CFL1 ADAMTS12 SERPINF1 RAC1 NOV MMP27 HIST1H4E ITGA6 ZP1 UCMA WNT9B C6orf15 CHL1 COL6A5 RPS16 VTN SLC1A3 RAN PHOSPHO1 SFRP1 HSPA8 COL27A1 RAB8A FBN3 DEFA1 TECTB GFOD2 HIST1H4C LAMC1 MMP16 COL5A3 COL6A3 ADAMTSL4 FKBP1A EFTUD2 ADAMTS8 MMP19 HIST4H4 COL4A5 OGN ANXA2P2 LAMC3 FGFBP3 LEPREL1 TINAGL1 WNT5B TNFRSF11B COL11A1 DSP EDIL3 ASPN RPTN CD93 SPOCK1 SPOCK2 GPC4 VWF CRTAP COL20A1 MAMDC2 SNCA HSD17B12 FRAS1 GPC5 SLPI CCT6A COL8A2 ALPL CTGF LAD1 SLIT1 AMELX CILP TGFB1 COL2A1 AMELY CSTA LAMA2 WNT11 LAMA3 HMCN1 DSG1 ANG PAPLN TGFB1I1 COL4A4 MMP15 TINAG SLC25A5 FMOD BMP7 TNXA RPS5 TNXB ADAMTS19 LTBP1 GLDN WNT2B COL19A1 MEGF9 MMP2 COL28A1 WNT3A BMP1 ZP4

GO_OUTER_MITOCHONDRIAL_MEMBRANE_PROTEIN_COMPLEX Any protein complex that is part of the outer mitochondrial membrane. TOMM7 TOMM22 TOMM5 TOMM40L PINK1 TOMM40 ANKZF1 TOMM6 TOMM20 TOMM20L TOMM70A SAMM50

GO_MITOCHONDRIAL_PROTEIN_COMPLEX A protein complex that is part of a mitochondrion. TOMM7 NDUFC1 COX8C TOMM70A NDUFA4 FXC1 UQCRB NDUFC2 TIMM22 UQCR10 NDUFA9 ATP5G2 NDUFA13 SLC25A6 NDUFV2 SDHB GRPEL2 HADHA NDUFB1 COX6A2 ETFB C22orf32 TOMM40L POLG SUCLG2 NDUFS8 TIMM17B UQCRC2 COX4I2 NDUFA8 NDUFA3 COX8A CHCHD3 TOMM22 TOMM5 COX5A ATP5E NDUFB3 PDK2 NDUFA1 ATP5G3 COX4I1 PPIF USMG5 NDUFB9 NDUFAF1 GRPEL1 WDR93 UQCRC1 TIMM50 NDUFS4 NDUFS6 NDUFV3 TIMM23 CCDC109B PAM16 APOO DNA2 IMMT ATP5L2 TOMM40 ATP5H IMMP1L NDUFAB1 SDHA NDUFA4L2 C14orf2 NDUFA6 ATP5I MCCC1 TOMM6 PINK1 AFG3L2 BAX PDK1 NDUFA11 NDUFB7 C18orf55 EFHA1 SAMM50 NDUFB5 ATP5B SNCA C11orf83 SDHC UQCRFS1 NDUFB10 ATP5D C19orf70 MCCC2 MRPS36 CLPX TOMM20L TIMM8B COX6A1 NDUFA2 NDUFB6 DLAT TIMM10 ATP5A1 TIMM17A APOOL NDUFV1 ANKZF1 IMMP2L SDHD NDUFA12 ATP5G1 NDUFS3 PMPCB NDUFB2 NDUFB8 NDUFS2 NDUFA7 TIMM9 ATPIF1 ATP5C1 NDUFS7 ATP5O BCKDHB MICU1 NDUFB11 TIMM13 ATP5EP2 BCKDK NDUFS5 NDUFA10 FOXRED1 C15orf48 ATP5F1 NDUFA5 TOMM20 ATP5L ATP5J2 NDUFS1 BCKDHA ATP5J BCS1L NDUFB4 LOC100652748 PARK7 DBT CHCHD6 SPG7 MCU COX5B

GO_HOPS_COMPLEX A multimeric protein complex that associates with the vacuolar membrane, late endosomal (multivesicular body) and lysosomal membranes. HOPS is a tethering complex involved in vesicle fusion. VPS39 HOOK2 VPS18 AKTIP STX17 VPS16 C14orf133 UVRAG VPS33A VPS33B HOOK1 VPS11 HOOK3 VPS41

GO_CELL_PROJECTION_CYTOPLASM All of the contents of a cell projection, excluding the plasma membrane surrounding the projection. CNO AP3B2 UCHL1 ADA HPCA SPG7 OPA1 DTNBP1 CDKL5 GRIK2 MAP2K4 GABARAPL1 WDR81 KIF1B RANGAP1 NDEL1 KIF4A CANX WLS PAFAH1B1 SPAST DLG4 RAB21 KCNAB1 BLOC1S2 SNAPIN PQBP1 FMR1 AP3B1 HIF1A GRIK3 BLOC1S1 PLDN AP3M2 LRRK2 AP3S1 UHMK1 AP3D1 MUTED HAP1 KLHL17 BLOC1S3 SOD1 OPRM1 C4orf49 NEFL MAPK1 AKR1B1 KIF3B AP3M1 KIF1A AP3S2

GO_OUTER_MEMBRANE The external membrane of Gram-negative bacteria or certain organelles such as mitochondria and chloroplasts; freely permeable to most ions and metabolites. MYO19 RHOT2 AMBRA1 RHOT1 BCL2L11 NAV3 GLTPD1 CPT1C ACSL4 SLC8A3 TOMM22 TOMM5 HK2 MSTO1 TSPO BID TDGF1P3 DNM1L GPAM BNIP3L ATP5G3 ACSL6 SLC25A46 AKAP1 CYB5B TOMM40 ABCB6 FIS1 SMCR7 BCL2 GPAT2 PHB2 ITPR3 GK FAM73B TOMM6 BCL2A1 QTRTD1 C19orf46 PLD6 BRI3BP CYB5R3 LETMD1 GIMAP5 CPT1B CCDC155 SLC44A1 PPP1R15A MGST1 RTN4IP1 BCL2L2 ATP5G2 MYOC MTX1 BMF SYNJ2BP GJA1 AIFM2 TOMM34 IFI27 P2RX1 NME1 DAO MTOR QTRT1 GK2 SOX10 MCL1 TOMM7 SPG20 FUNDC1 TOMM70A TOMM40L CASP8 BAK1 NXNL1 MUL1 ANKH SARM1 MT3 RSAD2 OPA1 FAM82A2 RAB11FIP5 SPATA19 LRPPRC SH3GLB1 DAG1 SLC11A2 MFF MFN2 MAVS GUCY2F HAX1 ACSL3 LTC4S AGPAT5 C14orf49 C12orf5 SYNE2 LPIN1 MOSC2 MTERFD1 AGK BCL2L1 RAB32 BPHL VAMP1 SPATA18 VDAC1 FAM73A MOSC1 EMD MAOB ACSL5 MFN1 BNIP3 BCL2L10 PMAIP1 SLC11A1 VDAC3 KMO ACACB RAF1 VAT1 SLC24A1 ARMCX3 DDX3X DHCR7 MLXIP DMPK MTX2 BAD MAOA EPHA4 TMEM173 HADHB VDAC2 SNN CISD2 SMCR7L TOMM20 PGAM5 BOK CYP27B1 FUNDC2 RETSAT LRRK2 ACSL1 PI4KB SNCA WASF1 ASS1 MTX3 PPP1CC RNF185 GDAP1 NLRX1 CPT1A MTCH2 BAX PINK1 GHRHR MARCH5 VPS13C SAMM50 MOAP1 PSEN1 COASY RPS6KB1 C4orf49 ATF2 CNP TMEM109 ANKZF1 SYNE1 SIGMAR1 CYB5A HK1 CISD1 NUTF2 GUCY2D PPP2R2B USP30 TOMM20L ST20 PGR ARG1

GO_APICOLATERAL_PLASMA_MEMBRANE The apical end of the lateral plasma membrane of epithelial cells. CLDN3 THBD MPDZ CCDC165 CLDN6 CLDN7 CLDN4 TMEM114 PALM NEDD4 CXADR CLDN8 OCLN FZD6 CLDN5 TJP1 JUP

GO_EUCHROMATIN A dispersed and relatively uncompacted form of chromatin. HIST1H1C HIST1H1A TRNP1 RRP1B RUVBL2 H1F0 ANKRD2 H2AFZ NR1H4 DNTT SETD1A TRIM28 CTNNB1 NELF RBMX ALKBH1 ASH2L CBX2 PPARGC1A SIRT1 TCF7 JUN CBX3 UHRF1 RNF2 HSF1 TRIM24 HIST1H1D DNMT3A KLF4 TBP H3F3C CREB1

GO_MICROTUBULE_END Any end of a microtubule. Microtubule ends differ in that the so-called microtubule plus-end is the one that preferentially grows by polymerization, with respect to the minus-end. ACTR1B SVIL CLIP2 KIF2C KIF18B MAPRE3 SPAG5 SLAIN2 MAPRE2 CDK5RAP2 MYO5A NCKAP5L MAPRE1 CKAP5 CLIP1 ACTR1A CLASP1 DST FAM175B ASPM NIN C15orf23

GO_ROUGH_ENDOPLASMIC_RETICULUM_MEMBRANE The lipid bilayer surrounding the rough endoplasmic reticulum. ARL6IP1 NAT8L CFTR C1orf9 SRP9 RP9 PLOD3 PI4KB UBA1 SSR4 ZC3H12A BCAP31 SEC61B SRPRB SRPR DNAJC3 PLOD1 PLOD2 GNRH1

GO_COMPLEX_OF_COLLAGEN_TRIMERS A complex of collagen trimers such as a fibril or collagen network. COL4A2 COL7A1 COL4A4 COL11A2 COL27A1 COL2A1 COL6A1 COL1A1 COL4A5 DCN TNXB COL4A6 COL5A2 COL4A1 COL1A2 COL6A3 COL5A3 COL3A1 COL8A1 COL11A1 LUM COL4A3 COL5A1

GO_NUCLEAR_INCLUSION_BODY An intranuclear focus at which aggregated proteins have been sequestered. TPR NUP153 RANBP2 NUP98 PABPN1 NXF1 RAD18 ANKRD32 STUB1 ATXN3 NBN ATXN1

GO_RESPIRATORY_CHAIN The protein complexes that form the electron transport system (the respiratory chain), associated with a cell membrane, usually the plasma membrane (in prokaryotes) or the inner mitochondrial membrane (on eukaryotes). The respiratory chain complexes transfer electrons from an electron donor to an electron acceptor and are associated with a proton pump to create a transmembrane electrochemical gradient. NDUFB2 NDUFA1 COX7A2L NDUFB3 PMPCB NDUFS3 CYC1 SDHD NDUFA12 COX5A COX8A NDUFA3 NDUFS5 NDUFS6 NDUFS4 WDR93 NDUFB11 UQCRC1 UQCRQ NDUFAF1 SURF1 NDUFB9 NDUFC2-KCTD14 NDUFS7 NDUFS2 NDUFA7 NDUFB8 COX4I1 NDUFS1 UQCRH NDUFA5 NNT NDUFV3 C15orf48 COX7B FOXRED1 NDUFA10 COX7B2 COX5B OXA1L PARK7 NDUFA6 SDHA NDUFA4L2 NDUFB4 BCS1L NDUFAB1 NDUFA4 COX7A2P2 NDUFB5 COX8C NDUFC1 NDUFB7 NDUFA11 HIGD2A COX7A1 UQCRFS1 NDUFV2 SDHC NDUFA13 CYCS NDUFA9 C11orf83 UQCR10 SNCA UQCRB NDUFC2 NDUFA2 COX6A1 UQCR11 HIGD1A NDUFB1 COX6A2 UQCRHL SDHB NDUFB10 NDUFV1 COX4I2 NDUFA8 UQCRC2 COX15 NDUFS8 COX7A2 NDUFB6

GO_T_CELL_RECEPTOR_COMPLEX A protein complex that contains a disulfide-linked heterodimer of T cell receptor (TCR) chains, which are members of the immunoglobulin superfamily, and mediates antigen recognition, ultimately resulting in T cell activation. The TCR heterodimer is associated with the CD3 complex, which consists of the nonpolymorphic polypeptides gamma, delta, epsilon, zeta, and, in some cases, eta (an RNA splice variant of zeta) or Fc epsilon chains. ZAP70 CARD11 CD8B PTPN6 SKAP1 SYK CD8A CD3E CD4 CD6 BCL10 ALCAM TRAT1 CEACAM1 STOML2 CD3G CD247 CD3D APBB1IP

GO_NUCLEAR_MEMBRANE_PART Any constituent part of the nuclear membrane, the envelope that surrounds the nucleus of eukaryotic cells. SUN1 C19orf46 TMEM43 P2RX1 C11orf85 P2RX3 P2RX7 P2RX4 P2RX2 P2RX6 LEMD2 LBR SUN2 LEMD3 P2RX5

GO_TRANSFERASE_COMPLEX A protein complex capable of catalyzing the transfer of a group, e.g. a methyl group, glycosyl group, acyl group, phosphorus-containing, or other groups, from one compound (generally regarded as the donor) to another compound (generally regarded as the acceptor). DHTKD1 KBTBD3 STK38 MED14 STT3A RING1 C20orf20 PRDX3 POLE4 PRPSAP2 MSL2 PRMT5 CDK14 WDR4 PHF16 RECQL5 TAF1 UBE2L3 ACVR1B NAA10 PRKACA CXXC1 KLHL4 RB1CC1 INTS6 SPOP CCNF DCAF10 UBR1 USP51 CHD8 ZDHHC9 YEATS4 CUL4A TNFAIP1 KLHDC5 WWP2 RAD18 HDAC9 UBE2S RNF2 GTF2F1 PIK3C2G ASB14 MLL5 GRINL1B ZER1 DAD1 CUL5 TSPAN17 RNF216 INTS12 PRKAA1 POLR3E GOLGA7 PEX2 KBTBD8 HDAC2 CCNL1 POLR2B INTS5 TAF12 TADA2A SETD1A PPP1CA OGDH RBBP7 BRE ANAPC11 KLHL32 ANKIB1 MSL1 ERCC8 SPTLC3 C10orf120 CTR9 ARIH1 MED17 TAF7 CUL9 ZNF335 PPP1CB POLR2C KLHDC8A TAF2 KLHL29 GOLGA7B CDK12 FNTB CAND1 DPM2 ORMDL3 PPP1CC WDR61 RPRD1B INTS2 KLHL38 ASB2 PHF1 DMAP1 ZZZ3 DCUN1D3 PRKACB TCEB1 EEA1 SORBS1 INTS7 GPR37 PHKA1 NRBF2 CDK4 POLR3H UBE4A PYCARD PAF1 PHKG1 DDB2 KAT5 ASB3 MAX KIAA1267 PRKAR2A UBE2A DCAF7 DCUN1D2 RNF8 CDC20 CUL1 MRPS36 C10orf46 DCAF16 PIK3CD CSRP2BP BUB1B KLHL8 PDSS2 UBE2U POLR2K PHKA2 H2AFY2 KLHL21 LAS1L KLHL17 POLR2D CHUK ABTB1 KLHL12 MED6 KLHL15 TUSC3 ASB10 VAC14 TAF6 NAA16 METTL1 MCM3 KDM5A FBXO4 DPM1 AKAP4 PTAR1 MSL3 INTS1 CBX7 C11orf51 TAF13 ERCC2 BARD1 CCND1 ORMDL2 BTBD11 WDR75 FBXO18 MBIP UBE2C KBTBD5 TRMT6 ZNF768 MAD2L2 FBXO17 BTBD9 ANAPC1 KBTBD13 KBTBD12 PFKL ING4 KCTD10 BTBD2 OS9 DYDC2 CUL4B HCFC1 JARID2 PIK3C3 IKBKG TGFBR2 MAT2B SUGT1 FZR1 ERCC3 METTL14 KLHL20 POLR3B MAT2A C7orf52 KLHL18 TAF8 FBXO2 E2F6 DCAF6 FBXW4 ELP3 CDC23 CDK2 POLR3A PFKFB1 PRKAR1A PFKM MPHOSPH8 FBXW8 CREBBP ENG CCND2 PRKAG3 KAT8 PIAS4 SUPT3H HSPA1B PHKG2 RBX1 PIK3C2A ERCC5 FBXL12 KBTBD7 PRPS1 INTS4 GTF2A1L RUVBL2 KAT7 EPC2 HELB ZYG11B RNF20 CDC26 EZH1 MGA PIK3R3 TAF5 DPM3 MED24 KBTBD6 KLHL36 ATG3 FAM48A ANAPC5 MED27 USP22 TRIM37 TWISTNB MLL2 MEAF6 CAB39 OGDHL TRAF7 MED20 KDM6A C17orf49 ENY2 TBP KCTD17 MED18 BMI1 VHLL CCIN CCNC PHF17 PIK3CA SUZ12 RTF1 LEO1 MAP3K5 BRAP DCAF5 MAP3K13 ANAPC10 PIK3R5 C12orf41 RNF144A BECN1P1 BECN1 POLE2 ASB18 CCNK BRPF3 TRPC4AP POLR2F CCNE1 BRCA1 MORF4L1 ASH2L H2AFY DCAF17 YEATS2 FBXL19 BRCC3 CKS2 KLHL23 INTS3 MED1 SPTSSA CDKN1B PGGT1B GTF2H4 NEK10 ERC1 PHF19 MED4 POLR2I SENP3 PYDC1 PRKAR1B TEX10 TRMT61B CHM ERCC4 RBBP5 GTF2E2 FBXO27 NCOA6 MED30 RABGGTB UBE2D2 PUF60 DLST KLHL11 AUP1 DR1 GLMN KLHL25 CRCP OGT BACH1 FBXL22 ATG13 TBC1D5 POLA2 SMURF2 RB1 KLHL31 FBXO6 POLE PPARGC1A RNF11 TADA3 MED11 ZYG11A INO80C FBXO31 RPN2 KLHL14 UBE2E1 MED12 WDR5 DCUN1D1 SHARPIN TRIM40 RNF222 CTDP1 PIK3R2 ING5 HSPA1A CBX8 ENC1 MIB2 GTF2A2 DCUN1D4 FBXW5 GTF2H2 TAF5L MED7 MTOR KBTBD10 CDKN1A POLR2E POLR3K KLHDC7B TAF9B DLD POLG KLHL28 FBXO11 TAF3 SPOPL DNAJA1 KLHL22 EPC1 STUB1 C2orf67 EP400 HSPA8 SPTSSB POLR3C PHF20 GAN MED10 SKP1 TAF4B FBXW7 CHD6 UBE2I KLHL2 CHRAC1 SEL1L KRTCAP2 BTBD3 FBXO10 USP33 RNF31 FAM48B2 RABGGTA PIK3CG POLR3D SYVN1 PARK2 POLR3GL INTS8 BTBD16 CCND3 MED31 ATXN7L3 ORMDL1 C19orf2 NEDD4 POLR2L CHML KLHL26 AMFR PIK3R1 GTF2A1 TAF6L ASB15 FBXO44 RNF7 NAA35 POLE3 RNF19B PELP1 ATXN7 CCNT2 TAF1L TRRAP KLHL34 ANAPC16 DPY30 POLD3 POLR2H BRD1 MNAT1 TAF7L CDC27 VHL EDF1 FAM105B CKS1B WTAP DCAF12 UBE2B KBTBD2 ANAPC2 POLR2G TGFBR1 RNF168 TRAF2 ERCC1 COMMD1 GMCL1 CRBN BACH2 TAF10 ERN1 KCTD2 GTF2F2 CDK9 SNW1 FBXL7 ELP4 MYO6 CUL2 UBE4B ASB16 ABAT PRKAG1 LSMD1 CBX4 SKP2 RNF217 OSTC LMO7 TAF11 DCAF11 MSL3P1 NAA25 DTL KBTBD11 USP34 ASB4 USP27X UBE2D1 PAPD4 TOPORS CCNT1 IKBKB PRKAG2 SIRT1 REV3L KEAP1 FBXO21 CUL7 POLR1B PHC2 KLHL13 FBXL4 ACTL6A CCNL2 KCTD13 CCDC101 SPTLC2 FBXO25 UBE2N RNF4 KLHL33 PHKB PRKAB1 RBCK1 FBXO7 HSPD1 GTF2H1 POLR2M CUL3 POLR3F RNF144B CBLL1 DDOST MMS19 TERT KLHL24 DYDC1 RCHY1 KLHL10 DCAF8 ELP2 BTBD1 TP53 SAP130 BTBD6 EP300 STT3B FBXL15 FAM58A ANAPC13 MARCH6 TRIM21 SPTLC1 KIAA1310 FBXO9 HOXC10 RBBP4 PHC3 BTBD18 ANAPC7 NAA15 CDK7 TADA2B RPAP2 POLR2J2 KBTBD4 CDC16 FBXO24 GMCL1P1 AEBP2 CDK13 TADA1 PRKAR2B KLHL3 CBX5 TRMT61A PIK3R6 PHOSPHO2-KLHL23 DAB2 FBXL3 IPP PLRG1 RUVBL1 UBR2 KLHL7 CCNH ABTB2 INTS10 TAF4 CDC73 DNA2 CCNY BRD8 SESN2 RPN1 ACVR1 SUPT7L POLR1E MAGT1 KLHDC7A POLR3G FBXO8 PIK3C2B PIGV DCUN1D5 NAA30 PIK3CB PRKAB2 ACTB USP47 BRPF1 DYRK2 POLR2A DDB1 TAF9 FBXO39 POLD4 ANAPC4 METTL3 NAA20 CDK6 KLHL1 MLL4 POLR1C ULK1 PINK1 PHF15 PRKAA2 CBX2 WDR82 EZH2 MCRS1 PCGF6 FAM48B1 BTRC CD3EAP PRPF31 UBR3 MEN1 POLR1D PRDM4 POLR2J MLL3 C16orf53 MTF2 INSR EED CPSF3L UBAP2L ARIH2 PIK3R4 POLA1 UBE2J2 ATG14 FBXL21 RNF14 ACVR1C MORF4L2 IKBKAP FBXL2 SHFM1 ZNRD1 KLHL9 TBPL1 UBE2V1 PAXIP1 KIAA1429 FBXO15 NAA11 RNF19A KAT2A KAT6B KLHL30 SETD1B MED21 KCTD5 WWP1 PHC1 RPRD1A MLL POLD1 KAT6A RNF40 DCAF4 KAT2B FBXO32 PDSS1 KLHL5 GTF2H3 TCEB2 FBXW11 POLR1A FAM58BP ING3 IRS1 DCAF13 FBXL5 FNTA INTS9 NAA50 RPRD2 MAP3K7 BCCIP UBE2V2 PCGF2

GO_CYTOSKELETAL_PART Any constituent part of the cytoskeleton, a cellular scaffolding or skeleton that maintains cell shape, enables some cell motion (using structures such as flagella and cilia), and plays important roles in both intra-cellular transport (e.g. the movement of vesicles and organelles) and cellular division. Includes constituent parts of intermediate filaments, microfilaments, microtubules, and the microtrabecular lattice. CLMP SNTB2 NLRC4 CCDC37 WDR35 MYLPF GAS8 MYBPC2 MZT2B VPS4A KRT84 NEIL1 MAD2L2 KRTAP19-1 KRT16 MYO16 CCT2 DYNLL1 KLC2 KRTAP21-2 DLGAP5 ALS2 KRTAP29-1 RAB11B IFT46 DNAH7 KRTAP5-5 KLHL12 CRHBP WDPCP PKD2 KNCN SRC AGBL2 ACTN1 KRT14 KRT7 TSSK2 TCHP RADIL IFT122 NICN1 DIAPH1 CETN3 TTC28 DNAAF2 KRT75 PRKAR2A FLOT2 KRT76 NUSAP1 ARHGEF10 SPTBN4 MYO1H KRTAP23-1 PDCD6IP INVS TCTEX1D4 RCSD1 BUB1B KRT33B MYO3A FAM101A ARPC3 CDC20 MYH4 RPRD1B G6PD TOPBP1 MAP1LC3A HARBI1 TMSB15A KRTAP10-7 KRT83 NES FSCN3 AXIN2 DYNC2H1 POLR3H PLS1 MST1R KRT81 CCDC41 JUP CDC42 TMSB15B TLN1 SORBS1 REEP4 MYH7B KRTAP22-2 RHOQ MLF1IP PHF1 STAU2 PRKACB LRP8 PHAX DPYSL2 NPM1 BFSP2 ALDOB PLK1S1 LOC100653515 ROCK2 AHI1 CYLD WIPF2 TTLL6 SEPT6 MAP2 CLUAP1 DNM3 MYO5A NEFH CLIP1 ANKRD26 LRMP MARK4 IQGAP1 NIN MCPH1 FAM101B JUB RAC3 RIF1 NDRG2 TTLL1 ACTN4 EML6 CHAMP1 KBTBD8 MTSS1L SLMAP FAM154A KIAA0284 SGOL1 GDPD2 TUBGCP2 TCP11L1 TUBA1A BBS1 CASP8 KRT40 EPB41 ABLIM1 PPP4C FGF13 KRT32 KRTAP10-2 CCNF BIN2 IFT80 TMOD4 MAPRE2 PXN LIMA1 CDC25B CCDC61 AK8 DYNC1H1 SPICE1 TUBB LOH12CR1 RAD18 NEK1 DAAM1 NDEL1 FAM82A1 SCLT1 NUDCD2 KIF13B TMEM141 YIF1A KRTAP9-4 RASSF1 PTK2 MID1IP1 GNAI1 WDR1 KIF23 NME2 CTNNB1 KRTAP21-1 DES DCDC2 CALM2 WHAMM ARHGAP6 ARPC2 MYL12A CETN1 HOOK2 RASSF5 JAKMIP1 TPM3 ODF2L DNAH3 DDHD2 CCT7 DNAH10 ACTR1A MED12 PCNA CCDC164 BBS7 MYOM1 TNNC2 DYNLRB2 TNNT3 CEP152 GLI3 C6orf165 MAPRE3 GTSE1 MYH16 CALD1 AATF CCDC42B MYO7A KIF7 MPHOSPH9 MYL4 KRT34 BCL2L1 KIF14 ZNF274 KATNAL2 PCM1 C10orf92 ZNF207 KRT80 CEP112 IFFO2 DCTN2 CTTN ODF4 ANKRD53 YEATS2 SPAG6 TTK IFT74 COBL KIF21B EEF1A1 TUBB6 CAPN2 MAP4 DYNC1I1 CDK5RAP2 CCDC64 CYBA TRPV4 CEP120 MAP7D1 FAM125A CBX3 SNX10 C17orf28 NCAPG C15orf23 NEK10 LRGUK RFWD2 KRT4 LEO1 NEK2 KRTAP22-1 GNAI2 TUBB4A MYH15 AKAP11 RSG1 PHF17 MED28 CCDC146 KLC4 TERF1 NPHP4 LMNB2 MTUS1 PBXIP1 ADD2 TACC1 DNALI1 SVIL DDX6 KRT6B KIF18B RBM39 DNAH12 RPS6KA2 PLK3 CCDC38 KRTAP1-3 NDRG1 DPYSL3 PAFAH1B1 RHOU SPTA1 DNM1 TTLL13 RTTN ESPN MASTL SPTB SNPH TCEA2 MYH1 CDKL2 ECT2 DPF2 KIFC2 TMSB4Y KRTAP20-1 PLK4 C7orf11 ZW10 GNAI3 PINX1 KIF25 FMN2 BCL2L11 ARPC1A CEP41 NDN DLC1 PSMA1 CCDC141 KRTAP5-10 RUSC1 CEP70 IFT140 KRTAP9-1 KRTAP4-2 POC1B PPP2CA CD2AP C12orf55 RSPH9 TTLL7 TPPP INTU VIM LOC100130097 TSC1 TUBG1 CCDC13 MYBPC1 KRTAP4-8 FAM82A2 TTC26 MAF1 CAPZB WDR67 KRTAP10-5 PKP1 KRTAP12-2 CLDN5 KRTAP19-6 MYO5C DCXR AAAS CDK2 DIS3L DBN1 SPTBN5 ATF5 SH3PXD2A ILK MYO15A TUBB3 CCDC67 MYO18B SKI BICD1 TUBA1C NEDD9 ATG7 PTP4A1 ARPC4 FOPNL KRTAP1-1 ACTN3 SIPA1L3 ZNF12 TTLL9 TTC8 CENPV AURKB AKT1 EML5 SKA2 RASSF10 IFT57 MYH14 HAUS4 EPB49 PLEKHA7 DISC1 SEPT2 SLC8A1 SDCCAG8 HYLS1 KIF20B CCDC151 AURKC CSNK1A1 SHROOM2 ANXA11 XIAP KIF19 STOX1 DSTN INPP5E ATF4 ATG16L1 KRTAP11-1 KIAA1731 MYO1D MYO6 IFT172 MYH8 MYO1B KRT82 TBCD KRT71 EVI5 PARP4 CYLC1 DCTN1 VRK1 KNTC1 TUBGCP6 NEFL GEN1 TUBD1 MAP1LC3B WDR81 PRPF19 LANCL2 SEPT12 HOOK1 INCENP ZFYVE19 CDC27 KRTAP9-7 RACGAP1 CKAP2L CCDC114 STIL FBXL7 CCT4 RAPGEF3 BLOC1S2 CCDC40 KRTAP4-11 PARP3 CIB1 IFT43 MYL7 NUBP1 HDAC3 TAPT1 CEP104 CIAO1 DSP DCLRE1B TUBE1 MEFV MYLK DVL1 ASPM MAPK1 PSEN1 GEM KRT121P KRTAP13-2 TBCCD1 TRIP4 ATAT1 SAA1 LRRC16A RILPL2 KRTAP10-12 C19orf46 PRC1 CHEK1 KIAA0753 ATG5 FBXO5 KRTAP10-3 ANLN PIN4 VPS4B MYO1C MAP2K2 OR2A4 PTPN12 RILPL1 STMN1 HAUS3 NUP85 KRT31 RAB8A TBCE C10orf90 SERP1 ARPC1B CORO1A ACTR8 MYO1A HAUS1 TXNDC9 TCP1 CKAP5 FIGN RAN MAP3K11 TBC1D7 ALS2CR12 MYO9B KIF22 MAD1L1 CENPF CEP44 CEP97 MDM1 KRT38 STARD9 DCTN3 CCNB2 TULP3 SPATC1 RAC1 ACTR3 ADAM8 CDCA8 KRT6A RASSF3 KIF3A KRT1 DIDO1 RANGAP1 CIR1 KRT2 UBXN6 HYDIN KATNA1 SH3PXD2B CTDP1 USP20 UPF3B CCDC77 MAP2K1 MST4 HSPA1A AKAP9 FKBP15 GJA1 BOD1P PDE4D NME1 KLC3 TRIM63 MAPRE1 KRTAP5-1 MZT2A PLS3 NCOR1 IQGAP2 WRAP73 DCAF13 HSPA6 ALMS1 TSKS GSK3B C4orf47 CHMP1A BBS4 DNHD1 TMEM214 CEP76 PDE4DIP VANGL2 CEP192 KRT28 EXOC7 FSCN2 FNTA TNKS KRT17 MYRIP KRTAP9-3 TUBA3D RNF19A LLGL1 KRTAP10-1 ARL13B HDAC6 GNG12 PPP1R12A CEP55 SLC16A1 RAB11A SNAP29 LPXN KAT2B CHD3 PKN2 KRTAP5-3 FHL3 YWHAE BBS9 IST1 TUBB2B KAT2A KRT6C ZFYVE26 CDC6 CTAG2 SYTL4 KIF2C KRT13 WDR34 C1orf135 EZR PCNT DLGAP2 C21orf2 DCTN6 CCDC85B MYOM2 TUBGCP5 NUBP2 CDC45 PIK3R4 UXT KRTAP5-9 RSPH4A RAD51 MYL12B KIAA1751 TUBB4B SRPRB LCK SCYL1 NUP62 SPEF2 RABGAP1 FEZ1 KIF17 IKZF4 FAM110C KIF6 NRP1 KIF3C IFT88 WDR73 CDK6 ARHGEF5 TXNDC2 TUBGCP3 HNRNPK ARHGAP4 SHCBP1 DNAH5 FKBP4 DYX1C1 KIAA1984 NUDT21 KRT79 KRIT1 C9orf24 ACOT13 NUDC FMN1 CEP164 MAP2K5 CEP78 KIFC1 RAB11FIP3 RAD51D MYH10 SPAST UNC119 KRTAP19-8 DZIP1L MAP7 NCKIPSD PPP1R42 PSKH1 KRTAP4-3 CETN2 TBCB PRPH FAM83D DAB1 KRTAP5-2 TMEM67 KIF9 KRTAP19-5 DNAAF1 ODF3 SPAG8 SIRT2 DYNLRB1 GLI2 SPAG17 CRYAB KIF4B HOOK3 TUBA4B MYO3B PRKAR2B ARPC5L EYA3 C2CD3 CLIC4 SPTAN1 IFLTD1 APPBP2 RAB28 CAPRIN2 FAM110B B9D1 MAP6D1 ACTG2 MT3 NAV1 SPPL2B UBR4 CSNK1D RCC2 LASP1 CEP63 CEP89 EFHC1 LRPPRC DNAJB13 TUBA3E KIF5B RGS20 BIRC5 KRT39 SPECC1L RPS7 C1orf88 KRTAP20-4 KIAA0528 MAP9 KRTAP19-7 FLNA GNAT3 TPR HFE IQCG AZI1 OBSL1 TRIM59 KRTAP9-9 ACTR3B SLC2A1 CUL3 TNNI1 C1orf190 ERCC2 VIL1 KRTAP4-6 CLIP2 PDE4B DSN1 NEFM C14orf45 KRT77 KIAA1598 MLPH TTC19 MYO1F RP1L1 C1orf96 PTPN20B NUP107 ABCA2 TRAF3IP1 MYO9A IFFO1 GSN LAS1L BCL10 OLA1 AK1 SHROOM1 RANBP9 ACTG1 CALM1 RP1 CHD4 KLHL21 KRT8 KRTAP3-2 DDAH2 BFSP1 KIF18A MAPK14 C16orf48 TNNT1 MYL9 MCM3 MAP1LC3B2 TTLL8 FAM179B DYNC1LI2 TMOD3 DAPK3 RPGR PGM5 RAB3D ACTR1B LCA5 CCNB1 TFAP2A RAB6C KRTAP26-1 TCTE3 POC1A TPM2 TBL1X RPP25 MICALL2 CCDC78 NME7 FLG TRIM54 CCDC63 TTBK2 CCDC15 NEK6 SASS6 CLTC SPTBN2 KIAA0368 CGN FLII ARPC5 CCDC81 FNTB RABL5 KRTAP13-4 CDC42BPB C21orf56 FAM175B AFAP1L1 BBS2 ERCC6L KIF11 DYNC2LI1 RSPH1 GSG2 SSNA1 TAF1D SPRY2 SYNJ1 TNNI3 SLAIN2 KRTAP9-8 ZMYND10 CROCC KRT5 DCX EVC PSRC1 CEP19 ATP6V1D MYL5 NEIL2 DST FAM83H DNAI1 KRT36 TMSB10 SEPT7 SEPT1 MYO19 MZT1 PCDP1 ACTR3C CCDC165 CLIC5 FERMT2 FLOT1 GPER TUBA1B MACF1 KRTAP1-5 TBCA KRTAP13-3 LMOD1 TUBGCP4 KRT19 CNTROB CEP350 ODF2 PAWR LRRC48 CNTLN NEK7 TACC2 MYL3 TMUB1 RGS19 FAM96B LRWD1 NEBL TEK TMEM48 CEP68 KRTAP10-11 PLEKHH2 CYLC2 IQCB1 KIF20A EML3 MAD2L1BP SAC3D1 KIF2A MOBP TEKT3 DBNL KCNAB2 DNAH17 KLHDC5 KRTAP5-11 BOD1 KRT20 MAP1S KRTAP4-4 C14orf166 C12orf52 ID1 DCTN4 KRTAP5-6 MYO1G CCDC8 MAEA PLK2 ARMC4 TBL1XR1 RAP1GAP2 KIF1C GAS2 CCP110 TUBB1 WIPF3 AGBL4 PRKACA PROCR C16orf80 CEP72 TUBA3C WASH3P ACTN2 MAK PPP2CB NUMA1 CENPJ STIM1 BRSK2 RB1 SNX4 CCDC113 TUBA8 LRRCC1 KRTAP5-7 TRIM32 KIF27 KRTAP10-10 INO80 FHOD1 LATS2 KLC1 CAP1 MYH9 TADA3 CAMSAP2 ZNF322 TRIM55 WDR62 KIF15 DNAH6 HSPB11 PSEN2 TUBB2A CNTRL PIBF1 DNAL4 CCT5 GFAP KRT86 ACTR2 EMD MAPKAPK2 IFT81 TPPP3 FBF1 BRCC3 KRT74 NFE2L2 BRCA1 MAP1B WDR47 CCDC116 CDK1 KATNAL1 SPIN1 MYL1 PSMB5 C19orf20 ORC2 APEX1 CAPZA2 JTB KIF4A LMOD2 CCDC103 PKP4 ELL2 MYL6 ARL8B KRTAP8-1 TNNT2 CCHCR1 CCDC99 EML2 LMOD3 TTLL4 ARFGEF2 KRTAP3-1 ABLIM3 GABARAPL3 CCIN TTLL11 CCDC92 SMC3 KRTAP12-4 DYNC1I2 MYH3 VCAM1 NARF MICAL1 KIFC3 ARHGEF2 MYO10 RBBP6 HAUS2 PRKCZ KIF12 KRT9 BIRC7 CEP57L1 VMAC TTC23L CBX1 USH2A SKA1 FSD1 CAMK2B KRTAP27-1 MEAF6 CCDC39 CDH2 MIB1 KRTAP6-1 SPTBN1 MYO18A CAP2 CSPP1 REEP2 NEK9 CAPZA1 HERC2 CAMK2N2 APC SEPT11 NR3C1 KRTAP25-1 NDE1 EML4 ANKRD32 CGNL1 KRTAP4-5 NIT2 SLC8A3 KIF5C LMNA TUBAL3 SH3GL1 CLASP1 BIRC3 PXK C6orf204 DLG1 HSPB1 WIPF1 NEURL4 CENPE STX1A HCK KIF24 DNAJA3 PDLIM7 MYL2 HSPA1B KRTAP20-3 KRT12 CAPZA3 TBC1D30 KRT33A LDLRAP1 KRTAP6-2 TMOD2 IKBKG PIK3C3 PLEKHG6 KIAA1383 TSPYL2 GYS2 MYH2 SHROOM3 STX1B CAPG SMAD7 CEP95 KIFAP3 ACTC1 KRT23 HAUS8 PAK1 KRTAP12-1 KRT10 SPAG9 FNIP2 FAM161A LMNB1 NF2 TOPORS BIN3 SPAG5 MID1 CEP85 YES1 C18orf10 TMSB4X KIF5A TUBA4A KEAP1 C9orf9 CUL7 SLC1A4 IFT20 CAMSAP1 ZNF365 RIC8B C1orf192 AGBL5 KRTAP12-3 SLC25A5 TEKT2 KRTAP2-1 DCTN5 KRTAP4-12 OFD1 DTL SBDS KRTAP16-1 LLGL2 WASH1 REEP3 IFT27 NEK3 PLA2G6 RPGRIP1L TOP2A KRTAP24-1 UVRAG CAMSAP3 DYNLL2 KRT85 TNNI2 DCAF12 KRT26 TTLL3 KRTAP9-6 HAUS6 KIAA1009 ICK CEP290 IGBP1 HTT CCT6A LATS1 CCDC28B MYO5B SFI1 ZNF664-FAM101A CKAP2 CC2D1A MYH6 KRT222 TMOD1 CEP250 PLK1 SYNC DNAH11 KIF3B PLA2G3 CDC14A CCDC102A MAD2L1 RPGRIP1 POLB SLC9A3R1 TLK2 PEA15 WASL KRT72 TRIOBP CLTCL1 SEPT9 HAUS5 KIAA1377 MAP1LC3C FSCN1 GSDMC SS18 FAM110A CRMP1 SPATA7 RP2 MKS1 MYBPC3 HEPACAM2 PKHD1 CCDC96 MYO1E TEKT4 HSPA2 CORO1B VPS18 KRTAP10-6 TTC12 SHANK2 SKP1 STAU1 LZTS2 EML1 AMBRA1 POC5 KRTAP7-1 GAS2L3 FTCD FYN E4F1 FAM82B SYBU FRY AK5 ITSN2 SELS DCLK2 KRTAP10-4 ESPL1 USP33 FERMT3 BRCA2 KIF21A KRT35 LCP1 DNAH9 KRTAP6-3 GABARAPL1 DNAH14 DNAH8 SARM1 KIF26A SPEF1 PCGF5 KRTAP19-3 TTLL5 CLASP2 DNM2 UMOD USP2 KLHL22 PTPN23 MARCKS CAPN6 VPS37A BMF KRTAP1-4 SPAG16 CCT3 GOLGA2 FGFR1OP SYNM KIF1A MLLT11 MYO7B CNN2 MTUS2 FBLIM1 ARHGAP35 HSPH1 ARL3 KRT3 RAB11FIP4 KPTN MNS1 CDC42BPA CALM3 PPP2R3C KRT18 KRT78 SHROOM4 KRT37 ARL2BP KRT15 RDX FBXW11 GPSM2 TPM1 CDH1 TUBB8 CCDC14 TNNC1 KIF13A B9D2 LRIF1 KRTAP20-2 BRSK1 TIAM1 MARK2 DNAH1 BIRC6 MTPN ARL6 KRTAP2-4 CCBP2 SCTR ACTA1 KRTAP10-8 NEDD1 PPP4R2 RGS14 KRTAP10-9 AMOT MAPT CEP57 CASP14 AIF1 PHF7 MKKS AIF1L MAP1A CEP135 DNAI2 MYZAP HDAC4 KRTAP5-8 LRRC45 BBS5 ATG14 KRTAP4-1 TUBG2 INA UTRN KIF2B CDK5RAP3 AXIN1 APC2 DHX9 KRTAP17-1 TPT1 HAP1 KRT24 EIF3A KRT25 ID2 MTA1 KRTAP9-2 GRAMD3 KATNB1 SHCBP1L SYNPO NOX4 IFT52 DDX11 KRTAP5-4 SKA3 KRTAP19-4 TXNDC3 TBCC MBNL1 TNKS2 RUVBL1 KIF1B AKAP13 NINL ARL2 CEP170 KIAA0586 MYH13 TPM4 DYNLT1 ADD1 CCDC50 MAP6 KRT27 CCT8 TRAF5 MID2 CCDC155 CEP128 TEKT1 DYNLT3 RANBP1 KIF26B MAP7D3 ARL8A CDC16 CYP2A6 AURKA KRTAP3-3 PPP1R9A LOC730755 HAUS7 DZIP1 RAC2 MYBPH RPS3 PAX2 XRCC2 KRTAP13-1 USH1G KRTAP15-1 ODF3L2 NSUN2 RANBP2 KRTAP4-9 ATP6V0D1 KRTAP21-3 EPB41L2 TPX2 DNM1L BIRC2 ACTR10 FLNB DYNC1LI1 MYH7 PKP2 SUCLG2 PDLIM2 TTN LRRC49 ZYX MDH1 ROCK1 RAB11FIP5 CCDC124 SMEK2 MYADM ATM KIF16B C13orf15 C7orf31 XRCC4 MYH11 WDR43 FERMT1 SMEK1 APP BIRC8 BCAS3 DNAH2 GLI1 KRT73 NCKAP5L MMS19 PALLD KRTAP19-2 SSX2IP SNCG WRN MYL6B TSEN2 UPP2 PNN C9orf102 RELB RASSF7

GO_MYOSIN_FILAMENT A protein complex containing myosin heavy chains, plus associated light chains and other proteins, in which the myosin heavy chains are arranged into a filament. TRIM32 MYH13 MYBPH MYH6 MYBPC1 MYH14 ACTG2 MYH15 MYBPC2 MYH2 MYH7 MYH1 MYH11 MYH9 MYH4 MYOM1 MYH7B MYH3 MYH10 MYBPC3 MYH8 MYOM2

GO_ORGANELLAR_RIBOSOME A ribosome contained within a subcellular membrane-bounded organelle. MRPL3 MRPL27 MTG1 MRPS18B NSUN4 MRPL41 MRPS18C MRPL51 MRPL39 MRPL48 MRPL19 MRPL13 MRPS9 MRPL21 MRPS11 MRPS14 C1orf177 MRPS12 MRPS24 MRPL9 MTERFD2 MRP63 MRPS22 MRPL47 MRPL10 NDUFA7 MRPL15 MRPS5 MRPS26 C12orf65 MRPS33 MRPS28 MRPL20 MRPL35 MRPL40 MRPL32 MPV17L2 MRPS31 MRPL33 MRPS7 MRPS2 DAP3 MRPL36 MRPL43 MRPL28 GADD45GIP1 MRPL17 MRPL2 MRPL37 CHCHD1 MRPL42 ICT1 MRPL23 MRPS18A MRPS36 MRPS16 MRPL34 MRPL11 MRPL22 MRPL18 MRPS15 MRPL49 MRPS35 MRPL46 MRPS6 MRPS21 MRPL16 NSUN3 GTPBP5 MRPL55 MRPS17 MRPL12 MRPL52

GO_TORC2_COMPLEX A protein complex that contains at least TOR (target of rapamycin) and Rictor (rapamycin-insensitive companion of TOR), or orthologs of, in complex with other signaling components. Mediates the phosphorylation and activation of PKB (also called AKT). In Saccharomyces, the complex contains Avo1p, Avo2p, Tsc11p, Lst8p, Bit61p, Slm1p, Slm2p, and Tor2p. PRR5 SESN2 TTI1 MAPKAP1 PRR5L RPL23A PINK1 MLST8 RICTOR MTOR TELO2

GO_TELOMERE_CAP_COMPLEX A complex of DNA and protein located at the end of a linear chromosome that protects and stabilizes a linear chromosome. CTC1 XRCC6 CCDC79 POT1 XRCC5 TERT TEN1 TINF2 TERF1 TERF2IP ACD OBFC1 TERF2

GO_BANDED_COLLAGEN_FIBRIL A supramolecular assembly of fibrillar collagen complexes in the form of a long fiber (fibril) with transverse striations (bands). COL5A3 COL27A1 COL5A2 COL1A2 COL3A1 COL11A2 COL5A1 TNXB COL2A1 COL1A1 LUM COL11A1

GO_GAP_JUNCTION A cell-cell junction that is composed of an array of small channels that permit small molecules to pass from one cell to another. At gap junctions, the membranes of two adjacent cells are separated by a uniform narrow gap of about 2-4 nm that is spanned by channel-forming proteins called connexins, which form hexagonal tubes called connexons. MIP GJB6 GJB2 GJD4 GJA5 DSC1 GJA8 GJC1 TJP1 GJC3 PANX2 CALB2 GJB1 GJA1 GJA9 GJB5 GJB3 GJA4 GJA10 GJB7 GJC2 GJB4 GJA3 SPECC1L DBN1 PANX1 GJD2 GJD3 NOV PANX3

GO_GABA_RECEPTOR_COMPLEX A protein complex which is capable of GABA receptor activity. Upon binding of gamma-aminobutyric acid (GABA) it transmits the signal from one side of the membrane to the other to initiate a change in cell activity. Major inhibitory receptor in vertebrate brain. Also found in other vertebrate tissues, invertebrates and possibly in plants. Effective benzodiazepine receptor. GABRA3 GABRB1 GABRG1 GABRA6 GABRA4 GABRA5 GABRB2 GABRA1 GABRQ GABRD GABRE GABRR1 GABRA2 GABRR2 GABRB3 GABRG3 GABRR3 GABRG2

GO_INHIBITORY_SYNAPSE A synapse in which an action potential in the presynaptic cell reduces the probability of an action potential occurring in the postsynaptic cell. GLRA1 IGSF9 IQSEC3 CEP112 NPTN MAF1 GABRA2 GAD2 SYT11 SLC32A1 IGSF9B NLGN2

GO_PHAGOCYTIC_VESICLE_MEMBRANE The lipid bilayer surrounding a phagocytic vesicle. HLA-B HLA-C INPP5B HLA-E RAB5A TLR6 IRGM ATG5 VAMP7 ATP6V0D2 PIK3R4 ANXA3 DNM2 CYBA RAB20 DMBT1 RAB9A ATP6V0A4 TLR2 ATP6V0B RAB7B ATP6V0E2 RAB8B ATP6V0C SLC11A1 SYT7 RAB43 LAMP1 RAB38 ATP6V0D1 LAMP2 ATP6V0E1 RAB9B HLA-G B2M RAB32 TCIRG1 RAB22A RILP RAC2 RAB11FIP1 HLA-H RAB23 HLA-A RAB7A CORO1A OCRL RAB8A ATG12 RAB31 TLR1 RAB39 RAB34 HLA-F PIK3C3 RAB11B RAB10 CYBB ATP6V0A2 ATP6V0A1

GO_PERIKARYON The portion of the cell soma (cell body) that excludes the nucleus. CDK5 UCN PDE11A PI4K2A SYNPO BRD1 CNR2 LRIT3 SLC8A2 DRD2 KCNA1 MAPK1 KCNE3 NAIP KCNB1 NEUROG1 PDPK1 ASTN1 ASS1 KCNB2 CPNE5 LRRK2 ITGA8 ENO2 GRIK3 PCSK2 FMR1 ITGA1 GRIK5 GLRA1 CACNA1F DRP2 PDE10A NPFF BGLAP KCND2 AMIGO1 PENK CCL2 CTNND2 TTLL7 PTPRN GRIK2 CCK HTR5A ZNF259 KLHL24 MCRS1 HPCA IFNG FUS GLUL AIF1 CRYAB NELF SMN2 ADC SLC8A3 CNTNAP2 PDE9A GLRA4 GNRH1 PAM SLC5A7 PPP1CA OPRM1 ENDOG PPP5C HDAC6 TH CRHBP ASTN2 SIRT2 KCNC2 CTSL2 NDN DDN CCR2 GOT2 CNGA3 KCNAB1 KIAA1598 TMEM100 OPRK1 GNB2L1 EPHA4 RBFOX3 GLRA3 TOP1 CHRNA10 OLFM1 SMN1 CPNE6 SLC17A8 RUFY3 NGB NEURL KCNK1 CRH NTSR1 MAP2K4 KCNA2 EFNA2 RGS8

GO_GTPASE_COMPLEX NA GNAT2 GNG10 GNAO1 GNAI1 RGS9 GNA13 GNG7 GNG2 RGS6 GNAZ ARRB1 RGS19 GNA15 GNAT1 GNG5 GNGT2 RGS7 GNG11 GNG4 GNAI2 GNA14 GNB1 GNG8 GNG13 GNG3 GNG12 GNAI3 RGS11 GNAT3 GNGT1 GNA12

GO_SECONDARY_LYSOSOME Vacuole formed by the fusion of a lysosome with an organelle (autosome) or with a primary phagosome. NCF2 LAMP1 FTL SQSTM1 NCF4 LAMP2 MAP1LC3A NCF1 ADAM8 NCOA4 FTH1 CRHBP LRRK2 PIK3C3

GO_FILAMENTOUS_ACTIN A two-stranded helical polymer of the protein actin. SHROOM4 PAK1 CD2AP MYO1C FSCN2 ESPN TMSB4X TMSB15B MYO3A TPM4 DPYSL3 MYO6 FLNA LRRC16A FSCN3 MYO1A TPM1 PRKCZ FSCN1 PKD2 ACTG1 TMSB15A FERMT2 RAC3 IQGAP2 FERMT1 TMSB4Y TMSB10

GO_CATION_CHANNEL_COMPLEX An ion channel complex through which cations pass. SCN1A CACNA2D2 RYR1 KCNIP1 KCNE1 CACNA2D3 CAMK2D KCNH1 CACNA1I KCNA5 KCNJ6 KCNC1 CATSPERB KCNC2 SCN4B KCNF1 MICU1 CALM1 KCNMA1 KCNK6 CNTNAP1 SCN7A NOS1 PKD1L3 KCNAB1 HERPUD1 SNAP25 TRPC4 KCNN4 KCNT2 KCNT1 CACNA1D CNGB1 CACNG4 KCNC3 PDE4B CACNA1G KCNS2 KCNA2 CALM2 CATSPER3 FKBP1B MCU DLG2 KCNG2 SCN5A CACNA1H SCN3A EFHA1 CACNG6 DPP6 CATSPER4 KCNQ3 KCNK4 PPP2R4 KCND1 KCNB1 SESTD1 KCNJ2 KCNE3 CATSPER2 AKAP6 KCNB2 SCN9A KCNQ2 CATSPER1 CACNA1C CACNB2 CACNA2D4 CACNA1F SCN10A KCNA10 KCNMB2 KCNMB4 CACNG2 SUMO1 KCNE1L PKD2L1 KCNJ4 AMIGO1 CTTN LRRC26 SCN11A KCNN1 KCNE2 KCNJ16 KCNH2 SCNN1G VAMP2 KCNJ5 SCN2A CACNA1B CNTNAP2 TMEM146 CACNG1 KCNJ11 KCNQ1 CACNB1 SCN2B HCN2 KCNMB1 SCNN1B CACNB4 KCNIP4 KCNS3 ATP2A1 KCNJ3 CACNA2D1 HTR3A TRPC5 KCNG1 SCNN1A KCNG3 KCNG4 KCNQ5 C1orf101 HSPA2 CCDC109B KCNK1 PKD1L1 KCNH4 KCNIP2 KCNQ4 KCNA3 CACNG8 PRKACA CNTN2 CALM3 KCNJ8 KCNA1 KCNJ1 ABCC8 KCNU1 NOS1AP KCNIP3 AKAP9 CACNG7 SCN8A ABCC9 KCNK2 KCNS1 KCNMB3 PDE4D KCNJ14 KCNV1 CACNB3 KCNC4 CACNA1E CATSPERG KCND2 KCNA4 DLG4 KCNAB2 C22orf32 KCNA6 SCN3B SCN4A TRDN CASQ2 KCND3 RYR3 CACNA1A HTR3B SCN1B KCNV2 CACNA1S CACNG3 RYR2 KCNA7 STX1A

GO_PROTON_TRANSPORTING_TWO_SECTOR_ATPASE_COMPLEX_CATALYTIC_DOMAIN A protein complex that forms part of a proton-transporting two-sector ATPase complex and catalyzes ATP hydrolysis or synthesis. The catalytic domain (F1, V1, or A1) comprises a hexameric catalytic core and a central stalk, and is peripherally associated with the membrane when the two-sector ATPase is assembled. ATP6V1H ATP5E ATP6V1B1 ATP5B ATP6V1A ATP6V1F ATP6V1C1 ATP5D ATP6V1E1 ATP6V1B2 ATP5EP2 ATP6AP1 ATP6AP1L ATP5A1 ATP6V1C2 ATP5C1 ATP6V1E2

GO_NUCLEAR_SPECK A discrete extra-nucleolar subnuclear domain, 20-50 in number, in which splicing factors are seen to be localized by immunofluorescence microscopy. ZCCHC18 NOC3L SRY RCHY1 THRAP3 POLDIP3 NSRP1 SPOP WAC PYHIN1 RBM11 APBB1 EAF2 SRP54 ATXN2L MLL5 SON PIAS1 PPIH CIR1 PHF5A RBM27 BCLAF1 SRSF5 SRRM2 PABPN1 PRPF40A DDX39B GATAD2A CLK2 ODZ1 PRPF4 SRSF10 THOC4 FTO RING1 PNN EIF4A3 EPAS1 CBLL1 OGG1 PRKACA CXXC1 EIF4ENIF1 EFTUD2 PLRG1 SF3A3 YTHDC1 THOC2 RBM39 PSKH1 DDX42 NXF1 ALKBH5 LUC7L2 SMNDC1 CTR9 PNISR SARNP PDX1 LUC7L3 PIN1 TFIP11 SRSF6 SMC6 HIF1A TIMM50 GLI2 TUT1 RBM4 EP400 WT1 EAF1 CLK3 FAM206A SRRM1 CCNL1 CDK13 SRSF1 ELL PPP1R8 SETD1A SF3B1 C8orf80 THOC1 PIP5K1A ZNF638 ACIN1 NRIP1 LMNA C1orf124 MAML1 WTAP PRPF18 PRPF31 GATAD2B PRPF19 ZMIZ1 PRPF40B SNRNP70 ZCCHC12 NONO RFWD2 SF3A2 CDC5L ZBTB16 SUMO1 CHTOP APEX1 U2AF1L4 IFI16 MBD1 SRSF3 PLCB1 CD2BP2 BASP1 PPP1CC PQBP1 MAML2 YLPM1 ATOH8 CRNKL1 CDK12 NEK6 WBP11 RBM8A FYTTD1 NXT1 PSPC1 PRPF8 GPATCH2 SRSF4 CWC22 DDX46 U2AF2 PRPF6 CBY1 MECOM GLIS2 METTL3 PIAS2 TRIM69 RBM25 THAP7 MEOX2 MEF2C DDX3X THOC3 WBP4 PPIG SART3 ZNF830 PIAS3 TOPORS CCNL2 AKAP8L CSNK1A1 AFF2 THOC7 POU4F2 C8orf4 HIF3A AKAP17A HIPK1 ZC3H13 RSRC1 PSME4 DUSP11 RNPS1 SRSF2 ZC3H14 MAGOH TRIM22 RNF34 TOE1 GLI3 MAML3 DYRK1A DGKQ CBX4 KIAA1429 TARDBP AGGF1 SETD1B SAP18 CASC3 FAM76B RBM15 RREB1 PATL1 SCNM1 SLU7 ARL6IP4 PRPF3 ATPAF2 THOC6

GO_TRANSPORT_VESICLE Any of the vesicles of the constitutive secretory pathway, which carry cargo from the endoplasmic reticulum to the Golgi, between Golgi cisternae, from the Golgi to the ER (retrograde transport) or to destinations within or outside the cell. COL7A1 SYNRG ERP29 KDELR3 RPH3A SYT9 TRIM9 SYT4 ICA1 SPG21 SCAMP2 BRSK1 GRIN1 SYT12 VTI1B MYRIP RAB3B SNAP25 DDHD2 VAMP7 VANGL2 YIF1B AIMP1 GRIA1 SEC24C HLA-H RAB11A RAB3C MUTED SV2B VDAC1 SYT10 SYNGR1 HLA-DRB5 COPB1 CNIH2 HLA-DQA1 SORT1 BET1 FOLR1 BCL2L1 RABEPK SYNPR CUZD1 CDK16 YIPF1 HLA-DQB1 VAMP2 C1QTNF5 SVOP CRISPLD2 NRSN2 SYTL4 SLC18A1 GAD2 SYTL5 SEC23IP TGOLN2 ASTL ANKRD27 VMA21 SAR1B RAB1B LRRK2 SLC32A1 HAP1 FGFR4 SERPINA1 TMED2 STX4 SPRED2 LAMP1 SREBF2 EXOC3L1 SEC22B COPE MTMR2 PI4K2A RAB27B HLA-G SYT2 IER3IP1 CD74 RPH3AL CPA3 RASSF9 SYT7 DNAJC5 SCAMP1 HLA-B TPRG1L LMAN1 SYNGR3 SLC17A8 TOR1A DPYSL3 SYT11 CD55 YIPF3 DNM1 ZNRF1 KCNK9 STX2 SNCAIP NTS SEC24B COPS4 SLC2A4 SEPT5 PCSK9 AP1AR CD59 HLA-DRB3 SLC18A3 AP3S1 HLA-F LGI3 ATP6V0D1 DNM1L STON2 SEC24A SEMA4C AP2A1 MT3 HCK STX1A YIPF5 SURF4 SEC23B RAB11FIP5 APBA1 OTOF PLIN3 SLC18A2 COPG2 TRIP11 YIPF6 GOLIM4 C2orf40 CHGA SIPA1 LDLRAP1 DLG4 C12orf39 GALNTL2 BGN AQP2 APH1B ARCN1 SNAPIN SYT3 IL33 CNIH TMEM30A SEC31A COPS5 PCSK2 STX1B COPB2 AQP6 SCAP STXBP5 APP SCGN LMAN2L CHP ABCC8 SYN3 STX17 NRSN1 CPE STX11 AFTPH SFTA2 F8 DISC1 COPZ1 SNTB2 FGFR3 PDE4B DOC2A SEC13 SYT5 LMAN1L M6PR LMAN2 FGFRL1 SV2C SLC17A6 VDAC2 COPZ2 HLA-A DBH RAB26 SLC30A8 RAB11B GOSR2 MME KLHL12 PRG2 TGFA DMXL2 VTI1A OVGP1 C20orf30 INS RAB3D IGF2R UNC13D SYPL1 FURIN CLTA VAMP1 ADC KIAA1244 TRAPPC4 HLA-DPA1 HLA-DRB1 PTPRN NCALD DDC KDELR1 RAB6A CTTNBP2 HLA-DRB4 CLCN3 COPG NPTX1 HLA-DRA KIAA0368 PLDN SNCA VAMP3 APBA2 CLTC SYTL1 HLA-DQB2 VAMP4 AP1G2 TMEM163 SYN2 RAB14 HLA-DPB1 ATP13A2 MCFD2 B2M YIPF2 SLC2A8 DGKI SEPT6 RAB9A SSPN AREG CLTCL1 CTSZ SCG3 ATP7A PPT1 HLA-E HLA-C GRIN2A GABRA2 COPA SLC40A1 GIPC1 SEC23A USO1 AMPH TEX261 HLA-DQA2 TH GOLGA5 TMEM187 RAB3A RAB8A NRGN SYT1 GOPC GOSR1 STX19 C14orf1 CTSC IGF1 CLN3 TMED3 RAB27A KDELR2 TMEM168 PTPRN2 SYP VGF STX3 CAV2 CA4 WDR7 STX5 SYT13 ATP6V1G2 SLC17A7 PLEKHF2 SV2A SYNGR2 RABAC1 SLC30A3 SYTL3 DTNBP1 MFF HCRT RAB5A SLC17A5 SEC31B NDEL1 TMEM184A YKT6 CNST PCSK1 SYPL2 F5 TMED9 SYT6 SYT17 CLTB SEC24D SYNGR4 DEFA5 YIF1A GOLGA2 TMED10 CEACAM1 STEAP2 SYTL2 BLOC1S3 SYCN DRD2 SCAMP5 SLC6A17 CNIH3 AP1S1 TMED7 SYN1 C19orf26 SREBF1

GO_CONDENSED_NUCLEAR_CHROMOSOME_CENTROMERIC_REGION The region of a condensed nuclear chromosome that includes the centromere and associated proteins, including the kinetochore. In monocentric chromosomes, this region corresponds to a single area of the chromosome, whereas in holocentric chromosomes, it is evenly distributed along the chromosome. MIS12 MIS18BP1 CENPA BUB1B LOC728637 REC8 SS18L1 SGOL1 AURKC BUB1 CENPC1 SUV420H2 PLK1 CCNB1 SUV420H1 AURKB NDC80 DSN1 AURKA

GO_MULTIVESICULAR_BODY A type of endosome in which regions of the limiting endosomal membrane invaginate to form internal vesicles; membrane proteins that enter the internal vesicles are sequestered from the cytoplasm. RAB27B ATP13A2 CD300LG LAMP1 EGFR RAB27A HDAC6 ZP3 NDFIP2 CD63 TPT1 ZP2 CRHBP SLC2A4 BACE1 LRRK2 RAB11A PGA4 GJA1 SFTPB HGS KIAA0368 SLC40A1 HMP19 PGA5 SLC17A8 SFTPC TSG101 CST3 CTSH PGA3 STEAP3 LRAT CD74 NAPSA CD79A BST2 PMEL

GO_MCM_COMPLEX A hexameric protein complex required for the initiation and regulation of DNA replication. MMS22L MCM4 MCM3 MCM2 MCM8 MCM5 MCM6 TONSL MCM7 MCMBP MCM9

GO_SPLICEOSOMAL_COMPLEX Any of a series of ribonucleoprotein complexes that contain snRNA(s) and small nuclear ribonucleoproteins (snRNPs), and are formed sequentially during the spliceosomal splicing of one or more substrate RNAs, and which also contain the RNA substrate(s) from the initial target RNAs of splicing, the splicing intermediate RNA(s), to the final RNA products. During cis-splicing, the initial target RNA is a single, contiguous RNA transcript, whether mRNA, snoRNA, etc., and the released products are a spliced RNA and an excised intron, generally as a lariat structure. During trans-splicing, there are two initial substrate RNAs, the spliced leader RNA and a pre-mRNA. DHX38 TXNL4B RBM25 MAGOHB LSM7 PABPC1 HNRNPM WBP4 AKAP17A KIAA1967 PRPF4B YBX1 SF3B4 SNRNP35 HTATSF1 ZCCHC8 SRSF2 PRPF38B RNF113B DHX8 SNRNP27 ZCRB1 DHX15 GEMIN2 ZMAT5 MAGOH AGGF1 C14orf102 SNRNP25 PPWD1 CWC15 SNRPG LOC100130932 LSM4 C2orf3 RBM41 API5 SNRPA DQX1 PRPF3 SCAF8 RBMX SART1 RBMX2 SLU7 SNRNP48 PRPF19 XAB2 PRPF31 PRPF18 SYNCRIP SF3A2 KIAA1875 CDC5L SNRNP70 PRPF40B SNRPE U2AF1L4 LSM6 HNRNPH3 SF1 GPATCH1 SNW1 TTF2 CRNKL1 RALY PPIE SNRPN SNRPB2 RBM8A WBP11 RBM17 LGALS3 LUC7L SNRPC HNRNPK SNRPB CWC22 U2AF2 SNRPD3 AQR PRPF8 SNRNP40 HNRNPA1L2 PPIL1 TXNL4A PRPF6 DGCR14 NAA38 HNRNPR CWC27 RNF113A IVNS1ABP SF3A3 PLRG1 EFTUD2 SNRPA1 HNRNPF UPF1 DHX16 LSM2 LUC7L2 SMNDC1 PRPF38A BCAS2 USP39 FRG1 RBM28 TFIP11 HNRNPU SNRPD2 HNRNPA2B1 LUC7L3 LSM3 SNRNP200 SF3A1 HSPA8 DDX41 ADAR C19orf29 RBM5 DDX39A SRRM1 CDC40 HELB RBM22 SF3B1 SF3B2 PPP1R8 SRSF1 SNRPF SUGP1 PRCC HNRNPA1 RNPC3 DDX23 PTBP2 SF3B3 HNRNPH1 LSM5 HNRNPA3 WAC DHX32 DHX35 PHF5A SREK1 SON PPIH NHP2L1 CTNNBL1 WDR83 HNRNPC SF3B14 ZRSR2 SKIV2L2 SYF2 DDX39B PRPF40A ZNF326 RHEB SRRM2 ZRSR1 SF3B5 THOC4 PDCD7 ISY1 PRPF4 BUD31 EIF4A3 DDX5 PNN SNRPD1 PPIL3 PRPF39

GO_REPLISOME A multi-component enzymatic machine at the replication fork which mediates DNA replication. Includes DNA primase, one or more DNA polymerases, DNA helicases, and other proteins. POLA2 POLE4 POLE3 PRPF19 XPA RPA2 POLE PURA PRIM2 SMARCAL1 PURB TONSL PLRG1 ERCC5 PCNA POLD3 POLE2 POLD4 PRIM1 RPA1 MCM3 POLD1 POLA1 RPA4 RPA3 BCAS2 CHRAC1 CDC5L HELB

GO_ER_TO_GOLGI_TRANSPORT_VESICLE_MEMBRANE The lipid bilayer surrounding a vesicle transporting substances from the endoplasmic reticulum to the Golgi. HLA-C HLA-E HLA-B LMAN1 VTI1B SEC31B HLA-DRB4 SEC23IP SEC23A HLA-DRA HLA-DQB1 STX5 CD74 SEC23B HLA-DPA1 AREG HLA-DRB1 SEC13 TMED2 HLA-DRB5 VTI1A STX17 CNIH3 HLA-DQA1 CNIH2 SREBF2 SEC24A SEC22B TMED7 HLA-DPB1 SREBF1 FOLR1 B2M MCFD2 HLA-G HLA-A GRIA1 SEC24D USO1 SEC24C CNIH SEC31A VMA21 SEC24B HLA-H SAR1B TMED10 HLA-DQA2 GOSR2 SCAP CD59 TGFA HLA-F HLA-DQB2 HLA-DRB3

GO_CUL4_RING_E3_UBIQUITIN_LIGASE_COMPLEX A ubiquitin ligase complex in which a cullin from the Cul4 family and a RING domain protein form the catalytic core; substrate specificity is conferred by an adaptor protein. DCAF13 CUL4A CUL4B DCAF8 DDB2 PLRG1 DCAF6 DCAF10 DCAF12 DCAF17 GLMN RBX1 ERCC8 RNF7 TRPC4AP DTL DCAF4 DCAF16 DCAF11 FBXW5 CDKN1B CRBN DDB1 DCAF5 DCAF7

GO_DENDRITE_MEMBRANE The portion of the plasma membrane surrounding a dendrite. ATF4 C20orf103 GABARAPL1 ITGA8 PALM TRPV1 GPER GRIA1 HPCA DDN OPRD1 SHISA9 WLS SGCE ATP2B1 OPRM1 TACR3 GABRG2

GO_MEDIATOR_COMPLEX A protein complex that interacts with the carboxy-terminal domain of the largest subunit of RNA polymerase II and plays an active role in transducing the signal from a transcription factor to the transcriptional machinery. The mediator complex is required for activation of transcription of most protein-coding genes, but can also act as a transcriptional corepressor. The Saccharomyces complex contains several identifiable subcomplexes: a head domain comprising Srb2, -4, and -5, Med6, -8, and -11, and Rox3 proteins; a middle domain comprising Med1, -4, and -7, Nut1 and -2, Cse2, Rgr1, Soh1, and Srb7 proteins; a tail consisting of Gal11p, Med2p, Pgd1p, and Sin4p; and a regulatory subcomplex comprising Ssn2, -3, and -8, and Srb8 proteins. Metazoan mediator complexes have similar modular structures and include homologs of yeast Srb and Med proteins. THRAP3 MED24 CDK8 MED31 MED1 MED15 MED16 MED13L MED4 MED26 MED8 MED27 MED11 MED17 MED18 MED29 MED22 MED9 MED20 MED12 CCNC MED14 MED28 MED12L MED10 GLI3 PPARGC1B RBM14 MED6 MED19 MED30 MED13 MED21 MED7

GO_FILOPODIUM_TIP The end of a filopodium distal to the body of the cell. VIL1 CIB1 NLGN1 MYO10 UBE2K MYO5A FZD3 EPHB1 AP2A1 OSBPL3 TTYH1 FMR1

GO_CORNIFIED_ENVELOPE A type of plasma membrane that has been modified through addition of distinct intracellular and extracellular components, including ceramide, found in cornifying epithelial cells (corneocytes). LCE3A LCE5A KAZN CSTA EVPL LELP1 LCE2B C1orf68 SPRR4 SPRR2D SPRR2E LCE1D LCE1E SPRR2G LCE1F SPRR1A SPRR3 CDSN CRCT1 SCEL LCE2C TGM1 IVL LCE3C LCE4A ANXA1 LCE1A SPRR1B LCE3B LCE3D LCE1B RPTN CST6 SPRR2F CNFN LCE3E DSP PRR9 LCE2A LCE2D LCE1C SPRR2A EVPLL SPRR2B HRNR LOR

GO_HOLO_TFIIH_COMPLEX A complex that is capable of kinase activity directed towards the C-terminal Domain (CTD) of the largest subunit of RNA polymerase II and is essential for initiation at RNA polymerase II promoters in vitro. It is composed of the core TFIIH complex and the TFIIK complex. ERCC2 MNAT1 CCNH MMS19 ERCC3 GTF2H1 GTF2H2 CDK7 ERCC5 GTF2H3 GTF2H4

GO_SEMAPHORIN_RECEPTOR_COMPLEX A stable binary complex of a neurophilin and a plexin, together forming a functional semaphorin receptor. PLXND1 PLXNA2 NRP1 PLXNB3 NRP2 PLXNA3 PLXNB1 PLXNA4 PLXNC1 PLXNB2 PLXNA1

GO_MITOCHONDRION A semiautonomous, self replicating organelle that occurs in varying numbers, shapes, and sizes in the cytoplasm of virtually all eukaryotic cells. It is notably the site of tissue respiration. GHITM RPS6KA6 ATP5J PPARGC1A UBB MFN1 ATG13 DDX3X MRPL27 OXA1L SPARC COX17 GSK3A STOM MTRF1 FOXRED1 GLYATL1 CREB1 NNT SNN NOS1 NIPSNAP1 TMEM126B OXSM MRPL51 NDUFA5 HAGH VARS2 MPO PPP3R1 ACOT7 ECHS1 NADKD1 GSR ECI1 DNAJC15 TIMM13 QDPR SDS POLG2 ABCB7 MPV17L2 HOXB9 SLC25A35 GRAMD4 NDUFS3 RPP14 VDAC1 ROMO1 DCPS NDUFB2 ECH1 DLAT NDUFB6 CSDE1 ETHE1 FLAD1 DMGDH UBIAD1 CDK1 PTGES2 DECR1 C19orf70 NUDT8 SLIT3 APEX1 GLYATL3 NBR1 JTB NAGS SIRT4 LETM1 CAPN10 PDE2A C3orf15 C11orf83 FPGS HAO2 MAPK3 MRPS15 VHLL CPT1A MLYCD MRPL11 UQCRFS1 MTIF3 PDK1 NDUFA11 BECN1 ABCB10 MRPL52 BCAT1 EFHA1 COX7A2P2 COASY DCAF5 RAB38 MUTYH TRIM39 P4HA1 LYRM5 PEMT ACAD10 ACSM1 NDUFA4L2 SDHA UROS SFXN4 MRPS18B RDH13 FAM73B PRELID1 TOP1MT QTRTD1 AGXT2L1 C2orf18 NDUFV3 MAFF LRRC10 PPP1R15A FXN MPV17 OGDHL CYP1A1 MMAA FEN1 THOP1 SLMO1 HERC2 PRODH CYBB POR FIBP NR3C1 PKM2 SERAC1 C3orf78 PTCD1 MTCH1 ABHD10 NIT2 TOMM5 SLC8A3 C19orf12 TSFM PDP1 CAT SLC9A6 GPD1 RHBDD1 ABL1 MRPS28 MRPL20 ZDHHC8 TOMM40L GCK STARD6 LRP5 MAPK10 AGPS MRPL50 MUL1 IDE PNPLA7 RSAD2 AGXT2 SLC25A37 DNAJA3 DAOA COX4I2 YME1L1 NLN TMEM8B UQCRHL CYP2D6 COQ6 COX16 MRPS27 MAT2B CCBL2 FDX1L MTX1 STARD4 CYP11A1 MGST1 TOMM34 MTRF1L MRPL12 PITRM1 MRPS21 GPX4 SIRT5 THNSL1 RAI14 VAT1 SLC25A47 CPS1 ACACB MCART2 ETFA MRPL3 COMTD1 OAS2 CHCHD6 COA5 BCL2L13 TSTD1 CLYBL ASAH2 TTC19 C22orf29 SLC25A42 ATXN3 FASTK ACADM THG1L SLC25A14 LRRC59 HIF3A BRAF RPS15A NME4 SLC25A31 CLPB IDH1 SMURF1 NDUFB11 BFSP1 DDAH2 ALDH3A2 SP140 AGK MAPK14 TCIRG1 BPHL PARP1 GTPBP3 ME2 FAM73A TUSC3 HRSP12 RAB3D GLS PYCR2 RRP15 PNPT1 RNMTL1 DUSP26 TIMM17A KIAA0141 TXNDC8 APOOL IMMP2L LETM2 CYB5A HSD17B8 TRAF6 SSBP1 GSTZ1 PACRG NOP14 DHX29 BOLA1 FKBP8 TMEM186 ACSL1 LGALS3 SLC25A11 C14orf159 FASN DHX57 MRPL18 DHFRL1 GLYATL2 PYCARD IRG1 PSMB3 MARCH5 ALDH2 CXorf23 NTHL1 QRSL1 NVL ATP5H MRS2 TGM2 NDUFA6 ACP6 LIAS LDHD KRT5 MPST ACSF2 PFDN4 CCDC56 TMEM102 UCP2 MRPS12 AGAP2 OPA3 UXS1 MRPL13 PPP3CC TMLHE SLC25A32 BCAP31 COX4I1 ARMS2 MYO19 DNM1P46 SLC25A2 CASP1 MAPK9 USMG5 RHOT2 NRGN NDUFB9 NDFIP2 CCDC51 NDUFAF1 SLC3A1 WDR93 BZRAP1 HSPA1L FAM5C PMPCA CARKD GPER CPT1C MRPL40 COX5A TOMM22 HSD3B2 ARMC10 MTIF2 FDXR ME1 COX6B1 TACO1 THEM5 LDHB UQCRC2 MARS2 GMPPB TRAF3 SND1 ICT1 HADHA C12orf62 C6orf136 ATG4D TRMT1L ELK1 TMBIM6 ACSM3 MRPS18A MOBP HRK DTYMK YKT6 CASP2 TIMM22 C7orf30 PTCD2 ADO SLMO2 PTRH1 SUCLA2 ABCF2 SOD2 CYB5R1 DUS2L HIGD2A HSPA9 ALDH6A1 PRKACA TOMM7 MTCP1 MTO1 SPG20 PNKP MRPL44 ARL2BP ACSL5 CHCHD4 MAOB CASQ1 ARSB CASP9 C2orf47 RAI1 BCO2 KMO PRSS35 RAF1 MRPL38 TYMS NAPG SPG7 RIPK3 COX7B2 RPS14 RARS2 OMA1 C14orf119 ENOSF1 SLC22A4 TNNC1 C9orf89 MARK2 CISD2 TNRC18 ACN9 ATP5J2 FAHD1 ABCC12 BCKDHA CRAT NDUFS2 TIMM9 STAR AGPAT5 ATP5C1 G0S2 MRPL15 PDPR SLC25A44 DNAJC19 FAM32A SIRT3 BCKDK ADPRHL2 HSD17B10 PPOX MSRA TST PYCR1 PDHX ETFDH HOGA1 SDHD PLN SLC27A3 AADAT GLDC WBSCR16 MRPS5 REXO2 MRPS26 STOML2 MRPL28 EARS2 CD3EAP COX7C NOL7 COX11 COX15 RAB7L1 CPT2 DAP3 SEPT4 HS1BP3 MRPL45 TNFRSF1A C9orf46 DIABLO LIG3 PARL NDUFA2 SELRC1 RAB1B DUT GRSF1 NT5M HAP1 SLC25A43 LARS2 KIAA1279 GDAP1 COQ10A NDUFB7 NOX4 ALKBH1 ECHDC2 C18orf55 PTPMT1 C4orf49 RPUSD3 PACS2 BIK DNAJC5 ACSM4 STAP1 PDF GLYAT DDIT4 RAB8B KIF1B SMCR7 ARL2 MCCC1 HCCS LETMD1 GPT2 CTU2 METAP1D GNL3L PTEN PAM16 MRPL48 SMCP GLRX2 ATP5L2 GLRX C1QBP AGTPBP1 TFAP2C NEU4 RMND1 ZNF205 ECI2 TIMM50 NDUFS4 ACSL4 RPS3 NDUFA3 GNRH1 NOA1 AGXT CHCHD3 BAG5 SLC25A30 LACE1 RANBP2 MRPS10 TRIT1 GCDH MRPL35 GPAM PDK2 DNM1L DHX32 C17orf42 UCP3 SUCLG2 NOL6 COQ9 NDUFS8 TIMM17B MPG RAB11FIP5 FASTKD5 TERT MAGEA11 MDH1 ACAT2 ATP6V1E1 WWOX PRR5L YARS2 HIVEP1 SLC11A2 SLC25A45 HSD3B1 LGALS12 CCDC111 TFAM BNIP1 UQCR11 C22orf32 ADAP2 FBXO7 RTN4IP1 ISCA1 BCL2L2 IFI27 BLOC1S1 YRDC PALLD MECP2 SH3BP5 ZADH2 CYP24A1 SHMT2 PARS2 MCL1 TMEM11 RAP1GDS1 C9orf102 TXNRD2 NDUFA4 TRMU DHRS2 BSG TMEM14C SIRT1 MCCD1 NLRP5 LACTB2 ASB9 TP73 BCS1L ALAS2 PPM1E MTG1 AMT SLC25A18 GM2A COX5B BAD MAOA TIMM44 FOXO3 VDAC2 FDX1 HIBADH ATP5L ADHFE1 FUNDC2 NDUFA7 UBA1 ABAT SRP19 SLC25A10 IDH3B SLC25A5 C10orf2 BCKDHB MICU1 SYNE2 LIPT1 ACACA VARS THEM4 NUDT9 CMC1 NARS COQ5 ABCG1 EXOG NDUFA12 ATP5G1 ACSM2B MIPEP APEX2 CYP2E1 COX7A2L PRKCA C17orf90 PLA2G6 PRDX4 HDDC2 COX7A2 NT5C3 DYNLL2 MRPS2 TDH ACBD3 AARS2 ANKZF1 TUFM HADH NDUFB10 ATP5D BCOR MTHFD2L GNPAT ALKBH7 ERN1 TP53AIP1 PERP NDUFAF3 USP30 ARG1 NUDT1 YWHAB TAMM41 ACOT8 CECR5 ADCK3 CRY1 MRPL46 MACC1 CABS1 CLIC1 ERAL1 AS3MT BDH2 KYNU GSTK1 USP48 COX7A1 PNKD OCIAD2 NME3 ACSF3 HSDL1 POLD3 PLA2G15 HSCB PDK4 SARDH FH VPS13C PDK3 PCCB HLCS IMMP1L AKAP1 ADCK1 GK PHB2 NT5C ATP5I GSDMC PARK2 CYP11B2 TMEM65 TXNL1 PGS1 SHC1 ABCA12 NT5DC3 ATPAF1 FHIT ACAD9 BRI3BP GIMAP5 PTCD3 TRAK1 MRPL41 HSPA2 LIG1 SERHL2 CS YWHAH D2HGDH MRPL14 AMBRA1 PDE12 IFI6 FYN UQCRQ MRPL4 CYP11B1 FARS2 ADH5 NDUFS6 FAM82B SYBU GATC SOX4 ELK3 TFB1M ENDOG TSPO EHHADH PRELID2 TRIAP1 CBR4 SLC25A20 DIAPH2 NXNL1 ARG2 CHMP2B ACAD8 GABARAPL1 SARM1 FASTKD2 DLD ECHDC3 ALDH1L1 APOPT1 SQRDL NUDT2 BTD AKAP8 GBAS GPN1 ANXA10 SDHB SHMT1 NDUFB1 CHCHD1 TBRG4 NCBP1 DNM2 SLC25A15 UNG DNAJA1 HIGD1A MFF LOC100505876 PTPLAD1 NDUFC2 UQCRB RAB35 SDHAF2 LRRK1 SFXN5 BMF SYNJ2BP ABHD11 PROSC MRPL22 SLC27A2 GFM1 NFKB1 CDS2 ACO2 PTPN11 MTOR CHCHD10 DHRS4 GOT1L1 SLC25A39 PHB ABHD6 MRPL16 PMAIP1 COX4NB HSDL2 NDUFB4 BRP44 TAT PHYHIPL OGT TRAK2 CCT7 C15orf48 TRMT2B RRM2B CHCHD2 TMEM143 SLC25A16 C1orf177 STARD3 SGK1 MSRB3 CYB5R2 SMCR7L TOMM20 KIAA1967 C15orf62 GPX1 CASP4 OAS1 NDUFB8 SPRYD4 IDH3A BDH1 ALDH5A1 ATP5O DDX28 NAT8L TRMT61B CCDC90A NUDT6 LIPF MRPL32 MTERFD1 SLC25A27 ATAD1 MPDU1 RPL34 DLST DDAH1 ATPAF2 GLUL BCL2L1 DGAT2 TDRD7 KANK2 LYPLA1 C7orf73 FGR CRLS1 ACO1 MRPL1 YWHAQ CYBA PSAP PTRF HCLS1 SLC25A19 RHBDL3 CLPX TIMM8B ADSL COX6A1 MRPL2 C16orf61 PGR ADCK4 LRRK2 DARS2 ARGLU1 NFS1 CHDH RNF185 TUSC2 SLC25A40 ALDH7A1 SPHKAP C10orf129 PPP2R1A GSTP1 SOD1 MTUS1 C20orf72 UQCC NSUN3 XPNPEP3 TOMM40 PFDN2 SLC25A34 MTHFD2 MRM1 SLC25A46 RIPK1 DDX6 PARP9 TXN TPO C10orf10 DNLZ NME6 CCDC58 TMEM223 SLC44A1 DNM1 GOT1 CPT1B TIMM23 OXNAD1 MRPS18C MRRF PRKCE SNPH NUDT13 MRPS25 PPIF P2RY1 HEBP2 GDF5OS LDHAL6B PLA2G4B MTFP1 BCL2L11 DNAJC30 PINX1 FAM54A C6orf125 CYP17A1 HK2 HINT2 MRPS7 ACOX1 KRAS CYC1 MSTO1 PPP2CA BID MYL10 NDUFA1 MPV17L PDHB C19orf79 IARS2 SDSL ACOT9 DNM1P34 RFK LOC100130097 OPA1 FAM82A2 GRPEL2 SLC25A23 CYP27A1 COX6A2 KARS SH3GLB1 MUT AK4 AKR1B15 GLYCTK IVD MAVS SLC25A36 LAP3 DHODH TRMT1 HCFC1 SECISBP2 AIFM2 NDUFA13 GLS2 ACSS1 ACSM5 QTRT1 OGG1 C17orf89 CTPS2 COL4A3BP MRPS17 TOMM70A AIFM1 NOS1AP METTL12 TRNT1 STX17 SERHL DGUOK RAB40AL AP2M1 ZNF202 PLA2G4A AURKAIP1 NARS2 ATAD3A YBEY GTPBP8 EPHA4 TXNRD1 SLC9A1 STARD7 TMEM173 ADCY10 MRPS14 HADHB COQ3 CYP27B1 GIMAP8 FLVCR1 DYNLL1 SCO2 IFIT3 TTC35 RGS2 SFXN3 RAB11B FAM54B DNAJC27 HARS2 NDUFS7 YJEFN3 RAB24 ALAS1 RARS CHCHD7 OAT SRC SURF1 EFHD1 HEMK1 MRP63 ISCU ABCA8 NOL3 MOSC2 ACADVL VAMP1 RAB32 RHBDL1 ADC TCHP ANXA6 C12orf65 C2orf56 C10orf58 SLC25A22 ATF2 CROT GATM TIMM8A TIMM10 AARS PDHA1 LYN MRPL36 HDHD3 PDSS2 MRPL42 CA5A MCCC2 MRPS36 MRPL23 COX6B2 RPUSD4 FAM162A PET112 MRPL37 C10orf67 AK2 ABCG2 COMT MRPS30 MRPL49 YWHAZ MTX3 ASS1 LONP1 TPP1 SLC25A13 WASF1 KIAA0391 AGR2 METTL17 PPP1CC NLRX1 IBA57 SFN LOC347411 CASP8AP2 RILP PAK7 TP63 C7orf44 BCAT2 NDUFB5 ACAD11 MTHFD1L COX19 PIF1 ADAM12 NUBPL CARS2 OSGEPL1 MRPL24 OLFM4 MAPK8 RNF5 ABCB6 NSUN4 DPYSL2 CYB5B NDUFAB1 SLC25A28 AGPHD1 C14orf2 WARS2 GLUD1 LACTB PLIN5 CRYM COQ10B CYB5R3 CCDC90B SCP2 GZMB MRPL21 DUSP21 PIN1 DNM3 NEFH SLC25A41 CHPF PCK2 PRDX1 L2HGDH PPP3CA TMEM160 PPARGC1B SLC25A12 APOA1BP SFXN1 CYP2D7P1 DUSP18 MRPL9 RHOT1 EYA2 MRPL47 GLUD2 PHYH ATP2A1 PRODH2 AKAP10 NDUFAF2 NIF3L1 ATP6V1A OGDH CLN3 ACSBG2 NUDT19 DACT2 ACADL ATP5G3 GFM2 KCNJ11 UACA PPL CASP8 MRPL43 NDUFA8 OCIAD1 TIMMDC1 SLC25A26 C4orf52 SLC25A38 HAX1 ETFB SRI ABCE1 ATP5SL KIAA1683 AGXT2L2 CKB PRDX3 METTL20 PPM1K FTMT SLC25A25 STARD13 NDUFV2 DHTKD1 GK2 COX18 DHX30 C5orf63 GGNBP1 P2RY12 SUOX ALDH1B1 COX8C NDUFAF4 NME2 TDRKH GLOD4 CDK5RAP1 FUNDC1 LYRM4 ALDH1L2 KCNJ8 EFHA2 C21orf33 MMACHC BNIP3 NTSR1 FASTKD1 STARD5 CTSB C7orf55 VDAC3 TMEM70 PON2 ARMCX3 PARK7 DBT SLC25A3 MCU MLXIP HMGCL GSK3B MTX2 RPL35A ALDH4A1 MAPK12 RPUSD2 STK11 MRPS11 ECSIT UQCRH AGMAT CLU MDH2 MRPL19 ACADSB PPP1R13B CKMT1A OTC FAM65B MMADHC MRPS24 SLC16A1 RAB11A NDUFS5 SPNS1 PDSS1 CYP1B1 C6orf57 SPATA18 PSTK NMNAT3 CIDEA CPOX LYRM1 YWHAE RG9MTD1 DRG2 E2F1 C8orf38 KLK6 FASTKD3 ILF3 TRMT5 ATP5A1 C21orf2 ACOX3 NDUFV1 HSPB7 HK1 CA5B CISD1 MYOM2 ATIC MTHFD1 PDHA2 ACYP2 CTU1 TFB2M CMPK2 TOMM20L PANK2 RAD51 MRPL30 DCAKD MTPAP TMEM126A XRCC3 GTPBP10 MRPS6 MRPS35 CYCS C20orf7 QARS FEZ1 SUCLG1 MRPS23 CISD3 MACROD1 NIT1 PINK1 MCEE MRPL55 COX10 PI4K2A C17orf76-AS1 MOAP1 SAMM50 TXNDC2 ACADS ATP5B CERK PC SLC25A29 VRK2 MSRB2 CKMT2 FKBP4 AKR7A2 GPAT2 SLC25A21 TMTC1 NGB MRPL54 UCP1 YWHAG GOLPH3 PABPC5 BCL2A1 ACOT13 NMT1 BRP44L MYH10 MRPS9 HEATR1 GOT2 CCDC109B C3orf23 FAM72A BRD8 SESN2 IMMT DNA2 C12orf10 CDK7 MCAT ATAD3B MTCP1NB GRPEL1 TXN2 ZMIZ2 PPA2 AMACR IDH2 MTERF MTFMT ACOX2 CRYAB AK3 DNAJC11 OXCT2 PRKAR2B ATP5E ACSL6 CLIC4 MTFR1 TP53 CAPRIN2 OXR1 BAK1 FAM110B SLIRP PCBD2 MT3 CIAPIN1 AIFM3 CEP89 ATP5S SLC25A17 SCO1 SLC25A33 LRPPRC SORD HIBCH GADD45GIP1 DHRS1 MFN2 FADS1 HSPD1 PCCA UQCR10 SIAH3 ATP5G2 C7orf10 REEP1 SPATA5 HTRA2 MRPS16 GCAT CKMT1B SPTLC2 C18orf19 CHCHD8 CAPN1 RNASEL RNF144B AASS LIPT2 NDUFC1 MYCBP PTS PARG HSPA5 SCCPDH FXC1 ABCA9 OXCT1 TAZ BCL2L10 FSIP2 AKT1 MINOS1 MTHFS LOC100652748 SLC8A1 DISC1 RBFA IKBKE DMPK MMP2 SLC24A6 TP53BP2 LYRM7 NDUFA10 SNCB FECH ATP5F1 PGAM5 PUS1 BOK FBXL4 SLC9B2 MECR NDUFS1 TBC1D15 C12orf5 SFXN2 ATPIF1 ACSL3 HSD17B4 MTERFD2 LPIN1 FTSJ2 ATP5EP2 SUPV3L1 ALDOC VASN MRPS22 ATP7B TARS2 GARS LYRM2 MRPL33 PMPCB SARS2 MOSC1 BBOX1 MAP1LC3B MRPS33 ACSS3 RDH14 PLSCR3 ACAA2 WDR81 CNP TRAP1 KIAA0564 PRDX5 RAD51C RUNX1T1 VHL HMGCS2 ME3 BLOC1S2 TK2 MRPL17 PPP2R2B ST20 CAMK2A XAF1 SLC25A24 PI4KB SNCA GLRX5 STXBP1 HARS GBF1 SDHC GCSH SLC25A1 MTCH2 COX6C MRPL34 ESR2 AFG3L2 BAX STAT3 MMAB MRPS34 DHX34 SDHAF1 EEFSEC SLC25A48 PTRH2 GTPBP5 RPS6KB1 PDP2 PNPLA4 COQ4 MAPK1 PSEN1 HSH2D FIS1 GPD2 C19orf2 AUH HK3 HSPE1 SACS BCL2 C6orf203 ERBB4 TXNIP ABCB8 PLD6 GUF1 HSP90AB1 TOMM6 GNB2L1 RPUSD1 COX7B POLDIP2 PIN4 ABCD3 PECR FITM2 MRPL39 ARMC1 MAP2K2 APOO TH MRPL10 GNG5 TFDP1 RSAD1 NDUFC2-KCTD14 ACAT1 ANXA1 HAUS3 DEGS1 UQCRC1 C1orf31 MRPS31 ACSM2A COX8A MCART6 ABCD1 NDUFB3 BNIP3L MTERFD3 POLG SLC25A4 DIMT1 ALKBH3 FAM36A MRPL53 CHCHD5 COQ7 SPATA19 NFU1 RHBDL2 COQ2 FANCG TAP1 CLPP ACOT2 TRIM31 ALDH18A1 MYOC MAP2K1 GFER NDUFA9 HSPA1A NAIF1 ISCA2 PISD GJA1 SLC25A6 NME1 FOXO1 DAO MCART1 SOX10 BLID NDRG4 POLRMT PPTC7 PET117 MAPK8IP1 ATCAY ELAC2 BBC3

GO_PLATELET_DENSE_GRANULE_LUMEN The volume enclosed by the membrane of the platelet dense granule. FAM3C SPP2 CTSW TIMP3 APOH CLEC3B RARRES2 ECM1 LGALS3BP CDC37L1 ITIH4 ITIH3 SEPP1 SERPINA4

GO_PORE_COMPLEX Any small opening in a membrane that allows the passage of gases and/or liquids. VDAC2 C8B BAX C8G C6 C5 C7 VDAC1 VDAC3 BCL2 C9 NUP62 CD34 TOMM40L BAK1 TOMM40 C8A

GO_ACTIN_BASED_CELL_PROJECTION A cell projection supported by an assembly of actin filaments, and which lacks microtubules. SRCIN1 BAIAP2 PDZK1 CD302 AOC3 CNP GIF LHFPL5 TGFB1 EZR PODXL ITGAV MPP1 CA9 CLCA1 TRPV4 RAPGEF3 NFASC MYO3A UTRN DFNB31 INPPL1 ANTXR1 IGF2BP1 TMC1 AQP5 ABI1 TSPEAR FMR1 LRRK2 CIB1 HOMER2 STRC PROM2 PDPN VCAM1 SLC9A3R1 BBS2 ANKS4B AKR1B1 ACTA2 TWF1 CDK5 CDC42 PCDH15 RDX STRCP1 TTYH1 CDHR2 LY6G6D KITLG MSN PROM1 VIL1 IQGAP2 RAPH1 NF2 ITGA3 DOCK4 GRXCR1 FSCN2 CDH23 KIAA1598 GRXCR2 DEF6 EPHA4 MTM1 TBC1D10C FGD4 FAM65B SYNE2 ENPP7 B4GALT1 CTSL2 MYO6 PDGFA ARL4C PPP1R9B MUC20 CEACAM16 IFT20 EPS8 ACTA1 ANGPT1 UBE2Q1 TWF2 CDHR5 SLC27A4 MYO7A MYO1B ACTG2 LOXHD1 ELMOD3 ITGA6 APBB1 FZD9 TEK FGF13 ODZ2 DCXR GAP43 TIAM2 GPR124 ATP6V1E1 WWOX USH1C DAG1 DYNC1H1 MYO15A MYO1G ITGB1 EXOC4 PLEKHG6 NGDN KIF13B APP FAT1 ACTC1 CTNNB1 KPTN WASH3P SCARB1 GPR98 ACTN2 ATP6V1B2 MYO7B UBE2K OXTR FARP1 CRB1 FSCN1 TPRN SPATA13 DNALI1 MYO10 PALM TMC2 ITGB3 CLRN1 ESPN RUFY3 CA2 MYO1C CXADR GPM6A ARF6 NLGN1 ERMN ATP8B1 MYO5A USH2A DMD ACPP OSBPL3 FMN2 SLC4A7 DPEP1 MYO1A CIB2 VASP CLIC5 PPP1R9A ATP6V1B1 ENAH FOXA1 LCP1 ABI2 HYAL2 S100P CLIC4 TBC1D10A STARD10 AP2A1 FZD3 EPHB1 SLC10A2 ATP6V1A

GO_CHYLOMICRON A large lipoprotein particle (diameter 75-1200 nm) composed of a central core of triglycerides and cholesterol surrounded by a protein-phospholipid coating. The proteins include one molecule of apolipoprotein B-48 and may include a variety of apolipoproteins, including APOAs, APOCs and APOE. Chylomicrons are found in blood or lymph and carry lipids from the intestines into other body tissues. APOB APOC1 LPL APOE APOH APOA2 APOA1 APOBR LSR APOC3 APOC2 APOA4 APOA5

GO_PEPTIDASE_COMPLEX NA F7 TXNL1 USP34 UCHL5 SPG7 USP27X PSMD7 WFS1 PSMG2 SPCS3 UBE3A PSMA5 PSME2 ATXN7L3 FAM48A PSMA8 PSMD2 WDR75 TADA3 USP22 PSME3 PAAF1 PSMD6 ENY2 PSMB6 PSMA7 PSMB1 PSME4 PSMA4 PSME1 SHFM1 PSMA1 ADRM1 PSMD4 PSMC6 PSMD12 F3 PSMA2 SPCS2 ZFAND2A PSMD14 VCP FAM48B2 UBE3C PSMF1 PSMG3 PSMC4 SPCS1 TAF9B FAM48B1 UBR1 IDE PSMD11 UBQLN4 PSMB8 USP51 HSPB1 PSMD13 PSMA3 PSMD9 PSMD3 USP14 RAD23B PSMB5 SUPT3H PSMD10 PSMB11 PSMB10 UBXN1 UBQLN1 KIAA0368 PSMD8 PSMC2 PSMD1 PSMD5 PSMC5 TAF9 PSMC3 PSMC1 PSMB9 RAD23A PSMB2 PSMB3 PSMB7 PSMB4 AFG3L2 PSMA6

GO_CHROMOCENTER A region in which centric, heterochromatic portions of one or more chromosomes form a compact structure. INCENP CDCA8 MBD5 MBD6 ESCO2 SCMH1 SALL1 FMR1 AURKB TINF2 OIP5 CBX1 PIWIL2 CBX5

GO_NUCLEAR_NUCLEOSOME A complex comprised of DNA wound around a multisubunit core and associated proteins, which forms the primary packing unit of DNA in the nucleus into higher order structures. HIST1H2BC HIST1H2BN HIST1H2BK H2BFS HIST3H2BB HIST1H2BL HIST1H3A HIST2H2BD HIST2H2BE HIST1H3J HIST1H2BJ HIST1H3D HIST1H2BG IRF4 H2BFM HIST3H2A HIST1H2BM MPHOSPH8 HIST1H2BE HIST1H3B HIST1H2BF HIST2H2BF H2AFB2 HIST1H3C HIST1H3G HIST1H2BD HIST1H3I HIST1H2BI HIST3H3 HIST1H2BA H2AFB3 HIST1H3F HIST1H2BB H2BFWT HIST1H2BH HIST1H3H HIST1H2BO CENPA H3F3B HIST1H3E H3F3A H2AFB1

GO_GOLGI_LUMEN The volume enclosed by the membranes of any cisterna or subcompartment of the Golgi apparatus, including the cis- and trans-Golgi networks. WNT6 GPC1 CSPG4 MUC2 DEFA1 FMOD DEFA3 CGA F2 CSPG5 MUC20 DEFB103B INS HSPG2 LALBA MUC1 VTN PCSK5 ACAN FURIN MUC21 DEFA4 MUC16 GPC3 F7 MUC17 SDC4 LHB WNT3A NCAN PROZ WNT7A PCSK6 BLZF1 DEFB103A WNT3 WNT5A MMP16 PRELP AGRP MUC19 DEFB4A AGRN MUC4 MUC6 LUM MUC12 MUC15 MMP11 DEFB1 GPC4 MUC3B GPC2 MUC5B DEFA5 NGF BCAN GAS6 ZG16 RAB33B DEFA1B SDC2 WNT4 DCN PROC KERA OGN DEFA6 F9 PROS1 WNT7B VCAN HS3ST1 MUC13 F10 WNT5B PPIL2 MUC3A SDC1 MUC7 GPC6 FGF23 SDC3 OMD MMP14 SDF4 TGFB1 GPC5 MUC5AC GOLIM4 WNT1 BGN SOD3 MUCL1 BGLAP

GO_VESICLE_MEMBRANE The lipid bilayer surrounding any membrane-bounded vesicle in the cell. SVOP RAB11FIP2 SYTL4 CUZD1 RAB7B EXOC3 HLA-DQB1 YWHAQ TMEM199 VAMP2 GAD2 GRIA2 SEC23IP CACNG2 CYBA ABCC4 ENTPD7 PIK3R4 TNK2 NCK1 FCGR1B CD207 SLC18A1 TMEM225 TAOK2 C10orf112 AP3M2 LRP1 LRRK2 SLC32A1 ATP6V0A1 SCYL1 RND2 ANKRD27 ZDHHC17 VMA21 SAR1B SEC22B COPE SGSM1 ATP8B3 PI4K2A RNF144A HLA-G RAB27B FZD6 SYT2 ACRBP WNT7B TMED2 ABCA3 FZD4 DENND1A SPRED2 IL15RA SREBF2 LAMP1 RAB38 GAD1 VAMP5 N4BP3 UBB UBC RAB43 WHAMM TBC1D5 RPH3A SYT9 WAS CD46 SYT4 ICA1 WNT7A CALY SYNRG SPARC PDE6D AP2A2 SLC26A6 LDLR VAMP7 ANXA3 NCK2 SYT12 TYR VTI1B PLA2G4D DCT ATP2A2 RAB31 SV2B GRIA1 SEC24C C6orf64 OCRL WNT6 MYOF RAB11FIP1 HLA-H RAB11A CAMK2D SELP FOLR1 BCL2L1 SYNPR TBC1D4 AHNAK2 SYT10 SYNGR1 HLA-DRB5 COPB1 YWHAE CNIH2 HLA-DQA1 APPBP2 ATP6V0C FNDC3A ATP6V0E2 OTOF FLRT1 COPG2 SPIRE2 SLC18A2 TRIP11 CADPS ATP6V0B STX1A ITPR1 SEC23B RAB11FIP5 DMBT1 ATP6V0A4 DLG4 CD163 ATP6V0D2 SPACA3 SERPINA5 AQP2 SNX5 RPS27A IRGM CHGA LDLRAP1 PIK3C3 TYRP1 RAB10 COPB2 SCAP AQP6 WNT4 KIAA0528 STXBP5 TLR1 SCGN ARCN1 SNAPIN SYT3 CNIH RAB7A RAB13 FZD5 GPRC5C PTCH1 SLC30A5 TMEM30A SEC31A AP3B1 EPS15 GPR143 CPE AP3S2 HBEGF ABCC8 SLC45A2 SYN3 STX17 BSG ECE2 ROR2 SCARB1 RASSF9 KIFC3 SYT7 DNAJC5 ITGB3 SCAMP1 RAB8B KIF1B KIAA1199 TLR2 ITPR3 CD74 RPH3AL YWHAG MFSD10 DAB2 SYT11 SNX17 ZNRF1 CAMK2B HLA-B LMAN1 SYNGR3 SLC17A8 ATP10B GRIA4 BAIAP2L2 C9orf11 TOR1A NOS3 WNT5A TMEM67 MARCH3 SCNN1B VAMP8 PLA1A AZU1 CYBB DENND4C SLC2A4 RAB34 CD59 HLA-DRB3 AP3S1 ZP3 SLC18A3 HLA-F RAB23 FMN2 RAC2 SEC24B PACSIN2 AP1S3 SEC24A TEX101 SEMA4C AP2A1 LAMP2 ATP6V0D1 SNX24 ATP6V0E1 ZDHHC8 PIKFYVE DNM1L KCNQ1 SNX22 LAMP3 CLIC4 CAMK2G TMEM190 SUN1 AP1B1 CLVS2 FLOT2 HLA-DRB1 PTPRN NCALD TRAF2 MARCH8 SH3KBP1 SNX9 KIAA1244 ZNRF2 HLA-DPA1 COPG SMO APPL1 NPC1L1 NOSTRIN HLA-DRA PCDH7 WNT1 ANXA4 SNX15 IFNGR2 HTT KDELR1 MARCH1 HLA-DRB4 ITPR2 KCNJ4 PCSK4 CLCN3 CLCA1 YWHAB SNCA PHACTR2 STAB1 ATP6V0A2 C3orf58 SMAGP CAMK2A CLTC ASPSCR1 VAMP3 YWHAZ SNX21 HLA-DQB2 MYO5B TMX3 SFN RAB22A RILP TMEM163 RAB14 SYN2 ATP13A2 HLA-DPB1 WNT5B MARCO CAMKV MCFD2 B2M CAV1 AP1G2 AP1M1 WASL AP2M1 SNTB2 LRIG3 SLC11A1 SEC13 DOC2A CD36 ATP8A1 AP3B2 ITGA2B AFTPH WNT3A RALA COPZ1 SNX18 SV2C SLC17A6 CHMP4A TMEM173 DYSF COPZ2 RAB21 CACNG4 SYT5 RHBG IZUMO1 WNT3 RAB11B GOSR2 MARCH11 TGFA HLA-A DBH MYO6 PICK1 SLC30A8 RAB26 TCIRG1 TRIM72 SYPL1 CLTA VAMP1 RAB32 DMXL2 VTI1A PAM PECAM1 GDE1 STAB2 EGFR SLC30A3 GPRC5A GABARAPL1 ARHGAP21 DTNBP1 AGTRAP SPACA1 FCGR1A MDM2 GRIA3 CA4 STX5 ATP6V1G2 CACNG3 SLC17A7 AP1G1 RAB20 SV2A SYNGR2 HMP19 WLS SNX7 SYPL2 ADAM8 CLTB SYT6 STXBP2 DNM2 MSR1 APLP2 INPP5B RAB5A SLC17A5 TLR6 DBNL SEC31B TEKT3 SRI YKT6 TMEM184A OCA2 AP2S1 NECAP2 CEACAM1 ATG12 RAB39 SEC24D ZG16 SYNGR4 AP2B1 CYB5R1 AP1M2 TMED10 SYN1 TMED7 AP1S1 HIP1R C19orf26 SREBF1 PACSIN1 SYCN DRD2 EPN2 SCAMP5 SLC6A17 GPRC5B CNIH3 AREG ZDHHC13 CLTCL1 SCG3 SYNJ1 SLC2A8 PICALM CACNG8 SCARF1 GPR161 RAB9A GRB2 GABRA2 NECAP1 COPA ATG5 CLIP1 SPIRE1 ACPP GIPC1 SEC23A SH3GL2 HLA-C HLA-E SGIP1 SNX19 BACE1 TH HLA-DQA2 UBA52 YWHAH RAB3A VOPP1 ANXA7 RAB8A HIP1 ANXA1 SYT1 NRGN CD63 C20orf103 SNX33 CORO1A USO1 GPER AMPH HSPA8 CLVS1 AP3M1 ULK2 AP1S2 FZD2 APOB COLEC12 PTCHD2 PTPRN2 SYP FAM170B SLC5A6 STX3 CAV2 LNPEP CD9 RAB9B ARRB1 RAB27A TMED3 CADPS2

GO_ENDOCYTIC_VESICLE A membrane-bounded intracellular vesicle formed by invagination of the plasma membrane around an extracellular substance. Endocytic vesicles fuse with early endosomes to deliver the cargo for further sorting. RPS27A RAB5A TLR6 SFTPC GOLIM4 IRGM INPP5B LDLRAP1 CCL2 ARRB2 AMN WLS DLG4 ADAM8 TLR9 FMNL1 MSR1 LMBRD1 DNM2 KIF5B STX12 CD163 ATP6V0D2 ATP6V0B GRIA3 NCF2 DMBT1 AP1G1 RAB20 ATP6V0A4 CACNG3 SRGAP2 ATP6V0C FCGR1A MDM2 ATP6V0E2 DVL2 RAB11FIP4 HSPH1 RAB5C ROR2 SCARB1 DRD2 EPN2 OCLN AP1S1 SYK HBEGF RAB7A SFTPB PTCH1 RAB13 FZD5 HYOU1 AP1M2 EPS15 AP2B1 LPAR2 WNT4 AP2S1 PIK3C3 TYRP1 RAB35 RAB10 ATG12 CUBN TLR1 RAB39 STX7 MYO1E SNX3 HLA-B CPNE6 HLA-C HLA-E SGIP1 SH3GL2 ARF6 WNT5A GRIA4 NOS3 SYT11 LPAR1 CAMK2B CTLA4 GIPC1 ATG5 SAA1 CACNG8 SCARF1 TLR2 PICALM GPR161 LTF RAB9A EHD3 TLR7 SCGB3A2 CD74 SYT7 HSP90B1 DPP4 RAB8B APOE CD2AP RAB9B ATP6V0E1 HYAL2 CD9 ATP6V0D1 LAMP2 CAMK2G NLGN3 COLEC12 APOB AP1S2 FZD2 AP1S3 AP2A1 CORO1A RAB23 SLAMF1 CLVS1 RAC2 UBA52 CYBB ITGB5 HLA-DQA2 HLA-DRB3 SLC18A3 AMBRA1 HLA-F RAB8A RAB34 HBA2 APOA1 HBA1 FCGR1B WNT1 ITGAV RAPGEF2 NCF4 TIRAP HP CLCN3 CD207 HLA-DRB4 SFTPD CACNG2 CYBA ATG14 GRIA2 SMO HLA-DRA PIK3R4 NCF1 HPX NOSTRIN HLA-DQB1 SH3KBP1 UNC93B1 RAB7B HLA-DPA1 FLOT2 SFTA3 UVRAG AP1B1 CLVS2 INPP5F SFTPA2 HLA-DRB1 CAV1 WNT7B WASL RAB38 LAMP1 STX6 FZD4 AP1M1 MTSS1 RAB14 DAB2IP HLA-DPB1 RAB22A ITSN1 RILP RAB17 HLA-G BECN1 B2M MARCO WNT5B KIAA0368 PLEKHG5 PIK3C2B PLD1 ATP6V0A2 ATP6V0A1 CAMK2A LRP1 O3FAR1 STAB1 HLA-DQB2 SFTPA1 CLTC ANXA11 TF CACNG4 RAPGEF6 WNT3 ABCA1 LDLR ANXA3 DYSF VAMP7 WNT3A RALA CD36 WNT7A SPARC AP2A2 SLC11A1 UBC RAB43 AP2M1 UBB VPS11 LRP2 TBC1D5 HLA-DRB5 HSP90AA1 DRD3 HLA-DQA1 STAB2 EGFR CAMK2D IGF2R TCIRG1 RAB32 AMOT WNT6 OCRL GRIA1 HLA-A RAB11FIP1 MYO6 PICK1 RAB11A HLA-H CALR RAB5B RABEP1 RAB11B HBB RAB31

GO_H4_H2A_HISTONE_ACETYLTRANSFERASE_COMPLEX A multisubunit complex that catalyzes the acetylation of histones H4 and H2A. ACTL6A MEAF6 BRD8 DMAP1 EPC2 MORF4L2 EPC1 MSL3P1 TRRAP KAT5 MORF4L1 MSL3 EP400 RUVBL2 YEATS4 ING3 ACTB RUVBL1 C20orf20

GO_ENVELOPE A multilayered structure surrounding all or part of a cell; encompasses one or more lipid bilayers, and may include a cell wall layer; also includes the space between layers. SLC25A43 AHCTF1 GDAP1 MTA1 SLC22A18 SELRC1 TNMD AKIRIN1 RNF43 NXT1 PTPMT1 C4orf49 COQ10A HN1 C18orf55 PRKG2 NDUFB7 KLHDC2 COX15 COX7C COX11 DAP3 C11orf85 CPT2 MRPL28 STOML2 PARL NDUFA2 DIABLO MRPL45 POLA1 NLRP6 INA SLC25A44 DNAJC19 MYOF SIRT3 TIMM9 STAR AGPAT5 CRAT NDUFS2 ATP5C1 MRPL15 MRPS5 MRPS26 FAM76B REXO2 MRGPRF PPOX TMEM97 TST PLN SLC27A3 ETFDH SDHD MRPL38 TYMS COX7B2 BNIP2 SPG7 ACSL5 CHCHD4 MAOB TERF2IP ARL2BP KMO RAF1 TNRC18 CISD2 TOR1AIP2 ACN9 C9orf89 FAHD1 ATP5J2 OMA1 ATF6 BLOC1S1 IFI27 SCAI ADAP2 NUP153 RTN4IP1 BCL2L2 NELL1 APP ATP1B4 NDUFA4 TMEM43 POM121C ZNF224 DHRS2 TMEM11 MCL1 SHMT2 CYP24A1 NDUFS8 COQ9 TIMM17B LEMD2 KPNA6 AQP1 RAB11FIP5 UCP3 TMEM18 SHISA5 BNIP1 C22orf32 UQCR11 GUCY2F TMEM38B HSD3B1 GTPBP4 SLC25A45 SLC11A2 RAC2 TIMM50 DPY19L3 NDUFS4 ACSL4 RPS3 MTDH NEU4 MLIP NUP155 GPAM DNM1L MRPL35 POM121L2 NOA1 CHCHD3 NDUFA3 PCYT1A SLC25A30 CBX5 MRPS10 RANBP2 ARL2 CEP170 IPO4 MCCC1 BIK TNKS2 SMCR7 P2RX7 TNPO2 TOR1A SMCP TMEM57 ATP5L2 PAM16 RANBP1 MRPL48 HCCS LETMD1 NUCB2 RAB40B CCDC155 ERAL1 CLIC1 P2RX4 CABS1 GSTK1 TMEM194A MRPL46 PDK4 VPS13C IPO5 SUN5 OCIAD2 COX7A1 MAD2L1 FAM169A FANCL SLC16A3 WTAP TDH ANKZF1 NUP50 EIF5A NOC4L COX7A2 RTN4 NDUFAF3 USP30 ERN1 TAMM41 NUDT1 ARG1 HADH NDUFB10 ATP5D MTHFD2L MICU1 SYNE2 SLC25A5 SLC25A10 DCTN5 THEM4 NRM CERS3 NDUFA7 P2RX6 PRKCA COX7A2L COQ5 DTL EXOG ATP5G1 NDUFA12 MTG1 SLC25A18 COX5B DPY19L4 SIRT1 KPNA7 TMEM14C LMNB1 BCS1L ALAS2 SEC13 XPO4 FUNDC2 RETSAT ATP5L TIMM44 MAOA BAD NPIP VDAC2 SFXN5 BMF SYNJ2BP C15orf43 MRPL22 P2RX1 NDUFC2 UQCRB MNS1 MRPL16 PHB MTOR CDS2 SREBF1 DHRS4 SLC25A39 PARP11 CHCHD10 SQRDL NXNL1 SARM1 HIGD1A SLC29A2 LOC100505876 MFF PTPLAD1 NDUFB1 CHCHD1 SDHB EPC1 SLC25A15 DNM2 UQCRQ OSBPL3 MRPL4 CYP11B1 NAV3 PLA2G4C GLTPD1 XPO1 NDUFS6 ZC3HC1 MRPL14 UBE2I DUX4 AMBRA1 TSPO PRELID2 TDGF1P3 SLC25A20 TRIAP1 CHMP7 KPNA1 ATP5I PHB2 GK PGS1 CYP11B2 IMMP1L AKAP1 PTCD3 MRPL41 XPOT ACAD9 BRI3BP ABCA12 SNUPN GIMAP5 GLE1 MAPK3 MRPL11 UQCRFS1 MRPS15 CPT1A DPY19L2P2 FPGS C11orf83 COX7A2P2 COASY MRPL52 EFHA1 NDUFA11 ABCB10 BECN1 UBIAD1 CSDE1 NDUFB6 SUN2 NUPL2 SIRT4 LETM1 VAPA C19orf70 NUP37 PDCD6 BIN1 DNAJC15 ECI1 PSEN2 CSE1L TIMM13 KPNA3 VDAC1 NDUFB2 HRNR ROMO1 EMD KCNH1 ABCB7 MPV17L2 GRAMD4 NDUFS3 TRIM27 SLC25A35 DDX3X MRPL27 OXA1L COX17 MFN1 GHITM ATP5J NDUFA5 TMEM126B MRPL51 PTGER3 FOXRED1 OIT3 C2orf28 NIPSNAP1 NNT SNN TOMM34 TSGA10 MRPS27 MGST1 CYP11A1 MTX1 MRPS21 PAK1 CEPT1 MRPL12 CACYBP SLC25A37 OSBPL6 RSAD2 TOR1B PNPLA7 YME1L1 COX4I2 TOMM40L CERS2 MRPL50 MUL1 CCND2 COX16 UQCRHL NLN COQ6 CERS4 AEN PRODH HERC2 CYBB NUP133 TFPT ABL1 TMEM33 MRPL20 MRPS28 TOMM5 C19orf12 SLC8A3 MTCH1 CAT LMNA UBXN4 SFXN4 PRKCZ PRELID1 FAM73B QTRTD1 RDH13 MRPS18B KPNA2 PEMT SDHA NDUFA4L2 THOP1 SLMO1 TMEM120B QSOX2 NDUFV3 MPV17 ENY2 PPP1R15A TRA2B PLCB1 SLC25A11 MRPL18 DHFRL1 RAE1 MATR3 ACSL1 FKBP8 LGALS3 DNASE1 CDK4 MARCH5 DGKH TIMM17A LETM2 IMMP2L APOOL PNPT1 ANXA4 P2RX3 SUMO1 CYB5A HSD17B8 NDUFB11 SLC25A31 NUP188 ALDH3A2 FAM156A NME4 CMTM3 ADRA1A REPIN1 FAM73A MCM3AP AGK CUEDC2 SP140 BPHL NUP88 NELF PARP1 NUP98 DHCR7 MRPL3 INTS1 MCART2 BCL2L13 CHCHD6 SIRT5 S100A6 CPS1 ACACB VAT1 SLC25A47 SLC25A42 NUP107 POM121L12 SLC25A14 LRRC59 ATXN3 TTC19 YBX1 DTX2 ABCF2 RAP1GAP2 TIMM22 SLMO2 RANBP6 TMC6 SPG20 MRPL44 HIGD2A RB1CC1 TOMM7 UQCRC2 AGPAT3 NUP160 CCDC79 POM121 TBC1D20 TMEM48 COX6B1 DTYMK TMEM170A C12orf62 NUP93 HADHA ICT1 MRPS18A TMBIM6 SLC3A1 WDR93 KPNA4 NDUFAF1 ERBB2IP IPO9 GPER CPT1C PMPCA SLC25A2 DNM1P46 USMG5 COX4I1 MYO19 NDUFB9 NRGN RHOT2 NUP205 HSD3B2 SLC30A1 NR4A1 MRPL40 TOMM22 COX5A NDUFA6 CHMP2A LDHD MRS2 ATP5H DST GNAQ SLC25A32 MRPL13 UCP2 CCDC56 ALOX5AP MRPS12 C20orf7 GHDC FAM156B SUCLG1 MRPS23 GCH1 TMEM126A DDX19A MRPS35 CYCS NUP62 TMEM194B MRPS6 ATP5B SAMM50 MOAP1 SLC25A29 VRK2 IL15RA MRPL55 PINK1 COX10 DPY19L1 ATP5A1 NDUFV1 SENP2 KLK6 C8orf38 IPO7 HPN PANK2 TOMM20L MRPL30 ALOX5 HK1 GCHFR CISD1 SIX2 PIK3R4 C22orf28 MRPS24 B4GALT1 SPNS1 NDUFS5 C14orf49 UNC50 OTC CKMT1A NPC1 IST1 CIDEA CPOX ADRA1B SPATA18 PARK7 ARMCX3 SLC25A3 MCU SMAD1 HMGCL MLXIP EIF5A2 PARP16 GAPDH BNIP3 VDAC3 TMEM70 RRM1 UQCRH CLU ECSIT MDH2 TNKS MRPL19 MTX2 MRPS11 HTRA2 MRPS16 REEP1 CKMT1B GCAT CHCHD8 MRTO4 POLR2M HSPD1 ATP5G2 UQCR10 RGPD8 NUP62CL TAZ RNF6 FXC1 ABCA9 TPR WDFY3 AZI1 RNF144B NDUFC1 MT3 ITPR1 AIFM3 ATP5S CEP89 SENP1 CIAPIN1 IFLTD1 BAK1 DHRS1 ZMPSTE24 KPNA5 MFN2 SEPHS1 SCO1 LRPPRC SLC25A33 DAG1 SORD GRPEL1 MTCP1NB ATAD3B PRKAR2B ATP5E PHF8 ACSL6 WDR3 DNAJC11 IDH2 PLRG1 MRPL54 BCL2A1 GOLPH3 UCP1 CKMT2 GPAT2 SLC25A21 CCDC109B IMMT PRPF38A MRPS9 BRP44L SPAST GOT2 GRK5 MRPL34 SDHC SLC25A1 MTCH2 COX6C SLC25A24 SNCA PI4KB ROGDI AKAP6 SLC25A48 COQ4 PSEN1 CHMP4B RPS6KB1 GTPBP5 BAX STAT3 AFG3L2 MRPS34 CNP TRAP1 PPP2R2B C2orf42 MRPL17 GMCL1 ST20 GNAZ PUM2 CERS6 SIGMAR1 CERS5 MTMR6 HMGCS2 NUTF2 GUCY2D LPIN1 SCRN1 OSBPL8 EBP ATP5EP2 MYO6 MRPS22 ATPIF1 ACSL3 C12orf5 SFXN2 PMPCB MOSC1 ACAA2 PLSCR3 EGFR MRPS33 AKD1 IPO8 MGST2 CLGN MRPL33 IKBKE LOC100652748 SPAG4 SLC24A6 DMPK BCL2L10 BCHE SLC11A1 MINOS1 FBXL4 SLC9B2 PGAM5 ANXA11 BOK TMEM176B NDUFS1 FECH NDUFA10 ATP5F1 SLC25A6 PISD GJA1 EDNRB SEH1L NME1 DAO GABRB1 ALDH18A1 MYOC NDUFA9 GFER MAPK8IP1 ATCAY MCART1 SOX10 PRICKLE2 CHCHD5 MRPL53 PTGES MAD1L1 CENPF FAM36A PLCD4 SLC25A4 RANGAP1 SPATA19 COQ7 NUP210 COQ2 RHBDL2 UQCRC1 NUP210L MRPL10 XPO7 ACAT1 ANXA1 ANXA7 RNF180 PHF20 NUP85 NDUFC2-KCTD14 TRPC7 NDUFB3 BNIP3L COX8A C1orf31 TNPO3 PTCHD2 MRPS31 MCART6 RAN TOMM6 PML TXNIP ABCB8 GUF1 C19orf46 PLD6 GPD2 FIS1 BCL2 ATP11B ABCD3 MYO1C APOO MRPL39 ABCF1 COX7B CHDH DPY19L2 RNF185 RANBP17 SLC25A40 NRXN1 LRRK2 ADCK4 MX1 SOD1 BRAP UQCC SMAD3 GHRHR LMNB2 C15orf2 KIAA1161 MX2 SYNE1 MRPL1 C7orf73 CRLS1 FGR CLPX FAM188A CBX3 TIMM8B MRPL2 PGR COX6A1 MGST3 TEX10 SLC25A19 RHBDL3 ATP5O NAT8L RCC1 CCDC90A NDUFB8 TYRO3 IPO13 BDH1 PCM1 TMEM120A SLC25A27 SORT1 MTERFD1 MRPL32 BCL2L1 MVP BRP44 ELF4 HTATIP2 PMAIP1 EI24 NDUFB4 SMCR7L TOMM20 ZNF354C CHCHD2 C15orf48 AGFG1 ZNF383 SLC25A16 NDUFA13 AIFM2 DHODH FZR1 NOS1AP AIFM1 TOMM70A QTRT1 MRPS17 LOC100130097 PRICKLE1 OPA1 FAM82A2 DNM1P34 AAAS SLC25A36 MAVS SLC25A23 COX6A2 CYP27A1 GRPEL2 SH3GLB1 DNAJC2 BRIP1 P2RX2 EIF5AL1 BCL2L11 MTFP1 C6orf125 PPIF MRPS25 PLA2G4B BID NDUFA1 TMEM38A SUN1 TOR1AIP1 HK2 CYC1 MSTO1 MRPS7 CEP70 RRP12 RPS6KA2 ITPR3 SLC25A46 TOMM40 LBR SLC25A34 NXF1 MRPS18C TMC8 SNPH PAFAH1B1 CPT1B TIMM23 SLC44A1 DNM1 TM7SF2 PPP1CC NLRX1 PTGDS APEH MRPS30 INTS2 MRPL49 SLC25A13 NUPL1 WASF1 ASS1 MTX3 COX19 ACAD11 NDUFB5 MRPL24 IPO11 LOC347411 C7orf44 TIMM10 MRPL36 LYN SUN3 ATF2 P2RX5 TMEM109 SLC25A22 TIMM8A NUP54 GATM MRPL37 AK2 ABCG2 TXLNG MRPL42 MRPS36 MRPL23 COX6B2 EFHD1 SRC SURF1 ABCA8 MOSC2 SFXN3 LTC4S PGRMC2 SORL1 CHCHD7 EVX1 TNPO1 NDUFS7 DNAJC1 RBM15 ACADVL NUP214 IGF2R FAF1 ALG14 RAB32 RHBDL1 VAMP1 PLA2G4A AURKAIP1 ATAD3A CST3 FLVCR1 COQ3 CYP27B1 SCO2 TMEM188 EPHA4 TMEM173 MRPS14 HADHB HSD17B1 NDUFV2 STARD13 SLC25A25 RNF13 H2BFWT FUNDC1 NDUFAF4 DDX19B NME2 NUP43 GGNBP1 GK2 COX18 COX8C SUOX NDUFA8 MRPL43 CASP8 CDC14B TUBB TMEM201 NDEL1 C4orf52 SLC25A38 HAX1 GTF3C3 SLC25A26 TIMMDC1 DUSP18 MRPL9 RHOT1 SLC25A12 SFXN1 ATP2A3 CTDNEP1 MRPL47 L2HGDH ACADL ATP5G3 KCNJ11 NDUFAF2 PRODH2 MTMR8 OGDH NUP35 SCGB1A1 COQ10B CYB5B NDUFAB1 ABCB6 RNF5 TMPO C14orf2 SLC25A28 LRMP SLC25A41 POM121B LEMD3 MRPL21 DUSP21 DNM3 PPAPDC3 CCDC90B CYB5R3 CLIP1 KPNB1

GO_ANAPHASE_PROMOTING_COMPLEX A ubiquitin ligase complex that degrades mitotic cyclins and anaphase inhibitory protein, thereby triggering sister chromatid separation and exit from mitosis. Substrate recognition by APC occurs through degradation signals, the most common of which is termed the Dbox degradation motif, originally discovered in cyclin B. CUL7 UBE2C CDC23 ANAPC10 CDC20 UBE2S ANAPC11 ANAPC13 CDC26 ANAPC4 C10orf46 ANAPC16 MAD2L2 BUB1B ANAPC1 CDC27 C11orf51 ANAPC7 ANAPC2 CDC16 ANAPC5 FZR1

GO_RIBOSOME An intracellular organelle, about 200 A in diameter, consisting of RNA and protein. It is the site of protein biosynthesis resulting from translation of messenger RNA (mRNA). It consists of two subunits, one large and one small, each containing only protein and RNA. Both the ribosome and its subunits are characterized by their sedimentation coefficients, expressed in Svedberg units (symbol: S). Hence, the prokaryotic ribosome (70S) comprises a large (50S) subunit and a small (30S) subunit, while the eukaryotic ribosome (80S) comprises a large (60S) subunit and a small (40S) subunit. Two sites on the ribosomal large subunit are involved in translation, namely the aminoacyl site (A site) and peptidyl site (P site). Ribosomes from prokaryotes, eukaryotes, mitochondria, and chloroplasts have characteristically distinct ribosomal proteins. MRPL55 RPL15 METAP1 C7orf44 MRPS34 MRPL52 RPL41 RPL13 RBM3 RPL18A MRPL24 RPL24 GTPBP5 NSUN3 SNCA MRPS30 MRPL49 MRPS35 RPL36 RPL29 MRPS6 MRPL46 METTL17 RPL3 MRPL18 MRPL11 MRPL34 MRPS15 MRPS23 RPS28 FMR1 MRPL45 MRPL42 MRPS36 RPL6 MRPL23 APEX1 NCK1 EIF2D SF1 RPL13A HBA1 MRPL17 HBA2 MRPL37 MRPL2 MRPL30 RPS9 RPLP0 MRPL28 SRP68 RPL23A RPL8 RPLP2 RPL39P5 MRPS2 RPL5 MRPL36 DAP3 MRPL1 MRPL32 MPV17L2 RPL34 MRPL33 REPIN1 MRPS5 C12orf65 RPS24 MRPS26 NUFIP1 MRPS33 NAA11 RPS15A RPS5 NDUFA7 RPL39L MRPL15 MTERFD2 RPL37 MRPS24 RPL10 RPL7A RPL35 RPL21 MRP63 MRPS22 RPLP1 RPSA RPS17 RPL31 RPL17 RPL10L RPL35A RPS14 RPS12 C1orf177 MRPS14 MRPS11 EEF2 RPS3A RPS27 MRPL51 CANX FXR2 RPL28 MRPL19 RPL4 PPARGC1A RPL22L1 PRMT3 RPS26 NHP2 RPL38 MTG1 MRPL38 MRPL27 DDX3X MRPL3 RPS18 RPS4Y2 RPL26 RPS23 LARP4B MRPL12 RPL12 MRPS17 RPS29 NAA10 RPL18 MCTS1 MRPS21 RPS25 EIF2AK4 MRPL44 DNAJC21 MRPL16 EIF2S1 RPS27L RSL24D1 RPS21 RPL37A RPL27A RPS7 MRPS27 RPL22 C4orf43 HSPA14 MRPS16 RPS20 GCN1L1 MRPL22 RPL39 MRTO4 RPS10P5 CHCHD1 ICT1 MRPS18A NHP2L1 GADD45GIP1 RPS19 RPS27A RPL37AP8 RPS10 RPL23 RPL26L1 RPL3L EIF2AK2 RPL7 RSL1D1 MRPL50 RPS6KL1 NR0B1 MT3 RPL27 MRPL53 RPL7L1 RPL19 RRBP1 NUFIP2 MRPL43 RPS6 MRPL40 RPL14 MRPS31 MRPS7 RPL11 MRPS10 RPL30 RPS16 MRPL35 MRPS28 MRPL20 MRPL10 MRPL14 RPS2 UBA52 MRPS25 RPL32 IMP3 MRPL4 RPS11 MRPL9 RPL10A SERP1 RPL9 MRPL47 RPL36AL RPS3 RPL36A MRPL21 RPS4Y1 MRPS9 RPS8 GNB2L1 MRPS12 RPLP0P6 MRPS18C MRPL41 MRPL13 MRPL39 MRPL48 ABCF1 ZNF622 RPS15 RPS4X RPL13AP3 NSUN4 SURF6 MRPL54 APOD RPS13 MRPS18B

GO_PIGMENT_GRANULE A small, subcellular membrane-bounded vesicle containing pigment and/or pigment precursor molecules. Pigment granule biogenesis is poorly understood, as pigment granules are derived from multiple sources including the endoplasmic reticulum, coated vesicles, lysosomes, and endosomes. RAC1 CTNS SERPINF1 CCT4 SND1 GCHFR YWHAB OCA2 CTSD RAB5A GGH DTNBP1 CNP RAB7L1 ATP6V1G2 PDCD6IP P4HB MMP14 PMEL RAB27B PDIA4 NCSTN CAPG RAB17 ATP6V1B2 GPR143 SEC22B PDIA6 GANAB ANXA2P2 LAMP1 RAB38 SLC24A5 BSG HSPA5 SLC45A2 RAB5C SLC2A1 SYTL2 TPP1 YWHAZ SYTL1 CLTC ATP6V0A1 RAB35 MYH11 TYRP1 TMED10 SGSM2 ITGB1 ANKRD27 RAB2A TFRC RAB7A FASN GNA13 MYO5A NAP1L1 MYRIP CANX PPIB HPS4 GPNMB TYR ANXA11 RAB1A RPN1 CALU CTSB ITGB3 DNAJC5 SLC1A5 MREG HSP90B1 ERP29 SDCBP HSP90AB1 RAB9A STOM SLC3A2 TRPV2 RAB32 RAN STX3 ANXA6 ATP1A1 PDIA3 SYPL1 MYO7A ATP1B3 YWHAE TMEM33 RAB27A SYNGR1 HSP90AA1 ANXA2 CD63 DCT AHCY MLANA PRDX1 TH SLC1A4 HSPA8 FLOT1 RAB5B

GO_NEUROMUSCULAR_JUNCTION The junction between the axon of a motor neuron and a muscle fiber. In response to the arrival of action potentials, the presynaptic button releases molecules of neurotransmitters into the synaptic cleft. These diffuse across the cleft and transmit the signal to the postsynaptic membrane of the muscle fiber, leading to a change in post-synaptic potential. DNAJA3 SYNGR2 SV2A EFNA2 HDAC4 PDZRN3 TRIP4 DLG1 COL4A5 UNC13B CAV3 UNC13A MYH9 PRKAR1A SYNGR3 PTN P2RX7 KCNC4 POSTN UTRN CDH15 EPHA4 MYH10 ITGB1 SYNC RAPSN MUSK CHRNA1 FCHSD2 SYNGR4 NRG1 PPP1R9A APP F2R ANK3 LRP4 CIB2 CDK5R1 COLQ PSEN1 SERPINE2 SNTA1 FCHSD1 DES SYNGR1 ASCC1 ACHE EPHA7 PRKACA CDK5 TBC1D24 SPOCK1 CAMK2D SLC8A3

GO_ROUGH_ENDOPLASMIC_RETICULUM The rough (or granular) endoplasmic reticulum (ER) has ribosomes adhering to the outer surface; the ribosomes are the site of translation of the mRNA for those proteins which are either to be retained within the cisternae (ER-resident proteins), the proteins of the lysosomes, or the proteins destined for export from the cell. Glycoproteins undergo their initial glycosylation within the cisternae. SCGB1A1 ZC3H12A SLC7A11 ARSB PLOD3 C1orf9 RPN2 RPN1 SRPR CANX CFTR NAT8L TRAM1 C14orf49 CYBB BCAP31 SEC61B STAU1 LRPAP1 SSR4 UBA1 PCSK9 SRP9 LIN28A FKRP VTN RANGRF MPZ SEC62 GNRH1 TMEM97 GLUL CA4 MT3 SPPL3 HTR5A LRAT EPHA5 SEC61A1 TRAM2 P2RX3 HM13 HCRT CCL2 EDN1 BGLAP DNAJC3 PLOD1 NCF1 ARL6IP1 NUCB1 HSPD1 SRPRB TRAM1L1 RP9 SNCA PI4KB PTGDS APP MYOC CCDC47 KCNJ2 ADCYAP1R1 STX17 PSEN1 PLOD2 RAB14 TP63 CDKAL1

GO_ATPASE_COMPLEX A protein complex which is capable of ATPase activity. SLC9A1 ATP4B ATP12A ATP1B1 PLN ATP1A1 ABCA2 ATP1B4 ATP1B3 ABCB6 ABCG8 ATP1B2 FXYD1 ABCG5 ABCD4 ATP1A2 ATP1A3 ABCA7 FXYD2 ABCF2 ABCB8 GM2A ATP1A4

GO_RECEPTOR_COMPLEX Any protein complex that undergoes combination with a hormone, neurotransmitter, drug or intracellular messenger to initiate a change in cell function. CHRNB3 APP GABRA3 TLR1 STXBP5 ITGA8 HSPD1 TGFBR2 GABRA4 ADRB3 GPRC5C ABHD12 HTRA2 CSF2RB SLITRK5 GABRQ HFE2 ITGAL HFE SYK GPR61 GABRB3 GPR98 IL13RA1 IL12RB1 NOS1AP TSHR ALCAM LRP5 DLG1 GPR62 CAPRIN2 ADRB2 PLXNA4 OSMR HTR3B LOXL4 RAMP1 LRP1B TAS1R2 RAMP2 MCOLN1 DLG4 VWC2 GRM7 ENG ABCG5 IL10RB GPR160 PLA2R1 AIP ABCG8 CHRNA7 SHANK1 RET HTR3A MUSK P2RX2 SHISA9 GABBR1 PLXNA2 IL6ST PEX5L BMPR1A CD79B FLT4 TGFBR3 BIRC2 DDR1 ROR1 GRIN2C ITGB3 ITGAE RIPK1 DAB2 PLXNB2 SDCBP GABRA6 CD74 PLXNA1 GRIK4 TLR7 ITGA9 ITPR3 TLR2 RNMT CHRNG CHRNB1 VDR TRAF5 PTPN6 PTK2B NOTCH2 CHRNA10 GRIA4 IL29 ITGA5 ACVR1 PTPRA VWC2L NRP2 OLR1 TRIL LRP1 GABRA5 ITGB6 GABRB2 CD44 CD8B HTR3E PTPRB GPR37L1 GABRA1 GABRD NRP1 SMAD3 LRP5L CHRNE CD4 GABRG2 ADCYAP1R1 FLT1 GRIN3A GRIN2B STOML2 INSRR TRADD GABRR1 ITGB8 KLRD1 VIPR1 CARD11 CACNG2 ACVR1C GRIA2 MTNR1A RAMP3 DIABLO INSR TAOK2 TNFRSF1A ERBB2 ADRA2A HTR3D LY96 NOTCH3 ITGA4 TOLLIP HTR3C GRIA1 GPR101 GPR20 KLRC2 DLG3 CD40 PLXNC1 CNIH2 GABRG3 GPR84 CHRNA6 CD14 GRM1 IRAK1 IRS1 CD3G NPNT NR1H3 CD79A DLG2 ITGAM PKD1L3 NTRK3 GABRR2 PIGR LDLR MYH9 IL28RA CR2 TF GRIN1 PLXND1 IL6 CD6 CEACAM1 CACNG7 GABRB1 GABRG1 PLXNB1 GRIK5 VLDLR ITGA1 ITGB1 OLFM2 TFRC ACVR1B CD8A KCTD8 ZACN CNIH3 EGF ABHD6 PTH1R TSPAN32 ITGA6 CHRNA5 GRIK2 PORCN CACNG3 CD3D GRIA3 TFR2 CHRNA9 CHRNB2 SHISA6 TRAF3 ITGB7 TLR6 ITLN1 NOTCH1 CHRNA2 CHRNA3 PLXNA3 ITGB5 TAS1R3 ITGA7 CNTFR CPT1C TRPV3 CHRNA1 GPR63 ITGB2 RNF31 PTPRN2 IL6R NTRK1 NR3C2 KCNK1 ITGA11 LRP8 PLXDC1 RXRA FGFR1 CACNG8 ERBB4 CD247 TLR4 FCRL5 NT5DC3 NLGN1 GABRA2 GRIN2A SHANK2 GHR CHRND KCTD16 BMPR1B ITGB4 TM7SF2 B2M ZAP70 GRIN3B OLFM3 SKAP1 GPR37 SORBS1 TRAT1 TRAF2 GPR119 TGFBR1 CACNG5 LYN CD200R1 CHRNB4 GABBR2 GRIN2D IGF1R GABRE CHRNA4 LEPR CR1L PKD2L1 ITPR2 TRAF6 P2RX3 GABRR3 ITGAV CHUK BCL10 ITGA10 EPS8 BMP2 PLXNB3 NTRK2 GRID2 LIFR ITGA2 APBB1IP ITGAX LRP6 TRIP6 ITGAD KLRC1 AHRR ACVR2B EGFR SACM1L AHR CD3E SHISA7 SHISA8 LRP2 IKBKB ITGA3 RFFL IL4R IMPG2 ITGA2B ERBB3 ACVR2A IL23R MTTP TRPC1 CACNG4 CNGB1 NPR1

GO_CUL2_RING_UBIQUITIN_LIGASE_COMPLEX A ubiquitin ligase complex in which a cullin from the Cul2 subfamily and a RING domain protein form the catalytic core; substrate specificity is conferred by an elongin-BC adaptor and a SOCS/BC box protein. COMMD1 ZER1 TCEB2 ZYG11B RNF7 CUL2 VHLL ASB4 ZYG11A GLMN RBX1

GO_SPINDLE_MIDZONE The area in the center of the spindle where the spindle microtubules from opposite poles overlap. CENPE KIF18A PLK1 RCC2 RACGAP1 GEM PKP4 AURKB CDC6 KIAA1383 ARL8A AURKA ARL8B EML1 CTDP1 MAP9 CTTN APP CDCA8 OR2A4 CENPV KIF20B BUB1B UNC119 AURKC CDC42 KIF14

GO_EARLY_ENDOSOME_MEMBRANE The lipid bilayer surrounding an early endosome. DNAJC13 VAMP8 MARCH3 KIAA0319 ANXA1 TMEM165 LLGL1 PLA2G4B HLA-F C20orf103 RUFY1 HLA-A LRP6 EHD1 ZFYVE20 HGS OCRL CFTR RAB5B HLA-H MTMR4 SNX16 EHD4 CLVS1 FIG4 KCNH1 SLC9A6 WASH1 VAC14 EPHB1 SH3GL1 PMEPA1 HPS6 FAM21A NTRK1 STAM2 SYNDIG1 PIKFYVE EGFR SNX8 ATP9A TICAM2 ZFYVE9 VPS33B SNX20 SNX4 WNT3A SNX1 WASH6P SNX6 PML EPHA8 SH3GL3 EPHA4 RAB21 HLA-C HLA-E HLA-B SNX3 SNX19 DKK1 MMGT1 STX7 SNX13 SNX21 ZFYVE16 ABCA7 FZD5 SNX27 FGD2 EPS15 FAM21C STAM MTMR2 RAB14 TMEM163 PI4K2A CD8B B2M HLA-G RAB5C CAV1 ZFYVE28 WASH3P EEA1 CLVS2 INPP5F FCGR1A MARCH8 APPL2 CLCN4 RCC2 PLEKHF2 RAB11FIP5 HSD17B6 APPL1 CLIP3 SNX2 WLS RAC1 SNX5 TMEM9B REP15 INPP5B C18orf1 FCGR1B MARCH1 RAB5A KIF16B KREMEN2 CD207 TMEM184A CLCN3

GO_AXONEME_PART Any constituent part of an axoneme, the bundle of microtubules and associated proteins that forms the core of cilia (also called flagella) in eukaryotic cells and is responsible for their movements. KIAA1009 FAM154A CCDC114 SPAG17 CCDC42B DNAH17 DNAH1 TXNDC3 DNAI1 RSPH4A DNAI2 DNAH8 WDPCP DNAH6 C6orf165 DNALI1 ARL6 DNAH7 DYNC2LI1 CCDC37 HYDIN DNAH5 SPAG16 DNAH2 DNAH3 ARFGEF2

GO_AXOLEMMA The portion of the plasma membrane surrounding an axon; it is a specialized trilaminar random mosaic of protein molecules floating within a fluid matrix of highly mobile phospholipid molecules, 7-8 nm in thickness. ADORA1 CNTNAP2 KCNH1 CHRNA7 EPB41L3 SLC1A2 NRG1 ANK1 ROBO2 KCNC2 KCNJ11 SPTBN1 MYO1D MAPK8IP3

GO_LAMELLAR_BODY A membrane-bounded organelle, specialized for the storage and secretion of various substances (surfactant phospholipids, glycoproteins and acid phosphates) which are arranged in the form of tightly packed, concentric, membrane sheets or lamellae. Has some similar properties to, but is distinct from, a lysosome. RAB7A NAPSA SFTPB SPINK5 KLK5 SFTPA2 SFTPA1 CTSH SFTA3 LAMP1 SMPD1 LAMP3 KLK7 SFTPC ABCA3 CKAP4 ABCA12 SFTPD

GO_NUCLEAR_CHROMOSOME A chromosome that encodes the nuclear genome and is found in the nucleus of a eukaryotic cell during the cell cycle phases when the nucleus is intact. RBMX POLD1 KAT2A E2F1 EME2 SMARCC2 CTC1 HIST1H4A LRIF1 LOC728637 PCGF2 KLF4 TCF4 HEY2 TNKS RPA3 KDM4C ING3 HIST2H2BD TERF2IP RELA SMCHD1 PURA TTC21B HIST1H2AB TCF7L2 NR1H3 CHMP1A HIST1H2AC MXD1 POLD4 ALKBH1 DDX11 ACD SETX HNRNPK DDB1 HIRA HIST2H4B ACTB SWI5 XRCC3 SIRT6 TEX11 MCM7 CDC45 SS18L1 SYCE1L POLA1 ZSCAN4 ING2 TRIM24 RAD51 DLX5 EED SFR1 CDX2 MBD3 DFFA MEN1 C11orf85 HIST1H2BL WDR82 EZH2 HIST1H2AE MCRS1 HIST1H2AI DVL3 NRIP1 CBX5 MKI67IP RAD51AP1 ZMIZ2 MCM4 RUNX3 AURKA HIST1H4L H2AFJ SMARCD2 NPM2 CEBPB HIST1H2BI HMBOX1 HIST1H2AA XPA SIRT2 HIST1H3C GINS1 RBBP4 GAR1 ACTR5 TFIP11 HIST1H1B CCDC155 RAD51D RPA4 POLR3G CCNB1IP1 CDC73 DNA2 CHAF1B BCAS2 BRD8 MIS12 BLM RUVBL1 HIST2H2BE CALCOCO1 UPF1 TNKS2 HIST1H2AD IPO4 POT1 HIST1H4I PLRG1 FIGNL1 HIST1H4H OBFC1 WRN EXOSC9 TRIM28 PPARD WRNIP1 XRCC5 HIST1H2AH THOC4 SMARCA4 INO80E NCOA3 BAZ1B NCAPD2 TCF12 SYCE3 TEX12 REC8 HIST1H2BE HIST1H1E LRPPRC FKBP6 SAP130 BIRC5 RUNX2 SP100 OBFC2A H2BFM ZFP57 ATM NOL6 TERF2 NFRKB TTN SLX1A KLF1 TP53 JUND TERT HIST2H2AA3 SMARCAD1 THOC6 TOP2A UHRF2 SMARCA2 PBRM1 DNTT H2AFB3 H1F0 HORMAD1 T AURKC HIST2H2AA4 HIST2H2BF TRPS1 DNMT3A STAG3 SMAD4 TOP1 SUV39H1 THOC7 IRF4 ACTL6A TIPIN H2AFV TCF7 SOX18 AURKB STAT1 SCRT2 SIRT1 PAX6 ZNF385A TRRAP DCLRE1B POLD3 STAT3 LEPREL4 TCF3 ESCO2 POLE3 PLK1 SLX4 RAD9A PPP1R10 GINS2 INO80B DCLRE1A HIST1H2BF FOXD3 PMS2P3 PRPF19 NASP SIRT7 ERCC1 HAT1 HIST1H2BK RAD51C INCENP UBE2B NFATC1 BRCA2 ADD3 HIST1H3E HUS1 TCP1 ARID1A CHAF1A BRD4 CHRAC1 NCOA1 MCM2 RARG UBE2I HIST1H4C EP400 HIST1H3G SPI1 GABPA ACTR8 NCOR2 SMARCA5 CXorf27 RAD1 CHEK1 HIST1H3B HSPA2 HIST1H3D HIST1H2BM HMGA2 THRB SATB1 HIST4H4 DMC1 POLR3D PML HIST1H3A HIST1H2AK NACC2 POLR3GL TAL1 MCM6 SYCE2 HIST1H4K SMC2 CDCA5 SYN1 NCOR1 HIST1H2BB MIS18BP1 CBX8 ENC1 SMARCC1 MCMBP HIST1H1A C15orf43 HIST1H1C MTA2 HNRNPC USP3 SYCP1 OBFC2B CITED2 BRMS1L HIST1H4E HIST1H3J H2BFS SIN3B SSB TONSL NLRP2 JUNB NUFIP1 HIST1H2AM ATRX RCC1 H2AFB2 ERCC4 RPA1 PCNA CREB1 ORC1 INO80 KIAA1967 INO80C BRMS1 SMARCD3 HIST1H2BG SMC1A POLA2 NHP2 MBD2 POLE PPARGC1A HIST3H2BB FAM60A SP1 SYCP2 POLE2 TERF1 NUCKS1 SMC3 SMAD3 MMS22L FOXH1 SMAD2 HDAC8 ASF1A HIST2H2AB SUZ12 HIST3H3 MRE11A NEK2 MYOD1 BEND3 APEX1 H3F3C ORC2 MCM10 HIST1H4J HIST2H4A CBX3 JUN LIG4 GATAD2B ESR1 SUN2 IKZF1 CDK1 BRCA1 H2AFY MORF4L1 ASH2L DBF4B RAD21 PRKDC THOC1 HELB HIST1H2BO DFFB NAT10 TFPT SMC1B ATR SMARCB1 H2AFZ HIST1H2BA RUVBL2 GINS4 DPF2 TRNP1 ORC5 ERCC5 PINX1 CBX1 ORC4 TBP HIST1H1D FEN1 UHRF1 TNKS1BP1 HIST1H4D TOP1MT PMS1 UCHL5 CENPC1 KIFAP3 BUD31 CENPA NCAPD3 HIST1H2BH SYCE1 CSNK2A1 MLH1 HIST1H4B HIST1H3F SETD3 PHOX2B HIST1H3I AR SLX1B HIST2H2AC ASXL1 EME1 MPHOSPH8 HIST1H4F CREBBP ASF1B NDC80 RFX3 HORMAD2 SMARCAL1 HIST1H2BC ZBED1 TUBG1 PHOX2A NELF PARP1 SUDS3 H3F3A TEN1 MCM3 XRCC6 REPIN1 EHMT2 MIXL1 TARDBP SUV420H1 TOX4 UBE2U EIF2C3 HIST1H2BD H2AFY2 CHD4 KIAA0146 NR1D1 KLHDC3 GATA3 PHF12 HIST3H2A DSN1 MSH3 HIST1H2BJ RAD9B ORC6 SIN3A HIST1H2BN THOC3 IRF1 SUV420H2 WBP2 RRS1 CBX7 H2AFB1 HMGB2 TP63 H3F3B MEF2A HIST1H3H PIF1 TOPBP1 RNF212 PURB PPP1CC MBD1 PLCB1 LDB1 SYCP3 THOC5 BUB1B CDC5L MSH5 HDAC1 SMARCE1 ORC3 WAPAL POU4F1 SNAI2 CCNB1 MSH6 UBE2A ZNHIT1 MLH3 TIMELESS ETV3 KAT5 SGOL1 PAWR FER H2AFX SETD1A PPP1CA MUC1 NR1H4 MSH2 HDAC2 POLR2B RGS12 RAD50 STAT6 MCM5 DCLRE1C KDM1A FOXC1 PPP1CB HIST1H2AL ZRANB3 E2F4 RSPH1 ANP32E TINF2 THOC2 YY1 RXRA HIST1H2AG MSH4 ZNF238 RARA CTNNB1 SRF NBN H2BFWT APTX RING1 POLE4 RPA2 HUS1B POGZ H1FNT BUB1 RAD21L1 RNF2 LRWD1 HAND2 CCDC79

GO_MOTILE_CILIUM A cilium which has a variable arrangement of axonemal microtubules, contains molecular motors, and beats with a characteristic whip-like pattern that promotes cell motility or transport of fluids and other cells across a cell surface. Motile cilia are typically found in multiple copies on epithelial cells that line the lumenal ducts of various tissues. Motile cilia may also function as sensory organelles. NME5 TCTEX1D4 AK8 HK1 SEPT4 DNAJB13 CATSPERG C19orf20 ABHD2 AKAP3 SORD FSCB KIF2A RSPH4A TEKT3 AK2 DAAM1 PACRG PFKM RSPH9 IFT81 PGAM4 SEPT12 DNAI2 SLIRP RNF38 INTU ODF4 TXNDC8 SPEF1 SPAG6 PGK2 ENKUR IFT74 CCDC135 CEP89 CATSPER4 IQUB IQCG IFT88 FOPNL PRKACA IFT52 CATSPER2 WDR19 ROPN1B HEATR2 TXNDC2 SLC9A3R1 MNS1 DRD2 TXNDC3 NPHP1 BBS2 MAK CCDC103 CATSPER1 TSGA10 SLC9A10 SPEF2 CABS1 SPAG16 ACTL7A KLC3 SLC26A6 DEFB1 SPA17 ADCY10 SCNN1A SLC26A3 TSSK1B TEKT4 CCDC108 DNAH1 ATP2B4 LDHC DDX6 CCDC37 RSPH1 GAS8 LOC100653515 ALMS1 SQSTM1 TAS2R43 SPAG4 CATSPER3 BBS4 IFT172 SPAG17 CCDC42B FAM154A AKAP4 OXCT2 MKKS ODF2 HSP90AA1 SPATA6 IFT27 TEKT5 ALS2CR12 RPGR TMEM146 IFT46 CABYR IFT20 ANXA1 GSTM3 AK1 C10orf90 ARL13B C6orf165 SLC25A31 TEKT2 ODF3 CCR6 PTCHD3 HAVCR1 HIF1A ROPN1L KIAA1731

GO_ORGANELLE_ENVELOPE_LUMEN The region between the inner and outer lipid bilayers of an organelle envelope. SOD1 AIFM1 COX19 FXC1 GGNBP1 PINK1 EFHA1 SHMT2 CACYBP SUOX NDUFB7 CHCHD10 HTRA2 CHCHD8 BLOC1S1 SELRC1 CYCS SLMO2 MYOC APP GFER PANK2 DTYMK TUBB TIMM8B AK2 ALOX5 DIABLO HAX1 NLN HSD3B1 COX6B2 CHCHD5 TIMM10 LYN NDUFA8 OPA1 CEP89 CIAPIN1 PTGES STOML2 TIMM8A FGR GATM PNPT1 COX6B1 TRAP1 PRELID2 HSD3B2 CPOX TRIAP1 REXO2 IGF2R C1orf31 PPOX CAT MICU1 C6orf125 THEM4 TIMM13 NDUFS5 STAR TIMM9 SORL1 MTCP1NB NME4 CHCHD7 FBXL4 ACN9 THOP1 NDUFS1 SLMO1 CHCHD2 TIMM23 PARK7 ARL2 PRELID1 GOLPH3 TXNIP COX17 SIRT5 CHCHD4 BCHE ARL2BP

GO_ACTIN_CYTOSKELETON The part of the cytoskeleton (the internal framework of a cell) composed of actin and associated proteins. Includes actin cytoskeleton-associated complexes. NMT1 ADD1 MYH10 TPM4 DPYSL3 SPTA1 RHOU ORC4 CDH2 ESPN ZNF174 PEAK1 PKNOX2 BAIAP2L2 MYO18A CAP2 SPTBN1 SPTB MYO10 SVIL AKAP13 ZNF74 PRKCZ MYOZ2 IPP MYH13 FMN1 TAF5 USH1G SH3GL1 MYO3B CRYAB FHL2 CD2AP ARPC5L EPB41L2 ABL1 ALDOA STAG2 ACTR10 RLTPR CLIC4 KIF9 TMSB4Y MAP7D3 CAPZA1 NPFFR2 MYH1 PARD6A STK38L PPP1R9A SEPT11 SEPX1 ARPC1A FMN2 PHC3 DLC1 MYBPH CGNL1 RAC2 MYOZ3 MYL2 CDC42EP1 C9orf72 PDLIM7 CLDN5 MYO5C CORO2A MYADM CAPZA3 MYH7 FLNB SPTAN1 TTN PDLIM2 WIPF1 CASK ZYX ZBED1 SPPL2B HCK TSC1 ACTG2 STX1A CAPZB DNAJA3 LASP1 MYBPC1 PALLD HFE WASF2 CAPG ARPC4 PDLIM3 SIPA1L3 ACTN3 ACTC1 SLC2A1 MYL6B ACTR3B KLHL20 PAK1 FERMT1 SPTBN5 DBN1 TMOD2 MYH11 MYH2 ILK GYS2 KLHL33 SH3PXD2A MYO15A FLNA MYO18B CTNNA2 FHOD1 VANGL2 ACTR1A TNNC1 FSCN2 MARK2 MYOM1 CAP1 AVIL TNNT3 MYRIP MTPN MYH9 TNNC2 IQGAP2 MYL12A ARPC2 ARHGAP6 CORO2B WAS TPM1 RDX DAPK1 ANKRD23 BCAR1 TRIM32 TPM3 ARHGAP32 CDH1 MSRA KAT2B MYL4 LPXN ZNF268 MYO7A AIF1 PGM1 FHL3 AIF1L ACTR2 ACTA1 LLGL1 CCBP2 AMOT GNG12 LSP1 CALD1 MYH16 PPP1R12A NOTCH3 TRPV4 CYBA MYOM2 MYL1 BIN1 UTRN APBB3 SORBS2 CAPZA2 LMOD2 CORO1C NCAPG MYL12B STOML2 HDAC4 CTTN MYZAP STK17B DCTN2 EZR OPHN1 FGR ZNF185 BAIAP2 MCRS1 DCTN6 HSPB7 EEF1A1 CAPN2 COBL MTSS1 ACTA2 BAIAP2L1 NOX4 SYNPO ADD2 CRK VCAM1 TLN2 ARHGEF5 MYH3 HNRNPK APC2 MYOZ1 TNNT2 MYL6 HAP1 LMOD3 DDX58 MYH15 ABLIM3 MED28 MYO5A MYBPC3 TCERG1 MYL5 KALRN MYO1C MYO1E ANLN VPS18 ACTL7B CORO1B IQGAP1 SEPT9 TRIOBP AHNAK TNNI3 IVNS1ABP VILL FSCN1 CROCC WIPF2 POU6F1 LRRC16A APOB KRT19 ARC LMOD1 ONECUT2 FER PAWR FERMT3 GDPD2 RINL MYL3 ELL LCP1 MYO9B KLHL2 MYO19 RAC3 SEPT7 FAM101B TMSB10 PTPN12 C10orf90 NFATC2 FERMT2 CLIC5 ACTR3C VASP ACTN4 FYN SYNPO2L CORO1A CFL2 ARPC1B GAS2L3 MTSS1L MYO1A AMPH FLOT1 DCTN3 ADAM8 ACTR3 RAC1 RAB5A MOBP CFL1 HAX1 MARCKS DAAM1 DBNL IFIT5 TEK MAD1L1 NEBL TMEM48 MPRIP ABLIM1 CENPQ EPB41 PLEKHH2 TMOD4 BIN2 TTC17 NFE2 LIMA1 CDC42EP3 MYLK3 INTS6 PXN MYO7B ABL2 CNN2 GAS2 CCDC53 WDR1 RARA WIPF3 FBLIM1 PTK2 ARHGAP35 PLS3 SHROOM4 ACTN2 FYB CDC42BPA KPTN CTDP1 SH3PXD2B MAEA MYO1G FKBP15 DCTN4 BMF MYO1F MLPH FGD4 MYO16 TMSB4X SHROOM2 MYO9A KEAP1 NF2 ORC6 TMEM63B SMTN RAI14 VIL1 C1orf190 SCNN1D TNNI1 MYBPC2 MYLPF SEPT2 ABLIM2 EPB49 BIN3 MYH14 TARS YES1 MYO1B LLGL2 TNNT1 MYL9 MYH8 ADAM17 KNTC1 CDC42EP4 ABRA DENND2A DCTN1 NEB PGM5 DAPK3 TMOD3 DSTN H1F0 PPP1R9B SHROOM1 PPP1R12B GSN KLHL17 SRC ACACA KNCN ACTG1 PKD2 MYO6 MYO1D CORO6 BFSP1 ACTN1 RAPGEF3 RCSD1 ARPC3 FAM101A MYO3A TPM2 MYH4 MICALL2 PFN4 PDXP FLOT2 C2orf62 ACTR1B DYNLL2 LANCL2 SEPT12 SPTBN4 MYO1H TNNI2 PDLIM5 SRCIN1 SLC16A3 CTTNBP2NL MYOT CDC42BPB JUP TWF1 MST1R RAB22A PLS1 PSMB3 TMSB15B CCDC102A MYH7B AFAP1L1 SORBS1 CNN3 MYLK WASL SLC9A3R1 RHOQ SNCA SPTBN2 TOPBP1 WASF1 CGN SYNPO2 MYL7 ZNF664-FAM101A VCL TMSB15A MYO5B ERAL1 FAM129B CTNNA1 FSCN3 TMOD1 MYH6 NCOA5 ARPC5

GO_NUCLEAR_PERIPHERY The portion of the nuclear lumen proximal to the inner nuclear membrane. SRPK1 RGS12 GFI1B NCOR2 CFL2 KRT8 ZNF703 TGFB1I1 MYB SRRM1 RUVBL2 GFI1 DNTT PAXIP1 TNPO1 ATF4 CEBPB NUP205 NUFIP1 CLIC4 SFPQ GMCL1P1 MAPT ZNF350 THOC1 ATF7 KIF4B LMNA SCAF8 FIGN NELF PHB2 NUP98 NUP35 PHACTR3 JAK2 SATB2 PRKCZ IPO4 TEP1 ERCC8 PML CHMP1A SPARC YY1 NUMA1 TINF2 HNRNPM PPIG LMNB1 SATB1 RUVBL1 BLM AKAP8L NUP107 UHRF1 TNPO2 SUV39H1 ATXN3 XPOT DNMT3A KPNB1 LRIF1 ZNF326 DDX39B PRPF40A MBD1 AHCTF1 MAEA CENPW ARFGEF1 CAD PRKCD MATR3 ODZ1 NUP153 ENC1 RANBP6 PSPC1 ATXN7 SORBS1 EBNA1BP2 NARF IPO5 HLCS SMC3 TPR RNASEL PSMA6 GHRHR OGG1 CXXC1 LMNB2 SMARCAD1 RUNX1T1 SPTBN4 AKAP8 ATXN1 MEN1 YEATS4 HAT1 KIN TP53 CENPF CASK CFL1 PHF5A GMCL1 ALOX5 KIF4A NONO DCAF7 NUP93 TELO2 SNW1 POLA1 PIAS4 ATN1 MORC3

GO_BASAL_LAMINA A thin sheet of proteoglycans and glycoproteins, especially laminin, secreted by cells as an extracellular matrix. LAMA4 AMTN ACHE AGRN LAMA1 LAMC2 COLQ LAMC1 LAMB3 ANG FN1 COL4A4 LAMB1 ENTPD2 LAMA3 LAMA2 COL4A5 LAMB2 LAMA5 NID1 DLG1

GO_INTRINSIC_COMPONENT_OF_ENDOPLASMIC_RETICULUM_MEMBRANE The component of the endoplasmic reticulum membrane consisting of the gene products and protein complexes having either part of their peptide sequence embedded in the hydrophobic region of the membrane or some other covalently attached group such as a GPI anchor that is similarly embedded in the membrane. ELOVL2 HSPA5 PTPLA TBL2 MMGT1 ACER3 PIGU FAM158A ELOVL4 SLC37A4 PIGG ATF6 KIAA0090 EIF2AK3 ATF6B SLC27A2 SLC35B4 MARCH6 TAP2 DOLPP1 RTN1 INSIG1 BNIP1 TAP1 PTPLAD1 SEC61A1 ELOVL1 SLC35B2 IMP5 SPPL2B SPPL3 EXT1 SPCS1 SLC35B3 SLC27A5 PORCN RRBP1 BFAR PTPLB SLC35B1 SELS LRIT1 RHBDD1 CLN3 TMEM33 ANKLE2 BCAP31 DOLK HLA-DQA2 HLA-F HLA-DRB3 RNF180 TAPBP TEX261 PGAP3 INSIG2 TM7SF4 RTN2 TMCO1 TMEM85 SLC37A3 AMFR HLA-C HLA-E HLA-B FITM2 G6PC2 LBR TMEM66 WFS1 SYVN1 CD74 DPM3 HLA-DPB1 PTPLAD2 BSCL2 HLA-G ESYT1 PIGK ELOVL6 SPPL2A SREBF2 FKBP8 HLA-DQB2 ESYT3 TM7SF2 DPM2 ARL6IP1 SAMD8 UPK2 HLA-DRA ERN1 HM13 TMEM93 ZFYVE27 FITM1 DPAGT1 HLA-DRB4 UBXN8 SGMS2 SGPL1 C15orf24 RTN4 HLA-DRB1 HLA-DQB1 ESYT2 PIGT TMEM111 DHRS9 HLA-DPA1 ELOVL3 ABCB9 TECR AUP1 ELOVL7 PREB HLA-DRB5 DGAT2 CYP2E1 HLA-DQA1 SACM1L SLC37A1 EDEM1 PKD2 HLA-A MBOAT4 CALR HLA-H GPAA1 DERL3 RCE1 DERL1 SGMS1 XXYLT1 CANX TTC35 G6PC3 SLC37A2 STIM1 DERL2 G6PC COX4NB ELOVL5 FAM134B C19orf63 DHCR7 PIGS

GO_LAMELLIPODIUM_MEMBRANE The portion of the plasma membrane surrounding a lamellipodium. VASP EPHA2 ITGB3 FERMT2 PDPN DPP4 APC2 KCNA2 SYNE2 NCKAP1 PLEK2 ANTXR1 CSPG4 SLC39A6 PIEZO1 FAP PDXP ITGAV CFL1

GO_ADA2_GCN5_ADA3_TRANSCRIPTION_ACTIVATOR_COMPLEX A multiprotein complex that possesses histone acetyltransferase and is involved in regulation of transcription. Contains either GCN5 or PCAF in a mutually exclusive manner. The budding yeast complex includes Gcn5p, two proteins of the Ada family, and two TBP-associate proteins (TAFs); analogous complexes in other species have analogous compositions, and usually contain homologs of the yeast proteins. Both ATAC- or SAGA (see GO:0000124, SAGA complex) are involved in the acetylation of histone H3K9 and K14 residues. DR1 CSRP2BP KAT2B WDR5 HCFC1 MAP3K7 UBAP2L POLE4 POLE3 YEATS2 MBIP KAT2A TADA3 ZZZ3 CCDC101

GO_ENDOSOME_LUMEN The volume enclosed by the membrane of an endosome. CTSL1 CTSS RAB38 INS PGA5 CTSK SFTPC PRLR PRF1 LNPEP B2M RAB32 AP4S1 APOB PGA4 AP4E1 JAK2 SFTPB NAPSA CTSH CD63 PGA3 CTSB APP AP4B1 AP4M1

GO_NUCLEOPLASM_PART Any constituent part of the nucleoplasm, that part of the nuclear content other than the chromosomes or the nucleolus. SQSTM1 PHAX THOC2 THAP1 ZNF335 MLLT3 CTR9 TBL1Y MED17 TAF7 PIN1 TAF2 ZC3H3 PPP1CB POLR2C PEX2 C17orf70 TUT1 POLR3E GFI1 POLR2B INTS5 USP7 PPARGC1B HDAC2 CCNL1 SRSF1 PPP1CA NARG2 TAF12 TADA2A SETD1A ZNF638 SUPT5H MLLT1 MSL1 APITD1 RBBP7 WDR33 THRAP3 NOP10 NOC3L INTS6 SPOP YEATS4 USP51 FANCM CHD8 MDM2 HDAC9 GTF2F1 RNF2 TDP2 SRP54 MLL5 PIAS1 INTS12 SRSF5 GRINL1B BCLAF1 PABPN1 PRPF40A MED14 STK38 TBL1XR1 CLK2 TGS1 RPA2 MSL2 ODZ1 UBOX5 C20orf20 FTO RING1 POLE4 PHF16 RECQL5 TAF1 PRMT5 SALL1 EIF4A3 NBN PRKACA CXXC1 INTS1 CPSF4L THAP7 SATB2 THOC3 SIN3A MSL3 ERCC2 SART3 C14orf43 TCEB3C SUMO3 TAF13 PIAS3 AKAP8L FANCF MED13L PHF12 ZNF768 HIF3A C1orf51 WDR75 MBIP RSRC1 PSME4 ING4 C5orf41 TP53INP2 H2AFY2 HDAC7 CSTF2T CHD4 POLR2K RNF34 MED12L GEMIN5 HINT1 TRIM8 NXF3 LAS1L POLR2D TARDBP LRCH4 MED6 CASC3 SAP18 RBM15 SFPQ MKNK2 DAPK3 SUDS3 SCNM1 SP140 TAF6 ETV3 MAML1 CPSF1 SPTBN4 C19orf40 KAT5 PAF1 KIAA1267 ORC3 MAX NR2C1 CDC5L PHF21A HDAC1 ZCCHC12 CHTOP CSRP2BP ZFHX3 SUMO1 TBL1X IFI16 PPP1CC PLCB1 MBD1 CDK12 RPRD1B WBP11 NEK6 INTS2 TOPBP1 WDR61 DAXX INTS7 PHF1 HIPK2 DMAP1 ZZZ3 SNRPC TCEB1 ZNF217 POLR3H CASP8AP2 GLIS2 ANGEL2 SF3A3 SSU72 HDAC5 MED24 FAM48A STRA13 MED16 RBM39 MEAF6 MLL2 NXF1 SMN1 DDX42 MED27 TCEB3 LUC7L2 SMNDC1 ALKBH5 USP22 TRIM37 RFWD3 LUC7L3 TBP MED18 MED20 MED9 PCF11 ENY2 KDM6A C17orf49 ERCC5 SRSF6 FANCA RBM4 GTF2A1L SRRM1 TCEA2 CLK3 GINS4 RUVBL2 EAF1 KAT7 ATR INTS4 HINFP EPC2 SF3B1 TCEB3B TAF5 THOC1 COIL LMNA MGA EZH1 LIN52 ZCCHC18 ELL3 E2F6 APBB1 RBM11 UBN1 WAC ELP3 EAF2 APPL2 CREBBP CDK2 POLR3A KAT8 ZMYM2 PIAS4 SUPT3H SKI PRPF4 RDBP ERCC3 DYDC2 HCFC1 JARID2 KLHL20 POLR3B CSNK2A1 TAF8 ZNF473 EIF4ENIF1 OGG1 DDX3X TRERF1 ELF4 MAPK7 CRCP OGT DKC1 MBD2 PPARGC1A PPIG HNRNPM CPSF7 RB1 NOLC1 NHP2 BRMS1 C8orf4 MED8 TADA3 MED11 CPSF2 POU4F2 INO80C WDR5 ZC3H14 HDAC11 RPA1 MED12 AFF1 GEMIN2 FANCB CLP1 ERCC4 TRIM22 MAGOH RBBP5 TOLLIP NCOA6 GLI3 SP3 ATRX AGGF1 GTF2E2 LSG1 RBM14 ELOF1 RREB1 MED30 USPL1 NUFIP1 HIPK3 DR1 ATPAF2 TRIM27 ASH2L MORF4L1 H2AFY YEATS2 BRPF3 ATXN1 POLR2F PRPF18 INTS3 MED1 GATAD2B PHF19 MED4 SF3A2 GTF2H4 RFWD2 NONO TEX10 MAGEA2 DDX20 POLR2I SENP3 U2AF1L4 APEX1 CCNC PHF17 MED28 TTF2 ATOH8 LEO1 MRE11A HDAC8 ELL2 GEMIN7 RTF1 SUZ12 RERE PSPC1 ERCC6 DDX46 FANCE GPATCH2 MED13 MMS22L FAM60A C12orf41 SHQ1 TAL1 POLR3GL INTS8 EFTUD2 POLR3D NACC2 ZNF541 PML MED31 ATXN7L3 C19orf2 YTHDC1 SATB1 SAP30 POLR2L PNISR CHEK2 SARNP NXF2B GTF2A1 TAF6L PASD1 GEMIN4 NCOR2 SMARCA5 C2orf67 XPO1 EP400 HIF1A POLR3C MED10 TAF4B CHD6 UBE2I PHF20 ELL PIP5K1A C8orf80 FAM48B2 SRY POLR2E TONSL POLR3K TAF9B NFE2 POLDIP3 SIN3B NXF2 ODZ2 BRMS1L DDX1 ATXN2L FANCG RBM27 TAF3 CIR1 CPSF4 EPC1 OIP5 GEMIN6 DDX39B MTA2 SRRM2 SUPT4H1 ARNTL CTDP1 ING5 TH1L GTF2A2 NCOR1 MTOR ZNF496 GTF2H2 TAF5L MED7 USP27X IKBKE MEOX2 RBM25 BAHD1 USP34 GEMIN8 WBP4 EIF3E SIRT1 TOPORS CCNT1 THOC7 CHFR CSNK1A1 AFF2 ACTL6A CHD5 CCNL2 HIPK1 AKAP17A SUV39H1 DUSP11 TOP3A MED22 FBLL1 TOE1 ELP4 MYO6 CBX4 DGKQ ANKRD2 ANKS1B TAF11 MSL3P1 PRPF3 THOC6 C1orf86 WRAP53 WTAP MAGEA2B EDF1 ZNF451 ERCC1 MED15 PRPF19 POLR2G TBX15 TRIM16 ZMIZ1 TAF10 MED26 SNW1 APPL1 ZBTB16 GTF2F2 CDK9 FBL PQBP1 BASP1 CD2BP2 GINS2 SRSF3 HDAC3 CRNKL1 PELP1 BAHCC1 DNTTIP1 POLE3 SMC5 CIITA RBM8A FYTTD1 PRPF8 ATXN7 CCNT2 TAF1L HDAC10 U2AF2 CWC22 KIAA0947 POLR2H NOP58 BRD1 MNAT1 TAF7L PRPF6 TRRAP TRIM69 MECOM CBY1 FANCL DPY30 PLRG1 NUDT21 FAM118B CCNH INTS10 TAF4 CPSF6 WHSC2 RUVBL1 ZBTB1 PTEN BLM FRG1 PSKH1 CDC73 BRD8 PDX1 POLR3G MED29 SUPT7L TFIP11 ISG20 GAR1 WT1 SIRT2 RBBP4 GINS1 GLI2 MTDH SMC6 TIMM50 TADA2B POLR2J2 NACC1 RPAP2 FAM206A FANCC MLIP CDK7 PPP1R8 MED19 CDK13 TADA1 AEBP2 NPAT NRIP1 CBX5 SMN2 TP53INP1 ACIN1 DYDC1 RCHY1 TERT NSRP1 ELP2 PYHIN1 TP53 RRP8 TCEB3CL EP300 SAP130 PHF5A SON PPIH SP100 KIAA1310 GATAD2A RNF4 CCDC101 GTF2H1 POLR2M THOC4 SRSF10 PNN POLR3F EPAS1 RNF6 CBLL1 FIP1L1 MMS19 WDFY3 RPAIN MEF2C PARK7 GTF2H3 TCEB2 TCF7L2 BAZ2A ZNF830 ING3 TNRC18 INTS9 RPRD2 HR HEY2 CSTF2 CORT ZC3H8 SRSF2 PCGF2 RNPS1 ZC3H13 SALL2 RDM1 MAP3K7 RGS14 IKBKAP SHFM1 KIAA1429 MAML3 DYRK1A TBPL1 PAXIP1 HDAC6 C4orf44 SETD1B KAT2A N4BP1 KAT6B FAM76B RPRD1A SART1 SLU7 MLL KAT6A KAT2B PATL1 NSMCE2 MED21 CHD3 ARL6IP4 EZH2 MCRS1 C1orf124 FAM48B1 CDK8 HSPB7 ZNF259 SENP2 WDR82 MEN1 POLR1D POLR2J PRDM4 HDAC4 MBD3 PRPF31 EED PRPF40B CPSF3L ING2 RAD51 MLL3 C16orf53 SNRNP70 MTF2 MORF4L2 CDC45 LOC100506888 UBAP2L CPSF3 SS18L1 MORC3 YLPM1 MAML2 FMR1 COBRA1 LSM10 ACTB HIRA AFF4 NXT1 SUMO2 BRPF1 SRSF4 TAF9 POLR2A TDG MLL4 POLR1C PHF15 METTL3 PIAS2

GO_CENTRIOLE A cellular organelle, found close to the nucleus in many eukaryotic cells, consisting of a small cylinder with microtubular walls, 300-500 nm long and 150-250 nm in diameter. It contains nine short, parallel, peripheral microtubular fibrils, each fibril consisting of one complete microtubule fused to two incomplete microtubules. Cells usually have two centrioles, lying at right angles to each other. At division, each pair of centrioles generates another pair and the twin pairs form the pole of the mitotic spindle. CCDC151 CEP76 CEP19 DZIP1L C1orf96 CEP192 NIN CEP128 CETN2 HSPA6 CENPJ WRAP73 LOC100653515 ALMS1 TOPORS CETN1 AHI1 CEP170 TSKS LRRCC1 CROCC BBS4 CEP164 MKS1 RP2 SDCCAG8 KIAA0586 FAM154A CNTROB MPHOSPH9 TSSK2 WASH1 ODF2 RAN POC1B PCM1 CNTLN CEP135 CETN3 TUBD1 C2CD3 TOP2A CEP152 IFT20 HERC2 WDR62 RAB8A AURKA PLK4 C10orf90 NEDD1 FTCD POC5 DZIP1 AGBL2 CEP55 SIRT2 OFD1 KIAA1731 CEP41 STIL HSPA1B CEP120 STARD9 NUBP2 POC1A SPICE1 KIAA1009 CEP290 CCDC78 PARP3 HTT TTBK2 WDR34 FBF1 NEURL4 CCNF PCNT IQCB1 KIF24 TUBG1 CEP89 CEP63 MDM1 ROCK1 CCP110 CCDC92 CAPG IFT88 CCDC41 PLA2G3 CEP104 IFT52 AGBL4 C16orf80 TUBGCP3 WASH3P SASS6 SCLT1 HAP1 HSPA1A SFI1 CCHCR1 CCDC67 CEP250 PLK2 CCDC146 NUBP1

GO_NUCLEOID The region of a virus, bacterial cell, mitochondrion or chloroplast to which the nucleic acid is confined. POLRMT MTERF TFB1M HSPA9 SHMT2 ACADVL DHX30 ELAC2 TOP2A RG9MTD1 MTERFD3 TOP2B ATP5B VDAC1 LONP1 GRSF1 SUPV3L1 POLG2 C6orf125 SLC25A5 KIAA0391 DDX28 C10orf2 VDAC2 POLDIP2 HADHB LRPPRC HADHA LRRC59 DNA2 CLPX TFB2M TFAM SSBP1 FASTKD2 ATAD3A CPS1 C17orf42 POLG MPG TOP1MT TUFM TERT FASTKD5 DBT

GO_TERMINAL_BOUTON Terminal inflated portion of the axon, containing the specialized apparatus necessary to release neurotransmitters. The axon terminus is considered to be the whole region of thickening and the terminal bouton is a specialized region of it. SYT1 AP3D1 CALCA ILK STXBP1 SLC18A3 APP TH OXT LRRK2 SNCA NTRK2 ADORA1 SEPT5 RAB3A GRIK3 CAD KCNC2 GRIK5 GHRH PVALB P2RX4 RAB7A STX3 TBC1D24 VAMP1 CPLX2 AP1S1 SYN1 PTPRN2 SYP CABP4 NAPA CPLX1 STX6 SYNGR1 SLC18A2 OPHN1 CCK AP2M1 GRIK2 DPYSL2 NTSR1 UNC13B POLG AAK1 DNAJC5 SYT7 SV2A ADCYAP1 SYNJ1 CALB2 NMU PRSS12 SLC18A1 CNGB1 GRIN1 PFN2 P2RX3 RAB5A CALB1

GO_SPINDLE_POLE Either of the ends of a spindle, where spindle microtubules are organized; usually contains a microtubule organizing center and accessory molecules, spindle microtubules and astral microtubules. RASSF1 CEP95 CALM3 CTNNB1 CUL3 HAUS8 NUDCD2 CTDP1 MAP2K1 IKBKG PLEKHG6 FAM82A1 KATNA1 KIAA1383 NEDD9 GOLGA2 UMOD RANGAP1 KLHL22 KIF2A TMEM48 DYNC1LI1 MAD1L1 CEP44 CENPF CEP63 CEP89 FAM82A2 CDC25B TUBG1 SGOL1 CKAP5 ODF2 TUBGCP4 POC1B TPX2 NEK7 TUBGCP2 PPP2CA CCDC165 EML1 AURKA MZT1 DNAAF1 ZW10 NDE1 HAUS1 FAM82B FRY CEP19 UNC119 CCDC155 PSRC1 NIN CEP128 OR2A4 CSPP1 FAM83D LRMP VPS4B TBCCD1 KIF11 NPM1 PRC1 C19orf46 FAM110A CEP104 WDR73 DDX11 MAD2L1 SMC3 KATNB1 CDC14A TUBGCP3 ASPM FAM175B TOPBP1 NUP62 LATS1 PKP4 NEK6 NEK2 PLK1 FAM110C CCDC99 CKAP2 NUBP2 POC1A CKAP2L CDK5RAP2 TUBGCP5 C15orf23 CDC20 C1orf135 FBF1 BRCC3 CDC6 CCNB1 DYNC1I1 ANKRD53 SBDS MAPK14 TTC28 DCTN1 TUBGCP6 KNTC1 NEDD1 WDR62 RAB11A RGS14 CALM1 KLHL21 LATS2 AURKC KIF20B TNKS BIRC6 DLGAP5 DSN1 ALMS1 TOPORS CALM2 NUMA1 PPP2CB AURKB VPS4A CEP85 CETN1 RASSF10 SPAG5 GPSM2

GO_ENDOCYTIC_VESICLE_LUMEN The volume enclosed by the membrane of an endocytic vesicle. SCGB3A2 LTF HYOU1 SPARC CALR SAA1 HBB APOE HSP90B1 HP HSP90AA1 APOA1 HBA2 HBA1 HSPH1 HPX APOB

GO_CELL_CELL_CONTACT_ZONE Extended zone of intimate apposition between two cells containing one or more types of intercellular junctions, e.g., the intercalated disk of muscle. DSC2 GJA5 BAIAP2L2 CAV3 TIAM1 CDH2 RAP2B FLCN SLC9A1 FGFRL1 GJC1 HAMP DSG2 TMEM65 SCN1B ANKRD23 SCN5A AHNAK SLC8A1 OPALIN PKP2 DLG1 PCDH9 CTNNA3 FGF13 NRAP FLOT2 CTNNB1 PAK1 DES KCNJ11 VAMP5 PGM5 RANGRF KCNJ2 ANK2 OBSL1 ATP1B1 ATP1A1 SCN1A DSP TJP1 CAMK2D JUP ITGB1 FLOT1 ACTN1 SCN4B JAM3 CTNNA1 VCL RAP2C GJA1 ATP1A2 ANK3 AKAP6 PKP4 MYH1 YWHAH KCNA5

GO_MITOCHONDRIAL_MEMBRANE_PART Any constituent part of a mitochondrial membrane, either of the lipid bilayers that surround the mitochondrion and form the mitochondrial envelope. TIMM50 NDUFS6 NDUFS4 NDUFAF1 GRPEL1 UQCRC1 WDR93 RHOT1 RHOT2 NDUFB9 COX4I1 L2HGDH PPIF USMG5 NDUFA1 ATP5G3 ATP5E BID NDUFB3 NDUFA3 COX8A NOA1 CHCHD3 COX5A TOMM5 TOMM22 TOMM6 NDUFA6 ATP5I C14orf2 SDHA NDUFA4L2 ATP5H TOMM40 FIS1 ABCB6 IMMP1L NDUFAB1 PAM16 IMMT APOO ATP5L2 CCDC109B TIMM23 CCDC56 COX7B DUSP21 NDUFV3 NDUFV2 PISD NDUFA13 SLC25A6 SYNJ2BP UQCR10 NDUFA9 ATP5G2 HSPD1 UQCRB NDUFC2 TIMM22 TOMM70A FUNDC1 NDUFA4 TOMM7 NDUFC1 COX8C TMEM11 COX18 SOX10 OPA1 COX4I2 NDUFA8 NDUFS8 TIMM17B UQCRC2 SARM1 MUL1 TOMM40L BAK1 C22orf32 MFN2 MFF COQ7 SDHB GRPEL2 COX6A2 NDUFB1 CCDC90A ATP5EP2 NDUFS5 SURF1 MICU1 ATP5O NDUFB11 NDUFS7 ATP5C1 BDH1 NDUFB8 NDUFS2 NDUFA7 ATPIF1 TIMM9 STAR COX7A2L NDUFB2 PMPCB ETFDH ATP5G1 NDUFA12 SDHD NDUFS3 PPOX COQ5 CHCHD6 OXA1L SLC24A6 DMPK SPG7 COX5B MCU COX7B2 LOC100652748 ARMCX3 PARK7 BCS1L NDUFB4 TMEM70 ATP5J BNIP3 ATP5L ATP5J2 NDUFS1 FUNDC2 UQCRH NDUFA5 TOMM20 NNT ATP5F1 NDUFA10 FOXRED1 C15orf48 SDHC CPT1A GDAP1 UQCRFS1 C20orf7 C11orf83 LRRK2 SNCA C4orf49 SAMM50 NDUFB5 ATP5B COX7A2P2 NDUFA11 NDUFB7 ABCB10 C7orf44 C18orf55 EFHA1 COX7A1 PINK1 AFG3L2 APOOL NDUFV1 ANKZF1 LYN IMMP2L TIMM10 ATP5A1 COX15 TIMM17A COX7A2 NDUFB6 COX6A1 TAMM41 NDUFA2 TOMM20L COX6B2 NDUFB10 ATP5D C19orf70

GO_CHD_TYPE_COMPLEX A SWI/SNF-type complex that contains a subunit from the CHD(Chromodomain helicase DNA-binding) family. The CHD family is characterized by two signature sequence motifs: tandem chromodomains located in the N-terminal region, and the SNF2-like ATPase domain located in the central region of the protein structure. MBD3 APPL2 GATAD2B HDAC2 NACC2 RBBP4 MTA2 CHD4 GATAD2A RBBP7 CHD3 SALL2 APPL1 SALL1 HDAC1 CHD5 CSNK2A1

GO_COATED_MEMBRANE A single or double lipid bilayer with any of several different proteinaceous coats that can associate with membranes. Membrane coats include those formed by clathrin plus an adaptor complex, the COPI and COPII complexes. AP1M1 EPN2 CHMP4B AP1G2 AP3S2 COPE AP1S1 TMED7 VMA21 SEC31A AP2B1 AP1M2 GGA3 EPS15 AP3B1 SCN10A ARCN1 SEC24D CLTC SCYL1 AP3D1 COPB2 AP2S1 NECAP2 AP3M2 C3orf58 SCLT1 SEC31B LDLRAP1 CLTB COPG C20orf29 AP4E1 SEC23B AP1G1 GGA2 VPS39 COPG2 MUDENG NCALD AP4B1 AP1B1 VPS33A COPB1 TMED3 EGFR EPS15L1 AP2A1 CLTA DKFZp761E198 AP1S3 SEC24A AP3M1 IGF2R AP1S2 SEC24B PANK1 SEC24C KIAA0415 SLC18A3 AP3S1 CHMP1B ARL6 BAIAP2L2 VPS18 SGIP1 VPS41 CHMP4A SEC23A COPZ2 DNM1 KCNQ5 COPA NECAP1 SYNRG COPZ1 AP2A2 AP3B2 AFTPH PICALM SYNJ1 CHMP2A TBC1D5 VPS33B AP2M1 GGA1 AP4M1 CLTCL1

GO_EUKARYOTIC_TRANSLATION_INITIATION_FACTOR_3_COMPLEX A complex of several polypeptides that plays at least two important roles in protein synthesis: First, eIF3 binds to the 40S ribosome and facilitates loading of the Met-tRNA/eIF2.GTP ternary complex to form the 43S preinitiation complex. Subsequently, eIF3 apparently assists eIF4 in recruiting mRNAs to the 43S complex. The eIF3 complex contains five conserved core subunits, and may contain several additional proteins; the non-core subunits are thought to mediate association of the complex with specific sets of mRNAs. ABCE1 EIF3CL EIF3I EIF3H EIF3F EIF3L EIF3B EIF3M COPS5 EIF3J DDX3X EIF3A EIF3K EIF3C EIF3G EIF3E EIF3D

GO_MYOSIN_II_COMPLEX A myosin complex containing two class II myosin heavy chains, two myosin essential light chains and two myosin regulatory light chains. Also known as classical myosin or conventional myosin, the myosin II class includes the major muscle myosin of vertebrate and invertebrate muscle, and is characterized by alpha-helical coiled coil tails that self assemble to form a variety of filament structures. MYH14 MYH13 TRIM32 MYH6 MYH7 MYH11 MYH1 TTN SHROOM1 MYH2 MYLPF MYH3 MYOM1 MYH4 MYL3 MYL6B SHROOM4 MYL12B MYH9 MYL1 MYBPC3 MYH10 MYL9 MYH8 MYL5

GO_TRANSCRIPTIONAL_REPRESSOR_COMPLEX A protein complex that possesses activity that prevents or downregulates transcription. NCOR1 ZNF224 CTBP1 SALL1 CSNK2A1 RCOR1 LIN37 LIN9 HDAC3 SKI TBL1XR1 ZFPM1 SKOR2 MTA2 RCOR2 GATAD2A BAHCC1 C1D HDAC1 SMARCE1 YWHAB CORO2A TBL1X DDX20 ZBTB16 APPL1 MIER1 ETV3 LIN52 MBD3 HDAC4 APPL2 INSM1 TBX15 GATAD2B RRP8 CDX2 JUN ELANE ELP2 GATA1 ARID4A SMARCC2 N4BP2L2 DEPDC1 RBBP7 CBX5 HDGF CHD3 ZNF350 SIRT2 RBBP4 SMARCA5 NCOR2 CHD4 SKIL SPEN HDAC2 GFI1 GLI3 SP3 PRDM16 GPS2 JAZF1 SUV39H1 HEY2 CHD5 PHF12 RCOR3 RLIM TNRC18 PASD1 SALL2 HMGB1 REST BAZ2A NACC2 SIN3A BAHD1 RBBP8 SKOR1 TFAP4 CCND1 SIRT1 AKIRIN2 CTBP2

GO_PROTEIN_COMPLEX_INVOLVED_IN_CELL_ADHESION Any protein complex that is capable of carrying out some part of the process of cell-cell adhesion. CD80 CD28 ITGAL ITGB2 ITGA4 ITGAX ITGB6 ITGAD ITGA1 ITGA7 ITGB1 ITGB4 ITGA2 ITGA8 ITGB5 ITGA10 ITGA5 ITGAV MYH9 ITGB7 ITGB8 CTLA4 ITGA2B ITGAM NPNT LYN ITGA9 ITGAE ITGA6 ITGA11 TSPAN32 ITGA3 ITGB3

GO_NEURON_SPINE A small membranous protrusion, often ending in a bulbous head and attached to the neuron by a narrow stalk or neck. EPHA4 SYT11 NLGN1 NR1D1 DNM3 MYH10 GRM3 GIPC1 LPAR1 SIPA1L1 NOS1 TIAM1 ALS2 ZMYND8 PTEN GRIN1 GPM6A SHANK2 CANX PALM STRN NTSR1 ACCN1 PALMD NEURL PDE4B SLC8A1 DGKI CRIPT ARHGAP32 ARC SLC8A3 FRMPD4 SLC9A6 CRYAB SHANK3 ITPKA PRKAR2B SYNDIG1 PPP1CA CDK5R1 CNIH2 ARRB1 ASAP1 PPP1R9B SHANK1 PPP1R9A ANKS1B STRN4 SEPT11 NRGN KCNN2 ATP1A2 GRIA1 RGS14 GRID2 RGS10 GPER SEZ6 KCND2 LZTS1 DLG4 LRRC4 CTTNBP2 P2RX3 ARRB2 SRI COMT DTNBP1 APBB1 LAMA2 ODZ2 CTTN OPHN1 ARHGAP44 FUS FXR1 BAIAP2 GRID2IP MOB4 MAP1B HPCA MT3 SRGAP2 FBXO2 MTMR2 SLC8A2 CTNND1 SYNPO FARP1 STX4 KCNJ2 DVL1 CNN3 EEA1 ACTN2 DRD2 ARF4 ADORA1 APP MYL7 IGF2BP1 ATP1A3 ARFGEF2 P2RX4 PPP1CC PTPRO FMR1 ITGB1

GO_BLOC_1_COMPLEX A protein complex required for the biogenesis of specialized organelles of the endosomal-lysosomal system, such as melanosomes and platelet dense granules. Many of the protein subunits are conserved between mouse and human; the mouse complex contains the Pallidin, Muted, Cappuccino, Dysbindin, Snapin, BLOS1, BLOS2, AND BLOS3 proteins. BLOC1S3 C19orf50 SNAPIN CNO KIAA1033 SNAP47 PLDN BCAS4 BLOC1S1 SNAP25 DTNBP1 BLOC1S2 PI4K2A MUTED STX12

GO_DENSE_CORE_GRANULE Electron-dense organelle with a granular internal matrix; contains proteins destined to be secreted. SYT5 SOD1 MYRIP NCS1 VPS13A CRHBP SYT7 SCG2 ADAM8 SYT4 SYT1 SYT9

GO_CELL_BODY_MEMBRANE The plasma membrane of a cell that bears surface projections such as axons, dendrites, cilia, or flagella, excluding the plasma membrane on cell projections. KCNB2 FLRT1 KCNA2 KCNC1 SLC4A8 GABRA5 KCNC2 HPCA RGS8 UNC5A CX3CR1 DAB2IP KCND2 ATP2B1 AMIGO1 TACR3 KCNB1 KCNE3

GO_INTRINSIC_COMPONENT_OF_PEROXISOMAL_MEMBRANE The component of the peroxisomal membrane consisting of the gene products and protein complexes having either part of their peptide sequence embedded in the hydrophobic region of the membrane or some other covalently attached group such as a GPI anchor that is similarly embedded in the membrane. FIS1 PEX16 ABCD1 PEX11B PEX10 PEX26 PEX2 PEX13 SLC25A17 PEX3 PEX11G SLC27A2 PEX12 PEX11A

GO_PRESYNAPTIC_ACTIVE_ZONE A specialized region of the plasma membrane and cell cortex of a presynaptic neuron; encompasses a region of the plasma membrane where synaptic vesicles dock and fuse, and a specialized cortical cytoskeletal matrix. PPFIA2 PCLO DGKI SLC17A7 ERC2 GPER SV2A ADORA1 UNC13B SLC32A1 PPFIA3 RIMS4 PPFIA1 ERC1 UNC13C RIMS1 GRM7 RIMS3 NUFIP1 BSN UNC13A GAD1 NANOGNB RIMS2 SYT11 SYN1 SYP PPFIA4 FZD3

GO_NUCLEAR_OUTER_MEMBRANE The outer, i.e. cytoplasm-facing, lipid bilayer of the nuclear envelope; continuous with the endoplasmic reticulum of the cell and sometimes studded with ribosomes. NAV3 ITPR3 SYNE2 DHCR7 SYNE1 DMPK C19orf46 GLTPD1 TMEM109 NXNL1 C14orf49 LTC4S SNCA GUCY2F EMD PSEN1 RETSAT LRPPRC SIGMAR1 GUCY2D CCDC155 GHRHR NUTF2

GO_PLATELET_DENSE_TUBULAR_NETWORK A network of membrane-bounded compartments found in blood platelets, where they regulate platelet activation by sequestering or releasing calcium. The dense tubular network exists as thin elongated membranes in resting platelets, and undergoes a major ultrastructural change, to a rounded vesicular form, upon addition of thrombin. F2R SERPINA5 ATP2A2 MRVI1 ITPR2 ATP2A3 ATP2A1 ITPR1 EPB49 ITPR3 EHD1

GO_PROTEIN_PHOSPHATASE_TYPE_1_COMPLEX A protein complex that possesses magnesium-dependent protein serine/threonine phosphatase (AMD phosphatase) activity, and consists of a catalytic subunit and one or more regulatory subunits that dictates the phosphatase's substrate specificity, function, and activity. PPP1R9B PPP1R2P9 PPP1R2 PPP1R2P3 PPP1R15A PPP1CB NCK1 SHOC2 PPP1CA PPP1R15B PPP1R3B PPP1R2P1 PPP1R11

GO_TRANS_GOLGI_NETWORK_MEMBRANE The lipid bilayer surrounding any of the compartments that make up the trans-Golgi network. SCAMP2 AP4M1 RAB43 USP6NL ARFIP2 KIAA1432 RAB9A CD74 C11orf2 COG3 KIF13A RGP1 LGR6 M6PR STX10 HLA-DRB3 RAB31 TMEM165 LLGL1 CABP7 HLA-DQA2 COG2 COG4 COG1 CLVS1 ATP7B VPS53 ARL1 ARFIP1 CLTA RABEPK MYO1B VPS52 AP1S3 AP4S1 AP1S2 IGF2R HLA-DQA1 HLA-DRB5 VTI1A LGR5 HLA-DRB1 AP4B1 AP1B1 ARFRP1 CLVS2 HLA-DPA1 TMEM79 RHOBTB3 AP1G1 AP4E1 HLA-DQB1 MMP24 TGOLN2 HLA-DRA CLIP3 COG8 HLA-DRB4 RAB6A MARCH1 HLA-DQB2 APP CLTC VAMP3 COG7 COG6 CALN1 AP1M2 VPS54 STX16 GOLPH3L HLA-DPB1 AP1S1 STX6 AP1M1 SLC24A5 COG5 SCAMP5 SYS1 VAMP4

GO_GOLGI_ASSOCIATED_VESICLE_MEMBRANE The lipid bilayer surrounding a vesicle associated with the Golgi apparatus. CNGB1 PI4KA PKD1 HM13 KDELR1 LDLRAP1 COPG COPA CNGA2 COPZ2 CLTB AFTPH SPPL3 SPPL2B RHO SYNRG COPZ1 TMEM199 RASSF9 ZDHHC13 CLTCL1 COPG2 GPR89A IMP5 NCALD TMED2 AP1G2 RHOQ COPB1 TMED3 SPPL2A COPE TMED7 AP1S1 CLTA AP2A1 CNGA4 ARCN1 GJA1 CFTR ZDHHC17 COPB2 CSPG5 ITM2B C3orf58 CLTC NRGN SCYL1 SLC18A3 GOPC

GO_PHOTORECEPTOR_OUTER_SEGMENT_MEMBRANE The membrane surrounding the outer segment of a vertebrate photoreceptor. OPN1SW RHO CDHR1 PHLPP2 GNB1 GNAT1 OPN1MW2 PROM1 ROM1 CNGA1 OPN1LW DHRS3 CNGA3 GNAT2 OPN1MW NAPEPLD

GO_MYOSIN_COMPLEX A protein complex, formed of one or more myosin heavy chains plus associated light chains and other proteins, that functions as a molecular motor; uses the energy of ATP hydrolysis to move actin filaments or to move vesicles or other cargo on fixed actin filaments; has magnesium-ATPase activity and binds actin. Myosin classes are distinguished based on sequence features of the motor, or head, domain, but also have distinct tail regions that are believed to bind specific cargoes. MYO1A CGNL1 MYBPH MYH16 MYO1D MYO6 SHROOM1 MYH1 MYO19 MYO9B MYL3 MYO3B MYO7A MYH8 MYL9 MYO1B MYL4 TRIM32 MYH13 MYH14 MYLPF MYBPC2 MYL12A MYO10 MYH9 MYO9A MYO18A MYOM1 MYO1E MYO1C MYO16 MYL5 MYH10 MYBPC3 MYO1F MYO5A MYH6 MYO18B MYH15 MYO15A BMF MYO5B MYL7 MYO1G CGN MYL6 MYH2 MYH11 SHROOM4 MYL6B MYH7B MYH3 CCDC102A MYO7B MYBPC1 ACTG2 MYO1H TTN DYNLL2 MYH7 MYL12B MYO5C MYH4 MYO3A MYOM2 MYL1 MYL2

GO_LARGE_RIBOSOMAL_SUBUNIT The larger of the two subunits of a ribosome. Two sites on the ribosomal large subunit are involved in translation, namely the aminoacyl site (A site) and peptidyl site (P site). RPL39 MRTO4 MRPL22 MRPL11 RPL3 RPL29 MRPL46 RPL36 RPL27A RPL22 MRPL49 RPL37A NSUN3 RSL24D1 RPL18A RPL24 MRPL16 RPL13 RPL41 RBM3 RPL18 MRPL52 C7orf44 MRPL12 RPL12 MRPL55 RPL15 MRPL43 MRPL1 RPL7L1 MRPL36 RPL19 RPL39P5 RPL5 RPL27 RPL8 RPLP2 RPL23A RSL1D1 RPLP0 RPL3L MRPL28 RPL7 RPL26L1 RPL23 MRPL2 MRPL30 MRPL17 NHP2L1 RPL13A MRPL23 RPL6 ICT1 MRPL47 RPL36AL RPLP1 RPL9 RPL10A RPL35 RPL21 MTERFD2 RPL37 RPL10 RPL7A RPL32 RPL39L MRPL15 MRPL10 C12orf65 RPL30 RPL11 RPL34 MRPL33 RPL14 MRPL32 MPV17L2 RPL26 MRPL3 MRPL27 NHP2 RPL38 SURF6 RPL22L1 ZNF622 RPL4 NSUN4 RPL28 MRPL13 MRPL19 FXR2 MRPL51 MRPL41 RPLP0P6 RPL31 MRPL21 RPL17 RPL36A RPL10L RPL35A

GO_PHOTORECEPTOR_OUTER_SEGMENT The outer segment of a vertebrate photoreceptor that contains discs of photoreceptive membranes. MYO5A GNAT2 RP1L1 C2orf71 CNGA3 SAG PRPH2 CNGB1 PDE6B BBS7 MYRIP SHANK2 GNAQ OPN1MW2 ARR3 PROM1 RHO PCDH15 OPN1SW BBS4 MYO7A OPN1MW IFT140 GRK1 DHRS3 PDE6G GNB5 RPGR PPEF2 RAB27A IFT20 OPN5 CIB2 RP1 CDHR1 OCRL CNGB3 GNGT1 PTPRK GUCA1C GUCY2D GUCA1A RGS9 USH1C PDE6A GUCY2F GNA11 ABCA4 GUCA2B GNB1 IQCB1 MAP1B ATP8A2 INHA RGS9BP NAPEPLD GRK4 OPN1LW CNGA1 WDR19 TULP1 ROM1 MAK SPTBN5 GNAT1 CACNA1F GUCA1B MERTK PDC PHLPP2 PTGS1 GRK7

GO_NUCLEAR_EXOSOME_RNASE_COMPLEX_ Complex of 3'-5' exoribonucleases found in the nucleus. EXOSC6 EXOSC7 DIS3L2 EXOSC9 EXOSC8 DIS3 C1D DIS3L EXOSC10 EXOSC2 EXOSC3 EXOSC5 MPHOSPH6 EXOSC4 EXOSC1

GO_LYTIC_VACUOLE_MEMBRANE The lipid bilayer surrounding a lytic vacuole and separating its contents from the cytoplasm of the cell. COL6A1 STX7 RNF13 AP2S1 CD1B SLC29A3 AP1M2 AP2B1 RHEB ANKFY1 GNAI1 HPSE TLR3 AP1S1 ATP6V1B2 MTOR GNB2 EGF RAB5C SLC26A11 SLC30A3 VPS33A AP1G1 TMEM79 TPCN1 TLR9 LAMTOR2 OCA2 GNA11 SLC17A5 ITM2C LOH12CR1 SLC48A1 ABCD4 GOPC CD63 UBA52 HLA-DQA2 HSPA8 TMEM59 FLOT1 ATP6V1A LNPEP C1orf85 AP1S2 AP3M1 SCARB2 ARRB1 EEF1A2 ECE1 CLN3 TMEM192 VPS33B TMBIM1 DPP4 HSP90AB1 CLCN5 AHNAK GDAP2 SLC2A8 ACPP ATP6V1D VPS41 SPNS3 CYBASC3 TMEM106B VPS18 RNF152 GNAQ TMEM44 HLA-DQB2 AP3D1 CLTC ATP6V0A2 WDR11 PLD1 RAB2A P2RX4 ABCA5 NCSTN RAB14 HLA-DPB1 ATP13A2 RILP STK11IP AP1M1 PSEN1 MUDENG MARCH8 HLA-DRB1 GNB1 AP1B1 NEU1 HLA-DPA1 SPNS2 ATP6V1C2 ZNRF2 HLA-DRA HLA-DOA BLOC1S2 LAMTOR1 ATP11C HLA-DOB VPS35 HLA-DRB4 TMEM55B C7orf28B HM13 MARCH1 TMEM9B UBA1 VASN MYO6 DKFZp761E198 C10orf32 VAMP1 ANXA6 CLTA ENPP1 IGF2R TCIRG1 RRAGB LAMTOR3 HGSNAT OSTM1 RDH14 C18orf8 LRP2 PQLC2 MYLPF AP2M1 SIDT2 GPLD1 STARD3NL FAM176A TLR8 CD164 TMEM63A CP VPS16 RAB12 M6PR ABCA2 CUBN MMD MEF2BNB AP3B1 BLOC1S1 RAB7A TMEM8A SNAPIN SPHK2 DEPDC5 C3orf55 GPR143 SCARB1 STX17 MARCH9 C17orf59 IMP5 SLC12A4 ATP11A ATP6V0C SLC38A9 KIAA1324 TMEM150C ATP6V0A4 PNPLA7 SPPL2B SPPL3 CCZ1 SLC11A2 CTNS LMBRD1 CLEC16A ACP2 LITAF ATP6V1E1 MCOLN1 TM9SF1 SLC7A14 TMEM175 ANXA2 HLA-DRB3 PLA2G4F TMEM165 ARL8A VAMP8 AZU1 CFTR GNAI3 PLA2G4E AP2A1 RRAGA AP1S3 CLCN6 CTSA LAMP3 DIRC2 ATP6V0D1 LAMP2 SYT7 DNAJC5 DAB2 PLEKHM1 SLC36A1 TECPR1 TLR7 CD74 SLC30A4 GAA CD1D TSPAN1 C2orf18 ATP6V1H C19orf28 DRAM2 KIAA1609 RPTOR HLA-DMB WDR41 ARL8B LRP1 SPG11 CLN5 SNX14 PI4K2A TMEM9 DRAM1 LAMP1 SPPL2A TRIM23 SLC30A2 MFSD8 TPCN2 CLCN7 GPR137B EEF1A1 VPS39 HLA-DQB1 ATP6V1G1 TMEM55A PSAP C20orf29 OSBPL1A MARCH2 TM6SF1 MIOS PLEKHF1 CD68 ABCC10 GNB4 DNAJC13 DAGLB SLC15A4 SPNS1 C19orf50 HLA-DMA SLC15A3 ATP6V1C1 ABCB9 SORT1 GFAP TMEM74 MYO7A ZFYVE26 HLA-DQA1 SLC44A2 HLA-DRB5 NSF NPC1 VPS11 HBXIP ENPEP LAPTM5 STX8 AP2A2 NAPG C7orf59 VAMP7 IFITM3 C2orf28 STARD3 GBA ANPEP VTI1B

GO_NUCLEAR_MATRIX The dense fibrillar network lying on the inner side of the nuclear membrane. PRKCD CAD ARFGEF1 AHCTF1 CENPW MAEA MBD1 ZNF326 DDX39B PRPF40A ENC1 ODZ1 MATR3 HLCS SORBS1 ATXN7 PSPC1 CXXC1 OGG1 GHRHR PSMA6 RNASEL SMC3 ATXN1 AKAP8 SPTBN4 RUNX1T1 SMARCAD1 CASK TP53 CENPF MEN1 YEATS4 KIN HAT1 NONO DCAF7 ALOX5 KIF4A GMCL1 PHF5A CFL1 MORC3 ATN1 PIAS4 SNW1 POLA1 MYB TGFB1I1 ZNF703 KRT8 NCOR2 CFL2 GFI1B RGS12 SRPK1 CEBPB DNTT PAXIP1 GFI1 RUVBL2 SRRM1 SFPQ GMCL1P1 CLIC4 NUFIP1 NELF FIGN SCAF8 KIF4B THOC1 ZNF350 SPARC PML CHMP1A ERCC8 TEP1 PRKCZ SATB2 JAK2 PHACTR3 PHB2 RUVBL1 SATB1 LMNB1 PPIG HNRNPM TINF2 NUMA1 YY1 XPOT ATXN3 UHRF1 AKAP8L BLM LRIF1 DNMT3A

GO_H4_HISTONE_ACETYLTRANSFERASE_COMPLEX A protein complex which is capable of H4 histone acetyltransferase activity. RUVBL1 ING3 KIAA1267 YEATS4 MSL3 MORF4L1 YEATS2 KAT5 MAP3K7 KAT8 UBAP2L EPC1 CSRP2BP MORF4L2 WDR5 MBIP BRD8 TADA3 MEAF6 ACTL6A HCFC1 ACTB C20orf20 POLE4 POLE3 RUVBL2 MSL2 C2orf67 EP400 CCDC101 MSL3P1 TRRAP C12orf41 KAT2B DR1 MSL1 KAT2A EPC2 DMAP1 ZZZ3

GO_RETROMER_COMPLEX A conserved hetero-pentameric membrane-associated complex involved in retrograde transport from endosomes to the Golgi apparatus. The budding yeast retromer comprises Vps35p, Vps29p, Vps26p, Vps5p, and Vps17p. The mammalian complex shows slight variation in composition compared to yeast, and comprises SNX1 or SNX2, SNX5 or SNX6, VPS26A or VPS26B, VPS29, and VPS35. VPS26B TBC1D5 SDCCAG3 SNX27 RAB7A ANKFY1 SNX1 SNX6 SNX2 DENND5A FAM21C VPS35 SNX5 TRIM27 VPS26A MAGEL2 SNX3 VPS29 DCTN1 SNX8 M6PR

GO_MRNA_CLEAVAGE_FACTOR_COMPLEX Any macromolecular complex involved in cleavage or polyadenylation of mRNA molecules. CPSF6 CPSF7 TUT1 NUDT21 CLP1 CPSF4L SSU72 CPSF1 CSTF2T WDR33 CPSF3 PCF11 FIP1L1 PIP5K1A ZC3H3 CSTF2 CPSF2 CPSF4 CSNK1A1

GO_ARP2_3_PROTEIN_COMPLEX A stable protein complex that contains two actin-related proteins, Arp2 and Arp3, and five novel proteins (ARPC1-5), and functions in the nucleation of branched actin filaments. ARPC5 ACTR2 ACTR3B ARPC1B ARPC1A ARPC5L ACTR3C ARPC4 ARPC2 ACTR3 ARPC3

GO_NUCLEAR_EUCHROMATIN The dispersed less dense form of chromatin in the interphase nucleus. It exists in at least two forms, a some being in the form of transcriptionally active chromatin which is the least condensed, while the rest is inactive euchromatin which is more condensed than active chromatin but less condensed than heterochromatin. TBP KLF4 ALKBH1 NELF H3F3C RBMX CREB1 CBX3 TRIM28 TRIM24 HIST1H1D CTNNB1 SETD1A H2AFZ PPARGC1A RUVBL2 H1F0 SIRT1 TCF7 JUN NR1H4 HIST1H1C ASH2L TRNP1 HIST1H1A

GO_NUCLEAR_MEMBRANE Either of the lipid bilayers that surround the nucleus and form the nuclear envelope; excludes the intermembrane space. TMEM18 CERS2 SENP1 AQP1 TOR1B PRICKLE1 PNPLA7 ITPR1 LEMD2 OSBPL6 GTPBP4 DNAJC2 LRPPRC TMEM38B SEPHS1 GUCY2F AAAS CCND2 ZMPSTE24 SHISA5 TSGA10 NUP153 FZR1 MRTO4 SCAI IFI27 AZI1 WDFY3 CEPT1 TPR ZNF224 POM121C RNF6 PAK1 TMEM43 NOS1AP ATP1B4 LBR IPO4 ITPR3 CEP170 RPS6KA2 PLRG1 RRP12 CCDC155 PAFAH1B1 SPAST QSOX2 TMEM120B PRPF38A TMEM57 TOR1A TNPO2 TMC8 P2RX7 NUP133 MTDH DPY19L3 AEN CERS4 P2RX2 BRIP1 RANBP2 CEP70 LMNA TOR1AIP1 WDR3 SUN1 TMEM38A PHF8 NUP155 ABL1 TFPT NUPL2 SUN2 KLK6 SENP2 SYNE1 C11orf85 KIAA1161 DPY19L1 C15orf2 KLHDC2 SIX2 INA GCHFR PDCD6 NLRP6 TEX10 ALOX5 VAPA CBX3 FAM188A HPN TMEM194B MX1 NUP62 AKIRIN1 DDX19A GCH1 DPY19L2P2 NRXN1 MRPS23 MRPS15 AHCTF1 DPY19L2 LMNB2 PRKG2 GHRHR HN1 SMAD3 IL15RA BRAP NDUFB4 EI24 GAPDH SMAD1 ELF4 ZNF383 TNKS MRPL19 ZNF354C TOR1AIP2 TNRC18 UNC50 IPO13 C14orf49 RCC1 PSEN2 MYOF TRIM27 BCL2L1 TMEM97 SORT1 KCNH1 MRGPRF TMEM120A EMD FAM76B ADRA1B PCM1 PLCD4 TMEM48 TBC1D20 CDC14B POM121 NXNL1 CCDC79 NUP210 EPC1 GTF3C3 NUP93 HAX1 PTPLAD1 RANGAP1 TMEM201 SLC29A2 RANBP6 TMC6 RNF13 RAP1GAP2 P2RX1 EDNRB C15orf43 PRICKLE2 DHRS4 RB1CC1 DDX19B H2BFWT BCL2 TMPO C19orf46 PML NUP35 KPNB1 ALOX5AP LEMD3 GNAQ ATP11B NUP205 DUX4 PHF20 NUP85 ZC3HC1 CTDNEP1 XPO1 GLTPD1 ATP2A3 NAV3 ERBB2IP OSBPL3 TNPO3 PTCHD2 NR4A1 SLC30A1 TSPO NUP54 NOC4L TMEM109 P2RX5 NUP50 WTAP DGKH SLC16A3 GUCY2D NUTF2 SUMO1 SIGMAR1 CERS6 CERS5 PUM2 TXLNG NUDT1 P2RX3 ERN1 C2orf42 ANXA4 AKAP6 NUPL1 INTS2 SNCA PTGDS APEH MATR3 TMEM194A GRK5 PLCB1 TRA2B TM7SF2 CLIC1 P2RX4 FAM169A CDK4 SUN5 IPO5 PSEN1 LMNB1 BCL2L10 SIRT1 DMPK DPY19L4 DHCR7 INTS1 NUP98 NPIP HSD17B1 MRPS14 YBX1 DTX2 TMEM188 RETSAT TMEM176B NUP107 CST3 ADRA1A CMTM3 TNPO1 EVX1 LTC4S CERS3 NRM MYO6 DCTN5 OSBPL8 SCRN1 SYNE2 LPIN1 NELF CUEDC2 ALG14 MCM3AP AKD1 DTL RBM15 DNAJC1 EGFR P2RX6 REPIN1

GO_NUCLEAR_CYCLIN_DEPENDENT_PROTEIN_KINASE_HOLOENZYME_COMPLEX Cyclin-dependent protein kinase (CDK) complex found in the nucleus. RB1 CDK9 CCNT1 ERCC2 BCCIP SNW1 KDM5A CCNH HSPD1 HOXC10 TAF7 CDK12 CDK13 PUF60 CCNT2

GO_NUCLEOLAR_PART Any constituent part of a nucleolus, a small, dense body one or more of which are present in the nucleus of eukaryotic cells. It is rich in RNA and protein, is not bounded by a limiting membrane, and is not seen during mitosis. POLR2E POP4 NOP10 POP5 TAF1D DKC1 POLR2F BOP1 POLR1D SURF6 SIRT7 RRP9 CD3EAP POLR1A NHP2 UTP18 NOL6 PES1 RPP30 RPP21 POLR2L NHP2L1 CDKN2AIP IMP4 POLR1B TWISTNB WDR12 SUMO1 EIF3L HEATR1 RPP40 PWP2 MPHOSPH10 RRP7B CIRH1A POLR1E CLEC3B FBLL1 TAF1A FBL GAR1 WDR37 ZNRD1 POLR2K RPP38 WDR43 RRP7A IMP3 UTP15 TAF1C TBL3 UTP6 WDR3 POP7 NOL11 NOP58 POLR2H POLR1C TAF1B POP1 NOP56

GO_ENDOPLASMIC_RETICULUM_TUBULAR_NETWORK A subcompartment of the endoplasmic reticulum consisting of tubules having membranes with high curvature in cross-section. KPNB1 RAB18 STIM1 ARV1 RAB10 RAB3GAP1 ASPH PARP16 REEP5 ATL1 REEP1 KIAA1715 ATL3 TMEM110 ZFYVE27

GO_SYNAPSE The junction between a nerve fiber of one neuron and another neuron or muscle fiber or glial cell; the site of interneuronal communication. As the nerve fiber approaches the synapse it enlarges into a specialized structure, the presynaptic nerve ending, which contains mitochondria and synaptic vesicles. At the tip of the nerve ending is the presynaptic membrane; facing it, and separated from it by a minute cleft (the synaptic cleft) is a specialized area of membrane on the receiving cell, known as the postsynaptic membrane. In response to the arrival of nerve impulses, the presynaptic nerve ending secretes molecules of neurotransmitters into the synaptic cleft. These diffuse across the cleft and transmit the signal to the postsynaptic membrane. KCNA2 ARPC2 CLSTN2 SYT4 RPH3A GRIP1 GSK3A GRM8 EFNA2 LPHN1 GABRR2 RGS17 VAMP7 EFNB1 NOS1 NIPSNAP1 SYT12 MINK1 CANX MYH9 SOS1 OXT RAB3C GRASP C1orf70 ATP2A2 ARFGAP1 ANK1 PRRT1 CAMK2D KCNH1 MYO7A ATAD1 IFNGR1 SYNPR COL13A1 BCL2L1 VDAC1 SYT10 ZMYND19 SDK1 RASGRP2 GRM1 NUFIP1 CEP112 CNIH2 CHRNA6 SLC29A1 SVOP CTTN GRIN3A GLRA2 GRIN2B GRIK1 PCLO CDK16 MAP1B CAMK2N1 SYNE1 VAMP2 MPDZ GAD2 SLC6A3 GABRR1 DOC2B ERC1 RIMS3 PDE2A PKP4 LRRK2 LRP4 ARFGEF2 EGFLAM NRXN1 GABRA5 SPG11 DSCAM NPHP4 CDK5 TLN2 ANK2 STX4 KCNB1 ASCC1 GABRG2 SEPT3 TULP1 STX6 LAMP1 CPLX4 PJA2 GRIN2C ACCN1 NEURL SH2D5 CHRNG GABRA6 LRRC4C GRIK4 LRRTM2 PTK2B CHRNB1 DNM1 LPAR1 CDH2 MIB1 PDZD11 PTN AGRN ITGA5 GLRB CHRNA10 GRIA4 SNPH P2RY1 SHANK1 NDE1 SEPT11 MUSK STX2 PRKCG COPS4 SLC4A7 SLC8A3 PLD2 RUSC1 SLC9A6 SEMA4C SHANK3 ABL1 TANC1 HCN3 CDK5R1 DLGAP1 DLGAP3 APBA1 GRID1 APBB1 CCK UNC13B CADPS GRM5 PVRL3 SLC18A2 CASK DLG1 OTOF ARHGAP44 SNAP47 STX1A NPAS4 DNAJA3 MAF1 HTR3B FBXO2 SEZ6 PRSS12 DLG4 DACT1 NPFF KCNC4 PIP5K1C PRKAR1A GRM7 DNMBP SNTB1 GABRA3 STXBP5 ILK PTCH1 RAB7A SYT3 LRFN1 STX1B CPE LIN7B NLGN4X GABRQ C1QL1 ABCC8 NETO2 C9orf4 TRPV1 SNTB2 AP2M1 PIAS3 CLSTN1 PPFIA3 PDE4B ITGA3 SAMD4A ERC2 BAIAP3 EPHA4 NR1D1 ALS2 CNGB1 GSG1L CAV3 FGFR2 PRRT2 CADM2 ACCN2 RAB11B ASAP1 NTRK2 NDUFS7 IGSF9B DOK7 KLHL17 ATP1A2 SRC FCHSD2 NMNAT2 GRID2 SYPL1 GLRA4 FRMPD4 VAMP1 NELF OPRD1 VTI1A SHISA7 DMXL2 NANOGNB NPTN C20orf30 FLRT3 NLGN2 DDC PTPRN FAIM2 ZNRF2 CHRNB4 GABBR2 SH3KBP1 PDLIM5 GRIN2D TRAPPC4 LYN SUMO1 LZTS1 CHRNA4 P2RX3 GABRR3 LRFN2 RAPGEF2 CLCN3 KCNJ4 COMT SLC22A2 SYTL1 PPFIA1 AP3D1 ANK3 WASF1 LRRTM4 ABI1 PPP1CC GLRA1 FOSL1 GRIN3B CABP4 TMEM163 AXIN2 ACHE EEA1 PFN1 CHAT HOMER1 STRN LRP8 DPYSL2 LAMB2 PDZRN3 DGKI PICALM NRN1 SYNJ1 SSPN SEPT6 MPST LIN7C NMU NLGN1 OPRK1 GABRA2 DNM3 GIPC1 POSTN SLC40A1 SIPA1L1 SLC6A9 CALB1 SH3GL2 ADAM10 GRIN2A GPM6A OLFM1 CRTC1 MAGI2 CHRNA2 CHRNA3 PPP3CA CIB2 STX19 GOPC NAAA NRGN SYT1 CHRNA1 BZRAP1 RGS12 GHRH CPT1C GPER PTPRN2 SYP NLGN3 ARC RIMS2 CLN3 CHRM1 OPRM1 PPP1CA CHRM5 TMUB1 SLC5A7 CADPS2 RGS19 CHRNA5 SLC30A3 MDM2 FBXO45 KCNJ10 WDR7 MOB4 GRIA3 RABAC1 SYNGR2 SV2A CHRNB2 SYT6 SYT17 CABP1 DRP2 ProSAPiP1 SYPL2 USH1C RIMBP2 HCRT CCL2 NDEL1 ARRB2 UNC13A MAP1S SRI NRXN2 DBNL KCNAB2 HRH4 SCRIB ARF4 GABRG1 SYT8 CPEB1 GRIK5 ITGB1 GRIK3 SLC8A2 KCTD8 PRKACA FARP1 LRRTM1 RIMS1 SPG20 CNIH3 SLC6A17 ACTN2 CYFIP1 SNTA1 DRD2 DES ARR3 NTSR1 CBLN4 GRM2 ICA1 PALMD TRIM9 SYT9 CALB2 PARK7 WNT7A PCDH15 MAGEE1 MEF2C SNAP23 MTHFR DLG2 NCS1 ARHGAP32 GSK3B SNAP25 TIAM1 UNC13C GRIN1 BRSK1 NCK2 RAB3B PRR7 MYRIP VTI1B SV2B C4B F2R LRRTM3 FLRT2 LRRC7 GRIA1 RGS14 CHRM4 SNAP29 CHRM3 DNAJC6 DLG3 IL1RAPL1 MAPT SYNGR1 NSF COLQ GABRG3 NAPA SYTL4 HDAC4 OPHN1 FUS BAIAP2 GRID2IP DLGAP2 CBLN1 GRIA2 BAI3 SEMA4F SYTL5 UTRN LRRC4 APBB2 PFN2 ATP2B2 CDH8 SLC18A1 VWC2L SLC32A1 CACNA1C AXIN1 HOMER2 HAP1 GABRB2 FMR1 PVALB ZDHHC17 MTMR2 IL31RA SYT2 SYNPO PI4K2A GABRA1 GABRD DSCAML1 KCNJ2 DTNB CHRNE GAD1 DENND1A DNAJC5 PALM SYT7 SCAMP1 GLRA3 CDH15 SYT11 MYH10 ZNRF1 DMD IGSF9 TPRG1L SLC17A8 P2RX7 SYNGR3 PTEN DAB1 TOR1A TMEM57 SEPT5 CHRNA7 PPP1R9A SLC18A3 CALCA SNCAIP KCNK9 KCTD12 LRRC4B HTR3A DLGAP4 SRPX2 ZC4H2 CACNA1B SHISA9 GABBR1 CRYAB FZD3 PRKAR2B SYNDIG1 ENAH ATP6V0D1 LGI3 SERPINE2 DNM1L STON2 CACNA1A DTNA ITPR1 PPFIA2 MT3 UNC5C KCND2 DAG1 PRIMA1 VWC2 RGS20 ITGA8 SLC6A4 SCGN CHRNB3 APP SNAPIN GABRA4 COPS5 CTNND1 MECP2 CPEB2 GRM4 STX11 SYN3 CLSTN3 IQSEC3 GABRB3 GPR98 AKT1 HRH3 DOC2A WNT3A DISC1 SEPT2 SLC8A1 SYT15 SLC17A6 GPHN SDK2 SNCB SV2C CNKSR2 VDAC2 SYT5 MME PPP1R9B ARF1 ANKS1B EPS8 CPEB3 CPLX3 DDN LRP6 PICK1 NF1 KCNC2 CYFIP2 BAI1 D4S234E SHISA8 EGFR CBLN3 CACNG5 LAMA2 LGI1 AAK1 SRCIN1 RAB3GAP1 SIGMAR1 LRFN3 GABRE CTTNBP2 USP14 ELFN1 CAMK2A CHRND ADORA1 SNCA GHRL STXBP1 VAMP3 APBA2 PSD3 PLCB4 ATP1A3 IGF2BP1 MYL7 NETO1 P2RX4 PTPRO KCTD16 PLDN SYNC SPOCK1 OLFM3 SYN2 PCDH8 ITSN1 EPHA7 TBC1D24 DVL1 CNN3 RPS6KB1 CPLX1 PSEN1 COL4A5 CTBP2 CEL PPT1 TRIP4 KCNK1 CACNG8 NLGN4Y SLC2A8 NRCAM PARK2 ADCYAP1 CRIPT MAPK8IP2 GRM3 NRXN3 ZMYND8 ATP2B1 SHANK2 DRD4 RAB3A TH STRN4 RIMS4 KCNN2 RAB8A RAPSN FYN GRM6 HOMER3 HSPA8 RGS10 AMPH CPLX2 BCR CADM1 STX3 ITPKA C4A ARRB1 DTNBP1 SYTL3 GRIK2 POLG SARM1 ODZ2 SYT13 FXR1 HPCA SLC17A7 SRGAP2 CPEB4 ATP6V1G2 CNTNAP4 DNM2 CHRNA9 SHISA6 RAB5A SLC17A5 SHC4 MFF PENK GAP43 EEF2K PHACTR1 GABRB1 AKAP9 EMB SYNGR4 NRG1 OLFM2 SHARPIN P2RX1 CAD PPFIA4 PVRL1 AP1S1 SYN1 CNTN2 C19orf26 MAPK8IP1 GABRP HTR2B PACSIN1 BSN LIN7A ATCAY CHRM2 FCHSD1 KCNA1 SCAMP5

GO_CELL_JUNCTION A cellular component that forms a specialized region of connection between two or more cells or between a cell and the extracellular matrix. At a cell junction, anchoring proteins extend through the plasma membrane to link cytoskeletal proteins in one cell to cytoskeletal proteins in neighboring cells or to proteins in the extracellular matrix. DSCAML1 GABRD GABRA1 SYNPO NOX4 RPL15 BAIAP2L1 IL31RA DENND1A PDLIM1 CHRNE LCP2 HAP1 PANX3 PLCG1 ZDHHC17 FMR1 GABRB2 CD99L2 ITGB6 CLDN24 PLEKHG5 LRRC4 CLDN2 UTRN TMEM2 GRIA2 RPL13A RAP2B MIOS MYZAP RPLP2 EFHD2 GRID2IP EXOC3 PTPRR DLG3 CD2 EPCAM COLQ GABRG3 REXO2 AIF1L FLRT2 C4B FLNC CHRM3 CLDN22 AKAP12 RGS14 LRRC7 AMOT ITGA4 RPS14 TNC PRR7 BRSK1 DNAJB1 MARK2 TIAM1 SYT9 WAS CBLN4 ARHGAP32 CDH1 NCS1 RDX DLG2 SNAP23 CALB2 SSX2IP WASF2 S100A14 CTNND1 PALLD KIRREL OXTR BSG PNN SYN3 KIAA1462 APP CHRNB3 XRCC4 FERMT1 HYOU1 NCKAP1 PRIMA1 CDH5 RPS10 AMOTL2 MYADM VAPB PLIN3 PKP2 LIMS1 PDLIM2 TRIM25 FLNB PANX1 ATP6V0C NPHS2 UNC5C ANO7 CLDN3 ZYX GABBR1 SHISA9 GJC3 DOCK9 FRS2 PACSIN2 PDIA3 PTK7 DNM1L SYNDIG1 EEF1D TADA1 S100P EPB41L2 ANXA2 PPP1R9A PARD6A CHRNA7 HTR3A RAC2 LRRC4B RPL9 MTDH RPS3 KCTD12 RPS11 RPL10A DMD CCT8 ZNRF1 SYT11 TPM4 ADD1 GLRA3 BAIAP2L2 TOR1A RANBP1 SYNGR3 P2RX7 TPRG1L RUVBL1 SCAMP1 CLDN11 CLDN14 RPS15 SYT7 SNX1 HMGA1 COBLL1 DAB2 AFAP1 GJB3 ITSN1 OLFM3 CCDC85C TLK2 NPHP1 CNN3 PLAU GRB7 SMC5 VAMP3 PSD3 MRPL46 CLDN7 VAV1 PCDH12 DLL1 CHRND FAM129B CTNNA1 KCTD16 SLX4 CLIC1 TJAP1 P2RX4 NETO1 BASP1 CC2D1A ZC3HAV1 LAD1 EIF4G2 DUOX2 ARHGAP28 HEPACAM FHL1 YWHAB PODXL PERP FRMPD2 TGFBR1 PCDH9 CAMSAP3 RTN4 DLG5 RPLP0 LGI1 CACNG5 TSPAN4 SRCIN1 ATP1B1 NAMPT RPGRIP1L PECAM1 LMO7 P2RX6 ARF1 UBA1 XIRP1 RPS5 CYFIP2 TGFB1I1 KCNC2 SYNE2 CPLX3 RAP1B LDLRAD3 FMNL2 TRPC4 SDK2 PPIB GJD4 ENO1 EPB41L1 SYT5 TP73 NRAP RPL4 EIF3E NF2 LIMD1 YES1 SORBS3 TNS3 SCN5A GJB1 FBLIM1 RPL12 C19orf26 SYN1 SYNM PVRL1 CNN2 FYB CDC42BPA BSN LIN7A KRT18 HTR2B GTF2A2 PACSIN1 GRHL2 H1FX RSU1 PHACTR1 PDZD3 WTIP GOLGA2 EMB DNM2 NCR3 CNTNAP4 GAP43 PARVG PTPLAD1 MARCKS SHC4 MFF SLC17A5 ATXN2L NOV SARM1 GRIK2 ITGA6 ARHGAP21 CHMP2B SDC1 ESAM CLASP2 USP33 STX3 CAV2 FRMD4A CADM1 PAK6 FERMT3 PVR FZD2 BCR STARD10 LCP1 C4A CNN1 PRDX6 HEG1 RIMS4 NOTCH1 YWHAH AMPH HSPA8 HOMER3 RAPSN C2CD4B CORO1B PMP22 CXADR SHANK2 HIST1H3D MYO1E KCNK1 CCND3 ARFIP2 SEPT9 TRIOBP HCAR3 TMEM65 HIST1H3A FSCN1 NLGN4Y CACNG8 ZC3H15 FZD4 VAMP5 FLT1 RHOB TULP1 KCNB1 ANK2 TLN2 CLDN25 EFNA5 PKP4 SCARF2 GABRA5 EGFLAM DDX58 MAPK3 TJP3 ARFGEF2 GABRR1 VAPA PTPRM MPP7 ERC1 GRIN2B GRIN3A FBF1 SVOP MLLT4 VAMP2 MPDZ CAMK2N1 MAP1B GRIK1 COL13A1 PANX2 CAMK2D ACTR2 EMD OSBP POF1B PARVB UTP15 GRASP NOTCH3 SMPX PRRT1 CSPG4 RPL31 ARHGAP18 LPHN1 MYH9 HMCN2 MINK1 CAP1 EFNB2 RPS26 SYT4 BMPR2 ZFYVE21 PCDH1 DIAPH3 KCNA2 PPIA DDX3X ANKRD23 SMAD7 CLDN1 NLGN4X CAPG LIN7B PAK1 C9orf4 ACTC1 FAT1 MYH2 HIST1H3F GABRA3 DNMBP RAB10 CADM3 FES EIF4G1 HIST1H3I PLAUR SHROOM3 GCN1L1 F11R SPRY4 PIP5K1C SNX5 LPP DLG4 DACT1 HSPA1B PDLIM7 TBC1D2 PAICS AMTN ENG RPS19 HSPB1 OTOF DLG1 CASK KAZN CADPS UNC13B GRID1 UNC45A DLGAP1 WNK4 FZD1 RPL19 PARD6B DNAJA3 CD53 CLDN17 DCAF6 MMP14 HCK STX1A LSR SEMA4C SHANK3 PVRL4 CAT SH3GL1 KDR SLC8A3 TJP1 CD151 GPA33 DSG4 PRUNE CLASP1 ALDOA MICALL1 TENC1 SEPT11 APC MARVELD2 AHSA1 TIGIT CAPZA1 CGNL1 PRKCG PKM2 LRRTM2 PTPN6 PTK2B GRIA4 SPTBN1 STK24 ARF6 ITGA5 LAYN AGRN CDH2 SH2D5 NEURL ARHGEF2 KIFC3 PJA2 MRPS18B PRKCZ EPB41L3 ZAP70 C19orf21 CDK4 SKAP1 TMEM163 CDC42BPB HIST1H3H PFN1 CD226 ACHE PTPN1 AFAP1L1 PPFIA1 CLTC SPTBN2 SMAGP ITGB4 ARPC5 GLRA1 GIT1 FASN FLII ABI1 CGN VCL LRRTM4 CLDN16 DHX29 PVRL2 MICALL2 CD81 RPL23A PTPRN MKL2 NLGN2 RND3 PAF1 MAGI3 CHRNB4 OPALIN ZNRF2 ITGB1BP1 NELF C1orf172 C20orf30 TMOD3 PGM5 DMXL2 CDC42EP4 ATP1A2 GSN KLHL17 IGSF9B CLDN12 DOK7 OLA1 CLDN6 PCMT1 BZW1 GRID2 RPLP1 EHD4 EIF2A ACTG1 KRT8 S100A7 IDH1 XIRP2 EEF2 KIAA1598 LRRC59 CAV3 DSC3 PPAP2B PRKCH C11orf73 EVPL PCBP2 STXBP6 MSN TRPV1 CCND1 FGFR3 SDC4 CDHR2 SAMD4A CDH13 ERC2 FAT2 RALA HSPA9 SERBP1 OCLN SLC6A17 ACTN2 CNIH3 PROCR RIMS1 SWAP70 GIGYF2 PDPK1 GABRG1 ITGB1 PLEC CPEB1 STK38 ProSAPiP1 CABP1 DRP2 IRF2 PRX ARHGEF16 CHRNB2 DSG2 SND1 ILDR1 KIAA1524 KCNAB2 DBNL CD200 FABP7 MAP1S UNC13A YKT6 HCRT GTF2F1 MIP DSG3 MPRIP CEP68 SLC30A3 GPRC5A TEK LIM2 RABAC1 GRIA3 STX5 ATP1A1 SLC4A2 TBC1D10A PDXDC1 ARC ABCB4 PTPRN2 ARHGAP22 TMUB1 CADPS2 GAK PPP1CA HSPG2 CHRM1 PFKP GOPC FERMT2 CLDN4 CIB2 SEPT7 ITGB5 CHRNA2 RPS2 CPT1C GPER FLOT1 MACF1 RAP2C USO1 ERBB2IP GIPC1 CAPN5 PSMB6 ASH1L DSTYK MAGI2 GRIN2A DST GJB6 TGM2 HSP90B1 RPS4X STRN HOMER1 GJA3 GDI2 MPST EHD3 NRN1 PICALM RHO LPHN3 PI4K2A SYT2 NRP1 RPL24 HNRNPK ADCYAP1R1 ARHGEF5 KCNJ2 FGFR4 GIT2 SCYL1 ACTB HOMER2 VWC2L HAVCR2 JAK1 ATIC TNK2 NCK1 NFIA GJA8 ABP1 CSRP2 ARHGAP24 ATP2B2 HPN EPB41L5 DIXDC1 TFB2M OPHN1 FRMD6 EZR C1QTNF5 PAK4 MARVELD3 CBLN1 DLGAP2 P4HB GJB7 CBL MCRS1 RPL5 BAIAP2 ESYT2 SCN1A LPXN PTPRJ EPS8L1 DDR2 IST1 YWHAE EPS15L1 PKN2 RANGRF SYNGR1 CLDN10 FHL3 GJD3 LRRTM3 PAK2 USP8 PPME1 SV2B PPP1R12A SCN4B B4GALT1 WNK3 RPS17 CALR CHRM4 GRIA1 VANGL2 SNAP25 MPP5 CTNNA2 BAG3 GRIN1 RPS3A UNC13C CD46 TRIM9 CRKL CSDA RPL38 ICA1 GRM2 DCAF13 L1CAM CASS4 BCAR1 PARK7 COL17A1 OBSL1 GABRB3 SLC2A1 HSPA5 VEZT PARD6G ITGA8 SPECC1L RPS7 CAPN1 COPS5 FLNA GABRA4 EPS15 ANXA5 SNAPIN FZD5 KIF5B DAG1 KIAA1797 CDC42EP1 KCND2 RPL23 VWC2 LOC100288814 CAST CLCA2 FBLN7 FLRT1 ALCAM PARD3B SPTAN1 DTNA LASP1 SENP1 RPL27 CSNK1D UBAP2 NRIP1 FHL2 ZC4H2 SRPX2 STON2 CLIC4 AMOTL1 LGI3 MCAM ENAH ARPC5L CD59 RHOA C10orf54 HIST1H3C SIRT2 PDZD2 SLC9A3R2 LARP1 NMT1 CSRP1 BCAS2 PEAK1 FLCN SLC17A8 IGSF9 ITGB3 FMN1 NUDC YWHAG JAG1 KRIT1 DSP PCDH8 SYN2 TWF1 AKR1B1 DAB2IP CHMP4B MAPK1 PSEN1 TMEM204 RPS6KB1 AKAP6 EIF4H RPL29 SNCA ATP6V0A2 CAMK2A ABCA7 LIMK1 GRK5 SYNPO2 RPL6 GABRE LRFN3 NFASC SIGMAR1 GJC1 RPS9 RHOG SRP68 ICAM1 LYPLA2 GJA10 ARHGEF18 ADAM17 C1orf86 BAI1 DSG1 MYO1B TBCD CBLN3 LAMTOR3 PARD3 EGFR FNBP1L CD3E TWF2 HMCN1 EPS8 ANKS1B PPP1R9B TRPC6 MME KCNA5 ITGA2 THEMIS PTPRK VASN MYO6 PICK1 RPL7A ABCF3 SV2C SLC17A6 GPHN PCDHGA12 EIF2S3 SHROOM2 EPHA2 AQP3 PABPC1 DOC2A AKT1 STAT1 GNA12 ITK SDCCAG8 ITGA2B SEPT2 SLC8A1 PLEKHA7 DISC1 RPL18 PKP3 PARVA MAPRE1 EPN2 SCAMP5 KCNA1 CHRM2 ATCAY GABRP SH3PXD2B HSPA1A MAP2K1 GABRB1 RPL22 CEACAM1 SHARPIN GJC2 OLFM2 NEXN MDC1 GJA1 GJA9 CCNB2 RAC1 ACTR3 CHRNA9 RANGAP1 CFL1 CTNND2 ODZ2 KIF22 HIST1H3J DTNBP1 SRGAP2 CD99 SLC17A7 TMEM47 NPHS1 ASAP3 CKAP5 RAN CDH24 ADD3 HIST1H3E TXNDC9 PIP5K1A IMPDH1 SCARB2 PPP1R13L RPS16 GJD2 ANXA1 VASP G3BP1 ABCB11 PTPN12 PLXNA3 HIST1H3G HCAR2 CORO1A ARPC1B HOXC5 GRB2 EPS8L2 GNB2L1 HAMP HIST1H3B MAP2K2 KTN1 ANLN RAB1A PIK3R1 ATAT1 PLXDC1 CTBP2 DPP4 SGCA CRIPT HSP90AB1 CDK5 NPHP4 CD44 C8orf37 CPLX4 SEPT3 GABRG2 PTPRU PCBP1 ARGLU1 LRP1 LRRK2 MRE11A CADM4 DSCAM TRAF4 PRKCD NRXN1 RPL3 EIF5 ATN1 CLDN20 SORBS2 ARHGAP1 GAD2 CDK5RAP2 S100A11 TRPV4 CYBA CORO1C DSC2 CXCR4 GJA5 RFWD2 RIMS3 MRC2 LIG4 ZNF185 GLRA2 CTTN TES CAPN2 GJB4 YWHAQ CLDN15 CDK16 PCLO BCL2L1 SYNPR RND1 RPL34 ATAD1 AATF CCS CHRNA6 CNIH2 PUF60 RASGRP2 EVL SLK SDK1 ADAM9 AMICA1 C1orf70 IQGAP3 DOCK7 CALD1 EHD1 VAMP7 RGS17 CLDN23 CDSN GABRR2 CLINT1 PI4KA SYT12 RPH3A ARPC2 NHS C12orf53 SLC2A2 GRIP1 ADA RPS18 TSPAN9 CSK NOP56 GABRQ FBP2 LRRFIP1 SIPA1L3 ACTN3 ILK STXBP5 SH3PXD2A SLC5A1 HCFC1 CHP CLDN18 SNTB1 DBN1 NEDD9 JAM3 LRFN1 GJB5 RAB13 PKP1 SH3GLB1 CLDN5 ARHGAP31 LAP3 CLDN8 PLCB3 MAGI1 PRKD1 STEAP1 PVRL3 UBN1 DLGAP3 UBFD1 LDHA SCN1B CAPZB VIM ARHGAP44 RUSC1 KRAS C20orf11 PIKFYVE RPL30 TANC1 RSC1A1 CD2AP SHANK1 MYH1 NUMB ECT2 THY1 DLC1 COPS4 CEP41 INADL PTPRC MUSK RHOU CHRNB1 DCP2 C4orf19 TAGLN2 FAP LMLN SNPH JAM2 CHRNA10 SH3GLB2 GLRB MPZL1 MB21D2 PDZD11 TNKS1BP1 CTNNA3 PDGFRB GRIN2C SVIL DDX6 CDH3 TLE2 LRRC4C GRIK4 GABRA6 NDRG1 SDCBP CHMP5 HDLBP CHRNG RPS13 CDC42 NCSTN CPNE3 B2M GAPVD1 JUP GRIN3B KIT PPFIBP1 PHLDB2 CAV1 SORBS1 TLN1 FOXA2 IGSF5 ANK3 WASF1 YWHAZ LIMS2 SFN PPP1CC CHRNA4 AJAP1 ARPC3 LZTS1 SNX2 RAP1A KCNJ4 RAPGEF2 LRFN2 ITGAV GABRR3 RPL8 FLOT2 FLRT3 PRKAR2A TJP2 PDCD6IP LYN NEU1 ARHGAP26 SH3KBP1 PDLIM5 SNX9 GRIN2D GABBR2 EPB41L4B DDB2 SPTBN4 FAIM2 ANXA6 VAMP1 TCHP IGF2R NUP214 GLRA4 RRAS2 CLDN9 BVES RARS ASAP1 RAB11B TNS1 ACTN1 TRIP6 PKD2 BZW2 APBB1IP SRC GNA13 CRTAM ARHGAP17 RAB21 FGFRL1 SLC9A1 EPHA4 CADM2 PRRT2 GSG1L ARHGEF7 ADAM15 KLC2 ITGA3 CNKSR1 CLSTN1 CRB3 CLMP SNTB2 CLDN19 PRKCI GOLGA3 SLC3A2 PTK2 FARP1 RPS29 WDR1 KCTD8 MLC1 GPR142 DES CYFIP1 SNTA1 CTNNB1 SLC2A11 GNB2 GLOD4 NME2 LRRTM1 KIF23 KIRREL2 RPL37A SCRIB GRIK3 CD96 ITGA1 GRIK5 STX16 SYT6 RIMBP2 EEF1G FGF13 IFI30 EPB41 RPL7 PPL CHRNA5 SYMPK STARD8 RSL1D1 PXN LIMA1 SV2A BIN2 FBXO45 TRIM29 FER RIMS2 RPL14 NLGN3 SYP KCNJ11 GJB2 CHRM5 CD9 OPRM1 ACTN4 SYT1 RRAS JUB TNS4 CHRNA3 PRDX1 CDCA3 AQP7 ARFIP1 RGS12 CHRNA1 PNMA1 SIPA1L1 PPP1CB GABRA2 RPS8 NLGN1 IQGAP1 DSC1 OLFM1 LAT ADAM10 RUFY3 NOX1 TMPO CD97 ITGA11 NPM1 LIN7C SSPN AHNAK AHI1 GJA4

GO_CYTOSOLIC_PROTEASOME_COMPLEX A proteasome complex found in the cytosol of a cell. PSMC6 UBQLN4 PSMD2 PSMD1 PSMC2 UCHL5 PSMC4 PSMC5 PSMC3 PSMC1 PSMD14 IDE

GO_CONDENSED_NUCLEAR_CHROMOSOME A highly compacted molecule of DNA and associated proteins resulting in a cytologically distinct nuclear chromosome. LOC728637 CHEK1 CCNB1IP1 STAG3 AURKC CCDC155 RAD1 MIS12 BLM SUV39H1 HSPA2 DMC1 AURKB RAD9B SMC1A DSN1 RSPH1 RRS1 SYCE2 PMS1 CENPC1 CHMP1A SUV420H2 ADD3 HUS1 H2AFX RAD21 SGOL1 MKI67IP BRCA2 SMC1B BRD4 HORMAD1 SUV420H1 UBE2I AURKA RGS12 RAD50 EIF2C3 RCC1 SYCE1L RAD21L1 SYCP1 LRPPRC BUB1B TEX12 BUB1 SYCP3 REC8 SS18L1 PMS2P3 FKBP6 MSH5 RAD51 CCNB1 SUN2 CDX2 NOL6 TTN WAPAL NDC80 RAD51C BRCA1 INCENP TUBG1 HORMAD2 MLH3 SMC3 SYN1 LEPREL4 KIFAP3 SYCP2 MIS18BP1 SYCE1 MLH1 CENPA NCAPD3 RNF212 TEX11 NEK2 TOPBP1 RAD9A SYCE3 PLK1 HUS1B NCAPD2

GO_CELL_CORTEX_REGION The complete extent of cell cortex that underlies some some region of the plasma membrane PCLO PRKCZ MYO5B PKD2 CLASP1 CLASP2 PHLDB2 BSN PHLDB1 FABP1 GM2A HAMP NLRP5 TCHP

GO_CULLIN_RING_UBIQUITIN_LIGASE_COMPLEX Any ubiquitin ligase complex in which the catalytic core consists of a member of the cullin family and a RING domain protein; the core is associated with one or more additional proteins that confer substrate specificity. KCTD10 BTBD2 FBXO25 KBTBD3 FBXO7 FZR1 ENC1 CUL4B KLHL33 KLHL20 FBXW5 CUL3 KLHL4 KLHL18 CCNF KLHL10 DCAF6 DCAF10 KLHDC7B SPOP FBXO2 KLHL24 KBTBD10 KLHL28 BTBD1 CUL4A DCAF8 UBE2S SPOPL FBXW8 KLHL22 FBXL15 TNFAIP1 KLHDC5 CDC23 BTBD6 CUL5 TRIM21 ANAPC13 ZER1 KBTBD8 RBX1 FBXO9 SKP1 CDC16 KBTBD4 FBXW7 ANAPC7 KBTBD7 BTBD18 GAN ZYG11B BTBD3 KLHL3 KLHL2 KLHL32 CDC26 ANAPC11 PARK2 PLRG1 IPP KBTBD6 ERCC8 FBXL3 PHOSPHO2-KLHL23 KLHL7 ANAPC5 ABTB2 KLHL36 KLHL26 CUL9 KCTD17 KLHL29 KLHDC8A KLHDC7A FBXO44 RNF7 VHLL CCIN CAND1 KLHL38 USP47 ASB2 FBXO39 ANAPC10 DDB1 DCAF5 KLHL1 ANAPC16 KLHL34 ANAPC4 DDB2 DCAF17 DCAF12 CDC27 CKS1B TRPC4AP KLHL23 COMMD1 ANAPC2 BTRC KBTBD2 FBXL19 CKS2 CUL1 CDC20 CDKN1B CRBN BACH2 DCAF7 FBXL21 KLHL8 FBXL7 BUB1B C10orf46 DCAF16 KCTD2 KLHL9 KLHL21 CUL2 FBXL2 ABTB1 KLHL12 SKP2 KLHL17 FBXO27 KLHL15 KLHL30 KBTBD11 DTL FBXO4 DCAF4 FBXO32 DCAF11 KLHL11 KCTD5 KLHL5 BACH1 FBXL22 FBXW11 TCEB2 KLHL25 GLMN ASB4 DCAF13 C11orf51 FBXO6 KLHL31 KBTBD5 KLHL13 KLHL14 KEAP1 FBXO18 FBXL5 BTBD11 FBXO31 UBE2C ZYG11A CUL7 ANAPC1 KBTBD13 KCTD13 KBTBD12 MAD2L2 FBXO17 BTBD9

GO_OTHER_ORGANISM A secondary organism with which the first organism is interacting. TAP2 TMEM229A PI4K2A AXL SCO1 DYNLT1 C4BPB C4BPA IFIT1 LMAN1 DERL1 TAP1 PI4KA C4B RAB7L1 FMR1 BICD1 AQP1

GO_PML_BODY A class of nuclear body; they react against SP100 auto-antibodies (PML, promyelocytic leukemia); cells typically contain 10-30 PML bodies per nucleus; alterations in the localization of PML bodies occurs after viral infection. DAXX TOPBP1 SMC5 CIITA RPA2 MRE11A SKI ARNTL RNF4 EIF4ENIF1 PIAS2 RPAIN WDFY3 MTOR CASP8AP2 NBN RNF6 TDG HIPK2 SUMO2 KLHL20 HIRA ODZ2 MAX TRIM16 TP53 UBN1 NFE2 SENP2 TERT ZNF451 SPTBN4 MAGEA2B CDK9 MORC3 ZMYM2 PIAS4 MAGEA2 SUMO1 ZBTB16 PIAS1 SP100 RAD51 TDP2 NR2C1 MLIP ATR USP7 SP3 TRIM8 ATRX UBE2I ANKRD2 SMC6 TP53INP2 RGS14 CBX5 TRIM27 TP53INP1 NSMCE2 SP140 PATL1 KAT6A MKNK2 HIPK3 DAPK3 N4BP1 LRCH4 TOPORS THAP1 SATB1 SUMO3 RB1 EIF3E PPARGC1A SIRT1 PML TCF7L2 MAPK7 ELF4 IKBKE SQSTM1 PARK7 ISG20 TOP3A RDM1 RPA1 RFWD3 SRSF2 CHEK2 HIPK1 C1orf51 PTEN CHFR BLM AKAP8L

GO_CATALYTIC_STEP_2_SPLICEOSOME A spliceosomal complex that contains three snRNPs, including U5, bound to a splicing intermediate in which the first catalytic cleavage of the 5' splice site has occurred. The precise subunit composition differs significantly from that of the catalytic step 1, or activated, spliceosome, and includes many proteins in addition to those found in the associated snRNPs. PHF5A SF3A2 KIAA1875 CDC5L SNRPE SF3B14 WDR83 HNRNPC SKIV2L2 SNW1 GPATCH1 HNRNPA1 DDX23 HNRNPH1 XAB2 PRPF19 SF3B3 DHX35 HNRNPA3 SYNCRIP EIF4A3 AQR SNRPB HNRNPK CWC22 SNRPD3 PNN SNRNP40 PRPF8 PPIL1 DDX5 PPIL3 SNRPD1 PRPF6 DGCR14 CRNKL1 SYF2 SRRM2 PPIE SNRPN RALY RBM8A SNRPB2 THOC4 ISY1 BCAS2 FRG1 PRPF4B HNRNPU TFIP11 DHX8 SNRPD2 ZCCHC8 HNRNPA2B1 HNRNPR CWC27 DHX38 EFTUD2 PLRG1 SF3A3 LSM7 SNRPA1 HNRNPF PABPC1 MAGOHB HNRNPM LSM2 RBM22 SF3B1 CDC40 SF3B2 SRSF1 RBMX SNRPF SART1 SLU7 RBMX2 SNRNP200 SF3A1 LSM3 DDX41 MAGOH C19orf29 SNRPG CWC15 PPWD1 C14orf102 SRRM1

GO_U12_TYPE_SPLICEOSOMAL_COMPLEX Any spliceosomal complex that forms during the splicing of a messenger RNA primary transcript to excise an intron; the series of U12-type spliceosomal complexes is involved in the splicing of the majority of introns that contain atypical AT-AC terminal dinucleotides, as well as other non-canonical introns. The entire splice site signal, not just the terminal dinucleotides, is involved in determining which spliceosome utilizes the site. SNRNP48 SNRPD2 SNRNP35 ZRSR2 SF3B14 YBX1 SNRPF SNRPD1 SF3B4 SF3B2 SNRPE SF3B1 PHF5A SNRPB SNRPD3 RBM41 SNRPG PDCD7 SNRNP25 LSM7 SF3B3 SF3B5 ZMAT5 RNPC3 ZCRB1 DHX15

GO_CHROMATIN The ordered and organized complex of DNA, protein, and sometimes RNA, that forms the chromosome. BRMS1L HIST1H4E MYCN HIST1H3J CENPF LRWD1 KIF22 H2BFS SIN3B STAG1 HAND2 HMGN3 TTC37 HNRNPC OIP5 SALL4 USP3 RNF2 CITED2 CBX8 RING1 H1FX ENC1 RRP1B RPA2 HIST2H3A MCMBP H1FNT HIST1H1A SMARCC1 HIST2H3D POGZ PLK2 MTA2 PRM2 HIST1H1C RARA SUV39H2 HIST1H4K CDCA5 NCOR1 SALL1 SRF CTNNB1 TOP2B HIST1H2BB APTX H2BFWT ANP32E HIST4H4 TMPO SATB1 THRB HMGN1 MTBP PADI2 YY1 HIST1H3A NACC2 HIST1H2AK POLR3D RXRA HIST1H2AG POLR3GL TAL1 FOXC1 KDM1A STAT6 CHEK1 HIST1H3B HIST1H2AL NEDD4 CTR9 HIST1H2BM E2F4 HMGA2 HIST1H3D NR1H4 HDAC2 RARG HIST1H4C MCM2 KDM3A EP400 HIST1H3G ACTR8 GABPA NCOR2 CXorf27 SPI1 RAD50 PAWR RAN NFATC1 TCP1 ARID1A H2AFX CHAF1A HIST1H3E FER SETD1A ARRB1 ELL MUC1 NCOA1 HMGN4 NARG2 POU4F1 WAPAL NASP SNAI2 MSH6 EXOSC10 UBE2A SIRT7 HAT1 TIMELESS KDM4A HIST1H2BK ZNHIT1 UBE2B KAT5 ETV3 INCENP HIST1H2BF HIST2H3C SUMO1 SNW1 TNP2 FOXD3 EXOSC3 HDAC1 SMARCE1 EXOSC4 WDR61 BAHCC1 PELP1 PLK1 PPP1R10 PLCB1 MBD1 LDB1 INO80B SLX4 HMGB2 TP63 TRRAP H2AFB1 STAT3 H3F3B CDK4 HLCS TCF3 KIAA0947 HIST1H3H MEF2A HELLS ESCO2 TP73 HIST1H2BJ TCF7 EIF3E SCRT2 SIRT1 SOX18 STAT1 IRF1 SUV420H2 PAX6 HIST1H2BN SIN3A BAHD1 CBX7 WBP2 ZNF385A HIST2H2BF TRPS1 HIST2H2AA4 DNMT3A NR1D1 HSF1 EXOSC5 PHC2 SMAD4 KLHDC3 SUV39H1 GATA3 FBXO18 HIST3H2A TIPIN CHD5 PHF12 H2AFV FAM111A AKAP8L IRF4 ACTL6A MIXL1 TARDBP MBD4 DNTT ANKRD2 H1F0 H2AFB3 UBA1 HIST1H2BD UBE2U TOX4 T CHD4 H2AFY2 NELF PHOX2A H3F3A SUDS3 SFPQ DNMT1 SMARCA2 UHRF2 EHMT2 MIS18A RRP8 ASF1B NFRKB RFX3 H1FOO TP53 CBX6 KLF1 JUND HIST1H2BC SMARCAD1 HIST2H2AA3 AR HIST2H2AC ANKRD17 HIST1H2BE HIST1H1E MPHOSPH8 ASXL1 SAP130 EME1 RUNX2 CCND2 ZFP57 H2BFM CREBBP HIST1H4F HIST1H3F SETD3 SMARCA4 HIST1H4B INO80E PHOX2B BCAS3 HIST1H3I NCOA3 TNP1 BAZ1B C12orf48 TCF12 HTRA2 MECP2 PDS5A PPARD CENPA NCAPD3 TRIM28 BUD31 CSNK2A1 HIST1H2AH HIST1H2BH RUVBL1 HIST1H4D TNKS1BP1 HIST2H2BE DDX6 UBR2 CALCOCO1 HIC1 UPF1 UCHL5 CENPC1 HIST1H4I HMGA1 HIST1H2AD IPO4 HIST1H4H PRM3 PSIP1 ACTR5 CBX1 HIST1H1B POLR3G TBP BRD8 HIST1H1D CHAF1B UHRF1 HIST1H4L SMARCD2 H2AFJ NPM2 SMARCB1 CEBPB MAGED1 RUVBL2 DPF2 H2AFZ HIST1H2BI HIST1H2AA HIST1H2BA SIRT2 HIST1H3C ANKRD32 RBBP4 TRNP1 CBX5 DBF4B NRIP1 RNF20 RAD21 RAD51AP1 DFFB HIST1H2BO PRM1 CDKN2A RUNX3 TFPT SHPRH STAG2 MBD3 GATAD2B JUN MED1 DFFA ESR1 MEN1 CBX2 WDR82 HIST1H2BL HIST1H4G CAPN2 IKZF1 MORF4L1 ASH2L DVL3 H2AFY HIST1H2AE EZH2 MCRS1 HIST1H2AI NFE2L2 H3F3C ORC2 DSCC1 HMGN2 POLA1 HIST1H4J HIST2H4A ING2 TRIM24 RAD51 CBX3 SFR1 DLX5 EED ESCO1 HIST2H2AB ACTB SUZ12 ASF1A SIRT6 SWI5 HIST3H3 MCM7 HMGN5 MYOD1 AHCTF1 BEND3 KDM3B DDX11 FAM60A ALKBH1 SP1 PINK1 NUCKS1 SMC3 JMJD1C SMAD3 HILS1 HNRNPK HIST1H1T MAEL SMAD2 AFF4 HIST2H4B HIRA FOXH1 MAF L3MBTL1 ING3 KDM4C RB1 PPARGC1A TTC21B HIST1H2AB RELA MBD2 HIST2H2BD NR1H3 HIST3H2BB BAZ2A HIST1H2AC TCF7L2 MXD1 PARK7 CREB1 HIST1H4A PCGF2 INO80 TCF4 KLF4 PDS5B KIAA1967 INO80C ZC3H8 HR HEY2 MAU2 BRMS1 SMARCD3 TNRC18 HIST1H2BG ATRX HIST1H2AM RCC1 HP1BP3 H2AFB2 NIPBL RBMX RNF40 KAT6A JUNB NUFIP1 KAT2A E2F1 KAT6B EME2 SMARCC2 FAM178A

GO_CILIARY_PLASM All of the contents of a cilium, excluding the plasma membrane surrounding the cilium. ATG5 RP1L1 C1orf96 CCDC151 DNAH1 BBS7 CCDC164 CCDC39 DNAI1 TRAF3IP1 WDR35 GAS8 KIAA1984 DNALI1 DNAH5 CCDC37 DYNC2LI1 SEPT9 DNAH3 SPATA7 IFT57 SEPT2 IFT140 TTLL8 SPAG17 IFT172 CCDC42B MAPT FAM154A RPGRIP1L MAP1LC3B C12orf55 LRRC48 DNAH9 C10orf92 PCDP1 INPP5E WDPCP AMBRA1 DNAH6 C6orf165 DNAAF1 ARL13B KIF19 DNAH7 SEPT7 ARL6 GLI3 GLI2 ATG16L1 RP1 CCDC40 TULP3 PIK3R4 KIAA1009 ATG14 CCDC114 EFHC1 TCTEX1D4 AK8 DNAJB13 CCDC63 DNAH17 RSPH4A WDR34 DNAH8 DNAI2 BBS1 RSPH9 CENPF TTLL3 MAP4 SPEF1 SPAG6 RPGRIP1 KIFAP3 DYNC2H1 ATG7 MNS1 DCDC2 TXNDC3 MAK TMEM141 CCDC103 KIAA1751 PIK3C3 HYDIN ARMC4 SPAG16 GLI1 DNAH2 GNAT3 KIF17 ARFGEF2

GO_SWI_SNF_COMPLEX A SWI/SNF-type complex that contains nine or more proteins, including both conserved (core) and nonconserved components; the Swi2/Snf2 ATPase is one of the core components. ARID1A SMARCD2 ACTL6B SMARCB1 SMARCA4 RB1 SMARCD1 SMARCA2 SMARCC2 ACTL6A SMARCD3 NCR1 SMARCE1 ARID1B SMARCC1

GO_ESCRT_COMPLEX An endosomal sorting complex required for transport. TSG101 FAM125A VPS25 CHMP6 STAM2 CHMP4B CHMP4C STAM UBAP1 CHMP4A VPS36 CHMP7 UBAP1L VPS37B CHMP3 VPS37C CHMP2A HGS VPS28 VPS4A CHMP1A VPS37D FAM125B CHMP1B CHMP2B SNF8 VPS37A

GO_COHESIN_COMPLEX A protein complex that is required for sister chromatid cohesion in eukaryotes. The cohesin complex forms a molecular ring complex, and is composed of structural maintenance of chromosomes (SMC) and kleisin proteins. For example, in yeast, the complex is composed of the SMC proteins Smc1p and Smc3p, and the kleisin protein Scc1p. In vertebrates, the complex is composed of the SMC1 (SMC1A or SMC1B) and SMC3 heterodimer attached via their hinge domains to a kleisin (RAD21, REC8 or RAD21L) which links them, and one STAG protein (STAG1, STAG2 or STAG3). RAD21 SMC1A WAPAL DDX11 REC8 CDCA5 RAD21L1 SMC3 STAG3 SGOL2 SMC1B

GO_UBIQUITIN_LIGASE_COMPLEX A protein complex that includes a ubiquitin-protein ligase and enables ubiquitin protein ligase activity. The complex also contains other proteins that may confer substrate specificity on the complex. MED31 BTBD16 C10orf120 PARK2 ERCC8 SYVN1 KLHL29 KLHDC8A ASB15 KLHL26 AMFR CUL9 NEDD4 ARIH1 MED17 SKP1 MED10 FBXW7 GAN KBTBD8 STUB1 HSPA8 KLHL32 ANKIB1 RNF31 USP33 ANAPC11 BRE FBXO10 BTBD3 KLHL2 SEL1L KLHL28 CUL4A CCNF UBR1 DCAF10 KLHDC7B SPOP KBTBD10 CUL5 RNF216 TSPAN17 ZER1 SPOPL UBE2S RNF2 RAD18 ASB14 KLHL22 DNAJA1 TNFAIP1 FBXO11 KLHDC5 WWP2 MIB2 ENC1 RING1 CBX8 HSPA1A RNF222 DCUN1D1 SHARPIN KBTBD3 KLHL4 MED7 DCUN1D4 FBXW5 UBE2L3 BARD1 UBE2D1 C11orf51 TOPORS CBX7 ASB4 ANAPC1 KBTBD13 KCTD13 KBTBD12 MAD2L2 BTBD9 FBXO17 KLHL13 FBXL4 KBTBD5 FBXO21 KEAP1 FBXO18 BTBD11 PHC2 UBE2C CUL7 ABTB1 CBX4 RNF217 KLHL12 SKP2 KLHL17 ASB16 KLHL21 UBE2U UBE4B CUL2 KBTBD11 DTL FBXO4 DCAF11 KLHL15 ASB10 LMO7 COMMD1 UBE2A ANAPC2 ASB3 KBTBD2 TRAF2 RNF168 DDB2 UBE2B DCAF12 VHL FAM105B CDC27 CKS1B KLHL8 BUB1B FBXL7 DCAF16 C10orf46 KCTD2 CDC20 CUL1 GMCL1 CRBN RNF8 DCUN1D2 DCAF7 BACH2 RNF19B KLHL38 ASB2 FBXO44 RNF7 CAND1 UBE4A ANAPC16 KLHL34 GPR37 DCUN1D3 TCEB1 KLHL7 UBR2 ANAPC5 ABTB2 ATG3 KLHL36 IPP PLRG1 KBTBD6 MED24 PHOSPHO2-KLHL23 FBXL3 KCTD17 BMI1 KLHDC7A MED18 TRAF7 MED20 MED27 CDC16 FBXO24 KBTBD4 ANAPC7 KBTBD7 BTBD18 PHC3 FBXL12 RBX1 FBXO9 CDC26 RNF20 ZYG11B KLHL3 GMCL1P1 BTBD1 DCAF8 KLHL10 FBXW4 RCHY1 DCAF6 FBXO2 KLHL24 TRIM21 MARCH6 HSPA1B ANAPC13 FBXW8 FBXL15 CDC23 MPHOSPH8 BTBD6 IKBKG FBXO7 FZR1 SUGT1 KLHL33 CUL4B RBCK1 KCTD10 RNF4 OS9 BTBD2 UBE2N FBXO25 CBLL1 KLHL18 RNF144B KLHL20 CUL3 DCAF13 SMURF2 FBXO6 KLHL31 KLHL5 BACH1 TCEB2 FBXL22 FBXW11 GLMN KLHL25 PCGF2 UBE2E1 MED12 KLHL14 MED11 RNF11 FBXL5 FBXO31 ZYG11A RNF19A FBXO15 UBE2V1 FBXO27 KLHL9 FBXL2 AUP1 RNF40 FBXO32 DCAF4 MED21 KLHL11 WWP1 PHC1 KCTD5 UBE2D2 KLHL30 MED30 UBR3 KLHL23 MED1 BTRC FBXL19 CKS2 BRCC3 BRCA1 PCGF6 DCAF17 CBX2 TRPC4AP FBXL21 UBE2J2 RNF14 ARIH2 CDKN1B USP47 DCUN1D5 VHLL CCIN FBXO8 KLHL1 ASB18 PINK1 RNF144A ANAPC4 FBXO39 ANAPC10 BRAP DYRK2 DCAF5 DDB1

GO_AGGRESOME An inclusion body formed by dynein-dependent retrograde transport of an aggregated protein on microtubules. EPS15 SFMBT2 SLFN11 SYNE2 GIT1 UBQLN1 HDAC6 EVX1 RAB11B TRIM66 RNF32 PSEN1 XRN2 SEC62 MECOM DBF4B HOXD3 POLD1 CABIN1 CDH1 HSPB7 URB2 SQSTM1 PARK2 UBD ORC6 UCMA TRIM37 C7orf28B FAM125A EEF2 CCZ1 EID1

GO_NUCLEAR_TRANSCRIPTIONAL_REPRESSOR_COMPLEX A protein complex, located in the nucleus, that possesses activity that prevents or downregulates transcription. SALL1 HDAC1 SUV39H1 CHD5 CSNK2A1 TNRC18 RBBP7 PASD1 DDX20 SALL2 CHD3 APPL1 BAZ2A NACC2 SIRT2 BAHD1 RBBP4 MTA2 SMARCA5 CHD4 ETV3 GATAD2A LIN52 APPL2 MBD3 GATAD2B TBX15 RRP8 HDAC2 BAHCC1 SIRT1

GO_RUFFLE Projection at the leading edge of a crawling cell; the protrusions are supported by a microfilament meshwork. WWC1 MYADM CFL1 RAB5A DBNL PDLIM7 TESC SPRY4 PIP5K1C FGD1 DNM2 RAC1 SNX5 FGD3 FGD5 CLASP2 PDE4A KBTBD10 ROCK1 IFIT5 WIPF1 PACSIN1 NME2 PAK1 CYFIP1 ATP6V1B2 WASF2 PALLD ITGB1 NME1 ARF4 FERMT1 KIAA0528 AKT2 ITGA5 LAYN BMX MYO1C IQGAP1 ARF6 SNTG1 FAP EPS8L2 MYO5A CLIP1 FSCN1 SPRY2 SPATA13 ARHGEF2 ARFIP2 PTK6 MYO10 ITGB3 LCP1 CD2AP KSR1 ABL1 KLHL2 RLTPR SH3YL1 PACSIN2 PIP5K1A RINL ASAP3 FRMD4B MTSS1L ARAP3 INPP5K DLC1 MACF1 RPS3 S100B PARD6A RAB34 APC ANXA2 PLA2G4F EMR2 ITGAV TIRAP PODXL EPB41L5 FGD6 PDXP SAMSN1 RASA1 S100A11 TRPV4 SNX9 ARHGEF26 BAIAP2 ITGB1BP1 EEF1A1 COBL TNFRSF12A FGR CTTN EZR TLN1 TLN2 SLC9A3R1 RAB22A MTSS1 TWF1 MEFV CDK6 DDX58 ABCA7 INPP5J FGD2 PLEKHA1 CIB1 PDPN PLCG1 ARHGEF7 ALS2 TIAM1 MYH9 KANK1 MTM1 PSD FGD4 RDX TPM1 BCAR1 VIL1 MTMR14 NF2 S100A6 CDKL5 EPHA2 RASGRP2 AIF1L HSP90AA1 DIAPH1 PLEK PDE9A PTPRJ EPS8L1 PLEKHO1 AIF1 ARHGEF4 ADAM17 AMOT SRC THEM4 KIF18A ACTN1 CYTH3 MYO6 PPP1R9B TRPM7 EPS8 INPP5E MKLN1

GO_PROTEIN_DNA_COMPLEX A macromolecular complex containing both protein and DNA molecules. HIST1H2BO CHRAC1 HELB PRM1 SHPRH H2AFX HIST1H3E PAX2 HIST1H3C ANKRD32 GINS1 HIST1H3G ERCC5 LEF1 H2AFJ HIST1H4L XPA HIST1H4C GINS4 HIST1H2AA H2AFZ HIST1H2BI HIST1H2BA BCAS2 HIST1H2AL HIST1H1D HMGA2 HIST1H2BM HIST1H3D HIST1H1B HIST1H3B RPA4 POT1 HIST1H2AK HIST1H4I HIST1H3A HIST1H2AD HIST1H4H HIST1H2AG PLRG1 PRM3 HIST4H4 HIST1H4D TINF2 HIST2H2BE CENPA XRCC5 CTNNB1 H2BFWT HIST1H2BB HIST1H2AH HIST1H2BH OBFC1 HIST1H4K HIST1H3I HIST1H1A TNP1 HIST2H3D ATF6B PRM2 HIST1H1C HIST1H3F KCNIP3 HIST1H4B POLE4 H1FX RPA2 HIST2H3A MPHOSPH8 HIST1H4F H2BFM HIST2H2AC HIST1H2BE HIST1H1E H2BFS CCDC79 JUND SMARCAL1 TERT TONSL HIST1H2BC HIST2H2AA3 HIST1H4E TERF2 H1FOO HIST1H3J KAT6B CTC1 HOXA11 XRCC6 MCM3 TEN1 KDM5A H3F3A POLD1 KAT6A HP1BP3 HIST1H2BD CHD4 H2AFB2 H2AFY2 H1F0 HIST1H2AM H2AFB3 RPA3 NFYC H2AFV EYA1 HIST3H2A IRF4 HIST1H2BG PCNA HIST2H2BF HIST1H4A HIST2H2AA4 DDIT3 RPA1 PRIM1 HIST3H2BB HIST1H2AC TCF7L2 HIST1H2BN MYOG HIST1H2BJ POLA2 HIST1H2AB TERF2IP HIST2H2BD PURA POLE HIST1H3H HILS1 HIST1H1T HIST2H4B ACD POLE2 POLD3 POLD4 H2AFB1 SP1 JUP H3F3B TERF1 NFYB PURB GINS2 HIST2H2AB HHEX SUZ12 POLE3 HIST3H3 HIST1H4J HIST2H4A CDC5L SOX12 HIST1H2BF H3F3C CDC45 HIST2H3C POLA1 TNP2 HIST1H2BL HIST1H4G HIST1H2BK PRIM2 H2AFY NFYA HIST1H2AE HIST1H2AI NFE2L2 PRPF19 ORC3 MED1

GO_EXON_EXON_JUNCTION_COMPLEX A multi-subunit complex deposited by the spliceosome upstream of messenger RNA exon-exon junctions. The exon-exon junction complex provides a binding platform for factors involved in mRNA export and nonsense-mediated mRNA decay. TDRD3 EIF4A3 CASC3 SRSF1 SAP18 PNN RNPS1 POLDIP3 UPF3A WIBG UPF2 THRAP3 R3HCC1 MAGOH UPF3B MAGOHB THOC4 RBM8A UHMK1 UPF1 SMG6 C10orf28

GO_COATED_VESICLE Small membrane-bounded organelle formed by pinching off of a coated region of membrane. Some coats are made of clathrin, whereas others are made from other proteins. HLA-B SGIP1 SH3GL2 HLA-E HLA-C CPNE6 LMAN1 MYO1E SNX3 WNT5A VPS18 COPA NECAP1 VPS41 CTLA4 SEC23A PICALM IER3IP1 CD74 DAB2 RASSF9 CLTCL1 AREG DNAJC5 CTSZ ATP7A VPS33B MALL SCAMP1 KIAA1199 CD9 GPR107 CTSC ECE1 RGS19 RAB27A TMED3 DENND1B STON2 FZD2 AP1S2 SEC24A AP1S3 CCDC115 APOB ABCB4 AP2A1 PACS1 USO1 SEC24B HSPA8 PIK3C2A TEX261 CLVS1 HLA-DQA2 NUMB BCAP31 RAB3A ASTN2 SLC2A4 HIP1 PCSK9 RAB8A CD59 NRGN SYT1 HLA-DRB3 SLC18A3 GOPC HLA-F YIPF6 SFTPC DBNL SEC31B LDLRAP1 EDN1 F5 TMED9 CLTB DNM2 LMBRD1 YIPF5 STX5 SEC23B SLC17A7 AP1G1 GGA2 VPS33A SLC18A2 DVL2 COPG2 FCGR1A PACSIN1 SH3BP4 EPN2 STX17 ROR2 CNIH3 AP1S1 TMED7 MLC1 HIP1R SREBF1 HBEGF ARCN1 SEC24D SFTPB CNIH WIPI1 FZD5 AP2B1 YIF1A SEC31A AP3B1 EPS15 AP1M2 GOLGA2 TMED10 FCHO1 NECAP2 TYRP1 AP2S1 COPB2 RAB35 HSPD1 SCAP STEAP2 ASTN1 APP LMAN2L LMAN1L VTI1B LMAN2 CLINT1 DDHD2 LDLR SNX18 IDUA VPS16 VANGL2 COPZ2 AP3B2 AFTPH FAM109B F8 COPZ1 COL7A1 SYNRG AP2A2 AP2M1 TBC1D5 VPS11 SPG21 SEC13 VTI1A HLA-DRB5 COPB1 CNIH2 EGFR HLA-DQA1 IGF2R FCHO2 UNC13D SORT1 FURIN FOLR1 CLTA YIF1B GRIA1 HLA-A SEC24C OCRL MYO6 HLA-H GOSR2 KLHL12 ARF1 EPN3 TGFA KDELR1 FCGR1B CD207 HLA-DRB4 SLC18A1 GAD2 COPG SEC23IP SFTPD DENND1C HLA-DRA TNK2 TGOLN2 SNX9 HLA-DQB1 HLA-DPA1 TMEM199 VAMP2 INPP5F AP1B1 AAK1 CLVS2 SNAP91 SFTA3 HLA-DRB1 SFTPA2 NCALD DVL1 TMED2 AP1G2 FZD4 DENND1A AP1M1 SCYL2 SREBF2 GAD1 STX6 SEC22B COPE RAB14 HLA-DPB1 RAB27B HLA-G MCFD2 B2M VWF KIAA0368 FAM109A VMA21 SAR1B LRP1 SLC32A1 C3orf58 SCYL1 SFTPA1 VAMP3 CLTC SERPINA1 HLA-DQB2

GO_ENDOPLASMIC_RETICULUM_LUMEN The volume enclosed by the membranes of the endoplasmic reticulum. CTSC VTN P4HA3 COL4A6 PDIA2 CFP ERLEC1 APOB PDIA3 PTPRN2 RCN3 CLN6 DNAJB11 ARSH COL27A1 COL9A2 ARSD FKBP10 BACE1 TOR2A PDGFB ADAMTS7 CERCAM COL1A2 APOA4 COL5A3 COL22A1 COL6A3 WNT5A HRC TOR1A TOR3A MBTPS1 COL21A1 PDIA5 COL10A1 ARSK ADAMTSL4 COL4A2 F7 CTSZ P4HA1 COL24A1 ADAMTS13 HSP90B1 COL4A5 SRL HSPA5 STS COL4A1 COL23A1 P4HA2 PDIA6 GIP ARSA TMEM43 CES3 THBS1 ERP44 OS9 GAS6 PLAUR GCG C19orf10 ARSI HYOU1 PRKCSH SLC27A2 RCN2 WNT4 FMO1 COL6A1 ERAP1 ARSF ADAMTSL1 ARSG RCN1 EDN1 EDEM3 COL12A1 LIPC ARSJ FKBP7 SIL1 DNAJC3 F5 TOR1B ARSE ESD H6PD GPX8 FLT3 SUMF2 KDELC2 ADAMTS5 COL9A3 ERP27 TRDN CASQ2 COL3A1 MZB1 PDGFD ERO1L CALR3 INS FKBP14 SHH COL13A1 EDEM2 DNAJC10 WNT6 CYP2W1 LEPRE1 GLT25D1 COL9A1 COL4A4 COL11A2 CALR LRPAP1 COL18A1 CES1 PDGFA F2 CALU UGGT2 MTTP WNT3 PPIB CANX MINPP1 GLT25D2 PDGFC F8 COL17A1 WNT3A TXNDC5 COL28A1 TXNDC12 RNASET2 WNT7A PROZ ERP29 COL19A1 COL7A1 SERPINH1 BCHE CASQ1 ARSB CNPY3 RDH5 LEPREL1 DNAJB9 CD4 CES2 WNT7B GANAB PACS2 F9 SUMF1 COL11A1 B2M PTGS2 WNT5B COL14A1 PDIA4 F10 GBF1 APOA2 COL20A1 CRTAP KDELC1 GPX7 SPON1 EMID2 PROC SERPINA1 COL1A1 GHRL TXNDC16 COL8A1 APOA1 ERAP2 COL5A2 WNT1 COL8A2 COL16A1 COL15A1 COL4A3 BGLAP COL5A1 SDF2L1 C3orf64 P4HB FOXRED2 NTF4 SEP15 COL6A2 UGGT1 COL25A1 POGLUT1 COL2A1

GO_SPERM_FLAGELLUM A microtubule-based flagellum (or cilium) that is part of a sperm, a mature male germ cell that develops from a spermatid. RSPH1 SEPT12 DNAI2 SLIRP RNF38 ODF4 IFT81 DDX6 PGAM4 SQSTM1 TXNDC8 PGK2 ENKUR CATSPERG ABHD2 AKAP3 DEFB1 NME5 SLC26A6 TCTEX1D4 HK1 SPA17 AK8 SEPT4 DNAJB13 DNAH1 TEKT3 ATP2B4 AK2 PFKM PACRG SLC26A3 KIF2A GSTM3 AK1 SPEF2 CABYR PTCHD3 CCR6 ODF3 ODF2 IFT172 FAM154A CATSPER4 AKAP4 IQCG TEKT5 SLC9A3R1 IFT27 MNS1 DRD2 ALS2CR12 RPGR TXNDC3 TMEM146 HSP90AA1 SPATA6 TXNDC2

GO_RNA_CAP_BINDING_COMPLEX Any protein complex that binds to a specialized RNA cap structure at any time in the lifetime of the RNA. NCBP2 EIF4E1B PIWIL1 ZFP36 NCBP2L RNMT EIF4E3 FMR1 CYFIP1 FAM103A1 EIF4E2 EIF2C2 EIF4E NCBP1 C17orf85

GO_CHROMOSOME A structure composed of a very long molecule of DNA and associated proteins (e.g. histones) that carries hereditary information. C12orf32 RING1 NUDCD2 POLE4 RRP1B RPA2 H1FNT HIST2H3D HUS1B POGZ PLK2 RFC1 PRM2 CENPK RARA CFDP1 NUP43 NBN SALL1 SRF CTNNB1 APTX H2BFWT RECQL5 MYCN ZWILCH LRWD1 CCDC79 SGOL2 HAND2 NUP160 TTC37 BUB1 SALL4 RAD21L1 TELO2 BOD1 NDEL1 C11orf80 PES1 RAD18 RNF2 NR1H4 CHAMP1 TTC15 MSH2 HDAC2 POLR2B ZNF276 RIF1 SEPT7 FAM208A ZFR RGS12 RAD50 PAWR SGOL1 NSMCE4A APITD1 H2AFX FER TADA2A SETD1A PPP1CA HMGN4 NARG2 MUC1 RECQL ANP32E CCDC137 GSG2 HMGB3 TMPO RSPH1 RAD51B ERCC6L TINF2 HMGN1 YY1 C9orf114 THOC2 MTBP RBM19 SEPT6 RXRA ZNF238 SPO11 HIST1H2AG MSH4 SETD2 KDM1A FOXC1 PPP1CB STAT6 DCLRE1C MCM5 CLIP1 ASH1L TEX14 HIST1H2AL CTR9 SETDB1 E2F4 ZRANB3 BCL6 LRMP DAXX TOPBP1 WDR61 RNF212 PURB MBD1 PLCB1 LDB1 PPP1CC HMGB2 TP63 H2AFB1 H3F3B CDK4 NUF2 MLF1IP DMAP1 HIST1H3H PHF1 MEF2A HELLS PIF1 INTS7 POU4F1 WAPAL NUSAP1 ORC3 CCNB1 MSH6 SNAI2 UBE2A KIAA1267 ATF2 TIMELESS MLH3 ZNHIT1 PRDM9 KAT5 ETV3 SYCP3 BUB1B SUMO1 CCDC75 THOC5 TNP2 RNF8 SMARCE1 HDAC1 CDC5L RNF169 MSH5 NCAPH2 EXOSC4 ZWINT MIXL1 TARDBP MBD4 SUV420H1 EIF2C3 HIST1H2BD KIF18A UBE2U TOX4 CHD4 H2AFY2 PARP1 NELF PHOX2A MCM3 TEN1 PMF1 SUDS3 H3F3A TP53BP1 SFPQ REPIN1 EHMT2 XRCC6 MIS18A MSH3 HIST1H2BJ DSN1 SUMO3 ORC6 RAD9B SUV420H2 IRF1 HIST1H2BN THOC3 SIN3A THAP7 CBX7 RRS1 WBP2 NUP98 CENPL MPHOSPH10 KIAA0146 NSD1 NR1D1 NEIL1 EXOSC5 HSF1 KLHDC3 FBXO18 GATA3 PHF12 HIST3H2A NUP107 DYNLL1 PRPF4B AKAP8L SETD3 HIST1H3F NGDN HIST1H4B DYDC2 SUGT1 PHOX2B HIST1H3I SCMH1 KDM4D ITGB3BP IL33 KIFAP3 CENPA NCAPD3 CENPV BUD31 CSNK2A1 MLH1 SYCE1 HIST1H2BH NDC80 ASF1B RFX3 HORMAD2 SMARCAL1 TUBG1 ZBED1 PPP1R7 HIST1H2BC CENPE KAT8 AR SLX1B HIST2H2AC ANKRD17 PRDM7 MPHOSPH8 CDK2 ASXL1 EME1 CCND2 CREBBP POLQ HIST1H4F ZBTB4 NDE1 ZW10 SMARCB1 NUP133 ATR PLK4 APC RUVBL2 GINS4 DPF2 H2AFZ SMG6 HIST1H2BA ORC5 ANKRD32 TRNP1 ERCC5 PINX1 PRKDC RNF20 DBF4B RAD21 C1orf131 THOC1 NAT10 DFFB HIST1H2BO HELB SMC1B CDKN2A PPP2CA SHPRH TFPT STAG2 CLASP1 RFC4 TNKS1BP1 HIST1H4D RASSF2 STRA13 DDX6 HIC1 PMS1 UCHL5 CENPC1 TOP1MT RBBP6 PRM3 ORC4 CBX1 SKA1 PAFAH1B1 HMGB1 RFWD3 TBP FEN1 HIST1H1D UHRF1 MEAF6 HIST2H2AB CASC5 CENPO SUZ12 HDAC8 ASF1A NEK2 HIST3H3 MRE11A HMGN5 MYOD1 CCDC99 BEND3 KDM3B POLE2 SYCP2 PIWIL2 FAM60A SP1 SMC4 MMS22L SMC3 TERF1 NUCKS1 SMAD3 HIST1H1T SMAD2 MAEL PPP2R1A FOXH1 DCTN2 GATAD2B JUN LIG4 MED1 SUN2 ESR1 DYNC1I1 CAPN2 IKZF1 CDK1 MORF4L1 ASH2L H2AFY NFE2L2 BRCA1 APEX1 ORC2 H3F3C SETD8 MCM10 NUP37 HIST1H4J C15orf23 HIST2H4A KIF4A NCAPG CBX3 SPC24 HIST1H2AM ATRX HP1BP3 RCC1 POLG2 PSEN2 CENPM H2AFB2 ERCC4 NIPBL SSRP1 JUNB NUFIP1 ZNF207 SUPT16H NHP2 POLA2 SMC1A RB1 PPARGC1A MBD2 NUMA1 POLE PPP2CB HIST3H2BB NSMCE1 PCNA CIRH1A CREB1 RPA1 C9orf142 INO80 ORC1 KIAA1967 INO80C PPP2R5A FANCD2 MAU2 BRMS1 SMARCD3 HIST1H2BG CBX8 H1FX ENC1 HIST2H3A MCMBP HIST1H1A SEH1L SMARCC1 FBXO28 BOD1P MTA2 C15orf43 MDC1 HIST1H1C SUV39H2 HIST1H4K SMC2 CENPI SYN1 CDCA5 NCOR1 CTCF KIAA1530 HIST1H2BB TOP2B PHF2 MIS18BP1 HIST1H4E BRMS1L CENPQ HIST1H3J CENPF MAD1L1 KIF22 CLASP2 H2BFS SIN3B SSB STAG1 AKAP8 TONSL HMGN3 NLRP2 HNRNPC OIP5 USP3 DCTN3 SYCP1 RANGAP1 FBXO11 OBFC2B NDNL2 CITED2 CDCA8 RNF138 NUP85 RARG UBE2I HIST1H4C MCM2 KDM3A XPO1 EP400 HIST1H3G ACTR8 GABPA CXorf27 SMARCA5 NCOR2 SPI1 RAN BRCA2 NFATC1 CKAP5 TCP1 ARID1A HUS1 CHAF1A PHF6 ADD3 HIST1H3E DOT1L CHRAC1 ARRB1 BRD4 SETD7 ELL TOP3B NCOA1 HIST4H4 THRB SATB1 DMC1 PADI2 PML HIST1H2AK NACC2 HIST1H3A CDCA2 POLR3D HMG20B SYCE2 MCM6 POLR3GL TAL1 RAD1 PKHD1 CHEK1 HIST1H3B WHSC1 CHEK2 NEDD4 HSPA2 HIST1H2BM HMGA2 HIST1H3D RAD17 POLE3 SMC5 BAHCC1 SPC25 PELP1 SPATA22 CENPN ZMYND11 PLK1 PPP1R10 CENPW GINS2 INO80B RECQL4 FBL SLX4 RAD9A DPY30 DCLRE1B MAD2L1 DDX27 POLD3 TRRAP ANAPC16 LEPREL4 STAT3 KIAA0020 TCF3 HLCS KIAA0947 PSEN1 ESCO2 RNF168 PRPF19 NASP EXOSC10 SIRT7 UVRAG ERCC1 HAT1 WDR81 PTGES3 KDM4A PRIM2 HIST1H2BK UBE2B INCENP RAD51C HIST1H2BF DCLRE1A DDX18 HIST2H3C SNW1 HJURP EXOSC3 FOXD3 PMS2P3 PARP3 DNTT ANKRD2 HORMAD1 H1F0 UBA1 H2AFB3 CENPT DCTN5 T C1orf86 VCP THOC6 CENPB DTL TOP2A DNMT1 KNTC1 SMARCA2 PBRM1 UHRF2 DCTN1 SKA2 TP73 SEC13 TCF7 EIF3E SCRT2 SIRT1 AURKB STAT1 WHSC1L1 SOX18 RFC3 PAX6 BAHD1 SEPT2 SPAG5 RBBP8 ZNF385A HIST2H2BF TRPS1 AURKC TOP3A HIST2H2AA4 DNMT3A STAG3 MYBL2 SETDB2 PHC2 SMAD4 TOP1 SUV39H1 CHD5 H2AFV TIPIN THOC7 FAM111A IRF4 CSNK1A1 ACTL6A SMARCA4 THOC4 INO80E WDR43 XRCC4 BCAS3 FLNA NCAPD2 ZBTB38 TNP1 NCOA3 BUB3 BAZ1B C12orf48 SYCE3 MKI67 TCF12 PBX4 HTRA2 WRN EXOSC9 OBFC1 MECP2 PDS5A TPR PPARD WRNIP1 XRCC5 TRIM28 NSL1 HIST1H2AH MBD5 SLX1A TTN NOL6 RRP8 TERF2 NFRKB H1FOO TP53 CBX6 DYNC1LI1 KLF1 JUND RCC2 TERT DYDC1 HIST2H2AA3 SMARCAD1 LLPH REC8 TEX12 LRPPRC PPHLN1 HIST1H2BE HIST1H1E SP100 NCAPH FKBP6 SAP130 BIRC5 RUNX2 ATM NSFL1C OBFC2A ZFP57 H2BFM SMARCD2 H2AFJ HIST1H4L NPM2 MAGED1 CEBPB AURKA XPA HMBOX1 HIST1H2AA HIST1H2BI BOD1L SMC6 HIST1H3C SIRT2 RBBP4 GINS1 GAR1 CBX5 MKI67IP NRIP1 MCM4 ZMIZ2 SETMAR XRCC2 RAD51AP1 BIRC2 PRM1 DHX36 LOXL2 RUNX3 CENPP RUVBL1 TNKS2 UBR2 HIST2H2BE CALCOCO1 UPF1 POT1 PPP2R5C HMGA1 HIST1H4I HIST1H2AD TEP1 IPO4 FIGNL1 HIST1H4H PLRG1 PSIP1 CCDC155 ACTR5 TFIP11 HIST1H1B CCNB1IP1 POLR3G RAD51D RPA4 BCAS2 DYNLT3 BRD8 CDC73 DNA2 CHAF1B MIS12 BLM ESCO1 ACTB GTPBP10 SIRT6 TEX11 CHTF18 SWI5 EHMT1 XRCC3 FMR1 MCM7 AHCTF1 DDX11 ACD RFC5 POLD4 ALKBH1 PINK1 SETX JMJD1C DDB1 DMRTC2 HILS1 HNRNPK AFF4 SKA3 HIRA HIST2H4B MAF MBD6 MBD3 CLOCK CD3EAP ZNF330 CDX2 KIF2C DFFA MEN1 CBX2 DCTN6 HIST1H2BL WDR82 HIST1H4G C11orf85 DVL3 C1orf124 EZH2 HIST1H2AE MCRS1 HIST1H2AI SS18L1 DSCC1 CDC45 ZSCAN4 HMGN2 SYCE1L POLA1 RFC2 ING2 TRIM24 KIF2B RAD51 SFR1 DLX5 EED APLF PPP1R12A RBMX NSMCE2 KAT2B BANF1 POLD1 RNF40 KAT6A C16orf73 E2F1 KAT2A KAT6B EME2 SETD1B CTC1 SMARCC2 FAM178A L3MBTL1 ZNF830 KDM4C ING3 TTC21B HMGB4 HIST1H2AB HIST2H2BD TERF2IP RELA SMCHD1 PURA BAZ2A NR1H3 HIST1H2AC CHMP1A TCF7L2 CENPH EID3 MXD1 PARK7 LRIF1 HIST1H4A PRIM1 PCGF2 KLF4 TCF4 PDS5B LOC728637 TNKS RPA3 ZC3H8 HR HEY2 TNRC18

GO_CLATHRIN_COAT_OF_ENDOCYTIC_VESICLE A clathrin coat found on an endocytic vesicle. PICALM SGIP1 EPS15 AP2A2 EGFR LDLRAP1 AP2B1 EPN2 AP2M1 AP2S1 SLC18A3 AP2A1 TBC1D5

GO_APICAL_JUNCTION_COMPLEX A functional unit located near the cell apex at the points of contact between epithelial cells, which in vertebrates is composed of the tight junction, the zonula adherens, and desmosomes and in some invertebrates, such as Drosophila, is composed of the subapical complex (SAC), the zonula adherens and the septate junction. Functions in the regulation of cell polarity, tissue integrity and intercellular adhesion and permeability. RPGRIP1L CLDN9 EPCAM AMOTL1 PARD3 PKN2 POF1B BVES CLDN10 FRMD4A DLG3 TJP1 TBCD CGNL1 WNK3 MTDH CLDN22 INADL RAP2C AMOT AMICA1 VASP APC MARVELD2 CLDN12 RHOA CLDN6 CLDN4 PARD6A ECT2 MAGI2 JAM2 PMP22 CXADR SHROOM2 ARHGAP17 MPP5 CLDN23 ASH1L CDH1 LIN7C CLDN19 PRKCI PRKCZ PLEKHA7 CSDA CRB3 PLXDC1 CLDN11 CLDN14 NHS ARHGEF2 CLMP KIFC3 STRN CCND1 CCDC85C OCLN CTNNB1 NPHP1 LIN7A ADCYAP1R1 SORBS1 IGSF5 NPHP4 SYNPO CDK4 CLDN1 JUP LIN7B CTNND1 TRAF4 CTNNA1 JAM3 DDX58 SHROOM3 TJAP1 CGN TJP3 RAB13 F11R CLDN24 FZD5 ANK3 CLDN7 CLDN18 CLDN25 PDZD3 PARD6G VAPA CLDN8 PVRL2 AMOTL2 MPP7 RAPGEF2 LOC100288814 MICALL2 MAGI1 RAP2B FRMPD2 CLDN20 CLDN2 CDH5 CLDN5 CLDN16 ABP1 PARD6B CLDN15 CLDN3 MAGI3 ESAM MPDZ CLDN17 EPB41L4B DLG1 CAMSAP3 TGFBR1 FBF1 FRMD6 PVRL3 PARD3B C1QTNF5 SYMPK MARVELD3 TJP2 UBN1 WNK4

GO_BOUNDING_MEMBRANE_OF_ORGANELLE The lipid bilayer that forms the outer-most layer of an organelle. KIAA2018 ST20 RAB6A HMGCR PPP2R2B GUCY2D HLA-DRA BLOC1S2 NUTF2 GAL3ST3 RGS9 HLA-DOB LAMTOR1 VPS35 SIGMAR1 ENTPD4 HACE1 GALNT2 ATP6V1C2 ATP8A2 MARCH8 COMMD1 CNP RPS6KB1 PSEN1 CHMP4B AP1G2 VAMP4 MCFD2 NRAS HLA-DPB1 ATP13A2 PLOD2 SMPD4 SYN2 RAB14 BAX MAPK8IP3 MTCH2 WDR11 STEAP4 TMX3 GBF1 ABCA7 SNX21 AKAP6 MRC1 CAMK2A ATP6V0A2 SNCA PI4KB DIO3 LMAN2 LMAN1L PGAM5 BOK SLC35A2 RAB12 SLC17A6 ARF3 DHCR24 SNX18 SLN SV2C DMPK STARD3NL SNX6 WNT3A ACPL2 SEPT2 EPB49 IKBKE ITGA2B AP3B2 ATP8A1 CD164 DOC2A GALNTL5 GGTA1P BCL2L10 SLC11A1 RFFL LAMTOR3 MAP1LC3B EGFR RRAGB RDH14 MOSC1 TMEM167B MYO1B ENPP1 PICK1 GPR89B THBD SLC30A8 MYO6 VASN CYTH3 ATP7B HSD17B4 ATG16L1 LPIN1 HLA-A LRP6 TGFA B4GALT6 INPP5E C12orf5 RNF175 ACSL3 GOSR2 TRAPPC3 INPP5B LAMTOR2 MSR1 RAC1 TCTN3 ADAM8 CLTB GNPTG WLS SPATA19 HMP19 TMEM79 GGA2 RAB20 SLC17A7 CACNG3 ATP6V1G2 PEX14 RYR2 SPACA1 DECR2 GYLTL1B AGTRAP DTNBP1 SLC26A11 PKMYT1 ROM1 CHMP4C EPN2 MANEA ANXA8 SCAMP5 SLC16A13 SYCN DHRS7C OSBPL9 SOX10 ATP6V1B2 TLR3 NME1 GCNT2 DAO MGAT5B GJA1 SYNGR4 MYD88 RHEB MST4 MYOC ATG12 STEAP2 CEACAM1 SLC29A3 NECAP2 PECR MAP2K2 APOO RAB1A VPS4B SNX19 ATP11B ABCD3 TLR4 ATP2C2 ATG5 CYBASC3 A4GNT PML TMED5 RAB9A HSP90AB1 PLD6 C19orf46 TOMM6 TMEM130 PNPLA8 ST8SIA2 MALL BCL2 FIS1 DPP4 ATP9A SCARB2 KDELR2 BNIP3L TMED3 HPS6 ABCD1 SBF2 TMBIM4 IRAK2 FAM170B EPHB1 VPS52 SNX16 TEX261 ST6GALNAC2 TMEM59 GRM6 CORO1A ARL1 VPS37B ABCD4 ERGIC1 ANXA1 ANXA7 GOLGA8B RAB8A TH BACE1 TMEM5 TM6SF1 SLC18A1 TMEM93 VPS25 TOMM20L REP15 B3GNT8 ERBB2 ATP2C1 PIK3R4 TMEM55A C20orf29 HK1 ABCC4 CISD1 CHST5 GPR137B SLC9A8 ATP6V1G1 RAP2A VPS39 PXMP4 CLCN7 SYTL4 GAD1 SREBF2 IL15RA SLC30A2 SAMM50 MOAP1 PCYOX1L SYT2 HLA-G TMEM9 PI4K2A QPCTL ULK1 PINK1 COPE SAR1B SNX27 RND2 PAQR3 MAN1C1 SCYL1 SRPRB VPS37D TNKS ST8SIA6 GIMAP1 SGMS1 PDGFC HRAS MTX2 LDLR CHMP1A BBS4 COL7A1 MLXIP GALNT6 WNT7A ARMCX3 ICA1 ZFYVE9 CD46 VDAC3 TICAM2 BNIP3 PROM1 ZFYVE26 CHPT1 NAPA BBS9 SYNGR1 PDE6G NSF NPC1 SLC15A3 SPATA18 PREB RABEPK FOLR1 GNPTAB HLA-H MTMR4 RAB11A SNAP29 PEX11A RAB11FIP1 PCSK7 B4GALT1 SPNS1 HGS SEC24C GRIA1 LOC100288842 C19orf50 FAM18B2 SV2B ARL13B USP8 UNC50 LLGL1 ABCC10 C14orf49 CABP7 DNAJC13 MRVI1 PDE6A MFN2 TMEM175 CALCOCO2 PLOD1 CCZ1 SPACA3 EXT2 CD163 LRPPRC LITAF PSENEN MCOLN1 SLC25A17 RCC2 ITPR1 SPPL2B CSNK1D B3GALT6 MT3 MPPE1 CASQ2 SLC30A6 ATP11A ARFRP1 BAK1 KIAA1324 CDIPT CUL3 CNGA1 SLC2A1 CHPF2 GALNT4 UBAP1 WDFY3 DEPDC5 GPR143 COG6 EPS15 TMEM8A FZD5 CNIH PHLPP2 SNAPIN ZFYVE16 SCGN RAB33B OPTN GLIPR2 ATP5G2 CUBN TMEM35 SCAP WNT4 WNT5A DRAM2 TRAPPC6A HRC SLC17A8 UBAP1L GAA SCNN1A RAB11FIP3 SLC36A1 GOLPH3 CD74 BCL2A1 TLR7 C11orf2 SLC39A4 CHST1 GPAT2 ITGB3 GORASP2 ACSL6 SCARA3 LPCAT1 CYTH4 DHRS3 AP2A1 PEX5L AP1S3 SLC35B1 AP4S1 RRAGD FIG4 CFTR RAB23 HLA-DRB3 PPP6R1 CD59 RET TMEM67 CLCA1 CLCN3 PCSK4 SLC35C1 TMEM55B HLA-DRB4 GALNT11 TMEM9B DRD5 PCDH7 VPS26A B3GNT4 NOSTRIN GUCA1A ATP11C SNX2 CHST6 SMO NEU1 TRAPPC4 PITPNB JPH1 KIAA1244 B3GNT3 HHIP TMEM109 FLOT2 ATF2 EEA1 RHOQ TRAPPC9 B4GALNT4 GPSM1 CAV1 ABCA5 B2M C1GALT1 CDC42 MGAT4B MARCO NCSTN RILP FAM21C NLRX1 NCAM1 PEX5 HS6ST2 B3GALT4 GOLGA7B PLD1 PXMP2 PPP1CC B3GAT3 FNDC5 HLA-DQB2 MAP1LC3A MTX3 ASS1 WASF1 PHACTR2 C6orf89 PITPNM1 IZUMO1 M6PR CYP27B1 CNGB1 NDST4 ST6GALNAC5 RAB21 TMEM17 TMEM173 CNGA2 HADHB CP RGP1 TMEM63A EPHA4 GCC1 VPS4A COPZ1 RNF125 PRKCI GOLGA3 RASGRP1 GPR89A CLSTN1 MYLPF AP4M1 GPLD1 SNTB2 AP2M1 SACM1L STAB2 FAR2 SNX8 PAM HGSNAT C18orf8 ADC VAMP1 RAB32 GRK1 ANXA6 FURIN SYPL1 GCNT3 IGF2R MOSC2 GBP1 PEX13 NMNAT2 PKD2 KLHL12 GLG1 LTC4S RAB11B NTRK2 ITM2B GOLGA4 SLC22A17 HAX1 SRI GALNT8 GNA11 LOH12CR1 ERGIC3 SYT6 NAT8 SYPL2 ST3GAL5 SLC35B4 SYNGR2 SERINC3 SLC35B3 SV2A MOB4 EXT1 MMP24 MDM2 ZDHHC9 GBP4 STEAP3 BBS1 CASP8 VPS33A EHD2 GNB2 DRD2 FUNDC1 B3GNT5 GNAI1 NDST3 GK2 NAA60 YIF1A WIPI1 STX16 GUCA1B ZG16 COL6A1 MAN1A1 RNF13 LARGE GAL3ST2 GALNTL4 CHPF SNX3 SH3GL2 PEX26 HLA-C SEC23A HS3ST3B1 B3GALT5 COPA CYB5R3 GABRA2 OPN1SW GPR161 PEX12 SCARF1 AHNAK FUT11 CREB3L4 TTC1 SCG3 ZDHHC13 ABCB6 AREG HS2ST1 KIAA1432 CYB5B B3GAT1 ATP5G3 CLN3 CD9 LNPEP MAP4K2 ATP6V1A SYP COLEC12 AP3M1 TICAM1 COG1 ATP2A3 GNGT1 ATP2A1 GOLGA7 ST6GAL1 ARFIP1 NOTCH4 RHOT1 GALNT5 C20orf103 SYT1 ACER2 SNF8 GOLGA5 RAB6B RFWD2 C17orf28 TMEM225 SNX10 PGR MARCH2 FAM125A B4GALT7 CHST3 ASAP2 PPP6C B4GALNT1 CYBA CLIP3 PSAP GAD2 SYNE1 EEF1A1 HLA-DQB1 SCFD1 SLC35A4 TRAPPC5 SGMS2 RAB11FIP2 INPP5F STX6 CBFA2T3 LAMP1 SLC24A5 ABCA3 SYS1 HS3ST5 WNT7B GHRHR RAP1GAP ST6GALNAC1 GALNT9 SPG11 ARL6IP1 TSPEAR SLC50A1 RNF185 LRP1 LRRK2 RPN2 SEC16A ATP6V1G3 SYT12 SMCR7L PI4KA TOMM20 BBS7 TAS2R46 VAMP7 STARD3 AP2A2 SNX20 CLSTN2 RPH3A WHAMM PMAIP1 RAB43 LFNG ZC3H12A GGA1 HLA-DQA1 CNIH2 SYT10 HLA-DMA PEX16 SYNPR BCL2L1 MPHOSPH9 BET1 SORT1 MTERFD1 MYO7A ATAD1 TMEM74 RAB5B OCRL TRAPPC1 VPS53 CHST13 EHD1 ZFYVE20 YIF1B NAT8L PLEKHF1 GNB4 TFG MAVS PLCE1 STEAP1 TM9SF1 SLC9A9 C18orf1 SLC7A14 CLEC16A ATG9B GALNTL2 ATP6V0D2 SH3GLB1 STX12 ACP2 GUCA1C OPA1 FAM82A2 ATG16L2 YIPF5 ATP6V0B SLC35B2 IMP5 TAPBPL SLC18A2 SLC12A4 COPG2 ATP6V0E2 RYR3 SCARB1 TTC8 STX17 ZFYVE28 TOMM70A SLC45A2 NOS1AP HBEGF GOLPH3L C1GALT1C1 QTRT1 MGAT3 MGAT2 SEC31A GLCE RAB13 PTCH1 AIFM2 ARCN1 CHP TLR1 MMGT1 SPTBN5 RP9 MEF2BNB C19orf28 KIAA1609 HLA-B RHOU TSPAN1 SLC44A1 JPH4 SNX17 CPT1B CD55 MBTPS1 B3GNT1 UBE2D3 NDRG1 CHMP5 VPS28 ITPR3 KIAA1199 B4GALT2 TOMM40 B4GALT4 CYS1 SLC25A46 PLOD3 SUN1 ZDHHC21 MPV17L TMEM38A CHST7 PIKFYVE DIRC2 HYAL2 BID ACOX1 VPS36 MSTO1 GCNT4 CD300LG HK2 RRAGA SEC24A CTSA GXYLT1 BCL2L11 ST3GAL3 GNAI3 TAPBP RUFY1 PLA2G4F PLA2G4B FUT3 ST3GAL1 CHMP1B ST6GALNAC6 VAMP8 CSPG5 ARG1 C7orf28B IFNGR2 FUT7 USP30 NCF4 GNPAT APPL1 ACBD5 IRAK4 SPNS2 XYLT2 ACBD3 ANKZF1 SLC39A13 MUDENG NCALD CUX1 GNB1 ST6GAL2 ABO WASL COG5 VPS13C CNGA4 ST6GALNAC4 ATP6V1F WNT5B SSTR3 RAB17 GRK7 FAM57B LOC100507003 RAB2A FGD2 P2RX4 TMEM59L ASPSCR1 VAMP3 C3orf58 SRGN SLA2 STAB1 ABCD2 FUNDC2 RETSAT ST8SIA4 GAL3ST4 SYT5 VDAC2 PGAP2 CHMP4A MAOA BAD CD1E FKBP1B RAB41 FAM176A F8 TAS2R4 TLR8 RNF121 PQLC2 LRP2 SEC13 FKTN MAN1A2 XYLT1 INS PECAM1 D4S234E CORO7 LALBA GOLGB1 CYP2E1 DKFZp761E198 CHST10 PMEPA1 FUT9 WASH1 SLC35C2 GNPNAT1 ABCG1 OSBPL11 SYNE2 MMD2 ARF1 IFT20 UBA1 ARAP1 TMEM184A COG8 SEC31B FAM198B RAB5A SLC17A5 MFF SLC48A1 STXBP2 UMOD DNM2 TLR9 TMED9 F5 AMN HSD17B6 PLEKHF2 KBTBD10 CHMP3 IRF7 SARM1 ACAP2 B3GNT2 NXNL1 CHMP2B ARHGAP21 TM6SF2 GABARAPL1 GOLT1B ARL3 RAB11FIP4 CYTH1 RAB5C ZDHHC3 OPN1LW PEX19 DHRS4 SREBF1 C19orf26 PIEZO1 GALNT12 MTOR TMED7 SYN1 AP1S1 AP1M2 GOLGA2 TMED10 SLC27A2 BMF DSEL SYNJ2BP VPS37A NAT8B STX7 MANEAL RAB35 CD1B VPS18 SHANK2 RNF152 QSOX1 ZDHHC2 RAB2B VPS41 GIMAP5 HEPACAM2 NECAP1 GNAT2 BRI3BP CLCN5 MAN2A1 PRAF2 CACNG8 GK GDAP2 PHB2 SLC2A8 MAP1LC3C VPS33B TMBIM1 OPN1MW2 CLTCL1 ARFIP2 AKAP1 B4GALT3 RAB27A ARRB1 ERGIC2 RAB9B EEF1A2 CTSC ECE1 TSPO CHMP7 CAV2 STX3 C1orf85 AP1S2 FZD2 SLC35A3 HSPA8 GLTPD1 SYBU GALNT10 AMPH NAV3 FTCD AMBRA1 NOTCH1 RAB3A UBA52 HLA-DQA2 CSGALNACT1 TMEM138 INSR FCGR1B TNFRSF1A MIOS RAP2B ZFYVE27 UGCG TGOLN2 STK25 LTV1 CACNG2 CHSY1 GRIA2 ATG14 SAMD8 TMEM199 FUT1 BBS5 AP4E1 RAB7B EXOC3 UNC93B1 GALNT14 CHST2 SLC35A1 SPPL2A C4orf49 EBAG9 DENND1A TMED2 MFSD8 TPCN2 TRAPPC6B CD8B RAB27B C18orf26 KIAA0319L GCNT6 CHST9 ATP8B3 MTMR2 SEC22B GBP2 CALN1 ZDHHC17 GDAP1 DOPEY2 VPS37C SNX14 SCOC ANTXR1 WDR41 ATP6V0A1 GNAT1 SLC32A1 JPH2 RAB1B PROM2 ASPH CHST8 VTI1B TYR CISD2 NDFIP1 MAP3K7 UST ST8SIA1 ST8SIA5 TRAPPC2 GBA IFITM3 KIF13A SYNRG ARHGAP32 TAS2R43 C7orf59 A3GALT2P NAPG SPG21 SCAMP2 VPS11 ENPEP KMO HBXIP IRAK1 RAF1 C1orf9 SYT9 USP6NL BLZF1 MAOB ACSL5 STX8 CASQ1 LAPTM5 ENTPD6 COPB1 SLC44A2 CD14 PLN ATP6V1C1 TAB3 RYR1 LY96 SLC15A4 WNT6 CD68 TOM1L1 SRP9 TAB2 DAGLB COG2 ARL6 AGPAT5 GUCY2F GALNTL1 KIF16B DSE VAPB RPS27A SMPD3 DNAJC3 CTNS AQP2 SLC11A2 WDR83 HSD3B1 SERPINA5 ATP6V1E1 TMEM38B ROCK1 TMEM150C RAB11FIP5 CYTH2 PLIN3 IL17RD SLC38A9 WDR44 GOLGA8A ATP6V0C ECE2 BSG ATL1 CLSTN3 CHMP6 SYN3 MARCH9 CHSY3 GALNT3 MCL1 C3orf55 NAPEPLD ARFGAP3 VPS54 IFI27 BLOC1S1 ATF6 SPHK2 CANT1 EVC2 LMAN2L SNX13 APP TMEM167A RTN4IP1 BCL2L2 COPB2 SLC6A4 B3GALT1 ATP6V1H FUT6 M1 GAL3ST1 FUT8 SYNGR3 LMAN1 RHBDF1 PDE6B PKD1 CCDC155 ZNRF1 NDST2 PLEKHB2 LETMD1 SYT11 TM7SF4 HS3ST6 PLEKHM1 RHOD RPH3AL DAB2 EPHA8 SNX1 TECPR1 NAPB ST3GAL2 IER3IP1 TBK1 TLR2 SLC30A4 SCAMP1 RAB8B SMCR7 TNKS2 DNAJC5 SYT7 DNM1L GPAM RER1 STAM2 RRAGC FAM21A SYNDIG1 ATP6V1B1 FKRP LAMP2 ATP6V0D1 MGAT4A GNRH1 RAB1C PACSIN2 ACSL4 RAC2 SEC24B MIA3 B4GALNT2 JPH3 ZP3 MGAT4C SLC18A3 ANXA2 TMEM165 PDGFB ARL8A AZU1 MARCH3 COG4 LST1 ATG9A SNX25 ITPR2 MARCH1 TRAF6 HM13 KDELR1 WNT1 MITD1 HLA-DOA TM9SF2 CYB5A COPG GALNTL6 PKD2L1 HLA-DPA1 ZNRF2 FUT5 CLCN4 PTPRN FAM18A HLA-DRB1 ABCA4 RND3 AP4B1 AP1B1 CLVS2 STK11IP BBS2 CHST4 AP1M1 GCNT7 PROS1 CD1A DRD1 MARCH5 SLC33A1 TMEM163 RAB22A STAM ARFGEF1 TPST1 LMTK3 PEX11B TMEM44 CLTC AP3D1 TMEM115 ACSL1 FAM125B LGR6 DKK1 WNT3 CAV3 VMP1 B3GAT2 ABCA2 CACNG4 TMEM132A SLC35A5 COPZ2 IMPDH2 VPS16 ART1 NDST1 WASH6P AFTPH CD36 COG3 DHCR7 VAT1 RFNG ACACB GBP5 SIDT2 CERS1 OSTM1 PDGFD FAM73A VTI1A PEX10 DMXL2 GOLGA1 BPHL MAP1LC3B2 C10orf32 VAC14 CLTA AGK TCIRG1 RAB26 EHD4 POMGNT1 GBP3 ALDH3A2 DBH TMEM231 SSR4 PDGFA YKT6 OCA2 TEKT3 DBNL ITM2C TLR6 APLP2 PEX1 CABP1 RHOBTB3 AP1G1 TMED1 STX5 AGPAT3 GALNT13 CA4 GRIA3 TPCN1 PMEL FCGR1A SVIP TBC1D20 PLEKHM2 ST3GAL4 SLC30A3 CNIH3 GCNT1 SLC6A17 EGF ZFYVE1 WASH3P ANKRD28 SPG20 MBTPS2 TOMM7 HPSE LEPROT HIP1R GORASP1 B3GALNT2 CYB5R1 AP2B1 ANKFY1 SEC24D FAM20B RAB39 TRAPPC10 AP2S1 FUT10 TMF1 TMEM106B FUT4 MFNG GNAQ EMP2 B3GNT7 HLA-E SGIP1 UXS1 TMCO1 ATP6V1D ACPP ZDHHC7 EVC SPNS3 EHD3 MAN2A2 CHMP2A RHO STX18 PICALM PKD1L1 GRB14 CTSZ TPTE2 SLC9A7 PEX6 CHERP HSD3B2 NTRK1 TMEM192 B4GALNT3 PTPRN2 APOB OPN1MW TOMM22 CLVS1 PGAP3 FLOT1 MARCH4 GPER CPT1C USO1 RAP2C PEX2 SLC3A1 GOSR1 RHOT2 GOPC NRGN CD63 NDFIP2 HIP1 BCAP31 MYO19 KIAA0319 ST8SIA3 GBGT1 VAPA CD207 VPS29 ERC1 NAGPA SEC23IP OSBPL1A VAMP2 PTGES2 UBIAD1 MGAT5 VPS45 CUZD1 GOLT1A SUN2 SVOP COASY RAB38 TRIM23 SCYL2 FZD4 SPRED2 ACRBP HS3ST4 FUT2 RHOB TPST2 CHST14 BECN1 DRAM1 GALNT1 CAMK1G GGA3 GABARAPL3 CPT1A HS3ST2 RHBDD2 CLN5 MGAT1 VMA21 NUCB1 TCTN2 ARFGEF2 NOSIP SERPINA1 ARL8B COG7 ANTXR2 CHST11 ANPEP FAR1 TF TSG101 ANXA3 SNN C2orf28 NOS1 SH3GL3 CD1C SPARC PEX3 HS6ST1 B3GALNT1 CHST12 TTYH1 HS3ST3A1 SNX4 DDX3X SYT4 TBC1D5 UBC ARFGAP2 STIM1 JSRP1 KDELR3 UBB MFN1 NUMA1 EMD CLN8 HLA-DRB5 VDAC1 OSBP SEC16B ABCB9 SELP GFAP CAMK2D PCYOX1 KCNH1 A4GALT PSEN2 PEX11G NOTCH3 CHST15 RAB31 ATP2A2 DCT SEC61B LDLRAP1 RNF24 RAB30 KREMEN2 CHGA GOLIM4 IRGM APH1A SNX5 LMBRD1 GOLGA8IP DLG4 ATP6V0A4 DMBT1 ACAP1 SEC23B SURF4 SLC26A7 RSAD2 PNPLA7 STX1A SPPL3 TRIP11 C17orf59 APPL2 AGPS CASK CSGALNACT2 TRDN OTOF MUL1 FNDC3A GALNT7 RIC3 TOMM40L SLC35D2 ROR2 ST3GAL6 GPI ABCC8 GRK4 B3GNT9 CPE SCAMP3 AP3B1 PPP6R3 TOMM34 ATP6AP1 PDCD10 TMEM30A SLC30A5 RAB7A SYT3 RAB36 TAB1 MTX1 MGST1 AQP6 ACER3 MMD RAB10 PIK3C3 TYRP1 NOTCH2 SRPR ARF6 NOS3 MYO18A GRIA4 C9orf11 RPTOR B3GALT2 HLA-DMB CD1D PPP1R15A CAMK2B CNGA3 VTA1 C2orf18 B3GNT6 LPCAT2 QSOX2 FAM73B QTRTD1 PJA2 CD320 RASSF9 CAMK2G LAMP3 TMEM190 WBSCR17 RTN3 RAB15 ATP6V0E1 LGR5 PLA2G4E SH3GL1 CAT SLC9A6 SEMA4C SHANK3 TEX101 B4GALT5 PARM1 GBA2 CLCN6 SLC8A3 TOMM5 CDHR1 HLA-F STX10 RAB34 BET1L ST6GALNAC3 RHOV MICALL1 CYBB GABARAPL2 PLA1A

GO_MICROTUBULE_PLUS_END The growing (plus) end of a microtubule. In vitro, microtubules polymerize more quickly at the plus end than at the minus end. In vivo, microtubule growth occurs only at the plus end, and the plus end switches between periods of growth and shortening, a behavior known as dynamic instability. KIF2C CLIP2 KIF18B MAPRE2 SPAG5 SLAIN2 MAPRE3 MAPRE1 NCKAP5L MYO5A CDK5RAP2 CLIP1 CKAP5 DST CLASP1 C15orf23

GO_AXON_PART A part of an axon, a cell projection of a neuron. PACSIN1 BLOC1S3 MAPK8IP1 ROBO2 DRD2 KCNA1 CHRM2 SYN1 AP1S1 KIF1A UCN AP3S2 CNTN2 SNAPIN NRG1 RAB7A PTCH1 CAD GRIK3 BLOC1S1 AP3B1 GRIK5 KCNK2 SCN8A ILK SRSF10 APP KCNIP3 KIF13B MYOC KCNA6 RAB5A KCNAB2 SRI PENK RANGAP1 NDEL1 CCL2 DLG4 TNFRSF1B PRSS12 SERPINF1 ELK1 KIF5B DAG1 KCNC4 NPFF BOC OPA1 SV2A SCN1B POLG GRIK2 UNC13B APBB1 CCK DTNBP1 SLC18A2 DLG1 ATP6V0D1 TANC1 CHRM1 TPX2 KCNJ11 HCN3 CNTNAP2 AP3M1 CPLX2 GNRH1 SYP PTPRN2 MAG STX3 SLC9A6 TRPV2 NTS SIRT2 GPER RGS10 HIF1A GHRH TH CHRNA7 SEPT5 RAB3A CALCA SYT1 AURKA AP3S1 SLC18A3 CALB1 SLC17A8 SPTBN1 OLFM1 GPM6A OPRK1 PAFAH1B1 NMU ERMN SPAST UCN3 GOT1 DNM1 C1orf130 SYNJ1 NRCAM DGKI PRKCZ HNRNPR SEPT6 ADCYAP1 DPYSL2 FKBP4 SYT7 DNAJC5 KIF1B MBP TULP1 SOD1 C4orf49 STX6 CPLX1 EEA1 KCNQ3 CABP4 DAB2IP KIF3B SPOCK1 TBC1D24 EPB41L3 ADRA2C PDYN P2RX4 PLDN PVALB FMR1 MAPK8IP3 SNCA ADORA1 KCNQ2 AP3M2 LRRK2 SLC32A1 ANK3 AP3D1 HAP1 STXBP1 PNOC TUBB4A P2RX3 PFN2 SLC18A1 KIF4A CDH8 BLOC1S2 NFASC BIN1 UCHL1 SCN2A SPTBN4 COBL AAK1 WDR81 FLRT3 OPHN1 PTPRN SLC1A2 SYNGR1 NEFL NAPA PARD3 KCNH1 RNF40 CAMK2D GLUL OPRD1 VAMP1 SCN1A ANK1 GRIA1 KCNC2 CHRM3 ITGA2 MYO1D NTRK2 OXT CRHBP MUTED GRIN1 CNGB1 TIAM1 KIAA1598 EPHA4 KCNAB1 RAB21 CNTNAP1 CNO DLG2 AP3B2 CALB2 L1CAM RTN4R SPG7 NTSR1 DAGLA AP2M1 KCNA2 CRH

GO_ANCHORED_COMPONENT_OF_PLASMA_MEMBRANE The component of the plasma membrane consisting of the gene products that are tethered to the membrane only by a covalently attached anchor, such as a lipid group, that is embedded in the membrane. Gene products with peptide sequences that are embedded in the membrane are excluded from this grouping. ULBP1 CD14 HYAL2 NTNG1 LYPD3 GGTLC3 ULBP2 FOLR2 FOLR1 CD2 GGTLC2 GGTLC1 MFI2 GPC3 MDGA1 PRSS41 CD160 THY1 GGT2 PRSS8 NTNG2 CD59 RTN4RL2 CD24 EFNA5 GP1BA GGT3P RTN4RL1 GAS1 CPO RHBG TREH NRN1L PKHD1 ULBP3 RGMB GGT7 GGT1 GGT5 PRSS42 CA4 PRSS22 GGT6 GGTA1P EFNA1 GPIHBP1

GO_INTERMEDIATE_FILAMENT_CYTOSKELETON Cytoskeletal structure made from intermediate filaments, typically organized in the cytosol as an extended system that stretches from the nuclear envelope to the plasma membrane. Some intermediate filaments run parallel to the cell surface, while others traverse the cytosol; together they form an internal framework that helps support the shape and resilience of the cell. VMAC MYO5A CCT8 KRT27 CLIP1 KRTAP19-8 NEFH KRTAP10-3 NCKIPSD FAM83H DST KRTAP27-1 KRTAP6-1 SAP30BP KRTAP4-3 SHANK2 KRTAP10-6 KRT36 PRPH ZCCHC17 TACC1 KRT72 KRT6B BFSP2 PJA2 KRTAP13-2 KRT121P NUP35 KRT79 KRTAP1-3 KRT5 KRTAP9-8 KRT9 KRTAP10-12 KRTAP1-5 KRTAP13-1 KRT19 KRTAP5-10 KRTAP13-3 KRTAP15-1 KRTAP10-4 LMNA TRIM29 KRT35 STAG2 STXBP4 KRTAP9-1 KRTAP4-2 KRTAP4-9 KRTAP6-3 ZNF175 KRTAP21-3 RLTPR CLK3 KRTAP5-2 KRTAP3-3 KRT31 KRTAP19-5 KRTAP20-1 MDN1 KRTAP25-1 KRTAP7-1 LOC730755 C17orf70 STUB1 GPER KRTAP4-5 HOXA13 KRTAP12-2 PKP1 CTNS KRTAP20-3 KRTAP19-6 NSFL1C KRT6A KRT12 KRT1 KRT39 LDLRAP1 KRT20 KRTAP6-2 KRTAP5-11 NDEL1 KRT33A IFLTD1 KRT40 KRT32 PKP2 KRTAP10-11 KRTAP10-2 VIM KRTAP4-8 KRTAP10-5 KRTAP19-3 KRT38 SYNM KRT73 KRTAP19-2 KRTAP9-4 OBFC1 KRTAP5-1 MTRR KRTAP9-9 KRTAP1-1 NME2 PNN UPP2 KRT3 KRT23 DES KRTAP21-1 MNS1 KRT78 KRTAP12-1 KRT18 KRT2 KRTAP4-4 NFKBIL1 XRN1 KRTAP20-4 PLEC KRTAP5-6 GJA1 KRTAP19-7 KRTAP1-4 PADI6 BCAS3 NME1 KRTAP10-10 KRTAP19-1 KRT16 CSNK1A1 KRTAP20-2 KRTAP29-1 KRTAP21-2 KRT28 KRT17 KRT10 LMNB1 KRTAP4-6 KRT37 KRT15 EVPL IP6K2 KRTAP5-7 KRT84 KRT77 NEFM KRT14 KRT7 KRTAP16-1 KRT34 KRT82 CASP14 GFAP KRT86 CHD3 KRT71 TCHP PKN2 SMARCA2 KRTAP5-3 SEC62 KRT80 NEFL KRTAP24-1 KRT6C SLC1A4 KRTAP5-5 IFFO1 KRTAP9-3 KRTAP10-1 KRTAP2-4 KRTAP12-3 NFATC4 KRTAP10-8 SYNE2 KRTAP2-1 KRT8 SMG7 KRTAP10-9 KRTAP3-2 BFSP1 KRTAP4-12 KRTAP11-1 KRTAP9-6 PSMD10 KRTAP4-1 KRT33B KRTAP4-11 INA NDOR1 KRTAP5-9 FLG KRT75 IFFO2 KRT76 KRT13 FBF1 KRTAP5-8 KRT85 KRT74 KRTAP23-1 KRTAP26-1 DLGAP2 KRT26 KRTAP9-7 JUP KRTAP13-4 KRT81 NRP1 KRTAP12-4 LMNB2 DSP KRTAP5-4 KRTAP19-4 TLK2 KRTAP22-2 MICAL1 PHLDB2 NARF KRT4 EIF1AD PCDHB4 KRTAP22-1 KRTAP17-1 DDX60 KRTAP8-1 KRT24 KRT25 ERAL1 NES KRTAP10-7 KRT83 KRTAP3-1 SYNC KRT222 KRTAP9-2 MRPS23

GO_APICAL_PART_OF_CELL The region of a polarized cell that forms a tip or is distal to a base. For example, in a polarized epithelial cell, the apical region has an exposed surface and lies opposite to the basal lamina that separates the epithelium from other tissue. GIF SLC4A9 FLOT2 LHFPL5 EPB41L4B BST2 SLC12A2 CYP4F12 LZTS1 DUOX2 DCHS1 NPC1L1 AJAP1 PODXL RAPGEF2 SLC12A3 CIB1 STXBP3 DLL1 VAMP3 SPTBN2 MYO5B ABCA7 AQP5 PLD1 PTPRO RAB17 DYNC2H1 SLC34A1 CDC42 MUC13 TRPM6 SLC17A3 P2RY6 IGSF5 SLC9A3R1 LCT UPK1A MSN NLRP5 CRB3 NF2 GPIHBP1 LRP2 SLC14A2 PRKCI CD36 ERBB3 SLC22A5 SLC3A2 MUC17 GM2A CDHR2 SLC9A1 C5AR1 NOD1 EDA ADCY10 KCNMA1 MFRP SHROOM2 SLC19A1 RAPGEF6 ATP1B2 MUC20 KCNA5 WDPCP C1orf192 LGMN CD34 KNCN EDAR KCNC2 MYO6 MAL2 TCIRG1 DSG1 ADAM17 ATP1B1 ARHGEF18 TCHP STK39 HSP90AA1 VASH1 EGFR GPR116 SLC9A4 TEK IFIT5 SLC26A9 REEP6 CA4 SLC34A2 DSG2 AMN USH1C UMOD STXBP2 SLC6A20 TLR9 MIP SLC5A12 KCNK2 CEACAM1 KISS1 PDZD3 MGAM MST4 CACNB3 GJA1 PDE4D C20orf54 PIP MYO7B MLC1 CRB1 PTK2 UPK3A SLC5A8 SLC7A5 OCLN CTNNB1 SLC23A1 KCNA1 PTH1R AKR1A1 SHROOM4 DPP4 MREG DYNC2LI1 HOMER1 ITPK1 KCNK1 SLC1A1 SLC34A3 SLC2A5 CLCN5 HSP90AB1 HAMP PKHD1 CA2 EMP2 GJB6 DSTYK ATP2B1 SHANK2 ABCB11 SEPT7 CLDN4 NOTCH1 ANXA1 CCDC165 FLOT1 DPEP1 MYO1A CNTFR PRKAA1 ABCB4 STX3 SLC4A2 SLC10A2 P2RY4 SLC22A12 ATP1A1 ATP6V1A MUC1 CD9 SLC26A4 IL6R RAB27A ABCC6 CHL1 C1QTNF5 SLC29A1 C11orf34 EZR ACY3 PDZK1 MPDZ SCNN1G TRPV4 ABCC2 CYBA OOEP BYSL IGFBP2 SORBS2 UPK2 CCDC23 HPN ERBB2 MYL12B ATP2B2 SLC29A4 HOMER2 PROM2 TRPV5 GNAT1 RHCG SLC2A9 UPK1B SLC22A18 PRKG2 SLC22A13 FZD6 RAB27B NOX4 SLC12A1 ANK2 VCAM1 SLC7A9 F2RL2 PROM1 NHS TMEM114 NUMA1 ENPEP BMPR2 RDX GPSM2 SLC2A2 LDLR SLC26A6 PLB1 TMEM235 VAMP7 SLC22A4 VANGL2 SRR TF NEDD1 CSPG4 PSEN2 CTSL2 KCNE4 TNIK SLC25A27 MYO7A SLC22A11 KCNE1 CYP4F2 ATP6V1C1 FOLR1 ANO1 SLC9A3 SLC46A1 EPCAM DDR2 TDGF1 GPR64 CDHR5 DRD3 ADRB2 DVL2 NAALADL1 TUBG1 AQP1 ATP6V0A4 OSMR PARD6B ATP6V1E1 FN1 ATP6V0D2 MAL AQP2 SLC11A2 DUOX1 STC1 AMOTL2 PFKM AQP6 ITGA8 MGST1 SLC5A1 CUBN APP SHROOM3 SLC30A5 TMEM30A EPS15 GNAT3 GPR143 CLDN1 HFE OXTR KL SLC17A4 PLAT GPR77 SLC2A1 OTOG SIPA1L3 S100G SLC23A2 FAT4 PDGFRB CLCA4 SLC39A4 JAG1 PRKCZ ITPR3 ABCB1 ATP8B1 FAP SCNN1A SLC4A5 USH2A AHCYL1 AQP8 CYP4A11 SLC26A3 ACVR1 PTEN CDH2 DRAM2 DAB1 CHRNA7 PARD6A P2RY1 SCNN1B ABCG8 NUMB ABCG5 SI MARVELD2 INADL P2RX2 CFTR KIAA1919 SLC4A7 THY1 FABP1 SLC9A3R2 MTDH TJP1 EXOC1 CD300LG OTOA AP2A1 FZD3 ATP6V1B1 ATP6V0D1 HYAL2 CLIC4 AMOTL1

GO_PLASMA_MEMBRANE_RAFT A membrane raft that is part of the plasma membrane. SDPR EMP2 CDH2 CAV3 ATP2B4 NOS3 CDH15 TRPC4 KCNMA1 SELE NOS1 SMPD2 SCN5A JAK2 FASLG CDH13 CDH1 SLC22A6 IRS1 LRP8 BMPR2 CLN3 BVES RANGRF PRKCDBP ATP1B3 BMPR1A PACSIN2 ATP1A1 CAV2 ATP1B1 SRC LRP6 DLC1 MYO1D MYO1A KIF18A FLOT1 MYOF KCNA5 HDAC6 ATP1A2 F2R INSR CORO1C PRKAR1A KCND2 PTRF SLC6A3 SMO HTR2A LIPE HCK CBL PTGIS FLOT2 PRKAR2A EHD2 EZR CAV1 SPRED1 ADCYAP1R1 SLC2A1 LCP2 NOS1AP SCARB1 HMOX1 CTNNB1 MAPK1 MLC1 P2RY12 PRKACA PTGS2 PLVAP PTCH1 MAPK3 CTNNA1 TGFBR2 EFNA5 LRRK2 AKAP6 LRP4

GO_CD40_RECEPTOR_COMPLEX A protein complex that contains at least CD40 (a cell surface receptor of the tumour necrosis factor receptor (TNFR) superfamily), and other signaling molecules. TRAF3 CHUK RNF31 TRAF5 TRAF2 IKBKB HTRA2 TRAF6 DIABLO CD40 BIRC2

GO_SAGA_TYPE_COMPLEX A histone acetyltransferase complex that acetylates nucleosomal H3 and H2B and is required for the expression of a subset of Pol II-transcribed genes. The budding yeast complex includes the acetyltransferase Gcn5p, several proteins of the Spt and Ada families, and several TBP-associate proteins (TAFs); analogous complexes in other species have analogous compositions, and usually contain homologs of the yeast proteins. CCDC101 TADA2B HCFC1 TADA1 ATXN7 TAF9 TADA2A KAT2A TAF12 TAF5 KAT2B TAF6 FAM48B2 TAF5L TRRAP TAF9B FAM48B1 USP27X USP34 TAF4 ATXN7L3 FAM48A USP51 TAF10 TAF7 USP22 WDR75 SAP130 TADA3 TAF2 TAF6L ENY2 SUPT3H SUPT7L

GO_SOMATODENDRITIC_COMPARTMENT The region of a neuron that includes the cell body (cell soma) and the dendrite, but excludes the axon. ADAM21 TP63 CDC42 GRIN3B ATXN10 PYCARD OR11H4 PPP1CC GLRA1 LMTK3 SPTBN2 KCNB2 ASS1 CPNE5 ANK3 GDPD5 TGFB2 COMT P2RX3 ZWINT RAPGEF2 CHRNA4 HTR2A SUMO1 LZTS1 TRAPPC4 SRD5A1 SPTBN4 MAX DDC BRS3 PTPRN PMM2 DICER1 NPTN PAM VTI1A HSP90AA1 ADC FRMPD4 NELF KCNIP1 GLRA4 PDE9A GRID2 TMPRSS5 EPHB2 SMURF1 KNCN NDUFS7 IGSF9B CAPRIN1 KLHL17 ATP1A2 CRHBP ASAP1 ALS2 CST3 ARHGEF7 OSBP2 ADCY10 VPS16 EPHA4 TMEM100 KIAA1598 MLPH NR1D1 RTN4R AQP11 SAMD4A PIAS3 CIT PDE4B ELOVL5 CDKL5 TRPV1 OR10H5 CNIH3 EIF4A3 ACTN2 DRD2 RARA UCN FARP1 SLC38A2 SLC8A2 ADCY4 GRIK5 ITGA1 PLK2 ITGB1 GRIK3 CPEB1 OR10H3 UHMK1 PDPK1 KISS1 ARF4 NDEL1 CCL2 ARRB2 YKT6 MAP1S SRI FABP7 DBNL ELK1 DRP2 SEMA3A MOB4 FGF13 HTR1D ATXN1L SIAH2 SLC5A7 KCNJ11 NTRK1 CHRM1 OPRM1 PPP1CA TGFB3 ARC APOB FAM5C SYNGAP1 HTR2C GPER CPT1C TRPC5 RGS12 C20orf103 GOPC NRGN RAC3 CHRNA3 KNDC1 S100B OLFM1 GPM6A GNAQ MAGI2 CRTC1 RUFY3 CALB1 CPNE6 GIPC1 SIPA1L1 MYO5A OPRK1 NLGN1 DNM3 GABRA2 GNG13 CYGB DGKI PICALM GNG3 ATP7A STRN LRP8 PHAX DPYSL2 LAMP1 STX4 KCNB1 KCND1 PDE1B SOD1 RBM3 BECN1 PDYN CDK5 DFNB59 RAP1GAP CNR2 OR11H7 NRXN1 GABRA5 UBXN1 SPG11 CACNA1F ARFGEF2 INPP5J OR6T1 TUBB4A LRP4 GNAI2 LRP1 LRRK2 TMEM185A TAOK2 RCVRN NCF1 HTR1E UNC5A OR10H1 PTPRF C19orf20 CYBA BGLAP SLC6A3 MPDZ SLC31A1 COBL CAPN2 MAP1B CAMK2N1 CTTN GRIN3A NRSN2 EPHA5 SLC4A8 INPP5F FAM5B CNIH2 GRM1 IFNGR1 GLUL CAMK2D SORT1 KCNH1 SLC25A27 HTR1A PSEN2 SOS1 FBXO31 CANX AGRP KLHL14 MINK1 ANXA3 KCNAB1 AGFG1 KLC1 NOS1 NCDN EIF2C2 TRPM4 EFNA2 ADA GRIP1 BMPR2 SYT4 KCNA2 PPARGC1A UBB STRN3 NUMA1 SGCE PAK1 KLHL20 GRK4 FRMD7 CPE LYNX1 NLGN4X OR10H2 PCSK2 PTCH1 NLRP1 TUBB3 NGDN HCFC1 HTR1F ILK DHODH ENO2 DBN1 GRM7 NPFF PDE10A SEZ6 PRSS12 TNFRSF1B TIMP2 DLG4 MAF1 RPS6 MPL OPA1 FBXO2 ARHGAP44 SNAP47 TBX21 TTLL7 MUL1 KCND3 RIC3 S100A5 ADCY9 APBB1 CCK CDK5R1 HCN3 ABL1 TANC1 SLC12A5 PSD2 PMM1 SLC9A6 SHANK3 BMPR1A SLC8A3 NDN CYP17A1 THY1 CCR2 OR10J5 PRKCG PPP5C SEPT11 ASTN2 AVP CYBB P2RY1 SHANK1 GLRB CHRNA10 GRIA4 SNPH RPTOR SMN1 JPH4 LPAR1 CNGA3 PAFAH1B1 PTK2B ADNP APOD PLK3 PRKCZ ITPR3 SKOR1 ACCN1 NEURL ARHGEF15 ARHGEF2 CPLX1 PSEN1 MAPK1 DVL1 NAIP CNN3 MBP EPHA7 LSM1 SLC4A10 GLRX3 ATP13A2 DAB2IP PCDH8 RAB17 BRD1 PTPRO MAPK8IP3 ACVRL1 BMPR1B ATP1A3 IGF2BP1 MYL7 P2RX4 RBM8A PNOC GLRX5 TRPM2 PLCB4 OR10H4 CIB1 ADORA1 ARG1 SEZ6L AMIGO1 GNAZ ELFN1 CTTNBP2 DPYSL5 HTT LRFN3 HTR5A UCHL1 SRCIN1 GNB1 TGFB1 GAL RPLP0 LAMA2 PARD3 KCNN3 HCN1 GNAO1 OR10J6P ANG STMN2 MYO1D SRD5A2 NF1 PTPRK SLC38A7 KCNC2 CPEB3 DDN LRP6 ANKS1B IFT20 MME PPP1R9B KCNC1 SLC1A4 RPL28 TOP1 SYT5 KIF5A GPHN CNKSR2 IFT57 SLC8A1 ZNF385A GNB3 STAT1 BSN ATCAY CHRM2 BPTF KCNA1 TRPM5 GIP NEUROG1 MAPK8IP1 HTR2B CNTN2 PDE11A MTOR LRIT3 SYN1 CSNK1E CAD SHARPIN KCNJ14 NRG1 PDE1A KCNIP3 AKAP9 ENC1 GABRB1 KCNK2 RANGAP1 PENK CTNND2 RAB5A SERPINF1 WLS CNTNAP4 TAC1 INHA CPEB4 SRGAP2 OMP FXR1 SST EPHB3 HPCA SARM1 ODZ2 NOV DTNBP1 GABARAPL1 GRIK2 CHL1 RAB27A ARRB1 EEF1A2 OR5T3 ENDOG C4A KPNA1 GTF2I CX3CR1 STX3 SLC1A3 TMPRSS3 EPHB1 ITPKA DNER CPLX2 RGS10 RIN1 GRM6 STRN4 KCNN2 RAB8A STAU1 TH ATP2B1 SHANK2 HTR7 ZMYND8 AMFR ERMN GNB2L1 RBFOX3 GRM3 MAPK8IP2 ACAD9 CRIPT HNRNPR PPT1 KCNK1 SACS C19orf2 PLXDC1 DENND1A KCNJ2 KCNE3 SYNPO IFT52 PI4K2A MTMR2 NRP1 KATNB1 KLHL1 FMR1 PVALB SNX14 IAPP FEZ1 SLC32A1 GNAT1 HOMER2 ATP2B2 TACR3 ZFYVE27 HPN ZDHHC5 LRRC4 PREX1 GCHFR CCR4 ZNF259 FUS ASCL1 BAIAP2 MCRS1 GRID2IP OR5T2 OPHN1 KCNN1 NSF ERO1L SCN1A AIF1 HTR6 DLG3 MAPT IL1RAPL1 CTSL2 CHRM3 GRIA1 RGS14 C4B HDAC6 STAR VTI1B TIAM1 CLU GRIN1 SRR GNAS NCS1 ARHGAP32 GSK3B RGS8 MAGEE1 CRH GRM2 PALMD CASP5 RRM1 TRIM9 MAGOHB BNIP3 PURA NTSR1 NRSN1 SNCG KIRREL CTNND1 FLNA APP ASTN1 SRSF10 POLR2M ITGA8 NQO1 KCND2 EFHC1 KLHL24 PTBP2 IFNG MT3 ALCAM FLRT1 CACNA1A KIRREL3 PRKAR2B TPX2 SYNDIG1 HTR1B CACNA1B SMN2 GABBR1 CRYAB FZD3 IL6ST GNRH1 DBC1 CNTNAP2 ACSL4 HTR3A SIRT2 SNCAIP TXN2 PPP1R9A PPP1R1B AURKA CALCA FAM206A RET DAB1 GLRX2 IGSF9 SLC17A8 P2RX7 ADCY2 RELN PTEN GOT2 GLRA3 SYT11 MYH10 WFS1 APOE NGB FKBP4 PALM MAP2K4

GO_PROTON_TRANSPORTING_V_TYPE_ATPASE_COMPLEX A proton-transporting two-sector ATPase complex that couples ATP hydrolysis to the transport of protons across a concentration gradient. The resulting transmembrane electrochemical potential of H+ is used to drive a variety of (i) secondary active transport systems via H+-dependent symporters and antiporters and (ii) channel-mediated transport systems. The complex comprises a membrane sector (V0) that carries out proton transport and a cytoplasmic compartment sector (V1) that catalyzes ATP hydrolysis. V-type ATPases are found in the membranes of organelles such as vacuoles, endosomes, and lysosomes, and in the plasma membrane. ATP6V1B1 ATP6V0D1 ATP6V0E1 ATP6V1G3 ATP6V1H ATP6V1B2 TCIRG1 ATP6V1F ATP6V1C1 ATP6V0D2 ATP6V1D ATP6V1A ATP6V0B ATP6V1G1 ATP6AP1L ATP6V1C2 ATP6AP1 ATP6V1G2 ATP6V0A4 ATP6V0A1 ATP6V0A2 ATP6V0C ATP6V0E2

GO_SECRETORY_GRANULE A small subcellular vesicle, surrounded by a membrane, that is formed from the Golgi apparatus and contains a highly concentrated protein destined for secretion. Secretory granules move towards the periphery of the cell and upon stimulation, their membranes fuse with the cell membrane, and their protein load is exteriorized. Processing of the contained protein may take place in secretory granules. C9orf11 HPS4 SEPP1 C7orf68 TOR1A CPA3 SYT7 ITGB3 FAM3C SCAMP1 IL1B ITPR3 RPH3AL DEFA4 FIGF TEX101 STXBP5L RNPEP FGB LAMP2 ISLR SPINK8 ALDOA KLK7 SERPINE2 TMEM190 SUN1 LAMP3 PLA1A VAMP8 SCG2 AZU1 CLK3 DENND4C AVP SLC2A4 PDGFB ZP3 VEGFC GNAI3 SPAG8 HRG FN1 SPINK2 SERPINA5 ZPBP2 SPACA3 AKAP3 ABHD2 A2M IGF2 CHGA CAPZA3 FNDC3A SLIRP TRIP11 STX1A ITPR1 NCF2 RAB11FIP5 DMBT1 HEXB AZI1 TRIM36 CPE IQCF1 CDC37L1 PLAT LACRT BSG RAB10 HSPD1 TBXA2R SPINK1 STXBP5 VEZT APP GCG GAS6 SNAPIN RAB7A CFD ACR RAB13 SLC30A5 PCSK2 GNAT3 CRISP3 MMRN1 VAMP7 CKAP4 ANXA3 CLU TF TSSK1B MORN2 SERPINA3 MYRIP RAB3B THBS2 SPACA4 SYT9 RPH3A CD46 SYT4 ICA1 SNAP23 LEFTY2 TSKS SPINK5 NCS1 SPARC CRCP SELP PRSS57 RAB4A OXT MPO DEFA3 SV2B CTSL2 CALR CYBA SFTPD ABCC4 SPP2 APOA1 TMEM225 LRGUK SYTL4 ELANE CUZD1 EXOC3 BAIAP2 NAPSA AHSG VAMP2 CATSPER4 ATP8B3 RARRES2 SCG5 GHRHR RAB27B SYT2 ACRBP SOD1 STX4 TMED2 ABCA3 STK31 EBAG9 FSTL3 LAMP1 EXOC3L1 NPPC PATE4 SFTPA1 DEFA1B SERPINA1 TIMP3 COL1A1 CASC5 RND2 ASTL SERPINF2 ABCA12 F13A1 MYO5A QSOX1 CXADR TBC1D21 PLA2G1B SCG3 LGALS3BP ATP7A ACRV1 VPS33B SCGB1A1 LTF TCP1 ARC PTPRN2 TGFB3 FAM170B CAV2 STX3 PCSK5 BRCA2 CD9 SPACA7 ALB ECE1 IGF1 RAB27A SPESP1 RAB3A ANXA7 SYT1 CD63 ACTN4 NOTCH1 DEFA1 SKIL SERPING1 CTSG EDN1 PCSK1 CLEC3B F5 ADAM8 STXBP2 APLP2 CAPN11 HCRT SFTPC SRI TEKT3 TMEM184A PLA2G2A ITIH3 ZP1 KNG1 CTSW DLD SPACA1 KLK5 KLK13 PCSK1N CA4 FSTL4 THBS1 TEX22 C19orf26 SYCN GIP DRD2 VEGFB EGF ACTN2 ATP6V1E2 STX7 SYT8 ZG16 BMF SFTPB DEFA5 SERPINE1 ORM2 CYB5R1 TMED10 ITGA1 HEXA CAMP HGF TMSB4X ADAM15 ANXA11 SYT5 C9orf9 IZUMO1 VPS13A SPAG9 SLC11A1 PPFIA3 VEGFA CD36 ATP8A1 ITGA2B F8 CATSPER3 GARS UNC13D SYPL1 TSSK2 A1BG VAMP1 CYLC1 FABP9 PAM RAB3D INS PECAM1 PDGFA ZP2 LOXL1 CRHBP SERPINA4 DBH ACTN1 RAB26 SLC30A8 TCTEX1D4 TRH ANGPTL6 PCDH7 RAB4B PLG PF4 FGA TIMP1 TGFB2 FGG ITPR2 PCSK4 CLCA1 SMPD1 NUDT1 CLCN3 FLOT2 SFTA3 TGFB1 GAL ECM1 PTPRN CTSH APOH SFTPA2 ORM1 TXNDC8 POMC ENKUR RACGAP1 ITIH4 IQUB CDC42 PPBP SPINK13 CAV1 PROS1 CHGB OLFM4 KIT SNCA STXBP3 PHACTR2 ATP6V0A2 SRGN VAMP3 STXBP1 GHRL VWF TMX3 CARTPT POMT1 CTNNA1

GO_BASEMENT_MEMBRANE A thin layer of dense material found in various animal tissues interposed between the cells and the adjacent connective tissue. It consists of the basal lamina plus an associated layer of reticulin fibers. CD151 ANG EFEMP2 HMCN1 LOXL2 MATN2 LAMA4 HSPG2 VTN COL4A6 COLQ COL18A1 LOXL1 ANXA2 LAMA5 ERBB2IP TINAG COL4A4 FREM2 FREM1 FBN1 SMOC1 RELL2 USH2A PTN DST AGRN CST3 HMCN2 THBS2 LAMA1 LAMC2 LAMC1 TNC ENTPD2 APLP1 COL4A5 NTN1 CCDC80 LAMB2 NID1 COL17A1 FGF9 COL28A1 MEGF9 NPNT SPARC COL7A1 COL4A2 SMC3 LEPREL1 ACHE NID2 COL4A1 LAMC3 ANXA2P2 LAMB3 FRAS1 MMRN2 TGFBI EFNA5 EGFL6 LAMB4 TIMP3 FBLN1 ADAMTS1 EGFLAM LAMB1 COL15A1 VWA2 SPN COL5A1 FN1 COL4A3 DAG1 LAD1 SERPINF1 VWA1 TIMP1 AMTN COL8A1 COL8A2 VWC2 SMOC2 LAMA3 ITGA6 NTN4 LAMA2 FREM3 COL2A1 DLG1 CASK THBS4

GO_AP_TYPE_MEMBRANE_COAT_ADAPTOR_COMPLEX Any of several heterotetrameric complexes that link clathrin (or another coat-forming molecule, as hypothesized for AP-3 and AP-4) to a membrane surface; they are found on coated pits and coated vesicles, and mediate sorting of cargo proteins into vesicles. Each AP complex contains two large (a beta and one of either an alpha, gamma, delta, or epsilon) subunits (110-130 kDa), a medium (mu) subunit (approximately 50 kDa), and a small (sigma) subunit (15-20 kDa). AP1S1 AP1S2 AP3M1 DKFZp761E198 AP3S2 AP2A1 AP1G2 EGFR AP1M1 AP3M2 AP2S1 SLC18A3 AP3S1 KIAA0415 AP3D1 AP3B1 GGA3 EPS15 AP1M2 AP2B1 C20orf29 VPS41 SGIP1 LDLRAP1 VPS18 AP4M1 VPS33A GGA1 AP1B1 AP4B1 AP2M1 VPS33B MUDENG TBC1D5 VPS39 PICALM AFTPH AP3B2 AP1G1 GGA2 AP2A2 SYNRG

GO_PHOSPHATASE_COMPLEX A protein complex which is capable of phosphatase activity. WDR82 PPP2R5C ITPR1 PPP3CB SHOC2 PPP1R2P3 PPP4C PPP1R2 STRN3 STRN PPP2CB TMEM188 PPP2R5A PPP2R5E PPP2R5D PPP2R2B NCK1 PPP1CB NKD1 PPP1R15A SMEK2 PPP4R4 PPP4R1 PPP2R2D CTDNEP1 PPP1R11 TOX4 PPP1R12A PPP1R10 PPP1CC PPP4R2 STRN4 PPP2R3B CYCS PPP2R2A PPP3R1 PPP3CA PPP1R2P9 PPP1R9B PPP2R4 PPP2R1A PPP2CA PPP1R2P1 PPP1R3B PPP2R5B PPP1R15B PPP1CA PPP2R2C PPP2R3A

GO_M_BAND The midline of aligned thick filaments in a sarcomere; location of specific proteins that link thick filaments. Depending on muscle type the M band consists of different numbers of M lines. ANK1 LMOD3 CMYA5 SMPX KBTBD10 ENO1 ALDOA ANK2 MYOM1 S100A1 SPTBN1 PPP2R5A LMOD2 MYOM2 TRIM63 SMTNL1 NBR1 OBSL1 CRYAB FHL2 MYOM3 OBSCN

GO_POLYSOME A multiribosomal structure representing a linear array of ribosomes held together by messenger RNA. They represent the active complexes in cellular protein synthesis and are able to incorporate amino acids into polypeptides both in vivo and in vitro. EIF2C1 RPS4X CSDA NR0B1 DIS3L2 FXR1 FUS MCRS1 RPS6 MSI1 NUFIP2 RPL10L RPS4Y1 EIF2C2 UNK EEF2 EIF4B VBP1 FXR2 LSMD1 MSI2 EIF4H NAA30 ATXN2 PIWIL1 GCN1L1 NAA35 RPL7A RPS3 FMR1 PSMA1 CALR IMPACT PSMA6 DRG1 PIWIL2 LARP4B EIF2AK4 EIF2S1

GO_MICROBODY_PART Any constituent part of a microbody, a cytoplasmic organelle, spherical or oval in shape, that is bounded by a single membrane and contains oxidative enzymes, especially those utilizing hydrogen peroxide (H2O2). ATAD1 AMACR PIPOX AGXT PEX7 LONP2 PEX5L PAOX CAT ACOX2 PEX16 ABCD1 ACOX1 PEX10 EHHADH PEX6 MPV17L NUDT19 FAR2 ACSL6 ACOXL ACSL3 CRAT ABCD4 ARF1 DDO ECI2 HSD17B4 IDH1 PEX2 PEX13 FABP1 ALDH3A2 PEX11G ACSL4 PHYH HAO1 PEX11A SCP2 IMPDH2 ABCD3 PEX26 FAR1 MAP2K2 PECR NUDT12 FIS1 SYT7 TTC1 ACAA1 RAB8B PNPLA8 GRHPR PEX3 PEX12 CRYM BAAT PEX19 DHRS4 HACL1 ABCD2 ACSL1 HSPD1 ACOT8 TMEM35 HAO2 MGST1 PEX11B FNDC5 PXMP2 SLC27A2 MLYCD DAO PEX5 NUDT7 ACBD5 ACOT6 GNPAT PEX1 HMGCR MAVS CROT AGPS ACOT4 DECR2 PXMP4 IDE PEX14 SLC25A17 ACOX3

GO_RESPIRATORY_CHAIN_COMPLEX_IV A part of the respiratory chain, containing the 13 polypeptide subunits of cytochrome c oxidase, including cytochrome a and cytochrome a3. Catalyzes the oxidation of reduced cytochrome c by dioxygen (O2). NDUFA4 COX7B2 COX4I2 COX5B COX6A1 COX6A2 COX5A COX7B C15orf48 COX8A COX4I1 COX8C NDUFA4L2

GO_ORGANELLAR_LARGE_RIBOSOMAL_SUBUNIT The larger of the two subunits of an organellar ribosome. Two sites on the ribosomal large subunit are involved in translation: the aminoacyl site (A site) and peptidyl site (P site). MRPL19 MRPL13 MRPL2 MRPL51 MRPL17 MRPL41 MRPL23 ICT1 MRPL21 MRPL43 MRPL36 MRPL27 MRPL3 NSUN4 MRPL28 NSUN3 MRPL16 C12orf65 MRPL33 MRPL12 MRPL52 MPV17L2 MRPL32 MRPL55 MRPL47 MRPL22 MRPL11 MTERFD2 MRPL46 MRPL15 MRPL49 MRPL10

GO_EXOCYTIC_VESICLE_MEMBRANE The lipid bilayer surrounding an exocytic vesicle SYNGR1 SYN3 ABCC8 DMXL2 SLC6A17 DNM1L SCAMP5 DRD2 SYPL1 SYP PTPRN2 SYN2 TMEM163 SYN1 VAMP1 SYT2 PI4K2A SYNPR BCL2L1 SEMA4C C19orf26 SYNGR4 SNAPIN AMPH SLC32A1 RAB11B LRRK2 SV2B SLC18A3 SYT1 SYT5 SLC17A8 SLC17A5 SYNGR3 SYT12 SLC18A1 SLC17A6 SV2C SYT11 GABRA2 GAD2 ZNRF1 SYT6 SYPL2 STX1A SYNGR2 VAMP2 SV2A SLC17A7 ATP6V1G2 DTNBP1 SYT7 SVOP SLC30A3 ICA1 DOC2A SCAMP1 SYT4 SLC18A2 SYT9 OTOF RPH3A

GO_SMN_SM_PROTEIN_COMPLEX A protein complex formed by the association of several methylated Sm proteins with the SMN complex; the latter contains the survival motor neuron (SMN) protein and at least eight additional integral components, including the Gemin2-8 and unrip proteins; additional proteins, including galectin-1 and galectin-3, are also found in the SMN-SM complex. The SMN-Sm complex is involved in spliceosomal snRNP assembly in the cytoplasm. GEMIN5 SNRPG GEMIN8 GEMIN7 GEMIN4 GEMIN2 GEMIN6 STRAP SNRPD2 SMN2 SNRPD1 DDX20 SNRPF SNRPE SMN1 SNRPD3 SNRPB

GO_VESICLE_LUMEN The volume enclosed by the membrane or protein that forms a vesicle. SPACA7 FGB ISLR ALB ALDOA HSP90AA1 IGF1 INS EGFR APOB FIGF TGFB3 PRSS57 A1BG SERPINA4 DBH SERPING1 ACTN1 CALR HRG BACE1 PDGFA DEFA3 PDGFB ACTN4 VEGFC DEFA1 HBB CLU TF QSOX1 SERPINA3 SEPP1 HGF F13A1 MMRN1 TMSB4X FASLG ADA LEFTY2 SAA1 F8 SCGB3A2 SPARC LTF HSP90B1 SCG3 LGALS3BP FAM3C VEGFA APOE PROS1 HSPH1 GIP EGF VEGFB ACTN2 THBS1 ITIH4 CDC37L1 RARRES2 PPBP GAS6 ZG16 VWF GCG CFD DEFA5 SERPINE1 ORM2 PCSK2 HYOU1 SERPINF2 SRGN DEFA1B APP TIMP3 SERPINA1 GHRL IGF2 FGA HBA2 PF4 APOA1 TIMP1 HBA1 TGFB2 FGG HP FN1 PCSK1 HPX CLEC3B F5 SPP2 PLG A2M ORM1 POMC AHSG ITIH3 KNG1 ECM1 TGFB1 APOH CTSW

GO_NUCLEAR_BODY Extra-nucleolar nuclear domains usually visualized by confocal microscopy and fluorescent antibodies to specific proteins. TFIP11 ISG20 PDX1 RFWD3 LUC7L3 LUC7L2 SMNDC1 ALKBH5 BLM SMN1 NXF1 PTEN PSKH1 FRG1 DDX42 CPSF6 ZBTB1 RBM39 FAM118B NUDT21 PLRG1 SF3A3 COIL LMNA NRIP1 TP53INP1 ACIN1 CBX5 SMN2 NPAT THOC1 HINFP SF3B1 CDK13 PPP1R8 ATR MLIP FAM206A NACC1 CLK3 SRRM1 EAF1 RBM4 WT1 TIMM50 SMC6 GLI2 MTDH SRSF6 GAR1 PIAS4 ZMYM2 PHF5A CDK2 SP100 SON PPIH CREBBP EAF2 APBB1 UBN1 WAC TP53 PYHIN1 RBM11 TERT NSRP1 RCHY1 ZCCHC18 RPAIN EIF4ENIF1 OGG1 CBLL1 ZNF473 WDFY3 RNF6 EPAS1 KLHL20 PNN SRSF10 THOC4 PRPF4 SKI GATAD2A RNF4 RDM1 RPA1 SRSF2 ZC3H14 ZC3H13 PCGF2 RNPS1 CSTF2 C8orf4 HR POU4F2 ZC3H8 RB1 NHP2 ZNF830 NOLC1 HNRNPM PPIG PPARGC1A MAPK7 TCF7L2 DKC1 PARK7 MEF2C DDX3X ELF4 ARL6IP4 NSMCE2 ATPAF2 TRIM27 KAT6A SART1 SLU7 PATL1 USPL1 RREB1 N4BP1 HIPK3 FAM76B C4orf44 SETD1B AGGF1 LSG1 KIAA1429 ATRX GLI3 SP3 MAML3 DYRK1A GEMIN2 RGS14 TOLLIP MAGOH TRIM22 DDX20 U2AF1L4 APEX1 SS18L1 MORC3 MAGEA2 SF3A2 RAD51 SNRNP70 NONO RFWD2 PRPF40B GATAD2B PRPF31 PRPF18 HSPB7 ZNF259 SENP2 C1orf124 SHQ1 METTL3 PIAS2 DDX46 GPATCH2 TDG SRSF4 PSPC1 HIRA SUMO2 NXT1 GEMIN7 LSM10 MRE11A ATOH8 FMR1 MAML2 YLPM1 PIN1 SARNP CTR9 PNISR CHEK2 SATB1 THAP1 THOC2 PHAX YTHDC1 PML NACC2 SQSTM1 EFTUD2 ZNF638 C8orf80 PIP5K1A SETD1A SRSF1 NARG2 ELL USP7 CCNL1 UBE2I GFI1 EP400 TUT1 XPO1 HIF1A GEMIN4 NCOR2 OIP5 SRSF5 BCLAF1 RBM27 PIAS1 CIR1 RNF2 DDX1 TDP2 ATXN2L SRP54 MLL5 MDM2 ODZ2 SPOP POLDIP3 NFE2 SRY NOP10 THRAP3 NOC3L CXXC1 PRKACA MTOR ZNF496 NBN EIF4A3 FTO UBOX5 RING1 RPA2 ODZ1 CLK2 ARNTL TGS1 GEMIN6 DDX39B PABPN1 PRPF40A SRRM2 TOP3A C5orf41 RSRC1 PSME4 DUSP11 C1orf51 HIPK1 AKAP17A HIF3A AFF2 CSNK1A1 AKAP8L THOC7 CHFR CCNL2 SUMO3 PIAS3 TOPORS GEMIN8 SART3 SIRT1 EIF3E WBP4 THOC3 IKBKE RBM25 THAP7 MEOX2 PRPF3 THOC6 SCNM1 SP140 DAPK3 MKNK2 SFPQ RBM15 CASC3 LRCH4 SAP18 ANKS1B TARDBP DGKQ CBX4 ANKRD2 TRIM8 GEMIN5 RNF34 TOE1 TP53INP2 FBLL1 CDK9 IFI16 CHTOP ZBTB16 SUMO1 ZFHX3 CDC5L ZCCHC12 ZMIZ1 NR2C1 PRPF19 MAX TRIM16 ZNF451 WTAP MAML1 WRAP53 MAGEA2B SPTBN4 MECOM GLIS2 TRIM69 ANGEL2 CBY1 CASP8AP2 NOP58 PRPF6 SNRPC CWC22 U2AF2 HIPK2 KIAA0947 PRPF8 SMC5 CIITA TOPBP1 DAXX FYTTD1 RBM8A WBP11 NEK6 CRNKL1 CDK12 PPP1CC PQBP1 FBL SRSF3 MBD1 CD2BP2 PLCB1 BASP1

GO_CLATHRIN_COATED_VESICLE A vesicle with a coat formed of clathrin connected to the membrane via one of the clathrin adaptor complexes. SCAMP1 KIAA1199 MALL VPS33B ATP7A DNAJC5 CLTCL1 RASSF9 DAB2 CD74 PICALM CTLA4 VPS41 NECAP1 VPS18 WNT5A MYO1E SNX3 SGIP1 CPNE6 SH3GL2 HLA-DRB3 GOPC SLC18A3 SYT1 NRGN HIP1 RAB8A RAB3A BCAP31 ASTN2 SLC2A4 NUMB HLA-DQA2 PIK3C2A HSPA8 CLVS1 AP2A1 ABCB4 APOB AP1S3 FZD2 AP1S2 STON2 DENND1B RGS19 RAB27A ECE1 GPR107 CD9 FCGR1A SLC18A2 DVL2 VPS33A AP1G1 GGA2 SLC17A7 LMBRD1 DNM2 CLTB TMED9 EDN1 LDLRAP1 DBNL SFTPC ASTN1 STEAP2 RAB35 AP2S1 NECAP2 FCHO1 TYRP1 TMED10 EPS15 AP1M2 AP3B1 AP2B1 WIPI1 FZD5 SFTPB HBEGF HIP1R MLC1 AP1S1 ROR2 SH3BP4 EPN2 SPG21 VPS11 TBC1D5 AP2M1 AP2A2 SYNRG AFTPH AP3B2 FAM109B VPS16 SNX18 LDLR CLINT1 EPN3 MYO6 OCRL CLTA FURIN FOLR1 SORT1 UNC13D IGF2R FCHO2 EGFR HLA-DQA1 HLA-DRB5 VTI1A NCALD SFTPA2 HLA-DRB1 SFTA3 SNAP91 INPP5F AP1B1 CLVS2 AAK1 VAMP2 HLA-DPA1 HLA-DQB1 SNX9 TGOLN2 TNK2 DENND1C HLA-DRA SFTPD GAD2 CD207 SLC18A1 HLA-DRB4 FCGR1B HLA-DQB2 VAMP3 CLTC SFTPA1 SLC32A1 LRP1 FAM109A VWF RAB27B HLA-DPB1 RAB14 STX6 GAD1 SCYL2 AP1M1 DENND1A FZD4 AP1G2 DVL1

GO_INTRINSIC_COMPONENT_OF_THE_CYTOPLASMIC_SIDE_OF_THE_PLASMA_MEMBRANE The component of a plasma membrane consisting of gene products and protein complexes that have some covalently attached part (e.g. peptide sequence or GPI anchor) which is embedded in the cytoplasmic side of the plasma membrane only. RASA2 RASAL1 SPTA1 RASA4 RASA4B RASA3 RASAL3 DAB2IP NPHS2 MIEN1 RASA1 SYNGAP1 SPTB NF1 RASAL2

GO_MAST_CELL_GRANULE Coarse, bluish-black staining cytoplasmic granules, bounded by a plasma membrane and found in mast cells and basophils. Contents include histamine, heparin, chondroitin sulfates, chymase and tryptase. SNAP23 RASGRP1 PIK3CG LYN MRGPRX2 LAT2 SRGN ANXA1 MILR1 BTK KIT CHGA S100A13 LAT CPLX2 CXCR2 PLA2G3 NPPA AKR1B1 PIK3CD NR4A3

GO_PSEUDOPODIUM A temporary protrusion or retractile process of a cell, associated with flowing movements of the protoplasm, and serving for locomotion and feeding. LDB3 ACTN3 ARRB1 F2RL1 MAPK1 ACTN2 VAMP7 MAPK3 CAPN2 ACTN1 KBTBD10 CNP MSN MYOZ1 RAF1 RAB25 ACTN4

GO_AXON_INITIAL_SEGMENT Portion of the axon proximal to the neuronal cell body, at the level of the axon hillock. The action potentials that propagate along the axon are generated at the level of this initial segment. SCN8A CAMK2D KCNQ2 KCNQ3 CCK BIN1 SCN1A ANK3 NFASC SPTBN4 NRCAM

GO_CELL_DIVISION_SITE The eventual plane of cell division (also known as cell cleavage or cytokinesis) in a dividing cell. In Eukaryotes, the cleavage apparatus, composed of septin structures and the actomyosin contractile ring, forms along this plane, and the mitotic, or meiotic, spindle is aligned perpendicular to the division plane. In bacteria, the cell division site is generally located at mid-cell and is the site at which the cytoskeletal structure, the Z-ring, assembles. RAB11A ITGB1 CEP55 MAEA PPP1CC NDE1 RHOA MZT1 PLK4 MYH2 RHOC ECT2 SEPT7 PLEKHG6 TUBGCP2 TUBGCP3 RAB11FIP4 TUBGCP6 MYLK PKN2 RHOB PSTPIP1 WDR73 TUBGCP4 STAMBP LIMA1 RACGAP1 SEPT6 RALA SEPT2 ZFYVE19 RDX SPIRE2 SEPT12 PKN1 NF2 MEN1 SVIL PLCD3 PITPNM1 ARF6 MYH9 MASTL OR2A4 PDXP ANLN RAB21 SPIRE1 FSD1 TUBGCP5 DCTN3 RAB11FIP3 SSH1 MYH10

GO_U4_U6_X_U5_TRI_SNRNP_COMPLEX A ribonucleoprotein complex formed by the association of the U4/U6 and U5 small nuclear ribonucleoproteins. PPIH ZMAT2 SNRPB TXNL4A NHP2L1 SNRPE LSM6 PRPF3 PRPF6 SNRPD2 SART1 NAA38 TXNL4B LSM3 CD2BP2 SNRPN PRPF31 LSM5 LSM2 PRPF18 PRPF4 SART3

GO_ENDOPLASMIC_RETICULUM_SUBCOMPARTMENT A distinct region of the endoplasmic reticulum REEP5 PARP16 FKBP1A RAB3GAP1 ATL3 KIAA1715 REEP1 RAB10 CASQ1 ARV1 STIM1 RAB18 ATL1 ASPH ZFYVE27 TMEM110 RYR1 RTN2 KPNB1

GO_PHOTORECEPTOR_DISC_MEMBRANE Ovally-shaped membranous stack located inside the photoreceptor outer segment, and containing densely packed molecules of the photoreceptor protein rhodopsin that traverse the lipid bilayer. Disc membranes are apparently derived from the plasma membrane in the region of the cilium that connects the photoreceptor outer segment to the inner segment. GUCA1A GRK1 RGS9 GUCY2D GRK4 GUCA1C PDE6A GUCY2F PDE6B PDE6G GNB1 SPTBN5 GNAT1 ABCA4 GNGT1 GRK7 GUCA1B RHO

GO_PROTEASOME_CORE_COMPLEX A multisubunit barrel shaped endoprotease complex, which is the core of the proteasome complex. PSMA8 PSMB8 PSMA3 PSMA5 PSMF1 PSMA1 PSMB11 PSMB6 PSMB4 PSMA7 PSMB1 PSMB10 PSMA4 PSMA6 PSMB9 PSMB5 PSMB2 PSMB3 PSMB7 PSMA2

GO_PHOTORECEPTOR_CONNECTING_CILIUM A nonmotile primary cilium that has a 9+0 microtubule array and forms the portion of the axoneme traversing the boundary between the photoreceptor inner and outer segments. USH2A RP1L1 CEP290 CETN2 TBCC FAM161A TOPORS PCDHB15 CETN1 IQCB1 SEPT2 IFT57 BBS4 SPATA7 C21orf2 USH1G MYO7A NPHP4 IFT140 IFT52 KIFAP3 RPGRIP1 IFT122 WDR19 ARL3 NPHP1 MAK GNAT1 IFT20 SPTBN5 KIF17 RP1

GO_PRESPLICEOSOME A spliceosomal complex that is formed by association of the 5' splice site and the branch point sequence with specific snRNPs. The prespliceosome includes many proteins in addition to those found in the bound snRNPs. Commitment to a given pair of 5' and 3' splice sites occurs at the time of prespliceosome formation. Prespliceosome complexes are not active for splicing, but are instead an early step in the assembly of a spliceosomal complex. SNRPN PRPF40A SF3A1 CRNKL1 SYF2 LUC7L SNRPG LSM7 XAB2 PRPF40B SF3B1 SNRNP70 SNRPD3 SNRPB SF3A2 LUC7L2 U2AF2 SNRPC PRPF39 LUC7L3 SNRPD1

GO_BLOOD_MICROPARTICLE A phospholipid microvesicle that is derived from any of several cell types, such as platelets, blood cells, endothelial cells, or others, and contains membrane receptors as well as other proteins characteristic of the parental cell. Microparticles are heterogeneous in size, and are characterized as microvesicles free of nucleic acids. AFM IGHG3 ITIH4 PFN1 PSMC5 PROS1 ACTB YWHAZ C9 PON1 SERPINF2 C1QB C8A APOA2 CLIC1 PLG HPX SERPINC1 IGHD C8G IGKV4-1 HP HBG2 FGG DNPEP CPSF3L HBA1 APOA1 FGA HBA2 ANGPTL4 IGHA1 TGFB1 ZNF559-ZNF177 AHSG IGJ SLC4A1 IGHG2 PZP ORM1 A1BG FCN3 IGHV4OR15-8 C1S C1R C4BPA IGKV3D-11 IGLC2 HBB ACTA1 C4B GSN IGHA2 HBD F2 IGHV1OR21-1 CFHR1 ACTG1 ITIH2 EIF2A IGHM CP APCS C3 IGKV1D-33 SERPINA3 TF CLU FCN2 ZNF177 GRIPAP1 BCHE IGLC7 HSPA6 MSN STOM IGLC6 TMPRSS13 ITGA2B IGLC3 OAZ3 SLC2A1 ACTC1 HSPA1A IGLC1 C1QC ITIH1 IGHG1 ZBTB38 ORM2 KDM4D TFRC PRSS1 ANXA5 IGHG4 A2M IGKC FN1 HSPA1B HBE1 KRT1 ENG CD5L CPN2 IGKV1-5 KNG1 TRDC APOL1 IGKV2-40 CFB HSPA7 ACTG2 POTEF AGT HPR ALB C4A FGB VTN AMBP IGKV3-20 CFHR3 IGLL1 CIB2 HRG HSPA1L HSPA8 APOA5 GC SERPING1 F13A1 CFH HSPA2 APOA4 LOC440786 APOE LGALS3BP IGHV3-23 ACSM1 SDCBP POTEE IGHE IGLL5

GO_ENDOPLASMIC_RETICULUM_GOLGI_INTERMEDIATE_COMPARTMENT_MEMBRANE The lipid bilayer surrounding any of the compartments of the endoplasmic reticulum (ER)-Golgi intermediate compartment system. CD55 TM7SF4 COPZ2 ERGIC3 TMED9 F5 NAT8 LMAN1L KDELR1 LMAN1 YKT6 VMP1 LMAN2 CTSZ AREG TM6SF2 MPPE1 TBC1D20 WHAMM STX5 CSNK1D F8 SPPL3 TMED5 TMEM199 TMED1 COL7A1 SURF4 SEC23B PIEZO1 BET1 SEC22B TMED7 GORASP1 ADC MCFD2 SLC35C2 FOLR1 ERGIC2 TMED2 CTSC CNIH3 CNIH2 INS STX17 CLN8 TMED3 BCAP31 GOSR2 RAB1B C20orf103 TGFA NAT8B SERPINA1 ERGIC1 ASPSCR1 CD59 RAB2A CNIH GRIA1 TMED10 GOLGA2 VMA21

GO_SPECIFIC_GRANULE Granule with a membranous, tubular internal structure, found primarily in mature neutrophil cells. Most are released into the extracellular fluid. Specific granules contain lactoferrin, lysozyme, vitamin B12 binding protein and elastase. ANXA11 STX4 SNAP23 LTF CLCN3 OLFM4 CRISP3 STXBP3 CAMP ADAM8 VAMP1 ANXA3 STXBP2 STX3

GO_INTERMEDIATE_FILAMENT A cytoskeletal structure that forms a distinct elongated structure, characteristically 10 nm in diameter, that occurs in the cytoplasm of eukaryotic cells. Intermediate filaments form a fibrous system, composed of chemically heterogeneous subunits and involved in mechanically integrating the various components of the cytoplasmic space. Intermediate filaments may be divided into five chemically distinct classes: Type I, acidic keratins; Type II, basic keratins; Type III, including desmin, vimentin and others; Type IV, neurofilaments and related filaments; and Type V, lamins. KRT79 KRT9 KRTAP10-12 KRTAP9-8 KRT5 KRTAP1-3 BFSP2 KRT6B KRT72 KRT121P KRTAP13-2 KRTAP6-1 DST KRTAP27-1 FAM83H PRPH KRT36 KRTAP10-6 SHANK2 KRTAP4-3 MYO5A VMAC NCKIPSD KRTAP10-3 KRTAP19-8 KRT27 CLIP1 NEFH KRTAP7-1 LOC730755 KRTAP4-5 GPER KRTAP5-2 KRTAP25-1 KRTAP20-1 KRTAP3-3 KRT31 KRTAP19-5 KRTAP21-3 KRTAP6-3 KRTAP4-9 KRTAP4-2 KRTAP9-1 KRTAP10-4 KRTAP13-3 KRTAP15-1 KRT19 KRTAP5-10 KRTAP1-5 KRTAP13-1 KRT35 LMNA VIM KRTAP10-2 KRTAP10-11 KRT38 KRTAP19-3 KRTAP10-5 KRTAP4-8 KRT40 IFLTD1 PKP2 KRT32 KRT1 KRT12 KRT6A KRT33A KRTAP5-11 LDLRAP1 KRTAP6-2 KRT20 KRT39 KRTAP19-6 KRTAP20-3 PKP1 KRTAP12-2 KRTAP1-4 GJA1 KRTAP19-7 KRTAP5-6 NME1 KRT2 KRTAP4-4 KRTAP20-4 KRT23 UPP2 PNN KRT3 NME2 KRTAP1-1 KRT18 KRTAP12-1 KRT78 MNS1 KRTAP21-1 DES KRTAP9-4 KRTAP19-2 SYNM KRT73 KRTAP9-9 KRTAP5-1 NEFM KRT77 KRT84 KRTAP5-7 KRT37 KRTAP4-6 LMNB1 KRT10 KRT15 KRTAP21-2 KRT28 KRTAP29-1 CSNK1A1 KRTAP20-2 KRT17 KRTAP10-10 KRT16 KRTAP19-1 KRTAP10-9 KRT8 KRTAP2-1 KRTAP11-1 KRTAP3-2 KRTAP4-12 BFSP1 IFFO1 KRTAP9-3 KRTAP5-5 SLC1A4 KRTAP10-8 KRTAP12-3 KRTAP2-4 KRTAP10-1 KRTAP5-3 KRT6C KRTAP24-1 NEFL KRT80 CASP14 GFAP KRT82 KRT34 KRT7 KRTAP16-1 KRT14 TCHP KRT71 KRT86 KRTAP23-1 KRT74 KRT85 KRTAP5-8 KRTAP9-7 DLGAP2 KRT26 KRTAP26-1 KRT75 IFFO2 FBF1 KRT76 KRT13 FLG KRTAP5-9 KRTAP4-1 KRTAP9-6 INA KRTAP4-11 KRT33B NES KRTAP10-7 KRT83 KRT25 KRTAP9-2 KRT222 SYNC KRTAP3-1 KRTAP17-1 KRTAP22-1 KRT4 KRT24 KRTAP8-1 KRTAP5-4 NARF MICAL1 TLK2 KRTAP22-2 KRTAP19-4 KRT81 KRTAP12-4 NRP1 KRTAP13-4 JUP DSP LMNB2

GO_T_TUBULE Invagination of the plasma membrane of a muscle cell that extends inward from the cell surface around each myofibril. The ends of T-tubules make contact with the sarcoplasmic reticulum membrane. CACNB3 KCNJ3 CACNA2D1 CACNB2 CACNA1C SLC2A4 KCNN2 ANK3 ATP1A2 ADRA1A AKAP6 CAPN3 AHNAK2 NOS1AP ANK2 KCNJ2 KCNJ11 SLC30A1 STAC CAMK2D TGFB3 ATP1A1 RYR1 SCN1A RDX KCNJ5 SCN5A SLC8A1 AHNAK CACNA1S SCN1B CASQ1 EZR PPP3CB CACNA1D ATP2B4 SRI CAV3 SLC9A1 NOS1 DYSF BIN1 RTN2

GO_NADPH_OXIDASE_COMPLEX A enzyme complex of which the core is a heterodimer composed of a light (alpha) and heavy (beta) chain, and requires several other water-soluble proteins of cytosolic origin for activity. Functions in superoxide generation by the NADPH-dependent reduction of O2. CYBA CYBB NCF1 NOXO1 NOX4 NOX3 NOX1 NCF1B NCF4 NCF1C NCF2 NOXA1

GO_MUSCLE_MYOSIN_COMPLEX A filament of myosin found in a muscle cell of any type. TRIM32 MYH13 MYH6 MYH7 MYH1 MYH11 TTN MYH2 MYLPF MYH3 MYOM1 MYL3 MYH4 MYL6B MYL1 MYH8 MYBPC3 MYL9 MYL5

GO_PLATELET_ALPHA_GRANULE_MEMBRANE The lipid bilayer surrounding the platelet alpha granule. SNCA PHACTR2 SELP ITGB3 PCDH7 CD9 APLP2 CD36 ITGA2B TMX3 CYB5R1 SPARC PECAM1

GO_INVADOPODIUM A cell projection that emerges from the ECM-facing surface of a cell, is enriched in actin and associated cytoskeletal proteins, and displays localized proteolytic activity toward the substrate. SVIL DPP4 FAP ITGA3 WASH1 PLAUR FSCN1 NOX1 RUFY3 AFAP1L1 PAK1 ITGB1

GO_BETA_CATENIN_DESTRUCTION_COMPLEX A cytoplasmic protein complex containing glycogen synthase kinase-3-beta (GSK-3-beta), the adenomatous polyposis coli protein (APC), and the scaffolding protein axin, among others; phosphorylates beta-catenin, targets it for degradation by the proteasome. CSNK1A1 CTNNBIP1 RGS20 GSK3B RGS19 CTNNB1 GSK3A SIAH1 DACT1 APC2 AXIN2 AXIN1 APC CACYBP

GO_PERINUCLEAR_ENDOPLASMIC_RETICULUM The portion of endoplasmic reticulum, the intracellular network of tubules and cisternae, that occurs near the nucleus. The lumen of the perinuclear endoplasmic reticulum is contiguous with the nuclear envelope lumen (also called perinuclear space), the region between the inner and outer nuclear membranes. CAPN2 ADAM10 OSBPL7 OSBPL6 OSBPL3 PIK3R1 TRIM13 SYT6 GDPD5 BCAP31 CYBA

GO_INTEGRATOR_COMPLEX A protein complex that stably associates with the C-terminus of RNA polymerase II and mediates 3'-end processing of small nuclear RNAs generated by RNA polymerase II. INTS6 SHFM1 INTS1 INTS8 INTS7 INTS9 CPSF3L INTS4 INTS5 INTS3 INTS2 INTS10 INTS12

GO_WNT_SIGNALOSOME A multiprotein protein complex containing membrane-localized Wnt receptors and cytosolic protein complexes, which is capable of transmitting the Wnt signal. Contains at least a Wnt protein, LRP5 or LRP6, a member of the Frizzled (Fz) family, Axin and and a Dishevelled (DVL) protein. LRRK2 FZD1 APC WNT2 LRP5 LRP6 DVL3 DVL1 GSK3B CTNNB1 WNT3

GO_CYTOPLASMIC_UBIQUITIN_LIGASE_COMPLEX A ubiquitin ligase complex found in the cytoplasm. MARCH6 AUP1 USP33 ATG3 AMFR OS9 TCEB2 RBX1 VHL VHLL SEL1L CUL2 TCEB1 SYVN1

GO_NONMOTILE_PRIMARY_CILIUM A primary cilium which contains a variable array of axonemal microtubules but does not contain molecular motors. Nonmotile primary cilia are found on many different cell types and function as sensory organelles that concentrate and organize sensory signaling molecules. GUCA2B B9D1 ELMOD3 INHA RGS9BP CEP89 TUBG1 IQCB1 USH1C GUCA1C PDE6A GUCY2F TCTN1 GNA11 SPTBN5 GPR83 GUCA1B PDC PHLPP2 GRK4 OPN1LW KIFAP3 AZI1 NAPEPLD DCDC2 ROM1 MAK DRD2 ARL3 CNGA1 PKD1L1 OPN1MW2 DYX1C1 SEPT9 TBCC SPATA7 MKS1 OPN1SW RHO AHI1 C2orf71 CNGA3 PRPH2 USH2A MYO5A GNAT2 TMEM237 SHANK2 GNAQ CETN2 PDE6B RAB8A OPN5 CIB2 TMEM67 SEPT7 GNGT1 CDHR1 CNGB3 IFT140 DHRS3 OPN1MW USH1G RAB27A GNB1 ABCA4 MCHR1 C21orf2 CC2D2A TMEM216 MAP1B ATP8A2 PCDHB15 GUCY2D GUCA1A RGS9 NPY2R PKD2L1 TTBK2 DRD5 CEP290 C5orf42 TMEM107 GNAT1 ANO2 STRC PTGS1 GRK7 CACNA1F TCTN2 KIF17 MERTK RPGRIP1 DRD1 IFT52 NPHP4 GLIS2 SSTR3 NPHP1 WDR19 TULP1 TOPORS ARR3 FAM161A PROM1 BBS4 IFT57 SEPT2 PCDH15 CETN1 STRCP1 TMEM17 B9D2 RP1L1 C1orf96 SAG UNC119B MYRIP TRAF3IP1 CNGB1 BBS7 GRXCR1 IFT20 TMEM231 PTPRK C5orf30 RP1 OCRL PKD2 KNCN MKKS GRK1 MYO7A RPGR PPEF2 BBS9 RPGRIP1L PCM1 PDE6G GNB5 IFT122

GO_PLATELET_ALPHA_GRANULE_LUMEN The volume enclosed by the membrane of the platelet alpha granule. KNG1 VEGFA TGFB1 F8 LEFTY2 ORM1 SPARC AHSG F13A1 HGF FN1 TMSB4X PLG A2M F5 MMRN1 TIMP1 FGA QSOX1 PF4 IGF2 CLU FGG SERPINA3 TGFB2 SRGN PDGFA VEGFC APP SERPINA1 PDGFB ACTN4 SERPINE1 CFD SERPING1 GAS6 VWF SERPINF2 HRG ACTN1 ORM2 FIGF THBS1 A1BG PPBP TGFB3 PROS1 IGF1 ALDOA ALB FGB ISLR ACTN2 EGF VEGFB

GO_NUCLEOLUS A small, dense body one or more of which are present in the nucleus of eukaryotic cells. It is rich in RNA and protein, is not bounded by a limiting membrane, and is not seen during mitosis. Its prime function is the transcription of the nucleolar DNA into 45S ribosomal-precursor RNA, the processing of this RNA into 5.8S, 18S, and 28S components of ribosomal RNA, and the association of these components with 5S RNA and proteins synthesized outside the nucleolus. This association results in the formation of ribonucleoprotein precursors; these pass into the cytoplasm and mature into the 40S and 60S subunits of the ribosome. RPP14 ZNF274 OSBP NUFIP1 RREB1 VEZF1 ZNF207 DDX17 SSRP1 CCT5 NOL11 AATF VCX3B RPL34 RBBP5 DDX28 GEMIN2 ARNTL2 TIMM13 FTSJ3 RBM14 UTP15 GNL2 SPC24 TSG101 BCL9L TSEN15 INO80C C8orf4 FANCD2 S100A16 DGCR8 VCY1B ORC1 ZC3H14 DHX33 CIRH1A MED12 GORAB PRMT2 NANOG DKC1 HLTF HNRNPM RPS6KA6 NOLC1 NHP2 DDX47 FOXJ2 RPS26 ERCC6 EBNA1BP2 RBM3 PSPC1 PDCD11 CBFA2T3 GPATCH2 WDR46 TERF1 POP1 ZC3H15 LOC81691 SHQ1 RPL3 BEND3 VHLL DDX54 SPG11 HMGN5 SPATA24 SERPINB13 NEK2 COG7 LEO1 LRP1 SUZ12 RTF1 MRO NFS1 SNRPB2 RNF111 NUDT16 STK35 NONO CDV3 L1RE1 CA9 TAOK2 VCX3A TEX10 EXOSC2 MCM10 UTP11L SPIN1 POLR2I SENP3 APEX1 LSM6 DCAF17 H2AFY SIX1 POLR2F EEF1A1 MED1 WDR36 RRP9 CD2AP ABL1 ARL4D TMX1 TFPT STAG2 NAT10 DFFB CCDC86 HINFP NUAK1 C15orf29 TAF5 XPO6 VCX2 DDX24 HINT2 ACOX1 PRRX1 ZNF146 COIL NEK11 RNF20 PRKDC AEN FMN2 UTP14A PINX1 ZNF750 UTP23 GTF2H5 RBM4 KAT7 ARID3A SMG6 TRIM68 ENDOV SMARCB1 ARID5A MDN1 PLK4 MEAF6 SUB1 BTBD10 PRMT7 FEN1 TWISTNB PAK1IP1 MED27 SPTBN1 STK24 ZBTB33 NCL PTPN6 ORC4 KRI1 POP4 RPS13 ITPR3 PLK3 RBBP6 RNMT RRP12 BTN3A3 TOP1MT IPMK PFDN2 MYO10 DDX6 S100A3 TSEN54 RPS25 E2F5 TBL3 CAPG PNO1 ABT1 YPEL4 MXI1 TAF1B NOP56 LARP4B SMAD7 PLSCR1 SLC30A5 USP17 PNMA3 KLLN TRIM41 USP28 NGDN TSPYL2 RPS19 CCND2 CCDC106 BRIX1 MPHOSPH8 ZNF506 EME1 SKIV2L2 TTC3 NFIC SPTY2D1 RPS19BP1 PPP1R26 NSUN5P2 RPS6 NCF2 MAF1 RPL19 BOP1 SLC14A1 ELL3 ETV6 CIRBP SENP5 CASK ELP3 AGPS EXOSC1 XRCC6 SELENBP1 RNF213 MKNK2 CETN3 KDM5A SCNM1 SP140 ACADVL METTL1 KDM2A PARP1 ZBED6 MDFIC PITX1 POLR2K RPP38 NOL3 EFCAB4B NOP16 ABTB1 OLA1 MINA LAS1L MBIP VMP1 EXOSC5 C6orf89 RGS2 JAZF1 GATA3 WDR75 KDM2B PIH1D1 RBL2 CDC14C ZNF593 GRWD1 MPHOSPH10 URB2 RRS1 DHX40 GOLGA3 SIN3A TAF13 FAM64A CPS1 TCEA1 SDR9C7 STAU2 IP6K1 ZZZ3 NVL CDKN2AIPNL CDK4 PYCARD FAM21C HMGB2 SPRN PPP1CC CDK12 RAE1 TSR1 ARFGEF1 GTF2B SETD4 DAXX RPL36 RBM4B TRAF6 SNX15 EXOSC4 UBLCP1 CDC5L WDR55 ZCCHC11 NOP14 C14orf169 ZFHX3 SUMO1 NSUN5P1 CHTOP TNP2 IFI16 CHD2 MRPL23 PARP2 KAT5 TIMELESS ESF1 RRP15 HIRIP3 ISG20L2 RPL8 DDX21 NUSAP1 RPL23A TACC2 SRPK2 PPP1CA FOXA1 POP7 TMUB1 MLLT1 MRPL40 TBCA WDR33 NFIB USO1 RBM34 ADARB1 C9orf140 TUT1 RPS2 EXOSC8 C3orf17 SEPT7 RPP21 DEAF1 CHD7 ABHD14B AGAP2 DDX50 NIN RRP1 EIF3L MAP2 TCERG1 ZDHHC7 PPP1CB PGRMC1 PNMA1 GNL3 JHDM1D FGF22 TAF1D POP5 NMD3 NPM1 SURF6 RBM19 ZNF622 CCDC137 THAP1 DROSHA APTX ZCCHC9 DDX31 PNKP NBN RPS23 DIS3 NAA10 HSPA9 TGS1 ESRRA HUS1B RFC1 RRP1B RRP7A PER2 RASL11A BRWD1 PES1 KIF2A TDP2 SRP54 YPEL3 MAP1S WDR74 TNFAIP1 GTF3C3 ACTL6B G2E3 IK BCLAF1 SRSF5 CLEC3B NOC3L ZNF655 NOP10 CUTC RSL1D1 CDC14B FGF18 ATXN1L USP44 CRYL1 RPL7 MDM2 TAF15 FGF13 SF3B3 MPHOSPH6 REXO2 N4BP1 DDX52 THUMPD3 KAT6A CHD3 ARL6IP4 RPL21 ARL4A RPL35 ALS2CR8 FAM32A URB1 ZNRD1 IKBKAP CTSL2 HAND1 NEDD1 PA2G4 MRI1 NOL9 FAM9A DNAJB1 RPS3A THAP2 RCL1 RRN3 ERVK-16 RPS14 RDM1 TYMS SP110 ILF2 EIF6 BAZ2A BCAR1 SLC25A3 DCAF13 ACSL5 POLR1A L3MBTL1 CTSB ING3 TXNDC2 ANP32B PARP10 MBD6 POLR2A SETX RBPJ POLR1C DDX11 NOX4 NOVA1 HN1 TAF1A EIF3A FMR1 ERI1 PPAN SIRT6 DHX9 C1D HOMER2 GTPBP10 PIK3CB VPS25 RSPH4A RPL13A EED BHLHE40 CDK5RAP3 KIF2B RAD51 ETV4 MORF4L2 POLA1 RRP7B NIP7 BYSL RPL5 NOL7 NOC2L MCRS1 ZNF259 CDK8 CPT2 SERTAD3 POLR1D KLK6 FCF1 MOB2 CD3EAP ZNF330 NSUN5 EZR ILF3 EEF1D FAM21A PPID WDR3 PHF8 STON2 NSUN2 NOL4 CBX5 IL37 ACIN1 RPL11 MKI67IP NRIP1 RPL10A HAUS7 PWP1 CSTB WDR37 GAR1 WIBG TXN2 RPS11 MTDH PAX2 RPS3 WT1 RPL9 C1QBP RPAP2 IMP3 ADAR CDCA7L POLN RBM28 FRG1 GNL3L BLM FGF1 BCAS2 DAB1 DHX37 GLTSCR2 HEATR1 MRPS9 ISG20 POLR1E SAP30L VCX TFIP11 PHTF1 UBD PLRG1 ATF7IP2 DAB2 PLK5 VPRBP KLHL7 SCAF11 CENPP CAMK4 DDX5 TSEN2 RSL24D1 NOL12 XRCC5 EXOSC9 WRN OBFC1 RPAIN MKI67 SNAPIN THYN1 FBXO25 FLNA MRTO4 BCAS3 TRA2A UBE2N INO80E ATF3 NUP153 RPS7 WDR43 SMARCA4 C4orf43 RPS27A NHP2L1 RPL23 RPS10 WDR12 KIAA1310 ACAT2 NLE1 NOL10 MAFIP GTPBP4 DEDD2 NPM3 ITPR1 LLPH CIAPIN1 IDAS RCC2 TERT SPATA2 DNTTIP2 H1FOO TP53 OXR1 RRP8 NOL6 ZNF415 VRK1 APEX2 CCDC110 SNRPA TOP2A C2orf3 SBDS WBSCR22 DTL DEDD ANG SNAPC1 EXOSC6 EXOSC7 HOMEZ SRP19 FBLL1 RPL7A PARN ACACA DHX15 FTSJ2 CUL2 AKTIP TOE1 ASNA1 NF1 STOX1 SKP2 YPEL2 POLR1B REV3L TOP1 ZFP106 KIF20B RPP40 ZNF554 SEPT2 PPM1E RBBP8 ZNF385A RPF1 CEP85 DDX56 NF2 SIRT1 RPL4 NLRP5 STAT1 CCNT1 ZNF346 UTP3 ZMAT3 UTP18 DNAJB9 UTP6 ATXN7 TSEN34 PNMA2 IPO5 MNX1 LEPREL4 POLR2H YBX2 KIAA0020 NOP58 NOM1 VCY DDX27 CENPW INO80B FBL USP36 FAM129B DDX51 PLK1 PELP1 RAD17 REXO4 DNTTIP1 RPP30 FMR1NB NKRF UBTF EXOSC3 CRBN RPS9 SRP72 ZBTB16 HJURP SNAPC5 PWP2 NOL8 DCLRE1A DDX18 KDM4A EDF1 EXOSC10 SRP68 NOC4L TSPYL1 SIRT7 TRAIP GZF1 ERGIC2 LIN28A SETD7 DIAPH2 HUS1 MRPS31 DNAJC14 CHAF1A RORC PHF6 RAN NFX1 ATPBD4 SMARCA5 GEMIN4 HSPA8 C9orf3 XPO1 GLTPD1 SDAD1 TAF4B FBXW7 PAFAH1B2 TLE1 TAF1C RAB8A LYAR ZCCHC7 UTP14C MYO1C RBM15B PIN4 CEBPA POLR2L MALT1 LIN28B EMG1 HELQ GRB2 RG9MTD2 MYC C2CD4B OASL XPC ABCB8 JMJD6 PML HNRNPR MYBBP1A CRIPT SRSF9 ZCCHC17 C16orf88 ZNF771 NSA2 SMUG1 KRR1 TOP2B PHF2 MAK16 GTF3C1 RPF2 GPRC5B BMS1 KRT18 HMOX1 CTCF XRN2 RASL10A MUS81 RPL18 DMP1 RPL12 SMC2 NOP2 RCN2 ENC1 PHLDA1 MIDN UPF3B H1FX DIEXF FANCG CERKL SLC29A2 CDKN2AIP CDCA8 IMP4 PRKRIP1 FBXO11 ANKRD1 MIF4GD RBBP9 TCOF1 CHCHD1 NFATC3 SPATS2L TULP3 POLR2E PCGF5 POLR3K FXR1 RRP36 EWSR1 PEX14 WEE1 DDX23 CDKN1A TTF1 UTP20 RPL7L1 AKAP8 SURF2 PTBP1 LDOC1 PAPD5 DIMT1

GO_DNA_REPAIR_COMPLEX A protein complex involved in DNA repair processes including direct reversal, base excision repair, nucleotide excision repair, photoreactivation, bypass, double-strand break repair pathway, and mismatch repair pathway. MSH3 LIG4 MSH6 POLE ERCC1 NHEJ1 PMS1 XPC MLH3 ERCC8 MSH4 ZSWIM7 PMS2 KDM1A DCLRE1C C9orf142 PMS2P5 PHF21A MSH5 CETN2 ATM XRCC6BP1 RAD23B PMS2P3 POLE3 MSH2 POLE4 XPA C19orf39 XRCC4 ERCC4 PMS2CL SLX4 POLE2 WRN PRKDC POLD1 XRCC5 PMS2P1 CHRAC1 MLH1 RCOR1 XRCC6

GO_MICROTUBULE_ORGANIZING_CENTER_PART Any constituent part of a microtubule organizing center, a region in a eukaryotic cell, such as a centrosome or basal body, from which microtubules grow. NEURL4 IQCB1 TUBG1 KIF24 CCNF ROCK1 CEP89 MDM1 CEP63 CCDC13 STARD9 HSPA1B SPICE1 NEK1 SCLT1 HSPA1A CCDC67 PLK2 AZI1 CCP110 CAPG FOPNL MZT2A AGBL4 SSX2IP CEP72 C16orf80 KRT18 WASH3P TNKS2 LOC100653515 ALDOB AHI1 CEP170 ZMYND10 CROCC CEP164 RP2 KIAA0586 MKS1 KIAA0753 DZIP1L CEP19 NIN CETN2 CEP128 HERC2 C10orf90 RAB8A AURKA MZT1 PLK4 DZIP1 FTCD POC5 PAX2 CEP41 SIRT2 FLOT1 CNTROB TCP1 TUBGCP4 FAM154A ODF2 RAN HOOK3 CKAP5 TUBGCP2 CNTLN POC1B CLASP1 C2CD3 FBF1 WDR34 BRCA1 PCNT CEP120 TUBG2 TUBGCP5 STIL CDK5RAP2 KIAA1009 NUBP2 BLOC1S2 POC1A HTT CEP290 UXT CCDC78 PARP3 TTBK2 SASS6 HAP1 LCK SFI1 CCHCR1 CCDC146 NUBP1 CEP250 IFT88 PLA2G3 CCDC41 KATNB1 CCDC92 IFT52 TUBE1 CEP104 TUBGCP3 HSPA6 CENPJ WRAP73 ALMS1 TOPORS PDE4B SPAG5 TSKS MZT2B CETN1 BBS4 CCDC14 SDCCAG8 LRRCC1 CEP85 CCDC113 CCDC151 C1orf96 CEP76 EXOC7 CEP192 TNKS IFT20 WDR62 CEP152 NEDD1 AGBL2 KIAA1731 PIBF1 CEP55 OFD1 MPHOSPH9 TSSK2 WASH1 TUBGCP6 PCM1 TOP2A TUBD1 BBS9 CEP135 CETN3

GO_ENDOLYSOSOME_MEMBRANE The lipid bilayer surrounding an endolysosome. An endolysosome is a transient hybrid organelle formed by fusion of a late endosome with a lysosome. AP2A2 TLR7 AP2B1 TLR8 TLR9 CLEC16A CLTC CLTA AP2A1 AP2S1 TLR3 AP2M1

GO_TRANSCRIPTIONALLY_ACTIVE_CHROMATIN The ordered and organized complex of DNA and protein that forms regions of the chromosome that are being actively transcribed. AFF4 ELL NARG2 EXOSC4 EXOSC3 EXOSC5 ZC3H8 KIAA0947 CTR9 PSIP1 H2AFB1 TTC37 H2AFB2 HIST1H1C BCAS3 EXOSC10 PADI2 H2AFB3 ESR1 PELP1 WDR61

GO_PROTEINACEOUS_EXTRACELLULAR_MATRIX A layer consisting mainly of proteins (especially collagen) and glycosaminoglycans (mostly as proteoglycans) that forms a sheet underlying or overlying cells such as endothelial and epithelial cells. The proteins are secreted by cells in the vicinity. An example of this component is found in Mus musculus. TNR ACAN OTOA CD151 GPC3 TGFBR3 COL4A6 LOXL2 MFAP2 MMP13 EPYC ODAM ZP3 ANXA2 LAMA5 FBN2 COL9A2 SERAC1 GPC1 OPTC COL21A1 TFIP11 USH2A MATN3 FREM1 ADAMTS14 CHI3L1 WNT5A FGF1 ECM2 WISP3 ADAMTS7 PTN AGRN COL22A1 RELN COL1A2 NTN1 COL6A6 CCDC80 LTBP4 ENTPD2 PODNL1 COL24A1 ADAMTS13 COL4A2 COL10A1 CCBE1 TNN ADAMTS17 KAL1 CHADL KERA MFAP1 EGFL6 ADAMTSL2 WNT4 ADAMTS1 ADAMTS20 COCH TECTA DAG1 BGN VWA1 ENAM COL12A1 TIMP2 SPN CD248 FN1 VWC2 MMP28 NTN3 MUC5AC ADAMTSL3 SMOC2 CYR61 FBLN7 FBLN2 AMTN FLRT1 DLG1 CASK COL9A3 WNT9A THBS4 MMP26 WNT10B CILP2 ADAMTS16 ELN EFEMP2 WNT8B EMILIN2 ADAMTS18 COLQ MMP20 MGP COL3A1 LAMA4 LOX FLRT2 COL18A1 DGCR6 SOST CALR NDNF COL11A2 MMP23B HAPLN4 DSPP WNT6 LEPRE1 COL9A1 LUM SMOC1 MMP10 CRISP3 EMILIN3 THBS2 PRELP LAMA1 TNC CTHRC1 MMP7 LECT1 ADAMTSL5 HMCN2 APLP1 NPNT MFAP4 SPARC COL7A1 COL17A1 WNT7A MMP8 ADAMTS10 SPARCL1 MMP25 SMC3 CHAD LAMB3 MMP17 ADAMTS4 NID2 WNT7B MMP1 COL1A1 AMBN SERPINA1 TIMP3 SFTPA1 ADAMTS2 DCN TGFBI EFNA5 EGFLAM PXDN WNT8A SPON2 EMID2 IMPG1 SPON1 MMP9 COMP SLIT3 C8orf84 SFTPD WISP1 ADAMTS9 THSD4 MFAP5 RBP3 MATN1 COL16A1 ANGPTL4 COL5A2 CRTAC1 TFF3 NTN4 CRISPLD2 HAPLN3 RTBDN GPC6 MMP3 OMD PHOSPHO1 SLC1A3 SFRP1 COL6A5 CHL1 C6orf15 WNT9B MATN2 HSPG2 VTN FBN3 TECTB GFOD2 ERBB2IP OLFML2A COL27A1 POSTN SLIT2 RELL2 MMP11 LAMC2 MMP16 NYX LAMC1 KAZALD1 TIMP4 COL5A3 COL6A3 DST NAV2 LAMB2 MMP19 NID1 PODN ADAMTS8 LGALS3BP ADAMTS3 COL4A5 EFEMP1 ADAMTSL4 FGF9 DMP1 WNT2 SPOCK3 HPSE FBLN5 PRSS36 VIT ADAMTS6 COL4A1 MYOC WNT16 LAMB4 COL6A1 MMRN2 LAMB1 GPC2 FBLN1 BCAN ZG16 SERPINF1 IL1RL1 WNT10A BMP4 ADAMTS12 ADAMTSL1 NOV CPZ ZP1 UCMA ITGA6 ADAMTS5 MMP12 MMP24 SHH PAPLN PI3 ANG HMCN1 FMOD HAPLN2 TNXA TNXB LOXL1 HPSE2 GLG1 ZP2 COL4A4 DPT FREM2 TINAG TFPI2 LTBP1 GLDN FBN1 WNT3 ADAMTS19 CST3 MUC4 LGALS1 VEGFA EMILIN1 GPLD1 ZP4 CPA6 MMP2 MEGF9 LOC100507050 COL19A1 WNT2B BMP1 WNT3A IMPG2 HAPLN1 NCAN MEPE COL28A1 VCAN ASPN COL11A1 TNFRSF11B COL14A1 WNT5B SPOCK1 RPTN ANXA2P2 OGN LAMC3 WISP2 LEPREL1 ACHE MAMDC2 FRAS1 ABI3BP HSD17B12 SNCA GPC4 PTPRZ1 SPOCK2 ADAMTS15 CRTAP VWF SLIT1 LAD1 EMID1 COL15A1 VWA2 COL5A1 COL4A3 GPC5 CTGF TIMP1 COL8A1 ALPL WNT1 COL8A2 FREM3 SFTPA2 AMELY COL2A1 LTBP2 ECM1 TGFB1 LAMA3 FLRT3 WNT11 LAMA2 COL6A2 CILP AMELX

GO_IMMUNOLOGICAL_SYNAPSE An area of close contact between a lymphocyte (T-, B-, or natural killer cell) and a target cell formed through the clustering of particular signaling and adhesion molecules and their associated membrane rafts on both the lymphocyte and the target cell and facilitating activation of the lymphocyte, transfer of membrane from the target cell to the lymphocyte, and in some situations killing of the target cell through release of secretory granules and/or death-pathway ligand-receptor interaction. GZMA SNX27 CORO1A HAVCR2 LGALS3 BCL10 STX7 LCK CD6 CD3E CD28 PTPRJ CD37 ZAP70 PDCD6IP C17orf87 ICAM1 CD53 STOML2 DLG1 ALCAM EZR SOCS6 CD81 MYH9 PRKCQ PRKAR1A LAT GZMB DUSP3 CARD11 RHOH

GO_POLE_PLASM Differentiated cytoplasm associated with a pole (animal, vegetal, anterior, or posterior) of an oocyte, egg or early embryo. SNRPG HENMT1 TDRD9 DDX6 PIWIL1 TDRD1 ASZ1 EXD1 PIWIL2 CARHSP1 MOV10L1 DDX4 TDRKH MAEL TDRD5 PIWIL4

GO_INTRACILIARY_TRANSPORT_PARTICLE A nonmembrane-bound oligomeric protein complex that participates in bidirectional transport of molecules (cargo) along axonemal microtubules. IFT80 IFT57 KIF17 IFT74 HSPB11 TTC26 IFT20 TTC21B DYNC2LI1 TTC30A IFT46 IFT81 IFT43 TTC30B WDR35 TRAF3IP1 WDR19 IFT122 IFT27 KIF3B IFT88 RABL5 IFT172 CLUAP1 TTC21A IFT52 KIFAP3 IFT140 TULP3 TRIM59

GO_POSTSYNAPTIC_MEMBRANE A specialized area of membrane facing the presynaptic membrane on the tip of the nerve ending and separated from it by a minute cleft (the synaptic cleft). Neurotransmitters cross the synaptic cleft and transmit the signal to the postsynaptic membrane. GRIA4 CHRNA10 GRIN2A SHANK2 GLRB PTEN DMD SIPA1L1 CHRNB1 GRM3 GABRA2 NLGN1 LRRTM2 GLRA3 GRIK4 LRRC4C LIN7C GABRA6 SSPN CHRNG PICALM NLGN4Y CACNG8 SH2D5 NEURL GRIN2C STRN HOMER1 PJA2 ARRB1 TMUB1 CADPS2 CHRM5 TANC1 CHRM1 SYNDIG1 SEMA4C SHANK3 RUSC1 GABBR1 ZC4H2 BCR ARC HTR3A HOMER3 KCTD12 RAPSN MUSK CHRNA1 GOPC P2RY1 SHANK1 CHRNA7 CHRNA3 CHRNA2 GRM7 ARRB2 SHC4 ProSAPiP1 DRP2 CABP1 DAG1 DNM2 CHRNA9 CHRNB2 DLG4 KCND2 SRGAP2 HTR3B DNAJA3 GRIA3 FBXO45 DLG1 ODZ2 PVRL3 GRIK2 GRID1 APBB1 CHRNA5 DLGAP3 DTNBP1 DLGAP1 CHRM2 GABRB3 CNIH3 LIN7A LRRTM1 GABRP IQSEC3 CLSTN3 GABRQ NLGN4X KCTD8 LIN7B P2RX1 GRIK3 GRIK5 GABRA4 LRFN1 CPEB1 GABRA3 CHRNB3 GABRG1 GABRB1 SCRIB PRR7 GRIN1 MINK1 CNKSR2 EPHA4 GABRR2 GPHN ARHGAP32 NCS1 GRIP1 MAGEE1 DLG2 DISC1 CLSTN1 CLSTN2 TRPV1 CHRNA6 GABRG3 CNIH2 D4S234E COL13A1 OPRD1 NELF IL1RAPL1 ATAD1 DLG3 GLRA4 KCNC2 CHRM3 GRID2 PICK1 CHRM4 RGS14 LRRC7 GRIA1 ANK1 LRRTM3 F2R KLHL17 IGSF9B ARF1 ANKS1B GRASP NTRK2 COMT KCNJ4 LRFN2 GABRR3 GABRE CHRNA4 LRFN3 LRRC4 UTRN GABRR1 SIGMAR1 SEMA4F LZTS1 GRIA2 CBLN1 DLGAP2 MPDZ SYNE1 GRIN2D PDLIM5 SRCIN1 CAMK2N1 GABBR2 GRID2IP CHRNB4 GRIK1 FAIM2 GRIN2B GLRA2 GRIN3A NLGN2 CACNG5 CHRNE GABRG2 KCNB1 ANK2 GABRD CDK5 GABRA1 SYNPO EPHA7 PCDH8 GRIN3B FMR1 KCTD16 GABRA5 GLRA1 GABRB2 NETO1 ABI1 LRRTM4 ANK3 PSD3 ADORA1 HOMER2 CHRND

GO_PLATELET_DENSE_GRANULE Electron-dense granule occurring in blood platelets that stores and secretes adenosine nucleotides and serotonin. They contain a highly condensed core consisting of serotonin, histamine, calcium, magnesium, ATP, ADP, pyrophosphate and membrane lysosomal proteins. SERPINA4 ITPR1 ITIH3 CD63 APOH ECM1 LGALS3BP CTSW FAM3C TIMP3 LAMP2 SEPP1 HPS4 ABCC4 SELP ITIH4 CLEC3B RARRES2 CDC37L1 SPP2

GO_MITOCHONDRIAL_MATRIX The gel-like material, with considerable fine structure, that lies in the matrix space, or lumen, of a mitochondrion. It contains the enzymes of the tricarboxylic acid cycle and, in some organisms, the enzymes concerned with fatty acid oxidation. ERAL1 KIAA0391 MRPL34 DHFRL1 IBA57 GSTK1 MRPL18 GCSH MRPL49 MRPL46 GLRX5 LONP1 PDK3 PDK4 NUBPL SARDH FH SDHAF1 MTHFD1L BCAT2 PDP2 PCCB GTPBP5 CARS2 MMAB ACSF3 ALDH2 PDHA1 PRDX5 MRPS2 TUFM MRPL36 TRAP1 SSBP1 MRPL17 TK2 TP53AIP1 MRPL37 NUDT1 CA5A ME3 MRPL42 HMGCS2 ATP5D PDSS2 HADH ALKBH7 MRPL23 MRPS36 MCCC2 HSD17B8 MTHFD2L LIPT1 MTERFD2 C10orf2 BCKDHB IDH3B SLC25A5 THEM4 MRP63 ISCU SUPV3L1 MRPS22 NDUFA7 OAT NME4 ALAS1 NDUFS7 ABAT HARS2 MIPEP SARS2 PMPCB GLS PYCR2 C2orf56 C12orf65 MRPS33 TARS2 GARS ACADVL NUDT9 ME2 ACSM2B MRPL33 MRPL3 ETFA MTG1 MTHFS LYRM7 AMT LACTB2 SIRT5 DGUOK ALAS2 ATAD3A CPS1 NARS2 PUS1 COQ3 HIBADH LRRC59 NDUFS1 ADHFE1 SCO2 ACADM ATXN3 FASTK FECH NDUFA10 TIMM44 ATP5F1 HADHB MRPS14 FDX1 VDAC2 SUCLA2 GFM1 MRPL22 DHTKD1 SOD2 C7orf30 SDHAF2 AGXT2L2 PPM1K PRDX3 ISCA2 METTL20 NDUFA9 MRPL16 ALDH1L2 LYRM4 ELAC2 DHX30 ACO2 POLRMT ALDH1B1 SUOX ALDH6A1 HSPA9 NUDT2 BTD MRPL43 MARS2 POLG ACAD8 FDXR ARG2 THEM5 DIMT1 DLD FASTKD2 DTYMK ABCE1 ACOT2 ETFB CLPP CHCHD1 ATG4D HADHA ICT1 MRPS18A ACSM3 MRPL9 APOA1BP CARKD PMPCA HSPA1L MRPL47 FARS2 D2HGDH MRPL10 PCK2 ACAT1 PDE12 MTERFD3 GFM2 CBR4 ACADL MRPL40 MRPS31 ACSM2A OGDH TFB1M ERBB4 GLUD1 ACSF2 GUF1 LIAS NSUN4 NDUFAB1 WARS2 HSPE1 AGPHD1 AUH MRPL41 PIN4 CS TMLHE CHPF MRPL39 MRPL13 MRPL21 DUSP21 SHC1 POLDIP2 MRPS12 QARS MRPL11 ALDH7A1 MLYCD MRPS15 SUCLG1 LARS2 DARS2 LRRK2 PDE2A FPGS NFS1 MRPS35 MRPS6 NT5M GRSF1 SOD1 ATP5B ACADS NSUN3 COASY PC MCEE MRPL55 MRPL52 PDK1 ATP5A1 DAP3 DMGDH TDRD7 MRPL28 ETHE1 DLAT TRMT5 FLAD1 EARS2 NAGS SIRT4 TFB2M CLPX RAD51 MRPL2 DECR1 CA5B PDHA2 MRPS24 DDX28 ECI1 POLG2 BCKDK SIRT3 TRMT61B ECHS1 IDH3A NDUFS2 ALDH5A1 OTC GSR BDH1 PDPR MRPL15 ATP5C1 MRPS5 GLDC VDAC1 AADAT MRPS26 RG9MTD1 REXO2 C6orf57 PYCR1 MRPL32 TST MPV17L2 HSD17B10 PDSS1 ADPRHL2 NDUFS3 BCL2L1 DLST PDHX ETFDH DBT MRPL27 TYMS PARK7 HMGCL CASQ1 ARL2BP BCO2 ACN9 HAGH MRPL51 BCKDHA ACADSB GPX1 MRPL19 KIAA1967 MDH2 RARS2 MRPS11 C1orf177 CREB1 ALDH4A1 ACSS1 MRPS16 GLS2 BLOC1S1 ACSM5 PCCA HSPD1 ISCA1 CYP11A1 FDX1L TXNRD2 MRPS21 PARG OXCT1 TRNT1 DHRS2 RNASEL MRPS17 MCL1 MRPL12 PITRM1 PARS2 AASS LIPT2 SHMT2 MTRF1L AGXT2 NDUFS8 TERT FASTKD5 DNAJA3 MPG C17orf42 IARS2 TP53 SUCLG2 ACOT9 TFAM AK4 MUT HIBCH GADD45GIP1 CCDC111 IVD AKR1B15 CYP27A1 GRPEL2 LRPPRC KARS YARS2 GRPEL1 TXN2 PTCD1 C6orf125 RPS3 PPIF PRODH C1QBP NR3C1 LDHAL6B GCDH ATP5E TRIT1 PDHB PDK2 MRPS28 MRPL20 MRPL35 PPA2 AGXT ABHD10 AK3 MTERF TSFM MRPS7 PDP1 IDH2 ARL2 TOP1MT PABPC5 MRPS18B MCCC1 GLYAT ACSM1 ACAD10 MTHFD2 ACSM4 MRPS18C MMAA MRRF NUDT13 DNA2 GLRX2 MRPL48 PAM16 MRPS9 AGXT2L1 OGDHL GOT2 FXN GPT2

GO_GERM_CELL_NUCLEUS The nucleus of a germ cell, a reproductive cell in multicellular organisms. TRIP13 TNP1 MLH3 LHX8 ACTL7A TCFL5 TOPBP1 AURKA MLH1 HILS1 HSPA2 MARCKS TNP2 KIF6 H2AFX SYCP1 ARPM1 REC8

GO_PRE_AUTOPHAGOSOMAL_STRUCTURE_MEMBRANE A cellular membrane associated with the pre-autophagosomal structure. ATG12 STBD1 WIPI2 RAB1B RAB7A WIPI1 ATG16L1 ATG2B ATG2A ATG5 ULK1 RB1CC1 ULK2 ATG14 WDR45L WDR45

GO_CLATHRIN_COATED_PIT A part of the endomembrane system in the form of an invagination of a membrane upon which a clathrin coat forms, and that can be converted by vesicle budding into a clathrin-coated vesicle. Coated pits form on the plasma membrane, where they are involved in receptor-mediated selective transport of many proteins and other macromolecules across the cell membrane, in the trans-Golgi network, and on some endosomes. AP1S1 ITSN1 HIP1R SH3BP4 AP2S1 NECAP2 FCHO1 RAB35 LRP1 HSPD1 CUBN CLTC APP TFRC AP2B1 VLDLR EPS15 RAMP2 AMN CLTB EPN1 DNM2 TNK2 LDLRAP1 LRP12 ARRB2 FNBP1 INPP5F AAK1 AP4B1 SNAP91 LRP10 CTTN AP4E1 FCHO2 AP4S1 AP1S2 AP1S3 SORT1 CLTA AP2A1 ARRB1 DNM1L EPS15L1 EGFR CDHR5 SLC2A4 CXCR7 SLC18A3 EPN3 OCRL REPS1 MYO6 LDLR NECAP1 SELE SGIP1 TF LRP3 AP2M1 CLTCL1 TBC1D5 ATAT1 LRP2 KIAA1199 SYNJ1 PICALM AP2A2 DAB2

GO_NUCLEAR_OUTER_MEMBRANE_ENDOPLASMIC_RETICULUM_MEMBRANE_NETWORK The continuous network of membranes encompassing the nuclear outer membrane and the endoplasmic reticulum membrane. UGT2B11 PITPNM1 TMEM188 ATXN3 TTC35 LRRC59 XXYLT1 FDFT1 CALU TMEM132A COPZ2 RAB21 CYB5R4 UGT1A10 ZNRF4 TMEM173 SEC22C EDA ART1 LPGAT1 SLC9A1 TMEM174 AGPAT6 ATP10D COPZ1 RASGRP1 DHCR7 ARL6IP5 ALG1 CLSTN1 ELOVL5 FAM134B SPCS3 BCAP29 SLC37A2 ORMDL2 G6PC3 PLA2G4A SRD5A3 FAR2 SLC27A4 DNAJC1 SACM1L SLC39A1 SSR1 CERS1 VTI1A ILDR2 TMED4 CYP3A43 SPCS2 TUSC3 MSMO1 ELOVL7 ALG14 FAF1 DPM1 SEC61G FKBP14 CYP4F8 UGT2B4 ALDH3A2 SERINC1 CYP2R1 SEC63 PKD2 RDH16 ALG10 LTC4S SSR4 ITPR2 ATG9A HLA-DRB4 UBXN8 LSS KDELR1 HM13 MARCH1 SDF2L1 PAPPA-AS1 LPCAT4 SOAT2 ATP11C CYB5A COPG CYP4F12 HLA-DPA1 LRAT PITPNB ZDHHC16 CYP2A7 SRD5A1 FKBP3 VKORC1 JPH1 TMEM111 TBXAS1 SFTPA2 HLA-DRB1 NFE2L1 TMEM109 FLRT3 GPSM1 GPR37 PROS1 REEP4 CAV1 PTPN1 CYP2U1 DRD1 MARCH5 CDC42 SLC33A1 FKBP11 CYP19A1 DPM2 PLD1 GSG1 POMT1 UBXN7 MBOAT2 ORMDL3 LRRC8C TM7SF2 POMT2 CYP3A4 HLA-DQB2 PTGDS ACSL1 FKBP8 ACER1 ERN2 SLC37A3 SLC35D1 LRMP HLA-C HLA-E DST UFD1L AWAT2 SEC23A TMCO1 PGRMC1 SHISA2 ALOX5AP CYB5R3 COPA PPAPDC3 SPTLC3 STX18 CREB3L4 CTAGE5 TMPO ABCB6 RNF5 AREG HSP90B1 TMEM66 B3GAT1 C3orf39 TPTE2 TTC9 HMOX2 NR3C2 KSR1 ARMC10 CHERP HSD3B2 CLN3 HMGCLL1 APOB CYP7A1 TRAM1 PGAP3 CTDNEP1 PIGO GPER ATP2A1 DHRS7B CPT1C CYP4A22 ATP2A3 NOTCH4 RASGRF2 ATP8B2 SLC39A7 C5orf4 SDR16C5 BCAP31 ARV1 RDH11 OCA2 PLA2G2A TMEM170A LRRC8D SRI SFTPC DOLPP1 HSD11B1 LPIN2 TMBIM6 ERGIC3 ALG5 NAT8 SLC35B4 DNAJB14 DAD1 FAAH SLC35B3 RRBP1 PORCN TMED1 CYP4X1 AGPAT3 STX5 PMEL EXT1 ZDHHC9 WRB CREB3L2 LRRC8B FAM69B CALHM1 SVIP TBC1D20 CYP2F1 CYP4V2 POM121 ELOVL1 SHISA3 CNIH3 PDIA6 ALG8 CYP2C18 FMO4 SLC16A11 HSD11B2 UPK3A SLC28A3 EXTL2 YIF1A CYB5R1 SFTPB STT3A SEC24D TMC6 RNF13 MBOAT1 ABCA1 CASP4 CANX NOX5 SEC16A RPN2 CYB5R2 KLHL14 HHATL NOS1 SGK1 VAMP7 DERL3 C3orf52 TTYH1 ATL3 SPINK5 FMO5 TMEM189 CLSTN2 PIGH COX4NB EI24 JSRP1 MOGAT1 STIM1 KCNA2 ZC3H12A MAN1B1 KDELR3 DERL2 CNIH2 EMD HLA-DQA1 SEC62 ALG11 CLN8 HLA-DRB5 DGAT2 OSBP SEC16B CYP7B1 MPDU1 CYP4F3 PEX16 CAMK2D CYP4F2 BET1 SORT1 ABCB9 NCEH1 AUP1 NBAS SEC11C TMEM203 NOTCH3 PSEN2 PIGX YIF1B NAT8L ATP2A2 TYRO3 SEC61B LOC643181 PIGY DPAGT1 DUOXA1 MARCH2 VAPA ERAP2 FKBP2 PDCD6 LPCAT3 CYP4F22 UGT1A4 CYBA MGST3 FADS2 PTGIS RDH10 CYP26A1 SYNE1 UBIAD1 CYP1A2 HLA-DQB1 DHDDS SCFD1 OSTalpha PIGB SGMS2 SPTSSA XBP1 CD4 PIGK ELOVL6 ESYT1 BECN1 GHRHR UGT1A1 PTPLAD2 BSCL2 TTC9B GALNT1 ATL2 LPIN3 ARL6IP1 UBXN1 VMA21 DDRGK1 RNF185 EPHX1 ESYT3 MX1 SFTPA1 UBE2J1 MAP3K5 ANTXR2 REEP2 SRPR ALG13 PPM1L TMEM178 NOTCH2 TMC8 ATP10B HLA-B CYP1A1 CAMK2B PPP1R15A TMCC1 TMEM85 CH25H JPH4 CD1D INSIG2 MBTPS1 LPCAT2 SELK SDCBP DPM3 P4HTM ITPR3 PTGFRN FADS3 PTDSS1 PEMT NSDHL PJA2 ENTPD2 LBR PLOD3 CAMK2G CYP26C1 TMEM38A TMEM33 RTN3 TMX1 UBXN4 PLD2 SCD RHBDD1 SLC9A6 SLC8A3 C19orf12 SEC24A GBA2 CYP17A1 ERLIN1 CERS4 FMN2 EIF5AL1 TAPBP RNF170 LRRC8E HLA-F ZW10 POR CYBB DIO1 SEC22A CSPG5 CYB5RL ANKRD13C SLC35G1 APH1A IRGM INSIG1 TAP2 CYP2D6 TTC9C CYP2J2 SURF4 SEC23B SLC27A5 YIPF5 RSAD2 OSBPL6 SOAT1 CYP2B6 SPPL3 TAPBPL IMP5 SLC35B2 OTOF DLG1 COPG2 UBQLN4 TRDN RYR3 RAB18 CERS2 RIC3 SEC61A1 ALG12 TMTC2 STX17 PIGL ELOVL2 RNF103 POFUT2 CDS1 NOS1AP COL4A3BP CEPT1 ERP44 C8orf17 SEC31A OS9 PLAUR ARCN1 FAM69A ERAP1 STBD1 MGST1 SLC37A4 ACER3 MMGT1 RP9 RAB10 LMAN2 RETSAT FKBPL DGAT2L6 BOK HSD17B2 UFL1 LMAN1L PGAP2 RHBDF2 RCE1 DHCR24 HERPUD1 AGPAT4 SLN GPAA1 DMPK FKBP1B KIAA1715 GJB1 C19orf63 FAM176A TLR8 RNF121 SEZ6L2 SEC13 TRIM13 NECAB3 XYLT1 EGFR EBPL RDH14 OSTC RINT1 CYP2E1 JAGN1 CLGN REEP3 VCP MGST2 TECR ABCG1 UGT2B10 EBP NPLOC4 ASNA1 OSBPL8 SRD5A2 CYP21A2 TMEM129 RNF133 HLA-A SYNE2 DDN C6orf70 PTPN5 LPIN1 FA2H TGFA ERO1LB FMO2 FMO3 CYP2C9 RNF175 ACSL3 GOSR2 CERS3 UBA1 SEZ6L UGT2B15 IFNGR2 RHOG ERN1 RAB6A FITM1 HMGCR HLA-DRA GUCY2D NUTF2 GJC1 B3GALTL SIGMAR1 CERS5 CERS6 RAB3GAP1 XYLT2 ANO5 GALNT2 PIGT PIGF UCHL1 MFSD2A ICMT PLD3 RTN4 SEC11A TRAF2 OST4 SFTA3 C15orf24 EIF5A USP19 ANKS4B NCLN PSEN1 LMF1 DNAJB9 SLC17A3 ATP13A1 MCFD2 SMPD4 RAB14 PLOD2 SEC61A2 HLA-DPB1 C4orf34 SCD5 BAX AGMO FAM57B PIGC RAB2A TMX3 PIGQ AKAP6 AGPAT1 SNCA PI4KB HSD17B12 KTN1 FITM2 PIGA APOO PIK3R1 RAB1A CYP46A1 AMFR RAB2B RTN2 RAB9A FKBP1A C19orf46 TMED5 PML SYVN1 CYP3A7 LMF2 PNPLA8 BCL2 CYP2S1 NOMO2 CYP2C8 ORMDL1 KRTCAP2 ABCC6 SEL1L FKBP5 TMED3 KDELR2 VKORC1L1 C14orf1 DGAT1 ERGIC2 LRIT1 ERMP1 TMPRSS3 PTCHD2 DNAJC14 PIGN SELS TEX261 SPTSSB XPO1 RFT1 GLTPD1 GRM6 PLA2G4C NAV3 SERP1 FTCD OSBPL3 ABCD4 RNF180 PNPLA3 DEGS1 ANXA7 ERGIC1 NOTCH1 DEGS2 NUS1 HLA-DQA2 CYP39A1 DOLK PTPLAD1 MBOAT7 SC5DL SEC31B DNAJA1 RTN1 TAP1 TLR9 FKBP7 TMED9 RAC1 CYP4F11 NUP210 WLS UGT1A3 KBTBD10 PTGES AGPAT2 PNPLA6 SPCS1 EXTL3 GUCY2C SERP2 REEP6 RYR2 OSBPL7 MMP27 UGT1A6 AGTRAP NXNL1 DTNBP1 TM6SF2 PKMYT1 TBL2 HMOX1 MAPK8IP1 DHRS7C PTPLA DHRS4 SREBF1 PIGP C11orf9 PIGM PIEZO1 CDS2 TMED7 NDRG4 TLR3 MTOR LRIT3 TMED10 ATF6B SLC27A2 EIF2AK3 GJA1 MEST RHEB ELOVL4 ERLIN2 NAT8B PIGG ALG9 RAB3GAP2 TMEM106C FAM158A KCNK2 SGK196 RAB35 ASPH PIP4K2B IKBIP VTI1B FAM69C SEPN1 C9orf89 DERL1 GIMAP1 SGMS1 TOR1AIP2 TMED6 ALG3 CISD2 YIPF7 CKAP4 TMEM214 CYP26B1 LCTL EIF5A2 AGPAT9 PARP16 LRRC33 PIGW ARHGAP32 USP17L2 HSD17B3 PIGS MLEC C1orf9 CYP2C19 RDH5 CYP4Z1 FXYD3 G6PC CASQ1 MOGAT2 ACSL5 COPB1 ERO1L CYP8B1 APOL2 PREB FOLR1 PLN RYR1 EXTL1 CREB3 CYP1B1 MMP23B CALR HLA-H SEC24C EDEM1 CYP2W1 C22orf28 SGPP2 MBOAT4 MOXD1 DUOXA2 GRIA1 TXNDC11 UGT2B28 SRP9 SLC37A1 FLRT2 C14orf49 AGPAT5 PLP2 OSBPL5 SLC18A1 TMEM147 TMEM93 ZDHHC6 UGT3A1 ZFYVE27 HPN UPK2 PTDSS2 SFTPD SAMD8 ATG14 UBE2J2 PIGZ GRIA2 UBAC2 DHRS9 ELOVL3 SSR3 ESYT2 UNC93B1 UGT1A8 UGT1A9 MOSPD3 UGT2B7 MR1 SGPL1 SPPL2A SREBF2 C8orf83 IL15RA VRK2 CYP2A13 TMED2 RNF43 NOX4 HLA-G PTGS2 MRAP2 ATP8B3 SEC22B TRPM8 COPE PTGS1 EPT1 SAR1B ABHD4 CYP51A1 PIGV SDF2 NUP62 SQLE CREB3L3 TRAM1L1 SRPRB PLD4 RAB1B HSD17B7 USE1 JPH2 UGT1A7 PCYT2 M1 HRC G6PC2 TOR1A LMAN1 PSKH1 RPN1 RHBDF1 CYP4A11 AWAT1 AHCYL1 MAGT1 CCDC155 SPAST MRAP TM7SF4 LCLAT1 TLR7 CD74 MOGS PDIA5 WFS1 IER3IP1 PGAP1 ALG10B FKBP4 ACSL6 ANKLE2 TSPO2 DNM1L SCARA3 LPCAT1 ATP10A PCYT1A RNF139 DHRS3 CLN6 PTPLB GNRH1 RAB1C PNPLA2 FKBP9 SLC35B1 MTDH ACSL4 MIA3 HSD3B7 SEC24B PCYT1B CFTR HLA-DRB3 JPH3 RHOA CREB3L1 CYP2A6 CD59 CYP4B1 MLANA FAF2 STIM2 FKBP10 TMEM67 CYP3A5 GUCY2F MRVI1 FKBP6 ALG6 C19orf6 VAPB STT3B MOGAT3 ZMPSTE24 SGPP1 BNIP1 SHISA5 DNAJC3 PLOD1 EXT2 HSD3B1 KDSR LRPPRC SPTLC1 TMEM38B SSR2 MARCH6 MGLL PSENEN UGT2B17 HHAT BFAR SPPL2B ITPR1 KIAA0748 FLRT1 CASQ2 JKAMP PANX1 CDIPT POM121C ATL1 HSPA5 TMEM208 CLSTN3 TMEM110 STS TRIM59 CDKAL1 TMEM119 DDOST KIAA0090 MFSD3 SPTLC2 CNIH ATF6 HTRA2 CANT1 AADAC LMAN2L ALG2 GGCX FADS1 SCAP FMO1 PIGU COPB2

GO_MICROBODY_LUMEN The volume enclosed by the membranes of a microbody. HAO2 HSPD1 CRAT ACOT8 ACOXL DAO HAO1 MLYCD PHYH NUDT7 FABP1 IDH1 HSD17B4 DDO ECI2 ACOX1 PAOX BAAT ACOX2 CAT PEX7 LONP2 AMACR AGXT PIPOX NUDT19 FAR2 EHHADH HACL1 ACOT4 AGPS ACAA1 CROT NUDT12 CRYM ACOX3 IDE GRHPR GNPAT SCP2 ACOT6 FAR1 ABCD3

GO_NPBAF_COMPLEX A SWI/SNF-type complex that is found in neural stem or progenitor cells, and in human contains actin and proteins encoded by the ARID1A/BAF250A or ARID1B/BAF250B, SMARCD1/BAF60A, SMARCD3/BAF60C, SMARCA2/BRM/BAF190B, SMARCA4/BRG1/BAF190A, SMARCB1/BAF47, SMARCC1/BAF155, SMARCE1/BAF57, SMARCC2/BAF170, PHF10/BAF45A, ACTL6A/BAF53A genes. The npBAF complex is essential for the self-renewal/proliferative capacity of the multipotent neural stem cells. SMARCC1 SMARCE1 SMARCD3 ACTL6A SS18 SMARCA2 SMARCC2 SMARCD1 SMARCB1 SMARCA4 PHF10 ARID1A

GO_PERICENTRIC_HETEROCHROMATIN Heterochromatin that is located adjacent to the CENP-A rich centromere 'central core' and characterized by the modified histone H3K9me3. DNMT1 HELLS NCAPD3 ESCO2 CBX3 CBX1 CBX5 BAZ1B IKZF1 SUV420H2 KDM4A CENPC1 INCENP H2AFY LRWD1 ATRX

GO_CLATHRIN_COATED_ENDOCYTIC_VESICLE A clathrin-coated, membrane-bounded intracellular vesicle formed by invagination of the plasma membrane around an extracellular substance. SFTA3 INPP5F CLVS2 AP1B1 FCGR1A SFTPA2 DVL2 HLA-DRB1 HLA-DQB1 HLA-DPA1 AP1G1 SFTPD LMBRD1 DNM2 HLA-DRA FCGR1B SFTPC LDLRAP1 HLA-DRB4 CD207 RAB35 AP2S1 TYRP1 HLA-DQB2 CLTC SFTPA1 FZD5 SFTPB AP1M2 EPS15 AP2B1 HLA-DPB1 AP1S1 HBEGF ROR2 AP1M1 FZD4 EPN2 AP2M1 TBC1D5 PICALM AP2A2 CD74 LDLR CTLA4 MYO1E SH3GL2 CPNE6 SGIP1 WNT5A HLA-DQA2 SLC18A3 HLA-DRB3 MYO6 CLVS1 APOB AP1S3 AP1S2 FZD2 AP2A1 HLA-DRB5 CD9 HLA-DQA1 EGFR

GO_BASAL_PART_OF_CELL The region of a cell situated near the base. For example, in a polarized epithelial cell, the basal surface rests on the basal lamina that separates the epithelium from other tissue. PKD2 MET PRCP ERBB2IP AQP5 MYO1A ITGA2 ITGA1 EPS15 HOMER3 MUC20 CLDN4 CEACAM1 ANK3 DOCK7 CD34 CLASP1 GPR77 SLC23A1 PHLDB1 SHROOM4 PHLDB2 P2RY12 HFE AQP1 SLC27A5 ITGA9 OSCP1 CLASP2 ITGA6 KCNQ4 GKN2 TACSTD2 CLDN11 TEK SLC23A2 BMPR2 TF DST CLCA2 MYO1C LDLRAP1 EDN1 FAP ADCY10 SLC11A2

GO_CAJAL_BODY A class of nuclear body, first seen after silver staining by Ramon y Cajal in 1903, enriched in small nuclear ribonucleoproteins, and certain general RNA polymerase II transcription factors; ultrastructurally, they appear as a tangle of coiled, electron-dense threads roughly 0.5 micrometers in diameter; involved in aspects of snRNP biogenesis; the protein coilin serves as a marker for Cajal bodies. Some argue that Cajal bodies are the sites for preassembly of transcriptosomes, unitary particles involved in transcription and processing of RNA. TRIM22 FBLL1 SRRM2 GAR1 FBL FMR1 TGS1 XPO1 TOE1 EAF1 PRPF4 LSG1 LSM10 ANKS1B ELL NARG2 KIAA0947 HINFP USPL1 SNRPC DDX46 NPAT ZNF473 SART1 NOP58 SMN2 ANGEL2 PRPF3 COIL SHQ1 EFTUD2 NOP10 WRAP53 ZNF259 DKC1 HSPB7 FAM118B PHAX SART3 NOLC1 NHP2 PRPF31 DDX42 FRG1 SMN1 ZC3H8 CDK2 SMNDC1 ISG20 RDM1 OIP5

GO_PHAGOCYTIC_VESICLE A membrane-bounded intracellular vesicle that arises from the ingestion of particulate material by phagocytosis. ANXA11 SNX3 HLA-B HLA-E HLA-C ABCA1 SYT11 ANXA3 ATG5 VAMP7 CD36 TLR2 RAB9A LTF TLR7 SYT7 SLC11A1 RAB43 RAB8B ATP6V0E1 RAB9B LAMP2 ATP6V0D1 TCIRG1 RAB32 CORO1A OCRL SLAMF1 RAB23 HLA-A RAB11FIP1 RAB11A HLA-H RAC2 CYBB ITGB5 RAB11B AMBRA1 HLA-F RAB8A RAB34 RAB31 RAB5A TLR6 IRGM ITGAV NCF4 INPP5B CLCN3 CYBA ATG14 ADAM8 TLR9 PIK3R4 FMNL1 DNM2 NCF1 STX12 ATP6V0D2 ATP6V0B UNC93B1 RAB7B DMBT1 NCF2 RAB20 ATP6V0A4 SRGAP2 ATP6V0C UVRAG ATP6V0E2 RAB38 LAMP1 STX6 RAB14 RAB22A SYK RILP HLA-G BECN1 B2M RAB7A ATP6V0A2 ATP6V0A1 PIK3C3 RAB10 ATG12 TLR1 RAB39

GO_HEMOGLOBIN_COMPLEX An iron-containing, oxygen carrying complex. In vertebrates it is made up of two pairs of associated globin polypeptide chains, each chain carrying a noncovalently bound heme prosthetic group. HBM HBA2 HBQ1 HBA1 AHSP HBG2 HBE1 CYB5R3 HBB HBG1 HBD HBZ

GO_ENDOLYSOSOME An transient hybrid organelle formed by fusion of a late endosome with a lysosome, and in which active degradation takes place. CTSB CLTC AP2S1 AP2M1 HRG AP2A2 AP2B1 TLR7 TLR8 TLR9 CLEC16A AP2A1 CLTA TLR3 CTSS CTSL1 CTSK

GO_EXTRINSIC_COMPONENT_OF_MEMBRANE The component of a membrane consisting of gene products and protein complexes that are loosely bound to one of its surfaces, but not integrated into the hydrophobic region. NUCB1 PLDN CTNNA1 FMR1 PIK3C2B C20orf7 ESYT3 JAK1 GNAI2 ASPSCR1 LCK SYTL1 PIK3CA PIK3CB APC2 RGS7 GNGT2 GNAT1 EEA1 VPS13C PIK3R5 NRBF2 EPB41L3 ZAP70 FRK BECN1 BECN1P1 CNR2 ULK1 JUP TEC LYN RACGAP1 ALOX15B SNX9 ST14 PRSS22 EPB41L4B CDK16 ESYT2 STYK1 FGR WIPI2 GNG4 S100A10 TXK GNB1 MYZAP EZR AAK1 STOML2 SYTL4 GNAZ C17orf28 TAMM41 SNX10 EPB41L5 RGS9 PIK3CD PIK3R4 MITD1 TNK2 PLG NCF1 ATG14 EPB41L4A SNX2 SCUBE1 CYTH3 RGS11 GNG12 SRC ATP2A2 USP8 FARP2 EPN3 FRMD5 SRMS BLK MFGE8 SNX8 TDGF1 RS1 MTMR3 BST1 FRMD3 VAC14 STK39 GNAO1 COQ5 RNF40 JAK3 KCNIP1 GNA12 SNX6 CDH1 YES1 ITK SNX11 RGS8 SNX4 RDX GOLGA3 ALOX15 JAKMIP1 RPH3A PRNP MSN NF2 ZC3H12A S100A6 VPS13A WDR45 TF EPB41L1 TIAM1 GNA13 KCNAB1 ATG2B GML SNX18 GNG10 GNAT3 PRSS8 PLAUR RAB7A GNG8 WIPI1 OSR1 PIK3R2 CUBN GNA14 TYK2 MATK SYTL2 GFRA3 PIK3C3 FES ZFYVE1 CTNNB1 RGS6 NMT2 CYTH1 CSK FARP1 PTK2 SNX30 SMAD7 GNAI1 SYK TPR PVRL1 MTOR SOX10 ABL2 WDFY3 TNK1 OPA1 DMBT1 NBEAL1 DNAJA3 HCK SNX32 FCN1 EPB41 MMP27 SARM1 EHD2 SYTL3 DTNA SNX12 NSMAF KCNAB2 WDR45L GNA15 BTK PIK3C2G MAL SNX7 ATG2A SYT6 RAC1 UMOD SNX5 DLG4 MGLL COQ7 NBEAL2 GNGT1 VPS13D CNTFR PIK3C2A PRSS41 SNX16 SNX33 GNAI3 FYN BCL2L11 ERRFI1 PCSK9 MICALL1 ANXA1 APC ANXA2 FERMT2 RHOA GNG5 HIST1H2BA AZU1 NUMB RGS19 ARRB1 SERPINE2 RLTPR GNG7 ABL1 EPB41L2 MYLIP RGS1 CAV2 KRAS PIK3R3 NLRP10 FER MUC16 PACSIN2 PIK3R6 NOA1 PIK3CG SNX1 PML JAK2 GNG3 CYLD GNG13 GNG11 PTK6 IQGAP1 TOR1A TGM3 PAM16 BMX GNG2 PIK3R1 CDH2 SNX3 FOLR3 WDFY4 GFRA2 GFRA1 PTK2B DUSP21 GNAT2 NMT1

GO_N_TERMINAL_PROTEIN_ACETYLTRANSFERASE_COMPLEX A complex that catalyzes the transfer of an acetyl group to the N-terminal residue of a protein acceptor molecule. C7orf52 NAA15 NAA25 NAA30 NAA20 NAA10 NAA16 LSMD1 NAA11 NAA50 NAA35

GO_INTRINSIC_COMPONENT_OF_GOLGI_MEMBRANE The component of the Golgi membrane consisting of the gene products and protein complexes having either part of their peptide sequence embedded in the hydrophobic region of the membrane or some other covalently attached group such as a GPI anchor that is similarly embedded in the membrane. ST8SIA5 B4GALNT1 ST8SIA1 ENTPD4 SAMD8 QSOX2 B3GNT1 SLC35B4 ST3GAL5 ST8SIA4 ST8SIA6 MFNG ST8SIA3 QSOX1 SGMS1 CSGALNACT2 FAM18A CHST2 TBC1D20 RFNG ZDHHC9 ST8SIA2 SYT4 SLC35B2 ST3GAL4 SGMS2 LFNG UBIAD1 SLC35B3 CHST5 SLC39A13 ST3GAL2 IER3IP1 GALNT2 CHST12 SLC35B1 ST3GAL6 CHST4 SYS1 RER1 UNC50 MAN1C1 FAM18B2 CSGALNACT1 LARGE ST3GAL1 ACER3 ACER2 STEAP2 YIF1A ST6GALNAC2 TEX261 PCSK7 A4GALT YIF1B B4GALNT2 ST6GAL1 GOLGA7 LOC100507003 ST3GAL3

GO_MIDBODY A thin cytoplasmic bridge formed between daughter cells at the end of cytokinesis. The midbody forms where the contractile ring constricts, and may persist for some time before finally breaking to complete cytokinesis. SEPT6 PRC1 CYLD KIAA1377 TRIOBP GDI1 TACC1 GEM HSP90B1 SVIL TEX14 ARF6 OR2A4 IQGAP1 FLCN VPS4B TTC23L HEPACAM2 PSRC1 SPAST GNB2L1 RAB11FIP3 SSH1 MYH10 PIN1 SIRT2 GNAI3 VPS37B C7orf11 RHOA ANXA2 AURKA CCDC165 ECT2 ARL8A SEPT1 LZTS2 SEPT7 CLIC4 RAN TXNDC9 RCC2 KIF20A CENPE CENPF CEP44 ERH BIRC5 CDCA8 DNM2 SH3GLB1 CCDC124 RALB DCTN3 PTCH1 SDCCAG3 CTDP1 KATNA1 KIAA1383 PIK3C3 MAK CHMP4C KIF23 ARL3 HSPA5 RAB11FIP4 SCCPDH SPG20 SLC2A1 GNAI1 CTNND1 CAPG VPS4A RALA SPAG5 SEPT2 RDX TOPORS ARL2BP AURKB C6orf89 BIRC6 PITPNM1 KEAP1 ANXA11 KLHL13 KIF13A AURKC CHMP4A KIF20B TTC19 CEP55 KLHL9 MAPRE3 USP8 AGBL5 ALKBH4 TTC28 ZFYVE26 IST1 PKN2 KIF14 ANKRD54 RACGAP1 CDK1 RAP2A RASGEF1B ZFYVE19 INCENP ZNF330 SEPT12 PKN1 UVRAG RNF8 PDXP KIF4A JTB MITD1 PLK1 PPP1CC NUP62 GNAI2 ARL8B NEK2 PKP4 SHCBP1 CHMP4B FAM175B ASPM DDX11 CDC42 KIF3B KATNB1

GO_GAMMA_TUBULIN_COMPLEX A multiprotein complex composed of gamma-tubulin and other non-tubulin proteins. Gamma-tubulin complexes are localized to microtubule organizing centers, and play an important role in the nucleation of microtubules. The number and complexity of non-tubulin proteins associated with these complexes varies between species. BRCA1 MZT2B TUBG1 MZT1 PDE4B TOPORS CENPJ UXT CEP290 TUBGCP2 TUBGCP6 TUBGCP3 CKAP5 BLOC1S2 MZT2A TUBGCP4 TUBGCP5 TUBG2

GO_EXTRACELLULAR_MATRIX_COMPONENT Any constituent part of the extracellular matrix, the structure lying external to one or more cells, which provides structural support for cells or tissues; may be completely external to the cell (as in animals) or be part of the cell (as often seen in plants). LAMA2 ITGA6 NTN4 LAMA3 COL2A1 DLG1 CASK FREM3 THBS4 THSD4 SPN COL4A3 FN1 COL5A1 COL12A1 COL15A1 VWA2 LAD1 SERPINF1 VWA1 DAG1 COL5A2 COL8A2 TIMP1 COL8A1 AMTN MUC5AC SMOC2 VWC2 MFAP5 TGFBI EFNA5 SNCA FRAS1 MMRN2 DCN COL1A1 EGFL6 LAMB4 COL6A1 TIMP3 ADAMTS1 FBLN1 LAMB1 EGFLAM PTPRZ1 FBLN5 SMC3 ADAMTS10 COL14A1 COL11A1 ACHE NID2 COL4A1 LEPREL1 LAMB3 MFAP1 ANXA2P2 LAMC3 COL4A5 ENTPD2 APLP1 NID1 CCDC80 NTN1 LAMB2 FGF9 COL28A1 COL17A1 COL7A1 COL4A2 NPNT MEGF9 SPARC MFAP4 FREM1 FBN1 USH2A RELL2 SMOC1 LTBP1 LUM COL6A3 COL5A3 AGRN CST3 ADAMTSL5 DST COL1A2 HMCN2 PTN LAMC1 TNC THBS2 LAMC2 LAMA1 FBN2 COL18A1 TNXB LOXL1 ODAM LAMA5 ANXA2 COL27A1 TINAG ERBB2IP FREM2 COL4A4 COL11A2 ANG CD151 EFEMP2 SLC1A3 ELN TNR HSPG2 VTN MFAP2 MATN2 HMCN1 LOXL2 LAMA4 COL3A1 COL4A6 COLQ

GO_MEMBRANE_MICRODOMAIN A membrane region with a lipid composition that is distinct from that of the membrane regions that surround it. CD14 NPC1 VDAC1 RANGRF CNR1 TDGF1 KCNE1 MYOF RFTN1 HDAC6 F2R ABCA1 SELE EFNB1 NOS1 CD79A PARK7 FASLG C7orf59 GRIP1 SERPINH1 CDH1 STOM IRS1 NTSR1 SLC22A6 BMPR2 SMURF2 HBXIP ANK2 CD4 PGK1 KCNE3 ADCYAP1R1 PLLP LCP2 CD48 PPP2R1B TRPM8 PI4K2A PTGS2 MAPK3 OLR1 RTN4RL2 NFAM1 LRRK2 PROM2 EFNA5 LCK LRP4 GNAI2 TNFRSF1A RAP2B INSR CBLB CORO1C HK1 CLIP3 PTRF SLC6A3 UNC5A CARD11 RIT2 CBL TRADD PDZK1 PTGIS CAPN2 STOML2 ADCY1 LTB4R EZR EFHD2 S100A10 PRKAR2B HYAL2 BIRC3 LAMP2 BIRC2 KCNQ1 PIKFYVE BMPR1A PACSIN2 GPR56 KDR ACE2 TNR KRAS TNF GPC1 PTPRC GNAI3 DLC1 THY1 ARID3A SLC2A4 RET ANXA2 CDH2 ADCY2 NOS3 ATP2B4 CD55 CDH15 UNC5B GPM6B PTK2B DMD SMPD2 PRKCZ TLR2 JAK2 SDCBP RIPK1 KCNA3 NOS1AP SLC2A1 SPRED1 SCARB1 BSG KIRREL PLVAP CSK PTCH1 PLSCR1 TGFBR2 HSPD1 SLC6A4 PAG1 APP TLR1 BTK MYADM PRKAR1A CTSD KCND2 TNFRSF1B DAG1 MAL STX12 HCK NPHS2 SLC38A9 INPP5D DLG1 BVES EGFR LAMTOR3 DAPK3 PECAM1 FADD MAL2 SHH ADAM17 ATP1B1 OPRD1 FURIN SRC RAP1B FAS LRP6 SLC6A2 MYO1D KIF18A KCNA5 CRB2 ANGPT1 ATP1A2 BCL10 SDPR CAV3 SLC9A1 TRPC4 EEF2 KCNMA1 SCN5A CD36 CDH13 SDC4 IKBKB PRNP STOML3 SORBS1 CAV1 CD1A CD226 SLC9A3R1 MAPK1 RHOQ PSEN1 LAX1 SLC34A1 CASP3 ZAP70 CTNNA1 DLL1 CBLC AKAP6 P2RX3 PODXL LAMTOR1 SMO CFLAR LIPE HTR2A FAIM2 BST2 RGMB ICAM1 LYN PRKAR2A FLOT2 LAT2 TRAF2 TGFBR1 CLN3 OPRM1 ATP1B3 RGS19 PRKCDBP ABCB4 CAV2 EPHB1 ATP1A1 TRPC5 FYN FLOT1 MYO1A BACE1 CD24 S1PR1 EMP2 MYO1C LAT ATP2B1 CXADR RTN4RL1 IQGAP1 STAT6 AHNAK SGCA DPP4 LRP8 PPT1 MALL CNTN1 GHSR GPRC5B CTNNB1 HMOX1 MLC1 P2RY12 GNAI1 PRKACA SULF1 HPSE ARID3C GJA1 EDNRB ITGA1 ITGB1 P2RX1 ERLIN2 RAB5A TLR6 ITLN1 RFTN2 LAMTOR2 LDHB SYNJ2 TEK CASP8 EHD2

GO_NEURON_PART Any constituent part of a neuron, the basic cellular unit of nervous tissue. A typical neuron consists of a cell body (often called the soma), an axon, and dendrites. Their purpose is to receive, conduct, and transmit impulses in the nervous system. EPHA8 KIAA0586 WFS1 ROBO3 SCAMP1 KIF1B MAP2K4 KCNA3 SYT7 DNAJC5 TMEM57 TOR1A GLRX2 PTEN ADCY2 PDE6B SYNGR3 TPRG1L P2RX7 DMD UCN3 ZNRF1 SYT11 TPH1 GLRA3 ARID1B HTR3A LRRC4B ACSL4 KCNK9 KCTD12 SNCAIP NTS CALCA AURKA SLC18A3 PPP1R9A NTNG2 PPP1R1B FAM206A CHRNA7 SEPT5 DNM1L ATP6V0D1 SYNDIG1 TPX2 HTR1B MAG SMN2 GABBR1 CACNA1B SHISA9 TRPV2 USH1G DBC1 CNTNAP2 NRTN GNRH1 UNC5C ELMOD3 S100A4 PTBP2 CYTH2 GUCY2F NQO1 BLOC1S1 EXOC4 SRSF10 ASTN1 APP CHRNB3 FBXO7 SLC6A4 SCN8A ATL1 NRSN1 SYN3 GRM4 STX11 WRN SNCG NAPEPLD PALLD CTNND1 KIRREL ARHGAP32 SPG7 NCS1 DLG2 SNAP23 RDX RGS8 MAGEE1 AIPL1 CALB2 MAGOHB SYT9 PALMD CASP5 ARR3 VTI1B MTPN BRSK1 AVIL TIAM1 CHRM3 CTSL2 RGS14 LRRC7 FLRT2 C4B STAR ERO1L AIF1 MKKS MAPT DLG3 IL1RAPL1 HTR6 ZNF259 CNTN4 GRID2IP ASCL1 MOB2 FAM168B KCNN1 ZFYVE27 TACR3 PFN2 LRRC4 UTRN SYTL5 CCR4 ANKRD27 ZDHHC17 FMR1 PVALB PTGS1 SNX14 IAPP SCRG1 HAP1 KCNQ2 AXIN1 CACNA1C GNAT1 SLC32A1 DENND1A CCDC120 C4orf49 KCNE3 IFT52 IDO1 SYNPO KATNB1 IL31RA MTMR2 ADCYAP1 PARK2 SLC2A8 FSCN1 NLGN4Y CACNG8 TMC2 KCNK1 PPT1 OPN1MW2 HTR7 CXADR SHANK2 ATP2B1 ZMYND8 CA2 ACAD9 GNAT2 RBFOX3 RIN1 AMPH RGS10 HSPA8 HOMER3 HIF1A FYN CNGB3 RIMS4 STRN4 OPN5 STAU1 RAB3A RAB27A ARRB1 C4A ENDOG EEF1A2 TMPRSS3 ITPKA CADM1 STX3 CX3CR1 KPNA1 BCR DNER BOC EPHB3 FAM126A REEP6 SYT13 NOV SARM1 GABARAPL1 GRIK2 SYTL3 GAP43 MFF KCNA6 SLC17A5 RAB5A SERPINF1 DNM2 TAC1 CNTNAP4 P2RX1 KCNJ14 KCNIP3 KCNK2 ODZ4 GIP CALM3 KPTN BSN LIN7A HTR2B PACSIN1 C19orf26 OPN1LW KIF1A SYN1 SYNM AP1S1 MTOR PVRL1 SYT15 NF2 DPF1 KIF5A SYT5 CNTNAP1 VDAC2 CNKSR2 HERPUD1 CYFIP2 KCNC2 NF1 DDN FAS CPLX3 ABAT ARF1 SLC1A4 CEACAM16 LSM4 IFT20 NEK3 KCNN3 HCN1 TRPA1 OR10J6P FADD PDZD7 HTR5A SRCIN1 TGFB1 LHFPL5 PCDH9 GNB1 NPBWR1 RPLP0 LAMA2 CACNG5 ELFN1 HEPACAM AMIGO1 SEZ6L ARG1 HTT CTTNBP2 KCTD16 GRK7 PQBP1 P2RX4 NETO1 BASP1 PSD3 VAMP3 GHRL ADORA1 STRC OR10H4 SLC9A3R1 MBP CNN3 GLRX3 ADRA2C LSM1 BRD1 ITSN1 RAB17 SSTR3 SPOCK1 KIF3B SKOR1 PRKCZ APOD ADNP SH2D5 NEURL MAP3K12 ARHGEF2 PJA2 ATP2B4 GRIA4 SPTBN1 SMN1 MIB1 RPTOR USH2A CNGA3 C1orf130 PTK2B PRKCG STX2 OR10J5 CDHR1 SEPT11 CYBB AVP HCN3 PSD2 SLC12A5 ABL1 SEMA4C SHANK3 SLC9A6 KIF5C PMM1 SLC8A3 BMPR1A FBXO2 RGS9BP MPL RPS6 STX1A TBX21 MUL1 OTOF CASK DLG1 LPAR3 GUCA2B CADPS UNC13B CCK ADCY9 S100A5 APBB1 RIC3 ADAM22 DLGAP1 LDLRAP1 PIP5K1C PDE10A DLG4 TIMP2 TNFRSF1B SEZ6 AP3B1 STX1B SYT3 RAB7A HTR1F NGDN TMOD2 TSGA10 TSHZ3 ENO2 PAK1 NETO2 KLHL20 ABCC8 GPI ROBO2 C1QL1 AP3S2 FRMD7 CACYBP GRK4 LYNX1 NLGN4X LIN7B CPE EFNA2 BMPR2 SYT4 GPR44 NUMA1 DAGLA UBB PPARGC1A KCNA2 SSTR5 CANX CDH23 FBXO31 MINK1 SMARCD3 AGRP NOS1 KLC1 KCNAB1 ANXA3 CREB1 EIF2C2 GRXCR2 LPHN1 PSEN2 RAB39B QDPR ANK1 RAB3C SOS1 OXT FAM5B GRM1 NMB VDAC1 RASGRF1 IFNGR1 GPRIN1 HTR1A KCNH1 CAMK2D SLC31A1 MPDZ VAMP2 CAMK2N1 MAP1B GRIN2B GRIN3A SCN11A SLC4A8 SVOP EPHA5 NRSN2 KIF4A S100A1 ERC1 RCVRN DOC2B PTPRF C19orf20 UNC5A BIN1 BGLAP SLC6A3 UBXN1 GABRA5 CAMK1G MERTK ARFGEF2 TMC1 CACNA1F LRP4 OR6T1 PKP4 PDE2A DCC FSTL3 TULP1 PDE1B KCNB1 STX4 ANK2 BECN1 DFNB59 MPST CROCC SYNJ1 GNG3 PICALM CYGB RHO DGKI STRN HOMER1 MAGI2 GNAQ GPM6A GRIN2A CPNE6 DST SLC6A9 GIPC1 DCX GPER CPT1C SYNGAP1 HTR2C FAM5C BZRAP1 TRPC5 NFIB CLIC5 NRGN GOPC NAAA STX19 CIB2 S100B ARMS2 RGS19 CADPS2 PCDHGB1 HNMT PPP1CA CHRM1 NTRK1 OPN1MW ARC APOB PTPRN2 RABAC1 WDR7 IQCB1 SEMA3A SLC30A3 SIAH2 NRXN2 KCNAB2 DBNL FABP7 MAP1S UNC13A YKT6 HCRT USH1C ProSAPiP1 CABP1 DRP2 ELK1 KCNA4 ITGB1 PLK2 RAP1GAP2 PDC DPF3 OR10H3 CPEB1 PDPK1 SYT8 ODZ1 ARF4 KISS1 SLC6A17 ACTN2 MAK EIF4A3 CNIH3 GHSR RIMS1 ADCY4 SLC38A2 CDH13 ERC2 SAMD4A NEFM CNO STRCP1 PDE4B PPFIA3 SSTR2 TRPV1 ACADM OSBP2 SAG C1orf96 RP1L1 TMEM100 KIAA1598 MLPH VPS16 BAIAP3 GRID2 SLC6A2 SMURF1 CALM1 RP1 ATP1A2 KLHL17 IGSF9B CAPRIN1 BRAF C20orf30 NANOGNB RPGR PMM2 DMXL2 GNB5 VTI1A NELF KCNIP1 OPRL1 CHRNB4 SRD5A1 ZNRF2 GPR1 PTPRN POU4F1 NLGN2 MAX ABCA4 MPP1 PACRG MICALL2 ZWINT P2RX3 DFNB31 SUMO1 ARPC5 GLRA1 OR11H4 ABI1 AP3D1 PPFIA1 CPNE5 SPTBN2 LMTK3 GDPD5 SLC22A2 PFN1 BBS2 SLC6A13 EPB41L3 KCNQ3 PYCARD TMEM163 FOSL1 NGB ODZ3 APOE FKBP4 PALM DAB1 DRAM2 RELN SLC17A8 IGSF9 GOT2 MYH10 SPAST SIRT2 TXN2 ORAI2 RET STON2 KIRREL3 LGI3 PRKAR2B IL6ST FZD3 DHRS3 CRYAB KLHL24 PPFIA2 MT3 CSNK1D ITPR1 IFNG FLRT1 GPR149 ALCAM NGEF DTNA HNRNPA3 CACNA1A RGS20 TIAM2 PDE6A DAG1 KIF5B EFHC1 KCND2 COPS5 FLNA SNAPIN PHLPP2 TNFRSF21 NCAM2 SMARCA4 SCGN ITGA8 POLR2M GPR98 RNF6 CNGA1 CPEB2 GSK3B L1CAM BBS4 PCDH15 WNT7A PARK7 TRIM9 RRM1 ICA1 DOCK4 CRH GRM2 NTSR1 PROM1 PURA BNIP3 MYRIP RAB3B BAG3 CLU GRIN1 FSCN2 EXOC7 UNC13C GNAS SRR SNAP25 CTNNA2 SNAP29 RAB11A GRIA1 FAM65B LLGL1 HDAC6 SV2B NPBWR2 NAPA YWHAE NSF PDE6G SMARCC2 SYNGR1 SCN1A RNF40 DLGAP2 RIT2 CBL MCRS1 BAIAP2 FUS OPHN1 OR5T2 CRTAC1 SYTL4 SLC18A1 CDH8 ATP2B2 ZDHHC5 HPN APBB2 PREX1 SS18L1 NPY5R GCHFR FEZ1 AP3M2 HOMER2 GAD1 ARHGAP4 ADCYAP1R1 KCNJ2 PTGS2 LPHN3 PI4K2A SYT2 KLHL1 PINK1 NRP1 SETX HNRNPR CRIPT NRCAM SACS STMN3 ATAT1 PLXDC1 C19orf2 AMFR MYO1C NRXN3 NRN1L MAPK8IP2 GRM3 GNB2L1 ERMN INPP5K CORO1A GRM6 KCNN2 BCL11B RAB8A BACE1 TH STMN1 CHL1 NCOA1 OR5T3 SLC1A3 EPHB1 GTF2I CPLX2 IGHMBP2 ARID1A LOXHD1 ATP6V1G2 SLC17A7 SRGAP2 CPEB4 INHA SYNJ2 HPCA SST FXR1 OMP ODZ2 POLG TPH2 DTNBP1 PENK RANGAP1 SLC6A1 CTNND2 ANKS1A WLS SHARPIN SMARCC1 CAD CSNK1E KLC3 PDE1A FKBP15 NRG1 SYNGR4 AKAP9 MYOC GABRB1 ENC1 EEF2K SCAMP5 TRPM5 KCNA1 CHRM2 BPTF ROM1 ATCAY RDH12 BLOC1S3 MAPK8IP1 NEUROG1 PDE11A CNTN2 LRIT3 PPFIA4 CD27 GNB3 ZNF385A AP3B2 SLC8A1 SMARCD1 DISC1 WNT3A IFT57 MYH14 HRH3 DOC2A STAT1 TOP1 RPL28 UFL1 SV2C SLC17A6 KIF20B GPHN SLC38A7 ITGA2 PTPRK SRD5A2 MYO1D PICK1 LRP6 CPEB3 EPS8 ATF4 ANKS1B KCNC1 PPP1R9B MME NEFL PARD3 TWF2 SMARCA2 GNAO1 BAI1 GARS STMN2 ANG ATP8A2 SCN2A UCHL1 GAL AAK1 WDR81 GNAZ DPYSL5 RGS9 NFASC BLOC1S2 LRFN3 GUCY2D SIGMAR1 BMPR1B PLDN ACVRL1 MAPK8IP3 PTPRO LIMK1 ATP1A3 MYL7 IGF2BP1 PLCB4 TRPM2 APBA2 STXBP1 PNOC GLRX5 RBM8A SNCA CIB1 CAMK2A PSEN1 MAPK1 CPLX1 RPS6KB1 NAIP DVL1 TBC1D24 SLC4A10 EPHA7 PCDH8 FOS SYN2 ATP13A2 DAB2IP GRIK4 ITPR3 PLK3 ACCN1 GRIN2C ARHGEF15 GDI1 SNPH CHRNA10 GLRB ESPN PDZD11 GOT1 LPAR1 DNM1 SPTA1 JPH4 DPYSL3 PAFAH1B1 ATP8B1 THY1 CYP17A1 SLC4A7 COPS4 NDN PTPN13 CCR2 AP3S1 PPP5C EXOC8 SMARCB1 P2RY1 SHANK1 ASTN2 CDK5R1 TANC1 RUSC1 IFT140 PLD2 OPA1 SCN1B MAF1 TSC1 VIM SNAP47 ARHGAP44 TTLL7 SEMA6A SLC18A2 GRM5 KCND3 APBA1 DLGAP3 GRM7 PSPH NPFF KCNC4 GUCA1C PRSS12 PCSK2 TUBB3 LRFN1 MYO15A NLRP1 RAB13 PTCH1 STXBP5 ILK HCFC1 DBN1 SPTBN5 DHODH SLC6A11 SGCE STMN4 OR10H2 TRPM4 GRM8 GRIP1 SSTR4 CNTF ADA RPH3A ARPC2 CALM2 STRN3 GPX1 BBS7 GRXCR1 SYT12 KLHL14 VAMP7 AGFG1 RGS17 NCDN OCRL MUTED IRX3 DOCK7 NGFR CNIH2 NUFIP1 RASGRP2 CNR1 SYT10 BCL2L1 GLUL SYNPR SLC25A27 MYO7A OTX2 SORT1 CAPN2 COBL MAP4 CDK16 PCLO CTTN DCTN2 INPP5F TAOK2 RIMS3 OR10H1 HTR1E NCF1 GAD2 FAM107A CYBA TRPV4 DSCAM SPG11 NRXN1 INPP5J TSPEAR GNAI2 TUBB4A TMEM185A LRRK2 LRP1 LAMP1 STX6 RBM3 SOD1 WDR19 GABRG2 KCND1 CDK5 PDYN CNR2 OR11H7 RAP1GAP LIN7C SEPT6 OPN1SW TPRN GNG13 ATP7A DPYSL2 PHAX LRP8 CHAT CRTC1 IQGAP1 MARK4 OLFM1 ADAM10 CALB1 RUFY3 PRPH2 SIPA1L1 NEFH C2orf71 SLC40A1 DNM3 PIN1 GABRA2 NLGN1 MAP2 OPRK1 NMU ROBO1 MYO5A GNGT1 GHRH RGS12 AHCY SYT1 ACTN4 C20orf103 KNDC1 CHRNA3 RDH11 NDRG2 RAC3 KCNJ11 SLC5A7 OPRM1 CLN3 TGFB3 AP3M1 RIMS2 SYP SV2A SYNGR2 MOB4 FBXO45 KCNJ10 FGF13 CASP8 ATXN1L HTR1D GNA11 SRI NDEL1 CCL2 ARRB2 SYPL2 USP9X ACTL6B SYT6 SYT17 GRIK3 GRIK5 ITGA1 GUCA1B UHMK1 KIF13B SSTR1 SCRIB DRD2 CYFIP1 DCDC2 LRRTM1 UCN FARP1 RARA KCTD8 SLC8A2 RTN4R AQP11 ELOVL5 ITGA3 CIT PIAS3 AP2M1 OR10H5 BDKRB1 CDKL5 RGS2 CADM2 ARHGEF7 CST3 CNGB1 ALS2 ADCY10 RAB21 NR1D1 EPHA4 KNCN EPHB2 TMPRSS5 SRC NDUFS7 ASAP1 NTRK2 RAB11B CRHBP PAM PPEF2 NPTN DICER1 HSP90AA1 OPRD1 GRK1 ADC FRMPD4 VAMP1 PDE9A GLRA4 SYPL1 LYN TRAPPC4 SH3KBP1 PDLIM5 GABBR2 SPTBN4 AHCYL2 DDC BRS3 SLC1A2 FLRT3 TGFB2 COMT RAP1A SMARCE1 CLCN3 HDAC1 RAPGEF2 CHRNA4 HTR2A GUCA1A ARPC3 LZTS1 PPP1CC ANK3 KCNB2 SYTL1 ASS1 SLC6A12 EEA1 ADAM21 CDC42 TP63 AXIN2 CABP4 ATXN10 GRIN3B

GO_MKS_COMPLEX A protein complex that is located at the ciliary transition zone and consists of tectonic proteins, B9 domain-containing proteins and other proteins. Acts as a barrier that prevents diffusion of transmembrane proteins between the cilia and plasma membranes. In mouse, members of the complex include TCTN1, TCTN2, B9D1, MKS1, CC2D2A and other proteins. TMEM231 TMEM67 TMEM17 B9D2 B9D1 TCTN2 AHI1 TMEM216 CEP290 MKS1 CC2D2A TCTN1

GO_HIGH_DENSITY_LIPOPROTEIN_PARTICLE A lipoprotein particle with a high density (typically 1.063-1.21 g/ml) and a diameter of 5-10 nm that contains APOAs and may contain APOCs and APOE; found in blood and carries lipids from body tissues to the liver as part of the reverse cholesterol transport process. CETP SAA2 APOA2 SAA1 HDLBP APOL1 PON1 APOA5 APOM APOC4 APOC1 APOE APOH GPIHBP1 APOA1 APOA4 APOC2 APOC3 APOF CLU HPR ABCA1 APOO LIPC SAA4 LCAT

GO_CHROMOSOME_CENTROMERIC_REGION The region of a chromosome that includes the centromeric DNA and associated proteins. In monocentric chromosomes, this region corresponds to a single area of the chromosome, whereas in holocentric chromosomes, it is evenly distributed along the chromosome. CLIP1 PKHD1 CBX1 SKA1 PAFAH1B1 TEX14 DYNLT3 MIS12 MEAF6 CENPP RASSF2 ERCC6L C9orf114 MTBP STRA13 PPP2R5C SEPT6 CENPC1 CBX5 APITD1 CKAP5 RAD21 SGOL1 PHF6 PPP2CA SMC1B STAG2 CLASP1 ZW10 NDE1 CEBPB TTC15 CHAMP1 APC AURKA NUP85 NUP133 ZNF276 SEPT7 XPO1 PINX1 REC8 BUB1 OIP5 KAT8 SYCP1 DCTN3 RANGAP1 NDEL1 BOD1 BIRC5 CDCA8 NDC80 CENPQ CENPF LRWD1 KIF22 MAD1L1 DYNC1LI1 ZWILCH CLASP2 SGOL2 STAG1 RCC2 NUP160 CENPE SUV39H2 CENPK PDS5A CENPI CFDP1 TPR CDCA5 CENPV NCAPD3 NUP43 CENPA CTCF NSL1 MIS18BP1 PHF2 NGDN NUDCD2 WDR43 SUGT1 NCAPD2 BAZ1B ITGB3BP BUB3 SEH1L MKI67 FBXO28 BOD1P AURKC STAG3 DNMT3A LOC728637 PDS5B PPP2R5A TNKS SUV39H1 NUP107 CSNK1A1 DYNLL1 SEC13 SKA2 SUMO3 SMC1A DSN1 PPP2CB AURKB SUV420H2 SIN3A CENPH SPAG5 SEPT2 CENPL NUP98 CENPB PMF1 KAT2B TP53BP1 DNMT1 ZNF207 KNTC1 MIS18A DCTN1 SPC24 ATRX SUV420H1 CENPT DCTN5 PSEN2 KIF18A PPP1R12A CENPM ORC2 SS18L1 SYCP3 DSCC1 BUB1B NUP37 CCDC75 HJURP C15orf23 KIF2B CBX3 ZWINT WAPAL DCTN2 ZNF330 KIF2C CCNB1 WDR81 UVRAG KIAA1267 KDM4A DYNC1I1 DCTN6 IKZF1 H2AFY INCENP MAD2L1 ANAPC16 NUF2 SMC3 PSEN1 HELLS MLF1IP ESCO2 PPP2R1A SKA3 CENPO DAXX CASC5 NEK2 SPC25 CENPN FMR1 PLK1 AHCTF1 CENPW CCDC99 PPP1CC

GO_ZYMOGEN_GRANULE A membrane-bounded, cytoplasmic secretory granule found in enzyme-secreting cells and visible by light microscopy. Contain zymogen, an inactive enzyme precursor, often of a digestive enzyme. RAB3D DMBT1 CLCA1 VAMP2 TMED10 CUZD1 ZG16 GNAI3 TMED2 STX3 RAB27B SCAMP1 STXBP2 HSPD1 SRGN

GO_GOLGI_CISTERNA Any of the thin, flattened membrane-bounded compartments that form the central portion of the Golgi complex. ST3GAL3 ST6GAL1 B4GALT1 TMEM59 FUT3 BCAP31 SORL1 GOLGA5 ST3GAL1 ARAP1 CSGALNACT1 GOSR1 RAB34 INPP5E B4GALT6 GOLGA8B LLGL1 GOLGA1 B4GALT3 TMED3 BET1 SORT1 FUT9 B4GALNT3 B4GALT5 FUT11 GOLGA3 ST3GAL2 A3GALT2P COG3 GOLPH3 AP4M1 B4GALT4 GPR89A GGTA1P B4GALT2 CIT NECAB3 GAL3ST4 SGMS1 FUT8 UXS1 FUT10 GAL3ST2 FUT4 FUT6 CHPF PITPNM1 RAB21 STX16 CANT1 SAR1B GOLGA2 GALNT1 NUCB1 TMEM115 TMED2 B4GALNT4 FUT2 GCNT1 ATL1 CHSY3 GALNT3 CHPF2 GOLPH3L GALNT2 YIPF5 B3GALT6 FUT5 SCFD1 MOB4 FUT1 HACE1 ABO GOLGA8A ST3GAL4 CSGALNACT2 ST6GAL2 GOLIM4 APH1A FUT7 SMPD3 C17orf28 RAB30 NAGPA CHSY1 HMP19 PSENEN B4GALT7 GAL3ST3 ASAP2 GOLGA8IP

GO_CYTOPLASMIC_SIDE_OF_MEMBRANE The side of a membrane that faces the cytoplasm. GNAZ DIABLO TRAF6 HM13 RGS9 TNK2 RASA1 PTPN22 LYN TEC EEF1A1 RACGAP1 CDK16 SNX9 ESYT2 STYK1 TXK GNB1 GNG4 FGR TRAF2 EZR MYZAP KIT CHMP4B SPPL2A PTPN1 FRK ZAP70 CNR2 DAB2IP JUP ESYT3 JAK1 LCK ASPSCR1 GNAI2 RGS7 GNGT2 LRRK2 PKP4 G6PD GNAT1 RGS2 TIAM1 KCNAB1 GNA13 CHMP4A RAB21 SNX18 GNG10 YES1 CDH1 GM2A GNA12 ITK ALOX15 RGS8 PTPN7 IKBKB NTSR1 S100A6 MIEN1 PGM5 RASA2 GNAO1 JAK3 DSG1 CD2 GFAP KCNIP1 NF1 CYTH3 PKD2 GNG12 RGS11 ZFYVE20 SRC RASA4 CHUK ATP2A2 SRMS BLK GNA15 KCNAB2 LDLRAP1 BTK LOH12CR1 DNAJA1 RAB5A RASA3 SNX5 PTPN3 DLG4 TRAF3 TNK1 SPPL3 HCK SPPL2B DLG1 IMP5 DTNA PTPN4 NPHS2 RGS6 SHROOM4 CYTH1 RASA4B FARP1 PTK2 CSK GNAI1 SYK ABL2 PTP4A1 GNAT3 HTRA2 GNG8 GNA14 MATK TYK2 FES MAP2K2 SPTB IQGAP1 ATP2B1 TGM3 BMX ACP1 GNG2 RASAL2 PTEN TRAF5 SPTA1 PTK2B RASAL3 GNAT2 JAK2 GNG3 GNG13 CYLD GNG11 RASAL1 GEM PTK6 RGS19 ARRB1 RLTPR BIRC2 GNG7 ABL1 EEF1A2 RNF31 CAV2 RGS1 KRAS EXOC1 FER GNRH1 GNGT1 SYNGAP1 GNAI3 ERRFI1 FYN GNB1L RHOA FERMT2 TH CACNB4 GNG5

GO_AXON The long process of a neuron that conducts nerve impulses, usually away from the cell body to the terminals and varicosities, which are sites of storage and release of neurotransmitter. NRCAM SYNJ1 DGKI HNRNPR SEPT6 ADCYAP1 LRP8 DPYSL2 HOMER1 SACS PPT1 ATAT1 STMN3 CPNE6 DST CA2 RUFY3 CALB1 IQGAP1 OLFM1 GPM6A OPRK1 GABRA2 GRM3 DNM3 NMU ERMN ROBO1 NEFH NRN1L NFIB CORO1A RGS10 CPT1C GPER HIF1A GHRH TH BACE1 RAB3A SYT1 NRGN C4A NTRK1 CHRM1 SLC5A7 KCNJ11 CPLX2 AP3M1 PTPRN2 SYP IGHMBP2 STX3 EPHB1 HPCA BOC SEMA3A FXR1 OMP SLC17A7 SYNJ2 SV2A GRIK2 POLG DTNBP1 NOV FGF13 SARM1 KCNA6 SLC6A1 RAB5A GAP43 SRI KCNAB2 NDEL1 RANGAP1 CCL2 UNC13A PENK TAC1 KCNA4 SERPINF1 ELK1 FKBP15 NRG1 CAD GRIK3 GRIK5 KCNK2 MYOC KIF13B KCNIP3 UHMK1 BLOC1S3 PACSIN1 LRRTM1 MAPK8IP1 KCNA1 DRD2 ATCAY BSN CHRM2 PVRL1 KIF1A AP1S1 SYN1 SLC38A2 UCN CNTN2 AP3B2 CNO MYH14 DISC1 NEFM RTN4R AP2M1 STAT1 CST3 CNGB1 ALS2 CADM2 ACADM KIAA1598 VPS16 KIF20B EPHA4 ADCY10 CNTNAP1 RAB21 C1orf96 SMURF1 EPHB2 NF1 KCNC2 ITGA2 PTPRK SLC38A7 MYO1D NTRK2 CRHBP MME HCN1 NEK3 NEFL DICER1 PARD3 STMN2 GARS OPRD1 ADC VAMP1 UCHL1 SCN2A SRCIN1 SPTBN4 WDR81 AAK1 FLRT3 PTPRN TGFB1 SLC1A2 DDC P2RX3 HTT AMIGO1 TGFB2 HEPACAM COMT NFASC BLOC1S2 LRFN3 HTR2A P2RX4 IGF2BP1 ATP1A3 PLDN PTPRO MAPK8IP3 CIB1 SNCA ADORA1 GDPD5 AP3D1 ANK3 LMTK3 GHRL PNOC STXBP1 MBP ADAM21 DVL1 MAPK1 PSEN1 CPLX1 EEA1 KCNQ3 DAB2IP KIF3B SPOCK1 CABP4 LSM1 ADRA2C TBC1D24 EPB41L3 ADNP PRKCZ KCNA3 MAP2K4 DNAJC5 PALM FKBP4 SYT7 ROBO3 ODZ3 KIF1B MAP3K12 IGSF9 SLC17A8 SPTBN1 CHRNA10 TMEM57 SYT11 PTK2B MYH10 SPAST PAFAH1B1 GOT1 UCN3 C1orf130 SPTA1 DNM1 NTS HTR3A LRRC4B CYP17A1 SIRT2 CHRNA7 RET SEPT5 AURKA SEPT11 CALCA NTNG2 SLC18A3 AP3S1 TANC1 ATP6V0D1 TPX2 HCN3 KIRREL3 CDK5R1 NRTN CNTNAP2 GNRH1 SLC9A6 CRYAB FZD3 MAG TRPV2 MT3 OPA1 KLHL24 MAF1 SCN1B CCK APBB1 ADCY9 DTNA UNC13B ADAM22 DLG1 SLC18A2 LPAR3 SEMA6A MUL1 ALCAM GRM7 LDLRAP1 DLG4 TNFRSF1B PRSS12 KIF5B DAG1 NPFF KCNC4 TUBB3 SNAPIN PTCH1 RAB7A BLOC1S1 AP3B1 SCN8A NCAM2 SRSF10 ILK TNFRSF21 NGDN APP HCFC1 STMN4 KLHL20 ROBO2 PAK1 ATL1 RNF6 PALLD AP3S2 C1QL1 SNCG PARK7 CNTF DLG2 CALB2 L1CAM SPG7 NCS1 DAGLA NTSR1 KCNA2 CRH GRM2 SYT4 MINK1 AVIL GRIN1 TIAM1 CANX MTPN NCDN LPHN1 CTNNA2 KCNAB1 KLC1 ANXA3 CREB1 ANK1 GRIA1 CHRM3 RAB11A OXT IRX3 LLGL1 MUTED DOCK7 HDAC6 C4B CNR1 SYNGR1 NAPA YWHAE RNF40 KCNH1 MAPT IL1RAPL1 CAMK2D GLUL SCN1A COBL CNTN4 ZNF259 INPP5F EPHA5 OPHN1 SCN11A FAM168B ZFYVE27 PFN2 CDH8 SLC18A1 KIF4A SLC6A3 GAD2 C19orf20 BIN1 FEZ1 SPG11 DSCAM FMR1 PVALB LRRK2 AP3M2 KCNQ2 SLC32A1 HAP1 TUBB4A TULP1 SOD1 GABRG2 KCNB1 STX6 C4orf49 DCC KATNB1 IL31RA MTMR2 NRP1 SETX PINK1 RAP1GAP LPHN3 CDK5 PDYN

GO_CALCIUM_CHANNEL_COMPLEX An ion channel complex through which calcium ions pass. TRPC4 C1orf101 PKD2L1 HERPUD1 CACNG2 NOS1 CATSPERG CACNA1E CACNA1D CACNG4 CCDC109B HSPA2 C22orf32 CALM2 CACNA1A RYR3 PDE4B CACNA1G CASQ2 TRDN PKD1L1 RYR2 CACNA1H CACNG8 FKBP1B CACNA1S CACNG3 MCU CATSPER3 CATSPER4 CACNA2D3 CAMK2D EFHA1 CACNG6 RYR1 PRKACA CACNA1B CACNA2D2 CACNA1I CATSPER2 NOS1AP CACNB1 SESTD1 CALM3 TMEM146 PPP2R4 CACNG1 CACNB4 CACNA1C CATSPER1 CACNG7 AKAP6 CACNB3 MICU1 CALM1 CACNA2D4 TRPC5 CACNA1F ATP2A1 PDE4D CACNA2D1 CATSPERB CACNB2

GO_LATERAL_PLASMA_MEMBRANE The portion of the plasma membrane at the lateral side of the cell. In epithelial cells, lateral plasma membranes are on the sides of cells which lie at the interface of adjacent cells. RAB13 GJA1 NKD2 MYO1A PTPRO AXIN1 CLDN4 CEACAM1 APC ANXA1 CCDC165 ANK3 IQGAP3 CLDN7 CLDN12 D4S234E ATP6V1B1 BVES DVL1 KCNB1 EPCAM SNTA1 CTNNB1 DRD2 GJB2 ABCC6 CLDN1 DSG1 TBCD JUP FZD3 SCN5A ERBB3 ARL2 CDH1 CLDN15 CLDN3 TACSTD2 C1QTNF5 DLG1 PKD1 MARK2 MYO1C CORO1C IQGAP1 VANGL1 SLC26A5 DSG2 DMD VANGL2

GO_MALE_GERM_CELL_NUCLEUS The nucleus of a male germ cell, a reproductive cell in males. MLH1 HSPA2 MLH3 TNP1 HILS1 TRIP13 TCFL5 ACTL7A KIF6 TNP2 SYCP1 H2AFX TOPBP1 ARPM1 REC8

GO_SECRETORY_GRANULE_MEMBRANE The lipid bilayer surrounding a secretory granule. VAMP1 FAM170B TEX101 STX3 CAV2 PTPRN2 SELP PECAM1 LAMP3 TMEM190 SUN1 PAM RAB27A LAMP2 CD9 ZP3 ANXA7 SYT1 CD63 VAMP8 PLA1A AZU1 SLC30A8 RAB26 DBH VAMP7 C9orf11 IZUMO1 SYT4 ICA1 SCAMP1 ITGB3 SYT9 CD46 SLC11A1 SPARC RPH3AL ITPR3 CD36 ATP8A1 ITGA2B RAB27B SYT2 CPE ATP8B3 BSG SYCN TMED2 ABCA3 CAV1 ACRBP SNCA PHACTR2 TMED10 CYB5R1 SLC30A5 TMX3 ZG16 RND2 PCDH7 ADAM8 STXBP2 SPACA3 SERPINA5 ABCC4 ITPR2 PCSK4 TMEM184A CLCA1 TEKT3 SRI TMEM225 APLP2 TRIP11 SPACA1 FLOT2 DMBT1 VAMP2 CA4 ITPR1 CUZD1 EXOC3

GO_DNA_DIRECTED_RNA_POLYMERASE_II_HOLOENZYME Large protein complex composed of the RNA polymerase core complex and a variety of other proteins including transcription factor complexes TFIIA, D, E, F, and H which are required for promoter recognition, and the Mediator subcomplex. Catalyzes the synthesis of eukaryotic pre-mRNA. TAF5L TRRAP GTF2H2 MNAT1 POLR2H TAF8 MMS19 TAF7L TAF9 POLR2A INTS7 RECQL5 GTF2A2 TAF1L TAF1 WDR61 CTDP1 RTF1 RPRD1B LEO1 POLR2M GTF2H1 ERCC3 INTS2 MED14 GTF2F2 GRINL1B SUPT3H POLR2I INTS12 GTF2H4 TAF3 GTF2F1 CPSF3L MED4 TAF10 POLR2G ERCC1 TP53 POLR2J INTS3 PAF1 EDF1 INTS6 POLR2F TAF9B POLR2E TAF6 RPRD1A TAF5 TAF12 KAT2A MED6 TAF11 INTS4 POLR2D TBPL1 CDK7 GTF2E2 POLR2J2 RPAP2 GTF2A1L MED10 CHD6 POLR2B TAF4B INTS5 POLR2K SHFM1 MYO6 ERCC4 ERCC5 PEX2 POLR2C GTF2A1 TBP TAF2 MED18 CTR9 TADA3 CDC73 TAF7 POLR2L ZNF768 RPRD2 INTS9 TAF13 C19orf2 ERCC2 MED31 INTS10 TAF4 PPARGC1A CCNH INTS8 GTF2H3 INTS1

GO_PHOSPHATIDYLINOSITOL_3_KINASE_COMPLEX A protein complex capable of phosphatidylinositol 3-kinase activity and containing subunits of any phosphatidylinositol 3-kinase (PI3K) enzyme. These complexes are divided in three classes (called I, II and III) that differ for their presence across taxonomic groups and for the type of their constituents. Catalytic subunits of phosphatidylinositol 3-kinase enzymes are present in all 3 classes; regulatory subunits of phosphatidylinositol 3-kinase enzymes are present in classes I and III; adaptor proteins have been observed in class II complexes and may be present in other classes too. PIK3R5 PIK3R1 NRBF2 PIK3C2G BECN1 BECN1P1 PIK3R4 PIK3R3 VAC14 PIK3CD ATG14 PIK3R6 MTOR PIK3C2A PIK3C2B PIK3CG PIK3CA PIK3CB PIK3R2 PIK3C3

GO_VOLTAGE_GATED_CALCIUM_CHANNEL_COMPLEX A protein complex that forms a transmembrane channel through which calcium ions may pass in response to changes in membrane potential. CACNG2 C1orf101 CATSPERG NOS1 CACNA1E CACNA1D CACNG4 HSPA2 CACNA1A PDE4B CACNA1G TRDN CASQ2 CACNG8 CACNA1H CATSPER3 CACNG3 CACNA1S CACNA2D3 CATSPER4 CACNA1B CACNA2D2 CACNG6 CACNB1 CACNA1I NOS1AP CATSPER2 CACNG1 TMEM146 CACNG7 CACNB4 CACNA1C CATSPER1 CACNA2D4 CACNA1F CACNB3 CACNB2 CATSPERB PDE4D CACNA2D1

GO_RIBOSOMAL_SUBUNIT Either of the two subunits of a ribosome: the ribosomal large subunit or the ribosomal small subunit. RBM3 RPL41 RPL13 RPL24 RPL18A NSUN3 RPL15 MRPL55 MRPL52 C7orf44 RPL3 MRPL11 RPS28 MRPS15 MRPL49 RPL36 MRPS35 MRPL46 MRPS6 RPL29 RPL13A HBA2 MRPL17 HBA1 RPS9 MRPL30 MRPL2 MRPL42 RPL6 MRPL23 MRPS36 EIF2D RPL5 RPL39P5 MRPS2 DAP3 MRPL36 MRPL1 MRPL28 RPLP0 RPL23A RPLP2 RPL8 MRPS5 MRPS26 C12orf65 RPS24 MRPS33 MRPL32 MPV17L2 MRPL33 RPL34 MRPS24 RPL10 RPL7A MTERFD2 RPL37 RPL21 RPL35 RPLP1 RPSA MRPS22 RPS17 RPS5 RPS15A MRPL15 RPL39L RPS27 RPS3A MRPL51 FXR2 MRPL19 RPL28 RPL10L RPL35A RPL17 RPL31 RPS14 RPS12 MRPS11 MRPS14 MRPL27 MRPL3 DDX3X RPS4Y2 RPS18 RPL26 RPL4 RPL22L1 RPS26 RPL38 NHP2 MCTS1 RPS25 MRPS21 MRPL16 RPS21 RPS27L RSL24D1 RPS23 MRPS17 RPS29 RPL12 MRPL12 RPL18 MRPS16 RPS20 MRPL22 MRTO4 RPL39 RPL37A RPL22 RPS7 RPL27A NHP2L1 RPS27A RPS19 RPL26L1 RPL23 RPS10 RPS10P5 ICT1 MRPS18A RPL27 RPL19 RPL7L1 RPS6 MRPL43 RPL7 RPL3L RSL1D1 RPS16 RPL30 MRPS28 RPL14 MRPS31 RPL11 MRPS7 RPS11 RPL10A RPL9 RPS3 MRPL47 RPL36AL MRPL10 RPS2 IMP3 RPL32 MRPL41 MRPS18C MRPL13 RPS8 MRPS9 RPS4Y1 RPL36A MRPL21 GNB2L1 RPLP0P6 MRPS12 RPS13 MRPS18B NSUN4 RPS4X ZNF622 RPS15 SURF6

GO_NUCLEAR_CHROMOSOME_TELOMERIC_REGION The terminal region of a linear nuclear chromosome that includes the telomeric DNA repeats and associated proteins. ATRX TOX4 ERCC4 H2AFY2 PARP1 THOC6 MCM3 TEN1 H3F3A POLD1 CTC1 XRCC6 NHP2 PURA SMCHD1 TERF2IP THOC3 LRIF1 PCNA RPA1 HIST1H4A ORC1 TNKS THOC7 SIRT6 XRCC3 MRE11A MCM7 PPP1R10 PPP1CC SLX4 DCLRE1B ACD H3F3B TERF1 DDB1 HIST1H3H PIF1 HIST2H4B ORC3 LIG4 SUN2 HAT1 MEN1 ERCC1 WDR82 CDK1 C11orf85 H2AFY ORC2 APEX1 DCLRE1A ZSCAN4 THOC5 HIST2H4A HIST1H4J RAD51 CBX3 HIST1H4L MSH2 ATR HIST1H4C POLR2B HIST1H2BA HIST1H2AA HMBOX1 MCM2 ORC5 HIST1H3G HIST1H3C PINX1 RAD50 GAR1 BRCA2 CBX5 PRKDC MCM4 THOC1 HIST1H3E NAT10 PPP1CA HIST4H4 HIST1H4D TNKS1BP1 TNKS2 UPF1 THOC2 TINF2 HIST1H3A HIST1H4I PML POT1 MCM6 HIST1H4H PPP1CB ORC4 KDM1A MCM5 DCLRE1C TFIP11 CBX1 HIST1H3B RAD51D FEN1 DNA2 CDC73 HIST1H3D HIST1H3F HIST1H4B THOC4 RPA2 HIST1H3I C15orf43 WRN HIST1H4K OBFC1 NBN XRCC5 WRNIP1 HIST1H2BB HIST1H4E TERF2 HIST1H3J SSB CCDC79 TERT NLRP2 SP100 OBFC2B ATM HIST1H4F OBFC2A

GO_EXCITATORY_SYNAPSE A synapse in which an action potential in the presynaptic cell increases the probability of an action potential occurring in the postsynaptic cell. SHANK2 GRIN2A DAB1 MAGI2 MIB1 SLC17A8 ADAM10 SIPA1L1 SYT11 PTK2B NLGN1 LRRTM2 DNM3 GRM3 MAPK8IP2 CRIPT LIN7C CACNG8 DGKI NLGN4Y GRIN2C NEURL SH2D5 PJA2 HOMER1 LRP8 STRN CDK5R1 RGS19 ARRB1 CHRM1 SYNDIG1 TANC1 CRYAB RUSC1 SHANK3 SEMA4C SYP NLGN3 ARC SRPX2 BCR HOMER3 GPER FYN PPP1R9A GOPC NRGN SYT1 RAB8A CHRNA3 SHANK1 P2RY1 ARRB2 GAP43 RGS20 KCNAB2 DBNL DNM2 CABP1 DRP2 ProSAPiP1 DLG4 SRGAP2 CPEB4 SLC17A7 ITPR1 FBXO45 MT3 GRM5 DLG1 DLGAP3 DLGAP1 DTNBP1 GRIK2 LIN7A BSN NETO2 DRD2 LRRTM1 LIN7B NLGN4X SYN1 GRIK5 SHARPIN CPEB1 PTCH1 LRFN1 ITGA8 EEF2K FGFR2 ALS2 MINK1 GRIN1 EPHA4 NCS1 ARHGAP32 DISC1 DLG2 ITGA3 PDE4B NPTN CNIH2 NSF GRM1 BAI1 IFNGR1 NELF DLG3 MAPT PICK1 SRC LRRC7 GRIA1 RGS14 ANKS1B ARF1 IGSF9B KLHL17 EPS8 SOS1 PPP1R9B ELFN1 LRRC4 LZTS1 SIGMAR1 MPDZ DLGAP2 LYN BAIAP2 PCLO MAP1B PDLIM5 SRCIN1 CAMK2N1 GRIN3A CACNG5 DVL1 CNN3 SYNPO CDK5 MTMR2 SPOCK1 AXIN2 FMR1 NETO1 ABI1 P2RX4 PSD3 LRP4 PLCB4 CACNA1C AXIN1 PKP4 HOMER2 ADORA1

GO_DNA_DIRECTED_RNA_POLYMERASE_II_CORE_COMPLEX RNA polymerase II, one of three nuclear DNA-directed RNA polymerases found in all eukaryotes, is a multisubunit complex; typically it produces mRNAs, snoRNAs, and some of the snRNAs. Two large subunits comprise the most conserved portion including the catalytic site and share similarity with other eukaryotic and bacterial multisubunit RNA polymerases. The largest subunit of RNA polymerase II contains an essential carboxyl-terminal domain (CTD) composed of a variable number of heptapeptide repeats (YSPTSPS). The remainder of the complex is composed of smaller subunits (generally ten or more), some of which are also found in RNA polymerases I and III. Although the core is competent to mediate ribonucleic acid synthesis, it requires additional factors to select the appropriate template. POLR2K POLR2F POLR2E POLR2D POLR2G POLR2J2 C19orf2 PPARGC1A POLR2B POLR2J CHD6 POLR2A POLR2L ZNF768 POLR2I POLR2C POLR2H

GO_CILIARY_PART Any constituent part of a cilium, a specialized eukaryotic organelle that consists of a filiform extrusion of the cell surface. Each cilium is bounded by an extrusion of the cytoplasmic (plasma) membrane, and contains a regular longitudinal array of microtubules, anchored basally in a centriole. EHD1 HSPB11 SNAP29 PTCHD3 GLI3 CABYR ARL6 NEDD1 ARL13B C6orf165 DNAH6 PCM1 PDE6G C10orf92 BBS9 KIF7 CCDC42B MAPT MKKS TAS2R43 DNAH3 BBS4 WRAP73 CENPJ TTC21B PROM1 TTC30B CCDC164 BBS7 DNAH1 DYNLRB2 SPA17 SLC26A6 TAS2R46 B9D2 UNC119B KIF17 ARFGEF2 PHF17 TCTN2 TSPEAR RSG1 GNAT1 KIAA1751 CCDC103 PROM2 SPEF2 TMEM107 TXNDC2 WDR19 TXNDC3 CATSPER4 KIF3C IFT88 C8orf37 IFT52 NPHP4 SPAG6 IFT74 TMEM216 C21orf2 CC2D2A MAP4 BBS5 BBIP1 IFT81 EZR ODF4 FBF1 WDR34 DNAI2 RSPH4A SEPT4 HK1 PRKAR1B ATG14 PIK3R4 CDHR1 ODF3 DZIP1 CEP41 GLI2 DYNLRB1 TMEM67 DNAAF1 PRKAR2B POC1B C12orf55 C2CD3 SPAG17 USH1G IFT140 KIF5C DHRS3 SHANK3 CEP164 KIAA0586 DNAH5 CYS1 DNALI1 DDX6 KIAA1984 CCDC39 SLC26A3 PDE6B RTTN PKD1 CETN2 ATP2B4 CNGA3 DZIP1L SCNN1A USH2A PTCH1 PHLPP2 EPS15 GNAT3 GLI1 DNAH2 SPTBN5 PIK3C3 EVC2 APP C1orf88 GPI CNGA1 TTC8 AZI1 NAPEPLD ATG7 GRK4 NPHP3 FOPNL KIFAP3 SSX2IP TUBG1 TTC26 CEP89 PGAM4 RAB28 RSPH9 INTU CASK B9D1 PDE6A GUCY2F TBC1D30 PFKM PRKAR1A TCTN1 DNAJB13 GUCA1C EFHC1 KIF5B AKAP3 CATSPERG RP1 C5orf30 AGBL2 ATG16L1 PKD2 KNCN OFD1 CCR6 IFT20 TMEM231 DNAH7 KIF19 IFT46 AK1 GSTM3 INPP5E WDPCP C1orf192 HSP90AA1 IFT122 RPGR MAP1LC3B IFT27 RPGRIP1L AKAP4 IFT172 TCHP FAM179B TTLL8 GRK1 IFT57 TAS2R4 DISC1 SEPT2 C14orf45 SUFU CCDC37 FAM161A AKT1 GAS8 TOPORS WDR35 TRAF3IP1 KIF5A CNGB1 DYNLL1 CCDC151 DEFB1 TMEM17 C1orf96 RP1L1 CNGA2 GRK7 IFT43 C5orf42 BBS2 PRKACB PSEN1 SLC9A3R1 KIF3B CCDC41 SSTR3 RABL5 DYNC2H1 RPGRIP1 DRD1 CNGA4 TAPT1 ENKUR TTLL3 PRKAR2A ABCA4 HHIP DYNLL2 GNB1 SEPT12 ICK DRD5 CEP290 TTBK2 PACRG AK2 CCDC63 WDR60 SMO TCTEX1D4 CCDC114 PKD2L1 GUCY2D KIAA1009 POC1A GUCA1A CCDC40 ULK3 RGS9 GNGT1 SEPT7 AMBRA1 PCDP1 RAB8A DNAH9 LRRC48 SPATA6 ALS2CR12 TBC1D7 TMEM146 FAM154A OPN1MW ODF2 AHI1 CYLD RHO SQSTM1 OPN1SW SPATA7 MKS1 RP2 EHD3 CROCC TTLL6 GPR161 OPN1MW2 SEPT9 DYNC2LI1 SSNA1 PKD1L1 RSPH1 KIAA1377 DNAI1 PRKACG SHANK2 MARK4 NIN EVC GNAT2 TMEM237 CLUAP1 NEK8 CEP19 ATG5 PKHD1 GUCA1B KLC3 ARMC4 SPAG16 MOK HYDIN SCLT1 TMEM141 C16orf80 ARHGAP35 DCDC2 MAK ROM1 MNS1 DRD2 AGBL4 OPN1LW PRKACA IFT80 PGK2 SPEF1 CENPF BBS1 DNAH8 KIF2A KIF3A DAAM1 PTPN23 DNAH17 AK8 UMOD TCTN3 CLTB TULP3

GO_AXONEMAL_DYNEIN_COMPLEX A dynein complex found in eukaryotic cilia and flagella; the motor domain heads interact with adjacent microtubules to generate a sliding force which is converted to a bending motion. DNAI1 DNAH17 DNAH1 DNAH2 TXNDC3 DNAH3 DNALI1 CCDC114 DNAH7 DYNC2LI1 DNAH5 DNAI2 DNAH8 DNAH6

GO_SODIUM_CHANNEL_COMPLEX An ion channel complex through which sodium ions pass. SCN3B SCN4A SCN11A SCNN1B SCN8A SCN9A SCN4B SCNN1G SCN1B SCN10A SCN5A SCN2A SCNN1A SCN1A SCN3A SCN7A SCN2B

GO_ORGANELLE_MEMBRANE_CONTACT_SITE A zone of apposition between the membranes of two organelles, structured by bridging complexes. Membrane contact sites (MCSs) are specialized for communication, including the efficient traffic of small molecules such as Ca2+ ions and lipids, as well as enzyme-substrate interactions. ESYT2 ESYT3 TOMM20 RAB38 ACSL4 STX17 CANX ZFYVE1 ATG14 RAB32 ESYT1 PIK3R4

GO_CELL_LEADING_EDGE The area of a motile cell closest to the direction of movement. PHACTR4 DPYSL3 PTK2B SSH1 MYH10 PAFAH1B1 FAP LAYN ITGA5 AKT2 CLRN1 CDH2 SPTBN1 ARF6 ARHGEF2 PALM MYO10 CTNNA3 ITGB3 PRKCZ SNX1 SPATA13 PACSIN2 CNTNAP2 SH3YL1 SHISA9 SLC39A6 ENAH DOCK8 ABI2 ABL1 CD2AP AMOTL1 CCDC88A RLTPR CHRNA7 PARD6A RAB34 APC RHOA PPP1R9A PLA2G4F EMR2 ANXA2 RAB3IP STX2 FRMD4B PTPN13 RAC2 RPS3 DLC1 TESC PDLIM7 ARHGAP31 PIP5K1C SPRY4 DAG1 SNX5 MYADM TIAM2 APBB1 WIPF1 VIM ACTG2 TSC1 ARHGAP44 TUBG1 ROCK1 PDE4A WASF2 PALLD CTNND1 SSX2IP PSTPIP1 FAT1 ACTC1 ROBO2 SGCE PAK1 ITGA8 FERMT1 KIAA0528 ILK APP NCKAP1 RAB13 SCYL3 NEDD9 BCAS3 CTNNA2 VAMP7 MTM1 TIAM1 MYH9 KANK1 KITLG NHS DGKZ IQGAP2 KCNA2 ARPC2 RDX TPM1 CDH1 C17orf87 BCAR1 PTPRJ KCNH1 PLEKHO1 EPS8L1 ARHGEF4 AIF1 SLK AIF1L EVL PKN2 RASGRP2 PLEK C15orf38 ACTA1 PARVB HDAC6 GRIA1 ANK1 PLEK2 CSPG4 AMOT TRPV4 S100A11 APBB2 SORBS2 UNC5A TACR3 FGD6 EPB41L5 ARHGEF6 CORO1C CXCR4 SAMSN1 PTPRM FGR EZR CTTN BAIAP2 ARHGEF26 COBL SH3RF1 EEF1A1 BRK1 ACTA2 MTSS1 CDK5 CDK6 STX4 TLN2 GABRG2 APC2 PDPN PIK3CA PLCG1 INPP5J ANTXR1 DDX58 PLEKHG5 PLEKHA1 ABLIM3 SNTG1 MYO5A EPS8L2 CLIP1 DST BMX RUFY3 MYO1C CORO1B IQGAP1 ATP2B1 DPP4 ARFIP2 CDC42BPG PTK6 ABI3 SPRY2 FSCN1 LRRC16A FER PIP5K1A STX3 ASAP3 RINL GDPD2 OPRM1 LCP1 KSR1 KLHL2 KCNJ11 JUB S100B RAC3 SLC39A14 VASP C20orf103 FERMT2 BSPRY CORO1A FLOT1 INPP5K ARAP3 GPER AMPH MTSS1L MACF1 WLS RAC1 DNM2 ACTR3 FGD1 WWC1 CFL1 RAB5A DBNL NDEL1 HAX1 GABARAPL1 IFIT5 ABLIM1 HPCA PLEKHH2 FGD3 SRGAP2 PXN FGD5 CLASP2 KBTBD10 ATP6V1B2 PIEZO1 PTK2 PACSIN1 SWAP70 NME2 CDC42BPA CYFIP1 CTNNB1 WASH3P JMY SCRIB ARF4 PLXND1 NRG1 MYO1G ITGB1 NME1 SLC9A1 KIAA1598 SH3BGRL3 LDB2 DYSF FGD4 PSD ARHGEF7 ALS2 NRBP1 VIL1 S100A6 CDKL5 RAPH1 TRPV1 NF2 MTMR14 EPHA2 PRKCI STMN2 C1orf172 PDE9A OPRD1 ADAM17 HSP90AA1 DCTN1 TWF2 DIAPH1 LMO4 KCNC1 PPP1R9B TRPM7 MKLN1 INPP5E EPS8 ARF1 ATF4 DDN SYNE2 PKD2 SRC APBB1IP ACTN1 CYTH3 PTPRK THEM4 KIF18A KCNC2 MYO6 MYO1D WASF3 RASA1 SNX2 RAPGEF3 ARPC3 INPPL1 PODXL TIRAP ITGAV ANGPTL3 PDXP AAK1 TNFRSF12A FLOT2 SLC1A2 SRCIN1 SNX9 ITGB1BP1 ITSN1 RAB22A TWF1 CDC42BPB CDC42 EPB41L3 MEFV TLN1 MYLK SLC9A3R1 PHLDB2 WASL CIB1 ADORA1 WASF1 ABCA7 IGF2BP1 LDB1 FGD2 GBF1 ABI1 ITGB4 MAPK8IP3 PTPRO MCC CTNNA1

GO_NMDA_SELECTIVE_GLUTAMATE_RECEPTOR_COMPLEX An assembly of four or five subunits which form a structure with an extracellular N-terminus and a large loop that together form the ligand binding domain. The C-terminus is intracellular. The ionotropic glutamate receptor complex itself acts as a ligand gated ion channel; on binding glutamate, charged ions pass through a channel in the center of the receptor complex. NMDA receptors are composed of assemblies of NR1 subunits (Figure 3) and NR2 subunits, which can be one of four separate gene products (NR2A-D). Expression of both subunits are required to form functional channels. The glutamate binding domain is formed at the junction of NR1 and NR2 subunits. NMDA receptors are permeable to calcium ions as well as being permeable to other ions. Thus NMDA receptor activation leads to a calcium influx into the post-synaptic cells, a signal thought to be crucial for the induction of NMDA-receptor dependent LTP and LTD. GRIN1 GRIN2D GRIN2A PTK2B NLGN1 SHANK1 GRIN3B GRIN3A GRIN2B EPS8 GRIN2C

GO_ACETYLCHOLINE_GATED_CHANNEL_COMPLEX A homo- or hetero-pentameric protein complex that forms a transmembrane channel through which ions may pass in response to acetylcholine binding. ZACN CHRNA4 HTR3E CHRNA9 CHRNB1 CHRNB2 CHRNA10 CHRNA6 CHRNE CHRNA5 CHRNA7 CHRNA3 CHRNA2 CHRND STXBP5 HTR3D CHRNB3 CHRNG CHRNB4 HTR3C CHRNA1 HTR3A HTR3B

GO_NEURON_PROJECTION_MEMBRANE The portion of the plasma membrane surrounding a neuron projection. NRG1 GRIA1 DDN ANK1 GPER KCNC2 MYO1D MAPK8IP3 ADORA1 KCNC1 CHRNA7 ITGA8 C20orf103 ATF4 OPRM1 GABRG2 ROBO2 KCNJ11 SGCE CNTNAP2 KCNH1 EPB41L3 OPRD1 SHISA9 HPCA GABARAPL1 TRPV1 PALM SLC1A2 TACR3 SPTBN1 ATP2B1 WLS UNC5A

GO_ENDOCYTIC_VESICLE_MEMBRANE The lipid bilayer surrounding an endocytic vesicle. LDLR VAMP7 ANXA3 CACNG4 WNT3 AP2M1 UBB SLC11A1 RAB43 UBC TBC1D5 CD36 WNT7A WNT3A AP2A2 TCIRG1 CAMK2D RAB32 HLA-DRB5 HLA-DQA1 EGFR STAB2 RAB11B RAB31 HLA-A GRIA1 OCRL WNT6 RAB11FIP1 HLA-H PICK1 SMO GRIA2 CACNG2 CYBA NOSTRIN HLA-DRA PIK3R4 WNT1 FCGR1B HLA-DRB4 CD207 CLVS2 AP1B1 HLA-DRB1 RAB7B HLA-DQB1 HLA-DPA1 RAB22A RILP HLA-DPB1 WNT5B MARCO HLA-G B2M CAV1 WNT7B FZD4 AP1M1 LAMP1 RAB38 WASL LRP1 STAB1 ATP6V0A2 ATP6V0A1 CAMK2A CLTC HLA-DQB2 ATG5 CAMK2B HLA-B SGIP1 SH3GL2 HLA-E HLA-C GRIA4 NOS3 WNT5A SYT7 RAB8B TLR2 PICALM SCARF1 CACNG8 CD74 RAB9A GPR161 AP1S2 FZD2 AP1S3 APOB COLEC12 AP2A1 LAMP2 CD9 ATP6V0D1 ATP6V0E1 RAB9B CAMK2G HLA-DQA2 UBA52 CYBB RAB8A RAB34 HLA-DRB3 SLC18A3 HLA-F RAB23 CORO1A RAC2 CLVS1 DLG4 WLS CD163 ATP6V0D2 MSR1 DNM2 INPP5B RPS27A TLR6 RAB5A IRGM LDLRAP1 ATP6V0C ATP6V0E2 MDM2 FCGR1A ATP6V0B GRIA3 CACNG3 DMBT1 AP1G1 RAB20 ATP6V0A4 AP1S1 HBEGF EPN2 ROR2 SCARB1 PIK3C3 TYRP1 AP2S1 RAB10 WNT4 ATG12 TLR1 RAB39 RAB7A FZD5 PTCH1 AP2B1 AP1M2 EPS15

GO_FIBRIL Extracellular matrix material consisting of polysaccharides and protein. ODAM LTBP1 SLC1A3 ADAMTS10 FBN1 SNCA FBN2 THSD4 MFAP5 MFAP1 MFAP4 MUC5AC MFAP2 ADAMTSL5

GO_SPERM_PART Any constituent part of a sperm, a mature male germ cell that develops from a spermatid. DNAH1 IZUMO1 C9orf9 MORN2 TSSK1B ADAM15 SPA17 DEFB1 SLC26A6 CATSPER3 CRCP TSKS PPFIA3 CD46 SPAG9 SPACA4 RPGR IFT27 TEKT5 FABP9 HSP90AA1 CYLC1 TSSK2 AKAP4 IFT172 CALR CCR6 PTCHD3 AK1 SV2B GSTM3 LOXL1 CABYR LRGUK NUDT1 PACRG PCSK4 TMEM225 AK2 SEPT4 HK1 NME5 TCTEX1D4 RACGAP1 ENKUR TXNDC8 ODF4 DNAI2 SEPT12 FLOT2 IFT81 TXNDC3 STK31 SLC9A3R1 KIT TXNDC2 ACRBP CAV1 SPINK13 IQUB ATP8B3 CATSPER4 CTNNA1 POMT1 RND2 CASC5 SPEF2 PATE4 ATP6V0A2 TBC1D21 CXADR ATP2B4 C9orf11 SLC26A3 HSP90AB1 SQSTM1 ACRV1 RSPH1 DDX6 SUN1 TMEM190 ALS2CR12 TMEM146 SPINK8 SPATA6 SPACA7 ODF2 CAV2 FAM170B TEX101 ARC TCP1 FAM154A SPAG8 ODF3 SKIL ZP3 NOTCH1 RAB3A SPESP1 CLK3 PLA1A CAPZA3 PFKM TEKT3 KIF2A CAPN11 ABHD2 AKAP3 SERPINA5 ZPBP2 SPACA3 CATSPERG AK8 DNAJB13 SPINK2 NCF2 PGK2 TRIP11 SPACA1 DLD SLIRP RNF38 PGAM4 FNDC3A BSG MNS1 DRD2 GPX5 TEX22 IQCF1 AZI1 TRIM36 IQCG HEXB ITGA1 GNAT3 ACR BMF SYT8 VEZT TBXA2R SPINK1 ATP6V1E2

GO_CYTOSOLIC_RIBOSOME A ribosome located in the cytosol. RPL22L1 PPARGC1A SURF6 ZNF622 RPL4 RPS4X RPS15 RPL38 NHP2 RPS26 RPS13 RPS18 APOD RPS4Y2 DDX3X RPL26 RPS14 RPS12 RPL10L RPS4Y1 RPL35A RPS8 RPL36A RPL17 RPL31 RPLP0P6 RPS27 RPS3A MRPS18C RPL28 FXR2 RPS2 NAA11 RPS15A RPS5 RPL32 RPL39L RPL21 RPL10A RPL35 RPL7A RPL10 MRPL4 RPS11 RPL37 RPS17 RPS3 RPSA RPLP1 RPL36AL RPL9 RPL14 RPL34 RPL11 MRPS5 RPS16 REPIN1 RPL30 NUFIP1 RPS24 RSL1D1 RPL7 RPLP0 RPL3L RPLP2 RPL8 RPL23A RPL5 RPL39P5 RPL27 RPS6 MRPL1 RPL19 RPL7L1 RPS10P5 EIF2D RPL6 MRPS18A RPS27A HBA2 RPS19 HBA1 RPL13A NHP2L1 RPL26L1 RPL23 RPS10 RPS9 RPL22 RPS7 RPL27A RPL37A RPL29 RPL36 RPS20 RPL3 MRTO4 RPS28 RPL39 METAP1 RPS23 RPL15 NAA10 RPL18 RPS29 RPL12 C7orf44 EIF2AK4 RPS25 MCTS1 RPL13 RPL41 RPS21 RSL24D1 RPS27L RPL24 RPL18A

GO_INFLAMMASOME_COMPLEX A cytosolic protein complex that is capable of activating caspase-1. CARD8 NLRP3 NLRP6 PYCARD CASP1 NLRC4 CASP12 CASP5 NLRP1 AIM2 GSDMD CASP4

GO_PERINUCLEAR_REGION_OF_CYTOPLASM Cytoplasm situated near, or occurring around, the nucleus. CA4 CYLC2 MOB4 SERINC3 FZD9 VPS33A EHD2 TPD52L1 GBP4 SELM ITM2C MOBP SLC39A12 WWC1 HCRT MAP1S PLA2G2A CCL2 SYT6 CABP1 TFRC STX16 SEC24D RAP1GAP2 CMYA5 ITGB1 C14orf166 ODZ1 KCNS1 ATXN2 PER2 NME2 HEPH GNB2 ZFYVE1 CYFIP1 CTNNB1 MLC1 SERBP1 RARA HIP1R DGKI CYLD PICALM EHD3 TNFSF13B HSP90B1 MT1A ATP7A CDK2AP1 NFKBIE ADAM10 LAMC2 SLC30A7 MAGI2 LMTK2 DNM3 TNFSF12 SEC23A AGTR2 USO1 GPER SNF8 BCAP31 NDRG2 RAC3 S100B NDFIP2 ACTN4 CHERP HMGCLL1 TMEM192 GAK RNF207 STK33 LNPEP SRD5A1 ITGB1BP1 KAT5 NHLRC1 SRFBP1 LRAT LYN PLA2G5 FLOT2 CDC20 TRAF6 ARF5 RAPGEF2 ANXA4 CCDC78 HDAC1 CDC5L RAP1A BUB1B UPF2 ZNF35 PLD1 ARFGEF1 GDPD5 PTGDS HIF1AN CAV1 ACHE PRKACB OLFM4 DCUN1D3 ATXN10 SLC34A1 CDK4 HMGB2 MOSPD1 OAS2 TPD52 TSTD1 VPS4A CDH13 MTMR14 S100A6 MSN ITGA3 ALDH1A2 LDB3 CST3 M6PR DEF6 TMEM100 MLPH SLC9A1 KIAA1598 CYB5R4 MYO16 ADCY10 TMEM173 SRC SET EHD4 GBP3 BCL10 CXCR7 SLC2A12 VTI1A GOLGA1 HSP90AA1 PPP1R16B C17orf46 CASC3 RAB40C PAM MCM3 FAF1 AKAP4 IGF2R PDE9A ADC ANXA6 SNAP47 OSBPL6 TSC1 PDE4A RPS6 MAF1 SEC23B DAOA RYR3 APBA1 UNC45A APBB1 TPPP UBQLN4 DLG1 MT1E INHBA FBXW8 BTK ANKRD13C CHGA TNFRSF1B SEZ6 HSPA1B PRKRA RNF128 PTCH1 UBQLN1 BICD1 AATK SEC31A STBD1 FAT1 KLHL20 AIFM1 NOS1AP TAF8 CCAR1 HECTD3 PLVAP COL4A3BP PRKCZ APOD NDRG1 NEURL SPP1 PTN ANP32A CHODL CHI3L1 PRKCE ROS1 TRIM37 PAFAH1B1 TRAPPC2P1 PTK2B CTLA4 TSPAN1 DOCK6 CCR2 CTIF INADL PRKCG FMN2 COPS8 CYBB SLC2A4 NANOS1 VAMP8 TRIM68 STX10 RAB34 CD2AP RAB15 HYAL2 ABL1 CDK5R1 LAMP3 CCRN4L PIKFYVE SLC8A3 SEC24A DYNC1I1 VAMP2 CAPN2 PTGES2 COBL EPHA5 PPARG APBA3 DDX4 MT1B PTPRM VAPA CYBA PTPN22 GAD2 CDK5RAP2 ARHGAP1 APEX1 ATN1 SORBS2 ARFGEF2 RHBDD2 CCIN PRKCD GALNT1 CLN5 THBS3 TRAF4 PDE2A PKP4 MX1 TSNAXIP1 MAEL STX4 STC2 LAMP1 STX6 VAMP5 TARBP2 SCYL2 HCN4 SPINK5 MVP STOM MEX3D APLN SYT4 BRSK2 GSDMA HHATL DNAJB6 TF CLINT1 MT1X ABCA1 BDNF CYHR1 SELE NOS1 C2orf28 VAMP7 TOLLIP GGT2 VPS53 PSEN2 RASGRP3 SLC2A10 RAB3C PLEKHF1 GRASP EPN3 ATP2A2 AGGF1 OSBP DGAT2 SLK HRNR TRIP10 RAB4A SORT1 CAMK2D KCNH1 RASA2 KIAA0101 PLA2G16 FXR1 OSBPL7 SRCAP PTGES CDKN1A UCMA CENPF EIF2AK2 MT1F DNAJA1 TMEM184A RANGAP1 DCTN3 CDKN3 USP2 DNM2 SERPINF1 FGFR1OP NME1 LAMB1 EIF2AK3 CAPN6 HSPA1A MST4 USP20 STX7 MAPK8IP1 RAB11FIP4 PACSIN1 BPTF KRT18 RHPN2 HMOX1 KPTN GLB1 MS4A3 PARK2 PLCZ1 SEPT9 ATP9A VPS33B PNPLA8 STK16 BCL3 PIK3R1 AMFR MALT1 NEDD4 CORO1B MT1M SERHL2 MAP2K2 GNB2L1 TLR4 SPIRE1 PKHD1 SERINC5 OSBPL3 ENPP3 INPP5K BSPRY AMBRA1 EML1 MRFAP1 NOS2 ECE1 TRPC7 MAP3K4 CSF1 TRIAP1 MYO9B NPPA VPS52 CX3CR1 ABCD1 USP33 CAV2 NXT2 GALNT2 HERC5 PRDX5 SLC39A13 RAD51C KCNH2 LCE1D CNP TRAIP SEPT12 TAF10 RASD1 CTGF MT2A YWHAB PPM1F PUM2 RAB4B IGF2BP1 P2RX4 CLIC1 SYNC CEP250 PI4KB SNCA CIB1 AKAP6 EIF4H ASPSCR1 CCNT2 SLC17A3 RPS6KB1 TLK2 SLC9A3R1 RAB14 TWF1 MEIS2 AKR1B1 MAD2L1 SEPT2 EPB49 DISC1 TP53BP2 NF2 SPAG9 STAT1 NRBP1 TRIM13 KCNS2 SYT5 KIF5A CUL7 PPIB ARF3 KIF20B MMD2 MYO6 ANGEL1 PICK1 ITGA2 CYFIP2 ATP7B KCNA5 ACSL3 CD34 TGFA ARF1 TWF2 CYLC1 S100A13 CABP2 EGFR PRKCA MYO1B MAL2 STMN2 VCP CSNK1D MT3 S100A4 EIF4A2 PSMF1 ARHGAP10 WDR44 LIMS1 SLIRP SPIRE2 FLRT1 RASEF CALCOCO2 SIPA1 MRVI1 LRPPRC ZNF675 SLC11A2 GTPBP4 PKN3 KIF5B FZD5 SNAPIN FLNA MT1H COPS5 VPS54 OPTN APP NELL1 ALG2 WRNIP1 DEPDC5 S100A14 KIRREL HFE PSTPIP1 SNCG GALNT4 OBSL1 GALNT3 C9orf24 FKBP4 TNKS2 TSNAX AKAP13 RAB8B NANOS3 MT1G CIDEB DAB1 SPAST MAP6 RAB40B MAP7 WBP2NL RASIP1 MTDH SIRT2 SEC24B CDC25C ZP3 ANXA2 CDK7 PCSK9 AURKA PRKAR2B CLIC4 DNM1L BAG5 RANBP2 INHBB PTPRR FUS CBL ZNF259 RAB7L1 SH3RF1 MOB2 FAM168B EZR ERBB2 REP15 PTOV1 RAD51 NMRAL1 NDOR1 DOK1 PREX1 PLEKHG5 FMR1 CALN1 APC2 AXIN1 XRCC3 EIF3G EIF4E PINK1 ATP9B GBP2 NOX4 MAGEE1 GALNT6 FASLG PARK7 BNIP2 NCS1 GSK3B CDH1 APLP1 STX8 GAPDH CSDA CTSB TYR CLU NDFIP1 MYRIP RAB3B MTPN FBXL5 VTI1B NANOS2 ODC1 SNAP25 HRAS INF2 CKAP4 TRAPPC2 SEC24C CALR RAB11A CABP7 PPP1R13B AANAT HDAC6 PHEX PAK2 PKN2 NPC1 LPXN TSC2 AIF1 PLN

GO_CENTRIOLAR_SATELLITE A small (70-100 nm) cytoplasmic granule that contains a number of centrosomal proteins; centriolar satellites traffic toward microtubule minus ends and are enriched near the centrosome. ALDOB PIBF1 SDCCAG8 CCDC14 PAX2 BBS4 ZMYND10 CCDC113 FLOT1 OFD1 CCDC13 SPAG5 PCNT HOOK3 FOPNL SSX2IP KIAA0753 AZI1 C2CD3 KRT18 BBS9 PCM1 CEP72 EXOC7 CEP290

GO_SET1C_COMPASS_COMPLEX A conserved protein complex that catalyzes methylation of histone H3. In Saccharomyces the complex contains Shg1p, Sdc1p, Swd1p, Swd2p, Swd3p, Spp1p, Bre2p, and the trithorax-related Set1p; in mammals it contains the catalytic subunit (SETD1A or SETD1B), WDR5, WDR82, RBBP5, ASH2L/ASH2, CXXC1/CFP1, HCFC1 and DPY30. WDR5 CXXC1 DPY30 HCFC1 DYDC2 ASH2L RBBP5 SETD1B DYDC1 SETD1A WDR82

GO_LAMELLIPODIUM A thin sheetlike process extended by the leading edge of a migrating cell or extending cell process; contains a dense meshwork of actin filaments. PTK2 PSTPIP1 WASF2 CTNND1 PALLD PIEZO1 WASH3P CTNNB1 CYFIP1 ACTC1 FAT1 NME2 SWAP70 ILK APP PLXND1 SCRIB SCYL3 ITGB1 NEDD9 NCKAP1 RAB13 MYO1G FGD1 RAC1 DAG1 DNM2 ACTR3 TESC ARHGAP31 DBNL TIAM2 HAX1 CFL1 ABLIM1 APBB1 FGD5 PXN SRGAP2 ROCK1 TSC1 ACTG2 FGD3 PLEKHH2 STX3 SLC39A6 GDPD2 FER PIP5K1A KLHL2 CCDC88A RLTPR AMOTL1 ENAH ABI2 VASP APC FERMT2 RHOA PPP1R9A JUB SLC39A14 RAC3 MTSS1L RAC2 ARAP3 FLOT1 STX2 RAB3IP PTPN13 CORO1A MYH10 PHACTR4 SSH1 DPYSL3 PTK2B FAP CORO1B CLRN1 CDH2 RUFY3 ITGB3 CTNNA3 ABI3 DPP4 MYO10 SPATA13 SNX1 LRRC16A FSCN1 CDK5 MEFV ITSN1 ACTA2 WASL MYLK STX4 WASF1 PLCG1 PDPN PIK3CA APC2 CIB1 ABLIM3 CTNNA1 MCC PTPRO ANTXR1 FGD2 ABI1 IGF2BP1 PLEKHG5 APBB2 SORBS2 ARPC3 INPPL1 RAPGEF3 SNX2 TRPV4 CORO1C ARHGEF6 PDXP PTPRM ITGAV PODXL FGD6 ANGPTL3 CTTN FLOT2 SH3RF1 BRK1 SRCIN1 ITGB1BP1 AIF1 STMN2 PKN2 EVL TWF2 PARVB ACTA1 PPP1R9B C15orf38 WASF3 PLEK2 SYNE2 PKD2 APBB1IP AMOT CSPG4 VAMP7 DYSF FGD4 SLC9A1 KIAA1598 CTNNA2 SH3BGRL3 ARHGEF7 ALS2 EPHA2 VIL1 NHS KITLG NRBP1 KCNA2 RAPH1 NF2 IQGAP2 DGKZ CDH1 BCAR1 RDX

GO_LATE_ENDOSOME_MEMBRANE The lipid bilayer surrounding a late endosome. CHMP6 RHOB SPPL2A CHMP4C ANXA8 CHMP4B ATP13A2 RILP ABCA5 OSBPL9 RAB27B TMEM9 RAB7A VPS37C SNX14 PLD1 RNF13 MMD SLC29A3 FAM125B VPS37D VPS37A ARL8B MARCH1 FAM125A SLC9A9 ATG9A CLCN3 TMEM55B HLA-DRB4 C20orf29 LAMTOR1 MCOLN1 HMP19 LAMTOR2 MITD1 HLA-DRA SLC11A2 LTV1 TMEM55A VPS39 CHMP3 CLCN4 PMEL SLC38A9 CHMP2B SLC30A3 KIAA1324 VPS33A MUDENG HLA-DRB1 HLA-DRB5 NTRK1 VTI1A NPC1 LAMP2 LAMTOR3 RAB27A PIKFYVE CD300LG HLA-DMA VPS36 VAC14 ANXA6 HGS VPS37B SNX16 FIG4 OSBPL11 TMEM59 SNF8 ARL8A CHMP1B GOSR2 VAMP8 HLA-DRB3 ANXA2 TMEM165 NDFIP2 CD63 MICALL1 VPS4B HLA-DMB TSG101 TMEM106B VPS18 VTI1B VPS16 CYBASC3 VPS41 VAMP7 IFITM3 STARD3 CHMP4A VPS28 CHMP2A C7orf59 SLC30A4 STARD3NL VPS4A SLC11A1 STX8 VPS11 VPS33B GALNTL5 TICAM2 HBXIP

GO_INTRINSIC_COMPONENT_OF_PLASMA_MEMBRANE The component of the plasma membrane consisting of the gene products and protein complexes having either part of their peptide sequence embedded in the hydrophobic region of the membrane or some other covalently attached group such as a GPI anchor that is similarly embedded in the membrane. TSHR ATP1B4 ADCY3 C6orf192 MC1R EVI2B BSG SLC5A4 GPR32 BEST4 OXTR TMEM11 GRM4 GPR83 SLC6A6 ABHD12 PTGER1 MFSD3 CD160 SLC6A4 SCN8A TBXA2R PAG1 CHRNB3 APP CPO FZD10 HTR4 GUCY2F JAG2 AQP2 SLC11A2 GNRHR2 ZYX HVCN1 AQP1 RAMP1 CLEC4A BFAR FSHR PLXNA4 TMEM150C CLDN3 MS4A2 SLC10A5 NPHS2 SCN4A IL17RD HCN2 NCR1 DDR1 HTR1B SYNDIG1 NKG7 KCNQ1 SLC38A6 TNFSF10 PTK7 BIRC2 FLT4 CNTNAP2 FRS2 FCER1A SHISA9 TRPV2 GABBR1 KCNK9 ADCY6 GPC1 TSPAN16 HTR3A CHRNA7 SCNN1B PILRA NTNG2 FCER1G SLC18A3 ADCY2 PKD1 NCKAP1L ACVR1 GP5 P2RX7 GAL3ST1 OR11H6 PODXL2 SYT11 TM7SF4 GLRA3 KCNG4 LTBR TRAF5 OPN3 TLR2 OR5T1 NPY6R PCDHA8 DAB2 NCR2 PLXNB2 EPHA8 KCNA3 SLC5A10 LILRB3 TRHR TRGC2 KCNE3 RNF43 CD48 CSF1R CHRNE KIR2DS5 GGTLC2 NTSR2 CLCNKB IL1RL2 MC5R GABRA1 SLC22A13 GABRD SLC38A4 SCN3A CD8B NOX4 LTK GABRB2 TMPRSS9 ITGB6 UPK1B ADIPOR2 KCNQ2 PROM2 SLC39A10 KCNJ18 OLR1 PCDHAC2 SLC39A8 MC3R SIGLEC7 PDPN SLC38A5 C9orf46 TACR3 TNFRSF1A DIABLO INSR MTNR1A GRIA2 CEACAM6 CCR4 SAMD8 CACNG2 UPK2 ITGB8 RELT KCNJ15 FUT1 C3AR1 SCNN1G SLC35A1 GALR3 KCNN1 RRH CD14 EPCAM DRD3 MRGPRF SLC22A11 DLG3 CD2 KLRC2 HTR6 KLRK1 RYR1 NOX3 SELPLG OR9G1 ITGA4 LY96 CHRM3 KCNJ6 CCRL1 F3 F2R RASA4 FLRT2 TMEM150A CCBP2 SCTR OR10J1 CEACAM4 IL28RA SLC43A2 S1PR3 KCNT2 PIGR SLC26A6 SLC22A4 FFAR1 FASLG DLG2 SLC2A3 CD79A OR1E2 FPR1 ATP4A SLC10A3 SLC15A2 IRS1 CD180 STX8 LAPTM5 FAM26E ENPEP IRAK1 APCDD1 HTR2B ADAM23 CALM3 ABHD6 PHB TMPRSS11F PVRL1 TSPAN31 CD72 GPR65 SLC16A4 OPN1LW NEU3 NCF1B EMB KCNJ14 GPR152 LPPR1 CD70 P2RX1 KCNMB3 ODZ4 KCNK2 EPOR KCNIP3 MRGPRX1 KCNA6 SLC29A2 SLC17A5 OR9A1P OR9G9 SLC26A5 NCR3 SLC28A2 ATP12A SHISA6 SLC4A3 BOC KCNA7 SYT13 EPHB3 GBAS CLCN1 SLC7A10 PCDHB12 SDC1 ITGA6 GRIK2 FLT3 SLC2A13 TSPAN32 PCDHB11 CNPY2 GPR171 ATP1B3 IL6R HCRTR1 IL17RC CALCRL CADM1 RNF31 CAV2 ITGB2 PVR TMPRSS11D CX3CR1 KCNG1 CNGB3 ITGA7 HLA-DQA2 OPN5 S1PR1 ZDHHC2 CXADR SLC22A8 HTR7 DRD4 ATP2B1 C5 SHANK2 KCNQ5 RXFP3 RASAL3 KLRC4-KLRK1 PKHD1 KCNK5 SLC2A8 TNFRSF14 SLC39A2 CACNG8 SLC2A5 TNFSF15 NLGN4Y HCAR3 IL27RA CLCN5 KCNIP2 OPN1MW2 CEL KCNH4 KCNK1 P2RY11 ENTPD1 LCT ADAM29 ADRA1D CD58 PTGER4 OLFM3 SSTR3 DPP6 ADRA2C F10 CNGA4 P2RX4 GALR1 SLC6A16 PCDH12 STAB1 ADORA1 TNFRSF10A CHRND DLL1 OR10H4 EPHA3 TPSG1 NCF4 PODXL PERP IFNGR2 IL15 PPYR1 AMIGO1 KLB C8G SLC16A3 GPR88 BST2 RGMB HTR5A BSND SLC12A2 TSPAN4 KCNH2 PROKR1 NPBWR1 SLCO4A1 CACNG5 TGFBR1 PPAPDC1A KIR3DL1 TRAF2 PRRG1 P2RX6 HCN1 AVPR1A OR10J6P ABCG1 ATP1B1 TRPA1 FAS SLC25A5 NF1 KCNC2 LYVE1 TSPAN14 KCNH3 CLPTM1 SLC1A4 SLC19A2 CYSLTR2 GPR42 RHBG SLC12A6 GPR68 LY75 PTGER2 ATP13A3 CCR8 TRPC4 CD1E RCE1 TPBG CNTNAP1 KCNMA1 SCN5A KCNG2 CCR10 HRH2 GPR75 GM2A TACSTD2 CYSLTR1 CD47 KCNH8 KCNS2 SLC24A1 ABCC8 FAT1 GPR77 LANCL1 MRGPRD ROR2 CLDN1 NLGN4X MPP2 SLC17A4 TLR5 PLAUR PRSS8 SLC30A5 PLSCR1 GPRC5C LHCGR CADM3 CCR1 KCNK12 AQP6 TGFBR2 MMD STBD1 NRG3 HTR1F GABRA3 ADIPOR1 ENG APH1A RAMP2 FOLH1 DLG4 TNFRSF1B APH1B PCDH11X STX1A MMP14 SLC26A7 MPL GPR35 SLC14A1 CD53 NCF2 SLC13A2 ADCY9 ICAM2 ADORA2A LPAR3 DLG1 CD69 PCDHA3 SLC12A5 GGTLC3 LGR5 F2RL1 HCN3 SLC8A3 GPA33 ICAM5 CD151 CD37 PVRL4 HAS3 KDR CDHR1 OR10J5 TYROBP NOXA1 TIGIT LPPR4 CYBB LRRC8E OR52A1 PCDHB3 RASAL2 ITGA5 TSPAN13 GRIA4 ATP2B4 GP9 NOTCH2 SLC5A2 C7 REEP2 ICOS PTK2B PCDHA6 KCNG3 CNGA3 RXFP4 C1orf130 CD1D XCR1 GCGR CD5 NCF1C SLC23A2 TRPV6 FLT1 GYPB KCNB1 SLC7A9 FZD4 VAMP5 HCN4 SLC7A13 FLT3LG SLC6A19 PTPRB ESYT3 MERTK TNFRSF8 KCNH5 LTB4R2 GABRA5 OR11A1 IL12RB2 EFNA5 CALHM2 RHCG NPFFR1 TMEM22 CR1 OR6T1 JTB PTPRM FAM26D SLC6A3 NPY2R ABCC2 NAGPA VIPR1 PTPRF ABCB5 NMBR GABRR1 UNC5A FAM26F KCNH7 PRSS22 ST14 PCDHB15 GPC6 GRIK1 GGT5 VAMP2 SLC31A1 GPR25 SLC4A8 ADCY1 GPR33 COL25A1 EPHA5 GRIN3A GRIN2B BCAM TSPAN5 SCN11A PCDHA1 GRM1 CELSR1 SLC38A3 GPR31 CD40 GPR84 KCNH1 HTR1A SELP AVPR2 CXCR2 COL13A1 GPR52 RASA2 GPR101 TRIM27 IFNGR1 MET OR10X1 TOLLIP CSPG4 CLEC1B PSEN2 SLCO5A1 SGCB EPHA6 SLCO1B3 ADRA2A HTR3D ATP2A2 PCDHB6 PTGER3 MYH9 ABCA1 ANPEP SSTR5 SLC1A6 CD1C SLC16A8 PPAPDC1B KCNAB1 MEP1A CRHR2 ITGAM HS6ST1 STOM ATP1A4 PCDH1 HNRNPM STIM1 KCNA2 EFNB2 C9orf5 GPR44 BMPR2 PROCR KCNJ1 SLC7A5 LYPD3 UTS2R OR11H12 SLC23A1 GPR50 PTH1R CNIH3 LOC388630 SLC6A17 SLC38A2 XPR1 MEP1B SLC24A3 SLC16A11 KCNV1 OR10H3 ABCC11 MAEA HCRTR2 TSPAN2 ITGB1 LPAR2 KCNS1 ODZ1 TRO CACNG7 EFNA3 KLRF2 TLR6 MIP CD200 SLC5A12 KCNAB2 YKT6 SLC24A4 TRAF3 KCNA4 KCNK18 CHRNB2 TMBIM6 SLC6A20 GRIA3 PMEL CA4 HAS1 PORCN ATP13A4 KCNV2 PTPRG EDA2R SLC30A3 GPRC5A TEK SELL CALHM1 ENPP2 CCKBR NTNG1 NTRK1 CHRM1 SLC26A4 ICAM4 MFI2 XG OPN1MW ABCB4 PTPRN2 SLC17A2 SLC4A2 ATP1A1 SLC3A1 TRPC5 GPER CPT1C EPGN HTR2C SYNGAP1 CLDN4 ITGB5 CHRNA2 BCAP31 SLC39A14 CD63 ATP2B3 SLC6A9 RTN4RL1 KCNJ9 GRIN2A TNFSF12 TMPRSS11E STEAP1B C2orf83 KIR2DS4 FGFR1 PRSS42 TSPAN3 CD83 RHO MRGPRX2 ORAI1 TNFSF11 CCRL2 TGM2 CD226 CD1A GPR37 SLC6A13 IGSF6 IFITM5 CALHM3 SLC15A1 TACR2 UPK1A LY6E KCNQ3 SLC34A1 SLC22A24 SLC33A1 DRD1 KCNJ13 OR11H4 LRRC8C GLRA1 SCN10A SLC25A11 ITGB4 PTPRZ1 SMAGP SLC22A2 CXCR3 IGDCC3 CD81 GAS1 TRAF6 SLC4A4 P2RX3 IL1RAP MPP1 PVRL2 KCNE1L SUMO1 KCNMB4 TM9SF2 CHRNB4 CLCN4 OPRL1 HLA-DPA1 KCNE2 MRGPRG ABCA4 GPR1 NLGN2 TUSC3 SHISA7 TSPAN11 SLC22A10 SLC7A8 TCIRG1 SLC5A3 KCNIP1 MRGPRE CALM1 ITGAD RAB26 GRID2 SLC6A2 ZNRF3 BMP2 SLC16A7 PLXNB3 SLC2A12 ITGA10 ATP1A2 ADRA1A CAPRIN1 CACNG4 MUC4 MC2R GPR176 PPAP2B SLC25A14 LGR6 EPHA1 GGT3P C5AR1 ART1 EDA TSPAN19 KCNK6 SIGLEC6 CD36 EDNRA RDH8 CDHR2 SDC4 SLC24A2 SLC1A5 CD82 FGFR3 TRPV1 MRGPRX3 BCAP29 SSTR2 SLC14A2 TM2D1 SLC2A1 CNGA1 IL13RA1 PCDHB2 GABRB3 ITGAL HFE SLC16A12 KL MLNR C1QTNF1 FOLR2 CSF2RB HTRA2 TMEM8A OR56A1 GABRA4 SLC10A4 SCARA5 ITGA8 ECEL1 SLCO1B1 SLC20A1 TNFRSF21 KCNK15 EPHB4 PCDHA2 CLCA2 SLC10A1 SLC16A10 VWC2 MCOLN1 ABCC5 PSENEN SPN C6 KCND2 ADRB1 CD163 BTN1A1 DCLK1 SLC7A2 TNFRSF10B KIAA1324 FLRT1 ANKH SCN3B ALCAM PCDHA11 GPR149 SCN2B SLCO1A2 SLC6A5 SLC39A5 IL6ST ATP4B CCKAR SLC39A6 TNF SLC7A1 IFNAR1 GPR55 SLC43A1 KCNJ3 BEST3 MDGA1 PRSS41 RET MLANA FXYD4 PLA2R1 KCNMB1 CD59 HLA-DRB3 PRRG2 BEST2 P2RY14 MPP3 SCNN1A MAGT1 JAG1 SLC39A4 PPAP2A MS4A1 HRH1 KCNK16 PLXNA1 ITGA9 TLR7 OR3A2 CRHR1 PALM ITGB3 ODZ3 PCDHAC1 ADCYAP1R1 SLC5A5 KCNJ2 MMP17 F2RL2 NPR3 PI4K2A LPHN3 SLITRK6 SLC20A2 KIR3DS1 GYPC IL17RE VWC2L TRPV5 FGFR4 C9 PTPRA SEMA4D HPN CASR SLC26A10 SLC18A1 ATP2B2 RAMP3 SEMA4F ACVR1C GJA8 SLC6A18 NPY5R BEST1 ATP2C1 ESYT2 FPR2 GPR137B EPHA10 ICAM3 APLNR MILR1 OR5T2 NPC1 DDR2 PRLHR ADRA1B PTPRJ NPSR1 GPR20 EMR1 SLC22A31 FOLR1 SCN1A GRIA1 CHRM4 SCN4B HLA-H SLC16A1 ACCN4 CCR3 PHEX GJD3 NPBWR2 KCNK7 GRIN1 SGMS1 ATRN KCNH6 KCNN4 SNAP25 LDLR AXL ULBP3 TNFRSF13B CALY COL17A1 CLEC4M GGT7 SLC25A3 NPNT SLC22A6 NTSR1 PROM1 GPR172A PTPRS MIEN1 CSF2RA FXYD3 CD46 SLC7A3 GRM2 GGT6 PTGIR KCNU1 SLC16A13 SLC17A1 SLC22A20 SLC5A8 KCNA1 CHRM2 ROM1 NINJ2 C8B VIPR2 ZACN TLR3 CD27 AGTR1 RASA4B CNTN2 RYK NRG1 GJA1 EDNRB CD300C APOM OLFM2 C20orf54 GABRB1 ABCC9 GALR2 CEACAM1 STEAP2 IL6 PLXND1 AKAP9 TRHDE SLC6A1 NMUR1 RASA3 MSR1 CHRNA9 ADAM8 NPHS1 CACNG3 CD99 SLC7A7 SLC26A11 CSF3R SLC25A4 ODZ2 OR5T3 TRPC7 TNFSF4 ATP13A5 CLCN2 SLC22A12 P2RY4 EPHB1 CLEC1A GRM6 HAS2 OR56A5 CNTFR TRPV3 ENPP3 KCNS3 PLXNA3 ABCB11 BACE1 PCDHA7 TMEM5 IL9R DEGS1 MAS1L GHR NRXN3 MMP16 KTN1 GRM3 KLRF1 ATP2C2 TMEM150B SLC38A1 NRN1L TLR4 NRCAM SLC1A1 TMEM130 ATP13A1 P2RY6 SLC17A3 PSEN1 KCNK4 PCDH8 DAB2IP ATP13A2 SLC4A10 TNFRSF11B TRPC3 SLC12A7 TRPM1 EPHA7 NOXO1 GPR39 ATP1A3 STEAP4 BMPR1B GPC4 CACNB2 PTPRO ACVRL1 PCDHB4 TRPM2 MRC1 HPS1 KISS1R KCNK3 SLC12A3 KIR2DS3 GPC5 SIGMAR1 AQP9 IGF1R OPN4 GUCY2D AQP7P3 KCNMB2 HLA-DRA FXYD5 MFSD2A P2RY13 SCN2A KCNJ5 TNFSF8 TMPRSS11A ICAM1 ABCC1 ART3 IFNAR2 SLC4A9 TM4SF1 TMPRSS2 LRRC26 SHISA8 CD3E ADORA2B SLC31A2 ENPP1 ACCN3 ADAM17 BAI1 SEMA6C ITGAX ATP7B ITGA2 PTPRK EBP THBD MMP15 CEACAM5 ATP1B2 TRPC6 KCNC1 CCR5 KCNA5 MME EPS8 KCNK13 TGFA CD34 NPR1 TRPC1 KCNT1 SLC19A1 ADRA2B LMAN2 IL23R LRRN4 SCN7A ACVR2A LPPR3 TACR1 ITGA2B GPR6 CD164 AGER SLC8A1 PTGFR IL17REL SLC7A11 SLC11A1 AQP3 EPHA2 GPIHBP1 IKBKB HRH3 PCDHA10 GGTA1P NOS1AP TSPAN10 DCBLD2 SGCE SLC6A11 IL12RB1 SCARB1 OR10H2 BTN2A1 GABRQ HFE2 SLC22A16 ABCC3 PTAFR HBEGF RHCE ADRB3 SLC22A25 SLC5A1 TLR1 STXBP5 SLC7A14 MTNR1B SEMA6B PRKD1 STEAP1 CD40LG GRM7 SLC26A8 TNFRSF11A CEACAM8 CXCR6 MAL KCNC4 MICA OSMR HTR3B SCN1B QRFPR ASGR1 ADRB2 C2orf89 CLEC2D TMPRSS11B KCND3 SLC12A4 SLC18A2 SEMA6A GRM5 PVRL3 SLC13A1 MCHR2 ROR1 HYAL2 OR11G2 GPR12 TGFBR3 GPC3 CD79B GPR56 TNFRSF10D PLXNA2 TSPAN8 OR13F1 OR9A4 TNFRSF10C P2RX2 MUSK CCR2 OR1D4 P2RY2 PTPRC SIRPB1 SLC4A7 CMKLR1 THY1 KCNK17 FXYD1 SHANK1 NPFFR2 P2RY1 CSPG5 SLC2A4 IL10RB FCER2 SLC26A3 MPZL1 IL29 SPTB CHRNA10 IL11RA JAM2 GLRB PTPRD ATP8B1 CD55 CD19 MUC12 SLC4A5 GPR19 TSPAN1 CHRNB1 SPTA1 CTLA4 LPAR1 AQP8 CHRNG SMPD2 ITPR3 TPO SLC6A7 GABRA6 GRIK4 GP1BB SDCBP SLC19A3 KCNQ4 GPR34 SLCO6A1 ITGAE RASAL1 PDGFRB ACCN1 GRIN2C LPPR2 CLCA4 GABRG2 KCND1 SLC24A5 LRRC32 LAMP1 OR11H7 CNR2 UGT1A1 SLC5A11 CD44 F2RL3 FZD6 HTR3E KCNA10 CLEC5A DSCAM LILRB2 LGR4 NRXN1 LRP1 CADM4 SLC22A9 RTN4RL2 PTPRU TNFRSF25 TSPAN6 GYPE MRC2 GJA5 LRP12 NPR2 CDH4 FADS2 CYBA TRPV4 KCNK10 OR10H1 NCF1 HTR1E PCDHA4 INSRR MCHR1 MAS1 SLC29A1 FFAR2 SGMS2 GLRA2 LTB4R CTTN CNR1 ADAM9 CHRNA6 CDHR5 HLA-DQA1 GPR64 ULBP1 PLXNC1 CNIH2 OR51F1 IL2RG SLCO2A1 IL17RB KCNE1 PDGFRA GGT2 HTR3C TNFRSF4 TSPAN18 NGFR SLC7A4 SLC2A10 RASGRP3 TYRO3 CD52 PCDHA5 ADAM2 SLC22A14 OR1D2 FPR3 GABRR2 EFNB1 NTRK3 RHD IL2RB SSTR4 FXYD2 TSPAN9 EPHB6 KIR3DL2 GGT1 SLC2A2 SLC22A1 CD3G GRM8 CALM2 SLC22A3 SLC10A6 TSPAN12 ULBP2 SEMA6D SLC26A2 KCNJ8 CD28 DRD2 P2RY12 AQP4 SLC28A3 CD8A SLC8A2 TSPAN15 ACVR1B NPY1R CXCR5 TNFRSF18 CD33 TFRC AVPR1B CALCR GRIK3 GRIK5 PTH2R ITGA1 CD96 KIR2DL1 PLXNB1 GP6 CD6 GOLM1 SSTR1 LPPR5 SLC39A12 EFNB3 ITGB7 LRRC8D GNRHR SLC22A17 ST3GAL5 TSPAN17 IL17RA RGR SLCO2B1 MMP24 TFR2 KCNJ10 KIR2DL4 SLC34A2 CHRNA5 HTR1D STEAP3 SLC26A9 IL1R1 OPRM1 MUC1 CD9 CSPG4P5 SLC26A1 CD84 SLC5A7 TNFRSF6B KCNJ11 CHRM5 SLMAP NLGN3 SLC5A6 SLC10A2 LNPEP SLC16A5 SLC6A15 ATP6V1A SLCO1C1 NOTCH4 CHRNA1 SLC22A23 ATP2A3 ATP2A1 AQP7 EREG KCNIP4 CHRNA3 CD24 OR56A4 FCAR GRPR LRMP KCNJ12 CLEC2B NOX1 ALK GPR3 GPNMB TSPAN33 NLGN1 OPRK1 TAAR5 PILRB GPR172B ROBO1 SLC16A6 VSIG2 PRPH2 AGTR2 HS3ST3B1 SLC40A1 PPAP2C GJA4 OPN1SW SSPN ITGA11 CD97 BPI BDKRB2 TNFRSF9 GPR183 ATP7A NEO1 CAV1 SORBS1 GGTLC1 MST1R GRIN3B SLC13A3 NCSTN MARCO TM7SF2 AQP5 C8A CLCNKA PTGDR SLC6A12 O3FAR1 KIR2DL3 SCN9A SLC25A13 SIT1 KCNB2 DRD5 RAPGEF2 ITGAV FFAR3 OR9A2 CLCA1 GP1BA KCNJ4 PROKR2 RASA1 GPR4 AQP10 CHRNA4 HTR2A PCDH7 SLC2A6 GABBR2 GRIN2D B3GNT3 LYN SLC4A1 SLC28A1 P2RX5 KCNJ16 FLRT3 SLC13A5 EFNA4 TRAT1 EFNA1 HHIP RHAG STAB2 SACM1L TM4SF5 MPZ GLRA4 IGF2R ACVR2B GPR87 SYPL1 OR11H1 KLRC1 OPRD1 SIGLEC9 SLCO3A1 SLC22A7 VAMP1 TIE1 MRGPRX4 RHBDL1 AMHR2 PKD2 TRIP6 SLC6A8 GPR17 SLC6A14 KCNF1 SLC5A9 EPHB2 CCR6 SLC7A6 ADRM1 LIFR S1PR4 TLR10 NTRK2 SORL1 CHUK SLCO4C1 TREH CNGB1 FLVCR1 PTPRH GPR15 FGFR2 M6PR ACCN2 CADM2 SLC9A1 TSPAN7 SLC4A11 OR11H2 EPHA4 CNGA2 CRTAM ERBB3 SLC22A5 IL4R RTN4R SLC16A2 BDKRB1 CDON CCR9 OR10H5 SLC13A4 ITGA3 KCNC3

GO_COSTAMERE Regular periodic sub membranous arrays of vinculin in skeletal and cardiac muscle cells, these arrays link Z-discs to the sarcolemma and are associated with links to extracellular matrix. ANK2 AHNAK2 PGM5 KRT19 SYNM DAG1 DMD VCL FXR1 AHNAK KRT8 PLEC SMPX SDC4 HOMER1 FLNC SVIL ILK ANK3

GO_NUCLEAR_PORE_NUCLEAR_BASKET A filamentous, cage-like assembly on the nuclear face of the nuclear pore complex (NPC). In S. cerevisiae, Mlp1p and Mlp2p are two major components of the NPC nuclear basket. In vertebrates, Tpr is a major component. RANBP2 TPR NUP214 NUP153 POM121 POM121L2 POM121B POM121C NUP98 C15orf2 NUP35 POM121L12

GO_NUCLEAR_PORE Any of the numerous similar discrete openings in the nuclear envelope of a eukaryotic cell, where the inner and outer nuclear membranes are joined. GLE1 KPNB1 NPIP ENY2 SNUPN AGFG1 POM121L12 MYO1C NUP107 NXF1 TNKS POM121B XPOT XPO4 KPNA2 KPNA7 SEC13 NUP35 MVP NUP98 EIF5A2 IPO4 MCM3AP NUP214 RAN RANBP2 KPNA1 NUP88 POM121L2 NUP155 TMEM33 XPO7 KPNA3 NUP205 NUP85 NUP133 NUP188 KPNA4 EIF5AL1 NUP210L SUMO1 NUP37 NUP93 PIK3R4 NUP210 NUTF2 AAAS RANGAP1 KPNA5 POM121 NUP50 EIF5A MAD1L1 IPO7 TMEM48 NUP54 NUPL2 KPNA6 NUP160 C15orf2 SENP2 MX2 TPR PARP11 MAD2L1 DDX19B NXT1 RGPD8 NUP43 IPO5 POM121C NUP62CL NUP153 NUP62 NUPL1 DDX19A AHCTF1 RANBP17 RAE1 SEH1L

GO_BRUSH_BORDER The dense covering of microvilli on the apical surface of a epithelial cells in tissues such as the intestine, kidney, and choroid plexus; the microvilli aid absorption by increasing the surface area of the cell. TMPRSS15 CDHR5 SLC27A4 DNM1L RGS19 SLC46A1 SLC9A3 SLC26A4 HSP90AA1 TMEM27 SLC22A12 SLC5A6 FOLR1 MYO1B ADD3 MYO1D B4GALT1 ACTN1 MYO1A RALGDS SLC3A1 SLC6A14 SI ACTN4 EPS8 MME DAB1 SHANK2 MYH9 MYO1E MYO1C SLC26A3 ESPN GIPC1 GNA13 ATP8B1 SLC26A6 LCTL MYH10 MFSD10 HSP90AB1 GNA12 SLC2A2 CDHR2 MYH14 SLC22A5 ITPR3 SLC34A3 CD36 LRP2 KCNK1 ATP7A ENPEP PEMT VIL1 ANKS4B SLC9A3R1 SLC7A9 PTH1R SLC15A1 SLC17A3 TRPM6 SLC6A19 PEX19 SLC38A2 MYO7B PLS1 SLC34A1 FLII CLIC1 PLEC SLC5A1 MYL6 CUBN PDZD3 MYL12B DCXR CAPZA2 ITLN1 DRD5 ACTR3 SLC11A2 SLC6A18 USH1C NPC1L1 SOAT2 PDZK1 ATP6V0A4 LIMA1 AQP1 SLC34A2 CA4 EZR SCIN CYBRD1 FLNB

GO_GOLGI_APPARATUS_PART Any constituent part of the Golgi apparatus, a compound membranous cytoplasmic organelle of eukaryotic cells, consisting of flattened, ribosome-free vesicles arranged in a more or less regular stack. GALNTL6 COPG CHST6 B3GNT4 HM13 KDELR1 GALNT11 MARCH1 WNT1 ATG9A LST1 CLCN3 HLA-DRB4 SLC35C1 TRAPPC8 CLVS2 AP1B1 RND3 AP4B1 HLA-DRB1 FAM18A FUT5 B3GNT3 SNX9 HLA-DPA1 PITPNB TRAPPC4 PPIL2 MUC13 ABCA5 VCAN B2M CDC42 MGAT4B C1GALT1 SLC33A1 PROS1 GPSM1 B4GALNT4 CAV1 CHST4 TRAPPC9 GCNT7 F9 RHOQ AP1M1 TMEM115 HLA-DQB2 LMTK3 AP3D1 CLTC TPST1 KIAA0368 B3GAT3 HS6ST2 NCAM1 PLD1 GOLGA7B ARFGEF1 B3GALT4 RGP1 NDST1 DENND5A COPZ2 RAB21 CNGA2 MUC4 SLC35A5 NDST4 ST6GALNAC5 TMEM132A CNGB1 PITPNM1 CAV3 B3GAT2 LGR6 WNT3 C6orf89 M6PR PCSK6 GBP5 AP4M1 CLSTN1 GPR89A CIT RFNG NCAN RASGRP1 COG3 AFTPH PROZ GOLGA3 PRKCI GCC1 SDC4 COPZ1 RNF125 MUC17 IGF2R GCNT3 RAB32 ADC FURIN VAC14 CLTA VTI1A PDGFD GOLGA1 SACM1L C20orf30 PAM CERS1 GLG1 SORL1 KLHL12 ITM2B MUC20 PDGFA GBP1 NMNAT2 POMGNT1 RAB26 GBP3 SLC35B4 ST3GAL5 SYT17 CABP1 YKT6 GOLGA4 DBNL GALNT8 ST3GAL4 ZDHHC9 SVIP TBC1D20 GBP4 CA4 FGF23 GALNT13 STX5 AGPAT3 CHID1 MMP24 EXT1 MOB4 PCSK1N SLC35B3 AP1G1 SERINC3 RHOBTB3 GORASP1 NDST3 B3GNT5 LEPROT ANKRD28 MBTPS2 GCNT1 CNIH3 ZFYVE1 RNF13 TRAPPC10 LARGE ATXN2 MAN1A1 FAM20B SDC2 RAB39 BCAN DEFA5 WIPI1 STX16 SEC24D ZG16 NAA60 B3GALNT2 GPC2 AP2B1 YIF1A MMP11 COPA ZDHHC7 HS3ST3B1 SEC23A POSTN TMCO1 B3GALT5 B3GNT7 EMP2 ADAM10 UXS1 HLA-E SH3GL2 HLA-C FUT4 GAL3ST2 GALNTL4 MFNG CHPF FUT10 TMF1 AREG ZDHHC13 ABCB6 CTSZ DEFB103A KIAA1432 HS2ST1 CREB3L4 ATP7A RHO FUT11 STX18 MAN2A2 F7 MAP4K2 MUC21 B4GALNT3 CLN3 MUC1 HSPG2 B3GAT1 TPTE2 SLC9A7 GOLGA5 BCAP31 ACER2 RAB6B GOPC GALNT5 GOSR1 NRGN NDFIP2 GOLGA7 USO1 CHIC2 ARFIP1 NOTCH4 ST6GAL1 COG1 PGAP3 CLVS1 GPER MARCH4 SEC23IP NAGPA CLIP3 GAD2 BGLAP B4GALT7 SOD3 ASAP2 CHST3 PPP6C B4GALNT1 ERC1 GBGT1 RFWD2 ST8SIA3 FNBP1 VAPA C17orf28 SGMS2 SLC35A4 GOLT1A TRAPPC5 MGAT5 VPS45 HLA-DQB1 GPC6 PLEKHA8 OMD SCFD1 VAMP2 UBIAD1 PTGES2 RAP1GAP BECN1 CHST14 TPST2 SYS1 STX4 WNT7B HS3ST4 FUT2 HS3ST5 TRIM23 RAB38 CBFA2T3 STX6 SLC24A5 DEFA6 COG7 LRRK2 TGFBI SERPINA1 NOSIP DEFA1B ARFGEF2 NGF SLC50A1 RHBDD2 HS3ST2 GALNT9 CAMK1G GGA3 ST6GALNAC1 GALNT1 NUCB1 MGAT1 VAMP7 SEC16A AGRP PI4KA CHST11 RAB43 LFNG ARFGAP2 NUMA1 KDELR3 CLSTN2 SYT4 WHAMM HS3ST3A1 CHST12 LHB B3GALNT1 HS6ST1 SORT1 MPHOSPH9 BET1 HLA-DRB5 SEC16B OSBP CNIH2 HLA-DQA1 TRIP10 TFG CHST15 DEFA3 RAB31 OCRL CSPG4 TRAPPC1 CHST13 YIF1B VPS53 A4GALT NOTCH3 PSEN2 SH3GLB1 STX12 GALNTL2 GOLGA8IP APH1A PRKD1 IRGM GOLIM4 YIPF6 RNF24 LDLRAP1 LAP3 SULF2 MUC5AC RAB30 PLCE1 GALNT7 FNDC3A SLC35D2 RIC3 TAPBPL IMP5 SLC35B2 TRIP11 CSGALNACT2 COPG2 ARSE YIPF5 MMP14 SPPL3 SEC23B SURF4 C1GALT1C1 SCAMP3 MGAT3 GOLPH3L B3GNT9 KLHL20 ST3GAL6 ACER3 MMGT1 DNMBP RAB10 CHP RAB13 ARCN1 GAS6 RAB36 MUC3B BICD1 MGAT2 PDCD10 GLCE PPP6R3 SEC31A B3GNT1 DNAJC28 CD55 MBTPS1 QSOX2 LPCAT2 B3GNT6 RHOU MUC12 MUC19 HLA-B AGRN B3GALT2 RBFOX1 NOTCH2 MYO18A VCPIP1 NOS3 B4GALT4 PJA2 RASSF9 B4GALT2 PLK3 PARM1 GPC3 GXYLT1 GBA2 SEC24A PACS1 GCNT4 B4GALT5 HYAL2 WBSCR17 RTN3 LGR5 CHST7 PIKFYVE ZDHHC21 ST3GAL1 SLC2A4 FUT3 CSPG5 ST6GALNAC6 GABARAPL2 STX10 HLA-F ST6GALNAC3 MICALL1 BET1L RAB34 ST3GAL3 TAPBP ENTPD4 HLA-DRA GAL3ST3 IFNGR2 RAB6A FUT7 GPC5 KIAA2018 ABO NCALD TGFB1 CUX1 ST6GAL2 GALNT2 ATP8A2 SLC39A13 ACBD3 XYLT2 HACE1 SMPD4 RAB14 HLA-DPB1 DPY30 CNGA4 ST6GALNAC4 CBY1 MCFD2 WNT5B NRAS F10 AP1G2 VAMP4 OGN COG5 CPD PSEN1 SRGN C3orf58 PI4KB VAMP3 GBF1 MUC5B STEAP4 LOC100507003 RAB2A TMEM59L ABCA7 TJAP1 MAPK8IP3 GPC4 FAM57B DHCR24 ARF3 CD1E RAB12 DEFB1 PGAP2 BOK LMAN1L GAL3ST4 SLC35A2 LMAN2 ST8SIA4 XYLT1 MAN1A2 RFFL GGTA1P SEC13 FKTN NECAB3 F8 WNT3A ACPL2 RAB41 RNF121 TLR8 ATP8A1 AP3B2 GCC2 MYO1B TMEM167B ABCG1 GNPNAT1 PMEPA1 FUT9 CHST10 VRK1 GOLGB1 LALBA CYP2E1 FNBP1L D4S234E CORO7 INS EGFR IFT20 ARAP1 F2 GOSR2 RNF175 TGFA ARF1 INPP5E FMOD B4GALT6 MMD2 HLA-A MUC2 GPR89B ATP7B CYTH3 WLS CNST HMP19 TLR9 TMED9 CLTB RAC1 DNM2 GNPTG F5 TRAPPC3 COG8 FAM198B ARHGAP21 GYLTL1B AGTRAP B3GNT2 CLASP2 SDC1 GGA2 TMEM79 MUC7 TLR3 AP1S1 TMED7 MTOR MUC3A GALNT12 SREBF1 SULF1 SLC16A13 ARL3 PACSIN1 ZDHHC3 CYTH1 PKMYT1 MANEA SCAMP5 CHAC1 GOLT1B STEAP2 RAB35 AKAP9 MST4 MANEAL GJA1 RHEB DSEL GCNT2 TMED10 GOLGA2 AP1M2 MGAT5B RAB2B HEPACAM2 MUC15 VPS41 ATP2C2 QSOX1 RAB1A DEFB4A MMP16 APOO CLTCL1 ARFIP2 ATP9A ST8SIA2 MALL PNPLA8 STK16 RAB9A CLCN5 A4GNT MAN2A1 TMEM130 MUC16 AP1S2 VPS52 PCSK5 CAV2 TMBIM4 CTSC VTN B4GALT3 TMED3 ARRB1 KDELR2 CGA TMEM5 CSGALNACT1 BACE1 HLA-DQA2 DEFA1 RAB8A GOLGA8B NOTCH1 ERGIC1 GRM6 FTCD ARL1 TEX261 SLC35A3 GALNT10 TMEM59 SYBU AMPH INPP5K ST6GALNAC2 SAMD8 CHSY1 ATP2C1 STK25 NCK1 TGOLN2 TNFRSF1A UGCG B3GNT8 TAS2R16 SLC35A1 SDF4 CHST2 GALNT14 SDC3 UNC93B1 RAB7B RAB7L1 TMEM199 CHST5 AP4E1 SLC9A8 FUT1 ATP9B GBP2 SEC22B COPE C18orf26 HLA-G RAB27B HS3ST1 QPCTL CHST9 KIAA0319L GCNT6 TMED2 TRAPPC6B SREBF2 SPPL2A IL15RA EBAG9 DCN RAB1B PROC PAQR3 MAN1C1 SCYL1 DOPEY2 FAM109A SCOC SAR1B CALN1 ZDHHC17 SNAP25 HRAS UST LUM ST8SIA1 KIF13A PDGFC ST8SIA5 TRAPPC2 TYR GIMAP1 SGMS1 NCK2 NDFIP1 TNKS PRELP CHST8 BIRC6 ST8SIA6 BLZF1 USP6NL STX8 SPG21 SCAMP2 ICA1 A3GALT2P WNT7A GALNT6 FAM109B ARHGAP32 CDH1 SYNRG COL7A1 GNPTAB RABEPK FOLR1 PREB NSF DEFB103B COPB1 ENTPD6 NAPA CHPT1 CABP7 COG2 TOM1L1 FAM18B2 LLGL1 UNC50 SEC24C WNT6 GRIA1 LOC100288842 PCSK7 SNAP29 RAB11A HLA-H B4GALT1 PSENEN MUCL1 BGN EXT2 VAPB DSE GALNTL1 NSFL1C SMPD3 RGS20 GOLGA8A ARFRP1 KIAA1324 MAP6D1 CYTH2 SLC30A6 IL17RD NBEA B3GALT6 CSNK1D SPPL2B RAB11FIP5 ROCK1 CHSY3 GALNT4 CHPF2 GALNT3 MARCH9 CLSTN3 SLC2A1 CDIPT ECE2 CUL3 KERA ATL1 BSG SCAP WNT4 B3GALT1 COPB2 OPTN RAB33B TMEM167A APP LMAN2L C1orf88 GLIPR2 ATF6 CNIH ACR FZD5 SNAPIN CANT1 COG6 ARFGAP3 VPS54 MUC6 NDST2 LMAN1 FUT8 PKD1 RHBDF1 TRAPPC6A FUT6 WNT5A GAL3ST1 TNKS2 GORASP2 SCAMP1 PION C11orf2 CHST1 ST3GAL2 IER3IP1 GOLPH3 HS3ST6 TLR7 CD74 DEFA4 RAB1C GNRH1 MGAT4A AP4S1 AP1S3 CCDC115 SLC35B1 ACAN AP2A1 CYTH4 RER1 FKRP LPCAT1 SCARA3 MLANA COG4 SLC18A3 PPP6R1 MGAT4C HLA-DRB3 TMEM165 PDGFB CD59 CFTR GPC1 B4GALNT2 RASIP1 FIG4 MIA3 SEC24B

GO_ANCHORED_COMPONENT_OF_MEMBRANE The component of a membrane consisting of the gene products that are tethered to the membrane only by a covalently attached anchor, such as a lipid group that is embedded in the membrane. Gene products with peptide sequences that are embedded in the membrane are excluded from this grouping. GGT2 FOLR4 CD52 ENPP6 CEACAM5 ULBP1 TDGF1 FCGR3B BST1 CD14 RECK FOLR1 LPL CD2 NTM GGT1 EFNA2 GGT7 CDH13 RTN4R LY6G6D VNN1 PRNP GPIHBP1 LYPD1 GGTA1P GGT6 SPACA4 SMPDL3B VNN3 GGT3P TREH RHBG LY6D LYPD5 DPEP2 ART1 GML OMG DPEP3 ULBP3 NCAM1 GPC4 LSAMP CNTN6 CEACAM7 VNN2 LY6H EFNA5 LYPD2 RTN4RL2 CD48 ACHE MMP17 GFRA4 MMP25 SPRN GGTLC2 GGTLC1 LY6E CD58 NEGR1 CNTN3 PSCA GGT5 RGMB SEMA7A LY6K CNTN4 PRSS22 BST2 GPC6 EFNA1 EFNA4 NT5E ART3 C11orf34 CFC1 LY6G6C GPC5 RGMA GP1BA CD177 ALPL GAS1 TFPI LOC646627 PRSS21 GAD2 CEACAM6 CNTFR THY1 DPEP1 MDGA1 PRSS41 ALPPL2 OPCML GPC1 CD59 NTNG2 CD24 TECTB ART4 GGTLC3 PRND HYAL2 NTNG1 TEX101 TNFRSF10C MFI2 GPC3 OTOA NRN1 PRSS42 CNTN1 RTN4RL1 MSLN PKHD1 RAET1L NRN1L GFRA1 GFRA2 CD55 GPC2 CD160 PLAUR PRSS8 BCAN GFRA3 EFNA3 CD109 ALPI ULBP2 LYPD3 RAET1G ALPP LYPD6B HFE2 CPM CNTN2 FOLR2 LYNX1 LYPD4 MDGA2 CA4 SVIP SPAM1 XPNPEP2 CNTN5 ITLN1 CPO GP2 UMOD TECTA CEACAM8

GO_MHC_PROTEIN_COMPLEX A transmembrane protein complex composed of an MHC alpha chain and, in most cases, either an MHC class II beta chain or an invariant beta2-microglobin chain, and with or without a bound peptide, lipid, or polysaccharide antigen. HLA-DQA1 HLA-DRB4 HLA-DRB5 HLA-B HLA-C HLA-DMB HLA-E HLA-DMA HLA-DRA HLA-G B2M HLA-DOA HLA-DOB HLA-DPB1 HFE HLA-DPA1 HLA-H CD74 HLA-DQB1 HLA-A HLA-DRB3 HLA-DQB2 HLA-F HLA-DRB1 MR1 HLA-DQA2

GO_INNER_MITOCHONDRIAL_MEMBRANE_PROTEIN_COMPLEX Any protein complex that is part of the inner mitochondrial membrane. CHCHD6 SPG7 MCU COX5B NDUFA6 LOC100652748 ATP5I PARK7 BCS1L SDHA C14orf2 NDUFB4 NDUFA4L2 ATP5H ATP5J IMMP1L NDUFAB1 PAM16 ATP5L ATP5J2 APOO IMMT ATP5L2 NDUFS1 CCDC109B NDUFA5 TIMM23 ATP5F1 NDUFA10 FOXRED1 C15orf48 NDUFV3 ATP5EP2 TIMM50 NDUFS4 NDUFS5 NDUFS6 NDUFAF1 GRPEL1 MICU1 ATP5O UQCRC1 NDUFB11 WDR93 NDUFS7 ATP5C1 NDUFB9 COX4I1 NDUFB8 NDUFA7 PPIF NDUFS2 TIMM9 ATPIF1 USMG5 NDUFA1 NDUFB2 ATP5G3 PMPCB ATP5E NDUFB3 NDUFA12 SDHD ATP5G1 NDUFS3 NDUFA3 CHCHD3 COX8A COX5A APOOL NDUFA8 NDUFV1 COX4I2 IMMP2L TIMM10 ATP5A1 NDUFS8 TIMM17A TIMM17B UQCRC2 NDUFB6 COX6A1 C22orf32 NDUFA2 NDUFB10 SDHB GRPEL2 ATP5D C19orf70 NDUFB1 COX6A2 SDHC NDUFV2 UQCRFS1 NDUFA13 SLC25A6 UQCR10 C11orf83 NDUFA9 ATP5G2 NDUFC2 UQCRB TIMM22 SNCA NDUFB5 ATP5B NDUFA4 NDUFA11 NDUFB7 NDUFC1 EFHA1 C18orf55 COX8C AFG3L2

GO_TRANSPORT_VESICLE_MEMBRANE The lipid bilayer surrounding a transport vesicle. SPRED2 SREBF2 TMED2 AP1G2 PI4K2A HLA-G B2M SYT2 MCFD2 SEC22B TMEM163 SYN2 HLA-DPB1 VMA21 SAR1B CLTC HLA-DQB2 LRRK2 SLC32A1 SLC18A1 HLA-DRB4 CLCN3 HLA-DRA GAD2 SEC23IP HLA-DPA1 VAMP2 CUZD1 KIAA1244 HLA-DQB1 HLA-DRB1 PTPRN NCALD SVOP SYTL4 CNIH2 HLA-DQA1 DMXL2 SYT10 VTI1A SYNGR1 HLA-DRB5 BCL2L1 FOLR1 SYNPR CLTA VAMP1 SYPL1 RAB26 HLA-H SLC30A8 GRIA1 HLA-A DBH SEC24C SV2B TGFA RAB11B GOSR2 VTI1B SYT12 SYT5 VAMP7 SV2C SLC17A6 SYNRG AFTPH RPH3A SYT9 SYT4 DOC2A ICA1 SEC13 SNTB2 DRD2 SCAMP5 STX17 SLC6A17 CNIH3 ABCC8 SYN3 SYCN C19orf26 SREBF1 SYN1 AP1S1 TMED7 CPE TMEM30A SEC31A TMED10 SEC24D SNAPIN SYT3 SYNGR4 CNIH SCGN CEACAM1 AQP6 SCAP SEC31B CHGA LDLRAP1 TMEM184A SLC17A5 SYPL2 SYT6 AQP2 CLTB SEC23B ATP6V1G2 SLC17A7 RAB11FIP5 SV2A SYNGR2 STX1A CA4 STX5 OTOF SLC18A2 SLC30A3 DTNBP1 DNM1L SEMA4C AP2A1 SEC24A SYP PTPRN2 AMPH SEC24B USO1 NRGN SYT1 CD59 SLC18A3 HLA-DRB3 HLA-F HLA-DQA2 RAB3A HLA-B HLA-E HLA-C SLC17A8 LMAN1 SYNGR3 ZNRF1 SEC23A GABRA2 SYT11 CD74 RPH3AL SCAMP1 RASSF9 SYT7 AREG CLTCL1 SCG3

GO_MICROVILLUS Thin cylindrical membrane-covered projections on the surface of an animal cell containing a core bundle of actin filaments. Present in especially large numbers on the absorptive surface of intestinal cells. SLC10A2 ATP6V1A MYO7A TBC1D10A STARD10 CDHR5 CLIC4 SLC27A4 FOXA1 ATP6V1B1 HYAL2 S100P ANGPT1 PDGFA MUC20 IFT20 DPEP1 CTSL2 MYO1A MYO6 FMN2 ENPP7 GRXCR2 GRXCR1 CLRN1 ESPN CA2 MYO1C ITGB3 MSN VIL1 PROM1 IQGAP2 CDHR2 FSCN1 RDX CRB1 ATP6V1B2 MYO7B AKR1B1 OXTR SLC9A3R1 CTNNB1 SCARB1 ANKS4B BBS2 VCAM1 KIF13B PDPN LRRK2 PLEKHG6 PROM2 EXOC4 MYO1G AQP5 NFASC USH1C ATP6V1E1 WWOX RAPGEF3 CA9 CLCA1 ITGAV PODXL DCXR TGFB1 EZR CNP GIF TEK CD302 PDZK1 AOC3

GO_ACTIN_FILAMENT_BUNDLE An assembly of actin filaments that are on the same axis but may be oriented with the same or opposite polarities and may be packed with different levels of tightness. MYLK LCP1 SORBS1 FHL3 SIPA1L3 SHROOM4 PLS3 PGM5 PLS1 MYL9 CNN2 MST1R CRYAB PTK2 FBLIM1 SYNPO NOX4 ZNF664-FAM101A ACTN1 MYH6 ABLIM3 FAM101B SEPT7 SEPT11 ACTN4 ILK ACTA1 FERMT2 MICALL2 MYO1C MYL12B MYH9 CORO1B DAAM1 TPM4 PDLIM7 MYH10 CYBA FHOD1 VANGL2 FAM101A FSCN1 MYH14 ZYX TPM1 PXN TPM3 LIMA1 NEBL FLNB VIL1 TEK SEPT9 MYH7 SEPT12 ABLIM1 PDLIM2

GO_SITE_OF_DOUBLE_STRAND_BREAK A region of a chromosome at which a DNA double-strand break has occurred. DNA damage signaling and repair proteins accumulate at the lesion to respond to the damage and repair the DNA to form a continuous DNA helix. RPA2 ATF2 MRE11A APLF SMC5 PRPF19 RNF168 RNF138 SMARCAD1 RAD50 ANKRD32 KDM4D HUS1B SMARCAL1 SMC6 RFWD3 HUS1 C9orf142 H2AFX SETMAR VCP RAD18 FAM178A PARP3 ESCO2 PHF1 HELB RNF169 RAD51 NBN RNF8

GO_PROTEIN_KINASE_COMPLEX A protein complex which is capable of protein kinase activity. CKS1B CDKN1A CCNE1 CCNK PRKAA2 PHKG1 CKS2 TGFBR1 PRKAR2A NEK10 INSR CDK2 GTF2H4 PRKAR1A ENG ERN1 CCND2 PRKAG3 ERC1 CDK9 FAM58A ACVR1C PYDC1 SNW1 PRKAR1B CDK12 TRIM40 CCNC PRKAB1 PRKAB2 PRDX3 PHKB HSPD1 TGFBR2 MAP3K5 ERCC3 GTF2H1 IKBKG PRKACB MAP3K13 CDK14 PHKA1 CCNT2 SORBS1 PRKACA CDK6 GTF2H2 ACVR1B ULK1 MMS19 RB1CC1 CDK4 PYCARD MNAT1 DAB2 ATG13 GTF2H3 CCNT1 IKBKB CCND3 FAM58BP TBC1D5 RB1 CCNH CCND1 ERCC2 PRKAG2 IRS1 SESN2 CCNY TAF7 CCNL2 CAB39 ACVR1 MAP3K7 BCCIP PRKAA1 PHKG2 PHKA2 HOXC10 ERCC5 CCNL1 PRKAG1 CDK7 CHUK PRKAR2B PUF60 CDK13 KDM5A AKAP4

GO_COPI_VESICLE_COAT One of two multimeric complexes that forms a membrane vesicle coat. The mammalian COPI subunits are called alpha-, beta-, beta'-, gamma-, delta-, epsilon- and zeta-COP. Vesicles with COPI coats are found associated with Golgi membranes at steady state. COPZ1 COPB1 TMED3 ARCN1 COPZ2 COPG2 SCYL1 C3orf58 COPA COPE COPB2 COPG TMED7

GO_NUCLEAR_CHROMATIN The ordered and organized complex of DNA, protein, and sometimes RNA, that forms the chromosome in the nucleus. CREB1 PCGF2 INO80 TCF4 KLF4 KIAA1967 INO80C HEY2 BRMS1 HIST1H2BG SMARCD3 ING3 KDM4C TTC21B PPARGC1A HIST1H2AB HIST2H2BD RELA MBD2 NR1H3 HIST3H2BB HIST1H2AC TCF7L2 MXD1 RBMX JUNB NUFIP1 E2F1 KAT2A EME2 SMARCC2 HIST1H2AM ATRX RCC1 H2AFB2 H3F3C TRIM24 ING2 RAD51 SFR1 CBX3 DLX5 EED MBD3 GATAD2B JUN DFFA ESR1 MEN1 HIST1H2BL IKZF1 MORF4L1 H2AFY ASH2L DVL3 EZH2 HIST1H2AE MCRS1 HIST1H2AI DDX11 FAM60A ALKBH1 SP1 NUCKS1 SMAD3 HNRNPK SMAD2 HIRA FOXH1 HIST2H2AB ACTB SUZ12 ASF1A SIRT6 HIST3H3 SWI5 MYOD1 BEND3 ACTR5 CBX1 HIST1H1B POLR3G TBP BRD8 HIST1H1D CHAF1B UHRF1 RUVBL1 TNKS1BP1 HIST2H2BE CALCOCO1 UCHL5 HIST1H2AD IPO4 CBX5 DBF4B NRIP1 RAD51AP1 DFFB HIST1H2BO RUNX3 TFPT SMARCD2 H2AFJ SMARCB1 CEBPB NPM2 DPF2 RUVBL2 HIST1H2BI H2AFZ HIST1H2AA HIST1H2BA SIRT2 HIST1H3C TRNP1 RBBP4 AR HIST2H2AC HIST1H2BE HIST1H1E MPHOSPH8 SAP130 ASXL1 EME1 RUNX2 H2BFM ZFP57 CREBBP NFRKB ASF1B RFX3 TP53 KLF1 JUND HIST1H2BC HIST2H2AA3 CENPA PPARD NCAPD3 TRIM28 BUD31 CSNK2A1 HIST1H2AH HIST1H2BH SETD3 HIST1H3F SMARCA4 INO80E PHOX2B HIST1H3I NCOA3 TCF12 HIST2H2BF TRPS1 HIST2H2AA4 DNMT3A NR1D1 KLHDC3 SMAD4 GATA3 TIPIN PHF12 H2AFV HIST3H2A IRF4 ACTL6A HIST1H2BJ TCF7 SCRT2 SIRT1 SOX18 STAT1 IRF1 SUV420H2 PAX6 SIN3A HIST1H2BN CBX7 ZNF385A WBP2 NELF PHOX2A SUDS3 H3F3A SMARCA2 UHRF2 EHMT2 TARDBP MIXL1 DNTT H1F0 H2AFB3 HIST1H2BD UBE2U T CHD4 H2AFY2 HIST1H2BF FOXD3 HDAC1 SMARCE1 POU4F1 NASP SNAI2 MSH6 UBE2A SIRT7 HAT1 TIMELESS HIST1H2BK ZNHIT1 UBE2B KAT5 ETV3 HMGB2 TP63 TRRAP H2AFB1 STAT3 H3F3B TCF3 MEF2A HIST1H3H ESCO2 PLCB1 INO80B MBD1 LDB1 SLX4 FOXC1 KDM1A STAT6 HIST1H3B HIST1H2AL E2F4 HIST1H2BM HIST1H3D ANP32E SATB1 THRB YY1 HIST1H2AK NACC2 HIST1H3A POLR3D RXRA HIST1H2AG TAL1 POLR3GL PAWR NFATC1 TCP1 ARID1A H2AFX CHAF1A FER HIST1H3E SETD1A NCOA1 MUC1 NR1H4 HDAC2 RARG EP400 HIST1H3G GABPA ACTR8 CXorf27 NCOR2 SPI1 RAD50 HNRNPC USP3 CITED2 RNF2 BRMS1L HIST1H3J H2BFS SIN3B HAND2 RARA CDCA5 NCOR1 SRF CTNNB1 APTX HIST1H2BB H2BFWT CBX8 RING1 ENC1 MCMBP HIST1H1A H1FNT POGZ SMARCC1 MTA2 HIST1H1C

GO_PLASMA_MEMBRANE_RECEPTOR_COMPLEX Any protein complex that is part of the plasma membrane and which functions as a receptor. CHRNE SORBS1 CD4 ZAP70 HTR3E B2M CD8B SKAP1 OLFM3 GRIN3B ITGB4 BMPR1B ITGB6 VWC2L CHRND DIABLO INSR ITGAV TRAF6 CARD11 CHRNA4 ITGB8 GRIA2 CACNG2 ACVR1C LYN GRIN2D GABBR2 CHRNB4 GRIN2B TGFBR1 GRIN3A TRAF2 TRAT1 CACNG5 STOML2 CHRNA6 CNIH2 SACM1L CD40 GRM1 SHISA8 SHISA7 CD3E DLG3 ITGA2 GRID2 ITGAD TRIP6 GRIA1 HTR3C APBB1IP ITGAX ITGA4 TOLLIP EPS8 HTR3D ITGA10 BCL10 CHUK BMP2 IL28RA MYH9 GRIN1 CACNG4 TF IL23R CD3G NPNT ITGAM DLG2 ITGA2B CD79A IRAK1 ITGA3 IKBKB IRS1 ABHD6 IL13RA1 IL12RB1 CNIH3 NOS1AP ACVR1B HFE2 HFE SYK ZACN ITGAL CD8A OLFM2 ITGB1 ITGA1 GRIK5 HTRA2 CSF2RB ABHD12 TFRC STXBP5 IL6 TLR1 CD6 CHRNB3 CACNG7 TGFBR2 ITGA8 CEACAM1 ITGB7 VWC2 TLR6 ENG SHISA6 CHRNA9 CHRNB2 DLG4 TRAF3 PORCN CACNG3 HTR3B OSMR TFR2 GRIA3 CD3D DLG1 TSPAN32 ALCAM GRIK2 CHRNA5 ITGA6 IL6R BIRC2 IL6ST RNF31 GABBR1 ITGB2 SHISA9 CD79B BMPR1A CPT1C CNTFR HTR3A ITGA7 CHRNA1 IL10RB SHANK1 ITGB5 CHRNA7 CHRNA3 CHRNA2 GRIA4 CHRNA10 SHANK2 GRIN2A ITGA5 ACVR1 IL29 TRAF5 CHRNB1 NLGN1 PTK2B PTPN6 GRIK4 ITGA9 SDCBP DAB2 CHRNG TLR2 CD247 CACNG8 ITGB3 KCNK1 GRIN2C ITGA11 ITGAE

GO_CLATHRIN_COATED_VESICLE_MEMBRANE The lipid bilayer surrounding a clathrin-coated vesicle. EGFR HLA-DQA1 CD9 HLA-DRB5 CLTA AP2A1 AP1S3 FZD2 AP1S2 APOB CLVS1 HSPA8 MYO6 NRGN SYT1 HIP1 HLA-DRB3 SLC18A3 HLA-DQA2 RAB3A WNT5A SGIP1 SH3GL2 LDLR NECAP1 CD74 SYNRG AP2A2 DAB2 AFTPH PICALM AP3B2 TBC1D5 KIAA1199 AP2M1 RASSF9 DNAJC5 CLTCL1 AP1M1 FZD4 DENND1A EPN2 ROR2 GAD1 AP1G2 HIP1R HBEGF AP1S1 HLA-DPB1 AP2B1 EPS15 AP1M2 AP3B1 FZD5 VAMP3 CLTC HLA-DQB2 NECAP2 TYRP1 AP2S1 SLC32A1 HLA-DRB4 CD207 SLC18A1 DBNL LDLRAP1 FCGR1B CLTB HLA-DRA GAD2 SLC17A7 HLA-DPA1 VAMP2 AP1G1 HLA-DQB1 SLC18A2 HLA-DRB1 FCGR1A NCALD CLVS2 AP1B1

GO_OXIDOREDUCTASE_COMPLEX Any protein complex that possesses oxidoreductase activity. NCF1C PARK7 DBT NDUFA6 BCS1L NDUFB4 SDHA RRM1 GPD2 NDUFAB1 P4HA1 NDUFS1 BCKDHA NDUFA5 NOX1 OGDHL RRM2B NDUFV3 GMPR2 NDUFA10 FOXRED1 NOXA1 BCKDK NDUFS4 NDUFS5 NDUFS6 BCKDHB UQCRC1 WDR93 NDUFB11 NDUFAF1 NDUFB9 NDUFS7 NDUFB8 NDUFS2 NDUFA7 CYBB PDK2 NDUFA1 NDUFB2 CBR4 PDHB GLDC PMPCB NDUFB3 GPD1 GPD1L OGDH NDUFS3 NOX3 PDHX DLST NDUFA12 SDHD RRM2 NDUFA3 NDUFA8 NDUFV1 NCF2 PDHA1 NDUFS8 UQCRC2 P4HB DLD DLAT NDUFB6 NDUFA2 NCF4 MRPS36 PDHA2 NCF1 NDUFB1 CYBA NDUFB10 SDHB NDUFV2 DHTKD1 UQCRFS1 GCSH SDHC NDUFA13 GMPR NCF1B UQCR10 C11orf83 NDUFA9 UQCRB NDUFC2 SNCA NDUFA4 NDUFB5 NDUFA11 PDK1 NOX4 NOXO1 NDUFC1 NDUFB7

GO_TRANSCRIPTION_FACTOR_TFIID_COMPLEX A complex composed of TATA binding protein (TBP) and TBP associated factors (TAFs); the total mass is typically about 800 kDa. Most of the TAFs are conserved across species. In TATA-containing promoters for RNA polymerase II (Pol II), TFIID is believed to recognize at least two distinct elements, the TATA element and a downstream promoter element. TFIID is also involved in recognition of TATA-less Pol II promoters. Binding of TFIID to DNA is necessary but not sufficient for transcription initiation from most RNA polymerase II promoters. ERCC4 TAF9B EDF1 ERCC1 TAF4 TP53 TAF4B TAF13 TAF11 TAF1 TAF1L TAF10 TAF3 TAF12 TAF9 TAF7 TBP TAF8 TAF7L TAF5 TAF2 TAF6 SUPT3H

GO_INCLUSION_BODY A discrete intracellular part formed of aggregated molecules such as proteins or other biopolymers. PSMC5 PSEN1 XRN2 NBN MECOM TPR PINK1 HOXD3 WDFY3 EPS15 UBQLN1 GIT1 PABPN1 HAP1 HSPA1A TRIM66 FBXO7 SNCA LRRK2 NUP153 C7orf28B RAD18 FAM125A EID1 CCZ1 HSPA1B HSPB7 PSMC4 ATXN1 MT3 UCMA SEC62 DBF4B RANBP2 POLD1 MAPT BAG5 ANKRD32 STUB1 SYNE2 MIOX GYS1 SLFN11 SFMBT2 EVX1 HDAC6 ATF4 RNF32 RAB11B GPX1 ATXN3 TRIM37 CLU NXF1 NEFH EEF2 SNCB HERPUD1 CDH1 HSP90AB1 CABIN1 NEFM PARK2 UBD NUP98 PICALM SQSTM1 URB2 ORC6

GO_COMPACT_MYELIN The portion of the myelin sheath in which layers of cell membrane are tightly juxtaposed, completely excluding cytoplasm. The juxtaposed cytoplasmic surfaces form the major dense line, while the juxtaposed extracellular surfaces form the interperiod line visible in electron micrographs. PRKCI PLLP PTEN MBP PMP22 SIRT2 JAM3 MPDZ MPP5 AKR1B1 CD59 MAG C1orf130 MARVELD2 ANXA2

GO_VIRION The complete fully infectious extracellular virus particle. ERVW-1 ERVK-7 ERVFRD-1 ERVK13-1 ERVK-6 ERVK-5 ERVMER34-1 ERVK-21 HSBP1L1 ERVFC1-1 B7H6 ERV3-1

GO_SIDE_OF_MEMBRANE A cellular component consisting of one leaflet of a membrane bilayer and any proteins embedded or anchored in it or attached to its surface. GEM IGHV3-23 PTK6 TNFRSF9 NRCAM TNFRSF14 GNG3 IGLL5 GNG13 CYLD CD83 NLGN1 RASAL3 GNAT2 KLRC4-KLRK1 PKHD1 TLR4 HLA-C BMX HLA-E RTN4RL1 MAP2K2 IQGAP1 ATP2B1 TGM3 TH HLA-DQA2 CACNB4 CD24 GNG5 LILRB1 PDCD1 BCAP31 ANXA1 S1PR1 HEG1 GNB1L FERMT2 SLAMF1 FYN GNGT1 HSPA8 SYNGAP1 FER IL7R RNF31 CAV2 RGS1 CD9 EEF1A2 ECE1 RGS19 ARRB1 PTPN4 ITGA6 SELL TFR2 CA4 SDC1 PTPN3 TRAF3 RASA3 CHRNB2 LOH12CR1 DNAJA1 RAB5A GNA15 KCNAB2 IL1RL1 UMODL1 GNA14 IL6 GFRA3 MATK CD33 NRG1 TFRC ITGB1 P2RX1 ITGA1 P2RY12 AQP4 CD27 ABL2 IL2RA THBS1 CD8A RASA4B FARP1 PTK2 CXCR5 GNAI1 CYTH1 CD80 CD28 SHROOM4 S100A6 IGLC7 TRPV1 LAG3 GPIHBP1 ITGA3 IKBKB GGTA1P ALOX15 ITGA2B IGLC3 CD36 PTPN7 CDH13 YES1 GM2A MUC17 GNA12 CXCL9 ITK SNX18 IGHM GNA13 CHMP4A RAB21 RGS2 GGT3P CEACAM5 CLPTM1 BLK CCR5 CHUK IGHA2 CD34 PKD2 FAS HLA-A SRC NF1 CYTH3 ITGA2 IGHV1OR21-1 JAK3 ABCG1 DSG1 IGHV4OR15-8 KCNIP1 GNAO1 CD3E STAB2 MFGE8 PGM5 TMEM123 GNB1 IGHA1 TXK GNG4 HLA-DRB1 TRAF2 KCNJ5 SNX9 STYK1 CD200R1 LYN TEC SEMA7A ICAM1 IGHG2 HLA-DPA1 RACGAP1 RASA1 IGHD CHRNA4 RGS9 PLG HLA-DRA ITGAV TRAF6 FGA HM13 FGG GNAZ HLA-DRB4 CLCN3 GP1BA RGS7 G6PD ASPSCR1 CXCR3 HLA-DQB2 GLRA1 GPC4 NCAM1 GGTLC1 DAB2IP HLA-DPB1 JUP F10 FRK ZAP70 B2M PTPN1 CD226 KIT CHMP4B ITGAE RASAL1 GNG11 JAK2 MS4A1 IGHE CD5 CD74 PTK2B ICOS CD19 TRAF5 SPTA1 CTLA4 FCER2 GNG2 ACP1 PTEN RASAL2 HLA-B ITGA5 P2RX7 SPTB CHRNA7 SCNN1B IGLL1 SLC2A4 CD59 PCSK9 RHOA HLA-F CXCL10 FCER1G HLA-DRB3 TAPBP GNAI3 IL13 ERRFI1 PTPRC KCNJ3 THY1 EXOC1 CTSA CD79B FCER1A BMPR1A GNRH1 IL6ST KRAS TNF GGTLC3 GNG7 FGB MCAM CCR7 HYAL2 ABL1 SERPINE2 TGFBR3 BIRC2 RLTPR DTNA MS4A2 GPR125 NPHS2 DLG1 CD69 ALCAM IMP5 SPPL3 HCK SPPL2B IFNG FCN1 TNK1 TRDC SPN TNFRSF11A DLG4 SERPINA5 SNX5 CDH5 IGKC BTK FCRL6 CD274 ENG CD40LG CD276 LDLRAP1 CCR1 IGHG1 FES TGFBR2 ASTN1 IGLC1 CUBN TYK2 HTRA2 IGHG4 ANXA5 GNG8 GNAT3 HFE SYK PTP4A1 CSK FOLR2 IL12RB1 RGS6 CXCL12 NTSR1 MIEN1 ENPEP GGT6 ENOX2 ADA FASLG RGS8 CD79A GGT7 CDH1 IGLC6 GGT1 LDLR SPA17 GNG10 KCNAB1 IL2RB TNFRSF13B TF TIAM1 IL4 CANX ABCA1 ANPEP ACE TNFRSF13C SRMS CD200R1L F3 BDH1 RASA4 ATP2A2 GSR RGS11 GNG12 ZFYVE20 GGT2 AMOT CTSL2 B4GALT1 CALR HLA-H SLC22A11 IL2RG GFAP SELP CD2 KLRK1 EMR1 FOLR1 RASA2 HLA-DRB5 CD14 ADAM9 IGLC2 HLA-DQA1 CD40 C11orf34 EPHA5 CD86 FGR DNAI2 BCAM IL17A EZR MYZAP CDK16 P4HB FGF8 HLA-DQB1 ESYT2 GGT5 EEF1A1 SCNN1G CCR4 PTPN22 SCUBE1 KLRD1 FCGR3A TNK2 VTCN1 DIABLO TAS2R16 GNGT2 LRRK2 PKP4 IL12RB2 EFNA5 ANTXR2 RTN4RL2 GNAT1 MAP3K5 LCK GNAI2 PDPN ESYT3 TMC1 JAK1 ITGB6 NUCB1 CNR2 TRPM8 GGTLC2 IL31RA IGHG3 CD8B FLT3LG HLA-G CD4 VCAM1 LAMP1 SPPL2A CD244

GO_HISTONE_METHYLTRANSFERASE_COMPLEX A multimeric complex that is able to catalyze the addition of methyl groups to histone proteins. PRMT5 PHF1 TAF9 TAF1 DPY30 CXXC1 MLL4 PPP1CC STK38 DYDC2 SUZ12 ACTB JARID2 HCFC1 PELP1 C16orf53 MLL3 MTF2 EED RNF2 HDAC9 MLL5 PHF19 KAT8 SENP3 TEX10 E2F6 WDR82 MCRS1 DYDC1 EZH2 H2AFY ASH2L CHD8 PRPF31 MAX MEN1 KIAA1267 PRDM4 AEBP2 SETD1A PPP1CA SETD1B TAF6 EZH1 CBX5 MGA RBBP7 MLL RBBP4 H2AFY2 RBBP5 PAXIP1 HDAC2 LAS1L PHF20 NCOA6 RUVBL2 TAF7 INO80C TRIM37 MLL2 ZNF335 PPP1CB C17orf49 KDM6A WDR5 OGT RUVBL1 TAF4 SIRT1

GO_CLATHRIN_ADAPTOR_COMPLEX A membrane coat adaptor complex that links clathrin to a membrane. AP1S1 AP3M1 AP2A1 AP1G2 SGIP1 EGFR LDLRAP1 AP1M1 AP4M1 AP4B1 AP2M1 AP2S1 AP3M2 GGA1 AP1B1 SLC18A3 TBC1D5 AFTPH PICALM EPS15 AP2A2 AP1M2 GGA3 GGA2 AP3B1 AP1G1 SYNRG AP2B1

GO_NUCLEOTIDE_EXCISION_REPAIR_COMPLEX Any complex formed of proteins that act in nucleotide-excision repair. POLE2 POLE4 POLE3 XPA POLD1 ERCC1 POLE XPC ERCC8 CHRAC1 CETN2 SLX4 RAD23B ERCC4

GO_EXTRINSIC_COMPONENT_OF_ORGANELLE_MEMBRANE The component of an organelle membrane consisting of gene products and protein complexes that are loosely bound to one of its surfaces, but not integrated into the hydrophobic region. COQ5 ATG14 DUSP21 NOA1 COQ7 SOX10 ULK1 WDFY3 SNX5 ZFYVE1 C17orf28 SNX10 TAMM41 TOR1A PAM16 ZC3H12A USP8 ANXA1 MMP27 SARM1 GOLGA3 C20orf7 RAB7A OPA1 NUCB1 SNX16 PML

GO_SEX_CHROMOSOME A chromosome involved in sex determination. SMC6 SIN3B SMARCC1 H2AFY UBE2B H2AFY2 PBX4 SUZ12 SMARCB1 RING1 PLK4 SMC5 UBE2A H2AFZ SMCHD1 BIRC2 CDK2 DMRTC2 MAEL ESCO2 EED RNF2 DNMT3A H2AFX PCGF2 SUMO1 H3F3B H3F3A

GO_ENDOPLASMIC_RETICULUM_EXIT_SITE An endoplasmic reticulum part at which COPII-coated vesicles are produced. YIPF5 VAPB SEC31A MIA3 PDCD6IP VAPA TMED5 PDCD6 APOB PREB MPPE1 CTAGE5

GO_DNA_POLYMERASE_COMPLEX A protein complex that possesses DNA polymerase activity and is involved in template directed synthesis of DNA. CRCP DNA2 CHRAC1 REV3L POLD3 POLE2 POLE4 POLE3 MAD2L2 POLD4 POLE POLD1 POLG

GO_NUCLEAR_HETEROCHROMATIN A condensed form of chromatin, occurring in the nucleus during interphase, that stains strongly with basophilic dyes. The DNA of heterochromatin is typically replicated at a later stage in the cell-division cycle than euchromatin. CBX3 UHRF1 RNF2 EED ZFP57 MPHOSPH8 EME1 DNMT3A PCGF2 HIST1H1E FOXC1 CBX1 HIST1H1B H2AFY SUV420H2 IKZF1 SIRT1 TNKS1BP1 SATB1 ESCO2 UHRF2 NCAPD3 TRIM28 TCP1 CBX5 BEND3 SIRT2 SIRT6 ATRX H2AFZ SUZ12 RING1

GO_XY_BODY A structure found in a male mammalian spermatocyte containing an unpaired X chromosome that has become densely heterochromatic, silenced and localized at the nuclear periphery. SIN3B BIRC2 DMRTC2 SMARCC1 UBE2B ESCO2 MAEL PBX4 SMARCB1 PLK4 SUMO1 UBE2A H2AFX DNMT3A

GO_ACROSOMAL_MEMBRANE The membrane that surrounds the acrosomal lumen. The acrosome is a special type of lysosome in the head of a spermatozoon that contains acid hydrolases and is concerned with the breakdown of the outer membrane of the ovum during fertilization. SPACA1 TRIP11 ZP3 CD46 FLOT2 PLA1A RND2 CAV2 SERPINA5 SPACA3 TEX101 FAM170B ATP8B3 SUN1 TMEM190 PCSK4 BSG IZUMO1 TEKT3 TMEM225 C9orf11 ACRBP CAV1

GO_ENDORIBONUCLEASE_COMPLEX A protein complex which is capable of endoribonuclease activity. POP1 RPP40 EIF2C2 DGCR8 TARBP2 POP7 DICER1 ZFP36 TSEN34 TSEN2 RPP30 ERN1 RPP21 TSEN54 DROSHA EIF2C4 EIF2C1 RPP38 EIF2C3 POP5 CLP1 POP4

GO_EXOCYST A protein complex peripherally associated with the plasma membrane that determines where vesicles dock and fuse. At least eight complex components are conserved between yeast and mammals. EXOC3L1 MYRIP EXOC7 WASH1 EXOC1 RALB EXOC4 EXOC6B EXOC3 EXOC3L2 SEPT2 EXOC6 EXOC5 EXOC3L4 TNFAIP2 EXOC8 STXBP6 RAB10 EXOC2

GO_MEMBRANE_PROTEIN_COMPLEX Any protein complex that is part of a membrane. GABRA3 PIK3C3 TGFBR2 ATP6AP1 AP3B1 STX1B OS9 RAB7A AP3S2 SMAD7 SYK CLIC2 ABCC8 DLG1 TRDN LRP5 TOMM40L SEC23B COX4I2 NCF2 ATP6V0A4 CLDN17 STX1A CACNA1E APH1B SNX5 DLG4 LDLRAP1 INSIG1 ENG APH1A BET1L APC STX10 HLA-F CYBB NOXA1 STX2 FZD8 TOMM5 BMPR1A ATP6V0E1 NDUFA4L2 SDHA NCF1C CNGA3 NDUFV3 PTK2B PTPN6 KCNG3 INSIG2 GRIA4 SRPR C7 ITGA5 HLA-DMB CDH2 LRP4 PIK3CA C11orf83 UQCRFS1 UBXN1 VMA21 GABRA5 GGA3 CACNA1F EFHA1 BECN1 BECN1P1 NDUFA11 VAMP5 VCAM1 KCNB1 PIK3R5 PIGK STX4 GRIN2B GRIN3A SCN11A NDUFB6 VAMP2 GABRR1 SLC6A3 C19orf70 MPP7 ANO6 VPS29 DOC2B HTR3D KIAA0415 SEC61B SGCB TOLLIP NDUFS3 COL13A1 TRIM27 AUP1 KCNH1 CAMK2D NDUFB2 CD40 GRM1 VDAC1 HLA-DRB5 TBC1D5 SNTG2 KCNA2 ATP5J ATP1A4 ITGAM SNX4 TTYH1 NOS1 MEP1A KCNAB1 PKD1L3 FOXRED1 GNG10 MYH9 TSG101 TF NDUFA5 TSNARE1 TIMM22 GABRG1 AP2S1 KCNS1 CACNG7 AP2B1 ITGB1 SEC24D ABCF2 ANKFY1 CACNB3 KCNV1 PRKACA TOMM7 CNIH3 KCNJ1 VPS26B SVIP KCNV2 PORCN AP1G1 UQCRC2 GRIA3 STX5 CHRNB2 KCNA4 TRAF3 KCNAB2 YKT6 TLR6 CLIC5 NDUFB9 SGCZ STX19 GOSR1 USMG5 ITGB5 CHRNA2 BCAP31 COX4I1 CPT1C FLOT1 WDR93 TRPC5 NDUFAF1 ATP1A1 TOMM22 COX5A KRT19 RGS19 CACNG1 PKD1L1 GJA3 ATP5H SPTLC3 GNG3 SYNJ1 STX18 PICALM CD247 CHMP2A NDUFA6 ATP6V1D GRIN2A SGIP1 HLA-E UFD1L ERN2 GJB6 AP3D1 CLTC FAM125B ANO2 ITGB4 UBXN7 SCN10A GLRA1 ZAP70 CASP3 KCNQ3 SKAP1 AP1M1 CATSPER2 NRBF2 HLA-DRB1 KCNE2 AP1B1 AP4B1 LRRC8A IMMP2L APOOL HLA-DPA1 TIMM17A CHRNB4 ATP6AP1L HLA-DOA KCNMB4 PIK3CD PKD2L1 KCNE1L COPG SUMO1 HM13 TRAF6 ITGA10 ATP1A2 BCL10 SSR4 BMP2 GRID2 CATSPERB ITGAD NDUFB11 CALM1 KRT8 VAC14 CLTA CACNA2D2 TCIRG1 KCNIP1 PGM5 TUSC3 SHISA7 VTI1A PDE4B CACNA1G SPCS3 CHCHD6 AFTPH KCNK6 COPZ2 VPS16 CAV3 CACNG4 SGCD ABCA2 CHRNB3 PIGU C14orf133 COPB2 SCN8A KIAA0090 ABHD12 STX11 BEST4 DDOST NDUFA4 ATP1B4 CHMP6 SCN4A MS4A2 ATP6V0C SLC38A9 TIMM17B NDUFS8 CATSPERG DNAJC3 MARCH6 ATP6V1E1 SPTLC1 C22orf32 BNIP1 SLC18A3 ABCG5 FCER1G SCNN1B CHRNA7 FAF2 HTR3A SEC24B NDUFS4 TIMM50 RNF139 GABBR1 GJC3 CACNA1B SHISA9 CNTNAP2 CHCHD3 PIK3R6 NDUFA3 KCNQ1 BIRC2 FKRP GNG7 ATP6V0D1 HCN2 ATP6V1B1 RRAGC KCNA3 SNX1 DAB2 TLR2 NAPB GJB3 TRAF5 DMD CLIC6 GLRA3 KCNG4 BAIAP2L2 ATP5L2 ATP6V1H PAM16 ACVR1 RPN1 GNG2 APC2 KCNQ2 CACNA1C TRIL GNAT1 ATP6V0A1 GABRB2 ITGB6 VPS37C GABRD C18orf55 GABRA1 NDUFB7 NOX4 SCN3A CD8B SEC22B CLCNKB CATSPER4 CHRNE KCNE3 SESTD1 STOML2 KCNN1 AP4E1 SCNN1G CARD11 LTV1 ITGB8 UTRN ATG14 GRIA2 CACNG2 NDUFA2 DIABLO INSR SRP9 ATP5C1 ARL6 TIMM9 NDUFS2 KCNJ6 LY96 RGS11 ITGA4 NOX3 ATP6V1C1 PLN RYR1 SDHD DLG3 COPB1 GABRG3 ANO1 CD14 HBXIP IRAK1 VPS11 IRS1 STX8 COX7B2 CDH1 SYNRG SPG7 DLG2 MAGEE1 NAPG SNAP23 C7orf59 CD79A SLC26A6 IL28RA VTI1B ATP5J2 KCNT2 DERL1 PIK3R2 GNA14 STX7 KCNIP3 VPS37A KCNK2 NDUFC2 UQCRB KCNMB3 AP1M2 TMED10 NCF1B KCNJ14 AP1S1 TMED7 PVRL1 MTOR ABHD6 CALM3 TSPAN32 GRIK2 ITGA6 CHMP2B CLCN1 KCNA7 CHMP3 SHISA6 ATP12A STXBP2 NDUFB1 SDHB SEC31B KCNA6 HLA-DQA2 CACNA2D1 NDUFS6 ITGA7 KCNG1 CNGB3 RNF31 STX3 ITGB2 CHMP7 AP1S2 SELS ARRB1 ATP1B3 IL6R TMEM146 VTN IGF1 KCNK1 VPS33B KCNH4 KCNIP2 ORMDL1 IMMP1L CLTCL1 PIK3CG ATP5I CACNG8 VPS41 KCNQ5 SNTG1 SHC1 GNAT2 NECAP1 HSPA2 SHANK2 C5 VPS18 VAMP3 C3orf58 CHRND CTNNA1 CLIC1 DPP6 ATP6V1F OLFM3 GNB1 TGFBR1 NCALD TRAF2 MUDENG UVRAG C15orf24 CACNG5 ANKZF1 KCNH2 PIGT ATP5D C8G NDUFB10 AMIGO1 NCF4 ERN1 NDUFA7 KCNC2 FAS MICU1 ATP1B1 DKFZp761E198 ATP5G1 NDUFA12 VCP FADD CACNA2D3 OSTC BCS1L KCNS2 FKBP1B GM2A COX5B CATSPER3 KCNG2 SCN5A GJB1 CHMP4A KCNMA1 CNTNAP1 VDAC2 TRPC4 GPAA1 HERPUD1 ATP5L GJD4 STXBP5 TLR1 SNTB1 RP9 MMGT1 SEC31A ARCN1 NDUFA13 SDCCAG3 GJB5 GABRQ HFE2 IL12RB1 SGCE STX17 NOS1AP TOMM70A ATP6V0E2 COPG2 RYR3 KCND3 CACNA1S HTR3B OSMR SCN1B ATP6V0B SNAP47 STX12 ATP6V0D2 TAP2 KCNC4 COX6A2 GRPEL2 AP3S1 IL10RB SEC22A VAMP8 FXYD1 SHANK1 CHMP1B PPIF PIK3C2A GNAI3 TAPBP PANK1 STXBP5L VPS36 PIK3R3 CD79B SEC24A RRAGA NDUFA1 TGFBR3 GRIN2C KCNQ4 RIPK1 ITGAE TOMM40 GRIK4 GABRA6 SDCBP CHRNG VPS28 TIMM23 TMEM85 DNM1 CHRNB1 CHRNA10 GLRB HLA-B IL29 GNAI2 MAP3K5 ARL6IP1 RNF185 KCNA10 CACNG6 HTR3E STX6 KCND1 GABRG2 CD4 CLCA3P GLRA2 CTTN SPTSSA GJB4 SCFD1 HLA-DQB1 NCF1 CYBA GJA5 CORO1C COX6A1 FAM125A NDUFB8 ATP5O HTR3C HLA-DMA KCNE1 BET1 CHRNA6 CNIH2 HLA-DQA1 NDUFB4 COX4NB GGA1 CALM2 AP2A2 CD3G FXYD2 HOOK2 VAMP7 C15orf48 GABRR2 TOMM20 ATP6V1G3 RPN2 CD6 SCLT1 NDUFV2 GRIK5 ITGA1 TFRC STX16 STT3A ACVR1B COX8C GNAI1 CD8A SNTA1 KCNJ8 CTNNB1 VPS33A CASP8 CHRNA5 NDUFA8 TFR2 DAD1 ITGB7 PIK3C2G SYT1 CACNB4 CHRNA3 KCNIP4 SNF8 ATP2A1 GNGT1 CTDNEP1 CHRNA1 ATP6V1A AP3M1 ATP5G3 KCNJ11 GJB2 NEO1 C14orf2 NDUFAB1 ITGA11 RNF5 ABCB6 LIN7C SSPN GJA4 GNG13 SEC23A C1orf101 GABRA2 NLGN1 COPA HLA-C NOX1 SNX3 KCNB2 HLA-DQB2 SCN9A CLCNKA C8A ORMDL3 CACNA2D4 B2M FAM21C JUP GRIN3B CAV1 SORBS1 NDUFB5 GNG4 TRAT1 KCNJ16 FLOT2 LYN GRIN2D TMEM111 GABBR2 TIMM10 CHRNA4 VPS26A CFLAR SNX2 HLA-DRB4 KCNJ4 ITGAV GABRR3 CHUK NDUFS7 TRIP6 APBB1IP KCNF1 VAMP1 IGF2R GLRA4 FAF1 SNX8 SACM1L SPCS2 ITGA3 KCNC3 SNTB2 AP2M1 AP4M1 ORMDL2 COPZ1 VPS4A CACNA1H GNA13 SLC9A1 DENND5A SEC22C TTC35 M6PR TMEM188 CNGB1 ATP5G2 UQCR10 HSPD1 ITGA8 SPTLC2 GNAT3 GABRA4 EPS15 CSF2RB HTRA2 GNG8 NDUFC1 HFE ITGAL UBAP1 IL13RA1 RGS6 GABRB3 SCN3B CASQ2 ALCAM BAK1 CACNA1A ATP5S EXT2 DAG1 PSENEN C6 KCND2 VWC2 STT3B KCNMB1 HLA-DRB3 CASP10 ABCG8 KCNJ3 BEST3 RRAGD GRPEL1 CFTR IL6ST AP2A1 ATP4B HOOK3 AP1S3 CLIC4 SCN2B ATP5E ITGB3 GNG11 ITGA9 CD74 SCNN1A UBAP1L MAGT1 BEST2 IMMT CCDC109B C9 SCYL1 PIK3CB NUP62 VPS37D AP3M2 GNGT2 CATSPER1 VWC2L SRPRB PIK3C2B SNX27 MAGEL2 HLA-G COPE PINK1 PPP2R4 SREBF2 ATP5B KCNJ2 SAMM50 MR1 NDUFV1 TRADD CBL GJB7 VPS39 ATP5A1 ATP6V1G1 PIK3R4 BEST1 TNK2 GJA8 ACVR1C C20orf29 UGT3A1 VPS25 TOMM20L TMEM93 GJD3 SCN4B NDUFS5 SNAP29 HLA-H CALR GRIA1 GNG12 SEC24C SCN1A NAPA EPS15L1 VDAC3 FXYD3 MCU CHMP1A NPNT PIGS PARK7 SNAP25 KCNN4 CLIC3 SGCG CACNA1D GRIN1 IL6 AKAP9 NDUFA9 ABCC9 ATP6V1E2 NECAP2 FAM158A GABRB1 CEACAM1 GJC2 OLFM2 PDE4D SLC25A6 GJA1 GJA9 CNTN2 ZACN ATP6V1B2 C8B SCAMP5 TTYH3 EPN2 CHMP4C KCNA1 KCNU1 GABRP ATP6V1G2 INHA CACNG3 GGA2 SPCS1 RYR2 CD3D CLTB ADAM8 LAMTOR2 CHRNA9 GNA15 TAP1 GJD2 ABCD4 GNG5 CNTFR KCNS3 SPTSSB VPS37B UQCRC1 CLCN2 COX8A CPLX2 TMED3 SEL1L KRTCAP2 CACNB1 NDUFB3 BCL2 SGCA TOMM6 SYVN1 ABCB8 CLCC1 TLR4 GRB2 COX7B APOO AMFR KHDRBS1 PIK3R1 AKAP6 SNCA RGS7 ATP6V0A2 BMPR1B PLDN CACNB2 SDHC ABCA7 ATP1A3 NOXO1 BAX KCNK4 AFG3L2 HLA-DPB1 CHMP4B PSEN1 CPLX1 VAMP4 AP1G2 HOOK1 LRRC26 GJA10 KCNJ5 SCN2A ATP6V1C2 GABRE RGS9 IGF1R HLA-DRA KCNMB2 VPS35 LAMTOR1 HLA-DOB GJC1 GNAZ EPS8 CD34 KCNC1 ATP1B2 GOSR2 ATPIF1 KCNA5 NPLOC4 ITGA2 AKTIP ATP5EP2 LRP6 HLA-A ITGAX GNAO1 RRAGB EGFR LAMTOR3 CACNA1I SHISA8 DCTN1 CD3E PMPCB IKBKB TTYH2 GNA12 SNX6 ITGA2B AP3B2 LOC100652748 WNT3A C19orf63 ATP5F1 ACVR2A SCN7A IL23R NDUFA10 NDUFS1 KCNT1

GO_SODIUM_POTASSIUM_EXCHANGING_ATPASE_COMPLEX Sodium:potassium-exchanging ATPases are tetrameric proteins, consisting of two large alpha subunits and two smaller beta subunits. The alpha subunits bear the active site and penetrate the membrane, while the beta subunits carry oligosaccharide groups and face the cell exterior. ATP1A4 ATP1B3 FXYD2 ATP1B4 ATP1A3 ATP1A1 ATP1A2 ATP1B1 ATP4B FXYD1 ATP1B2

GO_PROTEASOME_REGULATORY_PARTICLE_BASE_SUBCOMPLEX The subcomplex of the proteasome regulatory particle that directly associates with the proteasome core complex. PSMD5 PSMC4 PSMC5 PSMC3 PSMC1 PSMD4 PSMC6 PSMD9 PSMC2 PSMD10 PSMD1 PSMD2

GO_PROTON_TRANSPORTING_ATP_SYNTHASE_COMPLEX_COUPLING_FACTOR_F_O_ All non-F1 subunits of a hydrogen-transporting ATP synthase, including integral and peripheral membrane proteins. ATP5I ATP5G3 ATP5L2 ATP5S ATP5L ATP5J2 ATP5H ATP5J ATP5F1 ATP5G2 ATP5G1

GO_EXTRINSIC_COMPONENT_OF_PLASMA_MEMBRANE The component of a plasma membrane consisting of gene products and protein complexes that are loosely bound to one of its surfaces, but not integrated into the hydrophobic region. GNAO1 KCNIP1 JAK3 TDGF1 MFGE8 RS1 EPN3 USP8 ATP2A2 BLK SRMS CYTH3 SRC RGS11 GNG12 KCNAB1 GNA13 GNG10 SNX18 TIAM1 TF S100A6 ITK YES1 CDH1 GNA12 ALOX15 RGS8 FRK ZAP70 JUP CNR2 EEA1 SYTL1 LCK GNAI2 GNAT1 RGS7 GNGT2 APC2 CTNNA1 FMR1 JAK1 ESYT3 TNK2 PLG RGS9 SCUBE1 GNAZ MYZAP GNB1 TXK S100A10 GNG4 FGR STOML2 AAK1 RACGAP1 TEC LYN STYK1 ESYT2 CDK16 SNX9 PRSS22 ST14 KRAS CAV2 RGS1 NLRP10 FER RLTPR SERPINE2 ARRB1 RGS19 ABL1 GNG7 RHOA ANXA2 FERMT2 ANXA1 APC PCSK9 NUMB HIST1H2BA GNG5 PRSS41 GNGT1 ERRFI1 FYN GNAI3 GNAT2 PTK2B TGM3 IQGAP1 CDH2 GNG2 BMX GNG11 PTK6 GNG13 CYLD JAK2 GNG3 GNAI1 SMAD7 FARP1 CSK PTK2 ABL2 PVRL1 SYK RGS6 CTNNB1 CYTH1 SYTL2 MATK TYK2 GNA14 CUBN FES GNAT3 GNG8 PRSS8 SNX5 RAC1 DLG4 GNA15 KCNAB2 BTK DTNA SYTL3 DNAJA3 TNK1 FCN1 HCK

GO_NEUROTRANSMITTER_RECEPTOR_COMPLEX NA SHISA6 CACNG2 GRIA2 PTK2B NLGN1 DLG4 VWC2 GRIN2A SHANK2 GRIA4 CACNG4 GRIN1 GRIN2C GRIN3A DLG1 GRIN2B CACNG5 GRIK2 CACNG3 PORCN GRIK4 CACNG8 GRIA3 DLG2 GRIN2D SHISA9 GRIN3B OLFM3 DLG3 CNIH3 SACM1L CNIH2 ABHD6 SHISA7 SHISA8 EPS8 VWC2L CACNG7 SHANK1 GRIK5 GRID2 OLFM2 CPT1C ABHD12 GRIA1

GO_SIN3_TYPE_COMPLEX Any of a number of evolutionarily conserved histone deacetylase complexes (HDACs) containing a core consisting of a paired amphipathic helix motif protein (e.g. Sin3p in S. cerevisiae, Pst1 in S. pombe or Sin3A in mammals) at least one class I histone deacetylase (e.g. Rpd3p in S. cerevisiae, Clr6 in S. pombe, or HDAC1 and HDAC2 in mammals), and at least one WD40 repeat protein (e.g. Ume1p in S. cerevisiae, Prw1 in S. pombe, or RbAp46 and RbAp48 in mammals). These complexes also contain a variable number of other proteins that direct histone binding, DNA binding, or add other functionality to the complex. SUDS3 FAM60A BRMS1 PHF12 CSNK2A1 ING2 SAP130 HEY2 NCOR1 HDAC1 HDAC2 BRMS1L MORF4L1 SIN3A RBBP4 SIN3B

GO_GOLGI_ASSOCIATED_VESICLE Any vesicle associated with the Golgi complex and involved in mediating transport within the Golgi or between the Golgi and other parts of the cell. COPA COPZ2 VPS41 CNGA2 TYR CNGB1 PI4KA ADAM10 PKD1 CLTCL1 ZDHHC13 RASSF9 SPG21 GPR89A ATP7A STK16 RHO AP3B2 AFTPH SYNRG COPZ1 SORT1 GNRH1 CCDC115 IGF2R PACS1 CLTA AP2A1 FURIN TMED3 COPB1 SLC2A4 BACE1 ITM2B CSPG5 ARF1 GOPC SLC18A3 NRGN RAB8A OCRL CHIC2 CFTR COPG TGOLN2 CLTB TMED9 HM13 KDELR1 LDLRAP1 IMP5 MAP6D1 NCALD COPG2 SPPL2B SPPL3 SCFD1 TMEM199 RAB14 COPE TMED7 AP1S1 CNGA4 RAB27B TMED2 AP1G2 PACSIN1 SPPL2A RHOQ STEAP2 C3orf58 LRRK2 COPB2 MST4 CLTC SCYL1 ACR GJA1 ARCN1 TMED10 ZDHHC17 NUCB1

GO_RIBONUCLEOPROTEIN_GRANULE A non-membranous macromolecular complex containing proteins and translationally silenced mRNAs. RNA granules contain proteins that control the localization, stability, and translation of their RNA cargo. Different types of RNA granules (RGs) exist, depending on the cell type and cellular conditions. L1RE1 DDX4 INA RPL6 LSM6 FAM195B PUM2 TIAL1 YTHDF2 TNRC6B DYNC1I1 POLR2G CLOCK RPLP0 TDRD7 TIA1 EIF2C1 MAEL LSM1 CDC42 PIWIL2 PSMA6 FMR1 APOBEC3D PQBP1 IGF2BP1 EIF2C4 GRB7 ACTB EIF4E GRSF1 DHX9 PABPC4 TOP1 TRIM71 RPL28 KHSRP APOBEC3H YBX1 MOV10L1 EIF2C2 DCP1B NANOS2 PSMA4 SAMD4A DDX3X DIS3L2 PABPC1 LIMD1 ZC3H12A PAN3 PNRC2 CNOT1 DCPS EDC3 MAPT PATL1 RQCD1 EIF2C3 DDX28 CNOT3 PIWIL1 POLR2D TRIM5 CAPRIN1 TDRD9 RC3H2 SNRPG LSM4 BTBD6 DDX1 RC3H1 TUBB ATXN2L PUM1 RAC1 CARHSP1 TRIM21 MEX3A OGFOD1 FASTKD5 NUFIP2 RPS6 ZC3H12D HNRNPL RBPMS CNOT7 FXR1 CNOT2 FASTKD2 CIRBP HNRNPA3 PATL2 MOV10 BTBD1 MEX3B EIF2S1 ZFP36 TNRC6A TDRKH PSMC3 LARP4B APOBEC3F EIF4ENIF1 DCP1A DHX30 TDRD1 ARNTL WTIP CPEB1 BTBD2 FAM195A DDX3Y UHMK1 ATXN2 PSMC2 LSM14A PAN2 HENMT1 IQGAP1 PIWIL4 SMN1 TDRD5 HNRNPU DCP2 NCL APOBEC3G ASZ1 TDRD6 SAMD4B SQSTM1 DDX25 NANOS3 ROCK2 RPS4X UPF1 DDX6 MBNL1 LSM2 CCRN4L TUBA1A PSMA2 ZFP36L1 LIN28A SMN2 EDC4 EXD1 NSUN2 RBM4 LSM3 G3BP1 MYH1 JUB STAU1

GO_GLIAL_CELL_PROJECTION A prolongation or process extending from a glial cell. SLC17A8 MT3 EIF2S1 FMR1 SIRT2 GFAP AKR1B1 PINK1 GPR56 KCNK2 EZR SCN7A APP NFASC GLUL

GO_ORGANELLE_SUBCOMPARTMENT A compartment that consists of a lumen and an enclosing membrane, and is part of an organelle. KLHL20 TMEM110 MARCH9 ATL1 CHPF2 GALNT3 GOLPH3L CHSY3 CANT1 RAB13 REEP1 VPS54 REEP5 COG6 BICD1 MGAT2 DNMBP RAB10 C1orf88 APP OPTN SMPD3 NSFL1C YIPF6 GOLIM4 PRKD1 APH1A RAB30 SULF2 LAP3 RGS20 PSENEN GOLGA8IP ARSE YIPF5 B3GALT6 KIAA1324 ARFRP1 RAB18 GOLGA8A NBEA CSGALNACT2 LGR5 AP1S3 AP4S1 B4GALT5 RASIP1 ST3GAL3 COG4 MLANA FUT3 ST3GAL1 TMEM165 RAB34 MICALL1 STX10 HLA-DRB3 FUT8 VCPIP1 MYO18A FUT6 RBFOX1 LPCAT2 MBTPS1 ST3GAL2 PLK3 C11orf2 CD74 GOLPH3 B4GALT4 SCAMP1 PION B4GALT2 FUT2 STX4 TMED2 SYS1 SLC24A5 STX6 RAB38 ATP9B RAB27B BECN1 SCOC FAM109A ARFGEF2 NUCB1 GALNT1 SAR1B GGA3 CALN1 LRRK2 TGFBI COG7 ZFYVE27 C17orf28 FNBP1 TAS2R16 CHSY1 CLIP3 NAGPA ASAP2 SOD3 B4GALT7 NCK1 TGOLN2 ATP2C1 SCFD1 RAB7B PLEKHA8 HLA-DQB1 FUT1 AP4E1 VAMP2 RAB7L1 CHST2 NSF HLA-DRB5 TRIP10 HLA-DQA1 SORT1 BET1 RYR1 RABEPK VPS53 OCRL B4GALT1 RAB11A COG2 CABP7 RAB31 LLGL1 TOM1L1 NCK2 SGMS1 BIRC6 ASPH SNAP25 VAMP7 KIF13A FAM109B A3GALT2P ATL3 CDH1 PARP16 STX8 CASQ1 USP6NL RAB43 STIM1 SCAMP2 CHAC1 SCAMP5 ZFYVE1 GCNT1 AP1S1 SULF1 STX16 WIPI1 AP2B1 GOLGA2 AP1M2 ATXN2 AKAP9 COG8 GOLGA4 HMP19 WLS CNST DNM2 RAC1 CLTB SYT17 MOB4 PCSK1N CHID1 MMP24 CA4 TMEM79 GGA2 AP1G1 CLASP2 RHOBTB3 ST3GAL4 CLN3 SLC9A7 TMED3 B4GALT3 VPS52 AP1S2 TMBIM4 B4GALNT3 ARL1 ST6GAL1 ARFIP1 USO1 GOLGA7 MARCH4 INPP5K AMPH GPER TMEM59 COG1 CLVS1 HLA-DQA2 ARV1 CSGALNACT1 BACE1 BCAP31 GOLGA5 GOLGA8B GOSR1 UXS1 FUT10 FUT4 CHPF GAL3ST2 POSTN RTN2 KPNB1 FUT11 RAB9A FKBP1A ARFIP2 ATP9A KIAA1432 CLTCL1 ATP7A VAMP4 B4GALNT4 AP1M1 CPD TRAPPC9 COG5 CHST4 HLA-DPB1 SMPD4 RAB14 DPY30 CBY1 TJAP1 KIAA0368 GBF1 ARFGEF1 TMEM115 CLTC VAMP3 HLA-DQB2 FUT7 RAB6A MARCH1 HLA-DRB4 ATG9A GAL3ST3 HLA-DRA ATP8A2 SNX9 FUT5 GALNT2 TRAPPC4 HACE1 HLA-DPA1 RAB3GAP1 AP4B1 AP1B1 CLVS2 ABO ST6GAL2 HLA-DRB1 CORO7 FNBP1L GOLGA1 GOLGB1 VRK1 VTI1A PAM C20orf30 IGF2R MYO1B CLTA FURIN RAB32 ADC FUT9 NMNAT2 ATP7B ARAP1 SORL1 INPP5E B4GALT6 ARF1 GAL3ST4 M6PR LGR6 PITPNM1 DENND5A RGP1 RAB21 GOLGA3 ATP8A1 COG3 KIAA1715 GCC2 AP4M1 NECAB3 CIT GPR89A GGTA1P

GO_LOW_DENSITY_LIPOPROTEIN_PARTICLE A lipoprotein particle, rich in cholesterol esters and low in triglycerides that is typically composed of APOB100 and APOE and has a density of 1.02-1.06 g/ml and a diameter of between 20-25 nm. LDL particles are formed from VLDL particles (via IDL) by the loss of triglyceride and gain of cholesterol ester. They transport endogenous cholesterol (and to some extent triglycerides) from peripheral tissues back to the liver. SORL1 APOB LDLR SELS APOE MSR1 LSR APOBR APOC2 APOF PLA2G7 APOM APOO APOA5

GO_SIN3_COMPLEX A multiprotein complex that functions broadly in eukaryotic organisms as a transcriptional repressor of protein-coding genes, through the gene-specific deacetylation of histones. Amongst its subunits, the Sin3 complex contains Sin3-like proteins, and a number of core proteins that are shared with the NuRD complex (including histone deacetylases and histone binding proteins). The Sin3 complex does not directly bind DNA itself, but is targeted to specific genes through protein-protein interactions with DNA-binding proteins. ING2 SIN3A HEY2 RBBP4 NCOR1 HDAC1 SIN3B MORF4L1 PHF12 CSNK2A1 FAM60A HDAC2 SUDS3

GO_U2_TYPE_SPLICEOSOMAL_COMPLEX Any spliceosomal complex that forms during the splicing of a messenger RNA primary transcript to excise an intron that has canonical consensus sequences near the 5' and 3' ends. SF3A1 DHX15 RNF113A PRPF40A SNRPN LSM7 PRPF31 PRPF18 LUC7L SNRPG SNRPD3 SNRPC C2orf3 LUC7L2 SNRPB SF3A2 CWC22 U2AF2 SF3B1 SNRNP70 RBM22 PRPF40B SF3B2 TFIP11 SNRPD1 SF3B14 SNRNP35 HTATSF1 LUC7L3 PRPF39 RNF113B

GO_SYNAPTIC_MEMBRANE A specialized area of membrane on either the presynaptic or the postsynaptic side of a synapse, the junction between a nerve fiber of one neuron and another neuron or muscle fiber or glial cell. GABRR3 LRFN2 KCNJ4 COMT SIGMAR1 LZTS1 GABRE CHRNA4 LRFN3 ZNRF2 FAIM2 SRCIN1 PDLIM5 GRIN2D GABBR2 CHRNB4 FLRT3 CACNG5 NLGN2 GRIN3B FOSL1 PCDH8 EPHA7 ABI1 NETO1 LRRTM4 GLRA1 KCTD16 CAMK2A CHRND ADORA1 ANK3 PSD3 EPHA4 GPHN CNKSR2 DISC1 ERC2 TRPV1 CLSTN1 ITGA3 D4S234E NPTN GLRA4 NELF OPRD1 DDN GRID2 PICK1 KCNC2 NTRK2 IGSF9B ARF1 NDUFS7 ANKS1B KLHL17 SHC4 UNC13A ARRB2 CNTNAP4 CHRNA9 CHRNB2 DNM2 ProSAPiP1 DRP2 CABP1 FBXO45 GRIA3 SRGAP2 DTNBP1 GRIK2 CHRNA5 ODZ2 RIMS1 LRRTM1 GABRP CHRM2 CNIH3 LIN7A KCNA1 PVRL1 KCTD8 CPEB1 GRIK5 P2RX1 GRIK3 OLFM2 ITGB1 SCRIB GABRG1 GABRB1 AKAP9 GRIN2A SHANK2 ATP2B1 GABRA2 GRM3 NLGN1 SIPA1L1 DGKI NLGN4Y CACNG8 SYNJ1 PICALM SSPN LIN7C HOMER1 STRN CHRM1 CHRM5 ARRB1 TMUB1 CADPS2 ARC SYP BCR RIMS2 STX3 GRM6 RAPSN CHRNA1 HOMER3 GPER CHRNA2 CHRNA3 GOPC RIMS4 SYT1 ERC1 SEMA4F GAD2 GRIA2 UTRN GABRR1 LRRC4 GRIK1 CAMK2N1 GRID2IP MPDZ SYNE1 CBLN1 DLGAP2 GRIN2B GRIN3A GLRA2 KCNB1 GABRG2 ANK2 CHRNE DENND1A MTMR2 IL31RA SYNPO GABRD CDK5 PI4K2A GABRA1 GABRB2 GABRA5 FMR1 NRXN1 ZDHHC17 PDE2A HOMER2 LRP4 UNC13C GRIN1 MINK1 PRR7 GABRR2 LPHN1 SNAP25 NIPSNAP1 GRIP1 DLG2 MAGEE1 NCS1 GRM8 ARHGAP32 KCNA2 CLSTN2 GRM2 GABRG3 CNIH2 CHRNA6 DLG3 IL1RAPL1 ATAD1 KCNH1 COL13A1 RGS14 CHRM4 LRRC7 ANK1 GRIA1 CHRM3 GRASP F2R C1orf70 LRRTM3 GRM7 KCND2 DLG4 DAG1 HTR3B DNAJA3 DLGAP3 DLGAP1 APBB1 GRID1 PVRL3 DLG1 CASK IQSEC3 CLSTN3 GABRB3 LIN7B CPE NLGN4X GRM4 GABRQ LRFN1 GABRA4 ITGA8 GABRA3 CHRNB3 PTEN GLRB GRIA4 SNPH CHRNA10 GLRA3 LRRTM2 SYT11 CHRNB1 DMD CHRNG LRRC4C GRIK4 GABRA6 SYT7 PALM PJA2 GRIN2C SH2D5 NEURL SYNDIG1 TANC1 SRPX2 GABBR1 ZC4H2 SHISA9 SEMA4C SHANK3 RUSC1 CRYAB SNCAIP MUSK KCTD12 PRKCG LRRC4B HTR3A SHANK1 P2RY1 CHRNA7

GO_TRANS_GOLGI_NETWORK_TRANSPORT_VESICLE A vesicle that mediates transport between the trans-Golgi network and other parts of the cell. AFTPH TMED10 SYNRG CLTCL1 SLC2A4 STEAP2 RASSF9 NCALD GOPC SLC18A3 SPG21 RAB8A NRGN CLTC ATP7A AP1G2 LDLRAP1 RAB14 SORT1 AP1S1 IGF2R CLTB RAB27B TMED9 TGOLN2 FURIN AP2A1 CLTA

GO_STEREOCILIUM_BUNDLE A bundle of cross-linked stereocilia, arranged around a kinocilium on the apical surface of a sensory hair cell (e.g. a neuromast, auditory or vestibular hair cell). Stereocilium bundles act as mechanosensory organelles by responding to fluid motion or fluid pressure changes. TWF2 BBS2 GPR98 DCDC2 SLC9A3R1 KPTN PDZD7 MYO7A MKKS IDO1 TRPA1 TSPEAR TMC1 KNCN MYO15A FAM65B SLC4A7 IFT20 CEACAM16 CIB2 STRC HOMER2 CLIC5 EPS8 MYO1C FSCN2 GRXCR1 ESPN CDH23 MPP1 ATP8B1 GRXCR2 DFNB31 USH2A USH1C TPRN STRCP1 RDX PCDH15 ELMOD3 LOXHD1 DOCK4 TMC2 LHFPL5

GO_SMALL_NUCLEOLAR_RIBONUCLEOPROTEIN_COMPLEX A ribonucleoprotein complex that contains an RNA molecule of the small nucleolar RNA (snoRNA) family and associated proteins. Most are involved in a step of processing of rRNA: cleavage, 2'-O-methylation, or pseudouridylation. The majority, though not all, fall into one of two classes, box C/D type or box H/ACA type. DKC1 GAR1 FBL NOP10 POP4 FBLL1 NAF1 LSM7 NHP2 RRP9 SNRPG LOC100130932 NHP2L1 SNRNP40 NOP56 SNRPF POP1 MPHOSPH10 LSM6 NOP58

GO_PRIMARY_LYSOSOME A lysosome before it has fused with a vesicle or vacuole. STX3 PRSS57 VAMP7 STXBP2 VAMP1 HEXA HEXB DEFA4 OLFM4 ANXA11 STX7 DEFA1B DEFA3 DEFA1 AZU1 VAMP8 MPO SNAP23

GO_APICAL_DENDRITE A dendrite that emerges near the apical pole of a neuron. In bipolar neurons, apical dendrites are located on the opposite side of the soma from the axon. FLNA MYO1D YKT6 CLU OSBP2 SLC17A8 CPEB3 SLC4A10 NEURL NELF PTK2B PPARGC1A SEZ6

GO_PRONUCLEUS The nucleus of either the ovum or the spermatozoon following fertilization. Thus, in the fertilized ovum, there are two pronuclei, one originating from the ovum, the other from the spermatozoon that brought about fertilization; they approach each other, but do not fuse until just before the first cleavage, when each pronucleus loses its membrane to release its contents. EZH2 SLC2A1 HNRNPL EED HSF1 TET3 AKAP8 CENPF RIF1 DPPA3 TBP CCNA2 CBX1 AURKA

GO_EXORIBONUCLEASE_COMPLEX NA EXOSC7 EXOSC6 CARHSP1 EXOSC9 DIS3 SKIV2L2 EXOSC2 AICDA EXOSC5 EXOSC3 MPHOSPH6 KHSRP ZFP36 EXOSC4 EXOSC1 DIS3L2 PNPT1 EXOSC8 DIS3L C1D EXOSC10 SUPV3L1 GTPBP1

GO_CYTOPLASMIC_REGION Any (proper) part of the cytoplasm of a single cell of sufficient size to still be considered cytoplasm\ KIF1A MAPRE1 PTK2 FBLIM1 WIPF3 BLOC1S3 CTNNB1 BSN ACTN2 SHROOM4 UHMK1 MAEA GRIK3 RALB WLS CABP1 USP2 MOBP DBNL RANGAP1 MARCKS NDEL1 GABARAPL1 GRIK2 DTNBP1 OR2C1 EPB41 AGTRAP HPCA BIN2 PLEKHH2 PXN CLASP2 AP3M1 ADD3 FER KRT19 POTEF OPRM1 PDZD4 RGS19 MYO9B ARV1 SEPT7 NOS2 ACTN4 CLIC5 FERMT2 CORO1A PLA2G4C FLOT1 MTSS1L MYO1A HIF1A FRY RASAL3 ERMN HAMP GIPC1 SPIRE1 DST ANLN NEDD4 LAMC2 ARFIP2 BFSP2 SEPT9 SEPT6 WIPF2 AXIN2 PLS1 AKR1B1 KIF3B GLRX3 C19orf21 CAV1 MAPK1 PFN1 WASL PHLDB2 SNCA EXOC2 AP3D1 SPTBN2 EXOC3L2 PQBP1 INSC MYO5B PLDN TMOD1 RAPGEF3 BLOC1S2 PFN4 CTTNBP2 CTGF TRAF6 FGA FGG MPP1 ITPR2 WDR81 FLOT2 LANCL2 SEPT12 TRAF2 UCHL1 SPTBN4 SLC4A1 CD302 C1orf172 LLGL2 WASH1 TCHP NELF FNBP1L HMCN1 NEFL PARD3 STOX1 DSTN MKLN1 KLHL17 WDPCP GSN RIC8B EPS8 TNFAIP2 PKD2 KNCN EXOC6 BFSP1 MYO6 MLPH TRPC4 RAB21 ARHGEF7 SHROOM2 FGFR2 NLRP5 NRBP1 STXBP6 RAI14 CDKL5 NF2 CNKSR1 AP3B2 CNO EPB49 SEPT2 GM2A HFE AP3S2 GRK4 SLC2A1 TMEM110 RAB10 DBN1 SPTBN5 WDR43 KIAA0528 MYH2 GYS2 SNAPIN BLOC1S1 EXOC4 AP3B1 FLNA NEDD9 DLG4 MELK CLDN5 FMNL1 MYADM PRKD1 EPB42 RGS20 GRM7 CAPZA3 SPTAN1 FLNB CYTIP SPIRE2 SCIN EXOC5 WIPF1 PDLIM2 TSC1 SPRR4 OPA1 LASP1 TLE6 PARD6B EXOC1 CLASP1 FGB EPB41L2 PHLDB1 PARD6A SEPT5 ASTN2 EXOC8 RHOA PPP1R9A ANXA2 AP3S1 FMN2 FABP1 DLC1 TPM4 PTK2B NCL MYH10 SPAST PAFAH1B1 SPTA1 AKT2 CDH2 SPTBN1 SPTB CAP2 FGF1 ARF6 MAP2K4 MYO10 EXOC3L4 KIF1B AKAP13 COL10A1 PRKCZ SOD1 C4orf49 EXOC3L1 LRRK2 AXIN1 AP3M2 HAP1 ACTB FAM110C GYPC MED28 FMR1 TRPV4 OOEP UTRN CAPZA2 KIF4A FNBP1 EZR MYZAP CTTN EXOC3 PCLO COBL CAPN2 EEF1A1 MYO7A RYR1 TRIP10 ACTR2 LLGL1 MUTED PSEN2 AKAP12 CALD1 KCNAB1 SELE NDFIP1 HMCN2 CAP1 EXOC7 CANX MYH9 ASPH MYRIP NUMA1 STIM1 CRIP2 ADA RDX EXOC6B FRYL SPINK5 GPSM2 CDH1 ARHGAP32 SPG7

GO_COP9_SIGNALOSOME A protein complex that catalyzes the deneddylation of proteins, including the cullin component of SCF ubiquitin E3 ligase; deneddylation increases the activity of cullin family ubiquitin ligases. The signalosome is involved in many regulatory process, including some which control development, in many species; also regulates photomorphogenesis in plants; in many species its subunits are highly similar to those of the proteasome. NOD2 AMOTL1 EPB41L2 HSPA5 DOCK7 COPS7B PLCG1 COPS7A COPS5 COPS8 TMOD1 FLOT1 THEMIS HSPA1L COPS4 WDR6 AMOT BASP1 NCKIPSD GPS1 GRB2 MYH9 LAT DYNLL1 COPS6 COPS2 COPS3 STOML2 VPRBP HSPA6 HSP90AB1 KIAA0748 HSPA7 ATP5A1

GO_BASOLATERAL_PLASMA_MEMBRANE The region of the plasma membrane that includes the basal end and sides of the cell. Often used in reference to animal polarized epithelial membranes, where the basal membrane is the part attached to the extracellular matrix, or in plant cells, where the basal membrane is defined with respect to the zygotic axis. RHBG TF MEGF11 SLC19A1 SLC12A6 CTNNA2 NOD1 LDLR SLC26A6 SLC4A11 TRPC4 C5AR1 SLC9A1 VANGL2 DLG2 FXYD2 ERBB3 CNNM2 CLDN19 SLC22A1 MEGF10 TACSTD2 SLC22A6 MSN C12orf53 BMPR2 GPIHBP1 ITGA3 AQP3 ADAM9 HSP90AA1 MPZ EGFR SLC38A3 EPCAM ENPP1 SLC7A8 DLG3 STK39 SLC22A7 FOLR1 ATP1B1 PKD2 ANK1 MET MYO1D SLC7A6 ATP7B KCNC2 CHRM3 NKD2 B4GALT1 SLCO1B3 MUC20 CEACAM5 CD34 TGFA SLCO4C1 ADRA2A SLC4A4 FRMPD2 ERBB2 KCNJ4 CDH17 CA9 AQP9 LEPR ABCC4 AJAP1 BEST1 ST14 BSND SLC4A1 FLOT2 CDH16 SLC29A1 ABCC1 KCNJ16 EZR P2RY6 STX4 ANK2 NAIP CNNM4 RAB17 DSP NOD2 SLC4A10 SLC41A1 AQP5 SLC2A9 RHCG SLC22A9 ADORA1 STXBP3 PROM2 CLDN7 ANK3 MYO1C CDH2 CA2 PKD1 DST ATP2B1 DSTYK ATP2B4 SLC22A8 CXADR SLC40A1 CD1D MAP7 ERBB4 HSP90AB1 ITGA9 LIN7C PALM KCNQ4 ATP7A SLC23A2 ATP6V1B1 ABCC6 ARRB1 IL6R KCNQ1 HPGD CD300LG TJP1 SLC39A5 MAP4K2 P2RY4 ATP1A1 SLC4A2 CADM1 AP2A1 CFTR CA11 ERBB2IP STX2 MYO1A FLOT1 SLC4A7 NUMB P2RY1 CLDN4 ANXA2 MARVELD2 PDGFB ANXA1 CLCA2 SLC10A1 SLC29A2 SLC26A5 LDLRAP1 ARRB2 SLC16A10 CLDN8 DLG4 AQP2 TLR9 UMOD DAG1 ATP12A CA4 KCNJ10 SLC26A7 OSCP1 SLC7A7 SLC27A5 SLC14A1 AQP1 TEK SLC9A4 ITGA6 OTOF DLG1 CASK HEPH SLC2A1 GPR77 S100G SHROOM4 LIN7A PTH1R CTNNB1 SLC23A1 SLC8A2 LIN7B MLC1 P2RY12 NDRG4 AQP4 TFRC EPS15 LPO SLCO1B1 CEACAM1 SCRIB

GO_VESICLE_COAT A membrane coat found on a coated vesicle. CLTA AP2A1 SEC24A COPE AP1S1 TMED7 TMED3 EPN2 COPB1 EGFR AP1G2 CLTC SCYL1 SLC18A3 COPB2 AP2S1 NECAP2 C3orf58 SEC24B VMA21 SEC31A AP2B1 EPS15 SEC24D ARCN1 SEC24C SEC23A COPZ2 CLTB COPG COPA NECAP1 SEC31B LDLRAP1 SGIP1 COPG2 TBC1D5 NCALD AP2M1 CLTCL1 SYNRG COPZ1 SEC23B AP2A2 AFTPH PICALM

GO_TOR_COMPLEX A protein complex that contains at least TOR (target of rapamycin) in complex with other signaling components. Mediates the phosphorylation and activation of downstream signaling components including PKB (AKT) or S6K. RPL23A PRR5L LARP1 MLST8 MTOR TELO2 RICTOR PINK1 AKT1S1 SESN2 PRR5 TTI1 MAPKAP1 RPTOR

GO_BRUSH_BORDER_MEMBRANE The portion of the plasma membrane surrounding the brush border. DRD5 SLC26A3 ITLN1 SHANK2 NPC1L1 ATP8B1 SLC26A6 SLC11A2 GNA13 SLC6A18 CA4 SLC22A5 CD36 SLC34A3 HSP90AB1 ATP6V0A4 MFSD10 PDZK1 SLC34A2 GNA12 CDHR2 AQP1 PEMT CYBRD1 KCNK1 LRP2 ATP7A SLC26A4 SLC17A3 SLC9A3 SLC46A1 HSP90AA1 TRPM6 SLC27A4 CDHR5 PTH1R SLC7A9 SLC9A3R1 SLC34A1 SLC6A19 TMEM27 FOLR1 SLC5A6 SLC22A12 PEX19 SLC6A14 SLC3A1 B4GALT1 CUBN SLC5A1

GO_ACROSOMAL_VESICLE A structure in the head of a spermatozoon that contains acid hydrolases, and is concerned with the breakdown of the outer membrane of the ovum during fertilization. It lies just beneath the plasma membrane and is derived from the lysosome. CAV2 TEX101 FAM170B TSSK2 ARC TCP1 TMEM190 SUN1 CYLC1 SPINK8 SPACA7 FABP9 NOTCH1 SV2B ZP3 CLK3 PLA1A RAB3A SPESP1 LOXL1 SPAG8 CALR SKIL CXADR IZUMO1 C9orf9 C9orf11 MORN2 TBC1D21 ADAM15 TSSK1B CD46 PPFIA3 ACRV1 SPACA4 SPAG9 CATSPER3 CRCP TSKS IQCF1 TEX22 SPINK13 HEXB ATP8B3 CATSPER4 IQUB AZI1 TRIM36 BSG STK31 DRD2 KIT ACRBP CAV1 CASC5 SYT8 VEZT ATP6V1E2 TBXA2R SPINK1 PATE4 ATP6V0A2 POMT1 ITGA1 GNAT3 CTNNA1 RND2 BMF ACR SERPINA5 SPACA3 ZPBP2 ABHD2 AKAP3 SPINK2 TCTEX1D4 TEKT3 TMEM225 LRGUK CAPZA3 NUDT1 PCSK4 CAPN11 SLIRP TRIP11 SPACA1 DLD FNDC3A FLOT2 NCF2 RACGAP1 TXNDC8 ENKUR

GO_ESCRT_III_COMPLEX An endosomal sorting complex required for transport. Consists of two soluble subcomplexes of highly charged coiled-coil proteins and is required for sorting and/or concentration of multivesicular body (MVB) cargoes. CHMP7 CHMP4A CHMP2B CHMP1B CHMP1A CHMP4B CHMP4C VPS4A CHMP2A CHMP6 CHMP3

GO_AXONAL_GROWTH_CONE The migrating motile tip of a growing nerve cell axon. TRPV2 KIF5B EPHA4 KIAA1598 PARD3 GPM6A OLFM1 TIAM1 MAPK8IP1 FLRT3 FKBP4 RTN4R L1CAM COBL PTCH1 BOC

GO_PCG_PROTEIN_COMPLEX A chromatin-associated multiprotein complex containing Polycomb Group proteins. In Drosophila, Polycomb group proteins are involved in the long-term maintenance of gene repression, and PcG protein complexes associate with Polycomb group response elements (PREs) in target genes to regulate higher-order chromatin structure. HDAC2 RING1 CBX8 SUZ12 JARID2 CBX4 SKP1 RBBP4 CSNK2A2 PHC3 H2AFY2 PHC1 RBBP7 EZH1 PCGF1 SCML2 PHF1 AEBP2 RYBP CSNK2B BAP1 CSNK2A1 YY1 CBX6 SIRT1 CBX2 EZH2 PCGF5 H2AFY CBX7 PCGF6 UBAP2L BCOR KDM2B BMI1 PCGF2 ASXL1 PCGF3 TRIM37 MTF2 PHC2 EED RNF2 PHF19

GO_DERLIN_1_RETROTRANSLOCATION_COMPLEX A protein complex that functions in the retrotranslocation step of ERAD (ER-associated protein degradation), and includes at its core Derlin-1 oligomers forming a retrotranslocation channel. VCP SVIP RNF139 RNF5 SELS SYVN1 SEL1L DERL1 HM13 AMFR RNF185

GO_LEADING_EDGE_MEMBRANE The portion of the plasma membrane surrounding the leading edge of a motile cell. MAPK8IP3 ANTXR1 ABCA7 DDX58 FGD2 PLEKHA1 PDPN APC2 ADORA1 CIB1 TLN1 GABRG2 CDC42 EPB41L3 TWF1 EEF1A1 FGR SLC1A2 EZR PDXP TIRAP ITGAV EPB41L5 TACR3 UNC5A TRPV4 THEM4 PTPRK KCNC2 MYO6 MYO1D PLEK2 DDN SYNE2 GRIA1 ANK1 SRC CSPG4 EPS8 ATF4 PPP1R9B KCNC1 PLEK RASGRP2 AIF1L HSP90AA1 DIAPH1 ARHGEF4 AIF1 ADAM17 OPRD1 PDE9A PTPRJ KCNH1 EPS8L1 PLEKHO1 C17orf87 TPM1 EPHA2 TRPV1 NF2 KCNA2 CDKL5 KANK1 TIAM1 ITGB1 NME1 NCKAP1 NRG1 MYO1G KIAA0528 ARF4 FERMT1 ITGA8 PAK1 SGCE PACSIN1 ROBO2 PIEZO1 FGD5 PDE4A CLASP2 HPCA ARHGAP44 GABARAPL1 IFIT5 WWC1 CFL1 SPRY4 PIP5K1C DNM2 RAC1 TESC WLS GPER AMPH INPP5K DLC1 RPS3 MACF1 RAB34 VASP APC FERMT2 EMR2 PLA2G4F C20orf103 CHRNA7 KCNJ11 LCP1 OPRM1 KSR1 SLC39A6 SHISA9 CNTNAP2 PACSIN2 SH3YL1 PIP5K1A SPATA13 SPRY2 ITGB3 ARHGEF2 DPP4 PALM SPTBN1 ATP2B1 ITGA5 AKT2 BMX MYO1C SNTG1 EPS8L2 FAP

GO_SEH1_ASSOCIATED_COMPLEX A protein complex that associates dynamically with the vacuolar membrane, and is proposed to have a role in membrane-associated trafficking or regulatory processes. In S. cerevisiae the complex contains Seh1p, Sec13p, Npr2p, Npr3p, Iml1p, Mtc5p, Rtc1p, and Sea4p. SESN3 NPRL2 WDR24 SEC13 DEPDC5 RRAGA SESN1 GATSL2 SEH1L WDR59 SESN2 NPRL3 GATSL3 MIOS

GO_MITOCHONDRIAL_RESPIRATORY_CHAIN_COMPLEX_IV A protein complex located in the mitochondrial inner membrane that forms part of the mitochondrial respiratory chain. Contains the 13 polypeptide subunits of cytochrome c oxidase, including cytochrome a and cytochrome a3. Catalyzes the oxidation of reduced cytochrome c by dioxygen (O2). COX4I1 COX6A2 COX5A COX8A C15orf48 COX8C NDUFA4L2 NDUFA4 COX6A1 COX4I2 COX5B

GO_RUFFLE_MEMBRANE The portion of the plasma membrane surrounding a ruffle. PLEK RASGRP2 LCP1 HSP90AA1 AIF1L DIAPH1 KSR1 AIF1 ARHGEF4 ADAM17 SH3YL1 PACSIN2 PDE9A PTPRJ PIP5K1A EPS8L1 PLEKHO1 THEM4 INPP5K DLC1 RPS3 MACF1 MYO6 SRC EPS8 APC RAB34 EMR2 PLA2G4F PPP1R9B KANK1 AKT2 ITGA5 BMX MYO1C TIAM1 SNTG1 EPS8L2 FAP SPATA13 SPRY2 TPM1 EPHA2 ITGB3 ARHGEF2 NF2 CDKL5 PAK1 PACSIN1 TLN1 TWF1 ITGB1 NME1 ABCA7 DDX58 FGD2 PLEKHA1 KIAA0528 PDPN CIB1 ARF4 FERMT1 PDXP TIRAP ITGAV WWC1 EPB41L5 CFL1 SPRY4 PIP5K1C RAC1 DNM2 TESC TRPV4 FGD5 EEF1A1 CLASP2 PDE4A FGR EZR IFIT5

GO_ER_TO_GOLGI_TRANSPORT_VESICLE A vesicle that mediates transport from the endoplasmic reticulum to the Golgi complex; bears a coat formed of the COPII coat complex proteins; such vesicles found associated with endoplasmic reticulum (ER) membranes at steady state, and are involved in ER to Golgi (anterograde) vesicle transport. SEC24A FOLR1 VTI1A CTSC HLA-DRB5 CNIH2 HLA-DQA1 HLA-DQA2 GOSR2 KLHL12 PCSK9 CD59 HLA-DRB3 HLA-F TGFA YIF1B HLA-A GRIA1 SEC24C USO1 SEC24B TEX261 HLA-H DDHD2 VANGL2 SEC23A HLA-B HLA-C HLA-E LMAN1 LMAN1L VTI1B LMAN2 AREG CTSZ SEC13 IER3IP1 F8 CD74 COL7A1 SEC22B TMED7 HLA-DPB1 SREBF1 HLA-G B2M MCFD2 TMED2 STX17 SREBF2 CNIH3 SCAP APP SERPINA1 HLA-DQB2 LMAN2L SEC24D CNIH KIAA0368 YIF1A VMA21 SEC31A TMED10 SAR1B GOLGA2 SEC23IP F5 HLA-DRA YIPF6 SEC31B HLA-DRB4 HLA-DRB1 YIPF5 HLA-DQB1 STX5 SEC23B HLA-DPA1

GO_KINESIN_COMPLEX Any complex that includes a dimer of molecules from the kinesin superfamily, a group of related proteins that contain an extended region of predicted alpha-helical coiled coil in the main chain that likely produces dimerization. The native complexes of several kinesin family members have also been shown to contain additional peptides, often designated light chains as all of the noncatalytic subunits that are currently known are smaller than the chain that contains the motor unit. Kinesin complexes generally possess a force-generating enzymatic activity, or motor, which converts the free energy of the gamma phosphate bond of ATP into mechanical work. KIF15 KIF13B NDE1 KIF19 KIF26B KIFC2 KIF9 KIF18A KIF1C KLC3 KIF25 KIF17 KIF5C KIF4B KIF21A KIFAP3 KIF14 KLC4 KIF6 KIF1A KIF3B KIF7 KIF3C YWHAE KIF23 KIF1B KIF26A KIF18B KIFC3 KIF22 KIF11 KIF2C KIF12 KIF21B CENPE LOC100130097 KIF20A KIF24 DISC1 KIF13A KLC1 KIF5B KIFC1 KIF27 KIF20B STARD9 PAFAH1B1 KIF2B KIF4A NDEL1 KIF5A LOH12CR1 KIF2A KLC2 KIF16B KIF3A

GO_MITOCHONDRIAL_INNER_MEMBRANE_PRESEQUENCE_TRANSLOCASE_COMPLEX The protein transport machinery of the mitochondrial inner membrane that contains three essential Tim proteins: Tim17 and Tim23 are thought to build a preprotein translocation channel while Tim44 interacts transiently with the matrix heat-shock protein Hsp70 to form an ATP-driven import motor. GRPEL2 C18orf55 TIMM23 TIMM17B SLC25A6 TIMM17A GRPEL1 LOC100652748 TIMM10 PAM16 TIMM50

GO_TRANS_GOLGI_NETWORK The network of interconnected tubular and cisternal structures located within the Golgi apparatus on the side distal to the endoplasmic reticulum, from which secretory vesicles emerge. The trans-Golgi network is important in the later stages of protein secretion where it is thought to play a key role in the sorting and targeting of secreted proteins to the correct destination. RHOBTB3 AP1G1 CLASP2 GGA2 TMEM79 CHID1 MMP24 PCSK1N CA4 NBEA ARFRP1 KIAA1324 RGS20 LAP3 RAB30 GOLGA4 COG8 YIPF6 PRKD1 CLTB SYT17 DNM2 RAC1 WLS CNST AP2B1 VPS54 COG6 AP1M2 BICD1 WIPI1 RAB13 STX16 C1orf88 ATXN2 APP OPTN RAB10 SCAMP5 CHAC1 GCNT1 KLHL20 MARCH9 GOLPH3L AP1S1 CD74 RAB9A GOLPH3 C11orf2 ATP7A PION SCAMP1 KIAA1432 ARFIP2 ATP9A CLTCL1 MYO18A RBFOX1 POSTN AMPH GPER MARCH4 INPP5K CLVS1 COG1 ARFIP1 ARL1 MICALL1 TMEM165 HLA-DRB3 STX10 BACE1 COG4 HLA-DQA2 MLANA SLC9A7 LGR5 CLN3 AP4S1 AP1S2 VPS52 AP1S3 AP4E1 RAB7L1 VAMP2 HLA-DPA1 ATP8A2 SNX9 RAB7B PLEKHA8 HLA-DQB1 HLA-DRB1 CHST2 AP1B1 CLVS2 AP4B1 HLA-DRB4 FNBP1 TAS2R16 ATG9A RAB6A MARCH1 SOD3 ATP2C1 HLA-DRA NCK1 TGOLN2 CLIP3 ARFGEF1 NUCB1 GGA3 CALN1 SCOC TJAP1 GBF1 ARFGEF2 KIAA0368 FAM109A CLTC VAMP3 HLA-DQB2 COG7 TGFBI LRRK2 COG5 TRAPPC9 SLC24A5 AP1M1 CPD CHST4 RAB38 STX6 VAMP4 SYS1 STX4 CBY1 BECN1 DPY30 SMPD4 RAB14 ATP9B HLA-DPB1 GCC2 CDH1 COG3 ATP8A1 FAM109B SCAMP2 STX8 RAB43 USP6NL AP4M1 M6PR BIRC6 LGR6 NCK2 KIF13A VAMP7 RAB21 SNAP25 DENND5A RGP1 ATP7B RAB11A VPS53 NMNAT2 OCRL LLGL1 RAB31 ARF1 COG2 ARAP1 SORL1 CABP7 PAM C20orf30 TRIP10 HLA-DQA1 GOLGA1 FNBP1L CORO7 VTI1A HLA-DRB5 RABEPK FURIN CLTA RAB32 ADC IGF2R MYO1B

GO_SPINDLE_MICROTUBULE Any microtubule that is part of a mitotic or meiotic spindle; anchored at one spindle pole. CALM1 BIRC8 KLHL21 TBL1XR1 KIF18A PLK1 RAB11A HDAC3 CDC16 CAPN6 CLTC AURKA MAP9 ZW10 BIRC3 CLASP1 PARP4 SKA3 ARL3 KNTC1 TUBGCP3 CALM3 CUL3 NCOR1 BIRC2 KIF3B POLB KIFAP3 HAUS2 CSNK1D TUBG1 SPAG5 CDK1 CDC27 BIRC7 PRC1 CLASP2 AURKB NLRC4 CALM2 NUMA1 KIF11 NUSAP1 SKA2 KIF18B NEIL2 KIF2A KLHL22 KIF3A KIF4A XIAP PSRC1 TUBG2 PAFAH1B1 SKA1 TBL1X AURKC C1orf96

GO_SPERM_PRINCIPAL_PIECE The segment of the sperm flagellum where the mitochondrial sheath ends, and the outer dense fibers (ODFs) associated with outer axonemal doublets 3 and 8 are replaced by the 2 longitudinal columns of the fibrous sheath (FS) which run the length of the principal piece and are stabilized by circumferential ribs. The principal piece makes up ~2/3 of the length of the sperm flagellum and is defined by the presence of the FS and of only 7 (rather than 9) ODFs which taper and then terminate near the distal end of the principal piece. PGAM4 CABYR IFT81 CCR6 ENKUR AKAP3 CATSPERG AKAP4 HK1 SPA17 CATSPER4 IFT172 TMEM146 PFKM TXNDC3 ATP2B4 IFT27 KIF2A

GO_NEURON_PROJECTION_TERMINUS The specialized, terminal region of a neuron projection such as an axon or a dendrite. SLC32A1 ADORA1 KCNK2 SNCA LRRK2 APP STXBP1 KCNIP3 PNOC ILK AP3D1 SRSF10 RAB7A SCRG1 P2RX4 FMR1 PVALB GRIK5 GRIK3 CAD CABP4 SYN1 AP1S1 PDYN TBC1D24 UCN ADRA2C PACSIN1 TULP1 FSTL3 CHRM2 CPLX1 STX6 BSN DRD2 KCNA1 FLRT3 GRIK2 AAK1 UNC13B POLG CCK MOB2 OPHN1 FLRT1 SLC18A2 PTPRN UCHL1 SV2A VAMP2 PRSS12 DLG4 ELK1 NPFF KCNC4 RAB5A P2RX3 PFN2 KCNA6 PENK CCL2 KCNAB2 SLC18A1 CDH8 SRI MME RAB3A CRHBP SEPT5 NTRK2 OXT TH SLC18A3 CALCA SYT1 NTS GHRH CHRM3 KCNC2 ITGA2 GPER RGS10 GNRH1 SYP PTPRN2 RNF40 CPLX2 VAMP1 GLUL OPRD1 SLC9A6 STX3 CHRM1 VTI1A SYNGR1 ATP6V0D1 TANC1 HCN3 NAPA SYT7 KCNA2 DNAJC5 NTSR1 DPYSL2 AP2M1 DGKI CALB2 SYNJ1 ADCYAP1 SEPT6 HNRNPR EPHA4 NMU OPRK1 DMD GOT1 UCN3 SLC17A8 CALB1 GRIN1 CNGB1 TMEM57

GO_COLLAGEN_TRIMER A protein complex consisting of three collagen chains assembled into a left-handed triple helix. These trimers typically assemble into higher order structures. EMILIN1 COL4A5 COL24A1 COL6A6 MBL2 ADIPOQ COL10A1 CCBE1 COL17A1 COL28A1 C1QL4 COL19A1 COL7A1 COL4A2 EDA GLDN C1QA C1QTNF2 COL21A1 LUM C1QTNF9B CTHRC1 COL6A3 COL5A3 COL22A1 COL1A2 FCN2 COLEC10 COL18A1 TNXB COL27A1 C1QTNF7 COL9A1 C1QL3 COL9A2 COL4A4 COL11A2 COLEC12 C1QTNF9 C1QTNF8 OTOL1 WDR33 FCN3 COL13A1 EMILIN2 LOX COL3A1 COL4A6 COLQ COL6A5 SCARA3 COL25A1 C1QTNF5 COL9A3 SFTPA2 COL2A1 FCN1 COL6A2 EMID1 COL12A1 COL15A1 SFTPD COL5A1 COL4A3 MSR1 C1QL2 COL8A1 COL5A2 COL8A2 C1QTNF6 C1QTNF3 COL16A1 C1QC DCN COL1A1 COL6A1 SFTPA1 COL20A1 EMID2 C1QB C1QTNF1 COL11A1 C1QL1 MARCO COL14A1 COL4A1 COLEC11 COL23A1

GO_EXTRACELLULAR_SPACE That part of a multicellular organism outside the cells proper, usually taken to be outside the plasma membranes, and occupied by fluid. SFRP4 MICA HIST1H2BC LOXL3 IFNA21 SPINK2 LIPC CEACAM8 CCL14 CCL19 NPFF FRZB FGF17 IL36G PRKAG3 C2orf40 IGF2 INHBA STC1 CD40LG SULF2 RBP4 GDF6 PATE2 IGHG1 MST1 EGFL6 MSTN CFD NPY AIFM2 ADAMTS20 JAM3 RPL39 PCSK2 HEATR8 KDM4D KAL1 HBEGF CLEC11A CHRD HFE2 CPM FLJ45831 SIPA1L3 C1QTNF4 PPY OAZ3 PRH2 TLE2 MTHFD2 ANGPTL2 ARTN TNFSF13 DKKL1 TPO LY86 FAM132B HDLBP SDCBP LRRC4C TCN1 FAP DPYSL3 TMC8 IL29 ECM2 WISP3 SCG2 SPACA5B CLEC18A IFNE GPC3 AGT GPT LOC439951 IL36RN F11 RNPEP FGB TGFBR3 KLK7 CXCL1 TFF3 ELANE CSN3 HAPLN3 CMTM2 AHSG CELA1 EEF1A1 BTC S100A11 PSAP SERPINA10 HPX SOD3 TIMM8B SPACA5 SERPINB11 C1QTNF6 HBG2 AMY1A RBP3 SSH2 IBSP LRRK2 NPPC EGFL7 PRH1 SFTPA1 SERPINB7 COMP XCL1 LILRB2 PXDN C1QB AMY1C CCL17 POP1 CLCA3P ABCA3 GSTP1 WNT7B SOD1 MTUS1 INHBE DEFA6 TNFSF14 KITLG KLK11 CXCL12 CRISP1 CNTF ADA C10orf99 IGLC6 GGT1 SCGB1D2 IL28B MMP10 MMP7 BMP3 AMY2A IL4 COL18A1 IGFBP5 KRT34 CXCL13 ADAM9 LOX IGLC2 CSF3 APOF RS1 USPL1 C1R CPZ FGF18 KLK13 PCSK1N KLK5 APOL1 EDN1 ADM PCSK1 APOC3 PLA2G2A CCL2 C1QC CKB CPAMD8 BMPER WNT16 TWSG1 GOLM1 TFRC FBLN1 SERPINE1 SSC5D ANGPT4 VLDLR FGL1 CELA2A ORM2 GPC2 THBS1 UCN IL26 ULBP2 CCL27 IL10 GNB2 CTSS LYZL6 BPI AREG TNFRSF9 CD97 IFNA7 CCL23 FGF9 FGF22 TNFSF13B SCGB1A1 COPA NLGN1 POSTN TNFSF9 IL20 OLFM1 LAMC2 PRDX1 FGF16 INSL4 ACTN4 APOA1BP SERPING1 EREG GHRH CTSG GDF15 NLGN3 KLK14 TGFB3 GDF7 MUC1 CD9 F12 TNFRSF6B CCL22 FLRT3 CRLF1 FREM3 APOH LTBP2 CST9L ORM1 PZP IL5RA COL6A2 AMH SEMA7A SLC4A1 PLEKHH3 COL15A1 HMSD TMIGD2 NODAL WDR60 KRT33B HBA1 TIMP1 PF4 SPINLW1 SEMA4A CLCA1 THNSL2 PCSK4 TGFB2 ABI3BP PTGDS YWHAZ FGFBP1 APOA2 KRT83 SAA2 AGR2 CARTPT PYY2 SFN C8A KRT81 ATXN10 SERPINB2 AFM B2M RNASE3 F9 PSMC5 KIT OLFM4 IL17C PCSK6 GPLD1 CPA6 DAND5 CHI3L2 CYTL1 HIST1H2BJ CCL28 VEGFA MBL2 ERBB3 RNASET2 IL4R CXCL9 CP FBN1 CAMP CST3 CRHBP GDF9 LOXL1 SORL1 EDN2 ITM2B ANGPT1 IGHA2 IL19 ITIH2 DPT ACTN1 ZSWIM5 IGF2R PAPPA FURIN FABP3 FUCA2 HABP2 SERPINA9 VASH1 C2orf56 OVOS2 PAM CTSL1 FLRT1 IFNG CFB ACTG2 MT3 FBRS PLA2G7 WNT9A KDSR LRG1 SPN GH1 FN1 CRISP2 DAG1 SORD CCL15 SEMG1 SPACA3 BTN1A1 GDF5 LYZ VWC2 WNT4 SPINK1 HSPD1 ENDOU SMARCA4 ANXA5 LPO CES5A CSF2 CCL3 HFE C1QTNF1 GPX5 KL SLC2A1 LGALS9 CPA4 APOE SERPINA6 LTBP4 MS4A1 KRIT1 IGHE BPIFA1 CEP164 CPXM1 NRG2 IFNA4 MSLN RELN WNT5A FGF1 GPX3 SEPP1 C7orf68 LTB IGLL1 VEGFC EPYC ODAM C20orf151 CD59 HYAL1 CSTB MDGA1 IL23A IGFL1 APOA5 GC DEFA4 SRPX2 IL16 ACE2 IL37 TNF SOSTDC1 MCAM CTRL SERPINE2 IL36B CXCL2 PGF AEBP1 PROL1 TCN2 KLK6 IFNK C1QTNF5 EZR NAPSA INHBB LCN2 GDF1 SFTPD WISP1 SCUBE1 ABP1 PXDNL SEMA4F PGC TFPI IGFBP2 IL3 ANGPTL4 SEMA4D CCL20 HP ALOX5 VWC2L APOC1 CPA5 ACTB SDF2 BIVM IL25 C9 MMP9 IFNW1 WNT8A NRP1 LEP ENOX1 SPARCL1 CES2 ADAMTS4 DDB1 IL15RA IL12A HSPA6 KCP PROM1 CRH CTSB WNT7A ENOX2 C1QL4 CPB1 COL7A1 ANGPTL1 AXL BMP8A IFNA16 LDLR SEMA4G PDGFC PTHLH FAM184A CLU LAMA1 IGKV1D-33 PGCP ATRN VNN3 CES1 SOST SCGB3A1 XDH SERPINA12 MTMR4 CALR B4GALT1 CHRDL2 C1S TFF1 RBMX GDF2 FAM178A CRP KAT2A IGKV1-5 KNG1 ALDH3A1 FAM132A SEMA3G SST KLK8 INHA FSTL1 FGF5 SIL1 MSR1 LTA CMTM4 KRT1 CFL1 NOG WFDC2 UMODL1 MMRN2 KRT2 PKHD1L1 OTOP1 MAN2B1 MYOC HSPA1A IL6 CCL3L3 APOM LAMB1 PNLIPRP1 C8B PRTN3 SULF1 CST4 IFNA5 CPB2 OAS3 PLA2G1B C6orf58 KRT121P SAA1 IGLL5 C3orf33 SERPINE3 TFF2 HAMP S100A8 CHEK1 NRN1L IL22RA2 COL6A3 GHR TAC4 APOO IL9R IL17F UBN2 DEFA1 CBR3 ANXA1 KRT31 CFL2 IL2 CCL4 SERPINA7 FGF12 CNTFR POTEF NPPA SFRP1 MUC16 VGF ALB CSF1 CXCL6 WNT9B TNFSF4 IFNAR2 CNP CSTA CTSH COL2A1 GAL TNFSF8 PRDX5 ICAM1 HIST1H2BK SERPINC1 IGHD IGFBP4 LPA SLIT1 CKLF ENO3 CBLN2 CTGF ALPL SMPD1 GPC5 C1QTNF3 SNCA CELA3A SEMA3F APOBR MUC5B COL20A1 PON1 GPC4 NCOA5 CST5 AKR1B1 IL7 LGALS8 FJX1 SPINK13 TNFRSF11B KLHL34 TINAGL1 FAM20C CCL4L2 BCHE LRIG3 TPI1 IL17D GRIPAP1 ZNF177 RETNLB CCL11 GPIHBP1 WNT3A BMP1 TMPRSS13 ITGA2B MMP2 WNT2B HGF SERPINB3 DEFB1 C1QTNF2 CST7 CELA2B LMAN2 PYY3 F2 TGFA CTSO MCF2L TINAG THBD VASN NRG4 ENPP1 ANG BMP8B CCL26 HDGF ELFN2 EGFR BMP10 CBLN3 CCK ZNF446 CER1 MANF HSPB1 IDE LSR TRIM75 APOL4 CDNF DMBT1 LOXL4 COL12A1 SEZ6 C9orf72 TIMP2 HSPA1B A2M CTSL3 IGKC C12orf39 CST9LP1 IL5 ENG KRT33A C4BPB HBE1 CHGA MUC5AC ENO2 ITIH1 NRG3 ERAP1 IGLC1 IL33 FGL2 C19orf10 PRSS1 GAS6 PRSS8 LIPG SERPINB5 PDE4C CPE NLGN4X FRMD7 CMTM8 GPI MRGPRD OTOG ACTC1 RETN CES3 LACRT GNLY ACSM1 ADAMTS13 CXCL16 SEMG2 SPP1 RNLS APOD ADNP ANXA13 CCL25 SERPINB4 KRT9 CFH HMGB1 SMR3B QSOX2 UTS2 PTN ERVH48-1 LOC440786 LOC100506013 CHI3L1 SEMA3B TCEB3 AVP TNFAIP6 CXCL10 LAMA5 IL36A LIF PLBD1 IL13 FRMD4B CSN2 STX2 CMTM5 FGFBP2 FIGF FGF2 IGFALS TG CAT CPN1 SEMA4C EDDM3A IL31 RTN3 ALDOA FGF10 CHIT1 COL25A1 IL11 SERPINA2 LCN1P1 PRSS33 GGH GPC6 CPA2 MMP3 OMD ST14 SECTM1 PTGIS CHIA UTP11L LIPH BGLAP SLIT3 IL27 SERPINB6 LOC646627 APOA1 VPREB1 A2ML1 REN KLK3 IFNA1 CCL16 SERPINB13 PATE4 TGFBI GAST COL1A1 TIMP3 SERPINA1 CMTM6 DEFA1B IL1F10 MERTK NUCB1 SMPDL3A FLT3LG TPSAB1 IL21 MMP8 XCL2 NPPB STX4 VCAM1 FLT1 STC2 FSTL3 EPX UBC UBB APLN BMPR2 ITGAM PPIA SPARC STOM C1QA CRISP3 SELE PNLIPRP2 ASIP MEP1A POTEI AGRP HIST1H2BG TF IGFBP3 ANPEP ABCA1 SERPINA3 EEF1A1P5 MPO TRY6 OXT IFNA10 DEFA3 HBD AIMP1 PIBF1 UCN2 PCYOX1 SELP TSKU NENF WNT8B KRT86 LRRC17 MMP20 COL3A1 APOC2 SCGB1D1 CD40 TDGF1 S100A9 INHBC C4BPA CPN2 ADAMTS5 ENPP2 CPA1 CD5L SEMA3A IL8 SERPINB8 FGF23 CHID1 PTPRG SERPINB12 SERPINB1 IK CABP1 CLEC3B SFTPC SERPINI2 BPIFC KISS1 CD109 YARS IFNA13 DEFA5 IL18 SFTPB TGS1 PIP CST6 SERPINB9 CA6 IFNB1 TAC3 MSMP MEP1B LYPD3 BPIFB1 AKR1A1 GCNT1 EGF CTSZ CES4A TNFSF11 ADAMTS3 DEFB103A CST8 ADIPOQ PODN LGALS3BP IL9 METRN CXCL11 LMCD1 EFEMP1 F7 F13A1 TNFSF12 KIAA0889 SLIT2 ACPP GNL3 TIMP4 IL22 MFNG CIB2 S100B PF4V1 IGKV3-20 CD63 CFHR3 CCDC165 CTRB2 EPGN HSPA1L PMPCA IL34 CCL8 APOB IL17B ICAM4 MFI2 MIA AZGP1 PRSS3 HSPG2 HPR C6orf174 CFI GRN CFP ESF1 C3P1 GKN2 ZNF559-ZNF177 GIF NLGN2 SFTPA2 ECM1 GREM2 VPREB3 POMC C1RL AFP CCL3L1 IGHG2 VWA2 CELA3B LYZL1 RALGAPA2 ANGPTL3 FGA WNT1 TTBK2 DNPEP ZCCHC11 DHH DKK3 LGALS3 CCDC147 SPTBN2 SEMA4B CXCL3 ADAMTS15 CECR1 PTX3 MRPL18 ITIH4 WFDC1 VCAN MUC13 HMGB2 ACTBL2 PPBP COL14A1 PROS1 ACHE CCL21 IL12B SCT WISP2 PFN1 CPNE9 MSN SMPDL3B LYZL4 PROZ POTEJ CD36 TMPRSS6 LOC100507050 CCL13 CDH13 LBP IGFBP1 IHH IGHM TSLP MUC4 LGALS1 PPFIBP2 WNT3 DKK1 BMP2 PDGFA CMTM3 CLCF1 KLHL17 GSN SEMA3D ACTG1 DBH SERPIND1 SERPINA4 EIF2A MIF CFHR1 IGHV4OR15-8 LPL SHH A1BG KIAA0556 PDGFD SELENBP1 DMXL2 MFGE8 CNOT1 FAM3B IL13RA2 IRF2BPL IGKV2-40 WNT10B HSPA7 PYY S100A4 TRDC RAMP1 THBS4 MDH1 IFNA2 FGF6 HIST1H2BE SERPINA5 VWA1 CPO PRSS2 RPS27A CX3CL1 BMP6 TCTN1 CTSD CST2 APP GCG IGHG4 EPO ZBTB38 BLOC1S1 MECP2 VSTM1 HEXB PLAT KERA CXCL14 GREM1 HIST2H2BE CPA3 CCBE1 IL1B IL18BP SERPINA11 SSPO IFNA14 NUCB2 BMP5 UCN3 SFRP5 GRP COL1A2 CTBS C1QBP HTRA1 AZU1 HIST1H2BI ZP3 ANXA2 PDGFB PCSK9 CALCA GPC1 MSMB HRG EBI3 GKN3P GNRH1 SFRP2 PRTG SAA4 PCOLCE DDR1 LOXL2 CDK13 SPINK8 MMP13 FKRP AMBP LAMP2 IFNA17 BPIFB2 TNFSF10 IGFBP6 GUSB IL17A PTPRR IGJ TNFSF18 ADAMTS9 CEACAM6 INA ALAD CPSF3L TNFRSF1A CTSK ASAH1 TPT1 DCN PROC SCRG1 IAPP SEMA3E SERPINF2 METRNL SPON2 CCL7 SPON1 ACTA2 DLK1 LECT2 OSM DSCAML1 IGHG3 IL28A APOC4 CBLN4 FASLG LEFTY2 RDX ATP4A SERPINH1 EDN3 PIGR APCS HDGFL1 LUM GBA CTHRC1 ACE PRELP C3 CORT TNC CST11 F3 HBB ACTA1 C4B IL1RN FLRT2 CETP WNT6 CTSL2 LY96 TST HGFAC PON3 DLG3 CCL5 ZFC3H1 FCN3 PRSS57 IGKV3D-11 CD14 DEFB103B COLQ C2 ENTPD6 MOV10 UCMA GDF10 FGF21 IL1A CTSW SLURP1 KLKB1 CXCL5 H2BFS BTD SEMA3C IFNA8 AMN CTF1 TAC1 CST1 SERPINF1 F5 IL24 LYZL2 ARSG WNT10A SERPINI1 BMP4 SPINT1 LIME1 CCL18 IL32 NDP CD70 FBLN5 DNAJC9 WNT2 GLB1 LCAT RAB11FIP4 IFNA6 CPXM2 CCL24 GPRC5B KRT78 HMOX1 IGFBP7 GIP IGHV3-23 CEL PPT1 TNFSF15 NLGN4Y NBL1 LTF ADCYAP1 POTEE SAAL1 MAN2A1 LGALS4 GLE1 CRELD2 CA2 QSOX1 DEFB4A APOA4 ZNF649 C5 LAMC1 HSPA2 PSAPL1 UBA52 PLTP CMTM1 HSPA8 DPEP1 CTRB1 SELS PVR FAM48B2 PCSK5 KRT35 GDF3 CST9 PRDX6 IGF1 CTSC C4A VTN LCP1 KLK12 PRDX4 WNT11 LGI1 SIAE IGHA1 TGFB1 CILP KRT85 PPP1R1A C8G DKK2 GNL1 IGKV4-1 IRAK4 PLG HIST1H2BF ANGPT2 IL15 HBA2 PODXL NUDT1 SLPI ARG1 LCN1 FGG SRGN C3orf58 GHRL CCL1 SH3BGRL CRTAP CLIC1 SPOCK1 PLA2G3 OLFM3 THPO C16orf79 LEFTY1 CTSF WNT5B PLA2G15 GFRA4 SERPINB10 PLAU OGN CPD AGA PTH IGLC7 OVOS KRT10 PRKAG2 TACSTD2 CSTL1 FKTN CMTM7 F8 IGLC3 ZG16B STAG3 GDF11 GLDN REG3A GKN1 AMY1B ENO1 FCN2 MASP1 TNXB CEACAM16 LGALS7B TNFAIP2 BMP7 FMOD LGI4 IGHV1OR21-1 GSDMD FETUB PDZD7 CREG1 NAMPT TTR SCGB2A1 OSTN BMP15 LALBA S100A13 PECAM1 INS PLA2G6

GO_AUTOPHAGOSOME A double-membrane-bounded compartment that engulfs endogenous cellular material as well as invading microorganisms to target them to the vacuole/lysosome for degradation as part of macroautophagy. ATG9A PEG3 SRPX C6orf106 FYCO1 TM9SF1 IRGM HTT CALCOCO2 PIK3R4 ATG9B NBR1 SH3GLB1 RALB C9orf72 ATG14 ENTPD4 TRIM21 ATG16L2 OSBPL7 UBQLN4 WIPI2 VPS33A GABARAPL1 STX17 ZFYVE1 WASH3P NRBF2 NCOA4 MEFV BECN1 PIP4K2C ATP13A2 WDFY3 ULK1 TBC1D17 TBC1D14 TBC1D25 GABARAPL3 WIPI1 RAB7A UBQLN1 MAP1LC3A OPTN HAP1 LRRK2 PIK3C3 PIP4K2B VPS18 VMP1 RPN2 ATG5 RAB12 VPS16 FTL TECPR1 SQSTM1 IL1B MAP1LC3C PIP4K2A VPS11 TBC1D5 ORAI1 MAP1LC3B C20orf30 CLN3 UBQLN2 DAPK2 VTI1A TBC1D12 LAMP2 TP53INP1 MAP1LC3B2 FTH1 WASH1 USP33 TMEM74 TICAM1 SNAP29 ATG16L1 TP53INP2 RAB23 AMBRA1 RAB24 GABARAPL2

GO_PROTEIN_PHOSPHATASE_TYPE_2A_COMPLEX A protein complex that has protein serine/threonine phosphatase activity that is polycation-stimulated (PCS), being directly stimulated by protamine, polylysine, or histone H1; it constitutes a subclass of several enzymes activated by different histones and polylysine, and consists of catalytic, scaffolding, and regulatory subunits. The catalytic and scaffolding subunits form the core enzyme, and the holoenzyme also includes the regulatory subunit. PPP2R2C PPP2R3A PPP2R2D NKD1 PPP2CA PPP2R1A PPP2R2B PPP2R5B PPP2R5D PPP2R4 PPP2R5A PPP2R5E PPP2R2A STRN3 STRN PPP2CB STRN4 PPP2R3B CYCS PPP2R5C

GO_TRANSCRIPTION_ELONGATION_FACTOR_COMPLEX Any protein complex that interacts with RNA polymerase II to increase (positive transcription elongation factor) or reduce (negative transcription elongation factor) the rate of transcription elongation. MLLT1 SUPT5H MMS22L TH1L NARG2 ELL ELOF1 CCNT2 ERCC6 AFF4 TCEB1 KIAA0947 TCEB3B NUFIP1 RDBP TCEA2 LEO1 EAF1 WDR61 ELL2 COBRA1 RTF1 AFF1 PEX2 TTF2 IKBKAP SUPT4H1 ELP4 SNW1 LOC100506888 CDK9 TCEB3CL THOC7 MLLT3 CDC73 TCEB3 CTR9 ZC3H8 TAF7 ELP2 RB1 ELP3 TCEB3C WHSC2 CCNT1 EAF2 TCEB2 TONSL ELL3 PAF1

GO_MRNA_CLEAVAGE_AND_POLYADENYLATION_SPECIFICITY_FACTOR_COMPLEX A multisubunit complex that binds to the canonical AAUAAA hexamer and to U-rich upstream sequence elements on the pre-mRNA, thereby stimulating the otherwise weakly active and nonspecific polymerase to elongate efficiently RNAs containing a poly(A) signal. CPSF4L SSU72 CSNK1A1 CPSF1 CSTF2T CSTF2 TUT1 CPSF2 CPSF4 FIP1L1 PIP5K1A ZC3H3 WDR33 CPSF3

GO_SUPRAMOLECULAR_COMPLEX NA BIRC8 KRTAP19-7 FLNA BCAS3 DNAH2 FERMT1 WDR43 KRTAP20-4 MAP9 PNN UPP2 CUL3 KRT73 KRTAP19-2 PALLD NCKAP5L KRTAP9-9 MT3 NAV1 LRRC49 CSNK1D RCC2 DYNC1LI1 IFLTD1 APPBP2 PKP2 MAP6D1 KIF16B BIRC5 RGS20 KRT39 LRPPRC TUBA3E KIF5B LOC730755 ODF3 HAUS7 SIRT2 RAC2 DYNLRB1 KIF26B KRTAP5-2 CDC16 KIF9 KRTAP19-5 KRTAP3-3 CYP2A6 AURKA KRTAP4-9 TPX2 KRTAP21-3 DNM1L BIRC2 KRTAP13-1 SPAG17 ODF3L2 KRTAP15-1 KIF4B TUBA4B HOOK3 CEP170 KRIT1 KRT79 FMN1 NUDC TBCC FKBP4 DNAH5 NINL KIF1B AKAP13 TBCB TEKT1 KRTAP4-3 PRPH DYNLT3 DYNLT1 TPM4 KIFC1 CCDC50 MAP6 SPAST CCT8 MAP7 KRT27 KRTAP19-8 MID2 NCKIPSD FEZ1 KRT25 EIF3A KIF17 MTA1 FAM110C KRTAP9-2 TUBB4B APC2 AXIN1 KRTAP17-1 SRPRB KRT24 SKA3 KRTAP5-4 TUBGCP3 TXNDC2 KRTAP19-4 ARHGAP4 KIF6 GRAMD3 KATNB1 KIF3C NRP1 KRTAP5-8 PCNT DLGAP2 KIF2C DNAI2 KRT13 EZR KRTAP5-9 CDK5RAP3 KIF2B TUBG2 KRTAP4-1 TUBGCP5 INA RGS14 GNG12 AMOT KRTAP10-9 RAB11A ARL6 TUBA3D KRTAP9-3 CCBP2 KRTAP10-1 KRTAP2-4 HDAC6 KRTAP10-8 SCTR ACTA1 AIF1L KRTAP5-3 MAP1A TUBB2B KRT6C MAPT CEP57 CASP14 AIF1 TPM1 TUBB8 KRT37 IQGAP2 KRT15 KRTAP20-2 MARK2 FSCN2 KRT28 TIAM1 DNAH1 KRT17 KIF13A TMEM214 FKBP15 GJA1 KRTAP1-4 KLC3 GOLGA2 NME1 CCT3 KRT2 CAPN6 KATNA1 KRT3 HSPH1 ARL3 CALM3 MNS1 PLS3 NCOR1 KRT78 SHROOM4 KRT18 KIF1A SYNM TRIM63 MAPRE1 MTUS2 KRTAP5-1 MDM1 CLASP2 KRT38 TTLL5 KRTAP19-3 GABARAPL1 KIF22 DNAH14 DNAH8 KIF26A SARM1 KRT6A KIF3A KRT1 KLHL22 RASSF3 DNM2 RAC1 KRTAP7-1 GAS2L3 CORO1A FYN SERP1 FAM82B MYO1A SYBU HAUS1 STMN1 LZTS2 KRT31 EML1 HAUS3 TBCE LCP1 DNAH9 KRTAP6-3 MYO9B ALS2CR12 SELS TCP1 KRTAP10-4 DCLK2 CKAP5 MAP3K11 KRT35 KIF21A FIGN FSCN1 SAA1 SS18 LRRC16A PRC1 KRTAP10-12 KRT72 HAUS5 SEPT9 KRTAP13-2 KRT121P ATAT1 MAP1LC3C MYO1C TEKT4 CORO1B MAP2K2 SHANK2 KRTAP10-6 VPS18 KRTAP10-3 CKAP2 SYNC PLK1 TMOD1 KRT222 HDAC3 ASPM DVL1 TLK2 DNAH11 KIF3B POLB MEFV DSP TUBE1 KRT85 INCENP CDC27 TTLL3 KRT26 RACGAP1 KRTAP9-7 WDR81 CAMSAP3 DYNLL2 HOOK1 IGBP1 CCT6A KRTAP9-6 HAUS6 CCT4 KRTAP4-11 KIAA1009 KRTAP2-1 TEKT2 KRTAP4-12 MYO6 KRTAP11-1 SLC1A4 KIF19 CAMSAP1 KRTAP12-3 DCTN1 PARP4 KNTC1 TUBGCP6 NEFL KRTAP24-1 TUBD1 MAP1LC3B KRTAP16-1 MYO1B TBCD KRT82 KRT71 REEP3 DISC1 SPAG5 EPB49 SLC8A1 HAUS4 BIN3 MID1 YES1 C18orf10 KRT10 AURKB LMNB1 EML5 SKA2 KIF5A CSNK1A1 TUBA4A SHROOM2 C9orf9 KEAP1 XIAP KIF20B AURKC TMSB4X TUBB3 SHROOM3 TUBA1C BICD1 KIAA1383 ACTC1 KRTAP1-1 HAUS8 ACTN3 KRT23 TTLL9 PAK1 KRTAP12-1 KIFAP3 TSC1 HCK CENPE VIM TUBG1 KIF24 FAM82A2 KRTAP4-8 KRTAP10-5 DNAJA3 CAPZB TTLL7 DLG1 TPPP WIPF1 KRT12 DCXR KRTAP6-2 LDLRAP1 KRT33A KRTAP12-2 PKP1 KRTAP20-3 KRTAP19-6 KIF25 KRTAP4-5 TMSB4Y KIFC2 KRTAP20-1 APC NDE1 KRTAP25-1 ZW10 EML4 CLASP1 BIRC3 KRTAP9-1 KRTAP4-2 CD2AP SLC8A3 KRTAP5-10 LMNA RUSC1 KIF5C TUBAL3 DNAH12 PRKCZ HAUS2 KIF12 NDRG1 CEP57L1 KRTAP1-3 KRT9 BIRC7 SVIL ARHGEF2 DDX6 KIFC3 KRT6B KIF18B ESPN TTLL13 KRTAP27-1 KRTAP6-1 SNPH REEP2 CSPP1 VMAC DPYSL3 PAFAH1B1 FSD1 SKA1 DNM1 ARFGEF2 EML2 TTLL4 KRTAP3-1 GABARAPL3 KRT4 KRTAP22-1 NEK2 TUBB4A KRTAP8-1 MTUS1 PBXIP1 DYNC1I2 MICAL1 NARF TTLL11 KLC4 KRTAP12-4 LMNB2 KRT74 MAP1B SPAG6 WDR47 TUBB6 KATNAL1 KIF21B CDK1 COBL MAP4 DYNC1I1 IFFO2 CTTN FBF1 ODF4 DCTN2 TPPP3 KIF4A C17orf28 LMOD2 C15orf23 CDK5RAP2 TRPV4 C19orf20 MAPRE3 TUBB2A GTSE1 TRIM55 C6orf165 DNAH6 KIF15 KATNAL2 ZNF207 KRT80 EMD DNAL4 KRT34 CCT5 GFAP KRT86 KIF14 JAKMIP1 RASSF5 HOOK2 KRTAP5-7 TUBA8 DNAH3 CALM2 NUMA1 STIM1 CENPJ WHAMM ARHGAP6 DYNLRB2 CAMSAP2 CCT7 KRTAP10-10 INO80 KIF27 KLC1 DNAH10 ACTR1A KRTAP5-6 TBL1XR1 KIF1C KRTAP4-4 FAM82A1 KIF13B C16orf80 NME2 KIF23 DES KRTAP21-1 TUBA3C ACTN2 GAS2 KRTAP9-4 TUBB1 RASSF1 WDR1 WIPF3 MID1IP1 KRTAP10-11 KRTAP10-2 MAPRE2 KIF20A EML3 TEK KRT40 KRT32 FGF13 TUBB KIF2A DNAH17 KCNAB2 TEKT3 KRT20 MAP1S KRTAP5-11 NDEL1 DYNC1H1 GPER MACF1 TUBA1B TMSB10 TTLL1 RAC3 EML6 FERMT2 NEK7 TCP11L1 TUBGCP2 RGS19 TUBA1A FAM154A TBCA KRTAP1-5 KRT19 LMOD1 TUBGCP4 KRTAP13-3 KIAA0284 ODF2 PAWR GDPD2 SYNJ1 SPRY2 SLAIN2 CYLD WIPF2 KRT5 TTLL6 KRTAP9-8 DPYSL2 DYNC2LI1 KIF11 BFSP2 RSPH1 FAM83H NEIL2 DST DNAI1 IQGAP1 NIN KRT36 DNM3 MAP2 DCX PSRC1 MYO5A CLIP1 NEFH TMSB15A KRTAP10-7 KRT83 NES FSCN3 NEK6 CLTC MAP1LC3A FAM175B REEP4 KRTAP22-2 RHOQ STAU2 DYNC2H1 PLS1 AXIN2 JUP KRT81 KRTAP13-4 TMSB15B KRTAP23-1 KRTAP26-1 TCTE3 ACTR1B KRT75 NUSAP1 KRT76 TRIM54 FLG INVS RCSD1 TBL1X KRT33B MYO3A ACTG1 CALM1 PKD2 KLHL21 KRT8 SRC RP1 KIF18A ACTN1 BFSP1 KRTAP3-2 KRTAP5-5 DNAH7 IFFO1 CRHBP BCL10 SHROOM1 AK1 RADIL NICN1 DYNC1LI2 RAB3D DAPK3 KRT7 KRT14 C16orf48 TTLL8 FAM179B MAP1LC3B2 TCHP KRT84 KRT77 NEFM NLRC4 CLMP SNTB2 CLIP2 KRTAP4-6 GAS8 DYNLL1 CCT2 KRTAP29-1 KLC2 KRTAP21-2 PTPN20B KRTAP19-1 C1orf96 RP1L1 KRT16

GO_BASAL_PLASMA_MEMBRANE The region of the plasma membrane located at the basal end of the cell. Often used in reference to animal polarized epithelial membranes, where the basal membrane is the part attached to the extracellular matrix, or in plant cells, where the basal membrane is defined with respect to the zygotic axis. P2RY12 GPR77 TF DST MYO1C CLCA2 SLC23A1 LDLRAP1 SHROOM4 MUC20 CLDN4 TACSTD2 ITGA6 KCNQ4 CEACAM1 TEK ANK3 SLC23A2 CD34 BMPR2 MET PKD2 AQP5 ERBB2IP ITGA9 OSCP1 MYO1A SLC27A5 AQP1 EPS15

GO_SMALL_NUCLEAR_RIBONUCLEOPROTEIN_COMPLEX A complex composed of RNA of the small nuclear RNA (snRNA) class and protein, found in the nucleus of a eukaryotic cell. These are typically named after the snRNA(s) they contain, e.g. U1 snRNP or U4/U6 snRNP. Many, but not all, of these complexes are involved in splicing of nuclear mRNAs. NAA38 TXNL4B LSM11 DDX23 SF3B3 LSM5 LSM7 PRPF31 SNRPA1 PRPF18 SART3 LSM2 LUC7L2 PHF5A SF3A2 SNRNP70 ZMAT2 PPIH NHP2L1 PRPF40B SNRPE LSM6 HTATSF1 SF3B14 SNRPD2 LUC7L3 ARFGEF1 LSM3 SNRNP200 TGS1 SF3A1 DDX39B PRPF40A GEMIN4 CD2BP2 CCDC97 SNRPN SF3B5 SNRPB2 LSM10 PRPF4 SNRPG LUC7L LSM4 SNRPB SNRPC SNRPD3 SNRPA SF3B1 PRPF8 SF3B2 SNRNP40 TXNL4A SNRPF SNRPD1 PRPF3 SLU7 SART1 RBMX2 PRPF6 PRPF39

GO_RNA_POLYMERASE_COMPLEX Any complex that possesses RNA polymerase activity; generally comprises a catalytic subunit and one or more additional subunits. MED4 CPSF3L TAF10 GTF2F1 GTF2H4 POLR3A TAF3 INTS12 POLA1 GRINL1B POLR2I SUPT3H GTF2F2 TAF9B POLR2E POLR3K POLR2F TERT EDF1 PAF1 INTS6 TP53 POLR2J INTS3 POLR1D ERCC1 CD3EAP POLR2G TAF1L TAF1 POLR3F INTS7 RECQL5 POLR3B GTF2A2 TAF9 POLR2A MMS19 TAF7L POLR1C MNAT1 POLR3H TAF8 POLR2H TAF5L TRRAP GTF2H2 MED14 GTF2H1 POLR2M ERCC3 INTS2 LEO1 RPRD1B RTF1 WDR61 CTDP1 RPRD2 ZNF768 INTS9 POLR2L POLR1B TAF7 TWISTNB TADA3 CTR9 CDC73 MED18 POLR3G TAF2 TBP POLR1E POLR2C GTF2A1 GTF2H3 POLR3GL INTS8 INTS1 CRCP POLR3D INTS10 TAF4 PPARGC1A CCNH C19orf2 ERCC2 MED31 TAF13 POLR1A PAPD4 POLA2 TAF11 MED6 KAT2A HELB TAF12 MCM3 TAF5 RPRD1A TAF6 PEX2 ERCC4 ERCC5 ZNRD1 POLR3E POLR3C SHFM1 MYO6 POLR2K CHD6 POLR2B TAF4B INTS5 POLR2J2 RPAP2 MED10 GTF2A1L CDK7 GTF2E2 POLR2D INTS4 TBPL1

GO_CELL_SUBSTRATE_JUNCTION A cell junction that forms a connection between a cell and the extracellular matrix. RPL13A MRC2 EPB41L5 DIXDC1 CORO1C RFWD2 CSRP2 CYBA TRPV4 ARHGAP24 SORBS2 P4HB RPL5 CAPN2 YWHAQ PAK4 LIG4 ZNF185 RPLP2 TES CTTN EZR FLT1 RHOB TLN2 PDLIM1 HNRNPK NRP1 NOX4 CD44 RPL3 MAPK3 CD99L2 ITGB6 JAK1 LRP1 SCARF2 GIT2 ACTB RPS3A PI4KA CAP1 TNC MYH9 RPL31 RPS14 SNAP23 RDX COL17A1 RPS18 TSPAN9 CDH1 L1CAM CASS4 PPIA BCAR1 NHS EFNB2 CD46 RPL38 ARPC2 ZFYVE21 EVL AIF1L FHL3 ADAM9 DDR2 ACTR2 YWHAE REXO2 LPXN AATF CSPG4 ITGA4 PPP1R12A SMPX RPS17 CALR AKAP12 FLNC FLRT2 PARVB DOCK7 ENG RPS19 FBLN7 LAP3 RPL23 RPS10 KIAA1797 HSPA1B CDC42EP1 PDLIM7 ARHGAP31 SPRY4 PIP5K1C LPP DAG1 MMP14 DCAF6 HCK RPL27 VIM ZYX LASP1 RPL19 SENP1 FLNB ATP6V0C FZD1 HSPB1 CASK FLRT1 PDLIM2 LIMS1 ALCAM FAT1 ACTC1 HSPA5 ACTN3 PAK1 BSG PALLD PLAUR NCKAP1 ANXA5 CAPN1 FLNA HYOU1 NEDD9 RAB10 FES FERMT1 ITGA8 RPS7 ILK CHP SNTB1 ITGA5 MPZL1 LAYN CDH2 CSRP1 PEAK1 ARF6 TPM4 PTK2B FAP ADD1 LMLN DMD RHOU RPS13 AFAP1 SDCBP HMGA1 YWHAG DAB2 SVIL ARHGEF2 RPS15 TLE2 ITGB3 PDGFRB PRUNE MCAM RPL30 CLASP1 ENAH TADA1 EPB41L2 ARPC5L PTK7 PACSIN2 CD151 PDIA3 CAT FHL2 KRAS RPS11 PTPRC RPL10A THY1 RAC2 RPL9 SLC9A3R2 RPS3 DLC1 NUMB CD59 TENC1 RHOA ITGAV CD81 RHOG RPS9 PVRL2 YWHAB FHL1 RPL6 NFASC ARPC3 ARHGAP26 SH3KBP1 SRCIN1 ITGB1BP1 PDCD6IP ICAM1 TSPAN4 RND3 RPLP0 FLOT2 PRKAR2A SRP68 FLRT3 RPL8 SORBS1 CNN3 CAV1 PLAU TLN1 PFN1 PPFIBP1 MAPK1 PHLDB2 TWF1 JUP CDC42 NCSTN C19orf21 CPNE3 B2M LIMK1 PPP1CC GIT1 SYNPO2 GRK5 FLII VCL ITGB4 ARPC5 CTNNA1 LIMS2 ATP6V0A2 WASF1 GRB7 CLTC PPFIA1 YWHAZ ARHGEF7 PPIB SLC9A1 GNA13 RAB21 ITGA2B RALA GNA12 SDC4 SORBS3 YES1 CDH13 TNS3 PCBP2 MSN SNTB2 RPL4 LIMD1 FGFR3 ITGA3 EPHA2 PABPC1 NRAP PGM5 LMO7 EGFR LAMTOR3 IGF2R NUP214 RRAS2 ANXA6 ADAM17 RPL7A TRIP6 ACTG1 SYNE2 S100A7 APBB1IP ITGA2 TGFB1I1 ACTN1 RPLP1 TNS1 MME RPS5 GSN ARF1 CFL1 PARVG PTPLAD1 MARCKS IRF2 RAC1 DNM2 ACTR3 CD99 PXN SDC1 CLASP2 LIMA1 RPL7 ITGA6 KIF22 STARD8 TEK MPRIP PROCR NME2 KIF23 CTNNB1 CYFIP1 ACTN2 GNB2 PARVA CNN2 FBLIM1 RPL12 RPS29 PTK2 RPL18 HSPA9 PLEC MDC1 GJA1 STX16 ITGB1 NEXN ITGA1 RPL37A RPL22 PDPK1 RSU1 HSPA1A MAP2K1 ADAM10 DST IQGAP1 CORO1B MAP2K2 RPS8 PNMA1 C2CD4B CAPN5 PPP1CB AHNAK GDI2 EHD3 HSP90B1 DPP4 CD97 RPS4X ITGA11 TRIOBP NPM1 TGM2 ATAT1 CCND3 CD9 RPS16 LCP1 HSPG2 CNN1 OPRM1 ARHGAP22 GAK SCARB2 FZD2 PIP5K1A USP33 SLC4A2 ASAP3 CAV2 PVR ERBB2IP ARPC1B FLOT1 HSPA8 ITGB5 CIB2 PTPN12 TNS4 JUB RPS2 ACTN4 ANXA1 VASP FERMT2 G3BP1 RRAS

GO_U2_TYPE_PRESPLICEOSOME A spliceosomal complex that is formed by association of the 5' splice site with the U1 snRNP, while the branch point sequence is recognized by the U2 snRNP. The prespliceosome includes many proteins in addition to those found in the U1 and U2 snRNPs. Commitment to a given pair of 5' and 3' splice sites occurs at the time of prespliceosome formation. LSM7 SNRPG LUC7L SF3A1 SNRPN PRPF40A PRPF39 LUC7L3 SNRNP70 SF3B1 SNRPC SNRPB U2AF2 SF3A2 LUC7L2 PRPF40B

GO_REPLICATION_FORK The Y-shaped region of a replicating DNA molecule, resulting from the separation of the DNA strands and in which the synthesis of new strands takes place. Also includes associated protein complexes. POLE3 POLE4 XRCC3 RPA2 GINS4 XPA RFC1 PURB BAZ1B ERCC5 GINS2 SMARCA5 POLD4 POLE2 RFC5 POLD3 XRCC2 POLD1 ZMIZ2 MMS22L H2AFX MCM3 DNMT1 CHRAC1 DMAP1 HELB NBN PIF1 PRPF19 RAD51B POLA2 RFC4 PURA POLE TP53 SMARCAL1 PRIM2 TOP1MT RFC3 SMARCAD1 RAD51C PLRG1 UBE2B TONSL RPA1 PRIM1 CDC45 PCNA RPA4 RAD51D POLA1 MCM10 CHEK1 TOP1 RFC2 CDC5L RPA3 BCAS2 BCL6 RAD18 UHRF1 ZRANB3 TIPIN

GO_DENDRITE A neuron projection that has a short, tapering, often branched, morphology, receives and integrates signals from other neurons or from sensory stimuli, and conducts a nerve impulse towards the axon or the cell body. In most neurons, the impulse is conveyed from dendrites to axon via the cell body, but in some types of unipolar neuron, the impulse does not travel via the cell body. NPFF DLG4 SEZ6 KCND2 PRSS12 GRM7 TTLL7 ALCAM CACNA1A APBB1 ADCY9 CCK KCND3 OPA1 FBXO2 MAF1 RPS6 MT3 ARHGAP44 SNAP47 GRK4 OR10H2 NLGN4X LYNX1 KIRREL CTNND1 SGCE PAK1 KLHL20 SRSF10 ILK NGDN HTR1F APP HCFC1 DBN1 PCSK2 FLNA TUBB3 PTCH1 CNGA3 JPH4 LPAR1 PTK2B SYT11 MYH10 GLRA3 GLRX2 GRIA4 GLRB ADCY2 PTEN RELN IGSF9 RPTOR SLC17A8 NEURL APOE ACCN1 ARHGEF15 ARHGEF2 MAP2K4 PALM SKOR1 ADNP PLK3 APOD WFS1 SLC9A6 CRYAB IL6ST SHANK3 FZD3 CACNA1B GABBR1 SLC8A3 DBC1 CNTNAP2 GNRH1 BMPR1A CDK5R1 HCN3 KIRREL3 TANC1 SLC12A5 PSD2 PRKAR2B HTR1B SYNDIG1 SEPT11 PPP1R9A PPP5C FAM206A SHANK1 P2RY1 RET AVP CYBB THY1 TXN2 PRKCG CCR2 OR10J5 OR10H1 LRRC4 PREX1 C19orf20 NCF1 HTR1E BGLAP CYBA GCHFR TAOK2 ZDHHC5 ZFYVE27 RCVRN OPHN1 CTTN OR5T2 INPP5F EPHA5 COBL CAPN2 MPDZ MAP1B GRID2IP MCRS1 CAMK2N1 FUS BAIAP2 PI4K2A CDK5 BECN1 IFT52 SYNPO PDYN CNR2 OR11H7 KLHL1 MTMR2 RAP1GAP DENND1A LAMP1 SOD1 RBM3 KCNE3 STX4 KCNJ2 KCNB1 KCND1 GNAI2 LRP4 OR6T1 HOMER2 LRP1 LRRK2 TMEM185A SLC32A1 SPG11 UBXN1 FMR1 GABRA5 INPP5J FEZ1 SNX14 ARFGEF2 GNAS KCNAB1 NOS1 ANXA3 NCDN EIF2C2 CANX MINK1 GRIN1 CLU TIAM1 TRIM9 GRM2 PALMD SYT4 BMPR2 PURA STRN3 BNIP3 NUMA1 NTSR1 PPARGC1A KCNA2 GSK3B ARHGAP32 NCS1 ADA MAGEE1 GRIP1 RGS8 IFNGR1 KCNH1 DLG3 IL1RAPL1 HTR1A MAPT SORT1 HTR6 FAM5B CNIH2 NSF ERO1L GRM1 C4B HDAC6 CHRM3 GRIA1 RGS14 DRP2 ELK1 CNTNAP4 WLS SRI DBNL CCL2 ARRB2 YKT6 RANGAP1 PENK MAP1S CTNND2 RAB5A NOV FGF13 SARM1 ODZ2 GABARAPL1 GRIK2 DTNBP1 ATXN1L HTR1D SRGAP2 CPEB4 HPCA MOB4 SEMA3A FXR1 EPHB3 UCN FARP1 RARA MTOR LRIT3 SYN1 ADCY4 SLC38A2 SLC8A2 KCNA1 DRD2 TRPM5 CNIH3 EIF4A3 ATCAY BSN CHRM2 ACTN2 BPTF HTR2B MAPK8IP1 AKAP9 KCNIP3 UHMK1 GABRB1 ARF4 PLK2 ITGB1 GRIK3 SHARPIN GRIK5 OR10H3 NRG1 CPEB1 KCNJ14 SIPA1L1 GIPC1 NLGN1 OPRK1 DNM3 GRM3 GABRA2 ACAD9 GNB2L1 GNAQ HTR7 MAGI2 CRTC1 SHANK2 GPM6A CPNE6 ZMYND8 AMFR RUFY3 CALB1 SACS PPT1 KCNK1 LRP8 STRN C19orf2 PLXDC1 DPYSL2 HNRNPR CRIPT GNG3 GNG13 DGKI STX3 ITPKA EPHB1 KPNA1 CPLX2 DNER ARC SLC5A7 ARRB1 RAB27A CHL1 OPRM1 C4A PPP1CA NTRK1 CHRM1 OR5T3 NRGN RAB8A KCNN2 C20orf103 STRN4 GOPC TH CHRNA3 KNDC1 STAU1 RGS10 RIN1 GPER CPT1C FAM5C HTR2C SYNGAP1 RGS12 GRM6 TRPC5 LRFN3 HTR2A CHRNA4 LZTS1 SUMO1 AMIGO1 COMT ELFN1 GNAZ ZWINT CTTNBP2 DPYSL5 P2RX3 HTT GNB1 MAX RPLP0 LAMA2 TRAPPC4 HTR5A SRCIN1 SLC4A10 LSM1 GLRX3 TP63 EPHA7 PCDH8 RAB17 BRD1 DAB2IP ATXN10 PSEN1 MAPK1 CPLX1 CNN3 DVL1 PLCB4 ANK3 RBM8A LMTK3 PNOC GLRX5 KCNB2 CIB1 ADORA1 OR10H4 BMPR1B PTPRO MAPK8IP3 ACVRL1 P2RX4 PPP1CC OR11H4 GLRA1 ATP1A3 IGF2BP1 MYL7 ADCY10 MLPH NR1D1 GPHN EPHA4 RPL28 OSBP2 ALS2 PDE4B ELOVL5 PIAS3 STAT1 CDKL5 OR10H5 TRPV1 SAMD4A AQP11 ZNF385A GNB3 IFT57 SLC8A1 ADC FRMPD4 NELF GNAO1 GLRA4 OR10J6P KCNIP1 DICER1 NPTN HCN1 KLHL17 ATP1A2 ANKS1B CAPRIN1 IGSF9B KCNC1 ASAP1 SLC1A4 PPP1R9B CRHBP IFT20 MME NF1 PTPRK KCNC2 MYO1D GRID2 CPEB3 EPHB2

GO_PALMITOYLTRANSFERASE_COMPLEX A protein complex with palmitoyltransferase activity. SPTLC3 ORMDL3 GOLGA7 GOLGA7B SPTLC2 SPTSSB ORMDL1 SPTSSA ORMDL2 SPTLC1 ZDHHC9

GO_MITOTIC_SPINDLE A spindle that forms as part of mitosis. Mitotic and meiotic spindles contain distinctive complements of proteins associated with microtubules. KIF18A SIRT2 RPS3 GOLGA2 PKD2 EML2 AGBL5 EML1 CLTC AURKA MZT1 MAP9 IKBKG PKP4 KIAA1383 ECT2 KAT2A MAPK1 MAK DIAPH1 ASPM KIF23 CDC42 ESPL1 MAD2L1 TPR CAPG CDC14A TUBGCP4 RCC2 CDK1 RACGAP1 C19orf46 MAP4 CLASP2 CENPE GPSM2 SPAG5 YEATS2 ATAT1 CTTN NUMA1 GEM MAD1L1 KIF22 TADA3 NIN OR2A4 RANGAP1 FAM83D C15orf23 DYNLL1 KLHL22 PKHD1 C1orf96 EFHC1 CDK5RAP2 KIF20B

GO_COPI_COATED_VESICLE A vesicle with a coat formed of the COPI coat complex proteins. COPI-coated vesicles are found associated with Golgi membranes at steady state, are involved in Golgi to endoplasmic reticulum (retrograde) vesicle transport, and possibly also in intra-Golgi transport. COPB1 TMED3 TMED2 KDELR1 PACSIN1 COPZ2 PACS1 COPA COPG CCDC115 COPE TMED7 TMED10 TMEM199 COPZ1 AP3B2 ARCN1 ARF1 COPG2 SCYL1 C3orf58 COPB2
[truncated: 300,371 more chars]
