# Supplementary material for: Exploring autophagy with Gene Ontology
Source: Autophagy. 2018 Feb 17;14(3):419–36. doi: 10.1080/15548627.2017.1415189 (PMC5915032; doi:10.1080/15548627.2017.1415189)
Supplement: supp_data_1415189.zip [file kaup-14-03-1415189-s001.zip › supp_data_1415189/supp_data_1415189_s03.docx]

**File S3.** Molecular function GO gene set used to perform Gene Set Enrichment Analysis. Analysis was performed as detailed in Materials and Methods.

GO_LYSOPHOSPHOLIPID_ACYLTRANSFERASE_ACTIVITY Catalysis of the transfer of acyl groups from an acyl-CoA to a lysophospholipid. ABHD5 LPGAT1 LPCAT2 LCLAT1 LPCAT4 LPCAT3 AGPAT4 TAZ MBOAT7 LPCAT1 AGPAT1 CRLS1 AGPAT5 MBOAT1 AGPAT2 AGPAT9 AGPAT6 MBOAT2 AGPAT3

GO_NUCLEOSIDE_TRIPHOSPHATASE_REGULATOR_ACTIVITY Modulates the rate of NTP hydrolysis by a NTPase. RIN3 PIK3R2 TBC1D19 RALBP1 DOCK1 PLXNB1 RAB3GAP2 ARHGAP25 SRGAP3 STARD13 ARHGDIG RAP1GAP2 TBC1D2B RASA4B AGAP3 TBC1D25 TBC1D14 SH3BP4 AGFG2 AGAP5 ARHGAP35 HTR2B CDC42EP5 RIMS1 TBC1D3C HSPH1 TBC1D20 ACAP2 RABEP2 ARHGAP21 STARD8 SRGAP2 CDC42EP3 SMAP2 RASA3 TBC1D8B PTPLAD1 RANGAP1 INPP5B GOPC DEPDC1B RAB3A RIN1 ARAP3 RGS10 SYNGAP1 RGS12 SYDE2 ALS2CL RGS3 RINL RGS1 ASAP3 RGS18 TBC1D10A BCR RGS19 ARHGAP22 ARRB1 ATP1B3 MYO9B TBC1D7 DEPDC1 ALDH1A1 TBC1D12 BAG2 NPRL2 EVI5L GDI2 RP2 DGKI GMIP TBC1D26 KALRN SIPA1L1 SLIT2 ARHGAP33 MYBPC3 CTGLF11P RASAL3 IQGAP1 GNAQ TBC1D21 CHML AGAP2 TBC1D3 TBC1D1 RGS7 ARFGEF1 SGSM2 RUNDC1 GIT1 PLCB1 TBC1D24 GAPVD1 AXIN2 AHSA2 ELMOD1 DAB2IP PFN1 ARHGAP8 WASL IPO5 GPSM1 RABGAP1L FAM13B WNT11 RAB3GAP1 RACGAP1 PREX2 ARHGAP26 ELMOD2 ITGB1BP1 RGS9 CHN1 RALGAPA2 RASA1 RIC8A ARHGAP28 RAPGEF2 RGS13 RIC8B ASAP1 ATPIF1 ATP1B2 ARAP1 TBC1D15 AGAP9 NF1 C16orf7 ARHGDIA DNAJC10 ATP1B1 SRGAP2P1 EVI5 TBCD LLGL2 DNAJC1 RGS4 GNB5 CDC42EP4 AGAP4 DEPDC7 ADAP1 SMAP1 SIPA1L2 SRGAP1 RALGAPA1 ARHGAP17 TBC1D10C TBC1D22A SH3BGRL3 DOCK2 RGS2 MYO9A ARHGAP23 ARHGAP36 PGAM5 ARHGAP20 STXBP5 TBC1D8 PLEKHG6 ADAP2 ARHGAP19 ABR ARFGAP3 RGS22 RAP1GDS1 VAV3 CPEB2 TBC1D17 DEPDC5 RGS6 BAG1 TBC1D16 SIPA1L3 TBC1D9B AGAP6 TBC1D10B ARHGAP10 ACAP1 ELMOD3 DNAJA3 TSC1 GRTP1 ARHGAP44 DNAJC2 A2M CDC42EP1 RUNDC3A ARHGAP27 GRPEL2 TBC1D2 ARHGAP39 ARHGAP31 RGS5 ARHGEF1 RGS20 ARHGAP40 TIAM2 SIPA1 NPRL3 TBC1D30 TBC1D9 ARHGAP11A ARHGAP15 ADPRHL1 GPSM3 AHSA1 TBC1D22B ECT2 THY1 DLC1 AGAP11 TBC1D3F GRPEL1 TBC1D3B DNAJC7 ERRFI1 STXBP5L TBC1D13 BAG5 TAGAP DNM1L TOR1AIP1 ARHGEF11 RASAL1 RALGAPB GDI1 ARHGEF15 AGAP1 KRIT1 ARL2 DNAJC24 ARAP2 SESN2 RANBP1 NCKAP1L RASAL2 GIT2 ARHGAP5 RABGAP1 LRRK2 AXIN1 ANKRD27 ARHGAP9 AGAP7 RTKN CHN2 TBC1D3H RAP1GAP SGSM1 ARHGAP4 ARHGAP11B OPHN1 JUN HMHA1 IPO7 ARHGAP29 CDC42EP2 ASAP2 PREX1 TNK2 ARHGAP1 BAG4 RGS21 BAI3 GPS1 ARHGAP24 ARHGEF6 TBCK PFN2 LLGL1 RASA4 SOS1 RASGRP3 ADPRH RALGDS RABEP1 SYDE1 RGS14 ARFGAP1 CHM RGS11 OCRL PREB PLN TBC1D4 ARHGAP42 RASA2 RGS16 RGSL1 TSC2 SGSM3 TRIP10 CDC42SE1 PCP2 RAB4A TBC1D5 BRSK2 WAS DOCK4 ARHGAP6 SH3BP1 ARHGEF12 ARL2BP USP6NL FAM13A ARFGAP2 IQGAP2 ARHGAP12 ARHGAP32 ARHGEF19 BNIP2 PDE6D RGS8 ACAP3 GPSM2 AGFG1 RHOH RGS17 ARHGAP30 GARNL3 ARHGAP18 ARHGDIB RIN2 C15orf62 BAG3 GPS2 DNAJB6 TOR1AIP2 DNAJB1

GO_HISTONE_DEACETYLASE_ACTIVITY_H3_K14_SPECIFIC_ Catalysis of the reaction: histone H3 N6-acetyl-L-lysine (position 14) + H2O = histone H3 L-lysine (position 14) + acetate. This reaction represents the removal of an acetyl group from lysine at position 14 of the histone H3 protein. HDAC9 HDAC7 HDAC10 HDAC5 HDAC1 HDAC3 SIRT3 HDAC11 HDAC2 HDAC8 HDAC6 HDAC4

GO_ENDONUCLEASE_ACTIVITY_ACTIVE_WITH_EITHER_RIBO_OR_DEOXYRIBONUCLEIC_ACIDS_AND_PRODUCING_5_PHOSPHOMONOESTERS Catalysis of the hydrolysis of ester linkages within nucleic acids by creating internal breaks to yield 5'-phosphomonoesters. KIAA0391 SLX4 ERVK-10 RNASEH2B RPP38 ELAC1 RNASEH1 ENDOV DNASE1 LOC100128274 DICER1 MRPL44 POP7 EXO1 ERVK-7 POP1 ERVK-8 EDC3 POP4 POP5 FAN1 RNASEH2A SLX1A ERVK-6 DROSHA LOC100133495 RPP30 RPP21 FEN1 DGCR8 DNA2 RNASEH2C DBR1 TATDN1 RPP40 EIF2C2 APEX1 TATDN2 RPP25 SLX1B

GO_TRANSFERASE_ACTIVITY_TRANSFERRING_ONE_CARBON_GROUPS Catalysis of the transfer of a one-carbon group from one compound (donor) to another (acceptor). WDR4 ECE2 ALDH1L2 N6AMT2 PRMT5 METTL14 METTL12 METTL5 FTSJD2 METTL19 SHMT2 SUV39H2 CXXC1 METTL2A METTL11B NOP2 TGS1 METTL21B FTSJ1 TYW3 CAD METTL21D HENMT1 METTL7A FAM86B2 LCMT2 METTL2B TRMT1 SETD3 METTL20 DYDC2 MLL5 METTL23 FBXO11 PRDM7 SHMT1 TRMT1L ALDH1L1 PRDM1 DYDC1 CIAPIN1 NSUN5P2 DNMT3B CARM1 PRDM13 METTL6 PRDM2 PRDM10 SMYD1 DIMT1 RRP8 HNMT METTL22 SETD1A GAMT DOT1L SETD7 SETMAR PCMTD1 NSUN2 PRDM15 TRMT61A MTFMT SMYD4 EZH1 TFB1M MTR METTL21CP1 METTL21C ASMTL METTL8 ASMT METTL21A FTCD LRTOMT PRDM16 SMYD2 SETDB1 MLL2 GNMT WHSC1 PRMT7 EMG1 ASH1L METTL18 RG9MTD2 SETD2 DPH5 PRMT8 RNMT KIAA1456 CAMKMT ZCCHC4 MEPCE FAM86A SMYD3 METTL11A PEMT MRM1 C9orf114 NSUN4 METTL4 BHMT C9orf41 CCDC76 PNMT NSUN7 PRDM14 BCDIN3D NSUN3 METTL15 MLL4 DPY30 MGMT MECOM METTL3 C12orf26 METTL17 C20orf7 FBL AS3MT GCSH FTSJD1 EHMT1 GART SETD4 SUZ12 TRMT112 TFB2M EED PRDM6 MLL3 COMT SETD8 NSUN5P1 SETD6 ATIC FAM86C1 LCMT1 ASH2L TRDMT1 PRDM9 EZH2 PRDM11 WDR82 PRDM5 EDF1 PRDM4 RRNAD1 MEN1 INMT NSUN5 GATM TARBP1 ICMT RNMTL1 TRMT5 SETD1B PRDM12 EHMT2 C2orf56 RG9MTD3 DNMT1 RG9MTD1 METTL7B METTL1 THUMPD3 WBSCR22 COQ5 MLL METTL10 FAM86C2P FBLL1 RBBP5 TRMT2A FTSJ2 HEMK1 C5orf35 C6orf186 METTL13 PRMT6 C7orf60 TRMT61B SUV420H1 C6orf211 PCMTD2 PCMT1 WDR77 FTSJ3 OTC COQ3 TPMT THUMPD2 IRF4 SETDB2 SUV39H1 PRMT1 METTL16 METTL15P1 DNMT3A PRDM8 TRMT2B WDR5 WBSCR27 NSD1 N6AMT1 PRMT2 TRMT11 TYMS COMTD1 NNMT SUV420H2 AMT C9orf156 PRMT10 ALKBH8 SMYD5 WHSC1L1 BHMT2 CPS1 PRMT3 FDXACB1 NSUN6

GO_WNT_ACTIVATED_RECEPTOR_ACTIVITY Combining with a Wnt protein and transmitting the signal across the plasma membrane to initiate a change in cell activity. FRZB FZD6 RYK FZD3 SFRP2 FZD2 SMO LRP5L SFRP1 FZD4 EGF SFRP5 FZD10 TSPAN12 LRP5 FZD7 SFRP4 FZD1 FZD9 FZD8 FZD5 LRP6

GO_C_ACYLTRANSFERASE_ACTIVITY Catalysis of the transfer of an acyl group to a carbon atom on the acceptor molecule. SPTSSB ACAA2 GCAT SPTLC2 SPTLC3 HADHB ACAT1 ACAA1 ACSM4 SPTLC1 ACAT2 SCP2 SPTSSA HADHA

GO_CARBOHYDRATE_KINASE_ACTIVITY Catalysis of the transfer of a phosphate group, usually from ATP, to a carbohydrate substrate molecule. HK3 GNE GALK2 GCK SGK196 KHK XYLB PFKFB4 RBKS HKDC1 HK1 HK2 PFKL PFKFB3 PFKM PFKFB1 GALK1 PFKP PFKFB2 NAGK

GO_UBIQUITIN_LIKE_PROTEIN_LIGASE_BINDING Interacting selectively and non-covalently with a ubiquitin-like protein ligase, such as ubiquitin-ligase. KLHL11 TRIB1 USP13 RNF40 DLG3 CD40 YWHAE DTX1 FAM178A HDAC6 PA2G4 UBE2V1 NDUFS2 NGFR CALR NKD2 TOLLIP UBE2V2 UBE2W DERL1 TSG101 CLU RB1 DIO2 BLZF1 PPARGC1A STX8 RELA HLTF GLMN GSK3B TCEB2 TMEM189 DBT BECN1 NLK FZD6 PIAS2 ASB18 PINK1 SMAD3 POLR2A XBP1 FZD4 MOAP1 SUMO2 SMAD2 UBE2J1 AXIN1 SLC22A18 TRAF4 MYOD1 UBXN1 JAK1 DDRGK1 BAG4 MAGEA2 PTPN22 UBE2J2 CXCR4 AICDA CDK5RAP3 MAGEC2 UBE2H BRCA1 UBE2R2 RNF20 RRAGA BAG5 DNM1L PRKAR2B ABI2 BID SHPRH CEBPB AURKA FAF2 HERC2 KBTBD4 CASP10 GABARAPL2 SMC6 RBX1 ANKRD32 FZD8 SNCAIP ERLIN1 TRAF5 CCDC50 LTBR SYT11 TRIM37 SMG5 TANK ABTB2 RIPK1 WFS1 UBE2NL SMAD7 UBE2K TRAF1 SCAMP3 PER1 XRCC5 CUL3 TRIM28 GPI HSPA5 MC1R CUL4B HSPD1 IKBKG FBXO7 CDC34 SKI UBE2N FZD5 ATF6 BTBD2 PRR5L PIAS4 LRPPRC TNFRSF1B ZNF675 HSPA1B EIF4E2 MPHOSPH8 BTBD6 PRKAR1A MFN2 MUL1 TP53 ATP6V0C BTBD1 JKAMP VCP WASH1 FAF1 EGFR MAP1LC3B PER3 ASB10 CASC3 ASB16 BCL10 ABTB1 AKTIP CUL2 RNF34 NPLOC4 UBE2U SLC25A5 ACTG1 POU5F1 BTBD9 TMEM173 ATXN3 UBE2C CUL7 BTBD11 CCT2 RFFL TPI1 PCBP2 ASB4 MID1 PAX6 RALA IKBKE SCN5A YOD1 FANCL AXIN2 CBS ZNF746 PRKACB USP19 GPR37 UBE2T NFKBIA MAP1LC3A DAXX YWHAZ BAG6 UBXN7 EGR2 VCL C10orf46 SUMO1 RNF8 PACRG CUL1 TRAF6 HM13 TRAF2 PTPRN ANAPC2 OTUB1 UBE2Z ASB3 PRKAR2A UBE2A KDM4A KCNH2 LYN UBE2B MAGEA2B UCHL1 UBE2G1 SNX9 RNF31 UBE2M TCP1 UBE2O ARRB1 PRDX6 BTBD3 NAE1 AMBRA1 USP7 UBE2I FBXW7 HSPA1L TUBA1B HIF1A HSPA8 STUB1 UQCRC1 TRIB2 ASB15 USP25 FHIT CHEK2 ARIH1 CUL9 MAP1LC3C TRIP4 BCL2 TRIOBP PML TXNIP MC4R TNFRSF14 SQSTM1 PARK2 HSPA9 PRKACA ACVR1B UBE2L3 SPG20 SMAD5 ERLIN2 HSPA1A UBOX5 CKB RPA2 FOXO1 USP2 TMBIM6 TRAF3 NEDD8 RALB CUL5 ARRB2 UBE2G2 RANGAP1 PIAS1 UBE2F ASB14 DNAJA1 UBE2S SPOPL RAD18 TUBB MDM2 CUL4A CASP8 GABARAPL1 GRIK2 CDKN1A TRIB3 SMAD6 SPOP

GO_DIACYLGLYCEROL_KINASE_ACTIVITY Catalysis of the reaction: NTP + 1,2-diacylglycerol = NDP + 1,2-diacylglycerol-3-phosphate. DGKH DGKI DGKK DGKE DGKB DGKG DGKQ AGK DGKZ DGKD DGKA

GO_3_5_DNA_HELICASE_ACTIVITY Catalysis of the unwinding of the DNA helix in the direction 3' to 5'. GINS2 RECQL4 RECQL5 BLM FBXO18 GINS1 ASCC3 GINS4 ERCC3 RECQL WRN CDC45

GO_EXTRACELLULAR_MATRIX_STRUCTURAL_CONSTITUENT The action of a molecule that contributes to the structural integrity of the extracellular matrix. HAPLN2 FBN3 TECTB FBN2 DSPP HAPLN4 COL11A2 COL4A4 COL9A2 OPTC COL9A1 COL27A1 EMILIN2 ACAN EFEMP2 ELN COL4A6 LAMA4 COL3A1 MGP COL24A1 COL4A5 COL4A2 COL19A1 MUC17 MEPE HAPLN1 IMPG2 NCAN MUC6 STATH LUM TFPI2 FBN1 MATN3 LAMC1 LAMA1 PRELP CHI3L1 MUC4 COL1A2 COL5A3 AMBN COL1A1 IMPG1 LAMB1 PXDN COMP BCAN FBLN1 COL14A1 COL11A1 VCAN MUC3A KAL1 COL4A1 TINAGL1 CD4 COL2A1 AMELY COL9A3 HAPLN3 AMELX ENAM BGN TECTA COL5A1 COL4A3 COL15A1 COL12A1 MATN1 MUC5AC MFAP5 COL5A2 TUFT1 COL8A2 FBLN2

GO_PEPTIDE_HORMONE_BINDING Interacting selectively and non-covalently with any peptide with hormonal activity in animals. MC4R IDE GALR3 MAS1 ACVR1 GHR INHBA NPR1 PIK3R1 NPR2 INSR AGTR2 C2CD2L IGF1R GCGR SLC40A1 GIPR CALCR EDNRB HCRTR2 GALR1 HSPD1 MC3R CRHBP GALR2 RXFP2 GHSR ECE1 HCRTR1 PTH1R AVPR1A NPR3 OXTR PRLR GHRHR

GO_IMMUNOGLOBULIN_RECEPTOR_BINDING Interacting selectively and non-covalently with one or more specific sites on an immunoglobulin receptor molecule. CLEC4D IGHE IGHG4 IGLL5 IGLC3 TRDC FLNA IGJ IGHV1OR21-1 IGLC6 IGHG2 IGHV3-23 FES IGLC7 IGHG1 IGLL1 IGKV3-20 IGHA1 IGHA2 FGR IGLC1 IGLC2 IGHV4OR15-8 IGHD IGKC IGHG3 IGHM

GO_RECEPTOR_ACTIVATOR_ACTIVITY The function of interacting (directly or indirectly) with receptors such that the proportion of receptors in the active form is increased. CXCL13 WNT1 IGF2 ACTN2 WNT5A NCOA2 WNT3 PRKCE GREM1 EGF NODAL SFRP2 WNT2 CDK5 ANGPT4 WNT3A GAS6 NRG1 WNT10B WNT7A WNT8A BAZ1B MED1 WNT4 EFNA5 VEGFA ANXA2 NRG3 PPARGC1B MED16

GO_COENZYME_BINDING Interacting selectively and non-covalently with a coenzyme, any of various nonprotein organic cofactors that are required, in addition to an enzyme and a substrate, for an enzymatic reaction to proceed. ME2 ETFDH PARP1 ACADVL PPOX MOSC1 FMO3 FMO2 GSR ACOXL ACOT7 IDH3A NDUFS2 DUS4L CRYZL1 MOSC2 QDPR SIRT3 DUS1L XDH IDH1 IDH3B KCNAB1 NOS1 CYB5R4 NNT ALDH1A3 DHCR24 TXNRD1 ACBD6 NOX5 UGDH ACADSB ACADM HIBADH CYB5R2 KMO ALAS2 PHGDH SIRT5 GAPDH MAOB MMACHC SIRT1 HMGCL FMO5 ETFA DHCR7 MTHFR NOX4 GLYR1 CTBP1 MICAL1 ACADS HACL1 ACBD4 ACAD11 SQLE TSTA3 G6PD GCH1 DBI SIRT6 DHFRL1 CRYZ BDH2 FASN TM7SF2 CHDH NDOR1 HSD17B8 AOX1 DECR1 ACBD5 HADH ME3 SOAT2 DHFR GAPDHS SIRT4 HMGCR DHFRP1 KDM1B SIRT7 NDUFV1 TXNRD3 FOXRED2 ACOX3 ACBD3 TDH SRD5A1 GPD1 CAT ADH4 ACOX2 GPD1L OGDH IDH2 ACOX1 ACBD7 HPGD ACADL CBR4 GCDH ACAT1 NOS2 AHCY POR CBR3 SPR ASPDH TH D2HGDH PRODH CYBB SIRT2 ECI2 DDO OGDHL KDM1A GCLC ACAD9 SCP2 CYB5R3 NOS3 CRY2 UXS1 OXNAD1 NOX1 DUS3L SDHA ATAT1 ILVBL CTBP2 ACAD10 LDHD CRYM GLUD1 GRHPR ALDH6A1 SUOX MTRR HSD11B2 FMO4 AIFM1 TP53I3 MTO1 TXNRD2 MICAL3 GFER NDUFA9 DPYD FMO1 CYB5R1 DHTKD1 DUS2L DAO AIFM2 COQ6 SORD HADHA WWOX DUOX1 IVD GMDS CYB5RL AGPS DLD ME1 ACAD8 CRYL1 AIFM3 LDHA MDH1 H6PD LDHB SOAT1 MICAL2

GO_RNA_POLYMERASE_II_TRANSCRIPTION_FACTOR_ACTIVITY_SEQUENCE_SPECIFIC_DNA_BINDING Interacting selectively and non-covalently with a specific DNA sequence in order to modulate transcription by RNA polymerase II. The transcription factor may or may not also interact selectively with a protein or macromolecular complex. CEBPA EBF4 HMGA2 ETV7 FAM200B AIRE SOHLH2 HEYL ZMYM3 REL TLE4 MYC FOXD4L6 HOXA5 TAL1 NR2F6 USF2 ZBTB20 NACC2 STK16 THRB SATB1 NEUROG3 KLF17 JDP2 TBX2 SOX4 RORC ONECUT2 C1orf85 NFATC1 ELK3 GTF2I NFX1 SKIL SPI1 GABPA HELT FOXR1 ZKSCAN3 E4F1 HES5 NKX6-2 HIF1A TFDP1 RARG BCL11B ONECUT3 NFATC2 PCBP3 SIM1 CIR1 PTF1A PEG3 ZMYM5 ZSCAN5C NFATC3 IRF7 PRDM1 SRY TFDP2 TAF9B STAG1 TTF1 EHF HOXD9 KLF12 NR0B1 GCM2 ARHGAP35 GRHL2 ZNF518A SOX2 CTCF USF1 PHB DLX3 SOX10 NFKB1 BATF3 ZNF496 SREBF1 HOXC11 FOXO1 CEBPZ ARNTL NR2E3 KCNIP3 NR5A2 BARHL2 IRF4 VAX2 SMAD4 DMRT1 MYBL2 ZSCAN5A MYPOP RFX1 TRPS1 POU5F1 QRICH1 FOXO3 MEOX2 PAX6 FOXD4L3 IRF8 GMEB1 SOX18 STAT1 SCRT2 MESP1 TCF7 TFAP4 POU2F2 TP73 MYOCD RARB C2orf3 KLF11 ZNF350 FOXA3 FOXF1 MTF1 RORB DDN T CEBPE RUNX1 ZFP90 PITX2 SIX4 NFKB2 CDX4 ATF4 DMBX1 FOXD3 BACH2 RXRB AKNA ZFAT ZBTB16 ZIC1 KIAA1958 DLX2 NPAS3 TXK KLF16 GZF1 GLIS1 MSGN1 TBX15 INSM1 TAF1L FOXL1 PTH TCF3 STAT3 PAX9 ZNF746 FOS ZSCAN5D MEIS2 HNF4G GRHL1 GATA4 ASCL2 CC2D1A EGR2 ZFPM1 ESR2 NEUROD1 TEAD1 HHEX ZNF18 ZNF174 SIX3 NR4A2 MEIS3 SOX15 BRD8 PDX1 VDR NR2E1 CAMTA2 ZMYM6 KLF13 PAX8 SOHLH1 HMGA1 NFE2L3 ZNF292 FOXR2 RUNX3 ZNF280A GATA6 DACH2 AEBP2 ZEB1 CHCHD3 HOXC13 BARHL1 RBPJL MYB WT1 CGGBP1 ZNF148 GLI2 FUBP3 TFAP2C CREB3L1 CEBPB TFAM PROP1 EP300 POU4F3 RUNX2 SOX17 HIVEP1 ARNT BARX1 E2F7 JUND NRL TP53 FOXP3 ARNT2 FOXP4 KLF5 OVOL2 HOXA10 SOX7 ALX4 TFAP2E RELB PPARD EPAS1 TSHZ1 PROX1 NFIL3 TCF12 ZNF536 OSR2 MAFG MITF ZNF219 GLI1 BCL6B ATF3 ATOH1 FOXO6 ZNF518B SPDEF ZSCAN21 HEY2 ZC3H8 ZBED5 KLF4 TCF4 FOXP1 HOXA2 MEF2C MXD1 SMAD1 NR1H3 RELA PURA ELK4 TAL2 ARID5B PKNOX1 THRA CSDA GATA2 MYOG FOXG1 MEF2B NR1H2 EBF2 CREB3 MAFA OVOL3 ALS2CR8 NFAT5 BARX2 HAND1 NKX2-1 ZNF131 NFATC4 CDX1 DLX5 PAX5 BHLHE40 ZIC3 PLAGL1 HSF2 NFIA NKX3-1 BHLHE22 ETV4 TBX3 SOX12 SIX2 FOXI3 PCGF6 ASCL1 GRHL3 MYF6 FOSL2 HNF1B AEBP1 HOXB5 CDX2 CUX2 CLOCK FOXO4 SOX1 MAF MZF1 HNRNPK SREBF2 TAF9 RBPJ MNT ISX FEZF1 POU3F2 BHLHA15 CREB3L3 NKX2-5 FOXC2 ETS1 BCL6 E2F4 ESRRB CTCFL SPIC TAF2 HMX1 FOXC1 ARX POU2F3 OLIG2 RFX6 RXRA BHLHE41 FOXQ1 YY1 NR2F2 ZNF281 ZSCAN1 CREB3L4 FOXA1 HES1 NR4A1 RXRG LEF1 NRF1 NFIB GPER TBX5 GFI1 NR1H4 ERF TSHZ2 IKZF3 ETV5 CSRNP1 FOXB1 IRF2 NFXL1 ELK1 OVOL1 PAX4 CSRNP2 EBF3 FOXE1 FOXD4 HAND2 ZSCAN29 ZGPAT KLF15 ISL1 CREB3L2 OTX1 ZGLP1 TAF1 SRF SALL1 FOXD4L1 FAM200A PLAG1 TFE3 SNAPC4 ZEB2 SOX11 RARA ARID3C DMTF1 ESRRA ERG TFAP2D EN1 FOXJ1 HIF3A GATA3 HSF1 ZNF280D NR1D1 SIM2 REST YBX1 NKX2-8 NHLH1 MSC HOXD13 IRF1 ZNF202 ESX1 BCL11A TFDP3 NR2F1 MEIS1 AHR ETV1 ESRRG IRF3 MEOX1 DBP FOXS1 PITX1 ZMYM1 ASCL3 BTG2 CEBPD MIXL1 TARDBP GATA5 SPIB NR2C1 MEF2D EBF1 CDC5L FOSB PPARA ZFHX3 IFI16 NR4A3 FERD3L ETV3 ALX1 FOXK2 GSX1 DLX4 SOX14 ATF2 SNAI2 NFE2L1 MSX1 POU1F1 TFAP2A POU4F1 MAX NKX6-1 FOXA2 RCOR1 MEF2A HOXD8 ZBTB7A ZNF217 NR1D2 ELF3 FOSL1 GLIS2 FOXN1 TP63 GTF2IRD2B PBX3 RFX4 PURB SKOR2 FOXE3 SUB1 PATZ1 SOX9 ELF1 TBX20 PITX3 FOXJ3 SCXB BATF2 MAFF SKOR1 FOXI1 SOX8 CC2D1B VAX1 DACH1 NEUROD2 ETV2 PRKRIR HINFP MSX2 MYBL1 ONECUT1 BMPR1A FLI1 RAD21 ZNF641 E2F8 ZNF750 ELF5 NDN ARID3A NR3C1 NKX3-2 CREBBP ZBTB4 NR6A1 SNAI1 HNF1A SNAI3 SP2 ZNF770 LMO2 ETS2 GCM1 NFIC SP4 FOXL2 ZMYM2 AR MAFB TGIF1 NPAS4 CSRNP3 NEUROD6 ZNF446 ETV6 RFX3 CRX MYF5 FOXB2 EAF2 ETV3L HNF4A ZNF639 NR1I2 MLX HEY1 FOXK1 SIX6 PLSCR1 ZNF280B FOXF2 SKI PHOX2B RORA HOXA7 TSHZ3 FOXP2 FOXD4L5 LYL1 ATF5 MLXIPL SOX21 HCFC1 JARID2 RAX HES6 NFYC STAT5B POU4F2 CEBPG EPM2AIP1 DDIT3 PLAGL2 CREB1 BACH1 ATF1 ELF4 MAFK HLTF C5orf54 PROX2 ARID3B TEF NHLH2 FOXJ2 ZNF444 NPAS1 ZNF274 CAMTA1 FOXD4L4 FOXI2 JUNB SCAND3 NR5A1 OTX2 ZBTB7B FOXM1 ARNTL2 NFIX GCFC1 SP3 GLI3 ZFPM2 ZNF434 PGR ZMYM4 GTF2IRD1 TEAD2 ZNF280C BSX ZSCAN5B TCF21 NFE2L2 SIX1 TLX1 FIGLA PRDM5 PBX1 GATA1 BATF ESR1 MED1 TFAP2B PPARG JUN PAX7 FOXH1 FEZF2 SMAD2 ZSCAN18 FOXD2 XBP1 NUCKS1 ZKSCAN2 SMAD3 NOBOX ELF2 SP1 FEV NR1I3 EGR1 TWIST1 MYOD1 FOXD1 HLF PCBP1

GO_SERINE_TYPE_ENDOPEPTIDASE_INHIBITOR_ACTIVITY Stops, prevents or reduces the activity of serine-type endopeptidases, enzymes that catalyze the hydrolysis of nonterminal peptide bonds in a polypeptide chain; a serine residue (and a histidine residue) are at the active center of the enzyme. KAL1 WFDC1 ITIH4 SPOCK1 SERPINB9 SPINK13 SPINT3 SERPINB2 PEBP1 SERPINB10 WFDC13 SPINK1 SERPINB13 SPINT1 CD109 CPAMD8 ITIH1 SERPINA1 APP SPINT4 SERPINB7 SERPINE1 SERPINF2 SERPINB5 WFIKKN2 SERPINC1 WFDC5 SERPINB12 HMSD WFDC8 SERPINA10 SPINK2 A2M SERPINB1 SERPINB6 TFPI SERPINA5 SERPINF1 SERPINI2 APLP2 SERPINB11 SPINLW1 WFDC2 A2ML1 SLPI SERPINI1 SERPINA2 WFDC6 SPINK4 ITIH3 SPINK7 SERPINB8 PZP PCSK1N SPINK14 PI3 PAPLN SPINK9 AGT ITIH6 FURIN RECK WFDC10A SPINT2 AMBP SPINK8 WFDC3 SERPINA9 OVOS2 SERPINE2 SERPINA4 SERPIND1 ITIH2 SERPING1 SERPINA12 SERPINA7 HRG WFDC12 CRIM1 SERPINA11 SERPINB3 TFPI2 ITIH5 COL6A3 WFIKKN1 SERPINA3 OVOS SERPINA6 SPINK6 SPINK5 COL28A1 SERPINE3 SERPINB4 SERPINH1 COL7A1

GO_OXIDOREDUCTASE_ACTIVITY_ACTING_ON_A_SULFUR_GROUP_OF_DONORS_NAD_P_AS_ACCEPTOR Catalysis of an oxidation-reduction (redox) reaction in which a sulfur-containing group acts as a hydrogen or electron donor and reduces NAD or NADP. TXNRD2 TXNDC2 TXNRD3 NXN TXNDC17 TXNRD1 NXNL1 DLD NXNL2 PRDX3 GSR

GO_CYTOKINE_BINDING Interacting selectively and non-covalently with a cytokine, any of a group of proteins that function to control the survival, growth and differentiation of tissues and cells, and which have autocrine and paracrine activity. CNTFR CCR6 VASN IFNAR1 ITGA4 GBP1 CCBP2 LTBP3 IL1RN CXCR7 PLP2 CXCR1 CCRL1 CCR5 IL9R IL17F IL1R2 IL6R TGFBR3 IL22RA1 CCR7 HYAL2 TGFB3 IL6ST CX3CR1 IL36RN IFNGR1 IL1RAPL1 IL2RG CXCR2 CD74 IL18BP CD36 NBL1 PARK7 ITGB3 LTBP4 BMPR2 TNFRSF9 IL12A IL10RA ACVR1 IL22RA2 IL2RB LTBP1 IL23R HMGB1 ITGB1 ACVRL1 PXDN DARC IL20RA CXCR3 CD109 CCR1 NRP2 TGFBR2 IL12RB1 GREM1 FZD4 KIT IL12B CSF1R KLHL20 ZFP36 HFE2 CHRD THBS1 NRP1 IL2RA IL31RA TCAP GREM2 IL20RB TGFBR1 ELANE CRLF1 CER1 TRIM16 IL1R1 IFNAR2 CXCR4 HAX1 IL1RL1 ITGAV ENG NOG TNFRSF1A GDF5 CXCR6 A2M TNFRSF11A

GO_NEUTRAL_AMINO_ACID_TRANSMEMBRANE_TRANSPORTER_ACTIVITY Catalysis of the transfer of neutral amino acids from one side of a membrane to the other. Neutral amino acids have a pH of 7. SLC7A5 SLC7A9 SLC38A3 SLC7A8 SLC6A5 SLC7A13 SLC6A19 SLC6A15 SLC36A3 SERINC5 SLC43A1 SLC38A7 SERINC1 SLC1A4 SLC32A1 SLC38A5 SLC6A9 SLC43A2 SLC36A2 SERINC2 SERINC4 SLC6A20 SLC38A1 SLC1A1 SLC3A2 SLC6A7 SLC7A10 SERINC3 SLC36A1 SLC1A5 SLC7A11 SLC36A4

GO_SODIUM_CHANNEL_REGULATOR_ACTIVITY Modulates the activity of a sodium channel. NEDD4 ATP2B4 CAV3 NEDD4L SGK1 NOS1 PTPN3 SGK3 SCN1B FXYD5 FXYD2 PKP2 FGF13 SCN3B C8orf44-SGK3 GPLD1 FXYD3 SNTA1 RANGRF SCN2B TMPRSS3 GPD1L CAMK2D SGK2 FGF12 SCN4B PRSS41 PRSS8 PCSK9 FXYD1 YWHAH SCLT1 FXYD4

GO_ANION_BINDING Interacting selectively and non-covalently with anions, charged atoms or groups of atoms with a net negative charge. P4HTM EGLN3 NCF1C MCCC1 PLOD3 UBR2 P4HA1 EGLN2 MTHFD2 AGRN NOS3 GLRB SESN2 UGT1A7 TPH1 AGXT2L1 GLRA3 GATSL2 GOT2 GOT1 GPT2 PDXDC2P FABP1 DPYS HDC MICALL1 P4HA3 GATSL3 CYP26C1 AGXT CLCN6 GPT PACSIN2 FABP5 PDXK DAPP1 AGXT2 FCN1 UGT2B17 SDSL LEPREL2 KIF16B WDR45L SPTLC1 KARS SERPINA5 CRABP1 PLOD1 PYGM SPTLC2 PAH GCAT CCBL2 ADAP2 PCCA TDO2 HNF4A P4HA2 PPARD NLGN4X SHMT2 FOLR2 TAT HMGCL SLC22A6 STARD5 G6PC RELA LCN12 RPH3A FABP6 WDR45 C17orf101 GRIN1 AMY2A ACE CYP26B1 ADSS NOS1 SELE SRR GNG12 LEPRE1 SDS SESN1 OTC AADAT GLDC RS1 YWHAE S100A9 PLEK SELP DDAH1 FOLR1 GLUL GADL1 CCBL1 CYP26A1 SGPL1 UGT2B7 OGFOD2 GRIN3A UGT1A9 GLRA2 PPARG GRIN2B UGT1A8 CLCN7 NAGS DHFRP1 CTH DHFR ST8SIA3 GAD2 UGT1A4 GCHFR NCF1 SNX14 PLEKHA1 MAPKAP1 JPH2 LEO1 APOC1 NFS1 PLCD1 C11orf83 SESTD1 PC PMP2 GSS GAD1 CLCNKB UGT1A1 BCAT1 MOCOS CHST14 SLC1A1 SPTLC3 GRHPR GLUD1 CLCN5 PLA2G1B NDUFAB1 NR2F2 ADIPOQ ST8SIA2 EGLN1 SNX3 TMLHE NEDD4 GNMT GCLC PIN1 S100A8 SCP2 ALOX5AP ACCSL TTPA PITPNC1 ARAP3 ADH5 PHYH GLUD2 CLVS1 ACCS TH NRGN NOS2 NR1H4 ALB CTSC RAG2 PDXDC1 CLCN2 STX3 SLC1A3 UBR1 OGFOD1 CLCN1 CRABP2 SLC34A2 PNP TPH2 ALKBH3 UGT1A6 FABP4 SHMT1 UGT1A3 NCF1B WIPI1 MYO1G FABP2 CAD PROSC AGXT2L2 SH3PXD2B PHLDA3 NME2 ANXA8 ZFYVE1 GOT1L1 RARA PLEKHA5 THNSL1 AKT1 CPS1 ACACB ALAS2 PCK1 ST8SIA4 MARK1 PITPNM1 RAPGEF6 ADCY10 UGT1A10 DBH DDAH2 GSDMD MOSC2 UGT2B4 ALAS1 RARS OAT ABAT CD34 FABP3 MOSC1 PAM ADSSL1 HRSP12 GLRA4 IGF2R CLCN4 AARS OXER1 AARS2 LRAT CLVS2 COMMD1 WIPI2 DDC RAPGEF2 UGT2B15 CLCN3 THNSL2 YWHAB PYGL PYGB CSAD GLRA1 GBF1 CLCNKA KYNU O3FAR1 PTGDS SNCA ASS1 BCAT2 HIF1AN LEPREL1 HLCS AKR1C2 FAM21C CBS AKR1C1 GAB2 ATP13A2 GRIN3B PLOD2 CEP104

GO_TRNA_BINDING Interacting selectively and non-covalently with transfer RNA. FARS2 YRDC PTCD1 SLFN11 AIMP1 EIF2A TRMT1 YARS SEPSECS RARS DARS2 TRNT1 EIF2AK4 EEFSEC TRMU PSTK IGHMBP2 NSUN2 METTL1 TYW5 THUMPD3 SSB KIAA1456 TERT AARS2 EEF1A1 AARS AARSD1 TRMT11 EARS2 FDXACB1 NSUN6 IFIT5 IARS2 FARSA ALKBH8 CARS XPOT THG1L XPO5 THUMPD2 IARS TRMT1L YARS2 RG9MTD2 KARS CTU2 MARS CTU1 RPL35A

GO_PASSIVE_TRANSMEMBRANE_TRANSPORTER_ACTIVITY Enables the transfer of a solute from one side of the membrane to the other, down the solute's concentration gradient. FXYD2 TTYH1 CCT8L2 MCU NCS1 TRPM4 CALM2 FXYD3 KCNA2 FAM26E VDAC3 GRIN1 TOMM20 CACNA1D NALCN CLIC3 C9orf7 NOX5 KCNH6 KCNT2 SLC26A6 KCNN4 GABRR2 PKD1L3 KCNAB1 GRIA1 HTR3C SCN4B KCNE4 ACCN4 KCNJ6 GJD3 HTR3D KCNK7 VDAC1 ANO1 CHRNA6 GABRG3 IL1RAPL1 DLG3 KCNH1 KCNE1 RYR1 PANX2 FXYD7 SCN1A KCNJ15 KCNH7 GRIK1 TRPC4AP SCNN1G KCNN1 GRIN2B GRIN3A GLRA2 CLCN7 SCN11A ANO6 HPN SLC26A10 TOMM20L GJA5 GRIA2 FAM26D GJA8 KCNK10 CACNG2 TRPV4 FAM26F BEST1 GABRR1 KCNA10 GABRB2 TMC1 CACNA1F KCNH5 GABRA5 CALHM2 KCNQ2 CACNA1C CATSPER1 JPH2 PDE2A AQP12B TRPV5 KCNJ18 SLC9A10 PANX3 PDPN KCNE3 TRPV6 TPCN2 GABRG2 KCNB1 KCND1 CLCA3P KCNJ2 SLC24A5 CHRNE TRPM8 FXYD6P3 CLCNKB CATSPER4 GABRD HCN4 ANO10 CACNG6 GABRA1 HTR3E SCN3A CHRNG ACCN5 KCNK16 ITPR3 GJB3 GRIK4 GABRA6 BCL2A1 KCNA3 KCNQ4 TOMM40 CLCA4 ACCN1 GRIN2C PKD1 SLC26A3 P2RX7 CCDC109B GRIA4 CHRNA10 GLRB BEST2 PTK2B KCNG3 KCNG4 GLRA3 SCNN1A TIMM23 CNGA3 AQP8 CHRNB1 CLIC6 KCNK9 P2RX2 GAR1 CFTR PIEZO2 BEST3 KCNJ3 HTR3A KCNK17 SCNN1B FXYD1 STIM2 CHRNA7 CYBB FXYD4 KCNMB1 ORAI2 ZP3 JPH3 LRRC8E SCN2B HCN2 TMEM38A KCNQ1 MCOLN3 HCN3 CLIC4 CLCN6 TRPV2 CACNA1B TIMM17B CLDN17 TRPM3 ITPR1 SLC26A7 ANO7 ANO3 SLC14A1 HVCN1 CACNA1S AQP1 SCN1B HTR3B CACNA1A TOMM40L PANX1 GRID1 KCND3 RYR3 SCN3B DLG1 SCN4A TMEM175 STEAP1 CLCA2 GRM7 KCNAB3 TMEM37 DLG4 TMEM38B MCOLN1 SLC26A8 KCND2 MAL CACNA1E AQP2 KCNC4 GAS6 GABRA4 SCN8A KCNK12 AQP6 GABRA3 KCNK15 CHRNB3 ABCC8 CNGA1 AQP12A GABRB3 BEST4 CLIC2 MCL1 GABRQ SCN5A KCNG2 CACNA1H LOC100652748 GJB1 FKBP1B AQP11 SLC24A2 CATSPER3 TTYH2 SCNN1D TRPV1 CACNA1G KCNH8 KCNS2 AQP3 SLC14A2 SLC24A1 KCNC3 GPR89A CNGB1 CACNG4 KCNT1 TRPC1 SHROOM2 ACCN2 SLC4A11 TRPC4 DENND5A REST CNGA2 PRF1 KCNMA1 VDAC2 SCN7A KCNK6 PKD2 CALM1 KCNF1 KCNC2 GRID2 GPR89B KCNC1 SLC1A4 KCNH3 TRPC6 ORAI3 KCNA5 TRPM7 KCNK13 CACNA1I P2RX6 HCN1 RHAG PKD2L2 KCNN3 GLRA4 KCNIP1 ACCN3 CACNA2D3 ANXA6 TRPA1 CACNA2D2 KCNJ5 CLCN4 GJA10 GRIN2D FXYD5 TIMM17A SCN2A CHRNB4 ANO5 KCNH2 BSND FXYD6 KCNJ16 P2RX5 KCNE2 TMEM109 LRRC8A CACNG5 NCALD ITGAV P2RX3 GABRR3 ST20 KCNJ4 ITPR2 TMEM63C ANO9 CLCN3 CLCA1 KCNK3 KCNE1L PKD2L1 MTMR6 AQP10 AQP9 GJC1 CHRNA4 GABRE KCNMB4 AQP7P3 KCNMB2 SCN10A GLRA1 P2RX4 LRRC8C CLIC1 CACNA2D4 AQP5 CLCNKA CACNB2 ANO2 SCN9A CHRND TRPM2 KCNB2 MCOLN2 CATSPER2 TRPM6 SLC17A3 CALHM3 PSEN1 KCNQ3 BAX KCNK4 GRIN3B TRPC3 TRPM1 ANO8 CNGA4 KCNJ13 CLCC1 CACNG8 CLCN5 ORAI1 KCNIP2 GJA3 BCL2 TMC2 KCNK1 PKD1L1 KCNH4 KCNJ12 NOX1 KCNJ9 GRIN2A GPM6A KCNQ5 GABRA2 SLC40A1 KCNK5 TMCO1 KCNG1 CNGB3 TRPC5 CHRNA1 AQP7 TRPV3 CACNA2D1 KCNS3 NMUR2 CACNB4 CHRNA3 KCNIP4 CHRNA2 CLIC5 KCNN2 GJD2 DENND5B OPRM1 TRPC7 SLC26A4 CACNB1 KCNJ11 SLC26A1 CACNG1 GJB2 TOMM22 CLCN2 RYR2 KCNA7 TPCN1 GRIA3 KCNJ10 LOXHD1 KCNV2 CLCN1 CACNG3 SLC17A7 APOL1 GRIK2 CHRNA5 SLC26A9 SLC26A11 CALHM1 CHRFAM7A KCNA6 MIP KCNAB2 SLC26A5 LRRC8D SLC24A4 CUL5 KCNA4 RASA3 ANO4 PKDREJ CHRNA9 KCNK18 CHRNB2 CACNB3 KCNV1 GJA1 KCNJ14 KCNMB3 GRIK3 P2RX1 GJC2 GRIK5 TIMM22 GABRG1 ABCC9 KCNK2 GABRB1 KCNS1 CACNG7 KCNIP3 KCNU1 GABRP SLC5A8 KCNJ1 SLC26A2 TTYH3 TRPM5 CALM3 KCNJ8 KCNA1 ZACN AQP4 PIEZO1 SLC24A3 TOMM7 PKD1L2

GO_CADHERIN_BINDING Interacting selectively and non-covalently with cadherin, a type I membrane protein involved in cell adhesion. PACSIN2 DOCK9 TJP1 SH3GL1 CD2AP EEF1D S100P ALDOA CDK5R1 NUMB CAPZA1 ANXA2 AHSA1 MICALL1 APC PKM2 SLC9A3R2 HIST1H3C TAGLN2 ADD1 LARP1 CCT8 MB21D2 RANBP1 SPTBN1 STK24 SH3GLB2 DDX6 RUVBL1 TNKS1BP1 CTNNA3 HDLBP PTPRT SDCBP CHMP5 NDRG1 COBLL1 NUDC SNX1 CTNND1 WASF2 CAPG NOP56 HSPA5 BSG LRRFIP1 DBN1 RAB10 HCFC1 HIST1H3F GCN1L1 F11R FLNA HIST1H3I EIF4G1 EPS15 TBC1D2 CDC42EP1 SNX5 KIF5B SH3GLB1 VAPB PLCB3 CAST PAICS UNC45A FLNB SPTAN1 TRIM25 PLIN3 PKP2 DLG1 UBAP2 CSNK1D CAPZB LASP1 LDHA UBFD1 EPS8L1 CCS RPL34 PKN2 PUF60 SLK EMD EPS15L1 EPCAM YWHAE IST1 PPME1 PAK2 USP8 EHD1 CALD1 NOTCH3 CTNNA2 ARHGAP18 MARK2 DNAJB1 PI4KA CLINT1 BAG3 MYH9 PROM1 DIAPH3 CRKL CD46 RPS26 RDX DDX3X PARK7 CDH1 RPL15 BAIAP2L1 ZC3H15 RPL24 PDLIM1 HNRNPK MRE11A ARGLU1 PCBP1 SCYL1 EIF5 S100A11 TMEM2 ATIC NCK1 ARHGAP1 MPP7 ERC1 PTPRM VAPA PAK4 CTTN TES EZR EFHD2 BAIAP2 ESYT2 EXOC3 CBL MLLT4 RPL14 PDXDC1 TBC1D10A HIST1H3E TXNDC9 PAK6 RAN TRIM29 CKAP5 PFKP PRDX6 PPP1R13L RPS2 PRDX1 SEPT7 ANXA1 VASP USO1 ARFIP1 MACF1 HSPA8 HIST1H3G EPS8L2 GNB2L1 HIST1H3B PSMB6 GIPC1 RAB1A ANLN HIST1H3D KTN1 CORO1B IQGAP1 SEPT9 ARFIP2 TMPO AHNAK PICALM FSCN1 HSP90AB1 HIST1H3A MAPRE1 PARVA CNN2 SERBP1 PKP3 GLOD4 GIGYF2 SWAP70 KRT18 EPN2 CTNNB1 SCRIB HSPA1A H1FX STK38 PLEC GOLGA2 ITGB1 SND1 KIAA1524 ARHGEF16 CCNB2 EEF1G ATXN2L RANGAP1 YKT6 DBNL HIST1H3J RSL1D1 CHMP2B PPL GPRC5A ITGA6 MPRIP STX5 MMP24 LIMA1 MYO1B TWF2 FNBP1L EGFR TMOD3 PCMT1 ASAP1 RAB11B OLA1 RARS IDH1 ABCF3 SRC EIF2A RPL7A BZW2 VASN EHD4 MYO6 BZW1 TRPC4 KIAA1598 FMNL2 EEF2 EPB41L1 KLC2 ENO1 EIF2S3 LRRC59 EIF3E STXBP6 STAT1 EVPL EPHA2 SLC3A2 SEPT2 GOLGA3 CDH13 JUP TWF1 DAB2IP GAPVD1 CNN3 TLN1 PTPN1 PHLDB2 PFN1 OLFM4 HIST1H3H CHMP4B PPFIBP1 PSEN1 SPTBN2 EIF4H YWHAZ RPL29 ANK3 CC2D1A ABI1 CGN VCL ZC3HAV1 P2RX4 FASN CLIC1 FAM129B CTNNA1 SFN SNX2 EIF4G2 RPL6 LAD1 YWHAB DHX29 TJP2 RTN4 MKL2 CTNNAL1 RPL23A SNX9 PDLIM5 FXYD5 LYPLA2

GO_INSULIN_RECEPTOR_SUBSTRATE_BINDING Interacting selectively and non-covalently with any of the insulin receptor substrate (IRS) proteins, adaptor proteins that bind to the transphosphorylated insulin and insulin-like growth factor receptors, are themselves phosphorylated and in turn recruit SH2 domain-containing signaling molecules to form a productive signaling complex. GRB2 PTPN11 IGF1R PIK3CA PIK3CB JAK2 PRKCZ PIK3R1 PRKCD INSRR INSR

GO_FLAVIN_ADENINE_DINUCLEOTIDE_BINDING Interacting selectively and non-covalently with FAD, flavin-adenine dinucleotide, the coenzyme or the prosthetic group of various flavoprotein oxidoreductase enzymes, in either the oxidized form, FAD, or the reduced form, FADH2. OXNAD1 CYB5R2 ACADM CRY2 NOX5 NOS3 ACADSB CYB5R3 DHCR24 TXNRD1 ACAD9 CYB5R4 KDM1A NOS1 FMO5 ETFA MTHFR LDHD MMACHC ACAD10 MAOB DUS3L KMO SDHA GCDH ACADL PPOX ACADVL ETFDH ACOX1 ACOX2 XDH DUS1L DDO DUS4L PRODH CYBB ACOXL D2HGDH POR FMO3 NOS2 FMO2 GSR KDM1B IVD CYB5RL NDOR1 AOX1 COQ6 MICAL2 FOXRED2 ACOX3 AIFM3 TXNRD3 ACAD8 DLD AGPS MTO1 TXNRD2 ACAD11 AIFM1 FMO4 ACADS MICAL1 NOX4 MTRR AIFM2 CHDH DAO CYB5R1 DUS2L FMO1 DPYD GFER MICAL3 SQLE

GO_WNT_PROTEIN_BINDING Interacting selectively and non-covalently with Wnt-protein, a secreted growth factor involved in signaling. LOC388630 ROR2 EGF FZD4 CTHRC1 FZD10 SFRP5 ROR1 RYK FZD6 FRZB FZD3 WLS SFRP2 SMO SFRP1 LRP5L FZD2 PTPRO PORCN FZD8 FZD5 LRP6 WIF1 LRP5 FZD7 APCDD1 FZD1 SFRP4 FZD9 C2orf89

GO_COFACTOR_TRANSPORTER_ACTIVITY Enables the directed movement of a cofactor into, out of or within a cell, or between cells. A cofactor is a substance that is required for the activity of an enzyme or other protein. SLC19A2 ABCB6 PDPN SLC6A8 SLC25A17 MFSD3 SLC16A12 ABCB7 SLC25A26 SLC33A1 HPX FOLR1 FOLR2 SLC48A1 FLVCR1 SLC25A42 SLC46A1 SLC19A1 SLC25A32 ABCG2 FLVCR2

GO_CAMP_RESPONSE_ELEMENT_BINDING Interacting selectively and non-covalently with the cyclic AMP response element (CRE), a short palindrome-containing sequence found in the promoters of genes whose expression is regulated in response to cyclic AMP. ATF6B E4F1 ATF6 HMGA2 TCF12 CREB3L4 CREB1 CREB3L2 CREB3L1 JUN CREB3L3 CREB3 ATF2

GO_PROTON_TRANSPORTING_ATPASE_ACTIVITY_ROTATIONAL_MECHANISM Catalysis of the transfer of protons from one side of a membrane to the other according to the reaction: ATP + H2O + H+(in) = ADP + phosphate + H+(out), by a rotational mechanism. ATP5B ATP5E ATP6V0E1 ATP6V1H TCIRG1 ATP6V1B2 ATP6V1E1 ATP5D ATP6V1C1 ATP6V1F ATP6V1A ATP6V0B ATP5A1 ATP6AP1L ATP6AP1 ATP5EP2 ATP6V0A4 ATP6V1E2 ATP6V0A2 ATP6V0C ATP6V0A1 ATP6V0E2 ATP5C1

GO_MRNA_3_UTR_BINDING Interacting selectively and non-covalently with the 3' untranslated region of an mRNA molecule. ZC3H12A AUH CIRBP PABPC1 CSDA ZNF385A CPSF1 PCBP4 FXR1 CPEB4 HNRNPR DAZ3 PUM2 HNRNPA2B1 RNPS1 DAZ1 IGF2BP3 HNRNPC LARP1 PUM1 CARHSP1 RC3H1 RBMS3 KHSRP HNRNPD SECISBP2L ZFP36L2 IGF2BP2 SECISBP2 TARDBP ELAVL2 PARN IGF2BP1 CPEB3 CPEB1 RBM4 TUT1 CRYZ FMR1 ELAVL1 RNF40 CPEB2 SERBP1 RNF20 ANGEL2 ELAVL4 ZFP36 RBM24 ZFP36L1 DAZL BOLL

GO_GLYCOPROTEIN_BINDING Interacting selectively and non-covalently with a glycoprotein, a protein that contains covalently bound glycose (monosaccharide) residues. These also include proteoglycans. TGFB1 APOH AGR3 CLASP2 SDC1 FOXRED2 FBXO2 CSNK1D IDE GPC6 TFR2 VIM STX1A DAG1 PTPRF FST SHB COL5A1 RASA1 RGMA GPC5 CTSK SERPINA1 LCK LRRK2 FLNA PIP VLDLR GPC4 GPC2 BMPR1B AGR2 OS9 COMP VCL ATP1A3 TFRC VWF CECR1 CNTN2 B2M HPSE HFE2 THBS1 BBC3 CTSS LACRT HSPA5 CD4 PLAT CTSB FBXO6 ITGA3 CNTN1 NID1 DNAJC5 CSNK1G2 HSP90AB1 SDCBP CDH1 SEMA5A F7 ITGAM SLIT2 FBXO17 DMD CFH LDLR MAP2 LMAN2 CANX PTN FCN2 LGALS1 AGRN COL5A3 FBXO27 HPSE2 FGF20 AZU1 HRG CALR PLA2G2D FYN EDEM2 GPC1 EDEM1 ACE2 ITGB2 SHH ERLEC1 BMPR1A GFAP SELP GPC3 IGF2R EGFR CTSL1 AZGP1 HSP90AA1 CLASP1

GO_INTERLEUKIN_1_RECEPTOR_BINDING Interacting selectively and non-covalently with the interleukin-1 receptor. IL1RN IL36G IL1A TLR5 IL36RN ERAP1 IL37 IL36A TLR9 IRAK4 IL36B IL1F10 IL1B TRIP6 TOLLIP

GO_OXIDOREDUCTASE_ACTIVITY_ACTING_ON_THE_CH_CH_GROUP_OF_DONORS Catalysis of an oxidation-reduction (redox) reaction in which a CH-CH group acts as a hydrogen or electron donor and reduces a hydrogen or electron acceptor. SRD5A2 DUS4L DUS2L SDHC BDH2 FASN TM7SF2 PTGR1 DUS1L DHDH RSAD1 ACOXL DHODH DPYD ACADL CPOX ACADS GCDH ACAD11 ACOX2 AKR1D1 ACOX1 SDHD AKR1C3 AKR1C2 ACADVL AKR1C1 TECR PPOX ACOX3 BLVRB COX15 BLVRA ETFA DHCR7 TECRL SRD5A1 ACAA1 DUS3L SDHA DECR2 TBXAS1 ACAD8 SRD5A3 LBR ACAD10 RETSAT MECR ACADSB PECR PTGR2 IVD ACADM DECR1 ACAD9 SDHB DHCR24

GO_HYDROLASE_ACTIVITY_ACTING_ON_CARBON_NITROGEN_BUT_NOT_PEPTIDE_BONDS_IN_LINEAR_AMIDES Catalysis of the hydrolysis of any non-peptide carbon-nitrogen bond in a linear amide. RCOR1 SALL1 AGA HDAC10 ARID4B PIGL VNN2 MTA2 ACR GLS2 HDAC3 MTA1 NAALAD2 FAAH2 SIRT6 ACER3 ASAH1 ASPG HDAC8 UPB1 HDAC9 SIRT4 PHF21A HDAC1 KLK3 FAAH DARS BTD SIN3B ACY3 SIRT7 PGLYRP2 HDAC4 MBD3 WDYHV1 BRMS1L SAP18 NADSYN1 GLS ARID4A ASRGL1 ACY1 SUDS3 AMDHD2 NIT2 RBBP7 CAT CHD3 HDAC7 CHD4 SIRT3 SIRT2 RBBP4 ACER2 HDAC6 NTAN1 PGLYRP3 HDAC2 BRMS1 VNN3 ACER1 PGLYRP1 REST NDST1 PGLYRP4 ASAH2 NDST2 SAP30L KDM1A HDAC11 HMG20B NGLY1 AFMID MTA3 VNN1 NACC2 HDAC5 SIN3A SIRT1 PDF SIRT5 ASPA SAP30

GO_ADRENERGIC_RECEPTOR_ACTIVITY Combining with epinephrine or norepinephrine and transmitting the signal across the membrane by activating the alpha-subunit of an associated heterotrimeric G-protein complex. GPR101 OR13F1 ADRA2C ADRB1 ADRA1D ADRA2B ADRA1B DRD2 OR56A4 ADRB2 ADRA1A ADRA2A OR5T1 ADRB3 OR56A1 OR56A5

GO_POTASSIUM_ION_TRANSMEMBRANE_TRANSPORTER_ACTIVITY Enables the transfer of potassium ions (K+) from one side of a membrane to the other. PKD2 KCNF1 KCNC2 KCNE4 KCNC1 KCNH3 ATP1B2 KCNA5 KCNJ6 ATP1A2 KCNK7 KCNK13 HCN1 SLC9A3 KCNN3 SLC12A9 KCNH1 KCNIP1 KCNE1 ATP1B1 KCNG2 FXYD2 CCT8L2 ATP1A4 ATP4A SLC24A2 KCNA2 KCNH8 KCNS2 SLC24A1 KCNC3 CNGB1 KCNT1 SLC12A6 KCNT2 KCNH6 KCNN4 SLC9A1 REST CNGA2 KCNAB1 KCNMA1 KCNK6 KCNA10 SLC9A2 ATP1A3 KCNH5 KCNQ2 KCNJ18 SLC9A10 KCNB2 SLC9A5 KCNE3 KCNB1 KCND1 KCNJ2 SLC24A5 KCNQ3 KCNK4 SLC12A7 HCN4 CNGA4 KCNJ13 KCNJ5 KCNJ15 KCNH7 SLC9A8 KCNH2 KCNJ16 KCNE2 KCNN1 HPN KCNJ4 KCNK3 KCNE1L PKD2L1 MTMR6 KCNK10 KCNMB4 KCNMB2 KCNK9 KCNG1 CNGB3 KCNJ3 KCNK17 KCNS3 KCNIP4 FXYD4 SLC9A11 KCNN2 KCNMB1 HCN2 SLC12A5 KCNJ11 KCNQ1 TMEM38A ATP1B3 SLC9A7 HCN3 ATP1A1 SLC9A6 ATP4B KCNK16 KCNIP2 KCNA3 KCNQ4 KCNK1 KCNH4 KCNJ12 KCNJ9 KCNQ5 KCNG3 KCNG4 CNGA3 KCNK5 KCNV1 KCNJ14 KCNMB3 KCNK2 ABCC9 KCNS1 KCNK12 KCNIP3 KCNK15 ABCC8 KCNU1 CNGA1 KCNJ1 TRPM5 KCNA1 KCNJ8 SLC24A3 KCNA7 KCNJ10 KCNV2 AQP1 SLC9A4 KCND3 SLC12A4 SLC9A9 TMEM175 KCNA6 KCNAB2 KCNAB3 SLC24A4 TMEM38B KCNA4 KCND2 ATP12A KCNK18 KCNC4

GO_PROTEASOME_BINDING Interacting selectively and non-covalently with a proteasome, a large multisubunit protein complex that catalyzes protein degradation. BAG6 ID1 SACS USP13 UCHL5 ADRM1 PSMG1 PSMF1 PSMD14 UBD USP14

GO_APOLIPOPROTEIN_BINDING Interacting selectively and non-covalently with an apolipoprotein, the protein component of a lipoprotein complex. MAPT LIPC LRP8 LRP1 LPL PCSK9 LRP4 LCAT PLG LPA LRP6 CANX ABCA1 VLDLR SCARB1

GO_STEROID_HORMONE_RECEPTOR_ACTIVITY Combining with a steroid hormone and transmitting the signal within the cell to initiate a change in cell activity or function. NKX3-1 PPARA NR1D1 VDR AR ABHD2 NR4A3 NR2C1 NR6A1 NR4A2 PGR PAQR7 ESRRB RXRB ESR1 PAQR9 NR2F2 THRB PPARG THRA NR0B1 NR2E1 NR0B2 NR2F6 RXRA NR1H3 NR4A1 NR5A1 RORC NR1D2 ESRRG RXRG OR51E2 HNF4G RARA HNF4A PAQR5 NR1H2 NR1I2 NR3C2 NR2F1 RARB PPARD RORA NR2E3 PGRMC2 RARG NR1H4 NR3C1 NR5A2 LEF1 RORB NR1I3 PAQR8 GPER ESR2 ESRRA PAQR6 NR2C2

GO_UDP_GLYCOSYLTRANSFERASE_ACTIVITY Catalysis of the transfer of a glycosyl group from a UDP-sugar to a small hydrophobic molecule. B4GALNT4 GCNT7 GXYLT2 UGT1A1 GCNT6 MGAT4B B3GAT3 PIGQ PIGC MGAT1 B3GALT4 GALNT1 GALNT9 UGCG B3GNT8 UGT3A1 GALNT11 UGT2B15 GBGT1 CHSY1 GALNTL6 CCDC126 UGT1A4 B3GNT4 B4GALNT1 B4GALT7 B3GNT3 C3orf64 MGAT5 GALNT2 XYLT2 UGT2B7 UGGT1 ABO UGT1A9 GALNT14 POGLUT1 UGT1A8 LALBA EXTL1 UGT2B10 GCNT3 ALG14 UGT8 GLT25D1 B4GALT1 A4GALT UGT2B4 POMGNT1 B4GALT6 UGT2B28 UGGT2 XXYLT1 UGT2B11 B3GAT2 HEXA UGT3A2 UGT1A10 GLT25D2 GALNT6 A3GALT2P B3GALNT1 OGT LFNG XYLT1 RFNG PIGH GCNT1 GALNT12 MGAT3 HEXB WDFY3 PIGP CHPF2 GALNT3 GALNT4 UGT1A5 B3GNT5 CHSY3 MGAT5B MGAT2 GCNT2 EXTL2 B3GALNT2 LARGE GYG2 B3GALT1 GYS2 GYG1 GALNTL1 GALNT8 UGT1A3 UGT2A1 GALNTL2 EXT2 ALG5 EXT1 GALNT13 HAS1 B3GALT6 EXTL3 UGT2B17 GALNT7 B3GNT2 CSGALNACT2 UGT1A6 GYLTL1B WBSCR17 UGT2A3 B4GALT3 B3GAT1 C3orf39 GXYLT1 MGAT4A B4GALNT3 HAS3 B4GALT5 GCNT4 B4GALNT2 HYAL1 GYS1 HAS2 GALNT10 CSGALNACT1 GALNT5 MGAT4C B3GALT2 B3GNT7 PIGA GALNTL4 CHPF UGT1A7 ALG13 MFNG B3GNT6 B3GNT1 B3GALT5 A4GNT PLOD3 B4GALT4 B4GALT2

GO_EXOPEPTIDASE_ACTIVITY Catalysis of the hydrolysis of a peptide bond not more than three residues from the N- or C-terminus of a polypeptide chain, in a reaction that requires a free N-terminal amino group, C-terminal carboxyl group or both. TPP1 ERAP1 TPP2 CPA5 PRSS16 NUDT16 APEH NAALAD2 BLMH NPEPL1 PRCP DPP10 AGBL4 DPP6 CPM CPE LOC440434 METAP1 PM20D2 FAM63A CPB2 XPNPEP3 PREP CNDP2 CPD MMP17 SCRN3 CPXM2 PEPD CPA1 DPP7 NAALADL1 GGH CTSH AEBP1 PREPL CPZ GGT5 CPA2 MMP14 UCHL1 METAP2 XPNPEP1 FOLH1 CPVL DPP8 LAP3 XPNPEP2 DNPEP FAM188A AGBL1 CPO ERAP2 ZMPSTE24 SCPEP1 HPN TRHDE PHEX AGBL5 AGTPBP1 MME BACE1 MMP15 C9orf3 SCRN2 CTSL2 DPEP1 AGBL2 GGT2 SCRN1 NPEPPS LNPEP TMEM27 ACE2 CPN1 F11 RNPEP ACY1 DPP9 CTSA AGBL3 ASRGL1 PGPEP1 AQPEP GGT6 ENPEP CPA4 PM20D1 CPA3 DPP3 CPA6 DPP4 LTA4H GGT1 CPB1 GGT7 FOLH1B RNPEPL1 FAM63B DPEP2 CPXM1 CNDP1 METAP1D FAP DPEP3 MMP16 GGT3P ACE PGCP ANPEP

GO_OUTWARD_RECTIFIER_POTASSIUM_CHANNEL_ACTIVITY Enables the transmembrane transfer of a potassium ion by an outwardly-rectifying voltage-gated channel. An outwardly rectifying current-voltage relation is one where at any given driving force the outward flow of K+ ions exceeds the inward flow for the opposite driving force. REST KCND2 KCND3 KCNA5 KCNA2 KCNA3 KCNIP2 KCNK2 KCNK18 KCND1 KCNQ1

GO_ENDORIBONUCLEASE_ACTIVITY Catalysis of the hydrolysis of ester linkages within ribonucleic acid by creating internal breaks. ERVK-6 TSEN54 RNASE8 DROSHA NOB1 SLFN14 LACTB2 ZC3H12A RNASEH2A EIF2C1 PLD6 POP5 CPSF4L RNASET2 POP4 APEX1 RPP25 CPSF3 DBR1 RPP40 EIF2C2 ZC3H3 RNASEH2C DGCR8 RCL1 CPSF4 FEN1 TSEN15 ERN1 RPP30 RPP21 LOC100133495 ENDOV ELAC1 ENDOU SMG6 RNASEH1 RNASE1 RPP38 RNASEH2B ERVK-10 RNASE4 KIAA0391 POP1 RNASEK ERVK-8 EDC3 RNASE2 RNASEL MRPL44 POP7 EXO1 ERVK-7 DICER1 HRSP12 TSEN34 TSEN2

GO_DISULFIDE_OXIDOREDUCTASE_ACTIVITY Catalysis of the reaction: substrate with reduced sulfide groups = substrate with oxidized disulfide bonds. GRXCR1 ERO1L TMX1 TXNDC2 GLRX GLRX2 PDIA2 STAB2 CCS TXNRD1 SH3BGRL3 PDIA3 GLRX3 TXNDC12 TXNDC8 ENOX2 TXN2 DNAJC10 TXNRD3 GSTK1 PTGES2 GSTO1 TXNL1 CHCHD4 STAB1 GSTO2 GSR ERO1LB GFER GLRX5 TXN

GO_CYSTEINE_TYPE_ENDOPEPTIDASE_REGULATOR_ACTIVITY_INVOLVED_IN_APOPTOTIC_PROCESS Modulates the activity of a cysteine-type endopeptidase involved in the apoptotic process. VIL1 TFAP2B CTSH NLRP12 PRDX5 TNFAIP8 MT3 BCL2L13 RPS6KA1 NKX3-1 NOD1 BAD CARD8 GNB2L1 FOXL2 CDKN1B XIAP SNCA CASP1 AVP PRDX3 RPS6KA3 LEF1 GAS6 BIRC8 NLRP1 APAF1 NOL3 DPEP1 PYCARD SERPINB9 CASP8AP2 CASP3 CD27 BIRC3 TNFSF14 ARRB1 RPS27L NGFRAP1 BIRC2

GO_SH2_DOMAIN_BINDING Interacting selectively and non-covalently with a SH2 domain (Src homology 2) of a protein, a protein domain of about 100 amino-acid residues and belonging to the alpha + beta domain class. DLC1 SRC SQSTM1 JAK2 PAG1 SIT1 NUP62 SH3PXD2B LCK KHDRBS2 ARHGAP5 AFAP1L2 LAT2 IRS1 LILRB1 SHCBP1 CTR9 CRK SYNGR3 DAG1 NLK INPPL1 PTK2 SYP TRPV4 LAX1 GNB2L1 SKAP1 PTPN6

GO_LOW_DENSITY_LIPOPROTEIN_PARTICLE_RECEPTOR_BINDING Interacting selectively and non-covalently with a low-density lipoprotein receptor. APOB LRPAP1 HSP90B1 AP2M1 APOE PCSK9 SNX17 SYT1 DNAJA1 LANCL1 CRP MESDC2 LDLRAP1 DKK1 APOA5

GO_RHO_GTPASE_BINDING Interacting selectively and non-covalently with Rho protein, any member of the Rho subfamily of the Ras superfamily of monomeric GTPases. Proteins in the Rho subfamily are involved in relaying signals from cell-surface receptors to the actin cytoskeleton. HACE1 RCC2 PAK3 CDC42EP2 SRGAP2 BRK1 NCF2 CDC42EP3 ROCK1 DVL3 PKN1 DVL2 NET1 CORO1C TNFAIP1 DAAM1 FMNL1 ARHGEF16 CDC42EP1 SRGAP3 FLNA NCKAP1 RAB7A RTKN WASF1 RALBP1 LRRK2 DOCK11 PFN1 CYFIP1 ARHGAP4 CAV1 CDC42EP5 SOD1 DVL1 CDC42BPB KIF3B DAAM2 WHAMM CIT ROCK2 CSDA DOCK4 AKAP13 ARHGEF2 ARFIP2 DIAPH3 STXBP6 IQGAP2 TRIOBP CDKL5 IQGAP1 C15orf62 FMNL3 NOX1 TIAM1 SRGAP1 INF2 FMNL2 RHOH KCTD13 ARHGDIB MTSS1L NOXA1 OCRL EPS8 DOCK7 IQGAP3 PARD6A ECT2 DIAPH2 MYO9B DAPK3 CDC42EP4 DIAPH1 ABI2 ITPKA SRGAP2P1 MAP3K11 EXOC1

GO_PROTEIN_SERINE_THREONINE_KINASE_INHIBITOR_ACTIVITY Stops, prevents or reduces the activity of a protein serine/threonine kinase. HEXIM1 CDKN1B PRKAR1A HEXIM2 SPRED2 CDKN2A PRKAR2B SPRED1 CDKN2D INCA1 WNK1 PKIA KAT2B PRKAR1B YWHAG CDKN1A CDKN2C PKIB SFN H2AFY SPRY2 PPP1R1B HSPB1 CDKN1C PRKAR2A PKIG PRKAG2 CIB1 CDKN2B

GO_TRANSITION_METAL_ION_TRANSMEMBRANE_TRANSPORTER_ACTIVITY Enables the transfer of transition metal ions from one side of a membrane to the other. A transition metal is an element whose atom has an incomplete d-subshell of extranuclear electrons, or which gives rise to a cation or cations with an incomplete d-subshell. Transition metals often have more than one valency state. Biologically relevant transition metals include vanadium, manganese, iron, copper, cobalt, nickel, molybdenum and silver. ATP7B SLC30A8 SLC39A3 TFRC SLC30A5 ZP3 SLC39A7 SLC39A8 SLC39A14 SLC39A10 SLC39A1 SLC30A1 SLC30A2 SLC31A2 ATP13A1 SLC39A6 SLC39A5 SLC31A1 SLC30A10 SLC30A4 SLC39A4 SLC39A2 SLC39A13 SLC25A37 TTYH1 SLC30A6 SLC39A11 ATP7A SLC25A28 SLC30A3 SLC11A1 ATOX1 SLC30A7 SLC39A12 TF ATP2C2 SLC40A1 SLC11A2 MCOLN1

GO_INORGANIC_ANION_TRANSMEMBRANE_TRANSPORTER_ACTIVITY Enables the transfer of inorganic anions from one side of a membrane to the other. Inorganic anions are atoms or small molecules with a negative charge which do not contain carbon in covalent linkage. BEST4 XPR1 CLIC2 SLC17A4 GABRQ GABRP SLC22A20 SLC17A1 SLC26A2 TTYH3 GABRB3 GABRG1 GABRB1 SLC22A25 AQP6 SLC37A4 SLC20A1 GABRA3 MFSD5 GABRA4 SLC26A8 ANO4 CLCA2 SLC26A5 SLC26A9 SLC26A11 ANKH SLC12A4 SLC13A1 CLDN17 SLC4A3 SLC26A7 ANO3 SLC34A2 ANO7 CLCN1 SLC17A7 APOL1 CLCN6 SLC17A2 SLC22A12 SLC4A2 CLCN2 SLC12A5 SLC26A4 SLC26A1 CLIC4 FXYD1 CLIC5 CFTR BEST3 SLC4A7 NMUR2 BEST2 GABRA2 GLRA3 SLC4A5 CLIC6 SLC26A3 SLC37A3 SLC22A8 GLRB ADAMTS8 CLCA4 SLC34A3 CLCC1 GABRA6 CLCN5 SLC34A1 SLC22A24 CLCNKB GABRD SLC12A7 ANO10 ANO8 SLC4A10 GABRA1 SLC12A1 SLC5A5 GABRG2 SLC17A3 CLCA3P ANO2 SLC22A9 SLC20A2 GLRA1 GABRB2 CLIC1 CLCNKA GABRA5 GABRE BEST1 GABRR1 ANO6 GABRR3 SLC26A10 SLC4A4 SLC12A3 CLCA1 ANO9 CLCN3 SLC4A8 SLC4A9 GLRA2 CLCN7 CLCN4 SLC4A1 ANO5 SLC12A2 BSND SLC12A9 SLC22A11 GLRA4 PCYOX1 ANO1 SLC22A10 GABRG3 SLC1A4 SLC37A1 ASNA1 SLC4A11 SLC26A6 GABRR2 CLIC3 SLC12A6 SLC22A6 TTYH2 SLC13A4 FXYD3 SLC37A2 TTYH1 SLC25A3

GO_TRANSCRIPTION_FACTOR_BINDING Interacting selectively and non-covalently with a transcription factor, any protein required to initiate or regulate transcription. RUNX2 NAB2 ASXL1 EP300 CREBBP TFAM TSC22D3 MAP3K10 AR KAT8 CAND2 ARNT MAFB GCM1 LMO2 DACT1 SOX17 TERT JUND SIK1 CNOT2 LHX3 ARNT2 FOXP3 APBB1 TP53 MLX TTC8 PPARD EPAS1 HNF4A KLF5 E2F5 PSMC3 PSMC1 HEY1 TAF1B EXOSC9 KCTD1 ID3 ATG7 PER1 MECP2 FOXF2 IFI27 NCOA3 SKI BCAS3 FLNA TCF12 UBA2 RNF4 MLXIPL BRD7 TRIM6 SMARCA4 SETD3 PSMC2 RORA ERCC3 HOXA7 CREM GPX3 SOX9 NR4A2 NCOA2 COMMD6 SIX3 RPTOR ID4 VDR POLR1E SCXB TBP PDX1 PPRC1 TBX20 ADD1 INSIG2 HMGB1 SOX8 HDAC5 MED24 HMGA1 SKOR1 CAMTA2 WFS1 MED16 ARHGEF2 MSX2 GMNN GATA6 PPID MED19 CDKN2A FHL2 PRKDC NRIP1 CBX5 ZEB1 GAS2L1 SIRT2 MTDH RPS3 PAX2 TOB2 HYAL1 PSMC6 CEBPB SMARCB1 PRDM16 PARD6A AIP C1QBP HNRNPD CDK5RAP3 BHLHE40 ZFPM2 MED4 DDX20 TBX3 APBB2 APBB3 SETD6 APEX1 SIX2 TCF21 HCLS1 NFIA PSMD10 NKX3-1 MAGEA2 CXXC5 BBS5 PRDM5 NFE2L2 ASCL1 YEATS2 FUS NR0B2 FIGLA NOC2L PPARG JUN FOXO4 BRF1 NAB1 HDAC4 ESR1 GATA1 CRTC3 MED1 CPNE1 XBP1 MED13 TAF9 CTBP1 TDG PDCD11 FOXH1 NFKBID SMAD2 SP1 PIAS2 NLK NFYB SMAD3 NUCKS1 JMJD1C RBPJ PSMA6 MYOD1 IKZF4 TWIST1 MTA1 E2F2 ATOH8 HDAC8 ACTB NUP62 EIF4E NKX2-5 DHX9 HEY2 HES6 CEBPG NFYC SMARCD3 BRMS1 BBS7 TRAPPC2 DDIT3 HDAC11 PRMT2 MED25 CREB1 MED12 DHX33 LRIF1 CHCHD2 TCF4 KLF4 CSDC2 TCF7L2 GSK3B TRIM32 BBS4 TRERF1 PARK7 MEF2C DDX3X MAFK RB1 NHLH2 THRA GATA2 PURA RELA PPARGC1A KAT6B HES2 MED30 E2F1 RBFOX2 KAT2A CSNK2B JUNB NR1H2 COMMD7 ATF7 TRIB1 MKKS KAT6A DR1 PIM1 KAT2B ERCC4 GCFC1 GSC NFATC4 SPEN NCOA6 PPP1R13B SOST RNF19A HAND1 SRI ANKRD1 SLC26A5 WWP2 PIAS1 GTF2F1 HDAC9 DNAJA1 TCP10L ELK1 AKAP8 HAND2 CRTC2 PRAME SRY THRAP3 RBL1 TAF9B HMGN3 TFDP2 TBX6 ISL1 ENPP2 NR0B1 BBS1 GABARAPL1 CENPF CTNNB1 SRF NBN BPTF USF1 NCOR1 GTF2A2 TAF1 ZGLP1 RARA MTOR BEX1 NFKB1 SOX10 ARNTL FOXO1 TRIM11 MED14 RNF222 RCOR2 MTA2 ID1 NKX2-2 CRTC1 TAF7 ESRRB MED17 ZMYND8 CEBPA ETS1 HMGA2 E2F4 PIK3R1 BCL3 AGTR2 FOXC1 PASD1 TLE4 MYC KDM1A TRIB2 GTF2A1 TCERG1 HEYL RXRA USF2 MYBBP1A ZNF541 OASL BHLHE41 TAL1 HES7 STK36 BCL2 HNRNPF CTBP2 NPM1 TEAD3 DACT2 TAF12 ARRB1 PPP1R13L FOXA1 HES1 NCOA1 NFATC1 NIF3L1 IGHMBP2 TBX5 HIF1A HES5 LEF1 EOMES GFI1B SPI1 SKIL NCOR2 ACTN4 USP7 PPARGC1B HDAC2 NR1H4 NFATC2 NAAA TLE1 MAPK9 TFDP1 UBE2I CHD6 SNF8 RARG TAF4B CDC5L HDAC1 GATA5 ANXA4 MEF2D HTT TBL1X NR4A3 IFI16 CDK9 PPARA SNW1 FOSB BCOR ZNHIT6 SUMO1 VHL PSMC4 ZNF516 STK4 MAGEA2B KAT5 DUSP26 PTPRN HES4 POU1F1 PSMD9 ATF2 ERCC1 MAPK1 DMAP1 HIPK2 MEF2A PSMC5 TRIP12 BBS2 TCF3 FOXA2 NFKBIA TAF1L RCOR1 HIF1AN CCNT2 HMGB2 STAT3 NOP58 MEIS2 NLRP3 TAF7L FOS SKOR2 PURB UBXN7 ZFPM1 HES3 CAND1 HDAC3 FBL GATA4 LDB1 EGR2 CIITA NEUROD1 CRY1 YWHAZ HHEX RNF25 PELP1 BBS10 DLL1 TNFRSF10A GTF2B KEAP1 GATA3 SMAD4 IRF4 RCOR3 PHF12 MAD2L2 NSD1 TMEM173 FOXO3 TRPS1 NR1D1 REST SORBS3 SIN3A TP53BP2 PAX6 SUFU RBBP8 FAM89B CCNT1 TP73 SIRT1 C14orf43 CCND1 ZNHIT3 AHR RARB ZBTB49 DAPK3 CNOT1 MED6 TAF11 MYOCD HDGF PARP1 MAPK14 TP53BP1 DRG1 FAF1 FOXA3 CREG1 ZNF703 T PITX1 RORB TRIP6 MDFI MDFIC CHD4 PBX2 HDAC7 PSMD4 BCL10 MIXL1 ATF4 CD34 DGKQ LMO4 ANKRD2 PITX2

GO_METALLOEXOPEPTIDASE_ACTIVITY Catalysis of the hydrolysis of a peptide bond not more than three residues from the N- or C-terminus of a polypeptide chain by a mechanism in which water acts as a nucleophile, one or two metal ions hold the water molecule in place, and charged amino acid side chains are ligands for the metal ions. C9orf3 MMP15 DPEP1 AGBL2 NPEPL1 CPA5 ERAP1 NUDT16 AGBL5 AGTPBP1 AGBL3 CNDP2 CPD CPB2 AQPEP MMP17 CPXM2 PEPD LNPEP AGBL4 ACE2 NPEPPS RNPEP CPM CPN1 METAP1 LOC440434 CPE ACY1 FOLH1B CPB1 LTA4H RNPEPL1 CPA2 MMP14 CPA1 ENPEP PM20D1 CPZ CPA4 AEBP1 CPA6 CPA3 ACE MMP16 DNPEP PGCP ANPEP XPNPEP2 LAP3 CPO AGBL1 ERAP2 TRHDE ZMPSTE24 METAP2 CPXM1 DPEP2 METAP1D CNDP1 DPEP3 XPNPEP1 FOLH1

GO_DNA_SECONDARY_STRUCTURE_BINDING Interacting selectively and non-covalently with DNA containing secondary structure elements such as four-way junctions, bubbles, loops, Y-form DNA, or double-strand/single-strand junctions. RECQL4 ERCC5 RAD51C XPC MSH6 DMC1 YY1 MEN1 XRCC3 NEIL3 NR0B1 HMGB3 RAD51B MSH2 RAD18 BLM RAD51 HMGB1 RAD51D XRCC2 WRN HMGB2

GO_GLUCOSIDASE_ACTIVITY Catalysis of the hydrolysis of glucosyl compounds, substances containing a group derived from a cyclic form of glucose or a glucose derivative. LCTL GBA2 MGAM KL AGL GAA SI GBA3 GANC LCT MOGS KLB GANAB

GO_CYCLIN_DEPENDENT_PROTEIN_SERINE_THREONINE_KINASE_INHIBITOR_ACTIVITY Stops, prevents or reduces the activity of a cyclin-dependent protein serine/threonine kinase. CDKN2B CDKN1C KAT2B INCA1 CDKN2D CDKN2A CDKN1B HEXIM2 CDKN1A CDKN2C HEXIM1

GO_PHOSPHATIDYLINOSITOL_3_KINASE_BINDING Interacting selectively and non-covalently with a phosphatidylinositol 3-kinase, any enzyme that catalyzes the addition of a phosphate group to an inositol lipid at the 3' position of the inositol ring. PIK3R1 HCST FAM83B IRS2 INSR DAB1 XBP1 CALM3 AXL DAB2IP DNM2 BECN1 IRS4 IGF1R ATP1A1 CORO1A PTPN13 FYN CBL CALM1 JAK2 INSRR FBXL2 PIK3AP1 TYRO3 IRS1 CALM2 PDGFRB PIK3IP1 LCK FAM83A

GO_PHOSPHATIDATE_PHOSPHATASE_ACTIVITY Catalysis of the reaction: a 1,2-diacylglycerol 3-phosphate + H2O = a 1,2-diacyl-sn-glycerol + phosphate. LPPR4 PPAPDC1A PPAPDC1B LPIN2 PPAP2C LPPR2 LPPR3 PPAP2B LPIN1 PPAP2A LPIN3

GO_PHOSPHOTRANSFERASE_ACTIVITY_PHOSPHATE_GROUP_AS_ACCEPTOR Catalysis of the transfer of a phosphorus-containing group from one compound (donor) to a phosphate group (acceptor). DLG1 CASK PPIP5K2 GUK1 TJP2 IP6K2 MAGI3 DLG2 MPP3 CARD11 DLG4 NME5 AK8 NME6 IP6K3 NME9 MPP1 AK2 AK7 PCK1 AK4 DTYMK NME7 PPIP5K1 CMPK2 NME4 AK1 NME1 AK5 PMVK RAD50 AK3 NME3 AKD1 DLG3 MPP2 IP6K1 NME2P1 TXNDC3 CMPK1 NME2

GO_TBP_CLASS_PROTEIN_BINDING Interacting selectively and non-covalently with a member of the class of TATA-binding proteins (TBP), including any of the TBP-related factors (TRFs). PSMC6 BRF1 HHEX THRA HNRNPF PSMC2 GTF2B PSMC4 CAND1 YEATS2 CAND2 TAF1B GTF2A1 DR1 PSMC5 GTF2A2 TAF1L TAF1 PSMC3 PSMC1

GO_SULFUR_COMPOUND_TRANSMEMBRANE_TRANSPORTER_ACTIVITY Enables the transfer of a sulfur compound from one side of the membrane to the other. SLC25A26 C2orf83 CTNS SLC26A8 SLC26A6 SLC26A1 SLC7A9 SLC26A5 SLC26A3 SLC26A2 SLC26A10 SLC26A4 SLC35B2 SLC13A1 SLC19A3 SLC1A4 SLC19A2 SLC26A11 SLC13A4 SLC7A11 SLC26A9 SLC38A7 SLC35B3 SLC1A1 SLC3A1 SLC26A7 SLC6A6

GO_CALCIUM_ION_TRANSMEMBRANE_TRANSPORTER_ACTIVITY Enables the transfer of calcium (Ca) ions from one side of a membrane to the other. TRPM2 MCOLN2 CATSPER1 JPH2 CACNA1C TRPV5 CACNG7 PDE2A CACNB2 GAS6 CACNB3 CACNA1F TMC1 CACNA2D4 CACNG6 TRPC3 TRPM1 PKD1L2 SLC24A3 TRPM8 CATSPER4 GRIN3B SLC8A2 PSEN1 SLC24A5 TRPM6 CATSPER2 TPCN2 TRPV6 CLCA3P GRIN3A CALHM1 CACNA1A PANX1 CACNG5 RYR3 CACNA1S CACNG3 LOXHD1 TRPC4AP TPCN1 RYR2 ITPR1 TRPM3 CACNA1E RASA3 CHRNA9 ATP2C1 PKDREJ CUL5 MCOLN1 PKD2L1 TRPV4 CACNG2 GRM7 ATP2B2 SLC24A4 TMEM37 ITPR2 ITGAV ATP2A2 JPH3 ATP2B3 ORAI2 ZP3 TRPC6 ORAI3 CACNB4 STIM2 TRPM7 CACNA2D1 ATP2A3 TRPV3 ATP2A1 PKD2 TRPC5 TRPA1 RYR1 CACNA1B TRPV2 CACNA2D2 IL1RAPL1 SLC8A3 CACNA2D3 PKD2L2 MCOLN3 CACNG1 OPRM1 DENND5B CACNA1I CACNB1 TRPC7 TMC2 CACNA1G SLC24A1 PKD1L1 ORAI1 TRPV1 MCU FKBP1B CATSPER3 SLC24A6 SLC24A2 NCS1 TRPM4 CACNA1H CACNG8 SLC8A1 ITPR3 SLC3A2 ATP2C2 PKD1L3 TMCO1 DENND5A TRPC4 C9orf7 CHRNA10 ATP2B4 ATP2B1 GPM6A GRIN2A CACNG4 PKD1 GRIN1 CACNA1D TRPC1 CCDC109B

GO_KINASE_BINDING Interacting selectively and non-covalently with a kinase, any enzyme that catalyzes the transfer of a phosphate group. MAPK8IP1 FBXW5 SASH1 TOP2B GPRC5B USF1 CALM3 TBL2 PTPN11 SYN1 MTOR ELAVL1 SREBF1 NRG1 RHEB FGFR1OP NME1 GOLGA2 SPAG16 CEACAM1 MYOC MAP2K1 PIK3R2 MIDN SHC4 CDKN2D SOCS1 MARCKS PRKRIP1 PTPN23 DNM2 RAC1 SPDYE1 RYR2 HPCA CDKN2C DUSP12 EEF1A2 CCNYL2 ARRB1 PIP5K1A JAKMIP3 MAP3K11 GTF2I SOCS5 ITGB2 NFATC1 BTG1 GDF3 TPRKB CAV2 HIF1A STUB1 ZC3HC1 RNF138 RAB8A PIK3R1 CEBPA GHR CHEK2 MOB1B GNB2L1 SHC1 KLRC4-KLRK1 GRB2 MAPK8IP2 FBXO5 TRIB2 PARK2 PRC1 HSP90AB1 CCND3 DVL1 TAX1BP1 TCF3 MAPK1 LAX1 DAB2IP GAB2 STAT3 DSP GLRX3 SLC12A7 BLNK GRK5 GATA4 MLKL RAD9A ACVRL1 MAPK8IP3 PLK1 CEP250 MYH6 LATS1 CBLC CIB1 STXBP1 CRY1 GRB7 HTT IGBP1 FAM83E TIRAP APPL1 PKIA ANGPT2 SRCIN1 RACGAP1 SLC12A2 CKS1B IFNAR2 TRAP1 PIK3IP1 TRAF2 TWF2 VRK1 CD3E IRS2 EGFR TOP2A PLA2G6 AVPR1A SPDYE3 PARN FAS PICK1 PTPRK KCNA5 SPDYE6 ANKRD2 DGKQ ACSL3 PPP1R12B PRKAG1 CCNL2 SDPR NPR1 BAD CNPPD1 FOXO3 SCN5A DACT3 PAX6 SIRT1 RFFL AKT1 SPAG9 PRKAG2 TP73 IKBKB FAM83A SPRED1 SLC2A1 ZFP36 CTNND1 TPR PEBP1 PRKCB FZD5 FLNA FBXO7 CCNE2 PRKAB1 PAG1 TRIM6 C1orf88 C13orf15 SP100 RGS20 KIF5B DNAJC3 SIK1 RCC2 TP53 ELP2 CDKN2B LIMS1 TTN PKP2 DVL2 NBEA TPX2 PRKAR2B GATA6 KCNQ1 FRS2 SPDYE5 BAG5 MAG WNK1 AP2A1 ADCY6 RPS3 C1QBP CDC25C PARD6A CEBPB PPP1R9A AURKA PTEN PKD1 UGT1A7 CCNY FAM83D PTPN14 YWHAG RHOD MAP2K4 STAP1 AKAP13 TNIP1 TPCN2 TDG WWC2 VRK2 PINK1 ACTA2 IL31RA MAPKAP1 FEZ1 EIF3A GNAT1 SPDYC AXIN1 BCL2L14 PLCG1 SPDYE4 LCK CDK5RAP3 POLA1 NCK1 UTRN PTPRR TRADD CBL ZNF259 MARVELD3 CDC6 HDAC4 EZR FAM150A PFKFB2 NSF PLEK E2F1 KLRK1 KAT2B MAPT DLG3 PTPRJ ATF7 RGS14 PPP1R12A PPP1R12C PPME1 TOM1L1 LLGL1 PAK2 TIAM1 BRSK1 FAM83B GCET2 ACE PRMT1 FNTA SRSF2 TSKS DLG2 MICALCL PARK7 NCS1 BCAR1 PARP16 GSK3B TCF7L2 SMAD1 RELA HNRNPA0 IRS1 CASP9 DOCK4 RAF1 WAS CDK5RAP1 CD28 CTNNB1 CD8A RB1CC1 TBC1D14 RARA PRKACA PTK2 ANGPT4 AVPR1B STK38 CACNB3 MAP2K7 KIF13B PDPK1 PRDX3 JAKMIP2 HDAC9 WWC1 ARRB2 TRAF3 TELO2 ARHGEF16 SRSF5 KIF20A LDHB MOB4 SV2A TRIB3 CDC25B PXN C2orf44 CEP68 KSR1 CNTLN NTRK1 SRSF1 PRKCDBP RGS19 DACT2 MAP4K2 SGOL1 ATP1A1 GYS1 DUSP2 PRKAA1 TICAM1 TRIM49 CD24 CCNL1 MSH2 EMP2 FAM83H ADAM10 LAT TEX14 IQGAP1 PGAM1 DUSP3 PIN1 DCX PPP1CB NEFH SHC3 CYLD TNNI3 SPRY2 SQSTM1 NPM1 KIF11 DPYSL2 PLK1S1 CAV1 CD226 PTPN1 TNIP2 CCNB3 STK11IP SPDYA MEF2A JUP SKAP1 AXIN2 SPDYE7P CPNE3 NOD2 CDC42 PPP1CC CDC37 SFN EXOC2 NEK6 YWHAZ DAXX SIT1 CLTC TRAF6 ITGAV CDC5L IL1RAP RICTOR NR4A3 ELMO2 C10orf46 LIPE ITGB1BP1 PDLIM5 MAML1 PPM1D CCNB1 SNAP91 PRKAR2A AP1B1 ATF2 TRAT1 HSP90AA1 FAM83C PPEF2 PER3 PAM FAF1 STK39 PARP1 RPS6KA4 HDAC7 SRC TRIP6 DIRAS1 CALM1 IBTK HINT1 BRAF CCNA2 DOK7 IGSF9B ANGPT1 BCL10 ARHGEF7 LDB3 EPHA1 MAP3K1 BORA PFKL PIH1D1 EEF2 UGT1A10 TMEM173 MAD2L2 SPDYE2L SUFU SDC4 CCND1 BARD1 MSN PAK1 STX17 PER1 SYK PTAFR CSK GCN1L1 OSR1 RAB13 GAS6 UBQLN1 SKI STX1B PRKCSH PDCD10 PDE3B TGFBR2 MST1 RPS6KA3 TAB1 CHP NRG3 ILK ATF5 SNAI1 PRKAG3 RPS19 ADIPOR1 CCND2 ZBTB4 PFKM MAVS PFKFB1 PRKAR1A DLG4 DACT1 MAP2K6 STX1A RPS6 DNAJA3 SHC2 LDHA CADPS HSPB1 SLC12A4 DLG1 ABI2 HYAL2 CDKN2A ABL1 CDK5R1 CCDC88A TNFAIP3 DBF4B PTPRC ERRFI1 STRADA THY1 FAM150B CAMK2N2 NEK9 PTPN2 APC TENC1 CAB39 RPTOR WWC3 SOX9 PTK2B PTPN6 PPP1R15A DNM1 PRKCZ JAK2 DUSP19 HDAC5 PJA2 MAP2K3 AKAP7 MAP3K12 PDGFRB GSTP1 CD4 ANK2 MAP3K13 SPRED2 TOB1 FIZ1 SMAD3 FLT3LG CDK5 DOK2 PRKCD TRAF4 MAP3K5 IL12RB2 LRP4 JTB CBLB TAOK2 PTPN22 TRPV4 CHIA CDK5RAP2 PRKAR1B HCLS1 NBR1 H2AFY MAPK4 CAMK2N1 CCNE1 EEF1A1 CCNK RAB11FIP2 CKS2 FGR PKN1 ADAM9 VDAC1 GFAP KCNH1 KIF14 FOXM1 BCL2L1 TRIB1 TRIM22 PIWIL1 CSPG4 TOLLIP MAP3K2 CDC25A USP37 RASGRP3 CEP152 PITPNM3 TRIM5 ADRA2A PPP2R5A TOM1L2 PCNA JAKMIP1 ATG13 RPS18 MVP GSK3A AP2A2 MAPK7 MAPK6 CENPJ CALM2 CCNYL1 BRSK2 RB1

GO_GAMMA_CATENIN_BINDING Interacting selectively and non-covalently with the gamma subunit of the catenin complex. APC DSG1 APC2 PTPRJ CTNNA1 TCF7L2 PTPRK CDH1 CDH2 LEF1 PTPRT

GO_LYSINE_ACETYLATED_HISTONE_BINDING Interacting selectively and non-covalently with a histone in which a lysine residue has been modified by acetylation. PHIP BAZ1B CARM1 BAZ2A BRD7 ATAD2B SMARCA4 BRD2 TAF1L TAF1 BRD4 TRIM24 MLL BRD9 PSME4 BRD3 BRDT

GO_CATION_AMINO_ACID_SYMPORTER_ACTIVITY Catalysis of the transfer of a solute or solutes from one side of a membrane to the other according to the reaction: amino acid(out) + cation(out) = amino acid(in) + cation(in). SLC1A2 SLC6A12 SLC32A1 SLC6A7 SLC36A1 SLC1A1 SLC36A3 SLC38A1 SLC6A20 SLC6A15 SLC6A5 SLC36A2 SLC6A11 SLC6A1 SLC6A13 SLC6A9

GO_SODIUM_AMINO_ACID_SYMPORTER_ACTIVITY Catalysis of the transfer of a solute or solutes from one side of a membrane to the other according to the reaction: amino acid(out) + Na+(out) = amino acid(in) + Na+(in). SLC6A1 SLC1A1 SLC6A13 SLC6A9 SLC6A11 SLC6A7 SLC6A12 SLC6A5 SLC1A2 SLC38A1 SLC6A20 SLC6A15

GO_NAD_DEPENDENT_PROTEIN_DEACETYLASE_ACTIVITY Catalysis of the removal of one or more acetyl groups from a protein, requiring NAD. HDAC11 HDAC1 HDAC10 HDAC9 SIRT4 HDAC6 HDAC4 HDAC8 HDAC2 SIRT6 SIRT1 SIRT7 SIRT3 HDAC3 SIRT2 HDAC5 HDAC7

GO_LIGASE_ACTIVITY_FORMING_CARBON_OXYGEN_BONDS Catalysis of the joining of two molecules via a carbon-oxygen bond, with the concomitant hydrolysis of the diphosphate bond in ATP or a similar triphosphate. GARS TARS2 NARS PARS2 SARS2 CARS2 SARS LRRC47 DARS2 YARS HARS2 RARS WARS HARS VARS LARS QARS FARS2 EPRS LARS2 POLG2 RARS2 FARSB YARS2 IARS MARS KARS MRPL39 VARS2 FARSA IARS2 CARS NARS2 EARS2 WARS2 FDXACB1 TARSL2 AARS DALRD3 AARSD1 TARS DARS MARS2 AARS2

GO_PHOSPHOTRANSFERASE_ACTIVITY_FOR_OTHER_SUBSTITUTED_PHOSPHATE_GROUPS Catalysis of the transfer of a substituted phosphate group, other than diphosphate or nucleotidyl residues, from one compound (donor) to a another (acceptor). DPAGT1 CDIPT CHPT1 SGMS1 CDS1 GNPTG PIGN PTDSS2 GNPTAB SAMD8 CEPT1 PIGO EPT1 PLD6 PGS1 AASDHPPT PIGF CRLS1 PIGG SGMS2

GO_RNA_POLYMERASE_II_CORE_PROMOTER_SEQUENCE_SPECIFIC_DNA_BINDING Interacting selectively and non-covalently with the regulatory region composed of the transcription start site and binding sites for transcription factors of the RNA polymerase II basal transcription machinery. SP3 RUVBL2 H2AFZ SOX3 SUZ12 CEBPB GBX2 SPI1 NLRC5 YAP1 ZFPM1 HOXB3 FOS RBPJ H3F3B H3F3A NFIL3 SOX11 MTF1 SMAD5 HNF4A GATA6 TBR1 RREB1 TFAP2B SMYD3 GATA1 STAT1 GATA2 TFAP2A H2AFY ZNF277 PAX8 IRF7 EZH2 WBP2 TAL1 SMAD1 PAX6 SOX8 KDM2B HEYL HSF2 TBP CDK9 STAT6 NSD1 MAZ ZNF335 EP300 HSF1 STAT5B NR4A2 GATA3 RUNX2

GO_UBIQUITIN_LIKE_PROTEIN_SPECIFIC_PROTEASE_ACTIVITY Catalysis of the hydrolysis of peptide or isopeptide bonds within small proteins such as ubiquitin or ubiquitin-like proteins (e.g. APG8, ISG15, NEDD8, SUMO), or between the small protein and a larger protein to which it has been conjugated. USP16 VCPIP1 USP46 USP22 ALG13 ATXN3 OTUD6A USP39 UFD1L MYSM1 LOC100287205 USP42 EIF3F USP25 USP9Y ZRANB1 OTUB2 USP17L2 UCHL5 USP32 USP34 USP17L3 USP27X FAM63B OTUD6B CYLD OTUD5 LOC100287404 TNIP1 TANK ZC3H12A USP40 USPL1 FAM76B BAP1 USP41 FAM188B USP33 ATXN3L USP50 USP31 JOSD1 TNFAIP3 STAMBP JOSD2 LOC100287178 USP21 USP13 USP29 FAM76A LOC100288520 COPS4 USP45 LOC100287144 LOC100287513 USP37 USP17L8 UCHL3 USP7 USP8 USP53 USP18 OTUD7A USP49 USP10 USP38 OTUD7B USP30 USP14 FAM188A USP9X USP2 USP12 SENP3 USP17L4 USP3 USP4 ANKZF1 FAM105B SENP1 SENP2 UFSP2 UFSP1 USP54 UCHL1 USP51 OTUD1 LOC100287441 SENP5 OTUB1 BRCC3 USP44 USP17L5 USP1 USP19 LOC100287478 USP43 USP15 USP24 YOD1 USP6 USP17L1P SENP6 LOC100287238 OTUD4 OTUD3 FAM63A USP48 COPS5 USP35 USP17 USP36 USP5 USP26 USP20 USP28 LOC100287364 LOC100287327 USP17L7 USP11 USP17L6P USP47

GO_DNA_APURINIC_OR_APYRIMIDINIC_SITE_LYASE_ACTIVITY Catalysis of the cleavage of the C-O-P bond 3' to the apurinic or apyrimidinic site in DNA by a beta-elimination reaction, leaving a 3'-terminal unsaturated sugar and a product with a terminal 5'-phosphate. ALKBH1 APEX1 OGG1 NEIL3 POLB NEIL1 APLF RPS3 NEIL2 APEX2 NTHL1 HMGA2 SMUG1

GO_ZINC_ION_BINDING Interacting selectively and non-covalently with zinc (Zn) ions. ADAMTS2 ZZEF1 TRAF4 UBR5 RNF185 GATAD1 NR1I3 ASTL PHF17 SMAD3 TRIM74 ZDHHC12 BRAP L3MBTL2 SOD1 CD4 ZNF185 AMZ1 CSRP3 FBXL19 TES RNF166 GATA1 ZCCHC24 SOD3 SF1 POLR2I MMP21 ZCCHC2 CXXC5 LMO1 PGGT1B PJA1 RNFT1 CBLB MARCH2 PGR PTGR2 RFWD2 DBF4 ZCCHC16 MT1B ZFPM2 TEX13A ERAP2 PHF19 TIMM8B TRIM5 USP49 QPCT RNF219 ZFYVE20 ADAMTS16 CCS ZNF207 LNX1 ZFAND2A ZBTB32 PYGO2 RPH3A ZFAND5 POLE MEX3D HLTF NSMCE1 CRIP2 ADA MSRB3 MMP10 CNOT4 CYHR1 SIAH1 ADAM33 RNF11 TRIM71 ADAMTSL5 APOBEC4 MMP7 TCF20 APOBEC2 TRIM41 PNMA3 SKI ADAMTS1 ADAMTS20 CPM PDLIM3 TRIM50 ZNF212 TET2 RNF103 NR1I2 LRSAM1 RFPL4A FHL5 PRICKLE1 ZCCHC18 ZNF84 AR RNF128 GCM1 ZCCHC6 NR6A1 RNF170 TCEA2 DPF2 KAT7 ZFPL1 MLLT10 RUFY1 RSF1 MGRN1 ZNF282 SEPX1 RNPEP VPS8 SEC24A ZDHHC21 PIKFYVE ZNF208 TRIM49B SHPRH RNF135 PHF10 TRIM7 PHF14 TRIM42 RNF17 TRAF7 DNAJC24 DNLZ ADAMTS14 ZSWIM4 BMI1 TRIM37 LIMS2 RNF212 GTF2B ZNF75D MORC2 MBD1 NR1D2 SNRPC PHF1 EEA1 RNF126 CRIP3 HIF1AN KLF7 HHIP ZNF3 NHLRC1 PDLIM5 RNF122 TIMM10 TRIM65 AARS NR4A3 RNF149 TRIM64 TNP2 CA5A ZFHX3 PHF21A GATA5 NR2C1 RBM4B KDM1B GDA MARCH11 CDA LMO4 RNF32 TRIM8 CRYZL1 POLR2K RNF183 TRIP6 AGBL2 PAPPA TRIM45 KDM2A ZSWIM5 KDM5A TRIM72 PAM EHMT2 PIAS3 PHRF1 CPA6 BARD1 RNF125 ZNF331 RASGRP1 ZNF385C NSD1 ZNRF4 ZNF593 ZCCHC8 NEIL1 ZCCHC14 NR1D1 KDM2B GATA3 LIMD2 MAP3K1 LMX1B PHF12 RING1 MICAL3 UBOX5 TRIM52 LIMS3 RNF13 TRIM49L1 ITGB1BP2 LTN1 RNF150 RASSF1 RPS29 ALPP RARA CXXC1 PCGF1 KDM5B DNAJC21 ISL1 USP51 ABLIM1 TAF15 MDM2 RNF39 ZDHHC9 TLL2 ADAM30 USP44 CPZ LONRF2 PXN ZNF385B LIMA1 MMP24 G2E3 ZFAND3 MKRN3 ZFAND2B SP140L RNF123 PIAS1 ZNFX1 RNF2 PPP1R39 RAD18 MLL5 NR1H4 JUB RNF26 ZFR TRIM49 TRIM29 ZBBX ANAPC11 LNPEP DCST1 ANKIB1 RNF207 RYBP TRIM60 ZNF622 PM20D1 NR2F2 RNF5 ZDHHC13 PEX12 LTA4H KDM4B RNF186 CYLD CLIP1 SEC23A KPNB1 MMP11 ADH6 KCMF1 SETDB1 CALB1 CPA5 MARCH7 EHMT1 GCH1 CRYZ RNF151 MMP9 ADAMTS10 QPCTL PHF15 MSRB2 DTNB MMP17 ADAMTS4 RNF145 DTX4 ZNF330 TRIML1 UBR3 AEBP1 SH3RF1 RLF CBL FUS PCGF6 PDLIM4 CSRP2 ABP1 CA5B TRIM24 ING2 LOC399939 ZDHHC5 ZDHHC6 PHEX TK1 HDAC6 RNF187 RNF19A ZDHHC1 APOBEC3B CALR MMP23B SEC24C CHD3 TCF19 PHC1 RNF40 MLL ADAMTS18 LPXN SLU7 KAT6B DTX1 TRIM58 FHL3 TRIM9 ING3 KDM4C POLR1A THRA CPB1 CHMP1A NR1H3 SP110 RNF224 CNDP1 ZNF24 MYT1 LHX2 KDM5D NANOS2 CA3 MYRIP TNKS ADAMTSL2 BAZ1B MT1H MPI RNF4 TRIM59 MBTD1 RNF144B CBLL1 MEX3B RNF6 MORC4 ERVK-6 LHX3 DTNA TP53 LASP1 MT3 UBR4 SORD TRIM21 MEX3A RNF167 TRIM10 SIRT2 WT1 GLI2 LHX8 ERVK-10 PHC3 FHL2 ACE2 ZMIZ2 PHF8 GATA6 TRIM46 MT1G NANOS3 UPF1 UBR2 CPA4 RFPL4B BLVRA ADAMTS17 VDR CPXM1 ZNF2 NR4A2 CSRP1 ZMAT2 RLIM ZDHHC15 ZNF385D TRIM43B ADAMTS7 CBLC SNCA MKRN1 XAF1 ESR2 RNF7 ZMYND11 APOBEC3D YAF2 LIMK1 GATA4 ZCCHC3 TRIM69 NEURL3 MEFV CXXC4 ATP13A2 AFG3L2 MARCH8 TRAIP ZFHX4 KDM4A APOBEC1 C4orf21 MEX3C LHX9 ZMAT1 ZDHHC22 SMPD1 MT2A OTUD7B ZNF90 TET1 RNF175 MME TRIM38 NPLOC4 SLC30A8 ZNF253 MMP15 ZCRB1 RNF133 RORB MDM4 ADH1C PAPLN ENPP1 DNMT1 ZFHX2 BBOX1 PMPCB ZMAT3 WHSC1L1 RFFL BHMT2 WBP4 MMP2 ZNF385A BMP1 ABLIM2 TOP3A TRPS1 PGLYRP4 BRF2 RFPL3 MECR PRICKLE3 XIAP SETDB2 ADAMTS19 CHD5 USP20 NR5A2 MAN2B1 ACMSD TRIM66 MIB2 TRIM67 TRIM31 RNF10 TRIM47 SHARPIN CAD SUPT4H1 TRIM40 NR2C2 RNF222 GTF2H2 PRICKLE2 SUV39H2 TRIM63 ZACN LMX1A WDFY1 CA1 CPB2 ADAMTS6 BPTF TP53I3 KNG1 EWSR1 ZSWIM6 TRIM34 ADAM8 ZMYM5 DCTD USP3 DIDO1 FBXO11 RC3H1 TRHDE MT1F ADAMTS12 ZCCHC7 RNF180 PHF20 RNF138 NEIL3 RARG UQCRC1 MYT1L MYLIP ACY1 RABGGTA IGHMBP2 LIN28A THRB TRIP4 ATXN7L3 PRICKLE4 ZCCHC4 SYVN1 PML ZCCHC23 RNF113A NR2F6 SCEL TLL1 ZCCHC13 S100A8 ZMYM3 PGLYRP1 LIN28B MMP16 RNF214 WHSC1 AMFR RNF111 SREK1IP1 MMP1 ZDHHC11B NRD1 TTF2 CA8 ABLIM3 DDX58 PTGR1 EGR1 MMP25 RNF144A PHF13 MMP8 SMPDL3A LOC440434 MYCBP2 ZKSCAN5 RNF157 TRIM39 TRIM23 MICAL1 RERE ZNF117 PPARG GRIN2B BRF1 GATAD2B GLO1 ESR1 PGLYRP2 BRPF3 BRCA1 MMP3 CPA2 NBR1 TRIM73 SF3A2 CA9 AICDA ZMYM4 SIRT4 RNF181 ZNF93 ING1 UNKL TRIM55 RNF115 CGRRF1 TIMM13 TRIM22 TRIM27 NR5A1 PHF3 S100A9 ZMAT4 ERVK-7 RABGGTB BAZ2B DTX3L MMP20 DPP3 PHF23 TRIM32 MKRN4P MTA3 RNPEPL1 MEP1A APOBEC3C ADAMDEC1 ADH1B ZNF257 SPRYD5 LONRF1 RNF113B ANPEP ANUBL1 MT1X APOBEC3H ERAP1 RBM10 RORA TRIM2 SLC30A5 PITRM1 ZNF184 RNF182 RNF146 TRAF1 LHX6 CPE HNF4A LANCL1 RNF38 MUL1 MAN2B2 TRIM61 S100A5 ZDHHC24 SEC23B MMP14 ISL2 IDE TRIM75 PIAS4 ZMYM2 LPP PDLIM7 LMO2 TTC3 ADAMTSL3 MMP28 RNF24 CREBBP DHX58 MT1E NR3C1 PGLYRP3 MICALL1 TRIM68 KDM5C DPYS HERC2 NANOS1 RBM4 RBX1 PRKCG ADH4 PDXK SHANK3 DBF4B ZNF146 RNF20 CPN1 TNFAIP3 ZDHHC8 BIRC3 LONRF3 NEURL S100A3 CA7 ADAMTS13 ARHGEF2 PJA2 CHURC1 QTRTD1 BIRC7 ZNF318 SF3A3 RBBP6 ZDHHC19 TRIM62 CHORDC1 RFWD3 USP22 KIAA0913 MLL2 UHRF1 MIB1 ZFAND1 RAG1 MATR3 RNF141 FNTB ADAMTS15 GLRA1 CECR1 MARCH5 TMEM163 ZZZ3 ZDHHC4 TCEA3 TCEA1 PTPN1 LOC729974 CDADC1 ZDHHC16 ZDHHC23 ZNRF2 ADH1A PPARA RNF165 THAP11 ZCCHC11 DNPEP RNF169 DHH RNF8 ZCCHC12 BAZ1A MICALL2 TRAF6 AGBL1 MARCH1 TRIM54 ZNRF3 RC3H2 RNF34 ZMYM1 CHD4 S100A7 ZCCHC10 SHH PARP1 ESRRG SP140 NR2F1 NEURL1B RNF213 PEX10 AQPEP VAT1 SIRT5 RNF112 S100A6 ALKBH8 FBXO40 DTX2 MYNN ING4 GTF2H2C PCGF3 LDB3 RPS27 ZSWIM1 ERVK-5 MSL2 ST18 ALPI ESRRA TRIM43 ZNF598 SEC24D DPF3 ZDHHC11 MEP1B AGBL4 CA6 ZNF195 CNDP2 ZFYVE1 ZCCHC9 PHF16 ZGLP1 ENPP2 CPA1 SIAH2 ADAMTS5 MMP12 UQCRC2 UBR1 CA4 NFXL1 DRP2 ZSWIM3 INTS12 TRAF3 SOLH CA13 ADAT2 RBM5 OTUD7A S100B TRIM3 ZNF276 MARCH4 PMPCA PTMS PEX2 MTR NR4A1 ZNF638 RXRG CNBP LNX2 RBAK L3MBTL4 ZFP37 NR3C2 TRIM64C THAP1 MT1A PDZRN3 ADAMTS3 YY1 RXRA MAN2A2 MARCH10 ZDHHC20 SQSTM1 LMCD1 JHDM1D ZDHHC7 ZRANB1 ASH1L ZCWPW2 TAF2 ESRRB ARIH1 GRIN2A NEIL2 CUL9 USP39 ZRANB3 RNFT2 LOC646862 RNF148 SIRT6 RNF114 ZDHHC17 MTA1 DTX3 PIAS2 RABGEF1 MLL4 BRPF1 PDLIM1 C11orf54 RNF43 EARS2 HDAC4 PRDM4 LHX5 ZNF92 ZNF259 ZSWIM7 ALAD UTRN TRIM44 MORC3 ARIH2 ZDBF2 ADAMTS9 FBXO30 RNF14 MLL3 S100A12 LIG3 MTF2 LOC100133495 RNF130 TIMM9 TAB2 CA12 LHX4 ZNF8 ZNRD1 SIRT3 ZMAT5 RGN TRIM4 TAB3 PHF7 NSMCE2 SH3RF3 KAT6A USP13 ZDHHC14 RNF220 NR1H2 ENPEP ZNF830 VPS11 GATA2 RAI1 L3MBTL1 TRIM26 TRIM17 SPG7 BAZ2A KLF4 PCGF2 ACE ZIM2 RTN4IP1 TRIM6 RBCK1 NUP153 GATAD2A BIRC8 ACR PRKCB TRIM56 APOBEC3F NAPEPLD ZADH2 TRIM36 TRIM28 PPARD LIMK2 MARCH9 TRIM25 PDLIM2 LIMS1 PRDM2 LHX1 BFAR DZIP3 RCHY1 MMP26 ZYX ZNF675 MARCH6 SCAPER EP300 CPO TADA2B MARCH3 AGTPBP1 ZNF645 SEC24B USP45 HRG VAT1L TRIM64B ZNF205 RNF139 RANBP2 GTF2H2D ZEB1 SETMAR BIRC2 RNF208 MMP13 TRIT1 ERI2 BHMT SCAF11 CPA3 PTER ZMYM6 NR2E1 RBM20 CRIP1 APOBEC3A DMD TRAF5 MID2 ZNRF1 ZSWIM2 APOBEC3G RNF223 CCNB1IP1 MKRN2 ADH7 RNF25 RNF19B ZNF407 ZRANB2 PHF11 P2RX4 HNF4G RNF44 SAMHD1 ZNF214 MNAT1 BRD1 LMO3 CPD TRIM48 SRSF7 ZFAND6 GALT TRAF2 TRIM16 RNF168 AARS2 MAN2C1 TRIM14 TRIM77P CIZ1 FHL1 RXRB ZMIZ1 RFPL1 RABIF AGBL5 TGFB1I1 DYTN LOC100129520 TRIM33 TRIM49L2 PRKCA RARB AGBL3 LMO7 S100A13 APEX2 UHRF2 TRIM13 NRAP AMZ2 ZNF346 TOPORS LACTB2 LIMD1 MID1 ZNF22 RNF121 IFIH1 RNF41 ZNF91 PEG10 USP16 SUV39H1 PHC2 CHFR DPF1 ING5 NR2E3 PYGO1 P2RX1 WTIP USP5 TRIM11 ZCCHC5 MTA2 FBLIM1 RSPRY1 ZFR2 MLLT6 CTCF CPXM2 ZDHHC3 TRIM15 RNF215 PHF2 ADAT3 PHF21B DUSP12 MMP27 APIP AKAP8 PDZRN4 LIMCH1 MICAL2 POLR3K PCGF5 TAF3 CPSF4 ADAMTSL1 ZCWPW1 BSPRY ZC3HC1 UBR7 DPEP1 C9orf3 ADH5 MORC1 TRIM35 RAPSN RNF31 USP33 NFX1 ERVK-8 NPEPPS RORC PHF6 RAG2 PAPPA2 ADAMTS8 MMP19 ZCCHC17 MAN2A1 PARK2 VPS41 ZDHHC18 RNF152 MT1M AIRE ZDHHC2 ZMYND8 POLR2L CA2

GO_MACROMOLECULE_TRANSMEMBRANE_TRANSPORTER_ACTIVITY Enables the transfer of a macromolecule from one side of the membrane to the other. TIMM17B TIMM17A LOC100652748 TFRC TIMM22 TOMM40L HNRNPA3 SIDT2 TOMM40 GPIHBP1 SIDT1 TOMM20 TOMM20L AZGP1 TOMM70A SEC61G TOMM22 TIMM23 MCL1 NUTF2 RAN SLC11A2 TOMM7

GO_SIALYLTRANSFERASE_ACTIVITY Catalysis of the transfer of sialic acid to an acceptor molecule, typically the terminal portions of the sialylated glycolipids (gangliosides) or to the N- or O-linked sugar chains of glycoproteins. ST6GALNAC1 ST6GALNAC2 ST3GAL3 ST6GAL1 C20orf173 ST3GAL2 ST8SIA2 ST6GALNAC3 ST6GAL2 ST3GAL1 ST3GAL4 ST6GALNAC6 ST8SIA3 ST3GAL6 ST8SIA4 ST8SIA6 ST6GALNAC5 ST6GALNAC4 ST8SIA1 ST8SIA5 ST3GAL5

GO_C_C_CHEMOKINE_RECEPTOR_ACTIVITY Combining with a C-C chemokine and transmitting the signal from one side of the membrane to the other to initiate a change in cell activity. C-C chemokines do not have an amino acid between the first two cysteines of the characteristic four-cysteine motif. CCBP2 CCR4 CCR1 CCR8 CCR9 CCR5 CCR3 CCR6 CCR7 CCR10 GPR75 CCR2

GO_CXCR_CHEMOKINE_RECEPTOR_BINDING Interacting selectively and non-covalently with a chemokine receptor in the CXCR family. PPBP ITCH PF4 CXCL13 CXCL6 YARS CXCL1 PF4V1 CXCL10 CXCL2 CXCL12 CXCL9 TFF2 CXCL3 IL8 CXCL11 CXCL5

GO_MAGNESIUM_ION_TRANSMEMBRANE_TRANSPORTER_ACTIVITY Enables the transfer of magnesium (Mg) ions from one side of a membrane to the other. SLC41A1 NIPAL1 MAGT1 CLDN16 NIPA1 SLC41A2 NIPA2 CNNM4 TUSC3 NIPAL4 MRS2 MMGT1 NIPAL2 ZDHHC13 ZDHHC17 CNNM2 NIPAL3

GO_HORMONE_RECEPTOR_BINDING Interacting selectively and non-covalently with a receptor for hormones. NCOA1 PPID TACC2 ARRB1 NPPA FHL2 NRIP1 RAN TOB2 LEF1 FYN GAS2L1 HIF1A KDM3A TRIM68 CCDC62 YWHAH RARG PPARGC1B ACTN4 NR1H4 CDK7 CEBPB NR4A2 NCOA2 TAF7 MED17 UCN3 VDR KDM1A PHB2 JAK2 RXRA MED24 HMGA1 OASL ADCYAP1 STRN PADI2 CTBP2 FKBP4 DYX1C1 MED16 TRIP4 DDX5 CTNNB1 BUD31 RNF6 NCOR1 PTPN11 MMS19 UCN NCOA4 RNF4 WIPI1 MED14 PRKCB NCOA7 NCOA3 BCAS3 SMARCA4 TYK2 EP300 ASXL1 PIAS1 ETS2 ZNF366 GH1 RUNDC3A FOXL2 THRAP3 HMGN3 PRAME CRX FLT3 ISL1 NR0B1 NR1H2 TAF11 DDX17 ZNHIT3 RARB MED30 CNOT1 UCN2 PARP1 GNAO1 TRIP6 SRC TGFB1I1 CALR NCOA6 SMARCD3 PTHLH STAT5B NSD1 GNAS FOXP1 MED25 PRMT2 MED12 PCNA LRIF1 PARK7 GRIP1 TCF7L2 STAT1 SIRT1 PPARGC1A RB1 KDM4C CRH FOXH1 PTH PSMC5 TRIP12 MED13 LEP JMJD1C STAT3 PRPF6 PIAS2 JAK1 MYOD1 DDX54 SOCS2 NPPC GTF2B CRY1 NUP62 DAXX MED4 TAF10 C16orf53 NKX3-1 SNW1 RNF14 NR4A3 TCF21 BRCA1 NR0B2 FUS KAT5 CCNE1 RERG MED1 PPARG PKN1

GO_PROTEIN_HETERODIMERIZATION_ACTIVITY Interacting selectively and non-covalently with a nonidentical protein to form a heterodimer. HIST1H2AH HIST1H2BH LIMK2 EPAS1 PPARD CENPA CUL3 MLX TFAP2E ALX4 HEXB ITGAL TAF8 QTRT1 MCL1 ATF6 MAFG UBA2 TCF12 HIST1H3I JAM3 HSPD1 ATF3 RBP4 FOXP2 TYRP1 IKBKG FBXO7 HIST1H3F BCL2L2 HIST1H4B ALG2 MLXIPL TLR1 VAPB ADIPOR1 INHBA HIST1H4F H2BFM BMP6 GUCY2F HIST2H2AC SOX17 HIST1H2BE SUPT3H ARNT SNX5 TAS1R2 EXT2 ADRB1 NPAS4 STX1A HIST2H2AA3 HIST1H2BC PSMF1 MYF5 TP53 BAK1 PANX1 SUCLG2 PVRL3 ARNT2 RRAGC HIST1H2BO CLNS1A DMRTA2 RRAGA GABPB2 GABPB1 KCNK9 RRAGD HIST1H3C ABCG8 CYBB H2AFZ P2RY1 HIST1H2BI HIST1H2AA HIST1H2BA H2AFJ HIST1H4L ABCG5 CEBPB ABCG4 PDGFB ADCY2 SOX15 UGT1A7 JAM2 NR4A2 SOX9 ADD1 PDX1 SPTA1 SCXB MID2 HIST1H4H TLR2 PRMT8 SDCBP HIST1H4I QTRTD1 HIST1H2AD BCL2A1 SNX1 FAM73B SOX8 ABTB2 TAF4 BIK RALGAPB HIST2H2BE ZBTB1 ODZ3 HIST1H4D NEUROD2 KCNB1 ADD2 SMAD2 PPP2R1A HIST2H4B PPP2R4 GAD1 TAF9 FZD4 XBP1 GCLM UGT1A1 SMC4 SMC3 SMAD3 KATNB1 NFYB UBA3 SYCP2 METTL3 EFHA1 KRT25 PVALB FMR1 KCNH5 TWIST1 IKZF4 MYOD1 CREB3L3 ADIPOR2 HIST3H3 HOMER2 NKX2-5 HIST2H2AB MPP7 ERBB2 HIST1H4J HIST2H4A VAPA BHLHE40 UGT1A4 IRAK3 SCUBE1 CYBA GAD2 POLA1 H3F3C DVL3 H2AFY MYF6 P4HB MAPK4 HIST1H2AE HIST1H2AI OSTalpha AOC3 HIST1H2BL HIST1H4G CAPN2 PGF TFAP2B KCNN1 PBX1 HNF1B JUN UGT1A8 UGT1A9 CXCL13 SYT10 ADRA1B SCGB1D1 YWHAE DR1 PDSS1 KCNH1 MAFA OSTBETA BCL2L1 H2AFB2 TYRO3 HIST1H2AM HAND1 CABYR SOS1 ADRA2A TYR HIST1H2BG NFYC CEBPG HEY2 FXR2 TCF4 AXL HIST1H4A DDIT3 ITGAM MEF2C ATF1 HIST3H2BB HIST1H2AC CD3G SMAD1 MAPK6 IL12A HIST1H2AB HIST2H2BD NTSR1 RELA BNIP3 MYOG RAF1 IRAK1 POLA2 SMC1A PKNOX1 DGKD ROPN1B GTF2A2 TOP2B HIST1H2BB H2BFWT USF1 DRD2 VEGFB PRMT5 NFKB1 PVRL1 RARA HIST1H4K AGTR1 SMC2 NR2C2 HIST2H3D SUPT4H1 ITGB1 RAB3GAP2 PEF1 KATNA1 ODZ1 ODZ4 HIST2H3A DMRT3 PIK3R2 POLE4 IKZF3 SRI SIM1 UGT1A3 TBX18 SYT6 ZHX2 CHRNB2 CD3D FXR1 TAF9B EXT1 H2BFS HAND2 INHA HIST1H3J FZD9 TPD52L1 ODZ2 HIST1H4E UGT1A6 NAE1 CHRAC1 TAF12 JDP2 BNIP3L H2AFX SOX4 HIST1H3E SUPT5H NR4A1 ITGB2 TGFB3 IRAK2 APITD1 CAV2 GABPA CXorf27 NOTCH4 SLC3A1 TAS1R3 HIF1A FLOT1 HIST1H3G TAF4B YWHAH PAFAH1B2 HIST1H4C PPP3CA NOTCH1 PIK3R1 HIST1H2BM SYT16 HIST1H3D HIST1H2AL HEYL HIST1H3B GCLC NRN1L ZHX3 GTF2A1 TAF6L TAS1R1 SOS2 HMG20B HIST1H2AG TAL1 BHLHE41 HIST1H3A HIST1H2AK RXRA USF2 BDKRB2 NPM1 HMG20A HIST4H4 BCL2 CAV1 TCF3 IL12B HIST1H3H MEF2A SDCBP2 FOS H3F3B BAX ZHX1 H2AFB1 ADRA2C APOA2 CENPW LIMK1 PAFAH1B3 ADORA1 ANO2 KCNB2 NEUROD1 POLE3 BHLHA9 MEF2D IKZF2 TIRAP PVRL2 CLCN3 KCNK3 TGFB2 ST20 DMBX1 SNX2 PDSS2 HIST2H3C RALGAPA2 TXLNG ENO3 HIST1H2BF DRAP1 ALX1 CHRNB4 TIMELESS HIST1H2BK FLOT2 LOC391742 SOX14 ATF2 CRLF1 TBX15 MAX TGFB1 FAM73A TAF11 CD3E TFDP3 GCA SAE1 EGFR TOP2A MEIS1 AHR NEFL ADCY5 PDZD7 SCGB2A1 TTR ABCG1 H3F3A TAF6 SRGAP2P1 H2AFY2 MICU1 CEBPE HIST1H2BD CENPT ITGA2 SOX6 BMP2 BRAF PDGFA H2AFB3 ADRA1A ATF4 CLCF1 CHUK CEBPD BOK HIST3H2A H2AFV SYT5 MTTP DMRT1 BTBD11 SMAD4 HGF SIM2 BAD HEXA RALGAPA1 HIST2H2BF UGT1A10 HIST2H2AA4 ERBB3 TPD52 SNX6 HIST1H2BN MID1 BCL2L10 BARD1 SOX18 TAF13 TFAP4 VEGFA HIST1H2BJ IKBKB ITGA3 KHDRBS2

GO_NARROW_PORE_CHANNEL_ACTIVITY Enables the transport of a solute across a membrane via a narrow pore channel that may be gated or ungated. KCNK12 KCNK2 PANX1 KCNK7 KCNK1 KCNK13 KCNK15 KCNK16 KCNK9 KCNK17 KCNK10 KCNK4 KCNK5 KCNK18 KCNK6 TMEM175 NALCN KCNK3 RHAG

GO_HEPARIN_BINDING Interacting selectively and non-covalently with heparin, any member of a group of glycosaminoglycans found mainly as an intracellular component of mast cells and which consist predominantly of alternating alpha-(1->4)-linked D-galactose and N-acetyl-D-glucosamine-6-sulfate residues. AGER IMPG2 LOC100507050 APLP1 PCSK6 VEGFA MMP7 ADAMTSL5 FGFR2 THBS2 PRELP FGFRL1 FBN1 SERPIND1 FGF4 PLA2G2D NDNF SOST TNXB LRPAP1 MPO PRG2 FMOD BMP7 RSPO3 MDK CXCL13 ZNF207 BSPH1 ANG LPL SELP PRSS57 COL13A1 HDGF CRISPLD2 RSPO4 COL25A1 PGF PLA2G5 APOH ELANE LTBP2 GREM2 RSPO1 ANGPTL3 CTGF PF4 PDCD5 COL5A1 SERPINA10 LIPH WISP1 SERPINC1 ABP1 SOD3 PTPRF SLIT1 LPA SLIT3 ADAMTS15 CECR1 FGFBP1 COMP THBS3 CCL7 PCOLCE2 HSD17B12 NRP2 ABI3BP AAMP FGFR4 RPL29 FGFBP3 WISP2 OGN NRP1 FGF9 CXCL11 CCL23 FGFR1 SAA1 REG4 LTF ADAMTS3 ADAMTS8 CEL CCDC80 APOE COL5A3 NAV2 PTN WISP3 GPNMB LAMC2 FGF1 ECM2 PAFAH1B1 CFH POSTN SLIT2 APOA5 CTSG HRG AZU1 RSPO2 CXCL10 PF4V1 EPYC CXCL6 VTN FGF10 SERPINE2 C6orf15 TGFBR3 SFRP1 GPR56 CCL8 FGF2 APOB F11 ZNF146 PCOLCE THBS4 FSTL1 ADAMTS5 SELL KNG1 NOV APLP2 LIPI CYR61 FBLN7 SMOC2 BMP4 TMEM184A CCL2 LIPC FN1 CLEC3B SERPINA5 FGF7 LXN CCL15 ADAMTS1 LIPG MSTN PTCH1 ODZ1 RPL22 C6orf25 APP VEGFB THBS1 KAL1 HBEGF ELSPBP1 CHRD

GO_RNA_POLYMERASE_II_CORE_BINDING Interacting selectively and non-covalently with RNA polymerase II core enzyme, a multisubunit eukaryotic nuclear RNA polymerase typically composed of twelve subunits. C14orf166 SMYD3 WAC EIF2C1 RPRD1B ELP2 SMYD2 ELP3 ZNF326 PAF1 ELP4 IKBKAP EIF2C2 PCF11 ELOF1 KIAA1530 RECQL5 KIAA1967 BRD4 CDC73 CTR9

GO_CYCLIC_NUCLEOTIDE_BINDING Interacting selectively and non-covalently with a cyclic nucleotide, a nucleotide in which the phosphate group is in diester linkage to two positions on the sugar residue. HCN3 HCN2 PDE5A CNGA1 PDE6G PRKAR2B HCN1 BVES PRKG2 HCN4 PDE11A CNGA4 KCNH1 RAPGEF4 PDE6H PDE4D PRKG1 PDE1A CNGB3 C20orf152 PDE2A PRKAR1A RAPGEF2 CNGB1 CNGA2 CNGA3 TMEM173 PDE10A PRKAR1B RAPGEF3 FKBP1B PDE4A PDE6C POPDC3 PDE4B CNP PDE3A PRKAR2A

GO_HISTONE_DEMETHYLASE_ACTIVITY Catalysis of the removal of a methyl group from a histone. KDM3B KDM2A KDM1A KDM6A UTY C14orf169 KDM5A KDM5B KDM2B JMJD1C KDM5D HR PHF8 KDM1B PHF2 KDM4C JARID2 ARID5B KDM5C KDM3A JMJD5 JMJD6 KDM4A KDM4D JHDM1D KDM6B KDM4B

GO_DYNEIN_BINDING Interacting selectively and non-covalently with dynein, the multisubunit protein complex that is associated with microtubules. GLUL NEFH DYNC1H1 SMC3 RAB11FIP3 RILP KATNB1 PAFAH1B1 DYNLRB2 BICD2 PPP1R42 DCTN1 HTT HEATR2 DNAAF1 ATMIN DYNC1LI1 DNALI1 SNCA WDR81 DYNC2LI1 WDR43 SPTBN5 CENPF DYNC1I1 DYNLRB1 RAB7L1 FMR1 BICD1 BCL2L11

GO_SINGLE_STRANDED_RNA_BINDING Interacting selectively and non-covalently with single-stranded RNA. TRA2B DDX58 FMR1 PABPC4 LSM14A CBX8 MSI2 EIF4H LONP1 DDX60 EIF2C4 ZFP36 EIF4A3 U2AF2 SNRPC A1CF FXR1 ADARB2 RBPMS MCRS1 LUZP4 ATXN1 HNRNPA1 IFIT5 TIA1 EIF2C1 RBM11 CBX6 SYNCRIP PTBP1 PNPT1 HNRNPH1 POLR2G DLX2 DDX1 DHX58 L1RE1 STRBP HNRNPC PIWIL1 MTERFD2 EIF2C3 CBX4 ENDOV POLR2D ANXA1 PATL1 MTERFD1 L1TD1 CBX7 IFIH1 DDX3X TLR8 JMJD6 MSI1 TLR7 LACTB2 THRA HNRNPF RBM7 PABPC1 KHDRBS2 DIS3L2 KHDRBS1 NXF1 PABPC3 ZC3H14 EIF2C2 EIF4B HNRNPU

GO_TRANSCRIPTION_COACTIVATOR_BINDING Interacting selectively and non-covalently with a transcription coactivator, any protein involved in positive regulation of transcription via protein-protein interactions with transcription factors and other proteins that positively regulate transcription. Transcription coactivators do not bind DNA directly, but rather mediate protein-protein interactions between activating transcription factors and the basal transcription machinery. TERT ZBTB49 FOXO1 TFAM CCNT2 NFATC1 PASD1 NR4A3 CDK9 PPARA RORA

GO_NUCLEAR_LOCALIZATION_SEQUENCE_BINDING Interacting selectively and non-covalently with a nuclear localization sequence, a specific peptide sequence that acts as a signal to localize the protein within the nucleus. IPO5 POM121C POM121B POM121L2 KPNA5 TNPO2 BRAP POM121L12 NFKBIA KPNA1 KPNB1 CABP1 TNPO3 NUP214 IPO4 KPNA6 KPNA4 C15orf2 NUP98 TNPO1 RANBP6 NUPL1 KPNA3 NUP153 POM121 KPNA7 KPNA2 IPO13

GO_PLATELET_DERIVED_GROWTH_FACTOR_RECEPTOR_BINDING Interacting selectively and non-covalently with the platelet-derived growth factor receptor. LYN ERN1 PDGFD PTEN ITGA5 PDGFRA VEGFA IL1R1 PDGFRB ITGB3 PDGFC PDGFB FIGF PTPRJ PDGFA

GO_PROTEIN_TRANSPORTER_ACTIVITY Enables the directed movement of proteins into, out of or within a cell, or between cells. DSCR3 PGAP2 KPNB1 TIMM23 SNUPN XPO5 TOMM20 XPO4 TNPO2 KPNA2 KPNA7 TOMM40 TSNAX GPIHBP1 C11orf73 LOC100652748 COG3 AP2A2 IPO4 XPO6 TNPO3 IPO8 AP4S1 TOMM22 AP1S2 CALCRL SEC61G TOMM5 NUP214 AP1S3 RAN KPNA1 CHMP7 AP2A1 AZGP1 RANGRF SEC62 RAB4A IPO13 XPO7 KPNA3 COG2 TIMM9 AP3S1 CCT6B TNPO1 USO1 KPNA4 RUFY1 XPO1 CSE1L IPO9 RAMP2 RAMP3 VPS35 SLC11A2 VPS26A NUTF2 TOMM20L VPS29 RAP1A KPNA5 AP1B1 AP4B1 TOMM40L IPO7 NUPL2 VPS26B TIMM10 ITGB1BP1 KPNA6 TIMM17B TIMM17A AP1G1 RAMP1 TMCO6 MLC1 COX18 AP1S1 TOMM7 AP3S2 MCL1 EIF4ENIF1 TOMM70A AP1G2 IPO5 IPO11 AP2S1 TIMM22 RANBP6 CALCR RANBP17 TFRC ZFYVE16 ARFGAP3 AP2B1

GO_PROTEIN_SERINE_THREONINE_PHOSPHATASE_INHIBITOR_ACTIVITY Stops, prevents or reduces the activity of a serine/threonine protein phosphatase, an enzyme that catalyzes the reaction: protein serine/threonine phosphate + H2O = protein serine/threonine + phosphate. PPP1R14D PPP1R17 PPP1R2 PPP1R14B PPP1R1C PPP1R1B PPP1R8 PPP1R1A PPP1R14C PPP1R11 PPP1R14A

GO_NUCLEIC_ACID_BINDING_TRANSCRIPTION_FACTOR_ACTIVITY Interacting selectively and non-covalently with a DNA or RNA sequence in order to modulate transcription. The transcription factor may or may not also interact selectively with a protein or macromolecular complex. RCOR1 ZNF217 ELF3 FOSL1 ZHX1 HMGB2 GLIS2 ZNF35 GTF2IRD2B RFX4 PURB SKOR2 ZNF607 FOXE3 SPIB SOX5 CDC5L ZNF613 FOSB PPARA ZFP28 ZNF354A ZXDA IKZF5 NFYA ALX1 ETV3 GSX1 FOXK2 SCRT1 NFE2L1 TSC22D2 POU4F1 MAX TFAP2A MSX1 TFDP3 ZNF546 NR2F1 ZNF157 SP140 ESRRG POU3F1 ETV1 MEOX1 ZBED6 TAF6 IRF3 SUPT6H FOXS1 ZMYM1 ASCL3 HSFX1 ZNF280D HIF3A SIM2 HIVEP3 REST ZNF470 ZNF200 MYNN HOXB7 YBX1 MSC NKX2-8 POU3F4 ESX1 ZNF551 ZNF420 ZGLP1 TAF1 FOXD4L1 SRF ZEB2 SOX11 ZNF605 DMTF1 ESRRA ZFP36L2 ST18 EN1 ZNF879 DMRT3 ID1 TSHZ2 ZFP30 ZNF16 SALL4 ELK1 NFXL1 IRF2 TBX6 CSRNP2 FOXE1 ZNF655 PAX4 ZKSCAN4 YEATS4 CREB3L2 NR3C2 L3MBTL4 ZFP37 ZFP36L1 RFX8 TADA2A ARID4A RXRG CNBP NR4A1 NFIB LEF1 GPER ZNF234 TBX5 GFI1 SHOX ZNF624 ZNF584 ZNF565 SOLH BCL6 ESRRB NKX2-2 TAF2 ZHX3 HMX1 ARX KDM1A OLIG2 ZNF238 POU2F3 BHLHE41 RXRA ZNF281 TEAD3 YY1 HMG20A ZSCAN1 ZNF354B CITED1 ZSCAN18 ZNF484 ZNF773 RERE CTBP1 FOXD2 XBP1 ZKSCAN5 ZNF461 NOBOX NUCKS1 NFYB IRF6 EGR1 ZNF514 FOXD1 ZNF434 ZMYM4 GTF2H4 FIGLA NFE2L2 PRDM5 IKZF1 MED1 TFAP2B PBX1 ESR1 ZNF586 GATAD2B PPARG JUN ZNF117 CAMTA1 FOXD4L4 ZNF45 JUNB FOXI2 ZNF37A ZBTB7B NR5A1 ZNF23 TRIM22 AFF1 ARNTL2 VSX1 ZNF215 ZNF225 ZNF93 RAX GLI4 NFYC ZNF322 STAT5B CEBPG TADA3 PLAGL2 CREB1 MAFK BACH1 ATF1 NANOG TRERF1 MTA3 C5orf54 ZNF595 ZBTB48 NPAS1 ZNF197 RB1 FOXJ2 DMRTA1 ETV3L IZUMO2 HNF4A ZNF154 MLX BUD31 LHX6 ID3 IRF5 TAF1B SMAD7 HEY1 PLSCR1 TSHZ3 HOXA7 RORA PHOX2B ZNF564 ZFP3 LYL1 SNAI1 ZBTB4 CREBBP RHOXF1 SP2 SNAI3 HNF1A NFIC LMO2 HOXC4 MAFB TGIF1 ZMYM2 FOXL2 NPAS4 TBX21 CSRNP3 E2F6 TBX4 NEUROD6 CRX ZNF446 ZNF789 ZNF334 ZFP82 ETV2 DMRTB1 ZNF175 CCRN4L ZFP112 MYBL1 MSX2 BMPR1A TAF5 MGA ZNF146 RAD21 ZNF641 ZNF750 ELF5 PFDN1 ZNF761 GBX2 NR3C1 ZNF81 PAX1 UHRF1 ZNF71 KRBOX1 ELF1 HOXD10 PATZ1 SOX9 HMGB1 ZFP14 ZNF226 SCXB MAFF BATF2 SKOR1 CC2D1B TSC22D1 ZNF211 FOXN4 TFCP2L1 ZNF74 ZNF831 ZNF616 ZNF138 TAF1L ANKRD30A HOXA4 TCF3 TBR1 MNX1 PTH MEIS2 STAT2 ZNF746 GRHL1 HNF4G CC2D1A EGR2 ZNF419 SALL3 ZRANB2 VAV1 ZNF780B HHEX NEUROD1 REXO4 PAX3 SCML1 IKZF2 RXRB POU6F2 SNAPC5 ZFAT DRAP1 ZNF302 ZNF516 ZNF30 KIAA1958 MSGN1 INSM1 TBX15 NPAS3 KLF16 MYOCD ZFP42 ZNF415 ZNF561 GOLGB1 C12orf28 RARB C2orf3 SLC2A4RG FOXA3 FOXF1 SCML2 ZBED4 MTF1 HOMEZ ZNF548 RFXAP DDN ZNF140 ZNF345 CEBPE PITX2 HSFY2 SOX6 NFKB2 CDX4 POU3F3 ZNF121 CNOT8 BARHL2 RCOR3 IRF4 VAX2 SMAD4 ZNF91 MYBL2 MYPOP ZNF445 TFEC FOXO3 POU5F1 MEOX2 PAX6 SCRT2 MESP1 NPAS2 POU2F2 TP73 ZNF518A SMAD5 ARHGAP35 GRHL2 USF1 PHB DLX3 CTCF C11orf9 NFKB1 ZNF496 BATF3 ZNF835 SREBF1 MTA2 ARNTL CEBPZ DMRTC1B NR2E3 SOX3 KCNIP3 PCBP3 ZNF880 PTF1A ZNF263 ZNF696 SIM1 TBX18 ZHX2 ZSCAN5C TAF9B IRF7 SMAD6 NFE2 TTF1 STAG1 DMRTC1 HOXD9 KLF12 NR0B1 GCM2 ZNF182 ZNF573 TBX2 ZNF284 JDP2 ZNF711 RORC SOX4 ELK3 NFX1 NFATC1 C1orf85 GABPA FOXR1 HELT SPI1 SKIL GSC2 HES5 HIF1A E4F1 ZKSCAN3 TAF4B NOTCH1 EBF4 BCL3 ETV7 ZNF649 AIRE SOHLH2 MYC TLE4 PARK2 FOXD4L6 HOXA5 ZNF541 NACC2 ZNF691 USF2 NEUROG3 STK16 SATB1 SMAD9 SCAND2 AFF4 HIRA GPBP1L1 ZSCAN30 DMRTC2 MZF1 ISX ZNF165 MNT MLL4 RBPJ TEAD4 TMEM229A FEZF1 AHCTF1 MTA1 E2F2 BHLHA15 ZNF132 POU5F2 PAX5 ZIC3 PLAGL1 ZSCAN4 BHLHE22 CREBZF SOX12 EGR4 TBX3 MYF6 ASCL1 ZNF726P1 ZNF92 FOSL2 HNF1B CLOCK ZBTB17 FOXO4 NR1H2 FOXG1 MEF2B PRRX2 EBF2 ZNF268 MAFA ATF7 ZNF19 OVOL3 ZNF286A NFAT5 ZNF8 NKX2-1 PA2G4 ZNF131 ZSCAN21 ZNF544 ZBED5 PCGF2 KLF4 TGIF2 ZNF568 MEF2C MXD1 TCF7L2 BLZF1 TAL2 RELA ELK4 L3MBTL1 GATA2 RAI1 ZNF133 ZNF454 ARID5B PKNOX1 KLF5 PPARD ZNF726 ZNF224 TSHZ1 TRIM28 HOXA10 KLF10 ALX4 TFAP2E SOX7 ZNF83 RELB MECP2 PROX1 ZNF275 NFIL3 ATF6 MITF GATAD2A TCF12 PBX4 GLI1 ZBTB38 BCL6B SOX13 ZNF585A ZXDC ZFP1 ATOH1 ZIM2 ZNF41 HIVEP2 UBP1 TFAM ZNF167 EP300 RUNX2 HIVEP1 ZFX NRL JUND LHX1 ZNF33B KLF1 PRDM2 FOXP4 TRIM25 ARNT2 RUNX3 ZNF280A AEBP2 DMRTA2 HOXC13 ZEB1 CHCHD3 RBPJL GABPB1 ZNF205 ZSCAN16 ZNF841 MYB CGGBP1 TADA2B TFAP2C ZNF500 ZNF772 SIX3 ZNF26 ZBTB25 DMRT2 PDX1 PAX8 CAMTA2 NR2E1 KLF13 ZMYM6 HMGA1 ZNF611 NFE2L3 HOXB2 NKX6-1 ZNF286B FOXA2 ZBTB7A ZNF192 PHF1 MEF2A HOXD8 ZNF615 HOXB3 NR1D2 TP63 TBX19 FOXN1 ZNF135 ZNF235 PBX3 ZNF75D ZNF540 NR2C1 MEF2D ZKSCAN1 GATA5 SNAPC2 HDAC1 EBF1 ZNF304 ZFHX3 LZTS1 IFI16 NR4A3 ZNF658 FERD3L DLX4 SNAI2 RCAN1 SOX14 ATF2 ZNF3 POU1F1 KLF7 ZNF429 MEIS1 AHR KDM5A ZNF790 PHOX2A DBP PITX1 BTG2 LMO4 ZNF213 MIXL1 TARDBP EVX1 CEBPD LMX1B FOXJ1 POU2F1 GPBP1 HSF1 GATA3 NR1D1 ZNF323 HOXA3 C5orf41 NHLH1 ZNF134 IRF1 SIN3A HOXD13 BCL11A C14orf43 ZNF202 TAF13 ZNF629 NME2 OTX1 SALL1 CTNNB1 KDM5B SNAPC4 TFE3 FAM200A PLAG1 RARA ARID3C ZNF267 TFAP2D ERG BNC1 ZNF668 ASCL5 ZNFX1 ERF IKZF3 ETV5 CSRNP1 NKX2-6 ZNF623 FOXB1 EBF3 OVOL1 ZNF493 ZNF532 ZSCAN29 FOXD4 HAND2 ZGPAT ZNF542 MYCN ISL1 KLF15 ZNF549 HES1 FOXA1 TAF12 HSF4 ZNF571 SLC30A9 ZNF782 TRIM29 ZNF600 NRF1 ZNF187 ASCL4 ZNF227 ZNF780A NR1H4 ZNF728 ZNF480 E2F4 DEAF1 ETS1 FOXC2 TAF7 CTCFL SPIC FOXC1 STAT6 RFX6 POU6F1 NR2F2 FOXQ1 CREB3L4 ZNF432 SMAD2 FEZF2 FOXH1 TSC22D4 CBFA2T3 ELF2 ZKSCAN2 SMAD3 ZSCAN10 ZNF699 FEV SP1 ZNF239 GATAD1 NR1I3 ATOH8 TWIST1 MYOD1 PCBP1 CREB5 HLF STAT4 ZFPM2 ZBTB11 KLF3 GTF2IRD1 PGR BSX ZNF280C TEAD2 TCF21 ZSCAN22 ZSCAN5B LZTR1 TLX1 ZNF254 SIX1 ZNF717 PGBD1 ZNF391 ZNF75CP BATF GATA1 PAX7 ZNF274 ZNF207 RREB1 OTX2 SCAND3 AATF FOXM1 ZSCAN2 GCFC1 NFIX ZNF621 LOC100293516 ZNF829 SP3 HSFY1 GLI3 GAS7 C7orf29 HSFX2 TCF20 ZNF354C POU4F2 ZNF155 HES6 ZNF251 EPM2AIP1 ZNF383 TFCP2 DDIT3 ELF4 PROX2 MYCL1 HLTF ARID3B TEF ZNF444 NHLH2 ZNF501 NR1I2 E2F5 ZNF250 ZNF639 TBX22 ZNF341 ZNF490 ZNF12 DLX6 FOXK1 TCEAL1 ZNF280B SIX6 SKI SCMH1 FOXF2 FOXP2 HCFC1 SOX21 JARID2 FOXD4L5 MLXIPL ATF5 NR6A1 ZNF770 CBFB SP4 ETS2 GCM1 AR ZBED1 ZNF382 RFX3 ZNF483 ETV6 MYF5 UBN1 KLF2 EAF2 FOXB2 HOXB8 PRKRIR HINFP ONECUT1 FLI1 E2F8 HOXC6 NDN ZNF630 MLLT10 ZNF394 HOXD4 KAT7 ARID3A NKX3-2 SPZ1 ZNF397 SLC26A3 ZNF567 SUB1 TBX20 PITX3 TBP FOXJ3 FOXI1 BTAF1 SOX8 DACH1 VAX1 HIC1 NEUROD2 ZIC2 ZNF793 PTTG1 FOXL1 SCAND1 FOS ZSCAN5D STAT3 PAX9 MECOM GATA4 ASCL2 ZFPM1 ESR2 ZNF534 ZNF18 FUBP1 TEAD1 TAF10 ZNF90 FOXD3 STAT5A BACH2 DMBX1 BTBD8 ZBTB16 AKNA ZNF792 ZIC1 RUNX1T1 EDF1 ZNF451 ZNF585B ZNF367 ZNF85 GLIS1 HOXC8 DLX2 TXK GZF1 KLF11 ZNF350 ZFP2 PBX2 RORB ZNF570 RUNX1 ZFP90 TULP4 T ATF4 SIX4 DMRT1 RFX1 ZNF677 ZSCAN5A TRPS1 QRICH1 ZNF808 FOXD4L3 RFX7 TCFL5 IRF8 GMEB1 STAT1 SOX18 TFAP4 ZNF79 ZNF449 TFEB TCF7 FOXN3 NEUROG1 SOX2 ZNF320 SOX10 ZNF300 FOXN2 HOXC11 GTF2H2 TAF5L EGR3 ZNF643 RCOR2 HNRNPAB MKL1 NR2C2 FOXO1 HOXB4 SUPT4H1 ATF6B NR5A2 ZNF888 CITED2 CIR1 IRF9 PEG3 ZSCAN23 ZMYM5 NFATC3 TFDP2 CNOT7 ZNF471 PRDM1 SRY ZNF260 CBFA2T2 ZNF43 EHF ZNF396 KLF17 RFX2 ZNF552 ONECUT2 RFX5 GTF2I MYT1L TBX1 EOMES NKX6-2 RARG TFDP1 KDM3A NFATC2 BCL11B ONECUT3 HMGA2 FAM200B CEBPA HEYL ZMYM3 HOXC5 REL NR2F6 TAL1 ZBTB20 RFXANK THRB SOX1 MAF SREBF2 TAF9 ZNF7 MSRB2 HNRNPK HOXD3 ZIM3 TCF25 ZNF436 POU3F2 IKZF4 CREB3L3 NKX2-5 ZNF28 DLX5 CDX1 BHLHE40 ETV4 ZNF836 HSF2 NFIA NKX3-1 SIX2 PCGF6 ZNF189 GRHL3 FOXI3 CBL TCF15 AEBP1 ZNF606 ZNF193 CDX2 HOXB5 ZNF816 CUX2 ZNF229 ZNF283 E2F1 ZNF311 CREB3 MLL TCF19 HOXB6 ALS2CR8 BARX2 ZNF658B ZNF232 HAND1 ZNF577 ZNF583 ZFY NFATC4 ZNF821 ZNF518B SPDEF ZNF355P ZC3H8 HR ZNF528 HEY2 HSF5 TCF4 TBX10 POU5F1B MYT1 ZNF24 FOXP1 SALL2 HOXA2 ZNF73 GTF2H3 NR1H3 SMAD1 MLXIP TBPL2 PURA THRA CSDA MYOG ZNF660 CREBL2 OVOL2 EPAS1 ZNF883 ZNF814 ZNF317 OSR2 RNF4 MAFG ZNF536 ZNF219 ZIK1 ATF3 FOXO6 KLF9 POU4F3 TSC22D3 ZSCAN20 PROP1 ZNF287 ZNF498 PHF5A SOX17 ARNT BARX1 ZNF418 E2F7 TP53 TCF7L1 FOXP3 ZNF221 DACH2 E2F3 GATA6 ZNF837 PRDM15 ZFP161 BARHL1 ZNF33A HOXA6 GLI2 FUBP3 WT1 ZNF148 ZNF223 CEBPB CREB3L1 ZNF256 ZNF174 ZNF628 MEIS3 SOX15 WNT5A BRD8 NR4A2 CREM ZNF2 LBX1 VDR PHTF1 ZNF681 SOHLH1 ZSCAN12 TAF4 ZNF292 FOXR2

GO_RNA_BINDING Interacting selectively and non-covalently with an RNA molecule or a portion thereof. PCDH20 DUS3L NSA2 C16orf88 HIST4H4 AKAP1 ZCCHC17 DHX16 YTHDC1 SRSF9 MYBBP1A RBM6 KIAA1456 OASL FSCN1 HNRNPUL1 CSTF1 SNUPN RG9MTD2 HNRNPA2B1 PABPC3 RBFOX3 SARNP CS PNISR EMG1 ELAVL3 PIWIL4 XPOT PTCD3 MRPL41 IGF2BP2 MBNL3 ADAD2 TSN MRPL14 UBE2I STAU1 XPO1 RBM47 FARS2 HSPA8 MRPL4 SPI1 SMG1 DDX41 SLFN11 NFX1 TFB1M PHF6 C15orf52 TOP3B EEF1A2 PAPD5 NXF2 ETF1 FASTKD2 NR0B1 LARP1B EIF2AK2 KIN PATL2 MOV10 AKAP8 SSB EIF2S2 DDX23 SPATS2L HNRPLL NCBP1 TLR9 CHCHD1 TBRG4 YTHDC2 STRBP C1orf35 RBM27 CPSF4 IMP4 CDKN2AIP ATXN2L PCBP3 DIEXF H1FX CELF5 MRPL22 GFM1 CEBPZ CCT3 ZNF326 HIST1H1C DDX39B SRRM2 HIST1H4K RPL12 C2orf15 A1CF OTUD4 EIF3M PRPF39 XRN2 MRPL16 KRT18 BMS1 RPS21 MAK16 KRR1 EIF2AK4 UTP18 ZNF346 RPL4 LACTB2 G3BP2 EIF3E PABPC1L2B RPF1 IFIH1 TLR8 RBM25 RPS4Y2 TARS RBM18 TMSB4X POU5F1 PEG10 ZFP106 DUSP11 PPIB AKAP17A AFF2 ENO1 CNOT8 EYA1 ARF1 UBA1 RNASEH1 RPS5 H1F0 LOC100130932 LSM4 XIRP1 PARN DHX15 WDR6 SRP19 SLC25A5 PAPOLG PRPF3 VCP RBM12 WBSCR22 SBDS BOLL TOP2A EIF4E3 GOLGB1 EIF3I POLR2G DDX49 RTN4 EIF5A RPLP0 NOC4L EXOSC10 ZBP1 AARS2 TUFM TNRC6B SLC16A3 BST2 WRAP53 HERC5 RPL39P5 ADARB2 DDX18 NOL8 EIF4G2 SRP72 CCT6A NUDT1 NKRF REXO4 GRB7 EIF2C4 FYTTD1 RBMXL3 CRNKL1 DDX51 ZRANB2 CTNNA1 ERAL1 USP36 FBL ZC3HAV1 RBM43 PPP1R10 LSM1 GLRX3 SUGP2 DDX27 NOM1 SAMHD1 KIAA0020 PRPF6 SRSF7 CWC22 IPO5 PRPF8 UTP6 ZNF579 CPSF6 VSIG8 SCAF11 RPS15 TSNAX MBNL1 MSI1 RBM20 HIST1H1B CTU2 AHCYL1 ISG20 FARSB RBMS2 ZC3H4 SNRPD2 PPRC1 APOBEC3G DHX37 ADD1 GLTSCR2 MKRN2 RPN1 GNL3L RBM28 PCSK9 RPL32 ANXA2 IMP3 EIF1AY C1QBP RPL9 LSM3 TIMM50 RPS3 MTDH WIBG RPS11 KCTD12 RPL10A KHDC1 RANBP2 SMN2 NOA1 PDIA3 NOL4 NSUN2 DHX36 DND1 CDC40 SRRT CDK13 FAM133B EEF1D HNRNPH1 RRP8 C17orf85 NOL6 TRIM25 CBX6 DYNC1LI1 FLNB DHX32 DHX35 DNTTIP2 C17orf42 FASTKD5 TERT DZIP3 S100A4 BICC1 NUFIP2 NSRP1 PTBP2 ZYX YARS2 ZRSR2 GTPBP4 HIST1H1E R3HDM2 NQO1 CCDC124 RPS10 PURG SON EP300 NHP2L1 TFAM C9orf23 RPS27A SRSF10 LSM14A WDR43 ADK YRDC UBE2N PARP12 EIF5B TRIM56 APOBEC3F EIF1AX DUSP14 PARS2 MECP2 TRIM28 DDX55 TRMU PNN DDX59 MAGOHB LSM7 EIF1B SERPINH1 PUS10 HEATR6 BAZ2A RPL26 EIF6 TYMS MEF2C RDX RDM1 PDCD4 MOV10L1 CKAP4 CNOT6 MYEF2 SRSF2 BCCIP RARS2 RPS14 NAP1L1 RNPS1 ERVK-16 CSDC2 RCL1 CISD2 CCDC108 MARK2 PA2G4 SRP9 ATP5C1 MRPL15 FAM32A RPL21 GPATCH8 ZFC3H1 SART1 THUMPD3 PATL1 HSD17B10 TST TDRD10 MRPS26 WBSCR16 MRPS5 EIF3CL SETD1B EARS2 NSUN5 HNRNPCL1 DDX53 CD3EAP TARBP1 RPLP2 FBRSL1 MRPL28 TRUB2 DAP3 NOL7 TRDMT1 BYSL EIF3H UBAP2L METAP2 ADAD1 MORC3 MRPL45 MLL3 TDRD3 RBM23 DHFR SNRNP70 PRPF40B RPL13A SNRPE DHFRP1 PRRC2C EIF4E DUT GRSF1 LSM10 DHX9 DCN TPT1 PPAN FMR1 ERI1 EIF3A SNRPN NOVA1 DDX11 DOM3Z RPL15 RPUSD3 POLR2A GANAB RPL13 NSUN7 SUMO2 DDX25 THOC2 RPS4X YY1 HSP90B1 LSM2 GDI2 MEPCE RAVER1 EIF3J SYNJ1 GNL3 MRPS12 KDM1A HNRNPU TCERG1 RPS4Y1 RRP1 PABPN1L MRPL13 DDX50 RBMS1 HUWE1 CCDC165 FAM98A RBM5 STIP1 EXOSC8 RPS2 TUT1 TUBA1B MACF1 USO1 TBCA ZNF638 MRPL40 CNBP EXD1 GRN ZFP36L1 CHERP LSM5 YTHDF1 R3HDM1 MTIF2 C6orf221 RRBP1 RNPC3 THRAP3 BCLAF1 TRMT1L UNK SND1 ICT1 SREK1 HEXIM1 GTF2F1 C1orf52 SRP54 PES1 PTCD2 PTRH1 KHDRBS3 ATXN2 YARS ISY1 C14orf166 PEF1 ZFP36L2 SYF2 DUS2L KIF1C PRPF40A ZNF598 PLEC CPEB1 SOX11 SNRPD1 HSPA9 ALDH6A1 DIS3 C11orf68 SERBP1 MRPL44 SRF EIF4A3 EIF2S1 RPS27L ZCCHC9 C16orf80 APTX GIGYF2 RIMS1 MTO1 FDXACB1 NUDT4 MSN SIDT2 SART3 PCBP2 ALKBH8 SAMD4A THOC3 OAS2 PABPC1L2A CPSF4L NUP98 MRPL3 DCD DHX40 CBX7 IGF2BP3 YBX1 EIF2B5 EEF2 GRWD1 IMPDH2 WDR75 LRRC59 FASTK THG1L FDPS AKAP8L PRPF4B LGALS1 RPS27 SEPSECS PSMD4 LAS1L SRSF8 PRIC285 CAPRIN1 ABTB1 RPS15A RC3H2 GEMIN5 BZW1 RPLP1 EIF2A SEC63 SUPT6H PARP1 METTL1 ESRP2 RBMX2 ASCC3 SARS RPGR API5 CNOT1 HRSP12 XRCC6 CASC3 REPIN1 NUDT5 EXOSC1 CDC42EP4 RNMTL1 RPL23A PNPT1 ESF1 CPSF1 PCBP4 RPP25 PABPC1L CHTOP NSUN5P1 SUMO1 DAZ1 ZCCHC11 NOP14 CDC5L DHX29 ZC3H7B SSBP1 CLTC RPL36 PAPOLB WBP11 MATR3 LGALS3 KHNYN KIAA0664 PURB MRPL18 DHFRL1 EPRS RAE1 FASN DHX57 UPF2 TRA2B SLC25A11 ADAT1 EIF3K COL14A1 ANGEL2 HMGB2 NVL PFN1 PTPN1 HBP1 ZNF74 HIST1H4D CELF2 NKAP KPNA2 SF3A3 RNMT RBBP6 KRI1 FNDC3B EIF3F RPLP0P6 RPL36A LUC7L3 ZC3H18 NDUFV3 HMGB1 SPTBN1 SOX9 MYO18A RBFOX1 ALG13 SRPR SMN1 CELF4 XPO5 ANP32A NR3C1 C19orf29 CLK3 SRRM1 NANOS1 RBM4 PTCD1 SRSF6 RBPMS2 PKM2 RNF20 TSFM DDX24 C1orf131 THOC1 CCDC86 CCRN4L MRPS28 MRPL20 DENR ALDOA HSPB1 MANF FNDC3A RPL19 LSM11 RPS6 BTF3 GTPBP1 FAM120C RPS19BP1 EIF4E2 PAPOLA ERH DHX58 RPS19 EPPK1 NGDN RBM10 CCBL2 PRPF4 RPL27A SECISBP2L EIF4G1 GCN1L1 MRPL12 LARP4B PABPC4L EIF4ENIF1 MTRF1L CCAR1 ABT1 PNO1 NTPCR RALYL EIF2B1 METTL14 MFAP1 NCBP2L RPS25 MRPS21 PSMC1 RPS26 SMC1A NHP2 DDX47 UBC HNRNPM PPARGC1A PAN3 TRIM32 PPIA MBNL2 DDX3X MRPL27 SLTM SPATS2 APOBEC3C DHX33 RPL31 DBR1 EIF2C2 RPS12 MTRF1 DCP1B DHX8 CANX MYH9 FXR2 S100A16 DGCR8 EEF1A1P5 EIF4A1 CPSF2 QKI GNL2 RPL39L RBM14 UTP15 FTSJ3 SEC61B RFTN1 RPSA PRR3 YTHDF3 AIMP1 MAGOH SMG7 SCAF8 EIF4G3 SSRP1 ZMAT4 C4BPA RBFOX2 DCPS RBMY1J NUPL2 JUN CSDE1 PAIP2 DMGDH BRCA1 DARS MARS BAG4 U2AF1L4 APEX1 EIF2D UTP11L EXOSC2 SEC23IP SF3A2 L1RE1 HIST1H4J SAMSN1 NUDT16 DAZ4 SNRPB2 NOSIP SUZ12 MRPL11 HMGN5 DDX54 MRPS15 SRSF12 DDX58 PIWIL2 ZC3H15 MTIF3 NUCKS1 SNRPB DDX46 SNRPD3 SNRNP40 PSPC1 HNRNPA1L2 EBNA1BP2 AUH SNRPA1 HSPE1 HNRNPF OAS3 NAA38 HNRNPR SFSWAP JMJD6 HSP90AB1 EFTUD2 NXF2B RPUSD1 GRB2 GNB2L1 LIN28B KTN1 ABCF1 MRPL39 PIN4 RBM15B KHDRBS1 ANXA1 LYAR ZCCHC7 NOVA2 ANXA7 G3BP1 DDX39A IFIT1B MRPL10 HIST1H4C CORO1A RAN UBE2O IMPDH1 C1orf31 MRPS31 IGHMBP2 TCP1 NUDT16L1 IREB2 RBMXL1 SF3B2 RPS16 GTF3A LIN28A DIMT1 HIST1H4E RPL3L PTBP1 CPEB4 UTP20 RPL7L1 POLDIP3 TROVE2 SYNJ2 CNOT7 EWSR1 FXR1 RRP36 HNRNPC PUM1 TCOF1 MIF4GD DIDO1 PRKRIP1 RC3H1 DDX1 UPF3B CBX8 DDX3Y ALDH18A1 SRBD1 EEF2K FAM50A RPL22 HENMT1 CSNK1E NME1 PUSL1 NOP2 HNRNPAB CDK11B GTF2H2 POLRMT RPL18 ELAVL1 TLR3 MAPRE1 SOX2 TBL2 ELAC2 RPF2 NLRP11 KHDRBS2 ZMAT3 UTP3 PABPC1 CCNT1 DDX56 ZNF385A NAP1L4 TRUB1 KIAA0430 STRAP PRPF38B MTHFSD TOP1 EIF2S3 RPL28 THOC7 ANXA11 THUMPD2 PUS1 CBX4 SNRPG USP10 C11orf31 SUPV3L1 HLA-A MTERFD2 ZCRB1 RPL7A CPEB3 FBLL1 EXOSC6 HDGF CELF3 EXOSC7 THOC6 ELAVL4 ANG TARS2 DNMT1 ACAA2 SNRPA ROD1 DQX1 LARP6 RBMY1B TWF2 SARS2 GTPBP2 DLX2 TRAP1 CNP SRP68 RBM33 EIF2C1 EDF1 APOBEC1 RPL6 PWP2 CDK9 TIAL1 MEX3C PUM2 SNW1 ZMAT1 CCT4 RPS9 EXOSC3 UBTF RPP30 RBMY1F PCDHGA9 FUBP1 RPL29 ESRP1 RBM8A EIF4H STXBP1 PELP1 C14orf21 MKRN1 NCOA5 IGF2BP1 ZCCHC3 SRSF3 DSP SRSF11 YBX2 NOP58 CSTF3 U2AF2 RPL18A EEFSEC DAZL CCNT2 DHX34 EIF4E1B NANOS3 UPF1 FKBP4 TEP1 TLR7 YWHAG HIST1H4I SAMD4B PABPC5 NUDT21 MRPL54 AARSD1 HIST1H4H L1TD1 GOT2 SAFB CXorf57 LARP1 PSIP1 HEATR1 MRPS9 IMMT CSRP1 PRPF38A FAU ZMAT2 ZNF385D FRG1 RBM12B NAA15 ADAR MCAT HIST1H4L WT1 FUBP3 GAR1 CSTB SNIP1 ACIN1 SUGP1 RPL11 MKI67IP MTERF RAD51AP1 WDR3 RBM22 PPP1R8 ERVK-6 SLIRP LOC440563 HNRNPA3 KIAA1324 CAPRIN2 RCC2 EIF4A2 PRRC2A LLPH HNRNPL RBPMS RPL27 UBAP2 NPM3 NOL10 SF3B14 TRIM21 RBMXL2 PPHLN1 LRPPRC MEX3A IFIT1 PHF5A RPL23 CAST RBM42 OBFC2A THOC4 ENDOU RPS7 HSPD1 HELZ COPS5 SCAF1 PUS3 FLNA TRA2A MRTO4 MKI67 PEBP1 DDX12P EXOSC9 FIP1L1 TYW5 TPR CPEB2 RNASEL DAZAP1 MEX3B NOL12 XRCC5 ZFP36 TNRC6A DDX5 CSDA CRKL THRA RPL38 RBM7 PURA HNRNPA0 FASTKD1 DCAF13 SMAD1 EIF5A2 PARK7 GTF2H3 ILF2 SF3B4 HIST1H4A MRPS11 RPUSD2 RBM44 SNRNP35 HTATSF1 RPL17 RPL35A ZC3H13 ZAK NANOS2 CSTF2 METTL16 PRMT1 ZC3H8 MDH2 SLBP RPS3A NOL9 UNC50 SPEN TDRD9 KIAA1429 SRP14 APOBEC3B CALR URB1 RPS17 TRMT2A SRPK1 C22orf28 MRPS24 RPL35 HOXB6 CHD3 ARL6IP4 PSTK RBMX RNF40 SLU7 YWHAE PPIL4 DDX52 RBM41 RG9MTD1 MPHOSPH6 RBM24 PKN2 BUD13 ILF3 EZR PRPF31 FCF1 FASTKD3 SCAF4 MCRS1 EZH2 P4HB LUZP4 U2SURP FUS NOC2L ATP5A1 RPL5 PUS7L CTU1 NIP7 CPSF3 HMGN2 OOEP LARP4 TFB2M MRPS35 DDX19A DDX60 EIF3G GTPBP10 MSI2 MRPS6 C1D EIF1AD TUBB4B MTPAP LUC7L RGNEF SUCLG1 CRYZ MRPS23 NUDT7 YLPM1 QARS PDIA4 PAIP1 METTL3 PSMA6 HNRNPK RPL24 AQR NBPF10 SRSF4 PRDM14 HIST2H4B SKIV2L HMGB3 ERI3 DROSHA CCDC137 ROCK2 RBM19 C9orf114 ZNF622 PHAX NSUN4 SURF6 SCG3 NPM1 RNASEH2A NMD3 LTA4H HNRNPUL2 AHNAK KPNB1 MRPL21 RPS8 HBS1L EIF3L MYO5A ZC3H3 RBM26 ACTN4 CELF1 HDAC2 PPARGC1B FAM208A PRDX1 POLR2B ADARB1 ZFR SNRNP200 SF3A1 RBM34 MRPL9 TRNAU1AP RBBP7 WDR33 SUPT5H RPL14 HEXIM2 POP7 GFM2 SETD1A SRSF1 SRPK2 SF3B3 C7orf64 TAF15 RPL7 ATXN1L IFIT5 RSL1D1 ZNF385B MRPL43 TNRC6C ZC3H11A NOP10 NOC3L SRSF5 CARHSP1 DYNC1H1 LARP7 ZC3H7A NXF5 RBMS3 ZNFX1 EEF1G UHMK1 RRP7A RPL37A EIF3D RRP1B ZC3H10 PABPN1 RPS20 TFRC ZRSR1 RARA DCP1A DHX30 PRRC2B RPS23 DNAJC21 DDX31 MCTS1 UBE2L3 DDX19B TDRKH DIS3L2 NSUN6 SNTB2 BARD1 FAM103A1 SIN3A RNASET2 RBM38 RRS1 SLC3A2 ZNF385C HADHB MRPS14 MPHOSPH10 EIF4B PIWIL3 ZCCHC8 KDM2B IFIT3 EXOSC5 KHSRP TRMT6 ZNF768 POLR2D RARS TNPO1 TARDBP HARS2 TNS1 NOP16 LRRC47 NXF3 ACTN1 DDX60L NOL3 EIF2C3 EIF1 RPL10 TRIP6 CSTF2T LSM14B RPS24 C12orf65 DICER1 RBM15 SFPQ HSP90AA1 SAP18 IFIT2 DIAPH1 NUSAP1 DDX21 EIF3C RPL8 ISG20L2 HNRNPH2 FAM120A TIA1 YTHDF2 SRFBP1 AARS FKBP3 CHD2 MRPL23 C14orf93 IFI16 DAZ3 THOC5 MRPL42 MRPL37 PET112 SMARCE1 EXOSC4 LOC649330 RBM4B RPUSD4 MYH4 YWHAZ ASS1 LONP1 APEH MRPS30 PABPC4 SAFB2 IBA57 TSR1 PPP1CC C7orf44 CPNE3 EIF3B SNRPC STAU2 RPL41 TPD52L2 DDX43 CCDC47 ANKHD1 RAVER2 TXN RBM39 MRM1 DDX6 SRRM4 LBR FARSA UCHL5 RRP12 HDLBP POP4 RPS13 TARSL2 IARS PCF11 DNM1 NCL DCP2 HIST1H1D SMNDC1 LUC7L2 SMG5 ALKBH5 EEF1E1 SUB1 NXF1 DDX42 PUS7 ENDOV PPP5C RBM46 EIF2B3 SMG6 GSPT2 UTP23 RBM45 PSMA1 PINX1 UTP14A EIF5AL1 CTIF SNRPF PRKDC MRPS7 CLNS1A HELB SF3B1 NAT10 RPL30 CDKN2A CIRBP DEK CARS IARS2 RBM11 UBFD1 HNRNPA1 BOP1 NSUN5P2 VIM KARS DNAJC2 PRKRA RBMY1C ANKRD17 SKIV2L2 ZCCHC6 ARHGEF1 BRIX1 HIST1H4F DPPA5 NCBP2 EEF1B2 APOBEC2 HIST1H4B SECISBP2 TRMT1 EIF2B4 XRN1 DIS3L RTCD1 RDBP RP9 RPL39 ARCN1 CELF6 MRPS17 NOP56 TRNT1 LRRFIP1 TBL3 CPSF7 FLYWCH2 CCDC59 SUPT16H NOLC1 MBD2 PPIG THUMPD1 ZC3H12A HLTF MEX3D DDX10 DHX38 FAM98B DKC1 RPS18 JAKMIP1 TRMT11 AGFG1 CIRH1A ZC3H14 CNOT4 EIF2B2 DNAJC17 TRIM71 KIAA1967 GSPT1 TCF20 C3orf26 NGRN AGGF1 FAM46A GTF2E2 ELAVL2 NAF1 CWC15 OAS1 C19orf66 C7orf50 RPL37 DDX28 PIWIL1 RPL34 EDC3 AATF NOL11 CCDC9 MTERFD1 MRPL32 DDX17 ZNF207 NUFIP1 PUF60 TES RRP9 RSRC2 TDRD7 SYNCRIP WDR36 EEF1A1 PDAP1 SYNE1 MAP4 ATXN1 MRPL1 ACO1 ZCCHC24 SORBS2 HNRNPH3 LSM6 SF1 PTRF MRPL2 HNRNPD HIST2H4A NONO KIAA0907 MAZ RTF1 PCBP1 RBM17 LRP1 DARS2 HNRPDL UPF3A RPS28 GPATCH4 UBR5 RPL3 RCAN3 RALY PPIE EIF5 ZNF239 POP1 LOC81691 WDR46 LOC255308 TARBP2 PDCD11 NSUN3 RBM3 ASCC1

GO_TRANSFERASE_ACTIVITY_TRANSFERRING_HEXOSYL_GROUPS Catalysis of the transfer of a hexosyl group from one compound (donor) to another (acceptor). CSGALNACT1 POFUT1 FUT3 GALNT5 MGAT4C LOC152586 B4GALNT2 HYAL1 GYS1 HAS2 GALNT10 DPY19L3 MGAT4A B4GALNT3 HAS3 B4GALT5 GCNT4 WBSCR17 UGT2A3 B4GALT3 C3orf39 B3GAT1 PLOD3 B4GALT4 ALG10B MTAP B4GALT2 FUT11 DPM3 A4GNT B3GNT6 B3GNT1 AGL B3GALT5 B3GALT2 RPN1 B3GNT7 FUT8 PIGA FUT10 UGT1A7 MFNG CHPF C15orf58 GALNTL4 ALG13 FUT6 FUT4 LARGE GYG2 B3GALT1 ALG9 ALG2 GYS2 GYG1 STT3A MGAT5B MGAT2 EXTL2 GCNT2 B3GALNT2 GALNT12 HEXB MGAT3 DDOST WDFY3 PIGM PIGP C1GALT1C1 CHPF2 B3GNT9 GALNT3 GALNT4 B3GNT5 UGT1A5 CHSY3 POFUT2 ALG8 GCNT1 ALG12 GBE1 GALNT7 KDELC2 CSGALNACT2 B3GNT2 UGT1A6 GYLTL1B ALG1L2 EXT1 GALNT13 HAS1 B3GALT6 EXTL3 UGT2B17 DAD1 UGT1A3 UGT2A1 EXT2 GALNTL2 ALG5 PYGM GALNTL1 STT3B GALNT8 ALG6 B4GALT6 ALG10 UGT2B28 LOC100288842 GLT25D1 B4GALT1 A4GALT UGT2B4 POMGNT1 UGT2B10 EXTL1 GCNT3 DPM1 ALG14 UGT8 FUT9 TUSC3 LALBA OSTC ALG11 LFNG XYLT1 RFNG PIGH ALG1 GGTA1P GALNT6 A3GALT2P B3GALNT1 OGT DPY19L4 HEXA UGT3A2 UGT1A10 GLT25D2 UGGT2 ALG3 RPN2 TYMP UGT2B11 B3GAT2 DPY19L2P2 POMT2 SDF2 PIGV B3GAT3 PIGQ KDELC1 DPY19L2 PIGC MGAT1 B3GALT4 POMT1 DPM2 GALNT1 GALNT9 UGT1A1 C1GALT1 GCNT6 MGAT4B FUT2 B4GALNT4 GCNT7 UGT2B7 ABO UGGT1 GALNT14 UGT1A9 GLT6D1 UGT1A8 POGLUT1 PIGB DPY19L1 B3GNT3 C3orf64 MGAT5 GALNT2 FUT5 FUT1 XYLT2 CHSY1 PYGB PYGL PIGZ GALNTL6 CCDC126 UGT1A4 B3GNT4 SDF2L1 B4GALNT1 B4GALT7 UGCG FUT7 B3GNT8 UGT3A1 ALG1L GALNT11 UGT2B15 GBGT1

GO_METAL_CLUSTER_BINDING Interacting selectively and non-covalently with a cluster of atoms including both metal ions and nonmetal atoms, usually sulfur and oxygen. Examples include iron-sulfur clusters and nickel-iron-sulfur clusters. POLE ERCC2 LIAS FDX1 FXN DEM1 FECH DNA2 GLRX2 NDUFS1 REV3L CISD2 RSAD1 ABAT NDUFS7 NDUFS2 ISCU RPS3 BRIP1 MOCS1 XDH ETFDH POLD1 IREB2 TYW1B ELP3 NDUFV1 AIFM3 PRIM2 CIAPIN1 NDUFS8 TYW1 ACO1 RSAD2 NUBP2 AOX1 NFU1 POLA1 CISD1 SDHB NFS1 ISCA1 UQCRFS1P1 ISCA2 FDX1L GLRX5 NARFL DPYD NDUFV2 RFESD UQCRFS1 NUBP1 CISD3 PPAT CMAHP GLRX3 CDKAL1 DDX11 RTEL1 ACO2 MUTYH NUBPL CDK5RAP1 NTHL1

GO_NEUROPILIN_BINDING Interacting selectively and non-covalently with a member of the neuropilin family. SEMA4F SEMA4G SEMA4B SEMA4C SEMA3G SEMA3F SEMA3A SEMA3D SEMA4D SEMA3E SEMA4A SEMA3C SEMA3B SEMA7A

GO_DRUG_BINDING Interacting selectively and non-covalently with a drug, any naturally occurring or synthetic substance, other than a nutrient, that, when administered or applied to an organism, affects the structure or functioning of the organism; in particular, any such substance used in the diagnosis, prevention, or treatment of disease. TTC9B PDE4D P2RX1 ACR PPIE P2RX4 FASN CHRNB3 DHODH CHKA PPIC PDE2A FKBP8 SLC6A4 PPARD CHRM2 DRD2 NME2 GSTP1 NR1I2 HTR2B FOLR2 RARA HMGB2 GABRA1 CEP104 IL2RA FKBP11 HMGCS1 PNP FKBP3 CHRNB4 MT3 PPARG DHFR PPIH PGGT1B FKBP6 SRP54 NKTR FKBP2 DHFRP1 MT2A CHRNB2 FKBP7 CYP2D6 PDE10A TTC9C HTR2A ABP1 NPC1L1 PPARA PYGL SMO SIGMAR1 SLC6A3 HTR2C CHRM3 FABP1 ATP5O ATP1A2 CHRNA2 DCK PPIF CYP2C9 CYP4B1 PPP3CA FKBP10 TOP2A TTC9 DRD3 RARB FKBP5 HTR1B CHRM1 ALB PPID CNR1 ATP1B1 FOLR1 NFATC1 FKBP14 FKBP9 SFRP1 NAMPT HSP90AB1 FKBP1A FKBP1B TLR7 FSCN1 TYMS TLR8 PPP3CB FKBP4 PPIG GLRB ACE FKBPL DRD4

GO_NEUROPEPTIDE_RECEPTOR_ACTIVITY Combining with a neuropeptide to initiate a change in cell activity. NMUR2 GPR139 GALR1 HCRTR2 GPR83 SORCS1 NPBWR2 SSTR1 NPFFR2 NPFFR1 GALR2 HCRTR1 PRLHR TACR2 OPRM1 NPY1R GPR143 SSTR3 NPSR1 NTSR2 NPY6R QRFPR SSTR4 GAL SORCS2 BRS3 SSTR2 NTSR1 PROKR1 NPBWR1 GALR3 MCHR1 SSTR5 PROKR2 NMUR1 MC2R SORCS3 PPYR1 KISS1R TACR3 TACR1 NMBR NPY5R NPY2R

GO_ATPASE_ACTIVATOR_ACTIVITY Binds to and increases the ATP hydrolysis activity of an ATPase. MYBPC3 AHSA2 ATP1B1 DNAJC24 DNAJC2 DNAJB1 DNAJB6 TOR1AIP2 ATP1B3 RAB4A TOR1AIP1 DNAJC1 ATP1B2 RAB3A AHSA1 DNAJC10 DNAJC7

GO_LIPOPROTEIN_PARTICLE_RECEPTOR_ACTIVITY Combining with a lipoprotein particle and delivering the lipoprotein particle into the cell via endocytosis. A lipoprotein particle, also known as a lipoprotein, is a clathrate complex consisting of a lipid enwrapped in a protein host without covalent binding in such a way that the complex has a hydrophilic outer surface consisting of all the protein and the polar ends of any phospholipids. CD36 LRP6 APOBR VLDLR LRP1 STAB1 LRP8 OLR1 LRP10 LRP2 CXCL16 LRP12 STAB2 SCARB1 LDLR ILDR1

GO_ANNEALING_ACTIVITY A nucleic acid binding activity that brings together complementary sequences of nucleic acids so that they pair by hydrogen bonds to form a double-stranded polynucleotide. ANXA1 RECQL EIF4H EIF4B BLM FXR1 RECQL4 ZRANB3 SMARCAL1 FMR1 RAD54L SMARCA1

GO_MONOVALENT_INORGANIC_CATION_TRANSMEMBRANE_TRANSPORTER_ACTIVITY Enables the transfer of a inorganic cations with a valency of one from one side of a membrane to the other. Inorganic cations are atoms or small molecules with a positive charge that do not contain carbon in covalent linkage. SLC6A2 ATP5EP2 KCNC2 SLC5A9 KCNF1 SURF1 SLC6A8 SLC6A14 PKD2 KCNK13 ATP1A2 KCNA5 KCNC1 ATP1B2 KCNH3 KCNN3 COX7A2L HCN1 ATP5G1 ATP1B1 SLC5A3 KCNIP1 ACCN3 SLC12A9 TCIRG1 SLC24A6 SLC24A2 COX5B SLC8A1 SLC3A2 SCN5A KCNG2 SLC24A1 KCNC3 KCNH8 KCNS2 SLC13A4 SLC11A1 SCNN1D ATP5L ACCN2 SLC12A6 KCNT1 SHROOM2 SLC9B2 CNGB1 KCNMA1 KCNK6 SCN7A CNGA2 ATP5F1 REST SLC4A11 SLC9A1 COX6C SLC6A16 TMCO3 ATP1A3 SCN10A SLC36A3 SLC9A5 KCNB2 TRPM2 ATP6V0A2 SCN9A SLC6A12 CNNM4 SLC6A13 SLC17A3 CNGA4 KCNJ13 SLC12A7 ATP6V1F SLC13A3 COX7A1 KCNQ3 SLC34A1 KCNK4 KCNH2 SLC28A1 ATP6V1C2 ATP6AP1L KCNJ5 SCN2A SLC1A2 COX7A2 SLC13A5 SLC4A9 KCNJ16 KCNE2 KCNJ4 KCNK3 SLC4A4 KCNMB2 KCNMB4 COX6B2 MTMR6 ATP5D CYB5A PKD2L1 KCNE1L KCNS3 CNGB3 UQCRC1 KCNG1 UQCRQ KCNN2 COX4I1 SLC9A11 ABCB11 KCNIP4 KCNJ11 ATP5G3 SLC9A7 SLC5A7 ATP1B3 ATP6V1A SLC6A15 SLC5A6 SLC1A3 ATP1A1 SLC10A2 SLC17A2 C1orf31 COX5A COX8A SLC34A3 ATP5I SLC1A1 KCNK1 KCNH4 ATP5H KCNIP2 KCNJ9 SLC6A9 KCNJ12 KCNK5 SLC38A1 KCNQ5 COX7B KCNMB3 KCNJ14 KCNV1 KCNIP3 UQCRFS1P1 TRAPPC10 ABCC9 ATP6V1E2 KCNK2 UQCRB KCNS1 SLC6A17 TRPM5 SLC23A1 KCNJ8 KCNA1 SLC17A1 KCNJ1 KCNU1 SLC24A3 COX8C SLC8A2 SLC28A3 ATP6V1B2 ATP6V1G2 KCNV2 SLC34A2 SLC17A7 KCNJ10 KCNA7 COX6B1 SLC9A4 SLC24A4 KCNAB2 SLC6A1 KCNA6 SLC6A20 KCNK18 ATP12A KCNA4 SLC28A2 KCNE4 SCN4B ATP5O KCNK7 ATP5C1 ACCN4 KCNJ6 SLC9A3 SLC46A1 SCN1A ATP6V1C1 KCNE1 KCNH1 SLC10A3 TRPM4 ATP4A COX7B2 ATP1A4 CCT8L2 FXYD2 SLC10A6 ATP5J KCNA2 SLC1A6 KCNH6 KCNT2 ATP6V1G3 UQCRH NALCN NNT KCNAB1 KCNN4 C15orf48 SLC22A18 KCNH5 UQCRFS1 SLC20A2 KCNA10 SLC9A2 SLC9A10 SLC32A1 ATP6V0A1 KCNJ18 KCNQ2 SLC24A5 KCNB1 KCND1 KCNJ2 SLC5A5 COX7A2P2 KCNE3 ATP5B SLC6A19 SCN3A COX10 SLC41A1 HCN4 CATSPER4 SCNN1G SLC9A8 COX11 ATP5A1 ATP6V1G1 COX7C KCNJ15 KCNH7 COX15 SCN11A KCNN1 COX6A1 HPN SLC6A18 KCNK10 SLC6A3 KCNK17 KCNJ3 SLC4A7 KCNK9 KCNMB1 FXYD4 SCNN1B HCN3 SLC47A2 TMEM38A KCNQ1 ATP5E ATP6V0E1 ATP6V0D1 HCN2 SCN2B ATP6V1B1 SLC12A5 ATP4B SLC9A6 SLC8A3 SLC6A5 SLC36A1 SLC6A7 KCNK16 ACCN5 ACCN1 NDUFA4L2 SLC23A2 KCNA3 KCNQ4 SLC5A2 ATP6V1H ATP5L2 SLC4A5 SCNN1A CNGA3 KCNG3 KCNG4 SLC36A2 SLC10A4 ATP6AP1 SLC6A6 SLC9B1 KCNK15 UQCR10 ATP5G2 SLC5A1 SLC20A1 SCN8A KCNK12 SLC6A4 SLC5A4 SLC9B1P1 SLC6A11 CNGA1 ABCC8 NDUFA4 ATP1B4 SLC17A4 SCN1B SLC13A2 ATP6V0A4 ATP5S COX4I2 AQP1 ATP6V0B SLC1A7 SLC13A1 ATP6V0E2 SCN3B SLC12A4 SCN4A ATP6V0C KCND3 SLC10A5 SLC47A1 UQCR11 KCNAB3 SLC10A1 SLC9A9 TMEM175 SLC11A2 KCNC4 ATP6V0D2 KCND2 TMEM38B COX6A2 UQCRHL ATP6V1E1

GO_CALMODULIN_BINDING Interacting selectively and non-covalently with calmodulin, a calcium-binding protein with many roles, both in the calcium-bound and calcium-free states. MYO1C MYO1E ATP2B4 IQGAP1 NOS3 SPTBN1 PPP3CC ATP2B1 MYH10 DCX MAP2 MYO5A MAP6 ADD1 SMTNL1 CAMK2B WFS1 MAPKAPK3 MYH13 ORAI1 STRN GEM MYO10 SYT7 AKAP5 CNN1 MYLK2 KCNQ1 MYO9B RRAD CAMK2G ADD3 SLC8A3 TJP1 ITPKA RGS1 PCYT1A OBSCN RASGRF2 PHKG2 MYO1A PPP3CA MYH1 KCNN2 NRGN PCDP1 SYT1 NOS2 STRN4 ATP2B3 MYO5C CAMK1 PLCB3 MIP GAP43 UNC13A MARCKS CAMKK1 IQCF5 RYR2 SRY ITPKB IQCB1 UBR4 EWSR1 CEP97 MARCKSL1 UNC13B SPTAN1 RYR3 MYH7 CASK EPB41 RIT1 TTN MAP6D1 ADCY3 DDX5 MAPKAPK5 CAMK1D CAMK4 NDUFAF4 SNTA1 PDE1C CAMKK2 IQCG SLC8A2 CNN2 IQCF1 USP6 IQCF3 PDE1A MYO15A MYO1G MYH11 EEF2K MYH2 PHKB SNTB1 GRIN1 RGS2 MYH9 CAMSAP2 MYO1F SLC9A1 KCNN4 CNGA2 NOS1 SCN5A DAPK1 SLC8A1 MYH14 REM1 TRPM4 SNTB2 STRN3 TRPV1 IQGAP2 PPP3CB DAPK2 PLA2G6 MKNK2 KCNN3 PNCK EGFR RGS4 MYH8 KCNH1 MYO7A MYO1B CAMK2D RYR1 ARPP21 RGS16 MKNK1 PHKA2 CALD1 ITPKC FBXL2 MYO1D MYO6 ATPIF1 PPP3R1 CAMSAP1 IQGAP3 MYH4 CTH ATP2B2 PCP4 INVS CDK5RAP2 IQCF2 TRPV4 MYO3A PCNT RIT2 ENKUR SPATA17 EDF1 PHKG1 VAMP2 ADCY1 MAPKAPK2 AEBP1 KCNN1 CAMSAP3 TRPV6 CNN3 PDE1B MYLK ADD2 PHKA1 ASPM MYH3 EEA1 KCNQ3 CAMKV MYO5B MYH15 PLCB1 KCNH5 MYH6 CAMK1G SPHK1 CACNA1C TRPV5 CAMK2A IQCF6

GO_ARACHIDONIC_ACID_MONOOXYGENASE_ACTIVITY Catalysis of the incorporation of one atom from molecular oxygen into arachidonic acid and the reduction of the other atom of oxygen to water. CYP4F2 CYP2C8 CYP2C9 CYP2A6 CYP2F1 CYP2C19 CYP4A11 CYP2D6 CYP2B6 CYP2E1 CYP2C18 CYP2A13 CYP2A7 CYP2J2 CYP4F12

GO_BETA_2_MICROGLOBULIN_BINDING Interacting selectively and non-covalently with beta-2-microglobulin. CD1E HFE CD1C CD1B CD1D FCGRT MICA CD1A HLA-A HLA-E HLA-H

GO_RHO_GUANYL_NUCLEOTIDE_EXCHANGE_FACTOR_ACTIVITY Stimulates the exchange of guanyl nucleotides associated with a GTPase of the Rho family. Under normal cellular physiological conditions, the concentration of GTP is higher than that of GDP, favoring the replacement of GDP by GTP in association with the GTPase. ARHGEF26 FGD3 FGD5 MCF2 PREX2 NGEF ARHGEF38 NET1 VAV2 ARHGEF10 ARHGEF40 FGD6 ARHGEF6 ARHGEF1 TIAM2 ECT2L PREX1 ARHGEF16 FGD1 PLEKHG4 FGD2 PLEKHG2 PLEKHG5 RGNEF ARHGEF37 PLEKHG6 VAV1 DNMBP C9orf100 ABR DOCK1 ARHGEF33 ARHGEF5 DOCK11 RGL2 ARHGAP4 ITSN1 FARP1 PLEKHG1 VAV3 PLEKHG4B SOS2 SPATA13 ARHGEF19 ARHGEF2 ARHGEF12 ARHGEF15 PLEKHG7 AKAP13 ARHGEF7 ALS2 TIAM1 DOCK2 ARHGEF17 EPS8L2 KALRN FGD4 MCF2L ARHGEF3 MCF2L2 RASGRF2 ALS2CL ITSN2 SOS1 TRIO ECT2 FARP2 ARHGEF11 RASGRF1 PLEKHG3 ARHGEF25 BCR EPS8L1 ARHGEF10L ARHGEF9 ARHGEF18 ARHGEF4 OBSCN

GO_PHOSPHOLIPASE_BINDING Interacting selectively and non-covalently with any phospholipase, enzymes that catalyze of the hydrolysis of a glycerophospholipid. PARK2 CALM1 PRKCZ SRSF3 SNCA CALM2 DGKQ LMNB1 PDPK1 WAS PLA2R1 APOC2 CALM3 NEFL PAFAH1B1 PTPN11 SELE

GO_PHOSPHATIDYLINOSITOL_3_4_5_TRISPHOSPHATE_BINDING Interacting selectively and non-covalently with phosphatidylinositol-3,4,5-trisphosphate, a derivative of phosphatidylinositol in which the inositol ring is phosphorylated at the 3', 4' and 5' positions. FERMT2 NRGN JPH2 ARAP1 ASAP1 PIRT ADAP2 ARAP3 CYTH3 ARHGAP9 MYO1G MAPKAP1 GBF1 ZFYVE16 DAPP1 GAB2 MYO1B FAM21C PARD3 RAG2 ZFYVE1 ANXA8 PHLDA3 RS1 COMMD1 IQGAP2 NPM1 MYO10 AKT1 ADAP1 OGT RACGAP1 ARAP2 IQGAP1 KIF16B BTK

GO_GTPASE_ACTIVITY Catalysis of the reaction: GTP + H2O = GDP + phosphate. GNB1 RERG RND3 RAB7L1 TUFM TUBB6 EEF1A1 MX2 RAP2A RAB6C RIT2 RASD2 TUBG2 SEPT4 GNL1 NUDT1 GNAZ RAP1A RHOG RAB6A ARF5 RASD1 TUBB4A MX1 GTPBP10 ARL8B GNAI2 ARHGAP5 GNAT1 TUBB4B GNGT2 LRRK2 TSR1 ATL2 SAR1B RAB2A EIF5 ERAL1 RND2 RAB27B TUBE1 CDC42 RAB33A RAP1GAP RAB14 RAB22A GBP2 RAB17 TRIM23 RAB38 GTPBP5 RHOQ LOC255308 RHOB EEFSEC GBP5 RAB43 RNF112 MFN1 TUBB8 TUBA8 GTPBP8 GNA12 RALA ATL3 DDX3X GNB3 MTG1 EEF2 RAB21 GNA13 GNAS GBP6 ARHGDIB ARF3 GNG10 HRAS EIF2S3 RAB3B EEF1A1P5 TUBA4A GSPT1 NKIRAS2 LSG1 ARF1 GNL2 RAB31 NKIRAS1 RAB3C ARL4C DNAJC27 RAB11B TUBA3D RAB11A RAB5B GBP3 TUBB2A RAP1B GBP1 DIRAS1 GNAO1 RND1 RAB32 GTPBP3 RRAS2 ADSSL1 RAB3D RRAGB TUBD1 TUBB2B RAB4A GTPBP2 GNB5 RIT1 GBP4 DNM1P34 RAB28 RAB18 MTIF2 ARFRP1 GPN1 OPA1 TUBG1 RABL2A GTPBP1 GTPBP4 DNM2 RAC1 TUBA3E RALB MFN2 GNA11 RAB30 GNA15 GNAL REM2 RAB5A IRGM SRP54 TUBB RAB33B GNA14 ARF4 RAB10 RAB35 TUBA1C GNAT3 GFM1 MRAS GNG8 EIF5B RABL2B RAB7A RAB13 TUBB3 RHEB GIMAP7 GNAI1 TUBB1 RHOF BMS1 GNB2 ATL1 TUBA3C RASL10A ARL3 RAB5C RHOJ RAB8B GNG11 SEPT9 TBCC GEM RAB9A GUF1 RHOD EFTUD2 ARL2 GNG3 DNM1 RHOU GNL3 GNAT2 HBS1L DNM3 ARF6 SRPR GNAQ RAB1A GNL3L MMAA IRGC RRAS RHOT2 RHOA RAB8A ARL8A RAB3A RAC3 SEPT5 GNG5 DNM1P46 RAB6B RRAGD TUBA1B GNGT1 EFTUD1 RAC2 GSPT2 RHOT1 RAB23 GNAI3 DIRAS3 ARL1 TUBAL3 RAN KRAS TUBA4B GBP7 MSTO1 RRAGA NOA1 RRAD TUBA1A RAB27A GFM2 DNM1L EEF1A2 RRAGC ARL4D

GO_ISOMERASE_ACTIVITY Catalysis of the geometric or structural changes within one molecule. Isomerase is the systematic name for any enzyme of EC class 5. TOP1 FKBPL PPIB GNPDA2 PUS1 SRR RPUSD2 TOP3A BPGM ENOSF1 EIF2B2 PPIA DKC1 ERP29 PUS10 FKBP1B TRUB1 TXNDC5 ALOX15 PPIG TPI1 PGM5 TOP2A PMM2 ECH1 EBPL PPIL4 PPIAL4D ERO1L PGM1 PBLD FKBP14 EBP MIF HSD17B4 ECI1 YJEFN3 TXNDC11 ERO1LB DCT MRI1 PPWD1 PGM2 GSTZ1 RPE LSS RPUSD4 NKTR FKBP2 LOC729020 PGM3 HPGDS PUS7L PTGIS DHRS9 RPE65 PTGES2 RPIA TRUB2 FKBP3 P4HB TBXAS1 IDI1 PTGES3 PPP2R4 SREBF2 PPIAL4C RPUSD3 PIGK PPIL1 PPIAL4B RENBP PDIA4 FKBP11 PPIL2 MCEE HMGCS1 TTC9B PPIE TMX3 PPIC TSTA3 FKBP8 PTGDS PGAM1 GLRX2 QSOX1 PIN4 PPIAL4E PIN1 QSOX2 RPUSD1 FKBP1A TOP1MT CWC27 PDIA5 SPO11 ISYNA1 GNE HYI ITGB3 PGM2L1 ITPK1 PPIAL4F FKBP4 PDIA2 TTC9 FKBP5 HSD3B2 TMX1 PPID EHHADH TOP3B RANBP2 PMM1 PPIL6 PDIA3 AMACR FKBP9 GNPDA1 APOA1BP ECI2 PGAM2 PUS7 IDI2 PPIF FKBP10 TMX4 PPIH FKBP6 DSE MUT FKBP7 ALOXE3 TTC9C HSD3B1 PTGES C10orf125 PPIAL4G GALM PGAM4 ERP27 PDIA6 GPI TOP2B PPIL3 EIF2B1 ERP44 DDT PUSL1 PUS3 ALOX12 MPI GLCE DSEL C14orf149 EIF2B4 PDILT GALE

GO_CHAPERONE_BINDING Interacting selectively and non-covalently with a chaperone protein, a class of proteins that bind to nascent or unfolded polypeptides and ensure correct folding or transport. C1orf182 PARK2 ERP29 SYVN1 PFDN4 TBCC HSPE1 BAG2 PRNP DNAJA4 SACS DNAJB6 CLU DNAJB1 AMFR BAG3 TIMM44 CP DNLZ DNAJC10 GRPEL1 CALR ST13 DNAJB4 TIMM9 STIP1 TBCE AHSA1 ATP1A2 CTSC FGB GNB5 ALB HES1 DNAJC1 RNF207 TBCD USP13 TBCA DNAJB2 BAG5 OGDH ATP1A1 TIMM10 WRAP53 TSC1 SLC25A17 DNAJB7 PFDN6 TP53 BAK1 DNAJA1 PACRG BIRC5 CDKN1B GRPEL2 UBL4A BAG4 DNAJC3 DNAJA2 DNAJB5 SDF2L1 ATP1A3 VWF CDC37 HYOU1 GET4 HSPD1 DNAJB8 HSPA5 HSCB SOD1 BAG1 AHSA2 CXorf41 CDC37L1

GO_CHLORIDE_CHANNEL_REGULATOR_ACTIVITY Modulates the activity of a chloride channel. SLC9A3R1 VTI1B WNK1 SGK1 SGK3 SGK2 BSND WNK3 STX1A CFTR STX7 C8orf44-SGK3 STX8 CHRNA7 VAMP8 WNK4 TRPV1

GO_TRANSLATION_INITIATION_FACTOR_ACTIVITY Functions in the initiation of ribosome-mediated translation of mRNA into a polypeptide. EIF6 EIF2S2 EIF3J EIF4A2 EIF3E EIF1B MTIF2 EIF3C EIF2S3 DHX29 EIF4A1 EIF2B2 EIF3L EIF4E2 EIF4G2 EIF2C2 EIF2D EIF3F EIF4B EIF2B5 EIF3H EIF5B EIF3K EIF5 EIF2A EIF3A EIF1 EIF4G1 COPS5 EIF3D EIF2B3 EIF1AY EIF1AD EIF2B4 EIF4H EIF4E EIF3G EIF4E1B EIF3I EIF3CL MCTS1 EIF4E3 DENR EIF2S1 LOC255308 EIF3B EIF2B1 EIF3M MTIF3 EIF4G3 EIF1AX

GO_DNA_DEPENDENT_ATPASE_ACTIVITY Catalysis of the reaction: ATP + H2O = ADP + phosphate; this reaction requires the presence of single- or double-stranded DNA, and it drives another reaction. HELB DHX36 TOP2A XRCC6 SMARCA2 CHD3 XRCC2 IGHMBP2 CHD1L ASCC3 MCM4 RBBP4 RAD50 BRIP1 CHD4 ANXA1 G3BP1 CDK7 CHD6 RUVBL2 ATRX DNA2 FBXO18 BLM RAD51D CHD1 ERCC8 RFC3 DDX3X GTF2H3 MCM6 SPO11 RAD51B RECQL RFC4 RUVBL1 ERCC2 DMC1 CCNH BPTF NBN XRCC5 TOP2B RECQL5 PIF1 ERCC6 GTF2H2 WRN DDX11 DDX12P RFC5 RTEL1 MNAT1 RFC1 TTF2 MCM7 RECQL4 RAD17 SMARCA4 MRE11A XRCC3 DHX9 GTF2H1 CHTF18 ERCC3 RFC2 GTF2H4 RAD51 SMARCA1 RAD18 POLQ DSCC1 CHD2 SMARCAL1 RAD51C CHD8 RAD54B

GO_DEMETHYLASE_ACTIVITY Catalysis of the removal of a methyl group from a substrate. PHF2 PHF8 JMJD1C KDM5B KDM5A UTY KDM3B KDM2A KDM6B CYP51A1 KDM4D KDM5C ALKBH2 KDM3A FTO ALKBH4 JARID2 CYP1A1 KDM1B HR ALKBH5 KDM5D KDM2B C14orf169 KDM6A KDM1A KDM4B JHDM1D CYP1A2 JMJD6 KDM4A JMJD5 MMACHC ALKBH3 ARID5B KDM4C

GO_UBIQUITIN_LIKE_PROTEIN_CONJUGATING_ENZYME_BINDING Interacting selectively and non-covalently with a ubiquitin-like protein conjugating enzyme such as ubiquitin conjugating enzyme. RNF19B RNF217 RNF19A DCUN1D5 RNF180 RNF138 RNF185 TOLLIP RNF114 DCUN1D1 RPS3 RNF40 ANKIB1 TRIM72 RNF144A RNF144B DCUN1D4 DCUN1D3 SIAH2 GRIK2 RNF166 RNF5 PARK7 PARK2 RNF125 PPARA MARCH6 RNF14 RASD2 FOXL2 ZMYM2 ARIH2 TRAF6 DCUN1D2 ARIH1

GO_MONOCARBOXYLIC_ACID_BINDING Interacting selectively and non-covalently with a monocarboxylic acid, any organic acid containing one carboxyl (COOH) group or anion (COO-). NR1H4 PTGDS O3FAR1 PCCA SNCA APOC1 FABP2 FABP1 ADH5 UGT2B4 STX3 FABP5 RARA AKR1C2 IGF2R AKR1C1 UGT1A1 S100A9 PC CYP26C1 HLCS PPARD PMP2 HRSP12 ALB HNF4A FABP3 NME2 UGT1A9 PPARG ACACB UGT1A6 UGT1A8 LCN12 FABP6 PLA2G1B NDUFAB1 STARD5 UGT2B7 NR2F2 LRAT CRABP2 CYP26A1 MCCC1 UGT2B17 OXER1 SERPINA5 UGT1A10 CRABP1 PYGL S100A8 SCP2 CYP26B1 UGT1A3 ALOX5AP UGT1A4 UGT2B15 UGT1A7 FABP4

GO_PEPTIDYL_PROLINE_DIOXYGENASE_ACTIVITY Catalysis of the reaction: peptidyl L-proline + 2-oxoglutarate + O2 = peptidyl hydroxy-L-proline + succinate + CO2. OGFOD1 EGLN3 P4HA2 PDIA2 P4HA3 P4HB LEPRE1 EGLN1 LEPREL1 LEPREL2 EGLN2 P4HA1

GO_VOLTAGE_GATED_ANION_CHANNEL_ACTIVITY Enables the transmembrane transfer of an anion by a voltage-gated channel. An anion is a negatively charged ion. A voltage-gated channel is a channel whose open state is dependent on the voltage across the membrane in which it is embedded. CLCN4 CLCN5 BSND CLCN1 CLCNKA GPR89A CLCN7 VDAC3 ANO1 SLC17A3 ANO6 VDAC1 CLCN3 CLCNKB CLCN6 VDAC2 CLCN2

GO_TAU_PROTEIN_KINASE_ACTIVITY Catalysis of the reaction: ATP + tau-protein = ADP + O-phospho-tau-protein. CDK5 BRSK2 PRKAA1 GSK3A PHKG2 GSK3B MARK1 MARK4 PHKG1 MARK2 CSNK1D BRSK1

GO_STRUCTURAL_CONSTITUENT_OF_RIBOSOME The action of a molecule that contributes to the structural integrity of the ribosome. SLC25A15 SLC25A45 SLC25A26 MRPS18A SLC25A33 SLC25A23 RPS10P5 RPL37AP8 RPS10 SLC25A38 RPL23 RPL26L1 SLC25A36 RPS19 RPS27A SLC25A4 UCP3 RPL3L RPL7 MRPL43 RPS6 RPL7L1 RPL19 SLC25A17 SLC25A37 RPL27 RPL18 MRPL12 RPL12 RPS29 MRPS17 SLC25A39 MCART1 RPS23 RPS27L RPS21 RSL24D1 MRPL16 MRPS21 RPS7 RPL27A RPL22 RPL37A RPL39 MRTO4 SLC25A25 MRPL22 RPS20 MRPS16 SLC25A6 MRPS12 RPLP0P6 RPL36A MRPL21 RPS8 UCP2 RPS4Y1 MRPS9 MRPL13 SLC25A32 SLC25A41 MRPS18C MRPL41 SLC25A21 SLC25A28 SLC25A34 RPL13AP3 RPS15 RPS4X UCP1 MRPS18B RPS13 RPL11 MRPS7 SLC25A30 MCART6 MRPS31 RPL14 MRPL35 MRPL20 SLC25A20 RPL30 RPS16 RPL32 IMP3 RPS2 MRPS25 UBA52 MRPL10 SLC25A2 MRPL14 RPL36AL MRPL47 RPS3 RPL9 RPL10A SLC25A12 RPS11 MRPL4 MRPL9 MRPS36 SLC25A19 MRPL23 RPL6 MRPL42 MRPL37 MRPL2 MRPL30 RPS9 MRPL17 RPL13A RPL8 RPLP2 RPL23A SLC25A22 RPLP0 MRPL28 MRPL1 MRPL36 DAP3 MRPS2 RPL39P5 RPL5 MRPS34 MRPL52 C7orf44 RPL15 MRPL55 RPL18A MRPL24 SLC25A29 RPL24 RPL13 RPL41 SLC25A48 RPL29 MRPL46 MRPS6 SLC25A13 MRPS35 RPL36 MRPS30 MRPL49 SLC25A24 SLC25A40 MRPS23 MRPS15 RPS28 SLC25A1 MRPL11 MRPL18 MRPL34 SLC25A11 RPL3 SLC25A43 SLC25A16 MRPS14 MRPS11 RPS12 RPS14 RPL31 RPL17 RPL10L RPL35A RPL28 MRPL19 SLC25A14 MRPL51 RPS3A RPS27 SLC25A42 SLC25A47 RPL38 RPS26 RPL22L1 RPL4 RPL26 SLC25A18 SLC25A3 MCART2 RPS4Y2 RPS18 MRPL27 MRPL3 RPL34 SLC25A35 MRPL33 MRPL32 SLC25A27 MRPS33 RPS24 MRPS5 RPL39L MRPL15 RPS15A RPS5 NDUFA7 RPS17 MRPS22 RPLP1 RPSA MRP63 SLC25A31 SLC25A44 RPL35 SLC25A10 SLC25A5 RPL21 RPL37 RPL7A MRPS24 RPL10

GO_PROTON_TRANSPORTING_ATP_SYNTHASE_ACTIVITY_ROTATIONAL_MECHANISM Catalysis of the transfer of protons from one side of a membrane to the other according to the reaction: ADP + H2O + phosphate + H+(in) = ATP + H+(out), by a rotational mechanism. ATP5G1 ATP5C1 ATP5F1 ATP5G2 ATP5D ATP6V0C ATP5H ATP5EP2 ATP5G3 ATP6AP1 ATP5A1 ATP5E ATP6AP1L ATP5B ATP5O

GO_STORE_OPERATED_CALCIUM_CHANNEL_ACTIVITY A ligand-gated ion channel activity which transports calcium in response to emptying of intracellular calcium stores. ORAI2 ZP3 TRPC3 TRPC6 ORAI3 TRPC4 ORAI1 STIM2 TRPC1 TRPC5 TRPC7

GO_HISTONE_METHYLTRANSFERASE_ACTIVITY_H3_K4_SPECIFIC_ Catalysis of the reaction: S-adenosyl-L-methionine + histone H3 L-lysine (position 4) = S-adenosyl-L-homocysteine + histone H3 N6-methyl-L-lysine (position 4). This reaction is the addition of a methyl group onto lysine at position 4 of the histone H3 protein. SETD3 DYDC2 RBBP5 ASH2L DYDC1 WDR82 SETMAR ASH1L MLL MLL4 WDR5 DPY30 CXXC1 SETD1B MLL5 MLL2 SETD1A MLL3

GO_4_IRON_4_SULFUR_CLUSTER_BINDING Interacting selectively and non-covalently with a 4 iron, 4 sulfur (4Fe-4S) cluster; this cluster consists of four iron atoms, with the inorganic sulfur atoms found between the irons and acting as bridging ligands. ISCU NUBP1 BRIP1 PPAT MOCS1 ISCA2 ISCA1 NDUFS7 RSAD1 NARFL DPYD NDUFS2 MUTYH IREB2 NUBPL NTHL1 CDK5RAP1 TYW1B CDKAL1 ETFDH DDX11 POLD1 RTEL1 ACO2 NDUFV1 PRIM2 LIAS NDUFS8 TYW1 ACO1 RSAD2 POLE ERCC2 DNA2 NDUFS1 REV3L NUBP2 DEM1 NFU1 POLA1 SDHB

GO_RAC_GUANYL_NUCLEOTIDE_EXCHANGE_FACTOR_ACTIVITY Stimulates the exchange of guanyl nucleotides associated with a GTPase of the Rac family. Under normal cellular physiological conditions, the concentration of GTP is higher than that of GDP, favoring the replacement of GDP by GTP in association with the GTPase. PREX2 DOCK2 SPATA13 TIAM1 ALS2 FARP2 VAV3 ARHGEF4 FARP1 EPS8L2 EPS8L1 ARHGEF2 VAV1

GO_1_PHOSPHATIDYLINOSITOL_3_KINASE_ACTIVITY Catalysis of the reaction: 1-phosphatidyl-1D-myo-inositol + ATP = a 1-phosphatidyl-1D-myo-inositol 3-phosphate + ADP + 2 H(+). FGF4 FGF19 PIK3C2B PIK3C2A PIK3C3 FGF16 FGF20 PIK3R2 FGFR4 PIK3CB PIK3CA IRS2 FGF10 GAB1 FRS2 FGF2 PTPN11 KL PIK3R3 FGF9 FGF22 FGF8 FGFR1 FGF23 PIK3CG IRS1 FGFR3 FGF18 FGF3 PIK3C2G ATM PIK3R1 KLB FGFR2 FGF1 GRB2 FGF6 FGF5 PIK3CD FGF7 PIK3R4 TLR9 FGF17

GO_MHC_CLASS_I_PROTEIN_BINDING Interacting selectively and non-covalently with major histocompatibility complex class I molecules; a set of molecules displayed on cell surfaces that are responsible for lymphocyte recognition and antigen presentation. CD244 DERL1 TUBB TAP1 HLA-E ATP5B VCP CD8B CD8A KLRC4-KLRK1 KLRK1 PILRB LILRB2 ATP5A1 TAPBP PILRA BCAP31 LILRB1 TUBB4B

GO_DNA_POLYMERASE_ACTIVITY Catalysis of the reaction: deoxynucleoside triphosphate + DNA(n) = diphosphate + DNA(n+1); the synthesis of DNA from deoxyribonucleotide triphosphates in the presence of a nucleic acid template and a 3'hydroxyl group. POLQ CCDC111 LOC100133495 REV3L POLA1 POLH POLM TERT TEP1 MYBBP1A PAPD7 DKC1 POLG POLE PTGES3 POLA2 PAPD5 TERF2 ERVK-6 CHRAC1 ERVK-7 LOC100128274 POLD1 TERF1 POLI POLK POLD4 POLB ERVK-8 POLD3 POLE2 POLG2 ERVK-10 HMBOX1 POLN POLE3 POLE4 POLD2 DNTT POLL

GO_2_IRON_2_SULFUR_CLUSTER_BINDING Interacting selectively and non-covalently with a 2 iron, 2 sulfur (2Fe-2S) cluster; this cluster consists of two iron atoms, with two inorganic sulfur atoms found between the irons and acting as bridging ligands. CIAPIN1 CISD3 RFESD NDUFV2 ISCU AIFM3 UQCRFS1 XDH FDX1L GLRX5 ISCA1 UQCRFS1P1 ISCA2 GLRX2 NDUFS1 CISD2 AOX1 FXN FDX1 CMAHP CISD1 SDHB FECH

GO_TRANSCRIPTION_FACTOR_ACTIVITY_DIRECT_LIGAND_REGULATED_SEQUENCE_SPECIFIC_DNA_BINDING A DNA binding transcription factor activity that is directly regulated by binding of a ligand to the protein with this activity. Examples include the lac and trp repressors in E.coli and many steroid hormone receptors. NR6A1 ESRRB BRD8 RXRB NR4A2 NR1D1 PPARA NKX3-1 NR4A3 VDR AR NR2F6 NR1H3 RXRA NR2F2 ESR1 NR0B1 THRA PPARG THRB NR1I2 NR1H2 HNF4A PPARD NR2F1 RARB AHR ESRRG NR1D2 RXRG NR5A1 RORC STAT3 NR4A1 RARA C1orf85 HNF4G RORB NR1I3 ESRRA ESR2 GPER RARG RORA NR2E3 NR5A2 NR1H4

GO_CARBONATE_DEHYDRATASE_ACTIVITY Catalysis of the reaction: H2CO3 = CO2 + H2O. CA3 CA1 CA8 CA9 CA2 CA11 CA14 CA4 CA13 CA5A CA6 CA7 CA12 CA5B

GO_ADENYLATE_CYCLASE_BINDING Interacting selectively and non-covalently with the enzyme adenylate cyclase. ADCY5 AKAP5 CALM2 ADRB2 AKAP6 CAP1 ADCYAP1R1 CALM1 ADCY2 AKAP12 CAP2 CALM3

GO_PROTEIN_BINDING_INVOLVED_IN_PROTEIN_FOLDING Interacting selectively and non-covalently with any protein or protein complex (a complex of two or more proteins that may include other nonprotein molecules) that contributes to the process of protein folding. DNAJB8 CLGN PDCL3 HSPA1A RIC3 HSPA1B PFDN2 CD74 CALR CCT6A PFDN1 CCT3 CCT2 CALR3

GO_DIPEPTIDYL_PEPTIDASE_ACTIVITY Catalysis of the hydrolysis of N-terminal dipeptides from a polypeptide chain. DPP3 DPP4 FAP DPEP3 PRSS16 DPP6 DPP7 DPP10 DPEP2 PRCP DPP8 NAALAD2 DPEP1

GO_RETINOID_X_RECEPTOR_BINDING Interacting selectively and non-covalently with a retinoid X receptor. NR1H2 NR0B2 FUS NCOA1 HMGA1 NR4A2 RARB RARG NCOA6 NSD1 VDR NRIP1 NR1H4 MED25 PPARG

GO_PHOSPHATASE_BINDING Interacting selectively and non-covalently with any phosphatase. CHCHD3 SH3YL1 FER FLT4 WNK1 SBF2 SH3GL1 ELL PPP1CA CTSC KCNQ1 ANKLE2 PPP1R3C LILRB1 STAU1 ANAPC7 PPP1R3G PPP1R9A STRN4 PPP6R1 SLC9A3R2 PPP1R35 PHACTR4 PPP1R36 GRB2 GNB2L1 STAT6 PPP1R15A CDH2 SNX3 PIK3R1 MASTL ATP2B4 SMG5 IQGAP1 MAGI2 ROS1 CRY2 STRN HSP90B1 ANAPC5 BCL2 VRK3 EIF4EBP1 MYOZ2 TPRN CSK SPRED1 STX17 CTNNB1 RPA2 MAP2K7 CEACAM1 PHACTR1 PPP2R2A PIK3R2 SYTL2 PPP6R3 EIF2AK3 ITGA1 FOXO1 AP3B1 PPP1R18 DLG4 TRAF3 CNST CARHSP1 CDH5 PPP1R39 TP53 DLG1 CSRNP2 PPP1R26 DZIP3 PPP1R32 JAK3 MAPK14 TSC2 DLG3 VCP HSP90AA1 IRS2 ELFN2 PPP1R21 KAT2A EGFR PARD3 PPP1R9B PPME1 SH2D4A MET SMG7 FBXL2 KCNN4 SLC9A1 BAD CEP192 STAT5B STAT1 STRN3 AKT1 PPP3CB MVP PHACTR3 PPP1R3F SMAD3 SKAP1 STAT3 DAB2IP JUP ANAPC4 PTPN1 SOD1 SMAD2 MAPK1 ARPP19 SLC9A3R1 CSF1R RPS6KB1 PPP2R4 SPHK1 NEK2 MAP3K5 LCK CRY1 AKAP11 PPP1R27 PPP1CC MAPK3 MAST2 SFI1 JAK1 PPP1R37 LILRB2 PPARA SLC6A3 TCTEX1D4 ENSA HMGCR ERBB2 IGBP1 CDKN1B ELFN1 CDC5L GRIN3A PPARG TRAF2 SHOC2 SPTBN4 CDC27 CTTNBP2NL TRPC4AP

GO_RACEMASE_AND_EPIMERASE_ACTIVITY Catalysis of a reaction that alters the configuration of one or more chiral centers in a molecule. GALM APOA1BP DSEL C14orf149 DHRS9 C10orf125 GLCE GALE GNE YJEFN3 DSE LOC729020 RPE MCEE AMACR SRR RENBP

GO_TRANSLATION_REGULATOR_ACTIVITY Any molecular function involved in the initiation, activation, perpetuation, repression or termination of polypeptide synthesis at the ribosome. LARP1 IGF2BP3 DAZ1 RPS14 DAZ3 AIRE TRIM71 RPS9 CELF4 PABPC1 EIF4EBP2 CIRBP NEURL PURA SAMD4B PAIP2 CPEB4 SAMD4A EIF4EBP1 RARA PAIP1 EIF4EBP3 CPEB2 RPS27L IREB2 BOLL DAZL ZNF540 CELF1 IGF2BP2 NANOS1 PAIP2B FMR1 CPEB1 CPEB3 IGF2BP1

GO_TRANSFORMING_GROWTH_FACTOR_BETA_BINDING Interacting selectively and non-covalently with TGF-beta, transforming growth factor beta, a multifunctional peptide that controls proliferation, differentiation and other functions in many cell types. TGFBR2 CD109 LTBP3 LTBP4 TGFBR1 CD36 ACVRL1 VASN THBS1 LTBP1 TGFB3 ENG HYAL2 ITGAV ACVR1 TGFBR3

GO_ACTIN_DEPENDENT_ATPASE_ACTIVITY Catalysis of the reaction: ATP + H2O = ADP + phosphate. This reaction requires the presence of an actin filament to accelerate release of ADP and phosphate. MYH14 MYO1C MYH9 MYH6 MYO1D MYO7A MYH10 MYO10 MYH7 MYO1B MYL6 MYO3A

GO_RAL_GTPASE_BINDING Interacting selectively and non-covalently with Ral protein, any member of the Ral subfamily of the Ras superfamily of monomeric GTPases. MYO1C PRKCH EXOC4 FLNA LSM2 EXOC2 RNF41 EXOC8 RAB34 PIH1D2 RALBP1 EXOC5 USP33

GO_PHOSPHOTYROSINE_BINDING Interacting selectively and non-covalently with a phosphorylated tyrosine residue within a protein. SAMSN1 LDLRAP1 MAPK1 CBLB CBL PTPN5 MAPK3 ZAP70 FGR STAP1 PTPN3 PTPN6 CBLC GRB2

GO_PEPTIDASE_ACTIVITY Catalysis of the hydrolysis of a peptide bond. A peptide bond is a covalent bond formed when the carbon atom from the carboxyl group of one amino acid shares electrons with the nitrogen atom from the amino group of a second amino acid. LOC100287238 USP6 USP17L1P PRSS46 ECE2 CASP7 USP17L5 PLAT ADAM28 ADAMTSL2 ECEL1 USP17L7 ENDOU ADAM20 COPS5 CAPN1 ACR PRSS23 HTRA2 PRCP IGHG4 CTSD CLCA2 CPO PRSS2 ZMPSTE24 ERVK-6 PGA3 PSMB8 SENP1 SPPL2B MMP26 FCN1 IGKV2-40 CFB ACE2 PDIA3 CTRL CLC MMP13 PCSK9 HTRA1 AGTPBP1 CASP10 AZU1 USP45 CAPNS1 TMPRSS12 PRSS41 GZMA CPXM1 LOC100287205 METAP1D ADAMTS7 RHBDF1 RELN TNIP1 CPA4 CAPN14 CPA3 CAPN12 OTUD6B SENP7 IGKV3D-20 ADAMTS17 ADAMTS10 IGHG3 METAP1 PSMA6 C3orf37 OTUD3 PM20D2 LOC100287478 SPPL2A ADAM7 MMP17 ADAMTS4 USP24 CPA5 USP47 USP17L6P PROC MMP9 ATG4B TMPRSS9 METAP2 USP12 PGC ADAMTS9 XPNPEP1 PRSS55 PARL HPN KLK4 CTSK PSMA3 AEBP1 NRIP2 KLK6 SENP2 NAPSA TMEM27 FCN3 PRSS57 CASP14 USP13 HGFAC C1S ADAMTS18 TMPRSS15 FAM76B C2 FAM188B IGKV3D-11 C4B USP8 UCHL3 PHEX F3 MMP23B PCSK7 CTSL2 FAM76A CNDP1 PSMA4 OMA1 PSMB1 ACE C3 IGKV1D-33 PGCP USP46 CTSB GGT6 CASP5 CASP9 ZFYVE9 ENPEP CHMP1A SPG7 USP17L2 CPB1 GGT7 FOLH1B TMPRSS4 PARK7 PSMB7 C11orf9 OTUD4 PRTN3 GZMK TMPRSS11F ADAMTS6 ATG4A USP1 CPB2 STAMBPL1 PEPD CPXM2 ADAM23 USP20 CAPN6 USP5 BLMH CASP12 RHBDL2 USP2 ADAM8 F5 USP3 USP4 CLPP USP38 ADAMTS12 ADAMTSL1 TRHDE MMP27 CTSW DDI2 KEL IGKV1-5 SPCS1 KLKB1 KLK8 ERMP1 TMPRSS11D NPEPPS USP31 PCSK5 ESPL1 TMPRSS3 USP33 ATG4C ACY1 SFRP1 DPP9 PAPPA2 PSMA2 KLK12 CTSC ECE1 C4A USP18 PRSS37 BACE1 C9orf3 DPEP1 CTRB1 TMEM59 USP29 LOC100287144 UQCRC1 USP25 USP42 TLL1 OVCH2 USP9Y MALT1 MMP16 CTSE PRSS38 PSMA5 MMP19 ADAMTS8 IGHV3-23 IMMP1L DPP4 LTF USP17L3 ADAMTSL4 DPP10 CTSF F10 DPP6 TPSB2 AFG3L2 KLK1 USP19 COLEC11 CPD PSEN1 AGA ADAM29 ADAM18 TINAGL1 PLAU CAPN3 CELA3A PRSS16 LOC100287327 USP48 USP36 LPA PLG PRSS53 KLK10 PSMB10 IGKV4-1 CRBN TPSG1 USP14 PGPEP1L OTUD7B USP30 SEC11A PRSS44 CTSH OTUB1 LOC100287441 TMPRSS2 PREPL FAM105B ANKZF1 TMPRSS11A UCHL1 UFSP1 PRSS54 ADAM17 PAPLN LONP2 LOC100287178 USP21 STAMBP AGBL3 PMPCB GCA MIPEP USP41 AGBL5 USP10 MME F2 MMP15 SCRN1 CTSO TINAG KLK15 RHBDF2 RHBDD3 RCE1 HGF OTUB2 ADAMTS19 USP16 MASP1 FCN2 CELA2B AMZ2 LOC100287404 IGLC7 MMP2 USP34 BMP1 TMPRSS13 F8 IGLC3 USP27X PITRM1 CPM PSMB2 CPE CMA1 TPP2 ERAP1 USP28 GZMM IGLC1 MST1 IGHG1 USP35 PCSK2 HTRA3 CFD FGL2 ADAMTS20 ADAMTS1 USP17 PRSS8 PRSS1 CTSL3 APH1B PPPDE1 IGKC NLN PRSS12 FOLH1 MMP28 ADAMTSL3 LAP3 APH1A PRSS29P SCPEP1 IMP5 NAALADL1 SENP5 OTUD1 TMPRSS11B ADAM22 C2orf89 YME1L1 IDE SPPL3 MMP14 USP54 TNFAIP3 RNPEP F11 RHBDD1 CPN1 CTSA PGPEP1 ASRGL1 KLK7 LONRF3 PSMA1 COPS4 CAPN9 LOC100288520 PGA4 EIF3F MBTPS1 ADAMTS14 LMLN CASP6 FAP CAPNS2 ALG13 USP22 COLEC10 THOP1 VCPIP1 LOC440786 BACE2 CLCA4 TANK PSMA8 ADAMTS13 UCHL5 USP32 OTUD5 SENP6 TPSAB1 MMP8 MMP25 C6orf103 UNC5CL LOC440434 FAM63A KLK2 CPNE1 PRSS27 XPNPEP3 CLCA3P PIGK SCRN3 MMP1 NRIP3 NUDT16 PSMD1 CAPN10 ADAMTS2 USP11 NRD1 RHBDD2 C1QB ASTL SENP3 MMP21 PSMB5 RHBDL3 PDCD6 PSMB11 THSD4 PGA5 REN PRSS58 KLK3 RBP3 CLPX FAM188A ERAP2 BRCC3 GGH ELANE AMZ1 TASP1 PRSS47 PRSS33 UFSP2 CAPN2 GGT5 CELA1 CPA2 MMP3 PRSS22 ST14 ADAMTS16 JOSD2 C1R OVCH1 USPL1 ADAM9 MMP20 IGLC2 CAPN8 USP53 IGLV1-51 USP49 TRY6 PRSS56 IGLV7-43 SEC11C PSEN2 GGT2 MPND USP37 LOC100287513 ADAMDEC1 DPEP2 MMP10 MEP1A DPEP3 ADAM33 C1QA PSMA7 LONRF1 ANPEP CASP4 KY MMP7 ADAM2 ADAMTSL5 PSMD2 ZC3H12A KLK11 GZMH DPP3 KLK9 IGLC6 GGT1 RNPEPL1 AGBL4 PRSS45 PSMB9 MEP1B IGHV2-5 TMPRSS7 PRSS36 PSMB4 CTSS LOC388630 PREP CNDP2 USP43 MBTPS2 PEF1 C1QC CASP2 MST1P9 PIP CELA2A USP26 CTRC USP9X PCSK1 ATG4D NDEL1 SRI XPNPEP2 CAPN11 PRSS48 CPA1 DPP7 USP51 CPZ LONRF2 USP44 ADAM30 TLL2 CASP8 ADAMTS5 MMP12 KLK13 UQCRC2 MMP24 KLK5 LNPEP JOSD1 USP50 KLK14 ATXN3L CFI USP40 F12 PSMD14 PRSS3 BAP1 OTUD7A IGKV3-20 USP7 TTC15 SOLH CAPN7 CASP1 SCRN2 PMPCA CTSG USP17L8 CTRB2 CAPN5 TAF2 GZMB TMPRSS11E MMP11 PSMB6 ZRANB1 UFD1L ADAM10 USP39 PM20D1 CTSZ ASPRV1 ADAMTS3 F7 TPSD1 LTA4H PRSS42 CYLD PSMB3 YOD1 NCSTN PYCARD DDI1 CASP3 F9 OSGEPL1 ADAM21 SEC11B MMEL1 ADAM12 USP15 LOC100287364 TPP1 LONP1 APEH PRSS50 HTRA4 NAALAD2 ADAMTS15 PPPDE2 NPEPL1 PRSS21 CELA3B USP17L4 CFLAR CPVL CLCA1 PCSK4 DNPEP DHH DPP8 AGBL1 HM13 ADAM11 IGHG2 IMMP2L C1RL SHH RHBDL1 PAPPA FURIN HABP2 CTSL1 AQPEP SPCS2 LGMN CORIN TMPRSS5 AGBL2 IHH ADAM19 CAPN13 ATXN3 GGT3P MYSM1 ADAM15 XRCC6BP1 OTUD6A MBL2 CPS1 SPCS3 SENP8 PCSK6 CPA6 ADAM32 TYSND1 YBEY MASP2 PROZ FAM63B DCD TMPRSS6

GO_AMMONIUM_ION_BINDING Interacting selectively and non-covalently with ammonium ions (NH4+). IGJ HTR3B RPE65 ESYT2 CHMP2A CHMP3 CHRNB4 CHRNG RASGRP1 GPR119 BCHE CHRNA5 PITPNM1 PITPNA CHRNA10 APOA1 APOA4 CHRNA9 CHRNB1 CHRNB2 SERPINA5 CHRNA4 IGHM NF1 APOA5 HTR3A CHRM3 CETP CHRNA1 APOA2 HTR3C CHRNB3 SLC18A3 HTR3D CHRND CHRNA2 CHKA CHRNA3 CHRNA7 APOC1 CHRNE CHRNA6 SLC5A7 GPR12 SESTD1 ACHE CRP PCTP HTR3E PCYT1A ZACN

GO_ANION_ANION_ANTIPORTER_ACTIVITY Catalysis of the transfer of a solute or solutes from one side of a membrane to the other according to the reaction: anion A(out) + anion B(in) = anion A(in) + anion B(out). SLC4A8 SLC22A6 SLC26A9 SLC22A9 SLC26A11 SLC37A2 SLC4A9 SLC37A4 SLC37A1 SLC4A3 SLC26A7 SLC25A11 SLC4A1 SLC26A6 SLC4A11 SLC26A8 SLC4A5 SLC22A7 SLC4A10 SLC4A2 SLC26A3 SLC26A10 SLC26A4 SLC37A3 SLC26A2 SLC22A8 SLC26A1 SLC26A5

GO_INTRACELLULAR_LIGAND_GATED_ION_CHANNEL_ACTIVITY Enables the transmembrane transfer of an ion by a channel that opens when a specific intracellular ligand has been bound by the channel complex or one of its constituent parts. HCN2 TPCN2 CNGB1 CNGA1 HCN1 ITPR2 MCOLN1 RASA3 CNGA2 CNGA3 TRPA1 HCN4 RYR1 CNGA4 KCNA10 RYR2 TPCN1 PKD2 ITPR3 CFTR CNGB3 ITPR1 FKBP1B AQP1 JPH2 TRPV1 RYR3 JPH3

GO_NADPH_BINDING Interacting selectively and non-covalently with the reduced form, NADPH, of nicotinamide-adenine dinucleotide phosphate, a coenzyme involved in many redox and biosynthetic reactions. KCNAB1 MTRR CBR3 DECR1 CBR4 DHFR QDPR CRYZ FASN HMGCR TP53I3 GRHPR DHFRP1 SRD5A1

GO_POLY_PYRIMIDINE_TRACT_BINDING Interacting selectively and non-covalently with any stretch of pyrimidines (cytosine or uracil) in an RNA molecule. DIS3L2 KHDRBS2 HNRNPH1 MSI2 PNPT1 PABPC1 PTBP1 RBM11 PABPC4 FMR1 ATXN1 MSI1 MCRS1 HNRNPC PATL1 U2AF2 KHDRBS1

GO_RECEPTOR_AGONIST_ACTIVITY Interacts with receptors such that the proportion of receptors in the active form is increased. NODAL SFRP2 WNT4 WNT2 VEGFA CXCL13 WNT3A GAS6 WNT1 WNT10B WNT7A WNT8A WNT3 WNT5A GREM1

GO_OXIDOREDUCTASE_ACTIVITY_ACTING_ON_THE_ALDEHYDE_OR_OXO_GROUP_OF_DONORS Catalysis of an oxidation-reduction (redox) reaction in which an aldehyde or ketone (oxo) group acts as a hydrogen or electron donor and reduces a hydrogen or electron acceptor. GAPDHS ADH7 FAR1 ALDH1A2 BCKDHA MRPS36 PDHA2 OGDHL ALDH3B1 ALDH4A1 AOX1 ALDH1A3 PDHA1 ALDH1L1 AKR1C4 ALDH9A1 ALDH3A1 GAPDH DLAT ALDH8A1 DLD LOC440905 ALDH1A1 PDHB FAR2 ALDH1L2 AKR1B1 ADH4 ALDH2 OGDH AKR1B10 PDHX ALDH6A1 ALDH1B1 AKR1C3 BCKDHB XDH DHTKD1 ALDH3A2 ALDH3B2 ALDH16A1 ADH5 ALDH7A1 HAO1 ALDH18A1 ALDH5A1

GO_OLFACTORY_RECEPTOR_ACTIVITY Combining with an odorant and transmitting the signal from one side of the membrane to the other to initiate a change in cell activity in response to detection of smell. OR11H1 OR10J6P OR2T6 OR5AK2 OR56B2P OR6Q1 OR4N2 OR4Q2 OR52E6 OR5V1 OR8K3 OR2V2 OR2Y1 OR2W6P OR1F1 OR51S1 OR52Z1 OR2T33 OR9Q2 OR5A1 OR52L1 OR51F2 OR4C6 OR5W2 OR51H1P OR1S2 OR6Y1 OR4F15 OR52N4 OR51I1 OR51G1 OR52I1 OR11H2 OR5H15 OR4L1 OR4A8P OR51A7 OR6C1 OR2D2 OR6C6 OR52I2 OR2H2 OR5D16 OR2S2 OR51D1 OR52B6 OR8H2 OR5AN1 OR2L8 OR10H5 OR4D6 OR52R1 OR4N4 OR4D5 OR56A3 OR8H3 OR2AK2 OR5A2 OR5H2 OR3A3 OR10A3 OR4F16 OR4X2 OR51G2 OR4E2 OR4A4P OR4C16 OR4A5 OR1J2 OR2T3 OR4D9 OR2H1 OR13C9 OR5AC1 OR8U9 OR4C12 OR8D1 OR10H4 OR4C46 OR52E8 OR2A7 OR7A2P OR11H4 OR8J1 OR2AP1 OR10J3 OR1L3 OR51I2 OR1F12 OR2AG2 OR10G3 OR52W1 OR4K3 OR6C75 OR8I2 OR14J1 OR52K2 OR4K17 OR13C3 OR1F2P OR5B2 OR2W5 OR5AR1 OR9A2 OR8B2 OR10AG1 OR2F1 OR2G2 OR10G7 OR8S1 OR5L2 OR5K4 OR2T2 OR4X1 OR4K15 OR7A10 OR6C74 OR5AC2 OR5P2 OR2G6 OR1L6 OR5T3 OR10C1 OR1N1 OR52B4 OR2L3 OR4A16 OR6B3 OR13G1 OR6M1 OR56A4 OR5D14 OR4E1 OR13C5 OR10D4P OR52B2 OR2T10 OR9I1 OR14A2 OR4A15 OR6C2 OR14L1P OR8D2 OR2W3 OR52E2 OR56A5 OR2T8 OR7C2 OR4D10 OR2AG1 OR4K2 OR4M1 OR1B1 OR5C1 OR5AL1 OR2T11 OR4F17 OR10G9 OR5B21 OR4C13 OR51A4 OR5M3 OR2A4 OR2L5 OR51M1 OR2C3 OR4K1 OR9G4 OR2T35 OR2A14 OR5H14 OR7D2 OR6C68 OR6J1 OR8G2 OR7C1 OR4D2 OR5B3 OR2AJ1 OR51B2 OR4F29 OR2AE1 OR10S1 OR1K1 OR8U8 OR2K2 OR2M7 OR2T4 OR4C15 OR6K2 OR5AS1 OR11H12 OR2A12 OR8D4 OR1M1 OR4K13 OR5M9 OR10H3 OR13C8 OR7G2 OR13H1 OR51A2 OR10W1 OR5L1 OR2M3 OR5AP2 OR2J2 OR12D2 OR9A1P OR9G9 OR5H6 OR56B1 OR2C1 OR2J3 OR7D4 OR2A1 OR4S2 OR5M11 OR5J2 OR10G6 OR10P1 OR52N5 OR8K1 OR51F1 OR8K5 OR1N2 OR6C65 OR7A17 OR8J2 OR5K2 OR52K1 OR5R1 OR8G1 OR12D1P OR10J1 OR5M1 OR10X1 OR9G1 OR6F1 OR10K1 OR4D1 OR5G3 OR7G3 OR6V1 OR5D13 OR8J3 OR51V1 OR14C36 OR1D2 OR13C4 OR10G8 OR2W1 OR4C45 OR8U1 OR2B3 OR8B4 OR7A5 OR7G1 OR4M2 OR10A4 OR1E2 OR1J1 OR2L13 OR1E3 OR4F21 OR2T27 OR2I1P OR52A4 OR8B8 OR11H7 OR52M1 OR2D3 OR1I1 OR2M2 OR4C3 OR11A1 OR2B2 OR6T1 OR10AD1 OR1Q1 OR2B8P OR5I1 OR1J4 OR1G1 OR2Z1 OR52N2 OR51E1 OR1L8 OR2G3 OR6K3 OR2A42 OR13C7P OR5H1 OR52J3 OR10H1 OR52D1 OR13D1 OR6X1 OR52L2P OR10AC1P OR56B4 OR1P1 OR2B6 OR5T2 OR6N2 OR4N5 OR6A2 OR52A5 OR2T5 OR13J1 OR2B11 OR6C76 OR14A16 OR4D11 OR10V1 OR6P1 OR9K2 OR4K14 OR4Q3 OR2AT4 OR9A4 OR51Q1 OR13F1 OR2L2 OR4F4 OR10A2 OR5D18 OR10A5 OR4B1 OR2T29 OR2T7 OR2F2 OR8G5 OR11G2 OR52N1 OR13C2 OR10A7 OR2A5 OR6N1 OR52A1 OR4S1 OR8H1 OR3A1 OR1A2 OR10J5 OR5P3 OR1D4 OR1D5 OR6S1 OR52E1 OR10Q1 OR10G4 OR51J1 OR1S1 OR8G3P OR2A25 OR4C5 OR5F1 OR11H6 OR6C4 OR5M10 OR10R2 OR51B4 OR3A2 OR4F6 OR10J4 OR2M4 OR51B6 OR5T1 OR4C11 OR1E1 OR51L1 OR5B12 OR10T2 OR5B17 OR13A1 OR51T1 OR52P1P OR8B12 OR10H2 OR10Z1 OR52E5 OR5M8 OR6C3 OR2A2 OR51E2 OR9Q1 OR6B1 OR52E4 OR4F5 OR52H1 OR5K3 OR1L4 OR10K2 OR4K5 OR10G2 OR6C70 OR7E24 OR5H8P OR10D3 OR2T34 OR5K1 OR2V1 OR6B2 OR56A1 OR10A6 OR2T12 OR1L1 OR14I1 OR12D3 OR11L1 OR2T1 OR5AU1 OR2J1 OR4P4 OR2M5 OR8B3 OR1A1 OR51B5 OR4A47 OR5AK3P OR4F3 OR6K6 OR1C1 OR8A1

GO_L_ASCORBIC_ACID_BINDING Interacting selectively and non-covalently with L-ascorbic acid, (2R)-2-[(1S)-1,2-dihydroxyethyl]-4-hydroxy-5-oxo-2,5-dihydrofuran-3-olate; L-ascorbic acid is vitamin C and has co-factor and anti-oxidant activities in many species. EGLN1 LEPREL1 P4HA3 C17orf101 P4HA2 TMLHE PAM PLOD2 PLOD1 LEPRE1 DBH PHYH OGFOD1 P4HTM EGLN3 P4HA1 EGLN2 OGFOD2 PLOD3 LEPREL2 ALKBH3

GO_RAGE_RECEPTOR_BINDING Interacting selectively and non-covalently with the RAGE receptor, the receptor for advanced glycation end-products. HMGB1 S100A8 S100B HMGB2 S100A7 S100P S100A13 S100A4 FPR1 S100A12 S100A9

GO_STRUCTURE_SPECIFIC_DNA_BINDING Interacting selectively and non-covalently with DNA of a specific structure or configuration e.g. triplex DNA binding or bent DNA binding. CHD4 ERCC4 H2AFY2 RCC1 H1F0 PITX2 TET1 SMARCC2 KDM5A H3F3A WBP2 NHEJ1 FAN1 PPARGC1A MBD2 RELA THRA MYOG ACTL6A VAX2 HR FOXO3 RECQL4 MYOD1 HMGN5 XRCC3 HIST3H3 ACTB SUZ12 LONP1 PIF1 PRDM14 TDG HIST1H3H SMAD3 JMJD1C H3F3B STAT3 HMGB2 KDM3B GRHL1 GRHL3 H2AFY RAD51C EZH2 MED1 MSH6 ZBP1 MEN1 GATA1 ERCC1 INSM1 GATAD2B CLOCK MBD3 SMARCE1 HDAC1 RAD51 HIST2H3C HMGN2 APEX1 H3F3C RAD50 ERCC5 RPS3 HIST1H3G RBBP4 HIST1H3C RUVBL2 KDM3A H2AFZ SMARCB1 SMARCD2 NEIL3 ENDOV MSH2 HDAC2 NOTCH1 ACTN4 HMGN4 DHX36 SETMAR XRCC2 HIST1H3E HIST1H3A XPC RXRA NKAP YY1 VAX1 HMGN1 DMC1 NEUROG3 RAD51B ZIC2 HMGB3 THRB HMGA2 BLM BCL6 FOXC2 HIST1H3D HIST1H1D HIST1H3B HMGB1 RAD51D KDM6A PSIP1 HIST1H1B MTA2 HIST1H1C HIST1H1A HIST1H3I HIST2H3D SMARCC1 HIST2H3A SMARCA4 HIST1H3F ATOH1 GRHL2 CENPA SRF CTCF PER1 RARA WRN HMGN3 H1FOO HIST1H3J NR0B1 EP300 RAD18 HIST1H1E HNRNPC

GO_DAMAGED_DNA_BINDING Interacting selectively and non-covalently with damaged DNA. RBBP8 DDB2 REV1 GTF2H3 BRCA1 MSH4 XPC MPG ERCC1 XRCC1 MSH6 TP53 TP73 MSH3 RAD18 EP300 CREBBP NEIL2 POLQ RAD23B FANCG MSH5 CRY2 RPA3 FEN1 NEIL1 HMGB1 POLH DCLRE1A UNG RPA1 DCLRE1C PCNA APEX1 RAD1 ERCC4 RPS3 RPA2 XPA ERCC3 CUL4B MSH2 NEIL3 APTX LOC100133315 XRCC6 NBN PNKP XRCC5 TDG DDB1 POLD1 POLI POLK H2AFX RAD23A HMGB2 POLB MGMT TP63 DCLRE1B OGG1

GO_L_AMINO_ACID_TRANSMEMBRANE_TRANSPORTER_ACTIVITY Enables the transfer of an L-amino acid from one side of a membrane to the other. L-amino acids are the L-enantiomers of amino acids. SLC32A1 SLC25A2 SLC7A4 SLC1A4 TMEM44 SLC43A1 SLC3A1 SLC7A1 SERINC5 SLC36A3 SLC7A6 SLC38A7 SERINC1 C3orf55 SLC6A5 SLC7A8 SLC6A15 SLC7A13 SLC7A5 SLC38A3 SLC7A9 SLC36A4 SLC1A5 PQLC2 SLC7A3 SLC36A1 SERINC3 SLC7A10 SLC6A7 SLC7A7 SLC36A2 SLC25A15 CTNS SLC6A20 SLC38A1 SLC7A2 SERINC4 SERINC2 SLC38A5 SLC6A9 SLC7A14 OCA2 SLC43A2

GO_OXIDOREDUCTASE_ACTIVITY_OXIDIZING_METAL_IONS_NAD_OR_NADP_AS_ACCEPTOR Catalysis of an oxidation-reduction in which the oxidation state of metal ion is altered and NAD+ or NADP+ acts as an electron acceptor. STEAP1 STEAP4 CYB561 STEAP2 MMACHC STEAP3 CYBRD1 POR MTRR STEAP1B FRRS1

GO_AMINO_ACID_TRANSMEMBRANE_TRANSPORTER_ACTIVITY Enables the transfer of amino acids from one side of a membrane to the other. Amino acids are organic molecules that contain an amino group and a carboxyl group. SLC7A5P1 SLC7A8 SLC6A5 SLC6A15 SLC1A3 SLC38A10 SLC38A3 SLC38A6 SLC1A4 SLC7A4 SLC25A2 SLC43A1 SLC25A12 SLC6A14 SLC7A1 SLC3A1 SERINC5 SLC7A6 SLC38A7 SERINC1 SLC17A6 SLC36A2 SLC38A1 SLC17A8 SLC6A9 SLC1A6 SLC7A5P2 SLC43A2 SLC7A11 SLC1A5 PQLC2 SLC7A3 SLC3A2 SLC1A1 SLC36A1 PEX3 SLC6A7 SLC38A2 C3orf55 SLC6A19 SLC38A4 SLC7A13 SLC7A5 SLC6A13 SLC6A17 SLC6A11 SLC7A9 SLC38A8 SLC32A1 SLC6A12 PDPN TMEM44 SLC38A11 SLC25A13 SLC6A6 SLC36A3 SLC6A16 SLC6A20 CTNS SLC7A2 SLC25A15 SERINC2 SERINC4 SLC6A18 SLC38A5 SLC7A14 SLC6A1 OCA2 SLC16A10 SLC25A22 SLC36A4 SLC38A9 SLC1A2 SLC1A7 SERINC3 SLC7A7 SLC17A7 SLC7A10

GO_LIGAND_DEPENDENT_NUCLEAR_RECEPTOR_TRANSCRIPTION_COACTIVATOR_ACTIVITY The function of a transcription cofactor that activates transcription in conjuction with a ligand-dependent nuclear receptor from a RNA polymerase II promoter; does not bind DNA itself. C1orf85 CCAR1 SLC30A9 ZMIZ2 FGF2 BUD31 MED30 MED13 ACTN2 NCOA1 ACTN4 PPARGC1B WDR77 PRIC285 RBM14 NCOA6 CCDC62 ACTN1 NCOA3 NCOA7 MED14 PRKCB MED12 KDM1A ENY2 PPRC1 TADA3 NCOA2 ZCCHC12 MED17 USP22 TSG101 MED4 SFR1 MED16 PPARG PKN1 SRA1 ATXN7L3 CALCOCO1 PSMC3IP PPARGC1A MED1 CARM1 MED24 HMGA1 TRERF1 DCAF6 THRAP3 SS18 ZCCHC18

GO_NADP_BINDING Interacting selectively and non-covalently with nicotinamide-adenine dinucleotide phosphate, a coenzyme involved in many redox and biosynthetic reactions; binding may be to either the oxidized form, NADP+, or the reduced form, NADPH. GMDS DHFR NOS3 DUOX1 NOX5 NOX1 DHFRP1 GAPDHS HMGCR NDOR1 NNT KCNAB1 NOS1 DECR1 H6PD CRYM GRHPR SRD5A1 FMO5 DHCR7 MTHFR ME1 GAPDH CBR4 FMO4 TP53I3 MTRR CAT CRYZ QDPR CRYZL1 DHFRL1 IDH1 FASN TM7SF2 POR CBR3 SPR NOS2 FMO3 FMO2 GSR DPYD FMO1 ASPDH G6PD

GO_ODORANT_BINDING Interacting selectively and non-covalently with an odorant, any substance capable of stimulating the sense of smell. OR10Q1 OR5G3 OR8D2 OR14L1P OR5P3 OR14A2 OR9I1 OR5W2 OR5M1 OR9Q2 OR5A1 OR8H1 OR5R1 OR8G1 OR5D14 OR6B3 OR8G5 OR8J2 OR5K2 OR5D18 OR8K3 OR5AK2 OR8K5 OR9K2 OR8K1 OR10V1 OR5P2 OR14A16 OR5AC2 OR5H2 OR5A2 OR5B17 OR13A1 OR5H14 OR5B12 OR8H3 OR9G4 OR8B4 OR8U1 OR5M3 OR5AN1 OR8H2 OR5M10 OR5D16 OR5B21 OR5F1 OR5AL1 OR5C1 OR14C36 OR8J3 OR5H15 OR5D13 OR8J1 OR5M9 OR5I1 OBP2A OR6B2 OR5K1 OR8D4 OR8D1 OR5AS1 OR8U9 OR5AC1 OR5K3 LCN9 OR8U8 OR9Q1 OR5M8 OR5B3 OR8B8 OR8B12 OBP2B OR8A1 OR5J2 OR5K4 OR5L2 OR5M11 OR5AK3P OR5H6 OR8B2 OR5AR1 OR5B2 OR8B3 OR5AP2 OR8I2 OR5H1 OR14J1 OR5AU1 OR5L1 OR10W1 OR11L1 OR14I1

GO_SINGLE_STRANDED_DNA_DEPENDENT_ATPASE_ACTIVITY Catalysis of the reaction: ATP + H2O = ADP + phosphate; this reaction requires the presence of single-stranded DNA, and it drives another reaction. RAD18 POLQ PIF1 RFC3 RFC2 HELB RAD51 DNA2 CHTF18 RFC4 RFC5 DSCC1

GO_INOSITOL_PHOSPHATE_PHOSPHATASE_ACTIVITY Catalysis of the reaction: inositol phosphate(n) + H2O = inositol phosphate(n-1) + phosphate. This reaction is the removal of a phosphate group from an inositol phosphate. INPP5B PTEN INPP4B INPP1 MINPP1 INPP4A IMPA1 INPP5A INPPL1 INPP5J SYNJ1 OCRL INPP5K IMPA2 INPP5F INPP5D INPP5E ITPK1 IMPAD1

GO_NEUROTRANSMITTER_BINDING Interacting selectively and non-covalently with a neurotransmitter, any chemical substance that is capable of transmitting (or inhibiting the transmission of) a nerve impulse from a neuron to another cell. CHRNA3 CHRNA5 CHRNA7 CHRNA2 CHRND HTR3D CHRNB3 SLC18A3 CHRNB4 CHRNG CHRNA1 IDE HTR3C HTR3A CHRM3 HTR3B ZACN GRIN3B CHRNA4 NIPSNAP1 CHRNB2 CHRNB1 CHRNA9 HTR3E ACHE GRIN1 CHRNA6 CHRNA10 SLC6A11 CHRNE

GO_SNORNA_BINDING Interacting selectively and non-covalently with small nucleolar RNA. NUDT5 UTP6 NHP2L1 DIEXF TBL3 NUDT16L1 NOP14 IMP4 WDR3 BMS1 NUDT1 NUFIP1 NOP58 HEATR1 NOP56 ISG20 PWP2 NOP10 GAR1 TSR1 DKC1 NUDT7 RRP9 DDX21 NUDT16 IMP3 NUDT4 NHP2

GO_LYSOZYME_ACTIVITY Catalysis of the hydrolysis of the beta-(1->4) linkages between N-acetylmuramic acid and N-acetyl-D-glucosamine residues in a peptidoglycan and between N-acetyl-D-glucosamine residues in chitodextrins. LYZ LYZL2 SPACA5 LALBA LYZL6 LYG2 CHIA LYZL1 LYG1 SPACA5B LYZL4 SPACA3

GO_FMN_BINDING Interacting selectively and non-covalently with flavin mono nucleotide. Flavin mono nucleotide (FMN) is the coenzyme or the prosthetic group of various flavoprotein oxidoreductase enzymes. CREG1 DHODH HAO2 NOS1 NOS2 MTRR NDOR1 POR TYW1 PNPO CREG2 TYW1B NOS3 NDUFV1 HAO1

GO_THIOLESTER_HYDROLASE_ACTIVITY Catalysis of the reaction: RCO-SR' + H2O = RCOOH + HSR'. This reaction is the hydrolysis of a thiolester bond, an ester formed from a carboxylic acid and a thiol (i.e., RCO-SR'), such as that found in acetyl-coenzyme A. PNKD ACOT12 LOC344967 ACOT6 GNPAT BAAT ACOT1 ACSBG2 HIBCH HAGH NUDT19 ACOT2 PPT2 ACOT8 LYPLAL1 ACOT7 LYPLA1 ACOT9 PPT1 ACOT4 THEM5 OLAH ESD FASN UFSP1 NUDT7 UFSP2 LYPLA2 ACOT13 THEM4 ACOT11

GO_TOXIC_SUBSTANCE_BINDING Interacting selectively and non-covalently with a toxic substance, a poisonous substance that causes damage to biological systems. CYP4B1 DSG1 PHAX AZU1 CHRNA7 ASS1 TMEM181 EPHX2 ALB A4GALT GUCY2C

GO_ATP_DEPENDENT_DNA_HELICASE_ACTIVITY Catalysis of the reaction: ATP + H2O = ADP + phosphate; this reaction drives the unwinding of the DNA helix. FBXO18 GTF2H4 BLM CHD2 CHD1 DDX3X MCM6 RUVBL1 RECQL ERCC2 HELB NBN XRCC5 RECQL5 PIF1 XRCC6 CHD3 DDX12P DDX11 WRN RTEL1 ASCC3 MCM4 CHD1L IGHMBP2 MCM7 RAD50 BRIP1 RECQL4 CHD4 G3BP1 MRE11A RUVBL2 ERCC3 DHX9

GO_NON_MEMBRANE_SPANNING_PROTEIN_TYROSINE_KINASE_ACTIVITY Catalysis of the reaction: ATP + a non-membrane spanning protein L-tyrosine = ADP + a non-membrane spanning protein L-tyrosine phosphate. TXK STK16 FGR PTK6 EIF2AK2 ITK TEC LYN YES1 TNK1 STYK1 WEE1 HCK JAK2 SGK223 TNK2 MELK CLK1 RIPK2 PTK2B PEAK1 BMX BTK MATK TYK2 LCK BLK DYRK1A FES PKDCC SRMS PRKCD WEE2 BAZ1B JAK1 SRC FYN ZAP70 FRK CSK PTK2 ABL2 JAK3 SYK FER ABL1

GO_RRNA_BINDING Interacting selectively and non-covalently with ribosomal RNA. RPF2 NOL12 MRPL20 MRPL16 RNASEL MTERFD1 TST ANG SBDS RPL11 MRPS7 MRPS17 RPL12 MTERFD2 RPL3 RPL37 RPS11 ERAL1 DDX28 MRPS15 UTP23 RPLP1 RPS3 ERI1 MRTO4 RPL9 MRPL18 MRPL11 PPAN RPS5 IMP3 MRPS6 DIEXF PTCD3 RPL23 IMP4 RPS9 EMG1 RPS14 KDM2B RPS4Y1 PTRF EEF2 RPLP0P6 MRPS11 RPS18 RPS4Y2 RPL5 RPS13 RPF1 FASTKD5 RPL19 RPS4X RPLP0 NSUN4 CIRBP RPL8 FASTKD2 RPLP2 RPL23A DDX21

GO_PHOSPHOLIPID_TRANSLOCATING_ATPASE_ACTIVITY Catalysis of the movement of phospholipids from one membrane bilayer leaflet to the other, driven by the hydrolysis of ATP. ATP8A2 ATP8B2 ABCA7 ATP8A1 ATP10D ABCA4 ATP11A ATP9A ATP10B ATP8B4 ATP11B ATP10A ABCA1 ABCB4 ABCB1 ATP8B1 ATP8B3 ATP9B ATP11C

GO_METALLOAMINOPEPTIDASE_ACTIVITY Catalysis of the hydrolysis of N-terminal amino acid residues from a polypeptide chain by a mechanism in which water acts as a nucleophile, one or two metal ions hold the water molecule in place, and charged amino acid side chains are ligands for the metal ions. TRHDE ERAP2 MMP17 AQPEP DNPEP ANPEP XPNPEP2 MMP16 XPNPEP1 METAP1 LOC440434 RNPEP METAP1D LNPEP METAP2 NPEPPS MMP14 RNPEPL1 LTA4H C9orf3 MMP15 ENPEP ERAP1

GO_TRANSPORTER_ACTIVITY Enables the directed movement of substances (such as macromolecules, small molecules, ions) into, out of or within a cell, or between cells. HCN3 SLC12A5 SEC14L3 ATP6V0E1 SLC9A6 CLCN6 SLC41A2 SLC8A3 TOMM5 CSN2 NUP133 LRRC8E ABCG4 CYBB GRIA4 ATP2B4 SLMO1 SLC5A2 MFSD4 ATP10B XPO5 CNGA3 PTK2B KCNG3 PRELID1 MFSD10 SFXN4 APOD SLC23A2 NDUFA4L2 RASSF9 ATOX1 KPNA2 CD320 SLC45A4 ABCC8 SLC17A4 AP3S2 EIF4ENIF1 NIPA2 CLIC2 ATP6AP1 GAS6 PLSCR1 SLC30A5 SLC37A4 GABRA3 STARD4 CFHR4 AQP6 KCNK12 HBE1 CRABP1 CACNA1E LMBRD1 RAMP2 UQCRHL DLG4 SLC14A1 COX4I2 ATP6V0A4 SLC13A2 SLC25A37 CLDN17 SLC26A7 DLG1 LRP5 SEC61A1 TOMM40L GRID1 SLC35D2 SLC36A4 SLC38A3 VDAC1 OSBP SLC46A1 PANX2 FXYD7 KCNH1 PCYOX1 ABCB9 ABCB7 SLCO5A1 CSE1L HBD HTR3D ATP2A2 KPNA3 SLCO1B3 ABCA1 PLSCR2 SLC1A6 TF KCNAB1 PKD1L3 NNT SLC16A8 SLC23A3 ATP1A4 PEX3 TTYH1 CCT8L2 C9orf5 LCN12 FABP6 ATP5J KCNA2 SLC7A9 SLC12A1 COX7A2P2 TRPV6 KCNB1 SLC41A1 HCN4 SLC7A13 SLC6A19 ABCB10 FXYD6P3 UQCRFS1 KCNH5 GABRA5 TMC1 CACNA1F SLC38A11 CALHM2 RHCG PDE2A AQP12B ANO6 APOA1 VPS29 ABCB5 GABRR1 FAM26F FAM26D SLC6A3 ABCC2 SLC31A1 OSTalpha KCNH7 PLEKHA8 GRIK1 GRIN3A GRIN2B NUPL2 SCN11A SVOP SLC4A8 NIPAL2 SLC9A7 SLC30A1 CACNG1 SLC26A4 AZGP1 SLC4A2 ATP1A1 TOMM22 COX5A ABCB4 APOB SLC17A2 IPO9 CLVS1 SLC3A1 ATP8B2 USO1 KPNA4 TRPC5 CLIC5 ATP2B3 SLC39A7 SLC25A2 CHRNA2 COX4I1 SLC39A14 SLC25A32 KCNJ9 GRIN2A GPM6A CPNE6 SLC6A9 SLC37A3 ATP6V1D TMCO1 C2orf83 SPNS3 CYGB PKD1L1 ORAI1 ATP5H MRS2 GJA3 SLC23A1 SLC6A17 KCNJ1 SLC7A5 STRA6 SLC24A3 TOMM7 SLC16A11 SLC38A2 XPR1 FABP2 AP2B1 SLC16A14 CACNB3 KCNV1 ABCF2 ABCC11 SLMO2 RANBP6 KCNS1 AP2S1 TIMM22 GABRG1 TRAPPC10 CACNG7 FABP7 KCNAB2 SLC5A12 OCA2 SLC24A4 FABP4 MIP SLC15A5 KCNK18 CHRNB2 PKDREJ SLC6A20 KCNA4 ATP13A4 KCNV2 SLC30A10 AP1G1 TPCN1 GRIA3 TMEM184B NUP160 KIF20A VPS26B CALHM1 CHRFAM7A COX6B1 SLC30A3 POM121 SLC27A4 SLC22A10 TUSC3 PCTP NUP88 CACNA2D2 SLC7A8 TCIRG1 SEC61G SLC5A3 KCNIP1 SERINC1 SLC6A2 GRID2 CALM1 SLC25A31 SLC2A12 CCT6B ATP1A2 TRPM7 SLC16A7 SLC46A2 MTTP CACNG4 SLC35A5 SLC25A42 NUP107 ABCA2 POM121L12 PRF1 KCNK6 REST ATP10D SLC24A2 CNNM2 NUP98 COG3 C11orf73 CACNA1G SLC14A2 SLC1A5 SIDT2 GLTP SLC37A2 S100A6 TRPV1 SLC38A8 CALHM3 SLC15A1 CATSPER2 SLC6A13 SLC33A1 PPBP KCNJ13 TTPAL SLC34A1 KCNQ3 SLC22A24 SLC27A6 LRRC8C GLRA1 SCN10A SLC25A11 AP3D1 TMEM44 ANO2 SLC22A2 ITPR2 SLC22A15 SLC4A4 P2RX3 KCNMB4 CLDN16 PKD2L1 KCNE1L CYB5A TIMM17A CHRNB4 CLCN4 ITGB1BP1 ATP6AP1L NIPAL3 AP4B1 CLVS2 KCNE2 AP1B1 LRRC8A ABCA4 SLC38A6 NUP155 KCNQ1 POM121L2 ATP6V1B1 HCN2 ATP10A ATP6V0D1 CACNA1B TRPV2 PACSIN2 SEC24B HTR3A FABP1 MIA3 KCNK9 JPH3 ABCG5 ZP3 SLC18A3 CHRNA7 SCNN1B MB TNPO2 ATP5L2 ATP6V1H PKD1 RBP7 P2RX7 CLIC6 ABCC13 GLRA3 KCNG4 IPO4 SLC30A4 GJB3 ACCN5 SLC10A7 HBG1 KCNA3 TSNAX SLC5A10 BSG POM121C SLC5A4 ATP1B4 NDUFA4 C6orf192 MCL1 BEST4 C3orf55 PLSCR5 MFSD3 ARFGAP3 SLC6A6 CHRNB3 SLC6A4 NUP153 SCN8A KCNAB3 UQCR11 TMEM37 SERINC4 AQP2 CTNS SLC11A2 ATP6V1E1 TMEM38B HVCN1 RAMP1 AQP1 ANO7 TIMM17B RLBP1 KPNA6 SCN4A GLTPD2 ABCA6 UCP3 PANX1 SLC10A5 SLC38A9 ATP6V0C GABRG3 ANO1 SLC44A2 ATP6V1C1 RYR1 OSTBETA SLC22A11 IL1RAPL1 DLG3 SLC15A4 CETP ATP5C1 HBB STAR TIMM9 PLP2 COG2 KCNJ6 ABCA13 C9orf7 SLC43A2 KCNT2 ATP5J2 ABCC12 DISP1 SLC22A4 SLC26A6 COX7B2 ATP4A SLC10A3 NCS1 SLC2A3 FAM26E SLC15A2 APOC4 CHRNE SLC27A1 TPCN2 KCNE3 GABRA1 ANO10 GABRD SLC22A13 SCN3A SLC38A4 ATP8B3 CATSPER4 TMCO6 CLCNKB ATP9B ZDHHC17 SLC22A18 GABRB2 PANX3 PDPN JPH2 CACNA1C KCNQ2 ATP6V0A1 SLC39A10 KCNJ18 SLC32A1 SLC39A8 IPCEF1 OSBPL5 SLC38A5 GRIA2 CACNG2 SCNN1G COX15 KCNJ15 COX7C COX11 SLC2A14 SLC35A1 KCNN1 SLC41A3 SLC25A20 ATP1B3 TRIAP1 ABCC6 PRELID2 KPNA1 CHMP7 AP1S2 CALCRL STARD10 GLTPD1 CACNA2D1 XPO1 SLC35A3 KCNG1 UQCRQ SEC14L2 TTPA CNGB3 NIPAL4 SLC2A7 SLC22A8 ATP2B1 XPOT APOA4 SNUPN KCNK5 KCNQ5 ABCA12 CLCN5 ATP5I SLC39A2 SLC2A8 CACNG8 SLC2A5 SLC39A11 ADAMTS8 TMC2 KCNH4 KCNK1 KCNIP2 CALM3 XK SLC35E3 SYN1 AP1S1 PIEZO1 SLC16A4 SLC27A2 KCNMB3 P2RX1 SFXN5 EMB KCNJ14 KCNIP3 UQCRB KCNK2 RBP2 TMEM184A SLC26A5 KCNA6 SLC29A2 SLC48A1 SLC17A5 ANO4 ATP12A SLC25A15 SLC28A2 SLC7A10 CLCN1 SLCO1B7 CRABP2 SLC4A3 KCNA7 SLC2A13 GRIK2 SLC9A4 COX7A2L KCNN3 P2RX6 SLC38A10 HCN1 FABP9 ATP1B1 TRPA1 ATP5G1 ABCG1 CACNA2D3 ASNA1 KCNC2 C16orf7 SLC25A5 SLC25A10 CPLX3 ORAI3 KCNH3 SLC1A4 SLC19A2 XPO4 SLC12A6 ATP5L RHBG MFSD9 VDAC2 KCNMA1 PGAP2 ATP13A3 C20orf79 FABP12 TRPC4 GM2A SLC25A18 COX5B FKBP1B CATSPER3 SCN5A KCNG2 GJB1 KCNS2 KCNH8 PQLC2 LRP2 SLC24A1 KPNA7 IPO5 ATP6V1F ANO8 CNGA4 COX7A1 SLC6A16 P2RX4 CLIC1 CSN1S1 SLC9A5 ABCD2 CHRND ANO9 CDH17 TMEM63C HBA2 SERINC2 ATP5D FXYD6 BSND SLC12A2 SPNS2 KCNH2 SLC39A13 SLC16A3 COX7A2 TMEM184C NCALD SLCO4A1 CACNG5 TMEM38A XPO6 SLC4A7 KCNK17 TAPBP RUFY1 P2RX2 KIAA1919 AP3S1 FXYD1 SEC22A SLC2A4 CHRNA10 GLRB NXF1 SLC26A3 TIMM23 SLC4A5 SLC44A1 CHRNB1 AQP8 HIAT1 SLC36A2 ABCB1 ATP8B1 CPNE7 GABRA6 SLC6A7 GRIK4 CHRNG ITPR3 GRIN2C ACCN1 CLCA4 KCNQ4 SLC19A3 SLCO6A1 TOMM40 SLC6A11 OAZ3 SCARB1 TOMM70A SLC45A2 GABRQ COL4A3BP SLC22A16 ABCC3 RHCE SLC39A9 FABP5P3 SLC5A1 CHP MMGT1 RBP4 SLC22A25 SLC25A36 GRM7 SLC9A9 SLC7A14 STEAP1 ATP6V0D2 MAL KCNC4 TAP2 SLC26A8 COX6A2 SLC45A1 SLC27A5 CACNA1S ANO3 HTR3B SCN1B SLC1A7 SEC14L6 ATP6V0B SLC12A4 SLC18A2 ATP6V0E2 SLC35B2 SLC13A1 SLC47A1 KCND3 RYR3 RAB4A CHRNA6 SEC62 APOF SYNPR SLCO2A1 KCNE1 ATP5O HTR3C PITPNM3 SLC7A4 SLC2A10 IPO13 PITPNA NOX5 SLC22A14 TOMM20 ATP6V1G3 RHD SLC25A16 DSCR3 C15orf48 GABRR2 SLC22A1 SLC2A2 AP2A2 TRPM4 BRP44 FXYD2 SLC10A6 HIATL2 CALM2 SLC22A3 SLC24A5 CPNE1 PMP2 CLCA3P ABCA3 GABRG2 KCND1 CACNG6 SLC5A11 HTR3E NIPA1 UPF3A SLC50A1 KCNA10 RANBP17 SLC9A10 SFTPA1 LRP1 SLC22A9 GJA5 SLC29A4 HBG2 COX6A1 HPX SLC25A19 SEC14L4 KCNK10 TRPV4 TRPC4AP C15orf2 AKR1C4 GLRA2 SLC35A4 SLC29A1 SLC26A1 SLC5A7 ATP5G3 KCNJ11 GJB2 OPRM1 DENND5B SLC5A6 SLC10A2 SLC16A5 SLC6A15 SLCO1C1 ATP6V1A SLC44A3 SYP SLC30A9 ATP2A3 ATP2A1 AQP7 NMUR2 CHRNA1 SFXN1 PITPNC1 SLC25A12 SLC22A23 SEC14L5 SLC17A9 CHRNA3 KCNIP4 CACNB4 ARV1 SLC9A11 SLC7A5P2 SLC30A7 POM121B KCNJ12 SLC35D1 NOX1 KPNB1 SLC40A1 GPR172B GABRA2 SCP2 SLC16A6 NUP35 ATP7A SLC25A28 ZDHHC13 ABCB6 KCNJ8 SLC2A11 SLC26A2 COX8C PKD1L2 AQP4 SLC28A3 COX18 SLC8A2 MLC1 GRIK3 GRIK5 VLDLR TFRC CALCR UQCRFS1P1 LRRC8D SLC22A17 SLC25A38 SLC39A12 SLC25A26 SYPL2 SLCO2B1 CUL5 SLC35B4 SLC44A5 SLC34A2 APOL1 SV2A SERINC3 SLC35B3 KCNJ10 CHRNA5 SLC26A9 SLC39A1 RHAG FABP3 SLCO3A1 SLC22A7 ANXA6 ADC GLRA4 NUP214 IGF2R SYPL1 SLC7A5P1 ABCA8 SLC7A6 PKD2 SLC6A14 SLC6A8 SURF1 KCNF1 SLC5A9 TNPO1 SLCO4C1 SFXN3 M6PR ACCN2 PITPNM1 CNGB1 FLVCR1 SLC16A9 CNGA2 STARD7 DENND5A SLC9A1 SLC4A11 SLC16A2 AQP11 CACNA1H GOLGA3 SLC22A5 SLC3A2 GPR89A KCNC3 AP2M1 SCNN1D AP4M1 SLC13A4 IPO11 CNNM4 LCN9 TRPM6 CPNE3 ABCA5 GRIN3B SLC13A3 CLCNKA TMCO3 CACNA2D4 APOA2 AQP5 SLC25A13 NUPL1 MCOLN2 KCNB2 SLC6A12 PTGDS SCN9A SLC35C1 RAP1A ABCG2 CLCA1 CLCN3 KCNJ4 ITGAV HBA1 GABRR3 VPS26A COX6B2 CHRNA4 SVOPL SLC2A6 ATP11C AQP10 SLC4A1 SLC28A1 GRIN2D TIMM10 SLC13A5 NUP54 SLC1A2 P2RX5 KCNJ16 TMEM109 SLC25A22 RBM22 CLIC4 SLC47A2 MCOLN3 SCN2B ATP5E SLCO1A2 AP2A1 FABP5 SLC39A6 ATP4B AP1S3 SLC39A5 SLC6A5 SLC35B1 AP4S1 APOA5 GC KCNJ3 BEST3 SLC7A1 GAR1 PIEZO2 SLC43A1 CFTR KCNMB1 ORAI2 STIM2 ABCG8 FXYD4 HBM CCDC109B SLC17A8 SCNN1A MAGT1 BRP44L BEST2 BCL2A1 SLC36A1 UCP1 SLC39A4 KCNK16 NGB APOE SLC25A21 NUP62CL GABRB3 ABCA9 SLC9B1P1 SLC2A1 AQP12A CNGA1 FOLR2 TPR SLC16A12 MFSD5 GABRA4 SLC10A4 SLC9B1 ZFYVE16 HBQ1 SLC20A1 CUBN ATP5G2 KCNK15 UQCR10 SLCO1B1 KPNA5 SLC16A10 TMEM175 CYB561 SLC10A1 CLCA2 SLC7A2 ABCC5 MCOLN1 SLC25A33 KCND2 ATP5S LASP1 SLC25A17 ITPR1 TRPM3 SCN3B ANKH SLC30A6 HNRNPA3 CACNA1A HIATL1 ATP11A RANGRF NPC1 SLC9A3 FOLR1 SLC22A31 SCN1A SLC15A3 SCN4B SPNS1 SLC16A1 KCNE4 GRIA1 SLC37A1 GJD3 SV2B KCNK7 ABCC10 ABCA10 ACCN4 CLIC3 KCNH6 UQCRH NALCN CACNA1D GRIN1 ATP8B4 KCNN4 MCU SLC25A3 VDAC3 SLC7A3 STARD5 GPR172A SLC22A6 FXYD3 SLC25A29 SLC30A2 ATP5B SLC5A5 KCNJ2 COX10 TRPM8 SLC2A9 SLC9A2 SLC20A2 HBZ NUP62 RBP5 CATSPER1 TRPV5 SLC18A1 ATP2B2 HPN TOMM20L SLC26A10 SLC6A18 BEST1 ATP2C1 RAMP3 ABCC4 GJA8 SLC9A8 LCN2 PDZK1 ATP6V1G1 ATP5A1 CLCN7 IPO7 C8orf38 CACNB1 TRPC7 APOL3 CLCN2 SLC22A12 NIPAL1 SLC1A3 RAN ABCD1 COX8A ATP13A5 C1orf31 TNPO3 RFT1 TRPV3 KCNS3 SERINC5 UQCRC1 KCNN2 GJD2 ABCD4 ABCB11 XPO7 ABCD3 ATP11B ATP2C2 SLC44A4 SLC38A1 COX7B TNFAIP8L3 ABCB8 SLC1A1 SLC34A3 CLCC1 BCL2 ATP9A KCNA1 FLVCR2 TTYH3 TRPM5 KCNU1 SLC16A13 SLC17A1 GABRP SLC5A8 SLC22A20 ATP6V1B2 ZACN APOM GJC2 C20orf54 SLC25A6 GJA1 SLC39A3 RALBP1 UPF3B SLC29A3 GABRB1 ATP6V1E2 ABCC9 STEAP2 CEACAM1 TAP1 SLC6A1 PLEKHA8P1 RASA3 CHRNA9 TCOF1 CACNG3 SLC17A7 ATP6V1G2 LOXHD1 SLC7A7 RYR2 SLC25A4 SLC26A11 PKD2L2 PLSCR3 SLC31A2 CACNA1I RBP1 SLC12A9 IPO8 ACCN3 OSBPL8 SLC38A7 ATP7B EBP GPR89B SLC30A8 ATP5EP2 LRP6 KCNK13 TRPC6 ATP1B2 GOSR2 KCNC1 SFXN2 KCNA5 SLC19A1 SLC9B2 SLC35A2 SHROOM2 TRPC1 KCNT1 ATP5F1 SCN7A SV2C SLC12A8 SLC17A6 SLC24A6 AP3B2 ATP8A1 LOC100652748 SLC8A1 AQP3 GPIHBP1 SIDT1 TTYH2 SLC7A11 SLC11A1 PLSCR4 PSEN1 CPLX1 ATP13A1 SLC17A3 AP1G2 SLC4A10 TRPM1 SLC12A7 TRPC3 SLC45A3 KCNK4 BAX ATP13A2 CACNB2 COX6C SLC25A1 SLC36A3 ABCA7 ATP1A3 TRPM2 SLC25A24 ATP6V0A2 ST20 KCNK3 SLC12A3 NUTF2 GABRE AQP7P3 KCNMB2 VPS35 GJC1 MTMR6 AQP9 ANO5 FXYD5 SCN2A MFSD2A LCN15 ATP8A2 GJA10 ABCA11P KCNJ5 ATP6V1C2 ABCC1 SLC4A9

GO_BIOACTIVE_LIPID_RECEPTOR_ACTIVITY Combining with a bioactive lipid and transmitting the signal across the membrane by activating an associated G-protein; promotes the exchange of GDP for GTP on the alpha subunit of a heterotrimeric G-protein complex. A bioactive lipid is a lipid for which changes in lipid levels result in functional consequences in a variety of cellular processes. GPR174 S1PR2 S1PR3 LPAR2 S1PR5 GPR6 SPHK2 LPAR1 FFAR1 LPAR4 LPAR3 S1PR1 SPHK1 S1PR4

GO_PROTEIN_TYROSINE_PHOSPHATASE_ACTIVITY Catalysis of the reaction: protein tyrosine phosphate + H2O = protein tyrosine + phosphate. PTPRF SSH3 PTPN3 CDKN3 PTPN22 PGP MTMR6 PTPN23 DUSP9 SSH2 PTPRM PTPRN DUSP12 PTPN4 CDC14B PTPRG DUSP15 CDC25B PTPN18 DUSP26 PTPRR DUSP10 PTP4A2 DUSP5 PTPN9 DUSP28 DUSP14 PTPRB PTPN11 MTMR2 CDC14A PTP4A1 DUSP1 RNGTT PTPMT1 PTPN1 PTPRU MAP2K1 PTPRA PTPRQ PTPRO PTPRZ1 TPTE PTPRE MTM1 DUPD1 CDC14C DUSP16 DUSP21 SSH1 PTPN6 PTPN14 DUSP13 PTPRD DUSP11 DUSP3 PTPN21 MTMR1 ACP1 KIAA1274 PTEN PTPRH PTPN20B EYA1 MTMR7 DUSP22 PTPRS MTMR14 EYA4 PTP4A3 PTPRT DUSP19 DUSP4 PTPN7 PTPRJ PTPRN2 MTMR8 PTPLB TPTE2 UBASH3B MTMR3 EYA3 DUSP6 PTPN2 DUSP8 PTPN12 DUSP7 CDC25C PTPRK DNAJC6 MTMR4 EYA2 TIMM50 DUSP23 DUSP18 PTPN5 PTPDC1 CDC25A DUSP2 MDP1 PTPRC PTPN13

GO_PLATELET_DERIVED_GROWTH_FACTOR_BINDING Interacting selectively and non-covalently with platelet-derived growth factor. COL6A1 PDGFRB COL1A1 COL2A1 PDGFB COL5A1 PDGFA COL3A1 PDGFRA COL1A2 COL4A1

GO_INTRAMOLECULAR_OXIDOREDUCTASE_ACTIVITY_INTERCONVERTING_ALDOSES_AND_KETOSES Catalysis of an oxidation-reduction (redox) reaction in which the hydrogen donor and acceptor, which is an aldose or a ketose, are the same molecule, and no oxidized product appears. TPI1 EIF2B2 EIF2B1 MRI1 HYI EIF2B4 GPI GNPDA2 RPIA MPI GNPDA1

GO_TRANSMEMBRANE_RECEPTOR_PROTEIN_KINASE_ACTIVITY Combining with a signal and transmitting the signal from one side of the membrane to the other to initiate a change in cell activity by catalysis of the reaction: a protein + ATP = a phosphoprotein + ADP. EPHA10 EPHA5 FLT3 TEK TGFBR1 EFNA4 EPHB3 INSRR ACVR1C IGF1R ERBB2 EPHB4 EFNB3 INSR NRP2 TGFBR2 EPHA3 FGFR4 EFNA3 MERTK BMPR1B ACVRL1 NRP1 MST1R ACVR1B HFE2 LTK EPHA7 RYK FLT1 KIT ROR2 CSF1R FGFR3 EPHA2 LTBP4 BMPR2 PDGFRB ERBB4 EPHB6 FGFR1 EFEMP1 EPHA8 FGFRL1 EPHA4 AXL NTRK3 LTBP1 ACVR2A ACVR1 FGFR2 ALK EPHA1 ROS1 RET NTRK2 TYRO3 PDGFRA MET EPHB2 MUSK CRIM1 EPHA6 ACVR2B FLT4 IGF2R BMPR1A EPHB1 AMHR2 TRIM27 TIE1 KDR ROR1 DDR1 NTRK1 PDGFRL DDR2 NPTN TGFBR3 EGFR

GO_DNA_BINDING_BENDING The activity of binding selectively and non-covalently to and distorting the original structure of DNA, typically a straight helix, into a bend, or increasing the bend if the original structure was intrinsically bent due to its sequence. CEBPG TOP2A FOXL1 HMGA2 TFAM TOP2B HMGB2 FOXC1 CRIP1 HMGB1 TERF1 FOXD4 FOXI1 LEF1 TRERF1 HHEX HMGB3 FOXD1 GATA1

GO_NEUROPEPTIDE_RECEPTOR_BINDING Interacting selectively and non-covalently with a neuropeptide receptor. LOC100506013 PRLH HCRT QRFP NMB ASIP GNAO1 UCN3 GNAS MRAP2 UCN EDN1 EDN3 UCN2 NMU NPPA MRAP TAC1 GHRH ADCYAP1 POMC CRH CCKBR GAL KISS1 EDN2 APLN SHANK1

GO_PHOSPHOLIPID_TRANSPORTER_ACTIVITY Enables the directed movement of phospholipids into, out of or within a cell, or between cells. Phospholipids are a class of lipids containing phosphoric acid as a mono- or diester. ATP11A ABCA4 ATP9A MFSD2A ATP8A2 ATP8A1 TNFAIP8L3 PRELID1 ANO3 ANO7 ATP10D ABCB1 ATP8B1 ATP11C ANO4 ATP10B ATP8B4 APOA1 ANO6 ATP11B OSBPL5 ANO9 MTTP PITPNM1 ABCA1 PITPNA SLMO1 PLSCR2 PITPNM3 SLMO2 CETP PITPNC1 PLSCR1 ABCA7 ATP8B2 PLSCR5 OSBPL8 GLTPD1 ABCB4 ATP8B3 ATP9B ABCG1 ATP10A PRELID2 TRIAP1 PLSCR4 PLSCR3

GO_SOLUTE_PROTON_SYMPORTER_ACTIVITY Catalysis of the transfer of a solute or solutes from one side of a membrane to the other according to the reaction: solute(out) + H+(out) = solute(in) + H+(in). MFSD3 SLC36A1 SLC35A3 SLC2A9 SLC15A4 SLC36A3 SLC2A8 SLC35A4 SLC2A12 SLC2A13 SLC2A10 SLC32A1 SLC15A2 SLC35A1 SLC15A1 SLC45A2 SLC17A5 SLC45A4 SLC35A2 SLC35A5 SLC2A6 SLC15A3 SLC11A2 SLC33A1 SLC45A3 SLC36A2

GO_LRR_DOMAIN_BINDING Interacting selectively and non-covalently with a LRR domain (leucine rich repeats) of a protein. MKL1 NRL POLR2J CRX ATF4 ZXDC LRRFIP2 ERC1 DAPK3 CDC5L ROBO1 PMF1 AATF PAWR KRAS DDIT3 STK11

GO_LYSOPHOSPHOLIPASE_ACTIVITY Catalysis of the reaction: 2-lysophosphatidylcholine + H2O = glycerophosphocholine + a carboxylate. CLC MGLL PLB1 LGALS13 PLA2G15 PLA2G4E PNPLA7 ABHD12 PLA2G4C PLBD1 ABHD16A GDPD1 PNPLA6 PLA2G4A LYPLAL1 PLA2G4D PLA2G4B ASPG ENPP2 PLA2G4F GDPD3 LYPLA1 PNPLA8

GO_OXIDOREDUCTASE_ACTIVITY_ACTING_ON_A_HEME_GROUP_OF_DONORS Catalysis of an oxidation-reduction (redox) reaction in which a heme group acts as a hydrogen or electron donor and reduces a hydrogen or electron acceptor. COX5A COX6A2 C15orf48 COX8A COX7B COX7A1 CYB5A C1orf31 COX8C COX6B2 COX10 NDUFA4 COX7A2P2 COX6A1 COX7A2L COX4I1 NDUFA4L2 COX6B1 POR COX7A2 COX15 COX11 COX7C SURF1 COX7B2 COX5B COX4I2 COX6C

GO_COA_HYDROLASE_ACTIVITY Catalysis of the reaction: X-CoA + H2O = X + CoA; X may be any group. GNPAT BAAT ACOT6 ACOT12 LOC344967 ACOT2 NUDT19 ACOT1 ACSBG2 HIBCH PPT1 ACOT9 ACOT4 THEM5 ACOT8 ACOT7 NUDT7 ACOT13 THEM4 ACOT11

GO_MANGANESE_ION_BINDING Interacting selectively and non-covalently with manganese (Mn) ions. XPNPEP1 DCP2 ADCY10 IMPA1 B4GALT7 PCK1 ADCY2 XXYLT1 CCDC111 TDP2 LAP3 PAPOLA ARG1 ME1 GYLTL1B MPPE1 PPM1A GALNT2 PPM1N HMGCL PPM1B SLC24A2 PPM1M PIM1 ABL2 ATP13A2 GLUL GALNT3 WRN PPEF1 PEPD ABL1 XPNPEP3 PPEF2 CDIPT NEK4 FAM20C DYRK2 ENDOU LARGE MRE11A MTPAP NUDT16 NPEPL1 B4GALT1 SOD2 GALNT1 NUDT7

GO_LIGASE_ACTIVITY Catalysis of the joining of two substances, or two groups within a single molecule, with the concomitant hydrolysis of the diphosphate bond in ATP or a similar triphosphate. UBA5 CARS XRCC1 LRSAM1 IARS2 RNF38 MUL1 TTLL7 SUCLG2 TRIM25 RCHY1 UBR4 DZIP3 SLC27A5 TRIM21 MARCH6 TTC3 PIAS4 KARS TTLL2 RNF128 YARS2 RNF167 TRIML2 PAICS PCCA RTCD1 XRCC4 TRIM41 TRIM2 TRIM6 RBCK1 ACSS1 RNF4 UBA2 ACSM5 ATG7 CBLL1 TRIM36 RNF182 TRIM56 PARS2 CTPS2 TRIM50 LIPT2 RNF146 RNF144B MARCH9 RNF103 TRIM28 TTLL9 PELI1 RNF6 UBR2 ACSM1 FARSA ACSM4 MTHFD2 PJA2 RNF135 NEURL ATG3 TARSL2 RBBP6 AARSD1 RIMKLA MCCC1 BIRC7 RFWD3 TRIM62 CCNB1IP1 PELI2 PFAS TRAF7 ZSWIM2 FARSB ZNRF1 IARS MID2 TTLL13 RLIM MIB1 UHRF1 MKRN2 TRIM37 MARCH3 TRIM68 ZNF645 RNF170 MGRN1 CARNS1 RBX1 ACSL4 ACSBG1 CTPS RNF139 RNF20 RANBP2 TNFAIP3 SHPRH BIRC3 BIRC2 ACSL6 UBR3 LIG4 BTRC DTX4 TRIML1 EARS2 ACSS2 DARS IRF2BP1 BRCA1 CBL SH3RF1 MOCS3 RNF14 C19orf20 MARS MTHFD1 LGSN ARIH2 RNF181 TRIM24 CBLB MARCH2 PJA1 RFWD2 LIG3 DARS2 GART GMPS FPGS RNF111 MARCH7 NOSIP LARS QARS RNF185 TTLL4 RNF114 DTX3 LARS2 SUCLG1 TTLL11 MYCBP2 PIAS2 C10orf129 RNF144A UBA3 ASNS SLC27A1 RNF43 TRIM39 BRAP GCLM GSS TRIM23 PC AASDH ACSL5 TRIM9 HLTF TRIM17 NSMCE1 TRIM32 MKRN4P SIAH1 CNOT4 RARS2 ADSS VARS2 BIRC6 RCL1 TRIM71 RNF187 RNF115 RNF19A UNKL RNF130 KIAA1586 TRIM5 C22orf28 TRIM22 POLG2 RNF40 RNF220 GLUL SLC27A3 NSMCE2 TRIM4 TRIM27 UBE2CBP DTX3L DTX1 LNX1 TRIM58 SIAH2 MDM2 TTLL10 ATG10 UBR1 TTLL5 MARS2 FAAH TRAF3 MKRN3 RNF216 NAPRT1 ZFP91 ACSM3 RAD18 PPP1R39 RNF2 UBE2F PIAS1 RNF123 MSL2 TRIM31 MIB2 RNF13 UBOX5 RING1 YARS LTN1 TRIM11 SUCLA2 SLC27A2 CAD PPCS TRIM63 RNF5 PELI3 WARS2 PDZRN3 PARK2 DALRD3 MARCH10 ACSF2 TTLL6 SYVN1 LIAS GCLC RIMKLB AMFR KCMF1 LIG1 RNF152 MRPL39 ARIH1 UBA6 TTLL1 UBR7 UBE2I TTL RNF180 ACAT1 RNF138 WARS GATC STUB1 MARCH4 E4F1 FARS2 ACSM2A UBE2M MYLIP ATPBD4 RNF31 NFX1 NAE1 ACSBG2 TRAIP PRPF19 RNF168 TRAF2 MARCH8 NHLRC1 UCHL1 PARP2 AARS ZNRF2 ZNF451 AARS2 TTLL3 MEX3C RNF149 MTHFD2L MCCC2 PARP3 TRAF6 MARCH1 RNF169 RNF8 PET112 RNF19B RNF212 CBLC RNF25 ACSL1 RAG1 MKRN1 ASS1 HARS EGR2 FAAH2 EPRS ACSF3 UBE4A SLC27A6 PPIL2 TRIM69 FANCL UBA7 NEURL3 MARCH5 MTHFD1L QRSL1 PCCB HLCS AACS CARS2 RNF126 RFFL BARD1 FDXACB1 ACACB TRIM13 CPS1 TOPORS NARS2 PIAS3 TARS MTHFS RNF125 MID1 ASNSD1 ZNRF4 DTX2 RNF41 CHFR UFL1 XIAP ZNRF3 UBA1 ATPIF1 CBX4 ACSL3 TRIM38 TRIM8 LRRC47 RNF217 RC3H2 MARCH11 RARS HARS2 VARS ACACA TMEM129 RNF133 TRIM33 RNF34 UBE4B GARS TARS2 NARS ACSM2B TTLL8 PARP1 UHRF2 NADSYN1 SARS2 SARS SLC27A4 ADSSL1 ACSS3 NEURL1B SAE1 RNF213

GO_AMIDE_TRANSMEMBRANE_TRANSPORTER_ACTIVITY Enables directed movement of an amide, any compound containing one, two, or three acyl groups attached to a nitrogen atom, from one side of the membrane to the other. SLC38A3 SLC15A1 CDH17 ABCB9 AQP9 AQP10 AQP7P3 SLC38A1 SLC15A3 SLC14A1 SLC38A7 AQP7 SLC15A4 SLC15A2 AQP3 SLC14A2

GO_POTASSIUM_CHANNEL_REGULATOR_ACTIVITY Modulates the activity of a potassium channel. ARPP19 YWHAE CAV1 KCNE3 DPP10 DPP6 WNK1 KCNE1 KCNIP1 KCNS3 SGK2 KCNMB3 KCNV1 CHP AKAP9 KCNIP3 KCNMB1 KCNA5 KCNIP4 KCNS1 KCNK2 ABCC9 CAV3 AMIGO1 KCNAB3 DRD4 KCNAB2 NEDD4L ENSA KCNMB2 KCNAB1 SGK1 KCNMB4 SUMO1 RASA1 SGK3 KCNE1L PRKCZ PIAS3 C8orf44-SGK3 LRRC26 DLG1 ADRB2 KCNIP2 KCNE2

GO_TRANSCRIPTION_COFACTOR_BINDING Interacting selectively and non-covalently with a transcription cofactor, any protein involved in regulation of transcription via protein-protein interactions with transcription factors and other transcription regulatory proteins. Cofactors do not bind DNA directly, but rather mediate protein-protein interactions between regulatory transcription factors and the basal transcription machinery. KLF4 NR1D1 PPARA CDK9 PASD1 FOXO3 NR4A3 NFATC1 HDGF PHF12 CCNT2 SIX3 TFAM ZBTB49 CDC5L CHD6 RORA FAM89B CNOT2 MAFK NFE2L2 FOXO1 TERT

GO_TRANSFERASE_ACTIVITY_TRANSFERRING_PENTOSYL_GROUPS Catalysis of the transfer of a pentosyl group from one compound (donor) to another (acceptor). PARP9 MTAP GYLTL1B POGLUT1 SIRT1 TNKS2 ART3 XYLT1 SIRT5 TIPARP XYLT2 PARP16 PNP APRT PARP14 QTRTD1 PARP8 PARP2 C6orf108 HPRT1 PARP6 QPRT NAPRT1 UMPS ART1 TNKS UPP1 TYMP XXYLT1 SIRT4 PARP3 LRRC9 SIRT6 LARGE SIRT3 SIRT2 PARP12 ZC3HAV1 PDC PPAT PDCL3 PARP11 PARP1 ART5 TXNDC9 QTRT1 NAMPT PARP15 GXYLT1 PDCL2 GXYLT2 ART4 UPP2 PARP4 PRTFDC1 PDCL PARP10

GO_IRON_ION_BINDING Interacting selectively and non-covalently with iron (Fe) ions. PTGIS CYP4F12 CYP26A1 LCN2 CYP2A7 CYP1A2 ALOX15B TBXAS1 ALOX12B OGFOD2 ETHE1 HBG2 ALOX5 HBA2 HBA1 AOX1 CYP4F22 C14orf169 AGMO TET3 CYP51A1 CYP3A4 HBZ SNCA ALKBH2 HIF1AN LEPREL1 CYP2A13 CYP2U1 ALKBH1 PLOD2 SCD5 CYP19A1 FTL ALOX15 CYP2C19 CYP4Z1 ALKBH8 FBXL5 CYP27B1 C17orf101 TF HAAO NOS1 FDX1 CYP26B1 CDO1 FECH CALR ISCU CYP2R1 LEPRE1 XDH CYP2W1 CYP21A2 FA2H HBB MINA TET1 CYP2G1P HBD CYP2C9 PPEF2 CYP4Z2P ADI1 CYP3A43 CYP2E1 CYP8B1 CYP7B1 BBOX1 MSMO1 CYP4F3 FTH1 CYP4F2 CYP4F8 CYP1B1 CYP2J2 FTH1P19 CYP4X1 OGFOD1 ACP5 CYP2B6 ALKBH3 LEPREL2 CYP2F1 CYP4V2 TPH2 SC5DL ABCE1 HBE1 CYP2D6 ALOXE3 PLOD1 CYP4F11 CYP27A1 NFU1 ALOX12 PAH HBQ1 ALKBH6 FTMT ISCA1 FTO ISCA2 CYP11A1 CYP20A1 P4HA2 CYP2C18 HEPH PHF2 PPEF1 CYP24A1 ACO2 TET2 TYW5 KIAA1456 LTF JMJD6 CYP11B2 EGLN3 P4HTM CYGB JHDM1D CYP3A7 HBG1 CYP2S1 P4HA1 EGLN2 CYP2C8 PLOD3 TMLHE NOS3 HBM EGLN1 CYP46A1 CYP1A1 CH25H CYP4A11 DNAJC24 FXN TPH1 CYP17A1 CYP4A22 MIOX CYP2D7P1 CYP11B1 CYP2A6 C5orf4 CYP4B1 FTHL17 TH CYP39A1 CYP3A5 KDM3A CYP26C1 PHF8 P4HA3 SCD CYP27C1 IZUMO3 MFI2 CYP7A1

GO_CARBOXYPEPTIDASE_ACTIVITY Catalysis of the hydrolysis of the terminal or penultimate peptide bond at the C-terminal end of a peptide or polypeptide. CPA5 AGBL5 PRSS16 AGTPBP1 NAALAD2 CTSL2 AGBL2 BLMH PRCP AGBL4 ACE2 CPN1 CPM CPE CTSA FAM63A AGBL3 CPB2 CNDP2 CPD PEPD CPXM2 DPP7 CPA1 NAALADL1 AEBP1 CPA4 CPZ CPA3 CPA6 CPB1 FOLH1B CPA2 FAM63B CPXM1 CNDP1 FOLH1 ACE CPVL PGCP CPO FAM188A AGBL1 SCPEP1

GO_CHANNEL_REGULATOR_ACTIVITY Modulates the activity of a channel. A channel catalyzes energy-independent facilitated diffusion, mediated by passage of a solute through a transmembrane aqueous pore or channel. KCNE2 CACNG5 LRRC26 FXYD5 BSND RASA1 KCNE1L CACNG2 NPY2R SUMO1 KCNMB4 KCNMB2 ENSA AMIGO1 PRKG1 CACNA2D4 SGK2 NRXN1 CABP4 DPP6 DPP10 KCNE3 CAV1 SLC9A3R1 ARPP19 GPLD1 STX8 STIM1 TRPV1 FXYD3 GRM2 PIAS3 FXYD2 CRISP1 LOC400986 FKBP1B KCNAB1 SGK1 NOS1 CACNG4 PHPT1 VTI1B CAV3 KCNA5 SCN4B WNK3 CACNA2D3 KCNE1 CAMK2D KCNIP1 PLN RANGRF YWHAE ADRB2 WNK4 DLG1 PKP2 SCN3B FGF13 STX1A ITPR1 CACNG3 SCN1B PTPN3 SGK3 KCNAB3 SRI KCNAB2 GRM7 NRXN2 KCNS1 KCNK2 ABCC9 SCLT1 CACNG7 PDZD3 STX7 CHP AKAP9 KCNIP3 KCNV1 PRSS8 NPY PRKCB KCNMB3 LYNX1 TMEM110 SNTA1 GNB2 CNIH3 KCNIP2 BCL2 C8orf44-SGK3 CACNG8 TNNI3 PRKCZ FKBP1A HPCAL4 GRM3 GNB2L1 NEDD4L NRXN3 CCDC109B TSPAN13 NEDD4 DRD4 ATP2B4 PACSIN3 CHRNA7 KCNIP4 FXYD1 STIM2 VAMP8 SNF8 YWHAH FXYD4 KCNMB1 PCSK9 CFTR FGF12 PRSS41 KCNS3 WNK1 GPD1L TMPRSS3 RSC1A1 SCN2B AMBP SLC30A1

GO_SEQUENCE_SPECIFIC_DNA_BINDING Interacting selectively and non-covalently with DNA of a specific nucleotide composition, e.g. GC-rich DNA binding, or with a specific sequence motif or type of DNA e.g. promotor binding or rDNA binding. ZNF708 JDP2 TBX2 ZNF711 SOX4 RORC NFX1 ELK3 ZNF502 NFATC1 HELT SMG1 FOXR1 GABPA GSC2 SPI1 SKIL HIF1A E4F1 ZKSCAN3 TAF1C TSN NOTCH1 DUX4 IRX5 ETV7 ZMYND8 AIRE SOHLH2 HNRNPA2B1 MYC ZNF736 HOXC12 FOXD4L6 PARK2 HOXA5 NACC2 MYBBP1A ZNF691 USF2 NEUROG3 SCAND2 SATB1 PHF21B GRHL2 ZNF56 ARHGAP35 SMAD5 DLX3 USF1 XRN2 CTCF BATF3 NFKB1 MTOR SREBF1 MTA2 CEBPZ ARNTL DMRTC1B ZNF492 NR2E3 KCNIP3 SOX3 ZNF263 PTF1A OBFC2B TBX18 IRF7 TTF1 NFE2 SMAD6 DMRTC1 HOXD9 GCM2 KLF12 NR0B1 ZNF561 MYOCD MBD3L5 HLX GOLGB1 C2orf3 RARB FOXF1 ZNF682 FOXA3 MTF1 ZBED4 ZNF548 DDN CEBPE HSFY2 SOX6 PITX2 NFKB2 CDX4 POU3F3 VAX2 IRF4 ACTL6A BARHL2 SUV39H1 SMAD4 DNMT3A MYPOP MYBL2 POU5F1 FOXO3 MEOX2 BAHD1 PAX6 SIRT1 MESP1 TP73 POU2F2 ZNF679 TAF1L ZNF138 HOXA4 TBR1 TCF3 MNX1 MEIS2 NLRP3 ZNF746 GRHL1 HNF4G EGR2 CC2D1A ZNF737 SALL3 ZNF419 BAHCC1 CRY1 HHEX NEUROD1 PAX3 RXRB ZFAT ZNF277 ZNF516 TBX15 INSM1 CUX1 KLF16 TLX2 RUNX3 NANOGP8 AEBP2 DMRTA2 HOXC13 RBPJL TLX3 ZNF205 ARGFX ZSCAN16 PAX2 RBBP4 CGGBP1 MYB TFAP2C THAP9 SIX3 ZNF500 ZNF772 PKNOX2 EMX1 DMRT2 ZNF730 PDX1 CRIP1 PAX8 KLF13 NR2E1 CAMTA2 HMGA1 SMYD3 HOXB2 CALCOCO1 NFE2L3 TSNAX KLF5 PPARD TFAP2E SOX7 KLF10 ALX4 RELB HOXA10 TRIM28 PROX1 MECP2 NFIL3 MITF ATF6 PBX4 GATAD2A ZNF664 TCF12 BCL6B GLI1 ZBTB38 IRX2 SOX13 ATOH1 UBP1 HIVEP2 EP300 TFAM GMEB2 ZNF430 RUNX2 HOXB1 ZNF675 HIVEP1 ZFX DNMT3B TERT NRL JUND LHX1 KLF1 FOXP4 ARNT2 MEF2B FOXG1 HOXA11 NR1H2 CTC1 PRRX2 ZNF735 EBF2 HOXD12 MAFA ATF7 DLX1 NFAT5 LHX4 ZNF8 NKX2-1 ZSCAN21 TNRC18 MTPN KLF4 MXD1 MEF2C BAZ2A TCF7L2 TAL2 ELK4 TERF2IP RELA GATA2 PKNOX1 ARID5B ZSCAN30 MZF1 DMRTC2 ISX MNT ZNF165 DBX1 JMJD1C RBPJ ACD FEZF1 BHLHA15 MTA1 PAX5 PLAGL1 ZIC3 CREBZF ZSCAN4 SOX12 TBX3 MYF6 ASCL1 ZNF92 LHX5 HNF1B FOSL2 FOXO4 CLOCK MBD3 HDAC4 NR3C2 RFX8 RXRG NR4A1 NFIB LEF1 RAD50 TBX5 SHOX ZNF584 MSH2 CHD7 ZNF335 BCL6 ZNF519 ESRRB NKX2-2 TAF2 KDM1A ARX HMX1 MCM5 ZNF238 POU2F3 BHLHE41 RXRA ZNF281 YY1 THAP1 TAF1 ZGLP1 FOXD4L1 SRF PRMT5 ZNF195 SOX11 MBD3L2 DMTF1 ESRRA EMX2 EN1 ZNF876P NANOGP1 DMRT3 EVX2 SHOX2 SALL4 ELK1 IRF2 NFXL1 FOXE1 CSRNP2 PAX4 THRAP3 ZKSCAN4 ZNF479 LRWD1 CREB3L2 XRCC6 GSX2 NR2F1 ZBTB49 POU3F1 ESRRG HOXB13 ETV1 ZBED6 MEOX1 IRF3 CHD4 FOXS1 H2AFY2 ASCL3 MBD4 HSFX1 C1orf51 REST HIVEP3 PIH1D1 ZNF200 HOXB7 YBX1 MSC IRX6 WBP2 NKX2-8 ZNF676 POU3F4 ESX1 ORC6 ZNF551 NKX2-4 ZNF217 RHOXF2B FOSL1 ELF3 HMGB2 GLIS2 LDB1 UPF2 ZNF35 PURB SKOR2 RFX4 RAG1 FOXE3 SPIB CDC5L CHTOP PPARA FOSB IKZF5 NFYA GSX1 FOXK2 ALX1 ETV3 MLH3 NFE2L1 ZNF714 TFAP2A IRX4 POU4F1 MAX MSX1 DMRTB1 ETV2 MBD3L1 MSX2 MYBL1 GBX1 PRRX1 ZNF750 ELF5 RUVBL2 H2AFZ GBX2 NR3C1 UHRF1 RPTOR KRBOX1 ELF1 SOX9 PATZ1 HOXD10 ZBTB33 MAFF BATF2 ORC4 SCXB SKOR1 VENTX CENPC1 CC2D1B FOXN4 ZNF211 TFCP2L1 ZNF831 HNF4A ETV3L IZUMO2 ZNF154 MLX LHX6 TAF1B HEY1 HOXA7 RORA PHOX2B TSPYL2 LYL1 SNAI1 CREBBP ZBTB4 RHOXF1 SNAI3 HNF1A ERH NFIC LMO2 HOXC4 TGIF1 MAFB ZNF562 FOXL2 CSRNP3 NPAS4 ISL2 NEUROD6 CRX DBX2 FOXD4L4 HMX2 CAMTA1 ZNF486 JUNB FOXI2 ZBTB7B NR5A1 HOXB9 VRTN HOXA1 SMG7 VSX1 ZNF215 RAX STAT5B CEBPG LOC647589 NFYC ZNF724P CREB1 PLAGL2 ZNF257 DHX33 MAFK SLTM ATF1 NANOG BACH1 MTA3 PPARGC1A DMRTA1 FOXJ2 MAEL PSPC1 ZNF773 RERE XBP1 FOXD2 NOBOX NFYB KDM3B ZNF395 EGR1 BEND3 ZNF431 FOXD1 SUZ12 ZNF100 MCM10 ZNF543 ORC2 APEX1 FIGLA NFE2L2 PRDM5 TFAP2B MED1 ESR1 PBX1 GATAD2B ZNF586 ZNF117 PPARG JUN KLF17 RFX2 ONECUT2 RFX5 ZNF552 EOMES TBX1 NKX6-2 RARG KDM3A MCM2 NFATC2 ONECUT3 BCL11B DPRX HMGA2 CEBPA WHSC1 HEYL HOXC5 REL NR2F6 TAL1 THRB BCL2 FOXN3 BPTF SOX2 HOXC9 ZNF66P LMX1A SOX10 MBD3L4 FOXN2 SUV39H2 HOXC11 HNRNPAB MKL1 NME1 FOXO1 NR2C2 ATF6B SMARCC1 HOXB4 NKX1-1 NR5A2 MKX ZSCAN23 USP3 NFATC3 HNRNPC DUX5 PRDM1 ZNF713 EHF ZNF396 DNMT1 ZFHX2 ZNF350 SNAPC1 ZNF704 CENPB PBX2 ZNF570 RORB RUNX1 ZNF253 T STOX1 ATF4 SIX4 DMRT1 TOP1 RFX1 TRPS1 RFX7 FOXD4L3 STAT1 SOX18 GMEB1 ZNF79 TFAP4 ZNF98 ZNF449 TCF7 FOXL1 NKX2-3 FOS PAX9 STAT3 ASCL2 GATA4 ZMYND11 ZFPM1 ESR2 ZNF90 UBTF BACH2 FOXD3 DMBX1 BTBD8 ZBTB16 ZNF792 AKNA LHX9 CDK9 ZIC1 EDF1 ZFHX4 EIF2C1 HOXC8 GLIS1 ZNF85 GZF1 TXK DLX2 GATA6 HOXA9 ZNF837 BARHL1 ZFP161 PRDM15 NRIP1 KDM6B FUBP3 GLI2 HOXA6 LHX8 ZNF148 HOXC10 WT1 HMBOX1 CEBPB SMARCD2 ZNF256 CREB3L1 MEIS3 CRY2 CREM NR4A2 LBX1 SAFB VDR SOHLH1 ZSCAN12 POT1 UPF1 FOXR2 CREBL2 XRCC5 EPAS1 OBFC1 MAFG OSR2 ZNF536 YAP1 ZNF219 HSPD1 ZIK1 ATF3 ZNF805 HOXD1 SMARCA4 FOXO6 POU4F3 PROP1 ZSCAN20 ZNF498 SOX17 BARX1 ARNT ZNF716 ZNF418 E2F7 TP53 ZNF295 TCF7L1 ZNF718 LHX3 TERF2 FOXP3 SMARCC2 E2F1 KAT2B CREB3 MLL HMX3 HOXB6 ALS2CR8 BARX2 HAND1 ZNF232 RAX2 WDR77 NFATC4 ZNF583 ZFY GSC SPDEF ZC3H8 RRN3 HEY2 PRMT1 HR TCF4 HSF5 LHX2 POU5F1B SALL2 FOXP1 ZNF24 ZNF460 HOXA2 NR1H3 SMAD1 PURA CSDA THRA MYOG ZNF660 RHOXF2 SOX1 MAF SREBF2 HNRNPK SETX HOXD3 IKZF4 POU3F2 CREB3L3 ACTB NKX2-5 CDX1 DLX5 TRIM24 ETV4 NKX3-1 NFIA HSF2 SIX2 ALX3 CDC45 GRHL3 PCGF6 EZH2 FOXI3 KIF2C AEBP1 ZNF193 CUX2 CDX2 HOXB5 ZNF549 FOXA1 HES1 MUC1 HSF4 ZNF114 NRF1 ZNF187 GTF2E1 ASCL4 HESX1 ACTN4 SOX30 HDAC2 NR1H4 E2F4 ZNF480 FOXC2 ETS1 CTCFL NKX1-2 SPIC FOXC1 SIX5 STAT6 RFX6 POU6F1 NR2F2 FOXQ1 TINF2 CREB3L4 OTX1 SALL1 UNCX SNAPC4 TFE3 PLAG1 CXXC1 RARA ARID3C NLRC5 RFC1 TFAP2D ERG HOXD11 PER2 ASCL5 ERF ETV5 IKZF3 NKX2-6 CSRNP1 NKX6-3 FOXB1 OVOL1 ZNF532 HAND2 FOXD4 ZGPAT ISL1 SFPQ MEIS1 TEN1 H3F3A PHOX2A DBP PITX1 LMO4 MIXL1 ZNF213 CEBPD EVX1 LMX1B PHF12 FOXJ1 POU2F1 HSF1 GATA3 KDM2B NR1D1 HOXA3 C5orf41 ZNF323 NSD1 SATB2 NHLH1 IRF1 HOXD13 ZNF331 SIN3A BCL11A ZNF626 ZNF680 PIF1 FOXA2 NKX6-1 ZBTB7A HOXD8 MEF2A ZNF192 NR1D2 HOXB3 H3F3B TP63 TBX19 FOXN1 MBD1 SAFB2 PBX3 MBD3L3 ZNF75D LONP1 NR2C1 MEF2D ZKSCAN1 GATA5 SMARCE1 HDAC1 PHF21A ZNF304 ZFHX3 NR4A3 IFI16 CHD2 ZNF658 FERD3L DLX4 SNAI2 ZNF695 ATF2 SOX14 POU1F1 ORC3 HOXB8 HINFP ZNF264 FLI1 E2F8 GTF2H5 NDN ORC5 HOXC6 HOXD4 ZNF394 DPF2 ARID3A PRDM16 SMG6 ZW10 SMARCB1 NKX3-2 ZNF397 NCOA2 SMG5 TBX20 PITX3 TBP NCL KDM6A FOXJ3 FOXI1 SOX8 HIC1 ZNF732 VAX1 ZNF793 NEUROD2 NR1I2 ZNF639 CENPA VSX2 PER1 DLX6 DRGX FOXK1 DUX1 SKI FOXF2 FOXP2 JARID2 MLXIPL ATF5 FOXD4L5 NR6A1 LEUTX OTP ZNF506 GCM1 ETS2 HOXA13 AR ZBED1 MAF1 HNRNPA1 EN2 MYF5 RFX3 ETV6 KLF2 EAF2 SP5 FOXB2 ZNF274 RREB1 OTX2 ZNF727 ZSCAN2 FOXM1 GCFC1 LOC100293516 GLI3 SP3 HSFY1 IRX3 HSFX2 C7orf29 POU4F2 HES6 CHCHD2 MED12 DDIT3 TFCP2 ELF4 HLTF TEF ARID3B MBD2 NHLH2 SMAD2 FEZF2 FOXH1 ELF2 SMAD3 TERF1 FEV ZSCAN10 SP1 NR1I3 GATAD1 UPF3A ZNF141 MYOD1 PCBP1 HLF CREB5 STAT4 MAZ ZBTB11 NONO PGR HNRNPD CXXC5 ZNF273 BSX ZSCAN22 IRX1 H2AFY TLX1 SIX1 PGBD1 ZNF75CP GATA1 BATF PAX7

GO_METHYLATED_HISTONE_BINDING Interacting selectively and non-covalently with a histone protein in which a residue has been modified by methylation. Histones are any of a group of water-soluble proteins found in association with the DNA of plant and animal chromosomes. LRWD1 MSH6 RRP8 CHD8 L3MBTL1 ING3 JHDM1D CBX7 MSL3 CBX2 KDM4A CHD1 WDR5 NCAPG2 SPIN1 CDYL2 ING4 KAT8 CHD5 PHF19 UHRF1 ING2 TDRD3 TRIM24 CDYL MPHOSPH8 MTF2 ING1 CBX4 FAM156A ATRX ING5 CBX8 SUZ12 FAM156B RBBP5 PWP1 CCDC101 FMR1 ZMYND11 GLYR1 DPPA3 TP53BP1 CDY1B CDY1 CBX5 PHF13 PHF2 LOXL2 PHF1 RAG2 PHF8 L3MBTL2 NCAPD3

GO_PHOSPHORIC_DIESTER_HYDROLASE_ACTIVITY Catalysis of the hydrolysis of a phosphodiester to give a phosphomonoester and a free hydroxyl group. EDNRA SMPD2 PLCZ1 GDPD1 PLD6 FAN1 PDE6D GPLD1 SMPDL3B BDKRB2 PDE4B FAM83B PDE6B PLCL1 PLCD3 PLCXD1 PDE7A ENPP7 CHRM3 ENPP3 CCR5 ENPP6 PDE5A CHRM1 PLCH2 PDE6G CHRM5 GDE1 PDE9A ENPP1 PDIA3 PLD2 GDPD4 CCL5 GDPD2 TDP1 PDE4A PDE6C CNP PDE3A TBC1D10B GNB1 PLD3 ENPP2 MPPE1 CCKBR PLCD4 PLCXD3 SMPD3 PLCB3 CASR TDP2 PLCE1 SMPD1 PDE6A PDE8A PLCG2 NOTUM PDE10A APEX1 PDE1A PLCB1 PLCB2 PLD1 PDE3B PLCXD2 GPCPD1 PDE4D CCR1 ADORA1 PDE2A GDPD5 PLD4 PLCB4 PLCD1 PDE7B PLCG1 GDPD3 PDE1B HMOX1 F2RL2 PLCL2 PDE1C NAPEPLD PDE8B SMPDL3A PDE4C PDE6H SMPD4 PDE11A PLCH1

GO_MOTOR_ACTIVITY Catalysis of the generation of force resulting either in movement along a microfilament or microtubule, or in torque resulting in membrane scission, coupled to the hydrolysis of a nucleoside triphosphate. MYH3 MYH7B DYNC1I2 KIF3B KIF3C KLC4 KIF6 SMC3 DNAH11 DYNC2H1 CCDC102A KIF17 CGN MYH15 MYO5B MYH6 MYL6 MYH4 KIF2B KIF4A MYO3A MYO1H TCTE3 DYNC1I1 KIF21B KIF2C DCTN2 DYNLL2 DNAI2 DYNC1LI2 DCTN1 KIF7 MYO1B MYO7A MYH8 DNAL4 KIF14 MYH16 MYO1D MYO6 KIF18A DNAH7 KIF19 KIF15 DNAH6 KLC2 KIF5A DYNLL1 MYH9 DNAH1 MYO9A DYNLRB2 KIF27 KIF20B DNHD1 MYO1F MYO16 DNAH10 KLC1 KIF13A MYH14 DNAH3 BBS4 KIF23 MYL6B MYO7B KIF1A MYO1G MYO15A KIF1C MYO18B DNAH2 KLC3 MYH11 KIF13B MYH2 KIF2A KIF16B KIF3A MYO5C DNAH17 STARD9 KIF5B DYNC1H1 KIF20A KIF24 LOC100130097 CENPE DNAH14 MYH7 KIF22 APPBP2 DYNC1LI1 KIF26A DNAH8 MYL3 DNAH9 MYO9B KIF21A MYO3B KIF5C KIF4B KIF25 DYNLRB1 MYO1A CGNL1 KIFC2 MYO19 KIF9 MYH1 KIF26B MYO1E MYO1C DNAI1 DYNLT3 MYO5A KIFC1 DYNLT1 MYH10 PIN1 DNAH12 MYH13 KIF12 KIF11 DNAH5 MYO10 DYNC2LI1 KIFC3 DNALI1 KIF1B KIF18B

GO_PROTEIN_C_TERMINUS_BINDING Interacting selectively and non-covalently with a protein C-terminus, the end of any peptide chain at which the 1-carboxy function of a constituent amino acid is not attached in peptide linkage to another amino-acid residue. RAD51 YWHAB KCNK3 ERBB2 CDC20 CTGF PRRC2C TBL1X ZBTB16 CEP120 MLLT4 YWHAQ OPRL1 HSPB7 MPDZ VGLL1 ATXN1 BAIAP2 LIG4 BCAM ECM1 ERCC1 SDCBP2 CTBP1 SREBF2 ERCC6 SP1 IFT52 CEP250 PPP1CC EML2 CIITA LCK TOPBP1 MRE11A CIB1 CAV3 MED12 HRAS NEIL1 EIF2C2 SIAH1 TCF4 BAIAP3 VPS4A GRIP1 PIAS3 HIC2 PABPC1 TAF13 TAX1BP3 PPP2CB ERCC2 SIRT1 NEFL CEP135 DAPK3 TOP2A SAE1 XRCC6 PEX16 ATP1B1 NIPBL DLG3 PICK1 ERCC4 SRC BCL10 ATP2A2 ATF4 IFT46 KPNA3 DBNL PIAS1 SIPA1 PFKM EP300 PROP1 MAGI1 PIAS4 PLEKHB1 PEX1 DLG4 EFHC1 MIF4GD RABAC1 SDC1 NCF2 VIM DLG1 TERF2 YEATS4 CENPF CTNNB1 XRCC5 PHB TOP2B SASH1 FOXN3 CSK TNNI3K MID1IP1 FBLN5 PCGF1 MAPRE1 ITGB3BP YAP1 MDC1 SYNJ2BP MKI67 FBLN1 ID1 PRDX3 MAP2K1 ATXN2 OPTN XRCC4 ERCC3 PDZD3 SCLT1 RBFOX1 PEX26 DST VPS4B MYO1C CNGA3 SNX17 VTA1 DNM1 SNTG1 HPCAL4 NCL PEX12 DAB2 SDCBP PHB2 JAK2 VGLL2 CITED1 CALCOCO1 PEX6 KCNJ11 HSPG2 OPRM1 PXK ABL1 PPP2CA CD2AP KSR1 VPS36 AP2A1 SHANK3 COIL CACNA1B BRCA2 FIGN TJP1 NPAT HOMER3 PRKAA1 SLC9A3R2 CORO1A MSH2 SYT1 USP7 CDK7 PPP1R9A GOPC HESX1 STIP1 SHANK1 TRIM3 SNF8 RAB3A

GO_BICARBONATE_TRANSMEMBRANE_TRANSPORTER_ACTIVITY Enables the transfer of bicarbonate from one side of a membrane to the other. Bicarbonate is the hydrogencarbonate ion, HCO3-. SLC26A9 SLC4A9 SLC26A11 CFTR SLC26A7 SLC4A1 SLC4A7 SLC26A6 SLC4A11 SLC26A8 BEST1 SLC4A5 SLC26A10 SLC26A4 SLC4A4 SLC26A2 SLC26A3 SLC26A5 SLC26A1

GO_HYDROLASE_ACTIVITY_HYDROLYZING_N_GLYCOSYL_COMPOUNDS Catalysis of the hydrolysis of any N-glycosyl bond. ART5 NEIL1 OGG1 APEX1 PCNA UNG BST1 SMUG1 NEIL2 NTHL1 TDG CD38 MUTYH ADPRH NEIL3 CCNO MBD4 ADPRHL1 C6orf108 MPG RPS3 MAN2A2 MAN2A1

GO_DOUBLE_STRANDED_DNA_BINDING Interacting selectively and non-covalently with double-stranded DNA. REST KDM2B PIH1D1 NR1D1 YBX1 NSD1 LMX1B PHF12 FOXJ1 HSF1 C1orf51 FBXO18 GATA3 BCL11A ORC6 ESX1 ZNF551 ZNF626 MSH3 ZNF680 MSC NHLH1 WBP2 NKX2-8 IRF1 ZNF676 POU3F4 HOXD13 ZNF331 SIN3A ESRRG ETV1 H3F3A ZBED6 MEOX1 IRF3 PHOX2A XRCC6 SFPQ MEIS1 LMO4 MIXL1 TARDBP CEBPD MBD4 CHD4 H2AFY2 DBP ASCL3 PITX1 ZFHX3 PPARA FOSB NR4A3 IFI16 ZNF658 CHD2 NR2C1 MEF2D GATA5 SMARCE1 HDAC1 PHF21A MSH5 ZNF304 ZNF695 MSH6 NFE2L1 ATF2 ZNF714 TFAP2A MAX POU4F1 POU1F1 ORC3 MSX1 NFYA IKZF5 GSX1 FOXK2 ETV3 ALX1 FERD3L DLX4 MLH3 NR1D2 FOSL1 ELF3 HOXB3 H3F3B TP63 HMGB2 TBX19 GLIS2 FOXA2 NKX6-1 NTHL1 ZNF217 ZBTB7A HOXD8 MEF2A MBD1 LDB1 PURB SKOR2 SAFB2 PBX3 RFX4 FOXC1 ARX SIX5 HMX1 MCM5 STAT6 CHD7 ZNF335 E2F4 ZNF480 BCL6 RBMS1 ESRRB ZNF519 CTCFL ZNF281 YY1 CREB3L4 RAD51B HMGB3 RFX6 POU2F3 MSH4 BHLHE41 POU6F1 RXRA RXRG ZNF638 APITD1 ZNF549 RFX8 MUC1 ZNF114 ZNF584 ACTN4 MSH2 HDAC2 NR1H4 NRF1 NFIB LEF1 RAD50 ASCL4 TBX5 ELK1 IRF2 NFXL1 ASCL5 ETV5 IKZF3 ZGPAT ZNF479 CREB3L2 ISL1 PAX4 OVOL1 THRAP3 HAND2 TFE3 PLAG1 ZNF195 CXXC1 RARA SOX11 ZGLP1 OTX1 APTX PNKP SALL1 PRMT5 SRF CTNNB1 AFF3 TFAP2D EN1 ZNF876P PER2 DMRT3 ARID3C NLRC5 ESRRA RFC1 ZNF724P CREB1 MED12 ZNF257 DHX33 PCNA DDIT3 C7orf29 RAX STAT5B CEBPG POU4F2 NFYC HES6 TEF ARID3B MBD2 FOXJ2 DMRTA1 NHLH2 MAFK ELF4 SLTM ATF1 BACH1 HLTF ZBTB7B OTX2 MTERFD1 NR5A1 ZNF727 ZSCAN2 ZNF486 JUNB HES2 RREB1 GLI3 SP3 GCFC1 LOC100293516 MCM10 BSX ZNF273 ZNF543 APEX1 ORC2 STAT4 MAZ ZBTB11 NONO GTF2H4 PGR HNRNPD TFAP2B MED1 ESR1 BATF PBX1 GATA1 GATAD2B ZNF586 ZNF117 JUN PPARG FIGLA TLX1 H2AFY SIX1 NFE2L2 PRDM5 NOBOX SMAD3 TERF1 NUCKS1 KDM3B SP1 ZNF395 SMAD2 FEZF2 PSPC1 ZNF773 XBP1 MRE11A FOXD1 SUZ12 HLF ZNF100 EGR1 BEND3 NR1I3 DDX58 ZNF431 ZNF141 MYOD1 TBX20 PITX3 HMGB1 TBP KDM6A BATF2 ORC4 SCXB RPTOR UHRF1 NCOA2 ELF1 FEN1 SOX9 PATZ1 FOXN4 ZNF211 VAX1 ZNF732 STRA13 ZNF793 NEUROD2 FOXI1 SKOR1 PMS1 CENPC1 CC2D1B SOX8 FLI1 PRKDC ETV2 DMRTB1 MYBL1 MSX2 ZNF264 HINFP DPF2 ARID3A RUVBL2 H2AFZ SMARCB1 ZW10 NKX3-2 GBX2 NR3C1 ZNF750 ERCC5 E2F8 GTF2H5 ORC5 ELF5 NDN NFIC ETS2 GCM1 LMO2 TGIF1 ZNF562 MAFB AR FOXL2 NR6A1 SNAI1 ZBTB4 CREBBP HNF1A SNAI3 ZNF506 MYF5 ETV6 CRX RFX3 EAF2 SP5 ZBED1 NPAS4 MAF1 NEUROD6 PER1 TAF1B FOXK1 HEY1 MLH1 NR1I2 HNF4A ZNF639 ZNF154 CENPA MLX FOXP2 HOXA7 RORA PHOX2B JARID2 ATF5 TSPYL2 MLXIPL LYL1 SKI FOXF2 RFX1 MYPOP MYBL2 POU5F1 TRPS1 ACTL6A IRF4 VAX2 BARHL2 DMRT1 AIM2 SUV39H1 SMAD4 TOP1 MESP1 SIRT1 SOX18 STAT1 GMEB1 ZNF79 TP73 TFAP4 POU2F2 ZNF98 ZNF679 MEOX2 RFX7 BAHD1 PAX6 ZNF682 FOXF1 ZNF350 CENPB ZNF704 MTF1 ZBED4 ZNF561 MYOCD EGFR C2orf3 RARB STOX1 PITX2 ATF4 CDX4 NFKB2 SIX4 ZNF548 ZNF570 DDN MTERFD2 ZNF253 RUNX1 CEBPE T ZBTB16 BTBD8 ZFAT ZNF792 AKNA CDK9 ZIC1 ZNF90 UBTF RXRB MND1 FOXD3 BACH2 DMBX1 EIF2C1 TBX15 INSM1 ZNF85 GLIS1 TXK CUX1 GZF1 KLF16 DLX2 ZNF277 RAD51C KDM4A ZNF516 MEIS2 FOS ZNF746 PAX9 STAT3 GRHL1 ZNF138 TBR1 TCF3 BAHCC1 CRY1 HHEX NEUROD1 EGR2 ZNF737 CC2D1A ASCL2 ZNF419 GATA4 ZMYND11 ESR2 ZFPM1 HEYL SOHLH2 MYC PMS2 ZNF736 REL HMGA2 ETV7 ZMYND8 CEBPA AIRE DMC1 NEUROG3 SATB1 NR2F6 TAL1 HOXA5 PARK2 NACC2 XPC ZNF691 RFX5 ZNF711 ONECUT2 ZNF552 SOX4 NFX1 ELK3 ZNF502 NFATC1 ZNF708 KLF17 RFX2 JDP2 TBX2 MTERFD3 RARG KDM3A MCM2 TAF1C NEIL3 NFATC2 ONECUT3 DUX4 BCL11B HELT GABPA EOMES SPI1 SKIL HES5 NKX6-2 E4F1 ZKSCAN3 USP3 TBX18 NFATC3 HNRNPC PCBP3 PTF1A KIN ZNF713 HOXD9 DMRTC1 EHF KLF12 PRDM1 IRF7 SMAD6 NFE2 AKAP8 BATF3 NFKB1 ZNF66P SOX10 LMX1A MTOR SUV39H2 HOXC11 SREBF1 PHF21B ARHGAP35 SMAD5 ZNF56 USF1 CTCF SOX2 XRN2 NR5A2 KCNIP3 SOX3 MTA2 MKL1 NME1 ARNTL DMRTC1B CEBPZ ZNF492 SMARCC1 KLF4 TCF4 ZSCAN21 TNRC18 ZC3H8 CORT HEY2 RRN3 HR TAL2 PURA RELA THRA CSDA GATA2 MYOG ARID5B ZNF660 MXD1 PARK7 MEF2C HOXA2 ZNF460 BAZ2A TCF7L2 SMAD1 KAT2B MLL CREB3 MAFA HMX3 NR1H2 SMARCC2 MEF2B ZNF735 EBF2 HAND1 ZNF8 ZFY ZNF583 NFATC4 NKX2-1 ALS2CR8 DLX1 BARX2 NFAT5 ETV4 HSF2 NKX3-1 NFIA SOX12 SIX2 TBX3 CDC45 PAX5 CDX1 DLX5 PLAGL1 ZIC3 RAD51 AEBP1 KIF2C HNF1B MEN1 FOSL2 MBD3 CLOCK CUX2 HOXB5 CDX2 MYF6 ASCL1 PCGF6 EZH2 ZNF92 SETX MNT JMJD1C RBPJ DDX11 SOX1 TDG SREBF2 HNRNPK DMRTC2 MZF1 CREB3L3 XRCC3 ACTB NKX2-5 DDX60 FEZF1 BHLHA15 MTA1 POU3F2 ZNF730 RAD51D PDX1 SAFB CRIP1 SIX3 ZNF772 PKNOX2 CRY2 NR4A2 CREM DMRT2 SMYD3 NFE2L3 PAX8 KLF13 HMGA1 SOHLH1 BARHL1 SETMAR PRDM15 XRCC2 RAD51AP1 MTERF RBPJL NRIP1 GATA6 RUNX3 AEBP2 ZNF837 DMRTA2 TFAP2C HMBOX1 CEBPB SMARCD2 ZNF256 FUBP3 PAX2 GLI2 RBBP4 ZNF148 CGGBP1 MYB WT1 ZNF675 BARX1 HIVEP1 ZNF716 ZNF418 POU4F3 EP300 TFAM ZSCAN20 PROP1 GMEB2 ZNF430 RUNX2 TP53 KLF1 ZNF718 ARNT2 TERF2 ZFX E2F7 TDP1 NRL JUND PROX1 MECP2 NFIL3 KLF5 XRCC5 KLF10 ALX4 RELB TFAP2E HOXA10 ATF3 ZIK1 HSPD1 ZNF805 LSM14A SOX13 SMARCA4 ATOH1 MITF OSR2 ATF6 ZNF536 TCF12 GATAD2A ZNF664 BCL6B GLI1 YAP1 ZNF219

GO_HEAT_SHOCK_PROTEIN_BINDING Interacting selectively and non-covalently with a heat shock protein, any protein synthesized or activated in response to heat shock. USP19 GPR37 CSNK2A1 NFKBIA ZFP36 NOD2 CDC37L1 IQCG TPR DNAJC9 CDC37 ARNTL TOMM34 EIF2AK3 LIMK1 HSPA1A DAXX NUP62 HDAC8 BAG6 ADORA1 PACRG PDXP FKBP6 CDKN1B DNAJA1 ERN1 TFAM DNAJC2 DNAJA2 HSPA1B BCOR TELO2 DNAJA3 CDK1 NASP UNC45A BAK1 PPEF2 FKBP5 KCNJ11 AHR RNF207 PPID KDR OGDH FAF1 HSPA8 RPS3 ST13 HSPA1L HIF1A STUB1 APAF1 DNAJC7 DNAJC10 GBP1 METTL21A HDAC6 PPP5C HDAC2 STIP1 LMAN2 FGF1 HSF1 DNAJB6 DNAJB1 KPNB1 CREB1 GRXCR2 CHORDC1 DMPK PARK2 MVD DNAJA4 IRAK1 C11orf73 SACS FKBP4 HSPA6 UNC45B NPAS2

GO_ENDONUCLEASE_ACTIVITY_ACTIVE_WITH_EITHER_RIBO_OR_DEOXYRIBONUCLEIC_ACIDS_AND_PRODUCING_3_PHOSPHOMONOESTERS Catalysis of the hydrolysis of ester linkages within nucleic acids by creating internal breaks to yield 3'-phosphomonoesters. DNASE2 SLX1B RNASE2 MUS81 TSEN15 HRSP12 EME1 GEN1 EME2 TSEN34 TSEN2 SLX1A TSEN54 DNASE2B RNASE8 RNASE1 C17orf42 XRCC3 RAD51C RNASE4 SLX4 RNASET2

GO_ESTROGEN_RECEPTOR_BINDING Interacting selectively and non-covalently with an estrogen receptor. NSD1 FOXL2 PRMT2 PCNA NKX3-1 ZNF366 C16orf53 TAF10 ISL1 PPARG TRIP4 PADI2 STRN RERG PPARGC1A MED1 DYX1C1 PHB2 FUS NRIP1 PARP1 MMS19 CTNNB1 DDX17 ARRB1 CNOT1 NCOA1 DDX5 PPID PPARGC1B NCOA6 CCDC62 DDX54 LEF1 WIPI1 SRC

GO_ORGANIC_ACID_TRANSMEMBRANE_TRANSPORTER_ACTIVITY Enables the transfer of organic acids, any acidic compound containing carbon in covalent linkage, from one side of the membrane to the other. SLC16A8 SLC36A2 SLC16A6 SLC17A6 SLC16A9 SLC38A1 SLC17A8 SLC6A9 SLC7A5P2 SLC43A2 SLC1A6 SLC1A5 SLC7A11 SLC7A3 SLC10A6 PQLC2 SLC1A1 SLC3A2 PEX3 SLC6A7 SLC10A3 SLC16A2 SLC36A1 SLC7A8 SLCO2A1 SLC6A5 SLC7A5P1 SLC22A12 SLC10A2 SLC1A3 SLCO1C1 SLC6A15 SLC16A5 SLC38A10 SLCO1A2 SLC38A3 SLC38A6 ABCB11 SLC1A4 SLC7A4 SLC25A2 SLC16A7 SLCO1B3 SLC6A14 SERINC5 SLC3A1 SLC7A1 SLC25A10 SLC43A1 SLC25A12 SERINC1 SLC38A7 SLC7A6 SLC16A1 AQP9 ABCC2 SERINC4 SLC6A18 SERINC2 CTNS SLC6A20 SLC7A2 SLCO2B1 SLC25A15 SLC7A14 SLC6A1 SLC10A1 SLC38A5 SLC16A10 SLC5A12 OCA2 SLC25A22 SLC38A9 SLC36A4 SLC10A5 SLC13A5 SLC1A2 SLC1A7 SLC16A3 AKR1C4 SLC7A7 SLC17A7 SLC7A10 SLC13A2 SERINC3 SLC13A3 SLC16A4 SLC38A2 C3orf55 ABCC3 SLC7A13 SLC6A19 SLC38A4 SLC5A8 SLC6A13 SLC7A5 SLC17A3 SLC6A11 SLC7A9 SLC38A8 BSG SLC6A17 SLC6A12 SLC32A1 SLCO1B1 CEACAM1 SLC25A13 PDPN TMEM44 SLC38A11 SLC36A3 SLC16A14 EMB SLC6A6 SLC25A11 SLC2A9 SLC10A4 SLC25A1 SLC6A16

GO_MOLECULAR_FUNCTION_REGULATOR A molecular function that modulates the activity of a gene product or complex. Examples include enzyme regulators and channel regulators. STIM1 ARFGAP2 TBC1D5 BRSK2 PSD4 SPINK5 ACAP3 ARHGAP12 ARHGDIB ARHGAP18 DCP1B FAM20A ANXA3 NOS1 KCNAB1 IGFBP3 PPP2R5A SERPINA3 SOS1 FGF4 RALGDS CAMK2D SPINK9 AUP1 RGSL1 ARHGAP42 RGS16 RASA2 TRIB1 RASGRF1 APOC2 BMP2K PCP2 TRIP10 MED1 TFAP2B SERPINA2 ESR1 BRCC3 SHOC2 GRIN2B JUN CAMK2N1 CCNK NPY2R WFDC5 GPS1 BAG4 SERPINB6 APOA1 FGD6 RCVRN ENSA FNBP1 SH2D3A PDCD5 A2ML1 CDKN1B SERPINB13 EFNA5 SERPINA1 PIK3CA TIMP3 DENND4B ARHGEF33 ARHGAP5 ARFGEF2 NGF PRKG1 AGAP7 SCG5 PIK3R5 STX4 CISH TRIM23 SPRED2 ARHGEF2 PLEKHG7 C8orf44-SGK3 MED16 PRKCZ JAK2 NCF1C BIRC7 SERPINB4 HPCAL4 PHACTR4 CAMK2B GCGR PPP1R15A DOCK6 CAB39 PTN RPTOR IQSEC1 RASAL2 HPS4 ATP2B4 SPTBN1 TSPAN13 CAMK2N2 TBC1D22B AVP HERC2 PPP1R3C CXCL10 AHSA1 ARHGAP15 APC APAF1 DNAJC7 ERRFI1 CSN2 NOXA1 PSME1 FGF2 ITIH6 DBF4B PSD2 FGF10 ARHGEF11 DOCK8 BIRC3 SOCS7 CAMK2G DENND1B PPP1R2 WNK4 HSPB1 AGAP6 ELP3 GUCA2B DLG1 PPP1R7 MMP14 STX1A SHC2 DNAJA3 ACAP1 DAOA TBC1D2 DLG4 TIMP2 PLEKHG4 A2M FOXL2 CST9LP1 IL5 TBC1D30 PRKAR1A MAT2B IL3RA PLEKHG6 DNMBP ITIH1 ARHGAP19 TAB1 TBC1D8 NRG3 GCN1L1 PLEKHG2 GAS6 PRSS8 SERPINB5 EIF2B1 PPP2R2C LYNX1 RGS22 PITRM1 TBC1D16 CCND1 TRPV1 FGFR3 VIL1 PPP1R2P3 SMAP1 BCL2L13 DENND2D SH3BGRL3 NOD1 TBC1D22A FGD4 EIF2B5 SAG ARHGAP36 CACNG4 MBIP CAV3 DKK1 WNT3 MYO9A DOCK2 OAZ2 BMP2 PDGFA AFAP1L2 BCL10 SERPIND1 SERPINA4 C6orf127 SET CALM1 RPLP1 AGAP9 KCNIP1 RECK SRGAP2P1 CDC42EP4 GNB5 DENND2A RPGR C3P1 KCNE2 ITGB1BP1 SUMO1 RALGAPA2 COL4A3 KCNE1L RICTOR KCNMB4 SOCS3 ANGPTL3 RGS13 ANXA4 WNT1 FNIP1 WBP11 SPTBN2 DAXX ABI1 PLCB1 LRRTM4 RUNDC1 GIT1 SOCS2 ARFGEF1 WFDC1 PI15 ITIH4 CASP3 CDK4 PYCARD PROS1 PFN1 CST8 ANP32E PODN TNNI3 DGKI FGFR1 SPRY2 GDI2 CARD16 ALOX5AP PPP4R4 ARHGEF17 CTGLF11P SLIT2 ARHGAP33 SHC3 TIMP4 PSME3 AGAP2 NEDD4L LRTM1 GRIN2A RTN4RL1 GNAQ CASP1 PRPSAP1 GOPC SYDE2 ALS2CL LEF1 SYNGAP1 WDTC1 CCL8 TBC1D10A PPP2R3A RINL ARHGEF9 RGS1 WFDC10A SLC30A1 RGS19 ARHGAP22 CPN2 TEK RABEP2 CCKBR TBC1D20 FGF23 SERPINB8 TRIB3 MCF2 GTF3C4 SERPINB12 ARHGEF16 TMBIM6 SERPINB1 FGF7 CABP1 SERPINI2 APLP2 GTF2F1 HEXIM1 DBNL NRXN2 KCNAB2 CACNG7 ARF4 CD109 KCNS1 RIN3 PDPK1 SFTPB KCNV1 PDC RAP1GAP2 DUS2L CST6 ZEB2 SERPINB9 DIS3 PLEKHG4B TBC1D2B SPOCK3 RIMS1 RPS27L ACTN2 CNIH3 SH3BP4 EGF ARL2BP FAM13A USP6NL STX8 IRS1 ARHGEF12 CASP9 HBXIP WAS GPSM2 RGS8 C7orf59 BNIP2 CABIN1 ARHGAP32 SERPINH1 MTMR9 KLF4 BCCIP GARNL3 RHOH FFAR1 MARK2 TIAM1 TOR1AIP2 DNAJB1 C3 BIRC6 VTI1B CST11 ADPRH TOM1L1 C4B RASA4 IL1RN FLRT2 RGN RGS14 PPP4R2 RGS11 RABEP1 CCL5 PLN FAM150A PPP1R2P1 CTC1 UGT1A8 ARHGEF26 FGF8 RASGEF1B CACNG2 BAI3 SPP2 CDK5R2 EID1 LRRC4 PFN2 ARHGEF40 TBCK DCN AXIN1 PLEKHG5 FBXO8 SERPINF2 ANKRD27 PPP1R37 SGSM1 DENND4A RABGEF1 ARHGAP11B KCNE3 DENND1A EBAG9 CCNH PSME2 AGAP1 PODNL1 AKAP13 ARL2 PPP2R5C IL18BP SSPO SERPINA11 GFRA1 NCKAP1L ADH7 UGT1A7 C15orf58 ATP6V1H RANBP1 PACSIN3 M1 FAF2 CHRNA7 ANXA2 PPP1R1B PCSK9 PDGFB TBC1D3B PTTG3P HRG SEC14L1 RAC2 LRRC4B SFRP2 DOCK9 FRS2 BAG5 NRTN PIK3R6 PCOLCE LRRC19 AMBP PPP1R3B SPINK8 BIRC2 TAGAP ANKLE2 RCBTB2 DNM1L C17orf42 ARHGAP10 HSPB2 CYTH2 PKP2 WNT10B PKIB ELMOD3 PSMF1 PPP4R1 ARHGAP39 PPP1R14D FGF6 RAPGEF5 DNAJC3 SERPINA5 C13orf15 HSPBP1 SIPA1 RGS5 KCNAB3 TBXA2R CST2 ADAP2 APP ARHGAP20 PRKCB UBA2 BIRC8 BCAS3 EPO ARFGAP3 RAPGEF4 SH3BP5 SPINT3 VAV3 RAP1GDS1 TMEM110 SPRED1 WFDC13 NGFRAP1 BCAR3 GREM1 PRKAG2 OVOS CSTL1 COL28A1 FKBP1B GM2A BAD PPP1R16A PSD PPP1R14C SOCS4 MTMR12 ARAP1 ARF1 RIC8B RABIF DNAJC10 MCF2L2 ARHGDIA C16orf7 NF1 FETUB LLGL2 CACNA2D3 VCP ATP1B1 CACNG5 FAM13B WNT11 TGFB1 BST2 PPP1R1A STK4 CKS1B BSND PREX2 ARHGAP28 SH3BP5L WFDC8 IGBP1 NCF4 LCN1 SLPI ELFN1 AMIGO1 KLB VAV1 PPP1R2P9 GHRL PSD3 TBC1D1 FGD2 PPP1R10 SLX4 SPOCK1 ITSN1 DPP6 SERPINB10 GFRA4 CAPN3 ANXA2P2 WASL IPO5 SLC9A3R1 R3HDML KCNIP2 SPINK6 GMIP GUCA2A CACNG8 LTF RP2 SHC1 RASAL3 MYBPC3 GFRA2 TRIB2 CHML APOA4 C5 TBC1D21 MALT1 NEDD4 MOB1B DRD4 YWHAH PSAPL1 RAB3A MON1A DEPDC1B NOTCH1 FYN RGS3 ARHGEF3 ITSN2 FRY RIN1 AMPH RGS10 DPEP1 ARAP3 RCAN2 BCR TMPRSS3 CTSC CST9 C4A DOCK3 ARRB1 ATP1B3 ARHGAP21 RAPGEFL1 EIF2AK2 ITIH3 SPINK4 ACAP2 PPP1R1C CDKN2C RPS6KA1 CDKN1A SGK3 AZIN1 FRS3 SERPINF1 CST1 CARD18 CDKN2D PTPLAD1 SERPINI1 PDZD3 RAB3GAP2 PHACTR1 SPINT1 KCNK2 KCNIP3 GFRA3 PIK3R2 STX7 TBC1D19 NCF1B FGFR1OP LOC390940 KCNMB3 TBC1D25 WNT2 AGAP3 HSPH1 RGL4 HTR2B ARHGAP35 CYTH1 AGFG2 GPRC5B CALM3 PSMD2 CALM2 KITLG ARHGAP6 PYGO2 CRISP1 FXYD2 OGT GLMN RIN2 EIF2B2 PCNA RGS17 SGK1 IL2RB AGFG1 DNAJB6 KIAA1967 GPS2 C15orf62 RASGRP3 FGF20 DOCK7 TRAPPC1 OCRL PDGFRA ARFGAP1 CHM RCC1 KCNE1 IL2RG CCS PTTG2 ARHGEF4 CXCL13 RASGRP2 PPP2R5B CDC42SE1 RAB4A PSMD3 CXCL1 CKS2 APBA3 H2AFY FAM116B AHSG CCNE1 PSAP BTC SERPINA10 PRKAR1B NCF1 ARHGAP1 ASAP2 CLPX SERPINB11 DBF4 RTN4RL2 PSMD1 LRRK2 SH2D3C SERPINB7 CCNC RCAN3 NRXN1 PRKCD PXDN ARHGAP9 SGK2 PPP2R1B RAP1GAP UGT1A1 CDK5 CHN2 ERCC6 GSTP1 GMFG PPP2R1A CHAD TNFSF14 GCLM RALGAPB GDI1 ARHGEF15 ARTN GRIN2C PDGFRB RASAL1 TANK DUSP19 UCHL5 LRRC4C PPP1R36 SPTA1 DNAJC24 ITIH5 CDC20B PRKCE NCOA2 SPTB ARAP2 ECT2 FXYD1 VAMP8 EIF2B3 GPSM3 ADPRHL1 STRADA PINX1 TBC1D3F DLC1 THY1 FAM150B AGT GPC3 CTSA IL36RN PIK3R3 STXBP5L ARHGEF10L HYAL2 CDKN2A RSC1A1 TOR1AIP1 CDK5R1 LRRC15 ADRB2 VAV2 TBC1D9B IQSEC2 ARHGAP44 PPP1R26 TSC1 GRTP1 SCN1B GRPEL2 ARHGAP31 RUNDC3A PRKRA GUCA1C SPINK2 FGF17 DNAJC2 MAL IGF2 TBC1D9 GRM7 ARHGEF1 PPP2R5E PLCE1 SPTBN5 FZR1 ABR RPS6KA3 EIF2B4 CHP SPINT4 STXBP5 SH3PXD2A HERC1 NPY NLRP1 SKI KAL1 DOCK10 HBEGF PLEKHG1 SIPA1L3 DOCK11 OAZ3 NLRC4 GPLD1 VEGFA PIAS3 SIPA1L2 RASGRP1 ERBB3 RTN4R ADAP1 AGAP4 RGL3 RGP1 PSME4 DENND5A TBC1D10C TFPI2 ARHGAP17 ALS2 ARHGEF7 CST3 RAPGEF6 WFIKKN1 RGS2 FGFR2 RNH1 LRPAP1 ASAP1 LTC4S CRB2 FARP2 ANGPT1 RASGEF1C ITIH2 ADRM1 IBTK NOL3 PI3 FAF1 TEN1 JAK3 ADC FURIN PPP1R16B HSP90AA1 ARHGEF25 DNAJC1 WFDC3 SERPINA9 OVOS2 NET1 ARHGEF38 FLRT3 RCAN1 PRKAR2A RABGAP1L ARHGEF10 APOH SPTBN4 KIAA1244 CST9L GRIN2D IL5RA PZP SBF1 ARHGAP26 TRAPPC4 DNMT3L NODAL HMSD CFLAR RASA1 PPP2R2D CHN1 GUCA1A CDC20 TIMP1 RAPGEF2 SPINLW1 GP1BA RAP1A PHACTR2 LOC100507055 CACNA2D4 APOA2 B3GAT3 CDC37 NCAM1 DPM2 WFIKKN2 SFN AHSA2 PDE6H ELMOD1 CABP4 AXIN2 CASP8AP2 SERPINB2 GAPVD1 GPSM1 CAV1 PIF1 KIT MAPK8 NPM1 KIAA1432 TINF2 EVI5L CTAGE5 ATP7A SOS2 FGF22 FGF9 SCGB1A1 LMTK2 CARD17 PPP1R35 GMFB AGTR2 SIPA1L1 LAT IQGAP1 NYX FGF16 SNF8 CD24 KNDC1 KCNIP4 CCNL1 C6orf126 TAOK3 PPARGC1B RASGRF2 RGS12 SERPING1 EREG WFDC12 PI16 TBC1D12 PSMD14 DENND5B HEXIM2 STARD8 FGF18 PKIG FGF13 SMAP2 MMP24 PCSK1N FGD5 PTPN3 FGD1 TBC1D8B PPP1R39 APOC3 ELMO1 SRI ABCE1 PPP2R2A SCLT1 CPAMD8 PLXNB1 PRPSAP2 PRDX3 RING1 DOCK1 ANGPT4 SERPINE1 FBLN1 GUCA1B RFC1 ARHGDIG STARD13 IL2RA P2RY12 TBC1D14 DCP1A FARP1 PTK2 UCN LRRTM1 UBE2L3 CDK5RAP1 AGAP5 GNB2 SNTA1 FXYD3 CSF2RA IQGAP2 SH3BP1 PTGIR DOCK4 FAM58BP GRM2 NLRP12 LOC400986 PHACTR3 WNT7A PARK7 PDE6D ARHGEF19 PARP16 COL7A1 CARD8 ARHGAP30 STK11 PHPT1 NCK2 GRIN1 SERGEF BAG3 FNTA PPME1 LLGL1 LRRTM3 PAK2 SERPINA12 WNK3 IKBKAP SCN4B PPP1R12A SYDE1 EPS8L1 KAT2B SGSM3 TSC2 ANKRD54 INCA1 PREB TBC1D4 PDE6G RANGRF CSNK2B YWHAE PROL1 ARHGAP29 WFDC6 IPO7 OPHN1 HMHA1 CDC42EP2 GCHFR ARHGAP24 RGS21 NKX3-1 NCK1 TNK2 TFPI PREX1 DOCK5 WFDC10B ANGPTL4 IL3 ERBB2 FGF3 ARHGEF6 APOC1 PTPRA RABGAP1 FGFR4 GIT2 JAK1 RTKN RAPGEF1 PPP1R27 WNT8A RGNEF PINK1 TBC1D3H MMP17 ARHGEF5 PPP2R4 ARHGAP4 RGL2 APOE SOCS6 SERPINA6 KRIT1 POT1 YWHAG SPATA13 NRG2 FLCN CCDC109B FGF1 WNT5A SESN2 CCNY STK3 DENND4C FXYD4 STIM2 RET PPP2R3B KCNMB1 CFTR AGAP11 CSTB GRPEL1 RAB3IP PRSS41 APOA5 TBC1D13 GPD1L CYTH4 WNK1 PRKAR2B PPP1R8 SCN2B SERPINE2 NSMAF CDKN2B NGEF TBC1D10B SPTAN1 SCN3B FLRT1 RASGRP4 SPINK7 ITPR1 MT3 FN1 CDC42EP1 ARHGAP27 TESC BGN CCZ1 FAM58A ADRB1 ARHGAP11A CAST TIAM2 NPRL3 RGS20 BIRC5 ARHGAP40 SPINK1 WNT4 CCNE2 ANXA5 CSF2RB RASGEF1A OAZ1 BAZ1B DEPDC5 DENND3 CSF2 TBC1D17 CPEB2 CCL3 KL OBFC1 PEBP1 PPP1R14B IQSEC3 RGS6 RGL1 BAG1 AKT1 CCNT1 LYPD1 SEPT2 WNT3A RALGPS2 TNFAIP8 DEPDC7 DMPK SNCB SERPINB3 SLN RALGAPA1 SRGAP1 MADD CCNL2 CST7 PGAM5 XIAP ARHGAP23 KCNA5 TBC1D15 PPP1R9B ATP1B2 ATPIF1 PRKAG1 PPP1R12B LRP6 MCF2L CLPS MMP15 ELP4 CYTH3 CRIM1 TBCD SPINK14 PAPLN NRG4 RAB3IL1 EVI5 MGST2 ARHGEF18 PRLR ELFN2 SPINT2 IRS2 FNBP1L SAE1 EGFR LAMTOR3 RGS4 NEFL LRRC26 CSTA GDNF CTSH ELMOD2 APOBEC1 FXYD5 PRDX5 PLAA RACGAP1 RAB3GAP1 RIC8A LAMTOR1 RAPGEF3 SERPINC1 ECT2L DENND1C KCNMB2 LPA RGS9 PKIA PPP2R2B USP14 HPS1 PSPN CAMK2A SNCA CIB1 PPP1R17 RGS7 GBF1 SGSM2 PCOLCE2 PPP1R11 FAM116A KIDINS220 CST5 ESR2 ARHGEF37 SPOCK2 DAB2IP DPP10 SPINK13 NOXO1 ASPN TBC1D24 DENND2C RENBP CCNT2 PTTG1 NAIP ARPP19 ARHGAP8 C19orf2 BCL2 BAG2 NPRL2 TBC1D26 ERBB4 SERPINE3 HSP90AB1 FKBP1A TXNIP GNB2L1 EPS8L2 GRB2 GRM3 KALRN PIK3R1 TBC1D3 NRXN3 COL6A3 MMP16 MAP2K2 FBXW7 TRIO ANXA1 ARL1 IL2 SERPINA7 KCNS3 FGF19 FGF12 SOCS5 OBSCN RGS18 ASAP3 SBF2 DEPDC1 ALDH1A1 PLEKHG3 MYO9B LRRC66 TBC1D7 KNG1 FGD3 RALGPS1 CDC42EP3 ST5 SRGAP2 CACNG3 FGF5 LXN LAMTOR2 RASA3 INPP5B SOCS1 PPP2R5D PPP1R14A WFDC2 RANGAP1 PRKRIP1 UMODL1 C9orf100 CDKN1C ARHGAP25 ABCC9 AKAP9 CBX8 MAP2K1 RALBP1 SH3PXD2B NRG1 SRGAP3 CD27 RASA4B CST4 MAPK8IP1 TBC1D3C CDC42EP5

GO_NEUROPEPTIDE_BINDING Interacting selectively and non-covalently and stoichiometrically with neuropeptides, peptides with direct synaptic effects (peptide neurotransmitters) or indirect modulatory effects on the nervous system (peptide neuromodulators). MC4R SSTR4 NMUR2 GALR1 MRGPRX2 OPRL1 MC3R GALR2 NPBWR1 GPR149 SSTR2 SSTR1 NPBWR2 GPR44 GPR1 ADCYAP1R1 OPRM1 SSTR5 SSTR3 OPRK1 OPRD1

GO_ALCOHOL_BINDING Interacting selectively and non-covalently with an alcohol, any of a class of alkyl compounds containing a hydroxyl group. ABCG1 SYP DPM1 ANXA6 ADH4 APOF TSPO NPC1 RBP1 TSPO2 STAR TRPC6 ASTN2 OSBPL3 TRPC5 ERLIN1 CETP OSBPL8 APOA5 TRPC4 SCP2 STARD3 GLE1 OSBP2 ADH7 APOA4 PLCL1 RBP7 TRPC1 ABCA1 PRKCE STARD5 RPH3A APOE ITPR3 APOD NR1H3 ADAP1 XPR1 TRPC3 OSBPL10 SYT2 CAV1 CDIPT PMP2 ADAP2 RBP2 PROM2 RBP4 SCAP STARD4 RBP5 ERLIN2 APOA2 PTCH1 SOAT2 OSBPL2 C8G CRABP1 APOC3 APOA1 RBP3 ITPR2 OSBPL5 STARD6 CYTH2 OSBPL7 SOAT1 ITPR1 RLBP1 NPC2 LRAT CRABP2

GO_INTRACELLULAR_CHLORIDE_CHANNEL_ACTIVITY NA BEST1 ANO10 ANO4 ANO8 CLCA2 ANO1 ANO6 ANO9 CLCA1 TTYH3 ANO2 CLCA4 NMUR2 ANO5 ANO3 ANO7

GO_CYSTEINE_TYPE_ENDOPEPTIDASE_ACTIVITY_INVOLVED_IN_APOPTOTIC_PROCESS Catalysis of the hydrolysis of internal, alpha-peptide bonds in a polypeptide chain by a mechanism in which the sulfhydryl group of a cysteine residue at the active center acts as a nucleophile, and contributing to the apoptotic process. CASP7 CASP4 CLC CASP12 CASP9 CASP5 CASP14 CASP2 CASP6 PYCARD CASP10 CASP3 CFLAR CASP8 CASP1

GO_G_PROTEIN_COUPLED_AMINE_RECEPTOR_ACTIVITY Combining with an extracellular amine and transmitting the signal across the membrane by activating an associated G-protein; promotes the exchange of GDP for GTP on the alpha subunit of a heterotrimeric G-protein complex. OR10J5 HTR3C OR10H3 CHRM4 OR11H4 ADRB3 HTR2C ZNF219 CHRM3 OR56A1 OR56A5 OR10H4 HRH4 OR56A4 HTR1F ADRA1A OR6T1 HTR3D ADRA2A CHRM1 HTR1B OR5T3 HTR2B TAAR6 ADRA1D ADRA1B CHRM2 CHRM5 DRD2 HTR6 ZACN HTR1A OR10H2 OR10J6P OR11H7 HTR3E GPR101 OR13F1 ADRA2C TAAR1 HRH1 OR5T1 HRH2 HTR5A OR10H5 HTR1D ADRB2 TAAR8 OR5T2 HRH3 TAAR9 ADRA2B HTR4 HTR7 TAAR3 TAAR5 TAAR2 HTR1E HTR2A ADRB1 OR10H1

GO_VOLTAGE_GATED_POTASSIUM_CHANNEL_ACTIVITY Enables the transmembrane transfer of a potassium ion by a voltage-gated channel. A voltage-gated channel is a channel whose open state is dependent on the voltage across the membrane in which it is embedded. KCNH2 KCNV2 KCNJ10 KCNJ5 KCNA7 KCNJ15 KCNH7 KCND3 KCNJ16 KCNE2 KCNJ4 KCNK3 KCNAB2 KCNAB3 KCNA6 KCNK18 KCNC4 KCND2 KCNA4 KCNE1L KCNH5 KCNJ14 KCNA10 KCNV1 KCNB2 KCNJ18 KCNK2 KCNQ2 KCNS1 KCNA1 KCNJ8 KCND1 KCNB1 CNGA1 KCNJ2 KCNJ1 KCNE3 ABCC8 KCNU1 KCNJ13 CNGA4 HCN4 KCNQ3 KCNG2 KCNK1 KCNC3 KCNH4 KCNS2 KCNH8 KCNA2 KCNIP2 KCNQ4 KCNA3 KCNH6 KCNT2 KCNJ9 KCNT1 CNGB1 KCNJ12 KCNK5 KCNMA1 KCNK6 CNGA2 KCNAB1 CNGA3 KCNG3 KCNG4 REST KCNQ5 KCNS3 KCNE4 KCNJ3 KCNC2 CNGB3 KCNF1 KCNK9 KCNG1 KCNA5 KCNJ6 KCNC1 KCNH3 HCN3 KCNJ11 KCNQ1 HCN1 HCN2 KCNE1 KCNH1

GO_TUMOR_NECROSIS_FACTOR_RECEPTOR_SUPERFAMILY_BINDING Interacting selectively and non-covalently with any member of the tumor necrosis factor receptor superfamily. TRAF1 CASP8AP2 CASP3 DAB2IP FADD TNF BRE BID TNFSF14 TNFSF4 TNFSF10 NGFRAP1 LTB MYD88 FEM1B PIDD NGF TRAF4 NOL3 CD70 TNFSF12 CFLAR TRAF3 EDA LTA TRAF6 SIVA1 MADD TNFSF9 CD40LG TRIM37 STAT1 RIPK1 CASP8 TNFSF11 TMBIM1 TNFSF13 TRAF2 TRAP1 FASLG TRADD TNFSF8 TNFSF15 TNFSF18 TNFSF13B

GO_AMINOPEPTIDASE_ACTIVITY Catalysis of the hydrolysis of N-terminal amino acid residues from in a polypeptide chain. MMP14 RNPEPL1 LTA4H DPP3 DPP4 CTSH ENPEP DPP7 TRHDE ERAP2 DPP8 XPNPEP2 LAP3 ANPEP DNPEP MMP16 XPNPEP1 FAP DPEP3 METAP1D DPEP2 METAP2 PRCP NPEPL1 BLMH NAALAD2 DPEP1 CTSL2 MMP15 C9orf3 PHEX PRSS16 ERAP1 TPP2 PEPD MMP17 AQPEP XPNPEP3 DPP9 LOC440434 METAP1 F11 RNPEP DPP6 DPP10 NPEPPS LNPEP

GO_FERROUS_IRON_BINDING Interacting selectively and non-covalently with ferrous iron, Fe(II). HEPH TF HAAO FXN ALKBH1 DNAJC24 TET2 CDO1 FECH KIAA1456 ISCU ALKBH6 ACP5 ALKBH3 ISCA2 FTO ISCA1 EGLN2 ALKBH8 TH ALKBH2 SNCA

GO_TRANSCRIPTIONAL_REPRESSOR_ACTIVITY_RNA_POLYMERASE_II_CORE_PROMOTER_PROXIMAL_REGION_SEQUENCE_SPECIFIC_BINDING Interacting selectively and non-covalently with a sequence of DNA that is in cis with and relatively close to a core promoter for RNA polymerase II (RNAP II) in order to stop, prevent, or reduce the frequency, rate or extent of transcription from an RNA polymerase II promoter. ZNF536 ASCL2 CC2D1A SKOR2 ZFPM1 ZNF219 ESRRA SKI FOXO1 FOXP2 TSHZ3 ATF3 MLXIPL EN1 HHEX KCNIP3 NKX6-1 RCOR1 CTCF MZF1 ZNF217 SALL1 TSHZ1 SREBF2 TCF3 ZBTB7A ZNF746 BATF3 PROX1 NFIL3 PRDM1 OVOL1 ASCL1 PRDM5 DLX4 GATA1 ZGPAT SNAI2 KLF16 CUX2 GZF1 TBX15 INSM1 TFAP2A CREBBP ZFPM2 NR2C1 SNAI1 TSHZ2 BHLHE40 ETS2 PPARA TBX3 NFXL1 TGIF1 IFI16 TCF21 E2F8 SPI1 SKIL GCFC1 HELT ZKSCAN3 ZNF148 ZFP90 NKX6-2 HES5 TFAP2C BTG2 HAND1 SP3 GFI1 NKX3-2 NFATC2 NFATC4 HES1 KLF17 C2orf3 JDP2 TBX2 HINFP AEBP2 IRF3 NFX1 SKOR1 MXD1 BACH1 HOXA2 NR2E1 PROX2 NACC2 ZBTB20 BHLHE41 CC2D1B YY1 RELA NFE2L3 DACH1 BCL11A ZNF281 SCRT2 NEUROG3 HMGA2 GATA3 POU4F2 HSF1 MYPOP NR1D1 REST

GO_CULLIN_FAMILY_PROTEIN_BINDING Interacting selectively and non-covalently with any member of the cullin family, hydrophobic proteins that act as scaffolds for ubiquitin ligases (E3). SKP1 KCTD17 DCUN1D5 KCTD2 ANAPC11 KCTD5 DCUN1D4 PARK2 CCDC22 RBX1 DCUN1D3 DCUN1D1 RNF7 DCUN1D2

GO_SMAD_BINDING Interacting selectively and non-covalently with a SMAD signaling protein. EID2 MEN1 IPO7 JUN TGFBR1 PPM1A SMAD6 SNW1 TGIF1 USP9X C13orf15 C18orf1 COL5A2 ANKRD1 TGFBR2 AXIN1 RNF111 GATA4 TCF12 FLNA ACVRL1 SKI BMPR1B SKOR2 PURB FOS SMAD3 AXIN2 ACVR1B SMAD7 SMAD2 USP15 FOXH1 MEF2A HIPK2 TOB1 CTNNB1 PURA YY1 CITED1 SMURF2 SKOR1 FKBP1A DAB2 PML SMAD1 PAX6 ZC3H3 USP9Y HMGA2 ACVR1 COL1A2 RANBP3L MAGI2 SMAD4 BMP2 PRDM16 TGFBRAP1 TRIM33 SKIL SMURF1 STUB1 TGFB1I1 RANBP3 BMPR1A PMEPA1 PARP1 COL3A1 MYOCD TGFBR3

GO_RNA_HELICASE_ACTIVITY Catalysis of the reaction: NTP + H2O = NDP + phosphate, to drive the unwinding of a RNA helix. DHX33 MOV10L1 DHX37 DHX8 EIF4A1 DDX42 DDX50 DDX47 DDX25 UPF1 DHX16 DDX6 DDX56 DHX38 DDX10 DHX40 DDX3X DDX24 IGHMBP2 DDX17 DHX36 DDX52 HELB DQX1 TDRD12 G3BP1 DDX39A TDRD9 SNRNP200 SUPV3L1 DDX41 DDX28 DHX15 DDX18 DDX20 SKIV2L2 YTHDC2 DHX29 DDX4 DDX1 DDX53 DDX49 DDX21 DHX35 DHX32 RAD54B EIF4A2 DDX23 DDX27 DHX30 EIF4A3 DDX55 DDX31 DDX46 SKIV2L DDX59 DHX34 DDX19B DDX43 DDX5 DDX3Y DDX19A DHX9 DDX54 DDX51 DDX39B DHX57

GO_PROLINE_RICH_REGION_BINDING Interacting selectively and non-covalently with a proline-rich region, i.e. a region that contains a high proportion of proline residues, in a protein. PFN1 NEDD4 ABI2 ABL1 GHR CSK TCERG1 ITSN1 BAIAP2L1 RABAC1 YAP1 FAM59A BAIAP2 CYLD PRPF40A WBP4 CCND1 APBB1

GO_GUANYLATE_KINASE_ACTIVITY Catalysis of the reaction: ATP + GMP = ADP + GDP. DLG2 MAGI3 MPP1 TJP2 MPP2 DLG4 DLG3 GUK1 DLG1 MPP3 CASK CARD11

GO_TROPOMYOSIN_BINDING Interacting selectively and non-covalently with tropomyosin, a protein associated with actin filaments both in cytoplasm and, in association with troponin, in the thin filament of striated muscle. S100A6 LMOD1 NEBL PYCARD TMOD2 TNNT1 TNNT2 LMOD3 TMOD4 LMOD2 TNNT3 CALD1 TMOD1 TMOD3

GO_CALCIUM_CHANNEL_REGULATOR_ACTIVITY Modulates the activity of a calcium channel. STIM2 NPY CACNA2D4 PRKCB PRKG1 NRXN1 SGK2 CABP4 CACNA2D3 PLN TMEM110 AMBP GNB2 SLC30A1 STIM1 GRM2 C8orf44-SGK3 TNNI3 CRISP1 ITPR1 STX1A FKBP1A FKBP1B NPY2R SGK3 GRM3 HPCAL4 SGK1 CCDC109B PHPT1 NRXN3 CAV3 PACSIN3 NRXN2 GRM7 SRI TSPAN13

GO_TELOMERIC_DNA_BINDING Interacting selectively and non-covalently with a telomere, a specific structure at the end of a linear chromosome required for the integrity and maintenance of the end. XRCC5 OBFC2B SMG5 HNRNPD CTC1 PIF1 XRCC6 APEX1 ACD KDM1A OBFC1 TEN1 HNRNPA2B1 NCL TERF1 UPF3A POT1 TERT HNRNPA1 SMG1 SMG7 RAD50 UPF2 TERF2 UPF1 PURA SMG6 TINF2 HMBOX1 TERF2IP

GO_SCAFFOLD_PROTEIN_BINDING Interacting selectively and non-covalently with a scaffold protein. Scaffold proteins are crucial regulators of many key signaling pathways. Although not strictly defined in function, they are known to interact and/or bind with multiple members of a signaling pathway, tethering them into complexes. CASP8 PANX1 CASP5 MDM2 IKBKB CACNA1G CIT KRT15 NLGN4Y SCN5A CACNA1H VIM PARK7 KRT5 KCNH2 CRIPT DLG4 NLGN1 MAP3K7 NOS1 NCK2 ATP2B4 MAP2K2 NOS3 KCNA5 DLL1 P2RY1 SHANK1 LRP4 TREM1 CHUK NLRP1 KRT8 GJA1 SRC MAPK3 GRID2 TREM2 PDE4D ADCY5 NLGN3 PHF6 NLGN4X DSP SHANK3 CAV2 KRT18 KCNQ1

GO_DNA_HELICASE_ACTIVITY Catalysis of the reaction: NTP + H2O = NDP + phosphate, to drive the unwinding of a DNA helix. DHX9 ATRX ERCC3 RUVBL2 GINS4 MRE11A MCM2 G3BP1 GINS2 RECQL4 CHD4 C10orf2 RAD50 BRIP1 MCM7 SUPV3L1 GINS1 ASCC3 MCM4 SETX IGHMBP2 MCM3 CHD1L RTEL1 DDX12P DDX11 WRN CHD3 ERCC6 RECQL5 PIF1 XRCC6 NBN XRCC5 HELB RAD54B ERCC2 RUVBL1 RECQL CHD8 MCM6 DDX3X ERCC8 INO80 CHD1 MCM5 CHD2 CDC45 ZRANB3 BLM GTF2H4 DNA2 FBXO18

GO_ACTIVE_ION_TRANSMEMBRANE_TRANSPORTER_ACTIVITY Catalysis of the transfer of an ion from one side of a membrane to the other up the solute's concentration gradient. This is carried out by binding the solute and undergoing a series of conformational changes. Transport works equally well in either direction. ATP13A3 SLC4A5 ATP5F1 ATP2C2 ATP6V1G3 ATP6V1H ATP2B1 ATP2B4 SLC12A6 SLC13A4 ATP5H ATP7A SLC23A2 ATP8A1 SLC34A3 FXYD2 SLC1A1 ATP4A ATP1A4 SLC17A2 PCYOX1 TCIRG1 ATP13A5 SLC12A9 ATP6V1A ATP5G1 ATP4B ATP1A1 ATP1B1 ATP6V1C1 ATP5E ATP6V0E1 ATP6V0D1 SLC12A5 ABCC6 ATP5G3 ATP1B3 ABCC10 ABCB11 ATP1B2 ATP2B3 ATP5C1 ATP1A2 ATP2A2 CFTR ATP5O ATP5EP2 ATP7B ATP2A1 SLC4A7 ATP2A3 ATP5D ABCC2 ABCC4 ATP6V1E1 ABCC5 ATP2C1 ATP12A SLC4A4 SLC12A3 ATP2B2 ATP6V0C SLC4A9 ABCC1 SLC1A2 SLC13A1 SLC13A5 ATP6V0E2 SLC12A4 ATP6V1C2 ATP5A1 ATP6AP1L ATP6V1G1 ATP6V0B SLC13A2 SLC12A2 ATP6V0A4 SLC34A2 ATP6V1G2 SLC17A7 ATP13A4 SLC13A3 ATP13A2 SLC34A1 ATP6V1B2 SLC17A4 SLC12A7 ABCC3 ATP6V1F SLC17A3 SLC17A1 ATP13A1 SLC5A5 ABCC8 SLC12A1 ATP5B SLC23A1 ATP6V0A2 ATP6V0A1 ATP6V1E2 ABCC9 ATP5G2 SLC20A1 ATP1A3 ABCC11 SLC20A2 ATP6AP1

GO_OPSONIN_BINDING Interacting selectively and non-covalently with an opsonin, such as a complement component or antibody, deposited on the surface of a bacteria, virus, immune complex, or other particulate material. APCS C1QBP CD93 CR1 C4A CRP ITGAV MASP2 PTX3 PHB CALR

GO_ADENOSINE_DEAMINASE_ACTIVITY Catalysis of the reaction: adenosine + H2O = inosine + NH3. CECR1 ADA ADARB2 ADAT1 ADAL ADARB1 ZBP1 ADAR ADAT2 ADAD2 ADAD1

GO_NEUROTROPHIN_RECEPTOR_BINDING Interacting selectively and non-covalently with a neurotrophin receptor. NGFRAP1 NTF4 BDNF NTRK1 PIK3R1 NGF ZNF274 NTF3 PLCG1 SHC1 EFNA5 FRS2 GRB2

GO_BILE_ACID_TRANSMEMBRANE_TRANSPORTER_ACTIVITY Enables the transfer of bile acid from one side of the membrane to the other. Bile acids are any of a group of steroid carboxylic acids occurring in bile, where they are present as the sodium salts of their amides with glycine or taurine. SLC10A2 ABCC3 SLC10A6 SLCO1C1 SLCO2B1 ABCB11 SLCO1B1 SLC10A5 SLCO1B3 CEACAM1 SLC10A3 SLC10A4 SLCO1A2 AKR1C4 SLC10A1

GO_RNA_POLYMERASE_II_CARBOXY_TERMINAL_DOMAIN_KINASE_ACTIVITY Catalysis of the reaction: ATP + (DNA-directed RNA polymerase II) = ADP + phospho-(DNA-directed RNA polymerase II); phosphorylation occurs on residues in the carboxy-terminal domain (CTD) repeats. CDK12 CDK1 CCNK CDK8 GTF2H3 CDK7 GTF2H1 ERCC3 CCNH ERCC2 GTF2H4 MAPK1 CDK13 CDK9 GTF2H2 MNAT1

GO_PHOSPHATIDIC_ACID_BINDING Interacting selectively and non-covalently with phosphatidic acid, any of a class of glycerol phosphate in which both the remaining hydroxyl groups of the glycerol moiety are esterified with fatty acids. JPH2 COMMD1 NRGN MICALL1 PLCD1 C11orf83 PITPNC1 MAPKAP1 GSDMD PACSIN2 ATP13A2 RAPGEF2 SESTD1 MARK1 RAPGEF6 PITPNM1

GO_EPHRIN_RECEPTOR_ACTIVITY Combining with an ephrin to initiate a change in cell activity. EPHA7 EPHB1 NTRK3 EPHA4 EPHA1 EFNB3 NTRK1 EPHB4 EFNA4 EFNA3 EPHA2 EPHA5 EPHA3 EPHA10 EPHA8 EPHA6 EPHB2 EPHB6 EPHB3

GO_ENDODEOXYRIBONUCLEASE_ACTIVITY Catalysis of the hydrolysis of ester linkages within deoxyribonucleic acid by creating internal breaks. DNASE2B RAD51B SLX1A ERCC1 DMC1 C17orf42 FAN1 RBBP8 RAD51C DNASE2 DCLRE1C SLX1B APEX1 TATDN2 RAD51D NEIL1 TATDN1 RAD51 DNA2 EME1 FEN1 NEIL2 ZRANB3 HMGA2 MBD4 BIVM DNASE1 ENDOV NEIL3 MRE11A XRCC3 APLF DNASE1L3 RPS3 ERCC5 SLX4 RAD50 ERCC4 ALKBH1 POLB OGG1 MUS81 XRCC2 SETMAR EXO1 GEN1 DICER1 LOC100128274 APEX2 NTHL1 EME2 SMUG1

GO_ATPASE_REGULATOR_ACTIVITY Modulates the rate of ATP hydrolysis by an ATPase. GRPEL1 DNAJC7 DNAJC10 BAG2 BRSK2 AHSA1 ATP1B2 ATPIF1 RAB3A PFN1 ATP1B3 RAB4A DNAJC1 TOR1AIP1 BAG3 BAG1 TOR1AIP2 DNAJB6 DNAJB1 PFN2 HSPH1 DNAJC24 PLN ATP1B1 DNAJC2 BAG4 MYBPC3 BAG5 AHSA2 GRPEL2

GO_MRNA_3_UTR_AU_RICH_REGION_BINDING Interacting selectively and non-covalently with a region containing frequent adenine and uridine bases within the 3' untranslated region of a mRNA molecule. HNRNPD CPEB1 CPEB3 CPSF1 ZFP36 KHSRP ZFP36L1 ZC3H12A ZFP36L2 ELAVL1 CPEB2

GO_C2H2_ZINC_FINGER_DOMAIN_BINDING Interacting selectively and non-covalently with a C2H2-type zinc finger domain of a protein. The C2H2 zinc finger is the classical zinc finger domain, in which two conserved cysteines and histidines co-ordinate a zinc ion. LEF1 EHMT2 SRRM2 HMGA2 ZXDA THAP7 U2AF2 WT1 EBF1 TAF9 EHMT1 MBD2 GATA1 ZXDC GATA2

GO_TRANSMEMBRANE_RECEPTOR_PROTEIN_PHOSPHATASE_ACTIVITY Combining with a signal and transmitting the signal from one side of the membrane to the other to initiate a change in cell activity by catalysis of the reaction: a phosphoprotein + H2O = a protein + phosphate. PTPRM PTPRH PTPRF PTPRB PTPN6 PTPRD PTPRN2 PTPRK PTPRG PTPRO PTPRZ1 PTPRE PTPRR PTPRC PTPRU PTPRA PTPRS

GO_PDZ_DOMAIN_BINDING Interacting selectively and non-covalently with a PDZ domain of a protein, a domain found in diverse signaling proteins. SLC34A1 LIN7B CXXC4 FZD4 PSEN1 SLC9A3R1 SNTA1 RPS6KB1 USHBP1 SDC2 SNTB1 GJA1 F11R PLEKHA1 KIDINS220 EXOC4 GRIK5 MPP6 DLG4 ADRB1 ARHGEF16 CCDC88C RAPGEF2 ERC1 KCNJ4 CLCN3 GRIK2 ARHGAP29 DTNA FZD1 BAIAP2 TMEM88 PDZK1 TBC1D10A FZD2 DLG3 ACCN3 LLGL2 LNX2 FZD3 SLC22A12 ADAM17 CADM1 KIF14 SHISA9 ACOX1 NSF SLC9A3 LNX1 TGFBR3 GNG5 ATP2B3 GRASP GNG12 GRIA1 CFTR FZD8 CRIM1 GRID2 SLC26A6 NLGN1 PLEKHA2 SLC22A4 MPP3 SRR LPAR1 NKD1 GIPC1 ACVR2A PTEN ATP2B4 MAP2K2 CXADR ATP2B1 FZD7 CIT SNTG2 DOCK4 SSTR2 PARK2 SLC22A5 MUC17 LIN7C CRIPT

GO_SOLUTE_SODIUM_SYMPORTER_ACTIVITY Catalysis of the transfer of a solute or solutes from one side of a membrane to the other according to the reaction: solute(out) + Na+(out) = solute(in) + Na+(in). SLC6A16 SLC6A2 SLC10A4 SLC6A6 SLC5A9 SLC6A8 SLC6A14 SLC20A1 SLC5A1 SLC32A1 SLC6A12 SLC6A4 SLC6A17 SLC5A4 SLC5A7 SLC23A1 SLC6A11 SLC6A13 SLC5A5 SLC6A15 SLC6A19 SLC10A2 SLC5A6 SLC1A3 SLC28A3 SLC13A3 SLC5A3 SLC6A5 SLC28A1 SLC10A3 SLC13A2 SLC6A7 SLC1A1 SLC1A7 SLC1A2 SLC23A2 SLC10A6 SLC13A5 SLC10A5 SLC5A2 SLC1A6 SLC6A9 SLC10A1 SLC6A1 SLC6A20 SLC38A1 SLC6A18 SLC28A2 SLC6A3

GO_UBIQUITIN_LIKE_PROTEIN_LIGASE_ACTIVITY Catalysis of the transfer of a ubiquitin-like protein (ULP) to a substrate protein via the reaction X-ULP + S --> X + S-ULP, where X is either an E2 or E3 enzyme, the X-ULP linkage is a thioester bond, and the S-ULP linkage is an isopeptide bond between the C-terminal glycine of ULP and the epsilon-amino group of lysine residues in the substrate. CUL1 TRAF6 RNF181 MARCH1 RFWD2 PJA1 TRIM24 CBLB RNF165 FBXO30 RNF14 UBE2J2 RNF149 ARIH2 C10orf46 UBE2B RNF122 ZNRF2 CBL UBE2G1 NHLRC1 VHL ZNF451 UBE2A MED1 UBE2H TRAIP RNF166 UBR3 MARCH8 PRPF19 ANAPC2 RNF145 BTRC UBE2Z RNF43 UBE2T RNF126 BRAP PPIL2 UBE4A NEURL3 RABGEF1 FANCL RNF144A CDC42 RNF44 PIAS2 UBR5 RNF185 RNF7 VHLL RNF114 ASB2 RNF25 RAG1 CBLC RNF19B NOSIP RNF111 UBE2J1 FANCF CHFR XIAP TRIM71 UBE2W UBE2C MED11 UBE2V2 SIAH1 RNF41 MED12 FBXO40 FBXW11 FBXL22 TMEM189 RNF121 TRIM32 ASB4 HLTF RNF125 RFFL FBXO6 TOPORS C18orf25 SMURF2 UHRF2 MED30 FBXO4 ITCH RNF220 TRIM72 WWP1 MED21 MDM4 SMURF1 HECW1 TMEM129 UBE4B AKTIP CUL2 RNF34 UBE2U RNF19A SKP2 RNF217 RNF115 RNF175 ZNRF3 KIAA1586 UBE2V1 RNF2 UBE2S RC3H1 RAD18 UBE2G2 WWP2 RNF24 PIAS1 UBE2F RNF216 CUL5 MARCH6 RNF128 PIAS4 UBR1 BFAR DZIP3 CUL4A SIAH2 LRSAM1 MDM2 TRIM25 RNF38 PELI1 RNF6 CUL3 UBE2K CBLL1 RNF146 MED7 RNF144B RNF4 RNF222 TRIM11 LTN1 CDC34 UBE2N MIB2 RNF13 MSL2 TRIM6 SIAH3 CUL4B UBOX5 UHRF1 AMFR RLIM TRIM37 MED17 ARIH1 RNF152 MED27 NEDD4 MED18 CCNB1IP1 ZNRF1 MED20 ZSWIM2 C19orf68 PELI2 RBBP6 UBE2NL PARK2 SYVN1 MED24 PJA2 RNF5 MED31 UBR2 UBE3A PDZRN3 NEURL RAG2 UBE2M ANKIB1 UBE2O ANAPC11 UBE2R2 RNF139 MYLIP MGRN1 RBX1 STUB1 TRIM3 UBE2I ZNF645 SKP1 MED10 RNF138 RNF180

GO_LIPASE_ACTIVITY Catalysis of the hydrolysis of a lipid or phospholipid. LIPI CASR PLCB3 SMPD3 PLA2G2A SMPD1 PLCE1 LGALS13 PLCG2 SEC23IP PLA2G12A MGLL RARRES3 FAAH LIPC LIPH ABHD2 ABHD5 LIPE NOTUM PNPLA7 PLA2G7 PNPLA5 PNPLA6 PLA2G5 PLA2G12B LYPLA1 PLCD4 PLD3 ENPP2 CCKBR PLA2G2E LIPA PLCL2 PNPLA4 AOAH PLA2G2F F2RL2 HMOX1 ABHD6 DDHD1 PLA2G3 SMPD4 C2orf43 PNLIPRP1 SMPDL3A OC90 NAPEPLD PLCH1 PLA2G15 PLCB1 PNLIP ABHD16A ABHD12 LIPG PLCB2 PLD1 PAFAH1B3 PLD4 PNLIPRP3 LIPK CCR1 ADORA1 AADAC PLA2G10 GDPD3 PLCG1 PLCB4 ASPG PLCD1 PLCL1 FAM83B PLCD3 PAFAH1B1 PLB1 DDHD2 PNLIPRP2 EDNRA SMPD2 PLD6 GDPD1 PLCZ1 BDKRB2 LYPLAL1 SMPDL3B PLA2G1B GPLD1 PLA2G4A DAGLA CEL PNPLA8 LIPM ABHD3 LIPN CHRM1 PLCH2 CLC CHRM5 PLA2G6 LIPF PDIA3 PNPLA2 LPL NCEH1 CCL5 PLD2 PLA2G4E PLA2G16 ENPP7 PLBD1 PLA2G4C PNPLA1 PLA2G2D CHRM3 PROCA1 PAFAH1B2 PLA2G4D CCR5 DAGLB PLA1A CES1 PLA2G4F PLA2G4B PNPLA3

GO_CALCIUM_DEPENDENT_PHOSPHOLIPID_BINDING Interacting selectively and non-covalently with phospholipids, a class of lipids containing phosphoric acid as a mono- or diester, in the presence of calcium. SYT10 CPNE1 C2CD4A C2CD4D ANXA8 ANXA2P2 TC2N ANXA6 CPNE3 SYT2 SYT14 SYT3 ANXA5 ANXA8L1 ANXA1 SYT1 ANXA7 PLA2G4B KIAA0528 SYTL2 SYTL1 ANXA2 SYT8 SYT12 ANXA4 DOC2B SYT5 SYT16 ANXA11 C2CD4C MCTP2 SYT11 SYTL5 DYSF C2CD4B ANXA3 SYT17 SYT6 ANXA13 ESYT2 SYT13 SYT14L PCLO ANXA10 RPH3AL ANXA9 SYT15 SYTL3 PLA2G4A SYTL4 SYT7 RPH3A SYT9 MCTP1 DOC2A SYT4

GO_ATPASE_ACTIVITY_COUPLED_TO_TRANSMEMBRANE_MOVEMENT_OF_IONS_PHOSPHORYLATIVE_MECHANISM Catalysis of the transfer of a solute or solutes from one side of a membrane to the other according to the reaction: ATP + H2O = ADP + phosphate, to directly drive the transport of ions across a membrane. The reaction is characterized by the transient formation of a high-energy aspartyl-phosphoryl-enzyme intermediate. ATP6V0D1 ATP1B3 PCYOX1 ATP1A1 ATP6V1C1 ATP1B1 ATP4B ATP1A3 ATP7B ATP2A1 ATP2A3 ATP6V1E2 ABCB11 ATP1B2 ATP1A2 ATP2A2 ATP2B3 ATP6V1G3 ATP2B4 ATP2B1 ATP2B2 ATP6V1E1 ABCC4 ATP2C2 ATP12A ATP2C1 FXYD2 ATP6V1C2 ATP6V1G1 ATP6V1G2 ATP1A4 ATP4A ATP7A

GO_HISTONE_LYSINE_N_METHYLTRANSFERASE_ACTIVITY Catalysis of the reaction: S-adenosyl-L-methionine + histone L-lysine = S-adenosyl-L-homocysteine + histone N6-methyl-L-lysine. The methylation of peptidyl-lysine in histones forms N6-methyl-L-lysine, N6,N6-dimethyl-L-lysine and N6,N6,N6-trimethyl-L-lysine derivatives. MECOM DPY30 CXXC1 SUV39H2 EZH1 MLL MLL4 SETMAR SETD7 SETD1A DOT1L EHMT2 SETD1B DYDC2 SMYD2 SETD3 EHMT1 PRDM16 SUV420H1 RBBP5 NSD1 SETD2 WDR5 ASH1L SETD8 PRDM7 MLL3 SUV39H1 PRDM6 SETDB2 WHSC1 MLL2 MLL5 SETDB1 SMYD1 WHSC1L1 PRDM2 MEN1 SMYD3 WDR82 SUV420H2 EZH2 DYDC1 ASH2L PRDM9

GO_AMINO_ACID_BINDING Interacting selectively and non-covalently with an amino acid, organic acids containing one or more amino substituents. NEDD4 NOS3 YWHAB SESN2 GLRB GNMT GRIN1 NAGS NOS1 GOT2 KARS SRR PIN1 TPH1 SHMT1 GCHFR GLRA3 GATSL2 AARS2 TAT GLUD1 AARS UBR1 GRIN2B ALAS2 GRIN3A GLRA2 DDC UBR2 TPH2 YWHAE GSS GATSL3 GLDC CEP104 SLC1A3 DDAH1 SHMT2 AGXT GLRA4 GRIN3B CAD PAH DDAH2 GLUD2 GLRA1 OTC RARS TDO2 NOS2 ASS1 DPYS LEO1 TH SESN1

GO_RAB_GUANYL_NUCLEOTIDE_EXCHANGE_FACTOR_ACTIVITY Stimulates the exchange of guanyl nucleotides associated with a GTPase of the Rab family. Under normal cellular physiological conditions, the concentration of GTP is higher than that of GDP, favoring the replacement of GDP by GTP in association with the GTPase. SBF2 DENND2C RABGEF1 DENND1C RAB3IL1 DENND5A RGP1 RIN2 DENND4A DENND3 DENND1B DENND1A DENND5B DENND2A ALS2 MADD RIN3 DENND4B KIAA1432 DENND4C RAB3GAP2 TRAPPC4 DENND2D ST5 FAM116A RAB3GAP1 RAB3IP SBF1 TRAPPC1 FAM116B

GO_TRANSCRIPTION_COACTIVATOR_ACTIVITY Interacting selectively and non-covalently with a activating transcription factor and also with the basal transcription machinery in order to increase the frequency, rate or extent of transcription. Cofactors generally do not bind the template nucleic acid, but rather mediate protein-protein interactions between activating transcription factors and the basal transcription machinery. CEBPA NKX2-2 MED17 TAF7 ESRRB TCERG1 SERTAD2 GTF2A1 KDM1A TAF6L VGLL2 SS18 RXRA MED31 CTBP2 YY1 ATXN7L3 NPM1 CITED1 NEUROG3 TRIP4 UBE3A NCOA1 TADA2A SOX4 ARID1A SLC30A9 C1orf85 RAN RAP2C GABPA E4F1 TFDP1 CCDC62 NR1H4 ACTN4 PPARGC1B GTF2F1 WWC1 CITED2 MLL5 ANKRD1 PIAS1 HCFC2 WWTR1 LPIN2 ACTL6B THRAP3 CD3D HAND2 SRCAP NFE2 ISL1 GTF2A2 PHF2 TAF1 UBE2L3 CTNNB1 ACTN2 MAK SOX10 TAF5L SOX11 RARA MED7 MKL1 MED14 SMARCC1 NCOA7 NR2C2 JMY PER2 ACTL6A MYSM1 USP16 GATA3 ING4 TFEC MED23 WBP2 SMARCD1 WNT3A GMEB1 SRA1 TFAP4 MED6 MYOCD SMARCA2 TAF11 NR2F1 USP21 PMF1 KDM5A POU3F1 MTF1 LPIN1 RFXAP TGFB1I1 ACTN1 MED12L PITX2 BCL10 NFKB2 PRIC285 TAF10 MED26 ZCCHC12 SMARCE1 RXRB SNW1 MAML1 KAT5 EDF1 ATF2 MKL2 POU1F1 PSMD9 TFAP2A KLF7 MAX HIPK2 MEF2A TCF3 SCAND1 JUP TAF7L PRPF6 ELF3 GATA4 PQBP1 YAF2 ESR2 NEUROD1 CIITA SUB1 SIX3 MED27 USP22 NCOA2 PPRC1 TBX20 SUPT7L MED20 ENY2 NFKBIB MED24 HMGA1 NFE2L3 CALCOCO1 PSMC3IP TAF4 MED16 TADA1 HYAL2 HINFP BIRC2 ZEB1 NPAT ZMIZ2 FGF2 NRIP1 RNF20 FHL2 PRRX1 ARID1B MTDH GTF2A1L TADA2B PRDM16 AIP SMARCD2 MAGED1 SMARCB1 CDK7 GMEB2 CREBBP EP300 ASXL1 SAP130 CBFB SP100 WWOX SOX17 SP4 ARNT SUPT3H DCAF6 RBPMS ZCCHC18 ZFX FHL5 JUND CARM1 IDAS TRIP11 DDX5 NR1I2 PSMC3 BUD31 TRIM28 PPARD MMS19 MYCBP CCAR1 NCOA4 ABT1 ATF6 PRKCB CITED4 RNF4 NCOA3 COPS5 YAP1 BRD7 HCFC1 SETD3 SMARCA4 TSG101 BCL9L SMARCD3 POU2AF1 TCF20 TADA3 NFYC UTF1 PRMT2 MED12 GRIP1 PARK7 TRERF1 RIPK3 NR1H3 TRIM32 ARL2BP PPARGC1A HTATIP2 RB1 ARID5B ECD SMARCC2 MED30 DTX1 KAT2A DDX17 JUNB NR5A1 KAT6A KAT2B MED21 NCOA6 MAML3 HAND1 TBPL1 NFATC4 RBM14 WDR77 ZFPM2 SFR1 MED4 TRIM24 TDRD3 HSF2 RNF14 SOX12 APEX1 BRCA1 PCBD1 FUS VGLL1 CCNE1 MED1 TFAP2B PPARG PKN1 JUN RERE PDLIM1 TAF9 MED13 MNT IL31RA PIAS2 BRDT NR1I3 MAML2 PHF17 POU3F2 MTA1 MYOD1 LPIN3 DYRK1B

GO_RETINOL_BINDING Interacting selectively and non-covalently with retinol, vitamin A1, 2,6,6-trimethyl-1-(9'-hydroxy-3',7'-dimethylnona-1',3',5',7'-tetraenyl)cyclohex-1-ene, one of the three components that makes up vitamin A. Retinol is an intermediate in the vision cycle and it also plays a role in growth and differentiation. RBP3 CRABP2 LRAT RLBP1 RBP1 RBP7 ADH7 RBP5 ADH4 CRABP1 RBP4 C8G RBP2

GO_RIBONUCLEOPROTEIN_COMPLEX_BINDING Interacting selectively and non-covalently with any complex of RNA and protein. SRPRB SECISBP2L C7orf30 EIF4H UHMK1 SECISBP2 BAG6 SNRPB2 CD2BP2 CPEB1 GCN1L1 EIF3K PQBP1 ERAL1 NME1 FMR1 ERI1 SEC61A2 RNASEL PRPF6 MTOR CPEB2 MTIF3 MTRF1L NAA10 SNRPD1 HSPA5 EEFSEC EIF2S1 EIF4A3 SNRPC NVL PRMT5 SNRPD3 MTIF2 SRP68 SEC61A1 SLFN14 EIF5A YTHDF1 ETF1 PRPF31 APOBEC1 EZH2 CPEB4 BOP1 LETM2 SRP72 ICT1 WDR12 RICTOR SRP54 PES1 LETM1 SNRNP70 PPIH ABCE1 DHX29 C1QBP EIF1AY SMG6 OLA1 NAA15 SRP9 CPEB3 SMG7 WIBG EIF5AL1 EIF2A TIMM50 RPSA IMPACT EFTUD1 ITCH IGHMBP2 PIM1 SBDS PHF6 NAA16 PPP1CA SNRPA C12orf65 NPM1 ZC3H12A RNF135 EIF6 DDX3X GTPBP6 IFIH1 EIF5A2 OXA1L GUF1 NMD3 LETMD1 MTRF1 EIF4B EEF2 HNRNPU RPN2 MRRF PTCD3 PRMT7 SRPR SMG5

GO_N_METHYLTRANSFERASE_ACTIVITY Catalysis of the transfer of a methyl group to the nitrogen atom of an acceptor molecule. ASH1L METTL15P1 WDR5 SETD2 PRMT2 NSD1 SETDB1 MLL2 IRF4 WHSC1 PRMT7 SETDB2 GNMT PRMT1 METTL16 SUV39H1 PEMT SMYD3 WHSC1L1 FDXACB1 PNMT PRMT3 TRMT11 RNMT PRMT8 CAMKMT C9orf156 SUV420H2 NNMT FAM86A SETMAR MLL TFB1M EZH1 METTL21C METTL10 SETD1B EHMT2 DOT1L HNMT SETD1A SETD7 SUV420H1 PRDM16 SMYD2 WDR77 RBBP5 FBLL1 PRMT6 METTL21A C5orf35 HEMK1 SETD8 PRDM7 SETD6 TRMT1L TFB2M MLL5 FBXO11 MLL3 PRDM6 RRNAD1 PRDM2 MEN1 SMYD1 DIMT1 ASH2L PRDM9 EZH2 DYDC1 WDR82 CARM1 MLL4 DPY30 CXXC1 SUV39H2 MECOM METTL15 PRMT5 N6AMT2 METTL21D SETD4 EHMT1 SETD3 TRMT1 METTL20 DYDC2 FBL

GO_CHEMOKINE_RECEPTOR_BINDING Interacting selectively and non-covalently with any chemokine receptor. YARS CCL1 CXCL10 PF4V1 CCL16 CCL18 CCL7 CCL3L3 JAK1 CCR2 CXCL3 XCL1 CCL4 XCL2 CCL5 PPBP CCL26 S100A14 CCL17 CCL8 ITCH STAT3 CCL3 CCL4L2 CCL24 CCL22 CNIH4 CXCL14 CXCL13 CCL21 CXCL6 CCL27 CXCL16 CCL28 CXCL1 CCL11 CCRL2 CXCL2 STAT1 CXCL12 CCL3L1 CXCL9 TFF2 CCL13 IL8 CCL23 CCL25 CXCL11 CXCL5 CCL15 CKLF CCL19 CCL14 DEFB1 CX3CL1 C5 CCL2 PF4 CCL20 DEFB4A

GO_BASIC_AMINO_ACID_TRANSMEMBRANE_TRANSPORTER_ACTIVITY Catalysis of the transfer of basic amino acids from one side of a membrane to the other. Basic amino acids have a pH above 7. SLC7A7 SLC38A7 SLC38A3 SLC7A1 SLC3A1 SLC38A1 TMEM44 SLC7A2 PQLC2 SLC7A3 C3orf55 SLC7A4

GO_LIGAND_DEPENDENT_NUCLEAR_RECEPTOR_BINDING Interacting selectively and non-covalently, in a ligand dependent manner, with a nuclear receptor protein. ARID1A SLC30A9 PROX1 UBA3 NCOA1 TRIM24 TADA3 NCOR1 NCOA2 SMARCE1 C1D PPARGC1A MED1 NR1H4 ISL1 TRIP4 NCOR2 NCOA3 JUND BAZ2A

GO_GLUTATHIONE_PEROXIDASE_ACTIVITY Catalysis of the reaction: 2 glutathione + hydrogen peroxide = oxidized glutathione + 2 H2O. GPX6 LTC4S GSTT1 MGST1 GSTM2 GSTK1 GPX8 GPX7 MGST3 CLIC2 ALOX5AP GPX2 GSTA1 GPX5 MGST2 PRDX6 GSTP1 GSTZ1 GPX1 GPX3 GPX4

GO_LOW_DENSITY_LIPOPROTEIN_RECEPTOR_ACTIVITY Combining with a low-density lipoprotein particle and delivering the low-density lipoprotein into the cell via endocytosis. OLR1 LRP8 STAB1 LRP1 LDLR LRP2 CXCL16 LRP10 LRP6 CD36 VLDLR LRP12 STAB2

GO_VOLTAGE_GATED_CHLORIDE_CHANNEL_ACTIVITY Enables the transmembrane transfer of a chloride ion by a voltage-gated channel. A voltage-gated channel is a channel whose open state is dependent on the voltage across the membrane in which it is embedded. CLCN2 CLCN7 CLCN6 CLCNKB CLCNKA CLCN1 CLCN5 CLCN3 BSND CLCN4 ANO6 ANO1

GO_LIGASE_REGULATOR_ACTIVITY Modulates the activity of a ligase. GLMN TRIB3 CAPN3 CDC20B UBE2L3 CDC20 TRIB1 RING1 CBX8 TRIB2 AUP1 FBXW7 FZR1

GO_INSULIN_LIKE_GROWTH_FACTOR_BINDING Interacting selectively and non-covalently with an insulin-like growth factor, any member of a group of polypeptides that are structurally homologous to insulin and share many of its biological activities, but are immunologically distinct from it. ITGA6 HTRA1 IGFBP5 NOV ITGB3 IGFBP6 HTRA3 CRIM1 HTRA4 ITGB4 IGFBPL1 IGFALS IGFBP1 IGF2R WISP1 IGFBP4 IGF1R IGFBP2 IGFBP3 ITGAV ESM1 CYR61 CTGF WISP3 WISP2 KAZALD1 IGFBP7 INSR

GO_GLUTAMATE_RECEPTOR_ACTIVITY Combining with glutamate and transmitting the signal from one side of the membrane to the other to initiate a change in cell activity. GRM4 GRM3 PTK2B GRIA2 GRIN3B GRM7 GRIA4 GRIN2A GRM1 GRIN1 GRIN2B GRIN3A GRM2 GRM5 GRIN2C GRIK2 GRID1 GRIK3 GRIK4 GRID2 GRM8 GRIK5 GRIN2D GRIA3 GRIA1 GRM6 GRIK1

GO_BETA_AMYLOID_BINDING Interacting selectively and non-covalently with beta-amyloid peptide/protein and/or its precursor. BACE1 ITM2B CHRNA7 SORL1 ARMCX5-GPRASP2 APBA2 ITM2A FZD5 TM2D1 ACHE APBB1 APBA1 BCHE COL25A1 DLGAP3 APBA3 BACE2 CLSTN1 PION APOE GPRASP2 IDE FBXO2 CD74 MAPK8IP2 APBB2 APBB3 LDLRAD3 CST3 ITM2C APOA1 TGFB2 LDLRAP1

GO_EXODEOXYRIBONUCLEASE_ACTIVITY Catalysis of the sequential cleavage of mononucleotides from a free 5' or 3' terminus of a DNA molecule. RAD9A TREX2 EXD2 POLD1 DCLRE1C DCLRE1A DEM1 ISG20 APEX1 RAD1 DCLRE1B C20orf72 APEX2 APTX EXO1 FEN1 C16orf73

GO_CARBOHYDRATE_TRANSPORTER_ACTIVITY Enables the directed movement of carbohydrate into, out of or within a cell, or between cells. SLC50A1 AQP5 SLC5A9 KIAA1919 SLC2A9 AQP7 SLC35A3 SLC2A7 AQP6 SLC2A10 SLC2A4 SLC5A1 SLC2A12 SLC45A4 SLC2A1 SLC45A2 SLC5A4 SLC2A11 AQP4 PPBP SLC45A3 SLC2A8 SLC2A3 SLC2A5 AQP1 SLC2A2 SLC2A14 SLC35A1 AQP3 SLC2A13 SLC35A4 SLC35A2 SLC35A5 MFSD4 MIP SLC17A5 M6PR SLC5A2 AQP10 AQP9 AQP7P3 AQP8 SLC2A6 AQP2

GO_MODIFIED_AMINO_ACID_BINDING Interacting selectively and non-covalently with a modified amino acid. ANXA9 MTHFR RASGRP1 TYMS MTHFS CPS1 SYT9 GSTM1 SYT4 MMACHC SYT7 MARK1 SLC19A1 GNMT CPNE6 GSTM4 SYT5 SDPR FOLR3 AXL HMGB1 DPEP1 GSDMD OSBPL8 HSPA8 FTCD GSTM3 SYT1 GSTM2 TIMD4 FOLR4 FONG MFGE8 SYT10 RS1 SLC46A1 FOLR1 FCHO2 TRIM72 PTGES2 RPE65 PTGES SCIN GAP43 DHFR OSBPL5 MUT DHFRP1 SYT6 FASN GAS6 MGST1 PLCD1 SYTL2 JPH2 CPNE1 SCARB1 GSS CD300A LANCL1 SESTD1 GSTP1 OSBPL10 FOLR2 NOX4 CBS GPR143 THBS1

GO_TRANSCRIPTIONAL_REPRESSOR_ACTIVITY_RNA_POLYMERASE_II_TRANSCRIPTION_REGULATORY_REGION_SEQUENCE_SPECIFIC_BINDING Interacting selectively and non-covalently with a sequence of DNA that is in the regulatory region for RNA polymerase II (RNAP II) in order to stop, prevent, or reduce the frequency, rate or extent of transcription from an RNA polymerase II promoter. ZFPM2 NR2C1 BHLHE40 DMBX1 FOXD3 BACH2 PPARA ZNF280C ZBTB16 TBX3 TCF21 IFI16 FERD3L ETV3 PCGF6 ASCL1 PRDM5 DLX4 GATA1 SNAI2 AEBP1 MSX1 CDX2 KLF16 CUX2 GZF1 GLIS1 INSM1 TFAP2A TBX15 NKX6-1 FEZF2 RCOR1 MZF1 SREBF2 TCF3 ZBTB7A ZNF217 ZNF746 MNT ISX ASCL2 CC2D1A FEZF1 ZFPM1 PURB SKOR2 HHEX VAX2 GATA3 HES6 HSF1 ZNF280D POU4F2 ZC3H8 NR1D1 MYPOP REST POU5F1 FOXO3 HOXA2 BACH1 MXD1 MSC PAX6 PROX2 RELA SCRT2 ESX1 BCL11A TCF7 ARID5B CSDA C2orf3 ZNF350 IRF3 GCFC1 ZFP90 ASCL3 BTG2 SP3 HAND1 NFATC4 PROP1 CREBBP ZBTB4 TSHZ2 SNAI1 PCBP3 SNAI3 ETS2 NFXL1 HIVEP1 TGIF1 NFATC3 OVOL1 PAX4 PRDM1 E2F7 HOXD9 ETV6 ZGPAT KLF12 ARHGAP35 ETV3L MLX CTCF PPARD TSHZ1 SALL1 PROX1 ZEB2 BATF3 NFKB1 NFIL3 ZNF536 ZNF280B MITF ZNF219 FOXO1 BCL6B SKI ESRRA ATF3 FOXP2 TSHZ3 MLXIPL KCNIP3 EN1 JARID2 BCL6 HMGA2 ETV7 HMX1 ARX NR2E1 SKOR1 NR2F6 CC2D1B BHLHE41 ZBTB20 NACC2 VAX1 NFE2L3 YY1 ZNF281 DACH1 SATB1 NEUROG3 KLF17 ZNF280A HES1 JDP2 HINFP TBX2 MSX2 AEBP2 ZEB1 CHCHD3 NFX1 SPI1 E2F8 SKIL HELT E4F1 ZKSCAN3 CGGBP1 ZNF148 NKX6-2 HES5 TFAP2C GFI1 NFATC2 NKX3-2

GO_PHOSPHATIDYLINOSITOL_MONOPHOSPHATE_PHOSPHATASE_ACTIVITY Catalysis of the reaction: phosphatidylinositol monophosphate + H2O = phosphatidylinositol + phosphate. FIG4 SYNJ2 SACM1L MTMR4 MTMR1 PTEN MTMR3 SYNJ1 MTMR7 MTM1 MTMR6 MTMR8 MTMR14 MTMR2

GO_POLY_A_BINDING Interacting selectively and non-covalently with a sequence of adenylyl residues in an RNA molecule, such as the poly(A) tail, a sequence of adenylyl residues at the 3' end of eukaryotic mRNA. SYNCRIP ZC3H14 TIA1 PABPC4 PABPC3 KHDRBS2 PABPC1 DDX3X RBPMS DDX1 KHDRBS1 EIF4A3

GO_BITTER_TASTE_RECEPTOR_ACTIVITY Combining with soluble bitter compounds to initiate a change in cell activity. These receptors are responsible for the sense of bitter taste. TAS2R7 TAS2R5 TAS2R4 TAS2R43 TAS2R31 TAS2R45 TAS2R8 TAS2R40 TAS2R39 TAS2R50 TAS2R10 TAS2R16 TAS2R41 TAS2R20 TAS2R14 TAS2R19 TAS2R42 TAS2R13 TAS2R3 TAS2R1 TAS2R9 TAS2R46 TAS2R60 TAS2R38 TAS2R30

GO_REPRESSING_TRANSCRIPTION_FACTOR_BINDING Interacting selectively and non-covalently with a transcription repressor, any protein whose activity is required to prevent or downregulate transcription. RBPJ STAT3 NFYB SP1 MKKS CBX5 GTF2A2 GATA6 DMAP1 CTNNB1 BBS2 TCF3 GMNN TTC8 CTBP1 BBS10 HDAC2 GSC HHEX EIF4E MIXL1 SKIL HDAC7 MTA2 CHD4 MTA1 ZNF703 SKOR2 SKI ARNTL PPARA TBP TCERG1 HMGB1 MAGEA2 DDX20 TCP10L TLE4 MYC HDAC9 BBS7 ZMYND8 RUNX2 SRI HDAC1 RELA BBS1 HDAC4 RBBP8 SKOR1 PARK7 NOC2L KAT5 MAGEA2B TCF7L2 SIN3A HDAC5 BBS5 PRDM5 BBS4

GO_MACROMOLECULAR_COMPLEX_BINDING Interacting selectively and non-covalently with any macromolecular complex. ZNF274 ADAM9 MYO7A CCDC42B MYL4 PIM1 KIAA0101 HLA-DMA PRMT6 PLAC8 LEPRE1 IMPACT RCC1 NCOA6 ATRX GNB4 GLI3 SP3 AMICA1 ADAM2 RPN2 TNNC2 POU4F2 KIAA1967 ORC1 MTM1 MED12 PCNA CETN1 HDGFRP2 CD3G STRN3 POLE MBD2 ZC3H12A RPH3A PYGO2 WHAMM ARPC2 FEZF2 CD4 GMFG SMAD2 ERCC6 ADD2 LAMB3 SMAD3 CD44 DOK2 COMP MYOD1 LRRK2 LRP1 HIST3H3 ASF1A GNAI2 MRC2 HNRNPD CORO1C NONO TRPV4 TEX10 BRD3 H3F3C HCLS1 NR0B2 MORF4L1 SIX1 H2AFY IFT74 SYNE1 BRK1 SLFN14 GATA1 MADCAM1 PKN1 FGR CKS2 CD2AP HINFP LRRC15 TGFBR3 GPR56 IFNE CD300LG TSPAN8 FLI1 EZH1 KRAS GNAI3 EIF5AL1 ARPC1A THY1 ST13 SMG6 SHANK1 ARID3A GRB10 ATR SMARCB1 FCER2 MRRF CDC20B MB21D2 ESPN WISP3 SPTB HIST1H1D SMG5 NCOA2 TRIM37 ECM2 PRMT7 BMI1 PAFAH1B1 FAP KDM6A PCF11 SPTA1 DNM1 VPS28 HDAC5 UCHL5 RIPK1 SVIL VAX1 CTNNA3 RNF135 ZIC2 ACTN3 CHADL NCAPD3 SCARB1 CENPA PER1 ARPC4 PTCH1 PCSK2 JAM3 SKI RDBP FZR1 ATF5 SECISBP2 ILK EGFL6 JARID2 HCFC1 IGF2 CORO2A LIPC ANKRD17 AR PKP1 DNAJC2 VIM SLC27A5 BOP1 IFNA21 CAPZB DEK TAPBPL EHMT2 HSP90AA1 RPS24 C12orf65 CTSL1 MEIS1 STAB2 SFPQ KRT14 H3F3A ACVR2B KLRC1 KDM5A RAB32 SRC ACTN1 ADRM1 C12orf44 SORL1 ADAM15 PHF12 GATA3 M6PR HSF1 FBN1 NSD1 GNA13 MYO16 EIF4B SATB2 HOXD13 SIN3A CCDC37 ITGA3 TLN1 NKX6-1 SORBS1 SNRPC PHF1 MEF2A FAM21C H3F3B PLS1 TP63 PPP1CC MBD1 APOA2 NES C8A FSCN3 RPRD1B TULP2 ABI3BP WASF1 GATA5 CDC20 RAP1A ZNF304 SMARCE1 HDAC1 PHF21A GMNC CFLAR RCSD1 SNX2 HTR2A ARPC3 SNX9 SEMA7A ZNHIT1 LYN ZNF683 ATF2 SOX14 SNAI2 CTNNAL1 POU1F1 BAP1 HMGN4 CD9 PSMD14 ATAD2 SETD1A SUPT5H SLC30A9 COLEC12 PRKAA1 JUB HESX1 POLR2B HDAC2 ACTN4 FCAR SMYD2 RRAS PEX26 FOXC2 ADAM10 E2F4 SETDB1 GPNMB MUM1 CTR9 IQGAP1 GMFB SCARF1 IFNA7 NMD3 ERCC8 WIPF2 LRP8 ITGA11 NPM1 APOL5 NID1 RECQL5 CDK5RAP1 SETP18 NME2 CTNNB1 CYFIP1 GPR126 GNB2 CTSS DHX30 THBS1 MLC1 ACVR1B GNAI1 NAA10 WDR1 RARA ITGB1BP2 FBLN1 ARID3C HIST2H3D VLDLR ITGA1 ERG GP6 PPP2R2A RING1 UHMK1 RNF2 TUBB RAD18 TMEM201 ABCE1 GNA11 ARRB2 NDEL1 RAD21L1 TMOD4 NOC3L PXN NDUFA8 LIMA1 CASP8 ISL1 FANCM NSF FAM178A CRP SMARCC2 YWHAE IST1 KAT6B DDR2 KAT2A CSNK2B RNF40 ANKRD54 POLD1 MLL EPS8L1 KAT2B RBMX APOL2 IKBKAP CALR BARX2 C14orf49 HDAC6 NCK2 FSCN2 AUTS2 DPPA2 HR BAG3 LHX2 LDLR IFNA16 CTNNA2 GNAS CALY SLC25A3 CENPH EIF5A2 NPNT IQGAP2 CTSB CORO2B THRA MYOG NID2 PRDM14 PDCL ULK1 SPARCL1 PI4K2A MMP9 IFNW1 SRPRB GIT2 ACTB NUP62 NKX2-5 EED UXT RAD51 TRIM24 ING2 NFIA HMGN2 WISP1 ABP1 DOK1 CDC45 SIX2 TRADD P4HB EZH2 GRHL3 NOC2L PDZK1 WDR82 MEN1 IFNK ICAM3 EZR PRPF31 GATA6 PHF8 RLTPR AP2A1 TXN2 KDM6B EFTUD1 SIRT2 HIST1H3C GLI2 HMBOX1 NAA15 CREB3L1 NPM2 CEBPB SMARCD2 FRG1 FLCN IFNA4 CETN2 CHAF1B CDC73 MEIS3 SOX15 FGF1 MYH10 SAFB UBD MS4A1 KRIT1 CD74 ITGA9 PSMG1 UPF1 LTBP4 APOE MBD5 OVOL2 HSPA5 XRCC5 CPEB2 TPR ITGAL RNASEL DEPDC5 AZI1 RPAIN C1QTNF1 COCH RNF4 BAZ1B YAP1 GNAT3 FLNA SCAP HSPD1 SMARCA4 PROP1 FN1 LRPPRC DAG1 REC8 LASP1 JMJD5 ELP2 TP53 H1FOO SCIN TERF2 IRS2 PBRM1 CD3E SMARCA2 VRK1 DNMT1 EGFR SNRPA MYH8 ENPP1 MYO1B CENPB ADAM17 GNAO1 PBX2 CPEB3 ITGA2 ELP4 PICK1 MYO6 CUL2 MYO1D CBX4 PPP1R9B CLSPN ANKRD2 DSTN PGAM5 SHROOM2 ANXA11 TOP1 DMRT1 TRPS1 ACVR2A ITGA2B MYH14 SMARCD1 DISC1 GNA12 GPIHBP1 CCNT1 EEFSEC CCNT2 STAT3 DAB2IP FOS TWF1 NRAS ASPN GATA4 CD2BP2 NUPR1 NCOA5 PLDN CACNB2 ZMYND11 ACVRL1 HDAC3 PCOLCE2 HSD17B12 PELP1 CIITA BEND6 EIF4H USP14 CTGF GNAZ HIST2H3C HLA-DOB IGF1R RGS9 HLA-DRA CDK9 APOBEC1 ICAM1 SIRT7 AAK1 SRP68 EIF2C1 DLX2 NCOA1 SETD7 HIST1H3E CHAF1A IGHMBP2 ARID1A EPHB1 RAN EOMES UQCRC1 SMARCA5 CFL2 ARPC1B CORO1A HIST1H3G MYO1A KDM3A ANXA7 NFATC2 AMFR HMGA2 PIK3R1 KHDRBS1 WHSC1 MAPK8IP2 HIST1H3B ERMN REL PMS2 FCGR2A TAL1 LRRC16A CRIPT FKBP1A GUF1 HSP90AB1 LZTFL1 CTBP2 C19orf2 SACS THRB IFNA5 KIAA1530 NEUROG1 ANXA8 PLS3 C8B CDCA5 SOX10 NRG1 SHARPIN SMARCC1 HIST1H1A NME1 MCMBP NEXN FOXO1 HIST2H3A NR5A2 NDUFA9 CITED2 DDX1 CFL1 GNA15 TUB USP3 TULP3 HNRNPC MSR1 ACTR3 PRDM1 RBL1 HMGN3 ADM2 CPEB4 LOC389493 SIN3B POLG CENPF HIST1H3J WDR13 VDAC1 ELOF1 COL3A1 ACTR2 NR5A1 GFAP SSRP1 NIPBL NAP1L2 DOK4 SMG7 DSPP HP1BP3 RPSA VSX1 MPO TTC5 ADRA2A ATAD2B IFNA10 SMARCD3 MYH9 STAT5B DOK3 EIF2C2 MTRF1 ATF1 DDX3X SNX4 OXA1L MTA3 SPARC INSL3 PPARGC1A SMC1A TBC1D5 TULP1 RERE TLN2 MYH3 PIK3R5 VCAM1 MUTYH SNRPD3 FSTL3 RAB38 NFYB MTIF3 NUCKS1 SMC3 PHF13 BECN1P1 KDM3B CCDC99 ZNF431 EGR1 UQCRFS1 HMGN5 VHLL TGFBI SNRPB2 SUZ12 LETM1 APOA1 CDKN1B COL16A1 IFNA1 SMARCA1 SLC6A3 KLRD1 PTPRF APEX1 BIN1 KRT74 ASH2L CDK1 ESR1 TFAP2B MED1 PPARG JUN GATAD2B BRD2 CLASP1 ABL1 ABI2 EVPLL FCGR2C ICAM5 CD151 RAD21 RNF20 KDR PKM2 RBX1 ANKRD32 H2AFZ RUVBL2 AP1AR NR3C1 PTPN2 LAMA5 HLA-DMB ITGA5 UHRF1 RPTOR SPTBN1 SOX9 HOXD10 PATZ1 MYO18A SRPR PTK2B KCTD17 CBX1 USH2A ENY2 SKOR1 TNN ADNP S1PR2 FOXN4 ADAMTS13 NKAP MYO10 MLH1 LACRT PAK1 CAPG SYK SMAD7 MTRF1L SHROOM3 RAB7A GCN1L1 HIST1H3I TSHZ3 SECISBP2L RBM10 TAB1 HIST1H3F POLQ CREBBP CYR61 ENG POLR3A LDLRAP1 TIMP2 DLG4 LMBRD1 FMNL1 CENPE MMP14 SEC61A1 APBB1 ICAM2 PRDM13 WAC ADAM22 ELP3 WIPF1 XRCC6 ADSSL1 MFGE8 NAA16 MEOX1 SMURF1 H2AFY2 EIF2A DOK5 CHD4 KRT8 HDAC7 FCGRT CORO6 PDGFA KLHL17 ITGA10 OLA1 SHROOM1 DOK7 PPAP2B MYSM1 DOCK2 KLHDC3 CAV3 KIAA1598 RBL2 REST VPS16 YBX1 EEF2 CD36 WBP2 GTPBP6 CBX7 CDH13 VIL1 CCND1 CPS1 EVPL ACHE PTPN1 CD226 NVL WISP2 HIST1H3H TCEB1 SKAP1 SLC34A1 CDK4 CASP3 COL14A1 GIT1 LDB1 ABI1 EIF3K SKOR2 ARPC5 RFX4 LGALS3 BAG6 CD81 SSBP1 MICALL2 ANGPTL3 RNF169 DHX29 RNF8 PPARA RICTOR COL4A3 COL5A1 HLA-DOA ITGB1BP1 C19orf40 MLH3 PAF1 LETM2 LCA5 ADAM11 MSH6 HLA-DRB1 MAX TFAP2A PPP1CA PFKP PEX6 TADA2A ICAM4 MSL1 KRT19 APITD1 LEF1 TOX3 ERBB2IP GPER MACF1 CIB2 BCAP31 MSH2 UFD1L BCL6 DST FAM83H EMP2 CHD7 NKX2-2 GNAQ ASH1L TAF2 MCM5 RELL2 ARX KDM1A HNRNPU RXRA STRN HMGN1 KIF11 PODN CITED1 LAMB2 APTX ZGLP1 SRF PRMT5 EIF2S1 EIF4A3 ACTN2 IFNB1 ABL2 FAM123B SNRPD1 WIPF3 CPEB1 ITGB1 PLK2 AP2B1 NISCH PIP C7orf30 FCHO1 C14orf166 IFNA13 ID1 PDPK1 SRP54 TSHZ2 PES1 DBNL MAP1S TELO2 HADHA ICT1 USH1C PEX1 ELK1 UQCRC2 CRTC2 NEBL LRWD1 ADAMTS5 MTIF2 CHD8 FCGR1A YTHDF1 AIF1L FCGR3B EPCAM EBF2 C16orf73 MAPT ITCH USP13 KLRC2 CRAMP1L AIF1 KCTD5 DLX1 CTSL2 COG2 FLNC SRP9 TNRC18 S1PR3 CORT PCGF2 TNNC1 LUM EIF6 MTHFR MEF2C RIPK3 SERPINH1 CABIN1 ELK4 IRS1 RELA GATA2 L3MBTL1 MBD6 RBPJ JMJD1C MNT ACD ANTXR1 ITGB6 MTA1 FMR1 ERI1 ANKRA2 GNAT1 SIRT6 DCN CTSK FCGR1B TDRD3 SNRNP70 POLA1 FCGR3A UTRN PHIP ASCL1 PRKAA2 IGJ CBX2 HNF1B FOSL2 FOXO4 CLOCK MBD3 HDAC4 ATP6V1B1 MMP13 AMBP ATP6V0D1 DDR1 LOXL2 IFNA17 FCER1A ZEB1 FRS2 SFRP2 HOXC13 PCOLCE SVEP1 RBPJL CBX5 RANBP2 WIBG CIC RBBP4 FABP1 SSFA2 TIMM50 HRG EIF1AY TADA2B C1QBP PDGFB PCSK9 PPP1R9A FCER1G PTEN NCKAP1L ACVR1 FCGR1C PKNOX2 PDX1 ADD1 LETMD1 IFNA14 HIST1H1B VCX CCDC155 CAMTA2 CCBE1 SNX1 HMGA1 CALCOCO1 SMYD3 ESM1 NDUFA4 RELB EXO1 TRIM28 TSHZ1 WASF2 MECP2 SHMT2 WRN NCKAP1 PRKCB GLI1 BCAS3 NCAPD2 MYH11 SLC6A4 GTF2H1 ATOH1 EP300 TFAM ATM CCDC111 GNAL RUNX2 CX3CL1 PPIH HIST1H1E IFNA2 WDR12 THBS4 NRL PSMF1 DNMT3B PANX1 MS4A2 PKP2 TTN RARB TOP2A INS SBDS FADD ATP1B1 VCP SYNE2 RAP1B H1F0 TNXB PITX2 NFKB2 ACTL6A VAX2 PPIB SUV39H1 SMAD4 DNMT3A FOXO3 IFIH1 BAHD1 PAX6 SIRT1 TP73 UBE2T ATXN7 NFKBIA SLC9A3R1 PRPF6 SEC61A2 EDIL3 GRHL1 ERAL1 PQBP1 MLKL CRTAP VWF FCGR2B EGR2 TMOD1 PLK1 CTNNA1 DOK6 STAB1 ADORA1 SRGN BAHCC1 DNTTIP1 NEUROD1 HHEX IGBP1 YWHAB CDH17 C8G SRP72 IRS4 KCTD2 WRAP53 TSPAN4 EIF5A GNB1 MSGN1 TRAF2 INSM1 RNF168 VTN LCP1 IGF1 C6orf15 RAG2 JDP2 ARRB1 BRD4 PHF6 ITGB2 FERMT3 SKIL FYN GABPA EP400 ZKSCAN3 HSPA8 HES5 NOTCH1 STRN4 TSN ZMYND8 COL5A3 PTCD3 HIST1H3D MYO1E CXADR CORO1B AIRE SHC1 GNAT2 MYC TLE4 FSCN1 SGTA POLR3D HIST1H3A TRIOBP ADAMTS8 SATB1 NEUROG3 IFNA6 GRHL2 TOP2B ADAM23 PHF21B CTCF FYB C15orf42 DLX3 SHROOM4 FBLN5 MTOR SYNM NFKB1 PTPN11 SREBF1 DMP1 DDX39B HIST1H1C ZNF326 MTA2 TMED10 GNA14 ING5 MARCKS PTF1A FST DNM2 FRS3 UMOD IFNA8 STAG1 CDKN1A TTF1 SMAD6 ITGA6 FLT3 NOV ETF1

GO_DOPAMINE_RECEPTOR_BINDING Interacting selectively and non-covalently with a dopamine receptor. ATP1A3 GNA12 SLC9A3R1 DRD3 ARRB2 DLG4 VPS35 PALM PTPN11 GNA13 GNAS DNM1 CLIC6 DNM2 PPP1R1B

GO_ATPASE_COUPLED_ANION_TRANSMEMBRANE_TRANSPORTER_ACTIVITY Catalysis of the transfer of a solute or solutes from one side of a membrane to the other according to the reaction: ATP + H2O + anion(out) = ADP + phosphate + anion(in). ABCC3 ABCC9 ABCC1 ABCC5 ABCC2 ABCC4 ABCC10 ABCC6 ABCC8 CFTR ABCC11

GO_POTASSIUM_TRANSPORTING_ATPASE_ACTIVITY Catalysis of the transfer of a solute or solutes from one side of a membrane to the other according to the reaction: ATP + H2O + K+(out) = ADP + phosphate + K+(in). ATP4B ATP1A2 ATP1A1 ATP1B1 ATP12A ATP1B2 ATP4A ATP1A4 ATP1B3 ATP1A3 FXYD2

GO_LONG_CHAIN_FATTY_ACID_COA_LIGASE_ACTIVITY Catalysis of the reaction: ATP + a long-chain carboxylic acid + CoA = AMP + diphosphate + an acyl-CoA; a long-chain fatty acid is a fatty acid with a chain length between C13 and C22. ACSBG1 ACSL5 ACSL1 ACSL3 SLC27A6 SLC27A3 ACSBG2 SLC27A1 SLC27A2 SLC27A5 SLC27A4 ACSL4 ACSL6

GO_PHOSPHATIDYLINOSITOL_BISPHOSPHATE_BINDING Interacting selectively and non-covalently with phosphatidylinositol bisphosphate. MARK1 LDLRAP1 TIRAP SYT5 PFN2 TULP3 SNX18 SLC9A1 ALOX15 SNX20 KRIT1 SCIN RPH3A SYT9 STXBP6 VIL1 COMMD1 SYT7 RAG2 KCNQ1 ANXA8 PFN1 PARD3 ACTN2 PHLDA3 TULP1 SYT10 RS1 TWF2 KCNJ2 KCNJ1 SESTD1 FRMPD4 FAM21C EXOC1 FCHO2 TWF1 MYO1B FAM123B GSDMD MTSS1L FAM123C FAM123A PLCB1 TTPA MYO1G MAPKAP1 SYT1 SYTL2 ANXA2 SNX21 RAB35 JPH2 PIRT ADAP2 ASAP1

GO_GABA_RECEPTOR_ACTIVITY Combining with gamma-aminobutyric acid (GABA), and transmitting the signal from one side of the membrane to the other to initiate a change in cell activity. (GABA, 4-aminobutyrate) is an amino acid which acts as a neurotransmitter in some organisms. GABRG3 GPR156 GABRB3 GABRP GABRG2 GABRR3 GABRE GABRD GABRA1 GABRQ GABBR1 GABRR1 GABRA2 GABRR2 GABRA6 GABRA5 GABRA4 GABRB2 GABBR2 GABRA3 GABRG1 GABRB1

GO_OXIDOREDUCTASE_ACTIVITY_ACTING_ON_A_SULFUR_GROUP_OF_DONORS_DISULFIDE_AS_ACCEPTOR Catalysis of an oxidation-reduction (redox) reaction in which a sulfur-containing group acts as a hydrogen or electron donor and reduces disulfide. TXNDC8 TXN2 TXNDC12 ERO1L DNAJC10 SEPX1 TXNDC2 MSRB2 TXNL1 MSRA MSRB3 ERO1LB TXN

GO_HYDROLASE_ACTIVITY_HYDROLYZING_O_GLYCOSYL_COMPOUNDS Catalysis of the hydrolysis of any O-glycosyl bond. PARG OTOG GLB1L LYZL6 GANAB LCT MANEA HEXB GLB1 NEU3 KL HPSE NEU2 AMY1C MGAM ACER3 NAGLU MAN1C1 MAN1A1 ENGASE MAN2B1 MGEA5 LYZL2 LYZ GANC HEXDC SPACA5 AMY1A GLB1L2 KLB KIAA2018 CHIA EDEM3 NAGPA LYZL1 SPACA3 KIAA1161 CHID1 GBA3 NEU1 MAN2C1 GBE1 MAN2B2 SPAM1 GUSB HYAL2 OVGP1 FUCA2 LALBA HYAL4 MANBA GLA CHIT1 ABHD10 ADPRHL2 CTSA GBA2 EDEM2 HYAL1 EDEM1 HPSE2 ACER2 OTOGL NEU4 GALC SPACA5B SI AMY1B CTBS AMY2B TREH CHI3L1 ACER1 LYG2 AMY2A IDUA LCTL LYG1 HEXA GAA HYAL3 AGL GBA MAN2A2 MAN2A1 GM2A MOGS MAN1A2 GLB1L3 MAN1B1 NAGA CHI3L2 FUCA1 GNE KIAA1199 LYZL4

GO_PROTEIN_TYROSINE_KINASE_ACTIVATOR_ACTIVITY Increases the activity of a protein tyrosine kinase, an enzyme which phosphorylates a tyrosyl phenolic group on a protein. CCL5 FAM150A ERCC6 EGF GREM1 CD24 EFNA5 PAK2 AFAP1L2 GHRL NRG3 GAS6 NRG1 ERBB3 ABI1 ANGPT4 FAM150B

GO_PHOSPHOLIPASE_A2_ACTIVITY Catalysis of the reaction: phosphatidylcholine + H2O = 1-acylglycerophosphocholine + a carboxylate. ABHD3 PNPLA3 PLA2G4B PLA2G2E PLA2G4F PNPLA8 PLA2G10 PLA2G12B PLA2G4A PLA2G1B PLA2G5 PROCA1 PLA2G4D PAFAH1B3 PAFAH1B2 PLA2G2D PLA2G7 PLA2G4C PLBD1 PLA2G16 PLA2G15 PLA2G4E OC90 RARRES3 PLA2G3 PLB1 PLA2G12A PAFAH1B1 PLA2G6 PLA2G2F PLA2G2A

GO_RNA_POLYMERASE_CORE_ENZYME_BINDING Interacting selectively and non-covalently with an RNA polymerase core enzyme, containing a specific subunit composition defined as the core enzyme. RECQL5 ERBB2 KIAA1530 ELOF1 CDC73 BRD4 CTR9 KIAA1967 EIF2C2 SCAF8 PCF11 SPTY2D1 ZNF326 PAF1 ELP4 IKBKAP MAF1 ELP2 RPRD1B C14orf166 SMYD3 WAC EIF2C1 ELP3 SMYD2

GO_GLUCOSE_BINDING Interacting selectively and non-covalently with the D- or L-enantiomer of glucose. HK3 HKDC1 GCK PYGL G6PD HK2 HK1 SLC2A8 UGP2 SLC2A3 GYS1

GO_CORE_PROMOTER_PROXIMAL_REGION_DNA_BINDING Interacting selectively and non-covalently with a region of DNA that is in cis with and relatively close to the core promoter. The transcribed region might be described as a gene, cistron, or operon. SOX10 TFE3 ZNF66P BATF3 ZNF195 PLAG1 HOXC11 CXXC1 SREBF1 ZNF56 OTX1 SALL1 SRF CTCF KCNIP3 NR5A2 EN1 DMRT3 ZNF876P MTA2 ZNF492 ESRRA DMRTC1B CEBPZ SMARCC1 ELK1 NFATC3 HNRNPC ZNF479 ZGPAT ZNF713 DMRTC1 EHF CREB3L2 ISL1 OVOL1 PRDM1 SMAD6 ONECUT2 RFX5 ELK3 ZNF502 KLF17 RFX2 MUC1 ZNF708 RFX8 JDP2 ZNF114 TBX2 NFATC2 BCL11B HDAC2 NFIB GABPA NRF1 HELT SKIL SPI1 LEF1 NKX6-2 ZKSCAN3 ZNF736 SIX5 MYC CHD7 E2F4 CEBPA CTCFL ZNF519 NKX2-2 ZNF281 NEUROG3 RFX6 HOXA5 POU2F3 BHLHE41 NACC2 FOS FOSL1 ELF3 MEIS2 STAT3 ZNF746 GRHL1 ZNF138 NKX6-1 FOXA2 TCF3 ZBTB7A ZNF217 MEF2A HHEX NEUROD1 TEAD1 ZNF737 CC2D1A EGR2 ASCL2 RFX4 SKOR2 AKNA FOSB PPARA IFI16 NR4A3 ZIC1 NR2C1 ZNF90 MEF2D UBTF HDAC1 SMARCE1 ZNF695 EIF2C1 ZNF714 POU1F1 ZNF85 TBX15 INSM1 MAX TFAP2A GZF1 KLF16 TXK GSX1 FOXK2 DLX4 ZNF516 ZNF682 ETV1 MEOX1 IRF3 MTF1 MEIS1 C2orf3 PITX2 CDX4 NFKB2 ATF4 MIXL1 SIX4 CEBPD CHD4 DDN CEBPE ZNF253 PITX1 T RFX1 MYBL2 PIH1D1 NR1D1 MYPOP YBX1 FOXJ1 ACTL6A IRF4 HSF1 DMRT1 SMAD4 GATA3 MESP1 BCL11A ZNF626 GMEB1 SOX18 STAT1 TP73 POU2F2 TFAP4 ZNF679 ZNF98 ZNF680 MEOX2 NKX2-8 IRF1 ZNF676 RFX7 PAX6 ZNF331 HOXD13 PER1 KLF5 ZNF639 HOXA10 TFAP2E ZNF805 ATF3 HOXA7 FOXP2 PHOX2B SMARCA4 ATOH1 MLXIPL MITF TCF12 ZNF664 ZNF536 GLI1 SKI ZNF219 NFIC ETS2 ZNF675 GCM1 MAFB ZNF562 TGIF1 FOXL2 ZNF716 AR POU4F3 NR6A1 TFAM GMEB2 CREBBP SNAI3 ZNF430 ZNF506 RUNX2 CRX ETV6 RFX3 MYF5 KLF1 ZNF718 ARNT2 NPAS4 NRL JUND NEUROD6 RBPJL FLI1 ETV2 DMRTB1 AEBP2 HINFP ZNF264 DMRTA2 MYBL1 DPF2 H2AFZ NKX3-2 SMARCD2 SMARCB1 CEBPB NR3C1 E2F8 GLI2 PAX2 FUBP3 NDN MYB ZNF148 RBBP4 PITX3 TBX20 PDX1 ZNF730 TBP BATF2 KDM6A UHRF1 ELF1 NCOA2 DMRT2 PATZ1 NFE2L3 ZNF732 FOXI1 PAX8 SKOR1 KLF13 CC2D1B NOBOX RBPJ SMAD3 ZNF395 SP1 SMAD2 FEZF2 SOX1 SREBF2 DMRTC2 MZF1 HNRNPK FOXD1 NKX2-5 ZNF100 ACTB FEZF1 ZNF431 ZNF141 POU3F2 MYOD1 ETV4 ZNF273 BSX ZNF543 NFIA SIX2 TBX3 PAX5 DLX5 ZIC3 PGR MED1 BATF GATA1 PBX1 HNF1B ESR1 FOSL2 MBD3 CLOCK GATAD2B JUN CUX2 ZNF117 SIX1 TLX1 ASCL1 MYF6 FIGLA ZNF92 PRDM5 OTX2 ZBTB7B CREB3 ZNF727 MAFA SMARCC2 NR1H2 MEF2B ZNF735 ZNF486 JUNB EBF2 GLI3 SP3 HAND1 NFATC4 ALS2CR8 GCFC1 NFAT5 TCF4 KLF4 ZNF724P ZNF257 CREB1 DDIT3 ZSCAN21 RAX NFYC CEBPG POU4F2 ARID3B RELA MBD2 MYOG FOXJ2 DMRTA1 ELF4 HOXA2 ZNF460 MEF2C MXD1 SMAD1 TCF7L2 HLTF

GO_POLYUBIQUITIN_BINDING Interacting selectively and non-covalently with a polymer of ubiqutin. TNIP2 PRPF8 PARP10 FAM175B ZFAND6 RAD23A TNFAIP3 VCP C1orf86 BRE SHARPIN UBXN1 EPS15 UIMC1 UBQLN1 PSMD4 BAG6 OPTN OTUD7A HDAC6 TAB2 IKBKG FAM175A RNF169 ATRIP UFD1L RAD23B OTUD7B RAD18 ZRANB3 ZRANB1 DZIP3 RNF125 SQSTM1 IKBKE C1orf124 UBQLN4 ZBTB1 RNF168 TNIP3 BRCC3

GO_QUINONE_BINDING Interacting selectively and non-covalently with a quinone, any member of a class of diketones derivable from aromatic compounds by conversion of two CH groups into CO groups with any necessary rearrangement of double bonds. VKORC1 SQRDL AOC3 DHODH NDUFS2 HHIPL2 NDUFS7 HHIP HHIPL1 AOC2 TP53I3 CBR4 VKORC1L1 SDHB ABP1 ETFDH SDHD

GO_GDP_DISSOCIATION_INHIBITOR_ACTIVITY Prevents the dissociation of GDP from a GTPase, thereby preventing GTP from binding. ITGB1BP1 GPSM1 GPSM2 CHM ARHGDIA RGS14 CHML SESN2 RANBP1 GDI2 ARHGDIG SH3BP4 ARHGDIB GDI1

GO_MACROLIDE_BINDING Interacting selectively and non-covalently with a macrolide, any of a large group of structurally related antibiotics produced by Streptomyces species. FKBP5 FKBP6 FKBPL TTC9 FKBP2 NFATC1 TTC9C FKBP7 FKBP14 FKBP9 FKBP11 FKBP1B TTC9B FKBP1A FKBP3 FKBP10 FKBP8 FKBP4

GO_THIOL_DEPENDENT_UBIQUITIN_SPECIFIC_PROTEASE_ACTIVITY Catalysis of the thiol-dependent hydrolysis of a peptide bond formed by the C-terminal glycine of ubiquitin and another protein. ZRANB1 USP9Y OTUB2 USP42 EIF3F USP25 OTUD6A UFD1L MYSM1 VCPIP1 USP46 USP16 USP22 ATXN3 ZC3H12A TANK TNIP1 USP27X FAM63B OTUD5 CYLD USP17L2 USP34 UCHL5 USP32 STAMBP JOSD2 USP21 USP13 ATXN3L USP33 USP31 JOSD1 TNFAIP3 BAP1 USP49 USP10 USP7 USP8 UCHL3 USP18 OTUD7A USP37 USP29 USP45 USP3 USP4 USP9X USP2 USP12 OTUD7B USP30 USP14 USP38 USP44 OTUD1 USP51 OTUB1 BRCC3 UCHL1 ANKZF1 FAM105B OTUD4 FAM63A OTUD3 USP6 YOD1 USP15 USP1 USP19 USP11 USP47 USP20 USP28 USP5 USP36 USP48 USP35 COPS5

GO_SEMAPHORIN_RECEPTOR_ACTIVITY Combining with a semaphorin, and transmitting the signal from one side of the membrane to the other to initiate a change in cell activity. PLXNA3 PLXNB1 NRP1 PLXNB3 NRP2 PLXNA2 PLXND1 PLXNA1 PLXNA4 PLXNB2 PLXNC1

GO_GTP_RHO_BINDING Interacting selectively and non-covalently with the GTP-bound form of the Rho protein. TRIOBP NET1 STXBP6 WHAMM PKN1 RTKN ROCK1 CDC42EP3 CDC42EP2 KCTD13 EXOC1 CDC42EP1 CDC42EP4 CDC42EP5 C15orf62 TNFAIP1

GO_VITAMIN_D_RECEPTOR_BINDING Interacting selectively and non-covalently with the vitamin D receptor, a nuclear receptor that mediates the action of vitamin D by binding DNA and controlling the transcription of hormone-sensitive genes. SNW1 MED1 MED16 MED12 TOB2 THRAP3 MED4 MED14 TAF11 RXRA MED24 MED30 MED13 TAF7 MED17

GO_NUCLEOSOME_BINDING Interacting selectively and non-covalently with a nucleosome, a complex comprised of DNA wound around a multisubunit core and associated proteins, which forms the primary packing unit of DNA into higher order structures. H2AFY NOC2L HMGN3 CABIN1 HIST1H3A ZNHIT1 HIST1H3J H1FOO MBD2 HMGN1 MBD3 RNF168 L3MBTL1 GATAD2B UHRF1 HMGA2 HIST1H3D ACTL6A SMARCE1 HDAC1 SMARCA1 MUM1 RNF169 HMGN2 HIST1H3B HIST2H3C H3F3C HNRNPC MTA2 CHD4 SMARCA5 RNF4 RCC1 HIST1H3I HP1BP3 SMARCC1 HIST1H3C HMGN5 HIST2H3D RBBP4 HIST1H3G HIST3H3 H2AFZ HIST2H3A SMARCD2 HIST1H3F ACTB SMARCA4 SMARCB1 DNTTIP1 HDAC2 VRK1 SMARCC2 HMGN4 CENPA HIST1H3H ARID1A HIST1H3E H3F3B H3F3A

GO_MISFOLDED_PROTEIN_BINDING Interacting selectively and non-covalently with a misfolded protein. DNAJB9 HSPA5 DNAJC10 EDEM1 CLU STUB1 F12 TOR1A HSPD1 HDAC6 DNAJC3 BAG6 SDF2L1

GO_HMG_BOX_DOMAIN_BINDING Interacting selectively and non-covalently with an HMG box domain, a protein domain that consists of three helices in an irregular array. HMG-box domains are found in one or more copies in HMG-box proteins, which form a large, diverse family involved in the regulation of DNA-dependent processes such as transcription, replication, and strand repair, all of which require the bending and unwinding of chromatin. TCF12 MEF2C OLIG2 EGR2 PAX6 JUN POU3F3 HHEX DLX5 PAX3 GATA3 ALX4 HOXC4 UTF1 SP1 HOXA3 MEOX1 PRRX1

GO_AU_RICH_ELEMENT_BINDING Interacting selectively and non-covalently with a region of RNA containing frequent adenine and uridine bases. ELAVL4 EXOSC9 EXOSC7 ELAVL1 TIAL1 CPEB2 DND1 HNRNPD ELAVL3 ZFP36 EXOSC4 KHSRP ZFP36L1 ZFP36L2 ZC3H12A TIA1 HNRNPA0 EXOSC8 MEX3D APOBEC1 CPEB1 CPEB3 CPSF1 NUDT21

GO_DEATH_RECEPTOR_ACTIVITY Combining with an extracellular messenger (called a death ligand), and transmitting the signal from one side of the plasma membrane to the other to initiate apoptotic or necrotic cell death. NGFR EDA2R TNFRSF10A TNFRSF9 TNFRSF21 FAS TNFRSF14 TNFRSF18 TNFRSF10B RELT TNFRSF8 TNFRSF4 TNFRSF10D TNFRSF11A CD27 TNFRSF1B LTBR TNFRSF11B TNFRSF10C TNFRSF25 TNFRSF19 TNFRSF1A TNFRSF6B CD40

GO_ORGANIC_CATION_TRANSMEMBRANE_TRANSPORTER_ACTIVITY Enables the transfer of organic cations from one side of a membrane to the other. Organic cations are atoms or small molecules with a positive charge that contain carbon in covalent linkage. SLC22A2 SLC22A3 SLC7A8 SLC22A4 SLC22A16 SLC22A13 SLC22A5 SLC22A14 SLC22A1 SLC25A20 SLC25A29

GO_ATPASE_ACTIVITY_COUPLED Catalysis of the reaction: ATP + H2O = ADP + phosphate; this reaction directly drives some other reaction, for example ion transport across a membrane. DDX39B ABCC11 RFC1 ABCC9 ATP6V1E2 KATNA1 FIGNL2 RALBP1 HSPA1A DDX3Y TOP2B RECQL5 DDX19B DDX31 BPTF EIF4A3 NBN RTEL1 DHX30 ATP6V1B2 GTF2H2 DDX23 ATP6V1G2 ATP13A4 RAD54B LONRF2 CHD8 RAD18 TAP1 DDX1 YTHDC2 ATP12A PEX1 ATP8B2 RAD50 DDX41 ATP2A1 ATP2A3 HSPA8 SNRNP200 ABCB11 CHD6 ANXA1 ABCD4 DDX39A ATP2B3 G3BP1 ATP5G3 ATP1B3 PEX6 ABCC6 ATP13A5 IGHMBP2 ABCB4 ATP1A1 ABCD1 ATP6V1A FIGN MCM6 SPO11 ERCC8 ABCB8 DHX16 DMC1 ATP9A ABCB6 ATP5H DDX25 RAD51B ATP7A RECQL ABCD3 ATP11B DDX50 MYO1C VPS4B MYO1E ATP2B1 ABCA12 ATP2C2 ATP6V1D ABCA7 DHX57 ATP1A3 RECQL4 MYH6 DDX51 ABCD2 ATP6V0A2 RAD17 LONP1 DDX43 ATP13A1 PIF1 DHX34 PSMC5 MNAT1 AFG3L2 ATP13A2 ATP6V1F ABCA5 DDX27 ATP8A2 RAD51C ATP6V1C2 ATP6AP1L PSMC4 ABCC1 ABCA4 DDX21 DDX49 DHX29 ABCG2 CRBN ATP11C ATP5D CHD2 DDX18 MYO3A DHX15 CHD4 KIF18A ATP7B ASNA1 ABCA8 SUPV3L1 MYO1D ATP5EP2 ATP1B2 ATP1A2 TDRD12 XRCC6 DQX1 SMARCA2 TOP2A TCIRG1 ABCG1 MYO1B CHD1L ASCC3 LONP2 ATP1B1 ATP5G1 ATP8A1 DHX40 MYH14 ATP10D VPS4A DDX56 RFC3 ERCC2 CHD5 ABCA2 FBXO18 ATP13A3 ATP5F1 ATP6AP1 PSMC2 ERCC3 GTF2H1 ATP5G2 SMARCA4 ABCC8 DDX5 PSMC1 PSMC3 C9orf102 DDX59 DDX55 ABCA9 XRCC5 ABCC3 WRN DDX12P ATP6V0B EIF4A2 YME1L1 SMARCAL1 ATP6V0A4 ATP6V0C ATP11A MYH7 DHX35 DHX32 ATP6V0E2 ABCA6 POLQ HSPA1B ABCC5 ATP6V1E1 TAP2 BRIP1 TAPBP CFTR RBBP4 ABCG8 RUVBL2 PSMC6 ABCG5 CDK7 ABCG4 ATP6V0D1 ATP10A ATP5E LONRF3 ATP6V0E1 HELB DHX36 XRCC2 MCM4 DDX24 ATP4B FIGNL1 DDX6 UPF1 CCNH MYO10 RFC4 RUVBL1 BLM DDX42 ATP10B ATP2B4 DNA2 ATP6V1H MYH10 RAD51D ABCC13 ATP8B1 DHX37 SPAST ABCB1 CCT8 TTF2 MCM7 DDX54 MRE11A XRCC3 DHX9 ATP6V0A1 CHTF18 MYL6 DDX19A ATP5B ERCC6 SKIV2L ABCA3 MYH3 DDX46 KATNB1 ATP9B ATP8B3 ABCB10 DDX11 RFC5 ATP5A1 ATP6V1G1 KATNAL1 DDX53 CLPX DDX4 RFC2 GTF2H4 RAD51 ATP2B2 SMARCA1 ABCC4 ABCC2 DSCC1 DDX20 ATP2C1 ABCB5 DDX28 ATP5O TDRD9 ABCC10 ABCA13 ABCA10 ATRX ATP2A2 ATP5C1 NSF KATNAL2 DDX17 DDX52 ATAD1 MYO7A ABCB9 ABCB7 PCYOX1 CHD3 ATP6V1C1 DDX3X FXYD2 GTF2H3 ATP1A4 DDX10 DHX38 SPG7 ATP4A HSPA6 DDX47 ATP6V1G3 ATP8B4 EIF4A1 MYH9 ABCA1 TNNT3 ABCC12 CHD1 LONRF1 DHX8 MOV10L1 DHX33

GO_TRANSFERASE_ACTIVITY_TRANSFERRING_NITROGENOUS_GROUPS Catalysis of the transfer of a nitrogenous group from one compound (donor) to another (acceptor). AGXT2 TAT CCBL1 AMT GAPDH CCBL2 AGXT2L2 OAT ABAT GATM GFPT2 AADAT PSAT1 BCAT2 GPT AGXT AGXT2L1 GFPT1 GOT1L1 BCAT1 GOT1 GOT2 GPT2

GO_TRANSFERASE_ACTIVITY_TRANSFERRING_AMINO_ACYL_GROUPS Catalysis of the transfer of an amino-acyl group from one compound (donor) to another (acceptor). GGTLC1 GGTLC2 CHAC2 F13A1 QPCTL A2LD1 GGCT GGTLC3 TGM1 CHAC1 EPB42 TGM7 GGT3P TGM3 TGM4 QPCT TGM2 GGTA1P GGT6 ATE1 TGM5 GGT2 TGM6 GGT1 GGT5 GGT7

GO_PEPTIDE_ANTIGEN_BINDING Interacting selectively and non-covalently with an antigen peptide. HLA-DPA1 FCGRT CLEC4M HLA-DQB1 MAML1 TAPBP HLA-A HLA-DRB3 HLA-F HLA-DRB1 TRBV12-3 TRGV3 HLA-DQA1 SLC7A9 SLC7A5 HLA-DRB5 HLA-B TAP1 HLA-E HLA-C HLA-DRA DHCR24 CD209 HLA-DPB1 SLC7A8 HFE

GO_TRANSCRIPTION_FACTOR_ACTIVITY_RNA_POLYMERASE_II_CORE_PROMOTER_PROXIMAL_REGION_SEQUENCE_SPECIFIC_BINDING Interacting selectively and non-covalently with a sequence of DNA that is in cis with and relatively close to a core promoter for RNA polymerase II (RNAP II) in order to modulate transcription by RNAP II. ETV1 MEOX1 IRF3 MTF1 MYOCD MEIS1 C2orf3 PITX2 BTG2 CDX4 NFKB2 MIXL1 ATF4 SIX4 CEBPD DBP DDN ZFP90 CEBPE PITX1 REST MYBL2 NR1D1 MYPOP YBX1 FOXJ1 BARHL2 IRF4 HSF1 SMAD4 GATA3 SCRT2 BCL11A MESP1 GMEB1 SOX18 POU2F2 TFAP4 TP73 MEOX2 NKX2-8 IRF1 PAX6 HOXD13 FOS ELF3 FOSL1 MEIS2 STAT3 ZNF746 GRHL1 TP63 GLIS2 RCOR1 NKX6-1 TCF3 ZBTB7A ZNF217 MEF2A HHEX NEUROD1 TEAD1 CC2D1A EGR2 ASCL2 ZFPM1 RFX4 SKOR2 FOSB AKNA ZFAT PPARA IFI16 NR4A3 ZIC1 MEF2D NR2C1 EBF1 SNAI2 ATF2 POU1F1 INSM1 TFAP2A TBX15 POU4F1 MSX1 KLF16 TXK GZF1 GSX1 FOXK2 DLX4 ONECUT2 SOX4 NR4A1 ELK3 NFX1 KLF17 HES1 FOXA1 JDP2 TBX2 GFI1 NFATC2 NR1H4 BCL11B ONECUT3 NFIB NRF1 GABPA HELT SKIL SPI1 LEF1 HES5 NKX6-2 HIF1A ZKSCAN3 HEYL MYC EBF4 E2F4 HMGA2 CEBPA FOXC2 ETS1 CTCFL ZNF281 YY1 NEUROG3 STK16 HOXA5 POU2F3 BHLHE41 ZBTB20 NACC2 TFE3 BATF3 PLAG1 HOXC11 SOX11 SREBF1 OTX1 SALL1 DLX3 CTCF SOX2 SRF KCNIP3 EN1 NR5A2 FOXO1 CEBPZ ARNTL ESRRA ELK1 NFATC3 NFXL1 TSHZ2 PTF1A ZGPAT EHF GCM2 CREB3L2 KLF15 ISL1 EBF3 IRF7 PRDM1 OVOL1 HAND2 OTX2 ZBTB7B CREB3 MAFA CAMTA1 MEF2B NR1H2 JUNB EBF2 SP3 GLI3 HAND1 NFATC4 ALS2CR8 GCFC1 ARNTL2 NFAT5 KLF4 TCF4 CREB1 PLAGL2 DDIT3 ZSCAN21 RAX NFYC CEBPG POU4F2 STAT5B ARID3B TEF RELA GATA2 MYOG FOXJ2 ELF4 BACH1 HOXA2 MEF2C MXD1 PROX2 SMAD1 HLTF NOBOX RBPJ NUCKS1 SP1 SMAD2 SOX1 SREBF2 MZF1 HNRNPK FOXD1 NKX2-5 HLF EGR1 POU3F2 ETV4 BSX NFIA SOX12 SIX2 TCF21 TBX3 PAX5 DLX5 ZFPM2 ZIC3 PGR BHLHE40 TFAP2B GATA1 PBX1 BATF ESR1 FOSL2 CLOCK JUN CUX2 TLX1 SIX1 ASCL1 FIGLA PRDM5 HOXC13 BARHL1 ONECUT1 RBPJL FLI1 ETV2 AEBP2 HINFP MYBL1 TFAP2C NKX3-2 CEBPB NR3C1 CREB3L1 E2F8 ZNF750 NDN FUBP3 GLI2 MYB WT1 ZNF148 PITX3 TBX20 BATF2 ELF1 MEIS3 PATZ1 NR4A2 SOX9 DACH1 NFE2L3 ZNF292 FOXI1 PAX8 NR2E1 CAMTA2 KLF13 SKOR1 CC2D1B PROX1 NFIL3 KLF5 ZNF639 HNF4A EPAS1 TSHZ1 HOXA10 TFAP2E ATF3 FOXP2 TSHZ3 HOXA7 PHOX2B ATOH1 MLXIPL PLSCR1 MITF TCF12 ZNF536 SKI ZNF219 NFIC GCM1 ETS2 MAFB TGIF1 FOXL2 AR SNAI1 POU4F3 NR6A1 TFAM CREBBP SNAI3 HNF1A RUNX2 CRX ETV6 ARNT2 NPAS4 NRL NEUROD6

GO_CARBON_OXYGEN_LYASE_ACTIVITY Catalysis of the breakage of a carbon-oxygen bond. PCBD2 CA4 ACO1 PCBD1 APIP UBA5 POLQ CA14 CA9 PTPLAD1 THNSL2 GMDS CA5A HADHA CA5B ALAD ENO3 APEX1 C14orf149 FASN CA8 ENO2 ENO4 NTHL1 FH PTPLA SMUG1 PDDC1 CA1 XRCC5 CA6 CBS PTPLAD2 ACO2 PTS ECHDC2 ALKBH1 POLB OGG1 PARK7 UROS THNSL1 CA7 AUH ENO1 NEIL2 PUS1 CA2 HMGA2 CA3 NEIL1 AGXT2L1 HADHB ENOSF1 UROC1 HSD17B4 CA11 CARKD RPS3 ECHS1 APLF CA12 CA13 NEIL3 APEX2 EHHADH XRCC6 PTPLB TGDS

GO_STEROL_TRANSPORTER_ACTIVITY Enables the directed movement of sterols into, out of or within a cell, or between cells. Sterol are steroids with one or more hydroxyl groups and a hydrocarbon side-chain in the molecule. APOA2 CETP APOA5 ARV1 STARD5 STAR ABCG8 STARD4 APOE ABCG5 ABCG4 APOA4 NPC1 OSBP APOA1 ABCA1 ABCG1 C20orf79 APOB SCP2

GO_VOLTAGE_GATED_CATION_CHANNEL_ACTIVITY Enables the transmembrane transfer of a cation by a voltage-gated channel. A cation is a positively charged ion. A voltage-gated channel is a channel whose open state is dependent on the voltage across the membrane in which it is embedded. KCNJ9 NOX1 KCNJ12 KCNK5 CNGA3 KCNG3 KCNG4 KCNQ5 CACNG8 KCNK1 KCNH4 TMC2 KCNIP2 KCNQ4 KCNA3 HCN3 CACNG1 KCNJ11 KCNQ1 CACNB1 HCN2 OPRM1 CACNA1B KCNS3 KCNJ3 CACNA2D1 CNGB3 KCNK9 KCNG1 CACNB4 GRM7 KCNAB2 KCNAB3 KCNA6 KCNK18 KCNC4 CACNA1E KCNA4 KCND2 KCNV2 CACNG3 HVCN1 CACNA1S KCNJ10 KCNA7 TPCN1 CALHM1 KCND3 CACNA1A KCNJ8 KCNA1 CNGA1 KCNJ1 ABCC8 KCNU1 KCNJ14 GAS6 KCNV1 CACNB3 CACNG7 KCNK2 KCNS1 KCNH6 KCNT2 KCNT1 CACNA1D CNGB1 CACNG4 KCNMA1 KCNK6 CNGA2 KCNAB1 REST NCS1 CATSPER3 KCNG2 CACNA1H KCNC3 CACNA1G KCNH8 KCNS2 KCNA2 HCN1 CACNA1I CACNA2D2 RYR1 KCNE1 IL1RAPL1 KCNH1 KCNE4 KCNC2 KCNF1 PKD2 KCNA5 KCNJ6 KCNC1 KCNH3 KCNJ4 KCNK3 ITGAV CACNG2 KCNE1L KCNH2 KCNJ15 KCNJ5 KCNH7 CACNG5 KCNE2 KCNJ16 KCND1 KCNB1 KCNJ2 KCNE3 CATSPER2 TPCN2 KCNJ13 CNGA4 HCN4 CACNG6 CATSPER4 KCNQ3 CACNB2 KCNH5 CACNA2D4 CACNA1F TMC1 KCNA10 KCNB2 KCNJ18 KCNQ2 CATSPER1 CACNA1C

GO_INSULIN_RECEPTOR_BINDING Interacting selectively and non-covalently with the insulin receptor. SNX1 DOK6 PHIP SNX4 DOK4 DOK2 DOK5 SRC PDPK1 DOK7 IRS1 INSL3 GRB10 INS PTPN1 IGF2 SORBS1 IRS2 IGF1 PIK3R1 IGF1R IRS4 DOK1 FRS3 LMBRD1 DOK3 FRS2 PTPN11 SHC1 ENPP1 SNX2

GO_HYDROLASE_ACTIVITY_ACTING_ON_ESTER_BONDS Catalysis of the hydrolysis of any ester bond. ACOT7 PPP3R1 FAM135B PLA2G4D TATDN3 ENPP6 EXD3 CDC25A ERCC4 SMG7 OCRL NT5C1A NCEH1 PPP1R3D LIPF PLA2G16 GDPD4 EDC3 ERVK-7 DCPS DAGLA POLE PPP2CB PAN3 ZC3H12A PPP3CB NT5DC4 GDPD1 EIF2C2 DDHD2 DBR1 LOC344967 PLB1 BPNT1 DNASE1L2 MTM1 PPAPDC1B DUSP16 PNLIPRP2 NANP HAGH KIAA1274 ENDOD1 C14orf126 VARS2 DGCR8 TSEN15 MRE11A RNASE1 PDE2A PTPRU NUDT16 GDPD3 LARS INPP5J OLAH LPIN3 IMPA2 SMPDL3A C2orf43 PTP4A2 REXO1L1 PTPRB RNASE6 POP1 LOC81691 NT5DC1 C20orf72 PDE1B LIPA PPP2R1A MYH3 AOAH STK31 INPP5F SLFN14 LYPLA1 RNASE13 PNPLA5 UFSP2 LIPH SEC23IP PTPN22 EXOSC2 PTPRF PTRHD1 PPP6C APEX1 SSH2 PTPRM PLA1A SMG6 PPP1R3C PROCA1 LPPR4 PLA2G4B TENC1 ENDOV PLA2G4F PPP5C DUSP6 PTPN2 ERCC5 PTPDC1 AEN PTPN13 PTPRC WDR85 PLBD1 ABHD10 PLD2 PLA2G4E PDP1 TG RNASE11 PPP2CA PIKFYVE DFFB CAMK2G CCRN4L TSEN54 GNS ASPA MTMR7 LPPR2 SSU72 DUSP19 PTPRT POP4 SMPD2 DCP2 PTPN6 DUSP27 PAFAH1B1 PTPRD IARS ACP1 SMG5 FEN1 PPM1L PAPL CCR1 DIS3L XRN1 TAB1 LIPG ABHD16A PDE3B TPTE NTPCR NLGN4X PDE4C PPM1M PTP4A1 PPP2R2C DDHD1 BAAT OGG1 LIPJ RNASE9 DUSP1 FBP2 CES3 INPP5D IARS2 DNASE2B DLG1 ACOT9 CNOT2 PNPLA7 ARSE ESD C16orf57 PPM1N PDE4A LIPC PDE8A RNASEH2C ACP2 PLCG2 SLX1B FAM135A PDE10A PSPH ABHD2 PLCXD3 PLCB3 LIPI PFKFB1 EME1 SULF2 PLCE1 MBD4 CNOT6L MDP1 RPP38 PDE9A LPL DTD1 BPHL EXOSC1 C12orf65 PPEF2 CNOT1 HRSP12 DICER1 SACM1L PLA2G4A GPLD1 SMPDL3B RAD9B MTMR14 DNASE1L1 G6PC3 PDE4B DIS3L2 RNASET2 DUSP4 CPSF4L PTPN7 EDNRA MINPP1 ABHD1 NEIL1 BPGM DNASE2 CDC14C PPAP2B PTPN20B DLGAP5 SGSH PTPRH EXOSC5 RAG1 GDPD5 DNASE1L3 DNASE1 HARBI1 PPP1CC FASN KIAA0391 PLCB1 PLD1 PLCB2 PTPRZ1 PDE8B ACOT12 PDE6H RNASE3 INPP4A DUSP5 NTHL1 PTPN1 ACHE ILKAP PPM1D PLA2G5 PTPRN PNPT1 ACOT4 NLGN2 ISG20L2 NT5E SBF1 PTPN18 PPM1A AARS DUSP26 DUSP15 PPM1G PPP2R2D LIPE INPPL1 RPP25 EXOSC4 INPP4B PDXP TMEM55B UBLCP1 DUSP7 EXOSC8 PPP3CA FBP1 DUSP2 RAD50 DUSP18 DUSP23 ARSD PGAP3 TREX2 CTDNEP1 EYA2 NLGN3 MTMR8 EXD1 PTPRN2 ATP1A1 GDPD2 PPP1R15B PPP1CA ACSBG2 PLCH2 CHRM1 AZGP1 POP7 NUDT19 CHRM5 TPTE2 DUSP22 BDKRB2 CES4A RNASEH2A DROSHA ERI3 RAD51B SYNJ1 PSPHP1 POP5 NT5C3L ACP6 EYA4 ARSK DUSP21 NLGN1 ZC3H3 PLCXD1 DUSP3 DCLRE1C ACPP PPAP2C PPP1CB NEIL2 ERN2 RPP21 ZRANB3 ACOT1 PPIP5K1 PGAM1 PHLPP1 PPP3CC MTMR1 PLCD3 PTPRQ PPP2R2A ALPI PTRH1 PPM1K PNLIP DIS3 ALPP RNASE12 NT5C2 APTX PLA2G2F MRPL44 PDE1C PNKP PDE3A CDC14B FANCM PPIP5K2 THEM5 PPP4C PLA2G2E NOB1 ENPP2 CCKBR PTPRG CDC25B ARSJ FAAH PTPN3 SND1 ICT1 NOTUM LPIN2 STYXL1 LPPR5 TDP2 RNASE10 MAP1S PLA2G2A CES1 DAGLB APLF PDPR PPME1 SGPP2 RNASE4 RGN ENPP7 DNAJC6 CHRM3 MTMR4 POLD1 PON3 PTPRJ ZC3H12B CCL5 MTMR3 AADACL2 PFKFB2 PDE5A EME2 PDE6G STYX REXO2 C16orf73 G6PC ARSB PTPRS PON2 PDE6D FAN1 CNOT6 IMPA1 FAM83B PHPT1 CTDSP2 CA3 RCL1 XRCC3 CHKA ASTE1 PLD4 CYCS PLCD1 BIVM LCK NT5M PTPRA PLCG1 PLA2G10 ABHD4 N4BP2 GPCPD1 PLCXD2 ERI1 LARS2 NUDT7 DOM3Z MTMR2 PTPN9 DUSP28 ALKBH1 DUSP10 C11orf54 CES2 F2RL2 PTPMT1 PLCL2 PTPRR ACY3 RPE65 HDHD1 POLA1 PXDNL LGALS13 TMEM55A CPSF3 TATDN2 PHOSPHO2 LOC100133495 CASR RAD51 RPAP2 CDC25C PPP2R3B POLN PGLS PGAM2 RPS3 ERVK-10 FIG4 TIMM50 PPA2 XRCC2 PTPLB SETMAR PNPLA2 PDIA3 PPP1R8 CLC PPP1R3B ERI2 EYA3 LOC100128274 LYPLAL1 PGAP1 PTER PPAP2A RNASE7 AARSD1 ACOT13 RAD51D SSH1 PTPN14 ISG20 DEM1 DUPD1 PTEN HDDC3 PDE6B DNA2 G6PC2 ELAC1 ENDOU LIPK AADAC PHLPP2 NT5C1B ABHD12 CPPED1 CES5A NAPEPLD RNASE2 RNASEL WRN EXOSC9 DUSP14 INPP5A PLCH1 TSEN2 STS RNGTT EXO1 TBC1D10B C17orf42 PGAM4 PELO ERVK-6 MPPE1 SLX1A CILP2 ACP5 TDP1 PDE6C PLA2G7 GALNS MGLL TATDN1 PPP4R1 LHPP ACPT SSH3 SGPP1 SMPD3 HIBCH EPHX2 PDE6A RNASEH1 DUSP8 C12orf5 CCR5 INPP5E PARN VARS PDE7A PTPN5 LPIN1 PNPLA1 TOE1 PLA2G2D THEM4 PTPRK MYH8 ANG ENPP1 EXOSC6 EXOSC7 EXOG APEX2 PLA2G6 GEN1 GDE1 LACTB2 ENOPH1 BCHE ABHD3 LIPM HDHD2 RBBP8 C20orf3 PPM1E ACPL2 PPM1B CTDSPL RPP40 DUSP13 DUSP11 LPPR3 PLCL1 EYA1 PGAM5 CNOT8 PAFAH2 REV3L ADORA1 PNLIPRP3 PAFAH1B3 ACOT8 REXO4 PLCB4 PTPRE SLX4 CTDSP1 RAD9A RNASEH2B MYH6 PTPRO PON1 OC90 PNKD PLA2G3 SMPD4 CDC14A PLA2G15 POLB SAMHD1 DCLRE1B TSEN34 PPM1H PNPLA4 PDP2 PTRH2 ERCC1 CNP EIF2C1 EXOSC10 RNASE8 PPAPDC1A GNB1 PLD3 NT5C3 SIAE UFSP1 RAD51C CILP LYPLA2 IDS ACOT6 MTMR6 DCLRE1A PFKFB4 ABHD5 GNPAT PPM1J PPP2R2B ALPL RPP30 ERN1 PPM1F EXOSC3 SMPD1 PTPN12 PP2D1 PAFAH1B2 PNPLA3 NEIL3 TSN PDE12 G3BP1 ALPPL2 ARSH PLA2G4C INPP5K EXD2 ENPP3 AADACL4 RNASEK ERVK-8 PHOSPHO1 UBASH3B ENDOG LIPN PPT2 INPP1 DMC1 PLA2G1B ITPK1 CEL PNPLA8 PPT1 NT5C ZC3H12C NLGN4Y PRORSD1P PLCZ1 PLD6 PTP4A3 NT5DC3 REXO1 PMS2 RAD1 CA2 HMGA2 PTPN21 PAN2 ASPG CTDP1 PDE7B IMPAD1 MAP2K1 ARSF PDE1A PDE4D LPPR1 ARSI NME1 PFKFB3 MUS81 PNLIPRP1 PTPN11 PPTC7 PDE11A SULF1 PPEF1 SMUG1 HMOX1 ABHD6 ARSA CA1 XRN2 ELAC2 PLA2G12B POLG PTPN4 DUSP12 PLCD4 CNOT7 CTDSPL2 ACOT11 SYNJ2 PNPLA6 H6PD ZC3H12D CDKN3 RARRES3 PLA2G12A PGP NT5DC2 INPP5B DDX1 PPP2R5D DUSP9 PTPN23 ACOT2 ARSG CPSF4

GO_IMMUNOGLOBULIN_BINDING Interacting selectively and non-covalently with an immunoglobulin. FCGR1B FCGR1C CD4 FCGR3B AMBP FCER2 CD300LG FCGR2C FCER1A FCGR2A FCGR3A UMOD FCGRT FCGR2B VWF IGJ PIP HRG LGALS3 MS4A2 FCGR1A FCAR FCER1G

GO_NF_KAPPAB_BINDING Interacting selectively and non-covalently with NF-kappaB, a transcription factor for eukaryotic RNA polymerase II promoters. PSMA6 FAF1 COMMD7 SETD6 APEX1 COMMD6 NFKBIA ANXA4 BRMS1 NFKBID CDKN2A HIF1AN CPNE1 CDK5RAP3 HDAC1 PPARD RNF25 RELA NPM1 TAF4B HDAC2 FOXP3 BCL10 AKAP8 TP53BP2 GSK3B MTDH HDAC3

GO_MAP_KINASE_ACTIVITY Catalysis of the reaction: protein + ATP = protein phosphate + ADP. This reaction is the phosphorylation of proteins. Mitogen-activated protein kinase; a family of protein kinases that perform a crucial step in relaying signals from the plasma membrane to the nucleus. They are activated by a wide range of proliferation- or differentiation-inducing signals; activation is strong with agonists such as polypeptide growth factors and tumor-promoting phorbol esters, but weak (in most cell backgrounds) by stress stimuli. MAPK4 MAPK3 MAPK15 MAPK11 MAPK8 MAPK13 MAPK7 MAPK1 MAPK9 MAPK14 MAPK6 MAPK10 NLK MAPK12

GO_DIACYLGLYCEROL_BINDING Interacting selectively and non-covalently with diacylglycerol, a diester of glycerol and two fatty acids. UNC13A CDIPT CHPT1 UNC13C RAPGEF2 RASGRP1 RASGRP2 DGKD RASGRP4 RASGRP3 UNC13B

GO_FAD_BINDING Interacting selectively and non-covalently with the oxidized form, FAD, of flavin-adenine dinucleotide, the coenzyme or the prosthetic group of various flavoprotein oxidoreductase enzymes. CYB5R1 DAO DDO MICAL2 MICAL3 KMO AGPS MMACHC PRODH CYB5RL MICAL1 CRY2 AIFM1 CYB5R2 OXNAD1 COQ6 MTRR CYB5R4

GO_RNA_POLYMERASE_II_ACTIVATING_TRANSCRIPTION_FACTOR_BINDING Interacting selectively and non-covalently with an RNA polymerase II transcription activating factor, a protein involved in positive regulation of transcription. HEY2 BHLHE40 CREBBP EP300 CREB1 MAD2L2 TBX3 TBX20 LMO2 BHLHE41 SIN3A DUSP26 TBX6 NFE2L2 POU1F1 ISL1 RB1 NHLH2 JUN ATF2 NCOR1 CTNNB1 HIPK2 HNF4A EXOSC9 TP53BP1 BEX1 SMAD3 T IFI27 ZFPM1 EGR2 LDB1 EOMES SETD3 NEUROD1 PITX2

GO_NAD_BINDING Interacting selectively and non-covalently with the oxidized form, NAD, of nicotinamide adenine dinucleotide, a coenzyme involved in many redox and biosynthetic reactions. GLUD1 UXS1 SIRT4 SIRT3 SIRT2 SIRT6 SIRT1 HADH HPGD CRYL1 SIRT7 SIRT5 ALDH1A3

GO_SULFOTRANSFERASE_ACTIVITY Catalysis of the transfer of a sulfate group from 3'-phosphoadenosine 5'-phosphosulfate to the hydroxyl group of an acceptor, producing the sulfated derivative and 3'-phosphoadenosine 5'-phosphate. NDST3 CHST14 TPST2 CHST10 HS3ST1 WSCD1 CHST9 HS3ST4 HS3ST5 CHST4 CHST7 HS6ST3 SULT1B1 SULT1C3 CHST15 SULT2B1 SULT1A2 TPST1 DSEL CHST13 HS6ST2 HS3ST2 SULT1C2 SULT1A1 NDST1 CHST6 NDST2 HS3ST3B1 UST GAL3ST3 CHST3 GAL3ST4 SULT4A1 NDST4 GAL3ST2 CHST8 GAL3ST1 CHST11 SULT1A3 HS2ST1 CHST2 SULT6B1 SULT2A1 HS3ST3A1 CHST12 SULT1E1 CHST1 SULT1C4 CHST5 HS3ST6 HS6ST1

GO_ATPASE_BINDING Interacting selectively and non-covalently with an ATPase, any enzyme that catalyzes the hydrolysis of ATP. SNTA1 TAF9 BBC3 CAV1 ANK2 PEX19 NOP58 PDE4D UBXN1 FBL LCK ATP6V0A1 ATP6V0A2 SNX10 S100A1 PGR NHP2L1 NSFL1C AR SDF2L1 ATP6V1E1 PTPN3 ZNHIT6 RALB NKAIN1 ATP6V0A4 ATP6V1G1 SVIP EZR ESR1 SLC26A9 RAB4A ATP1B3 NUFIP1 TOR1AIP1 NR1H2 ATP1B1 PLN FXYD7 SELS TCIRG1 UBE4B PKD2 ANK1 METTL21A DNAJC10 TRPC5 GABARAPL2 ATP1B2 TRPC6 ATPIF1 RAB3A FXYD4 ABCA1 HRC ATXN3 PEX26 DNAJB1 UFD1L TOR1AIP2 DERL1 ATP6V1G3 ADCY10 CHMP4A USP25 SLN PIH1D1 SYVN1 RDX RALA WFS1 BRSK2 ALDOB FXYD3

GO_RETINOL_DEHYDROGENASE_ACTIVITY Catalysis of the reaction: retinol + NAD+ = retinal + NADH + H+. ADH7 DHRS7C SDR9C7 RDH12 HSD17B6 AKR1C3 ADH1C DHRS3 ADH4 ADH1A DHRS9 RDH10 RDH8 SDR16C5 BMP2 RDH11 RDH16 RDH5

GO_GUANYL_NUCLEOTIDE_BINDING Interacting selectively and non-covalently with guanyl nucleotides, any compound consisting of guanosine esterified with (ortho)phosphate. RAB12 IRGQ ARF3 TMEM173 CNGA2 GNA13 RAB21 EEF2 CNGB1 PCK1 NKIRAS2 GIMAP8 NPR1 TUBA4A EIF2S3 THG1L RAB40AL RHOBTB1 RNF112 RHOBTB2 GBP5 MTG1 GTPBP6 RAB41 RALA SEPT2 GNA12 GTPBP8 RAB37 REM1 DRG1 RRAS2 GTPBP3 ANXA6 RAB32 GNAO1 HSP90AA1 MB21D1 RHEBL1 GTPBP2 ERAS IFT27 TUBD1 ADSSL1 RAB40C RRAGB RAB3D RAB11B DNAJC27 ARL4C ARL14 NKIRAS1 ARF1 OLA1 RAB24 DIRAS1 GBP1 RAP1B GBP3 RAB26 EHD4 GNL1 GUCY2D RAB4B RAPGEF2 RASD1 RAB6A ARF5 RHOG RAP1A GNAZ RND3 RERG RABL3 SEPT12 LANCL2 RAB6C TUFM RABL5 RAB17 RAB22A RAB19 RAB14 IFI44L PDE6H RAB33A NRAS CDC42 SAMHD1 TUBE1 CNGA4 RASL12 EEFSEC RHOQ GTPBP5 ARL10 CIITA RAP1BL ERAL1 RAB2A GPN2 TSR1 DNM3 HBS1L GNAT2 GNL3 GIMAP5 RAB2B AGAP2 ARL17B RAB1A GNAQ NIN ARL11 ARFIP2 GEM ARL5C SEPT9 SRL TGM2 RHOJ GLUD1 EFTUD2 RP2 RAB9A GUF1 SEPT6 EHD3 HSP90AB1 RASL11B C8orf80 GUCY1A3 RAN RAB9B EEF1A2 RAB27A GFM2 TUBA1A RAB6B SEPT7 RAC3 PCK2 RAB3A SEPT1 RAB8A RHOT2 RRAS RERGL IRGC ARL1 RHOT1 CNGB3 RAP2C TUBA1B NMUR2 GLUD2 GMPPB RALB RAC1 SEPT8 DNM2 GUCY1A2 TUBB SRP54 RAB5A GPN3 REM2 GNA15 GNA11 URGCP EHD2 MTIF2 GBP4 RIT1 NUDT2 RABL2A GUCY2C GPN1 SAR1A RAB20 TUBB1 PDE11A GNAI1 AGAP3 RAB5C ARHGAP35 LOC100507096 ARL3 RASL10A TUBA3C RHOF BMS1 RAB35 ARF4 GNA14 RAB39 LRRK1 RASL11A RHOC PDE1A RHEB RABL2B GFM1 NME1 HRAS EIF2B2 GNAS GBP6 ADSS RHOH SMCR7L GSPT1 GIMAP1 EEF1A1P5 RAB3B MFN1 RAB43 GIMAP6 NOLC1 GIMAP2 DAPK1 ATL3 TUBA8 TUBB8 RND1 PDE6G PDE5A RAB4A DRG2 TUBB2B TUBA3D RAB3C ARL6 RAB31 GNL2 RAB25 GVINP1 ARL5B ARL13B LSG1 EHD1 MOCS1 ARL4A TUBB2A RAB5B RAB11A RAB39B SEPT4 TUBG2 RASD2 ARL15 RAP2B INSR NPR2 RIT2 RAB7B RAP2A MX2 TUBB6 EEF1A1 SEPT10 SEPT14 RAB7L1 GBP2 SCG5 C10orf129 PRKG2 RAB27B MFHAS1 SEPT3 UPRT RHOB ARHGEF5 LOC255308 RAB38 TRIM23 LRRK2 RAB1B TUBB4B GCH1 RAB44 SRPRB GNAT1 PDE2A ARHGAP5 NUDT16 GNAI2 ARL8B GTPBP10 MX1 TUBB4A RND2 PRKG1 RSG1 EIF5 RTKN FPGT RANBP17 ATL2 SUCLG1 SAR1B CNGA3 RAB40B RHOU DNM1 MMAA GNL3L ARL9 ARF6 SRPR ACSM1 AGAP1 GUCY1B3 FKBP4 RAB8B ARL5A ARL2 RHOD NOA1 RRAGA RASL10B RAB1C AK3 DIRAS2 GBP7 TUBA4B KRAS TUBAL3 ARL4D RAB15 RRAGC TPX2 DNM1L GIMAP4 RRAD SEPT5 ARL8A RAB34 SEPT11 PRPS1 RHOV RHOA DIRAS3 GNAI3 RAB23 GSPT2 RAC2 EFTUD1 ARL13A RRAGD SEPHS1 TUBA3E RAB42 PDE10A GTPBP4 AK4 IRGM RASEF C9orf69 RAB30 GNAL MFN2 GUCY2F ARFRP1 DYNC1LI1 RAB28 RAB18 DNM1P34 SPAG1 SUCLG2 GTPBP1 TUBG1 OPA1 PDE6C HHAT ARL16 GIMAP7 CNGA1 RNGTT ATL1 RAB10 ERCC3 C9orf86 RAB40A RAB33B RAB36 TUBB3 RAB13 RAB7A EIF5B MRAS ACSM5 GNAT3 TUBA1C

GO_ARYLSULFATASE_ACTIVITY Catalysis of the reaction: a phenol sulfate + H2O = a phenol + sulfate. ARSD ARSA SULF2 ARSK ARSI ARSG ARSH ARSE SULF1 ARSF ARSB ARSJ

GO_OXIDOREDUCTASE_ACTIVITY_ACTING_ON_PAIRED_DONORS_WITH_INCORPORATION_OR_REDUCTION_OF_MOLECULAR_OXYGEN_REDUCED_FLAVIN_OR_FLAVOPROTEIN_AS_ONE_DONOR_AND_INCORPORATION_OF_ONE_ATOM_OF_OXYGEN Catalysis of an oxidation-reduction (redox) reaction in which hydrogen or electrons are transferred from reduced flavin or flavoprotein and one other donor, and one atom of oxygen is incorporated into one donor. CYP2U1 CYP2D6 CYP4F8 CYP1B1 CYP19A1 CYP2C18 CYP2A13 CYP3A43 CYP2E1 CYP1A1 CYP3A4 CYP4Z1 CYP2S1 CYP2F1 CYP2A6 CYP4B1 CYP2C8 CYP3A5 CYP4F12 CYP2J2 CYP2A7 CYP4X1 CYP3A7 CYP2D7P1 CYP1A2 CYP2B6

GO_MICROTUBULE_BINDING Interacting selectively and non-covalently with microtubules, filaments composed of tubulin monomers. FAM154B FMR1 RAE1 PLK1 EML2 KIF17 MAST2 MAP1LC3A MX1 TPT1 LRRK2 SNCA APC2 TXNDC3 FAM175B REEP4 FAM190B TUBGCP3 CNN3 POLB KIF3B KIF3C KATNB1 KIF6 RACGAP1 MAP4 DYNC1I1 KIF21B MX2 KATNAL1 MAP1B EZR HOOK1 CAMSAP3 NUSAP1 KIF2C SUN2 WDR81 KIF2B KIF4A VAPA TRIM54 DPYSL5 CEP290 UXT C19orf20 TRPV4 CLIP3 TUBGCP5 LZTS1 CDK5RAP2 RAB11A KIF18A RP1 CHD4 MAPRE3 RGS14 KIF15 HDAC6 CAMSAP1 KIF19 MAP1LC3B S100A9 ZNF207 MAP1A KATNAL2 TUBGCP6 MAP1LC3B2 KIF14 WDR52 KIF7 SBDS MAPT CEP57 NEFM MID1 JAKMIP1 CETN1 GAS8 SKA2 PRNP WHAMM STIM1 CLIP2 GAPDH NUMA1 CAMSAP2 FNTA TIAM1 TRAF3IP1 KIF5A C1orf96 KIF13A KIF20B FKSG2 KIF27 GOLGA2 KIF1C GLI1 KLC3 REEP1 CHP KIF13B KIAA1383 WDR43 KATNA1 FES CAPN6 KIF23 ARL3 OGG1 MTUS2 MAPRE1 KIF1A GAS2 CLASP2 EML3 HDGFRP3 OPA1 RCC2 MDM1 PEX14 KIF24 MAPRE2 KIF20A LOC100130097 CENPE TPPP KIF26A MAP6D1 DNM1P34 FGF13 KIF22 NDEL1 MAP1S BIRC5 KIF2A KIF16B VAPB KIF3A JAKMIP2 DNM2 KIF5B STARD9 LRPPRC MACF1 RPS3 SPAG8 GAS2L1 SYBU BCL2L11 KIF25 GAS2L3 FTCD EML4 NDE1 PPP5C APC EML1 CCDC165 GAS2L2 KIFC2 KIF9 MAP7D3 KIF26B DNM1P46 GABARAPL2 CCDC88A DNM1L TUBGCP2 CLASP1 HOOK3 KIF21A CRYAB KIF5C CEP350 KIF4B TUBGCP4 JAKMIP3 FAM154A CEP57L1 NDRG1 PRC1 KIF12 CRIPT FMN1 KRIT1 KIF1B MAP1LC3C KIF18B KIF11 KIFC3 ARHGEF2 DPYSL2 FAM83D MARK4 CETN2 NEIL2 DST SGIP1 MID2 DNM1 VPS41 CLIP1 NEFH SKA1 MAP6 SPAST PSRC1 S100A8 PAFAH1B1 MAP2 KIFC1 DCX DNM3

GO_SULFURIC_ESTER_HYDROLASE_ACTIVITY Catalysis of the reaction: RSO-R' + H2O = RSOOH + R'H. This reaction is the hydrolysis of any sulfuric ester bond, any ester formed from sulfuric acid, O=SO(OH)2. ARSD ARSI ARSK ARSH ARSE GNS ARSF ARSB SULF2 ARSA ARSG STS SGSH SULF1 ARSJ IDS GALNS

GO_HYDROLASE_ACTIVITY_ACTING_ON_CARBON_NITROGEN_BUT_NOT_PEPTIDE_BONDS Catalysis of the hydrolysis of any carbon-nitrogen bond, C-N, with the exception of peptide bonds. CAD PADI6 AMPD2 GLS2 MTA2 ACR APOBEC2 ASPG ACER3 PIGL ARID4B SALL1 APOBEC3F BTD SIN3B BRMS1L WDYHV1 ARG2 ADAL HDAC9 FAAH DCTD SIRT2 RBBP4 ADARB1 PADI1 PGLYRP3 HDAC2 NAAA ADAD2 ADAR ADAT2 DPYS PADI3 ACER2 ARID4A OPLAH ASRGL1 CAT RBBP7 AMDHD2 NIT2 ACY1 HDAC5 CRMP1 NACC2 AFMID HMG20B NGLY1 ASPA SAP30 DPYSL2 PADI2 PDF MTHFD2 ACER1 PGLYRP1 APOBEC3A NDST2 SAP30L KDM1A AMDHD1 APOBEC3G DPYSL3 NAALAD2 MTA1 FAAH2 HDAC3 APOBEC3D CECR1 CD101 VNN2 ADAT1 HDAC8 UPB1 SIRT6 GCH1 ASAH1 HDAC10 AGA PADI4 MTHFD1L RCOR1 ALLC NIT1 ACY3 AMPD1 DARS DPYSL4 ADARB2 APOBEC1 HDAC4 MBD3 SIRT7 PGLYRP2 ZBP1 YDJC AICDA KLK3 PHF21A HDAC1 ARG1 SIRT4 GDA DPYSL5 MTHFD1 ATIC ADAD1 MTHFD2L APOBEC3B DDAH2 SIRT3 HDAC7 CHD4 HDAC6 NTAN1 CDA AMPD3 GLS NADSYN1 SAP18 DDAH1 CHD3 SUDS3 SIN3A MTA3 VNN1 ADA CPS1 SIRT5 SIRT1 VNN3 AGMAT BRMS1 APOBEC3H APOBEC4 APOBEC3C HDAC11 NDST1 PGLYRP4 ASAH2 REST

GO_PROTEIN_PHOSPHATASE_1_BINDING Interacting selectively and non-covalently with the enzyme protein phosphatase 1. PPP1R15A FER TCTEX1D4 PHACTR4 CDC5L KCNQ1 PPP1CA PPP1R39 PPP1R9A SHOC2 PPP1R9B LILRB1 PHACTR1 STAU1 LILRB2 PHACTR3 AKAP11 PPP1CC

GO_KINASE_ACTIVITY Catalysis of the transfer of a phosphate group, usually from ATP, to a substrate molecule. FGF19 WEE2 AK5 IPPK PAPSS2 FYN SMG1 FUK TRIO DOLK GALK2 PDGFRL PMS2P1 TESK2 STK32B MYLK2 MAP3K4 ITPKA EPHB1 IRAK2 MAP3K11 PAK6 OBSCN TLK1 BCR PRPS1L1 PKLR PIP5K1A DCLK2 MAP3K14 PIK3CG PASK ERBB4 GK STK36 SPEG STK16 PGM2L1 NPRL2 ITPK1 KCNH4 HK3 ADCK1 CCND3 FN3K STK17A MAP2K2 CHEK2 BMX PIK3R1 HMGA2 KALRN NEK8 C9orf96 TRIB2 GRB2 ADRBK1 CHEK1 CAD EIF2AK3 CSNK1E FGFR1OP NME1 NRG1 CDK11B PIK3R2 LRRK1 MST4 ERLIN2 MAP2K1 SGK196 ALDH18A1 EEF2K CALM3 CDC42BPA CDKL3 PKMYT1 EIF2AK4 CAMK1D GTF2H2 RBKS RYK PFKFB3 CAMKK2 PIP5K1B MTOR PTPN11 GUCY2C MAP3K8 RPS6KA1 CDKN1A WEE1 EPHB3 ITPKB GUK1 FASTKD2 EIF2AK2 FLT3 CDK20 STK10 MAP4K5 CERKL CAMK1 AKT3 CCNB2 TLR9 NIM1 SGK3 TBRG4 SGK110 FGF5 IP6K3 CRIM1 CSNK1G3 MMD2 MKNK1 PRKAG1 KCNH3 DGKQ CDK3 BLK PRKCA NEK3 MAPK13 EGFR IRS2 PAPSS1 DAPK2 VRK1 STK40 C21orf7 AKD1 NRG4 MVK YES1 ITK DMPK IKBKE EPHA2 KCNH8 PIP4K2A CCNT1 IKBKB AKT1 NRBP1 AURKB PRKAG2 MARK1 HIPK1 NPR1 PCK1 CSNK1A1 DGKE RPS6KC1 AURKC LTBP1 ACVR2A NME9 BMPR1B PLK1 ACVRL1 GRK7 UCKL1 LIMK1 MLKL GRK5 GK5 CIITA VAV1 LATS1 PI4KB ADCK3 EPHA3 CAMK2A TLK2 MAPK1 KIAA1804 FAM20C RPS6KB1 PDK3 C9orf103 PDK4 MYLK CCNT2 TAF1L TRRAP FRK CAMKV PI4K2B EPHA7 MAP3K15 MNAT1 NME3 TWF1 RPS6KB2 TEC STK4 KCNH2 STYK1 CUX1 TXK TGFBR1 AAK1 KLB STAT5A ICK TK2 ERN1 IGF1R PFKFB4 CDK9 GUCY2D IRAK4 CSNK2A2 CSNK1G1 PDGFB AURKA CDK7 RET DCK PTK7 PDK2 NEK4 CDK13 DDR1 RPS6KA5 WNK1 AK3 MYO3B FRS2 FLT4 PIK3R6 MAP2K5 EPHA8 TBK1 MAPKAPK3 LTBP4 GNE AKAP13 MAP2K4 CCNH VPRBP PLK5 CKMT2 AK7 STK3 PEAK1 FGF1 RELN ACVR1 DGKK PSKH1 MPP3 NRG2 STK32A MARK3 ADK SCYL3 CKMT1B BAZ1B CSF2RB SPHK2 PRKCB PHKB PRKAB1 TYK2 GTF2H1 TRIM28 PIM3 ULK4 CMPK1 LIMK2 CAMK4 STRADB MAST1 TNNI3K KL PIP4K2C CCL3 RNASEL CSF2 FASTKD5 ROCK1 MAPK15 SIK1 CSNK1D PRKX TTN GUCY2F EPHB4 ATM DCLK1 PKN3 FGF6 SEPHS1 TNIK BCKDK WNK3 PMVK SRPK1 DAK TK1 PAK2 CKMT1A SRMS DYRK1A NUAK2 DDR2 PNCK CSNK2B PKN2 PFKFB2 PSTK CCL5 DLG3 IP6K2 GSK3B RIPK3 DLG2 GTF2H3 ITGB1BP3 DAPK1 IRAK1 ADCK5 RAF1 DGKD MAP4K4 FASTKD1 IRS1 CSF2RA PSKH2 PIP4K2B BRSK1 MARK2 SGMS1 NOL9 STK11 HKDC1 SGK223 MAPK12 MAP3K7 SIK3 MAP3K3 ZAK AXL PIK3C2B N4BP2 JAK1 SCYL1 FGFR4 LCK PIK3CB PRKAB2 SPHK1 CHKA NRP2 MOS VRK2 YSK4 CSF1R DYRK2 TXNDC3 CERK TSSK6 PANK4 PRKG2 PI4K2A LTK CDK6 ULK1 DYRK3 PINK1 NRP1 PRKAA2 CDK18 CDK8 FGF8 PRKD3 CD86 FASTKD3 PAK4 EPHA10 DCAKD TRIM24 TBCK INSR ERBB2 PANK2 FGF3 WNK2 IL3 CMPK2 CARD11 ARAF PIK3R4 STK25 TNK2 RIOK2 RIPK2 IRAK3 HK1 ACVR1C PRKAA1 EREG PRKY RAD50 RIOK3 TAOK3 MAPK9 FGF16 GAK SEPHS2 PFKP KSR1 NEK7 NTRK1 SRPK2 MAP4K2 STK33 ULK2 FER CCL8 EFEMP1 FGF22 SQSTM1 FGF9 DGKI FGFR1 CDK10 GSG2 VRK3 ROCK2 AGPHD1 CDC42BPG PTK6 DSTYK MARK4 ALK TEX14 FGFR1OP2 ERN2 KSR2 PRKACG PPIP5K1 C19orf35 DCX RIPK4 LMTK2 SBK1 MOK TSSK4 PLK2 CLK2 STK38 PDPK1 MATK UHMK1 EFNA3 FAM20B CKB PKDCC MAP2K7 CD28 EGF MAK CDK14 PNKP NME2 CD80 TAF1 PTK2 ACVR1B PRKACA DGKA NT5C2 PIP5KL1 ABL2 GK2 MYLK3 DYRK4 TRIB3 CDKL4 XYLB FGF23 PGK2 PPP4C PPIP5K2 CKM ETNK2 ALPK1 KHK TEK MAP4K3 FGF18 EFNB3 NEK1 SGK494 CCL2 MAST3 MYLK4 ADPGK DTYMK PIK3C2G FGF7 PIPSL BUB1 CDK17 ADCK2 AK8 PHKA2 SRPK3 CALM1 EPHB2 SRC STK32C NME4 CHUK AK1 NTRK2 PDGFA TRPM7 BRAF CDK11A RIOK1 MKNK2 NPTN DAPK3 HSP90AA1 CDK15 MAP3K6 TSSK2 RPS6KA4 GRK1 AMHR2 STK39 TIE1 PBK ACVR2B IGF2R JAK3 MAPK14 AGK HSPB8 CDC7 SIK2 SHPK ERBB3 PRKCI OXSR1 CSNK1A1L CIT ERCC2 CSNK1G2 CCND1 CDKL5 FGFR3 DGUOK FGFR2 MAP3K1 EPHA1 FASTK PRPF4B XRCC6BP1 PRKCH CDK19 ANKK1 FGFRL1 PFKL EPHA4 CLK1 CDK12 CDC37 MAST2 MAP4K1 LMTK3 NEK6 KIT HIPK2 MAPK8 NME2P1 IP6K1 PRKACB TRPM6 PRPS2 PHKA1 TTBK1 ZAP70 CPNE3 CDK4 PAK7 TP53RK CDC42BPB MST1R LYN PHKG1 MAGI3 IL5RA DGKH UCK2 TRAT1 EFNA4 CCNB1 TJP2 TGFB2 MPP1 AK2 TTBK2 NAGK SNX15 NME7 ULK3 PIK3CD MYO3A NME5 NADK BUB1B TSSK3 PHKG2 ALPK3 PIK3C2A HMGXB3 STRADA PRKCG PKM2 DGKB MUSK PANK1 PRPS1 ATR CHKB PLK4 STK38L HUNK CDKL2 NEK9 CLK3 AVP NUAK1 PIKFYVE TAOK1 CDK5R1 CAMK2G TGFBR3 FGF10 PXK ROR1 ABL1 PRKDC NEK11 PDXK KDR PIK3R3 BMPR1A FGF2 HK2 HIPK4 RPS6KA2 JAK2 PRKCZ PLK3 RASSF2 PDIK1L MAP3K12 PDGFRB C8orf44-SGK3 MAP2K3 RIPK1 IPMK PIM2 DGKG STK24 SOX9 MASTL ROS1 PRKCE AKT2 MAPK11 CAB39 CAMK2B PTK2B CD19 NME6 AATK OSR1 ILK RPS6KA3 PIK3C3 TGFBR2 FES MMD ERCC3 IL3RA PAK1 ROR2 MAPKAPK5 CSNK2A1 CSK COL4A3BP HFE2 GRK4 HBEGF SYK UCK1 MPP2 EIF2AK1 TNK1 PAK3 HCK MAP2K6 RFK RPS6KL1 DLG1 MAPK10 CASK GCK WNK4 PFKFB1 CDK2 PRKCQ ALPK2 PFKM GLYCTK AK4 NRBP2 BTK PRKD2 IL5 MAP3K10 PRKAG3 GALK1 PRKD1 ZMYM2 CAMKK1 PIP5K1C FGF17 DLG4 MAP3K9 CDKL1 MELK ITPKC EPHA6 CNTRL GRK6 PDGFRA CLP1 MET TPK1 FGF4 MAP3K2 NADKD1 TYRO3 ETNK1 NEK5 FGF20 C9orf95 BMP2K GAB1 HIPK3 SLK TRIB1 SBK2 TRIM27 PI4KAP2 ADRBK2 KCNH1 PIM1 CAMK2D MAPK7 GSK3A SNRK EPHB6 MAST4 BRSK2 BMPR2 CALM2 KITLG MAPK6 PAN3 RPS6KA6 DGKZ STAT5B FN3KRP MINK1 PI4KA TSSK1B CLK4 SGK1 LATS2 NTRK3 FAM20A KCNH5 SGK2 PRKCD CAMK1G PRKG1 CCNC MERTK MAPK3 PI4KAP1 NRK DYRK1B PIK3CA DCLK3 LRRK2 MAP3K5 NEK2 ADCK4 STK31 SCYL2 MAP3K13 COASY FLT1 UPRT PGK1 PIK3R5 CDK5 NLK PDK1 CCNK CDK1 CCNE1 INSRR MAPK4 CDK16 TTK TESK1 PKN1 FGR STK17B MAPKAPK2 FGGY PANK3 EPHA5 SGMS2 GTF2H4 TAOK2 NPR2 LRGUK NEK10 NAGS STK35 STK19 BTC

GO_PHOSPHATIDYLINOSITOL_3_KINASE_ACTIVITY Catalysis of the reaction: ATP + a phosphatidylinositol = ADP + a phosphatidylinositol 3-phosphate. This reaction is the addition of a phosphate group to phosphatidylinositol or one of its phosphorylated derivatives at the 3' position of the inositol ring. PIK3R1 FGF1 FGFR2 CD19 GRB2 NRG2 FGFR1 ERBB3 FGF22 ERBB4 FGF9 PIK3CG FGFR3 KITLG IRS1 PDGFRB IRS2 FGF10 GAB1 EGFR FGF2 NRG4 FRS2 PIK3R6 PIK3R3 FGF4 FYN PDGFRA PIK3C2A EREG FGF19 FGF16 FGF20 PDGFA PDGFB ATM ERBB2 FGF3 PIK3C2G KLB BTC FGF5 FGF6 TLR9 PIK3R4 FGF17 FGF7 PIK3CD FGF23 FGF8 FGF18 TRAT1 CD86 PIK3R5 CD80 KIT CD28 EGF PTPN11 HBEGF KL NRG1 PIK3C2B PIK3C3 VAV1 PIK3CA PIK3CB PIK3R2 FGFR4 LCK

GO_CYCLIN_DEPENDENT_PROTEIN_KINASE_ACTIVITY Catalysis of the phosphorylation of an amino acid residue in a protein, usually according to the reaction: a protein + ATP = a phosphoprotein + ADP. This reaction requires the binding of a regulatory cyclin subunit and full activity requires stimulatory phosphorylation by a CDK-activating kinase (CAK). CDK2 CDK20 ICK CDK19 CCNB2 CDK9 CDK17 CDKL1 CDK1 CDK18 CDK8 CCNK CDKL4 CDK16 CDK10 CCND3 CDKL5 CCNB1 CDK14 MAK CDK5R1 CDKL3 CDK13 CDK15 CDK5 CDK6 CDK4 MOK CDK12 CDK11B CDK7 CDKL2 CDK3 CDK11A

GO_RECEPTOR_SIGNALING_COMPLEX_SCAFFOLD_ACTIVITY Functions to provide a physical support for the assembly of a multiprotein receptor signaling complex. GRIP1 RGS14 MAPK8IP3 CD3G GRIP2 G3BP2 AXIN1 HOMER2 LRRK2 SPAG9 SHANK1 NUP62 DLG5 CD3E ROPN1B NCK2 SHANK2 LDLRAP1 CARD10 MAGI2 MMS19 NCK1 DEDD2 SHANK3

GO_COFACTOR_BINDING Interacting selectively and non-covalently with a cofactor, a substance that is required for the activity of an enzyme or other protein. Cofactors may be inorganic, such as the metal atoms zinc, iron, and copper in certain forms, or organic, in which case they are referred to as coenzymes. Cofactors may either be bound tightly to active sites or bind loosely with the substrate. GLDC AADAT SDHD ETFDH PPOX SIRT3 QDPR SDS XDH DUS1L PNPO GSR NDUFS2 IDH3A ACOT7 ACOXL ACADSB NOX5 CYB5R2 SRR NNT NOS1 KCNAB1 ACBD6 HMGCL TYMS MTHFR FMO5 TAT PHGDH KMO MMACHC GAPDH MAOB MICAL1 GAD1 CTBP1 ACADS NOX4 ASNS MOCOS BCAT1 TKT CRYZ SUCLG1 CHDH NFS1 SQLE HAO2 SIRT6 DBI GCH1 DHFR CTH DHFRP1 GAPDHS SIRT4 AOX1 NDOR1 ABP1 GAD2 DECR1 AOC3 FOXRED2 ACOX3 CCBL1 NDUFV1 GADL1 SGPL1 CREG2 AOC2 GCDH ACOX1 IDH2 GPD1L CAT ACOX2 PDXK ADH4 GPD1 GPT AGXT SIRT2 PDXDC2P PGAM2 ECI2 SPR POR HDC CYBB PRODH ASPDH CRY2 NOS3 OXNAD1 GPT2 OGDHL GOT1 GOT2 AGXT2L1 UROS ILVBL SDHA ACAD10 TXNRD2 AIFM1 MTRR SHMT2 GCAT SPTLC2 AIFM2 FMO1 DHODH DPYD CCBL2 CYB5RL IVD DUOX1 PYGM SORD COQ6 SPTLC1 WWOX LMO2 MDH1 LDHA AIFM3 TYW1 SOAT1 AGXT2 AGPS SDSL MOSC1 PARP1 ME2 CREG1 ACADVL MOSC2 DUS4L CRYZL1 IDH1 IDH3B ABAT NDUFS7 OAT FMO2 ALAS1 FMO3 ACADM UGDH HIBADH ALDH1A3 CYB5R4 TXNRD1 DHCR24 DHCR7 ETFA ALAS2 SIRT1 THNSL1 SIRT5 ACBD4 HIF1AN ACAD11 BCAT2 HACL1 GLYR1 CBS KYNU BDH2 DHFRL1 TM7SF2 FASN TSTA3 G6PD THNSL2 KDM1B HMGCR CSAD HSD17B8 SOAT2 ME3 HADH ACBD5 PYGB PYGL ACBD3 TXNRD3 SRD5A1 VKORC1 TDH DDC HHIP SIRT7 ACADL CBR4 VKORC1L1 TYW1B ALB OGDH HPGD PDXDC1 ACBD7 PHYH ACCS HAO1 DDO CBR3 AHCY ACAT1 NOS2 TH D2HGDH NOX1 UXS1 ACCSL KDM1A CYB5R3 SCP2 GCLC ACAD9 CRYM LDHD GLUD1 GRHPR SPTLC3 ATAT1 HHIPL1 DUS3L NDUFAB1 CTBP2 ACLY MTO1 TP53I3 FMO4 ALDH6A1 HSD11B2 SUOX GOT1L1 PROSC DAO DUS2L CYB5R1 DHTKD1 NDUFA9 GFER MICAL3 AGXT2L2 HHIPL2 GMDS SDHB SHMT1 HADHA H6PD MICAL2 SQRDL LDHB DLD ME1 CRYL1 ACAD8

GO_TRANSMEMBRANE_RECEPTOR_PROTEIN_TYROSINE_KINASE_ACTIVITY Combining with a signal and transmitting the signal from one side of the membrane to the other to initiate a change in cell activity by catalysis of the reaction: ATP + a protein-L-tyrosine = ADP + a protein-L-tyrosine phosphate. INSR EFNB3 EPHB4 ERBB2 IGF1R INSRR EPHB3 EFNA4 TEK EPHA5 FLT3 EPHA10 CSF1R ROR2 KIT FLT1 EPHA7 RYK LTK MST1R NRP1 MERTK EFNA3 FGFR4 EPHA3 NRP2 ROS1 EPHA1 FGFR2 ALK NTRK3 AXL EPHA4 FGFRL1 EFEMP1 EPHA8 FGFR1 EPHB6 ERBB4 PDGFRB EPHA2 FGFR3 EGFR NPTN PDGFRL DDR2 NTRK1 DDR1 ROR1 TRIM27 KDR TIE1 EPHB1 IGF2R FLT4 EPHA6 CRIM1 MUSK EPHB2 MET PDGFRA TYRO3 RET NTRK2

GO_BETA_TUBULIN_BINDING Interacting selectively and non-covalently with the microtubule constituent protein beta-tubulin. EMD TBCA CCT5 TBCD SIRT2 SLC6A2 BCAS3 GJA1 ARL8B C1orf88 HDAC6 GABARAPL2 SNCA ARL8A RGS2 PDCD5 NDEL1 MAP1S PACRG UXT VAPB HTT SYT11 LRPPRC SPAST TRPV4 RACGAP1 BBS4 PEX14 ADNP IFT74 TTLL7 FGF13 GABARAPL1

GO_PHOSPHATASE_REGULATOR_ACTIVITY Modulates the activity of a phosphatase, an enzyme which catalyzes of the removal of a phosphate group from a substrate molecule. SBF2 PPP2R3A FRS2 RCAN2 ANKLE2 BMP2K PPP1R8 PPP2R5B PPP1R16B PPP1R3B PPP1R2P1 ELFN2 PPP2R3B PPME1 PPP1R12B PPP1R1B PPP1R9B PPP1R3C MTMR12 BMP2 PPP1R12A PPP4R2 CALM1 SET SAG PPP1R15A PPP1R16A PPP1R36 PHACTR4 PPP1R35 LMTK2 PPP4R4 PPP2R5A IGFBP3 PTN PPP1R14C PPP1R2P3 ANP32E CALM2 C19orf2 DMPK CABIN1 PPP2R5C PHACTR3 PPP1R14B PPP2R1B ZEB2 PPP2R2C CALM3 ARPP19 PPP2R4 PPP2R1A PHACTR2 PPP1R2P9 WBP11 PPP1R17 PPP2R2A PHACTR1 PPP1R37 PPP1R11 PPP1R27 B3GAT3 RCAN3 PPP1R10 PPP1R14D TESC PPP2R2D PPP4R1 ELFN1 PPP2R5E PPP1R14A PPP1R39 PPP2R2B PPP2R5D GTF2F1 ENSA IGBP1 PPP1R1C SHOC2 EIF2AK2 PPP1R2 RCAN1 SBF1 PPP1R1A PPP1R7 PPP1R26

GO_FATTY_ACID_SYNTHASE_ACTIVITY Catalysis of the reaction: acetyl-CoA + n malonyl-CoA + 2n NADPH + 2n H+ = long-chain fatty acid + n+1 CoA + n CO2 + 2n NADP+. MCAT ELOVL4 ELOVL5 ELOVL7 ELOVL1 ELOVL3 OXSM OLAH ELOVL2 ELOVL6 FASN

GO_ORGANOPHOSPHATE_ESTER_TRANSMEMBRANE_TRANSPORTER_ACTIVITY Enables the transfer of organophosphate esters from one side of a membrane to the other. Organophosphate esters are small organic molecules containing phosphate ester bonds. SLC37A4 SLC37A1 SLC25A19 SLC35B2 SLC25A24 SLC25A33 SLC37A2 SLC25A36 SLC25A17 SLC35B3 SLC25A42 SLC25A6 SLC37A3 ABCC11

GO_FUCOSYLTRANSFERASE_ACTIVITY Catalysis of the transfer of a fucosyl group to an acceptor molecule, typically another carbohydrate or a lipid. POFUT1 FUT3 FUT9 POFUT2 FUT2 FUT7 FUT5 FUT11 FUT8 FUT1 FUT4 FUT6 FUT10

GO_BINDING_BRIDGING The binding activity of a molecule that brings together two or more molecules through a selective, non-covalent, often stoichiometric interaction, permitting those molecules to function in a coordinated way. FSCN2 NCK2 CNTNAP1 SLC9A1 AP2A2 TRIM17 COL19A1 TP53BP2 BIN3 SLA ARHGAP6 EVPL VPS11 CRKL CNKSR1 AP2M1 IRS1 SPAG9 GAB1 NUFIP1 NEFL CLNK COL11A2 SRC TRIM22 ANK1 PPP4R2 TRIM5 RBM14 SPRR1B FLRT2 EPS8 AFAP1L2 SH2D3A FGG FGA TIRAP MPP7 COL8A2 NCK1 ARHGAP1 DDX20 IRS4 SORBS2 SKAP2 CHN1 SH3BGR SPRR1A PDCD6 BAIAP2 DVL3 TRADD CSTA TRAT1 FCRL2 GRAP CUX1 TJP2 FLRT3 MEN1 ARHGAP4 TOB1 HSH2D ANK2 CRK CAV1 SORBS1 DSP COL11A1 CHN2 BLNK COL14A1 DAB2IP GAB2 BAIAP2L1 SKAP1 STAM MAPK8IP3 SOCS2 FSCN3 DOK2 TNNT2 SH2D3C ANK3 SH3BGRL GRB7 SLA2 LAT VPS18 SH2B3 BAIAP2L2 RPTOR PIK3R1 KHDRBS1 BCL3 AMFR COL1A2 NEFH GNB2L1 SHC1 GRB2 MAPK8IP2 DAB2 SDCBP NMD3 PICALM DUSP19 FSCN1 AKAP13 SH2D2A GRB14 FKBP4 HOMER1 STAP1 ARRB1 CNTLN ABI2 KSR1 C14orf1 GRAP2 FGB CRADD CBX5 AP2A1 CAV2 FRMD4A RUSC1 FRS2 ST13 TEX261 ITSN2 GAS2L1 STUB1 RAD50 SH2B2 ANXA1 PSMC6 GRB10 FBXW7 LDLRAP1 IVL WWC1 SHB SH2B1 TCAP LASP1 BFAR STX1A SH3BP2 TRDN FLRT1 DVL2 SH2D1B LOR SH2D1A CD28 MAPK8IP1 VAV3 OBSL1 ARPC4 PTPN11 MMS19 BICD1 AP2B1 GATAD2A GAS6 PAG1 TRIM6 OPTN ADAP2 AP2S1

GO_CATION_TRANSPORTING_ATPASE_ACTIVITY Catalysis of the transfer of a solute or solutes from one side of a membrane to the other according to the reaction: ATP + H2O + cation(out) = ADP + phosphate + cation(in). ATP5O ATP1A3 ATP6AP1 ATP2A3 ATP7B ATP2A1 ATP5EP2 ATP1B2 ATP6V1E2 ABCB11 ATP6V0A1 ATP6V0A2 ATP2A2 ATP5G2 ATP1A2 ATP5C1 ATP2B3 ATP5B ATP6V0D1 ATP13A1 ATP6V0E1 ATP5E ATP1B3 ATP5G3 ATP6V1B2 TCIRG1 ATP13A5 ATP13A2 ATP6V1C1 ATP6V1F ATP1B1 ATP1A1 ATP4B ATP6V1A ATP5G1 ATP6V0B ATP8A1 FXYD2 ATP6AP1L ATP6V1G1 ATP5A1 ATP6V1C2 ATP13A4 ATP1A4 ATP6V1G2 ATP4A ATP6V0A4 ATP5H ATP6V0C ATP7A ATP6V0E2 ATP6V1G3 ATP2B4 ATP6V1H ATP2B2 ATP2B1 ATP6V1E1 ATP13A3 ATP5D ATP12A ATP2C2 ATP5F1 ATP2C1

GO_G_PROTEIN_COUPLED_PHOTORECEPTOR_ACTIVITY Combining with incidental electromagnetic radiation, particularly visible light, and transmitting the signal across the membrane by activating an associated G-protein; promotes the exchange of GDP for GTP on the alpha subunit of a heterotrimeric G-protein complex. GNAT2 OPN1MW2 OPN1MW OPN5 OPN3 ELOVL4 OPN1LW OPN4 RRH RHO OPN1SW

GO_RNA_POLYMERASE_II_TRANSCRIPTION_COREPRESSOR_ACTIVITY Interacting selectively and non-covalently with an RNA polymerase II repressing transcription factor and also with the RNA polymerase II basal transcription machinery in order to stop, prevent, or reduce the frequency, rate or extent of transcription. Cofactors generally do not bind DNA, but rather mediate protein-protein interactions between repressive transcription factors and the basal transcription machinery. ELANE BEND6 TBX15 TLE1 SOX3 C19orf2 OLIG3 TFAP2B ZNF451 SIN3A SIN3B RBBP8 HDGF TCP10L TCERG1 TBX18 HEYL HDAC1 CTBP1 RERE ZMYND8 CITED2 N4BP2L2 UXT PHF12

GO_FIBROBLAST_GROWTH_FACTOR_BINDING Interacting selectively and non-covalently with a fibroblast growth factor. FGFBP1 GPC1 FGFR1 SCN5A RPS2 FGFR3 GLG1 FIBP FGFR4 ITGB3 RPS19 CXCL13 FGFBP3 ITGAV API5 TGFBR3 S100A13 FGFR2 KLB THBS1 FGFRL1 CEP57 KL

GO_KINASE_ACTIVATOR_ACTIVITY Binds to and increases the activity of a kinase, an enzyme which catalyzes of the transfer of a phosphate group, usually from ATP, to a substrate molecule. DAXX GHRL NRG3 MAP2K1 PIK3CA TAB1 EFNA5 EPO ABI1 ANGPT4 GAS6 NRG1 ITSN1 GPRC5B EGF CALM3 GREM1 ERCC6 CKS2 FGF13 TGFB1 WNT11 CKS1B CDKN1A STK4 MT3 FAM58A CDK5R2 NKX3-1 C13orf15 IGF2 TOM1L1 PAK2 BCL10 AFAP1L2 CD24 RPLP1 FAM150B IL2 CALM1 STRADA CCL5 WNK1 DBF4B LAMTOR3 CDK5R1 FAM150A PRKAG2 CALM2 PARP16 LTF SPRY2 DUSP19 ERBB3 FAM20A STK11 MALT1 MAP2K2 MOB1B IQGAP1 STK3 RPTOR ALS2 MARK2 CAB39 MADD NCKAP1L

GO_CATION_CATION_ANTIPORTER_ACTIVITY Catalysis of the transfer of a solute or solutes from one side of a membrane to the other according to the reaction: cation A(out) + cation B(in) = cation A(in) + cation B(out). SLC8A2 SLC9A1 SLC8A3 SLC24A3 SLC41A1 SLC9A6 SLC22A4 SLC9A3 SLC9A9 SLC9B2 SLC24A4 SLC24A5 SLC9A7 SLC9A11 SLC11A1 SLC9A4 SLC47A1 SLC9A5 SLC24A1 SLC9A10 SLC22A5 SLC3A2 SLC8A1 SLC9A2 SLC9B1 SLC24A6 SLC24A2 SLC9A8

GO_LYSINE_N_METHYLTRANSFERASE_ACTIVITY Catalysis of the transfer of a methyl group from S-adenosyl-L-methionine to the epsilon-amino group of a lysine residue. SMYD1 WHSC1L1 PRDM2 MEN1 SMYD3 FAM86A CAMKMT WDR82 SUV420H2 EZH2 DYDC1 ASH2L PRDM9 NSD1 SETD2 SETD6 WDR5 ASH1L SETD8 PRDM7 SUV39H1 MLL3 PRDM6 WHSC1 SETDB2 MLL2 IRF4 SETDB1 MLL5 METTL20 DYDC2 SETD3 SMYD2 SETD4 EHMT1 PRDM16 SUV420H1 METTL21D METTL21A C5orf35 RBBP5 METTL10 METTL21C MECOM SUV39H2 CXXC1 DPY30 EZH1 MLL MLL4 SETMAR N6AMT2 SETD7 DOT1L SETD1A EHMT2 SETD1B

GO_RIBONUCLEOTIDE_BINDING Interacting selectively and non-covalently with a ribonucleotide, any compound consisting of a ribonucleoside that is esterified with (ortho)phosphate or an oligophosphate at any hydroxyl group on the ribose moiety. TTN SUCLG2 ABCA6 PRKX MYH7 DHX32 DHX35 DYNC1LI1 ROCK1 SIK1 HSPA7 GTPBP4 YARS2 RAB42 GUCY2F GNAL RUNX2 KIF16B ATM TYK2 SCN8A MYH11 HYOU1 DNAH2 UBE2N SCYL3 ADK MRAS PRKCB EIF5B UBA2 SPHK2 WRN PARS2 TNNI3K PIP4K2C RAPGEF4 DDX55 CMPK1 ATL1 ULK4 DDX59 C9orf102 PSMC3 SYN3 ADCY3 TRMU LIMK2 KIF1B RAB8B RUVBL1 ACSM4 VPRBP PLK5 MAP2K4 AGAP1 EPHA8 MCCC1 RHOD MYH13 MAPKAPK3 ARL2 TBK1 FARSB CCT8 RAB40B DHX37 ABCC13 AK7 TOR1A P2RX7 DGKK GNL3L ACVR1 ADCY2 ABCG5 AURKA ARL8A SEPT5 DCK KIF26B ACSL4 RAC2 ADCY6 LACE1 CACNA1B UBE2R2 RAB1C FLT4 XRCC2 NOA1 GIMAP4 PTK7 PDK2 DNM1L DHX36 TPX2 TRIT1 RRAGC RPS6KA5 DDR1 RUNX3 ATP10A HCN2 CDK13 ATP6V1B1 NLRP14 EARS2 DDX53 RAB7L1 SEPT14 CBWD3 PRKAA2 PRKD3 RAB7B STK25 ARAF TUBG2 RASD2 NLRP6 SEPT4 UBE2J2 LIG3 INSR TBCK KIF2B RAP2B WNK2 GNAT1 C20orf152 DHX9 RAB1B MCM7 LARS2 N4BP2 FPGT LTK RAB27B DDX11 UBA3 PRKG2 ATP9B ATP8B3 ACTA2 GBP2 HSPA12A YSK4 DYRK2 CSF1R MOS TSSK6 GIMAP6 IRAK1 RAF1 SMCHD1 ACSL5 SPG7 TUBB8 ATP4A IP6K2 RIPK3 DAPK1 ITGB1BP3 RHOH SGK223 MAP3K7 SLC22A4 KIF13A MOV10L1 MAP3K3 RARS2 SIK3 HSP90AA5P PSKH2 ABCC12 KCNT2 PIP4K2B DNAH1 MARK2 BRSK1 ARL5B ACTA1 NUAK2 DYRK1A ABCA13 SRMS ARL6 BCKDK MKKS PDE5A KIF26A DNAH8 MOV10 FLT3 DNAH14 EIF2AK2 SRCAP RPS6KA1 TTLL5 GPN1 GUCY2C EPHB3 ITPKB NLRP2 NUDT2 DDX23 NIM1 GUCY1A2 DNM2 ATP12A IP6K3 YTHDC2 SGK3 SGK110 UBE2G2 CDK20 DNAJA1 RAB5A AKT3 CAMK1 GNA14 LRRK1 RAB35 CCT3 P2RX1 GFM1 SLC27A2 EIF2AK3 DDX39B CBWD6 AGAP3 SMC2 MYO7B PIP5K1B SYN1 CAMKK2 PFKFB3 KIF1A MTOR CDKL3 BMS1 SPATA5L1 CDC42BPA RASL10A EIF2AK4 HSPH1 ARL3 TOP2B ARHGAP35 RAB5C ADCK1 STK16 SEPT9 ARL5C DHX16 ARFIP2 ACLY OASL CLCN5 RP2 PIK3CG PGS1 GK SPEG STK36 RAB2B GIMAP5 GNAT2 ABCA12 HELQ ADRBK1 ATP2B1 UBE2D4 STK17A LIG1 HSPA2 MYO1E NMNAT1 IRGC NOS2 RAB3A UBE2I GALK2 HSPA8 AK5 FARS2 GATC EP400 SLFN11 CNGB3 FYN SMG1 DDX41 NAV3 PAPSS2 PAK6 GUCY1A3 KIF21A ATPBD4 ITPKA PKLR DCLK2 C8orf80 RASL11B BCR PRPS1L1 ABCC6 NLRP7 RAB27A EEF1A2 TYW1B DNAH9 RAB9B DDX49 TGFBR1 RERG STK4 TUFM PSMC4 AARS2 TTLL3 TEC UBE2B STYK1 UBE2G1 PFKFB4 DDX18 GTF2F2 BBS12 ATP5D GNL1 IRAK4 CCT6A ERN1 RASD1 ICK RAD17 SMC5 GK5 EPHA3 BBS10 LATS1 ADCK3 ABCD2 GRK7 DDX51 PLK1 MYH6 NLRP4 RAB2A RECQL4 GPN2 MYO5B P2RX4 ERAL1 MLKL CNGA4 UBA7 DDX27 NLRP3 KIF3B DNAH11 ACSF3 RAB17 NME3 PCCB ARL10 HLCS KIAA1804 TLK2 ENTPD1 PDK3 PDK4 UBE2T PIP4K2A UBE2D1 BCS1L PAPD4 MCM8 NLRP5 PRKAG2 RAB37 MVK YES1 TARS RAB41 IFIH1 MTG1 IRGQ TIMM44 ATP13A3 MARK1 DGKE TUBA4A GAL3ST4 NKIRAS2 PCK1 KIF5A IQCA1P1 ARF1 CDK3 ARL4C UBA1 CSNK1G3 UBE4B ASNA1 RAP1B ACACA DHX15 VARS C10orf2 VCP ACSM2B ATP1B1 PAPOLG CREG1 LONP2 ABCG1 NARS DRG1 TOP2A IFT27 MAPK13 PRKCA NEK3 ERAS HCN1 STK40 P2RX6 PAPSS1 RPS6KL1 CASK MAPK10 WNK4 GCK ADCY9 PDE4A DNAJA3 CBWD1 TNK1 YME1L1 KIF24 TOR1B IDE GTPBP1 TYW1 HCK CENPE PDE10A PIP5K1C CDKL1 MELK HSPA1B MAP3K9 PAICS PAPOLA RAB30 PRKAR1A EPB42 PRKD2 C9orf69 MAP3K10 IRGM DHX58 POLQ MYH2 FES TGFBR2 PIK3C3 PSMC2 RAB10 ATP6AP1 RAB7A RAB36 GRK4 MAT2A EIF2AK1 NTPCR UCK1 SYK ROR2 PAK1 CCT8L1P PSMC1 CSNK2A1 MLH1 ABCC8 MAPKAPK5 ACTC1 MAP3K12 ARL5A C8orf44-SGK3 PDIK1L MYO10 IPMK PIM2 ENTPD2 ACSM1 KIFC3 PMS1 RIMKLA KIF12 PRKCZ JAK2 HIPK4 CAMK2B ORC4 CNGA3 ADCY8 PTK2B CHORDC1 ARF6 SRPR ROS1 ATP2B4 ARL9 MYO18A NOS3 STK24 ATP10B AKT2 MMAA MAPK11 ABCG4 POR HUNK PSMC6 RHOV PRPS1 RAB34 SEPT11 RUVBL2 NEK9 CLK3 ATAD5 TRIP13 PHKG2 ALPK3 PKM2 APAF1 DGKB CARNS1 PRKCG TUBAL3 KDR NEK11 PDXK KIF5C BMPR1A DDX24 ACSBG1 CLCN6 UBE2E3 RRAD HCN3 CAMK2G NUAK1 TAOK1 CREG2 RAB15 ABL1 PXK ACSS2 FLAD1 PANK3 EPHA5 MAPKAPK2 ADCY1 KATNAL1 MOCS3 CDK1 DARS ABCB5 MARS DDX20 ABCC2 STK19 SMARCA1 KIF4A DDX4 STK35 PIK3CA UBE2J1 HAO2 ARL8B FPGS GMPS NUDT16 ARHGAP5 PDE2A CAMK1G ATL2 DDX54 TTF2 TTLL4 NRK PRKG1 DDX58 MERTK MAPK3 MFHAS1 ABCB10 PDK1 ASNS SCG5 HCN4 SMC4 SMC3 TTLL11 TRIM23 RAB38 GSS COASY MAP3K13 SCYL2 DDX46 STK31 PGK1 MYH3 FLT1 RHOB UPRT BMPR2 DDX47 SMC1A BRSK2 PAN3 RPS6KA6 DGKZ MFN1 GSK3A ATP1A4 TUBA8 SLFN12 MAPK7 CCT8L2 MAST4 CHST12 DDX3X DHX33 NOS1 LATS2 GBP6 ADSS DHX8 KIF27 INO80 VARS2 EEF1A1P5 EIF4A1 ABCA1 HSPA4 MYH9 TSSK1B MINK1 DNAH6 ATAD2B CHST15 KIF15 GNL2 ATP2A2 RAB31 ETNK1 NADKD1 RAB3C RAB39B EPHA6 TUBB2A TPK1 MET TRIB1 ABCB7 CAMK2D CCT5 ABCB9 C9orf95 HIPK3 BMP2K ACTR2 ALPK1 ME1 ETNK2 CHD8 MTIF2 TEK URGCP KHK PDE3A RAD54B RHOBTB3 TRIB3 MARS2 HFM1 ATP13A4 DYRK4 KIF20A RABL2A THRAP3 CDKL4 TTLL10 PEX1 ACSM3 BUB1 RALB GMPPB CDK17 MAST3 DNAH17 SGK494 UBE2F GPN3 REM2 ITM2C KIF2A SRP54 DTYMK MYLK4 YARS FAM20B TTLL12 PDPK1 RAB39 ARF4 PKDCC MAP2K7 KIF1C CLK2 PLK2 STK38 ABCC11 MYO1G ABCF2 SUCLA2 PRKACA HSPA9 TUBB1 ABL2 ADCY4 MAK PNKP EIF4A3 TUBA3C KCNJ1 LOC100507096 TAF1 SWAP70 VRK3 DDX25 TGM2 SRL GSG2 RAD51B PTK6 KIF11 ERCC6L HSP90B1 EHD3 ACSF2 DGKI FGFR1 SPO11 UBE2QL1 HNRNPU RIMKLB GNL3 MCM5 SBK1 TEX14 DSTYK GNAQ ERN2 CHD7 ZRANB3 ARL17B AGAP2 CUL9 DDX50 ATP2B3 RHOT2 RIOK3 MSH2 FBP1 ACTR3C MYO19 SEPT1 SEPT7 MAPK9 HAO1 HSPA1L TUBA1B CARKD TUT1 WARS RAP2C ATP8B2 RAD50 ATP1A1 CBWD2 ABCB4 GAK PEX6 KSR1 NEK7 PFKP NTRK1 MSH6 ABCA4 UBE2A ACTR1B RND3 MAGI3 MLH3 RAB6C CLCN4 DGKH ULK3 PIK3CD NADK PYGL TTBK2 DHX29 MSH5 P2RX3 NME7 NAGK RAP1BL PAPOLB LMTK3 MAP4K1 NEK6 ACSL1 CDK12 EPRS DHX57 HSPA12B ZAP70 RAB33A ACTBL2 CDC42BPB CDK4 RAB22A RABL5 ACOT12 QRSL1 IP6K1 NME2P1 NVL TTBK1 MSH3 CSNK1A1L PDE4B ACACB FDXACB1 CPS1 TRPV1 GBP5 FGFR3 RHOBTB2 RNF112 ERCC2 CSNK1G2 OAS2 ATP10D OXSR1 RALA DHX40 GTPBP6 CNNM2 C8orf45 EEF2 ANKK1 NOD1 MYO1F THG1L UBE2C EPHA1 FASTK MYO9A ABCA2 CDK19 PRKCH PRPF4B OLA1 PRIC285 AK1 ATP1A2 NME4 CCT6B TRPM7 BRAF EHD4 RAB26 GBP3 KIF18A UBE2U CLPB CHD4 STK32C DIRAS1 ACTG1 STK39 TTLL8 PBK GTPBP3 CHD1L MCM3 AGK ASCC3 MAPK14 RAB3D RAB40C ADSSL1 DAPK3 RIOK1 SARS MAP3K6 DYNC1LI2 XRCC6 RHEBL1 ATP11A RAB28 TP53 ARFRP1 UBA5 APRT HHAT PDE6C EIF4A2 CSNK1D ACTG2 MAPK15 SMARCAD1 TTLL2 KIF5B PKN3 DCLK1 TUBA3E SEPHS1 ABCC5 MFN2 RASEF EPHB4 RAB33B RAB40A SMARCA4 C9orf86 HELZ HSPD1 PCCA GNAT3 CKMT1B BAZ1B MKI67 SPATA5 MTRR DDX12P MAST1 PEBP1 ARL16 SLFN12L UBE2K RNASEL WRNIP1 ABCA9 XRCC5 RNGTT PIM3 CAMK4 HSPA5 CNGA1 STRADB DDX5 ACTR3B GNE FKBP4 NWD1 DNAH5 CKMT2 UPF1 MAP2K5 TEP1 FIGNL1 AARSD1 MVD STK32A ATAD3C SPAST MARK3 MYH10 RAD51D KIFC1 PEAK1 STK3 DNA2 PSKH1 BLM TOR2A CDK7 RHOA ATAD3B ABCG8 KIF9 RET SMC6 RRAGD EFTUD1 CFTR RAB23 CSNK2A2 CSNK1G1 TUBA4B GBP7 MYO3B KIF4B WNK1 AK3 CTPS RASL10B MCM4 NEK4 ACSL6 OPLAH PRKAR2B CLCN7 SLFN13 KIF2C PAK4 CDC6 EPHA10 CDK18 CDK8 NDUFV1 ATP5A1 RAP2A RIT2 ATP2C1 PIK3R4 MTHFD1 TNK2 NDOR1 RIOK2 ARL15 NUBP2 IRAK3 HK1 ACVR1C ABCC4 RIPK2 ATP2B2 DCAKD RFC2 RAD51 CMPK2 ERBB2 PANK2 GTPBP10 ACTB PIK3CB FGFR4 DDX60 SCYL1 DDX19A LCK CHKA GCH1 RAB44 SRPRB MTPAP CHTF18 SPHK1 TUBB4B XRCC3 GART SAR1B SUCLG1 PIK3C2B KIF17 JAK1 RTKN QARS RND2 RFC5 CDK6 PANK4 PI4K2A DYRK3 ULK1 PINK1 SETX KIF3C KIF6 NLRP8 PPP2R4 PC VRK2 ARHGEF5 SKIV2L CERK ATP5B RRM1 GIMAP2 NLRP12 DGKD MAP4K4 HSPA6 AASDH GSK3B POPDC3 ILF2 SRR HKDC1 MAPK12 STK11 GNAS AXL ZAK CHD1 PDS5B HRAS RAB3B ATP8B4 GIMAP1 NOL9 ARL13B CKMT1A TK1 RAB25 PAK2 ABCA10 ABCC10 TUBA3D TDRD9 WNK3 PMVK TNIK RAB11A ARL4A DAK MOCS1 C22orf28 SRPK1 C9orf167 PSTK CHD3 PNCK RAD54L2 DDR2 DRG2 TUBB2B DDX52 PFKFB2 PDE6G PKN2 NMNAT3 NSF GUK1 KIF22 MAP3K8 RAB20 WEE1 SEPT8 ACTR3 RAC1 STARD9 STK10 MAP4K5 GNA15 KIF3A ENTPD8 TAP1 DDX1 HSPA1A MST4 DDX3Y MAP2K1 FIGNL2 EEF2K KATNA1 ABCC9 SGK196 ALDH18A1 NME1 CAD CSNK1E PDE4D CDK11B PDE1A RHEB RBKS RYK PDE11A ATP6V1B2 PKMYT1 CAMK1D NLRP11 HSPE1 PNPLA8 RHOJ HK3 ITPK1 HSP90AA2 OAS3 DMC1 ATP9A GEM RAB9A HSP90AB1 GUF1 ABCB8 MAP3K14 EFTUD2 MCM6 PASK ERBB4 C9orf96 PMS2 ATP2C2 KALRN NEK8 CHEK1 TOR3A GCLC ABCF1 CHEK2 MAP2K2 VPS4B RAB1A MYO1C ABCD3 ATP11B BMX ABCD4 DDX39A RERGL G3BP1 FUK TTL RAB8A CHD6 MCM2 ABCB11 TRIO MYO1A WEE2 ACTR8 SMARCA5 IPPK ARL1 MAP3K11 RAN ABCD1 TLK1 OBSCN FIGN IRAK2 EPHB1 P2RY4 IGHMBP2 PIP5K1A TCP1 ACSM2A ATP13A5 NLRP10 UBE2O MYO9B STK32B MYLK2 MAP3K4 TESK2 TRAP1 LANCL2 SEPT12 TXK RABL3 ABCC1 AAK1 KIAA0564 ABCA11P ATP8A2 RAD51C CDK9 GUCY2D RAB4B DNAJA2 IGF1R GAL3ST3 RAPGEF3 CCT4 GNAZ SLFN5 TK2 RHOG RAB6A CIITA SLFNL1 CAMK2A PI4KB ACVRL1 NUBP1 UCKL1 BMPR1B GRK5 HARS ATP1A3 ABCA7 LIMK1 RASL12 TUBE1 CAMKV EPHA7 PI4K2B MAP3K15 RENBP NRAS FRK IFI44L RAB14 AFG3L2 TWF1 SYN2 RPS6KB2 ATP13A2 MMAB FAM20C GTPBP5 RPS6KB1 MAPK1 DHX34 ATP13A1 C9orf103 EEFSEC MYLK NAIP IKBKB EPHA2 AKT1 NRBP1 AURKB ITK DMPK GNA12 DDX56 SEPT2 MTHFS MYH14 ATP8A1 UBE2Q2 IKBKE ACVR2A AURKC RPS6KC1 ARF3 GPHN RAB12 KIF20B EIF2S3 HIPK1 CHD9 CHD5 NPR1 CSNK1A1 UBE2Q1 PRKAG1 NKIRAS1 BLK ARL14 ACSL3 DGKQ KIF19 MYO6 SUPV3L1 MYO1D RUNX1 ATP7B ABCF3 MKNK1 GNAO1 MYO1B ENPP1 GARS TARS2 AKD1 MYH8 RRAGB ACSS3 EGFR TUBD1 DAPK2 GTPBP2 VRK1 SARS2 TWF2 SMARCA2 DQX1 MB21D1 SPAG1 TTLL7 DNM1P34 RAB18 IARS2 CARS HSP90AB2P PAK3 SLC27A5 OPA1 SMARCAL1 TUBG1 RFK MAP2K6 LOC100130097 TAP2 CAMKK1 KARS SKIV2L2 NLRP13 ALPK2 PFKM RAD54L GLYCTK PFKFB1 CDK2 PRKCQ HSP90B2P MAGI1 PRKD1 PRKAG3 GALK1 MYO5C AK4 NRBP2 BTK RPS6KA3 UBE2E2 ILK DHODH ERCC3 RTCD1 TUBA1C CDC34 MYO18B AATK ACSM5 NLRP1 RAB13 ACSS1 TUBB3 MYO15A NDUFA13 CTPS2 GIMAP7 ABCC3 CSK HSP90AB3P FICD TTLL9 TRNT1 RFC4 KIF18B SRXN1 PDGFRB FARSA GUCY1B3 DDX6 MAP2K3 RIPK1 TARSL2 PLK3 RPS6KA2 BTAF1 DNAH12 DNM1 RHOU IARS PFAS NME6 ATP8B1 ABCB1 UBE2D3 PRKCE DGKG MASTL DDX42 KTI12 TTLL13 PPP5C STK38L ATR PLK4 CHKB MDN1 KIFC2 P2RY1 CDKL2 MYH1 PIK3C2A ORC5 ARL13A GSPT2 MUSK PANK1 P2RX2 STRADA GNAI3 DIRAS3 BRIP1 KIF25 KRAS PRKDC DIRAS2 RRAGA HK2 NAT10 PIKFYVE HELB SMC1B ARL4D SHPRH ROR1 STK17B FGR LIG4 PKN1 UBE2H SLFN14 INSRR EEF1A1 TUBB6 SEPT10 KIF21B MX2 TTK TESK1 MAPK4 UGP2 CDK16 TRPV4 PRKAR1B NPR2 LRGUK NEK10 TAOK2 CLPX TUBB4A MX1 DCLK3 GNAI2 DYRK1B MAP3K5 NEK2 DARS2 LRRK2 PRKCD MAT1A PALM3 SGK2 RANBP17 EIF5 LARS RSG1 MYH15 NLK C10orf129 CDK5 KIAA0232 LOC255308 ERCC6 ABCA3 SEPT3 HS3ST5 NOLC1 MAPK6 RAB43 TRPM4 DNAH3 SNRK DDX10 DHX38 HLTF EPHB6 ATL3 ACTR1A SGK1 DNAH10 UBE2E1 NTRK3 CCT7 EIF2B2 ORC1 UBE2W CLK4 GSPT1 SMCR7L PI4KA LSG1 GVINP1 NEK5 TYRO3 OAS1 ATRX ITPKC RAB5B MAP3K2 EHD1 DDX28 CLP1 GRK6 PDGFRA PNPO KIF14 RND1 SBK2 GLUL PIM1 KIF7 ATAD1 MYO7A ADRBK2 DDX17 RAB4A KATNAL2 UBE2D2 SLK RIT1 FANCM CKM PPIP5K2 NLRC3 GBP4 MAP4K3 EHD2 SAR1A MYLK3 KCNJ10 PGK2 XYLB DYNC1H1 ADCK2 AK8 GNA11 NEK1 ABCE1 TUBB ENTPD3 PIK3C2G UBE2S MATK UHMK1 HSPA14 KIF13B RHOC RASL11A HSPA4L CKB H1FNT MOK TSSK4 RFC1 RABL2B NLRC5 HSP90AB4P DGKA GNAI1 HSP90AA4P ACVR1B PTK2 GK2 PIP5KL1 RTEL1 DHX30 DDX31 CDK14 RHOF KCNJ8 IQCA1 NME2 DDX19B KIF23 UBE2L3 RECQL5 RECQL ROCK2 WARS2 CDK10 ATP7A ABCB6 CDC42BPG SEPT6 TTLL6 DALRD3 GLUD1 MSH4 LMTK2 CYB5R3 MYO5A DNM3 HBS1L RIPK4 ARL11 NIN ALK MARK4 MCM9 KSR2 PRKACG PPIP5K1 NAV2 RRAS TAOK3 TTLL1 RAC3 PCK2 RAB6B UBA6 PRKAA1 GLUD2 NMUR2 SNRNP200 ATP2A1 ATP2A3 RHOT1 PRKY ATP6V1A MAP4K2 STK33 UBE2M FER ULK2 SEPHS2 TUBA1A GFM2 KCNJ11 ATAD2 SRPK2 ACSBG2 UBE2Z DDX21 UCK2 PRKAR2A P2RX5 PHKG1 LYN MYO1H AARS MYO3A MCCC2 CHD2 ATP11C TSSK3 BUB1B CLCN3 PET112 AK2 ABCG2 RAP1A MYH4 ARF5 RAPGEF2 LONP1 ASS1 NLRP9 TSR1 NLRX1 HSPA13 MAST2 ABCA5 NOD2 CDC42 PDE6H RAB19 MST1R PAK7 DYNC2H1 TP53RK PRKACB AACS CARS2 PSMC5 MAPK8 HIPK2 KIT RHOQ HELLS MTHFD1L MYH7B DDX43 NUBPL PIF1 PRPS2 TRPM6 NARS2 ATAD3A DNAJA4 CIT CDKL5 DGUOK RHOBTB1 NLRC4 RAB40AL REM1 GTPBP8 CDC7 SHPK SIK2 VPS4A SLC22A5 ADCY7 ERBB3 PRKCI MYO16 RAB21 GNA13 CNGA2 TMEM173 ADCY10 PFKL EPHA4 CLK1 MAP3K1 FBXO18 FGFR2 GIMAP8 CNGB1 CCT2 RAB24 HARS2 TDRD12 RARS CHUK CDK11A NTRK2 ITM2B DNAH7 RAB11B DNAJC27 ABCA8 DDX60L EPHB2 SRC GBP1 SRPK3 NMNAT2 AMHR2 RAB32 TIE1 ANXA6 TSSK2 RPS6KA4 GRK1 ADCY5 RRAS2 ACVR2B JAK3 MKNK2 DICER1 POTEKP CDK15 BVES NADSYN1 HSP90AA1

GO_SECONDARY_ACTIVE_TRANSMEMBRANE_TRANSPORTER_ACTIVITY Catalysis of the transfer of a solute from one side of a membrane to the other, up its concentration gradient. The transporter binds the solute and undergoes a series of conformational changes. Transport works equally well in either direction and is driven by a chemiosmotic source of energy, not direct ATP coupling. Chemiosmotic sources of energy include uniport, symport or antiport. SLC7A1 KIAA1919 SLC4A7 SLC35A3 SLC9A11 SLC12A5 SLC26A4 SLCO1A2 SLC26A1 SLC5A7 SLC9A7 SLC47A2 CLCN6 SLC6A5 SLC8A3 SLC17A2 SLC9A6 SLC4A2 SLC22A12 SLC10A2 SLC1A3 SLC5A6 SLC6A15 SLC16A5 SLCO1C1 SLC2A8 SLC1A1 SLC34A3 SLC6A7 CLCN5 SLC36A1 SLCO6A1 SLC5A10 SLC23A2 SLC10A7 SLC26A3 SLC37A3 SLC6A9 CCDC109B SLC17A8 SLC22A8 SLC5A2 SLC36A2 SLC16A6 SLC4A5 SLC38A1 SLC9B1 SLC16A14 SLC25A6 SLC6A6 MFSD3 SLC10A4 SLC6A4 SLC22A25 SLCO1B1 SLC20A1 SLC5A1 SLC37A4 SLC45A4 SLC16A13 SLC17A1 SLC26A2 SLC5A8 SLC45A2 SLC7A5 SLC22A20 SLC23A1 SLC6A11 SLC9B1P1 SLC6A17 SLC5A4 SLC16A12 SLC38A2 SLC28A3 SLC16A4 SLC8A2 SLC17A4 SLC24A3 SLC16A11 SLC4A3 SLC1A7 SLC26A7 SLC17A7 SLC7A10 SLC7A7 SLC34A2 SLC13A2 SLC47A1 SLC9A4 SLC36A4 SLC10A5 SLC26A11 SLC26A9 SLC12A4 SLC2A13 ANKH SLC13A1 SLC9A9 SLC6A1 SLC7A14 SLC10A1 SLC17A5 SLC16A10 SLC5A12 SLC24A4 SLC26A5 SLC26A8 SLC45A1 SLC28A2 SLC15A5 SLCO2B1 SLC11A2 SLC7A2 SLC6A20 SLC6A8 SLC6A14 SLC5A9 SLCO5A1 SLC15A4 SLC16A1 SLC7A6 SLC6A2 SLC7A4 SLC1A4 SLC16A7 SLCO1B3 SLC2A10 SLC2A12 SLC37A1 SLCO4C1 SLC9A3 SLC38A3 SLC22A10 SLC22A11 SLC7A8 SLCO2A1 SLC12A9 SLC5A3 SLCO3A1 SLC22A7 SLC15A3 SLC22A5 SLC3A2 SLC8A1 SLC25A3 MCU SLC25A18 SLC22A1 SLC24A6 SLC24A2 SLC10A3 SLC16A2 SLC15A2 SLC1A5 SLC22A6 SLC37A2 SLC11A1 SLC13A4 SLC7A11 SLC10A6 SLC7A3 SLC24A1 SLC35A2 SLC9B2 SLC35A5 SLC12A6 SLC19A1 SLC1A6 SLC46A2 SLC9A1 SLC26A6 SLC16A8 SLC4A11 SLC12A8 SLC17A6 SLC22A4 SLC16A9 SLC25A16 SLC36A3 SLC9A2 SLC20A2 SLC25A11 TMCO3 SLC2A9 SLC6A16 SLC22A18 SLC6A12 SLC22A9 SLC32A1 SLC9A10 SLC9A5 SLC12A1 SLC5A5 SLC17A3 SLC6A13 SLC24A5 SLC7A9 SLC15A1 SLC34A1 SLC22A24 SLC13A3 SLC33A1 SLC4A10 SLC41A1 SLC12A7 SLC45A3 SLC5A11 SLC7A13 SLC38A4 SLC6A19 SLC16A3 MFSD2A CLCN4 SLC9A8 SLC4A1 SLC28A1 SLC12A2 SLC35A1 SLC4A8 SLCO4A1 SLC4A9 SLC25A22 SLC13A5 SLC35A4 CLCN7 SLC1A2 SLC4A4 SLC26A10 CDH17 CLCN3 SLC12A3 SLC6A3 SLC6A18 SLC2A6

GO_POLY_A_SPECIFIC_RIBONUCLEASE_ACTIVITY Catalysis of the exonucleolytic cleavage of poly(A) to 5'-AMP. PDE12 PAN2 CNOT6 PAN3 TOE1 CNOT1 CCRN4L CNOT7 PARN CNOT2 CNOT6L CNOT8

GO_LACTATE_TRANSMEMBRANE_TRANSPORTER_ACTIVITY Catalysis of the transfer of lactate from one side of the membrane to the other. Lactate is 2-hydroxypropanoate, CH3-CHOH-COOH; L(+)-lactate is formed by anaerobic glycolysis in animal tissues, and DL-lactate is found in sour milk, molasses and certain fruit juices. SLC16A7 SLC16A6 SLC16A4 SLC16A12 SLC16A8 SLC16A5 SLC16A11 SLC16A13 SLC16A3 SLC16A1 SLC5A12

GO_LYASE_ACTIVITY Catalysis of the cleavage of C-C, C-O, C-N and other bonds by other means than by hydrolysis or oxidation, or conversely adding a group to a double bond. They differ from other enzymes in that two substrates are involved in one reaction direction, but only one in the other direction. When acting on the single substrate, a molecule is eliminated and this generates either a new double bond or a new ring. GADL1 ACO1 PCBD1 PTGES2 CCBL1 GLO1 SGPL1 ADCY1 DDC CA14 ADSL THNSL2 NPR2 CA9 CTH CA5B ME3 GAD2 CA5A APEX1 GUCY2D ENO3 CSAD ALAD FASN ASL MLYCD CA8 NPL CRY1 SCLY FH TSEN34 HACL1 NTHL1 GAD1 PTPLAD2 CBS DDTL HAL NPR3 IRG1 POLB ALKBH1 MOCOS ECHDC2 ADCY7 C20orf3 PARK7 HMGCL THNSL1 AMD1 PUS1 PCK1 ENO1 NPR1 DERA FAHD1 CA3 BCKDHA NEIL1 FECH ODC1 SRR ENOSF1 ADCY10 HADHB MOCS1 DAK HSD17B4 UROC1 BCKDHB ALDOC SDS APLF CA12 LTC4S ECHS1 RPP14 BST1 XRCC6 GLDC APEX2 PAM ADCY5 HOGA1 ADC MGST2 ME2 GLUL CA4 PCBD2 TYW1 PPCDC APIP GUCY2C SDSL ADCY9 UBA5 ME1 POLQ GMDS GUCY2F CD38 PAICS PTPLAD1 SHMT1 HADHA ALOXE3 GUCY1A2 PRHOXNB PISD C14orf149 DDT ENO2 AGXT2L2 ACMSD CCBL2 ENO4 GGCX SMUG1 PTPLA TSEN2 ADCY3 CENPV XRCC5 CA1 PDDC1 ACO2 CA6 ADCY4 OGG1 SHMT2 PTS UROS MVD GUCY1B3 CA7 AUH ALDOB HMGA2 CA2 NEIL2 ADCY2 UXS1 CRY2 AGXT2L1 HCCS ALOX5AP HMGB1 ADCY8 GOT1 UMPS CA11 ADCY6 FTCD RPS3 ACCS CYP17A1 PDXDC2P CARKD PCK2 UROD HDC NEIL3 POLL ACAT1 CA13 TYW1B ALDOA HMGCLL1 EHHADH PTPLB PDXDC1 ECHDC1 GUCY1A3 TGDS

GO_PHOSPHOLIPID_BINDING Interacting selectively and non-covalently with phospholipids, a class of lipids containing phosphoric acid as a mono- or diester. SYT2 DENND1A C2CD4A CPNE1 TULP1 PIGK KCNJ2 SESTD1 PLCD1 C11orf83 NUP62 JPH2 ARHGAP9 PIK3C2B SNX14 MAPKAP1 SNX27 PREX1 NCF1 SYTL5 ZCCHC2 OSBPL1A HS1BP3 ING2 SNX10 OSBPL5 APOA1 PFN2 DOC2B OPHN1 STOML2 SYTL4 RPE65 BBS5 VAMP2 PCLO SYT14L PLEKHA8 ESYT2 NR5A1 FCHO2 ZFYVE26 RS1 OSBP SYT10 OTC PLEKHF1 PIRT ARL6 F3 MYOF MTM1 ANXA3 AXL PITPNA ABCA1 WDR45 SYT12 RPH3A SYT9 TICAM2 SYT4 ZFYVE9 IQGAP2 ARHGAP32 OGT SNX20 SNX4 SNX31 PSD4 GOLPH3L PEBP1 SNX30 COL4A3BP PTAFR WDFY3 NUP62CL ZFYVE28 SCARB1 KIAA0528 ANXA8L1 SH3PXD2A SNX13 ADAP2 PIGU FES ZFYVE16 GAS6 ANXA5 SYT3 SNX5 NBEAL2 WDR45L C6orf145 LDLRAP1 NSFL1C BTK KIF16B LPAR3 SCIN MPPE1 NSMAF PLA2G7 ARHGAP44 ITPR1 DAPP1 VPS36 PLD2 SH3YL1 EXOC1 C2CD4D KCNQ1 RLTPR SNX22 CCDC88A PXK PSD2 SNX24 PLA2G4B TIMD4 ANXA2 THY1 FABP1 APOA5 PIK3C2A SNX17 PLEKHA2 GOT2 LPAR1 SYT11 HMGB1 ATP8B1 BAIAP2L2 SPTBN1 ARAP2 C8orf44-SGK3 RASAL1 APOE STAP1 AGAP1 SYT7 MYO10 PEMT TECPR1 SNX1 NCF1C RPH3AL GOLPH3 PLEKHA3 ANXA13 KRIT1 ITPR3 PLA2G15 F10 NOXO1 CPNE3 OSBPL10 FAM21C TWF1 DAB2IP GAB2 PFN1 ANXA2P2 EEA1 CD300A AIDA GRB7 SPTBN2 SNX21 SYTL1 MAP1LC3A SNCA TULP2 PLD1 SNX29 PON1 GRK5 FGD2 APOA2 GBF1 PLCB1 FAM123A MITD1 SNX2 ITPR2 SNX25 TIRAP ANXA4 NCF4 SNX15 C2CD4C FRMPD2 WIPI2 SEPT12 LANCL2 APOH COMMD1 SNAP91 TEC RACGAP1 ARHGAP26 SNX9 ZFYVE19 SPTBN4 ANXA6 SYT14 FRMPD4 ABCG1 TRIM72 MYO1B SNX8 MFGE8 PARD3 TWF2 ASAP1 ARAP1 CEACAM5 GSDMD RNF34 NF1 CYTH3 OSBPL8 TULP4 MCF2L SMURF1 RPS6KC1 DYSF PSD ZCCHC14 SNX18 SLC9A1 GPAA1 BAD MARK1 MCTP2 PITPNM1 ANXA11 PHF12 PAFAH2 SDPR SYT5 WDR35 CPS1 DOC2A VIL1 PLEKHA5 PLA2G4A AKT1 STXBP6 TRPV1 SNX6 SYT15 SNX11 ADAP1 ANXA9 RASGRP1 PRKCI ALOX15 HIP1R DGKA THBS1 FAM123B ANXA8 ZFYVE1 WDFY1 ACTN2 ARHGAP35 PACSIN1 PHLDA3 KCNJ1 SH3PXD2B SYT8 NR5A2 SYTL2 RAB35 APOM NISCH ANKFY1 NCF1B MYO1G WIPI1 SNX7 TULP3 SYT17 SYT6 SGK3 GAP43 TUB PLA2G2A APOC3 PIK3C2G BPIFC EPB41 SYTL3 SNX12 ANXA10 NBEAL1 BIN2 SNX32 SYT13 SBF2 LPAR4 APOB TC2N RAG2 HIP1 ANXA7 SYT1 ANXA1 NRGN FERMT2 AMPH MTSS1L DPEP1 GLTPD1 ARAP3 FAM123C HSPA8 SNX16 ARFIP1 SNX33 PLA2G4C TTPA PITPNC1 SEC14L2 C2CD4B ARHGAP33 GLE1 WDFY4 SHC1 IQGAP1 PLEKHA4 CPNE6 SGIP1 SNX19 SYT16 MYO1E SNX3 MCTP1 ARFIP2 NPM1 TNFAIP8L3 PASK NUP35 PICALM

GO_RNA_POLYMERASE_II_REPRESSING_TRANSCRIPTION_FACTOR_BINDING Interacting selectively and non-covalently with an RNA polymerase II transcription repressing factor, a protein involved in negative regulation of transcription. RBPJ STAT3 PPARA TBP TCERG1 SP1 MKKS BBS7 GTF2A2 GATA6 DMAP1 BBS2 HDAC1 TTC8 BBS10 BBS1 HDAC2 GSC MIXL1 RBBP8 MTA2 CHD4 SIN3A MTA1 TCF7L2 BBS5 BBS4

GO_PHOSPHATIDYLINOSITOL_BINDING Interacting selectively and non-covalently with any inositol-containing glycerophospholipid, i.e. phosphatidylinositol (PtdIns) and its phosphorylated derivatives. SYT7 MYO10 NPM1 ARFIP2 C8orf44-SGK3 KRIT1 ITPR3 PASK PLEKHA3 PICALM NCF1C GOLPH3 TECPR1 SNX1 TNFAIP8L3 ARHGAP33 PLEKHA2 SNX17 SNX19 MYO1E SNX3 PLEKHA4 IQGAP1 ARAP2 ANXA2 FERMT2 HIP1 NRGN SYT1 PITPNC1 TTPA ARFIP1 SNX33 PIK3C2A FAM123C SNX16 MTSS1L THY1 ARAP3 DPEP1 SH3YL1 EXOC1 PLD2 DAPP1 SBF2 VPS36 SNX24 PXK SNX22 CCDC88A RAG2 KCNQ1 SNX12 MPPE1 SCIN EPB41 SNX32 ITPR1 SGK3 SNX5 SNX7 TULP3 KIF16B BTK PIK3C2G LDLRAP1 TUB WDR45L GAP43 C6orf145 FES PIGU ADAP2 RAB35 SYTL2 SNX13 SH3PXD2B SH3PXD2A WIPI1 MYO1G ZFYVE16 ANKFY1 NCF1B NISCH FAM123B WDFY3 GOLPH3L HIP1R SNX30 COL4A3BP KCNJ1 PHLDA3 ACTN2 SCARB1 ZFYVE28 ANXA8 ZFYVE1 WDFY1 TRPV1 IQGAP2 VIL1 PLEKHA5 AKT1 STXBP6 ZFYVE9 RPH3A SYT9 WDR35 SNX31 SNX20 SNX4 ALOX15 ADAP1 OGT SNX11 ARHGAP32 SNX6 ZCCHC14 GPAA1 SNX18 SLC9A1 RPS6KC1 MTM1 PHF12 SYT5 WDR45 PITPNM1 MARK1 PITPNA ASAP1 PIRT ARAP1 CEACAM5 PLEKHF1 MCF2L TULP4 OSBPL8 GSDMD RNF34 CYTH3 MYO1B FCHO2 FRMPD4 TWF2 RS1 OSBP SYT10 SNX8 ZFYVE26 PARD3 COMMD1 SNAP91 WIPI2 SEPT12 LANCL2 ESYT2 PLEKHA8 SNX9 ZFYVE19 RACGAP1 BBS5 HS1BP3 SNX2 ZCCHC2 MITD1 NCF1 FRMPD2 PFN2 TIRAP NCF4 SNX15 ITPR2 OSBPL5 SNX25 ING2 SNX10 TULP2 JPH2 SNX21 PLCD1 GRB7 SNX27 MAPKAP1 FGD2 GBF1 PLCB1 FAM123A SNX14 PIK3C2B PLD1 ARHGAP9 SNX29 TWF1 GAB2 DAB2IP FAM21C NOXO1 KCNJ2 PIGK SESTD1 AIDA TULP1 EEA1 PFN1 DENND1A

GO_GDP_BINDING Interacting selectively and non-covalently with GDP, guanosine 5'-diphosphate. GNAT1 RAB11B RAB10 RAB35 ARF1 RRAS RAB31 RAB8A ARL8B PRPS1 RAB2A RAP1B RAP2C RAB7A GNAI3 RAB5B RAB14 RAB17 RAB22A GNAI1 KRAS RAB27B RAN RAB9B RRAGC ARL3 RAB5C RHOB TRIM23 RAB40C RAB4A RAB27A RERG RAB18 RAB28 GEM DYNC1LI1 RAB8B SUCLG2 SEPT12 RALA RAB9A RAB7L1 RAB12 RALB RAB21 SRP54 RAP2B RAB5A SMCR7L PCK1 RAB3B

GO_MIRNA_BINDING Interacting selectively and non-covalently with a microRNA, a 21-23 nucleotide RNA that is processed from a stem-loop RNA precursor (pre-miRNA) that is encoded within plant and animal genomes. LIN28A TRIM71 SOX2 DND1 TARBP2 EIF2C2 HNRNPA2B1 POU5F1 MEF2C FMR1 EIF2C3 RBM4 EIF2C1 ZC3H12A PNPT1 EIF2C4

GO_SYNTAXIN_BINDING Interacting selectively and non-covalently with a syntaxin, a SNAP receptor involved in the docking of synaptic vesicles at the presynaptic zone of a synapse. VPS18 ABCA1 SNPH SYT5 TXLNB SYT16 SYT12 VAMP7 C2CD4B SYT11 SNAP25 RPH3AL SYT15 NAPB DAPK1 SNAP23 NAPG DOC2A SYT4 VPS11 SYT9 RPH3A SYT7 STX8 RAB4A NAPA C2CD4D ABL1 NSF STXBP4 SYT10 SYT14 TXLNA STXBP5L LLGL2 BET1 TC2N VPS52 CPLX2 RNF40 RAB11A SNAP29 SYBU CPLX3 PTPN2 STX10 SYT1 LLGL1 UNC13A DOC2B C2CD4C STXBP2 SYT17 TMED9 SYT6 TXLNG SYTL5 VAMP2 SYT13 SNAP47 SYT14L SCFD1 TXLNG2P SYTL4 SYTL3 CACNA1A STX6 CPLX1 C2CD4A CPLX4 ABCC8 SYT2 HECTD3 SEC22B TMED10 GOLGA2 PLDN VPS54 STX16 SYT3 SYTL2 STXBP1 SYT8 SYTL1 VAMP3 STX7 STXBP5 LRRK2 STXBP3 SLC6A4

GO_PHOSPHATE_ION_BINDING Interacting selectively and non-covalently with phosphate. ADSSL1 PNP SLC34A2 GNG12 CHST14 OTC RPH3A ADSS MTHFD2 G6PC RELA

GO_DNA_N_GLYCOSYLASE_ACTIVITY Catalysis of the removal of damaged bases by cleaving the N-C1' glycosidic bond between the target damaged DNA base and the deoxyribose sugar. The reaction releases a free base and leaves an apurinic/apyrimidinic (AP) site. NEIL2 NTHL1 SMUG1 MUTYH MPG TDG RPS3 NEIL1 CCNO UNG MBD4 APEX1 OGG1 PCNA NEIL3

GO_CYTOSKELETAL_ADAPTOR_ACTIVITY The binding activity of a molecule that brings together a cytoskeletal protein and one or more other molecules, permitting them to function in a coordinated way. NCK1 ANK3 SORBS2 OBSL1 BAIAP2L1 SDCBP BICD1 GAS2L1 BAIAP2L2 BAIAP2 ABI2 ANK2 BIN3 NCK2 ANK1

GO_G_PROTEIN_COUPLED_RECEPTOR_BINDING Interacting selectively and non-covalently with a G-protein coupled receptor. GNA14 USP20 MYOC WNT16 YARS HSPA1A CCL18 KISS1 CCL3L3 PDE4D NDP GRIK3 EDNRB SFTPB AGTR1 UCN RYK RTP4 GNAI1 WNT2 ARRDC3 PTPN11 CALM3 PHB CCL24 GPRC5B CCL27 RLN3 CCKBR CXCL5 IL8 DNM2 TAC1 EDN1 ADM WLS USP4 GNA15 WNT10A GNA11 ARRB2 CCL2 PENK HCRT DNAJA1 S1PR1 PF4V1 GOPC PSAPL1 STUB1 HOMER3 GHRH HSPA8 IL2 CCL4 FYN USP33 SFRP1 CCL8 NPPA WNT9B CCL22 ARRB1 CXCL6 CCRL2 HOMER1 BDKRB2 TFF2 ADCYAP1 CXCL11 PARK2 NPB GNG3 CCL23 SAA1 ADRBK1 DNM3 NPW NMU GNAT2 GNAQ NEDD4 MAGI2 C5 DEFB4A QRFP GHRL CCL1 PNOC ADORA1 RTP3 PYY2 RTP2 ITGB4 RTP1 CECR1 CXCL3 ATP1A3 PPBP ADRA2C WNT5B STAT3 SLC9A3R1 PTH PSMC5 CCL4L2 CCL21 DVL1 GNB1 BAMBI GAL RSPO1 WNT11 PDCD6IP CCL3L1 UCHL1 POMC CKLF VPS35 GNAZ PACRG WNT1 RAPGEF2 PF4 EDN2 ARAP1 ZNRF3 INSL5 PICK1 CALM1 LRP6 CCL26 GNAO1 AVPR1A RPGRIP1L RSPO3 FZD7 CCL11 CCL28 STAT1 WNT2B CCL13 CXCL9 GNA12 RALA WNT3A GNA13 DEFB1 PYY3 WNT3 TYK2 WNT4 NMS BICD1 GNAT3 ADRB3 FLNA GCG NPY REEP1 CCL3 S100A14 CXCL14 PPY ROR2 DVL2 FZD1 RAMP1 WNT9A WNT10B PYY FCN1 ADRB1 NPFF CCL14 CCL19 CCL15 HSPA1B DLG4 CX3CL1 CALCA CXCL10 PPP1R1B NPFFR2 SHANK1 P2RY1 AVP C1QBP GNAI3 CCR2 AGT ARHGEF11 PRLH UTS2D CXCL16 PALM S1PR2 SDCBP CCL25 JAK2 UCN3 CLIC6 DNM1 MRAP LOC100506013 WNT5A REEP2 GNAI2 HOMER2 CCL16 GNAT1 CCL7 WNT8A XCL1 JAK1 MRAP2 XCL2 PDYN CCL17 CNIH4 WNT7B RNF43 CXCL1 CXCL2 PROK2 DVL3 PSAP CCL20 ADRA2A ATP2A2 C2orf85 OXT WNT6 WNT8B CCL5 ITCH UCN2 DRD3 NMB CXCL13 FEM1A CRH CXCL12 ARHGEF12 CALM2 APLN APLP1 WNT7A GNAS ASIP PROK1 EDN3 ACE C3 CORT CTHRC1

GO_CHEMOKINE_BINDING Interacting selectively and non-covalently with a chemokine. Chemokines are a family of small chemotactic cytokines; their name is derived from their ability to induce directed chemotaxis in nearby responsive cells. All chemokines possess a number of conserved cysteine residues involved in intramolecular disulfide bond formation. Some chemokines are considered pro-inflammatory and can be induced during an immune response to recruit cells of the immune system to a site of infection, while others are considered homeostatic and are involved in controlling the migration of cells during normal processes of tissue maintenance or development. Chemokines are found in all vertebrates, some viruses and some bacteria. HMGB1 CXCR2 A2M CX3CR1 CXCR6 ZFP36 ITGAV CCR7 CCRL1 CCR5 CXCR1 CCR1 PLP2 CXCR7 CCBP2 ITGB3 CXCR3 ITGA4 DARC ITGB1 CCR6

GO_ENZYME_REGULATOR_ACTIVITY Binds to and modulates the activity of an enzyme. AVP ECT2 CAMK2N2 TBC1D22B PPP1R3C CXCL10 AHSA1 GPSM3 APC ADPRHL1 ARHGAP15 ERRFI1 DNAJC7 APAF1 TBC1D3F PINX1 CSN2 STRADA DLC1 NOXA1 PSME1 FAM150B THY1 CTSA GPC3 AGT PIK3R3 ITIH6 DBF4B STXBP5L HYAL2 CDKN2A BIRC3 ARHGEF11 SOCS7 CDK5R1 TOR1AIP1 LRRC15 ARHGEF15 GDI1 RALGAPB RASAL1 TANK DUSP19 BIRC7 NCF1C SERPINB4 UCHL5 LRRC4C PHACTR4 PPP1R36 PPP1R15A DNAJC24 ITIH5 RPTOR PTN CAB39 CDC20B RASAL2 PRKCE ARAP2 ATP2B4 ABR FZR1 MAT2B ITIH1 ARHGAP19 PLEKHG6 CHP NRG3 TAB1 TBC1D8 RPS6KA3 SH3PXD2A SPINT4 STXBP5 GCN1L1 NLRP1 GAS6 SERPINB5 SKI PPP2R2C KAL1 PITRM1 RGS22 SIPA1L3 TBC1D16 OAZ3 PPP1R2 GUCA2B ELP3 HSPB1 AGAP6 TBC1D9B ARHGAP44 PPP1R26 PPP1R7 GRTP1 MMP14 TSC1 DNAJA3 DAOA ACAP1 ARHGAP31 GRPEL2 TBC1D2 SPINK2 GUCA1C PRKRA TIMP2 RUNDC3A A2M DNAJC2 CST9LP1 MAL FOXL2 IGF2 TBC1D9 TBC1D30 PPP2R5E PRKAR1A ARHGEF1 GRM7 RASGRP3 SOS1 OCRL CHM ARFGAP1 RALGDS SPINK9 RGSL1 CCS AUP1 RGS16 RASA2 PTTG2 ARHGAP42 TRIB1 PPP2R5B APOC2 BMP2K CDC42SE1 TRIP10 RAB4A PCP2 ARFGAP2 PSMD2 CALM2 ARHGAP6 BRSK2 PYGO2 TBC1D5 SPINK5 ACAP3 OGT GLMN ARHGAP12 RIN2 ARHGAP18 ARHGDIB DCP1B ANXA3 RGS17 PCNA FAM20A AGFG1 DNAJB6 IGFBP3 PPP2R5A GPS2 KIAA1967 C15orf62 SERPINA3 PSMD1 RTN4RL2 SERPINB13 LRRK2 EFNA5 PIK3CA SERPINA1 TIMP3 ARHGAP5 SERPINB7 NGF RCAN3 CCNC AGAP7 PRKCD ARHGAP9 RAP1GAP UGT1A1 PPP2R1B CHN2 SCG5 STX4 PPP2R1A PIK3R5 GMFG GSTP1 ERCC6 CHAD CISH TRIM23 GCLM TNFSF14 SPRED2 TFAP2B SERPINA2 PSMD3 ESR1 CXCL1 CKS2 SHOC2 BRCC3 APBA3 JUN H2AFY CAMK2N1 CCNE1 AHSG CCNK WFDC5 PSAP GPS1 PRKAR1B SERPINA10 BAG4 NCF1 ARHGAP1 ASAP2 SERPINB6 RCVRN CLPX APOA1 ENSA SERPINB11 A2ML1 PDCD5 DBF4 CDKN1B PRPSAP1 CD24 CASP1 CCNL1 GOPC TAOK3 C6orf126 ALS2CL SYDE2 SERPING1 LEF1 RGS12 WDTC1 SYNGAP1 WFDC12 CCL8 TBC1D10A PPP2R3A PI16 RGS1 RINL PSMD14 WFDC10A TBC1D12 HEXIM2 RGS19 ARHGAP22 NPM1 EVI5L TINF2 CST8 CTAGE5 ANP32E ATP7A PODN DGKI SPRY2 GDI2 SCGB1A1 PPP4R4 LMTK2 CARD16 ALOX5AP CTGLF11P CARD17 PPP1R35 ARHGAP33 SLIT2 SIPA1L1 GMFB TIMP4 AGAP2 PSME3 LRTM1 NYX GNAQ RTN4RL1 IQGAP1 PPP2R2A CD109 PLXNB1 CPAMD8 PRPSAP2 DOCK1 RIN3 PDPK1 PRDX3 RING1 SERPINE1 FBLN1 ANGPT4 SFTPB GUCA1B PDC RAP1GAP2 ARHGDIG STARD13 RFC1 DUS2L ZEB2 CST6 SERPINB9 DCP1A TBC1D14 SPOCK3 TBC1D2B UCN UBE2L3 RIMS1 LRRTM1 CDK5RAP1 AGAP5 RPS27L EGF SH3BP4 PKIG CPN2 STARD8 RABEP2 CCKBR TBC1D20 FGF13 SERPINB8 SMAP2 PCSK1N MMP24 TRIB3 SERPINB12 GTF3C4 SERPINB1 TMBIM6 TBC1D8B CABP1 SERPINI2 GTF2F1 APLP2 PPP1R39 APOC3 HEXIM1 ABCE1 DBNL BMP2 OAZ2 RNH1 LTC4S ASAP1 CRB2 BCL10 AFAP1L2 ITIH2 SET C6orf127 CALM1 SERPINA4 SERPIND1 RPLP1 ADRM1 NOL3 IBTK AGAP9 FAF1 PI3 TEN1 ADC SRGAP2P1 RECK FURIN CDC42EP4 PPP1R16B HSP90AA1 GNB5 DNAJC1 SERPINA9 WFDC3 OVOS2 CCND1 VIL1 NLRC4 PPP1R2P3 SIPA1L2 ERBB3 ADAP1 SMAP1 RTN4R BCL2L13 AGAP4 NOD1 PSME4 SH3BGRL3 TBC1D22A TBC1D10C ARHGAP17 TFPI2 SAG ARHGAP36 ALS2 CST3 WFIKKN1 MBIP RGS2 DOCK2 MYO9A LOC100507055 WBP11 PHACTR2 DAXX PLCB1 LRRTM4 APOA2 ABI1 GIT1 B3GAT3 RUNDC1 DPM2 WFIKKN2 SOCS2 CDC37 ARFGEF1 SFN ITIH4 ELMOD1 AHSA2 PI15 WFDC1 PDE6H PYCARD AXIN2 CASP8AP2 CDK4 CASP3 SERPINB2 GAPVD1 PROS1 GPSM1 PIF1 CAV1 MAPK8 PFN1 RCAN1 FLRT3 C3P1 PRKAR2A RABGAP1L APOH CST9L ITGB1BP1 ARHGAP26 SBF1 PZP HMSD DNMT3L RICTOR RASA1 PPP2R2D CFLAR RALGAPA2 COL4A3 SOCS3 GUCA1A CHN1 TIMP1 RGS13 CDC20 ANGPTL3 SPINLW1 RAPGEF2 ANXA4 GP1BA FAF2 PPP1R1B ANXA2 PDGFB PPP2R3B TBC1D3B GRPEL1 CSTB AGAP11 HRG PTTG3P APOA5 RAC2 LRRC4B SFRP2 PIK3R6 BAG5 TBC1D13 FRS2 LRRC19 WNK1 PCOLCE PRKAR2B SPINK8 PPP1R3B AMBP PPP1R8 BIRC2 DNM1L SERPINE2 ANKLE2 TAGAP CCNH AGAP1 PODNL1 PSME2 SERPINA6 APOE SOCS6 ARL2 KRIT1 YWHAG PPP2R5C POT1 SSPO SERPINA11 NCKAP1L ATP6V1H CCNY SESN2 RANBP1 UGT1A7 STK3 M1 CST2 SPINK1 ADAP2 CCNE2 APP ARHGAP20 UBA2 BIRC8 ANXA5 EPO ARFGAP3 BCAS3 OAZ1 DEPDC5 SH3BP5 CCL3 TBC1D17 CPEB2 VAV3 SPINT3 PPP1R14B PEBP1 RAP1GDS1 OBFC1 WFDC13 SPRED1 BAG1 NGFRAP1 RGS6 GREM1 HSPB2 C17orf42 ARHGAP10 NSMAF TBC1D10B CDKN2B SPINK7 FLRT1 MT3 PKIB ELMOD3 PSMF1 ARHGAP39 PPP4R1 TESC ARHGAP27 PPP1R14D FN1 CDC42EP1 DNAJC3 BGN SERPINA5 FAM58A ARHGAP11A C13orf15 CAST HSPBP1 NPRL3 SIPA1 TIAM2 ARHGAP40 RGS5 RGS20 BIRC5 ADPRH CST11 PPME1 C4B TOM1L1 RASA4 FLRT2 PAK2 LRRTM3 LLGL1 RGN SERPINA12 RGS11 PPP4R2 RGS14 RABEP1 SYDE1 IKBKAP PPP1R12A SGSM3 KAT2B ANKRD54 TSC2 CCL5 INCA1 TBC1D4 PLN PREB PPP1R2P1 PDE6G CTC1 FAM150A CSNK2B IQGAP2 USP6NL FAM13A ARL2BP ARHGEF12 SH3BP1 DOCK4 FAM58BP CASP9 WAS NLRP12 GPSM2 PARK7 RGS8 PHACTR3 PARP16 CABIN1 ARHGEF19 BNIP2 PDE6D SERPINH1 COL7A1 ARHGAP32 KLF4 CARD8 MTMR9 GARNL3 BCCIP ARHGAP30 RHOH STK11 MARK2 DNAJB1 TOR1AIP2 BAG3 BIRC6 C3 DCN AXIN1 APOC1 RABGAP1 GIT2 RTKN PPP1R27 SERPINF2 PPP1R37 ANKRD27 PINK1 SGSM1 TBC1D3H ARHGAP11B MMP17 ARHGAP4 PPP2R4 EBAG9 PROL1 ARHGAP29 WFDC6 IPO7 HMHA1 OPHN1 UGT1A8 CDC42EP2 ARHGAP24 GCHFR NKX3-1 BAI3 RGS21 TNK2 NCK1 SPP2 WFDC10B PREX1 LRRC4 TFPI EID1 CDK5R2 PFN2 ANGPTL4 ARHGEF6 TBCK PSAPL1 RAB3A FBXW7 DEPDC1B NOTCH1 ANXA1 RGS3 IL2 ARL1 FRY SERPINA7 RGS10 ARAP3 DPEP1 RIN1 RCAN2 BCR RGS18 SOCS5 ASAP3 SBF2 CST9 CTSC ALDH1A1 DEPDC1 C4A TBC1D7 LRRC66 MYO9B ATP1B3 ARRB1 R3HDML C19orf2 NPRL2 SPINK6 BAG2 TBC1D26 GUCA2A GMIP TXNIP RP2 HSP90AB1 LTF SERPINE3 RASAL3 GNB2L1 MYBPC3 TRIB2 KALRN TBC1D3 PIK3R1 APOA4 COL6A3 CHML MALT1 MMP16 TBC1D21 C5 MAP2K2 MOB1B SPINT1 ARHGAP25 CDKN1C UMODL1 PDZD3 PHACTR1 RAB3GAP2 MAP2K1 CBX8 TBC1D19 SH3PXD2B RALBP1 PIK3R2 NCF1B NRG1 FGFR1OP LOC390940 SRGAP3 CD27 TBC1D25 AGAP3 RASA4B HSPH1 TBC1D3C CST4 MAPK8IP1 ARHGAP35 CDC42EP5 HTR2B GPRC5B AGFG2 CALM3 KNG1 ARHGAP21 EIF2AK2 ITIH3 SPINK4 ACAP2 PPP1R1C CDC42EP3 CDKN2C CDKN1A RPS6KA1 SRGAP2 AZIN1 LXN SERPINF1 CST1 RASA3 CARD18 SOCS1 INPP5B PPP2R5D CDKN2D RANGAP1 PTPLAD1 PPP1R14A WFDC2 SERPINI1 PRKRIP1 TBC1D15 MTMR12 ATPIF1 ARAP1 ATP1B2 PPP1R9B PRKAG1 ARF1 PPP1R12B RIC8B DNAJC10 LRP6 ARHGDIA C16orf7 MMP15 CLPS CRIM1 NF1 ELP4 PAPLN LLGL2 FETUB TBCD SPINK14 VCP EVI5 PRLR ATP1B1 MGST2 ELFN2 SPINT2 LAMTOR3 RGS4 EGFR SAE1 OVOS PRKAG2 AKT1 CSTL1 CCNT1 SEPT2 TNFAIP8 COL28A1 DMPK DEPDC7 GM2A BAD SERPINB3 SNCB SLN PPP1R16A RALGAPA1 SRGAP1 PGAM5 CST7 MADD PPP1R14C CCNL2 ARHGAP23 XIAP SOCS4 PPP1R17 CIB1 RGS7 PPP1R2P9 SNCA GHRL TBC1D1 PPP1R10 SLX4 KIDINS220 PCOLCE2 PPP1R11 SGSM2 SPOCK2 CST5 DAB2IP SPOCK1 ITSN1 ASPN NOXO1 SPINK13 RENBP TBC1D24 SERPINB10 PTTG1 CCNT2 NAIP CAPN3 IPO5 ANXA2P2 WASL ARPP19 ARHGAP8 WNT11 FAM13B CSTA CTSH TGFB1 APOBEC1 ELMOD2 PRDX5 PPP1R1A BST2 PREX2 CKS1B RACGAP1 PLAA RAB3GAP1 STK4 SH3BP5L SERPINC1 ARHGAP28 RIC8A WFDC8 LPA PKIA RGS9 IGBP1 USP14 NCF4 PPP2R2B SLPI LCN1 ELFN1

GO_ION_CHANNEL_BINDING Interacting selectively and non-covalently with one or more specific sites on an ion channel, a protein complex that spans a membrane and forms a water-filled channel across the phospholipid bilayer allowing selective ion transport down its electrochemical gradient. KCNAB1 TRAPPC2 NOS1 KCNG4 HERPUD1 GRINA HRC CAV3 NEDD4L PKD1 PHPT1 PDE4B PRNP AP2M1 CALM2 KCNIP2 HOMER1 YES1 FKBP1B HSP90AB1 FKBP1A SCN5A SLC8A1 GPD1L PACS1 KCNH1 RIMS2 CAMK2D KCNQ1 YWHAE RNF207 ARRB1 KCNJ11 NANOGNB NPTN HSP90AA1 VDAC1 TSPO RANGRF CHERP DIAPH1 ACTN4 RIMS4 PPP1R9A GOPC PIRT FXYD1 KCNC1 YWHAH USP10 ACTN1 FGF12 SCN4B KCNC2 KCNE4 PKD2 CALM1 SRC FYN CDH5 KCNE1L SUMO1 FAM115A SRI FHL1 RIMS3 HTT DLG1 PKP2 S100A10 TRDN FGF13 SCN3B PANX1 KCNE2 BAK1 KCND3 LYN YWHAQ FAM115C TCAP STX1A RYR2 C19orf26 PYCARD PACS2 CTNNB1 SNTA1 CALM3 ACTN2 CAV1 KCNE3 ABCC8 ANK2 ACTN3 KCNB1 RIMS1 HAP1 ANK3 AKAP9 KCNIP3 AKAP6 CIB1 LRRK2 ABCC9 PDE4D PRKCSH KCNH5 FMR1 SCN10A ID2

GO_MICROTUBULE_MOTOR_ACTIVITY Catalysis of movement along a microtubule, coupled to the hydrolysis of a nucleoside triphosphate (usually ATP). KLC2 KIF5A DYNLRB2 DNAH1 KIF27 KIF20B DNHD1 KIFC1 KIF13A KLC1 DNAH10 DNAH12 BBS4 DNAH3 KIF12 DNAH5 KIF11 DNALI1 KIFC3 KIF18B KIF1B DNAH9 DYNC1LI2 KIF7 DNAL4 KIF14 KIF21A KIF4B KIF5C KIF25 DYNLRB1 KIF18A KIF9 KIFC2 KIF26B KIF19 DNAH7 DNAH6 KIF15 KIF3A KIF16B KIF2A DNAH17 KIF4A KIF2B STARD9 DYNC1H1 KIF5B KIF20A KIF24 CENPE LOC100130097 DYNC1I1 KIF21B KIF2C DNAH14 DYNC1LI1 APPBP2 KIF22 KIF26A DNAI2 DNAH8 KIF23 DYNC1I2 KIF3C KIF3B DNAH11 KIF1A SMC3 DYNC2H1 KLC4 KIF6 KIF17 KLC3 DNAH2 KIF1C KIF13B

GO_O_METHYLTRANSFERASE_ACTIVITY Catalysis of the transfer of a methyl group to the oxygen atom of an acceptor molecule. COQ3 BCDIN3D COMT PCMTD1 FTSJD2 LCMT1 FTSJ2 ASMTL ASMT COMTD1 FTSJD1 LRTOMT C6orf211 HENMT1 PCMTD2 PCMT1 MRM1 ICMT FTSJ3

GO_RNA_POLYMERASE_BINDING Interacting selectively and non-covalently with an RNA polymerase molecule or complex. ELP2 EIF2C1 WAC SMYD3 ELP3 CCNT1 PHRF1 STOM PAF1 MAF1 EIF2C2 YTHDC2 PCF11 BIN1 SPTY2D1 ERBB2 TAF10 CDC73 NEDD4 CTR9 PPIB RRN3 KIAA1967 RPRD1B C14orf166 SMYD2 PABPN1 ZNF326 IKBKAP GSG1 ELP4 SCAF8 PKN2 RECQL5 ZFP36 CCNT2 ANP32B ELOF1 KIAA1530 BRD4

GO_PALMITOYL_COA_HYDROLASE_ACTIVITY Catalysis of the reaction: palmitoyl-CoA + H2O = CoA + palmitate. ACOT7 LOC344967 ACOT8 THEM5 BAAT ACOT4 PPT1 GNPAT ACOT1 THEM4 ACOT2

GO_ARMADILLO_REPEAT_DOMAIN_BINDING Interacting selectively and non-covalently with the armadillo repeat domain of a protein, an approximately 40 amino acid long tandemly repeated sequence motif first identified in the Drosophila segment polarity protein armadillo. Arm-repeat proteins are involved in various processes, including intracellular signalling and cytoskeletal regulation. CNOT1 TCF7L2 RGS20 RGS19 CTNNBIP1 LEF1 STRN4 CHD8 AXIN1 AXIN2 CALCOCO1 STRN STRN3

GO_ACID_PHOSPHATASE_ACTIVITY Catalysis of the reaction: an orthophosphoric monoester + H2O = an alcohol + phosphate, with an acid pH optimum. ACP1 ACP6 ACP5 ACPL2 PPIP5K1 PAPL MINPP1 ACP2 PPIP5K2 ACPT ACPP

GO_ENZYME_BINDING Interacting selectively and non-covalently with any enzyme. ABCA1 STAT5B PPP2R5A BRMS1 TSG101 CAP1 SELE CREB1 EIF2C2 TOM1L2 ARHGDIB MAPK7 STOM GSK3A TMEM189 ATG13 BRSK2 RB1 TBC1D5 PPP3CB DIAPH3 INSL3 CENPJ DIO2 UBC PPARGC1A STIM1 CD40 RABGGTB VDAC1 APOC2 ELOF1 TRIB1 SCAF8 FXYD7 NIPBL KCNH1 NR5A1 CAMK2D GFAP PPP1R3D NKD2 CSE1L NOTCH3 MET AIMP1 ANK1 CSPG4 TOLLIP SMG7 TRIM22 ADRA2A ATP2A2 GRASP HIST1H2AM CDKN1B AICDA S100A1 ERC1 ENSA JTB APOA1 FGD6 DDX20 NBR1 SERPINB6 BAG4 BIN1 SLC6A3 CHIA MCM10 MLLT4 CCNK BRCA1 CAMK2N1 GRIN3A PPARG JUN TSPAN5 SHOC2 ESR1 TOB1 XBP1 SNRPD3 SPRED2 FZD4 MAP3K13 MICAL1 STC2 ANK2 RNF144A FLT3LG BECN1 UBXN1 GGA3 CCDC99 MAPK3 EGR1 DDRGK1 LRP4 UBE2J1 SERPINA1 TIMP3 IL12RB2 SERPINB13 SPTBN1 SOX9 HPS4 CAP2 ATP2B4 ROS1 USP22 RANBP3L ARF6 CYP1A1 CDH2 RPTOR XPO5 CAB39 CBX1 PPP1R15A PTK2B PHACTR4 PTPN6 HMGB1 SERPINB4 BIRC7 JAK2 PRKCZ ATG3 MAP3K12 SEMG2 AKAP7 ARHGEF2 ANAPC5 PJA2 KPNA2 DENND1B CCDC88A ABL1 ABI2 CAT DBF4B RNF20 TNFAIP3 SH3GL1 EXOC1 RBX1 ANKRD32 PHKG2 NOXA1 ERRFI1 FZD8 ERLIN1 RAB34 APC TENC1 MICALL1 POR PTPN2 PPP1R3C TRIM68 GABARAPL2 NEK9 HERC2 CAMK2N2 TBC1D22B PRKAR1A TBC1D30 SP2 ZBTB4 RPS19 CCND2 ADIPOR1 SNAI1 CST9LP1 PIAS4 RPS19BP1 ZMYM2 FOXL2 KAT8 A2M SPTY2D1 FMNL1 C9orf72 TIMP2 DACT1 EIF4E2 DLG4 HSPA1B TNFRSF1B TBC1D2 DAOA ATP6V0A4 SHC2 DNAJA3 NCF2 RPS6 PPP1R32 DMBT1 STX1A ELP3 DLG1 MUL1 HSPB1 CADPS UNC13B WAC ADORA2A PAK1 TBC1D16 GPI SMAD7 TRAF1 SCAMP3 SYK ATP6AP1 PPP6R3 PDE3B PDCD10 STX1B AP3B1 UBQLN1 PLAUR GAS6 OSR1 PLSCR1 OS9 GCN1L1 RAB7A CUL4B NRG3 TAB1 TBC1D8 IKBKG TGFBR2 MAT2B BTBD11 BORA ATXN3 EPHA1 CAV3 UBE2C AKAP8L LDB3 PRKCH CDK19 YBX1 UGT1A10 EEF2 BTBD9 FGD4 TBC1D22A MLPH PIH1D1 SDC4 SUFU RALA SPDYE2L MSH3 SP7 MSN STXBP6 PCBP2 TYSND1 SART3 CCND1 DAPK3 PER3 RAB3D CASC3 DMXL2 PPP1R21 CDC42EP4 ASB10 SRGAP2P1 STK39 PARP1 MAPK14 TCIRG1 SCNM1 RNF34 UBE2U ACTG1 CALM1 DIRAS1 HDAC7 BCL10 IGSF9B CCNA2 DOK7 RANBP9 ABTB1 BRAF IL1RAP NOP14 CDC5L RNF8 DCUN1D2 PACRG DYM MICALL2 TRAF6 CUL1 HM13 SDF2L1 C10orf46 LIPE RICTOR PPARA SUMO1 CYB5A NPC1L1 PAF1 MAML1 ETV3 ITGB1BP1 CPSF1 PTPRN ECM1 AP1B1 UBE2A CCNB1 DCUN1D3 PFN1 SPDYA CCNB3 STK11IP PTPN1 TNIP2 CD226 GPR37 RAD23A MARCH5 PYCARD SKAP1 CASP3 CDC42BPB SKOR2 UBXN7 EPRS RUNDC1 VCL PLCB1 LDB1 CLTC BAG6 DAXX CYP3A4 NEK6 MAGI2 PGAM1 HIST1H2AL TEX14 ARIH1 CUL9 UFD1L FAM83H TIMP4 EMP2 SHC3 KDM1A DCX GDI2 RXRA BHLHE41 SPRY2 SQSTM1 TNNI3 DGKI VRK3 AKIRIN2 YY1 STRN HSP90B1 CST8 KIF11 LSM2 RGS19 PPP1CA CNTLN NTRK1 KSR1 SLC4A2 ATP1A1 PAWR TBC1D10A PEX7 H2AFX APOB IPO9 FLOT1 TUT1 HSPA1L TUBA1B WDTC1 LEF1 TRPC5 MSH2 USP7 RPS2 UBE2F MOBP CTNNBL1 PIH1D2 TMBIM6 ARHGEF16 TELO2 SERPINB12 TRAF3 RALB RHOBTB3 TRIB3 AP1G1 LDHB CSRNP2 KIF20A IQCB1 TBC1D20 SVIP CEP68 SIAH2 CUL4A SELL SRF SH3BP4 SPG20 RIMS1 TBC1D2B HSPA9 PRKACA SERPINB9 RB1CC1 CST6 ITGB1 CACNB3 ANKFY1 STK38 RIN3 PDPK1 SYTL2 RPA2 MAP2K7 C14orf166 ALPI CASP2 ACE GCET2 DNAJB1 TOR1AIP2 BRSK1 FAM83B DERL1 TIAM1 INF2 RHOH SRSF2 UBE2V2 KLF4 TCF7L2 NCS1 RDX MLEC DLG2 MEF2C CYP2C19 WAS RAF1 CASP9 STX8 IRS1 RELA USP6NL BLZF1 GLDC MEF2B NR1H2 FAM150A ATF7 PLN RYR1 MAPT DLG3 USP13 KLRK1 AKAP12 SIRT3 FBXL2 RGS14 PA2G4 UBE2V1 NKX2-1 TOM1L1 ADPRH NDUFS2 F3 CST11 TBCK CDK5RAP3 EID1 UTRN ARIH2 UBE2J2 POLA1 SYTL5 RNF14 RASD2 RAB7L1 ZNF259 HIST1H2AI PTPRR FOXO4 HDAC4 STOML2 PRDM4 MAGEC2 DENND1A POLR2A TDG CSF1R TPCN2 SUMO2 CRK NXT1 ANP32B PIAS2 ACD RABGEF1 IL31RA ACTA2 SGSM1 RNF114 PPP1R37 ANKRD27 SERPINF2 SLC22A18 EIF3A MAPKAP1 SPDYE4 EIF4E PLCG1 AXIN1 SPDYC ATP6V0A1 ANKRA2 GNAT1 TNPO2 RANBP1 UGT1A7 ADCY2 PKD1 PTEN SIX3 EXPH5 HIST1H1B TRAF5 DMD PTPN14 SYT11 CCDC50 LTBR IPO4 CCDC64B RHOD HMGA1 RPH3AL CAMTA2 NR2E1 CCBE1 WFS1 MYOZ2 AKAP13 STAP1 MAP2K4 AKAP5 SMYD3 TNKS2 DHX36 KCNQ1 DNM1L ANKLE2 BICD2 PPP1R3B LAMP2 TPX2 UBE2R2 MAG RNF139 RANBP2 CBX5 BAG5 CHCHD3 SH3YL1 FRS2 FLT4 CNTNAP2 SETMAR PTPLB RBBP4 RPS3 ADCY6 HAUS7 SNCAIP TBC1D3B CYP2A6 AURKA PPP1R3G PPP1R9A ANXA2 ZP3 PARD6A C1QBP FAF2 BTBD6 NHP2L1 NSFL1C KIF16B C13orf15 VAPB SERPINA5 DNAJC3 PRR5L CDH5 ATP6V1E1 ZNF675 MARCH6 WWOX JUND DZIP3 BFAR ZMYND15 ROCK1 DNMT3B RAB11FIP5 SIK1 NFRKB PKP2 LIMS1 TTN PANX1 ATP6V0C TRIM28 HOXA10 SPRED1 MC1R MMS19 CTNND1 RAPGEF4 MECP2 EXOC4 UBE2N BCAS3 SPHK2 NCKAP1 ACR ATF6 PRKCB TRIM6 PAG1 PRKAB1 APP FBXO7 SLC6A4 CST2 PPIB XPO4 CUL7 RCOR3 ENO1 TUBA4A CHMP4A RNF41 FOXO3 POU5F1 FMNL2 CNPPD1 BAD MID1 YES1 PAX6 DACT3 SCN5A FAM83A CSTL1 TP73 TOPORS ZNF346 PRKAG2 ZP4 SPAG9 SIRT1 LMNB1 AVPR1A PRKCA PLA2G6 TOP2A INS CYP2E1 MYOCD ATP1B1 WASH1 MTF1 VCP SPDYE3 PDCD2 LONP2 LLGL2 FADD UBE4B TSPAN14 FAS C10orf2 MAGEA3 PARN DNAJC10 SLC25A5 ASB16 ABAT IFT20 RNF217 SPDYE6 ELFN1 SLPI YWHAB TIRAP ERN1 FAM83E IGBP1 HTT ANGPT2 APPL1 BCOR CKS1B SLC12A2 CTTNBP2NL KCNH2 UBE2G1 SRCIN1 MAGEA2B UBE2B GNB1 TGFB1 INSM1 TRAF2 PIK3IP1 NUP50 TSPYL1 SLC9A3R1 HDAC10 IPO5 TCF3 HLCS UBE2T NFKBIA MBP BLNK GLRX3 POLB FANCL ZNF746 KIF3B CEP250 MYH6 PLK1 MLKL FBL SFI1 MYO5B VWF EGR2 FGD2 TBC1D1 GRB7 CRY1 LATS1 RNF19B TNFRSF10A RNF152 HSPA2 MOB1B NEDD4 MALT1 XPOT TBC1D21 CHML VPS41 TRIB2 FHIT KLRC4-KLRK1 SHC1 NACC2 TNFRSF14 HNRNPUL1 PARK2 EIF4EBP1 MC4R MAP1LC3C CCND3 ARFIP2 RUSC2 TRIOBP ATP1B3 IL6R DIAPH2 BRD4 ARRB1 BTBD3 LCP1 EHHADH CST9 EEF1A2 PRDX6 CTSC CAV2 RNF31 GDF3 NFATC1 ITPKA USP33 BRCA2 ITGB2 BCR SELS PHF6 JAKMIP3 RIN1 XPO1 STUB1 HIF1A HSPA8 FYN NOTCH1 AMBRA1 STRN4 ZC3HC1 UBE2I STAU1 YWHAH RAB3A OBFC2B PTPN23 ANKRD1 UBE2G2 MARCKS PTPLAD1 SPOPL SHC4 CDKN2D DNAJA1 CST1 UNG DNM2 USP2 YTHDC2 CNST AKAP8 CDKN1A CDKN2C SMAD6 CHMP3 SPDYE1 DUSP12 BRMS1L ACAP2 SYTL3 GRIK2 GABARAPL1 HMOX1 CALM3 PHB USF1 GPRC5B DCUN1D4 TOP2B SMAD5 RAB11FIP4 SREBF1 PEX19 MTOR SYN1 TBC1D25 PTPN11 EIF2AK3 SLC27A2 SPAG16 CD70 GOLGA2 FGFR1OP ZNF326 MTA2 PIK3R2 ERLIN2 RAB3GAP2 PHACTR1 C15orf62 FANCD2 UBE2W KIAA1967 ATP6V1G3 PCNA GLMN HLTF AP2A2 TRAK2 MVP RPS18 JAKMIP1 RASSF5 RPH3A WHAMM CCNYL1 STRN3 CALM2 POMZP3 MAPK6 RAB4A NUFIP1 CSF3 ADAM9 BCL2L1 KLHL11 FANCI FOXM1 KIF14 SORT1 USP37 CHM CDC25A ZFYVE20 EHD1 MAP3K2 OCRL PIWIL1 IQGAP3 TRIM5 DOCK7 PITPNM3 NCOA6 CEP152 NGFR C6orf211 IPO13 RASGRP3 GLI3 TAOK2 SNX10 CBLB CXCR4 CORO1C HNRNPD PGR CBX3 HCLS1 TCF21 PRKAR1B CDK5RAP2 CCDC64 TRPV4 MAGEA2 PTPN22 NKAIN1 EEF1A1 CCNE1 TRPC4AP BRK1 MAPK4 CYP1A2 H2AFY APBA3 ELANE PKN1 FGR CKS2 UBE2H RNF166 RAB11FIP2 GCLM TARBP2 LAMP1 SOD1 CD4 GSTP1 SMAD2 SP1 CUTA CDK5 FZD6 NLK SMAD3 PTPLAD2 FIZ1 RAP1GAP UGT1A1 ASB18 MYOD1 TRAF4 LILRB2 PRKCD AKAP11 DOK2 RNF185 RANBP17 COMP LRP1 LRRK2 NEK2 MAP3K5 MASTL LUC7L2 SMG5 TRIM37 PRKCE WWC3 GSTM4 IARS DNM1 PCF11 TBP PPP1R36 FAP PAFAH1B1 HDAC5 NDRG1 DUSP19 UBE2NL TANK TNKS1BP1 MAGEA1 PDGFRB RFC4 RIPK1 HIC1 GDI1 MAP2K3 HINFP GMNN DFFB TOR1AIP1 CDK5R1 CRADD SHPRH HYAL2 CDKN2A BID STXBP5L PRKDC RRAGA XPO6 THY1 FAM150B STRADA PTPRC PUS7 ADPRHL1 EXOC8 SMG6 ECT2 PLCE1 PFKFB1 MAVS TBC1D9 MPHOSPH8 PFKM PRKAG3 AR GCM1 PRKRA LDHA PAK3 MAF1 GRTP1 MAP2K6 PPP1R26 SLC12A4 TBC1D9B XRCC1 ADRB2 STX17 DOCK11 NOS1AP TSPAN10 NOP56 ARPC4 CSK PTAFR PER1 PRKCSH SKI CDC34 BICD1 RAB13 NLRP1 BTBD2 ATF5 STXBP5 ILK CHP RPS6KA3 DIS3L MST1 MAP3K1 RAPGEF6 CST3 CCT2 ARHGEF7 ALS2 ADCY10 LDB2 TMEM173 MAD2L2 TBC1D10C DENND5A SLC9A1 RGP1 PFKL RGL3 RNF125 PPP1R3F CIT ITGA3 PIAS3 GAS8 PHRF1 BARD1 CDKL5 C2orf56 PAM PPEF2 SFPQ UHRF1BP1 FAM83C HSP90AA1 DIAPH1 RPS6KA4 FURIN UNC13D JAK3 IGF2R SUDS3 ADCY5 TRIM72 FAF1 IBTK NOL3 ADRM1 PKD2 TRIP6 GBP1 SRC GSTM3 GSTM2 TNPO1 ANGPT1 NDUFS7 CRB2 LRPAP1 HINT1 SORL1 ZP2 RAP1A HDAC1 ITGAV TIMP1 CDC20 MEF2D NR2C1 ELMO2 NR4A3 RASA1 CFLAR TCTEX1D4 ZNHIT6 ZFHX3 DNMT3L ZNHIT1 LYN PHKG1 SNX9 PDLIM5 CST9L SPTBN4 UBE2Z ASB3 TRAT1 DDC ATF2 KIAA1267 RABGAP1L SNAP91 PRKAR2A PPM1D NET1 FLOT2 IPO11 KIT MAPK8 MEF2A PRKACB ZBTB7A CAV1 CDC42 CPNE3 NOD2 SPDYE7P YOD1 RILP AXIN2 MST1R ATXN10 JUP SFN GSG1 PEX5 CDC37 PPP1CC MAST2 WASF1 SIT1 MAP1LC3A YWHAZ SYTL1 LONP1 RPRD1B EXOC2 CTR9 IQGAP1 LAT TAF7 TSPAN33 PEX26 ADAM10 ETS1 NOX1 SNX3 CDK2AP1 NEFH STAT6 PPP1CB KPNB1 PPP1R35 PIN1 DNM3 MYO5A DUSP3 PLIN5 HIST1H2AG CYLD DAAM2 TPRN ALDOB PLK1S1 GSTM1 ROCK2 EVI5L KIAA1432 DPYSL2 NPM1 BDKRB2 RNF5 DACT2 PRKCDBP HES1 N4BP2L2 SRSF1 TBC1D12 SGOL1 MAP4K2 RIMS2 AP3M1 FER SUPT5H UBE2M ANKIB1 MTSS1L TICAM1 TRIM49 PRKAA1 DUSP2 GYS1 HDAC2 SMYD2 CCNL1 PPP3CA CD24 GOLGA5 SRI TNFAIP1 DAAM1 GOLGA4 ARRB2 PIAS1 UBE2S WWC1 RAD18 PPP1R39 TUBB JAKMIP2 HDAC9 MLL5 ASB14 TBC1D8B SRSF5 CARHSP1 FGD1 CUL5 PTPN3 NEDD8 TSPAN17 PXN FGD5 SPOP CDC25B SV2A MOB4 NPC2 MDM2 IL1R1 CASP8 C2orf44 SLC26A9 CYFIP1 CTNNB1 SNTA1 CD28 GNB2 RECQL5 CDK5RAP1 UBE2L3 NME2 THAP5 ACVR1B PTK2 RARA TBC1D14 CD8A TSPAN15 ITGA1 PABPN1 SERPINE1 AVPR1B ANGPT4 UBOX5 MICAL3 PRDX3 KIF13B CKB PPP2R2A RRN3 FNTA HEY2 PRMT1 MYRIP CLU CEP192 PDE4DIP KCNN4 LDLR GSK3B SMAD1 PARP16 BCAR1 PDE6D PARK7 MICALCL DBT PHACTR3 TSKS TCEB2 DOCK4 KDM4C CSDA BNIP3 HNRNPA0 IQGAP2 FXYD3 HSPA6 YWHAE KAT2A E2F1 DTX1 PLEK NSF RANGRF FAM178A PKN2 ECD PFKFB2 TBC1D4 PTPRJ RNF40 TSC2 POLD1 KAT2B SGSM3 IKBKAP PPP1R12A CALR RAB11FIP1 PAK2 LLGL1 PPME1 PPP1R12C HDAC6 HAND1 RNF19A RAD51 RFC2 ERBB2 PREX1 NCK1 NKX3-1 GCHFR CDC42EP2 CBL TRADD P4HB ATP6V1G1 DVL3 EZR IPO7 CDC6 SYTL4 MARVELD3 VRK2 WWC2 TAF9 ARHGAP4 PPP2R4 ADCYAP1R1 MOAP1 ADAMTS4 TBC1D3H ANAPC4 PTGS2 RFC5 PINK1 ULK1 PPP1R27 FEZ1 RTKN JAK1 LCK DCUN1D5 ACTB RABGAP1 BCL2L14 SPHK1 FMNL3 CDC73 HRC FAM83D CCNY CRY2 GOT2 RAB11FIP3 TEP1 CD74 YWHAG GOLPH3 POT1 NUDT21 KRT79 TNIP1 ITGB3 ABTB2 HOXA9 GATA6 PRKAR2B WNK1 AP2A1 TMEM127 NRIP1 ACIN1 TNF SPDYE5 TBC1D13 PEX5L APOA5 SIRT2 SMC6 SLC9A3R2 CSTB METTL21A CFTR ANAPC7 PLA2R1 CEBPB NPM2 PPP6R1 CASP10 WDR70 CDC25C KBTBD4 FXYD4 RGS20 MFN2 BIRC5 SP100 KIF5B CDC42EP1 FN1 LRPPRC RCC2 NBEA DVL2 FOXP3 EXOC5 JKAMP TBC1D10B ELP2 CDKN2B TP53 BTBD1 LGALS9 XRCC5 CUL3 EPAS1 ZFP36 SLC2A1 DDX5 RGPD8 HSPA5 PEBP1 RNF144B TPR UBE2K FLNA FZD5 C1orf88 SMARCA4 CCNE2 OPTN PCCA HSPD1 NPR1 SDPR CST7 CCNL2 SRGAP1 SLN SERPINB3 KCTD13 DHCR24 ASB4 IKBKE GNB3 GPIHBP1 IKBKB HDHD2 CCNT1 TFAP4 STAT1 TPI1 RFFL AKT1 BCHE NEFL MAP1LC3B EGFR PARD3 PARP4 IRS2 TWF2 VRK1 ELFN2 CD3E PRDM12 MDM4 EVI5 IPO8 NPLOC4 PTPRK ELP4 AKTIP PICK1 CUL2 SH2D4A EPS8 PRKAG1 PPP1R12B ATP1B2 ATPIF1 TRPC6 DGKQ ACSL3 CBX4 PPP1R9B ANKRD2 TBC1D15 PHLDB3 KCNA5 HMGCR TAF10 NUTF2 PKIA SERPINC1 VHL CDC27 HACE1 KDM4A RAB3GAP1 RACGAP1 UCHL1 ANAPC2 OTUB1 CSTA TRAP1 IFNAR2 EIF2C1 MAPK1 ARPP19 PLSCR4 U2AF2 RPS6KB1 USP19 TAX1BP1 DVL1 CCNT2 SLC12A7 DSP NOXO1 COTL1 CBS STAT3 NOP58 DAB2IP GAB2 TAF7L LAX1 CST5 ESR2 MAPK8IP3 HDAC3 ACVRL1 SGSM2 RAD9A GATA4 SRSF3 GRK5 STXBP1 AKAP6 CIB1 CBLC SNCA ATP6V0A2 CHEK2 AMFR GHR CEBPA EGLN1 PIK3R1 MYO1C FBXO5 USP25 ASB15 MAPK8IP2 GRB2 GNB2L1 ADAMTSL4 SYVN1 TXNIP PML HIST1H2AK HSP90AB1 PRC1 TAL1 TBC1D26 THRB BCL2 TRIP4 DPP4 RFXANK TBC1D7 SCARB2 CHL1 MYO9B HPS6 NAE1 ELL NCOA1 CCNYL2 TPRKB SBF2 BTG1 MAP3K11 GTF2I SOCS5 ABCD1 UBE2O TCP1 RABGGTA PIP5K1A C2orf67 UQCRC1 NCOR2 ACAT1 RAB8A RNF180 RNF138 C10orf90 TRIO TH BACE1 LILRB1 MCM2 XPO7 FBXW7 PRKRIP1 RANGAP1 ANKMY2 SOCS1 CITED2 KIF3A TULP3 RAC1 PPP1R18 SRGAP2 CDC42EP3 GGA2 HPCA PRDM1 RYR2 FGD3 SNX12 POLG TBL2 NCOR1 BBC3 CDC42EP5 TBC1D3C FBXW5 KIAA1530 SASH1 CST4 MAPK8IP1 ELAVL1 PRTN3 PDE4D SRGAP3 CAD DCUN1D1 NME1 FOXO1 NRG1 RHEB RALBP1 MIDN MAP2K1 MYOC HSPA1A CEACAM1

GO_ATPASE_ACTIVITY Catalysis of the reaction: ATP + H2O = ADP + phosphate + 2 H+. May or may not be coupled to another reaction. PSMC5 ATP13A1 DDX43 PIF1 DHX34 ATP6V1F ABCA5 DDX27 DNAH11 DYNC2H1 MNAT1 AFG3L2 KIF3B ATP13A2 MYH6 DDX51 ABCA7 DHX57 RECQL4 ATP1A3 LONP1 RAD17 ABCD2 ATP6V0A2 DHX29 ABCG2 CRBN PMS2P3 MYH4 CHD2 DDX18 MYO3A ATP11C ATP5D PSMC4 MLH3 ABCA11P ATP8A2 RAD51C ATP6V1C2 ATP6AP1L KIAA0564 DDX21 DDX49 ABCC1 MSH6 ABCA4 RNF213 TOP2A DQX1 XRCC6 HSP90AA1 SMARCA2 ATP1B1 ATP5G1 VCP ABCG1 MYH8 TCIRG1 CHD1L MYO1B ASCC3 LONP2 ATP7B KIF18A ASNA1 ABCA8 SUPV3L1 MYO1D ATP5EP2 PICK1 DHX15 CLPB CHD4 ABCF3 ATP1A2 OLA1 TDRD12 ATF7IP DNAH7 KIF19 ATP1B2 FBXO18 ATP5L KIF5A CHD5 ABCA2 ATP5F1 ATP13A3 KIF20B ATP10D DDX56 VPS4A RFC3 ATP8A1 DHX40 MYH14 ERCC2 DDX31 BPTF EIF4A3 NBN TOP2B RECQL5 DDX19B KIF23 GTF2H2 RTEL1 KIF1A DHX30 ATP6V1B2 RFC1 KIF1C ABCF2 DDX39B ABCC11 FIGNL2 RALBP1 HSPA1A KIF13B DDX3Y ABCC9 ATP6V1E2 KATNA1 DNAH17 ABCE1 TAP1 RAD18 DDX1 KIF3A KIF2A ATP12A DYNC1H1 PEX1 YTHDC2 STARD9 ATP6V1G2 ATP13A4 RHOBTB3 DDX23 KIF20A CHD8 DNAH8 KIF26A KIF22 RAD54B LONRF2 ATP5G3 ATAD2 PEX6 ATP1B3 ABCC6 PMS2P1 MYO9B DNAH9 ATP1A1 ABCD1 ATP6V1A KIF21A FIGN ATP13A5 IGHMBP2 ABCB4 ATP2A1 ATP2A3 MACF1 HSPA8 SNRNP200 ATP8B2 RAD50 SMARCA5 DDX41 MSH2 ANXA1 ATP2B3 ABCD4 DDX39A G3BP1 ABCB11 MYO19 CHD6 ABCF1 ATP2B1 ATP11B ABCD3 DDX50 MYO1C VPS4B MYO1E ATP2C2 ATP6V1D PMS2 ABCA12 TOR3A ERCC8 ABCB8 ATP5I MCM6 SPO11 DDX25 RAD51B ATP7A RECQL DMC1 DHX16 ATP9A ABCB6 ATP5H KIF11 DDX46 PPP2R4 ATP5B ERCC6 MYH3 SKIV2L ABCA3 ABCB10 DDX11 RFC5 KATNB1 KIF6 ATP9B KIF3C ATP8B3 TTF2 MCM7 DDX54 KIF17 MYL6 DDX19A MRE11A XRCC3 ATP6V0A1 DHX9 CHTF18 KIF4A RFC2 RAD51 GTF2H4 KIF2B SMARCA1 ATP2B2 CLPX DDX4 DSCC1 DDX20 ATP2C1 ABCB5 ABCC4 ABCC2 KATNAL1 KIF21B ATP5A1 ATP6V1G1 DDX53 KIF2C DDX52 DDX17 NSF KATNAL2 CHD3 ATP6V1C1 KIF14 ATAD1 MYO7A PCYOX1 ABCB9 ABCB7 KIF7 DDX28 ATP5O ATP2A2 DNAH6 ATP5C1 KIF15 ATAD2B TDRD9 C19orf39 ATRX ABCC10 ABCA13 ABCA10 EIF4A1 MYH9 ABCA1 DNAH1 TNNT3 ABCC12 ATP5J2 CLU ATP8B4 ATP6V1G3 PSMD6 KIF13A MOV10L1 DNAH10 DHX33 LONRF1 CHD1 DHX8 INO80 KIF27 DHX38 ATP1A4 DDX10 HLTF SPG7 ATP4A DDX3X FXYD2 GTF2H3 DDX47 ATP5J HSPA6 ABCA9 DDX55 WRNIP1 XRCC5 DDX5 ACTC1 ABCC8 DDX59 C9orf102 HSPA5 PSMC3 PSMC1 MLH1 ABCC3 WRN DDX12P ATP6AP1 DNAH2 ATP5G2 SMARCA4 PSMC2 HSPD1 GTF2H1 ERCC3 POLQ KIF16B TAP2 KIF5B HSPA1B PMS2P5 ATP6V1E1 ABCC5 EIF4A2 YME1L1 SMARCAL1 ATP6V0A4 ATP6V0B CENPE LOC100130097 KIF24 TOR1B IDE ATP6V0E2 ABCA6 ATP6V0C ATP11A MYH7 DHX32 DHX35 HELB DHX36 ATP6V0D1 ATP10A ATP5E LONRF3 ATP6V0E1 KIF4B KIF5C ATP4B ACIN1 XRCC2 DDX24 MCM4 RBBP4 RSF1 TAPBP BRIP1 KIF25 CFTR CARNS1 PSMC6 MDN1 ABCG5 CDK7 ABCG4 KIF26B KIF9 ABCG8 KIFC2 RUVBL2 ATP2B4 MYO18A TOR1A DNA2 ATP6V1H BLM DDX42 ATP10B CCT8 RAD51D MYH10 KIFC1 ABCC13 ATP8B1 DHX37 ABCB1 SPAST KIF12 PMS1 DNAH12 FIGNL1 RFC4 KIF18B KIF1B RUVBL1 DDX6 KIFC3 UPF1 CCNH MYO10 DNAH5

GO_TRANSFERASE_ACTIVITY_TRANSFERRING_ALKYL_OR_ARYL_OTHER_THAN_METHYL_GROUPS Catalysis of the transfer of an alkyl or aryl (but not methyl) group from one compound (donor) to another (acceptor). MAT2A MGST2 COX10 GSTA1 MMAB GSTT2B PDSS1 RABGGTA CLIC2 CLIC4 GSTM5 RABGGTB TRIT1 GSTP1 GSTM3 MGST1 GSTM2 CLIC5 LTC4S NUS1 GSTO2 GDAP1L1 GDAP1 GSTK1 MAT1A FNTB CLIC1 CHM HMBS GSTA3 HPGDS COQ2 CLIC6 PDSS2 MGST3 ALOX5AP PGGT1B FNTA CLIC3 CTH GSTA2 GSTZ1 EEF1E1 SMS FDPS GGPS1 GSTA4 EEF1G GSTM4 FDFT1 GSTA5 NANS AGPS DHPS DPH2 GSTM1 SRM GSTT1 UBIAD1 PTAR1 GSTO1 DHDDS GSTT2

GO_DNA_DIRECTED_DNA_POLYMERASE_ACTIVITY Catalysis of the reaction: deoxynucleoside triphosphate + DNA(n) = diphosphate + DNA(n+1); the synthesis of DNA from deoxyribonucleotide triphosphates in the presence of a DNA template and a 3'hydroxyl group. REV3L CHRAC1 CCDC111 POLQ POLB POLE2 POLD3 POLD4 POLH POLI POLD1 POLA1 POLK PAPD7 POLG2 MYBBP1A POLM POLL POLA2 POLN PAPD5 POLE4 DNTT POLE3 POLD2 ERVK-6 POLG POLE

GO_OXIDOREDUCTASE_ACTIVITY_ACTING_ON_THE_CH_NH2_GROUP_OF_DONORS Catalysis of an oxidation-reduction (redox) reaction in which a CH-NH2 group acts as a hydrogen or electron donor and reduces a hydrogen or electron acceptor. LOX VCAM1 LOXL2 AOC2 GLDC IL4I1 ABP1 MAOA GLUD1 DDO PNPO LOXL4 GLUD2 DAO LOXL3 AOC3 LOXL1 ASPDH MAOB

GO_STRUCTURAL_MOLECULE_ACTIVITY The action of a molecule that contributes to the structural integrity of a complex or assembly within or outside a cell. UBA52 MRPL14 TECTB MRPL10 FBN3 NUP85 KRT31 ANXA1 ARPC1B MRPL4 COL27A1 RPL36AL MRPS31 ADD3 CRYGS KRT35 OBSCN MCART6 MYLIP CAV2 RPS16 SLC25A20 CLTCL1 COL4A5 KRT72 KRT121P VILL SPRR2D GNB2L1 MAPK8IP2 MYBPC3 SNTG1 LCE1F STATH MRPL41 COL5A3 SHANK2 ACTL7B LAMC1 RPL22 KRT2 AKAP9 ISCA2 SLC25A6 NEXN LAMB1 MRPL22 MCART1 SYNM MUC3A RPL18 RPL12 SLC25A39 MAPK8IP1 KRT3 SASH1 ROPN1B LOR RPS21 KRT78 KRT18 MRPL16 RPL3L LCE3A SLC25A4 KRT38 RPL7L1 DCTN3 CLTB ACTR3 SLC25A15 LAMTOR2 KRT1 KRT6A CRYBB1 NDUFA7 RPS5 ANKRD2 SPRR1B SLC25A5 SLC25A10 RPL7A LCE3B COL4A4 MRPS22 KRTAP11-1 KRT82 MYH8 MRPL33 KRT71 TUBGCP6 CD3E CYLC1 LCE2A NEB MRPS33 KRTAP24-1 LAMTOR3 TUBD1 NEFL LMNB1 G3BP2 SPAG9 KRT10 RPL4 DISC1 SEPT2 SMARCD1 RPS4Y2 SPAG4 COL19A1 SORBS3 SLC25A18 EPB41L1 TUBA4A LCE3C RPL28 SLC25A24 AKAP6 MRPL46 RPL29 CLDN7 LCE3D SLC25A1 CTNNA1 ACTL7A MAPK8IP3 MRPL34 SYNC KRT222 MYOT ITSN1 COL11A1 DSP TUBE1 MRPS34 TINAGL1 LCE1C CAPN3 MBP SLC25A48 LAMC3 NPHP1 RPL18A SLC9A3R1 LAMA3 LCE5A RPLP0 LAMA2 AMELY DLG5 CSTA COL2A1 CRYBA2 RPL39P5 MRPS2 LELP1 AMELX KRT85 LCE1D KRT26 LAMTOR1 GNL1 RPL6 LAD1 MRPL17 COL8A2 FGG RPS9 MAP7D3 SEPT5 FBN2 IMP3 RPL32 KRTAP3-3 RPL10A OPTC RPS11 RPS3 SLC9A3R2 MYBPH RPL9 CHCHD3 RPL11 TUBA4B SLC25A30 ACAN CRYAB EPB41L2 KLHL3 POM121L2 COL4A6 MRPL35 NUP155 SCARA3 COL24A1 CLDN14 CLDN11 RPS15 SLC25A21 AKAP13 KRT79 JAG1 UCP1 PLP1 ADD1 MATN3 MRPS9 TPM4 MUC6 DMD MAP7 KRT27 COL1A2 PRPH RPS7 NUP153 COPB2 MYH11 ISCA1 MRPS16 MRTO4 MMS19 KRT73 PRR9 PNN MYL6B LCE2D CRYAA RSL24D1 POM121C NUP62CL IFLTD1 COL9A3 UCP3 SPTAN1 TTN PKP2 ERVK-6 RPL27 CLDN3 SLC25A17 SLC25A33 SLC25A45 BGN DEDD2 DAG1 ENAM TUBA3E RPS27A LOC100288814 RPL23 RPS10 KRT39 TUBA3D MRPL15 ACTA1 LLGL1 RPL21 SLC25A44 RPL35 RGS14 COL9A1 MRPS24 RPS17 CLDN22 MAPT FRMD3 EFEMP2 LAMA4 MAP1A CLDN10 MRPS5 CPOX KRT6C COPB1 TUBB2B GRIP2 KRT37 RPL38 HBXIP KRT15 TPM1 C7orf59 RPL26 TUBB8 CRYGC SLC25A3 CTNNA2 RPS14 RPL35A RPL17 LCE1E LUM MRPS11 CRCT1 KRT28 NCK2 RPS3A LAMA1 PRELP MRPL19 KRT17 ASPH TUBB4B HOMER2 AXIN1 MRPS6 AMBN KRT24 NUP62 ACTB MRPS35 CLDN24 KRT25 EIF3A SLC25A43 MRPS23 IMPG1 UPK1B SPRR2F RPL15 MRPL55 COPE LCE3E TUBGCP3 RPL13 RPL24 WWC2 SLC25A29 MRPL28 FRMD6 RPLP2 KRT13 RPL5 KRTAP5-8 DAP3 TUBG2 TUBGCP5 SEPT4 MYOM2 NCK1 INA CLDN2 VPS25 EPB41L5 RPL13A COL5A2 FLG2 MRPL30 RPS2 CLDN4 SEPT7 SLC25A2 NUP205 HIP1 SLC25A12 ERBB2IP MRPL9 MRPL47 TUBA1B RPL14 KRT19 TUBGCP4 ODF2 KSR1 MYL3 TUBGCP2 CRYGB TUBA1A CARD10 BFSP2 RPS4X ROCK2 LAMB2 SLC25A28 AHNAK CROCC KRT5 COPA RPS4Y1 RPS8 UCP2 MRPL21 MAP2 MYL5 MRPS12 NEFH SLC25A41 POM121B KRT36 MRPL13 MAGI2 SLC25A32 ERVK-21 RPL37A ERVK-5 BCAN FBLN1 RPS20 PLEC SLC25A25 RB1CC1 TUBB1 RPS23 RPS29 HSPB6 COL4A1 ACTN2 RPS27L CRYBA4 DES TUBA3C SPRR2A SNTA1 KRT40 POM121 PPL RPL7 NEBL YEATS4 TMEM48 PSMD13 KRT32 EPB41 CYLC2 LIM2 MRPL43 TELO2 NUP93 RPS10P5 ACTL6B MRPS18A SLC25A26 MOBP MIP TUBB WWC1 KRT20 RPL26L1 SLC25A38 ARRB2 RPL37AP8 RPS15A FRMD5 IFFO1 CLDN12 CLDN6 HAPLN2 KLHL17 KRT8 NUP188 SLC25A31 RPL10 ACTG1 CRYBA1 RPLP1 ISCU MRP63 BFSP1 KRTAP3-2 KRT14 MYL9 KRT7 NUP214 CLTA ERVK13-1 BVES MPZ CNOT1 PGM5 CRYBB2 CLDN9 RPS24 RPL22L1 SMTN SNTB2 MSN SLC25A47 EVPL MYBPC2 MYLPF HAPLN1 IMPG2 NCAN MCART2 MRPL3 MEPE NUP98 KRT77 CLDN19 NEFM MUC17 KRT84 SPRR2E FBN1 RPL10L SLC9A1 KRT16 TFPI2 MRPS14 POM121L12 NUP107 MUC4 SLC25A42 RPS27 CAV3 SLC25A14 MRPL49 MRPS30 MATR3 SPTBN2 ANK3 RPL36 CLTC SLC25A13 NES KRT83 VCL SLC25A11 KIAA0368 ARPC5 MRPL18 EIF3B JUP KRT81 PLS1 VCAN COL14A1 EPB41L3 C7orf44 CDC42SE2 CAV1 TLN1 RPL41 CRYBB3 MRPL24 SLC25A22 KRT75 NUP54 RPL8 KRT76 RPL23A SPTBN4 JPH1 EPB41L4B MAGI3 MRPL36 MRPL42 COL15A1 COL5A1 COL4A3 EPB41L4A COPG SPRR2G ARPC3 CLDN16 KRT33B MRPL23 MRPS36 TPM2 FGA FLG MRPL37 MRPS25 SHANK1 LAMA5 NUP133 COL9A2 LCE1B TUBAL3 MRPS7 SHANK3 LMNA CD2AP FGB RPL30 MRPL20 TMEM33 EVPLL SLC25A34 KRT6B RPL13AP3 RPS13 C1orf68 DUSP19 MRPS18B KRTAP1-3 KRT9 COL4A2 SPRR3 RPL36A SPTA1 RPLP0P6 C1orf130 MAP7D2 MPZL1 AGRN MRPS18C LCE2C WWC3 CHI3L1 SPTBN1 SPTB RPL27A CLDN18 SNTB1 B7H6 TUBB3 TUBA1C BICD1 RPL39 CCDC6 KAL1 CLDN1 ARPC4 MRPS17 PDLIM3 MRPL12 MRPS21 ACTN3 KRT23 OTOG SPRR2B NDC80 COPG2 DLG1 TUBG1 PSMD11 CLDN17 VIM CRYGA SLC25A37 RPS6 TCAP RPL19 MYBPC1 SPRR4 COL12A1 MYL2 SLC25A23 CLDN5 TECTA MAL PKP1 MAGI1 RPS19 FBLN2 TUFT1 KRT12 LDLRAP1 IVL KRT33A MUC5AC EPB42 SLC25A36 CLDN8 COL18A1 RPL39L LCE1A ANK1 RPL37 RPSA COL11A2 HAPLN4 DSPP TUBB2A KRT34 MRPL32 GFAP SLC25A27 SLC25A35 ELN RPL34 KRT86 EMILIN2 COL3A1 MGP KRT80 ACTR2 ERVK-7 CDC42SE1 NUMA1 SNTG2 ARPC2 RPS26 RPS18 MRPL27 GRIP1 CD3G TUBA8 C9orf142 CLDN23 RPS12 RPL31 SLC25A16 MRPL51 MYOM1 LCE4A KRT4 LRRK2 TUBB4A CLDN25 COL1A1 MYL6 COMP RPL3 RPS28 SLC25A40 PXDN MRPS15 KRTAP3-1 MRPL11 LMNB2 MRPL52 NPHP4 ADD2 TLN2 CD4 ANK2 CRYGD LAMB3 IFFO2 CSRP3 LCE2B C15orf2 KRT74 MAP1B MAP4 CLDN15 MRPL1 HAPLN3 TUBB6 SPRR1A BGLAP MYL1 MAP7D1 CLDN20 SORBS2 SLC25A19 MPP7 MFAP5 MATN1 MRPL2

GO_LIPASE_ACTIVATOR_ACTIVITY Binds to and increases the activity of a lipase, an enzyme that catalyzes of the hydrolysis of a lipid. PLAA APOA5 GM2A STX4 APOC2 CCL5 ARF1 PDPK1 APOH NSMAF CCL8 CASP3 CCL3

GO_VOLTAGE_GATED_CALCIUM_CHANNEL_ACTIVITY Enables the transmembrane transfer of a calcium ion by a voltage-gated channel. A voltage-gated channel is a channel whose open state is dependent on the voltage across the membrane in which it is embedded. TMC1 CACNA1F CACNA2D4 PKD2 GAS6 CACNB3 CACNB2 CACNA2D1 CACNG7 CACNA1C CATSPER1 CACNB4 CACNB1 TPCN2 OPRM1 CATSPER2 CACNA1I CACNG1 CATSPER4 IL1RAPL1 CACNA1B CACNA2D2 CACNG6 RYR1 CACNG8 CACNA1H TPCN1 CATSPER3 NCS1 CACNG3 CACNA1S CACNG5 CACNA1A TMC2 CACNA1G CALHM1 CACNG4 CACNA1D ITGAV GRM7 CACNG2 CACNA1E

GO_CATION_TRANSMEMBRANE_TRANSPORTER_ACTIVITY Enables the transfer of cation from one side of the membrane to the other. TRPV4 KCNK10 SLC6A3 FAM26D FAM26F ANO6 COX6A1 NIPAL2 SCN11A SLC35A4 GRIN3A KCNH7 SLC31A1 TRPC4AP NIPA1 HTR3E SLC6A19 SLC41A1 CACNG6 HCN4 KCND1 KCNB1 CLCA3P SLC12A1 COX7A2P2 TRPV6 SLC24A5 PDE2A RHCG CALHM2 SLC9A10 TMC1 CACNA1F KCNA10 KCNH5 UQCRFS1 C15orf48 NNT RHD KCNAB1 PKD1L3 ATP6V1G3 SLC22A14 TF SLC1A6 NOX5 KCNA2 ATP5J SLC22A3 SLC10A6 CCT8L2 TTYH1 FXYD2 TRPM4 SLC22A1 ATP1A4 KCNE1 KCNH1 SLC46A1 CHRNA6 SLC2A10 HTR3D ATP2A2 HTR3C ATP5O COX6A2 UQCRHL KCNC4 CACNA1E ATP6V0D2 SLC9A9 GRM7 RYR3 KCND3 SLC47A1 SLC13A1 ATP6V0E2 SLC12A4 ATP6V0B SLC1A7 SLC25A37 SLC13A2 HTR3B SCN1B ATP6V0A4 COX4I2 CACNA1S SLC39A9 NIPA2 RHCE SLC22A16 SLC17A4 SLC45A2 ABCC8 SLC45A4 SLC6A11 MMGT1 KCNK12 SLC5A1 SLC30A5 GAS6 ATP6AP1 KCNG3 SLC36A2 CHRNB1 SLC44A1 SLC4A5 CNGA3 SLC5A2 ATP2B4 CHRNA10 ATOX1 KCNQ4 GRIN2C ACCN1 NDUFA4L2 SLC23A2 ITPR3 CHRNG SLC6A7 SLC8A3 SLC41A2 SLC9A6 ATP6V0E1 SLC12A5 HCN3 TMEM38A P2RX2 KCNK17 SLC4A7 CYB5A PKD2L1 KCNE1L SLC2A6 CLDN16 COX6B2 KCNMB4 CHRNA4 P2RX3 SLC4A4 ITGAV KCNJ4 ITPR2 KCNE2 P2RX5 KCNJ16 SLC1A2 SLC13A5 NIPAL3 ATP6AP1L CHRNB4 SLC28A1 SLC13A3 GRIN3B SLC34A1 KCNQ3 KCNJ13 SLC33A1 SLC6A13 TRPM6 CATSPER2 CNNM4 CALHM3 SLC15A1 SCN9A SLC22A2 SLC6A12 KCNB2 MCOLN2 TMCO3 CACNA2D4 SCN10A REST SLC4A11 SLC9A1 DENND5A KCNK6 CNGA2 SLC35A5 CACNG4 CNGB1 ACCN2 SLC13A4 TRPV1 SCNN1D KCNC3 CACNA1G SLC3A2 SLC22A5 CNNM2 CACNA1H SLC24A2 KCNIP1 SLC5A3 TCIRG1 SLC7A8 CACNA2D2 TUSC3 SLC39A1 RHAG TRPM7 ATP1A2 SLC2A12 SLC5A9 SURF1 KCNF1 SLC6A14 SLC6A8 PKD2 SLC6A2 KCNA4 SLC44A5 CUL5 PKDREJ SLC6A20 CHRNB2 KCNK18 SLC39A12 SLC24A4 KCNAB2 SLC30A3 CHRNA5 COX6B1 CALHM1 KCNJ10 TPCN1 SLC30A10 KCNV2 SLC34A2 ATP13A4 SLC8A2 SLC28A3 SLC24A3 PKD1L2 COX8C KCNJ1 SLC6A17 SLC23A1 KCNJ8 CACNG7 TRAPPC10 KCNS1 UQCRFS1P1 TFRC KCNV1 CACNB3 SLC40A1 TMCO1 SLC6A9 NOX1 KCNJ12 GRIN2A GPM6A SLC30A7 KCNJ9 ZDHHC13 MRS2 ATP5H ORAI1 PKD1L1 SLC25A28 ATP7A SLC17A2 SLC30A9 COX5A SLC44A3 ATP6V1A SLC6A15 ATP1A1 SLC5A6 SLC10A2 DENND5B OPRM1 CACNG1 KCNJ11 ATP5G3 SLC30A1 SLC9A7 SLC5A7 COX4I1 SLC9A11 SLC39A14 CHRNA2 CACNB4 KCNIP4 CHRNA3 ATP2B3 SLC39A7 TRPC5 CHRNA1 ATP2A1 ATP2A3 CACNG2 ATP2C1 SLC6A18 HPN ATP2B2 SLC41A3 KCNN1 SLC35A1 ATP5A1 COX11 COX7C ATP6V1G1 KCNJ15 COX15 SCNN1G SLC9A8 CATSPER4 TRPM8 SCN3A COX10 SLC22A13 ANO10 KCNJ2 KCNE3 SLC5A5 ATP5B TPCN2 CHRNE SLC30A2 SLC25A29 SLC39A8 SLC32A1 TRPV5 SLC39A10 ATP6V0A1 KCNJ18 KCNQ2 JPH2 CACNA1C CATSPER1 SLC20A2 SLC9A2 SLC22A18 SLC2A9 ZDHHC17 KCNN4 SLC22A4 CACNA1D GRIN1 NALCN UQCRH KCNT2 KCNH6 C9orf7 SLC15A2 FAM26E NCS1 SLC10A3 ATP4A COX7B2 MCU IL1RAPL1 SLC15A3 SCN1A ATP6V1C1 RYR1 SLC44A2 SLC9A3 ANO1 ACCN4 KCNJ6 KCNK7 ATP5C1 KCNE4 SCN4B SLC15A4 KCND2 TMEM38B MCOLN1 ATP6V1E1 SLC11A2 SLC10A1 TMEM175 TMEM37 UQCR11 KCNAB3 ATP6V0C SLC10A5 PANX1 CACNA1A SCN3B SLC30A6 SCN4A TRPM3 ITPR1 ATP5S AQP1 HVCN1 CNGA1 NDUFA4 ATP1B4 SLC9B1P1 SLC5A4 SCN8A SLC6A4 CHRNB3 UQCR10 KCNK15 ATP5G2 SLC20A1 SLC6A6 SLC9B1 SLC10A4 MFSD3 KCNG4 MAGT1 SCNN1A CCDC109B P2RX7 PKD1 ATP6V1H ATP5L2 KCNA3 KCNK16 ACCN5 SLC30A4 SLC39A4 SLC36A1 SLC39A5 SLC6A5 ATP4B CACNA1B TRPV2 SLC39A6 ATP5E ATP6V0D1 SCN2B ATP6V1B1 HCN2 MCOLN3 SLC47A2 KCNQ1 FXYD4 STIM2 SCNN1B CHRNA7 ORAI2 SLC18A3 ZP3 JPH3 KCNMB1 KCNK9 GAR1 KCNJ3 HTR3A ATP5D MTMR6 KCNMB2 SLC12A3 TMEM63C KCNK3 SLC4A9 CACNG5 COX7A2 ATP6V1C2 KCNJ5 SCN2A SLC39A13 KCNH2 SLC12A2 ATP13A2 COX7A1 KCNK4 CNGA4 SLC12A7 TRPM1 TRPC3 SLC45A3 ATP6V1F SLC17A3 ATP13A1 PSEN1 ATP6V0A2 CHRND SLC9A5 TRPM2 ATP1A3 SLC36A3 P2RX4 COX6C SLC6A16 CACNB2 TRPC4 ATP13A3 KCNMA1 SCN7A ATP5F1 KCNT1 TRPC1 SHROOM2 RHBG SLC9B2 SLC35A2 ATP5L SLC12A6 SLC11A1 SLC24A1 KCNH8 KCNS2 SLC8A1 KCNG2 ATP8A1 SCN5A CATSPER3 SLC24A6 FKBP1B COX5B ACCN3 CACNA2D3 SLC12A9 ATP5G1 ATP1B1 TRPA1 HCN1 P2RX6 CACNA1I KCNN3 SLC31A2 COX7A2L PKD2L2 KCNA5 KCNC1 TRPC6 ORAI3 ATP1B2 KCNH3 KCNK13 SLC30A8 ATP5EP2 ATP7B KCNC2 SLC28A2 CHRNA9 RASA3 ATP12A SLC17A5 SLC6A1 KCNA6 SLC9A4 SLC2A13 RYR2 KCNA7 ATP6V1G2 LOXHD1 SLC17A7 CACNG3 PIEZO1 ZACN ATP6V1B2 SLC17A1 KCNU1 TRPM5 KCNA1 ATP6V1E2 ABCC9 KCNK2 UQCRB KCNIP3 SLC39A3 KCNJ14 KCNMB3 P2RX1 KCNQ5 COX7B KCNK5 SLC38A1 SLC44A4 ATP2C2 ATP2B1 KCNIP2 KCNK1 KCNH4 TMC2 SLC39A11 CACNG8 SLC34A3 ATP5I SLC2A8 SLC39A2 SLC1A1 C1orf31 ATP13A5 COX8A NIPAL1 SLC1A3 TRPC7 CACNB1 SLC25A20 ATP1B3 ABCB11 NIPAL4 KCNN2 CNGB3 UQCRC1 UQCRQ KCNG1 SLC35A3 KCNS3 TRPV3 CACNA2D1

GO_3_5_CYCLIC_AMP_PHOSPHODIESTERASE_ACTIVITY Catalysis of the reaction: adenosine 3',5'-cyclic phosphate + H2O = adenosine 5'-phosphate. PDE7A PDE1B PDE1A PDE4D PDE3B PDE4A PDE8B PDE3A PDE2A PDE4C PDE8A PDE11A PDE7B PDE4B PDE10A

GO_GLUTAMATE_RECEPTOR_BINDING Interacting selectively and non-covalently with a glutamate receptor. PLCG1 DLG1 OPHN1 CAMK2A FLOT2 HOMER1 HOMER2 SHANK1 CALM2 HOMER3 FLOT1 RAPSN FUS NETO1 CALM1 DLG2 GNAS SHANK3 CACNG2 DLG4 DLG3 DNM3 NETO2 SHANK2 CANX RAB4A CALM3 DRD2 NEDD4 RASGRF1 SYNDIG1

GO_TRANSMEMBRANE_RECEPTOR_PROTEIN_SERINE_THREONINE_KINASE_ACTIVITY Combining with a signal and transmitting the signal from one side of the membrane to the other to initiate a change in cell activity by catalysis of the reaction: ATP protein serine = ADP + protein serine phosphate, and ATP + protein threonine = ADP + protein threonine phosphate. TGFBR3 ACVR1 HFE2 ACVR1B ACVR2A LTBP1 AMHR2 ACVR2B ACVR1C BMPR1A BMPR1B ACVRL1 LTBP4 TGFBR1 BMPR2 TGFBR2

GO_TRANSCRIPTION_FACTOR_ACTIVITY_TRANSCRIPTION_FACTOR_RECRUITING Interacting selectively and non-covalently with a specific transcription factor, which may be a single protein or a complex, and with another protein, macromolecule, or complex, recruiting that specific transcription factor to the transcription machinery complex and thus permitting those molecules to function in a coordinated way, in order to modulate transcription. A protein binding transcription factor may or may not also interact with the template nucleic acid (either DNA or RNA) as well. TTF1 DMTF1 MYB CDC5L MYBL1 NR1H2 TAF1B LIF SNAPC4 KLF4 MYBL2

GO_O_ACYLTRANSFERASE_ACTIVITY Catalysis of the transfer of an acyl group to an oxygen atom on the acceptor molecule. LCLAT1 LPCAT2 LPGAT1 AGPAT4 SOAT2 LPCAT3 LPCAT4 CPT1B GNPAT AWAT1 ABHD5 AWAT2 MOGAT3 DGAT2L6 MBOAT7 MOGAT2 CROT CHAT MOGAT1 CRLS1 GPAT2 SOAT1 AGPAT3 LRAT CPT2 HHAT AGPAT9 AGPAT6 PIGW AGPAT2 IFNB1 PNPLA2 LCAT PLA2G15 DGAT2 LPCAT1 DGAT1 GPAM TAZ MBOAT1 AGPAT5 CRAT AGPAT1 PNPLA3 MBOAT4 MBOAT2 CPT1C CPT1A

GO_PRE_MRNA_BINDING Interacting selectively and non-covalently with pre-messenger RNA (pre-mRNA), an intermediate molecule between DNA and protein that may contain introns and, at least in part, encodes one or more proteins. Introns are removed from pre-mRNA to form a mRNA molecule. DDX5 EP300 PRPF8 RBM41 TARBP2 SNRPC U2AF2 SOX9 SLBP RBM22 HNRNPA2B1 SLU7 SRSF2 PRPF39 LSM1 ZRSR2 U2AF1L4 HNRNPL SRSF6 RNPC3 ZRSR1 PTBP2 TRA2B RBM4 ERI1 PTBP1

GO_NUCLEOTIDE_KINASE_ACTIVITY Catalysis of the reaction: ATP + nucleoside monophosphate = ADP + nucleoside diphosphate. AK3 MPP3 CARD11 AK8 MPP2 DLG4 DLG3 PNKP CMPK1 MPP1 AK2 AK7 CMPK2 DTYMK AK4 AK1 GUK1 CASK DLG1 TJP2 AK5 MAGI3 RAD50 DLG2

GO_TRANSCRIPTIONAL_ACTIVATOR_ACTIVITY_RNA_POLYMERASE_II_TRANSCRIPTION_REGULATORY_REGION_SEQUENCE_SPECIFIC_BINDING Interacting selectively and non-covalently with a sequence of DNA that is in the transcription regulatory region for RNA polymerase II (RNAP II) in order to activate or increase the frequency, rate or extent of transcription from the RNAP II promoter. SRF SOX2 CTCF DLX3 GRHL2 OTX1 ZGLP1 SREBF1 SOX11 HOXC11 PLAG1 NFKB1 TFE3 CEBPZ ESRRA ARID3C NR5A2 PTF1A ETV5 IKZF3 IRF2 NFATC3 ELK1 CSRNP1 HAND2 STAG1 EBF3 CSRNP2 KLF15 ISL1 GCM2 CREB3L2 EHF FOXA1 NFATC1 ELK3 NR4A1 SOX4 ONECUT2 TBX5 HIF1A LEF1 SPI1 GABPA NRF1 NFIB ONECUT3 BCL11B NR1H4 NFATC2 CTCFL AIRE FOXC2 CEBPA E2F4 HMGA2 EBF4 REL FOXC1 MYC HEYL SOHLH2 POU2F3 HOXA5 RFX6 STK16 NEUROG3 CREB3L4 HOXD8 MEF2A GLIS2 TP63 GRHL1 PAX9 STAT3 MEIS2 ELF3 FOSL1 FOS RFX4 PBX3 GATA4 EGR2 NEUROD1 EBF1 RXRB GATA5 MEF2D ZIC1 NR4A3 PPARA ZFAT AKNA FOSB GSX1 FOXK2 ALX1 TXK MSX1 DLX2 POU4F1 MSGN1 TFAP2A GLIS1 POU1F1 ATF2 NFE2L1 MEIS1 MYOCD MTF1 MEOX1 ETV1 ESRRG FOXF1 PITX1 RUNX1 CEBPE DBP CEBPD SIX4 ATF4 TARDBP MIXL1 CDX4 NFKB2 PITX2 GATA3 SMAD4 DMRT1 IRF4 BARHL2 FOXJ1 YBX1 TRPS1 FOXO3 MYBL2 REST HOXD13 PAX6 IRF1 NKX2-8 NHLH1 MEOX2 POU2F2 TP73 TFAP4 SOX18 GMEB1 MESP1 TFAP2E ALX4 HOXA10 EPAS1 ZNF639 HNF4A OVOL2 KLF5 NR1I2 FOXF2 SIX6 TCF12 PLSCR1 MITF MAFG OSR2 ATF5 ATOH1 HCFC1 PHOX2B HOXA7 ATF3 RUNX2 HNF1A EP300 PROP1 TFAM NR6A1 POU4F3 AR FOXL2 BARX1 MAFB GCM1 LMO2 NFIC SOX17 NEUROD6 NRL CSRNP3 NPAS4 ARNT2 EAF2 MYF5 TP53 ETV6 CRX MYBL1 GATA6 ETV2 RAD21 FLI1 ONECUT1 BARHL1 HOXC13 WT1 MYB GLI2 NDN FUBP3 ELF5 ZNF750 CREB3L1 NR3C1 CEBPB TFAP2C ARID3A SOX9 NR4A2 PATZ1 MEIS3 ELF1 SUB1 SIX3 MAFF SCXB PITX3 TBX20 SOHLH1 HMGA1 KLF13 CAMTA2 NR2E1 PAX8 FOXI1 NEUROD2 ZNF292 HNRNPK MZF1 SOX1 MAF FOXH1 FEZF2 SMAD2 RBPJ NUCKS1 NOBOX MYOD1 BHLHA15 POU3F2 NR1I3 EGR1 HLF NKX2-5 FOXD1 CREB3L3 PGR PLAGL1 ZIC3 CDX1 DLX5 PAX5 SIX2 SOX12 TCF21 HSF2 NFIA ETV4 BSX NFE2L2 GRHL3 FIGLA MYF6 SIX1 TLX1 HOXB5 JUN ESR1 BATF GATA1 PBX1 TFAP2B EBF2 JUNB NR1H2 MEF2B CAMTA1 MAFA CREB3 ZBTB7B OTX2 NFAT5 BARX2 ALS2CR8 GLI3 CEBPG POU4F2 STAT5B NFYC RAX ZSCAN21 DDIT3 CREB1 PLAGL2 KLF4 TCF4 HLTF SMAD1 ATF1 BACH1 MEF2C MAFK ELF4 FOXJ2 PKNOX1 GATA2 MYOG RELA TEF ARID3B

GO_PROTEIN_LIPID_COMPLEX_BINDING Interacting selectively and non-covalently with a protein-lipid complex, any macromolecular complex that contains both protein and lipid molecules. LIPC MAPT LDLR THBS1 COLEC12 APOL2 MSR1 CRP APOA1 STAB2 SCARB1 STAB1 LRP8 SORL1 ANKRA2 APOL5 PCSK9 GPIHBP1 APOE CD36 SCARF1 APOA2 CDH13 VLDLR

GO_ALPHA_ACTININ_BINDING Interacting selectively and non-covalently with alpha-actinin, one of a family of proteins that cross-link F-actin as antiparallel homodimers. Alpha-actinin has a molecular mass of 93-103 KDa; at the N-terminus there are two calponin homology domains, at the C-terminus there are two EF-hands. These two domains are connected by the rod domain. This domain is formed by triple-helical spectrin repeats. PALLD MYOT PKD2L1 DAG1 LRRC10 RARA MAGI1 LDB3 CACNA1D KCNA5 CACNA1C NRAP PDLIM2 MYPN ALMS1 TTN PPARG KCNN2 XIRP2 SYNPO2 PKD2

GO_HISTONE_METHYLTRANSFERASE_ACTIVITY Catalysis of the reaction: S-adenosyl-L-methionine + histone = S-adenosyl-L-homocysteine + methyl-histone. Histone methylation generally occurs on either an arginine or lysine residue. SETD1B EHMT2 SETD1A DOT1L SETD7 PRMT5 SETMAR MLL4 MLL SUV39H2 EZH1 DPY30 CXXC1 MECOM FBLL1 RBBP5 PRMT6 FBL SUV420H1 EHMT1 PRDM16 SMYD2 SUZ12 SETD3 DYDC2 MLL5 SETDB1 EED MLL2 SETDB2 WHSC1 PRMT7 SUV39H1 PRDM6 MLL3 PRMT1 PRDM7 ASH1L SETD8 WDR5 SETD2 NSD1 PRMT2 ASH2L PRDM9 PRMT8 DYDC1 EZH2 WDR82 SUV420H2 CARM1 PRDM13 SMYD3 METTL11A PRDM2 MEN1 WHSC1L1 SMYD1 PRMT3

GO_OXIDOREDUCTASE_ACTIVITY_ACTING_ON_THE_ALDEHYDE_OR_OXO_GROUP_OF_DONORS_NAD_OR_NADP_AS_ACCEPTOR Catalysis of an oxidation-reduction (redox) reaction in which an aldehyde or ketone (oxo) group acts as a hydrogen or electron donor and reduces NAD or NADP. ALDH3B2 ALDH3A2 ADH5 ALDH16A1 ALDH7A1 ALDH5A1 ALDH18A1 PDHB ALDH1L2 FAR2 ALDH1A1 ADH4 ALDH2 OGDH AKR1B10 PDHX ALDH6A1 AKR1C3 ALDH1B1 ALDH9A1 PDHA1 ALDH1L1 AKR1C4 ALDH8A1 DLD LOC440905 ALDH3A1 GAPDH DLAT GAPDHS FAR1 ALDH1A2 PDHA2 ALDH4A1 ALDH3B1 ALDH1A3

GO_MONOVALENT_CATION_PROTON_ANTIPORTER_ACTIVITY Catalysis of the transfer of a solute or solutes from one side of a membrane to the other according to the reaction: monovalent cation(out) + H+(in) = monovalent cation(in) + H+(out). SLC9A4 SLC47A1 SLC9A1 SLC9A11 SLC9A6 SLC9A10 SLC9A5 SLC9B2 SLC9A2 SLC9B1 SLC9A9 SLC9A3 SLC9A8 SLC9A7

GO_G_PROTEIN_COUPLED_RECEPTOR_ACTIVITY Combining with an extracellular signal and transmitting the signal across the membrane by activating an associated G-protein; promotes the exchange of GDP for GTP on the alpha subunit of a heterotrimeric G-protein complex. OR6C74 OR2G6 OPN1MW OR5P2 OR1L6 OR1N1 CHRM1 GPR182 TAS2R1 OPRM1 TAS2R14 CHRM5 OR2L3 OR6M1 OR13G1 OR10D4P GPR153 OR56A4 CXCR1 OR13C5 GRPR OR2T10 OR52B2 OR4A15 OR9I1 OR14A2 OR6C2 OR52E2 HTR2C NMUR2 OR14L1P OR7C2 OR2T8 GPER OPRK1 OR2AG1 TAAR5 OR1B1 OR4M1 AGTR2 TAS1R1 OR4F17 OR2T11 SORCS3 OR4C13 GPR3 OR51A4 BDKRB2 GPR183 CCRL2 CD97 OR9G4 OR4K1 RHO OPN1SW MRGPRX2 OR5H14 GPR161 OR7D2 XPR1 OR4D2 P2RY12 CXCR5 GPR112 OR2AJ1 NPY1R OR8U8 OR10S1 GHSR OR2T4 OR6K2 TAS2R50 OR5AS1 OR11H12 UTS2R GPR50 DRD2 PTH1R GPR126 GPR142 HRH4 GPR128 SSTR1 OR2A12 GPR85 AVPR1B CALCR HCRTR2 OR5M9 OR10H3 OR7G2 PTH2R LPAR2 GRIK3 OR13H1 OR51A2 RGR OR5AP2 TAS2R19 OR2J2 GNRHR HTR1D OR5H6 GPR116 GPRC5A FZD9 OR2A1 CCKBR BAI2 OR11H1 GPR87 IGF2R MRGPRX4 OR5AK2 AMHR2 OR2T6 OR4Q2 OPRD1 OR52E6 MRGPRE OR4N2 GPR26 OR5V1 TAS2R13 OR2Y1 OR1F1 OR2W6P LPAR6 S1PR4 OR2T33 ADRA1A OR52Z1 OR52L1 CXCR7 TAS2R31 OR5W2 OR4C6 OR1S2 OR51H1P GPR17 OR52N4 OR6Y1 CCR6 OR52I1 OR11H2 C5AR1 OR51A7 OR6C1 GPR15 MC2R GPR176 LGR6 VN1R4 GPR82 BDKRB1 CCR9 MRGPRX3 OR10H5 OR2L8 OR4D6 SSTR2 OR52R1 EDNRA OR4N4 OR2AK2 OR8H3 OR56A3 OR3A3 OR4F16 OR10A3 DRD1 OR4C16 OR4A4P GPR37 OR2H1 OR13C9 TACR2 OR8U9 OR5AC1 O3FAR1 OR52E8 CXCR3 OR7A2P OR11H4 TAS2R5 GPR139 OR8J1 OR1F12 PTGDR OR51I2 OR10J3 OR2AP1 SMO OR10G3 GPR4 OR52W1 OR8I2 OR14J1 HTR2A OR6C75 OR4K3 FFAR3 OR4K17 OR52K2 DRD5 OR5AR1 GP1BA OR5B2 OR13C3 PROKR2 OR1F2P OR10AG1 OR8B2 OR9A2 MRGPRG OR2G2 GPR27 BRS3 OR8S1 OR10G7 GPR119 GPR1 OR5K4 GABBR2 OR4X1 OR2T2 OR4K15 OPRL1 OR7A10 OR6C76 OR9K2 GPR56 OR4D11 GPR158 OR51Q1 OR9A4 OR2AT4 OR4Q3 OR10A2 OR2L2 OR13F1 MCHR2 LGR5 OR2T7 OR2T29 OR11G2 OR52N1 GPR12 OR2F2 OR13C6P F2RL1 OR8G5 OR2A5 P2RY1 HCAR1 OR13C2 NPFFR2 OR8H1 EMR2 OR4S1 OR52A1 GPR160 OR1A2 OR3A1 OR5P3 CCR2 FZD8 OR1D4 P2RY2 OR10J5 OR1D5 OR6S1 GPR78 OR10G4 CMKLR1 OR10Q1 OR8G3P LPAR1 XCR1 GCGR RXFP4 GPR19 OR4C5 OR10R2 OR51B4 GPR34 OR4F6 PDGFRB TAAR8 VN1R2 OR2M4 OR5B12 OR1E1 OR4C11 OR13A1 OR10T2 GPR151 S1PR2 GPBAR1 OR10H2 TAS2R60 PTAFR OR52E5 OR10Z1 OR9Q1 OR2A2 OR6C3 MRGPRD LANCL1 OR52E4 GPR77 OGFR OR5K3 OR10K2 OR4K5 CCR1 HTR1F OR10D3 OR2V1 OR5K1 GPRC5C TPRA1 P2RY10 LHCGR NPY OR6B2 ADRB3 OR2T12 OR11L1 FRZB TAS1R2 OR2J1 CXCR6 ADIPOR1 TAAR9 GLP1R OR2M5 MTNR1B GPR113 GRM7 OR1A1 GPR125 ADORA2A OR4A47 FZD1 SFRP4 TAS2R8 ADRB2 OR4F3 GRM5 LPAR3 SORCS2 OR6K6 GPR173 OR1C1 OR8A1 QRFPR GPR35 OR52N5 AVPR2 CXCR2 GPR18 OR51F1 HTR1A OR8K1 GPR101 GPR52 GPR141 CELSR1 CNR1 GRM1 OR8J2 GPR174 OR52K1 GPR64 GPR84 GPR31 ADRA2A OR5M1 LPAR5 HTR3D OR10X1 HTR3C OR6F1 GPR110 OR5G3 OR4D1 OR7G3 LPHN1 FPR3 TAS2R38 OR5D13 TAS2R46 CRHR2 TAS2R9 OR14C36 OR1D2 GPR150 PTGER3 SSTR5 OR10G8 GPR44 OR7A5 OR2B3 OR7G1 OR1J1 OR10A4 ADORA3 OR2L13 SSTR4 CRCP GRM8 OR2I1P OR2T27 CNR2 OR11H7 F2RL3 OR52M1 FZD6 HTR3E GHRHR FZD4 GPR179 GPR162 NPFFR1 OR11A1 OR6T1 OR2B2 OR10AD1 OR1G1 DARC OR1J4 OR5I1 OR51E1 LTB4R2 OR1L8 OR2Z1 LGR4 OR13C7P OR2A42 OR6K3 VN1R17P NPY2R NMBR GIPR HTR1E OR5H1 VIPR1 OR10H1 OR52J3 OR52D1 OR13D1 TAS2R20 OR1P1 CXCR4 FFAR2 GPR33 MAS1 GPR146 OR2B6 MCHR1 LTB4R OR6N2 GPR132 PPARG GPR148 OR2T5 OR52A5 OR13J1 GPR25 OR5AC2 HPGD RXFP1 SFRP1 FZD2 CALCRL LPAR4 CX3CR1 P2RY4 OR10C1 OR5T3 GPR171 TAS2R42 OR52B4 GPR156 OR4A16 PDGFRL HCRTR1 OR6B3 SUCNR1 OPN5 OR4E1 OR5D14 GPR135 OXGR1 S1PR1 MAS1L GRM6 HCAR2 GPR63 TAS1R3 OR8D2 OR2W3 INPP5K OR56A5 RXFP3 OR4D10 GNAT2 OR4K2 GRM3 OR5C1 TAS2R3 OR5AL1 OR5B21 OR10G9 OR2A4 OR5M3 DRD4 HTR7 OPN1MW2 ELTD1 OR2L5 OR51M1 OR2C3 MC4R TAS2R7 OR2A14 OR2T35 OR6C68 HCAR3 OR7C1 GPR65 OR8G2 OR6J1 OR5B3 VIPR2 ZACN OPN1LW AGTR1 OR4F29 OR51B2 CELSR2 OR1K1 OR2AE1 HTR2B OR2K2 OR2M7 GPRC5B CHRM2 OR4C15 GALR2 GPR123 ELOVL4 OR8D4 MRGPRX1 OR1M1 EDNRB OR4K13 GPR152 OR13C8 OR5L1 OR10W1 OR2M3 NMUR1 OR12D2 TAS2R10 OR9A1P VN1R3 OR9G9 OR2J3 OR56B1 OR2C1 OR7D4 OR4S2 AGTRAP OR5M11 TAAR1 OR5J2 OR10G6 OR10P1 OR10J6P GPR115 OR6Q1 BAI1 OR56B2P VN1R5 OR8K3 OR2V2 ADORA2B AVPR1A TAS2R40 OR51S1 CCR5 CYSLTR2 OR5A1 OR9Q2 S1PR5 OR51F2 GPR97 OR51I1 OR4F15 OR51G1 OR4L1 OR5H15 CELSR3 CCR8 TACR1 OR4A8P OR2D2 OR6C6 OR52I2 OR5D16 GPR42 NPR1 OR2H2 OR51D1 PTGER2 OR2S2 ADRA2B OR52B6 OR8H2 OR5AN1 GPR68 CYSLTR1 PTGFR HRH3 TAS2R45 FZD7 GPR75 OR4D5 TAS2R4 GPR6 CCR10 HRH2 OR5A2 OR5H2 SSTR3 PTGER4 OR4X2 OR51G2 OR4E2 ADRA2C OR4A5 TAPT1 P2RY6 OR1J2 OR2T3 OR4D9 P2RY11 ADRA1D OR8D1 OR4C12 OR10H4 GPR157 OR4C46 ADORA1 OR2A7 GPR39 GALR1 OR1L3 OR2AG2 TAAR3 SIGMAR1 OPN4 GPRC6A KISS1R PPYR1 OR2W5 OR2F1 NPBWR1 PROKR1 GAL OR5L2 OXER1 P2RY13 GPR88 HTR5A SFRP2 OR2B11 TAS2R30 OR6P1 OR4K14 OR10V1 OR14A16 CCKAR GABBR1 OR4F4 FZD3 HTR1B OR10A5 OR4B1 OR5D18 CCR7 GPR21 OR10A7 OR6N1 SORCS1 GPR55 OR52E1 OR51J1 OR1S1 P2RY14 OPN3 OR2A25 SFRP5 OR5F1 OR6C4 OR11H6 GPR144 OR5M10 OR3A2 CRHR1 TRHR OR51B6 OR10J4 HRH1 PAX8 OR5T1 OR51L1 GPR133 OR52P1P OR51T1 OR5B17 NPY6R OXTR OR8B12 GPR143 GPR32 GPR61 OR5M8 OR51E2 MLNR GRM4 OR4F5 MC1R OR6B1 GLP2R TSHR TM2D1 OR52H1 GPR98 OR1L4 TBXA2R OR6C70 OR7E24 OR10G2 OR2T34 OR5H8P P2RY8 GPR83 FZD5 SPHK2 PTGER1 OR56A1 ZNF219 OR10A6 OR12D3 TAAR2 OR14I1 OR1L1 OR2T1 ADRB1 OR4P4 OR5AU1 FZD10 GPR124 OR8B3 OR51B5 HTR4 TAS2R39 OR5AK3P GPR149 GPR62 GNRHR2 FSHR RAMP1 GPR20 NPSR1 HTR6 EMR1 MRGPRF OR1N2 OR8K5 OR6C65 OR5K2 DRD3 OR7A17 ADRA1B PRLHR CCR3 CCRL1 GPR32P1 SCTR NPBWR2 OR10J1 OR12D1P OR5R1 OR8G1 GPR22 F2R CCBP2 RXFP2 OR10K1 OR9G1 CHRM4 OR6V1 CHRM3 FFAR1 OR8J3 OR51V1 OR13C4 EMR3 S1PR3 OR8U1 NTSR1 OR2W1 OR4C45 GRM2 PTGIR OR8B4 TAS2R43 OR4M2 OR1E2 OR1E3 FPR1 OR4F21 OR52A4 NTSR2 OR8B8 NPR3 GPR114 MC5R LPHN3 GPR37L1 OR2D3 TAAR6 VN1R1 OR1I1 ADCYAP1R1 OR2M2 F2RL2 MC3R OR4C3 ADIPOR2 SPHK1 OR1Q1 OR2B8P GPR111 OR2G3 OR52N2 LPHN2 NLRP6 MTNR1A BAI3 CCR4 NPY5R CASR TACR3 OR52L2P OR6X1 OR10AC1P TAS2R16 TAS2R41 OR56B4 GALR3 OR4N5 OR5T2 APLNR OR6A2 RRH GPRC5D C3AR1 FPR2 GPR45

GO_INORGANIC_CATION_TRANSMEMBRANE_TRANSPORTER_ACTIVITY Enables the transfer of inorganic cations from one side of a membrane to the other. Inorganic cations are atoms or small molecules with a positive charge that do not contain carbon in covalent linkage. SCNN1A SLC4A5 CNGA3 MAGT1 SLC36A2 KCNG3 KCNG4 ATP5L2 ATP2B4 CHRNA10 SLC5A2 ATP6V1H PKD1 CCDC109B NDUFA4L2 SLC23A2 ACCN1 KCNA3 KCNQ4 ATOX1 SLC6A7 SLC36A1 SLC30A4 SLC39A4 KCNK16 ACCN5 ITPR3 SLC9A6 ATP4B TRPV2 CACNA1B SLC39A6 SLC8A3 SLC41A2 SLC6A5 SLC39A5 KCNQ1 TMEM38A SLC47A2 MCOLN3 HCN3 ATP6V0D1 SCN2B HCN2 ATP6V1B1 SLC12A5 ATP5E ATP6V0E1 KCNMB1 ZP3 ORAI2 JPH3 SCNN1B STIM2 FXYD4 KCNJ3 SLC4A7 KCNK17 KCNK9 CACNA1E ATP6V0D2 SLC11A2 KCNC4 TMEM38B COX6A2 UQCRHL MCOLN1 ATP6V1E1 KCND2 GRM7 KCNAB3 TMEM37 UQCR11 SLC9A9 TMEM175 SLC10A1 ATP6V0E2 SCN3B SLC30A6 SCN4A SLC12A4 SLC13A1 SLC47A1 PANX1 CACNA1A RYR3 KCND3 ATP6V0C SLC10A5 ATP5S CACNA1S COX4I2 AQP1 SCN1B SLC13A2 ATP6V0A4 ATP6V0B SLC25A37 SLC1A7 TRPM3 ITPR1 SLC17A4 SLC39A9 NIPA2 SLC6A11 SLC5A4 SLC9B1P1 NDUFA4 ABCC8 ATP1B4 CNGA1 ATP5G2 SLC5A1 SLC20A1 KCNK15 UQCR10 SLC6A4 SCN8A KCNK12 MMGT1 ATP6AP1 SLC10A4 GAS6 SLC9B1 SLC6A6 SLC30A5 KCNAB1 PKD1L3 NNT KCNN4 C15orf48 C9orf7 SLC1A6 KCNH6 KCNT2 CACNA1D GRIN1 UQCRH TF NALCN ATP6V1G3 SLC10A6 ATP5J KCNA2 ATP1A4 COX7B2 MCU NCS1 TRPM4 SLC10A3 ATP4A FXYD2 TTYH1 CCT8L2 RYR1 ATP6V1C1 SCN1A IL1RAPL1 KCNH1 KCNE1 SLC9A3 SLC46A1 ATP2A2 KCNK7 ATP5C1 ACCN4 KCNJ6 SCN4B KCNE4 ATP5O SLC6A18 ATP2C1 SLC6A3 KCNK10 CACNG2 TRPV4 COX6A1 ATP2B2 HPN GRIN3A SCN11A NIPAL2 KCNN1 SLC9A8 TRPC4AP SLC31A1 SCNN1G KCNJ15 KCNH7 COX15 ATP5A1 COX11 COX7C ATP6V1G1 SLC41A1 CACNG6 HCN4 SLC6A19 COX10 SCN3A TRPM8 NIPA1 CATSPER4 SLC30A2 SLC24A5 SLC5A5 COX7A2P2 KCNE3 TRPV6 TPCN2 ATP5B KCND1 KCNB1 CLCA3P KCNJ2 SLC9A10 KCNQ2 CATSPER1 CACNA1C JPH2 PDE2A SLC32A1 SLC39A8 ATP6V0A1 SLC39A10 TRPV5 KCNJ18 KCNH5 UQCRFS1 ZDHHC17 SLC22A18 SLC20A2 KCNA10 SLC9A2 TMC1 CACNA1F ATP2C2 SLC40A1 SLC38A1 KCNK5 TMCO1 KCNQ5 COX7B KCNJ9 GPM6A GRIN2A SLC30A7 ATP2B1 KCNJ12 SLC6A9 SLC39A11 TMC2 ATP7A PKD1L1 SLC25A28 KCNK1 KCNH4 KCNIP2 ORAI1 ZDHHC13 ATP5H MRS2 SLC34A3 ATP5I SLC1A1 SLC39A2 CACNG8 SLC5A6 SLC1A3 ATP1A1 NIPAL1 SLC10A2 ATP6V1A SLC6A15 COX5A COX8A SLC17A2 C1orf31 ATP5G3 SLC30A1 KCNJ11 SLC5A7 ATP1B3 SLC9A7 CACNG1 DENND5B OPRM1 CACNB1 TRPC7 KCNN2 SLC39A7 ATP2B3 NIPAL4 ABCB11 CACNB4 KCNIP4 SLC9A11 COX4I1 SLC39A14 TRPV3 ATP2A1 ATP2A3 CACNA2D1 KCNS3 UQCRC1 KCNG1 UQCRQ CNGB3 TRPC5 RASA3 ATP12A PKDREJ SLC6A20 CHRNA9 KCNK18 CUL5 KCNA4 SLC28A2 KCNAB2 SLC24A4 SLC39A12 SLC6A1 KCNA6 CALHM1 COX6B1 SLC9A4 SLC30A3 ATP6V1G2 KCNV2 LOXHD1 SLC34A2 CACNG3 SLC17A7 SLC30A10 KCNA7 RYR2 TPCN1 KCNJ10 COX8C SLC24A3 PKD1L2 ATP6V1B2 SLC8A2 SLC28A3 TRPM5 SLC23A1 KCNA1 KCNJ8 SLC6A17 KCNU1 KCNJ1 SLC17A1 UQCRFS1P1 KCNIP3 KCNK2 ATP6V1E2 ABCC9 KCNS1 UQCRB CACNG7 TRAPPC10 KCNMB3 KCNV1 CACNB3 KCNJ14 SLC39A3 TFRC ATP5F1 CNGA2 KCNMA1 KCNK6 SCN7A TRPC4 SLC4A11 DENND5A SLC9A1 REST ACCN2 SLC12A6 ATP5L SLC9B2 CNGB1 CACNG4 KCNT1 TRPC1 SHROOM2 CACNA1G KCNS2 KCNH8 SLC24A1 KCNC3 SCNN1D SLC13A4 SLC11A1 TRPV1 FKBP1B COX5B CATSPER3 SLC24A2 SLC24A6 KCNG2 SCN5A CACNA1H CNNM2 SLC3A2 SLC8A1 TRPA1 ATP1B1 ATP5G1 CACNA2D2 SLC12A9 TCIRG1 SLC5A3 ACCN3 KCNIP1 CACNA2D3 SLC39A1 PKD2L2 KCNN3 SLC31A2 COX7A2L CACNA1I TUSC3 HCN1 ATP1A2 KCNK13 KCNC1 ATP1B2 KCNH3 ORAI3 TRPC6 KCNA5 TRPM7 KCNC2 ATP7B SLC6A2 SLC30A8 ATP5EP2 SLC6A8 SLC6A14 PKD2 SLC5A9 SURF1 KCNF1 KCNMB4 COX6B2 CLDN16 KCNMB2 KCNE1L PKD2L1 MTMR6 ATP5D CYB5A KCNJ4 ITPR2 KCNK3 ITGAV SLC4A4 SLC13A5 SLC1A2 COX7A2 KCNE2 KCNJ16 SLC4A9 CACNG5 KCNH2 SLC28A1 KCNJ5 SCN2A SLC39A13 NIPAL3 ATP6V1C2 ATP6AP1L TRPC3 SLC12A7 TRPM1 ATP6V1F KCNJ13 CNGA4 SLC34A1 KCNQ3 KCNK4 SLC13A3 GRIN3B COX7A1 PSEN1 CNNM4 ATP13A1 CATSPER2 TRPM6 SLC6A13 SLC17A3 TRPM2 KCNB2 SLC9A5 MCOLN2 SLC6A12 ATP6V0A2 SCN9A COX6C SLC6A16 CACNB2 SCN10A SLC36A3 CACNA2D4 TMCO3 ATP1A3

GO_OXYGEN_TRANSPORTER_ACTIVITY Enables the directed movement of oxygen into, out of or within a cell, or between cells. HBA1 HBA2 HBM HBQ1 CYGB HBE1 IPCEF1 HBG2 MB NGB HBZ HBG1 HBD HBB

GO_SNRNA_BINDING Interacting selectively and non-covalently with a small nuclear RNA (snRNA). DDX39B SNRPB2 LSM10 PRPF4 LSM4 GEMIN5 SNRPC SNRPB HEXIM2 RBM41 SNRPD3 RBM22 SNRPA PRPF8 CCNT2 NAA38 LSM11 TROVE2 RNPC3 RBPMS DDX21 PRPF31 LSM7 CCNT1 SNRPA1 EIF5A SART3 LSM2 SNRNP70 HEXIM1 NHP2L1 NCBP2 SNRNP35 CDK9 ISG20

GO_LIPID_TRANSPORTER_ACTIVITY Enables the directed movement of lipids into, out of or within a cell, or between cells. PLSCR5 SLC10A4 APOM SLC27A2 PLSCR1 APOA2 ABCA7 STARD4 SFTPA1 SLMO2 CEACAM1 SLC22A9 SLCO1B1 RBP4 ABCD2 CFHR4 PLSCR4 ABCA3 SLC27A1 COL4A3BP ABCC3 ATP8B3 SLC27A6 ATP9B SPNS2 SLC27A5 ANO3 ANO7 PLEKHA8 AKR1C4 MFSD2A ATP8A2 GLTPD2 SLC10A5 ABCA4 ATP11A OSBPL5 ANO9 SLC10A1 APOA1 ANO6 SLCO2B1 ANO4 PLEKHA8P1 ATP11C OSBPL8 APOA5 RFT1 GLTPD1 FABP1 SPNS1 PITPNC1 CETP ATP8B2 ABCG4 ABCG5 PITPNM3 SLCO1B3 ABCG8 STAR ABCB11 ARV1 TRIAP1 SLC27A4 PLSCR3 SLCO1A2 OSBP NPC1 FABP3 ATP10A APOF PRELID2 ABCD1 SLCO1C1 APOL3 SLC10A2 ABCB4 APOB SLCO2A1 ABCG1 SLC10A3 GM2A PRELID1 TNFAIP8L3 ATP10D APOD ATP8A1 APOE SLC10A6 APOC4 ATP9A STARD5 GLTP MTTP PITPNM1 ABCA1 PITPNA PLSCR2 SLMO1 ATP10B ATP8B4 APOA4 ATP11B ABCD3 SCP2 ABCB1 ATP8B1 ABCA12 SPNS3 C20orf79

GO_MICROFILAMENT_MOTOR_ACTIVITY Catalysis of movement along a microfilament, coupled to the hydrolysis of a nucleoside triphosphate (usually ATP). MYO10 MYO19 MYH7 MYH2 MYH14 MYO5B MYH13 MYO1D MYO6 MYH6 MYO5A MYO1B MYO7A MYH10 MYH8 MYO3A MYO1E MYH3 MYH4 MYO1C MYO9B MYH9

GO_FIBROBLAST_GROWTH_FACTOR_RECEPTOR_BINDING Interacting selectively and non-covalently with the fibroblast growth factor receptor (FGFR). FGF19 FGF8 FGF22 FGF9 FGF4 FGF23 FLRT2 FLRT1 FGF21 FLRT3 FGF18 FGF16 FGF20 KLB NPTN FGF1 FGF10 FGF3 KL FGF7 FGF17 FRS3 FGF6 FRS2 FGF2 FGF5

GO_GLYCEROL_TRANSMEMBRANE_TRANSPORTER_ACTIVITY Enables the transfer of glycerol from one side of the membrane to the other. Glycerol is 1,2,3-propanetriol, a sweet, hygroscopic, viscous liquid, widely distributed in nature as a constituent of many lipids. MIP AQP5 AQP7 AQP1 AQP4 AQP9 AQP10 AQP6 AQP3 AQP8 AQP2 AQP7P3

GO_MYOSIN_HEAVY_CHAIN_BINDING Interacting selectively and non-covalently with a heavy chain of a myosin complex. MYL12B AMPD1 STX1A MYL3 CORO1A USH1C PDLIM2 MYL9 MYBPC3 MYL2 SPTBN5 AXL MYL4

GO_LIPOPOLYSACCHARIDE_BINDING Interacting selectively and non-covalently with lipopolysaccharide. PSMB4 HMGB1 SELP TLR4 PTAFR DEFB114 BPIFC P2RX7 CD14 SCARB1 TRIL HSPD1 BPI DROSHA CD6 TLR2 RNASE7 BPIFA2 LBP SPON2 TREM2

GO_NEUROPEPTIDE_HORMONE_ACTIVITY The action characteristic of a neuropeptide hormone, any peptide hormone that acts in the central nervous system. A neuropeptide is any of several types of molecules found in brain tissue, composed of short chains of amino acids; they include endorphins, enkephalins, vasopressin, and others. They are often localized in axon terminals at synapses and are classified as putative neurotransmitters, although some are also hormones. NPY NTS POMC VIP PYY ADCYAP1 CARTPT PYY2 AVP OXT CCK PNOC CRH GAL GRP CALCB PRLH AGRP QRFP HCRT PENK CORT PPY PYY3 NPPA PDYN NPFF VGF UCN TRH C12orf39

GO_PEPTIDE_N_ACETYLTRANSFERASE_ACTIVITY Catalysis of the acetylation of an amino acid residue of a peptide or protein, according to the reaction: acetyl-CoA + peptide = CoA + N-acetylpeptide. SRCAP EDF1 KAT5 TAF9B MCRS1 CLOCK ELP3 ATF2 HAT1 KIAA1267 BAZ1A CDYL SAP130 TAF10 CREBBP EP300 SUPT3H EPC1 KAT8 KIAA1310 GTF3C4 CSRP2BP NAA60 NCOA3 NAA30 HCFC1 TAF9 CDY2B TAF1 CDY2A TAF1L NAA20 NAA10 C7orf52 TAF5L C12orf41 OGT MED24 ING3 ATAT1 NCOA2 USP22 TADA3 NAA50 TAF6L SUPT7L WDR5 ELP4 C2orf67 NAA15 PHF20 KAT7 NAA11 TADA2B TADA2A KAT6B KAT2A EPC2 ARRB1 TADA1 NCOA1 BRCA2 NAA16 NAA25 CDY1 CDY1B KAT2B TAF5 KAT6A

GO_HEPARAN_SULFATE_PROTEOGLYCAN_BINDING Interacting selectively and non-covalently with a heparan sulfate proteoglycan, any proteoglycan containing heparan sulfate as the glycosaminoglycan carbohydrate unit. GPC4 HRG SEMA5A GPC2 PLA2G2D ATP1A3 GPC1 GPC6 COMP FGF20 HPSE2 AZU1 GPC5 AGRN HPSE FST CFH GPC3

GO_VOLTAGE_GATED_ION_CHANNEL_ACTIVITY_INVOLVED_IN_REGULATION_OF_POSTSYNAPTIC_MEMBRANE_POTENTIAL Any voltage-gated ion channel activity that is involved in regulation of postsynaptic membrane potential. PKD2 SCN2A SCN10A SCN5A SCN4B SCN1B SCN8A SCN9A SCN4A SCN3B SCN11A HCN2 SCN2B HCN1 HCN3 CATSPER4 HCN4 SCN7A SCN1A SCN3A

GO_TYPE_I_INTERFERON_RECEPTOR_BINDING Interacting selectively and non-covalently with an interferon-type I receptor, a heterodimeric complex composed of an alpha subunit (IFNAR1) and a beta subunit (IFNAR2). IFNW1 IFNA8 IFNA21 IFNA7 IFNA10 IFNA13 IFNK IFNA17 IFNA1 IFNA4 IFNA6 IFNA5 IFNA14 IFNE IFNA16 IFNA2 IFNB1

GO_OXIDOREDUCTASE_ACTIVITY_ACTING_ON_PAIRED_DONORS_WITH_INCORPORATION_OR_REDUCTION_OF_MOLECULAR_OXYGEN_NAD_P_H_AS_ONE_DONOR_AND_INCORPORATION_OF_ONE_ATOM_OF_OXYGEN Catalysis of an oxidation-reduction (redox) reaction in which hydrogen or electrons are transferred from NADH or NADPH and one other donor, and one atom of oxygen is incorporated into one donor. FMO5 MICAL2 CYP4F12 CYP26A1 CYP11B2 CYP2C19 KMO CYP46A1 CYP1A1 CYP27B1 NOS3 COQ7 CYP26B1 NOS1 CYP4F11 COQ6 CYP4A11 CH25H CYP11B1 CYP51A1 CYP2C9 FMO1 FMO2 MICAL3 SQLE FMO3 NOS2 C5orf4 CYP11A1 CYP3A4 CYP8B1 CYP2E1 CYP7B1 FMO4 MICAL1 AKR1C1 CYP7A1 AKR1C2 CYP4F2 CYP4F3 AKR1C3 MSMO1

GO_G_PROTEIN_BETA_GAMMA_SUBUNIT_COMPLEX_BINDING Interacting selectively and non-covalently with a complex of G-protein beta/gamma subunits. GNAZ GNA11 GNA15 GNAL CETN2 GNAQ PIK3R5 GNA13 GNAS GNAO1 GNAI1 GNAT2 GNA12 GNAT3 CETN1 GNAI3 GNAI2 GNA14 ADORA1 GNAT1

GO_TRANSCRIPTIONAL_REPRESSOR_ACTIVITY_RNA_POLYMERASE_II_TRANSCRIPTION_FACTOR_BINDING Interacting selectively and non-covalently with an RNA polymerase II transcription factor, which may be a single protein or a complex, in order to stop, prevent, or reduce the frequency, rate or extent of transcription from an RNA polymerase II promoter. A protein binding transcription factor may or may not also interact with the template nucleic acid (either DNA or RNA) as well. PRRX1 HDGF POU3F1 AHRR SOX4 SMARCA2 HYAL2 MYOCD NCOA1 N4BP2L2 MED6 MIXL1 MINA TLE1 SPEN NOTCH1 NR1H4 GBX2 OLIG3 PITX2 MED12L T PITX1 EOMES CREB1 MED12 HEYL KLF4 TBX20 TCERG1 PHF12 ZMYND8 CITED1 C19orf2 BHLHE41 SIN3A RBBP8 WBP2 SOX11 SOX10 CTBP1 MEF2A HIPK2 TRIM28 RERE FOXH1 SMARCA4 SOX3 NKX2-5 BEND6 NEUROD1 POU3F2 CITED4 PHF17 MKL1 SOX12 TCP10L TBX18 PPARA WWOX LMO2 HDAC1 WWP2 BHLHE40 ANKRD1 CITED2 CREBBP UXT ZFPM2 TBX15 CLOCK MKL2 POU1F1 ISL1 ELANE JUN TFAP2B NFE2L1 ELP2 SIN3B ZNF451 TBX6

GO_CALCIUM_DEPENDENT_CYSTEINE_TYPE_ENDOPEPTIDASE_ACTIVITY Catalysis of the hydrolysis of nonterminal peptide bonds in a polypeptide chain by a mechanism using a cysteine residue at the enzyme active center, and requiring the presence of calcium. GCA CAPN11 CAPN3 CAPNS2 SRI CAPN13 PDCD6 CAPN5 C6orf103 CAPNS1 CAPN9 CAPN2 CAPN1 PEF1 CAPN10 CAPN7 CAPN6 CAPN14 CAPN12 CAPN8 SOLH

GO_CCR_CHEMOKINE_RECEPTOR_BINDING Interacting selectively and non-covalently with a CCR chemokine receptor. CCL2 CX3CL1 DEFB4A CCL20 CCL19 CCL14 CCL15 DEFB1 CCL13 CCL3L1 CCL25 CCL23 CCL11 STAT1 CCRL2 CNIH4 CCL22 CCL24 CCL4L2 CCL21 CXCL13 CCL26 CCL5 XCL2 CCL3 STAT3 CCL8 CCL17 CCL3L3 CCL7 XCL1 CCL4 JAK1 CCR2 CCL1 CCL18 CCL16

GO_GAP_JUNCTION_CHANNEL_ACTIVITY A wide pore channel activity that enables a direct cytoplasmic connection from one cell to an adjacent cell. The gap junction can pass large solutes as well as electrical signals between cells. Gap junctions consist of two gap junction hemi-channels, or connexons, one contributed by each membrane through which the gap junction passes. GJC1 GJA8 PANX2 MIP GJA5 GJB2 PANX1 GJA3 PANX3 GJD3 GJD2 GJA10 GJB3 GJA1 GJB1 GJC2

GO_INORGANIC_ANION_EXCHANGER_ACTIVITY Catalysis of the transfer of a solute or solutes from one side of a membrane to the other according to the reaction: inorganic anion A(out) + inorganic anion B(in) = inorganic anion A(in) + inorganic anion B(out). SLC22A24 SLC22A11 SLC4A11 SLC26A6 SLC4A2 SLC4A10 SLC22A12 SLC4A5 SLC4A4 SLC22A20 SLC26A3 SLC22A10 SLC22A8 SLC4A9 SLC22A25 SLC22A9 SLC22A6 SLC4A8 SLC4A3 SLC4A7 SLC4A1

GO_PHOSPHOPROTEIN_PHOSPHATASE_ACTIVITY Catalysis of the reaction: a phosphoprotein + H2O = a protein + phosphate. Together with protein kinases, these enzymes control the state of phosphorylation of cell proteins and thereby provide an important mechanism for regulating cellular activity. PPP3CB PPP2CB MTMR14 PTPRS PPM1B CTDSPL DUSP4 PTPN7 PPM1E MTM1 DUSP16 CDC14C DUSP13 DUSP11 KIAA1274 PPAP2B EYA1 PGAM5 CTDSP2 PTPN20B PTPRH DLGAP5 PHPT1 PPP3R1 DUSP8 PTPRK DNAJC6 MTMR4 CDC25A PTPN5 MDP1 MYH8 PTPRJ PPP1R3D STYX PPEF2 MTMR3 PTPRN PPM1D DUSP15 PPM1G SBF1 PTPN18 PPM1A PTPRR DUSP26 PTPRF PPP6C PPM1J PPP2R2D MTMR6 PTPN22 SSH2 PDXP PPM1F PTPRM UBLCP1 PPP2R2B CYCS PTPRU LCK PTPRA MYH6 PTPRZ1 PTPRO PTPRE CTDSP1 PPP1CC DUSP28 PTPN9 PTP4A2 DUSP10 DUSP5 PTPRB CDC14A MTMR2 PDP2 PTPMT1 PPM1H PTPN1 MYH3 PPP2R1A ILKAP MTMR7 DUSP22 EYA4 PTP4A3 SSU72 PTPRT DUSP19 PPAP2C PPP1CB DUPD1 SSH1 DUSP21 PTPN6 PTPN14 DUSP27 PTPRD DUSP3 PTPN21 PPP3CC PPM1L PHLPP1 MTMR1 PTEN ACP1 PPP2R3B TENC1 PPP5C PTPN2 DUSP6 DUSP7 PP2D1 PPP1R3C RPAP2 PTPN12 PPP3CA CDC25C TIMM50 CTDNEP1 EYA2 DUSP2 DUSP18 DUSP23 PTPDC1 PTPN13 PTPRC PDP1 PPA2 PTPLB MTMR8 PTPRN2 CAMK2G TPTE2 PPP1R15B PPP1CA PPP1R3B UBASH3B EYA3 PPP2CA DUSP12 PPP4C DLG1 PTPN4 CDC14B PPM1N PTPRG CDC25B CTDSPL2 STYXL1 SSH3 CDKN3 PTPN3 PPP4R1 PGP DUSP9 PTPN23 PPP2R5D CTDP1 PPM1K TAB1 MAP2K1 PTPRQ PPP2R2A TPTE PHLPP2 CPPED1 PPTC7 DUSP14 PPEF1 PPM1M PTP4A1 PPP2R2C PTPN11 RNGTT DUSP1

GO_PATTERN_RECOGNITION_RECEPTOR_ACTIVITY Combining with a pathogen-associated molecular pattern (PAMP), a structure conserved among microbial species, or damage-associated molecular pattern (DAMP), an endogenous molecule released from damaged cells), to initiate a change in cell activity. FCN1 CD36 TLR2 DMBT1 LY96 PGLYRP2 TRIM5 PGLYRP3 CD14 CLEC7A SCARB1 PGLYRP1 COLEC12 PGLYRP4 PTAFR TLR4 MARCO

GO_RETINAL_BINDING Interacting selectively and non-covalently with retinal, one of the forms of vitamin A. Retinal plays an important role in the visual process in most vertebrates, combining with opsins to form visual pigments in the retina. RBP3 CRABP2 RBP1 ALDH1A2 RBP7 RLBP1 CRABP1 OPN4 ADH4 RBP5 RBP2 OPN5 RBP4

GO_RNA_POLYMERASE_ACTIVITY Catalysis of the reaction: nucleoside triphosphate + RNA(n) = diphosphate + RNA(n+1); the synthesis of RNA from ribonucleotide triphosphates in the presence of a nucleic acid template. POLR2H POLR3H POLR1C POLRMT MED21 POLR3F POLR3B POLR2J3 POLR2A RPAP1 TRNT1 POLR2B POLR2M POLR2J2 POLR2D ZNRD1 POLR3E POLR3C POLR2K POLR3G POLA1 POLR2I MED20 POLR2C POLR1E PRIM1 CCDC111 POLR2L TWISTNB POLR1B POLR3A POLR1D POLR2J CD3EAP POLR1A POLR2G POLR2E POLR3K POLR3GL CRCP POLR2F PRIM2 POLR3D TERT

GO_PROTEIN_SERINE_THREONINE_KINASE_ACTIVATOR_ACTIVITY Binds to and increases the activity of a protein serine/threonine kinase. TGFB1 CKS2 MAP2K1 CALM2 CKS1B PARP16 STK4 LTF SPRY2 CALM1 STRADA CDK5R2 FAM20A MAP2K2 STK3 IQGAP1 CALM3 CDK5R1 IGF2 CAB39 ALS2

GO_EXTRACELLULAR_LIGAND_GATED_ION_CHANNEL_ACTIVITY Enables the transmembrane transfer of an ion by a channel that opens when a specific extracellular ligand has been bound by the channel complex or one of its constituent parts. CHRNB3 GABRA3 CHRND GABRB1 GABRG1 GRIK5 GABRA4 GABRA5 GRIK3 P2RX1 P2RX4 GABRB2 GLRA1 HTR3E GABRQ GABRA1 GABRD GRIN3B ZACN CHRNE GABRB3 GABRG2 GABRP CHRFAM7A GRIN3A GLRA2 GRIN2B GRID1 CHRNA5 GRIK2 P2RX5 HTR3B SLC17A7 GRIK1 CHRNB4 GRIA3 GRIN2D CHRNB2 CHRNA9 GABRR1 GABRE CHRNA4 GRIA2 P2RX3 GABRR3 HTR3D CHRNA2 CHRNA3 CHRNA7 GRID2 HTR3A CHRNA1 HTR3C GRIA1 P2RX2 GLRA4 GABRG3 CHRNA6 P2RX6 GRIN2C TRPV1 GABRA6 GRIK4 CHRNG CHRNB1 GLRA3 GABRR2 PTK2B GABRA2 GLRB GRIN2A CHRNA10 GRIA4 P2RX7 GRIN1

GO_PHOSPHATASE_ACTIVATOR_ACTIVITY Increases the activity of a phosphatase, an enzyme which catalyzes of the removal of a phosphate group from a substrate molecule. PPP2R4 CALM3 IGFBP3 B3GAT3 CALM1 GTF2F1 PPP1R15A BMP2 FRS2 CALM2 PHACTR4

GO_COMPLEMENT_BINDING Interacting selectively and non-covalently with any component or product of the complement cascade. PHB CRP C4A CR2 C5AR1 APCS C8G CD93 MASP2 C8A CALR PTX3 CFB CR1 CD59 C4B C1QBP

GO_ORGANIC_ANION_TRANSMEMBRANE_TRANSPORTER_ACTIVITY Enables the transfer of organic anions from one side of a membrane to the other. Organic anions are atoms or small molecules with a negative charge which contain carbon in covalent linkage. SLC5A12 SLC22A17 SLC26A5 SLC4A4 SLC26A10 SLCO2B1 BEST1 SLC26A8 ABCC5 ABCC2 SLC17A7 SLC4A1 SLC16A3 SLC1A7 SLC26A7 SLC13A5 SLC1A2 SLC26A11 SLCO4A1 SLC4A9 SLC25A22 SLC26A9 SLC23A1 SLC16A13 SLC26A2 SLC17A3 SLC22A20 ABCC3 SLC16A11 SLC16A12 SLC13A3 SLC22A24 SLC16A4 SLC25A1 SFXN5 SLC25A11 ABCC11 SLC25A13 SLC22A9 SLCO1B1 SLC22A25 SLC22A8 SLC1A6 SLC26A3 SLC17A8 SLC4A5 SLC4A11 SLC26A6 BRP44L SLC16A8 SLC17A6 SLC16A6 MFSD10 BRP44 SLC1A1 SLC10A6 SLC23A2 SLC25A21 SLC22A6 SLCO6A1 SLC7A11 SLC26A1 SLC22A10 SLCO1A2 SLC26A4 SLCO3A1 SLC22A31 SLC22A7 SLC22A12 SLC1A3 SLC16A5 SLCO1C1 SLC22A11 SLCO2A1 SLC4A7 SLCO5A1 SLC38A7 SLC16A1 SLC25A12 SLC22A23 CFTR SLCO4C1 SLC16A7 SLCO1B3

GO_UNFOLDED_PROTEIN_BINDING Interacting selectively and non-covalently with an unfolded protein. HSP90AB4P HTRA2 SRSF12 CCT3 CDC37 SCAP HSPD1 TUBB4B DNAJB8 HSPA1A SRSF10 AAMP NUDCD2 HSPA5 PFDN5 CRYAA NUDCD3 AFG3L2 HSPA9 HSP90AA4P HSP90AB3P SCG5 CDC37L1 DNAJA3 HSP90AB2P PFDN6 UGGT1 PTGES3 TRAP1 CLPX DNAJA1 CCT6A HSP90B2P CCT4 DNAJB13 GRPEL2 HSPA1B SIL1 DNAJB5 DNAJA2 NDUFAF1 GRPEL1 TAPBP DNAJB11 PFDN1 CALR HSPA1L ST13 HSPA8 RUVBL2 LRPAP1 AIP DNAJB4 TMEM67 MDN1 CCT6B ERO1LB CLN3 CALR3 HSP90AA1 PDRG1 CHAF1A CCT5 TCP1 ERLEC1 DNAJB2 CLGN MKKS CRYAB AHSP NAP1L4 AIPL1 PPIA RP2 SYVN1 HSP90AB1 SPG7 SERPINH1 NUDC PFDN4 NPM1 TTC1 PFDN2 HSPA6 HSP90AA2 HSP90B1 HSPE1 DNAJA4 LMAN1 DNAJB1 DNAJC4 UGGT2 CCT2 TOMM20 DNAJB6 CANX TOR1A HSPA2 CHAF1B PPIB GRXCR2 CCT7 APCS HSP90AA5P CCT8

GO_POLY_A_RNA_BINDING Interacting non-covalently with a poly(A) RNA, a RNA molecule which has a tail of adenine bases. DUSP14 TRIM56 EIF1AX APOBEC3F MECP2 DDX55 TRIM28 PNN SRSF10 WDR43 LSM14A UBE2N ADK PARP12 EIF5B YARS2 GTPBP4 NQO1 CCDC124 HIST1H1E R3HDM2 PURG RPS10 SON C9orf23 RPS27A NHP2L1 TFAM TRIM25 HNRNPH1 RRP8 NOL6 C17orf85 DHX32 DHX35 DNTTIP2 C17orf42 DYNC1LI1 FLNB NSRP1 S100A4 BICC1 NUFIP2 FASTKD5 DZIP3 PTBP2 ZYX PDIA3 NSUN2 NOA1 DHX36 CDC40 CDK13 SRRT FAM133B RPL32 IMP3 ANXA2 PCSK9 C1QBP MTDH RPS3 LSM3 RPL10A WIBG RPS11 KCTD12 HIST1H1B DHX37 ADD1 GLTSCR2 ZC3H4 RBMS2 SNRPD2 PPRC1 MKRN2 GNL3L RBM28 RPN1 CPSF6 VSIG8 RPS15 SCAF11 MBNL1 TSNAX MSI1 NOVA1 DOM3Z RPL15 POLR2A GANAB RPUSD3 SUMO2 RPL13 EIF4E DUT GRSF1 DHX9 TPT1 DCN FMR1 PPAN EIF3A METAP2 EIF3H UBAP2L BYSL MRPL45 DHFR SNRNP70 MLL3 TDRD3 DHFRP1 PRRC2C RPL13A NSUN5 HNRNPCL1 CD3EAP FBRSL1 MRPL28 TRUB2 DAP3 NOL7 ZFC3H1 HSD17B10 PATL1 SART1 MRPS26 WBSCR16 MRPS5 ATP5C1 MRPL15 PA2G4 RPL21 GPATCH8 FAM32A CKAP4 RARS2 RPS14 RNPS1 NAP1L1 SRSF2 MYEF2 BCCIP MARK2 CCDC108 CISD2 MAGOHB EIF1B HEATR6 RPL26 SERPINH1 RDX TYMS HIST1H4K C2orf15 RPL12 OTUD4 BMS1 KRT18 RPS21 XRN2 MAK16 KRR1 H1FX CEBPZ CCT3 MRPL22 GFM1 SRRM2 ZNF326 HIST1H1C DDX39B NCBP1 HNRPLL SPATS2L STRBP TBRG4 YTHDC2 CHCHD1 CPSF4 C1orf35 RBM27 ATXN2L PCBP3 DIEXF ETF1 FASTKD2 NXF2 PAPD5 MOV10 LARP1B EIF2AK2 SSB EIF2S2 AKAP8 DDX23 NFX1 TFB1M PHF6 C15orf52 TOP3B TSN IGF2BP2 MBNL3 UBE2I STAU1 MRPL14 HSPA8 RBM47 DDX41 SMG1 MRPL4 RG9MTD2 RBFOX3 SARNP HNRNPA2B1 CS PNISR EMG1 PTCD3 MRPL41 C16orf88 HIST4H4 NSA2 PCDH20 DUS3L YTHDC1 SRSF9 AKAP1 DHX16 ZCCHC17 OASL MYBBP1A RBM6 CSTF1 FSCN1 HNRNPUL1 SUGP2 NOM1 DDX27 LSM1 GLRX3 PRPF6 KIAA0020 IPO5 SRSF7 ZNF579 PRPF8 FYTTD1 REXO4 DDX51 ZRANB2 CRNKL1 CTNNA1 ZC3HAV1 PPP1R10 ERAL1 USP36 FBL DDX18 NOL8 SRP72 EIF4G2 CCT6A NKRF DDX49 RTN4 NOC4L EXOSC10 EIF5A RPLP0 TNRC6B TUFM HERC5 ADARB2 SLC16A3 BST2 VCP PRPF3 SBDS WBSCR22 RBM12 TOP2A BOLL GOLGB1 ARF1 RPS5 H1F0 LSM4 XIRP1 UBA1 SRP19 WDR6 SLC25A5 PARN DHX15 TMSB4X POU5F1 DUSP11 ZFP106 PEG10 PPIB AKAP17A ENO1 ZNF346 UTP18 EIF3E G3BP2 RPL4 RBM25 RPF1 PNO1 CCAR1 ABT1 LARP4B EIF4ENIF1 RALYL NTPCR MFAP1 METTL14 MRPS21 RPS25 PSMC1 NGDN RBM10 EPPK1 RPL27A SECISBP2L CCBL2 EIF4G1 GCN1L1 RPS19BP1 EIF4E2 ERH RPS19 HSPB1 MANF FNDC3A RPS6 RPL19 FAM120C GTPBP1 BTF3 TSFM RNF20 DDX24 C1orf131 CCRN4L CCDC86 MRPS28 MRPL20 ALDOA C19orf29 CLK3 SRRM1 RBM4 PTCD1 RBPMS2 PKM2 SRSF6 KRI1 FNDC3B HMGB1 RPL36A NDUFV3 ZC3H18 LUC7L3 ALG13 RBFOX1 SRPR SPTBN1 MYO18A ANP32A XPO5 CELF4 HIST1H4D CELF2 NKAP KPNA2 RBBP6 SF3A3 PIWIL2 ZC3H15 NUCKS1 SNRPB DDX46 SNRPD3 SNRNP40 PSPC1 EBNA1BP2 NOSIP NUDT16 SRSF12 DDX54 MRPS15 MRPL11 HMGN5 APEX1 BAG4 SEC23IP UTP11L HIST1H4J SAMSN1 SF3A2 JUN NUPL2 CSDE1 DMGDH PAIP2 DARS SSRP1 EIF4G3 RBFOX2 C4BPA RBM14 FTSJ3 UTP15 GNL2 SEC61B PRR3 YTHDF3 RPSA MAGOH DHX33 APOBEC3C RPS12 DCP1B DHX8 RPL31 DBR1 EIF2C2 CANX FXR2 S100A16 MYH9 EIF4A1 QKI NHP2 DDX47 RPS26 SMC1A HNRNPM UBC PAN3 PPIA SLTM SPATS2 MBNL2 DDX3X MRPL27 HSPA9 ALDH6A1 SNRPD1 SERBP1 C11orf68 EIF2S1 EIF4A3 RPS27L MRPL44 GIGYF2 RIMS1 MTO1 ZCCHC9 C16orf80 KHDRBS3 YARS ATXN2 PTCD2 PTRH1 PEF1 C14orf166 ZFP36L2 ISY1 KIF1C SYF2 CPEB1 PRPF40A ZNF598 PLEC UNK BCLAF1 TRMT1L SND1 SREK1 SRP54 C1orf52 PES1 GTF2F1 YTHDF1 R3HDM1 MTIF2 RRBP1 THRAP3 CNBP MRPL40 ZNF638 TBCA GRN CHERP ZFP36L1 FAM98A RBM5 HUWE1 CCDC165 RPS2 STIP1 MACF1 TUT1 USO1 MRPS12 HNRNPU GNL3 RRP1 TCERG1 MRPL13 RBMS1 DDX50 LSM2 THOC2 RPS4X RAVER1 GDI2 MEPCE ANGEL2 HMGB2 COL14A1 NVL PFN1 PTPN1 CLTC RPL36 KIAA0664 WBP11 MATR3 LGALS3 PURB DHFRL1 SLC25A11 TRA2B FASN DHX57 RPP25 SUMO1 DAZ1 CHTOP ZC3H7B NOP14 ZCCHC11 CDC5L DHX29 SSBP1 PNPT1 RNMTL1 RPL23A ESF1 PCBP4 CPSF1 PARP1 SUPT6H ASCC3 ESRP2 RBMX2 RPGR API5 HRSP12 CNOT1 CDC42EP4 REPIN1 XRCC6 CASC3 PRIC285 SRSF8 CAPRIN1 PSMD4 LAS1L RPS15A RC3H2 GEMIN5 BZW1 SEC63 GRWD1 EEF2 YBX1 IGF2BP3 WDR75 LRRC59 PRPF4B FDPS AKAP8L LGALS1 RPS27 SART3 PCBP2 SAMD4A DHX40 DCD MRPL3 EXOSC9 PEBP1 FIP1L1 CPEB2 TPR XRCC5 NOL12 MEX3B DAZAP1 ZFP36 TNRC6A DDX5 THOC4 RPS7 HELZ HSPD1 MRTO4 FLNA TRA2A MKI67 SF3B14 NOL10 NPM3 LRPPRC PPHLN1 MEX3A RPL23 PHF5A CAST RBM42 SLIRP HNRNPA3 KIAA1324 RCC2 EIF4A2 UBAP2 PRRC2A RBPMS LLPH HNRNPL RPL27 RPL11 ACIN1 MKI67IP SUGP1 MTERF SNIP1 RBM22 WDR3 ADAR MCAT HIST1H4L NAA15 FUBP3 GAR1 CSTB SAFB LARP1 CXorf57 PSIP1 GOT2 MRPS9 HEATR1 PRPF38A FAU IMMT CSRP1 FRG1 RBM12B FKBP4 UPF1 YWHAG HIST1H4I SAMD4B HIST1H4H MRPL54 NUDT21 PDIA4 METTL3 AQR NBPF10 SRSF4 HNRNPK RPL24 HIST2H4B EIF3G GTPBP10 MSI2 MRPS35 MTPAP LUC7L CRYZ MRPS23 SUCLG1 YLPM1 NIP7 HMGN2 LARP4 TFB2M EZR PRPF31 FASTKD3 FCF1 ILF3 SCAF4 U2SURP FUS RPL5 NOC2L ATP5A1 P4HB LUZP4 RBMX CHD3 HOXB6 ARL6IP4 RNF40 DDX52 YWHAE PPIL4 RG9MTD1 BUD13 RBM24 PKN2 SPEN SRP14 TDRD9 KIAA1429 CALR RPS17 URB1 APOBEC3B RPL35 SRPK1 TRMT2A MRPS24 C22orf28 SNRNP35 HTATSF1 SF3B4 RPUSD2 HIST1H4A MRPS11 ZAK ZC3H13 NANOS2 RPL17 RPL35A ZC3H8 SLBP MDH2 CSTF2 PRMT1 METTL16 RPS3A CSDA CRKL RBM7 DCAF13 PURA HNRNPA0 FASTKD1 ILF2 PARK7 POLRMT MAPRE1 ELAVL1 ELAC2 TBL2 NLRP11 RPF2 UPF3B FAM50A RPL22 ALDH18A1 NME1 CSNK1E CDK11B NOP2 HNRNPAB PUM1 HNRNPC TCOF1 DIDO1 RC3H1 DDX1 HIST1H4E DIMT1 PTBP1 POLDIP3 UTP20 RPL7L1 CPEB4 EWSR1 FXR1 RRP36 RAN C1orf31 MRPS31 TCP1 UBE2O NUDT16L1 IREB2 LIN28A SF3B2 RPS16 G3BP1 DDX39A NOVA2 LYAR ZCCHC7 ANXA7 HIST1H4C MRPL10 CORO1A NXF2B GNB2L1 GRB2 ABCF1 KTN1 MRPL39 LIN28B RBM15B PIN4 KHDRBS1 AUH HSPE1 HNRNPF SNRPA1 HSP90AB1 NAA38 HNRNPR EFTUD2 DSP SRSF11 CSTF3 NOP58 RPL18A U2AF2 DHX34 EEFSEC DAZL ESRP1 RPL29 RBM8A STXBP1 EIF4H FUBP1 PCDHGA9 MKRN1 PELP1 C14orf21 NCOA5 IGF2BP1 SRSF3 ZCCHC3 PWP2 RPL6 CCT4 TIAL1 MEX3C PUM2 SNW1 RPS9 RPP30 UBTF TRAP1 SRP68 RBM33 EIF2C1 EDF1 APOBEC1 ELAVL4 EXOSC6 CELF3 HDGF SNRPA ROD1 ACAA2 SARS2 TWF2 DQX1 SNRPG USP10 C11orf31 SUPV3L1 CPEB3 HLA-A ZCRB1 RPL7A PRPF38B STRAP RPL28 MTHFSD TOP1 PUS1 ANXA11 UTP3 ZMAT3 PABPC1 DDX56 NAP1L4 ZNF385A NOP56 TBL3 TRMT1 SECISBP2 HIST1H4B XRN1 RTCD1 RDBP RP9 ARCN1 DNAJC2 ANKRD17 SKIV2L2 PRKRA BRIX1 ZCCHC6 ARHGEF1 NCBP2 HIST1H4F DEK CIRBP UBFD1 HNRNPA1 BOP1 MRPS7 PRKDC NAT10 SF3B1 CLNS1A HELB CDKN2A RPL30 PUS7 RBM45 UTP23 GSPT2 UTP14A PCF11 DNM1 NCL ALKBH5 SMNDC1 LUC7L2 HIST1H1D DDX42 NXF1 SUB1 TXN RBM39 ANKHD1 RAVER2 LBR FARSA MRM1 DDX6 SRRM4 UCHL5 RPS13 RRP12 HDLBP POP1 WDR46 PDCD11 RBM3 RTF1 PCBP1 LRP1 RPS28 GPATCH4 HNRPDL PPIE EIF5 RPL3 RALY HNRNPH3 LSM6 SF1 ZCCHC24 SORBS2 PTRF HIST2H4A NONO MRPL2 HNRNPD MAZ TES WDR36 RRP9 RSRC2 TDRD7 SYNCRIP PDAP1 SYNE1 MRPL1 MAP4 EEF1A1 ACO1 EDC3 NOL11 CCDC9 MRPL32 AATF DDX17 ZNF207 PUF60 FAM46A ELAVL2 GTF2E2 NAF1 C7orf50 PIWIL1 DDX28 CIRH1A CNOT4 ZC3H14 KIAA1967 TCF20 C3orf26 NGRN GSPT1 SUPT16H NOLC1 CCDC59 FLYWCH2 CPSF7 ZC3H12A PPIG THUMPD1 MBD2 DKC1 FAM98B HLTF MEX3D DDX10 DHX38 RPS18 RARA DHX30 DCP1A RPS23 PRRC2B DDX31 DNAJC21 UBE2L3 UHMK1 EIF3D RRP1B RRP7A RPL37A ZC3H10 RPS20 TFRC PABPN1 CARHSP1 DYNC1H1 SRSF5 ZC3H7A LARP7 RBMS3 NXF5 ZNFX1 TAF15 IFIT5 RSL1D1 RPL7 MRPL43 NOC3L ZC3H11A WDR33 TRNAU1AP RPL14 SUPT5H POP7 SRPK2 SRSF1 HDAC2 ACTN4 CELF1 PRDX1 POLR2B FAM208A SNRNP200 SF3A1 RBM34 ADARB1 ZFR MRPL9 KPNB1 MYO5A MRPL21 EIF3L RPS8 RBM26 CCDC137 ROCK2 HMGB3 DROSHA ERI3 SURF6 SCG3 NPM1 C9orf114 ZNF622 RBM19 NMD3 LTA4H HNRNPUL2 AHNAK CPNE3 STAU2 SNRPC CCDC47 TPD52L2 YWHAZ ASS1 MRPS30 PABPC4 APEH TSR1 SAFB2 IBA57 PPP1CC C14orf93 IFI16 CHD2 MRPL42 DAZ3 THOC5 MRPL37 RBM4B RPUSD4 RPL8 ISG20L2 EIF3C DDX21 NUSAP1 TIA1 FAM120A HNRNPH2 YTHDF2 SRFBP1 FKBP3 RBM15 SFPQ RPS24 SAP18 IFIT2 DIAPH1 HSP90AA1 HARS2 TARDBP TNPO1 LRRC47 NXF3 TNS1 NOP16 EIF2C3 CSTF2T LSM14B EIF1 RPL10 TRIP6 EIF4B MPHOSPH10 HADHB MRPS14 ZCCHC8 ZNF768 TRMT6 KHSRP FAM103A1 SNTB2 RRS1 SLC3A2 RBM38

GO_IONOTROPIC_GLUTAMATE_RECEPTOR_BINDING Interacting selectively and non-covalently with an ionotropic glutamate receptor. Ionotropic glutamate receptors bind glutamate and exert an effect through the regulation of ion channels. SHANK1 FLOT2 DLG1 OPHN1 DLG2 RAPSN FUS NETO1 FLOT1 DLG3 DLG4 CACNG2 GNAS SHANK3 CANX RAB4A NEDD4 DRD2 SHANK2 NETO2

GO_MONOOXYGENASE_ACTIVITY Catalysis of the incorporation of one atom from molecular oxygen into a compound and the reduction of the other atom of oxygen to water. CYP1A1 CYP46A1 CYP27B1 TYR NOS3 TPH1 CYP26B1 NOS1 CYP4A11 CH25H FMO5 CYP3A7 CYP11B2 CYP2C8 KMO CYP4Z1 CYP2C19 CYP2S1 CYP7B1 CYP2E1 CYP8B1 CYP3A43 PAM CYP26C1 CYP7A1 CYP4F8 CYP1B1 CYP4F2 CYP4F3 MSMO1 CYP27C1 AKR1C3 DBH MOXD1 CYP11B1 CYP21A2 CYP2D7P1 CYP2W1 CYP2R1 CYP17A1 CYP4A22 CYP3A5 TH CYP39A1 CYP4B1 CYP2C9 NOS2 CYP2A6 C5orf4 FMO3 FMO2 CYP27A1 COQ7 CYP4F22 COQ6 CYP4F11 CYP2D6 PCBD2 CYP2B6 MOXD2P PCBD1 CYP1A2 MICAL2 CYP2A7 CYP4X1 CYP2J2 CYP26A1 FOXRED2 CYP4F12 PTGIS TPH2 CYP2F1 CYP4V2 TBXAS1 FMO4 NLRP11 CYP2C18 CYP2A13 MICAL1 CYP19A1 AKR1C2 AKR1C1 CMAHP CYP24A1 CYP2U1 CYP51A1 DOHH PAH AGMO CYP20A1 TYRP1 FMO1 CYP11A1 MICAL3 SQLE CYP3A4

GO_GTPASE_ACTIVATING_PROTEIN_BINDING Interacting selectively and non-covalently with a GTPase activating protein. TSC1 TUBB RGS14 GNAI3 CDH1 FMNL3 PIN1 AKT1 PLXNB1 FMNL1 GNAO1 GAPVD1 GNAI1 PLCD1

GO_PHOSPHOPROTEIN_BINDING Interacting selectively and non-covalently with a phosphorylated protein. YWHAB LDLRAP1 SAMSN1 NEDD4 CBLB LRP11 PAFAH1B1 PTPN3 PTPN6 GRB2 PIN1 PIH1D1 MID2 SAG CBL TBK1 THRAP3 LYN MID1 ARR3 FKBP4 TRPV1 STAP1 C19orf2 CSNK1G2 RB1 FGR BTRC CRK PLAT APTX YWHAE MAPK1 ARRB1 TBL2 RRAGA MTOR GPRIN1 IGF2R PHF6 ZAP70 SRC PTPN5 MAPK3 PKD2 TOX3 SFN PRKCSH FBXW7 CBX4 CBLC DPYS LEO1 SNCA

GO_DEATH_RECEPTOR_BINDING Interacting selectively and non-covalently with any member of the death receptor (DR) family. The DR family falls within the tumor necrosis factor receptor superfamily and is characterized by a cytoplasmic region of ~80 residues termed the death domain (DD). DAB2IP FADD CASP8AP2 CFLAR CASP3 MADD BID NGFRAP1 RIPK1 CASP8 TMBIM1 FEM1B PIDD NGF FASLG MYD88 NOL3

GO_INTRAMOLECULAR_TRANSFERASE_ACTIVITY Catalysis of the transfer of a functional group from one position to another within a single molecule. RPUSD1 RPUSD2 BPGM PMM1 PUS7L PGM1 PGM3 MUT RPUSD4 PUS1 GPI PGAM1 LSS RPUSD3 PGM5 PMM2 PGM2 PGAM4 PUS7 PGM2L1 PGAM2 TRUB1 TRUB2 PUS3 DKC1 PUSL1 PUS10

GO_TAU_PROTEIN_BINDING Interacting selectively and non-covalently with tau protein. tau is a microtubule-associated protein, implicated in Alzheimer's disease, Down Syndrome and ALS. HSP90AA1 APOE BIN1 HDAC6 AATF APBB1 SNCA S100B PPP2R2A FKBP4 DYRK1A

GO_RETINOIC_ACID_RECEPTOR_BINDING Interacting selectively and non-covalently with the retinoic acid receptor, a ligand-regulated transcription factor belonging to the nuclear receptor superfamily. FUS NR0B2 PRAME HMGA1 RARG MED1 CTBP2 NCOA6 PPARG NR1H4 ACTN4 NR1H2 NCOA1 CNOT1 RARB ASXL1 NR4A2 SNW1 LRIF1 NRIP1 PRMT2 MED25 VDR NSD1

GO_UBIQUITIN_UBIQUITIN_LIGASE_ACTIVITY Isoenergetic transfer of ubiquitin from one protein to an existing ubiquitin chain via the reaction X-ubiquitin + Y-ubiquitin -> Y-ubiquitin-ubiquitin + X, where both the X-ubiquitin and Y-ubiquitin-ubiquitin linkages are thioester bonds between the C-terminal glycine of ubiquitin and a sulfhydryl side group of a cysteine residue. PRPF19 UBOX5 PELI2 ANAPC11 UBE4A UBE2K PPIL2 RBX1 STUB1 UBE4B PELI1 AMFR UBR5

GO_PROTEIN_KINASE_C_ACTIVITY Catalysis of the reaction: ATP + a protein = ADP + a phosphoprotein. This reaction requires diacylglycerol. PRKCQ PRKCA PRKCE PKN2 PRKCH PRKD2 PRKD1 PKN3 CCL3 PRKCD PRKCG PRKCI PRKCZ PRKCB PRKD3 PKN1

GO_PHOSPHOLIPASE_C_ACTIVITY Catalysis of the reaction: a phospholipid + H2O = 1,2-diacylglycerol + a phosphatidate. PLCE1 F2RL2 PLCD3 PLCL2 CHRM5 PLCB3 PLCL1 CASR CHRM1 PLCH2 NOTUM PLCH1 CCL5 PDIA3 PLCG2 PLCZ1 PLCB2 CHRM3 EDNRA PLCB1 PLCD1 PLCB4 PLCD4 PLCG1 CCKBR CCR1 ADORA1 PLD4 CCR5 BDKRB2

GO_TUBULIN_BINDING Interacting selectively and non-covalently with monomeric or multimeric forms of tubulin, including microtubules. MTUS2 AGBL4 KIF1A GAS2 MAPRE1 WASH3P KIF23 ARL3 HSPH1 KIF13B CAPN6 C12orf52 KATNA1 KLC3 GOLGA2 NME1 KIF1C GJA1 DNM2 STARD9 MAP1S NDEL1 JAKMIP2 KIF3A KIF2A FGF13 KIF26A GABARAPL1 KIF22 MDM1 EML3 CLASP2 KIF20A MAPRE2 PEX14 CEP350 KIF21A BRCA2 TBCA PHF6 FAM154A JAKMIP3 TUBGCP4 TUBGCP2 EML1 CCDC165 MZT1 DNM1P46 STMN1 SYBU MACF1 FTCD GAS2L3 FYN NEFH CLIP1 VPS41 DNM3 DCX MAP2 S100A8 PSRC1 VBP1 MARK4 SGIP1 DST NEIL2 STMN3 MAP1LC3C DPYSL2 KIF11 CRIPT PRC1 TTLL6 PARK2 POLB KIF3B CNN3 REEP4 FAM190B FAM175B MAP1LC3A SNCA PLK1 RAE1 MAST2 BLOC1S2 LZTS1 PACRG CEP290 DPYSL5 HTT TRIM54 AGBL1 NUSAP1 CAMSAP3 HOOK1 NCALD WDR81 LYN RACGAP1 WASH1 MAP1LC3B2 SBDS STMN2 TBCD MAP1LC3B TUBGCP6 AGBL5 ARL4C KIF19 CAMSAP1 KIF18A OFD1 SLC6A2 RP1 CHD4 C1orf96 GPAA1 KIF20B RGS2 CAV3 KIF5A TRAF3IP1 PRNP PDE4B SKA2 GAS8 CLIP2 MID1 WASH6P NEFM OGG1 TPR STMN4 C1orf88 ATF5 CHP FES KIAA1383 WDR43 BCAS3 GLI1 REEP1 KIF5B DAG1 LRPPRC BIRC5 VAPB KIF16B TTLL7 DNM1P34 MAP6D1 TPPP RCC2 OPA1 HDGFRP3 RAB11FIP5 LOC100130097 CENPE KIF24 KIF4B CRYAB KIF5C HOOK3 DNM1L CCDC88A ALDOA CLASP1 APC PPP5C EML4 NDE1 KIF26B GABARAPL2 ARL8A AGTPBP1 KIF9 MAP7D3 GAS2L2 KIFC2 GAS2L1 SIRT2 SPAG8 NDN RPS3 KIF25 BCL2L11 SKA1 DNM1 MID2 RAD51D SYT11 KIFC1 PAFAH1B1 SPAST MAP6 CETN2 FAM83D PPP1R42 KIF18B KIF1B TBCC ARHGEF2 KIFC3 FMN1 C9orf24 KIF12 CEP57L1 NDRG1 KRIT1 ADNP KIF6 KATNB1 KIF3C TXNDC3 TUBGCP3 ARL8B MX1 RABGAP1 APC2 LRRK2 TPT1 FAM110C FMR1 FAM154B FEZ1 KIF17 TTLL4 EML2 C19orf20 CDK5RAP2 TRPV4 CLIP3 TUBGCP5 KIF4A VAPA KIF2B PDCD5 UXT DIXDC1 EZR TPPP3 IFT81 SUN2 KIF2C KATNAL1 KIF21B MX2 MAP4 DYNC1I1 MAP1B BRCA1 IFT74 WDR52 KIF14 MAPT CEP57 CCT5 KIF7 ZNF207 S100A9 EMD MAP1A KATNAL2 HDAC6 KIF15 RAB11A RGS14 MAPRE3 KIF13A KLC1 B9D2 INO80 KIF27 FKSG2 FNTA CAMSAP2 BRSK1 TIAM1 WHAMM NUMA1 GAPDH STIM1 PPARGC1A CENPJ BBS4 CETN1 JAKMIP1

GO_ENZYME_INHIBITOR_ACTIVITY Binds to and stops, prevents or reduces the activity of an enzyme. TIMP1 ANGPTL3 ANXA4 SPINLW1 USP14 LCN1 SLPI GP1BA ELFN1 SH3BP5L SERPINC1 HMSD WFDC8 COL4A3 LPA PKIA SOCS3 CST9L BST2 PZP PPP1R1A PRDX5 C3P1 PRKAR2A FLRT3 CSTA PROS1 PTTG1 SERPINB10 NAIP PIF1 ANXA2P2 IPO5 ARPP19 PDE6H PI15 WFDC1 ITIH4 SPOCK1 SPINK13 SERPINB2 ASPN RENBP PPP1R10 APOA2 LRRTM4 SOCS2 PPP1R11 WFIKKN2 SFN CST5 SPOCK2 SNCA CIB1 PHACTR2 PPP1R2P9 PPP1R17 PPP1R14C CST7 CST3 MBIP SOCS4 WFIKKN1 XIAP SERPINB3 SNCB SLN TFPI2 SAG COL28A1 TNFAIP8 RTN4R NLRC4 VIL1 PRKAG2 OVOS PPP1R2P3 CSTL1 ELFN2 SPINT2 WFDC3 SERPINA9 OVOS2 TEN1 SPINK14 PI3 FETUB PAPLN RECK FURIN SERPIND1 SERPINA4 LRP6 SET ITIH2 NOL3 CRIM1 IBTK RNH1 OAZ2 PPP1R9B ATPIF1 CRB2 SERPINI2 CARD18 PPP1R39 APLP2 CDKN2D APOC3 SOCS1 PPP1R14A HEXIM1 WFDC2 SERPINI1 PRKRIP1 ABCE1 SERPINB12 TMBIM6 SERPINB1 LXN SERPINF1 CST1 CABP1 SERPINB8 PCSK1N TRIB3 CDKN2C CDKN1A RPS6KA1 KNG1 PKIG SPINK4 ITIH3 PPP1R1C MAPK8IP1 CST4 LRRTM1 CST6 CD27 SERPINB9 UCN SPOCK3 SERPINE1 PDC FGFR1OP LOC390940 DUS2L PDZD3 UMODL1 PHACTR1 SPINT1 CDKN1C CPAMD8 CD109 PRPSAP2 PRDX3 TIMP4 COL6A3 C5 LRTM1 IQGAP1 NYX RTN4RL1 CARD16 GNB2L1 LMTK2 CARD17 PPP1R35 SLIT2 TRIB2 GMFB DGKI SPRY2 SERPINE3 TXNIP SCGB1A1 R3HDML NPM1 TINF2 CST8 C19orf2 ANP32E SPINK6 PODN WFDC10A CST9 C4A LRRC66 ARRB1 HEXIM2 PI16 SOCS5 LEF1 SERPING1 WDTC1 SERPINA7 FRY WFDC12 DPEP1 PRPSAP1 TAOK3 NOTCH1 ANXA1 APOA1 ANGPTL4 SERPINB11 ENSA A2ML1 CDKN1B GCHFR WFDC5 SERPINA10 GPS1 PRKAR1B SPP2 TNK2 NCK1 TFPI SERPINB6 LRRC4 WFDC10B H2AFY CAMK2N1 AHSG PROL1 SERPINA2 TFAP2B WFDC6 UGT1A8 APBA3 GMFG CISH CHAD SPRED2 TNFSF14 UGT1A1 SCG5 NGF RTKN SERPINB7 PPP1R27 SERPINF2 PPP1R37 SERPINB13 DCN RTN4RL2 APOC1 LRRK2 TIMP3 SERPINA1 KIAA1967 C3 BIRC6 GPS2 SERPINA3 RHOH ANXA3 SPINK5 PHACTR3 PDE6D CABIN1 COL7A1 GLMN SERPINH1 IQGAP2 PPP1R2P1 CTC1 PDE6G APOC2 CDC42SE1 KAT2B SPINK9 PTTG2 INCA1 TRIB1 PLN SERPINA12 PPP1R12A CST11 PPME1 C4B FLRT2 LRRTM3 HSPBP1 CAST BIRC5 PRKAR1A PPP1R14D SPINK2 TESC TIMP2 BGN DNAJC3 A2M SERPINA5 CST9LP1 PPP1R26 MT3 PKIB PSMF1 PPP1R2 CDKN2B HSPB1 SPINK7 FLRT1 SPRED1 WFDC13 OAZ3 KAL1 CPEB2 SH3BP5 SPINT3 OBFC1 PEBP1 PPP1R14B GAS6 BIRC8 ANXA5 SKI SERPINB5 OAZ1 SPINK1 CST2 ITIH1 APP RPS6KA3 CHP SPINT4 PTN UGT1A7 M1 ATP2B4 SSPO SERPINA11 PPP1R36 ITIH5 ARL2 DUSP19 POT1 UCHL5 YWHAG BIRC7 SERPINB4 LRRC4C PODNL1 SERPINA6 SOCS6 CDKN2A PRKAR2B HYAL2 AMBP PPP1R8 BIRC3 SPINK8 BIRC2 SOCS7 LRRC15 SERPINE2 GPC3 AGT ITIH6 WNK1 LRRC19 CSTB CSN2 PINX1 PTTG3P HRG LRRC4B CAMK2N2 AVP FAF2 ANXA2 PPP1R1B

GO_ACTIN_BINDING Interacting selectively and non-covalently with monomeric or multimeric forms of actin, including actin filaments. ANG MYH8 MYO1B WASH1 C17orf46 CORO7 ABRA TWF2 DIAPH1 TMOD3 EGFR NEB ADSSL1 TRPC6 TNS1 PPP1R9B TRPM7 CCR5 DSTN XIRP1 GSN KLHL17 EPS8 SHROOM1 SYNE2 GBP1 XIRP2 ACTN1 KIF18A PICK1 MYO1D MYO6 CORO6 WASF3 KIAA1598 MLPH MYO1F VPS16 TMSB4X EEF2 MYO16 KCNMA1 FMNL2 FGD4 SHROOM2 EPB41L1 TAGLN MYO9A SNTB2 MSN TNNI1 VIL1 SMTN NF2 NRAP MYBPC2 MYH14 EPB49 ABLIM2 WASH6P COTL1 PLS1 MYOT TWF1 EPB41L3 NOD2 TMSB15B MEFV C19orf21 TLN1 MYLK CNN3 SORBS1 MYH7B PFN1 WASL PHACTR2 DNASE1 WASF1 SPTBN2 MYO5B TMSB15A VCL SYNPO2 CGN FLII ARPC5 MYH6 PLDN TMOD1 FSCN3 CACNB2 CTNNA1 RCSD1 TPM2 PSTPIP2 MYO3A INPPL1 ARPC3 PFN4 MICALL2 MYH4 PACRG CTNNAL1 NCALD HOOK1 FXYD5 PDLIM5 TNNI2 MYO1H SPTBN4 ADD3 LMOD1 PAWR CNN1 LCP1 MYL3 DIAPH2 KLHL2 MYO9B JUB TMSB10 TNS4 YWHAH MYO19 ACTR3C VASP ACTN4 HIP1 GAS2L3 CFL2 ARPC1B CORO1A SYNPO2L TRPC5 MTSS1L MYO1A MACF1 SNTG1 MYBPC3 MYO5A ERMN EPS8L2 GMFB GIPC1 SPIRE1 DST ANLN MYO1E MYO1C CORO1B VPS18 TBC1D21 TRIOBP TBCCD1 FSCN1 VILL PARK2 DAAM2 TNNI3 RP2 WIPF2 CROCC KLHL4 SYN1 CNN2 ABL2 MYO7B PARVA PTK2 HIP1R WIPF3 WDR1 SNTA1 CYFIP1 KPTN WASH3P ANXA8 PLS3 ACTN2 SHROOM4 JMY MIB2 CEACAM1 PHACTR1 ENC1 MICAL3 PLEC MKL1 FKBP15 MYO1G MAEA ITGB1 PIP NEXN PPP1R18 USH1C ACTR3 TMEM201 MOBP CFL1 DBNL DAAM1 MARCKS PARVG MAP1S NEBL EPB41 ABLIM1 MPRIP HPCA LIMCH1 PLEKHH2 TMOD4 MICAL2 LIMA1 MYO7A EPS8L1 MYL4 AIF1 EVL AIF1L FHL3 POF1B ACTR2 EMD FLNC C14orf49 ALKBH4 PARVB HDAC6 IMPACT CALD1 LSP1 INO80 FHOD1 CTNNA2 TNNC1 INF2 AVIL CAP1 FSCN2 TNNC2 MYH9 GCET2 MYRIP TNNT3 DIAPH3 IQGAP2 WAS WHAMM SNTG2 ARPC2 CORO2B KLHL5 PHACTR3 RDX TPM1 TPM3 BAIAP2L1 KLHL1 MTSS1 SYNPO TULP1 TLN2 MYH3 GMFG ADD2 MSRB2 MICAL1 MYOZ1 HOMER2 LRRK2 MYPN TNNT2 MYH15 ANTXR1 LMOD3 MED28 ABLIM3 TRPV4 UTRN BIN1 HCLS1 CAPZA2 UXT DIXDC1 PFN2 CORO1C CXCR4 SSH2 LMOD2 OPHN1 EZR COBL SYNE1 RUSC1 SHANK3 MYO3B ENAH ALDOA PXK ABL1 EPB41L2 ARPC5L KLHL3 CLMN CCDC88A MYH1 CAPZA1 TMSB4Y STK38L PPP1R9A FMN2 ARPC1A SEPX1 GC SSFA2 TPM4 SSH1 PHACTR4 MYH10 ADD1 DMD LRRC10 SPTA1 CAMK2B ESPN PPP1R42 FRG1 SPTB NOS3 FMNL3 SPTBN1 CAP2 MYO18A PKNOX2 PRKCE SVIL MYO10 CTNNA3 IPP AFAP1 MYOZ2 FMN1 COBLL1 MYH13 CAPG WASF2 PALLD ARPC4 ACTR3B KLHL20 ACTN3 BCL7B MYH11 SLC6A4 TMOD2 DBN1 SPTBN5 KLHL33 MYH2 SNTB1 SHROOM3 MYO15A MYO18B FBXO25 FLNA MYL2 MYOZ3 LRPPRC DAG1 SSH3 FMNL1 CORO2A MYO5C FHOD3 CAPZA3 PANX1 SPTAN1 FLNB MYH7 SPIRE2 SCIN WIPF1 TTN MYBPC1 LASP1 PFN3 CAPZB S100A4 MARCKSL1

GO_RECEPTOR_SIGNALING_PROTEIN_ACTIVITY Conveys a signal from an upstream receptor or intracellular signal transducer, converting the signal into a form where it can ultimately trigger a change in the state or activity of a cell. STK25 BAG4 ARAF DOK1 ACVR1C INSR TAOK2 CDKN1B GNAZ TGFB2 ERBB2 TGFBR1 FLRT3 PAK4 TRAIP STK4 LYN MAPK4 MAP3K15 NLK SMAD3 PAK7 MAP3K13 YSK4 KIAA1804 MAPK1 MAPK8 KIT MOS SMAD2 PDCL DAXX PLCG1 MAP4K1 MAP3K5 ACVRL1 BMPR1B NRK MAPK3 DOK2 ACVR2A MAPK12 MAP3K7 ZAK MAP3K3 PIP4K2B MAP3K1 SMAD4 TIAM1 PGAM5 MINK1 BMPR2 MAP4K4 IRAK1 RAF1 MAPK6 IL4R MAPK7 RIPK3 SMAD1 IFITM1 OXSR1 IKBKE AMHR2 STK39 SBK2 C21orf7 MAPK14 ACVR2B EGFR MAPK13 MAP3K6 CD3E SLK ARF1 FLRT2 PAK2 BRAF TYRO3 TNIK MAP3K2 DOK5 RGS14 DOK4 ADRB1 DCLK1 MAP3K9 IL1RL1 MAP4K5 TIAM2 STK10 PLCE1 NEK1 ENG MAP3K10 FCGR1A FLRT1 MAPK10 PPP4C MAP4K3 NSMAF SMAD6 MAP3K8 PAK3 MAPK15 MAP2K6 SMAD7 ACVR1B SYK BAG1 PAK1 SMAD5 MST4 MAP2K1 TGFBR2 MAP2K7 CD19 STK24 MAP2K2 ALK STK3 MAPK11 ACVR1 MAP3K12 SMAD9 MAP2K3 MAP2K4 ADCYAP1 MAP2K5 MAP3K14 ERBB4 KDR MAP4K2 PAK6 MAP3K11 BMPR1A NEK4 TAOK1 MAP3K4 NCR1 TAOK3 MAPK9 TYROBP RGS12

GO_CYSTEINE_TYPE_ENDOPEPTIDASE_INHIBITOR_ACTIVITY_INVOLVED_IN_APOPTOTIC_PROCESS Stops, prevents or reduces the activity of a cysteine-type endopeptidase involved in the apoptotic process. CD27 SERPINB9 BIRC3 XIAP BIRC2 ARRB1 TNFSF14 AVP TFAP2B VIL1 SNCA RPS6KA3 PRDX3 TNFAIP8 PRDX5 LEF1 GAS6 BIRC8 MT3 RPS6KA1 DPEP1 NOL3

GO_EXONUCLEASE_ACTIVITY_ACTIVE_WITH_EITHER_RIBO_OR_DEOXYRIBONUCLEIC_ACIDS_AND_PRODUCING_5_PHOSPHOMONOESTERS Catalysis of the hydrolysis of ester linkages within nucleic acids by removing nucleotide residues from the 3' or 5' end to yield 5' phosphomonoesters. C16orf57 CNOT2 CNOT7 ISG20L2 PNPT1 DIS3L2 EXOSC10 PAN3 EXOSC3 FEN1 EXOSC5 CNOT8 EXOSC4 RAD1 APEX1 DEM1 ISG20 DCLRE1C DCLRE1A EXOSC2 CNOT6 DCP2 TREX2 EXD2 TOE1 CNOT6L RAD9A PARN PDE12 DIS3L PAN2 C16orf73 CCRN4L REXO2 CNOT1 XRN2 EXO1 APEX2 APTX C20orf72 DCLRE1B EXOSC9 EXOSC7 DIS3 POLD1

GO_METALLOCARBOXYPEPTIDASE_ACTIVITY Catalysis of the hydrolysis of C-terminal amino acid residues from a polypeptide chain by a mechanism in which water acts as a nucleophile, one or two metal ions hold the water molecule in place, and charged amino acid side chains are ligands for the metal ions. CPB2 CPD AGBL3 PEPD CPXM2 AGBL1 CPO CPN1 CPM CPXM1 AGBL4 ACE2 FOLH1 CPE FOLH1B CPB1 CPA2 AGBL2 AGBL5 CPA1 CPA5 CPA3 CPA6 AGTPBP1 AEBP1 CPA4 CPZ

GO_CARBON_SULFUR_LYASE_ACTIVITY Catalysis of the elimination of hydrogen sulfide or substituted H2S. ACCS CENPV CTH CCBL1 SCLY MGST2 ALOX5AP HCCS GLO1 CCBL2 LTC4S

GO_INOSITOL_TRISPHOSPHATE_KINASE_ACTIVITY Catalysis of the reaction: inositol trisphosphate + ATP = inositol tetrakisphosphate + ADP. CALM3 IP6K1 IP6K2 ITPKC CALM1 ITPKB ITPK1 ITPKA CALM2 IP6K3 IPMK

GO_MONOSACCHARIDE_TRANSMEMBRANE_TRANSPORTER_ACTIVITY Enables the transfer of a monosaccharide from one side of a membrane to the other. SLC2A6 PPBP SLC2A1 MFSD4 SLC5A2 SLC5A4 M6PR SLC2A10 SLC2A4 SLC2A14 SLC2A12 SLC5A1 SLC2A13 SLC2A3 SLC2A5 KIAA1919 SLC5A9 SLC2A8 SLC2A9 SLC2A2

GO_PEPTIDOGLYCAN_MURALYTIC_ACTIVITY NA LYZL4 PGLYRP3 SPACA5B PGLYRP2 PGLYRP1 LYG2 LYZL6 SPACA5 LALBA LYZ LYZL2 SPACA3 LYZL1 PGLYRP4 LYG1 CHIA

GO_MANNOSIDASE_ACTIVITY Catalysis of the hydrolysis of mannosyl compounds, substances containing a group derived from a cyclic form of mannose or a mannose derivative. MAN2B1 MAN1C1 MAN2B2 MAN1A1 MAN1B1 EDEM3 MAN1A2 KIAA2018 MAN2C1 MANEA MAN2A2 MAN2A1 MANBA EDEM1 EDEM2

GO_RNA_POLYMERASE_III_ACTIVITY Catalysis of the reaction: nucleoside triphosphate + RNA(n) = diphosphate + RNA(n+1). Utilizes a DNA template that contains an RNA polymerase III specific promoter to direct initiation and catalyses DNA-template-directed extension of the 3'-end of an RNA strand by one nucleotide at a time. Can initiate a chain 'de novo'. POLR3A POLR3F POLR3B POLR2L POLR3G POLR1C POLR2H POLR3H POLR3C POLR3E POLR2F CRCP POLR2K POLR3D POLR3K POLR2E POLR3GL POLR1D

GO_PHOSPHATIDYLSERINE_BINDING Interacting selectively and non-covalently with phosphatidylserine, a class of glycophospholipids in which a phosphatidyl group is esterified to the hydroxyl group of L-serine. PLCD1 SYT1 TIMD4 SYTL2 JPH2 OSBPL8 GSDMD HSPA8 GAS6 OSBPL10 FCHO2 THBS1 TRIM72 CPNE1 MFGE8 SCARB1 RS1 CD300A SYT10 SESTD1 SYT9 SCIN SYT4 SYT7 RPE65 ANXA9 RASGRP1 SYT6 HMGB1 AXL MARK1 GAP43 OSBPL5 CPNE6 SYT5 SDPR

GO_ACTIN_FILAMENT_BINDING Interacting selectively and non-covalently with an actin filament, also known as F-actin, a helical filamentous polymer of globular G-actin subunits. PKNOX2 TNNC2 SPTB MYH9 MYO18A CORO1B FRG1 SHROOM2 MYO1E FSCN2 ESPN MYO16 EEF2 SPTA1 TNNC1 VPS16 ADD1 ERMN CTNNA2 KIAA1598 MYH10 WIPF2 MYH14 FSCN1 ARPC2 CORO2B CTNNA3 TRIOBP IQGAP2 MYO10 VIL1 SVIL EGFR ADSSL1 ACTR2 ABL1 AIF1L LCP1 AIF1 MYO1B MYL4 MYO7A MYH8 PICK1 MACF1 CORO6 MYO1D MYO6 ACTN1 MYO1A SSFA2 ARPC1B CFL2 ARPC1A CORO1A SYNE2 PPP1R9A SHROOM1 KLHL17 ACTN4 DSTN C14orf49 FLNC JUB PPP1R9B MARCKS MAP1S CORO1C DBNL TMEM201 CFL1 CORO2A MICALL2 UXT UTRN ACTR3 BIN1 FMNL1 ARPC3 USH1C LRPPRC RCSD1 TRPV4 CAPZB SYNE1 LIMA1 LASP1 TMOD4 WIPF1 EZR TTN SCIN CTNNAL1 NEBL PANX1 SHROOM4 PLS3 ACTN2 CYFIP1 ANXA8 MYH3 TLN2 ADD2 TLN1 TULP1 WIPF3 WDR1 ARPC4 ABL2 PLS1 CACNB2 FLNA CTNNA1 NEXN PLDN TMOD1 ARPC5 FSCN3 SHROOM3 ANTXR1 MYH11 SLC6A4

GO_SERINE_TYPE_EXOPEPTIDASE_ACTIVITY Catalysis of the hydrolysis of a peptide bond not more than three residues from the N- or C-terminus of a polypeptide chain by a catalytic mechanism that involves a catalytic triad consisting of a serine nucleophile that is activated by a proton relay involving an acidic residue (e.g. aspartate or glutamate) and a basic residue (usually histidine). PREP CPD CPVL CPXM2 HPN SCPEP1 CPM F11 CPN1 CPXM1 CTSA CPE PRCP PRSS16 DPP7 CPZ PREPL AEBP1

GO_PROTEIN_SERINE_THREONINE_PHOSPHATASE_ACTIVITY Catalysis of the reaction: protein serine phosphate + H2O = protein serine + phosphate, and protein threonine phosphate + H2O = protein threonine + phosphate. PPP3R1 PPP1R3C PPP3CA PP2D1 RPAP2 PPP5C PPP2R3B DUSP23 TIMM50 MTMR4 CTDNEP1 PPP1R3D PPA2 MYH8 PDP1 PPP1CA PPP1R15B MTMR3 CAMK2G PPEF2 MTMR14 PPP2CB PPP3CB PPM1E SSU72 PPM1B PPP1CB CDC14C PGAM5 CTDSP2 PTEN PPM1L PPP2R2A TAB1 LCK CTDP1 PPM1K CYCS CTDSP1 PPP1CC PHLPP2 MYH6 PPP2R2C PPM1M CDC14A PPEF1 PPTC7 ILKAP MYH3 PPP2R1A PPM1H PDP2 CDC14B PPM1D PPP4C PPM1A PPM1N PPM1G MTMR6 PPP2R2D CDKN3 PPM1J PPP6C PPP2R5D PPP2R2B UBLCP1 PPM1F

GO_FRIZZLED_BINDING Interacting selectively and non-covalently with the frizzled (fz) receptor. FZD7 DVL2 BAMBI FZD1 WNT11 WNT9A SDCBP WNT2B WNT3A DVL3 WNT10B WNT7A WNT3 WNT5A WNT10A CTHRC1 WNT1 WNT16 MYOC GOPC WNT4 ZNRF3 WNT8A NDP WNT6 LRP6 RYK WNT2 WNT5B WNT8B SFRP1 ROR2 WNT9B RNF43 DVL1 RSPO3 WNT7B

GO_INTRAMOLECULAR_TRANSFERASE_ACTIVITY_PHOSPHOTRANSFERASES Catalysis of the transfer of a phosphate group from one position to another within a single molecule. BPGM PGM2L1 PMM1 PGM1 PGAM4 PGM2 PGM5 PMM2 PGAM1 PGAM2 PGM3

GO_ACETYLGLUCOSAMINYLTRANSFERASE_ACTIVITY Catalysis of the transfer of an N-acetylglucosaminyl residue from UDP-N-acetyl-glucosamine to a sugar. XYLT1 LFNG PIGH RFNG B3GNT2 MGAT5 C3orf64 B3GNT3 EXT1 OGT A4GNT XYLT2 EXTL3 B3GNT1 CCDC126 B3GNT6 HEXA B3GNT4 EXT2 B3GNT7 B3GNT8 MFNG ALG13 PIGA LARGE MGAT4C PIGC PIGQ GCNT2 B3GALNT2 EXTL2 POMGNT1 MGAT2 MGAT1 MGAT5B PIGP ALG14 MGAT4A HEXB EXTL1 GCNT3 MGAT3 B3GNT5 GCNT4 MGAT4B GCNT6 C3orf39 GCNT1 GCNT7

GO_HSP70_PROTEIN_BINDING Interacting selectively and non-covalently with Hsp70 proteins, any of a group of heat shock proteins around 70kDa in size. PPEF2 FGF1 PACRG RNF207 CDKN1B ERN1 DNAJA1 GPR37 DNAJB1 PPID CREB1 NOD2 DNAJC2 IQCG ST13 DNAJA3 RPS3 CDK1 STUB1 DNAJC10 MVD METTL21A PARK2 NUP62 SACS C11orf73 HDAC8 BAG6 STIP1

GO_TRANSLATION_REGULATOR_ACTIVITY_NUCLEIC_ACID_BINDING Any selective and non-covalent interaction with a nucleic acid involved in the initiation, activation, perpetuation, repression or termination of polypeptide synthesis at the ribosome. RARA LARP1 PAIP1 DAZ1 CPEB2 DAZ3 RPS27L BOLL CELF4 DAZL PABPC1 CELF1 ZNF540 PURA PAIP2B CPEB4 CPEB1 CPEB3

GO_PROTEIN_KINASE_C_BINDING Interacting selectively and non-covalently with protein kinase C. PRKCDBP DACT2 AVPR1A PLEK TDG TOP2A TOP2B TWF2 ADAM9 ABL1 GLRX3 DSP PRKCSH PICK1 FEZ1 HDAC7 GRK5 PRKCB SRC AVPR1B PARD6A HINT1 C1QBP UGT1A7 MARCKS ITGAV TIRAP HDAC9 LDB3 SDPR UGT1A10 DACT1 SRSF2 GNB2L1 TRPV4 SDC4 HDAC5 DACT3 YWHAG PDLIM5 SQSTM1 PKN1 HSPB1 PKP2 AKT1 IRS1

GO_PSEUDOURIDINE_SYNTHASE_ACTIVITY Catalysis of the reaction: RNA uridine = RNA pseudouridine. Conversion of uridine in an RNA molecule to pseudouridine by rotation of the C1'-N-1 glycosidic bond of uridine in RNA to a C1'-C5. TRUB2 RPUSD3 PUS3 DKC1 PUSL1 PUS10 RPUSD4 PUS1 TRUB1 RPUSD2 PUS7 PUS7L RPUSD1

GO_MITOGEN_ACTIVATED_PROTEIN_KINASE_KINASE_BINDING Interacting selectively and non-covalently with a mitogen-activated protein kinase kinase, any protein that can phosphorylate a MAP kinase. TRIB1 TRIB2 MAP3K11 PIN1 DAB2IP TAOK2 ARRB1 ACE IGBP1 MAPK8IP1 KSR1 DLG1 RAF1 BRAF MAPK8IP3 TRIB3

GO_NUCLEOSIDE_TRANSMEMBRANE_TRANSPORTER_ACTIVITY Enables the transfer of a nucleoside, a nucleobase linked to either beta-D-ribofuranose (ribonucleoside) or 2-deoxy-beta-D-ribofuranose, (a deoxyribonucleotide) from one side of a membrane to the other. SLC29A2 SLC25A6 SLC25A42 SLC25A17 SLC28A1 SLC29A4 SLC35B3 SLC25A24 SLC29A3 SLC29A1 SLC28A2 SLC28A3 SLC35B2

GO_ACTININ_BINDING Interacting selectively and non-covalently with actinin, any member of a family of proteins that crosslink F-actin. PDLIM5 PKD2 SYNPO2 XIRP2 TRPC5 RELA PROM1 CACNA1C TRPC6 KCNA5 KCNN2 PPARG CSRP3 TTN PDLIM2 MYPN ALMS1 NRAP MICALL2 CACNA1D LDB3 MAGI1 PKD2L1 PALLD MYOT NFKB1 RARA LRRC10 DAG1

GO_TRANSFERASE_ACTIVITY_TRANSFERRING_GLYCOSYL_GROUPS Catalysis of the transfer of a glycosyl group from one compound (donor) to another (acceptor). FUT7 PARP3 GALNT11 UGT2B15 GALNTL6 PYGB PYGL B3GALTL CCDC126 B3GNT4 SDF2L1 QPRT B3GNT3 C6orf108 FUT5 PARP2 GALNT2 C3orf64 XYLT2 UGGT1 ABO ART3 GLT6D1 POGLUT1 ST6GAL2 B4GALNT4 GCNT7 MGAT4B C1GALT1 ST6GALNAC4 KDELC1 PIGQ B3GAT3 PIGC ZC3HAV1 B3GALT4 POMT1 DPM2 B3GNTL1 POMT2 ST6GALNAC5 XXYLT1 ST8SIA4 UGT2B11 B3GAT2 ART1 HEXA UGT1A10 GLT25D2 DPY19L4 SIRT5 XYLT1 SIRT1 RFNG GALNTL5 ALG1 GGTA1P LALBA PRTFDC1 TUSC3 PARP4 OSTC UGT2B10 NAMPT GCNT3 PARP15 ALG14 DPM1 FUT9 PARP1 POMGNT1 UGT2B4 GLT8D1 ALG10 B4GALT6 GALNT8 DAD1 UGT2A1 ST3GAL5 UGT1A3 NAPRT1 ALG5 HPRT1 EXT1 ALG1L2 HAS1 GALNT13 EXTL3 PNP ST3GAL4 UGT1A6 B3GNT2 GYLTL1B GTDC1 ALG8 GCNT1 GALNT12 PIGM PIGP PARP11 B3GNT5 UGT1A5 PDC STT3A MGAT5B B3GALNT2 GCNT2 EXTL2 LARGE ALG9 B3GNT7 PIGA UPP1 MFNG FUT4 GALNTL4 CHPF FUT10 AGL B3GALT5 GLT8D2 PARP6 FUT11 A4GNT ST8SIA2 ART4 B3GAT1 C3orf39 B4GALT3 PDCL2 TXNDC9 B4GALNT3 ST6GAL1 GYS1 GALNT10 ST6GALNAC2 HAS2 CSGALNACT1 POFUT1 GALNT5 UGT3A1 LRRC9 B3GNT8 SIRT4 UGCG ALG1L GBGT1 DPAGT1 ST8SIA3 CHSY1 PIGZ UGT1A4 B4GALNT1 B4GALT7 DPY19L1 MGAT5 FUT1 TIPARP UGT2B7 UGT1A8 GALNT14 UGT1A9 GLT1D1 PIGB PDCL PARP10 FUT2 GXYLT2 UGT1A1 GCNT6 PDCL3 PPAT DPY19L2 PIGV MGAT1 GALNT9 ST6GALNAC1 GALNT1 DPY19L2P1 DPY19L2P2 SIRT6 SDF2 ALG3 UGGT2 RPN2 ST8SIA6 TYMP TNKS UGT3A2 ST8SIA5 ST8SIA1 A3GALT2P GALNT6 B3GALNT1 PARP14 PARP16 OGT LFNG PIGH ALG11 EXTL1 ART5 UGT8 GLT25D1 LOC100288842 C20orf173 B4GALT1 SIRT3 A4GALT UGT2B28 STT3B GALNTL1 ALG6 EXT2 GALNTL2 PYGM B3GALT6 APRT UGT2B17 ALG12 GBE1 GALNT7 KDELC2 CSGALNACT2 POFUT2 UPP2 ST3GAL6 MGAT3 HEXB QTRT1 C1GALT1C1 DDOST WDFY3 GALNT4 B3GNT9 GALNT3 CHPF2 CHSY3 PARP12 MGAT2 B3GALT1 GYG2 ALG2 GYS2 GYG1 RPN1 B3GALT2 FUT8 C15orf58 ALG13 UGT1A7 FUT6 B3GNT6 B3GNT1 UMPS ST3GAL2 PARP8 DPM3 QTRTD1 PLOD3 ALG10B B4GALT4 TNKS2 B4GALT2 PARP9 MTAP WBSCR17 UGT2A3 GXYLT1 MGAT4A HAS3 B4GALT5 GCNT4 B4GALNT2 HYAL1 ST3GAL3 SIRT2 DPY19L3 ST6GALNAC6 ST3GAL1 FUT3 ST6GALNAC3 MGAT4C LOC152586

GO_PHOTORECEPTOR_ACTIVITY The function of absorbing and responding to incidental electromagnetic radiation, particularly visible light. The response may involve a change in conformation. OPN1SW CRY2 RHO CRY1 OPN1LW ELOVL4 OPN3 RRH RGR OPN4 OPN1MW2 GNAT2 OPN5 OPN1MW

GO_THREONINE_TYPE_PEPTIDASE_ACTIVITY Catalysis of the hydrolysis of peptide bonds in a polypeptide chain by a mechanism in which the hydroxyl group of a threonine residue at the active center acts as a nucleophile. PSMA1 PRSS50 PSMA8 PSMA5 PSMA3 TASP1 PSMB8 PSMA2 PSMA6 PSMA4 PSMB10 PSMB6 PSMB11 PSMB4 PSMB1 PSMA7 PSMB3 PSMB7 PSMB5 PSMB9 PSMB2

GO_CYSTEINE_TYPE_ENDOPEPTIDASE_INHIBITOR_ACTIVITY Stops, prevents or reduces the activity of a cysteine-type endopeptidase, any enzyme that hydrolyzes peptide bonds in polypeptides by a mechanism in which the sulfhydryl group of a cysteine residue at the active center acts as a nucleophile. HRG DPEP1 NOL3 CST5 LEF1 CSTB GAS6 BIRC8 RPS6KA3 PRDX3 AVP CST2 SERPINB13 CST11 SNCA BIRC2 ARRB1 TNFSF14 CST9 CST4 PTTG1 NAIP BIRC3 CD27 SPOCK1 FETUB CST6 SERPINB9 BIRC7 RPS6KA1 AHSG CST9L TNFAIP8 PRDX5 MT3 CSTA CSTL1 TFAP2B KNG1 VIL1 NLRC4 CST8 BIRC6 XIAP WFDC2 LCN1 BIRC5 CST7 CARD18 CAST CST3 CST9LP1 CST1 CARD16 CARD17 SERPINB3

GO_RECEPTOR_REGULATOR_ACTIVITY The function of interacting (directly or indirectly) with receptors such that the proportion of receptors in the active form is changed. ANXA2 NRG3 PCSK9 IL1RN PPARGC1B WNT4 LRPAP1 EFNA5 WNT8A PXDN ESR2 BAZ1B ANGPT4 GAS6 NRG1 WNT2 IL36RN CCL5 CDK5 SFRP2 LYNX1 ACTN2 GREM1 EGF CXCL13 VEGFA LYPD1 MED16 MED1 IL18BP WNT3A WNT7A WNT10B AGTR2 NODAL WNT5A NCOA2 DKK1 WNT3 PRKCE FNTA ADH7 WNT1 IGF2

GO_PHOSPHOLIPASE_ACTIVATOR_ACTIVITY Increases the activity of a phospholipase, an enzyme that catalyzes of the hydrolysis of a glycerophospholipid. PLAA GM2A STX4 APOC2 CCL5 ARF1 PDPK1 NSMAF CCL8 CASP3 CCL3

GO_TRANSLATION_ELONGATION_FACTOR_ACTIVITY Functions in chain elongation during polypeptide synthesis at the ribosome. EEF1E1 EEF1A1P5 GFM2 EEF1G EEF1D EEF1A2 EEF1B2 GTPBP2 EEFSEC TSFM EEF2 HBS1L EIF5A2 EEF1A1 TUFM GFM1 EIF5AL1 GTPBP1 EIF5A ABTB1

GO_GTPASE_INHIBITOR_ACTIVITY Stops, prevents or reduces the activity of any enzyme that catalyzes the hydrolysis of GTP to GDP and orthophosphate. CDC42SE1 IQGAP1 GPS2 IPO5 PDE6D RTKN ARL2 DGKI TNK2 RHOH SLIT2 GPS1 CPEB2 IQGAP2

GO_FOUR_WAY_JUNCTION_DNA_BINDING Interacting selectively and non-covalently with DNA containing four-way junctions, also known as Holliday junctions, a structure where two DNA double strands are held together by reciprocal exchange of two of the four strands, one strand each from the two original helices. HMGB1 MSH6 XRCC3 XRCC2 YY1 MEN1 RAD51D DMC1 HMGB2 RAD51B MSH2 HMGB3 RAD51C RAD51

GO_G_PROTEIN_COUPLED_NUCLEOTIDE_RECEPTOR_ACTIVITY Combining with a nucleotide and transmitting the signal across the membrane by activating an associated G-protein; promotes the exchange of GDP for GTP on the alpha subunit of a heterotrimeric G-protein complex. P2RY6 P2RY10 P2RY2 GPR171 P2RY13 P2RY11 PTAFR P2RY8 P2RY4 P2RY14 P2RY1 GPR34 GPR87 P2RY12

GO_ACTIN_MONOMER_BINDING Interacting selectively and non-covalently with monomeric actin, also known as G-actin. TMSB15A MKL1 CORO1A LMOD3 MTSS1L COBL LIMA1 COBLL1 TMSB10 TMSB4Y PFN4 MYL3 TWF2 PFN2 ABL1 PFN1 NOS3 PRKCE PKNOX2 MYL2 MYL4 TWF1 MTSS1 ABL2 TMSB15B TMSB4X
[truncated: 385,857 more chars]
