# Supplementary material for: Exploring autophagy with Gene Ontology
Source: Autophagy. 2018 Feb 17;14(3):419–36. doi: 10.1080/15548627.2017.1415189 (PMC5915032; doi:10.1080/15548627.2017.1415189)
Supplement: supp_data_1415189.zip [file kaup-14-03-1415189-s001.zip › supp_data_1415189/supp_data_1415189_s04.docx]

**File S4.** Phenotype file used to perform Gene Set Enrichment Analysis. Analysis was performed as detailed in Materials and Methods.

59 2 1

# Controls Patients

0 0 0 0 0 0 0 0 0 0 0 0 0 0 0 0 0 0 0 1 1 1 1 1 1 1 1 1 1 1 1 1 1 1 1 1 1 1 1 1 1 1 1 1 1 1 1 1 1 1 1 1 1 1 1 1 1 1 1
